# Effects of RAL signal transduction in KRAS- and BRAF-mutated cells and prognostic potential of the RAL signature in colorectal cancer

**DOI:** 10.18632/oncotarget.3871

**Published:** 2015-04-19

**Authors:** Balázs Győrffy, Iwona Stelniec-Klotz, Christian Sigler, Katharina Kasack, Torben Redmer, Yu Qian, Reinhold Schäfer

**Affiliations:** ^1^ MTA TTK Lendület Cancer Biomarker Research Group, Budapest, Hungary; ^2^ Semmelweis University, 2nd Department of Pediatrics, Budapest, Hungary; ^3^ MTA-SE Pediatrics and Nephrology Research Group, Budapest, Hungary; ^4^ Laboratories of Functional Genomics and Molecular Tumor Pathology, Charité Universitätsmedizin Berlin, Germany; ^5^ German Cancer Consortium (DKTK), German Cancer Research Center, Heidelberg, Germany

**Keywords:** colon cancer, progression free survival, signal transduction

## Abstract

Our understanding of oncogenic signaling pathways has strongly fostered current concepts for targeted therapies in metastatic colorectal cancer. The RALA pathway is novel candidate due to its independent role in controlling expression of genes downstream of RAS.

We compared RALA GTPase activities in three colorectal cancer cell lines by GTPase pull-down assay and analyzed the transcriptional and phenotypic effects of transient RALA silencing. Knocking-down RALA expression strongly diminished the active GTP-bound form of the protein. Proliferation of *KRAS* mutated cell lines was significantly reduced, while BRAF mutated cells were mostly unaffected. By microarray analysis we identified common genes showing altered expression upon RALA silencing in all cell lines. None of these genes were affected when the RAF/MAPK or PI3K pathways were blocked.

To investigate the potential clinical relevance of the RALA pathway and its associated transcriptome, we performed a meta-analysis interrogating progression-free survival of colorectal cancer patients of five independent data sets using Cox regression. In each dataset, the RALA-responsive signature correlated with worse outcome.

In summary, we uncovered the impact of the RAL signal transduction on genetic program and growth control in KRAS- and BRAF-mutated colorectal cells and demonstrated prognostic potential of the pathway-responsive gene signature in cancer patients.

## INTRODUCTION

With nearly 800,000 new cases each year, colon cancer is the second most common malignancy in the world. Since several years, targeted therapies directed against receptor tyrosine kinases (RTK) as key components of the cellular signaling system are in clinical use. The NCCN guidelines (http://www.nccn.org, version 1.2014) list bevacizumab, cetuximab or panitumumab in combination with 5-fluorouracil chemotherapy as initial therapy option for advanced or metastatic disease. The therapeutic antibodies panitumumab and cetuximab can even be initially used as single agents in patients not eligible for combination therapy. RTK signals converge on RAS proteins, which serve as central molecular switches for intracellular communication and control of gene expression. Activating mutations in the KRAS gene are highly prevalent in colorectal and other cancers [1]. Therapeutic strategies targeting mutant RAS proteins directly have either failed in the clinic [2] or are still in early development [3]. In colorectal tumor patients, the mutational status of the KRAS isoform of the RAS gene family permits to roughly discriminate antibody therapy responders from non-responders. Therapies usually fail in KRAS-mutated tumors, while therapeutic benefit is observed in approx. one fifth of KRAS-wild-type tumors [4]. There is growing evidence showing that alternate mutations not affecting KRAS itself may also preclude efficient antibody therapy. Consequently, detailed patient stratification considering further critical elements of the RTK/RAS signaling system is likely to be essential, as is a detailed characterization of their impact on cytoplasmic signaling and gene expression control [5, 6].

The epidermal growth factor receptor (EGFR)/RAS signaling system regulates cellular proliferation, energy metabolism, survival and architecture, migration and angiogenesis via cytoplasmic effectors and transcriptional targets (for review see: [7]). Activated RAS proteins communicate with three major downstream effector pathways, the RAS-RAF-MAPK, the phosphoinositide 3-kinase (PI3K)-AKT and the RALGDS/RALA/B pathways [8]. Considerable efforts have been made to assign the individual branches of the signaling system downstream of RAS to specific phenotypic properties [9]. For example, the RAF pathway was linked to proliferation [10], apoptosis [11], energy metabolism [12] and angiogenesis. The PI3K pathway was associated with overlapping functions such as cell proliferation [13], and evasion of apoptosis [14] as well as with specific functions such as macrophage recruitment [15]. The RALGDS/RAL pathway composed of two small GTP-binding proteins RALA and RALB contributes to proliferation, anchorage independent growth [16], tumorigenicity [17], migration and metastasis [18, 19]. Moreover, the RALA pathway is known to stimulate metastasis of RAS transformed fibroblasts *in vitro* and *in vivo* [18]. In KRAS mutated human pancreatic carcinoma cells RALA is found to be necessary for anchorage-independent growth *in vitro* and for tumor growth *in vivo* [17]. In mouse models of KRAS mutated prostate cancer, RALB is shown to mediate tumor growth, cell migration and bone metastasis *in vivo* [20]. In colorectal cancer cells, the RALA and RALB pathways show antagonistic roles in regulating anchorage-independent growth [16].

Major efforts are underway to design inhibitors to block the RAF/MAPK and PI3K/AKT pathways and to use anti-MAPK and anti-PI3K drugs in clinical trials [21, 22, 23]. In contrast, the RAL pathway has not been targeted in a comparable manner [24]. In view of the functional relevance of the RAS/RAL pathway, further investigations on its contribution to cancer cell phenotypes and the deregulation of the transcriptome are warranted. Finding out if the RAL branch of the RAS signaling system impinges on distinct pathway targets or simultaneously on genes responsive to MAPK or PI3K pathways [25, 26] is of central importance for understanding its global function and for evaluating its relevance for cancer therapy.

In view of the role of RALA in RAS-induced tumorigenesis in human cells [27] and particularly its involvement in colorectal cancer [28], we investigated the role of RALA in colorectal cancer cell lines carrying KRAS mutations in codon 12, 13 or the BRAF V600E mutation. We silenced RALA expression by RNA interference, investigated the effect on cellular phenotypes and contrasted RALA-dependent transcriptional profiles with MAPK and PI3K-dependent ones. In addition, we studied the prognostic potential of RAL-pathway targets by performing a meta-analysis of publicly available microarray-based expression profiles of colorectal cancer patients with documented clinical outcomes.

## RESULTS

### RALA activity and RAL pathway-mediated phenotypic effects in colorectal cancer cell lines harboring different driver mutations

**Figure 1:**
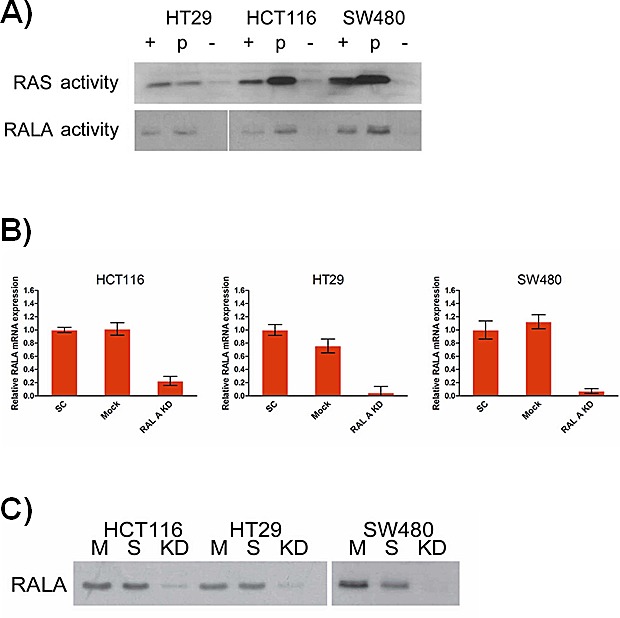
**A.** RAL and RAS activity assays using lysates obtained from SW480 (KRAS mutation in codon 12), HCT116 (codon 13) and HT29 (KRAS wild-type, BRAFV600E mutation) cells *(+: positive control; -: negative control*). **B.** TaqMan RT-PCR analysis of RALA mRNA levels in the same cells following RALA knock-down using siRNA described by [27] and controls (*p* < 0.05). (C) RALA activity assay following knock-down (SC: scramble siRNA transfected control, KD: RALA knockdown, M: mock - transfection reagents only).

RALA activity, as measured by GTP-binding, was highest in SW480 cells, harboring mutated KRAS G12V and in HCT116 cells harboring the GGC to GAC mutation in KRAS codon 13. RALA activity was also detectable in HT29 colorectal cancer cells, which are KRAS wild-type and carry a BRAF V600E mutation (Figure 1A). Transient silencing by siRNA reduced RALA mRNA expression from 77% (HCT116) to 95% (HT29) compared to both mock and scrambled siRNA transfection controls (Figure 1B). Reduced RALA expression resulted in strongly reduced GTP-binding in all three cell lines (Figure 1C).

**Figure 2:**
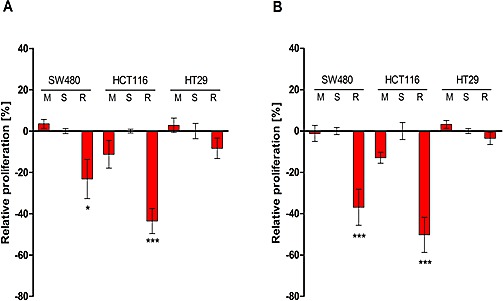
Effects of the anchorage-dependent **A.** and anchorage-independent **B.** growth of SW480, HCT116 and HT29 cells 48 h after treatment with scrambled siRNA-duplex (S), transfection reagents only (M) and RALA specific siRNA (R) determined by colorimetric XTT assays. The values were normalized in percent to S and the differences to S were calculated. Mean +/− SEM of three independent biological experiments with three technical replicas each is shown. *p ≤ 0.05; ***p ≤ 0.001.

Next we analyzed the impact of RALA silencing on anchorage-dependent and independent growth of the colorectal cancer cells. The proliferation of the two KRAS mutated cell lines was significantly reduced in both culture systems as compared to controls (Figure 2). BRAF mutated HT29 cells did not show any significant growth reduction following treatment with RALA siRNA. However, cell cycle analysis of HT-29 cells showed a slight increase in the sub-G1 peak on DNA histograms (Supplementary Figure 1), suggesting that the RALA pathway plays a minor role in cell survival. The migratory potential determined by scratch assays was highest in HCT116 cells as compared to SW480 and HT29 cells. Knock-down of RALA had no significant effect, indicating that this pathway does not significantly modulate cell migration under the conditions used for the three cell lines *(results not shown).*

### Pathway-restricted patterns of gene expression

**Figure 3:**
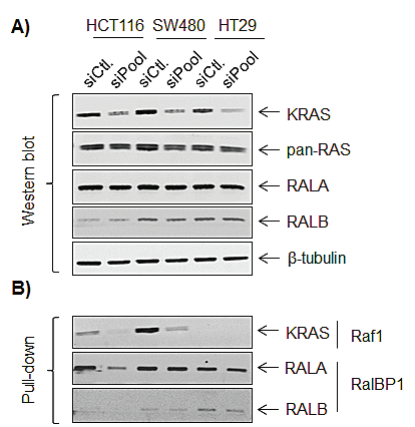
Effects of KRAS signaling on RALA and RALB expression and activity Western blot after KRAS siRNA **A.** and Western blot after pull-down **B.**. siCtl: control siRNA; siPool: set of four KRAS siRNAs (Dharmacon). Raf1: substrate of KRAS pull-down and RalBP1: substrates for RALA pull-down.

We have previously established pathway-specific gene expression signatures for MAPK and PIK3CA, suggesting a modular response of the transcriptome to oncogenic pathway activation [25, 26]. Since the RAL pathway is another important downstream branch of the RAS signaling system, we reasoned that there also exists a RAL-pathway responsive signature [9]. To confirm the impact of active KRAS on the activity of the RAL pathway, we silenced KRAS expression in the three colorectal cell lines. A direct link between KRAS and RALA activity was demonstrated in HCT116 cells, whereas RALA activity was not impaired in SW480 and HT29 cell lines after KRAS knockdown (Figure 3). This suggests that RALA activation is independent on upstream KRAS signaling in these two cell lines.

**Figure 4:**
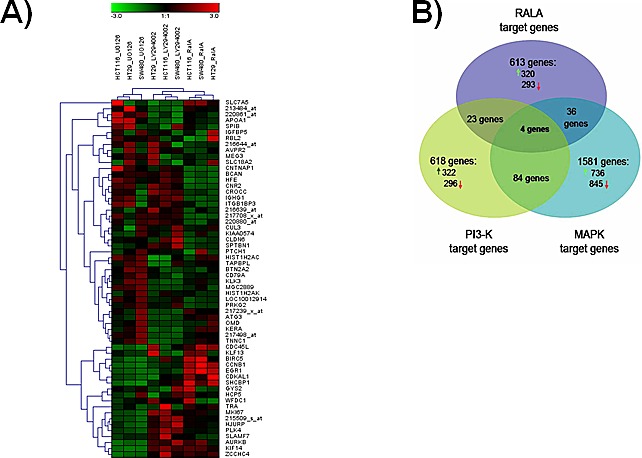
RAL pathway-restricted gene expression in colorectal cancer cell lines **A.** Euclidean distance clustering of 20 top genes (having the lowest p value in the class comparison) responding to one of the three pathways (total number = 60). Clustering indicates that the similarity of the pathway-responsive target gene pattern exceeds the differences between cell lines. **B.** Venn diagram showing the overall number of genes up-regulated and down-regulated due to inhibition of the MAPK, PI3K and RAL pathway, respectively, in three colorectal cancer cell lines. The MAPK pathway was inhibited by treating cells with the MEK inhibitor U0126, the PI3K pathway with LY294002 and the RAL pathway with a specific siRNA. Quality control of microarray data is summarized in Supplemental Table 1. The complete normalized dataset including all cell line arrays is available in Supplemental Table 2. The list of 613 genes regulated by the RAL-pathway is depicted in Supplemental Table 3. The list of 554 genes regulated by the RAL pathway only is listed in Supplemental Table 4. The raw data is accessible in GEO by the accession number GSE39857.

To determine the impact of the RALA pathway on gene expression, we interrogated Affymetrix microarrays using RNA prepared from cell lines, in which RALA expression was transiently silenced. Cells treated with scrambled siRNA were used as controls. Normalized gene expression for each sample are available in Supplemental Table 2. We compared the RALA pathway-dependent expression profiles with MAPK-and PI3K-regulated gene sets identified previously [25, 26]. Hierarchical clustering of the 20 top genes regulated by the RALA, MAPK or PI3K pathways revealed a significant difference between the pathway-restricted gene sets, particularly when considering marked differences between the individual cell lines (Figure 4A). Altogether, 613 genes were regulated by the RAL pathway (see Supplemental Table 3.). Of the RAL pathway-target genes, 89.7% were distinct from MAPK- and PI3K-pathway responsive targets (Figure 4B). Hence, the RALA pathway-responsive gene signature identified in the three colorectal cancer cell lines comprises 554 genes, irrespective of the mutational status of KRAS or BRAF. The list of these 554 genes regulated by the RAL pathway only is depicted in Supplemental Table 4. The genes encoding the inter-alpha-trypsin inhibitor heavy chain 5 (ITIH5), contactin-associated protein-like 2 (CNTNAP2), trafficking Protein particle complex subunit 6A (TRAPPC6A) and lipocalin-2 (LCN2) were the only ones commonly regulated by RAL, PI3K and RAF/MAPK pathways.

### Correlation between the RALA signature and clinical outcome

**Figure 5:**
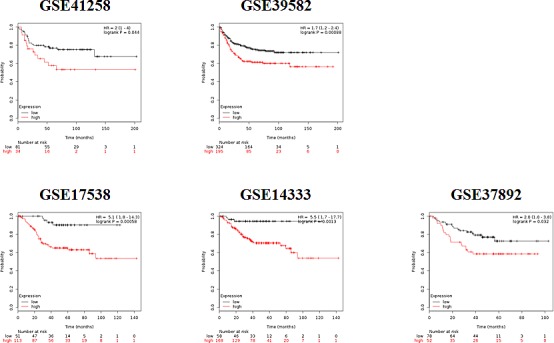
Kaplan-Meier survival plots derived by employing the signature of RALA responsive genes in five independent colon cancer datasets shows worse prognosis for those patients where a higher expression of RALA signature was observed

In view of the previously investigated role of RAL signaling in proliferation and malignant properties of cancer cells, we analyzed the correlation of RAL pathway-regulated genes with progression-free survival of colorectal cancer patients. We retrieved five independent datasets, in which gene expression profiles of tumors and clinical follow-up of patients were documented (Table 1). Patient tumors characterized by a higher expression of the RALA signature had a shorter relapse-free survival in each dataset (GSE41258: HR = 2, *p* = 0.044; GSE1433: HR = 5.5, *p* = 0.0013; GSE17538: HR = 5.1, *p* = 0.00058; GSE37892: HR = 2, *p* = 0.032; GSE39582: HR = 1.7, *p* = 0.00088). At the same time, the expression signature derived using the MEK inhibitor UO126 was not significant, and the signature established by the PI3K inhibitor LY2904 was only significant in three out of five dataset (GSE17538: HR = 2.7, *p* = 0.0097; GSE14333: HR = 2.5, *p* = 0.0074; GSE39582: HR = 1.8, *p* = 0.00048). Kaplan-Meier survival plots for the RALA signature in each dataset are displayed in Figure 5.

**Table 1:** Datasets used in the independent clinical validation of the RALA signature

GEO dataset ID	GEO platform ID	Samples with RFS	Mean follow-up (months)	Reference
GSE14333	GPL570	255	43.5	[48]
GSE17538	GPL570	164	47.2	[49]
GSE37892	GPL570	130	41.8	[50]
GSE39582	GPL570	560	48.6	[51]
GSE41258	GPL96	118	66.4	[52]

**Figure 6:**
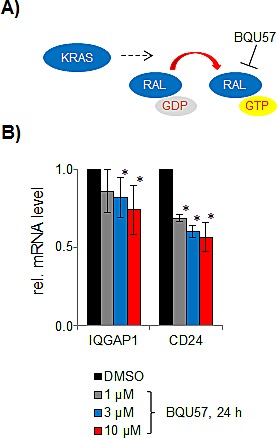
Effect of the RALA inhibitor BQU57 **A.** on mRNA expression of IQGAP1 and CD24 in SW480 cells **B.**

One of the RALA pathway dependent transcriptional targets, IQGAP1, encodes a multifunctional scaffold protein that interacts with various signaling proteins including MAP kinases. RAL pathway-dependent regulation of IQGAP1 potentially enables a feedback between RALA and MAPK signaling [29]. To support this link, we have validated the effect of RALA inhibition on IQGAP1 expression (Figure 6).

Colon cancer subtype classification including the chromosomal instable (colon cancer subtype 1 – CSS1), the microsatellite-instable (CSS2) and the sessile-serrated adenoma subtype (CSS3) was available for 326 patients for two datasets as published previously [30]. Of the genes included in the RALA signature, 31% had highest expression in the CSS1 subtype, 29% had highest expression in the CSS2 subtype and 40% had highest expression in the CSS3 subtype. There was no significant association between RALA signature and colon cancer molecular subtypes.

## DISCUSSION

The RALA pathway is robustly active in colorectal cancer cell lines harboring KRAS or BRAF driver mutations. Silencing of RALA expression resulted in growth reduction of KRAS-mutated SW40 and HCT116 cells, but not in BRAF-mutated HT-29 cells. This suggests that the RAL pathway contributed to the proliferative potential of cells, in which effector activation is triggered by GTP-bound KRAS proteins. Diminishing GTPase activity by RALA knock-down in HT-29 cells had no significant effect on proliferation supporting the notion that proliferation is mainly triggered by RAF/MAPK signaling. The migratory potential of these colorectal cancer cell lines was not affected by knocking-down RALA expression in contrast to other types of cancer cells [31]. This suggests that the migratory potential requires mechanisms controlled by other effector pathways.

Gene expression profiling of RALA pathway-depleted cells permitted identification of specific transcriptional targets. Overall, we recovered 554 genes responding to the RAL pathway but not to the RAF/MAPK and PI3K pathways. This finding supports the notion that the overall transcriptional response triggered by the RTK/RAS signaling system is delimited by individual effector pathways downstream of RAS and is essentially modular [32]. Surprisingly, we uncovered a large overlap of RAL pathway-responsive genes in the cell lines regardless of the expressed driver mutation in KRAS or BRAF. In the absence of mutated KRAS, the RAL pathway is likely to be indirectly activated by wild-type RAS proteins. It is well known that the RAF pathway stimulates expression of heparin-binding epidermal growth factor (HB-EGF) [33], which may then activate all downstream effector pathways via RAS as the central switch. The feedback from RAF to EGFR [34, 35] provides a mechanistic explanation for RALA activation caused by upstream alterations in EGFR or RAF genes.

We used RALA specific target genes that were commonly deregulated in all three cell lines for analysis of their correlation with progression-free survival in five independent colorectal cancer datasets involving 1,227 colorectal cancer patients, for whom data on relapse-free survival were reported. Using this strategy we were able to robustly uncover the correlation between survival and the RALA signature. Importantly, when compared to PI3K and MEK inhibition, the RALA signature was the only one significantly correlated to survival in each of the investigated datasets. Our results support the function of the RALA pathway as an additional collateral pathway further driving tumor progression. This suggest the potential of future therapeutic applications blocking the RALA pathway in addition to suppression of other effector pathways downstream of RAS, e.g. in patients receiving systemic therapies targeting RAF, ERK or PI3K.

The overall complexity of the RALA pathway-related signature requires further analysis of individual target genes in a systematic way in order to elucidate their functional contribution to cellular phenotypes and clinical features. Notably, there is growing evidence for cross-talk with other critical signaling pathways [36]. The down-regulation of the IQGAP1 gene encoding IQ motif containing GTPase activating protein homologue 1 upon RALA inhibition provides a likely example for an indirect interaction via transcriptional regulation and implies the RALA signaling pathway as an upstream regulator of MAPK signaling. The IQGAP protein family comprises three scaffold proteins, of which IQGAP1 is best characterized and known to interact with RAF, MEK and ERK and to be up-regulated in cancer [37]. In addition, IQGAP1 was shown to be correlated with invasion and poor prognosis [38]. Mice deficient in IQGAP were refractory to HRAS-driven carcinogenesis and depletion of IQGAP1 reduced invasion in RAS oncogene-driven cancer cells by suppression of ERK activity [29]. The interaction of ERK and IQGAP1, which is mediated via the WW domain of the scaffold, has emerged as a novel therapeutic target [37]. Moreover, in addition to the MAPK pathway IQGAP1 may modulate other oncogenic pathways by binding to E-cadherin and beta-catenin [39] and by regulating the activation state of Rho A/Rho C to promote breast cancer cell proliferation and migration [40].

Three independent colon cancer subtypes have been recently identified: the first subtype (CSS1) includes chromosomal-instable tumors, the second subtype include microsatellite-instable cancer, and tumors in the third subtype exhibit a very unfavorable prognosis and are refractory to epidermal growth factor receptor-targeted therapy [30]. Our results support the independence of the RALA pathway from the molecular subtypes thereby emphasizing the importance of RALA as an independent factor in colon cancer pathogenesis.

In conclusion, the RALA pathway impinges on the transcription of a distinct subset of target genes in colorectal cancer cells independent of the KRAS and BRAF mutational status. RALA pathway-responsive genes were unaffected by RAF/MAPK and PI3K signaling. In view of the correlation of RAL pathway-responsive genes and patient survival, further exploitation of therapeutic approaches [17] involving monotherapy as well as combinatorial therapies against non-mutated signaling proteins [41] are warranted.

## MATERIALS AND METHODS

### Cell culture

The colorectal cancer cell lines SW480, HCT116 and HT29 obtained from the ATCC. The cell lines were maintained at 37°C and 5% CO_2_ in a humidified incubator in D10-medium containing DMEM (Invitrogen) supplemented with 10% fetal bovine serum (Sigma-Aldrich), 2 mM ultraglutamine (Lonza, BioWhittaker), and 100 units/ml penicillin/streptomycin (Biochrom AG).

### KRAS and RALA activation assay

The activation states of KRAS and RALA proteins were analyzed by using GTPase Pull-down and Detection kits (RAL: STA-408-CB and pan-RAS: STA-400-CB) based on specific downstream effectors fused to GST, which bind to the active form of GTPases (Cell Biolabs). Cell protein lysates (500 μg) were incubated with 50 μl glutathione resin and GST protein binding domains for 1h to capture active small GTPases according to the manufacturer`s protocol. After washing, the bound GTPase was recovered by eluting the GST-fusion protein from the glutathione resin. The purified GTPase was detected by Western blot using specific antibodies supplied in the kit. Positive and negative controls were generated using GTPγS (0.1 mM) and GDP (1 mM) (Thermo Fisher Scientific).

### Transient transfections

One day before transfection 3 × 10^5^ HCT116 and SW480 as well as 1.5 × 10^5^ HT29 cells were plated in 10 cm dishes (BD Falcon) with D10-medium. SiRNA transfections were done twice in 24 h-intervals using Lipofectamine RNAiMAX (Invitrogen) for HT29 and HCT116 cell lines and Lipofectamine 2000 (Invitrogen) for SW480 cells according to the manufacturer's specifications. The following oligonucleotides were used to synthesize siRNAs using the Silencer^®^ siRNA Construction Kit (Applied Biosystems) according to manufacturer's specifications: RalA siRNA (1) : sense sequence: *5′-AGACAGGTTTCTGTAG AAGACCTGTCTC-3′;* antisense sequence: *5′-AACAGAGCTGAGCAGTGGAATCCTGTCTC-3′* [27]. RalA siRNA (2): sense sequence *5′-AACTAAGATA TCGATCTGGACCCTGTCTC-3′,* antisense sequence: *5′-AAGTCCAGATCGATA TCTTAGCCTGTCTC-3′;* non-targeted scrambled oligonucleotide for SW480 cells: sense sequence: *5′-AACGCGAGCTCGTGCGAGGGTCCTGTCTC-3′*, antisense sequence: *5′-AAACCCTCGCACGAGCTCCGCCCTGTCTC-3′*. Synthesized siRNAs were transfected at a final concentration of 1.5 nM. We used an alternative scrambled siRNA control for HT29 and HCT116 cells lines (silencer negative control no.1, AM4611, Applied Biosystems) at a final concentration of 60 nM. In addition, cells were transfected with transfection reagents only (Mock control). For KRAS knockdown we used On-target KRAS siRNA LQ-0050069-00-0005 purchased from Dharmacon (Lafayette, CO, USA).

### Inhibition of RALA by BQU57

The RALA inhibitor BQU57 [42] was purchased from Apexbio Ltd (Boston, USA). BQU57 was used in concentrations of 1 μM, 3 μM and 10 μM for 24 hours. To measure the effect of RALA inhibition on IQGAP1, RT-PCR was performed using the forward primer 5′-GCCAAGATGTATCTACTGTATCC-3′ and the reverse primer 3′-TCTGTGAAGTCAACCTTTCC-5′. The established RALA target CD24 [43] was used as internal control. For CD24, the forward primer 5′-CTACCCACGCAGATTTATTCC-3′ and reverse primer 3′-TGGCATTAGTTGGATTTGGG-5′ were used in RT-PCR.

### RNA isolation, cDNA synthesis and quantitative real-time RT-PCR analysis

Total RNA isolation and purification was performed using Qiagen RNeasy Mini Kit with QIAshredder according to the manufacturer's protocol. RNA concentration and integrity were analyzed spectrophotometrically with a NanoPhotometer (Implen) at a wavelength of 260 nm and 280 nm, respectively. RNA purity was assessed by determining the ratio of OD_260_/OD_280_ and was within the range of 1.8 to 2.0.

cDNA synthesis was performed with 50 ng/μl of total RNA per sample using TaqMan^®^ Reverse Transcription Reagents (Applied Biosystems) in a Techne Progene Thermal Cycler according to the manufacturer's protocol. Real-time RT-PCR reactions were performed in a StepOnePlus Real Time PCR System (Applied Biosystems) using TaqMan Gene Expression Master Mix (Applied Biosystems). Micro Amp Fast Optical 96-Well Reaction Plates and the following TaqMan gene expression assays were used: RALA (HS00800233_S1), UBE2D2 (HS003666152_m1) as endogenous control for HCT116 cells and Beta-actin (HS9999903_m1) as endogenous control for SW480 and HT29 cell lines. Relative transcript levels were determined by calculating 2deltaCt values.

### Proliferation assays

Cell proliferation was monitored semi-quantitatively in a sodium 3′ [1-(phenylyamino-carbonyl)-3, 4-tetrazolium]-bis-(4-methoxy-6-nitro)-benzene sulfonic acid hydrate (XTT)-based colorimetric assay using the cell proliferation kit II (Roche). To estimate anchorage-dependent and anchorage-independent growth, 5000 cells/well were seeded into untreated 96-well plates (BD Falcon) in a volume of 150 μl medium or into poly-HEMA coated 96-well plates, respectively. For the preparation of poly-HEMA coated 96-well plates, 100 υl of a 5 mg/ml stock solution of poly-HEMA (Sigma-Aldrich) dissolved in 96% ethanol by mixing at room temperature for 4 h were added to the wells and allowed to dry for 72 h at 37°C. Growth of cells was determined in triplicate experiments 48 h after the transient knock-down. Formazan formation was determined spectrophotometrically. The optical density of the medium without cells was subtracted from all probe values to obtain the final results. The statistical significance was calculated using ANOVA and Tukey's post hoc tests.

### SDS-Page and western blotting

Protein concentration was measured by staining with amido black assay (Merck) according to the manufacturer's specifications. Per sample, 50 μg of proteins and 5 μl of PageRuler Prestained Protein Ladder (SM0671, Fermentas) were fractionated by electrophoresis through 12% SDS-polyacrylamide gels (SDS-PAGE) and transferred to Protran nitrocellulose membranes (Whatman) by semi-dry blotting using Tris-base, Glycerol, (Merck), 10 % SDS, methanol (J.T.Baker, The Netherlands) in double-distilled water for 30 min with a constant current at 100 mA per membrane. The membranes were blocked with 5% non-fat dry milk (AppliChem GmbH) in TBST (Tris-buffered saline (TBS) + 0.05% Tween^®^-20, Serva) or with 3% BSA in TBST (Sigma-Aldrich, München). For the detection of active RAL and RAS proteins, we used the following primary mouse monoclonal antibodies: RALA included in STA-408-CB and pan-RAS included in STA-400-CB pull-down assays, RALB: #3523, KRAS: #2146 and beta-tubulin: #TA801672 (Cell Signaling). Anti-mouse IgG-Horseradish Peroxidase (HRP)-conjugate (1:5000 dilution, 325-035-045, Dianova) or anti-rabbit IgG-HRP-conjugate (1:10, 000 dilution; 7074, Cell Signaling) were used as secondary antibody. Protein bands were visualized using the ECL chemoluminescence detection system (GE Health Care) on X-ray films (Amersham Hyperfilm e).

### Scratch assays

To distinguish between migration and cell proliferation, we performed experiments with serum reduced medium (FCS concentrations between 0.1%-1%). The optimal FCS concentrations for migration assays were determined by XTT assays. Cells ceased proliferation 29 h after reduction of FCS concentrations 0.1% for SW480 and HT29 cells as well as to 0.5% for HCT116 cells. Prior to the scratch assay, cells were transiently transfected twice with siRNA in 10 cm dishes (BD Falcon). One day after the second transfection, 5 × 10^5^ HT29 cells, 2.1 × 10^5^ HCT116 cells or 6.7 × 10^5^ SW480 cells were plated into 12-well plates (BD Falcon) containing medium with 10% FCS. Under these conditions, the cells attached to form confluent monolayers. Then, cells were washed with serum-free medium to remove residual FCS and media with reduced serum were added. The monolayers were wounded 29 h later by scratching cells off the dish using a 100 μl plastic pipette tip for HCT116 and HT29 cells and 10 μl plastic tips for SW480 cells. To remove non-adherent cells, the wound was gently washed with serum free medium. Cell migration was documented using a digital microscope (Keyence BZ-8000, Keyence) at 40-fold magnification after defined time intervals for up to 96 h after wounding. The wound area in pixels was determined using ImageJ software (http://rsweb.nih.gov/ij/). The quantification was calculated using the following formula: (area of fresh wound - area after × hours) / × hours = pixels/hour.

### Cell cycle analysis

Trypsinized cells were denatured in cold 70 % ethanol overnight, pelleted by centrifugation and resuspended in PBS supplemented with 0.1 % TritonX / 0.5 % BSA and incubated with 10 mg/ml RNase at 37°C for 30 min. After centrifugation, the pellet was resuspended in dilution buffer and incubated with 20 μg/ml propidium iodide at room temperature in the dark for 20 min. DNA histograms were recorded in a FACSCalibur flow cytometer (BD Biosciences). The raw data were analyzed with the programs Cylchred and WinMDI 2.8 (Joseph Trotter, San Diego, USA).

### Whole genome transcriptome analysis

Transcriptional profiles were obtained by interrogating human whole genome arrays U133 2.0 (Affymetrix Inc.) with total RNA isolated 48 h after RALA siRNA transfer. To compare the RAL pathway-dependent profiles with those regulated by MAPK- and PI3K-signaling, we used raw expression data of GSE18232 and GSE18005 [25, 26] deposited in GEO.

The microarrays were MAS 5.0 normalized in the R statistical environment (http://www.R-project.org) using the Bioconductor package Affy (http://www.bioconductor.org). MAS 5.0 performed as one of the best among various normalization methods when using RT-PCR-based validation measurements of microarray data [44]. To eliminate the effects of different instrument-default settings for average expression on the GPL96 and GPL570 platforms, a second scaling normalization was performed on the matched gene set to adjust the average expression of each array to 1,000. Our data can be accessed in GEO using the accession number GSE39857.

As basic quality assessment of the Affymetrix microarrays, we measured the eight parameters according to the Affymetrix Whitepapers (http://www.affymetrix.com/support/technical/whitepapers.affx). We tested their method using an extended version of our previously published database [45]. The distribution of the arrays was assessed and outliers were identified as those having a parameter value outside of the range of 95% of samples. Detailed results for each sample are listed in Supplemental Table 1. For probe set quality control, we used Jetset [46], a tool that allows assessment of each probe set for specificity, splice isoform coverage, and robustness against transcript degradation.

### Feature selection and clustering

Normalized gene expression data were imported into BRB-ArrayTools 3.8.1 (developed by Dr. Richard Simon and Amy Peng Lam, http://linus.nci.nih.gov/BRB-ArrayTools.html). Intensity thresholding at the minimum value was performed, if the spot intensity was below the minimum value of 10. If less than 20 % of expression data had at least a 1.5-fold change in either direction from the gene's median value or the percent of data missing or filtered out exceeded 50%, the gene was discarded. Altogether 19, 961 probe sets passed the filtering criteria. Finally, probe sets with a median expression below 500 (MAS 5.0 unit) were excluded from the analysis.

In the next step, class comparison using paired t-test was performed to compare treated and control cell lines. Only probe sets with a minimum fold change of 2 between the two investigated groups were included. Finally, genes differentially expressed in the control samples (scrambled siRNA transfection and mock transfection, respectively) were excluded from the list of regulated genes. The significance threshold was set to 0.05.

Hierarchical clustering was performed using the Genesis software (http://genome.tugraz.at/genesisclient/genesisclient_description.shtml). In all analyses, average linkage clustering was computed for the experiments and genes. For visualization, the gene expression values were centered on the mean for each gene.

### GEO search for colon cancer samples

Colon cancer gene expression datasets with survival were identified in GEO using the search keywords “colon”, “cancer”, “gpl96”, and “gpl570” (http://www.ncbi.nlm.nih.gov/geo/). Only publications with available raw data, clinical survival information, and containing at least 100 patients were included. We considered only data obtained with Affymetrix HG-U133A (GPL96) and HG-U133 Plus 2.0 (GPL570) microarrays, because of their frequent application and the common representation of 22,277 probe sets.

### Meta-analysis of the colon cancer datasets

The raw .CEL files were processed as described above. The mean expression of all genes having a significance below 0.01 in siRNA-treated cells was designated as a RALA-signature. The package “survival” was used to calculate and plot Kaplan-Meier survival curves, hazard ratio (and 95% confidence intervals) and logrank P in each dataset separately as described previously [47]. The same analysis was also performed using the previously published PI3K and MEK signatures [25, 26].

To assess the correlations between RALA regulated genes and colon cancer subtypes, we used the samples in the clinical database according to their assignment to colon cancer subtypes as described in the original publication [30]. The correlation of all RALA target genes with colorectal cancer subtypes was assessed by comparing the number of genes showing the highest expression in each subtype by a chi-square test. Significance was set at *p* < 0.01.

## SUPPLEMENTARY MATERIALS, FIGURES, TABLES




