# Supplementary material for: Effects of RAL signal transduction in KRAS- and BRAF-mutated cells and prognostic potential of the RAL signature in colorectal cancer
Source: Oncotarget. 2015 Apr 19;6(15):13334–46. doi: 10.18632/oncotarget.3871 (PMC4537018; doi:10.18632/oncotarget.3871)
Supplement: Supplementary file 1 [file oncotarget-06-13334-s001.pdf]

Effects of RAL signal transduction in KRAS- and BRAF-mutated cells and prognostic potential of the RAL signature in colorectal cancer

Supplementary Material

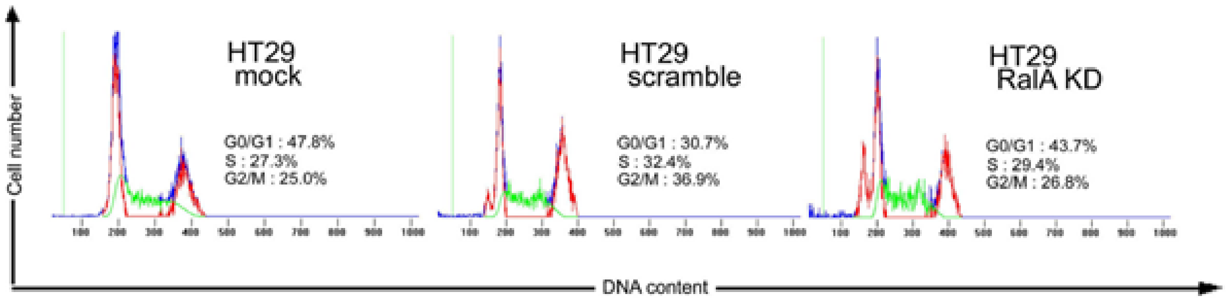

Supplementary Figure 1: RALA-pathway is important for the anti-apoptotic function in BRAF mutated cells. Effects on distribution of cell cycle phases determined by FACS analysis in propidium iodide treated HT29 cells 48 h after silencing of RALA (RALA KD) and treatment with scrambled siRNA-duplex (scramble), transfection reagents only (mock). Overall, in BRAF-mutated HT29-cells the number of SubG1-phase (apopototic cells) is increased. The amount of apoptotic cells 48 h after treatment of the HT29 cell line with RALA specific siRNA or non-sense siRNA (scramble) in percentage of the amount of total cells measured in the analysis was significant ($p<0.05$). These data indicate an anti-apoptotic function of RALA only in BRAF mutated cells. *G0/G1: Gap 0 or Gap 1 phases; S: synthesis phase; G2/M: Gap 2 or mitosis phases. One example of three independent experiments (biological replicas) is shown.*
