# Supplementary material for: Effects of RAL signal transduction in KRAS- and BRAF-mutated cells and prognostic potential of the RAL signature in colorectal cancer
Source: Oncotarget. 2015 Apr 19;6(15):13334–46. doi: 10.18632/oncotarget.3871 (PMC4537018; doi:10.18632/oncotarget.3871)
Supplement: Supplementary file 2 [file oncotarget-06-13334-s002.pdf]

	Background	Raw Q (Noise)	Scaling factor	percent present calls	bioB-, bioC-, bioD-spikes	GAPDH 3 to 5 ratio	Beta-actin 3 to 5 ratio
IST_11_SW480_Mock.CEL	55,89	3,212	2,666	43,03	OK	0,932	1,13
IST_12_SW480_Sc.CEL	39,56	2,102	5,133	39,86	OK	0,89	1,362
IST_13_SW480_RalA_KD	32	1,32	5,631	40,43	OK	1,32	3,138
IST_14_HCT116_Mock.CEL	35,45	1,484	4,591	40,61	OK	1,188	2,533
IST_15_HCT116_Sc.CEL	35,63	1,616	5,225	39,83	OK	1,095	2,504
IST_16_HCT116_RalA_KD.CEL	33,9	1,385	5,988	39,84	OK	1,111	1,769
IST_17_HT29_Mock.CEL	35,78	1,959	2,241	44,27	OK	1,084	2,032
IST_18_HT29_Sc.CEL	44,83	2,383	3,615	43,62	OK	1,22	3,88
IST_19_HT29_RalA_KD.CEL	40,46	2,18	2,869	44,41	OK	1,167	1,97
KJ_HCT116_Kontrolle.CEL	72,22	3,263	3,972	47,53	OK	0,885	1,043
KJ_HCT116_U0126.CEL	63,5	2,251	4,775	48,77	OK	1,267	1,154
KJ_HT29_Kontrolle.CEL	87,1	3,559	3,15	47,57	OK	0,895	1,061
KJ_HT29_UO126.CEL	80,23	3,098	4,34	47,34	OK	0,971	1,311
KJ_SW480_Kontrolle.CEL	76,36	3,912	4,937	37,67	OK	1,063	1,419
KJ_SW480_UO126.CEL	93,92	3,446	8,371	35,79	OK	0,866	1,883
TK_HCT116_DMSO_09.CEL	82,52	3,897	2,318	49,19	OK	0,813	1,008
TK_HCT116_LY294002_10.CEL	83,77	4,286	2,186	49,98	OK	0,88	1,027
TK_HT29_DMSO_04.CEL	97,05	5,303	1,755	48,69	OK	0,84	1,093
TK_HT29_LY294002.11_05.CEL	81,37	4,464	1,723	49,87	OK	0,947	1,114
TK_SW480_DMSO.11_01.CEL	98,4	5,506	1,395	52,23	OK	0,912	1,002
TK_SW480_LY294002_14.CEL	92,88	4,524	2,483	48,62	OK	1,013	1,349

AffyID	KJ_HCT116_Kontrolle	KJ_HCT116_U0126	KJ_HT29_Kontrolle	KJ_HT29_UO126	KJ_SW480_Kontrolle	KJ_SW480_UO126	TK_HCT116_DMSO_09	TK_HCT116_LY294002_10	TK_HT29_DMSO_04	TK_HT29_LY294002	TK_SW480_DMSO	TK_SW480_LY294002_14	IST_11_SW480_Mock	IST_12_SW480_Sc	IST_13_SW480_RalA_KD	IST_14_HCT116_Mock	IST_15_HCT116_Sc	IST_16_HCT116_RalA_KD	IST_17_HT29_Mock	IST_18_HT29_Sc	IST_19_HT29_RalA_KD
1007_s_at	4401	5484	1879	3083	3212	5264	3192	3292	1646	2718	3276	2730	1608	1221	1067	1827	1826	1557	1090	1343	1108
1053_at	958	483	673	330	1410	509	563	946	1013	593	878	1068	1958	1585	790	480	448	489	522	279	292
117_at	115	109	179	168	459	261	273	215	177	102	131	90	31	35	5	44	37	44	38	7	27
121_at	1720	1446	1747	1595	1860	2725	1579	1871	1664	1899	1939	1874	428	571	719	687	567	614	550	573	463
1255_g_at	64	9	47	52	55	13	68	106	98	75	64	80	17	14	12	2	14	4	3	5	5
1294_at	388	467	282	387	254	247	261	281	178	171	229	307	79	112	45	48	45	60	25	69	59
1316_at	161	175	171	185	274	152	158	183	142	172	153	143	71	45	45	59	60	66	50	46	39
1320_at	33	55	128	167	33	77	127	139	35	110	139	132	37	9	2	1	6	5	6	30	35
1405_i_at	9	4	14	14	12	24	7	9	9	8	9	5	2	2	2	9	25	178	43	114	163
1431_at	71	52	69	80	27	22	82	57	62	56	54	67	10	17	44	24	29	17	29	22	28
1438_at	136	32	699	194	45	51	136	199	777	760	489	232	65	34	31	4	7	35	67	66	64
1487_at	1000	850	1928	1174	837	1110	1140	756	1626	1379	1025	703	446	389	392	358	392	309	530	899	384
1494_f_at	384	328	391	570	220	132	415	381	336	425	395	378	49	55	54	51	66	69	60	62	52
1598_g_at	1987	1122	636	597	1847	1743	2478	2695	836	813	3516	3082	1049	1504	1269	591	979	774	269	498	478
160020_at	712	661	894	835	1278	1783	519	579	759	692	1042	780	354	286	367	146	196	58	379	192	152
1729_at	446	744	450	841	525	816	315	505	433	688	395	294	325	264	208	208	315	183	362	412	288
177_at	198	157	123	410	399	329	215	156	122	280	102	255	33	35	30	17	33	30	70	74	119
1773_at	329	370	283	226	191	58	424	375	211	238	281	123	92	91	29	140	95	150	124	56	114
179_at	1435	1401	1883	7200	1027	1064	1518	1322	2006	2200	1383	1127	209	47	195	256	285	159	381	289	221
1861_at	366	282	308	404	520	571	309	350	322	289	489	375	400	301	196	178	195	139	172	181	103
200000_s_at	5043	2913	3927	3992	2720	2443	4468	4712	3911	3420	4441	3246	2143	1937	1782	2131	1916	2601	2608	2813	2975
200001_at	8923	7664	8116	7902	5983	7590	9889	8263	9505	8163	9813	6868	7971	8368	6747	4896	4955	2972	3726	4510	2164
200002_at	15898	12504	14800	15188	15697	15212	16459	16433	16831	15919	17042	23177	18195	20425	13234	11774	14803	12747	11336	14113	8612

200003_s_at	23198	35012	20755	28478	20680	25650	21178	24328	19947	22381	19273	29531	26113	27891	20892	21507	21114	19119	15323	26277	16951
200004_at	9355	8704	13577	10039	17900	15438	11403	10987	12827	11417	13395	13386	18476	18351	18877	17401	15584	18953	17027	16269	19576
200005_at	7090	5890	4210	3545	3927	4354	6514	6327	4185	4085	5302	4957	7084	5228	4099	5645	6542	4460	3623	3308	3491
200006_at	12491	11306	10431	8878	14251	15984	12684	9951	9456	8706	10588	13675	13314	12006	13842	14186	13781	12663	12483	12013	8982
200007_at	11504	9103	9688	10492	7149	8017	8261	8912	8592	7959	6637	7287	7202	5699	7794	10372	10188	10782	8682	9002	8183
200008_s_at	2176	1544	5001	3511	3274	2293	3457	3135	5789	5568	4474	3594	2362	2146	1697	1521	1250	1320	2908	2038	2189
200009_at	4855	3932	9677	6915	6952	4333	6226	5215	11070	9828	8398	7593	7539	7700	8596	4833	4681	4033	8353	9611	7932
200010_at	11803	14972	11671	14397	10717	16353	19505	16162	12493	13546	15404	15261	18314	17665	13960	17736	17825	16738	13681	17459	13981
200011_s_at	2933	3214	4735	4170	2568	2064	4100	3441	5060	5382	5521	3803	3571	3153	3494	3302	3111	2136	3745	3143	2913
200012_x_at	11533	20596	16181	18920	15958	19523	18769	17649	20850	14838	15662	25711	16968	24764	23664	21060	22576	23827	14677	21540	18233
200013_at	12762	16115	18383	21147	13261	16446	19598	17791	22302	18855	13250	18684	21896	23280	14315	18039	17658	15526	19219	18848	12215
200014_s_at	4298	3848	3912	2479	4580	4963	4987	4079	3533	2697	3061	2767	7297	7402	8162	10556	9425	11214	9238	8426	9430
200015_s_at	5301	5044	4181	4727	4672	4263	5863	5628	4321	5237	3652	4472	6077	5729	5597	8800	8842	6970	7644	8642	9211
200016_x_at	19052	11780	15272	14811	19243	16029	16942	19323	14958	16443	15687	18340	20959	26274	26221	25932	24772	25823	18117	24664	23627
200017_at	16948	26406	18582	21624	24626	28465	20612	18548	21768	19544	18268	23656	24264	28549	26310	28293	28403	28415	20893	26988	22211
200018_at	16139	25400	22200	28584	19782	23657	22051	24076	23549	20594	18504	25713	22534	29518	26456	24736	27648	27896	18875	26899	22752
200019_s_at	19894	13738	19456	20556	15059	13836	18037	16562	19932	14773	15942	17669	18141	19503	18639	16539	15871	15529	13912	20753	12070
200020_at	2446	1923	2916	1692	3128	1799	3966	3122	3841	3971	3940	2642	4301	4178	4806	4628	4388	4104	5177	3195	4381
200021_at	26209	33689	30026	37632	28057	14895	25793	26128	31703	28109	23575	27817	16721	21759	26712	21806	23978	24815	16777	20628	15719
200022_at	19917	30079	21159	23299	21030	24875	20534	20518	19453	21401	18976	25797	20301	26473	19532	20506	22120	22098	17297	23721	16850
200023_s_at	9651	17141	6581	10161	7987	11584	7061	7474	6097	5692	7864	9575	12768	16136	16804	15632	16858	13308	12733	17432	14312
200024_at	21593	29282	17418	24014	14703	22026	16785	17159	18139	15970	14068	22410	16471	19668	17151	17625	18567	15748	11786	19240	12511
200025_s_at	17948	25915	19720	21707	18691	24802	19063	19065	20489	19558	16266	24790	22742	25416	21458	23163	24960	24637	19271	22668	20523
200026_at	11744	24589	16033	12185	7989	11409	15268	17673	12596	12699	11038	13919	13006	13899	13376	19664	21737	20090	10621	14574	10446
200027_at	6424	9245	7309	4956	2422	4267	7126	5431	5873	4735	2238	1695	2987	2700	4906	11368	10265	10205	9417	10550	12777
200028_s_at	3461	3454	4346	4749	5504	5670	4023	4302	6912	5866	5766	4504	5756	6100	8936	6602	5783	6225	6808	5190	6665
200029_at	21454	25264	17238	25707	18926	16903	19490	20015	17219	16366	17522	22306	18437	23509	23587	23555	25388	22042	14217	22347	17182

200030_s_at	17307	23726	15771	14227	10335	11953	17709	16976	14823	11874	12839	10660	13189	15971	16988	22802	23844	22796	13200	16381	15126
200031_s_at	28295	27052	28215	37690	20062	28809	26471	24853	29932	26989	20801	30408	24812	34042	28388	29657	31204	31531	23239	32959	26031
200032_s_at	22354	29339	19401	24887	25668	37445	23950	24012	19848	17523	17455	23994	20592	26421	23557	26041	28427	28240	17911	24918	22548
200033_at	11318	10574	9478	7806	15316	9309	9314	9496	9885	8728	9273	9744	14676	14033	14553	16507	16756	15257	14977	12867	15693
200034_s_at	13403	17543	14206	16950	13707	18710	15892	16120	16031	14323	12107	17179	19632	19747	18512	20406	19734	19047	14836	20994	17990
200035_at	2092	1077	1264	1975	1685	1064	1825	1673	1878	1564	3024	1085	907	876	1062	658	669	639	894	729	632
200036_s_at	18732	15629	12865	14988	10771	13426	14982	14114	9455	9351	9748	12326	12513	14410	12108	15772	16932	15719	10050	16053	10208
200037_s_at	5925	3552	4379	3096	10159	6270	4183	4877	5269	4857	6344	6081	15656	12483	8985	7303	7367	9771	11517	9097	12486
200038_s_at	20826	27173	18117	20619	11821	17248	18732	21832	16531	14476	11382	14194	17780	20628	18985	26362	26206	27392	17290	23706	19274
200039_s_at	8550	6214	6538	4673	5516	3275	8263	7015	8052	4945	7251	4541	7735	8160	7321	7903	9580	10292	7105	4983	3523
200040_at	5988	4883	4636	4052	3658	3252	5410	5319	4443	4225	4841	3769	5768	3208	4275	4567	4074	4761	5937	4698	5250
200041_s_at	7411	4248	8376	5226	4108	3166	9617	9358	8500	7616	7343	6115	3540	5258	7960	7906	8688	8042	8280	5678	5918
200042_at	1140	1077	1997	1558	2444	2021	1685	1680	2331	2070	3081	2389	2454	2931	2909	1663	2000	1467	2347	2469	1979
200043_at	7395	6822	8372	5601	10973	7777	10319	11046	8433	6907	11313	10951	13550	13169	12531	13296	12422	15301	12101	8403	7017
200044_at	8258	6338	6545	4611	3431	2235	4671	4942	5120	4555	3611	2901	6853	5838	4999	6568	5866	6000	6142	3673	3042
200045_at	1947	1493	1913	1557	1880	894	2264	1700	2502	2038	2072	1391	1198	1340	972	976	1084	1344	1379	1271	999
200046_at	2479	1920	3079	3615	2808	3442	4391	3168	2721	3245	3627	3848	4994	4150	2879	4235	4349	3046	3460	3130	2761
200047_s_at	5341	5472	3753	3163	5326	6078	3005	4306	2768	3451	3553	4473	6740	5253	6588	7425	6020	7263	8593	7920	10466
200048_s_at	5505	8181	7432	6217	3795	5115	5043	4586	6583	6451	4920	4914	7913	7209	7258	8228	9166	9021	9946	11404	10284
200049_at	815	903	769	715	726	775	1219	1283	815	853	1131	992	625	419	420	816	812	706	674	444	602
200050_at	2065	1839	2649	1456	3226	4049	2189	2092	1742	1989	2206	1901	3283	2526	3761	5440	4183	3878	5460	4306	7936
200051_at	593	473	811	688	805	582	666	596	899	948	780	667	946	861	905	694	591	536	1218	1200	869
200052_s_at	5372	2263	4357	2193	3644	2701	5234	4467	3113	2626	3449	3192	5352	3362	1734	3511	3744	3678	2933	1768	2009
200053_at	2032	1457	1367	2178	2155	1846	1915	1937	1394	1795	1160	1278	1743	1692	928	954	1036	688	611	1004	629
200054_at	1173	1152	1184	495	2437	2891	869	525	823	707	982	953	1113	906	1445	1007	862	753	1014	1245	1060
200055_at	4849	8823	5206	7356	2895	3568	5320	4311	3600	4348	5494	3897	6779	5883	8382	4824	5317	3529	4494	4680	2066
200056_s_at	734	567	741	641	942	982	525	577	585	478	613	549	1697	1644	888	1593	1631	1871	1928	1267	1329

200057_s_at 11323 6949 10735 12699 12828 10327 12169 11147 11858 12114
11384 12573 13059 13560 14067 14422 14447 16195 13686 16753 16839
200058_s_at 6485 3090 5769 3602 3791 4565 4767 5594 5845 5323 4498
4426 4132 2488 2141 2931 2658 3292 4422 3994 4057
200059_s_at 11626 8129 11413 13374 10221 9239 11816 12812 11135 11790
12376 12123 15413 15922 11471 12640 11973 12508 9527 8114 8760
200060_s_at 6381 5810 5884 3430 5662 2950 4342 5061 5831 5863 4390
4836 4410 2806 2413 2699 2540 2746 6279 3805 3859
200061_s_at 18799 27809 21798 29067 20599 24174 23219 25219 24967 22165
17548 22752 17680 21855 21866 22070 23736 23546 14832 20763 17844
200062_s_at 24706 38331 17841 27555 15443 23310 32227 29322 19278 19088
18325 19686 20761 25075 25332 30496 32945 33843 16854 23465 20538
200063_s_at 15005 14739 17478 14326 21830 20217 15124 18230 18358 17490
16310 15425 19595 22432 26148 24285 25383 26640 17088 22406 18500
200064_at 26959 12797 15930 7668 11348 7935 29807 25591 18408 14174
15882 12361 10109 10453 11744 16625 16152 16698 8690 7818 8024
200065_s_at 10275 13752 11343 9173 6884 7118 9773 8303 10714 9238 6657
6358 7849 6199 3300 5626 7391 6973 6044 5818 5903
200066_at 1437 1466 1541 1943 2904 2162 1753 1967 1647 1659 2035
2384 1996 2152 1612 1348 1491 1091 1380 1220 1355
200067_x_at 3806 4537 2873 4079 2445 3733 2593 2970 2768 2579 2098
1981 3371 3924 5105 5415 5510 4748 5124 5900 5716
200068_s_at 9542 8174 12010 9414 8229 13905 9654 11420 11154 11179
12899 9735 10930 11135 14726 16651 16361 15744 11006 14635 15911
200069_at 1136 1040 688 462 683 642 649 879 855 732 807
765 637 422 668 929 994 856 1293 1018 1433
200070_at 771 534 848 821 1014 1127 370 818 721 862 912
971 766 575 702 730 718 553 735 784 607
200071_at 2610 1619 1423 887 1520 1559 1895 1861 1255 1177 1235
1302 3334 2574 2835 6044 3599 4880 4306 4337 5679
200072_s_at 7280 3342 4494 2351 4128 1025 6832 5600 5958 4961 5578
4684 6685 6364 4318 4339 4029 4590 4445 3250 3629
200073_s_at 6584 3541 3831 1613 5807 3446 4121 4287 3473 3136 3273
3146 8430 6885 6825 7205 6564 8620 8605 6377 6976
200074_s_at 11212 10300 9107 10366 10354 11214 10225 9427 11470 9727 9838
12654 15271 15416 11626 12220 12787 10091 9111 13865 9791
200075_s_at 1684 2260 1843 2184 1076 767 1595 1552 2407 2105 1630
1587 2137 2391 1409 1220 1488 1132 2034 1792 973
200076_s_at 2058 1853 1236 1042 1208 1122 2037 1759 894 887 1443
1195 986 950 965 1378 1211 1211 664 541 456
200077_s_at 14870 16707 12961 14869 12413 14669 12878 13986 13609 12857
12944 14877 15611 13286 12728 12196 11878 11918 11059 10638 8374
200078_s_at 3280 2893 3007 2530 2010 1962 3171 2640 2803 2778 3181
2090 4482 4240 3710 2645 3066 2853 2144 3666 2229
200079_s_at 5223 3591 7067 4105 4734 2920 7143 6331 8351 6327 5722
4420 6458 5792 7096 7562 8210 6529 8946 10199 11133
200080_s_at 10068 10891 11526 11526 11967 12914 8276 14398 8890 13695
11995 10924 16509 18901 21585 19929 23047 25931 18261 22125 25747
200081_s_at 16922 24628 21557 24977 20578 26521 26639 28333 24306 21921
17820 27492 19470 25761 25222 28515 30113 29003 16943 26449 22531
200082_s_at 16599 18590 16808 21408 17202 23838 17949 18976 19576 18257
18154 20941 22334 25358 24150 22034 23075 20546 15128 21360 15996
200083_at 3585 4621 2229 1756 3240 2803 3298 3408 2262 2281 3174
2941 3933 3982 3045 3782 3327 3170 3180 2393 2895

200084_at	4827	3264	4183	3868	5629	6708	3682	4072	3693	3339	5445	5048	8441	7877	8791	7155	7420	7209	6623	5218	7065
200085_s_at	5480	3062	3645	5162	2848	3439	3518	3606	2985	3238	4529	5728	7417	7682	5458	4854	5115	4309	3509	3794	1750
200086_s_at	8088	5471	4167	5337	3087	3748	5676	5307	4150	3685	2882	3347	5740	5136	4306	7107	6436	5507	5167	4764	2287
200087_s_at	7676	7136	7965	5162	5036	6084	7974	7573	6268	6068	4886	6186	7570	7013	9398	14537	14274	13608	12365	14759	17140
200088_x_at	31692	36352	21277	25916	24120	40736	23943	27305	19271	21195	19259	36212	21122	25552	23021	27388	27069	26366	14232	24220	17684
200089_s_at	19586	16122	15171	13036	15276	18599	12748	15451	12451	12060	9099	14360	17318	18762	17858	20257	21954	21137	18114	24383	21950
200090_at	2237	3230	2343	1646	2819	2924	2215	2476	1854	2447	2670	3247	4984	7503	6933	4990	5857	5246	3878	6915	6964
200091_s_at	9643	11561	11060	10570	11723	20726	9115	12037	7333	9706	8645	12466	17201	17607	16821	19403	19617	17703	16274	18636	13911
200092_s_at	14104	19966	20128	23985	18790	23814	14892	17492	15609	18418	12804	20070	19201	24772	22475	21779	22143	22569	18218	24785	18853
200093_s_at	7050	6989	6397	5419	5688	5496	6010	6952	4895	4316	7196	7235	12069	15169	11609	11407	10738	12208	8974	10598	7138
200094_s_at	17705	28737	12193	10701	8620	14300	15950	14930	11348	11834	11026	10612	10010	9018	8983	15874	16405	11523	7683	13586	9836
200095_x_at	38643	40507	36048	36391	29324	26876	30610	38036	29061	29505	27136	30676	24545	29692	26867	29133	31254	31266	19297	29185	22438
200096_s_at	2269	2142	2948	4811	3341	4351	2839	2714	2921	3159	4423	4881	5822	7228	2935	2179	2867	1734	2261	2181	1738
200097_s_at	5088	3786	4792	3844	3721	3640	6572	7399	5346	6351	4680	5325	7565	5871	5621	7671	7253	7331	8029	5963	7915
200098_s_at	3010	2441	3102	2627	2680	2356	2413	3060	3719	3515	2781	2828	3259	3839	4100	3827	4457	4210	5133	5254	5451
200099_s_at	22044	29153	18638	22008	19980	29765	27505	27999	17020	19585	19055	24728	24081	29518	28284	31012	32652	34180	21932	27595	26484
200593_s_at	3286	2446	3879	1950	3674	1954	4343	4443	4570	3857	4124	3471	3590	3675	3108	2880	2297	2174	3142	2087	2037
200594_x_at	7672	6679	8436	5733	8419	8816	8705	8938	7399	7931	7359	7300	8687	6516	7987	9919	7716	8262	9154	8397	7999
200595_s_at	6371	4778	3641	2903	2607	3694	4640	5237	2262	3187	2469	2470	4549	3797	4529	10154	8625	11051	9066	7592	12195
200596_s_at	2060	1573	1489	1064	647	739	2955	3274	2008	2285	1232	1117	1854	1496	957	1897	1631	2108	1241	1614	1762
200597_at	836	792	654	596	837	532	892	914	721	960	499	674	542	447	433	1108	939	1207	1286	1237	1428
200598_s_at	4883	1345	4636	1611	2478	3177	4481	5540	3400	4254	3431	2326	3399	3034	1349	4804	5906	5691	2460	3607	3850
200599_s_at	10570	7067	9903	4538	4985	7331	9581	8965	8298	7843	6430	5488	8535	6834	5285	12830	15221	14152	6895	12008	12928
200600_at	2289	1471	6752	3171	4080	3923	2698	2730	5685	9397	6154	4640	4758	4000	3020	1613	1407	1575	3839	2176	2401
200601_at	4415	5232	7684	8246	9143	5167	3907	3255	5881	9780	13969	11874	3108	5316	7819	4754	2502	3174	5569	3376	1425
200602_at	1597	2608	1043	1498	1223	3210	596	992	573	1006	1143	915	1531	1331	1677	2650	2135	1990	2949	4296	3769
200603_at	5620	4828	2866	3116	6051	5874	3975	5613	2107	2602	3833	5262	6505	5720	4364	8022	6312	7098	5367	4050	6256

200604_s_at 2223 2145 1526 1384 1795 1203 2980 3440 1429 1613 3471
2139 1220 1019 1059 1375 1286 1267 312 341 446
200605_s_at 4358 4665 2853 2756 4183 3624 6165 5953 2688 2975 4417
3723 4576 3325 4076 5742 5323 4793 2996 1968 2538
200606_at 3998 4265 3204 2433 4870 6288 3244 4836 3236 3957 4835
4316 3879 2045 4387 2562 1868 3478 5405 5932 7450
200607_s_at 1364 777 3905 2461 1350 982 2082 2655 5123 5206 1482
1387 879 668 301 511 608 574 1682 719 1449
200608_s_at 4462 2456 6408 4161 3269 2326 2355 3571 4717 4949 2287
2344 5208 3692 2381 4301 3710 4790 9773 6689 9476
200609_s_at 2922 5053 3742 2356 3445 4568 3287 3125 4367 3951 3539
3170 5452 6368 7628 6388 6649 7360 6494 4768 5687
200610_s_at 15207 6786 12216 5747 11775 6229 11771 12531 14120 12475
10986 9419 9952 7394 7502 11887 8619 7729 10327 7563 7698
200611_s_at 3982 5097 4147 2314 2187 2427 4623 3946 3556 3652 4272
2613 2767 2883 2028 3010 3110 3861 3149 1856 1924
200612_s_at 1385 1524 5279 2956 1713 1395 1953 2315 3710 3305 2409
1950 1006 920 1005 1377 1179 1199 2782 2715 2399
200613_at 4599 2113 3402 4029 3670 3502 5010 5555 3747 3196 5092
2976 2730 3524 4370 4348 4904 5301 2938 2603 2615
200614_at 12915 11614 10110 11709 17591 13575 15067 15335 11903 9228
15330 16749 13815 15926 16040 13299 12461 15454 9991 11250 12080
200615_s_at 1380 2139 4692 2071 1083 1337 2581 2375 2803 2545 2118
1659 637 583 548 1285 1295 1675 986 1809 1972
200616_s_at 841 837 1325 1354 617 559 1238 1327 3034 3381 1269
968 2657 1969 947 1552 2385 792 3563 2438 1508
200617_at 1434 1569 4109 6531 4114 3592 2878 2324 6443 7602 2037
1890 962 876 1267 2284 2610 1457 4098 2950 2733
200618_at 3540 4044 7513 8209 7470 4896 4377 4454 7954 7711
11263 9491 11294 9611 8998 3465 2938 2432 6274 4689 3731
200619_at 4391 1691 3643 3066 1900 1841 3061 2951 5063 4284 2594
1836 2204 1711 1329 942 1135 1016 1569 1537 1172
200620_at 1897 2861 2402 5140 2423 3266 2217 2857 2589 3797 2859
3792 3705 3943 4527 4399 5224 4304 3519 5197 5661
200621_at 1372 730 2751 2177 2448 2322 1573 1148 2485 2931 3083
3053 2152 1882 3000 1329 1444 1183 2406 1486 1030
200622_x_at 1216 1170 1214 1720 953 869 1504 1437 1319 1656 1444
1098 1007 620 354 269 292 272 298 249 155
200623_s_at 2937 3267 2385 4355 917 321 3144 2779 2525 3308 2641
1666 555 857 982 1677 1707 1667 1591 718 821
200624_s_at 2674 2614 5252 4386 4541 2653 4468 5528 4849 4539 4967
3721 4567 5265 5513 6293 5033 6525 5937 4112 6688
200625_s_at 5751 4097 5347 4785 4646 3334 4949 5087 5290 4546 4666
2859 5013 5811 7680 8129 7596 7375 8749 5920 7712
200626_s_at 3299 2810 4558 4278 5024 3669 4969 4849 5100 4402 4406
3562 7512 6175 8338 8968 8888 8051 9509 7437 12652
200627_at 12616 10610 11108 7381 14509 9943 11131 9423 12896 10258
11165 10483 17043 18699 19872 20813 19529 18948 17348 17100 16951
200628_s_at 1935 473 2650 700 909 2524 2148 1137 1561 999 1522
890 795 616 1000 1139 1386 1596 764 1975 3213
200629_at 3153 1126 3555 698 2305 7222 1896 846 1149 897 1838
1310 1821 1447 2741 3140 3975 4157 1875 6619 6471
200630_x_at 13487 16014 9175 7434 4440 4218 15985 13483 10512 8577 7623
5361 15993 14836 19571 16167 16064 13619 12754 18140 13377

200631_s_at	4626	5288	8829	6098	6283	6590	7726	6813	6734	7058	6763	6504	11321	12446	13537	10729	11399	10434	10748	13037	12694
200632_s_at	2555	2446	1380	9865	1166	1149	1642	2173	677	1874	2601	2916	1340	905	720	970	871	646	182	434	768
200633_at	22772	28188	20767	24981	31141	28507	27495	26818	21542	21223	23446	32023	23410	29635	29129	26362	29058	31245	16985	22280	18144
200634_at	15555	10021	22834	8254	7295	4372	18394	11059	12970	14725	18413	8825	12435	10312	11680	11119	10277	10958	11793	8573	5501
200635_s_at	946	2881	1183	2724	106	266	1096	1153	1104	1855	139	176	130	106	37	240	283	188	357	483	359
200636_s_at	2570	4564	2309	5339	169	984	1913	2863	2501	2397	544	910	711	311	177	1512	1793	1310	2945	3030	2538
200637_s_at	1126	2192	1375	3082	59	275	1248	1122	1200	2053	253	126	68	69	51	237	163	261	339	294	279
200638_s_at	7584	7131	7958	5577	7094	7208	8990	10847	6869	7139	7865	6804	10142	8649	7729	10326	11176	11216	8334	6110	8212
200639_s_at	8169	7017	5635	5759	4778	4746	6844	10219	3947	3888	6040	5493	10134	7174	10056	12371	14149	9584	7764	5963	7824
200640_at	7053	7731	6569	7161	6896	7129	12121	14350	7704	7192	9040	8533	7681	7560	8765	12494	11419	9388	7178	5770	7155
200641_s_at	5908	4125	5292	4442	5488	5393	12671	10685	8415	7458	8730	6810	5985	4753	3755	6382	5986	6329	3103	1771	4366
200642_at	13485	8731	8284	6125	13309	10442	13868	14153	10948	9446	15453	15329	19384	21706	18113	14826	16131	15912	10830	7584	6040
200643_at	1816	3058	2020	1927	1557	1842	2205	2055	1535	1597	1001	1112	574	584	565	1034	1107	937	937	1241	966
200644_at	3907	4771	7111	8449	1942	1341	4973	6558	7760	8613	3053	3167	2986	2072	1834	3076	3590	3036	6531	4931	3598
200645_at	3077	5531	5503	11748	3506	5601	6463	6506	4636	5167	6437	4689	6931	6736	8313	8094	8841	6911	5326	8932	6005
200646_s_at	299	523	263	746	422	590	464	352	226	482	343	215	62	52	45	160	228	148	116	127	102
200647_x_at	11445	9479	10055	3913	10166	10024	8954	6479	10678	7832	10571	11775	8628	10427	11143	8892	9632	8529	8610	10699	9472
200648_s_at	644	187	490	1734	551	663	439	629	436	788	614	729	206	283	269	444	344	472	432	280	529
200649_at	679	1171	604	1719	36	568	794	771	472	992	684	392	77	164	244	422	629	319	294	355	347
200650_s_at	16080	11563	20134	10711	21463	14030	18325	17768	23357	18153	20168	18832	19573	23635	27303	21732	23051	24739	17932	18173	19700
200651_at	27511	20655	21243	24230	21254	32121	22682	24643	17664	20208	21550	29025	20890	27360	24641	27114	28277	26175	17487	25306	19674
200652_at	1953	2643	5243	6323	2010	2926	3098	3175	3586	3961	2962	2713	3994	3936	4398	6226	6683	5273	4830	8174	6236
200653_s_at	4130	4809	2995	3566	4621	2590	2818	2827	1865	2974	3085	3847	9705	7179	6404	4793	6084	3914	8817	4304	3484
200654_at	20581	20821	14713	15051	8001	10972	17832	16670	11629	12328	11526	9341	7896	10369	8250	16531	18254	16462	7531	13378	11568
200655_s_at	6449	7669	3485	6063	7646	3935	5009	4987	3389	4731	5266	8168	7954	9415	10100	9468	8845	10132	9772	7531	10081
200656_s_at	8727	6661	6857	3995	3472	5095	7759	7051	5116	5371	4366	2700	1999	2150	1420	3462	3270	2938	1888	3310	2753
200657_at	17448	14760	16618	13171	14677	10463	17090	16007	21238	16531	15694	14244	15608	18694	18880	16037	17120	18100	13235	18038	15493

200658_s_at	6598	4854	4335	2809	3038	3053	5957	4364	5815	4176	4107	3256	7450	6332	3947	3337	3416	3941	4722	3009	2182
200659_s_at	2293	2080	963	1332	1845	1730	1561	1902	1828	1785	1244	1234	1789	1771	1064	1677	1791	1770	1774	1059	1010
200660_at	2613	2766	12833	13988	7553	7726	3596	1890	10350	13431	14965	15311	14750	13338	15671	2898	3244	2494	9539	8862	4491
200661_at	1725	1706	2233	4547	1483	2278	1589	2229	2109	2481	2193	1958	3134	2877	1895	747	893	1425	1881	2033	1948
200662_s_at	4662	5492	3136	3228	2465	4197	4900	5482	3804	4484	2444	2557	5789	3852	5159	10919	8870	10101	10091	11324	14449
200663_at	8925	6934	10622	15452	11584	11192	9332	7882	11834	10320	12400	11398	12308	13854	9012	4177	5527	3222	5475	6929	3689
200664_s_at	2368	1577	1705	1017	1796	1127	1864	1701	1235	1354	2516	1697	1112	1077	1061	1106	1096	1299	623	486	484
200665_s_at	87	17	8	13	77	136	62	88	40	4	205	168	47	75	78	1859	1974	934	21	2	5
200666_s_at	4560	2859	2550	2647	3372	2139	3222	2739	1969	2133	4380	2929	2533	2124	2063	1919	1732	1808	1255	1248	1096
200667_at	2246	2904	1474	2020	1567	2212	2056	1907	1480	1481	1434	1651	2401	2025	3225	4190	3686	4355	3317	3049	3968
200668_s_at	7980	6803	5814	5090	4550	5331	7333	6940	5116	4366	5167	4340	7430	6807	8594	10960	12083	11695	9618	7971	11133
200669_s_at	4915	4191	3372	2671	2867	2931	5142	4694	3089	3199	3900	2883	5143	5059	4343	5858	6640	5021	4670	2741	3658
200670_at	1708	1540	3984	2870	980	1814	926	885	1541	1530	843	750	578	713	1061	2125	2245	2391	3641	4735	4947
200671_s_at	320	332	608	462	1052	961	449	282	499	936	985	729	102	84	3	3	19	11	67	39	88
200672_x_at	1015	1027	1274	1254	1325	1036	860	808	967	1760	1356	1141	403	354	224	94	147	128	283	329	343
200673_at	4903	4002	3581	4251	5974	9227	4137	4747	3058	3416	5172	6541	7722	8156	5611	8192	8043	7433	5410	6099	6565
200674_s_at	22475	34322	23243	32088	16001	20913	26732	22511	21796	21232	21540	22877	15614	23177	25769	24824	27453	26409	14757	22855	17850
200675_at	7248	9251	3732	3412	3222	2571	7005	8172	5122	4605	6074	3903	4454	3603	3419	4228	4091	3558	3341	2199	1662
200676_s_at	606	444	427	582	458	617	449	552	589	527	451	440	287	374	339	317	348	233	510	269	274
200677_at	3291	5733	3079	4053	10877	8856	2655	3375	3289	3678	11570	16487	11127	12823	13838	5126	5705	4719	4288	6921	7660
200678_x_at	1462	4457	3036	9645	1516	2047	2097	3744	2158	4959	2761	2898	1660	1817	1064	762	1045	825	1378	2439	1934
200679_x_at	5976	596	5412	1935	9983	3115	7366	6733	6946	5754	9351	10241	13163	11284	5423	3841	3893	4016	5649	2427	2661
200680_x_at	13995	3996	15415	8470	21782	8430	18520	18739	18012	17218	17317	24924	18631	23735	22755	17652	18599	19547	13910	13224	13703
200681_at	8181	8813	5506	3280	4069	2758	8518	9716	7405	6492	5584	3954	4584	5382	7201	10256	11631	11808	10094	6077	7951
200682_s_at	5256	2827	3816	3604	5898	3456	6006	5676	4186	3583	5759	5317	7812	6346	4287	4193	4126	4279	3990	2646	2040
200683_s_at	2098	1170	1184	1499	1479	767	2320	2076	1573	1653	1703	1733	1287	1183	996	2115	1855	1942	1669	870	1238
200684_s_at	979	578	796	1051	910	488	1077	916	1108	1073	996	1127	613	635	441	742	592	634	828	557	678

200685_at	169	142	197	203	349	141	115	124	229	268	125	366	503	435	350	239	239	275	1000	1034	1376
200686_s_at	3262	3665	2310	1752	2076	1592	2594	2650	2230	2064	1748	2143	3759	2253	1551	2759	3747	4083	6798	5227	7193
200687_s_at	2129	827	2086	1139	1772	911	2810	2045	3306	2770	1653	1558	1491	1210	1585	1873	1251	1706	3194	2686	3412
200688_at	804	294	370	459	595	496	462	310	508	527	553	479	312	290	254	195	237	237	378	515	361
200689_x_at	27844	25770	22137	31254	20555	26607	23445	23000	24738	20028	19370	24653	16891	20192	18618	16339	20176	19706	12644	19431	15877
200690_at	848	768	715	419	642	898	1008	453	907	679	577	422	631	552	617	1292	1339	1253	1351	1562	1809
200691_s_at	8832	8553	8599	3504	6055	8669	7481	4149	6710	4924	3859	3626	7000	5307	4743	7630	8345	7945	8338	8493	10180
200692_s_at	5252	3731	4952	1902	3531	4816	5720	4270	4506	3556	2801	2125	3555	2580	1609	3913	3421	4176	3898	3238	3836
200693_at	8421	11577	10670	7277	13900	15089	11166	12544	12200	9163	15626	18403	16294	14672	18052	12245	10991	10909	12429	9228	12059
200694_s_at	2197	2028	1626	1565	3442	2809	3017	2427	2392	1821	3375	3001	2866	2771	3258	2893	2782	3136	3075	2043	2163
200695_at	2729	2274	2054	2614	1999	1316	2593	2369	2125	1698	2460	1420	317	467	455	586	688	1075	724	463	338
200696_s_at	1807	2536	3653	10262	3468	4042	1358	1714	2582	5259	3458	2815	2003	1982	1479	1111	1117	963	2850	3595	3442
200697_at	1126	1147	1084	1133	1237	997	1953	1281	1773	1778	2020	1152	1128	1279	1022	829	832	791	1195	787	852
200698_at	2944	1821	4852	3068	4003	3498	3118	3047	4568	5142	4835	3875	4120	4295	4590	4968	4399	4539	6857	4743	6231
200699_at	1360	600	2815	2735	2172	1664	1246	1203	3207	3616	2953	2698	3376	4068	2851	1633	1733	1120	3223	3439	3054
200700_s_at	5708	7320	5796	4241	4892	5226	6210	5716	5044	5905	7244	5907	4817	4789	5688	7094	7271	7565	3515	4018	4057
200701_at	3819	2482	2849	6893	6701	7871	3101	3391	1917	2559	8989	13888	13698	16397	12229	7578	8019	6081	2974	5185	3555
200702_s_at	115	29	140	39	418	548	526	380	538	238	762	594	926	572	346	389	476	574	345	251	221
200703_at	9758	8346	9673	9357	9032	7183	6497	6780	9359	7973	9145	12667	12986	15702	13441	9823	11503	10984	12748	9589	5868
200704_at	1656	3327	2166	5703	1828	2278	2608	1982	2985	3332	2213	1272	1571	2447	4871	7327	7940	6576	5790	5221	6556
200705_s_at	21328	23315	16214	10136	18957	19558	18330	18935	15898	14221	12902	19631	17037	15644	18491	19720	19524	20325	13842	20490	16890
200706_s_at	2224	2109	2256	5843	1171	1123	1746	1933	2541	3395	1947	1126	1105	1118	1032	1319	1361	1103	2137	1679	1568
200707_at	1626	2306	1200	1915	849	1254	2186	2143	1536	1518	1484	674	220	382	1018	1204	1462	1057	894	892	573
200708_at	3757	4501	4621	2519	3494	3003	4865	4727	4137	4080	4046	2338	3316	3737	5831	5536	5711	5105	6498	4575	5274
200709_at	3883	3928	4638	4323	4570	2856	6772	7553	5441	5343	7093	6352	2679	3740	6012	6026	5760	5244	4528	2792	2215
200710_at	2488	3136	3102	4590	2466	4095	1978	2479	2802	5578	3041	2441	3180	3542	1975	1082	1031	851	1977	2138	1439
200711_s_at	1819	2109	1970	2319	3336	3132	2323	2293	1568	2447	3623	4287	4717	4980	5522	2991	2452	2928	2497	1743	2130

200712_s_at	1965	577	2120	1512	2551	2919	1754	1967	2786	2523	3719	2402	1769	1810	1189	1081	774	886	1173	581	970
200713_s_at	5251	3766	6678	5573	8648	6797	6330	5189	9298	7425	8966	8184	6846	8029	9703	5525	6149	5656	7327	6536	6847
200714_x_at	2945	3095	3989	5905	3058	4544	3260	3352	2597	4130	4025	3222	875	1350	1594	1946	2493	1286	1773	3033	2396
200715_x_at	23334	28663	18026	22551	13377	17420	18333	21979	14610	14826	12497	15467	12922	15241	17787	22397	24047	20624	13459	19869	16578
200716_x_at	31711	39493	31177	45294	26910	33819	30269	30284	31088	26279	22705	35594	21924	29278	21832	21234	23998	22255	16074	23884	17616
200717_x_at	28668	46386	29906	29047	32131	28648	37132	36005	29371	28665	24517	35471	23046	27721	26710	30880	31901	33521	20646	28269	22931
200718_s_at	8995	10032	9590	10522	18721	18127	10287	10379	9090	10127	12177	18623	18552	18705	20179	18083	18931	19109	15933	17279	17154
200719_at	328	277	410	391	653	409	511	445	633	563	766	576	858	1117	1078	800	755	580	857	490	604
200720_s_at	1230	755	975	669	381	669	1397	1202	787	901	1535	882	889	961	766	779	842	828	540	453	412
200721_s_at	1610	1007	857	1198	943	658	1668	1384	868	855	934	602	521	469	324	499	558	562	493	347	238
200722_s_at	3747	1838	3605	2949	3190	2193	4672	4005	4829	4945	5312	3511	1344	795	704	1167	916	913	1485	945	1629
200723_s_at	6558	5109	6728	7091	6671	5209	6131	8371	8373	8906	9661	7983	6585	5916	7966	7828	6314	7737	9166	6449	9292
200724_at	239	198	189	188	337	125	236	276	348	295	210	250	26	39	62	3	49	37	111	94	53
200725_x_at	18017	21561	22928	32494	16335	21084	19062	19307	26699	20226	18250	23518	15755	17058	17527	14281	16809	14880	11392	20104	15992
200726_at	6489	5916	5861	4821	4650	4842	3090	5082	6221	5845	5679	3547	6370	9617	10691	14021	12618	16757	13608	13899	15066
200727_s_at	979	543	1384	1119	1581	1149	1564	1309	2036	2175	2234	1272	209	227	251	191	170	216	255	122	148
200728_at	1494	1628	4600	4987	4415	3705	3723	3283	6566	5421	3716	3730	6886	6013	3352	1462	1348	1104	4838	3087	2817
200729_s_at	2150	1719	3376	2323	3939	3827	1995	2845	3157	3772	3689	2947	6238	4185	1396	1609	1393	1622	2650	1432	1588
200730_s_at	1767	1695	1143	751	624	595	2781	3357	1109	1319	927	719	898	699	588	2124	1934	1771	1246	536	678
200731_s_at	1369	1757	770	1093	493	836	2918	3745	720	969	573	480	389	508	470	1706	1627	1707	1019	548	610
200732_s_at	2286	3073	1069	1627	1054	1127	3532	3998	1327	1593	772	804	1155	1072	1268	4979	5096	5052	2008	1918	2142
200733_s_at	2449	2274	826	458	904	1156	753	1561	384	635	441	832	2866	1983	778	4077	2699	4051	4983	2262	3070
200734_s_at	1524	1473	3367	2903	2256	2131	2418	1961	3671	3829	4532	3295	3397	3732	6143	1898	2224	1709	3974	4638	2587
200735_x_at	14378	17028	18213	20725	17298	20668	19447	17787	19491	19102	18629	17595	20600	26032	23706	23828	24730	24038	19085	24491	21145
200736_s_at	9620	11177	3501	4400	8720	8173	6824	4653	3875	3556	7409	8141	13159	11447	9018	6856	7058	6179	6044	4675	3211
200737_at	1732	1528	3610	3269	1964	2276	2735	2634	3571	4098	2871	2715	3298	2560	1696	1652	1219	1486	3034	1901	2854
200738_s_at	9192	7120	11587	11682	11249	12416	8263	8808	10406	10921	9546	9424	15064	14781	14803	13401	11799	13663	13945	15245	15139

200739_s_at	410	237	846	1195	1807	1588	453	395	1442	1388	2977	2482	888	1173	849	243	279	355	638	307	662
200740_s_at	1372	918	3361	3893	7009	6770	1569	1123	4098	5129	7285	8801	8438	10314	11454	3019	3253	3065	8739	6380	7096
200741_s_at	17115	26210	18798	25402	11653	19044	18855	24204	19016	20747	14616	18272	18989	21344	13252	23123	22845	22473	15451	22806	17770
200742_s_at	704	1072	906	3031	500	2039	1428	2121	902	1919	1226	1018	384	570	999	973	1090	811	816	1304	1728
200743_s_at	904	1645	897	2096	1008	2912	965	1843	819	1296	1167	1325	1787	2153	2344	1422	1420	864	1361	3005	3525
200744_s_at	3506	3179	1531	1326	2550	2002	3317	3402	2198	1990	3192	1719	1955	2085	1391	1545	1721	1618	1081	932	923
200745_s_at	4964	4915	2946	2836	2614	1830	5016	5038	3274	3495	4237	3177	1611	1535	1356	1818	2152	1584	1257	1324	1129
200746_s_at	6037	6222	3272	3174	3216	2732	5507	5152	3204	3656	4775	3554	2771	2871	3782	6958	6436	5552	4040	3798	3548
200747_s_at	787	1198	1194	1732	1079	464	722	951	1959	1553	981	1287	822	534	431	275	262	524	800	565	493
200748_s_at	29232	45752	28559	54570	16813	51688	27142	21557	24247	21483	14749	21022	22710	26498	25688	23436	26955	21416	20858	32287	20284
200749_at	4628	2948	3306	1411	3337	2149	3140	3566	2154	2451	2599	3219	5351	3725	4447	6449	5914	6400	5063	4695	4923
200750_s_at	11151	7554	14810	6492	11457	3825	14290	13750	16186	12650	11429	12294	16610	17667	16200	15985	13761	15762	16238	14672	12521
200751_s_at	1377	657	3379	2005	3847	4281	4206	4518	3448	2611	3058	2976	9430	5574	2149	5308	4062	3677	5055	2483	4388
200752_s_at	975	1382	893	1509	720	1001	1321	757	811	929	1663	615	427	226	434	297	353	254	327	320	442
200753_x_at	3388	2120	1862	1059	3087	1207	3264	2490	3247	2660	2645	2312	4333	2979	1574	2007	1688	2433	2987	1005	1194
200754_x_at	8496	6815	6039	5617	8236	3972	11381	10320	13882	10114	13942	9287	11425	14661	17335	13780	13872	15929	11716	7773	9108
200755_s_at	405	336	1278	425	867	1153	847	690	1114	1139	1344	929	1613	1253	936	1663	1194	1076	1052	954	1089
200756_x_at	312	328	1004	605	411	981	1187	1006	1188	1349	1298	1179	469	520	321	392	434	346	341	248	433
200757_s_at	1375	1568	3095	1949	2168	2637	2784	2489	2242	2934	3165	2691	2275	2487	2021	2834	2986	2648	1949	2096	2128
200758_s_at	1892	1586	1455	1679	1027	1730	2344	2016	1181	1479	1845	1648	1493	983	690	894	797	615	439	528	402
200759_x_at	4161	2551	2512	3220	2603	4424	3617	3275	2190	2942	2540	1747	1401	928	640	857	899	843	581	877	631
200760_s_at	906	648	1480	2962	1243	1677	1499	1416	1813	1149	1593	1683	2063	2223	921	976	1264	954	1288	869	1208
200761_s_at	2302	2592	3007	8533	2370	3157	2419	2151	3491	2215	3110	3382	2899	3066	2188	2910	2635	2001	4392	3902	5300
200762_at	1070	838	1038	247	5157	2201	900	1263	535	2260	3529	5356	4128	3945	5497	2397	2269	1341	3100	5395	5372
200763_s_at	27665	33723	21599	32750	18704	28554	24199	27054	24050	21874	17123	24503	22795	25463	25593	27590	28974	31639	18162	26682	23052
200764_s_at	2854	1518	2676	3031	6323	3704	2586	3191	2632	2769	4880	4155	2873	2526	1435	843	1036	922	1031	928	1107
200765_x_at	4236	3602	4050	5233	7110	6362	4208	3965	4216	4886	7572	6505	7376	7354	7289	4318	4434	4196	4858	4248	5205

200766_at	320	634	377	1058	396	1024	294	296	262	296	662	339	291	380	312	206	170	193	164	244	236
200767_s_at	724	805	876	951	1072	883	762	760	929	896	772	503	457	335	139	127	146	142	198	184	153
200768_s_at	2596	1867	5709	3399	3645	2998	3628	2623	5928	4525	2781	3148	4192	4287	4638	4546	4012	3894	6200	5006	5999
200769_s_at	1537	521	2147	1790	1280	1278	1398	1572	2881	1535	1366	973	464	613	573	832	686	854	873	438	818
200770_s_at	595	484	553	447	948	1089	507	723	379	505	986	762	484	641	423	430	382	421	402	378	383
200771_at	704	1170	1160	1269	1069	1957	928	961	1109	1142	972	1149	734	707	441	689	920	687	983	1009	899
200772_x_at	21895	19948	14167	8009	11661	4502	18700	20831	18038	17207	13102	15576	20499	20562	10265	14890	13719	15642	10064	10616	7938
200773_x_at	18732	16865	14665	13089	14842	8477	13336	17118	15010	13110	11208	12982	15336	15498	13438	18292	17972	18013	13342	14920	13697
200774_at	6650	7968	8358	6027	7520	7456	5863	5467	6575	7952	6644	6474	11741	9811	11032	7650	7228	6668	12272	11575	13923
200775_s_at	9358	5003	8208	5665	11466	6354	8250	8218	7197	10039	9198	8851	10291	9774	7468	6505	5763	6144	5473	5423	5224
200776_s_at	1635	897	2842	1257	3849	3069	4194	4849	3004	2973	4954	3503	3990	4062	4251	4742	3368	4189	3869	1603	2862
200777_s_at	3948	3674	4860	3511	7016	3947	6597	6281	4325	4120	5644	5231	7311	5283	4782	5531	4403	5255	4839	3225	5155
200778_s_at	4768	3646	3643	3227	2368	2196	6363	6100	5289	6544	4589	3175	1151	1036	1692	3595	3014	3945	2174	1736	2369
200779_at	12228	16004	10702	4051	7317	13516	8383	4157	4618	3528	4419	3723	6332	6821	6004	9358	7939	14194	5096	13648	9712
200780_x_at	17370	22189	18901	26415	16218	21824	16614	17388	17893	17948	18483	16457	14955	19906	23926	17858	20689	17161	13480	18644	16364
200781_s_at	16279	24002	20284	20325	19165	21019	22112	23631	22512	20058	17167	23354	17140	23533	19832	19570	21450	20820	13148	19800	13826
200782_at	4591	3709	7413	3413	3139	2895	4196	4390	4613	7434	3741	4302	4625	4896	5540	7673	7752	6769	6820	7045	7274
200783_s_at	583	383	368	278	1414	997	807	912	542	281	1028	1457	1688	1080	860	1036	1005	1013	1611	1147	1176
200784_s_at	30	43	30	423	60	165	40	42	20	42	18	18	9	15	10	6	6	7	8	19	7
200785_s_at	163	101	115	427	219	517	138	137	120	183	107	99	13	8	79	48	83	51	55	42	64
200786_at	3362	3171	4182	3044	7523	5323	6120	4403	4762	3677	6644	6421	10951	10690	8320	6462	6285	5587	5421	5387	3400
200787_s_at	851	951	1597	748	1150	1234	1154	1066	1155	1574	2504	1524	549	707	1090	407	350	381	653	445	286
200788_s_at	926	870	1020	797	1416	1293	723	900	931	1201	2881	1963	1775	1574	901	497	581	538	573	428	259
200789_at	1180	1511	2816	4289	485	1596	1236	1219	2026	2633	1504	2336	968	1105	606	629	594	526	2153	2606	2085
200790_at	15198	11987	14254	3009	11389	6450	15006	13243	12346	8736	10562	9409	11239	14124	15425	13917	16319	13749	12286	12682	12584
200791_s_at	1509	1296	3307	3965	1974	1649	1764	2607	3029	4306	2660	2315	2862	2422	2096	2643	2658	2598	5071	4724	5214
200792_at	14359	9053	7709	6174	8515	4879	11921	9654	8575	7469	8958	7842	8549	12063	10724	14873	15090	15608	7903	6037	6736

200793_s_at	1987	1746	791	1689	1052	1263	2578	1188	1090	960	932	904	691	676	1134	2032	1259	869	1316	1350	1606
200794_x_at	4039	5568	4396	8527	6503	8060	5482	6347	4823	6054	6519	6912	10501	10376	10897	11255	10589	9548	7509	9604	11149
200795_at	120	103	149	173	287	83	199	145	72	161	136	143	8	24	35	14	21	54	35	4	39
200796_s_at	402	316	784	506	107	281	432	544	563	799	470	263	74	44	52	62	29	34	86	44	45
200797_s_at	1832	3016	6290	5198	2385	1553	2329	2377	3487	4830	2474	2853	2331	2068	1459	1362	1886	1304	2843	3122	3109
200798_x_at	1490	1220	4571	1974	1439	618	1609	2158	2755	3831	2356	1534	1742	1397	1602	1718	1619	1873	2947	1282	1988
200799_at	67	89	5471	14453	4218	4158	72	28	5088	9909	3724	5703	3952	3840	2708	57	43	19	8760	5588	5273
200800_s_at	3499	2232	2442	6948	2037	1463	1493	1636	2855	5458	1587	3089	1991	2420	1636	2101	1000	714	4211	2756	2376
200801_x_at	34796	36496	34653	40969	37304	41316	34601	29903	44788	31914	31404	39163	24690	33165	36259	24575	26125	30586	20715	21410	17698
200802_at	3944	2926	4912	3166	3522	5129	3907	1875	2943	2714	2689	2633	4052	3216	3832	4859	6414	4385	2479	8181	7049
200803_s_at	5408	3066	6849	7500	5844	6448	8557	9310	7464	9259	8799	6580	5026	5035	5022	4828	4653	3849	4249	3942	5214
200804_at	7582	6553	9530	13556	8257	7081	7791	8984	9555	11785	10015	7948	9635	11889	11889	10115	11040	9815	11588	13112	14577
200805_at	1114	1249	1381	929	2077	1803	1002	769	1228	1104	1462	1492	2023	960	961	637	713	483	728	888	658
200806_s_at	12477	4371	16507	4781	14218	10029	15202	14836	17832	15149	13479	11276	13293	12663	11943	10857	10309	11650	11388	9442	12771
200807_s_at	24374	20112	25574	13084	27390	22425	25581	23285	28411	22310	18375	19686	20681	25273	27461	26083	26079	29063	20369	25446	24888
200808_s_at	2146	3330	4201	1468	1163	1101	2290	1938	2278	2507	2665	1093	436	486	710	706	732	832	717	456	350
200809_x_at	30653	38666	22681	26392	24109	32842	27888	29341	20536	22680	19249	38990	19453	24047	22871	25355	25954	25084	12694	22066	15916
200810_s_at	2801	3281	533	728	1603	1321	2341	4001	657	1198	1644	2110	3956	3370	2098	4882	5662	3896	1604	1236	980
200811_at	4153	3704	1027	1156	1498	1543	3205	4803	1255	1631	1950	2153	2076	1214	781	1849	2119	1192	782	644	408
200812_at	7868	6377	4726	2040	6446	4589	6881	4502	6989	5161	6340	5472	9878	8692	7498	6224	5942	6911	5709	4928	4611
200813_s_at	853	304	1153	1077	682	988	1170	1227	1291	1760	1433	1436	458	497	332	192	258	254	369	326	421
200814_at	5232	4777	3653	6513	2377	2771	4555	4545	2842	3692	4086	2834	2359	3017	4416	5797	7695	7610	2632	5039	3592
200815_s_at	687	578	1287	892	1232	972	1324	1057	1694	1523	2525	985	1433	1435	1621	882	783	797	1163	1008	868
200816_s_at	1357	954	1268	1437	1317	1044	1301	1417	1480	1658	1370	1822	1661	2066	1654	1791	1802	1267	2307	2043	2274
200817_x_at	40385	40745	31301	47920	31358	23782	37584	35538	31153	30626	25891	31682	25470	32481	26934	28463	31474	27612	19294	28078	21445
200818_at	6895	7030	6066	7527	7263	8744	9467	8321	5926	5754	9884	9002	12122	11174	10608	8717	8979	10231	7106	7072	4819
200819_s_at	24713	34829	22356	28390	20732	24693	26303	28368	22579	21111	22286	28993	18870	25742	20740	20607	22156	22273	12466	18259	10672

200820_at	4184	2923	4586	2914	2779	2313	3807	2712	3711	2783	4292	4221	7330	6738	4744	2478	2893	3389	3447	3442	1943
200821_at	626	364	794	472	1524	3310	329	505	467	612	1049	982	3107	2107	1574	1011	945	1155	2670	2502	3364
200822_x_at	13559	8846	15652	12138	10878	5817	11176	10652	13434	10476	14340	8506	13761	12978	15774	11097	12036	12391	10314	9279	8051
200823_x_at	18159	13799	15655	26205	13076	16080	12879	9971	10048	9247	11533	16984	16476	16757	17285	15289	18210	14514	10306	17477	11020
200824_at	8659	9828	9152	10933	3839	5378	9604	9939	7732	7975	5857	4764	8796	5669	3544	3283	3383	3184	5590	5711	2797
200825_s_at	7620	2780	7509	2629	2981	4853	4212	3554	3574	4974	4075	2796	1814	1634	1581	2990	3309	3069	1054	2526	2880
200826_at	13206	7571	11547	11813	9051	8187	12748	11476	9587	9357	9234	11947	15497	15110	10649	10798	12438	10748	9044	10126	6121
200827_at	1361	998	509	419	579	716	1003	1050	527	303	831	481	404	420	389	795	854	1037	332	283	354
200828_s_at	7333	2637	7981	6382	7377	2403	8073	6598	8382	7857	8821	6830	6624	6421	4299	3039	3310	3566	4542	3496	3790
200829_x_at	3514	1994	4115	3373	3497	1390	3237	3348	4396	4567	4797	3523	5347	5829	6757	6287	5792	6205	6903	5573	5964
200830_at	6777	2904	3995	2021	9123	6456	4960	3899	3738	2892	7313	7519	7007	7667	4719	3288	3329	3020	3679	2738	2539
200831_s_at	7909	1940	3607	1773	1450	755	1950	2280	1763	1746	1514	397	607	676	766	2851	3198	2921	746	1148	1384
200832_s_at	12012	9326	10595	5017	4044	4217	3836	4743	3315	4094	3050	715	4046	5532	9561	20391	18771	16471	8728	15129	14641
200833_s_at	2204	2462	3867	2588	1895	1713	1526	2152	3048	4422	2805	2693	5114	4585	5242	5557	5576	5476	8824	7222	8363
200834_s_at	25378	27224	23327	28745	21201	24638	22019	22581	26778	23640	21085	33700	23987	31404	19387	18891	18377	16576	15601	22066	15294
200835_s_at	24	27	28	27	74	58	56	33	54	42	51	36	309	220	9	38	97	5	20	48	37
200836_s_at	729	856	886	857	260	454	1079	876	1148	998	941	762	384	259	93	55	28	82	119	34	46
200837_at	2996	1058	6777	5399	3553	2024	2609	2610	9086	8085	4828	4210	5166	5445	3829	2336	2645	2042	5554	4307	3518
200838_at	2671	3052	803	1143	1583	1802	1632	1837	545	1115	3412	4502	3836	3396	2868	2137	2062	1750	785	706	745
200839_s_at	3240	3938	1078	1875	3407	3412	2639	3659	1017	1560	5243	7933	4686	4799	6636	3693	4341	3843	1354	1574	1437
200840_at	10505	9857	9170	6019	11436	8270	9413	7148	11257	9364	7721	9256	12878	13000	16783	17444	17847	18733	16854	21393	21722
200841_s_at	355	89	713	449	440	418	963	802	970	894	551	479	91	101	105	423	437	299	271	194	459
200842_s_at	751	923	2130	791	734	1157	1903	2080	1705	1720	1053	1413	2473	1880	2195	4037	3979	3764	3248	4692	6107
200843_s_at	2274	2201	3199	1428	1482	2442	4104	2637	2594	2073	1389	1351	1629	1737	2550	4645	4039	5082	3395	4529	6477
200844_s_at	4813	4191	6974	7158	3308	6331	5259	4458	8057	6008	4791	3847	6044	6112	7434	7549	7165	7514	6643	6822	5487
200845_s_at	4909	3984	4331	4895	3817	6000	4147	4843	5275	4412	2808	3430	4823	4543	6538	9246	7977	9868	8769	6998	7775
200846_s_at	3381	2911	5776	5453	2576	1399	3684	3936	6208	7684	5166	4446	8270	6357	3896	2143	2301	2759	4506	3665	3351

200847_s_at	2365	2719	1748	1767	3218	3244	1546	1991	1037	1771	2271	2555	4465	5009	5672	6148	7308	6523	5268	7317	8674
200848_at	763	780	1034	1933	517	766	1342	1705	1537	1944	1114	1036	1099	440	330	330	378	339	894	849	971
200849_s_at	1056	990	1804	2140	888	931	1129	1412	1614	1705	1174	1020	1724	1987	1351	1432	1398	1224	2732	1829	2363
200850_s_at	3023	3669	4665	4445	3214	3764	3642	3980	4712	4964	3328	2561	2452	2127	2106	2171	2090	1926	4572	3139	3974
200851_s_at	2134	2250	1938	3250	1710	1750	2680	2416	2697	2934	1988	2031	1935	1693	1905	3320	3965	3408	4148	3822	4765
200852_x_at	2455	2844	3266	3059	3322	2212	3143	2685	3152	2918	4883	3207	4789	2514	1163	535	462	610	997	611	366
200853_at	18196	5497	10985	4369	11645	4362	14633	13296	12348	9353	12217	13619	17385	18252	12077	12959	12432	15685	11664	8655	7928
200854_at	245	225	1674	1157	1234	1588	178	322	1108	1163	1296	1069	734	824	1219	270	272	283	1813	1189	1376
200855_at	151	92	430	362	462	571	146	157	559	334	234	402	303	247	316	75	99	64	470	281	368
200856_x_at	168	86	539	433	445	697	158	190	317	409	428	273	268	252	362	93	81	139	766	588	686
200857_s_at	516	309	1449	1730	1076	1181	580	437	1365	1476	1148	1189	403	360	227	59	94	125	478	355	377
200858_s_at	19733	19084	13829	18243	10626	11732	12570	14377	11735	11361	9012	14229	15401	15700	10706	14656	15729	13947	12602	16855	13682
200859_x_at	2650	3718	12405	2377	3057	5041	2630	2138	6094	6489	10280	6270	1808	2485	1959	880	1175	807	2871	1943	1550
200860_s_at	2646	2967	4178	3963	2976	1918	3432	3613	4495	3895	3462	3274	1855	2303	2501	3545	3988	4227	4588	4222	4399
200861_at	716	422	1312	1355	766	672	1211	1434	1442	1367	1018	1092	172	422	444	465	503	504	787	785	826
200862_at	3856	2305	4931	4166	2559	1473	1442	2334	4331	4163	5442	1409	2489	1873	2431	2338	1983	1946	4394	3729	3380
200863_s_at	4073	3882	6141	8109	2566	2358	5590	5718	7306	9005	4826	3759	4578	5746	7729	9670	9429	10326	8511	7984	8215
200864_s_at	873	658	1504	1282	803	590	912	1098	1037	1690	551	654	850	673	538	1478	1379	1564	3290	1322	2014
200865_at	9	4	8	3	11	8	8	15	23	22	14	5	2	4	17	1	25	2	15	1	11
200866_s_at	2809	3180	3340	6050	987	2088	3795	4000	3841	5273	2491	2962	867	1072	663	1836	1775	1829	1131	1625	2236
200867_at	949	967	987	854	1318	867	939	896	971	1038	1231	1694	992	1217	939	769	753	847	1011	1176	1044
200868_s_at	1185	1270	1130	1082	1281	1321	1344	1355	1523	1273	2560	2510	1433	1420	1703	998	893	1109	541	641	552
200869_at	21917	33172	12686	12970	13777	15516	21647	18849	10611	11758	13352	25374	18495	19358	13573	19221	20183	22331	11530	21376	15163
200870_at	7955	7496	7285	4386	7722	8822	7191	6255	7939	7329	7497	5855	9446	8641	10225	8589	9130	8717	8872	8696	9073
200871_s_at	8272	14445	8938	16072	2379	5144	5588	5098	4263	7685	2697	4874	3689	5167	3227	5762	5925	5250	4208	7272	6095
200872_at	5277	4211	19431	34151	13582	8947	6115	6589	26222	28560	20180	28340	20763	27150	17995	6921	9607	8588	17672	15763	12671
200873_s_at	7590	4702	6165	3064	6567	4909	6921	7862	6811	6418	7208	7067	12071	11736	11533	12093	11626	14079	10164	8659	10098

200874_s_at	745	344	799	491	672	359	774	681	638	725	659	689	1031	930	416	656	722	560	544	433	541
200875_s_at	4370	2940	2212	790	4669	2165	3986	3282	3240	2147	3427	2901	8040	8772	8109	6140	6532	8295	6338	7463	6816
200876_s_at	9648	3955	5343	3670	8051	7305	10038	9176	5206	5687	8831	9389	12463	12242	11085	11091	11018	9452	7670	6706	4542
200877_at	15727	13319	12924	9807	13351	12670	16855	10777	12728	9713	14348	13775	12150	13416	14385	13095	13365	14613	10873	10381	11750
200878_at	1155	2563	2066	1099	4283	2551	1265	1832	1052	2108	3435	5493	4188	4295	6464	1029	1195	828	1452	1655	1552
200879_s_at	132	271	328	228	393	336	363	314	211	383	594	491	40	7	40	28	24	28	8	5	19
200880_at	2967	2561	2805	2574	2958	1982	2759	2592	2883	2365	2746	2594	3168	3017	3646	4776	3779	4765	4269	3393	4505
200881_s_at	6123	4002	6518	5105	6302	3384	5202	4984	6466	6298	5855	5687	7182	8294	5528	6655	5751	6337	7082	6062	6019
200882_s_at	5152	4693	3063	2531	3403	2584	3845	3249	2852	3472	2976	4514	4160	4018	3337	3834	3969	2460	3402	2920	1933
200883_at	3426	1721	4029	4655	1948	1988	3220	2765	4966	4200	2251	2079	2569	2356	1527	3014	2581	2333	5488	4166	5955
200884_at	3019	2926	2292	4374	1193	1029	2405	2348	2950	2749	642	408	368	324	269	1267	1158	1208	2169	1037	731
200885_at	2003	2693	3817	4301	1399	2341	2516	1900	2650	3695	3579	2075	4355	1910	1473	1854	1705	1422	2347	1379	967
200886_s_at	13387	10386	9411	6936	7961	5095	11883	9793	11475	7620	10022	6325	11528	10712	8983	9801	10319	10752	8786	8374	7513
200887_s_at	2332	3850	2458	3769	2565	2254	1936	2322	2510	3901	3048	3423	2546	2107	2681	2270	3332	9208	4793	7477	11451
200888_s_at	11178	17667	23760	27493	23088	23412	15352	16745	21678	18167	14668	23710	16831	18105	15532	13928	13751	10960	13569	18543	15167
200889_s_at	1647	944	1804	974	1032	1418	1909	1706	1654	1154	1258	1106	1282	915	537	1525	1625	1703	1896	1792	2963
200890_s_at	816	533	941	520	389	714	843	767	543	760	681	680	546	642	778	1363	1267	1273	933	1025	1761
200891_s_at	4046	4586	4223	3445	3550	5472	3880	3259	2295	2832	2702	3143	4802	4712	5111	8217	6896	7346	6773	11611	11429
200892_s_at	3113	1916	2881	1212	3876	2221	3257	3076	3396	2791	2602	3313	5354	3237	2151	2323	1797	2017	3001	1095	1501
200893_at	5809	4839	5514	3227	8659	5264	5972	5724	5249	4613	7123	7114	11920	11722	12177	9723	9372	9729	10619	8428	9539
200894_s_at	1135	661	849	416	997	769	1373	742	1189	713	929	558	1257	1010	700	544	504	422	682	280	179
200895_s_at	2721	2045	1626	1164	3035	2466	2217	1719	2588	2067	2585	1386	2861	1673	1358	1198	1189	971	1321	894	585
200896_x_at	5993	3668	9156	5737	3832	2100	6531	5549	9593	8161	7099	3229	4641	4448	5007	3392	4166	4419	6013	4106	2813
200897_s_at	3895	5750	160	175	793	1180	4322	5800	150	263	637	397	655	567	402	5651	5796	5028	591	777	1044
200898_s_at	606	967	648	1230	667	461	1574	2301	1090	2119	1059	1090	235	206	186	240	376	251	265	97	130
200899_s_at	1152	1376	842	1545	1528	1391	1489	1874	1131	2302	1198	1852	1371	966	826	1073	1377	1000	1670	674	1010
200900_s_at	771	165	982	698	456	765	1211	1506	1218	1445	1344	990	2425	2105	534	404	282	321	400	319	393

200901_s_at	1787	943	1762	1573	2650	2713	1980	2055	2012	1845	1822	1781	2124	1983	1866	1317	1401	1343	1927	1538	1408
200902_at	3109	2507	3300	2259	2551	3232	1761	2552	2404	3252	1653	1702	4105	5015	4671	8970	8960	8613	7274	7443	8070
200903_s_at	5776	5115	5555	2267	5905	3546	4179	3385	4676	5395	5064	4766	5898	6993	5881	4736	6431	5625	4861	5644	6277
200904_at	525	931	465	820	158	688	280	413	223	614	257	616	233	275	190	280	433	374	169	348	336
200905_x_at	2477	4642	1409	2435	1963	2368	2274	2345	973	1413	2116	2532	1531	1875	2043	1969	2280	2983	854	1570	951
200906_s_at	480	557	75	56	250	235	861	1141	52	31	146	108	196	135	53	241	230	252	60	28	81
200907_s_at	2053	2843	165	160	45	77	2867	3909	225	198	415	323	308	192	58	1336	1248	1033	210	187	298
200908_s_at	160	243	201	229	180	152	147	211	211	225	156	39	55	155	435	536	251	293	318	295	187
200909_s_at	24625	30772	19150	18362	18221	17663	23791	25195	15444	16882	19408	26994	26560	31928	22415	26479	26561	28229	15440	21834	14377
200910_at	12835	6552	8293	4587	4860	3188	10837	11363	9468	7154	5131	5624	8087	6472	5678	9641	10033	8494	7065	7467	7331
200911_s_at	1844	1545	1710	1073	2309	2233	2103	2254	1937	2208	2426	1879	1491	1872	1903	1493	1439	1201	1400	1956	1811
200912_s_at	8741	16178	11457	20636	11962	25334	6102	8191	7068	7569	7359	11759	11159	10543	14556	16464	13455	15890	10847	15701	18039
200913_at	1083	635	1411	740	1226	469	1526	1090	2357	2337	2766	1183	3032	1219	1305	617	442	422	1237	905	672
200914_x_at	1220	1027	1110	687	623	738	712	967	625	920	934	632	2256	1932	1407	1687	1272	751	2193	1668	699
200915_x_at	2639	3029	3479	2818	3655	5425	1787	3207	3104	3393	4438	1616	5368	5491	7152	6162	4828	5836	6803	6481	7753
200916_at	1835	1628	4617	3881	3409	2052	1791	1813	8001	8127	6165	3330	4740	3067	1957	513	966	636	4256	2149	2134
200917_s_at	247	178	602	360	359	246	537	668	1254	1033	939	468	42	109	132	185	261	204	200	102	200
200918_s_at	1969	2056	3174	2728	2536	2356	2434	2140	3389	3694	3326	2777	1636	3908	6334	3361	4570	3334	4385	3761	4328
200919_at	2532	1326	3836	1249	2264	1688	2283	2355	3469	3505	3678	3499	1891	2369	2868	2267	2299	1617	1928	1717	1772
200920_s_at	927	2961	1687	4124	676	962	586	1317	322	1131	514	1399	648	485	340	328	383	390	392	1638	1445
200921_s_at	1490	3721	1687	3714	693	1207	906	1657	524	1315	505	1283	981	664	727	1507	1226	1812	1676	4657	5577
200922_at	1935	939	1858	1716	1030	786	2164	1818	1981	1814	2692	1176	890	578	427	544	527	471	530	435	468
200923_at	5204	13796	6055	9241	1956	2228	4210	4786	6166	7048	3257	2296	1271	2068	1757	5420	6164	12543	5831	7477	6523
200924_s_at	2891	3340	3390	733	2298	3110	2316	916	1353	671	2736	1371	3563	3277	3486	1940	2142	2365	1317	5387	4362
200925_at	14101	11010	14374	20319	7517	9752	13597	12888	12109	11906	10693	12629	11917	14190	15426	16237	17083	18779	12315	16596	11032
200926_at	26320	38452	28196	35311	26332	27714	31391	29330	32248	27610	22022	26251	25170	32973	28329	32147	34991	33312	24124	32861	27553
200927_s_at	1339	1175	1318	1455	1601	1774	1512	1116	1808	1433	1511	1484	1192	911	1060	1828	1162	1204	1776	1333	1679

200928_s_at	108	92	81	135	107	169	101	133	100	88	109	96	32	55	70	110	76	66	82	52	74
200929_at	4769	2350	2604	2027	3854	5029	4009	4136	2476	2988	5196	4479	5590	5117	5140	4953	5310	4969	2698	3173	3636
200930_s_at	48	12	83	80	243	55	93	100	13	14	128	15	36	33	21	13	21	28	40	23	3
200931_s_at	4839	4530	5258	2625	3260	2381	3188	3518	4643	4593	4080	3952	2821	3124	4072	4874	4663	4870	6185	3677	5047
200932_s_at	1832	1341	745	1574	1467	1240	1045	1450	618	635	2099	1154	2759	3149	3254	2262	2221	2288	1031	1675	1461
200933_x_at	21949	30340	25295	27890	34550	36089	25907	28120	24160	24391	28964	35009	22525	28176	27222	24770	25333	24630	16340	23567	20066
200934_at	2737	1838	3299	1341	6183	3716	2465	2225	4213	3497	4436	4034	7082	4736	6389	4856	4269	4274	10552	6526	9281
200935_at	2123	682	1266	376	570	551	1354	810	518	492	850	468	134	130	124	325	355	245	186	371	306
200936_at	30768	39330	22494	19628	18649	15464	28029	29394	18129	17737	18092	20348	19592	18770	16950	23015	26171	23759	12428	16749	11583
200937_s_at	14671	16386	15812	15521	13830	15738	14258	16742	18273	13093	11453	12150	18446	18864	13006	16361	17052	14794	13852	18569	14253
200938_s_at	17	7	9	58	82	273	8	9	15	42	41	15	25	19	13	22	5	8	37	31	25
200939_s_at	32	39	175	28	23	27	24	67	128	112	203	60	53	24	17	4	4	7	5	5	1
200940_s_at	853	710	1138	1733	837	676	922	834	1370	1947	1559	1401	804	614	622	323	347	427	692	626	620
200941_at	2684	1153	849	1579	1744	2005	3120	2362	1184	1454	1659	2242	2623	3902	3616	5627	5731	5298	2664	2816	3287
200942_s_at	7223	2671	2697	2972	2344	2035	6662	4921	3655	2790	3179	2852	4261	3201	1999	4320	4643	4428	2858	1443	1390
200943_at	15097	17514	7254	6354	5583	8235	14539	15187	9574	7789	5042	6844	10412	7541	7149	14972	13679	14133	9979	9181	8958
200944_s_at	12195	13016	6879	5838	15664	11955	8866	7277	6936	7150	9863	13368	14899	14884	12633	12666	12310	13184	10256	10494	10085
200945_s_at	1746	1691	2340	1721	1548	1988	1288	1407	1407	1641	1437	1763	1472	1319	2268	3413	3493	3992	3429	3807	6439
200946_x_at	1795	1763	2707	2230	1391	1375	1827	1727	1949	2767	1589	1376	1232	1302	1740	2081	2028	1487	2362	2241	3224
200947_s_at	2082	1438	2358	2055	1619	2014	1512	1013	1873	2648	1309	1255	2492	1997	2050	2741	2426	2308	4074	4415	4850
200948_at	4374	5895	4602	4079	2550	1568	5274	5798	4054	3971	4902	2532	2247	1852	2937	3382	3590	2685	3341	1476	1305
200949_x_at	18182	21933	14323	18901	9789	14765	14480	19464	14044	14264	12589	13373	15849	19186	16302	19112	21940	20418	11191	18509	11993
200950_at	8747	4297	5348	4457	6507	4359	6964	5278	3670	2766	6556	8261	8381	9007	8432	5730	5453	5509	3008	2478	2701
200951_s_at	17	6	11	23	22	26	12	16	17	11	74	26	35	40	90	4	3	2	1	4	14
200952_s_at	91	77	54	31	180	188	113	53	71	53	85	16	2	13	28	20	7	30	21	6	16
200953_s_at	102	85	121	65	544	637	98	139	95	105	742	259	129	139	1189	27	28	6	41	22	2
200954_at	1810	2019	1594	1795	712	351	1153	1483	1420	1581	1506	376	1152	939	1113	1032	1155	1011	1773	1123	691

200955_at	2389	2040	3284	2659	3581	2330	2972	2743	3291	3308	3141	3279	4200	3899	4702	3731	3994	3425	4415	3744	4202
200956_s_at	2081	687	3124	1394	1555	1144	3601	2672	4185	3945	3729	1927	2368	1527	690	630	663	655	1124	817	872
200957_s_at	2252	1254	2607	1278	2304	1019	2707	1655	3714	2654	2879	1616	3588	3368	2749	2340	2461	1693	3017	2191	1600
200958_s_at	2401	2905	6252	4230	5356	4530	2923	3902	6233	6022	5521	4870	6496	7592	8140	7916	6928	7483	10561	8785	12401
200959_at	3503	2338	1426	715	4209	3595	2650	2165	1513	915	2656	1545	7676	5556	1728	1503	1351	1120	1276	1138	733
200960_x_at	5931	6900	4612	6390	5253	5467	5948	5947	5962	5498	9242	6076	11244	12560	11375	6111	7643	5208	4071	6060	3422
200961_at	1013	1307	1123	1482	1142	2075	688	797	966	1100	928	946	1542	1153	1329	1677	1771	1847	2254	2918	3776
200962_at	586	2210	648	1879	437	577	454	971	432	809	436	270	178	211	645	1000	856	924	608	1091	1131
200963_x_at	20646	33802	25310	33611	26015	32872	24024	24103	22547	23463	21501	33330	23600	32299	29389	29092	30215	29318	19331	27091	21521
200964_at	5490	5396	5803	5226	3317	2483	8458	8232	8520	7761	8654	4223	1827	2925	4245	3101	3812	4236	4185	2272	2504
200965_s_at	2741	1386	3236	4960	5058	5140	4718	3936	3909	3611	7375	7745	6724	6969	6708	5898	6290	4570	4054	5215	5424
200966_x_at	18057	15953	18114	15301	7541	7230	12678	9580	13829	11773	13928	9987	12883	13947	12557	11797	11867	12443	10844	10104	7969
200967_at	9799	4229	7937	4472	5278	8782	5716	5087	5330	4653	3732	4307	11104	11547	9622	15485	16708	13663	11795	18831	15600
200968_s_at	8938	3620	7752	4277	4119	4974	5370	4231	4677	4331	3775	4308	7085	7768	7234	12114	15164	10510	8197	16980	11490
200969_at	1820	1940	2433	2056	1413	2451	973	907	1493	1405	944	1392	2137	1801	1782	3097	2892	3749	4547	6064	8106
200970_s_at	2338	2303	4784	3712	2148	4146	1745	1457	3191	2697	2140	1369	2428	2703	3404	4168	3924	4641	5315	6348	7249
200971_s_at	4982	5272	10300	7717	4690	7124	3491	3010	9994	7302	6638	5601	6435	4869	5445	4832	5628	3634	8430	11784	9506
200972_at	3487	5020	5214	9979	3390	2778	2940	3729	4576	6924	3884	3695	4894	4583	3499	4921	4561	4433	6562	6714	7614
200973_s_at	1707	2039	2515	4349	1986	1437	2100	1863	2959	4067	2002	2207	3074	2731	1516	1880	1779	2190	2329	2374	2841
200974_at	497	865	397	357	1126	792	553	596	199	403	544	1005	634	567	392	246	210	247	109	97	96
200975_at	1668	1664	3920	5270	1983	2833	1908	1651	3892	3936	2385	1645	2534	3583	3587	3337	3332	3138	8378	7575	8859
200976_s_at	1670	1800	3336	4383	3890	7098	2097	2280	2447	3097	2642	1933	5072	4681	5426	1420	1775	1465	5851	7437	9427
200977_s_at	1363	2208	2608	3041	2672	3757	1662	2074	2250	2875	2665	2172	4143	4472	5975	3925	3799	3776	7274	9348	11201
200978_at	5980	6554	5361	7290	8350	6405	7812	8090	5230	5778	6879	7818	9440	11586	14211	12442	13394	12796	8510	8601	8525
200979_at	771	506	1271	1372	701	712	429	512	795	973	893	640	1001	901	645	480	415	569	1471	1163	1418
200980_s_at	2680	1771	3756	2963	2891	2263	2832	2361	5109	3740	5422	2397	6060	5793	4471	1938	2130	1688	3344	2142	1807
200981_x_at	18712	23569	20850	26361	16791	18850	15929	18841	18008	18953	20570	17618	12854	19037	21002	17250	19352	16240	12349	16996	14152

200982_s_at	909	536	15	19	2904	1821	751	607	23	46	6525	3602	2646	5033	4286	1331	1987	758	67	119	61
200983_x_at	1021	3317	11150	6433	2069	2091	2722	4289	6621	12388	5841	9549	3389	3319	1353	1030	1235	991	2528	2375	1516
200984_s_at	955	2085	8723	4038	3137	2678	2132	3117	5543	8435	4112	5740	3499	2607	2184	1871	1936	1512	3196	2562	2215
200985_s_at	1101	2696	8456	3982	2309	2451	1357	2732	4097	8535	4116	6939	3786	4302	3749	2718	3045	2217	4572	3865	3718
200986_at	87	39	7	56	63	523	68	107	21	63	129	165	25	27	18	79	29	36	38	4	15
200987_x_at	1982	1895	2476	1730	1375	974	3172	2493	3095	3098	3110	1932	1401	1595	1425	1077	879	1654	1791	1127	951
200988_s_at	402	321	869	785	733	527	745	421	1201	1125	846	696	384	372	482	351	285	347	633	222	434
200989_at	4870	4243	2777	3126	5032	7466	4330	3882	2513	2256	4139	2965	6672	6262	8871	12934	12765	12472	6203	6947	9502
200990_at	9523	5741	7265	5297	2987	2173	10368	10414	6999	7335	6572	3423	1946	2384	2501	3295	3063	2466	4404	2465	1906
200991_s_at	1139	1196	639	847	903	1129	1122	987	637	548	1542	1239	1925	1743	2287	1591	1674	1713	1166	1163	1276
200992_at	2576	2722	5738	3641	5788	7523	3317	3514	4956	5546	4932	5125	6376	4414	3438	2493	2309	1838	2980	3835	3353
200993_at	2322	2320	6337	3016	4304	3677	2637	3418	4106	5214	4552	4338	7152	6242	5062	3726	3184	3368	5659	5810	5918
200994_at	2068	1826	3993	2103	3183	4013	1423	2136	2724	3377	2805	2540	5248	4578	5761	4138	2933	4100	6788	7058	8792
200995_at	618	564	1306	687	1541	1209	892	743	1341	1389	1391	1306	2070	1983	2504	1368	1280	1434	3133	2390	3564
200996_at	4646	3053	4692	2554	6473	5455	4108	3880	4285	3329	5571	5033	7880	7113	6794	5022	4088	5889	6988	5131	6420
200997_at	2483	1069	1953	1776	2682	1226	1699	1108	1761	1146	1865	1561	2882	2767	2934	2260	2210	1982	2093	2528	2856
200998_s_at	1314	1175	1198	1081	1232	1408	1362	1003	640	723	1019	816	314	244	192	405	325	486	172	481	420
200999_s_at	2182	2697	1207	1487	1038	1344	1624	1966	531	808	1391	1269	1195	932	2052	6162	5291	6500	941	4641	4260
201000_at	4945	4910	5487	2248	2591	7409	3888	2644	3128	2485	1840	1748	1129	976	1325	2400	2644	2781	1727	4643	4380
201001_s_at	836	581	2229	1911	1582	1531	1911	2154	2471	2200	2926	1479	2250	1973	1276	1194	1076	1128	1836	1554	1551
201002_s_at	2258	2007	4647	4962	3253	2040	4321	4810	7282	5311	5463	3715	3712	4105	4623	4055	3546	3354	6323	4782	6118
201003_x_at	3147	3075	3002	3974	1632	1556	3286	2631	2823	4858	1705	2054	633	738	536	1364	1285	1262	2446	1853	1921
201004_at	7395	9801	11918	17609	5187	6764	6034	4219	11552	11016	6630	8491	8189	8616	6220	4411	5818	3954	7277	14803	6754
201005_at	5281	5190	4739	6738	12688	7492	4835	5123	4527	5873	14486	19385	16355	17765	16297	8020	8400	7127	7326	7480	5983
201006_at	255	184	250	336	261	175	288	375	233	298	262	328	75	58	149	27	78	64	60	59	54
201007_at	2823	2978	2694	4864	3744	5327	2802	3324	1980	2361	4426	4122	5154	5446	7308	4797	4564	4532	4131	5227	6392
201008_s_at	65	39	274	1526	399	137	72	90	606	3909	809	3978	216	179	74	5	12	3	88	58	125

201009_s_at 415 285 581 2521 815 351 480 457 883 4187 969
4102 287 294 127 90 58 46 190 202 249
201010_s_at 230 117 537 3271 1078 387 301 281 997 5585 1337
6655 435 433 425 9 34 20 608 333 661
201011_at 4747 3018 4186 2752 3398 3862 2359 1862 2918 2855 2271
2256 3639 4148 2855 4677 4977 4821 4743 7303 7405
201012_at 8657 8171 10711 2500 224 194 11710 11235 4734 2907 274
163 1672 2287 1668 16762 17472 21137 5278 8520 10478
201013_s_at 7656 4454 4707 1838 4360 2436 7227 6550 5764 4172 5176
2777 7137 5971 8339 9625 9554 10987 9610 6140 8425
201014_s_at 3833 1388 3217 868 3053 1591 4987 4572 5675 3022 4019
1898 4598 3688 4001 5023 4282 4105 5562 2599 3340
201015_s_at 3294 3906 4725 6060 1622 2024 2507 3534 3271 5811 2410
1641 1043 921 1342 2231 2514 2334 2940 2483 2347
201016_at 2786 1893 2492 1041 3029 1755 1373 1404 1828 1978 2137
1857 4167 3639 2430 2193 1728 2491 5489 3431 5437
201017_at 663 742 1213 1057 520 602 913 847 1331 1824 1271
1252 1934 1565 1594 1329 1623 1409 3267 2022 3710
201018_at 659 558 1455 1110 1295 845 979 724 1878 2240 1454
1282 2229 1550 2034 1773 1490 1464 4199 3449 5418
201019_s_at 2802 2101 5460 2559 3949 2826 2117 2077 4675 4741 3853
4092 8539 7535 5552 2444 2213 2064 7286 5256 5901
201020_at 2110 2270 1860 1331 2140 1059 2291 2194 2422 2493 2535
2586 3167 2809 1398 1793 1757 2040 1853 957 1058
201021_s_at 7943 8006 6221 8779 8841 11736 6039 6627 5659 6721 7630
8120 11096 9273 7138 6550 5760 6653 6825 6034 6699
201022_s_at 9979 10617 9381 10174 11932 13911 8177 8846 7997 7881
11175 11529 13582 15179 17126 14697 15271 13906 10866 13691 13036
201023_at 1237 1341 1888 2847 2749 2319 1284 1776 1651 2194 2931
3124 3657 3259 3755 2437 2493 2697 4073 3365 4918
201024_x_at 2590 1872 1807 590 1614 1360 2443 2099 2115 2020 1441
1029 3508 3985 5007 4917 3945 4345 5526 3945 3229
201025_at 548 380 757 587 745 677 681 566 912 907 831
331 841 730 744 1000 882 1065 1711 994 1023
201026_at 541 405 456 194 329 344 591 945 567 812 647
297 219 285 654 848 452 643 919 684 313
201027_s_at 635 564 1346 738 1593 1095 2152 1474 1940 1213 1425
704 1375 1166 1770 1166 1068 1078 1454 1077 865
201028_s_at 869 1100 184 328 1296 1262 1096 969 281 379 2912
2054 1688 1616 1357 451 482 456 163 186 138
201029_s_at 2031 3147 373 823 3034 3380 2412 2527 641 669 5305
4629 3572 3699 5012 1698 2106 1562 785 811 437
201030_x_at 15432 14728 20670 14702 16836 17130 18968 18362 24038 18798
14890 11751 16222 18558 22481 22560 25114 24344 17881 19747 18115
201031_s_at 10835 3583 3515 2217 2170 2737 5906 6370 3120 2709 2646
2752 9493 7443 6741 9730 7644 9117 10555 8920 8727
201032_at 844 1007 2546 3695 1656 2173 1454 1231 3182 3015 2850
2104 1806 1790 983 607 793 603 1617 1295 1277
201033_x_at 35129 47754 34827 41013 27268 28535 37231 42271 36373 31597
26225 40661 18962 24801 26773 25570 27056 28335 15782 23739 20344
201034_at 1345 2056 2962 6842 2693 2497 1613 2101 2157 4352 2295
3070 2914 2617 3124 1904 1431 1604 3544 5028 6661
201035_s_at 1139 742 1123 1534 1539 1204 1175 1156 756 988 1428
1362 1115 1196 1116 380 290 471 625 480 496

201036_s_at	1611	1128	1082	1679	2124	1400	632	838	740	815	1056	1195	2226	2047	2324	1860	1344	1841	3305	2782	3310
201037_at	5952	5743	7627	2563	972	982	5649	4012	6152	5687	2260	1866	1706	2206	1715	4850	5272	4735	5012	3519	3760
201038_s_at	681	658	1133	921	870	574	1145	1141	1315	1132	800	648	1389	982	1016	1469	1351	1115	3412	1727	1726
201039_s_at	1674	1628	1138	794	1364	964	1619	1673	1065	1219	1489	1273	1288	767	643	793	511	431	648	345	233
201040_at	1065	1134	1190	1632	790	484	1234	1533	927	1224	1683	738	984	1295	1351	1395	1374	1321	1029	797	955
201041_s_at	538	2525	570	484	619	856	546	622	221	366	722	896	804	985	978	2037	2502	3250	1066	3631	4230
201042_at	318	236	270	299	6119	2000	329	326	107	217	9211	6960	8421	5944	2860	138	246	109	112	126	97
201043_s_at	614	172	932	635	836	434	1316	1027	1944	1474	1073	537	1705	923	371	676	461	222	1172	595	470
201044_x_at	12	11	11	13	55	48	9	18	12	14	10	13	13	49	5	35	17	66	8	7	59
201045_s_at	13	22	47	78	388	465	118	18	48	80	9	46	19	8	14	5	39	11	44	9	18
201046_s_at	2205	2620	1598	1483	836	1349	1969	2113	1314	2008	2220	1061	1178	1548	2560	3072	2534	2566	1903	1333	1058
201047_x_at	2470	2315	5026	4207	2768	4424	2364	1999	3232	3173	2612	2698	2825	2325	5080	4024	3355	3622	5608	6856	7023
201048_x_at	253	172	414	278	602	654	273	317	598	456	368	607	84	26	84	85	54	39	151	126	270
201049_s_at	32638	40774	27535	40234	24301	26624	36960	33257	29335	24026	22589	30753	19855	28402	25412	26933	30799	31199	15728	24693	19983
201050_at	564	1334	787	1848	361	589	868	983	599	871	797	650	212	187	253	439	425	407	276	491	420
201051_at	3356	2881	4904	5617	3491	2279	5717	5322	7682	5920	3849	2544	6216	3448	1637	2362	2618	1641	4132	1745	2043
201052_s_at	1958	2005	1727	1049	1913	1892	1738	1620	1474	1428	2658	2731	1829	937	1346	1035	760	956	1033	859	877
201053_s_at	720	875	556	521	1101	1080	612	587	652	953	968	1286	2051	1156	1840	1145	1166	1090	2626	2096	2683
201054_at	3317	4675	1913	2356	3912	3830	3988	4686	1583	1880	2633	4353	5287	3031	4426	5616	4426	3665	3134	2116	2682
201055_s_at	1409	1373	1012	901	1368	853	897	1003	979	1055	1367	867	2049	1169	660	743	785	431	890	609	472
201056_at	288	313	522	1124	357	728	387	552	437	1029	475	466	231	345	220	367	321	350	463	713	409
201057_s_at	549	809	1359	1891	1467	1454	823	1024	993	1635	1214	769	497	601	710	831	747	952	975	1773	1834
201058_s_at	540	1899	8	32	70	1086	612	428	21	8	924	136	127	102	169	194	218	237	4	2	2
201059_at	1606	2280	1883	2883	1215	898	1227	1218	2038	2064	1042	1086	1186	1190	1457	3292	3318	3306	7054	6221	6866
201060_x_at	422	349	252	219	345	290	409	392	322	274	300	263	71	49	122	702	620	807	77	66	35
201061_s_at	398	377	164	136	416	499	424	330	120	138	189	265	101	88	151	696	630	706	36	49	19
201062_at	12	4	3	1	3	5	3	3	3	1	1	5	2	3	2	14	2	1	1	2	1

201063_at	2184	3179	2387	662	8624	6285	1791	1442	1414	1016	7265	5222	10865	11871	12051	5312	5547	4835	4350	5086	5066
201064_s_at	6816	10327	4794	4190	3824	3554	5696	4785	6041	4943	3316	3909	3643	2882	2906	4387	4692	4260	5060	3956	5558
201065_s_at	2131	1383	2005	3313	2735	2505	2446	2604	2111	2159	4079	2820	2800	2688	2069	1733	1650	1589	1710	1565	2028
201066_at	8427	7305	3201	2130	1970	1386	7417	5269	4317	2372	2898	2198	5498	5126	3635	6255	7409	5889	5336	4291	2187
201067_at	1095	635	874	1265	1035	1368	1200	937	740	1307	1563	1656	1667	2031	1504	1016	1044	934	1158	713	1247
201068_s_at	4507	2278	4403	2840	10246	7997	5080	4129	4863	3767	11456	11626	16049	17133	16884	7997	6287	8144	7888	5465	6810
201069_at	128	90	247	193	390	231	174	137	131	197	178	250	141	119	147	70	82	78	50	74	57
201070_x_at	1627	574	1257	1526	948	771	1333	1817	1766	1639	1858	1512	552	435	337	370	443	354	313	257	282
201071_x_at	7459	6199	7055	6990	8614	5874	8061	7701	7020	6225	7210	6928	6537	6476	8721	8442	8225	8331	7893	7681	8426
201072_s_at	480	398	505	410	89	179	753	776	808	753	640	599	513	456	196	251	266	126	160	227	122
201073_s_at	861	854	1252	810	460	520	1644	1715	1383	1588	1271	659	290	338	136	202	217	285	278	120	181
201074_at	2106	1872	2907	1573	2484	1349	2373	2867	2122	2380	2358	2251	2748	2528	2252	3351	2493	3089	4135	2805	2521
201075_s_at	1255	945	2041	1035	1480	1079	2047	2191	1798	1822	1808	1072	888	633	414	470	483	579	806	436	534
201076_at	2082	893	996	674	2157	1825	1901	1880	1377	1132	1649	1253	1698	1195	799	1166	1236	1190	1035	917	452
201077_s_at	7406	3844	5186	2968	4737	3995	6783	5902	6247	4988	6439	5574	8510	6474	5720	5907	5717	5646	4377	4707	2896
201078_at	5452	5472	5401	6194	4427	5297	3531	4777	3952	5446	5692	4772	5157	4734	5625	6503	6423	6286	6911	6469	8031
201079_at	4356	4231	2710	3546	1703	2379	3645	3470	4090	3684	3337	2478	2460	2018	1124	1369	1636	1354	2167	1081	1097
201080_at	545	457	891	767	639	336	586	607	805	865	728	817	448	263	220	199	221	237	271	242	256
201081_s_at	382	184	568	418	330	349	518	471	669	720	618	520	100	95	98	58	56	74	102	69	72
201082_s_at	738	564	575	843	748	836	953	996	669	841	921	554	190	262	349	260	229	337	385	256	247
201083_s_at	151	116	286	107	282	296	282	354	289	273	331	161	638	314	159	364	332	269	591	343	470
201084_s_at	3425	2692	5123	3404	3825	2655	4591	4919	5340	5343	4639	2608	5358	4713	6856	8508	7740	7861	10462	7571	11144
201085_s_at	755	757	393	420	400	368	776	827	470	780	830	598	692	537	339	383	291	226	301	264	250
201086_x_at	2912	2996	2600	2420	3776	2022	3558	3139	2645	3029	3216	2771	2215	1714	1961	2124	2007	2149	2156	1896	2342
201087_at	1294	1147	2180	1280	1205	949	1811	1681	1836	2034	1943	1901	309	448	220	328	308	230	266	143	235
201088_at	8297	3848	5380	2112	10631	6837	5874	6114	5437	4598	6360	8350	11065	9909	6368	7652	6763	7163	9789	6736	7702
201089_at	1230	1007	1384	1165	2482	2498	2141	1871	1890	1618	3725	2789	2113	2798	3793	1507	1282	1232	1310	819	895

201090_x_at	25015	12428	25150	9454	27244	16302	28831	23814	30987	23244	25584	33294	18700	25375	21998	14544	15135	17956	13537	12075	10340
201091_s_at	3008	1237	2367	1036	5601	2607	2185	2363	2266	2150	2932	3268	7703	7251	3223	2961	2995	3800	4836	2891	4506
201092_at	4429	4630	5362	4818	7946	5340	4551	4545	7512	6741	7325	7979	9859	9266	9058	4941	4795	3592	8355	5809	6812
201093_x_at	1172	1426	3140	4599	2146	1384	1355	1310	4874	5205	1688	2373	2372	2203	1474	824	1065	1209	3196	1788	1772
201094_at	17870	27156	19275	22968	19011	21817	20165	22168	17046	15267	13018	25898	18115	21256	18086	22324	22512	21736	14742	22156	14476
201095_at	2367	3581	4642	5134	1597	2008	1235	1660	3861	4945	3471	3740	1827	2324	2945	2051	2145	2031	2833	2980	2962
201096_s_at	2983	2510	5970	5030	6559	6516	4819	3655	4866	4633	7565	7083	9344	7165	5239	5037	5234	4383	5493	4573	6393
201097_s_at	6022	4626	9294	8810	8677	13367	5148	5872	6759	7230	9366	9605	13780	13042	12198	11486	11428	11811	10413	11112	12390
201098_at	1839	1935	1868	2517	4642	5510	2100	1801	1869	2214	3765	5041	5542	5310	5880	4330	4362	4514	4256	3924	5727
201099_at	843	955	969	685	1288	1635	795	1104	880	1023	1396	1092	1044	1206	1664	812	903	1161	1074	1201	2053
201100_s_at	949	1682	1594	1870	2356	3442	1540	2020	1238	1433	2513	1855	2587	3245	3718	1627	1843	1772	1411	2169	2415
201101_s_at	69	32	1567	313	1321	632	2075	2337	2808	2432	1968	846	1598	1053	425	680	690	651	469	340	398
201102_s_at	896	1152	501	610	1112	939	1024	1114	654	857	698	490	271	323	301	420	362	359	360	271	207
201103_x_at	3667	4887	4433	5057	2801	7616	4108	5937	4225	4147	4431	4101	2672	2317	2417	3065	3651	3384	3262	4371	4764
201104_x_at	1524	1195	2425	2990	2052	2852	2609	3069	2226	2537	2676	2453	476	348	442	588	730	858	686	839	741
201105_at	13886	5516	1785	3165	10347	9656	8594	6273	547	169	13902	19123	14937	16350	6622	7372	11052	6501	433	1275	576
201106_at	6558	6794	4548	7038	4775	8197	3677	4505	3628	5644	5540	5796	12551	10774	8633	6275	8174	6193	5272	7041	4211
201107_s_at	9	24	24	25	22	30	29	9	112	17	30	6	2	22	21	10	7	21	16	5	4
201108_s_at	242	489	399	366	209	167	232	248	575	398	300	289	47	101	11	72	43	56	92	129	161
201109_s_at	200	467	198	160	30	327	62	115	256	239	207	353	168	120	58	206	125	111	204	108	187
201110_s_at	111	500	102	65	168	695	27	96	106	92	129	279	388	136	123	558	326	284	574	280	370
201111_at	2361	1137	1639	551	3223	1836	2423	1944	1846	1174	2012	1842	3687	2527	4325	4083	3154	3806	4330	2819	4013
201112_s_at	4020	2528	7809	3690	8198	3432	6078	6589	9872	7479	8887	8354	10111	11064	12511	8891	7675	9610	13282	8058	11081
201113_at	8833	9433	9814	6637	3209	2083	7820	5922	8395	5869	4670	3161	4197	4649	4543	5498	5583	4915	5323	4401	3011
201114_x_at	10246	7596	8489	4900	13700	10279	9988	7776	11309	8797	11721	14976	14302	12768	15420	7735	7886	8660	10514	10027	10067
201115_at	1863	1315	1422	729	1856	1256	1675	1631	1584	1642	1977	1819	2153	1549	1250	1020	1042	1087	1754	932	854
201116_s_at	964	1851	45	44	48	28	847	864	48	38	37	50	6	21	33	925	974	896	10	54	12

201117_s_at	898	1343	5	5	51	3	423	336	41	13	13	8	1	1	1	1030	742	765	1	69	80
201118_at	6145	1544	3635	2153	2210	1864	3849	3624	2248	2844	2987	2188	3755	3348	2937	4939	4981	3052	2706	3188	3773
201119_s_at	8772	6613	11256	8968	8725	5150	11761	10134	15106	12760	11986	12309	11842	10119	6793	5067	5994	4799	6565	7165	2569
201120_s_at	2589	3160	3692	2023	3688	2293	2744	2879	4082	3194	4905	4468	3282	2335	904	821	711	813	1768	1014	2065
201121_s_at	4346	4485	5384	3773	5366	3643	4257	4330	5527	5034	5875	5815	7865	8907	9475	7342	6919	7022	9770	8368	10121
201122_x_at	5772	2853	4042	1852	2724	2029	5670	4955	4094	3403	3947	2610	8978	4565	3420	3188	2778	3375	4997	4437	3403
201123_s_at	14464	6925	10099	4342	6313	3010	16378	11986	13405	9575	11384	6045	16310	15452	11461	10824	11384	13774	11203	7492	8070
201124_at	402	456	605	391	566	607	288	422	440	713	613	1659	454	482	178	284	381	209	407	315	378
201125_s_at	2174	1895	3691	976	8567	2142	1151	1500	2918	3003	6152	9595	6035	4457	4464	2483	2782	2060	2720	2733	2980
201126_s_at	655	569	799	576	874	614	651	677	815	864	682	422	436	457	337	301	389	311	398	421	315
201127_s_at	1958	1019	2096	643	1920	1064	1113	1221	1105	1152	1157	1213	976	730	1001	968	644	704	927	909	706
201128_s_at	7865	5304	6912	3656	7136	4963	5570	4931	9286	6352	5943	5006	6382	4868	7448	7375	7079	6366	7047	7666	8180
201129_at	1639	1209	926	1031	1241	700	1275	1280	1015	1154	648	1124	958	1062	1665	2267	1855	1367	2070	1210	1782
201130_s_at	370	147	1248	1476	315	525	480	381	1203	1785	287	180	4	3	9	53	19	26	67	72	138
201131_s_at	2367	3222	8455	10077	1559	3272	1931	2197	7397	9416	1967	979	1007	416	768	2956	4073	2416	8242	9537	9539
201132_at	1136	933	1436	2152	1887	2561	1565	2060	1211	1867	1435	2182	3720	2705	1851	2701	2414	2113	3590	2228	2456
201133_s_at	413	456	688	763	496	761	510	816	487	609	505	927	639	357	224	684	755	722	738	998	1275
201134_x_at	8321	12465	8696	14843	3810	8167	9914	9757	8165	10103	6195	7573	11001	11808	11074	16099	16001	16630	12616	13465	9492
201135_at	3367	2345	6217	7399	3406	2200	2588	2681	7741	6618	4062	3100	4306	4649	7359	3422	3714	3787	8444	6554	4892
201136_at	2851	1361	3812	1993	7745	3478	3348	4125	3376	3226	8290	8148	10959	10239	9079	5122	6167	4461	5068	5455	3592
201137_s_at	138	39	46	172	195	58	186	133	95	109	105	96	9	25	5	72	46	56	32	16	6
201138_s_at	1070	841	1781	841	1781	1638	1525	1459	2391	1804	1998	1919	3928	2797	2404	1572	1295	1421	3735	2380	2448
201139_s_at	1328	658	1299	1187	2110	2079	1308	723	1444	1421	1400	604	3384	2812	3238	1957	1365	1665	5086	2721	3072
201140_s_at	2824	1929	2328	2562	1861	1902	1395	1103	1444	1604	1893	889	1564	986	1440	916	1001	860	1648	837	831
201141_at	216	266	98	132	219	194	311	263	103	134	360	220	166	417	191	199	268	252	71	44	13
201142_at	1598	1253	874	758	1344	1340	2377	1564	1411	1066	1889	1248	1666	2594	1657	2362	2197	2155	1686	1290	1955
201143_s_at	448	246	193	226	1223	1434	596	602	440	354	659	708	1180	898	490	830	871	755	615	513	645

201144_s_at	3594	2923	3394	1309	4277	3725	4359	3172	3182	2402	5474	3969	6752	7100	9011	6179	6367	6514	6032	4774	4528
201145_at	1250	1513	4080	2099	1796	2884	2512	1822	2190	1746	2231	1570	2778	2797	3134	2638	3505	2139	2148	3749	2783
201146_at	4047	3731	3367	3639	3200	4257	2710	2881	1821	3121	2726	2035	4228	3880	2569	5419	4883	5907	6285	5197	7620
201147_s_at	9	16	18	17	363	968	34	21	15	11	502	348	160	105	54	61	28	40	8	7	5
201148_s_at	93	90	38	35	128	622	97	160	41	46	411	176	16	58	178	31	6	11	26	9	11
201149_s_at	180	130	195	190	498	945	322	282	193	222	662	369	246	97	100	8	7	27	61	32	30
201150_s_at	89	57	106	16	313	1504	106	119	79	34	253	457	156	176	115	37	16	46	3	3	13
201151_s_at	724	203	1390	892	473	349	453	673	1058	1029	681	557	615	311	77	253	167	286	671	234	435
201152_s_at	1717	731	3668	3638	1611	1236	1544	1676	3841	3526	2321	1347	2915	2073	707	962	879	1493	3149	2016	2824
201153_s_at	2350	1300	3315	3767	1812	1240	1420	1738	3479	2580	1678	1157	2572	2008	1834	3336	3316	3578	6027	3997	5050
201154_x_at	30650	27208	27455	27184	19346	22977	25728	25427	21597	20440	16473	23167	15936	22930	23497	24488	26863	24763	16834	25467	21338
201155_s_at	2389	1297	1243	957	2588	1795	2442	1487	1836	1121	2523	1715	1096	887	861	647	717	478	497	405	316
201156_s_at	1304	1305	1691	1384	987	981	1167	1095	1102	1347	1290	838	1480	1075	933	822	704	846	1126	796	660
201157_s_at	2295	1089	2003	1168	2548	1336	2185	1945	2427	1915	3319	2084	3376	3764	3370	3265	2947	2882	2885	1417	1901
201158_at	620	252	233	396	550	308	197	288	209	383	560	417	561	609	506	400	396	322	253	247	246
201159_s_at	287	67	297	216	100	55	248	329	329	352	463	376	57	49	45	21	7	46	60	14	51
201160_s_at	8751	10368	9041	6077	8303	6270	7309	7560	9039	10580	8418	8934	10405	11453	12060	10992	9774	9967	11550	13488	13612
201161_s_at	5268	5242	4583	3401	2995	2874	4657	4195	5860	5386	4492	4112	4525	5548	5360	3354	4150	2943	3707	3885	3524
201162_at	12	10	16	13	1692	219	19	16	13	14	1416	3515	2348	2905	2338	6	11	5	9	7	6
201163_s_at	5	24	50	5	2727	829	30	30	5	15	1077	4126	3083	2981	2013	16	19	8	15	1	1
201164_s_at	1537	1282	1122	1262	1502	962	1528	1696	868	1351	1215	1820	817	849	767	1173	848	779	823	1311	1434
201165_s_at	2002	2474	2250	2433	2135	2177	2050	2755	1806	3015	2381	2540	1751	1532	868	1379	1412	1586	2316	1923	1348
201166_s_at	2299	3345	1860	3104	2322	2763	1942	2313	2016	2495	2061	2556	3314	3070	5125	5563	5221	5230	5123	6362	8738
201167_x_at	978	809	998	542	712	736	1391	900	1141	1170	1489	861	148	43	113	197	99	189	118	72	70
201168_x_at	3620	4165	3493	1655	2139	1083	4479	3546	3940	3690	4451	1606	715	223	934	1151	642	1224	1239	734	555
201169_s_at	418	271	178	18	278	77	284	186	62	77	627	271	64	72	73	47	19	66	5	34	3
201170_s_at	1232	804	861	40	1647	1433	478	373	122	35	2221	768	1810	2196	3660	2222	2449	2250	388	634	595

201171_at	58	105	76	118	21	183	91	66	103	103	102	102	60	129	294	233	183	163	177	199	174
201172_x_at	3393	3343	4258	7069	4188	5740	4595	3955	4681	5521	6471	5360	5529	6643	4728	3625	3189	2625	3278	3073	2110
201173_x_at	4068	3956	2775	1617	3230	1762	4195	2831	3901	3481	3164	2033	4320	3478	3447	3312	3551	3292	2957	2216	1863
201174_s_at	1513	1308	1120	808	636	826	1093	982	764	908	1052	868	863	733	792	1651	1479	1684	722	1276	1271
201175_at	2021	1299	4293	2642	1967	1615	2572	2169	5186	3768	3104	2087	4283	4757	4379	3608	3722	3054	6068	4670	4328
201176_s_at	3096	2384	2960	2208	4492	4568	2410	2965	2688	2539	2885	2531	2232	1854	2348	2218	2030	2181	1850	1324	1979
201177_s_at	2199	1680	3561	1887	4074	3601	1487	1448	3417	3294	2685	3970	6917	6597	5382	3932	2922	3332	8871	7256	8841
201178_at	2417	2614	1784	2315	2120	2670	3146	2829	2181	2219	3088	2242	1863	2350	3038	2791	2785	2296	1987	2106	2068
201179_s_at	1287	869	1289	759	1491	1097	1773	1602	1410	1356	1316	1312	1289	1245	1153	1205	869	1210	1055	623	1380
201180_s_at	4817	4072	3940	2823	5121	3197	5304	4344	4711	4236	4299	4430	5402	4681	6305	6932	6615	6798	7491	6004	7997
201181_at	347	265	203	222	308	415	433	339	238	257	220	260	426	437	353	543	392	536	616	460	622
201182_s_at	569	454	640	543	429	232	804	1194	854	1092	995	532	684	574	388	302	304	183	349	412	185
201183_s_at	1856	847	1728	1474	990	1105	2347	2499	1947	1959	2029	1570	977	662	365	344	342	300	299	433	265
201184_s_at	2732	1794	2059	2065	1934	1584	2498	2741	2430	2018	2865	2131	1601	1323	1065	1053	966	990	1232	1884	1760
201185_at	261	213	262	384	331	556	249	259	324	330	539	406	203	209	305	14	7	102	67	152	138
201186_at	795	1321	761	776	1456	1462	1071	850	780	974	863	772	1266	1316	863	1042	895	1015	979	806	713
201187_s_at	204	238	100	269	412	348	315	396	262	214	186	147	25	31	50	57	26	11	51	26	21
201188_s_at	1233	1106	1327	1380	676	1035	1660	1605	1200	1572	996	529	293	337	399	339	414	588	417	409	481
201189_s_at	2109	2007	1957	2624	924	2113	2160	2164	1648	2566	1526	1196	1426	1501	1652	1843	2418	1701	1697	1447	1444
201190_s_at	945	1463	1202	1311	1707	1950	1560	1445	1405	1950	1671	2227	1783	1921	1929	1533	1698	1367	2057	2069	2494
201191_at	516	675	842	952	781	868	766	870	835	1448	1271	1330	783	577	522	516	456	400	741	847	779
201192_s_at	730	787	1032	720	1138	1153	1157	1174	1476	1614	1838	1238	1350	1267	1299	583	681	542	789	551	444
201193_at	4507	3604	3968	4639	3168	6660	3154	3255	3164	2527	2520	2733	3550	4005	4734	7607	7095	5061	5375	8554	11468
201194_at	1929	943	735	1749	770	648	2940	3130	1995	764	961	1200	1732	1319	1037	1543	1820	1460	1790	427	315
201195_s_at	6799	12122	6431	363	6603	4564	7546	2706	3604	1166	7500	2545	5670	4691	6398	6645	6228	6434	3446	4046	3124
201196_s_at	1087	498	2092	891	1706	1015	1439	1536	2489	2475	2280	1674	1786	1974	2487	2884	2499	2295	3218	2287	3552
201197_at	1024	614	1409	764	2243	1412	1104	1048	2205	2217	1695	1594	3568	2845	2208	3908	4315	4319	7019	4306	7751

201198_s_at 2445 1470 2626 1750 2749 1400 4528 4130 2984 3045 4063
2994 2932 2588 1740 1969 1662 1882 2279 1105 1233
201199_s_at 3110 2205 3059 2124 4102 1923 4656 3496 3951 3602 4571
3314 3369 4931 4569 3460 3140 3554 3707 1932 1651
201200_at 2539 4075 2014 2406 1828 3428 1650 2578 1054 2541 1495
2836 2465 2402 4435 5996 6315 4664 6438 8110 9309
201201_at 7061 7788 9016 10424 4513 6182 6826 8019 9011 8618 5108
5728 8004 5693 7351 6324 5406 6190 5890 5332 4348
201202_at 5580 2781 3903 1887 5406 2014 3204 6160 4717 4363 7105
7141 10383 8397 9659 8255 6782 7423 9578 6477 7540
201203_s_at 393 557 182 141 470 543 363 402 127 212 323
34 66 29 5 6 21 12 11 19 2
201204_s_at 2139 2635 619 555 1919 2563 1764 1463 763 718 1676
465 1066 727 508 520 512 422 343 602 411
201205_at 261 213 239 225 513 509 183 227 190 218 209
216 53 60 52 69 45 103 44 65 63
201206_s_at 663 901 464 494 232 473 668 586 309 454 514
320 487 328 84 57 84 65 29 62 72
201207_at 976 1097 1500 1516 1561 1716 1346 1205 1053 1514 2083
1513 858 923 1192 610 698 704 814 681 684
201208_s_at 262 174 415 322 294 386 421 399 244 449 699
478 121 107 98 72 75 106 109 86 78
201209_at 5551 3690 4235 4278 2961 2725 5683 6304 4366 4669 2898
2701 4007 3568 3889 7802 6985 6965 6747 4515 4805
201210_at 6553 5720 4748 3505 8305 7464 4802 5603 4153 3782 5104
5143 10751 8068 6809 7429 6619 6139 7571 5334 5666
201211_s_at 1523 1594 732 663 1262 969 1358 1033 993 1001 1526
992 145 159 168 244 248 189 184 105 143
201212_at 880 752 975 1090 1017 1465 628 938 729 1078 964
831 807 702 552 348 454 371 705 1219 974
201213_at 234 186 225 340 27 20 232 169 174 334 239
269 225 118 196 135 128 56 314 312 420
201214_s_at 802 449 803 1218 950 876 837 748 831 883 802
1115 1457 1440 1224 1012 1072 750 1614 1813 1538
201215_at 2355 2878 4209 6058 3142 4823 2086 2152 3120 2110 3390
2256 4743 3899 6292 4653 4300 4523 7537 7912 11544
201216_at 5929 5821 3513 4149 1949 2607 5087 3694 2723 2630 2477
1950 3847 3079 1559 2606 3167 1716 1385 2176 1506
201217_x_at 36160 38743 25109 25029 22046 32814 32351 33271 28214 24415
19964 26040 17953 23464 24250 30854 32582 29838 16613 24227 18750
201218_at 4808 4986 3367 2579 2276 2524 2600 2519 2673 2604 1720
1967 3111 1725 2609 4650 5032 5408 6595 7064 7928
201219_at 647 665 677 563 243 366 759 586 582 685 445
349 436 384 1021 1234 1539 1413 1634 1397 2070
201220_x_at 3986 4186 5271 4044 3185 3237 3351 3122 4589 4224 2672
2041 2903 2563 3662 3596 3721 3698 5161 5916 6515
201221_s_at 2531 2248 3464 2748 1486 848 3409 2532 5176 4423 3354
1355 1822 1592 968 736 442 340 1436 600 640
201222_s_at 4096 3331 3841 2258 5701 5187 3407 4162 4580 3712 5104
4813 6359 4557 3293 3932 4399 3736 3824 2759 2484
201223_s_at 1793 1523 1743 1169 3111 4451 1654 2099 1751 2221 2946
2311 4866 4222 3475 3270 2662 3595 2865 2464 2854
201224_s_at 726 389 621 612 121 224 1203 1099 1069 813 712
719 321 500 520 755 764 477 598 426 351

201225_s_at 3822 2106 2256 2562 1366 1282 5010 4370 3310 2995 2655
2360 3019 2347 3327 7225 5799 7333 5967 2779 3494
201226_at 6575 2635 5293 3347 1310 1609 6679 4228 3255 2145 1327
1844 1653 2254 1061 3421 2579 2868 2092 2319 1357
201227_s_at 5900 3078 4042 2762 1764 2154 4497 3531 2812 2316 1531
1717 2118 1936 2053 5248 4351 4515 3439 3169 2026
201228_s_at 480 241 473 362 383 603 342 569 595 612 476
353 275 215 149 103 66 122 175 157 141
201229_s_at 721 318 880 554 964 1032 535 509 712 797 1008
875 1080 769 653 217 137 176 482 350 254
201230_s_at 818 734 826 855 917 1240 1243 941 941 1256 964
1087 637 803 798 622 872 434 858 802 971
201231_s_at 19831 9042 15400 4993 13852 6613 15950 16971 13690 10966
15709 10564 16997 18631 15847 19167 17091 18887 12647 11506 11443
201232_s_at 1644 501 1009 676 1893 1236 2198 1434 1532 1094 2292
1973 4368 3540 1279 1094 1339 1406 1679 1093 911
201233_at 595 179 368 308 1238 1078 727 474 724 435 1103
899 1674 1292 553 448 354 448 446 300 268
201234_at 1577 1656 2657 2590 1226 1911 1255 1818 1726 2014 2646
2036 1369 2048 2344 1320 1435 1119 1607 1645 1562
201235_s_at 191 322 231 780 473 429 395 319 122 368 205
187 56 49 32 69 55 55 28 37 64
201236_s_at 665 654 320 2407 458 297 905 819 474 822 441
503 49 145 169 628 625 309 636 562 498
201237_at 687 654 1365 1001 1274 1106 656 702 1164 1651 911
1028 2602 2496 1950 1962 2023 2841 5766 2494 5555
201238_s_at 2557 2392 3485 3084 3434 3842 2271 2084 2966 3905 2751
2359 6215 4891 5561 4927 4037 5389 7122 5932 8426
201239_s_at 2541 1545 5787 4715 3207 3950 3421 2470 5927 5291 4242
3237 4372 3985 3326 4216 4682 3685 5038 4612 4693
201240_s_at 4925 4210 8475 8234 5902 8739 5141 4521 9790 7870 5334
5432 6707 9261 10328 11759 13986 11728 10811 13523 13586
201241_at 2606 1890 4101 1989 3544 2659 5376 4815 5809 4722 4843
3761 4972 5523 7367 6107 5913 6801 6843 5127 6897
201242_s_at 1039 1401 1928 3685 617 499 739 946 2103 3451 1067
703 832 745 413 1398 1249 1086 3239 3253 2538
201243_s_at 1856 3521 3451 4114 1068 839 2373 1846 5175 5938 1896
1360 932 678 916 3767 2782 2931 8404 7631 7680
201244_s_at 1589 1395 1332 1482 1817 1183 1703 1797 1424 1433 1873
1313 789 950 715 863 846 1010 1193 963 1059
201245_s_at 1606 1210 1799 2148 1478 1429 1632 1318 1754 1688 1778
993 1467 1410 1512 1317 1295 1406 1447 1464 1221
201246_s_at 455 505 492 466 441 258 539 538 545 478 587
200 177 51 143 155 92 104 193 148 134
201247_at 874 880 818 846 590 464 505 497 649 650 568
319 148 249 182 295 424 409 367 227 211
201248_s_at 815 933 846 833 503 305 775 777 677 739 748
398 144 233 193 234 243 217 118 101 100
201249_at 544 108 186 189 855 993 452 203 174 340 696
523 491 515 570 175 143 187 167 170 209
201250_s_at 3512 1796 2797 2642 8012 11599 3225 2978 2328 3838 8256
9706 7033 7360 9955 2665 2805 2461 2521 3730 3731
201251_at 14924 9586 17889 12872 7461 1938 17197 16748 20251 17769
16635 10779 1857 3284 6109 5963 6316 5768 5229 3263 2924

201252_at	1846	758	1693	1015	1894	1309	1338	1395	1932	1138	2641	1420	3913	3545	2749	1841	1580	1665	2171	1805	1787
201253_s_at	2331	2216	1249	1548	2399	2061	1459	1357	1374	1863	1222	1949	2988	2198	1364	3460	2346	3407	4096	4181	2880
201254_x_at	24935	38971	30973	38421	29386	35163	36462	34698	33236	29203	24042	34205	22285	31343	34804	31626	34372	36607	20729	31137	26189
201255_x_at	3309	2495	1579	1650	1900	1309	3274	3253	1361	1901	2491	1294	1174	1043	1907	2419	2031	1666	1349	1614	1279
201256_at	2713	3969	4413	5961	4159	7480	4178	3478	3781	3856	3725	3687	4014	4140	5337	5234	5605	4571	4675	5934	5272
201257_x_at	30142	48489	30768	33144	30096	38752	38700	38098	26942	27257	29054	39828	24203	34415	36925	36871	38786	42103	21360	34296	28437
201258_at	21221	17860	18172	25664	16733	24375	17806	17635	16922	17944	13936	24498	20363	22855	13953	16838	14763	13443	12829	19032	15123
201259_s_at	1024	1322	3615	2570	2667	2106	3131	3333	5148	4224	5949	5383	3960	3435	3485	1894	1993	1879	2891	1762	2828
201260_s_at	2298	1691	2641	1849	3729	4619	1768	1950	2198	2148	2403	2735	3867	3839	3473	3883	3192	3867	4864	3834	5177
201261_x_at	284	193	499	457	393	232	286	263	692	544	155	176	2	64	58	69	73	68	392	323	335
201262_s_at	24	34	256	224	271	238	8	70	649	338	48	6	4	11	21	47	24	59	82	35	69
201263_at	3571	3145	6725	2643	2884	3444	5105	2680	5896	4187	2884	1886	3537	2784	3496	4776	4433	4947	6821	7499	8396
201264_at	4050	4754	2712	1853	238	878	2572	3010	1180	2257	1839	2555	1362	1540	2114	3087	3898	2706	1578	1815	1125
201265_at	189	168	205	146	383	488	171	228	214	177	115	155	19	22	14	41	20	7	8	13	19
201266_at	8307	11487	8240	9330	3835	7274	8342	7334	6549	8113	4770	5956	4029	3437	3151	9320	9127	9390	5830	4991	6239
201267_s_at	2274	433	2127	1057	3828	1724	2059	1477	2537	2102	3384	2105	5933	5834	2397	1499	1677	1381	1970	1286	947
201268_at	17829	20557	14260	8466	8367	5787	17075	14411	14535	10447	11989	11129	12756	13924	12269	14336	13995	16121	9576	10978	6678
201269_s_at	188	193	96	291	471	527	138	269	173	212	146	231	45	48	57	19	59	59	46	49	34
201270_x_at	1610	1153	1388	1252	1528	1529	1280	1421	1205	1479	1977	1704	1004	992	1201	1099	945	1240	1138	912	1028
201271_s_at	794	732	1289	1201	1549	1089	739	772	1323	1395	1688	1436	2435	1860	1369	440	421	263	755	628	445
201272_at	2533	2063	260	247	231	163	2931	2681	226	278	262	199	92	104	65	2783	3004	2553	182	415	444
201273_s_at	6738	5520	6933	5991	7451	6504	5956	6723	6647	6060	5935	9166	12316	9270	7848	8416	7738	8368	11899	10084	14344
201274_at	3223	2510	4851	3413	3387	2110	4379	3781	5588	5449	5135	3591	4433	5357	6389	5091	4978	5316	5570	5212	4136
201275_at	1671	682	2363	1141	3883	1379	1640	1408	2007	1194	2206	1840	4859	4820	4791	3636	2510	2214	2730	3311	1741
201276_at	849	658	1337	1827	1526	1567	398	814	823	1045	1474	1273	591	509	494	474	476	493	583	705	701
201277_s_at	8024	5093	8044	3078	10635	4342	7612	7167	12958	9920	11428	9075	14924	11902	9883	6608	7634	5875	10202	6480	4875
201278_at	282	200	308	488	11	17	278	238	203	291	222	221	40	29	12	137	152	154	139	187	248

201279_s_at	192	142	270	678	142	133	189	268	203	415	108	83	10	45	64	77	91	113	105	142	153
201280_s_at	24	55	76	290	73	99	98	79	17	179	30	132	23	22	3	20	2	42	63	88	131
201281_at	3487	3349	3144	2118	3582	1662	3759	2586	3798	3474	5499	3511	5309	4011	1764	836	1139	1325	1977	1154	946
201282_at	685	1068	1320	1658	137	141	1114	637	995	1423	1939	938	145	195	212	116	91	131	245	143	149
201283_s_at	19	44	206	437	126	79	60	18	173	287	206	121	28	38	41	1	14	1	90	47	44
201284_s_at	2320	1527	2421	2018	2054	1434	1721	1993	1876	2161	2008	1541	2170	2569	2060	1514	1850	1756	2537	1695	1183
201285_at	1150	1624	1735	2990	983	2086	1966	2278	2274	2924	2325	2338	1649	1444	1696	1578	1770	1138	1886	2262	2529
201286_at	300	271	723	901	1069	1118	415	249	1385	843	829	538	758	478	649	293	368	379	968	581	494
201287_s_at	1368	870	2887	2456	1999	1907	1363	1102	2517	2261	2888	1193	947	660	581	513	484	375	946	739	562
201288_at	11	129	1200	1393	36	24	27	25	863	551	13	6	4	4	2	92	57	87	2581	967	816
201289_at	652	2092	1004	550	1526	3768	362	471	206	305	1781	4102	1940	1356	698	1707	1555	1693	665	779	731
201290_at	4647	5585	8877	9283	4352	4386	4721	5747	10139	9171	4657	5728	8112	8437	7839	10063	11202	10181	11943	18056	13855
201291_s_at	168	52	1544	155	1765	35	747	1360	1679	2008	2690	4340	5182	5737	3399	836	677	637	2930	1147	1384
201292_at	893	428	2866	453	5637	267	1521	1949	2054	2491	4602	4500	6539	6848	6255	1971	1524	1897	5974	1885	3619
201293_x_at	30586	29540	34550	32247	37089	29702	38802	37814	36748	33375	34041	39563	24123	31623	36132	29130	32591	32708	19451	27338	22004
201294_s_at	221	185	103	215	146	529	256	346	146	207	212	229	66	79	73	82	167	62	64	76	90
201295_s_at	69	62	15	116	76	306	44	190	47	71	27	133	18	43	28	62	34	44	31	2	28
201296_s_at	1353	1288	1042	876	863	2310	920	1368	749	830	833	834	650	697	1492	2549	2317	2047	2309	1785	2356
201297_s_at	706	468	479	396	816	824	570	558	447	588	762	584	772	665	740	423	371	531	495	368	604
201298_s_at	2310	2257	1483	1156	2884	2141	1662	1795	1659	1500	2895	2291	3130	3074	3353	2270	1908	2151	1919	1224	1447
201299_s_at	1016	371	784	832	900	974	912	928	535	748	866	598	404	354	114	139	125	166	184	118	149
201300_s_at	4933	4441	2858	838	5854	4779	2476	3018	1386	2163	4042	5923	6667	5592	4411	5956	5736	5857	4477	6931	9635
201301_s_at	1859	2072	4990	16433	1585	3700	1734	1859	4937	8212	1398	1958	1821	1585	2761	2005	2347	1792	8014	7788	8450
201302_at	1386	2664	4521	14240	1013	2334	1431	1424	3643	6704	1225	1578	1301	1292	4057	3322	2786	2839	9975	9083	12760
201303_at	7115	7149	2976	2962	4390	4294	8831	7276	5389	5551	6906	7262	6752	6081	6929	7035	6405	5850	3990	3533	2389
201304_at	1218	1163	1630	1474	1362	1989	986	1367	1219	1431	1104	943	2923	1781	1244	3450	2503	3229	5699	3604	5113
201305_x_at	3137	1748	5867	1747	2697	2061	3714	3119	4644	2987	4960	3352	9573	7974	4577	2840	2495	2431	5851	6163	4478

201306_s_at	9186	8631	13347	7496	11881	8064	10629	7676	15571	10298	11311	9588	10911	13418	14877	11646	11987	13130	11503	14956	14519
201307_at	1969	1210	1537	1474	1861	1270	2361	2346	1674	1761	1894	1514	1225	1793	1530	1844	1650	1843	1305	1046	988
201308_s_at	136	66	213	131	250	130	329	467	318	215	372	269	131	97	64	68	78	97	120	66	142
201309_x_at	164	300	184	345	139	281	248	388	238	183	226	112	66	63	45	121	121	87	81	73	74
201310_s_at	352	794	312	1265	517	1199	723	757	496	250	343	358	223	228	361	713	728	638	492	695	977
201311_s_at	361	859	555	2023	140	247	791	1056	561	583	85	178	64	49	14	851	794	822	875	878	972
201312_s_at	1427	2669	1567	5953	300	277	1391	1878	995	1367	136	118	80	58	39	3415	2662	2333	2983	3282	4026
201313_at	771	626	555	437	471	675	868	876	214	748	1042	759	775	623	515	333	596	285	158	186	170
201314_at	808	1206	556	731	477	699	603	655	914	742	416	432	395	352	203	402	335	465	501	236	205
201315_x_at	5370	9221	1572	1296	6082	6450	6392	4708	2214	1625	12598	5547	13603	13026	12633	7370	9546	14851	3093	5955	4235
201316_at	339	428	411	513	797	434	497	544	605	570	785	640	1379	1074	892	691	718	637	1204	659	852
201317_s_at	4356	3709	5807	4741	7527	6231	6705	6224	6290	5579	9613	9647	12828	15156	10150	6863	6677	8065	8029	4786	4411
201318_s_at	5751	6797	10850	11750	9578	12326	5588	6742	7646	12358	7605	10547	14213	13900	9405	8552	8079	8616	12362	13343	10726
201319_at	1676	2753	2002	2408	1731	3005	1859	2212	1731	2242	1876	3520	6600	2270	3859	3510	3049	2447	5274	2953	3593
201320_at	594	433	496	833	526	402	460	527	507	588	628	557	280	159	403	206	317	183	344	368	332
201321_s_at	377	566	688	762	1086	817	376	391	504	580	611	851	722	443	317	225	220	249	632	577	650
201322_at	13763	13486	14292	11694	9502	7737	20823	11719	20756	15100	15083	11129	13023	13073	12728	12185	10787	13646	11792	12806	12470
201323_at	1413	1036	1324	612	1898	1510	1416	973	1607	1360	1472	1680	3016	2650	2775	2668	2584	2765	4108	2611	2798
201324_at	599	442	770	171	2357	1294	288	132	399	590	1961	2725	3590	3634	2488	1433	1469	1176	1501	962	1079
201325_s_at	393	410	520	189	624	810	302	269	535	495	1467	1971	989	1246	787	551	714	545	451	300	229
201326_at	4572	6164	2417	1339	6449	2932	3784	3089	2031	2394	5158	3701	5642	4960	7623	5774	4292	5360	4484	4404	5410
201327_s_at	6029	3916	4507	1992	6595	3769	3990	4005	4755	3445	5322	4549	9979	9434	6168	5830	4888	6240	8593	5754	7754
201328_at	574	560	2079	4845	1736	1701	612	429	3852	3695	1268	969	1348	673	1084	431	328	399	4616	4579	4599
201329_s_at	790	676	1671	3205	2060	2125	666	468	2878	2995	1037	632	355	289	241	326	161	301	974	879	933
201330_at	1184	1134	1308	1331	1796	1764	2100	1854	1477	1793	1831	2001	5416	4676	6742	7064	6195	5903	7333	5172	7407
201331_s_at	812	1298	1729	3313	517	1189	655	534	1925	1600	958	661	583	877	1475	794	925	810	2102	1654	1302
201332_s_at	193	163	290	715	60	50	172	61	415	436	198	147	14	5	6	4	6	7	24	28	5

201333_s_at	275	209	331	365	76	69	314	340	306	433	452	290	172	220	145	247	171	178	135	151	176
201334_s_at	1627	2396	1546	3132	1384	2506	1927	2531	1101	1897	2592	2989	1282	1112	1583	2152	1888	2212	2177	2382	2860
201335_s_at	22	68	39	119	181	271	41	59	13	66	105	112	70	72	107	67	33	78	118	73	112
201336_at	3143	2692	2438	3613	2058	2688	2496	4038	2328	2728	4002	4488	4992	7339	6921	6441	7889	5317	5212	5768	6594
201337_s_at	1134	415	864	1045	895	997	1579	1499	1524	1121	1674	1491	867	1080	706	1462	1240	1124	1076	884	1262
201338_x_at	5738	6731	6278	3748	9524	12848	5361	4281	6177	5077	8634	8468	11282	14343	17973	8567	10787	11061	10068	10487	11020
201339_s_at	1425	1994	1957	4198	1666	2927	1663	1316	1762	2008	1376	1650	1876	1700	1864	2738	2585	3081	3018	2501	3400
201340_s_at	191	180	507	806	179	48	130	153	737	935	398	770	303	169	133	67	82	44	199	206	122
201341_at	489	472	4417	4450	2574	3456	702	883	4914	6879	3145	4321	2117	2196	1734	1162	987	822	2624	2094	1768
201342_at	2631	1045	1559	1108	1946	1420	1878	1977	1441	1083	1660	1669	3072	2257	2295	1641	2012	1929	1476	1260	943
201343_at	1668	2387	2564	1732	2926	2568	1931	2066	2154	2345	3087	2498	3145	2903	4096	2622	2585	2178	3146	2460	2276
201344_at	210	223	321	269	465	548	211	191	261	261	355	480	448	507	578	255	223	193	537	443	597
201345_s_at	2705	2983	1906	1434	4064	2412	1974	1818	2472	2320	4359	3079	4480	3781	8127	3095	2654	2815	2862	2683	1927
201346_at	1105	1208	1918	1780	3824	2580	1538	1987	2066	2075	2594	1964	2158	2777	2568	1567	1650	1239	1696	1305	1554
201347_x_at	2151	2309	955	1045	1112	2205	2413	2122	1157	1062	2083	1873	3079	2672	1711	1243	1426	1116	983	827	805
201348_at	239	94	1652	5978	1248	2938	239	168	1238	1670	2511	1551	763	1057	941	19	21	15	867	409	460
201349_at	2101	1598	2234	3249	2169	1324	1507	1763	2071	3297	2066	1976	704	1099	1440	2052	1358	1688	3747	2000	1983
201350_at	1073	1209	1111	1937	737	525	1676	1357	1758	2099	1693	1131	506	697	515	502	460	448	982	548	606
201351_s_at	4802	3403	4204	3201	3410	3051	4073	5079	3737	4108	3482	2715	3512	2816	3028	4269	4341	4928	4560	4189	5302
201352_at	4159	5022	3717	3210	2882	3323	3750	3890	3739	3547	2678	2945	3261	4267	4231	9538	8081	8690	6436	9073	9766
201353_s_at	511	603	750	731	535	442	692	644	458	604	713	564	149	192	171	98	168	119	57	77	12
201354_s_at	327	27	46	370	730	740	390	208	401	528	492	337	339	185	120	83	206	180	117	198	78
201355_s_at	520	428	343	388	223	181	453	463	333	380	324	356	104	182	68	134	101	90	107	106	31
201356_at	2470	2580	1862	1314	2059	1598	4206	3583	2887	3034	2766	2082	2231	1298	1264	2431	2444	1917	2783	2359	2376
201357_s_at	1102	696	859	608	429	93	1134	1044	1145	1191	1103	748	256	216	148	147	157	188	280	217	137
201358_s_at	5411	6169	4167	3919	4985	7169	3991	4489	3424	3761	3972	5092	6185	6005	8132	9492	9260	11260	7948	11185	13863
201359_at	3215	3322	2973	2905	3686	5370	2930	3461	2050	2324	3040	3738	2876	3076	4266	5258	4688	4960	3937	5253	7324

201360_at	1633	2747	1270	2372	2300	4275	1848	2192	1259	2004	6731	5726	5024	3299	5783	833	1217	1249	1136	1247	610
201361_at	967	749	1450	1287	1076	543	1399	1086	2295	1544	1904	1018	682	849	697	687	731	551	911	463	338
201362_at	409	892	707	1102	466	1313	484	409	880	893	300	398	391	493	426	1043	856	831	1803	1061	1591
201363_s_at	452	709	720	767	386	798	388	534	765	871	335	390	347	169	115	409	454	421	1127	598	906
201364_s_at	1986	1647	1356	2319	1260	855	2182	2569	1945	1838	2052	1262	814	552	303	517	477	551	292	153	144
201365_at	1221	1079	819	1469	854	867	1305	1485	918	1152	1198	833	536	423	165	255	301	427	247	174	128
201366_at	1941	1253	1602	1474	1942	3052	1125	1017	1264	1391	1329	1700	1873	1749	1303	1728	1471	1514	3824	2498	4120
201367_s_at	122	49	445	714	11	86	175	133	338	362	198	31	4	25	27	24	3	2	55	18	50
201368_at	1163	3339	1941	3751	805	1803	1334	1393	2139	2437	1373	948	1009	862	1432	3042	2958	3183	5224	4479	4933
201369_s_at	106	195	309	619	610	532	62	172	466	344	179	72	91	52	65	69	45	55	258	160	141
201370_s_at	386	364	265	215	426	496	348	334	235	277	202	276	424	234	223	636	408	340	649	264	513
201371_s_at	3268	3635	3502	2911	4397	3216	3836	3659	4938	3317	3751	2860	3820	3263	4208	4782	4877	4922	5749	4075	5681
201372_s_at	5	39	16	13	136	99	8	55	7	59	10	85	25	6	34	36	19	35	45	32	44
201373_at	839	843	549	737	414	179	1007	908	514	910	920	457	220	169	321	216	220	382	235	265	371
201374_x_at	7	7	53	8	191	71	11	9	5	13	9	5	41	7	15	48	25	41	28	31	3
201375_s_at	4081	4647	3487	2981	2107	2743	3400	3872	3369	3560	2597	2238	4455	4811	4513	7824	7594	7270	6128	4670	6245
201376_s_at	4118	732	3599	2368	2367	1506	3221	3108	4699	4766	2882	2715	1811	1748	1156	1440	1389	1260	2049	1352	1580
201377_at	1032	311	1154	652	1575	572	1811	1099	2007	1087	1410	1046	1021	1020	1573	1042	925	939	1758	1103	1542
201378_s_at	2114	440	2101	1104	1332	310	3968	2784	3167	1989	2270	1195	370	382	558	473	422	346	766	383	615
201379_s_at	2774	2281	3601	2266	2280	1939	1924	1137	2721	2296	3117	2338	2562	1858	1543	1276	1290	851	1704	1384	1286
201380_at	912	796	453	519	800	1355	812	670	532	730	1174	885	704	663	541	492	517	570	271	333	287
201381_x_at	5025	3132	4407	2193	2862	1696	3980	3589	5199	3876	3425	3212	4980	5018	5417	7015	5574	6144	8993	5017	4722
201382_at	110	100	102	145	465	254	78	190	157	148	95	98	72	85	41	89	100	174	120	156	127
201383_s_at	708	219	639	1383	753	892	839	829	573	1008	1012	976	292	250	179	146	140	159	191	171	167
201384_s_at	1311	1276	1157	2859	1431	2059	1080	1425	856	1377	1303	2312	1354	1146	1031	1149	1099	1028	1356	1297	1378
201385_at	5726	4025	5258	3480	4840	2467	6584	7739	5124	5792	5322	4142	6875	5247	8685	11161	9413	10039	10481	8305	11830
201386_s_at	3221	2060	3799	2321	3079	1353	7250	4947	4932	3804	3864	2417	2554	3075	4512	5743	5714	6138	5322	3393	4415

201387_s_at	183	130	178	116	249	375	204	247	222	205	416	357	181	176	135	10	10	46	52	9	7
201388_at	2507	1384	1590	979	1937	1054	2634	1903	1604	1673	3229	1545	2643	1963	634	400	466	414	425	338	171
201389_at	680	504	123	185	976	962	678	591	84	165	388	297	150	263	262	317	654	410	74	92	60
201390_s_at	6387	5479	3951	3888	2334	1782	5338	5512	3026	2848	4804	4314	4777	4643	6157	7230	7698	6683	3803	3977	2049
201391_at	4719	3782	3501	1808	2261	1556	3317	2949	4390	2918	1848	1738	1733	1460	2470	3626	3768	3871	4333	2868	2995
201392_s_at	430	791	667	533	340	947	554	569	448	727	614	555	509	548	70	53	90	59	39	149	100
201393_s_at	1656	2356	1740	1107	1321	3393	1215	969	1112	1776	1434	1059	1326	1008	391	328	339	213	625	1207	785
201394_s_at	1207	611	849	1267	603	732	924	1193	578	753	820	804	302	360	190	301	257	280	151	193	166
201395_at	1478	1077	966	1220	1134	1697	1157	1182	776	1329	1155	1160	1147	1260	1360	1285	1402	1287	820	966	1020
201396_s_at	631	742	302	347	173	477	660	418	264	404	466	418	329	84	213	157	127	256	194	100	129
201397_at	2694	2519	1839	396	1843	2170	3275	1152	987	830	1151	846	1370	1049	1322	3014	3519	2167	904	1992	1600
201398_s_at	5772	6363	4053	4386	2238	5238	4471	4242	1955	2668	2714	2873	3024	3518	4566	11318	11672	11288	5020	9425	11314
201399_s_at	4903	4954	2990	3249	1632	2618	6180	5563	2368	2854	3029	2644	803	842	874	5406	4446	3899	1086	1494	3456
201400_at	9315	3171	10648	9027	6189	5467	8402	5239	9163	7558	7751	8914	12785	12442	9247	7415	6768	7009	10261	8637	6105
201401_s_at	337	248	583	446	246	165	486	400	690	762	593	185	42	54	7	36	43	70	109	84	77
201402_at	38	29	23	30	427	153	42	54	27	25	23	24	11	33	14	18	63	77	20	76	17
201403_s_at	3850	3080	3067	4157	3300	3847	3772	3989	2024	2819	3989	5479	4555	4363	3289	2796	3075	2966	1342	2094	1208
201404_x_at	1300	757	955	881	1211	676	1786	1054	1278	952	1005	801	385	708	351	681	511	425	618	346	288
201405_s_at	2989	1856	3132	2962	5712	5066	2960	2134	3234	3216	7056	7235	6866	6190	5729	1932	1866	1653	2414	1954	1146
201406_at	16684	30035	24378	26119	32080	29207	20910	21189	21195	23948	21841	32191	25187	29631	25361	22020	18893	16716	17833	26328	15673
201407_s_at	1442	1068	817	560	1200	1910	743	1075	518	604	985	896	2228	1712	1158	1590	1152	1394	2371	1634	1712
201408_at	447	428	650	476	819	1141	526	540	368	373	925	711	1184	1208	987	717	650	699	1440	1229	1641
201409_s_at	758	731	1017	1142	1381	2141	876	1041	673	826	1340	1124	2088	2245	1873	1202	1275	1550	2774	1582	2170
201410_at	2854	2713	4193	2385	2941	3463	2625	2210	4232	4240	2579	2623	3452	3385	4568	3699	3254	3088	4967	4214	5328
201411_s_at	890	640	1241	909	1190	1498	359	652	1053	929	708	1087	1791	1836	1330	300	224	429	1251	884	1039
201412_at	1078	2338	1674	2385	906	1235	1293	1252	1330	1673	1339	929	816	597	683	1385	1126	1149	1528	1622	1384
201413_at	1853	1435	2368	2813	1087	1332	1530	1812	1956	2546	1116	1294	1188	1457	1644	2815	2354	2798	3349	3350	4208

201414_s_at	1451	1573	1138	762	2282	1424	1242	1306	1192	1233	1511	1636	1570	1396	1386	852	829	756	633	381	454
201415_at	1697	1478	1283	1315	2176	1692	1497	1148	887	1087	1829	1680	3195	2549	3039	1652	1875	1626	1389	1155	1477
201416_at	1195	3658	1978	1937	524	495	1480	3346	768	609	486	283	1070	431	552	3847	2890	3645	722	2209	1313
201417_at	1614	3429	3443	3185	859	939	3938	5947	1968	1043	962	681	1828	2726	3500	12779	11730	12133	4208	6448	7761
201418_s_at	679	1224	669	389	176	118	1171	1459	488	89	256	192	113	64	68	209	116	34	15	102	45
201419_at	922	695	1099	790	979	925	1080	885	991	830	1347	804	596	387	453	168	166	207	405	315	224
201420_s_at	1955	1283	2465	1084	1277	949	2490	1269	2632	2122	2024	1273	1229	1054	1029	931	867	1038	1290	834	693
201421_s_at	1490	1179	1591	1051	2136	855	1842	1387	2431	1796	1873	1532	842	621	596	603	544	1006	1016	1087	672
201422_at	1470	2356	830	1154	609	1153	2356	1318	1220	932	779	910	866	735	802	986	1304	1321	1133	591	632
201423_s_at	906	886	2151	1124	1269	1334	1165	1081	2696	2180	1450	1443	964	953	814	755	651	748	1668	1440	1768
201424_s_at	675	580	722	457	980	1669	253	422	670	655	429	709	1298	1174	1461	1167	1034	1009	3243	2726	3673
201425_at	1979	2429	3338	5710	1944	2391	1758	1335	2848	2847	2061	1655	962	813	913	1006	1146	851	2202	3265	2410
201426_s_at	280	190	255	70	13182	7511	273	240	261	363	12323	18912	11667	14765	8759	189	215	165	447	1616	1293
201427_s_at	50	359	15	1032	10	74	163	369	4	13	36	11	34	27	70	549	532	513	105	458	519
201428_at	935	1524	970	1719	1483	1183	504	711	680	948	1084	524	924	628	322	347	292	271	235	400	316
201429_s_at	31071	44593	29866	41860	38335	50457	35868	36097	32182	29510	26640	41364	27370	32839	34883	33638	36965	37236	21549	27906	25097
201430_s_at	247	54	79	84	289	50	419	300	60	161	179	331	74	37	53	59	37	8	53	17	34
201431_s_at	360	376	94	80	401	199	379	622	171	433	294	896	83	68	105	163	132	178	60	115	112
201432_at	3548	2999	3262	10161	1218	1630	4586	4678	3648	4155	1511	3035	1613	1404	1117	2558	2372	1878	4891	1980	3927
201433_s_at	3702	3239	3601	1119	3264	2285	4430	3308	6588	4298	5413	3034	3823	4887	4994	3710	4119	3440	4766	2721	2293
201434_at	663	774	1015	1001	1762	1164	1026	793	950	830	1617	1810	2173	2395	2366	1466	1524	1309	1202	1206	765
201435_s_at	1560	1147	2636	1273	2397	1618	3599	3242	2507	2176	2379	1703	2255	2031	2544	4054	3536	3426	3926	1542	1809
201436_at	483	426	174	138	265	466	309	342	185	143	151	171	412	470	445	878	683	906	650	318	506
201437_s_at	1306	651	1089	423	1376	1039	942	945	547	709	664	643	1558	1405	1072	2075	1664	1666	3392	1363	1805
201438_at	40	45	20	40	184	35	42	88	27	59	179	186	63	57	47	21	41	12	9	6	3
201439_at	1146	1199	798	722	412	343	1104	1053	535	760	611	327	104	115	90	191	222	257	189	145	138
201440_at	1124	673	1550	1297	1113	606	796	1093	1474	962	1744	995	970	935	740	604	766	586	1083	811	999

201441_at	2877	4081	5811	8362	3803	4447	4321	4328	5551	4492	5277	5731	8832	7526	5504	3648	3881	3180	5096	4646	2243
201442_s_at	180	152	225	195	202	47	133	131	151	124	136	178	22	43	30	5	4	18	6	4	2
201443_s_at	2676	3333	1679	2355	4100	6176	2237	3325	1025	2735	3839	4424	7864	6972	8255	5926	7165	5976	4319	5435	8337
201444_s_at	1548	1999	1027	1252	2588	3032	1913	2885	1227	2268	3048	4521	3721	2732	1526	1123	1182	1086	903	856	1359
201445_at	2021	3888	513	565	181	167	1709	1818	391	316	98	107	37	25	28	4596	4446	3876	929	1246	1200
201446_s_at	1065	929	1384	1750	1032	845	1232	1473	1165	1490	1317	1078	216	248	353	805	770	805	522	430	802
201447_at	263	353	434	916	434	622	407	524	290	404	426	375	154	180	246	368	354	312	286	301	558
201448_at	500	530	612	1385	436	673	550	868	491	590	389	836	557	412	408	472	396	376	913	1067	1471
201449_at	155	206	320	679	377	508	213	355	399	302	317	254	216	197	168	233	248	212	341	353	443
201450_s_at	430	472	480	651	312	585	314	590	368	453	399	427	211	179	159	363	303	345	634	506	1056
201451_x_at	7	15	68	9	301	245	40	28	11	61	80	69	20	66	47	49	16	12	106	54	46
201452_at	25	101	69	28	14	144	94	46	37	189	74	114	82	68	7	8	55	9	8	11	33
201453_x_at	5178	5334	9498	5603	9850	8841	4281	4587	9177	10173	8645	9379	11188	13144	16755	10017	11014	10315	13659	10288	10612
201454_s_at	835	940	718	1139	789	711	1313	1560	743	658	767	993	1827	2798	1934	2485	2174	2188	1661	1500	2479
201455_s_at	2209	2114	1065	2340	1375	1133	2143	2328	1236	1282	1727	1617	969	1324	2075	2316	2119	2548	1526	779	788
201456_s_at	2147	659	2509	1852	1818	1075	2494	2530	1993	2492	2064	2718	1872	1550	1480	1793	1558	1785	2216	1359	2446
201457_x_at	8975	2326	5247	2866	5774	2682	7790	7716	6108	4931	6377	5087	6531	8711	9186	9631	10333	11262	7091	4149	5211
201458_s_at	3613	2232	4134	3263	2886	2071	3635	4184	3515	4288	3535	3675	3326	3313	4454	3591	4338	4743	4970	3382	5080
201459_at	3733	2541	3540	1543	2678	2252	2804	2381	3351	2379	2878	2447	4883	4141	4356	4441	4952	5310	5493	4151	3842
201460_at	1011	1197	948	1375	1221	895	799	983	840	1270	1205	772	666	588	434	422	485	431	532	415	420
201461_s_at	294	560	677	409	329	361	374	496	261	548	595	285	54	74	81	56	70	43	87	61	43
201462_at	1802	1792	978	863	2810	3630	1742	1954	702	758	4609	3270	4471	4825	3221	1610	1733	1232	604	631	672
201463_s_at	4580	2202	3827	6920	5703	9825	2805	3180	3380	3789	6906	7069	10272	10735	8971	5124	5060	4704	6466	5540	4721
201464_x_at	843	5281	2539	3303	2089	3549	473	717	1137	2221	1633	1719	2096	1295	1140	611	785	767	2326	3443	2407
201465_s_at	245	1147	372	533	59	81	78	100	123	482	323	320	242	177	74	13	9	15	47	94	71
201466_s_at	175	805	415	704	340	1043	94	149	96	281	156	248	647	588	394	195	179	250	955	1574	1621
201467_s_at	4579	6737	2164	4881	1232	2893	5643	5086	2181	2791	1654	1279	2262	1710	1006	1757	1333	1310	1547	843	723

201468_s_at	8348	13960	3140	11447	3592	4772	8704	9162	4181	5507	2966	3427	5299	2330	3958	10809	7692	8900	6035	7095	9159
201469_s_at	966	940	861	754	455	403	1310	1144	808	1006	1418	529	129	124	200	416	326	312	186	84	155
201470_at	6268	4919	5160	4043	4367	3069	6633	5294	6302	7530	5962	6078	6678	8139	8296	6600	6458	6167	7045	5348	3286
201471_s_at	6181	9045	6484	8082	3875	8114	5304	5102	3202	4953	5247	4534	4596	4895	2198	3139	3289	3140	1900	3503	2867
201472_at	2643	2630	6891	6291	4992	4276	4449	3915	7763	6625	5598	3271	7493	9679	10209	8557	8133	8600	11451	7482	9916
201473_at	1503	1833	1215	812	162	42	1069	1499	557	1441	534	568	155	294	247	292	332	381	369	330	167
201474_s_at	1654	1423	1195	718	833	1234	1567	1479	997	2239	1832	1134	363	420	254	636	602	339	249	287	234
201475_x_at	5636	3593	6345	1518	3307	5195	5736	2532	3733	1741	3660	2211	1841	2083	3391	2745	2686	2480	1738	3140	2598
201476_s_at	1139	388	1479	494	2411	706	2299	2447	1874	1413	3481	2423	1013	727	573	382	392	599	629	315	442
201477_s_at	2970	1356	2101	812	4525	1227	2657	2743	2142	1913	3891	3724	9201	8070	7314	5825	4934	5798	6994	3948	4625
201478_s_at	1695	1687	7895	2089	4661	2096	4133	3017	13233	10504	6336	4181	5748	4852	4871	2741	2611	1990	5186	4421	5169
201479_at	4047	3157	9244	3527	7237	4073	4926	3584	14730	10339	7066	4915	8665	7946	10848	6168	6255	5669	10717	9250	9619
201480_s_at	1119	862	784	1375	863	978	1128	843	1314	1301	1165	778	588	612	549	501	619	623	684	545	559
201481_s_at	859	759	7382	13333	56	501	684	110	5101	12599	561	700	463	592	655	1000	908	828	4273	5370	4071
201482_at	536	597	738	900	759	508	659	496	844	812	1104	735	623	551	443	137	198	202	128	111	150
201483_s_at	2496	2766	1384	1482	1270	1196	3509	4881	1480	1371	2774	2725	3756	3254	1946	3190	3488	3109	1413	1835	1065
201484_at	1106	1103	966	1138	994	1004	1907	2527	1009	1349	1595	1493	1536	1375	862	778	845	812	577	430	593
201485_s_at	3248	3630	2252	1994	1853	1165	1998	2543	2305	1748	1822	1633	2845	3231	2997	5264	5101	3681	4445	2325	4091
201486_at	4383	4061	2775	1614	2634	1927	2080	2765	1569	1807	1789	1894	4622	3128	2768	5630	5108	4271	6225	4194	6825
201487_at	709	507	4608	1606	646	418	1089	540	6128	4556	408	324	907	853	1591	1103	961	1025	6003	4862	5729
201488_x_at	1839	921	1861	1831	1193	1434	2904	2841	1580	2274	1984	1472	4014	2945	1843	1695	1754	1549	3117	2608	2094
201489_at	3497	2431	2923	903	2286	1213	3065	1576	4510	2612	3618	1751	2616	1747	2157	2169	1946	2015	3046	2638	1862
201490_s_at	2335	1502	2025	1022	1462	969	2292	1535	3773	2471	2767	1400	1492	864	626	681	403	993	1041	493	731
201491_at	4211	2977	2846	1249	2833	1939	3491	2734	4463	2737	3529	3192	5178	4577	5163	4236	3791	3763	4530	3950	2631
201492_s_at	30012	46426	34828	43954	37548	36475	36627	36308	36244	29485	27614	45785	21857	29404	35762	32653	35021	37101	18793	26828	24774
201493_s_at	1263	1088	1820	1913	1970	2328	1440	1767	1787	1933	2105	1802	2009	1356	1621	1784	1722	1521	3243	2502	3652
201494_at	1356	1460	1228	2424	1296	1786	1814	1809	1201	1312	1971	1837	1731	1680	2746	2358	2062	2595	1473	1315	1564

201495_x_at	11	7	11	10	21	26	8	11	16	10	10
	11	4	4	5	3	6	3	6	2	2	
201496_x_at	49	10	15	6	198	324	15	13	60	82	45
	10	40	67	42	17	19	81	41	34	22	
201497_x_at	37	28	33	59	80	120	45	32	45	43	27
	26	4	35	4	8	10	42	7	19	3	
201498_at	1618	1145	971	697	1109	629	1337	1275	1177	1040	1023
	716	1328	597	628	963	947	977	2329	1670	1896	
201499_s_at	4685	2838	3521	2188	3127	2082	4644	3923	6308	4865	3888
	3137	1390	1335	987	1146	1165	1117	1477	1407	1256	
201500_s_at	1434	1942	1735	2540	1467	1856	1908	1953	2146	2182	2278
	2018	1313	821	1590	1651	1698	1696	2990	2884	2836	
201501_s_at	1929	1894	986	954	1042	1216	1324	1246	879	914	715
	838	1304	1172	1234	2597	2293	2323	2275	1941	2542	
201502_s_at	982	7447	579	1310	111	634	849	1181	757	607	2339
	2380	657	696	428	1240	1285	1436	270	432	316	
201503_at	5908	2713	3238	3056	5604	3794	3126	3913	3994	3101	4339
	5319	8009	6028	7952	8346	6807	7718	9004	4733	6356	
201504_s_at	960	288	829	577	1207	2246	1696	1429	1600	1263	1984
	1758	947	812	738	666	580	634	934	492	656	
201505_at	516	499	713	308	452	185	278	426	324	425	254
	211	353	409	347	470	397	414	812	1298	2069	
201506_at	19	30	3344	3143	3956	2448	29	30	2051	1920	4896
	8594	3920	5509	7298	17	62	25	3826	4677	4965	
201507_at	814	580	1120	1252	1203	921	1214	1066	1141	904	1245
	1047	1974	1788	1036	933	894	744	862	543	441	
201508_at	20	26	2972	2019	1060	925	76	86	3515	5375	4474
	1528	512	495	341	36	23	37	568	257	142	
201509_at	847	508	579	535	914	808	706	632	666	593	863
	699	1741	1545	1381	942	903	714	776	716	559	
201510_at	505	943	373	2318	579	720	334	255	384	525	173
	181	83	73	78	295	199	193	526	589	633	
201511_at	1798	1277	1643	1411	1552	747	1215	1229	1985	1931	1832
	1156	1362	1403	981	671	742	638	839	546	426	
201512_s_at	1225	1027	1203	863	715	598	1693	1357	1474	1469	794
	518	595	540	547	1190	1140	1244	1224	1379	1566	
201513_at	337	297	279	279	588	844	334	240	321	282	470
	396	626	322	325	458	237	280	635	354	581	
201514_s_at	2118	678	1390	1372	3089	1575	1969	1672	1252	1523	1741
	2428	1197	1014	576	531	475	691	660	537	612	
201515_s_at	4788	2814	3097	2891	4064	5131	5377	4202	4122	3251	4782
	5909	4962	3652	5313	5438	4498	5074	5428	4031	5404	
201516_at	2422	2089	2306	488	1215	1048	3820	2239	2385	1639	2670
	1095	2001	1905	1887	1603	1937	1976	1480	1271	810	
201517_at	2184	1288	1564	1311	3095	3290	1497	1655	1545	1739	2014
	3190	2310	2150	2063	2009	1939	1662	1988	1436	1123	
201518_at	3334	3320	3095	2484	4148	3745	4656	5033	3113	2783	3601
	2262	5160	5036	4264	6313	6067	5728	4870	2336	1957	
201519_at	2142	1615	2680	2448	1626	1352	2494	2155	3143	2953	1525
	1186	1401	1320	2088	3068	3444	5164	5898	4757	6392	
201520_s_at	4010	4193	3790	2210	2506	1523	4892	5103	4255	3716	3796
	2314	3036	3431	5018	5294	4944	5044	4367	3285	2901	
201521_s_at	1296	445	992	852	1495	2193	1292	1048	1056	1210	1028
	1601	1260	666	507	514	481	469	491	407	406	

201522_x_at	359	151	368	140	599	387	352	205	421	292	334	289	41	59	63	88	39	68	55	54	50
201523_x_at	1519	920	1868	1822	1934	876	3534	3122	3666	2590	2371	2557	2905	2851	2170	2101	1955	1997	2709	1241	1293
201524_x_at	3277	2309	6009	3051	3631	2111	4852	4583	7569	6511	5169	4100	6767	6218	5835	6768	6015	5242	8866	4335	4093
201525_at	315	208	277	1121	287	216	317	286	296	270	243	289	61	65	45	31	60	33	69	100	77
201526_at	2581	1736	1963	2364	1417	1094	3175	2779	2222	2361	2041	1010	1789	892	580	784	649	736	601	544	324
201527_at	3016	1612	2249	4185	3056	3768	3514	4142	3071	2095	3701	2750	3605	3553	4333	2706	3242	2491	1891	2905	1089
201528_at	3100	2289	2402	2362	3076	1580	3204	3152	3202	3256	2901	3490	4519	4268	4550	5204	4731	4408	4854	4118	4955
201529_s_at	2122	1433	2950	1496	2315	1160	3287	2754	2903	3403	3948	2728	1992	2237	2003	2243	2010	2166	2176	1388	2007
201530_x_at	28631	13222	23248	17348	24296	17948	24127	22649	25370	19684	19785	13769	17847	21837	19614	17880	18246	18959	14918	15918	13630
201531_at	1172	2119	1512	1469	561	597	761	1908	862	1261	847	545	433	444	703	1223	1012	2422	1749	1727	1408
201532_at	3650	3777	5220	2916	6181	5464	5971	5405	5314	4186	6198	4595	9225	7796	6359	6284	6032	6471	5423	3558	3948
201533_at	10455	6125	1538	1708	1469	3404	6857	7650	1461	1294	4210	2854	9920	10868	7131	9412	9593	12773	5821	3754	3447
201534_s_at	298	538	372	679	613	1037	241	421	372	421	929	685	1168	282	347	209	212	167	947	1431	1772
201535_at	585	460	441	641	727	1614	464	487	447	457	937	1028	1030	1194	1961	760	801	560	1073	1523	2007
201536_at	661	540	1066	1185	844	2534	535	534	690	682	1187	674	664	778	486	259	314	259	631	769	589
201537_s_at	529	262	418	397	122	716	547	375	466	328	738	166	197	165	197	170	134	133	139	69	84
201538_s_at	405	223	326	275	378	512	404	327	399	233	570	172	196	151	106	67	68	89	60	95	68
201539_s_at	233	208	79	105	390	312	274	251	104	117	563	364	70	89	97	125	134	92	50	42	33
201540_at	771	1000	199	162	1467	3970	1084	755	206	239	1946	2110	504	787	2024	3413	3357	1757	49	245	194
201541_s_at	1052	1019	1005	1128	2003	2052	568	591	1006	915	1808	2645	3815	2963	2058	655	760	742	1096	1116	731
201542_at	868	1066	1435	1896	1622	1250	1342	1788	2048	2236	1649	1727	2258	2308	3250	4015	3481	4488	3494	2586	4061
201543_s_at	966	465	2002	938	1527	1262	1476	1019	2202	1603	1893	1026	1234	1182	1499	859	828	1066	1333	793	658
201544_x_at	6168	6922	4756	4228	3219	3040	6295	5578	4253	4028	4058	2884	5756	4271	3690	3788	3506	2730	2690	2686	1538
201545_s_at	1183	642	1249	616	986	649	926	1006	795	948	851	823	1121	518	371	674	554	461	605	429	258
201546_at	1689	1400	2328	2588	2109	2237	2370	1982	2636	2042	2007	1332	1643	1395	2048	2078	1961	2576	2376	2491	2687
201547_at	75	26	37	36	110	121	45	9	40	66	31	60	3	7	39	3	5	2	31	4	3
201548_s_at	368	465	640	813	166	312	716	577	504	688	442	387	90	150	212	343	291	355	247	343	510

201549_x_at	390	541	878	1494	529	609	554	771	613	745	466	473	210	226	309	593	767	688	815	1048	1081
201550_x_at	38831	49089	34722	24200	31857	30840	40082	35832	32660	33759	29419	45046	21966	27384	34032	32384	33965	35093	19533	26932	24409
201551_s_at	1351	1803	1545	1602	587	790	1921	1441	2293	2337	1971	1027	569	630	219	215	195	289	367	432	290
201552_at	4143	5162	4044	4788	3829	4545	3156	3303	3339	3522	3104	2049	5254	4719	2818	2752	2814	2462	3413	4644	3749
201553_s_at	9361	14750	7903	12009	10327	15027	8276	7520	8371	7987	7781	9114	11392	13026	11085	9314	7075	8345	9287	13059	9993
201554_x_at	1556	1443	2446	1967	1502	2220	1662	1783	2729	1504	2633	1684	2997	3482	4421	2652	2220	2206	3313	2907	3502
201555_at	3781	1040	3170	1348	3067	531	5207	4227	5065	3569	4916	4015	3466	2031	1333	1274	1191	1211	1726	853	656
201556_s_at	302	400	522	369	209	532	329	351	409	581	503	236	81	42	173	121	95	88	154	259	132
201557_at	540	450	688	533	33	317	388	589	338	537	480	327	80	130	197	210	295	219	173	218	140
201558_at	2164	1297	1755	1230	4602	2490	1673	1383	1890	1277	3348	2950	4672	4796	5243	2514	2680	2463	2880	2639	2411
201559_s_at	638	668	1034	577	1012	1490	928	812	658	1078	1136	1267	195	201	294	258	270	228	104	171	315
201560_at	1290	1702	2495	1005	2335	4200	1035	941	820	1347	2223	2526	2715	3148	5568	4600	4030	3514	2932	4610	6501
201561_s_at	1860	2365	1885	2428	2077	1560	1848	2213	1631	2014	1587	1156	548	631	476	898	887	1154	841	669	646
201562_s_at	1189	985	1436	1345	746	492	1646	1335	2470	1595	942	700	549	332	465	645	545	690	1156	442	587
201563_at	3315	3049	4570	2337	1579	1400	4078	2891	6220	5240	2082	1376	1755	1293	2674	4771	3860	4625	7742	3167	4626
201564_s_at	1013	1216	543	182	179	235	1332	1907	621	409	742	152	109	119	186	403	430	417	158	112	118
201565_s_at	3215	3013	1070	1079	5016	5559	1956	4600	1128	1343	4785	3128	17012	12576	7438	5599	4728	4713	4412	2827	4140
201566_x_at	409	934	389	435	1634	1149	621	569	341	593	1101	652	6221	2215	2299	2285	1868	1083	2659	746	1644
201567_s_at	1122	1148	1100	684	2271	3219	1045	841	1029	1273	1064	589	1506	1196	1408	1455	1441	1450	1550	1567	1764
201568_at	10169	6919	7612	10227	8804	11136	8165	7076	7619	8068	10494	13168	12827	7846	4711	5391	4960	5085	5916	6672	3470
201569_s_at	921	676	586	621	1071	1072	1134	781	1082	799	1020	919	2345	2164	1715	1497	1809	1400	1863	1357	1173
201570_at	1527	1420	1378	1164	1343	1058	2060	1568	1398	1185	1738	1381	2448	2189	1856	1596	1604	1276	1572	1384	1050
201571_s_at	796	591	399	336	683	697	920	1040	444	544	1387	697	1016	892	653	564	505	703	311	410	372
201572_x_at	1536	810	792	367	856	529	1508	1355	965	583	1779	1235	1083	956	976	1183	1132	1056	718	623	595
201573_s_at	2451	1664	2471	1320	3297	1764	2572	1949	3195	2503	2518	1905	2882	2557	3429	3052	2639	2720	3616	2687	3185
201574_at	3584	3511	4876	2442	4541	4553	4697	3716	4843	3761	4802	3173	6019	5093	3415	2142	2132	1436	2490	1652	1283
201575_at	1540	1005	1130	1159	2632	2948	1838	2053	1151	1064	2106	2276	3692	3646	3906	3121	3372	3071	2937	2738	2824

201576_s_at	1277	1435	1761	3009	1667	2938	1279	1420	1230	1572	2506	1991	2080	2491	1890	1465	1063	1055	2043	2335	3464
201577_at	15542	5793	9785	2180	8042	4044	15320	9102	10390	8321	7946	6513	13052	11278	11161	13632	13611	12151	8482	6555	4368
201578_at	704	982	9788	4609	972	2049	977	1083	5968	6708	1185	1377	720	606	1028	1000	947	990	5889	3988	3454
201579_at	9356	7764	2291	1505	2866	1614	8230	9456	1805	2334	1742	2100	1374	1595	1337	4557	4768	7025	2240	2354	2775
201580_s_at	329	107	305	244	796	407	433	389	304	398	780	873	555	447	495	188	208	144	277	300	346
201581_at	361	265	90	79	948	786	533	438	111	140	999	1206	1332	1301	982	774	882	741	568	1042	1436
201582_at	581	556	406	303	323	644	401	467	321	274	341	293	265	222	458	642	661	781	489	536	798
201583_s_at	3306	1929	1468	825	1343	1850	2769	2106	1209	1104	1298	1286	923	1102	1504	3484	3238	3483	1157	1348	1875
201584_s_at	4449	2141	2242	622	2532	1509	3336	2501	2515	1535	3358	1641	5541	5616	4033	4051	3554	4315	3630	2869	2417
201585_s_at	2553	791	2107	801	3018	1477	2017	1561	2035	1679	1727	1572	3218	1963	1231	1184	850	820	1813	1090	813
201586_s_at	4143	1752	4617	1393	3747	1114	4775	4543	4372	3950	4123	4120	7303	5881	4810	4426	3344	4009	6537	4468	3397
201587_s_at	2867	4255	14038	5591	9102	7392	2907	2220	13892	11441	8368	6538	13804	11420	9171	3307	3961	3443	13460	10926	9236
201588_at	6260	7299	3903	4051	2672	2400	5274	4500	3392	3321	2905	2683	4088	4882	6263	10247	9996	10428	6436	7564	8095
201589_at	2192	1063	2420	1107	4260	1957	1970	2293	2707	2621	2950	3662	5777	3821	3521	2558	1738	2712	5522	4935	6125
201590_x_at	20458	28308	24937	19715	31229	34608	23298	24465	21720	27070	25060	43330	17877	25307	27382	21972	23673	22722	13204	16624	13740
201591_s_at	1380	2786	944	2043	568	868	1012	1284	922	843	840	868	529	552	740	1947	1884	1598	890	1128	992
201592_at	10347	13355	14568	17678	3777	4023	9170	9063	14365	12503	4525	4928	6555	8550	8886	17013	21104	18177	16724	23671	20070
201593_s_at	1867	1387	3236	2236	1311	1333	4225	3018	5669	4661	3522	2049	6052	5285	6699	5089	4921	4489	5078	4258	4249
201594_s_at	1249	920	1629	1211	2377	1736	1929	1499	1977	1515	2325	1424	2539	2901	2090	2158	2077	1871	2632	2131	2594
201595_s_at	2113	1392	2561	1719	2908	2528	2247	1813	2763	2267	2182	1581	4564	3696	3849	3392	3144	3681	6047	4605	6730
201596_x_at	21663	33220	34759	40847	38429	21316	24058	22775	42159	33969	29804	38120	24451	31928	31197	17994	20602	17629	19088	26554	21560
201597_at	6338	7778	8874	8798	5238	9039	6877	6945	5605	7231	5146	6955	7246	8014	8439	11295	12482	11643	9523	7726	5727
201598_s_at	843	1326	1019	1984	612	1215	599	698	1116	790	1074	581	360	269	262	194	199	148	229	301	138
201599_at	4207	3087	2044	2199	4767	3733	4233	4229	1286	1855	3869	4835	5750	5598	7313	10731	9517	11437	5935	7010	9554
201600_at	16016	16624	10167	7639	10984	7607	13640	14094	10970	8769	11155	11795	14667	16929	17263	17635	18931	17795	12181	15863	12965
201601_x_at	419	973	437	560	1164	1068	274	470	728	553	2261	719	1054	911	1752	267	713	4275	440	3206	1861
201602_s_at	24	92	241	253	60	85	176	231	214	236	206	177	202	147	127	77	34	58	142	199	89

201603_at	705	661	617	675	592	743	496	589	487	609	573	576	1196	910	720	1135	947	1333	2169	1916	2757
201604_s_at	347	358	978	723	636	566	648	794	661	693	550	483	458	540	635	881	685	639	982	757	1046
201605_x_at	2049	1564	1971	1460	1544	1072	2170	1991	1882	2277	3144	1557	1606	1819	1177	1216	1325	1220	1140	882	636
201606_s_at	2549	2235	2406	1361	1469	1571	2447	2417	3299	2340	1592	1464	3027	1820	2708	3320	3309	2957	4483	3025	2777
201607_at	900	748	700	751	664	722	961	885	954	977	570	508	463	325	681	1012	902	991	1408	968	1815
201608_s_at	1849	1845	2547	1354	1871	1558	2206	1983	3548	2702	1782	1563	1696	1993	2869	3762	3755	4361	4033	3065	4118
201609_x_at	2532	3529	1381	1042	1526	1394	2482	2365	1548	1497	2128	1891	1566	1820	1735	2934	3088	2571	1923	1499	1760
201610_at	70	19	72	52	23	44	62	73	12	56	67	30	6	22	5	14	21	8	4	2	27
201611_s_at	777	528	802	824	1106	714	925	771	874	1027	802	754	831	668	890	920	907	816	1402	922	832
201612_at	2029	2341	2078	1954	1353	1539	2524	2040	2088	2141	1869	1717	3661	4256	3991	4644	5101	4695	5782	4947	7034
201613_s_at	524	498	321	687	412	544	195	322	242	506	355	309	318	360	360	702	742	935	757	951	771
201614_s_at	2697	2393	2216	1104	2036	1114	2115	1589	1889	2116	1819	1431	2700	2072	1968	2031	1717	1696	2606	1965	1762
201615_x_at	152	195	61	17	230	806	284	257	16	31	199	145	305	160	146	92	80	109	25	46	42
201616_s_at	558	539	16	22	564	753	533	501	51	17	475	500	172	196	196	278	241	221	9	64	78
201617_x_at	566	453	19	17	330	607	478	579	1	4	257	382	336	142	135	342	339	255	14	47	85
201618_x_at	1423	1541	644	485	694	563	1031	1134	698	882	1174	1156	585	935	722	697	707	796	591	320	301
201619_at	6609	5014	6229	4845	3508	2478	6414	6639	7429	6204	4134	3362	5516	6134	8792	11445	11339	13534	8801	6626	8190
201620_at	3101	3368	1378	2082	975	1582	1993	2316	1176	1827	1171	690	307	531	399	567	671	679	404	292	304
201621_at	618	538	518	687	478	149	749	612	571	486	853	256	229	284	33	170	279	153	186	59	72
201622_at	1271	950	2192	1977	1292	1806	1098	957	1774	2067	1980	1553	828	844	1229	797	1003	736	1115	1615	1317
201623_s_at	4591	5130	2570	2958	3772	3228	4145	4019	3282	4299	5089	2647	12245	13539	17166	12786	10423	11614	9707	11348	12577
201624_at	1774	1647	1461	1055	1288	2505	1177	1598	735	1198	1275	1144	3009	3866	3312	3528	2510	3287	4579	2751	5115
201625_s_at	1257	681	855	715	852	513	421	385	547	676	836	285	690	807	687	628	648	454	400	678	634
201626_at	2703	855	975	888	1413	1619	549	563	872	1100	810	408	1778	1729	2108	3899	2527	3340	2525	4442	5121
201627_s_at	3295	1423	2084	1719	2091	1114	1080	982	1188	1402	1873	499	1128	1287	2360	3174	2968	1961	968	2179	2249
201628_s_at	1695	2269	1934	2497	1982	1481	2360	2576	1789	2274	3309	2370	4170	3462	3220	2587	2681	2184	2534	2492	2199
201629_s_at	1304	515	1797	675	1454	732	1407	879	1769	1691	2247	1584	2741	2654	2194	921	953	947	1402	969	937

201630_s_at	2457	1472	2328	2168	3207	2763	1815	2263	2646	2823	2592	2893	3544	3716	3451	2828	2874	3093	3501	3356	3201
201631_s_at	18403	14809	3681	936	12163	5315	12692	11561	2351	2956	14185	14656	13652	13276	16027	17250	16029	16132	3433	4487	2754
201632_at	1647	1370	1879	1654	1285	1133	1537	1388	2249	1563	1910	1220	1876	2010	1581	1678	1504	1819	3284	1945	2224
201633_s_at	2879	991	780	1606	4104	2286	2934	2387	990	732	2444	1341	4298	3845	3139	4386	2705	3995	946	1065	1367
201634_s_at	2626	1112	487	876	2465	1079	1827	1990	396	391	1556	1154	4688	4342	4019	6082	4684	5417	1604	1750	2389
201635_s_at	1447	237	902	1231	1574	1138	2992	2305	993	1139	2550	2108	1926	1261	409	622	689	467	278	217	401
201636_at	1386	828	910	680	2449	2217	1545	1560	864	773	1683	1847	2929	2158	1984	2146	2153	2375	1580	900	945
201637_s_at	2765	1758	1417	1719	3201	3162	2548	2898	1375	1217	3755	3224	4464	5391	5498	5585	5484	4904	2708	3238	3646
201638_s_at	263	366	152	158	235	270	518	583	322	405	435	143	34	20	68	65	8	35	66	7	7
201639_s_at	2035	2557	666	619	335	499	1554	2033	938	844	756	371	516	522	234	678	521	575	412	316	290
201640_x_at	1023	1197	1390	1362	735	860	1133	1176	1350	1804	1478	719	336	425	507	442	371	525	620	284	335
201641_at	52	204	578	1098	236	380	21	20	656	685	240	167	36	70	64	36	189	542	499	905	343
201642_at	2133	2060	1784	1852	2087	1622	1700	1529	1525	1870	2264	2899	1699	1524	1225	1535	1424	1307	864	1314	1196
201643_x_at	1860	2070	1797	2089	2430	3530	2010	2308	1581	2140	1894	2710	1387	1293	1759	1825	1820	1799	2108	1552	1566
201644_at	1369	1140	921	892	521	355	1468	1227	1013	812	706	241	786	669	424	1120	1237	1223	1391	827	587
201645_at	15	10	959	88	364	804	12	12	870	77	219	133	71	86	60	6	5	16	869	551	585
201646_at	1188	699	875	1901	529	1394	958	1193	827	1342	765	584	274	270	191	318	343	322	372	585	644
201647_s_at	476	427	468	903	267	567	697	759	749	932	595	426	80	110	99	122	108	121	112	167	189
201648_at	1189	1923	2282	3364	1332	2043	1712	2032	1721	2516	1840	1826	1572	1559	1420	2018	1888	1454	1830	2843	2831
201649_at	865	1035	122	277	880	1461	1133	706	237	183	824	667	235	200	177	280	319	956	92	227	229
201650_at	12540	16650	6974	13193	15786	9834	11180	8885	4936	7322	14245	15172	19137	18219	11723	8575	9122	5886	4323	5318	3264
201651_s_at	3067	4559	2508	2439	4257	4355	2070	1865	2347	2257	3485	4522	3231	2505	2184	1925	2648	1643	2247	2824	2500
201652_at	3834	3107	1035	1213	1889	1747	3049	2319	874	660	1721	1230	3305	3205	2317	3979	3576	3671	1908	1939	1932
201653_at	4379	4058	3611	2812	3697	3255	3604	4041	3630	3143	2824	2315	4604	3756	5690	8132	8086	8055	7026	7801	9290
201654_s_at	38	237	270	273	23	39	34	107	154	348	78	48	2	4	3	7	2	3	3	3	3
201655_s_at	139	1605	860	1344	48	65	30	272	503	964	158	204	108	116	56	100	87	173	301	342	370
201656_at	1122	619	4526	1564	5132	2654	943	1008	3784	5258	2922	3759	4078	3475	2421	1293	1441	1123	6950	4118	5501

201657_at	247	144	293	281	241	429	319	298	221	281	198	163	388	165	160	377	246	586	749	894	1127
201658_at	578	328	570	811	705	896	635	513	605	595	421	532	543	645	676	1728	1628	1268	946	915	1304
201659_s_at	1070	736	1408	1138	801	1181	1251	1144	1391	1266	995	1124	1371	1241	1093	2005	1940	2056	1893	1576	1717
201660_at	1760	1753	1682	1658	1078	1258	1014	1139	1114	2025	833	696	870	1128	2036	2633	2498	2134	2839	2452	3515
201661_s_at	1749	1500	1721	1221	819	1093	849	1049	963	1737	560	461	1374	1216	1135	2191	1753	1515	2887	3160	4562
201662_s_at	3078	2390	3584	2108	1949	1062	1990	2193	2767	3289	1485	1191	1461	2007	2625	3728	3994	3906	5431	4815	5516
201663_s_at	1384	461	2785	685	5675	753	2120	2270	1560	2082	4903	3748	3288	4602	4078	1244	1243	1754	1845	852	1535
201664_at	1734	777	2900	856	7312	1134	1639	1785	1592	2115	4983	3883	7107	6850	7417	2359	1899	2645	4342	2501	4196
201665_x_at	26266	34695	26631	38931	23490	29164	24639	26175	30624	27358	19236	23857	17764	24773	22559	22847	24486	26721	14790	23799	18850
201666_at	4114	2558	3877	2792	2452	1635	4477	4521	4713	5532	4275	3259	5033	4026	3173	2731	3097	2676	4061	3918	2499
201667_at	5	1	9	14	51	58	7	50	13	6	26	16	8	1	1	1	1	14	11	14	2
201668_x_at	267	297	787	859	34	50	325	355	561	864	388	167	7	3	77	36	7	8	39	48	15
201669_s_at	1304	1343	3241	2790	1086	1086	1120	1244	1533	2545	966	1022	1174	801	848	1934	1712	1739	5962	3966	6109
201670_s_at	635	535	1324	1399	357	347	372	483	808	1138	675	452	430	299	174	200	175	147	393	214	220
201671_x_at	1036	945	1978	1888	1087	1250	885	1061	2101	2109	1450	1727	1913	1497	2090	1466	1039	863	3620	2620	3079
201672_s_at	3753	2810	6781	4524	5215	3865	3788	3486	7132	6541	5655	4541	4995	5682	6525	4322	4660	5506	9929	7506	8491
201673_s_at	1308	1714	1329	2451	734	607	1373	1354	1852	1546	1461	744	318	332	432	579	584	567	810	386	474
201674_s_at	734	780	1781	2336	914	758	1251	870	2503	2510	1474	1082	1445	1734	1037	646	680	720	1801	1753	1666
201675_at	1953	1851	2389	2096	1978	1795	2625	2235	2174	2683	2041	1651	2479	3953	3317	2744	3034	3489	6281	3919	5278
201676_x_at	9582	9091	6371	6441	8919	9287	9960	8168	5468	5280	9897	9403	11622	18340	16978	13359	15122	14437	9667	10731	9897
201677_at	376	462	370	565	686	578	347	413	470	632	433	327	197	288	246	332	213	187	402	204	203
201678_s_at	1434	799	1507	1668	2044	1146	1485	1209	1913	2063	1731	1797	1879	974	696	776	829	508	1412	940	1016
201679_at	495	225	461	202	698	251	696	490	586	569	742	378	66	38	28	48	12	39	58	18	17
201680_x_at	3254	2351	2789	1564	2587	1195	3956	1966	3532	2750	3526	2099	2539	3237	3781	1321	1423	3500	2809	2041	2277
201681_s_at	1708	1826	1203	1179	2282	2731	1353	1552	811	1557	1816	2054	2492	1967	1961	3108	2759	2112	2118	2132	1991
201682_at	3362	2467	1533	1397	5033	4742	1681	3259	2357	1205	3380	5556	6258	5917	7051	4146	4097	4427	4727	5419	5476
201683_x_at	373	107	368	454	455	458	398	348	559	430	640	298	391	153	115	97	113	137	110	76	56

201684_s_at	1327	932	1002	1108	1725	2176	1090	1322	958	815	1430	1360	715	428	399	388	318	509	458	252	381
201685_s_at	349	235	217	466	304	340	317	454	206	207	382	405	391	382	364	227	219	193	252	315	458
201686_x_at	848	398	1111	863	1196	937	849	1094	1454	1277	1687	1139	315	307	360	284	263	305	579	292	384
201687_s_at	1455	955	1691	1371	2264	2043	1271	1375	2297	2096	1919	1942	3059	2154	2345	2463	2218	2180	5258	2281	3343
201688_s_at	1541	1411	1150	1578	903	925	2324	2142	1920	2212	1349	1214	1043	770	1023	1559	1380	1522	1745	1108	1887
201689_s_at	2189	1999	1443	1670	1337	1396	2133	2588	1434	1824	1171	1052	1779	959	965	1952	1509	1615	2908	1905	2878
201690_s_at	3412	3175	2298	2725	1772	2182	3159	3085	2139	2707	1677	1439	3543	2925	4374	8040	6726	7922	6623	4412	6352
201691_s_at	134	122	197	244	63	195	66	73	131	261	138	102	85	75	92	177	116	111	248	159	269
201692_at	1142	1191	1070	678	1425	992	1228	1425	1402	1786	1511	1345	2907	1420	2765	1081	1183	961	1612	1033	840
201693_s_at	1028	238	312	106	540	156	836	1590	341	685	655	719	913	1540	1596	1515	1302	1541	1220	1150	856
201694_s_at	2377	364	1621	348	1479	235	1283	3971	594	1732	1711	2864	7361	7429	10409	6657	5967	8435	6135	6955	7712
201695_s_at	1461	1184	2602	1433	1610	538	1725	1082	2577	2524	1646	1093	1235	1396	2396	1993	2129	1665	2781	2215	1547
201696_at	1868	1510	1020	876	1647	1007	1842	1540	1255	1505	1636	1173	2675	2786	2929	2949	2970	3180	2086	1525	2122
201697_s_at	3309	3123	1963	702	2117	926	1989	3312	1767	1982	2278	2613	2551	1304	783	1133	1012	1552	2301	1481	1643
201698_s_at	8537	7659	7428	5928	3487	1961	6294	6760	6402	5880	4606	3544	7299	5556	4001	5756	4835	4640	3953	3341	1620
201699_at	1293	813	1293	940	2550	2102	1200	1244	1389	1038	2267	1439	5436	5549	6095	4133	3250	4159	5788	4563	6667
201700_at	878	606	608	521	2294	2071	900	719	876	771	3225	1666	2017	1985	2171	737	703	755	773	657	337
201701_s_at	595	376	423	397	550	744	405	527	174	408	341	537	441	368	202	551	454	403	743	846	782
201702_s_at	333	307	156	281	76	82	431	417	95	198	429	225	110	207	196	200	148	89	84	136	91
201703_s_at	1774	1279	1103	1055	859	839	1373	1327	1196	1182	1087	1044	517	295	411	470	458	313	372	359	290
201704_at	1371	1688	1187	2024	788	783	845	665	1231	1364	1175	655	790	885	904	846	1008	1003	1105	1378	1386
201705_at	1726	1904	1649	1502	1764	1177	1944	1822	1634	2228	2334	1587	2377	3280	4452	4671	4475	4234	4805	4380	5004
201706_s_at	318	226	726	816	220	539	629	740	808	848	691	493	284	236	503	368	369	307	677	390	527
201707_at	243	349	308	356	476	363	460	447	352	573	229	328	319	211	316	386	459	385	586	436	708
201708_s_at	841	847	1346	1936	416	426	1723	1850	1379	1927	777	545	301	278	170	304	363	286	350	280	227
201709_s_at	2097	2242	3087	4093	1116	1449	2745	3085	2645	2879	1477	1496	894	1508	1571	2323	2327	2389	2128	1819	1890
201710_at	882	380	1477	229	136	60	1276	951	1355	1212	1327	589	1016	1197	474	479	455	515	1151	456	404

201711_x_at	335	255	1453	901	1989	1752	1897	2348	1894	1864	2486	2295	387	401	329	187	177	234	313	262	411
201712_s_at	489	345	433	472	1329	1086	607	467	689	558	506	444	360	339	471	388	281	350	557	383	604
201713_s_at	946	1008	1320	1033	2368	2418	1401	1587	1658	1669	1813	1477	1705	1593	1877	1317	1445	1741	2378	1531	1958
201714_at	2598	1414	2016	480	1891	951	2544	2874	2480	2664	3063	2431	2672	2434	1979	1896	1721	1796	2345	1482	1246
201715_s_at	627	510	572	529	847	722	855	822	649	534	654	337	373	255	148	198	203	207	221	132	104
201716_at	1173	1282	1451	1601	1218	1099	1855	1587	1248	1648	1728	1403	1297	1445	1225	831	836	900	780	709	712
201717_at	4007	2047	4556	4148	3418	2017	4211	3179	4711	4759	4735	3374	3533	3997	5911	3890	3497	4329	4886	3337	3196
201718_s_at	548	198	1830	600	470	38	1073	878	3218	3828	1077	677	233	316	216	157	174	197	653	356	439
201719_s_at	1083	696	2112	870	1073	261	1040	790	4299	3725	713	1465	953	818	610	843	805	764	3543	1622	2320
201720_s_at	32	106	39	166	382	97	27	127	29	35	730	280	114	263	210	11	10	12	50	17	9
201721_s_at	251	139	187	176	826	552	186	183	280	194	1411	401	357	812	368	47	61	59	49	79	66
201722_s_at	1347	1557	1361	1407	570	810	1521	1510	1080	1135	568	530	649	588	700	3258	3289	3178	2932	1645	3001
201723_s_at	1319	1271	1371	1689	913	454	1935	1929	1573	1512	813	749	433	494	455	1389	1115	1255	1171	894	1095
201724_s_at	647	1034	689	956	212	292	800	830	694	612	279	255	324	419	484	2748	2214	2171	1807	1446	2063
201725_at	3490	2288	3456	1589	2895	1658	2724	2701	4668	2862	2005	1962	3110	3175	2338	2375	2724	2300	3969	2673	1954
201726_at	6011	6331	3803	3175	4334	4666	5806	4330	5210	4727	4959	4424	5738	5181	5830	5113	5338	5569	6643	4263	5516
201727_s_at	1386	1269	874	566	731	581	1986	1511	1375	1382	1582	1086	672	512	284	500	569	599	447	222	221
201728_s_at	503	367	600	612	636	333	770	878	1068	1291	959	821	108	143	112	104	80	35	153	137	80
201729_s_at	1482	1036	2459	2806	1762	2150	1834	1836	3821	3528	2780	1881	1555	707	663	567	419	323	955	893	674
201730_s_at	1032	920	1035	1273	410	415	1501	1706	1694	1919	782	724	460	782	1190	1606	1726	1230	1621	1435	1440
201731_s_at	2188	1199	3057	1949	1479	1356	2423	3324	2450	2934	1889	1105	874	774	502	996	763	1075	1386	845	722
201732_s_at	230	151	393	366	444	586	546	603	368	484	431	309	251	178	105	214	198	207	148	130	168
201733_at	589	220	288	328	313	427	292	205	377	222	225	248	93	100	77	136	215	165	92	119	195
201734_at	3220	2077	2366	1827	1614	2502	2531	2589	1894	2595	1549	1418	1418	1503	2239	4308	4350	3475	2277	3174	4314
201735_s_at	1515	1145	1861	1231	1654	1560	2287	1927	1632	1838	1455	1122	900	974	1287	1720	2143	2006	1347	1233	1845
201736_s_at	1711	1485	3036	2782	1225	1287	1623	1766	3616	3980	2565	1526	957	1479	1771	2144	2284	2086	1841	1931	2579
201737_s_at	198	92	1911	1593	1355	1189	140	240	1403	1954	1097	1516	236	253	152	59	45	54	620	511	765

201738_at	1740	2142	1534	1723	2117	2092	1338	1777	785	1198	2055	2060	3486	3545	2452	3967	3940	3957	3170	4299	3681
201739_at	397	24	1157	485	408	234	225	230	712	657	163	302	91	121	109	497	356	283	1875	1430	1649
201740_at	3490	3176	3477	4569	2959	2595	3416	3469	4245	4087	4572	3115	4920	4380	3478	2620	2249	2232	2887	2223	1297
201741_x_at	110	82	208	137	340	290	228	368	209	219	126	68	36	34	22	12	60	30	67	37	42
201742_x_at	5318	3225	5092	2621	4819	2326	6913	5862	7292	7182	7363	5668	1736	1279	1052	1821	1397	2150	1791	710	1065
201743_at	8	7	66	43	23	15	60	17	21	63	10	15	10	6	45	16	3	4	111	104	92
201744_s_at	1	1	15	17	3	4	1	1	3	1	1	1	4	1	1	1	1	2	162	329	327
201745_at	3522	2996	2836	2253	4201	4460	2837	2547	2458	2460	3057	3059	4728	4594	2905	3649	3074	3707	4583	3661	5396
201746_at	1537	2139	1476	1417	1069	1348	2030	2489	1580	1855	1961	2417	694	843	1571	2133	1951	1678	2751	2568	2534
201747_s_at	508	437	247	166	898	552	514	364	340	252	451	259	317	209	66	137	129	46	81	84	90
201748_s_at	1339	1310	684	622	1754	1098	1304	1101	583	879	790	999	952	733	428	427	561	261	504	475	525
201749_at	462	616	361	468	785	973	335	410	380	579	486	504	183	174	119	155	109	152	148	103	130
201750_s_at	99	305	199	230	162	356	209	189	325	96	90	64	75	28	82	30	22	18	7	9	7
201751_at	1070	1126	692	528	865	1154	1179	1293	657	955	1154	1533	1432	1447	1267	1265	1633	1410	743	1760	1557
201752_s_at	500	508	2290	4045	1447	836	1093	1423	2071	2920	1836	2157	1167	1067	1405	392	432	285	1302	1619	2782
201753_s_at	339	524	1758	3443	1269	1490	596	940	1215	2539	1228	1841	1475	905	866	269	292	215	1750	2484	4160
201754_at	9447	11942	7153	7098	7791	11493	11653	11505	4225	5944	7485	9881	9122	8724	12000	16757	17270	15442	7999	6059	4486
201755_at	1172	467	521	132	632	71	1295	901	751	451	853	373	429	557	288	550	483	832	625	182	235
201756_at	1984	1445	1732	1346	1828	1149	2193	2185	1858	1952	3269	2594	2154	1372	1222	1417	1322	1195	1520	1017	858
201757_at	4038	6181	10639	7773	3329	2677	11314	9234	11820	8687	4198	4553	5646	4957	6283	12365	12357	9272	10052	8434	4331
201758_at	1890	2354	2433	2857	2481	2123	1639	2114	2297	3038	1634	2312	3222	3942	4972	3981	4149	3840	3468	3939	4768
201759_at	282	158	99	132	361	266	249	176	107	82	136	106	111	171	66	133	81	116	120	126	52
201760_s_at	3229	2709	4326	3832	1541	1711	3098	2392	2608	2555	2356	1201	2439	3000	3734	5013	4548	3664	6070	5177	5887
201761_at	7498	6955	7349	991	5002	8922	5797	3390	6083	3508	2712	2921	5234	3897	6968	10905	12179	13436	10340	11816	14126
201762_s_at	3682	5036	2390	2982	4673	5112	3376	2502	2291	1982	4403	4948	4091	2310	2445	2355	2361	3347	2378	2847	1728
201763_s_at	478	241	414	410	102	253	466	462	482	608	370	246	257	286	328	353	293	262	283	154	242
201764_at	1591	1399	2006	1869	3340	1954	1863	1792	1464	1674	3577	4461	5788	7527	5900	2680	2990	2706	2398	2543	1865

201765_s_at	695	704	1263	2073	685	1055	632	680	1123	1368	888
	877	1299	967	458	368	385	351	915	1166	957	
201766_at	187	137	122	79	128	38	205	268	171	193	205
	52	7	35	36	48	23	35	34	6	23	
201767_s_at	1222	909	940	401	1324	473	1314	1019	1240	764	1222
	518	1229	1198	744	560	523	697	835	521	548	
201768_s_at	1322	695	1447	2408	1302	1177	1306	1502	1816	1971	1609
	1513	326	255	166	291	305	284	351	303	361	
201769_at	1172	1186	1664	3360	979	1924	1489	1214	1640	1880	1509
	1160	1857	1762	2297	2508	2069	2603	3337	2869	4118	
201770_at	3720	2351	3167	2633	1440	1712	2857	2749	3306	2994	2535
	1718	2843	2124	2149	2567	2274	3039	2791	3349	2385	
201771_at	2011	1922	1841	1794	922	900	2237	1453	2281	2110	1225
	1121	879	1043	1161	1957	1555	1896	1329	1256	909	
201772_at	3534	1950	1496	909	2326	1838	2359	3056	1306	1247	2133
	2296	1739	1199	1705	2525	2115	2090	1591	1749	2241	
201773_at	3326	3213	3360	3051	3045	4319	2435	2536	2396	2131	3057
	2557	2894	2941	3261	4043	3371	5268	5256	3486	5407	
201774_s_at	1443	212	1065	400	1755	646	1382	2625	1226	1277	1973
	2248	736	961	602	451	401	498	606	364	389	
201775_s_at	536	432	998	1064	763	605	889	962	1418	1683	944
	905	93	69	156	117	127	134	272	124	187	
201776_s_at	337	404	875	1076	429	710	416	788	852	1634	885
	828	578	506	507	447	566	528	1150	754	1208	
201777_s_at	872	214	491	551	513	353	457	668	809	696	658
	385	165	154	205	175	140	109	176	112	143	
201778_s_at	1401	1741	2383	4420	2815	3468	1660	2063	3030	4021	2281
	2746	1665	2140	2621	2402	2605	2109	3675	3493	3776	
201779_s_at	741	869	2252	2909	1446	1807	1479	1498	3931	2791	2432
	2475	2261	3010	2106	1269	1353	1185	2743	2667	3311	
201780_s_at	593	502	1209	895	1623	2161	557	566	1061	925	1127
	1136	1909	2073	1847	1724	1566	1504	3499	3120	5219	
201781_s_at	667	1088	1462	1644	680	950	651	592	1492	1297	1094
	617	589	463	418	422	345	285	568	546	548	
201782_s_at	1296	2349	2353	3167	1607	2454	1072	1104	2317	2078	1595
	1372	990	743	711	529	532	438	876	1127	933	
201783_s_at	1511	2579	2135	2540	1490	1119	1454	1586	2282	2126	2301
	1674	898	1129	1501	1275	1178	1140	1371	1273	1093	
201784_s_at	3954	2932	4415	3141	4917	7607	3605	3509	2811	3349	5315
	4580	8798	5718	5425	5317	4868	5500	6657	4076	6152	
201785_at	103	84	178	489	785	587	294	346	233	453	283
	338	84	85	107	38	54	44	64	17	50	
201786_s_at	4464	3840	3072	4658	2647	2990	4439	4051	3730	3374	2831
	2977	1526	1590	1885	3693	3800	6317	2850	4958	5136	
201787_at	53	112	24	32	128	203	34	53	25	29	37
	29	50	42	9	125	152	152	9	4	79	
201788_at	2802	2330	1587	1564	1840	2087	1768	2103	1897	1790	1720
	1832	1916	1772	1659	2613	2696	2662	2522	1813	1846	
201789_at	37	68	8	43	51	52	89	100	8	64	74
	43	16	30	23	2	39	20	22	17	20	
201790_s_at	3112	2247	3538	1976	1170	989	1651	1230	3242	2084	2818
	840	651	845	1621	1698	1543	1255	1863	2558	2652	
201791_s_at	2352	1137	4385	2005	2635	1009	865	765	2721	2282	1849
	566	1139	1769	3950	3273	2649	1652	2983	5505	4031	

201792_at	49	68	41	39	102	75	57	36	39	39	60	40	9	12	14	8	10	8	10	9	5
201793_x_at	311	74	227	184	43	39	523	416	277	349	414	353	36	34	10	13	32	22	6	2	42
201794_s_at	1005	534	1641	1790	920	857	1146	955	2027	1472	1379	1004	645	445	417	496	554	584	1052	797	806
201795_at	3790	1332	5205	2687	3281	1367	3187	2594	4232	2971	3630	2501	2554	2428	4899	6313	3903	4706	8429	5372	7144
201796_s_at	458	573	530	128	36	44	913	709	644	521	505	39	5	56	56	111	79	47	36	7	9
201797_s_at	1539	1476	859	253	445	351	1717	1291	825	565	728	236	455	406	400	1248	1236	1237	890	465	502
201798_s_at	6936	10354	4436	4796	3179	6125	4072	6781	2602	3803	3515	5327	2146	1585	2109	4908	5491	4128	3135	4279	5024
201799_s_at	609	502	1388	1309	628	869	1165	1008	1490	1737	1349	692	499	411	358	397	307	363	442	451	760
201800_s_at	757	669	1541	1888	1170	1627	1239	1118	1917	1845	1560	1470	925	872	766	656	714	546	1075	1275	1590
201801_s_at	663	427	564	405	400	262	1008	718	962	901	756	530	159	143	100	313	286	414	447	165	256
201802_at	738	420	699	347	528	120	865	769	851	903	725	503	151	106	199	229	220	282	275	177	136
201803_at	2514	2303	2036	2032	2930	2534	2961	3430	2642	2281	2794	2079	2717	2683	2950	5048	4089	5301	2746	2069	2491
201804_x_at	3344	3559	2671	2431	1592	2219	3204	2920	3655	4084	3478	3724	4386	2814	1299	1230	1428	998	1017	814	458
201805_at	568	483	1062	1234	884	1422	1436	1632	1192	1638	1487	1685	1478	1122	598	621	571	495	289	326	241
201806_s_at	205	227	374	206	386	59	254	241	116	173	158	106	98	118	108	73	109	118	67	89	58
201807_at	2285	3182	3647	3488	2843	3861	2673	3193	2885	3676	3294	3475	4728	5161	7441	5695	5700	5499	6538	6261	8885
201808_s_at	153	39	313	198	29	26	172	63	478	401	107	157	19	13	26	11	21	11	34	11	16
201809_s_at	165	129	399	154	297	306	227	117	483	236	95	111	29	9	5	17	66	13	280	133	112
201810_s_at	307	363	297	171	686	749	441	435	330	419	549	527	517	362	335	187	168	130	138	280	90
201811_x_at	958	1557	395	375	1597	4995	958	864	321	551	1001	926	1219	968	905	1090	822	889	528	527	521
201812_s_at	6961	9028	6799	11549	5619	8916	7908	7507	5892	6876	6782	8880	11598	7725	6169	5584	5561	6046	5020	5970	3507
201813_s_at	1068	774	594	565	663	1015	400	486	322	189	493	480	471	383	178	365	250	227	245	335	333
201814_at	1515	1518	956	1394	981	1477	816	876	713	597	807	755	617	510	525	1113	957	546	716	776	806
201815_s_at	532	347	454	424	531	813	538	509	330	368	519	265	127	138	84	123	106	122	99	170	137
201816_s_at	2322	2847	1015	1159	1555	3464	2086	2159	759	1153	1819	2167	3283	2276	2876	3881	3171	3344	3584	3702	6024
201817_at	1961	1219	2279	1580	2058	1488	1358	1404	2688	2637	1906	1377	1682	1578	1473	1520	1500	1355	2453	1785	1962
201818_at	1367	1415	1263	1967	408	228	1128	1384	1519	1505	375	279	518	404	297	503	498	347	709	490	494

201819_at	1282	1304	1557	1170	340	60	1104	847	1607	860	857	380	305	286	422	663	705	622	943	446	507
201820_at	106	18	18	22	14802	13144	127	36	12	154	21912	23636	12053	7702	7424	39	82	23	61	76	30
201821_s_at	1692	1463	2581	1518	1747	1682	2857	2437	3242	2491	2497	1364	3603	1603	1359	2436	2336	2197	3165	1280	1701
201822_at	71	95	68	76	249	317	155	137	100	119	101	102	110	76	109	212	156	223	184	141	147
201823_s_at	868	325	403	402	579	1689	910	785	349	377	792	655	727	450	554	638	561	610	341	394	561
201824_at	782	665	366	404	704	1340	546	512	322	320	469	457	692	433	324	520	436	552	372	337	705
201825_s_at	22	10	564	445	459	547	16	20	654	759	794	1161	885	1043	1169	58	11	54	1257	966	1019
201826_s_at	93	35	307	294	415	507	77	91	285	309	402	377	192	193	208	6	10	34	165	194	197
201827_at	1777	847	695	485	994	641	1533	1153	990	839	1086	623	1232	910	677	644	752	597	711	528	361
201828_x_at	1687	2786	496	570	276	254	2132	1882	528	470	387	392	62	108	123	1323	1531	1233	99	112	45
201829_at	959	404	2079	1664	3560	2817	518	755	1409	2147	2183	2334	2758	1178	1677	705	560	481	3552	2680	4129
201830_s_at	874	270	4826	2574	3581	2440	1105	909	3473	4581	5736	4707	4032	3064	4167	931	902	1009	5386	3516	5169
201831_s_at	1115	764	1032	559	286	348	1501	1775	819	1284	776	710	460	438	347	1381	1522	1427	744	939	1212
201832_s_at	2953	3267	2718	2187	2357	3368	3387	3237	2171	2300	1376	1581	1526	1628	3092	4954	5204	4967	2566	4104	5565
201833_at	3871	4247	3831	2021	2539	3511	1537	3083	2893	3290	2740	2142	4025	3765	3625	5509	4643	5835	6840	5293	6253
201834_at	184	400	269	482	279	125	346	317	151	238	306	372	256	197	203	341	376	262	270	387	303
201835_s_at	673	545	511	513	709	590	596	793	558	517	570	545	96	87	110	146	127	177	98	89	136
201836_s_at	364	420	258	348	220	275	241	282	229	215	281	248	202	135	230	273	250	296	242	212	308
201837_s_at	876	1175	861	1167	936	1591	942	804	622	605	969	674	1138	1011	885	768	934	975	762	885	1099
201838_s_at	185	174	248	309	331	245	318	265	289	373	300	331	148	138	196	114	148	142	166	118	132
201839_s_at	9386	8379	11622	9136	9774	10546	4696	7127	9361	10467	7150	5767	13138	9916	9349	12058	12882	10883	13242	14705	15475
201840_at	1418	1248	2329	1932	2389	2434	2117	2259	2019	1855	2061	4729	8003	2469	1301	1617	1393	1345	1795	1032	506
201841_s_at	4198	14532	2189	7879	3978	4139	3556	1820	1903	1009	5194	2701	6139	4537	4884	3476	3144	4236	2019	1678	755
201842_s_at	12	54	80	124	92	177	64	40	80	78	77	36	21	4	12	22	1	38	3	11	3
201843_s_at	8	43	31	61	98	28	9	46	27	36	11	21	3	18	12	12	19	1	13	10	9
201844_s_at	831	1243	1063	925	225	497	823	1074	890	1114	674	518	940	757	488	845	1288	721	786	726	728
201845_s_at	782	1032	985	742	1346	2924	771	891	702	1050	1154	1268	1530	1356	1411	2052	1765	1624	2049	1465	2215

201846_s_at	705	674	662	834	832	1199	725	742	688	864	1145	846	1035	657	227	231	182	173	445	310	217
201847_at	1429	1594	1649	2239	1344	3020	1729	1362	1135	1248	834	1386	1174	1071	2083	3341	3403	3411	2482	6461	8380
201848_s_at	507	603	54	47	84	112	719	578	58	68	53	90	7	9	10	342	294	359	11	32	2
201849_at	1037	1466	12	35	51	47	758	785	55	59	26	10	5	3	9	1848	1770	1957	12	5	13
201850_at	1413	2057	2158	3122	460	1553	1464	1094	1777	2429	1047	945	697	701	492	1051	1342	912	1340	1248	811
201851_at	1048	1000	578	510	749	769	977	881	633	517	901	508	560	633	851	872	553	964	441	418	444
201852_x_at	189	63	71	70	65	43	152	164	104	105	92	77	13	8	5	70	6	70	3	19	7
201853_s_at	4935	3788	1982	1010	3130	2619	3690	4252	2091	1623	5580	2559	2216	1408	2437	1790	1120	1691	978	1211	1131
201854_s_at	1693	1657	1283	1123	1089	985	2192	1920	1275	1332	1030	969	527	706	773	1958	2050	1786	1262	1065	1610
201855_s_at	1122	886	501	586	728	966	875	937	326	519	459	586	932	799	1121	2577	2236	2624	1555	1918	2854
201856_s_at	1037	701	982	934	847	642	1118	1160	966	1100	873	970	280	210	144	234	166	190	307	185	302
201857_at	2856	2982	2207	2499	2328	2056	2919	2457	2348	2200	1831	2056	3349	2625	4580	3548	5205	4662	5217	3320	6595
201858_s_at	16	7	11	13	16	43	13	13	8	15	10	10	1	5	2	5	2	6	1	3	2
201859_at	64	21	117	61	22	48	45	51	62	64	51	31	6	11	19	48	26	41	25	21	38
201860_s_at	321	332	18	71	351	85	135	139	58	53	557	390	118	104	85	311	256	171	21	22	23
201861_s_at	964	939	2188	1026	1017	641	1218	1094	1670	2281	968	811	1365	1221	1410	1676	1654	824	2655	2291	1937
201862_s_at	1765	856	3988	2062	2969	1574	1560	1495	2554	3375	1967	1059	2201	1910	1977	1971	1957	1438	4711	3373	4179
201863_at	1650	1532	1480	1543	1963	1482	1981	1908	1515	1829	1850	1445	1625	1827	1252	1549	1502	1465	1265	938	983
201864_at	3154	3538	3043	3920	1331	1263	2956	2569	2525	3913	1501	1492	432	387	392	1272	1397	1080	1169	848	784
201865_x_at	290	394	100	119	547	587	391	479	102	141	325	431	275	266	188	82	120	111	21	64	65
201866_s_at	267	339	221	171	51	51	363	492	175	249	253	251	58	121	62	154	127	175	18	37	37
201867_s_at	323	339	263	185	318	396	439	377	306	383	256	299	45	91	51	105	132	54	55	65	43
201868_s_at	254	260	182	91	37	12	265	288	207	300	88	78	36	8	35	40	8	7	22	6	19
201869_s_at	373	406	408	49	43	357	429	375	305	224	81	21	123	147	80	166	200	121	164	194	122
201870_at	1818	1720	2705	1911	2706	1473	2211	1709	2786	2942	3775	2278	3290	2753	3034	1274	1422	1445	2544	1399	1685
201871_s_at	1036	1010	1583	1746	1238	2084	1179	1099	1549	1725	1558	945	2417	2316	857	1176	1307	935	1704	2502	2063
201872_s_at	2409	1906	2707	878	2878	2543	4672	3691	3460	2358	2834	2321	3044	3033	4704	5347	4661	4795	3914	2736	4362

201873_s_at	1957	1400	1955	702	1480	1391	2394	2037	1830	1697	1373	1072	1508	1112	1208	2329	1782	2323	3452	1576	2333
201874_at	1775	1563	2310	1970	1133	1725	2480	2493	2537	2953	2489	1803	537	785	896	1001	910	852	825	785	786
201875_s_at	1430	684	723	705	804	771	841	797	982	1008	1022	1287	294	347	501	465	457	366	645	654	694
201876_at	1846	2579	2390	2212	2783	2047	1954	1803	2870	2802	2936	2179	4943	7385	6415	2665	2380	3029	3526	3209	3534
201877_s_at	1429	1566	1190	1185	1484	1789	1031	1285	983	1423	1285	1208	1637	1396	1927	2417	2441	1796	2686	2019	3280
201878_at	271	392	380	229	302	281	348	636	349	409	300	336	266	241	262	571	384	416	351	310	411
201879_at	486	534	850	982	653	594	1108	1585	1171	1381	792	914	254	154	109	316	305	245	396	227	291
201880_at	728	1619	872	1156	1238	899	1260	1644	902	1194	509	833	564	447	644	1610	1317	1396	1414	1060	1410
201881_s_at	523	691	765	512	691	628	1061	965	728	760	823	608	471	412	556	589	625	588	567	616	610
201882_x_at	44	75	117	48	76	58	41	44	88	22	65	137	14	7	11	9	46	4	57	101	10
201883_s_at	1015	701	392	441	804	825	1094	949	606	546	270	304	364	323	380	356	465	369	662	954	965
201884_at	214	220	659	718	159	168	326	309	876	1069	202	465	15	29	22	31	10	34	653	752	1178
201885_s_at	3948	5473	3574	2911	2195	1978	2886	4345	2214	3502	3448	3640	2135	1938	2563	5833	6130	5442	3898	2315	2803
201886_at	651	482	689	729	642	946	839	694	859	783	540	528	272	301	418	594	708	518	691	729	749
201887_at	577	789	1918	1160	1493	1856	767	660	1244	2073	1688	1491	1224	1048	944	599	778	653	1726	1617	2309
201888_s_at	167	140	456	202	452	540	187	239	290	314	362	402	263	367	201	266	286	187	408	408	413
201889_at	7234	2949	2581	1681	2137	2537	3458	3622	1775	2285	1639	1477	3221	4016	3525	9700	9643	7094	4643	4385	5339
201890_at	5705	2676	3193	883	7585	238	6715	6961	4489	4324	7136	9057	12132	11707	10795	9098	9877	11056	9042	4848	6004
201891_s_at	8907	18071	14537	21690	10701	19138	9816	10954	12259	13794	11742	14561	11875	14118	13509	14815	15913	22622	14486	20355	19028
201892_s_at	12634	14161	7538	2531	6801	5398	10986	12184	6700	6302	7599	7773	8130	7354	13659	18207	17780	16024	8491	9665	9926
201893_x_at	246	147	283	204	448	400	376	339	312	330	286	222	60	33	67	51	16	42	60	59	50
201894_s_at	2025	2179	2066	1469	1332	2121	2612	1884	1607	1319	1244	1863	1282	1289	2321	4216	4382	4706	2176	2503	3465
201895_at	759	888	1120	1390	869	886	906	685	890	1242	1437	1398	528	708	444	421	387	469	459	519	480
201896_s_at	964	342	974	538	1002	251	876	796	953	872	1035	846	737	483	385	287	260	338	1652	358	555
201897_s_at	2388	1752	4604	1364	3264	812	4297	4464	4221	3895	3617	5072	7056	6753	5717	6766	6573	5880	5465	4296	3293
201898_s_at	1372	1421	2172	1869	2162	1627	1631	1940	2324	2820	4161	3750	5188	4027	4101	1204	1244	957	2298	1983	1647
201899_s_at	1724	2203	2625	3061	3230	2905	1907	2051	1935	2743	3029	3078	4698	4752	7478	4629	4274	3812	4904	5808	5481

201900_s_at	3257	2609	2462	2512	892	1273	3067	2774	2127	2254	1526	1519	1194	974	1285	3035	3449	2452	2031	2841	1763
201901_s_at	1869	2557	1873	2545	1902	1805	2150	2506	2665	2194	2658	2136	3777	3728	2540	2676	2781	2145	3145	2724	2112
201902_s_at	54	153	148	110	54	67	139	210	231	341	269	112	62	18	111	40	34	51	21	108	71
201903_at	6091	4885	6103	5804	3447	3452	5128	4815	4226	4061	6867	5204	7503	9858	9815	6777	7134	7570	5373	4985	4063
201904_s_at	978	1133	1158	1523	3827	2646	954	742	1191	2288	5786	2725	4257	4582	5417	839	680	522	1881	1633	814
201905_s_at	302	146	400	422	1317	632	371	306	399	662	1765	953	932	995	905	311	201	288	397	380	513
201906_s_at	743	740	1095	2210	4403	3700	803	616	1596	2984	6920	4831	5627	5956	5235	814	1011	644	2358	2077	2502
201907_x_at	135	153	251	155	165	121	34	32	186	177	297	38	12	36	29	11	12	59	73	66	9
201908_at	660	1292	696	1179	738	1094	806	1156	611	960	1320	571	485	569	680	562	536	533	484	437	432
201909_at	2401	3909	212	167	243	327	4228	3671	238	221	125	171	13	19	54	816	611	599	9	17	13
201910_at	65	272	415	1181	972	1379	295	351	365	396	861	718	558	670	651	367	383	356	587	405	358
201911_s_at	307	602	545	1605	737	794	358	421	638	500	949	490	411	531	441	217	308	227	457	397	541
201912_s_at	2324	1356	2884	1857	2276	1977	1795	1405	3307	2344	2523	1396	3122	3425	3248	2457	2701	2768	5598	3719	4015
201913_s_at	1995	1938	1770	2007	1619	1149	1359	1423	1935	1237	2047	1654	1853	1912	1583	1285	1330	1058	1837	1850	1358
201914_s_at	409	325	533	674	416	597	490	476	745	630	578	410	1280	1078	1051	833	844	619	1279	2176	2183
201915_at	228	90	213	172	275	447	160	128	199	183	153	143	113	116	112	178	188	135	230	348	364
201916_s_at	893	809	2065	1620	1560	2295	939	941	1787	1680	1323	831	1654	1721	1997	1969	1879	1883	3683	4857	6342
201917_s_at	221	187	792	1379	728	758	541	702	788	855	1134	1225	1671	1531	1172	221	242	145	1135	2411	1431
201918_at	524	387	804	1036	646	818	684	1185	701	1094	1063	1168	992	1173	644	457	325	346	475	882	1008
201919_at	1708	2113	2954	4256	2478	2500	2414	2874	2831	2943	2724	2842	1680	2175	3120	3221	3073	2675	2894	4237	6388
201920_at	3671	2315	936	379	4168	4049	4505	3220	1700	1277	5905	3608	6540	6179	9648	8558	7557	8895	3156	1966	2654
201921_at	4276	4011	1659	1993	4365	4116	2071	2520	979	1725	2919	2535	5661	5369	4014	5008	4818	5463	3702	4283	4498
201922_at	5746	4950	4342	4942	3920	4065	6043	5252	5423	4738	4129	2898	7317	8407	9393	12915	12734	12386	11983	11860	9193
201923_at	5724	7253	2534	1296	3703	4331	6179	5293	3002	2486	4013	5249	5624	5005	6366	8437	8914	7089	5095	5265	5378
201924_at	874	1096	992	1972	1895	1696	1142	1606	747	1396	1251	1863	1114	834	1018	828	736	835	1873	1512	2414
201925_s_at	796	884	2010	419	69	484	539	458	482	2491	121	57	236	224	380	2285	1662	2404	1880	2741	4101
201926_s_at	495	720	1479	713	265	486	635	594	807	2580	121	245	147	114	153	724	863	589	916	1213	1106

201927_s_at	651	232	1447	869	844	488	645	904	1065	1474	1484
	770	1277	896	292	192	143	97	353	256	187	
201928_at	1136	911	1537	1240	2076	1658	829	1185	1363	1543	1691
	1668	2050	1706	2440	1205	1224	1222	2787	2609	3427	
201929_s_at	1046	635	1991	1402	1045	1097	772	1070	1650	2001	1613
	1102	781	679	452	381	350	347	798	425	516	
201930_at	4867	3814	4053	1965	5923	1176	5130	5914	5844	5282	6267
	7060	7819	7422	7214	5603	4077	5483	8704	5386	6333	
201931_at	3006	2740	6546	5260	2306	2564	2596	2052	7194	5403	2123
	1948	4312	4510	3271	4664	3613	4605	9625	7678	7984	
201932_at	788	1134	422	530	48	290	969	657	272	129	472
	447	205	241	311	614	527	545	156	248	329	
201933_at	1379	2348	1275	1104	827	657	1509	1131	1164	976	796
	582	578	329	334	708	692	643	857	447	331	
201934_at	5030	3297	2256	2020	4365	3329	3164	3650	2654	2608	3726
	4005	7356	3799	2894	3072	2795	2838	5791	4206	3500	
201935_s_at	311	471	756	819	749	1089	639	622	558	805	867
	760	494	488	301	225	214	283	276	358	365	
201936_s_at	276	406	305	378	525	468	203	290	183	233	237
	468	405	142	195	277	275	157	629	505	452	
201937_s_at	984	781	926	1151	1031	1133	1087	841	1312	1002	1337
	841	999	1021	777	722	739	601	997	591	590	
201938_at	7504	5278	2688	2572	3407	2279	4985	5272	2969	1951	4768
	2138	6967	8802	10273	13835	13502	16275	8031	5887	6449	
201939_at	1184	2403	1313	918	658	825	648	545	1307	1223	608
	542	1214	1056	611	1509	1422	1527	1731	1733	2047	
201940_at	558	540	1169	1370	603	1048	953	1145	911	1715	685
	931	384	337	463	568	545	528	838	985	1276	
201941_at	930	1197	1411	2051	1438	1900	1088	1211	1268	2479	1025
	1636	981	1103	1371	1441	1141	1699	2352	3495	3816	
201942_s_at	221	202	600	603	444	470	619	967	775	1061	675
	599	69	51	61	88	122	116	133	124	190	
201943_s_at	492	422	932	1009	766	796	515	1098	775	973	689
	576	280	278	458	526	430	474	544	640	883	
201944_at	2781	3036	3593	2523	2447	2926	3097	3573	4111	4556	2948
	2757	3738	4636	3862	3168	3009	3176	4460	4163	4165	
201945_at	456	463	358	435	1023	883	489	326	298	437	954
	462	171	187	254	129	221	153	122	183	91	
201946_s_at	1804	764	6480	2267	4665	2029	8719	8299	9780	8239	7085
	4938	5746	4445	4208	5299	4611	5802	4802	2779	4674	
201947_s_at	8364	6568	9537	5323	8413	4167	11448	9832	11746	9068	7569
	7841	11246	12217	11315	13722	13634	14539	12698	10671	12892	
201948_at	1097	574	1021	544	1028	818	920	991	1092	1017	740
	674	944	826	724	1063	931	1121	1588	1133	925	
201949_x_at	2371	1631	1512	1646	2495	2205	1830	1680	1243	1393	1563
	1090	1990	1386	952	1538	1274	1319	1812	1113	1113	
201950_x_at	2679	2933	2326	2316	1306	1110	3713	2916	2602	1936	3102
	1513	1615	1383	1191	1430	1537	1488	1028	558	611	
201951_at	647	837	11	13	18	13	792	1196	5	6	7
	15	15	2	4	1237	1149	932	123	124	167	
201952_at	2330	4136	269	251	192	118	2021	2739	229	203	134
	190	6	25	35	4378	4287	3960	379	725	918	
201953_at	1774	1892	3500	3395	1172	1299	1189	1153	2701	4089	1175
	1219	1575	1340	725	1030	1092	1016	2573	1979	1270	

201954_at	4440	8918	6114	6047	5010	2559	4483	4620	4944	5363	5347	4533	7508	8330	3702	2173	2548	2065	2520	2394	1596
201955_at	4523	3608	4697	3440	5655	3748	3326	3431	4311	3252	4889	4444	6324	7512	7325	6033	5982	6395	8110	7328	9848
201956_s_at	1979	1781	2055	4391	1309	1363	2642	2128	2051	3110	1759	1538	1187	1673	2705	3190	3343	3306	3172	3632	4174
201957_at	267	254	414	438	359	394	351	364	459	463	280	263	89	126	81	119	119	65	161	97	118
201958_s_at	7	9	12	9	12	12	7	9	12	14	6	11	2	6	2	1	4	3	2	3	3
201959_s_at	720	1050	951	898	1003	1141	938	1013	776	964	863	792	325	295	413	461	395	461	604	1211	1267
201960_s_at	737	725	1165	824	1223	1375	1120	921	1128	988	1080	882	1005	990	1518	1338	1035	1974	2565	4880	5294
201961_s_at	279	203	335	352	164	400	315	286	238	252	217	250	124	127	243	307	256	260	265	361	443
201962_s_at	200	181	704	374	129	552	309	330	621	600	327	308	198	270	200	243	182	164	253	322	266
201963_at	446	309	620	623	2081	4839	276	268	396	365	1346	1315	1858	1694	2349	630	523	475	1750	2695	3157
201964_at	1004	673	1157	865	1003	990	1034	1135	900	1307	1204	561	685	704	1015	1041	990	817	810	1368	1036
201965_s_at	613	287	530	357	471	657	461	587	516	546	765	524	275	281	156	130	150	149	149	201	116
201966_at	2436	2008	2444	3188	2085	1825	2999	2238	2986	2868	2277	1674	2599	2099	1508	2553	2945	2837	2633	2469	2734
201967_at	396	416	540	541	636	864	761	664	603	481	577	569	618	667	420	521	368	347	313	350	352
201968_s_at	3221	3508	2655	4467	1831	2340	2589	2931	3372	3800	2132	1152	1331	2111	2592	4136	4664	3699	4163	3794	4611
201969_at	369	245	370	264	438	267	497	744	698	559	296	336	255	271	253	320	452	244	729	295	299
201970_s_at	2809	639	3038	1308	2535	989	3090	4019	4921	3842	2982	2297	3970	3031	1799	2644	2363	1711	4226	2038	1829
201971_s_at	504	85	921	969	693	946	749	841	1196	939	1376	1160	484	330	352	284	212	269	493	337	446
201972_at	3039	2103	2777	4331	3889	7832	2078	1987	3362	2739	3992	3180	3155	2572	4904	5235	3887	5094	5674	4134	5544
201973_s_at	4222	4884	5829	4263	7956	7927	4203	4004	5574	4509	6829	7823	9309	6892	10796	5729	4786	5207	10099	10631	12100
201974_s_at	163	163	203	219	539	638	309	282	261	268	256	173	66	69	40	58	40	64	86	77	36
201975_at	583	717	914	438	687	1105	382	508	515	594	934	480	797	357	536	716	697	776	1815	1506	2346
201976_s_at	1068	1583	3098	2818	2836	2958	988	1147	2561	2435	2659	2938	2016	2079	2875	1310	1254	1412	2980	3077	2838
201977_s_at	304	283	368	573	309	247	264	442	377	524	242	392	214	241	259	309	285	308	315	340	454
201978_s_at	406	856	433	450	502	830	151	315	587	509	151	704	525	516	619	832	651	798	1087	750	862
201979_s_at	763	538	912	656	418	140	1053	920	1280	1122	1178	435	431	372	211	224	240	240	324	101	159
201980_s_at	769	876	1164	583	1182	1025	810	693	839	1082	1212	1019	2295	2403	2131	1563	1463	1304	2008	2166	2395

201981_at	16	46	39	18	81	211	42	38	58	92	64	20	16	33	7	5	6	3	13	10	5
201982_s_at	33	32	46	35	297	259	45	100	39	112	180	41	21	35	12	38	41	30	36	14	39
201983_s_at	389	825	937	790	533	703	841	1011	714	869	1461	2064	347	468	396	258	223	107	273	282	309
201984_s_at	575	443	822	410	873	1601	480	540	452	790	839	1146	359	283	562	224	255	199	466	430	502
201985_at	1072	791	2469	2355	715	1177	1006	1049	2414	2284	800	679	988	486	585	620	641	503	3212	2071	2957
201986_at	497	371	1324	1282	1634	1306	753	1045	1133	1177	1313	1062	943	730	401	482	329	374	829	1095	887
201987_at	1376	1562	1795	2239	2059	2518	1681	1843	1769	1832	1774	1536	2469	1800	4514	2733	2858	2640	2630	4515	5391
201988_s_at	524	470	558	788	1214	1403	481	600	455	704	1050	1096	374	378	417	385	348	178	368	413	462
201989_s_at	925	856	935	1164	1230	1840	523	912	701	1027	1416	1751	1356	1309	1637	987	1156	914	921	1668	1978
201990_s_at	250	240	275	486	660	917	239	319	238	411	686	764	693	667	728	330	379	254	486	984	1035
201991_s_at	6325	7202	4710	3285	2610	2923	3768	4922	3568	3898	2911	2679	3435	3263	2362	3988	3213	2514	5893	4074	3472
201992_s_at	413	389	951	564	867	657	810	810	1037	1036	728	626	332	328	262	341	335	364	517	453	528
201993_x_at	4237	1758	2351	1990	3192	2064	3505	2662	3184	2787	3077	1968	6097	7427	3778	5085	5390	4800	4578	4166	5062
201994_at	7307	5894	9776	9394	9873	10271	7547	7337	8972	8583	7447	7959	13059	14902	14241	13760	15121	15384	13520	16279	16991
201995_at	599	765	1629	1811	429	888	452	666	1444	2020	570	855	470	394	204	332	425	352	1363	917	1082
201996_s_at	112	140	136	129	179	160	256	327	182	243	239	290	264	113	44	90	103	81	128	77	69
201997_s_at	1036	1414	813	838	793	902	1228	1668	946	1249	1175	1237	815	663	798	1534	1518	1890	1211	1186	1360
201998_at	558	498	602	1036	697	908	665	527	500	376	433	368	175	180	466	597	563	390	389	940	1276
201999_s_at	4445	7626	2857	3501	2881	3428	6107	5280	2284	2859	3195	2831	6203	6032	6032	10120	9224	7306	4223	3734	3121
202000_at	1054	769	678	582	1226	724	951	848	811	597	843	802	716	714	515	983	895	750	687	502	413
202001_s_at	4347	2658	2614	2530	2540	2861	3482	3182	2408	1864	3495	3941	5296	3615	2000	2175	2077	2704	2923	2143	1311
202002_at	126	83	179	176	220	79	82	186	39	110	114	106	21	55	33	36	28	101	69	57	41
202003_s_at	359	255	1708	2263	471	110	578	488	1424	1895	937	901	1145	1580	1392	323	419	523	3077	3049	2828
202004_x_at	2867	1415	2865	2739	2359	1932	3827	3283	3323	3385	3016	2492	2726	2182	1795	1638	1560	1594	1898	1360	1078
202005_at	1008	1377	1700	3013	1307	1141	1064	933	1874	1477	1340	377	996	850	964	1021	966	903	1358	1073	937
202006_at	1287	1429	1765	1297	2577	3342	881	1411	1238	1639	1813	1752	2672	2663	2347	2309	2034	2332	3278	3308	4386
202007_at	5	4	11	6	180	20	3	4	5	4	348	137	217	584	141	2	2	1	1	52	33

202008_s_at	120	77	146	129	318	251	101	94	95	126	335	206	62	88	66	30	26	31	46	39	19
202009_at	676	568	534	498	727	284	621	586	622	586	749	628	573	339	123	268	145	261	238	225	171
202010_s_at	811	617	929	777	1375	2056	962	932	780	832	1352	1220	1181	722	1231	1238	1016	1273	924	927	1052
202011_at	2122	2448	4158	3170	3968	4010	3054	2804	2831	3811	3850	5280	4336	3792	4736	3677	3021	3522	4776	4635	6131
202012_s_at	1281	967	1458	1492	1656	1692	1264	833	1292	1280	2108	1910	1445	2092	1468	1268	1668	1324	1807	1225	1620
202013_s_at	606	718	659	744	492	560	625	463	599	662	583	613	397	565	702	863	922	790	786	1042	1271
202014_at	414	657	576	199	416	746	272	392	63	278	701	528	459	364	335	193	192	165	100	193	144
202015_x_at	57	6	14	34	194	230	38	79	47	49	70	68	16	19	27	2	24	29	8	29	4
202016_at	1928	1828	5308	4497	3535	3096	1593	1766	6838	5068	3550	2994	2847	3703	5285	2349	2441	1913	6370	3817	5666
202017_at	1532	2713	2230	14409	965	1410	2498	3431	2535	4894	2918	1181	379	424	892	752	1032	814	940	739	608
202018_s_at	17	12	12	13	22	339	17	16	16	22	16	13	2	6	3	4	4	2	4	4	2
202019_s_at	602	288	483	622	557	601	1102	1335	765	647	840	547	215	232	444	435	350	399	247	126	175
202020_s_at	1076	1123	841	1134	1271	1441	1512	2014	804	832	993	1626	1888	1859	2659	3426	3475	3585	2003	1296	2061
202021_x_at	9739	15128	14130	7078	8504	11390	10835	9752	8137	8376	9978	12933	11513	7841	7617	8212	8324	6392	6401	10716	9857
202022_at	1124	1180	590	948	407	348	666	878	538	718	335	299	60	123	171	1008	789	419	305	338	273
202023_at	385	311	191	273	36	164	142	239	37	87	290	268	309	242	129	379	437	241	70	435	313
202024_at	1480	557	777	974	884	1068	1651	1626	983	886	1107	643	849	994	905	1051	1162	1200	638	671	596
202025_x_at	874	743	1236	1763	1461	1325	844	734	1500	1368	775	897	535	296	391	249	398	338	468	540	339
202026_at	1947	1864	2366	2347	2074	2654	1639	1932	1935	1744	2311	3036	4091	3686	2979	3314	2283	3664	5783	3782	4625
202027_at	806	1653	747	193	789	1013	918	913	607	787	1003	797	756	748	434	1005	1206	829	669	626	539
202028_s_at	2037	3079	1765	2261	1287	1666	3022	3465	2604	2664	2065	1190	382	1222	3221	3119	2195	2599	1608	2617	1503
202029_x_at	29098	29749	20683	24204	23206	23290	22651	22781	18597	18015	16922	23427	19530	23254	22206	23681	25045	22099	13693	20445	12581
202030_at	607	893	456	670	440	501	814	963	569	581	624	535	324	234	251	487	463	436	471	345	358
202031_s_at	978	1168	880	1115	1089	622	1136	1403	1184	1679	1707	1398	2122	1057	4351	2591	1882	3157	3145	2245	2397
202032_s_at	500	354	690	931	763	843	375	412	589	721	389	138	245	228	226	116	186	221	318	375	298
202033_s_at	642	816	897	813	1178	1555	652	1176	488	829	1066	916	1286	1084	1629	1348	1231	1179	957	1171	1501
202034_x_at	472	227	155	125	653	974	178	363	198	161	267	307	829	528	523	745	549	726	643	829	1344

202035_s_at	45	28	46	59	286	113	56	50	47	57	24	36	19	31	42	13	14	23	18	19	19
202036_s_at	15	62	129	91	22	58	130	17	124	59	91	67	2	11	5	7	13	4	18	43	34
202037_s_at	189	207	217	255	482	414	281	195	195	155	220	163	20	30	40	37	47	35	50	74	48
202038_at	1790	1988	2652	2932	2541	3111	2474	3332	2349	2634	2528	2902	2815	3087	4606	4525	3721	4048	4532	4451	5905
202039_at	550	418	632	1355	531	902	851	670	957	1058	783	427	102	170	156	245	170	171	289	342	196
202040_s_at	271	237	639	782	727	653	284	364	526	882	777	935	593	760	822	299	336	376	836	1034	1107
202041_s_at	3618	3557	4014	4092	1993	2236	3429	3470	4102	4996	2942	4034	3231	2782	3051	2520	3011	2617	2802	3695	2842
202042_at	2193	770	1804	1017	1905	974	2002	1562	2311	1966	2935	1770	3678	2666	2168	1403	1403	1287	2010	1319	1219
202043_s_at	3666	2579	3326	2518	2540	1398	2164	2365	2914	2660	2157	2389	4531	4733	4262	3780	3337	4018	5853	3840	3856
202044_at	151	170	311	326	243	278	134	124	357	323	314	207	77	45	49	41	7	22	70	48	53
202045_s_at	224	420	501	605	745	585	460	441	478	605	512	331	145	78	69	93	54	74	124	90	48
202046_s_at	202	269	486	405	410	396	242	297	656	658	614	320	47	39	50	55	36	30	58	34	27
202047_s_at	2963	6716	905	985	1867	1778	3366	3319	1002	713	1759	998	718	314	620	1337	1065	690	490	774	535
202048_s_at	2309	2946	758	464	181	392	2072	1799	99	80	627	435	834	613	547	613	697	389	452	811	708
202049_s_at	466	474	506	556	412	693	725	718	371	506	345	363	320	280	226	613	502	486	409	530	541
202050_s_at	640	894	827	1143	649	935	1075	1271	685	932	492	617	318	276	223	594	517	494	648	635	654
202051_s_at	790	1046	1077	1195	774	875	1040	1069	789	857	652	621	537	521	622	1304	1318	1369	1538	1434	1841
202052_s_at	1000	687	1563	1851	382	261	857	732	1692	1388	389	392	567	296	798	1081	1123	1191	1676	1862	2427
202053_s_at	2630	2446	2310	4161	1649	2242	3372	2465	3195	4074	1487	1212	654	552	1049	3267	2394	2623	2328	1216	1279
202054_s_at	1278	1598	1141	3797	871	1122	1117	1219	1900	2398	630	956	457	354	508	1593	1568	1338	2990	1112	1325
202055_at	533	474	1122	788	940	653	802	777	1468	999	954	778	650	610	854	1023	811	1175	1328	855	1158
202056_at	208	263	514	335	469	787	436	379	694	580	409	484	505	356	660	691	556	538	1054	1007	949
202057_at	82	116	418	398	254	317	49	154	175	356	131	241	80	106	168	161	187	132	250	286	293
202058_s_at	443	360	1046	629	720	864	953	798	1204	1124	1209	759	337	390	522	471	376	498	518	403	587
202059_s_at	528	577	1521	1139	755	638	1313	1198	2448	2000	1967	637	982	1054	2693	2564	2278	2538	3451	2152	2455
202060_at	3647	2359	1999	1783	4038	2533	2808	2709	1658	2073	3539	2498	6676	6855	6506	5771	4087	6010	4159	3369	4105
202061_s_at	1685	1265	1914	1613	1920	3658	1108	1213	1029	1304	1626	1592	1138	1546	1967	2404	2403	2625	1483	3802	5161

202062_s_at	452	302	332	278	535	665	345	298	219	379	426	486	81	121	72	80	120	89	110	199	203
202063_s_at	74	16	90	166	29	204	25	34	71	52	53	180	35	33	7	59	79	65	81	83	154
202064_s_at	307	315	323	220	243	495	299	422	238	331	269	348	102	174	139	262	373	264	117	248	298
202065_s_at	361	271	722	488	231	535	444	432	821	774	533	354	135	93	106	104	107	88	130	104	153
202066_at	1193	946	1652	1278	1157	2156	920	871	1192	1642	1016	911	1984	1611	1618	1401	1310	1479	3536	4295	4964
202067_s_at	509	175	484	21	337	78	143	84	86	565	601	320	189	539	291	401	516	446	133	208	322
202068_s_at	3227	1407	2862	773	2919	531	904	1095	1339	2846	2541	1802	2587	3776	3091	4159	4444	4571	1910	2799	3295
202069_s_at	2298	957	1811	1060	1221	1118	1740	1593	2357	2553	1873	1132	1291	1178	1366	2619	2406	2723	2994	1632	2392
202070_s_at	767	354	964	495	1046	767	1036	819	1399	1289	1126	847	636	492	801	730	664	710	1286	864	1065
202071_at	2744	3101	2211	2420	2474	1943	2904	2758	1410	1943	4779	5840	3179	2691	2268	1491	1659	1649	1786	2320	2530
202072_at	5427	3111	732	781	203	452	4481	3767	1579	1588	125	248	486	719	876	2533	2699	2674	2211	1014	729
202073_at	347	1036	251	711	466	907	184	314	205	266	411	359	261	155	256	368	347	286	261	579	884
202074_s_at	396	704	602	1447	1494	3127	453	352	371	766	2038	1731	1362	1812	2139	892	1035	967	870	1648	1685
202075_s_at	181	184	354	393	107	390	69	70	484	366	418	331	154	116	110	19	48	42	199	195	219
202076_at	1879	2634	2318	2841	1790	2951	1375	1692	1015	1781	1968	2369	1977	2099	2403	2886	3612	3149	2999	5892	7167
202077_at	10304	8176	8641	7965	5633	4447	10212	7010	11228	7769	5861	4509	10491	9305	11473	13461	14065	14160	14095	10519	7478
202078_at	1580	850	1750	964	2759	2224	1950	1388	1749	1367	2585	2569	3974	4909	5034	3056	2866	2579	3362	2214	2136
202079_s_at	320	290	486	776	351	274	432	472	427	523	365	255	70	140	72	94	172	113	217	143	263
202080_s_at	778	596	715	1491	115	286	726	883	454	752	651	584	339	292	358	572	642	374	662	618	767
202081_at	10263	8293	5842	2854	3708	1285	6551	6437	4115	5124	4551	4069	3987	2857	3187	2704	2879	2424	2275	2636	1285
202082_s_at	169	218	416	127	449	222	256	306	340	280	985	488	1029	943	287	223	217	231	168	230	98
202083_s_at	102	158	179	113	318	310	178	128	272	113	587	354	469	545	464	129	174	156	158	205	256
202084_s_at	902	1645	1668	1351	1911	2720	1324	1490	1533	1483	3799	2653	1995	1923	1196	903	884	749	730	749	541
202085_at	635	343	2509	2412	1614	1700	295	181	2712	2848	2109	2152	2420	2284	2850	646	722	662	5350	3863	5004
202086_at	156	170	98	357	285	665	144	128	242	270	566	646	136	122	152	37	28	68	69	486	527
202087_s_at	1121	1100	254	266	1757	3098	1060	878	199	221	927	855	1201	825	535	856	1124	900	440	543	414
202088_at	5937	5289	2356	1073	1399	1423	4473	3953	1872	1645	1606	1445	1979	1334	2374	10177	10665	11872	4595	4396	5791

202089_s_at	2787	1556	1243	559	785	673	3034	2431	1469	1013	964	748	563	540	395	1490	1437	1422	632	505	411
202090_s_at	5226	6181	2750	3722	2473	3110	5482	4682	2878	2753	3199	5628	6710	5841	3779	4384	4728	4534	3110	2922	1657
202091_at	185	142	102	122	155	224	180	235	131	162	195	96	30	28	64	67	50	60	39	64	35
202092_s_at	1725	1662	1171	964	928	1309	2271	1652	1172	1020	1693	1827	867	787	798	1432	1460	1487	457	646	807
202093_s_at	448	532	568	427	814	508	518	544	577	566	732	544	861	937	732	698	561	460	664	542	586
202094_at	917	386	639	39	766	60	963	893	776	572	1039	631	816	1160	904	696	819	964	719	270	280
202095_s_at	3158	470	1205	150	1185	173	2828	2664	1890	1205	1829	1529	2861	3505	3277	2762	3014	2834	2448	879	779
202096_s_at	3250	3952	3003	3390	1460	1665	2777	1879	3334	2527	2312	1524	2289	2055	1818	3281	3119	2299	2542	2760	1075
202097_at	1400	1383	1891	898	1816	1294	1870	1898	1758	1658	2088	1909	1924	1116	1073	2929	1806	2436	3164	2066	3210
202098_s_at	943	1403	612	992	610	613	963	1131	631	644	648	578	509	614	447	925	1057	669	393	487	443
202099_s_at	173	231	175	118	210	445	306	362	341	348	263	129	28	48	10	15	21	16	18	21	15
202100_at	1360	798	2478	2506	2492	2031	930	810	1781	1945	1498	1560	1374	1515	2059	1712	1342	1129	3360	2547	3407
202101_s_at	622	309	1168	535	748	938	485	379	565	1083	793	617	421	327	287	289	215	220	654	365	644
202102_s_at	971	1588	1142	1234	788	1201	1015	835	934	1273	1289	842	948	1013	1462	1006	681	719	1082	1364	933
202103_at	292	189	265	194	312	374	237	220	296	267	293	197	189	93	152	50	90	73	102	100	109
202104_s_at	888	869	842	846	403	491	779	924	1232	1374	438	544	451	295	164	189	201	185	404	274	227
202105_at	804	965	1511	2280	1603	1974	1420	1467	1084	1182	1822	1415	1755	2112	2131	1492	1705	947	964	2641	1784
202106_at	675	600	750	696	69	407	572	586	693	945	590	524	341	254	180	273	281	291	307	445	464
202107_s_at	3969	1712	3718	940	4496	663	4668	3902	6612	4588	6904	4585	6363	5798	4557	3823	3603	3698	5723	2908	3156
202108_at	999	1297	993	1355	553	988	1285	1147	722	802	742	661	738	720	704	740	678	791	768	631	571
202109_at	2252	2326	2221	2343	1410	1566	2781	2648	3334	2759	2389	1418	875	1135	1658	1412	1588	1274	1309	1321	1206
202110_at	3891	4051	8518	9997	6660	10546	4330	4104	7215	6082	6185	7293	12032	13560	10993	7823	6831	7123	10591	9621	7252
202111_at	855	809	1971	969	30	26	784	892	2669	2157	995	201	248	255	235	416	392	523	790	587	608
202112_at	46	30	42	69	44	35	34	28	32	42	47	63	5	8	10	6	8	5	6	17	4
202113_s_at	746	663	1106	1273	1835	2557	1455	1565	1041	1286	1629	1834	2950	2215	1995	1336	1182	1103	1751	1238	1529
202114_at	675	564	671	722	674	1048	731	702	524	644	796	703	979	1194	1837	1552	1047	1390	1433	1313	1695
202115_s_at	1277	960	922	466	1508	1028	1263	1124	1224	896	843	621	666	398	318	407	317	374	431	240	221

202116_at	1175	631	1040	1064	777	1060	958	1062	665	669	1225	1026	1347	983	1875	2053	1795	1620	948	1774	1658
202117_at	1781	1442	2840	2351	2624	1531	1655	1560	3211	3051	2891	2698	2209	950	1865	1286	1327	1396	3620	2838	2868
202118_s_at	1105	620	423	273	502	508	1921	3134	440	921	1002	901	164	140	188	2788	1776	1880	603	340	753
202119_s_at	3411	3373	920	1260	1673	1160	3807	4062	1100	1590	1668	2075	2365	1720	3116	5533	4659	4996	1769	1632	2241
202120_x_at	3094	2479	3557	3139	3010	2439	3458	2263	3053	2505	2543	1659	2247	1175	1005	1033	1292	1156	2351	1373	625
202121_s_at	2656	2095	1911	4494	2680	4490	2870	2441	1939	2630	2987	3912	6074	4365	3565	1702	1782	1279	2437	2431	1285
202122_s_at	3948	4102	1739	1375	1432	915	3527	2961	1106	1516	1673	1243	998	1218	793	2179	2131	1956	626	410	378
202123_s_at	1081	1062	872	927	686	802	1334	1077	1286	1059	1107	969	458	241	463	610	750	577	581	602	579
202124_s_at	156	133	393	524	322	370	186	207	294	456	402	581	348	253	227	98	127	70	205	199	223
202125_s_at	373	456	1251	1156	1456	2094	721	625	955	1125	966	1467	1061	1062	848	546	688	436	1195	1562	1938
202126_at	1144	1118	903	729	1182	1136	1174	1262	867	900	921	1029	1278	1183	1446	2003	1658	1583	1690	1251	1538
202127_at	492	260	420	372	467	414	420	556	365	421	301	225	251	113	245	262	188	270	573	309	450
202128_at	941	1012	893	922	959	743	1287	1553	886	990	1526	1307	844	598	529	736	609	709	580	424	470
202129_s_at	483	203	521	513	515	669	501	633	416	601	1032	772	508	552	589	311	318	389	332	525	802
202130_at	1230	1494	1603	1868	2344	2431	1157	1292	941	1361	2152	2775	3314	3456	3865	1939	2407	2231	2181	5567	6821
202131_s_at	401	178	370	459	400	598	254	412	310	306	628	723	1345	804	349	188	404	365	866	1568	3172
202132_at	325	181	473	332	590	390	200	205	673	300	395	535	479	487	214	376	387	270	688	209	240
202133_at	896	865	1386	907	972	1046	443	596	2035	573	1096	1566	1798	2107	2208	3403	3229	2611	6790	2148	3402
202134_s_at	733	558	1318	475	856	367	478	375	1805	328	1073	475	299	237	314	316	213	317	1059	188	213
202135_s_at	1130	1341	1088	1588	1950	2313	836	1333	933	1241	1801	1534	1460	952	775	450	454	292	536	548	359
202136_at	1533	2188	1578	2003	1138	1365	1371	1982	1462	1332	840	837	764	909	1243	2791	2587	1708	2402	2800	3555
202137_s_at	503	348	522	345	445	482	331	472	455	602	338	241	129	80	52	139	200	172	370	252	230
202138_x_at	2887	4118	3964	1981	4272	6996	3193	2947	3568	3831	4879	3661	5412	5455	6798	2583	2667	2524	3548	4522	3152
202139_at	1893	2027	1131	1362	926	746	1578	1858	856	1396	1514	1367	2398	2046	1684	1721	1904	1654	1532	1216	1057
202140_s_at	673	1004	522	711	669	426	678	682	407	492	546	413	540	573	313	409	373	370	287	332	249
202141_s_at	1980	2191	1837	1557	2881	2623	2067	1989	2125	1947	2078	2165	3671	3806	4892	2876	3517	2890	3642	3799	3859
202142_at	947	1488	1436	1672	1434	1263	2109	2334	1460	2003	1533	1384	1477	1384	1371	895	1085	892	1243	822	809

202143_s_at	706	664	1270	872	924	809	1332	1354	1193	1112	1160	1217	1901	2181	1228	1006	956	794	1018	865	994
202144_s_at	3220	2517	1919	1234	2214	2237	3756	2915	2535	2333	2209	2012	5026	5925	5624	4860	5576	5995	4535	4813	4667
202145_at	1236	1474	307	36	201	79	1672	1754	264	354	928	617	220	207	224	407	582	499	74	81	45
202146_at	742	1208	1309	476	2951	7130	448	304	246	252	1555	1049	7265	5414	5037	881	562	1390	1183	4261	5797
202147_s_at	606	927	1598	578	2247	3091	469	321	479	440	1519	1159	3143	2330	1908	290	349	400	779	1659	2742
202148_s_at	2842	3134	1919	688	764	1360	3507	2387	1485	1059	702	725	436	488	592	1420	1431	1505	585	638	537
202149_at	38	82	372	728	887	800	34	77	280	384	620	569	1361	1109	950	29	24	28	1237	1239	1337
202150_s_at	130	103	311	808	616	433	113	132	429	519	685	364	236	158	111	51	29	51	138	130	136
202151_s_at	1101	1445	1004	1062	1181	714	983	1247	1216	1304	1777	1419	1775	1914	1252	884	948	694	893	592	447
202152_x_at	478	494	753	1245	792	552	718	277	420	583	553	522	371	374	298	257	207	235	463	405	241
202153_s_at	1115	1421	1629	918	939	466	1562	1015	2006	1920	1717	1624	2028	1889	2280	2398	2187	2553	4002	2167	3415
202154_x_at	5833	4216	5667	1406	14890	4220	5782	6253	4865	4602	8249	14336	16878	15554	4495	1209	1446	1177	3559	1083	811
202155_s_at	652	490	772	656	928	972	692	742	864	871	1028	824	348	263	283	194	189	222	258	283	263
202156_s_at	11	6	4	4	7	51	34	30	4	81	9	5	1	20	15	16	12	6	19	2	9
202157_s_at	193	77	106	140	274	180	127	77	116	215	90	57	34	8	38	17	13	32	48	57	19
202158_s_at	73	26	58	52	54	28	5	9	78	7	4	8	19	17	31	32	27	22	9	16	20
202159_at	1293	865	613	565	667	594	1060	895	846	854	686	769	631	536	520	659	606	809	545	776	685
202160_at	669	968	677	781	951	1551	741	838	557	661	610	499	781	291	349	463	405	607	1021	1312	1022
202161_at	1036	1578	513	422	60	534	1112	944	261	409	1025	479	274	391	452	525	562	664	271	357	197
202162_s_at	589	431	581	619	1126	968	1004	664	859	1224	546	845	436	286	219	274	242	336	448	347	411
202163_s_at	421	296	316	358	408	630	223	389	286	380	232	359	524	410	539	523	439	451	740	713	1008
202164_s_at	919	938	1150	1282	1020	1051	1257	1308	1185	1424	1107	990	571	583	444	559	465	628	691	543	738
202165_at	407	669	594	909	4563	4466	718	722	496	925	3519	3836	5072	3323	3860	1154	1072	696	1105	919	1392
202166_s_at	1611	1463	1472	1150	8712	5936	878	1093	1101	1198	7893	5877	9423	8028	6256	1376	1132	1674	2093	1301	1764
202167_s_at	1402	592	941	857	844	679	941	986	1026	1052	718	602	525	521	494	692	773	752	640	441	674
202168_at	2499	2704	3653	2385	1905	2033	3695	2969	4189	3491	3031	3075	3656	4535	5853	4978	4748	5493	5816	3954	4450
202169_s_at	3703	2297	3352	2255	1924	3350	2544	2648	2327	2746	2031	1998	3397	2534	4665	6343	5188	5738	5798	5213	8147

202170_s_at	1498	540	1557	565	1091	789	1459	1248	1464	1437	1137	1093	1305	1207	2005	1500	1631	1696	1807	1533	2261
202171_at	1626	1602	1461	1998	1643	1509	1728	2361	1291	1307	2261	2030	2114	1870	1676	2473	2452	2379	2519	2187	3554
202172_at	1097	1191	715	1227	1003	1246	986	1326	704	766	1403	1576	1475	1076	864	1091	1109	842	666	653	716
202173_s_at	619	909	1104	1498	750	589	853	1069	823	865	1467	2080	968	819	622	822	786	626	971	502	615
202174_s_at	788	852	651	964	1961	2090	1183	1719	399	651	2199	1903	2545	2201	1726	1227	1216	995	1047	1252	1445
202175_at	2196	1853	1110	678	1067	773	2083	2479	726	572	1487	868	191	82	141	330	316	111	95	150	94
202176_at	874	517	636	626	845	644	916	746	817	553	1124	764	585	582	390	458	529	373	417	246	269
202177_at	421	201	33	26	522	505	370	598	35	17	773	865	527	639	422	150	242	192	27	174	163
202178_at	1176	2269	768	1287	848	2272	988	1009	627	700	763	783	841	847	1168	927	1132	871	636	950	980
202179_at	721	760	621	480	234	231	1056	822	700	595	866	307	293	396	190	435	358	321	393	242	207
202180_s_at	661	1078	735	1423	69	94	712	606	803	907	207	210	62	80	18	506	349	340	336	463	333
202181_at	320	533	517	905	368	830	401	495	514	475	614	568	426	501	420	311	319	274	364	393	389
202182_at	777	788	1238	826	1511	1255	1049	961	930	1263	1049	889	588	368	420	247	255	244	683	427	423
202183_s_at	783	268	545	268	898	266	582	582	494	579	559	508	545	435	261	244	360	344	476	194	231
202184_s_at	1299	1140	1203	1142	997	1058	1064	1178	1080	1312	1025	1116	1176	1004	1463	1461	1118	1512	2233	1599	2251
202185_at	1570	1617	2726	1989	2467	1931	1931	1285	2575	3210	5939	1810	3750	4408	4881	1335	1610	1436	2289	2045	1855
202186_x_at	9	5	7	16	32	32	11	13	36	15	7	5	16	4	12	20	20	5	10	14	7
202187_s_at	775	833	1133	1084	656	570	839	1126	1116	1168	951	807	442	524	671	730	861	726	1021	872	1028
202188_at	1314	142	1207	316	1348	882	1386	1313	2594	1301	1353	769	1032	1105	937	1156	1049	1496	1701	895	1177
202189_x_at	8291	5485	5013	1900	4302	1977	6442	5466	4756	4435	6137	4714	5734	5980	8289	6630	7642	8353	6585	6505	5141
202190_at	689	247	393	520	847	513	517	527	705	426	766	958	913	592	668	449	362	392	817	668	923
202191_s_at	32	10	18	16	241	98	103	95	23	52	85	48	41	33	30	75	50	83	3	59	27
202192_s_at	390	383	423	581	741	954	420	537	138	309	361	349	83	72	89	182	153	174	41	31	94
202193_at	225	198	286	435	576	1537	249	261	458	537	593	418	809	419	470	343	426	342	1406	487	697
202194_at	1646	1086	2794	2222	1486	2720	1028	871	1833	1592	1464	1062	2507	3592	4400	4224	4448	5398	6431	6930	10893
202195_s_at	566	421	1169	669	326	392	521	463	906	862	537	494	276	376	507	592	521	541	1119	1039	1141
202196_s_at	16	6	1	1	264	1730	8	3	3	6	229	119	214	197	167	5	1	2	12	9	1

202197_at	652	782	730	731	405	774	656	860	693	643	878	589	311	387	293	375	319	442	332	262	226
202198_s_at	222	208	94	231	324	337	196	315	199	196	297	221	4	30	66	39	40	51	22	33	52
202199_s_at	867	380	872	683	779	1089	1155	1209	1209	1391	878	969	245	206	251	290	219	236	258	323	284
202200_s_at	1974	1418	1903	1159	1124	658	1822	1987	2052	2003	1757	1806	1880	1540	2480	2988	2282	2623	3520	3178	4627
202201_at	2031	4643	655	2814	632	2193	1995	1413	910	667	723	975	1360	1005	643	948	1082	929	775	684	451
202202_s_at	81	51	5	43	19	44	65	67	83	15	38	6	1	3	26	40	13	29	16	24	22
202203_s_at	1983	2131	568	548	1046	906	1434	1585	526	636	1025	1190	921	527	879	2024	1897	1563	826	616	680
202204_s_at	1091	1345	632	768	572	852	1385	1317	468	696	854	695	273	455	293	413	454	379	348	270	274
202205_at	775	533	2625	1986	739	556	564	945	2173	2450	1269	598	528	682	328	402	245	238	582	364	246
202206_at	269	359	1133	641	1638	1592	386	499	764	664	2889	1976	3062	2087	1167	206	185	100	276	243	255
202207_at	328	420	875	520	2728	2078	232	376	692	629	2372	2104	5313	5030	4151	571	631	366	552	466	688
202208_s_at	265	311	476	317	1364	1137	171	157	185	239	992	640	1933	1431	1087	242	233	128	193	114	72
202209_at	1615	918	2610	1896	1824	2185	2259	1903	2331	1648	2352	1659	4659	3102	2649	2855	2760	3837	4047	2320	2141
202210_x_at	321	406	555	87	1150	594	686	486	233	451	682	367	75	61	142	58	19	60	20	59	66
202211_at	1094	600	728	738	1704	2588	472	573	389	468	792	904	1232	1114	1361	952	1398	1187	1253	1181	1970
202212_at	1197	372	853	369	979	637	1997	1314	923	851	1063	429	697	629	580	823	807	655	608	470	377
202213_s_at	450	560	639	918	1915	4892	880	1201	765	928	2837	2268	1827	1260	1510	1298	1011	943	784	881	1167
202214_s_at	418	1300	883	1242	2269	4034	917	1374	657	767	2635	2980	2640	2357	2819	1951	1534	1608	1101	1524	1876
202215_s_at	705	817	700	680	340	521	572	747	720	776	497	398	560	463	398	471	423	467	852	434	625
202216_x_at	816	900	1069	910	1013	790	815	951	931	955	703	701	557	620	506	518	512	606	508	393	450
202217_at	2097	2303	1096	1661	1501	1433	1838	1519	1044	1371	1630	3339	2612	2878	3012	3228	3621	1963	2311	1557	1612
202218_s_at	205	102	73	52	275	400	151	228	59	63	439	166	147	106	136	55	25	38	25	54	33
202219_at	1189	1407	4204	1965	702	344	1658	1545	5805	4602	1360	747	961	869	696	497	532	430	1811	1403	1054
202220_at	1414	1280	1643	1567	1434	1357	1088	1114	1824	1904	1461	1053	970	922	1891	2569	2207	1875	2057	3679	5142
202221_s_at	377	281	434	453	602	679	245	335	278	349	480	260	255	251	401	357	232	350	702	369	551
202222_s_at	102	69	61	53	405	159	41	42	90	61	185	70	45	73	48	57	39	41	104	46	49
202223_at	1158	805	955	824	1200	1039	1734	951	1330	1058	1212	1003	1864	1860	2535	3082	2647	2202	1877	3152	3236

202224_at	2461	1754	1799	1351	1555	1234	1471	1775	1424	1902	2440	1685	1597	1560	1770	1515	1282	1230	1199	1246	1510
202225_at	1848	1468	1253	1103	2451	1786	951	1199	1049	1393	2064	1822	2150	2406	3199	2817	2722	3107	2370	1822	3005
202226_s_at	1954	982	1280	1032	1416	996	1533	1632	1102	1023	1973	1404	1165	1429	1530	1338	1180	1163	596	670	557
202227_s_at	648	692	1028	1367	1776	1434	908	1234	1018	1637	1134	1678	1872	1556	1283	935	923	743	1897	1535	1773
202228_s_at	4334	4381	6381	8550	3779	3407	3588	4542	6530	7356	3712	2919	5158	5580	4976	9078	7840	8654	12895	15233	16983
202229_s_at	361	291	47	303	76	86	302	344	435	422	401	371	38	62	63	44	74	58	10	32	6
202230_s_at	2846	2021	1348	817	1678	923	1809	1399	2055	2109	1920	1351	2129	1419	798	1381	1355	1245	1848	1058	1095
202231_at	4842	5230	4643	3816	6423	7857	4789	4516	4669	4033	4221	5802	10547	9151	7870	8045	7409	7612	11094	10721	11299
202232_s_at	3702	3518	4213	2777	4922	5897	3612	3353	3025	2664	3503	4340	8232	5805	5919	5860	5273	6083	8601	7706	8609
202233_s_at	7012	8093	9861	8937	4841	4515	10533	10158	11494	8876	6289	5321	8839	7787	6847	10136	10714	10667	9665	9295	6280
202234_s_at	538	468	597	352	1006	644	401	457	962	1126	1138	1627	803	776	1361	970	862	834	1821	855	1185
202235_at	151	190	263	144	554	255	184	161	262	481	327	582	440	195	242	237	185	205	676	440	599
202236_s_at	1502	1663	3473	1093	5499	2009	2732	2256	3964	4105	5882	6677	3042	2868	2741	2113	1711	2160	2745	1265	1969
202237_at	13	7	327	21	323	431	7	11	150	27	24	19	24	40	61	71	52	54	551	1210	761
202238_s_at	37	50	281	63	125	153	36	25	115	38	16	145	20	6	11	10	6	9	149	352	261
202239_at	3245	2265	2142	3207	3111	1633	2670	3653	2054	2850	2196	3400	1819	2027	2240	2314	2869	2684	2394	2012	4002
202240_at	1118	292	1331	167	514	333	1341	1156	1515	1287	1096	573	299	514	761	834	695	852	1539	387	485
202241_at	7270	10360	5994	2124	5047	2184	4744	6352	2927	4908	3951	3300	5768	4507	3802	11012	9377	11665	5270	8266	10069
202242_at	273	208	134	135	184	181	311	413	92	171	97	99	4	19	27	128	90	142	37	33	27
202243_s_at	6142	3304	3595	3381	5943	5592	4647	4494	3368	3119	5017	6999	9229	9034	6668	6189	6444	4998	4237	5635	3290
202244_at	7959	5468	4596	4485	6068	5400	7073	6729	4355	4252	6117	7287	10400	9908	8840	7230	7572	8211	6955	8226	6966
202245_at	1519	1143	1637	1305	1851	1606	1300	1277	1381	1045	1597	1189	1722	1978	3099	2201	2128	1637	1638	2503	1839
202246_s_at	5138	3888	5376	2949	4328	2766	4879	4162	5657	4449	4906	3255	7965	7544	7541	5895	5726	5490	5783	5960	4783
202247_s_at	1289	1072	925	479	595	534	1136	652	685	843	833	276	57	81	120	150	166	145	129	118	82
202248_at	684	303	674	288	826	536	559	445	427	407	475	221	136	47	56	117	118	221	261	121	113
202249_s_at	405	352	528	639	236	168	383	508	404	368	451	276	91	135	243	213	196	249	178	119	141
202250_s_at	513	370	396	780	271	257	538	636	528	630	328	323	204	226	147	280	227	174	360	258	254

202251_at	279	164	654	367	660	403	420	467	514	411	368	298	360	288	229	279	240	300	402	465	404
202252_at	2540	3508	2130	4202	2774	3834	3044	2966	1889	1741	2915	2835	2800	2867	2858	4448	5026	3394	2877	4274	2795
202253_s_at	499	583	747	1248	252	122	513	534	739	887	662	410	196	281	64	128	109	131	155	198	146
202254_at	41	303	228	701	186	388	225	96	286	412	215	231	126	103	127	219	202	246	540	320	419
202255_s_at	327	482	537	742	430	396	374	508	706	444	460	293	298	327	259	284	250	245	498	363	470
202256_at	533	310	522	464	790	820	511	334	512	397	443	449	335	306	277	294	272	204	332	230	223
202257_s_at	672	762	635	551	845	978	543	454	309	541	749	474	492	447	488	241	421	297	225	327	246
202258_s_at	745	833	700	1275	1116	1775	938	1275	601	600	1285	1239	1753	1498	1429	1119	1201	1097	1232	1079	1298
202259_s_at	320	427	358	609	524	668	419	596	302	365	519	510	496	504	341	543	404	496	588	380	628
202260_s_at	1438	2033	730	942	1615	1656	998	1528	607	1662	1981	3031	1256	1281	1886	1574	1609	759	641	1319	1486
202261_at	1660	1875	1394	1507	929	1345	1723	1715	1711	1832	1515	1264	855	651	549	890	1057	891	1094	1304	1102
202262_x_at	525	1064	608	1516	766	1681	588	823	423	496	911	905	779	581	287	248	312	30	59	226	171
202263_at	583	401	476	1058	515	730	486	462	490	753	671	620	494	653	549	590	784	822	926	777	694
202264_s_at	1728	1125	2098	1000	2355	1711	1680	1280	2768	2040	1632	686	1557	1273	917	597	564	787	1497	568	442
202265_at	1004	792	1776	1011	1284	2268	920	909	1056	713	841	777	1526	1497	1463	2155	2018	2563	3737	3647	5098
202266_at	2135	1287	1135	1445	1825	1652	1228	1293	1086	1220	1373	1374	2299	2382	2466	3322	2729	2666	3309	3046	4558
202267_at	445	422	1344	682	852	814	163	397	427	675	929	714	659	733	693	392	552	405	718	1025	1182
202268_s_at	4079	4587	3356	1482	3535	2870	5297	4254	3501	2882	3550	2428	5029	4853	3975	5760	6325	7339	7885	7289	8863
202269_x_at	11	60	8	26	121	106	5	4	20	8	7	5	2	14	6	6	3	36	38	59	51
202270_at	12	78	8	44	33	71	30	3	31	36	6	29	11	31	23	15	7	51	54	93	172
202271_at	718	775	932	1138	737	844	865	805	1193	1481	836	685	721	792	1154	1604	1681	1192	1965	1505	2186
202272_s_at	1287	961	1350	1354	689	734	1639	1411	1123	1392	1508	1017	1002	809	875	2077	1736	2151	2121	1308	2193
202273_at	38	21	19	38	263	312	33	34	40	24	31	28	10	49	30	20	15	16	20	16	2
202274_at	359	56	201	211	335	220	195	206	154	186	232	212	58	93	43	84	104	106	109	39	39
202275_at	1950	2795	7364	8908	863	1126	1617	2588	5788	8022	2194	1557	325	453	542	711	645	863	1405	1108	803
202276_at	1648	1487	3502	2074	3686	3386	3934	4142	3327	3948	8691	8224	10207	8633	6927	2933	2204	2398	2680	2395	1839
202277_at	4094	2596	3468	2837	4486	4167	3079	2816	2914	3446	3759	3630	5418	5480	6605	6979	6675	6037	7419	6575	8982

202278_s_at	1099	355	788	715	972	1036	959	1029	863	1180	1040	1174	619	455	305	393	376	309	395	439	816
202279_at	1376	2074	1545	1589	1377	1377	2219	1974	1449	1326	1960	1944	1946	2316	2444	3881	4186	3177	2176	2635	1762
202280_at	20	15	15	26	14	16	17	17	13	15	14	13	5	3	3	12	7	22	4	4	5
202281_at	848	1136	678	1024	554	482	761	842	672	830	659	259	285	265	236	314	290	368	278	318	262
202282_at	2663	1864	3022	2241	3714	3858	2861	1741	3468	1792	5177	3851	5715	5020	4712	1700	1903	1478	1820	3025	1501
202283_at	40	29	58	44	1067	1094	57	33	37	35	1265	592	1286	1116	780	101	82	37	78	284	291
202284_s_at	2973	5469	585	233	1002	677	3303	2254	44	548	1660	929	1211	1498	1442	2963	2955	2527	196	254	234
202285_s_at	66	54	24	62	43	55	45	91	47	82	51	63	10	11	9	37	7	43	40	53	37
202286_s_at	614	2909	1925	6540	3533	13374	598	478	884	428	3590	3803	4540	3781	7697	1661	1433	1667	1098	1727	1239
202287_s_at	71	12	69	116	197	226	78	44	86	113	55	67	47	35	6	38	41	30	59	6	3
202288_at	296	279	313	206	510	507	387	380	288	358	263	264	208	207	157	124	163	176	269	145	235
202289_s_at	905	957	533	651	473	998	457	475	393	519	212	343	304	216	328	550	453	512	1158	2094	2105
202290_at	3037	2653	1671	958	2977	2338	3442	2631	2592	2598	5733	2720	6035	4230	1911	1206	1386	1002	1255	816	510
202291_s_at	24	26	103	116	19	26	17	26	90	11	21	14	2	3	2	2	1	1	60	1	1
202292_x_at	2040	1281	1595	1680	1238	1086	2822	2282	2070	2249	2292	1273	812	371	425	699	806	925	448	544	319
202293_at	835	506	804	460	1332	910	580	632	821	525	1128	935	1034	966	1171	747	598	589	942	774	888
202294_at	782	366	673	347	2003	1571	518	570	575	650	823	1298	1392	1302	943	567	676	548	1198	932	1012
202295_s_at	840	1334	2503	7639	212	387	616	702	3590	2576	283	246	164	177	138	919	1277	770	6311	7091	4471
202296_s_at	2711	3479	2427	1751	1985	1596	3354	2700	2630	2626	3019	2003	3796	2942	2039	2228	2215	1879	1831	1371	998
202297_s_at	2228	2282	1280	953	1446	1150	1357	1004	1175	1251	1123	909	2104	1586	1527	1559	1873	1786	2131	1843	1287
202298_at	4080	4967	5429	7312	5406	11507	4041	4430	3572	4147	6148	5422	11789	12304	8779	6126	7845	9031	6609	3183	2974
202299_s_at	2469	2828	2227	3235	2749	4678	2968	2799	2494	2158	3350	2546	5686	4351	2948	2665	2519	2633	2461	2621	2057
202300_at	2772	3482	4068	4842	3259	4173	3328	3732	3913	2894	3715	2888	4182	3094	2405	2015	1873	1597	1635	1645	1337
202301_s_at	386	311	676	517	870	656	739	641	892	918	695	545	868	625	326	476	450	461	560	348	622
202302_s_at	1476	1049	1796	1847	1682	1544	1876	1909	2159	1980	1734	1400	2255	2263	3320	3005	3196	3105	3930	2574	3909
202303_x_at	1573	1281	1350	617	1280	782	2140	1754	1250	862	1130	911	1252	914	933	1674	1402	1867	1476	968	1027
202304_at	1031	1117	1164	1199	1028	1789	828	1021	712	792	881	787	1347	1104	1497	1696	2004	2259	1813	2720	5424

202305_s_at	591	1094	1529	1498	579	754	962	1036	1308	1972	981	857	843	837	694	438	675	491	1009	637	405
202306_at	3856	2909	7821	4759	5332	7048	5837	4516	6476	5071	6272	6382	5931	5936	6201	4460	5148	5269	4403	5050	3707
202307_s_at	618	1107	95	269	682	556	798	637	144	200	867	550	390	484	451	643	652	1390	156	395	389
202308_at	968	1136	770	936	246	288	857	969	702	808	514	210	365	339	169	480	535	489	398	428	381
202309_at	3949	1726	2264	872	3031	1849	2726	2799	3072	2583	2540	2560	4216	3050	3648	3599	3408	3378	6090	2689	3394
202310_s_at	192	307	81	193	173	79	204	292	80	78	58	55	41	45	30	141	85	109	39	51	29
202311_s_at	81	56	23	32	113	75	45	55	36	11	37	60	17	31	9	15	33	34	15	27	9
202312_s_at	188	120	132	47	599	255	133	131	161	138	176	65	20	70	82	64	84	28	64	66	35
202313_at	1978	2057	1670	1331	1633	1774	3012	2833	1933	1852	2234	1389	1931	1715	2151	2426	2465	2183	1847	1377	1674
202314_at	2107	1007	1249	469	2750	1364	488	561	482	432	912	741	2363	2057	2848	2562	2004	1839	1839	3467	2753
202315_s_at	778	998	1215	852	693	765	835	736	1193	1463	748	636	302	291	288	485	458	467	681	492	472
202316_x_at	283	238	190	472	37	28	647	276	256	407	571	581	90	108	72	91	120	86	70	57	94
202317_s_at	1077	815	861	1412	1157	1219	1133	1269	1117	1319	1060	1255	534	435	391	689	482	423	803	558	599
202318_s_at	927	944	1049	654	947	1115	824	1144	740	686	712	535	676	612	762	1437	1356	1406	1454	1206	1627
202319_at	144	80	195	114	294	337	111	156	90	158	121	155	133	81	119	143	91	139	278	212	367
202320_at	1136	1580	1247	1187	588	646	1420	1543	1374	1205	1214	522	251	279	211	315	327	396	319	279	287
202321_at	217	196	227	303	81	524	129	218	120	221	144	156	170	165	259	351	247	227	356	613	748
202322_s_at	738	1013	1058	1468	1414	1090	1081	1106	710	1431	1134	1452	1164	901	1296	919	907	953	1480	2442	2408
202323_s_at	392	185	399	361	533	559	419	488	558	661	374	427	324	209	104	140	121	85	127	101	104
202324_s_at	668	1024	1124	1245	896	911	896	781	1044	1064	618	684	1327	1122	2373	2880	2591	2827	2425	2887	3611
202325_s_at	4155	3555	3646	3884	4748	5874	5047	4580	3417	3406	5806	5174	11309	8011	6435	5469	5091	5603	5873	4202	2914
202326_at	688	1010	323	436	63	114	930	878	479	375	398	127	133	204	218	343	445	480	170	166	165
202327_s_at	213	168	178	79	173	75	171	208	86	145	125	108	19	50	9	70	85	10	12	22	15
202328_s_at	184	432	275	202	597	758	288	424	328	292	293	121	40	53	9	7	6	35	15	75	12
202329_at	1334	1392	1236	1366	925	556	1071	705	1560	1342	1003	667	814	357	573	1170	1119	696	1789	1952	1174
202330_s_at	1773	1525	1755	938	2082	894	1817	2233	2751	3146	1720	1474	3498	2496	1705	2497	1893	2212	5324	1433	1847
202331_at	450	521	707	1262	686	946	347	372	670	893	522	764	628	557	542	320	469	283	893	1078	1169

202332_at	200	318	212	202	1089	832	236	231	296	300	391	287	550	466	477	433	359	292	410	373	255
202333_s_at	1857	1365	1991	1698	2711	2763	1536	1646	1319	2056	1842	1896	3817	3361	2184	1876	1778	2367	2474	2465	2390
202334_s_at	919	809	940	719	906	887	492	614	728	748	888	804	1002	750	660	1464	1010	1362	1134	1684	2224
202335_s_at	78	38	109	87	69	67	37	41	62	94	81	114	40	23	37	16	7	12	55	72	56
202336_s_at	2773	10850	1925	4101	5662	7363	3880	5772	1440	1829	4168	3049	6769	8080	10225	7408	7417	7306	3311	5435	6410
202337_at	663	715	902	737	650	548	640	816	1147	901	503	589	468	484	573	648	651	510	1359	860	733
202338_at	1822	1174	2126	679	1769	308	2113	2935	2571	3008	1721	1919	2618	1073	802	1394	1272	1473	2794	1044	723
202339_at	291	234	372	288	99	234	253	211	413	291	155	52	93	54	54	63	16	79	107	39	56
202340_x_at	115	422	76	57	279	278	50	73	135	66	67	83	138	104	187	219	162	204	185	196	103
202341_s_at	577	747	209	332	1045	781	362	505	112	148	495	415	1948	2259	5324	2356	2018	1404	1467	1451	1962
202342_s_at	976	858	475	623	2814	2543	608	936	250	369	1700	1352	2702	2222	3333	2391	2238	1752	1792	1507	1978
202343_x_at	6312	8959	4739	8150	4218	6143	3783	3967	3507	4683	4819	10083	6979	7266	6512	4888	5596	4734	5414	7114	3792
202344_at	839	1297	651	384	274	320	759	715	478	512	630	307	268	214	131	294	303	469	301	314	278
202345_s_at	7325	5294	2564	171	2294	774	8007	8208	3433	2278	3129	2241	5726	3214	3702	7240	7066	8745	5546	1850	1964
202346_at	1674	1755	1008	861	950	1101	1692	1796	1264	1231	833	778	688	579	1067	2355	2019	2210	1400	850	1514
202347_s_at	1843	2011	1285	864	1676	1371	2374	2338	1983	1532	1835	1612	1952	1611	1469	1952	1565	2813	1656	1615	1215
202348_s_at	1150	698	1035	859	925	1153	917	924	694	913	1313	1191	1450	1204	960	686	627	848	611	704	959
202349_at	757	875	609	804	968	1064	804	681	601	723	999	966	672	672	1057	803	915	1008	850	1111	909
202350_s_at	143	439	41	85	231	183	134	215	20	56	82	156	135	69	33	111	113	92	35	24	49
202351_at	856	849	2170	1615	1117	1360	729	1095	1646	2120	1725	1808	2235	1952	1095	1365	880	1245	3015	4345	5510
202352_s_at	4782	4164	4613	1972	3631	1806	5223	4045	4926	3297	4465	2828	5048	5196	5874	6214	5429	6532	7045	3391	4425
202353_s_at	1654	1152	2565	2443	1567	1050	1948	1808	3052	2876	2274	1827	2621	1855	2029	2125	1786	1788	3895	2038	2068
202354_s_at	520	609	427	398	488	363	924	744	729	875	881	504	568	322	166	220	233	179	152	124	126
202355_s_at	839	1006	646	645	794	903	991	945	771	742	948	676	731	734	592	596	698	693	454	508	525
202356_s_at	1343	976	841	967	678	543	942	1283	1069	942	1209	752	708	560	610	546	674	481	683	616	654
202357_s_at	73	86	95	458	40	56	90	139	149	130	125	74	19	6	5	25	12	70	42	72	74
202358_s_at	122	260	415	515	279	786	423	351	308	449	668	513	253	192	228	182	207	145	244	302	383

202359_s_at	303	206	281	450	334	206	213	245	281	377	271	391	332	185	205	205	223	252	608	546	743
202360_at	1277	1639	1137	1336	1168	1266	1257	1375	1124	1266	1559	1355	1336	975	937	735	965	1033	1485	1183	973
202361_at	902	473	1359	1068	709	982	1044	1013	1520	1121	863	535	678	521	339	439	352	374	538	823	670
202362_at	1774	1468	1259	1526	1456	3674	1715	1748	1626	1706	1360	1449	2211	2563	4075	3802	3264	3343	6026	2771	3361
202363_at	90	123	85	74	230	231	113	191	146	217	129	85	11	48	2	5	5	3	4	22	31
202364_at	1994	3103	1455	2711	522	1439	2308	2716	1370	2502	631	1071	616	573	1066	3252	3854	2451	2299	2668	4061
202365_at	1033	1204	852	1186	1693	1953	863	1075	959	1381	1118	1326	1528	1148	1519	2070	1801	746	1588	1452	1091
202366_at	275	201	137	402	122	94	110	257	229	316	161	239	41	97	22	93	117	104	146	117	45
202367_at	631	1395	517	1080	399	1965	635	723	333	510	607	530	395	378	663	474	503	615	460	453	375
202368_s_at	537	524	707	502	56	208	538	566	609	964	455	368	147	194	176	249	287	222	259	165	209
202369_s_at	1095	723	1023	666	1032	1486	1134	1089	1019	1199	1020	818	728	738	675	1731	1282	1278	1025	930	1289
202370_s_at	3744	4028	4720	2686	4919	1715	3448	3193	4346	5371	4647	4342	4674	3063	7313	7077	4205	6208	6156	5420	8490
202371_at	898	1715	652	1039	2977	6243	918	1182	432	419	2408	2432	5536	3755	2779	1611	2195	1039	1349	2025	1573
202372_at	673	865	874	808	821	1051	837	875	642	1040	540	599	372	400	935	685	711	660	953	1044	1824
202373_s_at	648	511	1106	961	1199	1306	701	685	902	983	765	857	692	690	941	507	576	697	1214	1848	2739
202374_s_at	417	241	659	601	700	679	543	327	531	523	482	388	142	136	131	164	146	116	178	255	298
202375_at	553	242	305	153	175	775	188	195	36	109	132	167	164	177	188	787	687	884	341	659	1110
202376_at	60	109	247	140	58	36	74	323	233	222	237	114	4	12	4	122	106	63	11	198	171
202377_at	1300	1236	1559	2205	1073	1494	1630	1554	1504	2456	1929	1139	909	1042	1205	1682	1931	1683	1672	961	1576
202378_s_at	497	187	1291	2537	832	1293	792	764	1675	2444	1940	1766	1512	1695	1435	207	181	176	1222	1116	879
202379_s_at	291	496	537	614	186	310	588	1058	811	542	543	676	588	430	399	615	503	438	538	663	483
202380_s_at	435	384	434	597	625	466	563	656	530	616	598	342	158	169	141	199	209	155	198	219	208
202381_at	3794	3677	4031	2232	3046	3658	2123	2291	1762	2405	1808	1538	3622	4488	5941	9751	10662	10160	7216	7395	11412
202382_s_at	1218	884	1059	1198	1776	3466	1219	1242	1143	903	1586	1209	2499	2094	2245	1070	1100	1066	1812	1644	1798
202383_at	614	573	958	1114	711	892	863	842	817	964	972	571	107	138	147	282	250	246	331	185	143
202384_s_at	553	221	581	146	833	672	615	556	761	580	587	283	451	315	314	196	174	134	318	215	144
202385_s_at	848	597	1050	436	1159	790	1134	781	1180	1062	1187	611	615	644	833	641	618	703	1098	507	423

202386_s_at	516	637	553	855	399	437	371	735	333	570	308	511	210	217	249	698	727	603	418	732	1007
202387_at	597	545	525	546	708	891	620	658	838	713	577	757	584	447	328	599	639	470	798	590	727
202388_at	421	799	1199	595	190	175	595	814	834	621	30	50	51	62	77	1611	1509	2074	1462	1348	1695
202389_s_at	263	296	324	313	294	308	280	326	425	382	301	201	187	128	94	61	107	64	189	154	112
202390_s_at	33	92	90	190	107	60	46	51	119	123	50	40	64	66	21	62	36	50	81	76	54
202391_at	132	67	133	177	129	243	97	132	66	145	112	94	1	18	14	14	26	16	30	2	5
202392_s_at	647	551	522	362	99	125	513	562	594	421	456	276	348	352	249	407	367	556	267	144	124
202393_s_at	2961	1516	1732	679	929	427	2936	4060	923	1951	1768	2349	1870	1621	1670	3273	3022	3071	2623	1661	2873
202394_s_at	757	353	521	563	535	476	779	544	582	662	716	632	314	364	273	347	374	343	220	151	161
202395_at	1201	1832	1369	1677	1178	1395	1942	1866	1832	1356	1484	1470	1836	1794	2532	2812	3030	3082	2316	2013	2808
202396_at	1630	1271	1191	661	1666	977	1664	1520	1553	1495	1460	1004	1912	1611	1658	2200	1601	1754	3478	1931	2536
202397_at	7799	4957	1702	1120	649	404	7342	6356	2653	2654	1777	1849	5807	4698	4677	7119	7255	7079	7287	5994	5053
202398_at	64	111	241	182	183	196	130	164	328	231	190	145	126	107	114	94	83	125	165	159	167
202399_s_at	1655	804	1909	2360	1777	2216	1199	1173	1783	1845	1272	1561	1324	1421	1751	1142	1326	1325	2633	2060	2079
202400_s_at	197	195	103	98	125	39	166	137	201	119	220	132	42	60	6	48	7	12	43	12	18
202401_s_at	243	277	312	144	405	442	400	263	277	291	274	308	202	95	114	341	296	260	269	202	196
202402_s_at	1226	652	766	393	614	1118	800	420	486	299	600	457	634	509	630	981	838	733	294	715	624
202403_s_at	159	11	273	160	459	560	201	143	144	183	196	209	34	8	33	36	53	16	69	8	6
202404_s_at	50	7	66	87	136	22	23	15	75	34	54	41	1	18	3	15	21	3	20	16	9
202405_at	549	485	182	202	135	141	428	525	235	166	142	176	202	193	347	1133	1059	1015	687	393	560
202406_s_at	2667	2523	2235	1865	2319	1759	3167	2639	2732	2134	1842	1596	2086	1730	2056	2929	2417	2612	2654	2739	2658
202407_s_at	497	547	595	400	438	323	612	772	1019	1069	601	408	800	619	164	173	198	172	347	92	174
202408_s_at	1614	1556	1270	1458	1398	873	1276	1488	1944	1788	1229	1116	2455	2231	1139	1153	1301	1164	1976	922	920
202409_at	442	513	300	322	689	497	560	546	238	309	586	287	110	95	132	167	122	176	176	151	114
202410_x_at	21	12	9	14	59	31	15	22	17	10	11	16	6	3	3	9	6	10	6	3	5
202411_at	32	269	760	3850	416	611	252	132	1349	3126	206	229	174	168	276	23	223	3007	475	4296	3022
202412_s_at	430	227	654	369	503	430	640	616	1042	1175	1168	1054	2448	1242	618	403	381	339	779	500	367

202413_s_at	1740	1285	2973	1273	3930	1906	2011	2077	3479	2974	3721	3004	6051	6814	6847	4082	4363	5234	7386	5837	7787
202414_at	179	238	420	600	613	727	236	218	336	231	304	486	390	290	285	210	165	192	322	504	537
202415_s_at	1028	717	1040	665	1061	879	1022	918	1366	1027	1322	765	808	627	557	389	318	256	509	556	286
202416_at	1273	549	1374	881	1911	922	1672	1324	1616	1114	2429	1365	2423	3523	3191	1936	1919	1135	1401	1600	975
202417_at	1884	1664	1004	1051	713	434	1774	1545	1218	932	1504	1206	1234	1252	870	968	1053	692	817	452	365
202418_at	905	1219	2665	1915	1742	2047	1284	1016	2585	2853	1748	1570	1811	1852	2284	1197	1267	851	2969	3351	2326
202419_at	806	736	750	619	421	520	734	933	910	1083	620	514	788	1085	1426	2404	2329	1894	2207	1928	2105
202420_s_at	1794	687	1443	964	3530	1881	2029	1960	2333	1621	1612	1327	1779	1288	1192	986	807	722	1564	838	1291
202421_at	655	826	941	1950	522	617	1166	1148	1093	1572	674	675	314	389	290	494	478	608	818	846	1027
202422_s_at	439	310	532	431	700	470	172	234	234	444	463	357	785	483	481	439	281	264	998	818	812
202423_at	447	436	1270	1134	1567	1386	705	592	1209	1349	1629	1296	739	834	703	131	81	135	663	556	682
202424_at	2565	3701	1275	2612	524	1467	2967	2523	1506	1739	2960	474	1257	1190	593	860	890	1164	721	584	406
202425_x_at	2152	3004	936	1037	323	835	3049	3143	1093	1107	775	523	818	902	845	7313	4891	3692	1919	2429	2703
202426_s_at	213	280	161	407	789	787	254	289	280	215	277	134	155	120	225	98	163	150	150	153	95
202427_s_at	2466	2635	2078	2665	1656	1930	1985	2011	1908	1864	2301	2429	2077	1886	2205	1533	1460	1496	2555	2077	1586
202428_x_at	8657	8906	12382	19094	9145	4920	8248	6511	14194	11614	8232	6481	10118	9701	11240	9724	10518	9230	11475	10896	10907
202429_s_at	3970	5467	1753	1909	944	882	3507	4199	1135	1987	898	891	1150	1226	1948	7747	7889	7029	3246	3290	5213
202430_s_at	401	498	886	674	404	355	329	305	359	584	340	390	746	421	421	466	538	1395	2017	3960	6960
202431_s_at	6453	4542	9014	1696	3458	3345	4712	4933	6989	9061	3381	6712	4401	8656	15431	16694	15314	20369	9902	13903	13297
202432_at	1124	871	662	424	683	982	674	922	595	836	740	582	1034	968	832	1524	1681	1245	1754	880	1455
202433_at	2868	2275	2657	1467	1284	1369	3334	3043	2364	1764	2480	1503	1891	1663	839	3060	3953	3342	1406	1877	1527
202434_s_at	20	9	87	537	63	24	16	18	120	68	11	15	3	4	2	2	33	2	6	2	4
202435_s_at	60	120	498	5476	291	144	118	106	689	706	91	65	82	46	36	41	40	26	1379	659	1061
202436_s_at	319	612	1428	16170	264	226	250	318	2056	2109	195	152	84	90	73	102	100	72	2164	1431	2451
202437_s_at	98	487	1050	8375	107	152	72	104	1651	1379	47	50	31	58	40	117	76	68	3119	1085	2303
202438_x_at	78	73	266	154	139	137	118	133	171	232	281	300	64	41	50	78	95	55	87	119	135
202439_s_at	210	237	819	826	364	546	319	289	391	485	621	715	409	404	401	210	224	240	293	415	498

202440_s_at	194	218	471	574	221	314	266	216	476	460	443	375	211	214	244	93	168	82	240	235	242
202441_at	1737	1730	1824	1182	2043	1724	2276	2395	1745	2061	1499	1817	1858	1727	1453	3510	2943	3201	2539	2094	4029
202442_at	11241	13153	4322	4630	5974	5541	10621	11050	3729	4040	6121	6599	9854	11336	9181	16661	16940	17287	6180	6307	5763
202443_x_at	1385	1398	1869	2050	2032	2200	1683	1813	1972	1911	1859	1856	1080	1325	1242	1379	1396	1632	2064	1327	1374
202444_s_at	717	418	1036	788	521	578	1302	1668	1148	1174	1145	900	123	159	192	406	360	479	364	174	401
202445_s_at	116	100	164	185	132	211	105	179	80	194	118	187	38	41	53	64	69	78	95	94	94
202446_s_at	1183	1428	3284	2640	1555	1277	953	1254	2234	2665	1293	1720	1632	1343	1480	1789	2257	5999	3837	7809	10274
202447_at	966	1175	650	1271	386	658	1536	1532	425	442	335	270	300	352	376	1906	1955	2026	650	861	779
202448_s_at	255	298	285	407	232	280	322	351	317	354	509	282	32	23	22	37	25	23	20	22	5
202449_s_at	1040	1482	952	2509	1023	888	1100	1284	1086	1405	1286	875	887	930	338	315	305	293	627	579	350
202450_s_at	196	268	332	480	405	55	318	422	314	370	360	263	78	85	116	80	80	68	102	97	116
202451_at	738	972	1100	965	1039	2354	825	594	824	714	1042	1049	1661	1858	3032	1850	1570	1641	2292	3104	3552
202452_at	261	336	342	482	139	210	114	284	233	212	327	307	90	95	168	136	92	96	130	143	157
202453_s_at	557	333	1020	709	1075	899	867	666	1149	929	1271	970	737	947	865	567	642	588	930	716	1062
202454_s_at	402	472	1043	3526	683	517	448	607	1215	2546	807	659	413	493	609	232	177	181	1857	1731	2579
202455_at	291	448	210	554	71	94	420	462	272	432	318	304	93	131	206	64	101	115	132	185	102
202456_s_at	34	41	39	119	73	67	25	30	17	36	166	134	48	75	60	56	17	12	11	21	8
202457_s_at	4036	5115	1517	1513	856	783	5458	5673	1109	1902	831	637	1096	1414	1815	8437	8580	8510	2680	2440	3923
202458_at	423	1187	1828	2716	1397	1380	372	373	1789	1836	931	617	998	1435	1363	334	386	281	2607	2052	1475
202459_s_at	480	421	1944	2283	707	1387	537	544	1631	1857	942	676	262	290	287	155	153	98	573	1037	917
202460_s_at	591	541	1184	1112	724	1371	301	482	983	1104	685	905	350	462	377	182	238	273	918	1130	1511
202461_at	1443	1062	1428	1018	2966	2532	2005	1247	1361	1212	2415	1756	2560	3390	3327	1951	2035	1908	1791	1685	1339
202462_s_at	2879	1322	2657	2085	2355	1142	3161	2506	4810	2734	2395	1889	2379	1732	2425	3584	2975	3123	4312	2987	3159
202463_s_at	488	561	351	396	411	453	596	509	566	558	622	246	211	149	121	188	141	230	157	171	145
202464_s_at	525	805	699	582	309	380	502	463	565	525	387	387	224	189	458	1234	1122	1108	1003	644	769
202465_at	64	52	54	66	63	165	140	63	47	52	222	62	76	10	43	16	37	8	27	11	12
202466_at	1914	1966	2509	1217	2221	1011	1927	1748	2617	2832	2112	1923	1237	1696	2060	1773	1700	1808	1725	1394	1517

202467_s_at	2004	1993	2986	2900	2753	2064	3869	3150	3761	2795	2859	1857	3392	3608	5303	6791	5707	7979	5092	5550	6902
202468_s_at	2651	2514	2505	617	4042	2799	1725	2964	1361	2053	3614	3873	6187	5041	4372	3956	3648	4783	3894	2233	3091
202469_s_at	3270	1939	2310	1364	2551	1653	2355	2590	2286	2552	2332	1664	2157	2079	1554	2642	2464	2549	3389	2562	2400
202470_s_at	251	165	183	138	23	32	200	261	294	207	234	258	211	252	379	410	260	349	505	256	350
202471_s_at	1337	1434	3043	3699	1214	1050	1623	1458	3582	2619	1757	1335	2787	3079	1824	1663	2193	1529	3006	3172	2680
202472_at	421	299	502	585	206	404	454	409	416	471	348	364	268	210	328	302	259	366	437	259	308
202473_x_at	30	24	26	21	443	175	58	36	70	77	85	20	51	48	10	33	18	62	55	82	74
202474_s_at	1082	922	1295	907	1414	1046	1422	1310	1955	1518	1592	872	388	344	278	263	331	230	292	212	208
202475_at	5177	4422	3884	2987	2522	3463	4477	4483	4994	4225	4092	3205	5845	5094	5068	4196	4299	3747	3727	3429	1957
202476_s_at	245	297	429	433	289	370	327	268	601	486	519	250	84	106	54	6	34	50	132	41	67
202477_s_at	988	1029	1150	1167	936	1025	739	647	1488	1298	1003	610	661	646	511	300	324	414	561	593	415
202478_at	351	96	69	18	895	938	368	637	92	164	767	287	960	1099	921	329	310	211	4	13	37
202479_s_at	414	139	149	162	360	402	608	674	64	159	478	191	107	143	120	58	74	40	49	24	6
202480_s_at	254	133	206	257	286	212	358	239	246	238	176	186	122	121	86	121	161	184	142	106	106
202481_at	1713	4305	794	1584	440	450	945	775	298	414	618	386	279	451	464	1355	1754	1023	373	784	498
202482_x_at	130	41	72	45	220	114	187	127	365	200	213	39	54	96	17	18	65	5	36	9	13
202483_s_at	9881	5917	13339	3979	6112	2102	8010	6665	16154	12509	6584	5384	9810	8020	6643	5584	6337	6653	12258	7158	5471
202484_s_at	1865	1702	884	524	1098	895	1754	1581	938	857	1165	783	1389	1351	1391	2870	2751	3298	2329	1393	1710
202485_s_at	172	192	195	239	397	345	293	343	169	254	200	196	48	37	9	35	43	9	57	85	33
202486_at	2090	1139	1910	1367	1053	942	2156	1669	2162	1922	2058	1308	925	725	735	408	452	370	1248	819	967
202487_s_at	5076	2899	5591	5632	5604	2180	4933	5057	5564	6577	7369	8698	7338	6068	3974	2039	2517	1871	2776	2003	1363
202488_s_at	803	674	713	2237	902	792	841	821	792	936	726	579	121	161	218	322	284	246	430	619	529
202489_s_at	115	735	1126	8604	41	65	739	563	1355	2950	28	33	12	28	44	261	207	185	1161	1619	808
202490_at	41	52	88	109	40	50	40	82	110	85	94	63	70	77	55	75	61	39	158	75	107
202491_s_at	2106	1665	1626	1223	1966	2141	1444	1239	1385	1248	1558	1630	1547	1758	1817	1799	1653	1640	2208	2138	2496
202492_at	679	689	631	812	327	453	596	657	769	746	709	431	194	217	180	245	290	356	179	184	206
202493_x_at	11	9	9	9	21	31	8	11	7	8	7	6	2	2	1	1	2	3	4	29	1

202494_at	878	345	552	391	922	840	918	678	475	514	360	465	473	339	426	677	592	622	470	417	476
202495_at	988	890	437	617	543	586	763	743	492	639	581	651	704	335	270	438	331	619	680	615	474
202496_at	775	617	503	374	825	419	674	690	684	586	553	274	298	325	170	188	201	173	258	269	178
202497_x_at	42	34	18	22	10996	17520	45	41	27	21	12322	9095	9115	10524	5976	145	207	129	39	116	108
202498_s_at	97	16	19	49	3989	8196	57	117	12	39	3559	2370	5767	6810	1483	7	25	12	22	56	43
202499_s_at	108	43	11	14	9817	16193	102	115	58	4	8823	6103	14964	21533	14528	212	267	181	29	180	138
202500_at	318	719	197	570	286	567	219	251	116	95	503	339	337	213	110	9	79	42	34	92	41
202501_at	775	569	525	647	621	400	990	978	621	655	429	632	223	218	204	561	707	591	447	329	469
202502_at	1339	1071	1191	1473	1064	1118	859	1576	693	1578	1106	927	2353	1712	2283	3807	3022	3696	3491	3891	5609
202503_s_at	5255	3575	9586	3410	6093	837	9433	9480	11470	9490	7871	6319	9036	11253	11301	11467	12646	15673	11681	9251	8917
202504_at	242	259	397	494	2830	3888	240	232	431	834	9064	3158	3504	4435	4637	19	46	35	507	702	704
202505_at	3782	3517	2474	1502	4080	4404	6255	5286	3145	2741	5096	4218	7245	6214	6355	6355	6667	6223	6385	4788	3994
202506_at	6603	3979	3207	1649	1249	1263	4868	5872	2277	2591	1130	1122	1231	1183	1126	5902	5018	6099	3154	1987	2577
202507_s_at	242	210	220	327	372	496	235	305	205	273	281	269	48	79	84	99	65	75	113	109	57
202508_s_at	53	2	8	18	132	110	61	24	43	31	63	77	34	60	94	5	8	2	4	4	2
202509_s_at	138	107	68	110	29	46	82	137	74	105	357	55	17	3	40	29	20	11	16	4	17
202510_s_at	758	2418	328	1059	862	743	1530	723	803	447	3888	1953	177	163	234	807	938	678	142	159	134
202511_s_at	1503	1969	1575	1592	1277	1529	1582	1492	1572	1442	1172	1196	1599	1512	1612	3355	2382	3201	2778	2197	3141
202512_s_at	168	292	210	357	176	547	166	157	221	317	189	225	287	314	301	484	439	448	660	661	1115
202513_s_at	1461	1060	1332	1115	1201	875	1677	1485	1939	1722	1406	1042	454	485	434	464	388	526	489	378	361
202514_at	718	882	627	719	2574	3509	1022	940	567	513	3978	2090	1360	1179	1253	933	776	653	453	593	709
202515_at	1185	2108	1394	1579	4533	5296	1512	1430	1032	1142	4671	3668	2474	2350	2653	2125	1818	1360	1460	1530	1400
202516_s_at	216	148	308	256	965	1528	236	317	250	212	921	689	326	374	144	174	187	101	119	93	158
202517_at	152	127	332	277	650	778	212	150	324	249	468	459	171	191	169	19	50	83	50	19	43
202518_at	742	665	1198	1098	931	864	723	982	1394	1310	1650	1142	1284	950	796	561	509	496	722	558	320
202519_at	773	1615	1068	3913	822	917	1249	810	1690	1648	831	475	378	269	325	778	677	599	1374	1231	1424
202520_s_at	483	532	1556	1123	2441	2025	557	523	1255	1378	2382	2672	2426	2270	2291	342	432	528	2121	1630	2181

202521_at	2356	1650	2044	1736	2037	1853	2026	2296	2031	1739	1694	1792	1092	1400	1288	1923	1774	1680	2159	1673	2190
202522_at	2695	1652	3040	1965	3664	2611	3612	2907	3690	3758	3526	2511	2642	3507	4260	4484	5064	4666	3833	4762	5652
202523_s_at	179	196	220	133	120	103	258	401	286	299	200	226	76	64	72	67	39	73	71	52	66
202524_s_at	336	218	271	301	403	503	355	375	250	288	217	268	55	85	58	71	106	46	88	77	56
202525_at	508	914	758	1993	49	26	506	497	475	465	109	68	23	20	23	175	233	199	212	270	248
202526_at	81	66	60	66	76	52	70	115	43	48	51	54	1	23	22	82	57	54	26	22	13
202527_s_at	1020	979	130	80	122	78	639	1004	90	144	44	73	29	30	38	1149	884	946	93	69	153
202528_at	1115	855	639	759	792	976	1030	814	490	493	732	522	1899	1902	1004	821	739	775	489	529	378
202529_at	1846	2050	1549	2058	1748	2193	2482	2650	1537	1677	2068	2060	1868	1806	1632	1967	1903	1781	1567	2051	2049
202530_at	631	809	524	759	632	680	922	932	631	686	775	826	587	765	1074	1130	930	781	731	693	883
202531_at	605	1118	526	847	664	759	545	545	468	560	1082	946	454	602	421	327	399	424	255	366	295
202532_s_at	1004	314	1042	435	1216	438	531	1130	682	850	843	1076	1419	1412	693	781	617	876	2627	970	1429
202533_s_at	740	223	473	199	405	58	449	476	606	604	408	380	517	320	261	430	295	472	689	169	272
202534_x_at	1689	921	1914	920	2524	261	1292	1751	1450	2552	1846	2237	4824	3531	2533	1895	1657	1667	6261	2660	3022
202535_at	927	837	1617	1289	1692	900	682	408	1714	1870	1425	1531	2079	1706	790	449	570	231	1525	875	626
202536_at	2058	1367	882	229	914	1132	1265	1452	512	777	993	1072	1956	2034	2016	2541	2286	2637	2064	1702	2945
202537_s_at	771	771	872	578	1012	1363	897	879	549	769	1039	1082	1798	1730	1732	1012	869	829	1132	1014	1036
202538_s_at	1734	1015	1367	520	1524	1230	1545	1587	959	1132	1140	877	1159	1130	1688	2041	1425	1512	1576	1125	2050
202539_s_at	2347	630	1700	855	1155	660	1276	1374	1187	1208	908	480	1007	1084	897	1997	1512	1740	1460	1838	2428
202540_s_at	1974	788	1206	680	1419	683	822	1159	979	710	462	420	993	1099	1266	3074	2344	2017	1475	1685	2300
202541_at	635	619	799	507	755	766	829	697	801	744	675	514	931	411	599	1024	1000	1128	2264	867	1470
202542_s_at	1996	2555	2113	1599	2328	2139	3570	2522	2902	2179	1944	1694	2268	1867	2266	2735	2569	2549	2262	1750	1904
202543_s_at	1070	955	970	498	745	626	1772	1709	876	660	792	548	674	761	1405	2630	2472	2996	1562	912	1293
202544_at	1647	1882	1782	2116	1710	2528	2169	2023	1682	1442	1690	1306	1987	2832	3583	5063	4900	4827	3463	3445	4819
202545_at	291	361	446	1147	30	62	310	432	337	602	743	268	116	284	127	113	166	188	252	286	239
202546_at	527	686	3390	4225	3326	2721	1656	1421	2795	2373	4202	3712	4872	3214	3535	1072	877	924	1986	2152	1163
202547_s_at	382	425	482	438	319	384	501	709	583	580	642	584	80	118	152	101	128	175	175	109	133

202548_s_at	1199	1474	893	1270	1112	1294	1147	1116	1242	1655	1076
	1498	1059	878	925	1050	953	790	978	1220	1219	
202549_at	20	12	14	23	40	99	27	24	21	131	85
	134	12	4	2	10	7	3	5	3	3	
202550_s_at	1298	1185	2252	1670	2381	1908	1497	1193	2166	2407	3401
	2022	2811	2403	1736	925	829	651	1583	1452	979	
202551_s_at	312	523	606	263	482	710	192	364	286	498	679
	1048	811	778	350	490	469	421	493	388	446	
202552_s_at	142	202	349	181	399	837	125	165	187	218	294
	450	566	428	232	403	322	402	643	449	502	
202553_s_at	1502	1433	689	917	1451	1481	847	1128	599	580	830
	887	1359	1087	970	1516	1415	1235	1008	1403	1220	
202554_s_at	155	33	84	83	216	267	143	149	67	172	71
	161	15	34	42	22	47	58	25	14	21	
202555_s_at	28	30	164	392	54	267	56	102	41	401	111
	109	46	9	12	20	9	30	139	74	82	
202556_s_at	1154	727	1044	938	624	703	1336	1411	1486	1336	1165
	985	649	556	625	708	620	601	914	493	531	
202557_at	848	844	779	184	1304	2743	395	560	234	291	910
	1174	1588	1674	1589	1645	1706	2404	639	1847	2977	
202558_s_at	482	588	564	156	696	2391	429	472	150	210	512
	726	581	447	525	864	821	1141	630	991	1766	
202559_x_at	2368	1505	1198	1615	1480	1501	1567	1305	2299	1537	1143
	1069	909	932	551	1213	1198	987	1047	995	826	
202560_s_at	2773	3933	2245	2073	942	1185	2944	2949	2645	2084	968
	1159	868	674	1000	2829	2406	2210	2209	1265	1385	
202561_at	630	646	353	429	455	462	890	1423	297	411	271
	519	171	159	183	613	526	529	316	648	754	
202562_s_at	1626	841	1369	1529	2023	1455	1036	1337	866	959	1762
	1105	1970	1835	2739	2369	2212	2163	1762	2553	2006	
202563_at	30	5	37	36	285	151	36	40	11	15	34
	25	22	36	7	22	46	31	11	36	10	
202564_x_at	1294	1560	1373	1324	822	1113	922	962	739	1126	945
	961	807	840	406	527	815	556	684	835	527	
202565_s_at	700	707	827	546	657	1437	504	395	396	475	544
	760	305	403	259	529	558	483	689	809	1039	
202566_s_at	340	254	513	195	313	461	284	178	261	404	472
	606	86	119	51	80	52	82	83	118	101	
202567_at	2732	1826	4048	2469	3832	2625	4179	3523	5449	4018	4556
	3373	7102	6236	6071	7244	7514	6432	6072	4954	3041	
202568_s_at	745	588	690	631	926	958	856	767	619	597	937
	783	597	728	671	507	602	598	381	575	612	
202569_s_at	533	251	355	270	1027	919	281	358	248	298	394
	327	505	375	384	434	577	347	410	380	437	
202570_s_at	319	398	308	371	370	379	156	136	309	264	429
	400	417	221	159	98	96	207	198	275	185	
202571_s_at	353	268	335	649	621	595	277	230	384	335	269
	199	127	102	92	83	114	72	182	96	114	
202572_s_at	437	533	1031	1201	478	382	531	528	582	751	709
	352	156	167	249	206	168	271	313	232	270	
202573_at	1742	1772	985	777	1823	2559	886	1172	852	1036	1309
	709	794	943	680	1335	937	1329	1081	816	635	
202574_s_at	491	748	381	401	32	47	550	570	380	348	507
	187	314	288	84	160	148	126	156	106	57	

202575_at 1893 2608 2273 5737 2172 809 6008 5026 4105 2354 3455
1979 1868 3361 6302 3941 5796 3832 2036 2089 1512
202576_s_at 1164 584 1248 910 267 817 1126 1123 1409 1568 1103
656 680 499 527 683 669 780 944 1169 1106
202577_s_at 1306 633 667 411 1583 1506 1024 800 698 616 597
774 588 574 332 452 495 528 574 418 430
202578_s_at 845 297 613 392 520 564 877 797 759 805 789
582 310 232 265 416 367 402 218 145 262
202579_x_at 3298 3490 2637 2055 2460 2474 3209 3675 2353 2830 3577
3332 3120 2894 3428 4840 4506 5829 3621 3735 4930
202580_x_at 1233 277 2113 213 1783 77 1272 777 1973 1500 3192
1791 2122 2054 1842 680 665 784 1302 501 577
202581_at 6874 5713 1519 3101 1498 1351 3954 4058 2633 3529 1416
2201 1763 1552 1063 4980 4405 2763 4926 3445 3543
202582_s_at 2954 3299 1796 1477 2220 3089 2206 1950 1441 1325 2065
2540 2470 2540 2967 4307 4798 4181 3312 3757 4104
202583_s_at 1364 1170 1392 896 1135 1281 2104 1814 821 1001 1647
1041 683 727 441 686 669 679 301 386 407
202584_at 421 266 446 524 378 555 469 429 554 506 510
437 289 286 265 211 273 178 391 376 318
202585_s_at 224 124 300 149 60 199 250 228 285 246 212
226 86 86 121 109 108 100 97 103 81
202586_at 165 158 119 30 65 62 270 170 118 70 313
255 614 398 282 162 119 114 161 129 83
202587_s_at 953 853 808 973 498 650 1075 812 916 633 1089
803 1672 1087 1176 745 918 946 873 662 404
202588_at 206 367 224 362 107 243 302 208 264 291 175
300 95 48 116 122 141 137 23 124 82
202589_at 10500 8109 7139 1662 7539 1234 12427 13026 11012 9384
14007 11424 12620 17827 16655 17135 16349 18844 14927 5395 5106
202590_s_at 418 390 335 720 759 579 670 603 318 430 485
364 123 113 229 274 203 244 177 126 195
202591_s_at 3540 3008 6672 3957 5504 5019 4582 4109 6554 5782 5625
4438 12098 9667 8628 5921 5957 6000 9270 9434 6045
202592_at 1041 680 1299 2312 1258 1365 1366 1215 999 952 1529
1665 1351 968 1928 977 828 632 1247 1184 610
202593_s_at 1335 1957 3319 3028 1304 1046 1802 1931 3369 4141 2109
2095 1000 775 762 758 711 706 1630 1017 1141
202594_at 1117 1286 1304 1495 1016 1070 1193 1178 1259 1206 1308
904 1028 949 1125 1773 1394 1284 1295 976 1322
202595_s_at 930 653 882 758 888 563 1395 1217 1373 1142 1357
804 1011 1162 928 1156 1223 919 1215 703 534
202596_at 4548 1528 5749 4311 3302 2494 4374 4060 4622 4267 4112
2716 3940 3208 1858 1690 1512 1711 2690 1855 1400
202597_at 595 1113 1120 1905 443 637 753 905 805 1297 590
672 145 57 183 539 479 511 660 691 699
202598_at 1827 1390 1986 2623 2213 1670 2115 2275 1766 2173 2953
5446 2614 2557 1938 1367 1806 1156 1417 1457 608
202599_s_at 803 1311 1696 3211 388 497 453 1053 1496 1391 367
234 514 218 153 814 633 928 2336 2089 2441
202600_s_at 185 356 359 1063 133 153 284 379 470 369 114
206 126 61 7 74 61 63 251 195 278
202601_s_at 357 309 420 557 726 585 360 356 732 667 352
334 136 152 56 77 105 87 118 52 62

202602_s_at	1969	1347	2178	2809	1709	1556	1880	1553	3084	3268	1794	1929	3750	2000	1290	1813	1670	1666	5704	2997	3746
202603_at	2656	2907	2881	4410	2702	4103	2992	3222	3002	3325	1926	2043	2126	2261	2649	6191	5734	6137	7389	5987	8115
202604_x_at	438	336	513	738	323	676	656	736	627	818	456	514	144	123	96	389	503	397	458	397	582
202605_at	1176	1493	921	1261	1946	1895	1129	1163	1244	1909	2020	2679	1843	2359	1760	782	1125	1290	1727	1714	2001
202606_s_at	921	1232	1511	1326	1113	892	908	1181	1308	1188	1205	765	2019	1722	2561	1989	1376	1886	3889	2425	3880
202607_at	180	212	252	323	639	531	360	170	198	171	149	116	73	111	97	98	118	76	60	84	70
202608_s_at	19	13	41	21	40	55	82	32	56	22	77	26	11	15	13	20	24	12	39	45	5
202609_at	2121	3965	3519	3158	832	845	1652	2092	3277	5472	549	855	1064	767	1088	4487	4390	4625	9414	8187	11796
202610_s_at	889	345	632	348	1295	1596	598	614	508	544	1134	1237	727	1016	1142	645	683	543	676	645	957
202611_s_at	262	147	281	177	650	708	201	301	337	355	463	724	561	348	470	290	183	330	645	398	794
202612_s_at	81	67	16	65	62	105	52	70	45	20	33	98	1	19	2	5	6	24	4	26	4
202613_at	1872	804	2882	406	1884	591	1573	1326	2691	2601	1812	1083	1392	843	1053	876	848	823	2145	1174	1095
202614_at	1229	1412	1044	1097	1523	1981	1414	1479	709	1258	1137	1354	1518	1487	1622	2052	1639	2002	1960	1345	2425
202615_at	1111	1808	1354	1530	1917	1692	1228	1430	916	1758	1684	788	1591	1667	2668	980	943	780	1578	1537	1843
202616_s_at	238	420	612	696	219	321	383	426	654	823	553	476	219	181	167	146	149	114	343	360	298
202617_s_at	192	254	345	383	225	305	235	323	360	565	408	302	175	207	224	64	154	92	229	161	56
202618_s_at	302	398	676	636	990	1273	249	164	507	379	483	328	164	180	192	160	174	179	222	276	223
202619_s_at	213	259	1127	1261	274	235	453	435	1351	1542	80	69	20	15	15	280	256	259	711	432	721
202620_s_at	439	646	1517	1785	33	55	490	545	1512	1972	20	8	4	2	6	1355	1781	1193	4304	2605	5114
202621_at	852	1479	2113	1690	845	825	1252	1007	2218	1843	1840	1102	716	479	526	284	295	176	673	462	282
202622_s_at	344	359	861	550	548	465	543	457	405	512	293	241	232	133	338	296	297	369	465	526	397
202623_at	869	540	539	680	742	856	700	1122	437	735	729	1372	1098	1036	1061	1306	1410	1009	854	852	798
202624_s_at	888	710	758	813	276	215	1061	1002	657	787	757	493	92	96	108	144	158	132	111	89	94
202625_at	1086	785	157	159	335	290	692	669	265	267	463	295	381	193	270	596	677	1014	682	441	620
202626_s_at	541	463	346	274	283	321	766	520	340	433	385	396	321	309	308	500	509	403	222	227	223
202627_s_at	411	535	579	79	2272	1441	301	264	250	201	2777	3221	578	399	534	152	219	323	196	134	138
202628_s_at	374	579	631	19	3579	1365	188	37	62	32	3941	7039	1111	629	718	104	60	94	40	31	51

202629_at	849	1024	819	1046	954	1137	1067	1668	557	1005	1086	1372	986	1149	1328	1424	1233	1378	783	882	1094
202630_at	705	892	281	597	198	495	301	710	116	306	236	381	511	467	478	1847	1506	1530	689	989	1271
202631_s_at	325	350	274	299	499	543	537	492	268	243	414	416	242	229	119	333	361	328	193	237	284
202632_at	1050	1130	1751	1499	1091	844	1053	790	1349	999	3513	1348	1406	1346	1077	577	522	554	685	701	398
202633_at	1101	724	2231	1075	2499	1068	1138	1182	1778	2200	1867	1643	1875	1877	2736	1686	1647	1711	3856	2932	4205
202634_at	712	637	547	625	893	724	889	955	524	398	605	495	1187	868	945	1096	1307	1050	1166	663	476
202635_s_at	1423	1545	1000	888	1012	939	2001	1688	1121	1006	1178	1267	3062	1956	1626	2938	2472	2952	1681	1281	1042
202636_at	747	1465	650	2293	1519	1791	602	816	701	1333	1599	1043	1000	1339	2072	1761	1749	1151	1075	1248	1608
202637_s_at	265	170	429	389	375	961	282	392	421	414	2579	1293	294	364	335	94	61	45	60	199	118
202638_s_at	143	108	171	217	469	892	162	141	158	229	1887	1386	228	294	273	76	55	93	73	177	167
202639_s_at	187	174	130	48	529	480	110	239	76	53	284	46	65	99	214	225	265	296	152	61	135
202640_s_at	496	611	160	401	361	419	400	512	372	375	415	383	272	265	188	305	344	289	226	182	190
202641_at	1164	1327	1023	890	1923	2065	1904	1834	1032	1276	1197	1717	1366	1163	1239	1785	1562	1903	1288	1516	841
202642_s_at	1887	1995	1013	799	1891	1927	1514	1355	1014	970	2442	1551	1087	762	750	677	589	763	592	623	605
202643_s_at	206	367	297	265	110	109	281	104	254	284	303	348	26	70	63	55	114	106	124	101	126
202644_s_at	286	506	358	367	477	413	327	265	294	376	428	780	65	99	123	188	171	237	282	229	237
202645_s_at	1421	1199	1195	1385	1010	1114	1338	1536	1236	1296	1160	1056	604	687	787	983	969	822	929	1025	844
202646_s_at	3203	2664	2604	3732	1905	2220	3513	3886	3523	3323	2116	1243	2610	2609	1829	5345	5111	4352	5292	4200	5256
202647_s_at	586	569	945	742	646	677	1052	1097	1003	869	1028	439	701	398	510	1035	1118	769	1370	606	900
202648_at	73	382	141	47	34	60	25	249	213	48	43	31	40	195	447	435	227	328	146	266	168
202649_x_at	30479	34605	25942	31968	25217	33647	24515	23076	19560	19140	17820	31312	24879	28985	23252	23721	27444	25660	16957	25262	16038
202650_s_at	380	932	339	604	422	598	583	528	344	313	355	331	156	201	95	110	146	182	135	101	76
202651_at	971	686	827	391	911	1079	876	867	495	929	669	657	782	662	740	1384	1233	1016	1226	1328	1573
202652_at	81	30	137	107	63	40	60	113	24	116	173	78	10	31	41	17	61	16	4	6	3
202653_s_at	839	783	627	693	1035	1308	637	1107	535	570	769	670	2237	2135	1913	2845	2249	2895	2938	2139	3743
202654_x_at	673	521	937	855	975	1082	1118	1479	756	769	1033	872	671	735	465	871	880	1016	709	458	668
202655_at	4342	3107	4338	1089	2763	2813	2315	1774	1964	2393	2884	2423	3621	3870	3745	5369	6192	5006	2069	5337	3634

202656_s_at	1024	1510	1472	1235	998	1621	1756	1737	1303	1145	1281	915	643	665	702	1362	1316	1052	1124	1161	1327
202657_s_at	1503	2535	1350	1142	678	860	1782	2020	914	901	964	721	859	686	841	2193	2151	2930	2060	1663	1984
202658_at	816	692	894	1230	792	818	847	818	803	1064	812	770	508	487	810	1409	1045	775	895	947	823
202659_at	614	2137	392	1610	691	986	571	426	511	567	826	871	1065	1124	391	404	420	518	534	749	401
202660_at	94	99	37	151	313	329	86	164	21	68	149	256	168	158	224	75	50	82	341	665	638
202661_at	33	78	15	114	37	24	82	12	16	13	60	194	28	61	56	17	34	3	109	127	145
202662_s_at	112	73	62	189	371	473	111	156	45	96	199	280	132	153	89	21	18	47	98	179	165
202663_at	4	6	11	18	40	19	3	5	1	3	17	16	1	12	2	13	19	4	2	2	1
202664_at	64	35	30	31	55	11	36	36	17	15	27	6	1	3	2	13	2	2	8	26	32
202665_s_at	65	120	176	195	348	452	295	186	199	102	186	97	37	36	26	6	45	5	41	55	9
202666_s_at	962	626	1020	586	2276	1771	460	636	967	978	1259	1605	3365	2369	1875	1209	1001	1088	4763	1438	2257
202667_s_at	556	321	378	416	672	261	401	396	409	351	325	274	426	348	276	439	439	295	250	265	181
202668_at	2856	1663	2334	1470	410	196	1586	1791	1717	1786	337	562	294	286	437	2193	1724	1320	4412	5892	7116
202669_s_at	480	283	532	278	125	42	515	631	480	519	145	142	69	84	54	136	131	111	261	305	205
202670_at	2103	2619	2725	3374	1002	1070	1948	2712	4020	3400	2044	1645	1016	929	1302	2082	2009	1862	2718	1940	2370
202671_s_at	994	1223	2438	1791	1590	2677	1208	951	2835	2138	2581	1286	2836	2486	2346	805	945	853	2009	1210	991
202672_s_at	884	4675	747	228	801	1705	484	519	226	341	597	876	569	927	1001	839	864	1849	327	1466	1151
202673_at	2543	2204	3485	2764	7928	7237	2841	2729	3650	3950	4136	4356	10036	5889	5308	3393	3390	3281	8894	6240	7286
202674_s_at	33	38	90	92	95	194	50	38	95	131	82	46	46	28	18	6	10	11	88	46	40
202675_at	1978	1500	1427	1102	1105	917	2042	1463	1312	996	1465	1088	1971	1796	1622	2224	2277	2199	1907	1705	1476
202676_x_at	540	1180	906	925	128	216	572	472	919	695	371	266	437	250	210	166	192	122	285	259	177
202677_at	1441	1980	3342	2662	1667	1562	966	1134	1795	2193	843	769	862	677	1401	2055	2304	1682	3181	4242	5559
202678_at	3381	1730	4177	3126	2481	2087	2585	2346	5655	4479	2781	1947	3272	2911	2605	2754	2905	2644	4605	2407	1942
202679_at	1213	1390	1473	515	1520	1230	624	925	578	1047	2078	1899	1493	2043	2612	1150	1260	1229	697	1113	1465
202680_at	1630	2018	1327	1182	1155	1416	1480	1624	1358	1143	1164	1027	946	962	1017	1545	1370	1392	1276	1072	804
202681_at	822	512	515	758	1434	1826	686	764	902	665	1292	1057	755	572	633	667	509	924	653	335	357
202682_s_at	676	451	711	632	1309	1951	757	598	654	725	939	1262	1749	1558	1894	805	689	629	1159	1258	1402

202683_s_at	652	533	1087	736	929	926	693	885	879	1029	806	823	762	713	526	735	934	814	1222	928	917
202684_s_at	177	79	224	105	173	130	160	194	284	284	173	150	145	147	114	129	109	99	259	122	248
202685_s_at	233	286	460	131	58	87	212	367	193	102	372	501	95	178	60	58	232	137	45	95	99
202686_s_at	561	1077	1701	515	939	1693	704	975	618	552	2800	3760	4698	8484	8435	5418	7020	5053	2507	4045	4967
202687_s_at	52	54	35	295	223	298	42	40	23	63	124	78	105	113	23	9	25	2	95	147	295
202688_at	50	60	91	1145	302	296	53	53	60	99	281	103	145	163	136	11	2	26	184	383	579
202689_at	1504	892	973	976	1260	1199	996	871	1414	1522	881	1097	947	892	823	885	895	681	838	542	463
202690_s_at	3412	2368	3052	966	5568	3306	3517	4058	3428	2790	4889	4567	8797	6053	4873	4494	4650	4425	7095	3531	2424
202691_at	816	686	643	309	975	395	1383	1219	990	716	1072	803	1438	626	837	1219	1078	1240	1172	546	908
202692_s_at	729	712	549	630	566	285	1037	893	804	562	598	483	260	192	122	103	126	158	171	102	83
202693_s_at	892	897	792	378	1945	1598	612	720	499	548	1454	2583	2984	2642	3433	1455	1355	1024	1002	924	1280
202694_at	48	73	43	101	142	31	73	13	56	48	54	82	59	35	109	58	50	40	50	20	39
202695_s_at	385	311	392	325	700	814	347	475	268	327	502	583	306	286	570	273	220	246	117	117	124
202696_at	722	678	1396	1229	1827	1834	1194	1114	1662	1525	1764	1347	1527	1324	1256	942	1027	911	1278	856	807
202697_at	2053	1545	2142	1492	1471	1461	1942	1903	2597	2141	1993	1548	1781	1789	1511	1345	1241	1194	2002	1612	1312
202698_x_at	14678	12248	10317	11999	6478	7070	12552	10248	8347	8437	6511	6670	9375	9154	7786	12397	14488	9744	8003	7668	3788
202699_s_at	189	124	199	217	542	433	159	111	329	112	219	82	80	103	35	31	56	59	122	85	132
202700_s_at	111	159	110	266	139	177	93	146	197	161	117	75	71	113	97	52	32	118	108	69	59
202701_at	443	729	186	330	569	450	411	392	183	198	225	221	99	124	95	214	313	202	103	77	35
202702_at	725	785	765	700	763	507	742	913	747	731	971	569	235	352	440	575	431	564	416	422	573
202703_at	1296	813	924	1283	1373	1786	959	1221	1080	1269	1506	1296	1638	1882	1421	1268	1265	1382	1114	964	1327
202704_at	3313	4329	1697	2993	1210	1692	2019	2056	1741	2440	1286	1585	1945	2168	2436	5683	4793	5208	6773	7751	9809
202705_at	2475	703	3821	802	4277	390	2818	3407	3800	3312	4157	5038	5572	6539	5743	3137	3412	3660	5180	2589	2379
202706_s_at	1705	785	1668	838	1704	585	1301	1376	2740	2113	2059	1217	1679	1600	1361	1387	1297	1572	2177	646	823
202707_at	265	202	99	203	131	192	144	342	321	280	308	268	56	48	49	44	64	26	76	72	60
202708_s_at	276	296	298	892	561	1293	224	241	194	312	860	2074	748	728	854	87	71	84	128	585	712
202709_at	238	182	236	303	432	312	330	257	237	361	273	255	60	88	81	91	86	130	99	175	145

202710_at	1080	751	1190	1017	1234	2181	657	914	546	787	1298	1726	1605	1670	2713	1539	1497	1690	1088	1097	1593
202711_at	130	39	372	497	874	594	148	45	269	593	739	364	142	213	145	33	72	44	74	82	52
202712_s_at	550	608	404	795	441	570	735	487	740	579	361	378	280	268	333	614	495	598	584	349	333
202713_s_at	912	725	924	982	1222	981	1218	1008	1070	692	1164	885	806	1039	1481	1270	1228	1161	1035	809	715
202714_s_at	172	83	81	203	195	50	179	187	166	138	166	52	111	130	91	73	59	42	114	56	47
202715_at	1631	1651	1196	645	1539	1106	1586	1516	1409	1389	1839	1465	953	597	650	547	586	664	1115	865	725
202716_at	701	678	663	623	928	849	742	852	634	683	921	831	1181	916	581	527	809	719	372	386	341
202717_s_at	1177	1613	1156	1107	1333	2098	1320	1388	1038	1196	1596	1754	1430	1744	1717	1528	1193	1125	1532	1886	2401
202718_at	2999	4338	396	344	970	598	2097	2458	274	411	2105	1223	1471	1303	503	880	779	689	292	48	43
202719_s_at	607	661	1126	522	510	962	1383	1082	574	697	1097	1073	779	917	1203	1406	1382	1320	596	964	1336
202720_at	1900	2657	3243	2817	2590	3338	2472	2301	1663	2614	2405	2883	4475	4300	4802	5638	5782	6180	4117	5909	6985
202721_s_at	810	592	2181	1509	601	1043	1012	768	1973	1926	1272	899	530	544	520	875	899	965	899	2471	2704
202722_s_at	1506	1199	3521	1884	1215	1793	1479	1185	2410	2882	1309	1209	935	1126	1323	2085	2088	2395	2715	6062	8568
202723_s_at	441	363	187	132	58	58	297	402	231	247	142	324	61	100	137	199	137	101	402	288	235
202724_s_at	169	290	326	219	326	34	233	305	464	376	206	415	100	127	44	120	162	124	433	237	296
202725_at	1975	1574	1222	1130	1487	770	1923	2188	1110	808	2012	1181	457	406	702	520	483	859	425	334	399
202726_at	763	550	728	396	355	40	873	1124	1605	1261	759	548	435	314	158	163	114	250	531	131	115
202727_s_at	1224	1333	4115	1671	1068	1963	680	682	2106	3008	603	533	872	942	1047	1981	1894	1816	6464	6444	8530
202728_s_at	28	32	146	166	71	59	65	67	139	105	40	69	5	9	3	25	30	17	38	70	16
202729_s_at	333	189	392	573	385	407	286	207	264	390	195	72	29	85	69	167	170	163	359	270	438
202730_s_at	164	181	302	497	203	249	182	265	118	303	117	217	140	120	129	242	304	262	293	413	322
202731_at	520	476	534	1636	337	298	466	705	380	546	297	533	200	164	127	270	252	105	285	407	328
202732_at	732	991	206	182	1884	1365	909	930	74	148	2594	1523	2566	2552	1854	443	666	540	102	132	122
202733_at	954	893	875	1067	2657	5154	1011	888	633	1101	3995	3374	2498	2747	2732	945	1358	978	781	1633	1259
202734_at	1102	1232	732	704	929	797	1292	1111	739	889	938	571	145	218	345	536	624	379	397	239	181
202735_at	1156	462	709	332	2613	2181	543	629	709	474	632	951	1879	2036	2003	860	585	577	1146	1422	754
202736_s_at	3589	2698	1243	688	1381	642	4924	4322	2633	2327	2601	1605	4070	1658	1014	2480	2500	2166	2366	1368	1255

202737_s_at	6918	4907	2945	913	1934	590	6414	5635	4054	3133	3162	2198	3441	2390	2054	3437	3628	2818	2684	1814	1046
202738_s_at	581	536	880	1463	818	739	758	822	690	648	563	857	399	357	370	652	606	627	616	779	918
202739_s_at	552	428	589	716	510	847	468	521	345	281	385	391	274	424	298	505	465	433	604	498	876
202740_at	754	366	1538	953	1060	801	521	699	1197	1702	951	994	1289	1633	1083	872	869	719	1685	1175	1127
202741_at	3256	1973	1276	1476	1105	1145	3587	3072	938	820	993	1198	1017	1167	1806	10470	10136	9946	2507	1542	2539
202742_s_at	931	461	518	385	276	202	1517	1495	459	236	412	377	175	128	125	1598	1405	1425	360	148	421
202743_at	93	243	380	1800	184	325	136	170	345	307	246	236	121	200	136	89	128	117	450	437	498
202744_at	437	738	471	167	397	547	384	457	415	366	388	261	143	158	141	191	188	159	203	184	153
202745_at	562	544	939	700	561	779	698	837	664	917	649	523	769	695	508	1247	1189	1214	1721	1458	1921
202746_at	40	6	71	6	62	38	57	17	24	41	38	38	2	1	11	4	1	3	21	5	9
202747_s_at	36	24	33	40	44	54	42	25	41	45	31	34	7	27	7	13	7	24	14	4	15
202748_at	87	116	50	550	60	7	42	78	120	182	85	152	22	54	30	49	52	22	154	159	155
202749_at	1672	1490	1306	1481	1068	2232	1517	2371	661	1753	1643	2126	2093	1880	1901	1589	1582	1257	1607	1111	1651
202750_s_at	824	518	368	460	704	411	574	627	466	389	493	542	383	241	155	266	310	252	299	262	395
202751_at	124	80	159	138	60	67	187	165	155	179	142	158	45	53	78	90	113	87	97	76	98
202752_x_at	560	303	62	263	1772	3057	269	447	186	95	2119	1120	1169	1144	1051	78	105	81	154	152	142
202753_at	4390	3590	2614	2146	5352	2899	5505	3729	3773	2959	6812	5682	8800	8956	7705	4968	5116	4913	4219	2910	2975
202754_at	1737	970	1990	1168	1845	1936	1348	1696	1881	1659	1629	1337	1509	1388	1770	2109	1744	1961	3161	2199	3055
202755_s_at	184	624	110	74	826	744	280	232	205	108	441	38	71	77	26	128	148	93	88	56	33
202756_s_at	811	1741	625	573	1792	1473	1101	947	756	646	1079	866	869	750	648	546	686	390	443	197	198
202757_at	1767	1910	1740	1436	2159	1310	1688	1441	2015	2046	1798	1130	1143	1002	717	646	645	674	936	594	539
202758_s_at	962	1012	787	881	1010	1142	725	698	642	507	1016	725	940	599	553	404	360	316	451	513	321
202759_s_at	962	1270	1571	431	1724	1337	2263	2011	1308	1361	1937	2361	508	443	703	674	690	522	289	170	209
202760_s_at	295	330	266	127	437	370	551	554	273	314	466	789	266	157	124	151	187	114	90	41	61
202761_s_at	623	405	665	872	711	419	326	520	352	857	529	435	247	284	192	195	159	248	411	622	521
202762_at	1516	1120	2912	2603	4181	3291	1636	1826	2681	3560	2967	2217	2667	3203	3022	1505	1380	1206	3130	3899	4399
202763_at	488	338	332	260	892	500	334	426	211	300	642	548	1408	642	468	259	312	173	374	449	509

202764_at	238	204	347	468	268	636	189	284	371	274	435	276	142	150	132	90	137	97	97	89	92
202765_s_at	24	18	18	14	43	28	20	16	20	18	17	23	3	2	7	5	2	12	5	2	3
202766_s_at	74	41	8	14	19	11	40	46	95	38	11	39	5	15	2	18	5	2	10	29	7
202767_at	646	731	898	1172	614	818	859	809	619	1006	681	802	496	577	374	260	411	383	383	347	551
202768_at	32	13	53	36	29	31	52	33	55	61	44	19	96	80	90	341	314	393	238	545	628
202769_at	483	625	475	980	372	570	335	833	111	411	348	1154	439	205	376	796	1060	1040	611	919	1930
202770_s_at	438	426	355	625	184	500	215	625	111	222	180	527	148	189	199	603	646	591	279	382	910
202771_at	1226	2626	2242	1915	671	637	1153	1263	2095	2492	1389	1365	783	611	624	842	862	963	1675	872	915
202772_at	1176	821	731	1697	1226	1669	920	1110	545	788	826	971	718	659	419	344	561	324	363	729	513
202773_s_at	343	258	250	135	22	35	264	408	314	197	155	168	125	96	49	153	141	116	116	64	62
202774_s_at	946	1090	978	823	419	796	876	967	779	635	701	676	477	720	602	781	855	715	645	760	722
202775_s_at	656	826	555	591	432	380	734	666	518	499	557	246	165	250	432	627	665	599	512	275	314
202776_at	1491	1105	1537	1348	2355	1635	2335	1533	2379	1458	1647	1168	2316	2183	2833	3440	3237	2620	4224	2468	3034
202777_at	788	988	1232	1275	1450	2110	1455	1382	1286	1458	829	921	986	969	860	2042	1708	1958	1967	1459	2312
202778_s_at	283	397	800	1230	1601	2095	388	557	740	943	1329	1147	1697	1455	2050	561	551	691	1260	1555	2292
202779_s_at	8927	4385	13430	1604	6000	1256	10950	7652	12349	9831	11216	5030	10333	8676	5225	4831	4788	3353	4971	3048	1474
202780_at	1082	838	26	28	811	472	950	1130	52	105	938	842	1168	1048	838	1030	1102	913	81	509	497
202781_s_at	487	417	765	700	631	632	480	375	694	1198	961	1034	529	423	245	233	282	361	183	295	466
202782_s_at	476	418	563	671	399	762	564	445	571	801	733	552	530	283	221	184	158	182	250	206	318
202783_at	34	29	399	557	595	731	45	4	467	193	523	660	569	581	515	3	5	18	540	607	802
202784_s_at	24	38	572	749	623	550	8	12	447	275	480	451	252	279	199	2	2	2	248	306	539
202785_at	802	734	712	651	1080	943	779	838	460	633	773	1168	1893	1347	1092	1113	1918	967	1188	1358	553
202786_at	2699	4649	3859	3414	851	828	1677	1758	2006	3500	938	549	1113	969	1245	7478	6091	6981	5912	4580	5628
202787_s_at	558	463	423	269	1138	669	757	501	541	416	1232	403	632	802	725	386	427	411	227	197	151
202788_at	1311	1298	1643	1051	1383	1183	2451	1869	1520	1222	2561	1419	2476	2337	3181	1007	1020	1173	707	485	553
202789_at	405	328	254	352	950	1341	330	228	391	247	587	561	694	642	401	202	174	119	174	219	163
202790_at	2385	3557	3435	4016	1922	2110	2454	2191	4976	4661	2653	1224	1691	573	1061	1045	1095	1053	1179	1518	1073

202791_s_at	439	707	339	327	907	667	529	494	332	323	377	348	151	140	207	239	182	190	125	159	142
202792_s_at	196	221	122	239	36	164	64	154	195	162	149	238	62	53	83	53	98	76	86	75	60
202793_at	329	242	495	438	381	310	372	492	479	621	298	314	342	299	194	277	249	302	716	350	593
202794_at	1212	1096	4289	1211	1469	1216	1204	864	2779	4829	1937	1061	1271	1285	1237	811	673	1029	2721	2209	1836
202795_x_at	3077	2642	878	1446	1373	1027	2165	2951	678	1234	667	1445	644	420	705	1954	2060	2493	689	1098	881
202796_at	205	142	92	281	256	341	131	208	9	117	55	31	51	25	30	101	90	83	38	39	37
202797_at	811	894	1016	1005	1478	1889	600	830	768	551	1040	1119	1800	1830	2626	2075	1898	1990	3442	3323	4967
202798_at	1512	1176	770	954	1071	1299	1052	1339	803	788	927	883	807	1054	1482	2779	2505	2443	1643	1697	2461
202799_at	1585	1227	874	728	1439	1484	1521	888	749	528	1036	811	1308	1302	1001	890	862	834	735	832	408
2028_s_at	393	240	502	290	628	149	596	383	678	820	1006	615	186	135	165	70	69	71	174	52	108
202800_at	185	440	62	88	291	418	264	96	9	108	162	30	192	198	184	836	650	523	11	9	12
202801_at	651	574	558	1286	539	212	746	790	757	907	871	470	232	290	197	289	327	216	227	182	153
202802_at	798	644	297	274	84	386	625	479	219	239	213	253	447	462	342	668	689	593	409	547	531
202803_s_at	119	113	56	159	14	75	127	110	124	136	151	117	4	5	3	4	2	6	3	3	6
202804_at	2491	2427	2847	1460	1347	1748	2205	2594	2202	3346	1579	1524	1260	1269	1559	2830	2675	2045	2487	2163	2457
202805_s_at	1147	764	1700	830	400	478	1496	1302	1239	1820	1080	532	183	175	297	217	289	308	336	315	368
202806_at	1918	2167	1000	820	1339	769	1587	1490	519	382	1218	783	1028	1265	1787	1963	1726	1820	731	1014	779
202807_s_at	217	316	137	350	54	46	220	285	211	250	205	216	77	61	65	73	60	93	31	12	41
202808_at	1439	1620	1126	2398	1278	3423	1273	1478	1148	1252	2155	1477	1001	776	634	694	789	562	416	456	446
202809_s_at	1058	762	918	1099	715	512	871	479	668	583	560	524	282	323	476	776	762	910	744	932	1177
202810_at	2466	1979	2017	1968	2897	2086	3995	3244	2138	1994	3454	2526	3394	3506	4515	4506	4003	4337	2425	1755	1708
202811_at	1819	1224	1333	1253	1714	2717	1362	1152	1081	1164	1451	1333	1627	1532	2548	1969	1957	1730	2091	1821	2518
202812_at	1210	2963	236	1233	195	781	958	1341	248	363	530	454	235	223	119	198	290	250	97	174	100
202813_at	303	478	263	274	575	577	252	381	393	372	198	523	243	233	415	93	100	109	380	511	638
202814_s_at	1749	2152	636	811	2210	1388	815	1357	856	848	1238	1736	1420	1008	779	1131	731	1014	832	764	766
202815_s_at	372	506	149	323	359	403	395	459	211	228	581	775	545	313	199	387	355	321	184	240	146
202816_s_at	912	304	427	286	454	620	803	1041	450	671	419	344	449	296	213	481	295	516	386	272	391

202817_s_at	896	257	434	303	269	216	462	874	352	604	310	249	406	389	182	722	580	562	722	421	383
202818_s_at	601	274	659	296	66	56	942	537	827	882	821	701	539	526	436	374	438	315	418	346	167
202819_s_at	714	277	677	419	465	593	657	490	951	783	793	456	451	396	283	241	273	293	418	232	302
202820_at	534	579	326	541	411	567	335	414	448	391	168	269	503	222	183	259	208	248	715	804	860
202821_s_at	276	145	198	164	121	195	431	240	190	238	171	191	20	54	31	69	29	26	20	51	40
202822_at	877	3168	907	1027	1112	1450	1094	1317	541	669	1111	845	384	283	720	606	663	578	475	1226	1056
202823_at	673	563	202	149	304	371	290	524	177	210	289	288	776	460	500	1153	924	971	946	582	468
202824_s_at	4297	3326	2398	1558	3018	2189	3751	3422	2299	1976	2761	2576	6356	6307	6805	6474	6965	7484	4907	4064	3253
202825_at	826	715	625	473	1471	1576	755	736	443	632	1008	1140	1831	1446	1532	552	557	638	538	428	404
202826_at	1134	1406	1508	2686	1058	2100	1043	1215	1290	1384	1433	734	415	422	596	935	1044	640	963	1409	1269
202827_s_at	19	220	28	159	392	415	172	264	31	120	462	304	27	19	12	10	10	13	24	4	9
202828_s_at	44	46	14	14	95	261	37	17	9	13	57	36	74	70	28	7	9	6	6	4	4
202829_s_at	1283	1460	3398	3330	1813	2403	1442	1291	3515	3579	2048	1370	2798	3149	4509	4004	3452	3935	9086	6602	8665
202830_s_at	751	208	250	237	620	286	668	753	269	446	712	715	698	569	531	594	462	575	583	397	510
202831_at	120	172	10622	22142	496	501	237	169	10477	17052	438	139	163	154	258	24	26	2	11496	14087	8805
202832_at	446	608	1885	2457	1008	1386	356	623	1640	2542	726	664	466	555	840	590	540	621	2754	3298	4701
202833_s_at	86	17	1721	2916	1093	2068	146	124	518	1386	4129	2589	1746	1857	994	66	60	47	759	837	840
202834_at	303	174	321	630	335	324	138	239	244	355	132	260	45	46	50	79	45	51	100	209	227
202835_at	288	257	103	133	51	34	273	335	144	247	47	38	62	20	98	177	277	214	114	109	81
202836_s_at	6776	5551	2751	1875	1654	976	5532	3856	2901	2443	2363	1528	3206	2946	3556	6769	7622	6273	3951	4481	1606
202837_at	62	24	132	191	148	212	58	53	114	43	253	65	53	38	82	107	87	39	41	36	11
202838_at	1343	2386	1023	3313	3373	1676	1832	2549	945	1744	4133	4323	4443	6578	6813	2652	3321	2314	1915	1095	1645
202839_s_at	687	646	370	573	432	404	688	454	285	349	590	549	1922	1651	337	547	509	570	589	455	233
202840_at	984	619	1044	773	2412	1166	553	790	792	963	723	566	813	548	520	533	500	491	744	456	234
202841_x_at	1234	1197	1069	1249	410	367	1295	1207	1013	1333	999	730	274	333	396	415	383	336	418	447	292
202842_s_at	1736	1747	1965	1138	704	3350	800	779	487	774	1033	1206	977	1523	1411	1808	1976	4681	810	4229	4773
202843_at	295	184	281	88	168	806	105	87	50	89	91	72	251	197	216	220	238	969	171	811	950

202844_s_at	699	862	313	709	359	431	372	439	514	625	399	533	1186	737	442	464	408	328	943	1028	375
202845_s_at	2224	1949	2265	2870	1732	1677	1324	1828	2474	2572	2072	1660	1249	1279	2351	1798	2175	1334	2375	1338	1375
202846_s_at	410	304	701	1048	1009	865	509	591	800	1051	645	700	1114	1083	934	618	717	565	2040	1865	1718
202847_at	1152	1142	1224	639	783	1571	1112	516	527	608	597	753	511	577	932	987	1185	1216	442	1607	1381
202848_s_at	528	337	296	506	111	175	515	571	586	635	514	363	430	636	708	219	217	174	301	121	151
202849_x_at	187	482	186	191	209	180	252	363	214	472	304	783	400	411	472	200	151	166	413	52	42
202850_at	1794	1248	3101	3673	2152	2281	2419	2095	3136	3811	1464	1605	2353	2123	3249	2633	2170	2345	6852	3260	5501
202851_at	38	43	45	66	30	31	61	58	194	113	47	45	156	144	40	93	72	130	128	151	134
202852_s_at	742	921	863	763	1005	718	1157	743	937	1103	975	675	1304	1216	940	1227	1524	1543	1637	1085	1732
202853_s_at	1509	1490	1888	1822	2265	3115	1231	1298	1667	1835	1572	2108	2007	1895	2575	3432	2880	2822	4976	3109	4743
202854_at	3340	2861	4227	1843	5311	4127	2959	2692	5206	4400	5280	5324	7863	7925	9872	4638	3945	4514	7394	4175	5116
202855_s_at	3389	1914	389	341	774	538	3638	2637	559	481	2633	852	621	410	112	141	155	238	13	70	65
202856_s_at	4658	2479	453	379	1465	1050	3267	2489	460	400	2628	912	1554	1420	495	745	797	739	133	224	181
202857_at	4056	4636	2912	4382	3581	3767	3400	2926	2914	3353	3307	4473	5726	5931	4971	3410	3997	2617	3939	5424	2656
202858_at	8684	9006	4958	2265	6396	4264	7032	7234	6620	5059	7561	8507	9277	8985	5271	3745	4330	3328	2835	2748	2358
202859_x_at	157	501	482	109	111	122	144	137	217	119	387	366	36	28	32	291	410	655	425	669	845
202860_at	652	1028	802	773	231	468	684	603	757	569	559	82	252	114	92	176	290	133	324	365	403
202861_at	524	550	39	44	63	67	329	511	116	31	26	40	123	91	30	163	164	204	108	139	90
202862_at	355	235	696	445	531	465	313	304	649	612	551	310	424	438	273	192	232	168	994	378	435
202863_at	431	729	532	697	509	575	273	377	432	443	200	320	299	339	509	696	926	1861	1240	1431	1732
202864_s_at	368	578	404	413	539	771	372	503	318	293	262	153	302	436	223	453	632	1397	501	797	1157
202865_at	140	119	175	168	63	73	151	174	158	187	135	128	95	65	45	56	43	69	93	39	68
202866_at	589	472	848	1121	568	458	653	512	966	974	696	555	558	380	380	337	298	367	571	633	672
202867_s_at	476	290	696	892	594	900	550	567	693	956	472	456	385	305	240	265	336	221	525	323	301
202868_s_at	1766	1629	1423	1526	1895	1672	1667	1548	1627	1512	2005	1445	3936	2970	2453	2034	1961	1730	2696	1861	1679
202869_at	105	224	267	1543	368	509	126	86	419	418	207	231	72	138	244	54	166	1273	668	2419	2772
202870_s_at	4069	573	2490	297	2455	48	3824	4199	2684	2144	3876	2447	5181	4178	3439	2604	2852	3198	2652	1113	984

202871_at	426	813	719	716	1068	500	392	710	779	903	264	256	314	320	350	295	381	345	313	195	197
202872_at	542	414	499	481	499	716	600	800	322	372	486	320	709	479	615	998	836	1041	879	586	848
202873_at	372	122	210	239	236	47	554	662	389	377	397	353	54	55	55	79	70	71	61	42	71
202874_s_at	827	460	742	768	1075	1717	2005	2010	755	822	1138	889	1312	1309	812	1204	897	1761	848	809	1098
202875_s_at	512	451	115	178	205	116	323	240	79	84	217	108	195	177	232	258	336	338	110	141	88
202876_s_at	1552	963	293	751	1157	1007	509	503	245	306	466	336	377	164	171	259	251	123	140	185	98
202877_s_at	160	106	140	106	209	363	162	190	136	182	138	161	40	4	11	12	12	6	37	21	31
202878_s_at	134	151	165	219	246	235	148	199	144	222	92	241	4	19	62	36	26	48	13	2	29
202879_s_at	50	134	258	225	470	574	164	166	183	277	243	172	7	6	7	11	5	11	10	17	7
202880_s_at	341	504	319	358	458	535	246	321	281	254	395	367	269	243	180	128	177	177	179	166	102
202881_x_at	9	7	28	54	476	431	27	63	27	88	16	14	42	63	53	69	15	52	33	46	20
202882_x_at	4595	4475	3272	1632	4599	4001	3816	3262	3112	3031	3182	3778	6099	4244	4871	4421	4102	3870	4654	4903	4463
202883_s_at	679	528	890	679	1210	718	868	772	1275	952	1485	1195	1170	1148	767	610	779	409	928	699	565
202884_s_at	460	235	693	626	1218	603	837	689	1066	1085	1636	694	406	314	278	249	207	214	389	200	244
202885_s_at	135	92	66	322	111	59	70	92	186	211	77	30	25	8	5	6	15	6	25	31	5
202886_s_at	458	221	636	467	805	445	666	546	1070	787	1559	931	629	711	712	427	394	408	751	372	439
202887_s_at	4624	8942	3175	2032	1057	2395	3780	1159	712	333	883	494	689	323	479	1693	1608	2142	292	3569	1763
202888_s_at	48	12	94	47	30	32	41	15	48	38	31	14	2	13	28	2	23	24	8	5	13
202889_x_at	622	591	986	803	110	93	668	751	1163	1206	431	317	147	54	38	13	27	49	233	195	359
202890_at	930	1078	1506	1227	445	403	987	1082	1394	1673	460	650	594	521	582	1172	1100	1024	3947	3609	5408
202891_at	1433	1261	935	1456	763	1021	1114	1400	1070	792	620	975	593	532	490	642	805	817	612	751	618
202892_at	810	614	393	401	1183	941	836	874	421	415	1083	912	1339	1699	1363	1398	1203	1138	833	556	828
202893_at	819	563	878	1132	814	973	629	662	771	697	624	602	405	322	281	351	388	300	393	397	503
202894_at	1020	1024	1662	1640	727	691	819	913	1337	1950	772	628	449	437	395	368	572	493	644	844	522
202895_s_at	201	276	214	279	225	148	273	340	217	204	372	236	13	69	54	4	7	43	13	4	2
202896_s_at	532	654	129	296	375	325	542	534	187	274	733	604	158	232	157	175	180	122	65	37	73
202897_at	380	442	22	27	51	304	256	256	58	28	495	215	181	306	223	211	270	176	7	9	35

202898_at	681	695	60	39	264	78	765	805	154	81	657	395	324	292	214	325	392	244	14	54	65
202899_s_at	2487	1384	5396	2033	6595	2362	6259	5376	7645	5688	6505	5830	9695	9664	5606	4912	4297	6213	4780	2983	4234
202900_s_at	1377	1097	1578	724	1676	1102	1248	1006	1678	1491	1923	1599	3665	3050	3020	2119	2035	2156	4729	3164	4409
202901_x_at	28	17	42	224	65	52	19	33	47	39	65	57	23	14	34	10	19	30	126	149	211
202902_s_at	160	50	233	1059	329	67	52	70	195	196	348	238	62	61	34	2	15	18	250	563	731
202903_at	406	139	209	76	374	95	213	146	207	182	276	259	765	337	364	199	173	303	1058	547	893
202904_s_at	329	137	332	136	360	333	260	329	191	260	318	373	1241	948	898	771	674	853	1473	674	980
202905_x_at	565	418	631	357	364	509	582	900	708	667	516	465	444	455	277	389	415	469	367	318	379
202906_s_at	708	670	770	413	573	720	1023	1008	701	918	554	515	542	488	709	1083	997	1488	1641	1450	2325
202907_s_at	1568	1945	1299	998	1139	1341	2005	1779	1296	1498	971	926	1459	1076	1008	2538	2756	3331	3274	1610	2578
202908_at	1080	1153	962	1023	1288	1160	943	925	943	862	1592	1156	907	1015	1221	392	526	518	470	537	590
202909_at	445	681	583	449	779	894	486	872	375	760	757	725	1147	824	738	526	829	513	353	260	307
202910_s_at	442	327	369	285	821	442	347	384	591	342	890	471	894	1156	872	700	838	806	608	705	777
202911_at	1315	1072	1673	1138	3109	1477	1970	2065	2735	2163	3051	3667	3635	2509	1502	915	931	705	1996	1090	1344
202912_at	1578	2971	403	451	1214	1196	1468	2322	206	295	908	489	1676	1430	1014	2064	2147	2115	280	525	312
202913_at	435	498	681	733	151	323	723	578	601	675	524	385	40	109	123	102	144	141	151	147	130
202914_s_at	249	148	307	304	143	195	292	284	289	382	340	165	71	40	64	47	32	27	80	49	78
202915_s_at	900	820	1134	894	429	231	946	1128	1252	1356	767	656	625	386	270	421	402	427	626	387	399
202916_s_at	523	635	695	595	474	572	443	467	828	872	371	397	485	416	420	611	427	430	791	486	592
202917_s_at	160	43	100	119	85	144	68	136	92	80	88	74	25	38	9	14	3	32	12	4	7
202918_s_at	1782	1247	1443	896	2011	1489	1620	1657	1381	1412	2329	2010	4216	4334	3935	3473	3203	3685	2983	2690	3022
202919_at	1009	693	1179	901	1654	1207	1521	946	1390	1115	1663	1302	1588	1949	2181	1738	1619	1818	1540	993	1537
202920_at	118	66	66	23	366	417	134	73	66	82	205	160	165	250	179	84	113	121	30	24	52
202921_s_at	140	128	151	186	392	489	286	166	165	203	216	170	18	58	46	40	12	7	8	11	23
202922_at	1156	1502	1607	1499	742	1328	1236	1219	1078	1500	725	769	569	541	577	1352	1349	1341	1190	1129	1576
202923_s_at	1846	2518	2896	2218	1047	1473	2292	2118	2792	2618	1123	1273	1159	915	1010	2340	2381	1964	2375	2645	3337
202924_s_at	239	184	365	305	386	387	368	344	460	421	814	549	142	146	164	114	113	132	162	122	176

202925_s_at	605	403	616	759	1089	555	600	549	486	572	1035
	718	680	668	608	371	257	510	529	358	529	
202926_at	802	713	477	748	689	1071	735	833	476	552	770
	680	385	490	365	442	547	397	290	387	380	
202927_at	626	350	182	235	91	85	395	301	234	112	274
	156	853	354	312	170	237	159	418	236	127	
202928_s_at	332	137	126	124	192	55	294	212	28	196	186
	40	81	97	74	45	104	20	46	26	29	
202929_s_at	1599	1383	742	793	529	531	1793	1715	1021	851	739
	848	1641	974	639	980	1044	792	629	584	249	
202930_s_at	1614	920	1488	1383	1669	1149	1069	920	1144	988	1791
	978	2970	2856	3594	2238	1792	2018	3697	2696	4054	
202931_x_at	873	915	983	969	709	789	758	688	1353	970	995
	479	335	360	299	273	485	365	453	506	474	
202932_at	3007	1624	3953	2620	2120	1803	2254	2267	5449	4203	3280
	1693	2509	2162	2370	2578	2415	2422	5622	3314	4875	
202933_s_at	3430	1607	3024	1848	4008	4276	1491	1586	2578	2580	1757
	1846	4079	3281	3429	3534	3418	3394	8026	5352	6932	
202934_at	975	1136	1557	335	998	1633	763	325	1500	1054	597
	320	647	480	732	1275	1483	1547	3377	1775	1651	
202935_s_at	2831	2287	1666	2456	2899	1802	2296	2733	1529	2492	4021
	5335	2059	1870	1057	958	973	763	804	909	1048	
202936_s_at	4644	2642	2296	4190	6816	5549	3566	3988	2930	4081	6964
	11096	6189	4094	3590	2195	2030	1799	2048	2857	3135	
202937_x_at	550	636	703	268	110	312	632	333	803	586	1022
	493	952	743	437	182	227	161	456	207	161	
202938_x_at	466	311	378	304	247	673	492	507	437	567	456
	312	121	63	164	85	69	93	127	90	84	
202939_at	2727	2396	3535	3493	2539	1715	5554	2994	5966	3905	3250
	2071	3055	2539	3960	5722	4900	5469	5587	4208	5764	
202940_at	108	79	186	225	173	222	182	274	235	243	250
	239	57	65	110	15	8	6	51	81	56	
202941_at	4863	4275	4573	5485	3619	3292	4824	4071	5020	4418	4987
	4057	5518	6577	7289	5527	6062	6021	7500	5624	4023	
202942_at	1124	676	1763	2068	1911	1306	1412	1324	2426	2876	2078
	2256	4557	3262	1384	474	487	366	1637	1275	646	
202943_s_at	463	532	495	532	601	606	706	822	583	813	848
	716	223	210	328	368	381	347	325	182	203	
202944_at	180	163	167	295	389	296	331	416	233	328	370
	432	147	104	119	194	238	240	174	82	100	
202945_at	1457	890	1177	1054	935	667	1422	1136	1468	1630	1471
	1124	530	368	271	165	224	175	502	302	352	
202946_s_at	1069	865	661	674	707	657	863	830	631	844	460
	568	601	505	678	1234	1125	1082	1574	1282	1947	
202947_s_at	38	27	190	109	493	223	42	50	51	43	84
	68	8	18	30	36	43	17	31	17	59	
202948_at	392	276	69	336	11	15	319	172	116	35	34
	6	12	2	16	192	193	161	40	26	50	
202949_s_at	3928	4501	4418	929	2096	2498	2650	2078	1573	1940	2486
	3197	1766	2078	3318	3708	4977	3462	1891	2634	2405	
202950_at	1080	865	2291	2923	1353	1422	1497	1074	2395	1523	1094
	724	1692	1476	1664	1374	1204	1516	5069	3514	4979	
202951_at	812	1254	924	2051	1432	2548	1090	1060	761	780	1282
	1288	1189	1196	790	668	676	745	913	1020	962	

202952_s_at	54	27	9	17	132	133	8	13	16	8	16	10	26	4	21	2	2	13	2	4	3
202953_at	45	40	118	119	296	305	30	30	56	105	112	24	31	75	10	22	39	30	39	6	3
202954_at	4399	1573	4103	1039	4345	990	4444	4334	4734	4032	7618	8139	9359	7344	6273	3167	3604	3325	4153	1240	820
202955_s_at	447	583	650	883	506	555	526	666	769	703	559	451	192	275	184	244	192	228	411	382	469
202956_at	495	523	882	860	641	1111	651	589	615	818	539	449	370	427	645	634	717	798	1706	1706	2482
202957_at	139	82	199	172	220	285	89	137	151	194	183	124	8	12	77	48	31	54	41	58	31
202958_at	554	556	646	569	441	269	582	693	491	667	617	705	186	210	341	286	273	259	319	189	264
202959_at	345	242	248	391	393	578	318	362	161	314	304	308	342	407	532	501	527	465	592	668	1166
202960_s_at	628	459	395	789	504	883	521	552	302	405	600	429	992	636	919	1478	1411	1244	1513	1899	2210
202961_s_at	6214	3212	7602	7840	11873	6944	5951	5286	8997	7624	12836	11184	14882	12373	12010	4808	4324	3883	6569	4384	2862
202962_at	511	536	563	1070	382	624	435	482	423	1038	507	561	185	116	150	108	153	84	217	318	256
202963_at	912	917	1004	1549	610	934	795	846	1044	1298	762	520	683	702	790	1441	1299	1318	1682	1564	1598
202964_s_at	639	324	910	1301	887	645	814	804	736	1016	725	514	175	160	187	311	358	287	277	208	240
202965_s_at	19	139	100	72	77	237	30	25	16	15	91	28	48	12	49	78	10	127	3	4	22
202966_at	89	58	31	98	84	51	119	79	24	20	70	43	16	6	10	31	32	63	21	2	28
202967_at	336	272	770	3951	634	787	334	383	916	953	362	401	108	153	249	62	97	109	816	656	666
202968_s_at	380	238	244	213	383	336	362	241	428	640	259	138	138	66	140	253	197	196	588	432	439
202969_at	374	360	414	246	198	120	416	414	442	658	146	202	145	82	172	564	461	388	1089	780	1074
202970_at	152	206	190	321	202	409	184	139	171	415	134	160	66	41	36	139	70	42	217	118	187
202971_s_at	86	109	153	149	26	16	189	185	209	528	71	97	83	81	94	322	300	170	827	457	747
202972_s_at	343	249	358	582	32	349	307	304	256	422	146	137	45	35	66	86	53	114	89	65	86
202973_x_at	611	546	448	835	124	146	521	475	392	602	84	101	35	38	5	912	976	1047	788	1100	2051
202974_at	369	324	62	67	801	711	436	455	158	140	1100	682	638	618	664	248	305	209	60	11	53
202975_s_at	668	439	1134	1031	1093	1325	690	975	1153	946	1380	1612	1625	2136	1231	474	507	427	819	777	707
202976_s_at	1539	1517	2317	1498	2587	3397	1325	1275	1454	1194	1360	1778	3231	3634	3049	2857	2963	2884	3739	3661	4532
202977_s_at	26	22	31	27	71	85	30	24	52	124	36	24	50	8	3	7	48	9	58	31	25
202978_s_at	402	617	1133	608	756	636	498	768	1243	1202	1005	1237	550	517	514	385	299	391	648	560	998

202979_s_at	499	577	1194	641	954	1025	370	496	892	995	621	917	518	404	490	575	446	428	1675	849	1297
202980_s_at	237	218	163	136	315	310	135	191	173	136	139	246	209	126	148	314	274	298	413	385	485
202981_x_at	1554	1766	1678	1814	1171	1738	1389	1605	1604	1549	884	729	1163	1100	1179	2647	3053	2818	3252	3650	3789
202982_s_at	19	16	456	622	777	933	78	58	286	365	527	557	850	545	273	2	36	1	376	388	348
202983_at	2189	1732	47	50	170	188	2938	3331	35	64	54	79	14	20	39	2380	2054	2009	55	242	327
202984_s_at	181	5	142	137	286	255	163	234	226	256	361	375	1027	637	164	65	95	89	456	327	271
202985_s_at	1718	1032	1412	1129	2357	2004	1306	1272	1717	1501	2599	2557	2631	2374	2615	2391	2453	2300	3076	3242	3673
202986_at	40	140	12	18	158	172	232	198	12	22	161	176	71	71	14	117	103	106	4	4	17
202987_at	45	85	71	96	157	31	114	95	107	42	53	8	8	35	17	12	19	9	15	16	35
202988_s_at	8	2	4	8	15	48	5	22	4	3	6	8	2	2	2	3	1	2	3	20	20
202989_at	3	26	57	50	10	63	32	3	13	3	26	3	2	2	2	18	14	2	1	3	1
202990_at	2447	2962	962	1950	1350	1424	1783	2123	899	1308	1056	1291	1287	1450	2125	3577	3480	3381	1933	1598	2146
202991_at	283	309	193	277	445	292	398	306	297	419	205	219	252	224	68	119	35	90	67	98	41
202992_at	13	17	31	26	274	89	17	22	70	36	27	35	22	4	8	5	5	9	5	37	17
202993_at	246	359	308	257	106	77	811	596	674	270	492	197	297	210	157	231	233	240	351	383	253
202994_s_at	24	36	12	25	43	51	52	41	8	13	27	31	16	72	55	149	205	178	22	39	45
202995_s_at	221	214	69	125	181	184	249	241	124	119	90	155	26	35	49	98	123	136	13	56	43
202996_at	69	405	1236	1472	62	204	86	195	341	1210	411	387	451	442	257	199	173	147	388	442	290
202997_s_at	17	43	8	10	21	60	11	20	7	8	51	10	7	7	7	6	4	5	4	2	3
202998_s_at	783	1488	83	141	636	1218	529	934	116	32	1124	671	355	684	945	1053	1077	673	79	25	39
202999_s_at	32	79	35	72	225	220	115	29	119	28	26	109	9	13	56	10	9	9	29	2	7
203000_at	13	7	7	17	12	102	11	21	13	11	4	13	10	4	3	3	2	3	2	1	1
203001_s_at	56	24	7	49	40	223	33	63	15	73	80	15	3	20	21	18	12	13	13	4	3
203002_at	704	1879	1282	1509	2166	3679	457	603	773	1174	2409	3269	2821	2047	1787	1532	1582	1322	2329	1625	1916
203003_at	157	83	126	131	62	103	36	115	24	198	47	40	58	28	10	8	71	44	33	77	13
203004_s_at	156	114	102	101	98	62	164	125	151	20	118	52	25	11	42	55	130	101	73	77	58
203005_at	1040	1821	909	629	500	547	529	482	579	495	426	230	309	218	160	181	307	228	234	243	126

203006_at	816	1084	1169	1112	999	955	969	710	1371	970	951	773	576	616	692	730	729	810	1590	1170	1418
203007_x_at	1532	1282	1527	1111	819	1083	1512	1854	1904	1600	1400	1186	1515	1227	1283	2019	1428	1511	2650	1644	2920
203008_x_at	769	541	2793	2407	2999	3169	1018	839	2523	2668	3576	2952	4101	3921	5983	1302	1074	1044	4715	3903	4602
203009_at	106	176	246	834	142	97	65	38	155	260	87	29	13	28	64	12	32	56	93	59	19
203010_at	172	38	114	360	396	607	56	169	286	316	438	418	119	88	110	52	64	50	135	91	105
203011_at	2233	1922	893	1049	1812	1317	3413	3242	1496	1189	1150	1144	1439	1470	2811	5214	4187	5117	2428	1589	3105
203012_x_at	26946	35041	23609	44677	24160	37012	28993	29875	29930	26968	23451	30904	19968	26640	32024	29229	31440	32699	17170	25907	20931
203013_at	438	297	799	601	1038	954	689	624	1030	864	792	885	731	763	927	659	557	650	1006	802	1130
203014_x_at	480	501	366	495	129	120	286	293	341	453	205	204	83	112	83	214	150	153	153	112	104
203015_s_at	512	355	431	341	445	351	799	789	478	548	470	289	206	173	161	363	321	543	466	192	326
203016_s_at	791	466	756	563	794	535	966	1048	728	737	604	410	629	505	688	1950	1780	1674	1873	1275	1492
203017_s_at	639	646	419	197	498	341	640	694	436	509	270	235	351	298	435	1327	1250	1096	977	546	833
203018_s_at	450	268	277	180	52	215	541	441	254	204	135	194	224	119	216	516	452	485	553	377	541
203019_x_at	220	365	201	215	319	271	229	368	393	337	178	271	177	114	176	525	490	428	376	362	421
203020_at	229	246	270	1082	236	337	281	461	487	500	254	327	239	277	417	423	326	389	758	950	1370
203021_at	336	498	340	1342	592	1880	454	251	455	245	364	444	275	367	729	271	274	257	186	782	377
203022_at	2519	1299	1059	592	1139	317	2313	2532	975	1372	1816	2630	2680	1560	1148	786	716	906	1083	684	534
203023_at	1210	397	727	184	1732	1419	957	567	884	661	966	1049	2531	2450	1546	1155	1158	844	1616	1115	674
203024_s_at	1499	1433	1367	2043	2098	3123	1472	1327	884	864	2027	1501	2243	1467	1236	1190	1069	1314	1556	1889	2460
203025_at	1406	1315	2405	1566	2392	1423	1007	731	1964	1690	1892	1042	1989	1389	1272	625	588	413	1567	1528	797
203026_at	1413	1147	723	777	893	1196	1239	1345	661	756	1244	1051	363	443	344	556	641	571	326	449	450
203027_s_at	464	217	246	75	198	168	396	198	76	106	192	16	22	77	7	13	34	8	8	11	3
203028_s_at	2737	4299	4235	6316	1596	2483	2531	2305	3143	3867	1785	2251	3174	1885	964	1254	1343	979	2444	1997	927
203029_s_at	89	111	11	34	2836	1146	34	24	11	22	1993	2510	3053	1402	3505	107	150	100	75	204	332
203030_s_at	11	6	9	8	555	312	7	8	5	6	748	731	2123	2772	697	13	19	6	4	23	36
203031_s_at	1441	3210	1001	1028	400	551	1859	1785	860	1266	564	635	570	623	501	1035	1286	803	1082	1017	690
203032_s_at	517	304	300	195	422	404	392	471	467	479	462	418	1073	672	350	350	215	294	564	378	741

203033_x_at	2092	1375	2500	1443	2515	1312	3776	2437	2360	2071	2948	1808	4623	5013	7264	4951	4710	5169	6196	5362	6432
203034_s_at	34322	43504	30911	37454	29148	28733	28383	32950	32022	27846	24827	38478	23653	31609	27503	29540	31179	29818	19504	27226	23407
203035_s_at	482	718	426	507	410	431	394	594	537	625	368	260	207	225	222	392	390	376	428	310	272
203036_s_at	15	10	18	25	44	52	15	16	25	42	14	30	5	55	9	2	59	19	7	11	3
203037_s_at	171	221	465	670	1175	1152	295	302	431	1462	664	941	675	826	1573	197	225	226	310	322	288
203038_at	1147	1496	3067	4761	1019	1398	1198	1265	1917	3181	780	695	667	844	1180	2107	2065	2124	4963	7809	9255
203039_s_at	1110	865	1602	1301	1864	2129	1507	1247	1571	1269	1508	1273	2126	2279	2372	2063	1968	1893	3064	2339	2899
203040_s_at	2076	1839	2551	1103	1722	1377	1605	1846	2911	3108	2029	1865	1453	1350	1540	1489	1459	1623	2659	1439	1308
203041_s_at	680	1588	1035	2073	1229	2607	570	792	640	689	1205	1869	1997	1908	1448	1012	1423	929	1494	2673	3033
203042_at	258	577	266	599	514	1228	188	269	261	285	340	593	969	616	795	638	536	592	878	1731	2109
203043_at	561	698	361	564	761	586	600	561	376	394	870	574	503	490	325	303	361	292	298	355	281
203044_at	934	877	861	529	915	923	942	947	760	1122	745	721	1177	938	891	3019	2813	2487	2083	2104	2684
203045_at	519	900	273	307	588	602	578	362	219	238	716	584	586	734	598	409	474	445	301	307	223
203046_s_at	1438	715	1101	594	1364	710	1273	1479	1614	1585	1717	1596	2043	1479	1153	709	799	684	1380	666	677
203047_at	407	327	439	273	892	943	400	278	412	442	435	265	164	208	220	184	139	93	238	162	184
203048_s_at	224	263	749	661	862	1031	407	488	562	690	735	589	649	576	693	296	285	221	820	905	1246
203049_s_at	355	377	713	576	837	1863	203	350	415	552	520	457	1693	1266	1681	667	649	733	2521	3099	4119
203050_at	1168	966	766	1176	620	728	1632	1236	1192	784	617	602	294	293	261	537	343	470	514	590	848
203051_at	366	224	315	212	830	886	201	379	384	218	286	328	153	177	92	161	188	118	187	153	106
203052_at	85	162	183	178	344	219	152	177	86	127	173	137	57	82	115	20	87	69	47	21	8
203053_at	1433	1522	1427	1101	2529	1555	1638	1388	1302	1276	1244	992	2904	2784	3415	3959	3343	4441	3966	3193	3358
203054_s_at	524	465	754	1293	1063	1165	513	533	619	848	1357	1134	636	702	681	264	219	314	374	372	304
203055_s_at	642	422	1122	929	518	465	553	702	1049	1223	594	408	151	135	143	152	203	126	266	195	173
203056_s_at	116	23	172	230	329	132	216	125	99	155	210	288	54	81	29	15	5	4	15	5	26
203057_s_at	372	299	471	374	608	392	277	383	400	463	769	705	619	449	746	336	301	285	466	689	754
203058_s_at	283	462	1047	493	89	202	514	346	971	1358	68	132	38	17	44	267	221	211	2171	2344	3263
203059_s_at	263	292	545	433	70	20	379	310	710	971	142	186	8	32	21	69	23	81	232	222	315

203060_s_at	1000	1061	1514	927	93	70	490	544	1576	1822	92	75	51	73	53	713	652	662	6227	7530	11617
203061_s_at	365	23	164	52	27	26	411	53	51	45	48	23	110	91	14	24	50	48	55	64	8
203062_s_at	798	373	496	353	605	478	1023	881	605	643	516	549	376	275	558	830	711	768	769	467	627
203063_at	251	350	159	228	401	429	248	215	272	183	58	75	181	234	68	217	172	166	250	212	209
203064_s_at	1586	1896	718	691	779	925	1398	1230	712	724	1029	549	510	436	409	854	832	916	454	387	350
203065_s_at	2508	3449	978	327	144	157	2027	2470	991	1198	291	300	377	404	290	6967	6151	6159	4300	1050	868
203066_at	2576	1937	39	63	509	1364	1304	1556	17	11	541	708	376	290	336	1510	1766	1226	41	286	488
203067_at	1433	777	1457	1168	789	1302	1406	988	2111	1027	1320	718	1536	1475	1881	2059	1998	1825	3836	2436	4451
203068_at	636	410	296	75	507	453	408	211	166	166	610	676	544	363	799	731	813	695	682	467	496
203069_at	24	22	38	44	32	19	16	18	15	14	111	50	156	166	228	7	6	18	63	177	143
203070_at	26	27	50	40	139	23	61	22	55	28	21	23	20	32	14	6	59	5	42	24	7
203071_at	37	589	222	720	346	212	46	120	31	46	406	190	247	215	175	154	146	104	132	117	92
203072_at	499	686	267	282	506	456	380	393	284	320	310	258	104	69	136	129	168	103	107	132	92
203073_at	1200	832	1038	1291	1079	1076	1181	1083	1418	1325	1217	1018	712	783	811	1162	1148	1126	1403	1239	1652
203074_at	456	341	435	488	562	1410	505	492	462	425	507	749	104	98	90	44	55	49	61	24	18
203075_at	1254	2255	960	1222	755	1218	1238	1481	973	987	792	671	817	475	976	3179	2776	2276	2134	1846	2342
203076_s_at	1832	2721	1522	1977	591	1004	2115	1737	1470	1477	958	887	607	642	1108	2154	2111	1699	1163	1125	1467
203077_s_at	613	762	350	357	480	499	521	496	372	295	240	268	401	354	364	1047	1162	948	748	683	725
203078_at	110	97	80	58	110	173	61	48	47	45	68	50	132	111	66	213	167	170	445	158	341
203079_s_at	843	641	651	724	1134	954	742	595	661	436	702	375	807	757	913	1482	982	1090	1264	1131	1566
203080_s_at	222	315	720	574	470	740	327	402	365	421	345	273	316	173	299	254	291	194	381	572	689
203081_at	382	277	153	410	131	292	415	579	191	256	260	297	160	199	159	278	287	278	121	192	183
203082_at	1871	675	1283	808	1905	1149	3144	2102	2097	2089	1562	1489	1121	743	534	938	860	827	946	741	471
203083_at	148	85	20	67	63	26	42	174	179	285	57	99	21	2	7	9	41	43	25	51	29
203084_at	12	18	33	26	74	172	15	21	36	35	13	10	31	114	80	62	14	9	47	36	31
203085_s_at	685	597	2189	1223	537	243	808	896	1508	2342	1285	462	223	621	856	653	628	479	1123	1119	785
203086_at	261	262	571	402	370	59	355	496	518	537	500	245	172	207	121	194	159	202	229	139	280

203087_s_at	859	718	1346	689	1240	746	1295	1155	1406	1252	1165	924	1277	537	551	652	694	571	1741	1093	1641
203088_at	32	24	38	85	30	43	219	30	31	27	122	221	3	17	4	48	14	11	10	3	7
203089_s_at	822	1071	906	751	488	758	1162	925	867	601	1083	1022	1199	952	554	361	428	372	641	666	248
203090_at	980	675	1025	1556	994	667	991	1106	1061	1156	981	1215	898	693	718	748	720	666	619	685	437
203091_at	1509	428	1388	885	1205	543	1897	1242	1660	1416	1208	1139	1173	1476	1440	1107	962	1624	1339	703	1180
203092_at	893	560	312	136	711	829	543	269	260	166	300	106	432	565	590	649	727	607	423	540	406
203093_s_at	1082	771	897	391	964	1110	987	451	831	440	688	387	546	391	305	613	527	484	462	421	355
203094_at	732	528	532	495	1013	664	481	622	532	468	671	715	809	756	886	1262	1196	1117	641	588	604
203095_at	1527	1676	1286	1058	1828	3228	2178	1399	1805	1212	1329	1370	2662	2497	3033	2679	2484	2664	3261	3119	3655
203096_s_at	235	157	247	291	443	333	295	583	244	313	290	245	17	50	65	105	47	84	88	115	102
203097_s_at	927	1495	975	904	847	1118	755	1156	561	880	782	841	745	500	1502	2376	1908	2105	1510	2144	2698
203098_at	828	1029	552	597	896	931	657	1027	611	662	770	773	792	1024	1357	1687	1402	1253	903	1199	1568
203099_s_at	142	240	145	40	48	43	447	218	74	105	249	65	231	366	290	283	281	171	189	153	205
203100_s_at	601	585	395	247	686	793	456	541	531	464	522	434	603	696	794	944	741	699	823	565	860
203101_s_at	13	11	11	13	180	78	93	9	86	32	16	25	59	31	10	39	11	64	29	4	23
203102_s_at	1349	1248	1157	1062	1226	1216	701	706	606	685	1283	1345	2177	1784	2816	2281	2019	2271	2692	2917	3935
203103_s_at	2256	1645	2931	1921	3123	2434	2313	2191	4146	2882	3151	2990	4170	2920	2742	1381	1579	1156	3249	2327	1584
203104_at	85	30	76	17	76	129	101	128	120	94	51	40	23	14	18	3	19	18	15	49	17
203105_s_at	960	656	1279	625	1362	1176	1169	1167	1006	992	1349	984	985	946	1381	1276	970	1363	1149	1466	1740
203106_s_at	155	154	221	119	102	167	167	190	149	196	55	172	94	84	90	166	141	64	150	172	214
203107_x_at	36136	49189	32492	42181	36789	44164	36613	34861	37783	31425	26325	41112	20581	29010	29428	28672	31973	34421	18529	25557	22110
203108_at	4921	3500	7684	3285	2863	1711	2482	2733	4841	8739	4180	5404	1918	2826	4771	7591	9872	8367	8020	6549	6957
203109_at	2737	2120	2873	1588	2179	1861	2969	1677	3342	2531	4153	1820	5980	4167	2854	1988	2077	1834	2475	2152	1069
203110_at	77	249	182	171	102	120	175	232	103	292	368	369	71	84	129	73	146	7	65	14	57
203111_s_at	25	35	18	39	54	62	17	37	41	41	16	13	4	6	9	7	12	9	9	6	5
203112_s_at	841	665	457	382	423	284	490	781	349	411	296	216	316	278	203	463	322	425	451	262	270
203113_s_at	9145	16527	6299	6103	5686	7206	8459	7650	5659	5768	6415	6505	9653	8267	5212	6629	7906	4032	4099	6210	3663

203114_at	679	418	842	682	862	640	656	680	1195	527	679	461	1348	776	543	347	587	516	885	714	509
203115_at	311	226	307	384	36	20	351	383	249	405	114	134	113	93	72	366	278	235	306	249	353
203116_s_at	1569	1797	606	828	414	547	1595	1488	795	952	631	403	274	260	305	1625	1624	1605	586	400	550
203117_s_at	238	272	110	278	212	280	134	285	134	176	132	197	190	198	149	208	208	192	184	207	335
203118_at	204	186	347	313	99	99	168	108	384	416	608	338	343	493	468	219	329	244	443	293	267
203119_at	1888	1451	1876	692	2115	1717	1647	1320	1638	1349	1875	1085	1600	2019	2525	2161	2025	2002	2542	1417	1194
203120_at	750	544	414	551	647	1078	500	552	522	450	453	523	502	280	358	828	810	623	634	653	925
203122_at	213	380	346	305	360	234	68	249	345	224	273	225	345	504	495	408	401	306	564	552	543
203123_s_at	429	540	716	742	440	419	910	717	959	941	640	552	233	390	198	260	299	145	363	307	325
203124_s_at	635	954	677	704	554	618	790	629	919	767	742	418	508	706	648	1168	954	886	1515	1238	1738
203125_x_at	445	291	343	348	197	301	563	529	503	588	578	395	144	141	175	241	212	251	184	209	253
203126_at	618	844	1583	3086	594	443	726	895	2724	3328	813	1113	657	549	727	373	334	300	1768	945	728
203127_s_at	766	884	1195	662	1350	712	892	999	1061	1324	1281	980	855	1112	1331	824	801	982	1239	1161	1408
203128_at	343	152	633	785	87	63	497	702	541	669	573	582	383	350	337	124	125	152	511	553	468
203129_s_at	13	17	16	12	44	34	25	25	15	14	10	13	5	9	4	156	190	133	9	8	7
203130_s_at	517	671	183	147	217	242	591	573	80	179	367	341	41	63	90	348	342	290	57	9	37
203131_at	8	33	9	10	21	77	12	34	29	11	13	18	1	4	3	12	2	2	13	17	6
203132_at	795	502	705	380	1994	2184	515	750	670	928	1137	1181	3009	2762	2013	1223	940	1130	2700	1966	2627
203133_at	6121	3530	3964	4058	7677	8937	3937	4213	5073	3312	5729	4748	12090	7056	4597	4139	5425	4722	5219	6948	4637
203134_at	159	140	441	375	165	171	311	369	585	416	381	293	90	116	58	199	183	169	152	250	189
203135_at	845	601	339	383	459	482	731	644	380	398	460	476	378	458	593	784	591	641	407	571	514
203136_at	1480	1831	1465	2193	2082	3170	1723	1623	1183	1660	2877	2421	2563	1801	1037	635	834	603	459	958	560
203137_at	2650	2994	1443	1134	1939	2041	1709	1795	1184	1287	1822	2202	2741	1755	3141	4443	3378	4614	3347	2856	3853
203138_at	1294	713	2493	1566	3032	1656	1813	1733	2430	1892	2625	1771	5523	5718	4659	3009	2966	4241	5804	3250	4348
203139_at	292	411	571	3245	492	590	534	360	756	1159	142	150	32	40	37	236	241	157	983	1379	1671
203140_at	472	595	271	1535	276	341	317	541	317	440	166	302	120	176	168	678	753	839	363	1128	1529
203141_s_at	123	75	164	144	291	337	278	317	269	310	213	83	50	35	4	100	45	76	95	107	122

203142_s_at	636	810	1575	1485	488	120	1591	1967	1519	2161	1013	1170	515	572	660	1114	1007	832	1088	858	901
203143_s_at	337	94	572	102	388	292	457	542	391	846	372	363	79	89	69	129	100	141	161	115	104
203144_s_at	123	56	292	195	122	56	215	277	235	523	193	189	43	70	7	55	54	43	74	77	54
203145_at	964	319	939	287	1141	306	1044	1206	1312	941	1347	1348	1837	1760	1169	659	741	708	1377	445	590
203146_s_at	283	521	227	237	150	353	339	354	115	215	165	166	95	51	51	74	96	154	36	52	4
203147_s_at	467	359	369	446	198	146	333	309	330	218	487	287	515	482	133	234	233	280	223	232	185
203148_s_at	728	903	438	339	748	403	575	657	595	300	1018	768	1029	1042	1388	1440	1755	2638	1501	1790	2582
203149_at	599	866	1028	1009	610	605	992	879	900	974	866	464	404	394	270	284	256	295	280	254	148
203150_at	1750	1327	967	406	888	593	1617	951	916	758	1089	1046	1300	1305	2432	2140	1926	2227	1095	915	752
203151_at	525	415	411	636	480	148	629	611	428	559	335	441	25	39	48	73	96	40	60	56	54
203152_at	1364	1525	2410	3122	1333	1924	2394	1721	2707	2710	1502	1894	2135	2147	1871	2007	2022	2068	2672	2455	1530
203153_at	38	459	83	713	18	27	205	179	393	506	14	24	4	3	6	375	1696	11390	1276	5172	8006
203154_s_at	997	926	1150	1106	1064	172	719	1083	1618	1244	1086	617	624	372	590	558	483	557	1185	851	792
203155_at	245	516	293	837	78	242	440	548	439	390	203	103	135	117	94	209	234	375	263	270	296
203156_at	1066	1322	1945	1959	2059	2025	1567	1849	1727	2165	2182	2065	1434	950	1810	1452	1311	1583	2337	1796	2898
203157_s_at	253	201	408	365	734	363	341	428	401	288	512	542	113	121	204	179	135	88	109	180	185
203158_s_at	201	144	232	260	415	270	136	223	197	162	340	322	69	145	188	118	74	49	106	129	120
203159_at	488	476	614	760	1298	648	661	569	413	600	762	1007	357	549	1018	453	415	259	320	748	650
203160_s_at	753	656	471	393	888	559	912	706	610	609	776	638	652	439	807	723	788	733	497	472	393
203161_s_at	9	11	8	14	26	27	15	17	9	8	13	26	6	6	9	5	7	6	6	4	7
203162_s_at	844	845	1243	832	867	857	1041	937	1175	1353	1410	985	1146	1527	2604	1771	1849	1822	2924	3178	3070
203163_at	152	223	370	265	48	23	241	268	476	506	323	256	124	148	281	131	137	184	254	260	318
203164_at	544	455	1036	839	900	1310	591	540	488	496	661	914	670	771	644	789	784	635	549	1421	1578
203165_s_at	200	223	321	211	370	997	159	117	153	166	244	318	339	268	337	302	247	303	254	459	659
203166_at	1056	898	734	1185	1067	1132	1049	1128	645	650	1032	1087	1858	1536	1488	1733	1438	1435	1063	1085	812
203167_at	221	320	56	301	642	816	237	193	24	48	1245	1092	755	828	1171	183	197	195	76	49	67
203168_at	517	330	209	407	510	614	348	389	337	302	267	317	129	174	195	161	154	88	96	184	80

203169_at	398	389	491	349	1325	1064	413	340	587	389	395	418	473	252	174	277	194	176	315	383	296
203170_at	11	38	66	96	120	105	58	74	120	82	50	111	26	26	31	26	32	22	71	31	58
203171_s_at	530	454	604	334	551	711	342	393	676	591	875	729	846	656	1073	275	396	357	688	552	523
203172_at	352	502	378	144	429	421	77	80	67	409	104	52	243	128	169	145	63	127	144	217	100
203173_s_at	427	302	34	239	329	528	232	239	92	70	219	224	222	549	363	464	402	386	130	152	125
203174_s_at	488	367	420	550	553	395	420	476	338	341	559	323	138	139	171	65	144	147	80	59	34
203175_at	1771	1736	1542	1773	687	880	1328	1635	1544	1677	2081	833	1041	1233	1370	1608	1765	1342	938	884	515
203176_s_at	716	186	874	544	717	274	693	651	2102	1440	1037	632	1919	991	274	197	189	348	1072	527	1094
203177_x_at	1106	496	1879	736	1281	871	771	735	1664	1272	1164	1090	1927	1500	1910	1602	1166	1395	4538	3544	4563
203178_at	50	33	46	34	36	4	5	48	15	36	7	35	4	3	19	9	18	26	28	41	52
203179_at	396	511	266	670	487	775	286	550	318	498	559	706	403	461	393	337	304	319	300	257	254
203180_at	7255	22178	1531	10918	3384	2200	7428	5314	887	498	3742	1124	2535	2204	2898	12470	13544	9954	1480	566	632
203181_x_at	528	772	406	556	502	345	652	583	221	485	669	572	1851	2196	2426	1113	988	710	870	1171	585
203182_s_at	855	945	722	816	1832	1730	1177	885	809	760	1518	1315	2166	2072	2733	1746	1618	1434	1572	1299	1592
203183_s_at	519	367	619	499	474	380	628	769	559	668	1090	561	59	88	121	89	109	123	112	91	100
203184_at	103	52	66	67	109	133	72	86	48	91	60	85	20	17	12	20	27	26	29	31	33
203185_at	169	240	138	144	258	188	319	219	78	116	183	88	96	62	35	147	194	94	25	24	20
203186_s_at	3413	7899	5701	10367	13764	13807	7541	7518	10171	14875	14469	20408	20943	19208	16129	14277	14052	12352	12024	14924	8377
203187_at	848	1184	647	969	840	716	682	1169	676	756	757	514	183	144	75	136	155	226	208	150	123
203188_at	667	405	773	689	926	922	493	483	839	707	750	748	256	359	273	199	149	187	465	755	552
203189_s_at	975	994	1034	1020	847	986	704	581	954	840	851	589	1979	1742	932	957	892	1075	1416	1456	1042
203190_at	2823	3097	3405	3128	2243	1974	2219	1927	2494	2664	2045	1343	5378	4346	3375	2602	2547	2094	4220	4203	2170
203191_at	5	4	5	9	120	207	12	20	9	8	11	5	44	8	10	8	10	10	10	8	9
203192_at	483	667	425	810	580	595	551	450	578	541	567	338	253	268	188	201	226	149	214	139	128
203193_at	339	305	446	427	707	575	295	224	480	430	294	215	116	53	87	55	34	131	175	114	114
203194_s_at	896	253	590	437	1001	923	996	860	610	781	1303	1204	414	421	273	233	154	259	264	217	229
203195_s_at	503	369	547	458	696	933	852	924	733	697	1403	831	679	572	493	327	372	518	500	317	357

203196_at	541	854	1000	892	1082	1661	714	641	939	751	718
	445	587	370	842	1274	1093	854	1008	1482	2573	
203197_s_at	785	742	869	1305	93	273	420	986	807	990	790
	929	798	899	432	585	638	538	841	826	567	
203198_at	437	451	316	420	451	527	510	346	316	435	331
	337	804	608	428	637	508	634	503	335	332	
203199_s_at	226	146	346	389	476	652	350	321	429	493	303
	294	380	283	443	122	216	140	648	443	655	
203200_s_at	519	557	1195	745	1050	1218	664	673	1200	1037	1360
	1010	1962	2401	3420	1334	1457	2090	3142	3009	3812	
203201_at	630	780	819	269	378	269	776	566	1077	952	644
	381	502	490	680	758	881	765	1152	714	449	
203202_at	1578	1146	1023	608	601	624	1846	1929	977	953	759
	331	807	539	728	2887	2172	2819	1340	1148	2003	
203203_s_at	1099	779	695	294	653	798	1521	1343	492	321	344
	284	1052	531	344	1635	1149	1647	1039	555	705	
203204_s_at	557	436	625	768	668	581	539	528	381	605	443
	381	263	215	117	209	216	176	158	219	204	
203205_at	647	504	665	732	532	770	852	728	731	675	453
	424	236	300	190	299	349	269	224	284	207	
203206_at	829	762	638	630	807	884	1076	607	677	542	539
	441	165	142	266	181	205	164	240	269	184	
203207_s_at	979	446	1248	658	1024	800	1330	1355	1089	1139	1066
	763	633	617	790	1429	1294	1259	1339	872	1280	
203208_s_at	1298	881	1051	790	965	1437	1193	903	733	948	861
	862	1570	1122	1528	2222	2207	2487	2096	2680	3419	
203209_at	2015	595	1664	539	1501	375	1545	1478	1762	1568	1322
	1582	1718	1702	2138	2683	2227	3305	4115	1858	2751	
203210_s_at	1213	313	1167	397	548	47	1361	1103	1256	1110	1028
	1296	640	543	336	608	605	712	716	347	419	
203211_s_at	868	950	2035	1664	865	958	992	614	1935	1378	624
	398	1067	1309	901	1501	1506	1163	5391	2088	2712	
203212_s_at	191	266	1028	970	337	402	323	289	1428	920	426
	189	486	545	639	629	561	297	2092	753	948	
203213_at	2906	861	2454	560	2238	180	2629	2673	2339	2667	2798
	3072	6109	5830	8661	7228	6380	6559	10664	4855	8912	
203214_x_at	2437	641	2414	539	2465	246	3061	2840	3139	2969	2479
	3048	2447	2744	2270	2166	2219	2302	3217	937	1597	
203215_s_at	57	182	232	413	22	11	335	482	444	586	28
	29	136	123	273	663	576	499	697	866	775	
203216_s_at	1326	1530	1455	1530	481	736	967	1213	955	1581	246
	396	294	255	427	1978	1436	2064	2787	2455	3558	
203217_s_at	513	516	414	347	360	360	441	718	454	389	402
	356	146	149	180	472	382	379	254	205	243	
203218_at	872	867	745	891	1344	1140	771	776	840	995	1695
	1127	2041	2606	3009	1700	1606	1726	3011	1994	2793	
203219_s_at	2357	2302	1654	1320	1314	1224	1408	1189	1345	1734	1404
	1225	1336	959	804	1264	1132	879	1152	1036	523	
203220_s_at	257	328	412	499	400	481	176	455	496	560	125
	141	29	44	44	45	72	42	86	62	94	
203221_at	786	1538	1320	2451	536	894	563	595	1605	1765	354
	412	282	260	371	747	759	589	1686	2330	2634	
203222_s_at	271	467	442	715	87	42	123	235	460	594	14
	21	140	59	73	160	116	97	391	332	444	

203223_at	177	85	255	177	654	761	225	194	190	200	219	274	280	221	123	124	100	88	79	116	94
203224_at	1545	1443	658	1080	1042	1508	676	967	743	778	766	473	887	1189	854	2796	2370	3209	3409	1917	2807
203225_s_at	896	1007	711	520	770	620	1113	845	1504	706	812	522	977	984	952	1697	1816	2222	2877	1460	1065
203226_s_at	623	691	779	2116	713	2204	872	986	856	1340	1039	1253	609	511	541	474	571	354	473	497	710
203227_s_at	616	1279	800	2196	1065	2551	306	579	488	1075	983	1532	962	569	701	669	982	464	1188	1802	1696
203228_at	1115	1298	1321	1426	1302	1246	1423	1285	1786	1171	1056	1421	2287	1895	1940	1812	1912	1527	1881	1677	1190
203229_s_at	1261	944	665	935	287	220	855	1211	601	654	601	656	418	318	450	392	513	565	660	477	563
203230_at	1389	1342	950	537	2021	2268	677	846	539	776	448	530	543	446	472	567	539	575	516	516	381
203231_s_at	7	26	134	195	267	246	68	107	151	324	254	183	76	87	45	32	22	4	58	46	57
203232_s_at	202	386	868	879	1486	2178	261	269	550	990	1032	695	375	395	340	172	179	126	550	524	630
203233_at	587	564	553	702	73	38	458	454	633	887	263	182	40	79	103	197	256	166	413	265	274
203234_at	709	597	332	84	399	2006	404	169	218	31	800	481	1195	953	750	498	614	382	313	666	384
203235_at	635	568	633	103	478	163	680	449	534	531	628	207	382	604	731	555	663	453	519	340	282
203236_s_at	163	247	130	400	69	36	24	33	56	43	45	26	9	7	10	30	91	208	121	107	64
203237_s_at	46	45	33	35	55	44	41	51	48	34	134	31	13	13	14	12	3	22	23	11	22
203238_s_at	415	471	156	188	99	50	546	639	116	137	61	53	2	42	31	138	219	101	12	9	17
203239_s_at	816	715	974	1212	636	547	1016	710	894	901	1003	292	213	291	376	469	432	517	545	368	380
203240_at	48	123	38	44	103	56	24	63	39	59	38	39	35	7	7	15	19	35	48	67	91
203241_at	288	247	515	538	532	554	392	248	618	375	397	368	278	277	542	258	221	217	458	557	567
203242_s_at	450	263	643	348	462	130	364	622	443	717	347	375	57	130	138	180	141	166	165	136	182
203243_s_at	914	799	1546	758	1703	783	510	808	761	1266	514	407	525	390	438	920	758	920	2445	1778	2669
203244_at	1060	1064	951	1302	1568	1394	1305	627	1284	1252	1229	1136	1075	703	1248	635	499	575	875	629	591
203245_s_at	380	499	155	458	304	425	431	575	189	270	354	515	340	290	451	677	594	566	367	297	458
203246_s_at	1548	727	775	744	1003	1145	933	841	900	626	1020	1077	1162	1221	884	715	1006	811	1028	1070	941
203247_s_at	464	254	604	719	308	241	603	748	389	500	433	397	396	214	172	410	366	263	395	319	235
203248_at	406	288	381	453	291	222	682	595	280	393	324	220	149	115	102	269	331	268	165	167	186
203249_at	206	90	61	224	55	134	28	96	36	61	67	156	84	112	76	22	84	88	62	67	91

203250_at	1098	1111	1161	1225	1324	1423	1093	1581	1133	1194	1512	1320	1590	1206	1508	1718	1353	1976	2074	1089	1490
203252_at	1298	675	2382	1487	282	685	567	682	760	992	598	620	1432	1140	579	848	1050	1089	887	1597	982
203253_s_at	380	401	705	444	561	900	388	302	452	418	384	347	979	408	482	584	446	641	1286	1082	1450
203254_s_at	507	373	411	281	564	572	488	538	407	492	698	367	121	67	77	113	101	82	113	101	86
203255_at	205	397	364	491	533	429	269	352	392	363	580	519	601	873	1570	1061	852	854	1670	1085	1054
203256_at	3003	4198	985	1456	213	620	3201	4831	606	1022	354	308	555	229	530	2454	2917	2806	761	950	1146
203257_s_at	816	1197	977	2109	737	1172	656	694	809	886	797	1213	606	594	778	604	534	680	1060	1314	1069
203258_at	1509	1311	2697	1001	1296	879	1798	2015	2866	3407	2568	1560	832	644	422	597	482	265	1024	752	343
203259_s_at	3248	4108	3272	3555	1150	1588	4191	3389	3941	4108	1724	1813	1688	2076	2938	4917	5318	5118	5259	4604	3606
203260_at	169	140	155	188	137	151	233	179	106	133	134	96	75	76	153	290	319	264	170	195	163
203261_at	1482	1244	1411	1425	1384	1142	1933	1747	1338	1414	1516	1222	1826	2298	2791	4146	4470	4027	2184	1827	2204
203262_s_at	1320	2014	2630	1913	1674	1680	1154	1136	2471	2936	2037	1688	3143	2558	2643	866	876	603	1470	2512	1192
203263_s_at	171	146	137	294	58	297	175	158	201	172	217	227	108	128	147	105	81	92	114	119	136
203264_s_at	130	179	132	303	221	285	150	227	153	229	185	180	72	79	27	115	157	122	139	64	135
203265_s_at	319	268	319	230	463	652	697	501	506	456	578	475	301	257	314	541	525	575	355	367	351
203266_s_at	750	797	728	555	753	813	1138	913	811	702	716	592	437	570	805	1292	1504	1116	854	448	789
203267_s_at	474	656	780	667	66	177	619	558	920	662	816	388	637	576	659	385	339	305	515	362	332
203268_s_at	538	557	539	432	318	70	560	467	549	579	424	506	373	294	198	200	245	179	358	272	257
203269_at	992	1291	633	323	1196	1291	741	868	603	482	1711	1409	2808	2518	2749	1772	1663	1388	1071	1320	1568
203270_at	1200	517	633	308	767	141	896	685	1013	834	796	1058	1570	582	708	365	467	300	903	433	210
203271_s_at	247	549	56	281	96	89	399	310	51	66	74	210	130	124	62	92	132	128	21	11	8
203272_s_at	853	1090	625	542	1063	728	991	878	1030	914	1494	1379	2016	1298	566	554	628	522	477	350	173
203273_s_at	589	575	647	848	416	473	578	855	681	625	1117	714	822	699	421	234	313	244	349	222	169
203274_at	1455	1792	4098	6000	1847	1953	1696	2115	4264	6820	3706	2493	2094	1681	1539	655	674	605	2856	3268	2134
203275_at	314	223	313	467	333	496	276	344	219	347	725	865	147	215	203	138	144	177	158	188	200
203276_at	1101	461	1111	347	1586	125	970	1227	1325	1361	1350	845	1573	1250	1110	759	688	462	1863	597	401
203277_at	927	1002	1101	1040	1046	875	776	874	862	942	1226	910	679	561	645	934	898	1032	571	420	421

203278_s_at	364	369	205	221	617	997	272	228	108	68	333	325	246	241	327	286	316	297	175	453	496
203279_at	464	275	684	489	881	1125	354	372	440	542	568	622	300	335	284	176	120	267	303	456	667
203280_at	276	286	321	356	635	583	303	296	320	321	324	244	65	78	117	71	133	71	130	114	80
203281_s_at	42	302	31	58	148	163	34	42	40	32	38	40	43	92	7	32	21	95	55	75	116
203282_at	1427	1442	335	265	909	829	1043	929	258	389	932	687	1234	1050	1786	2604	2278	1999	683	1228	1681
203283_s_at	324	454	467	433	297	309	325	373	140	398	237	304	294	310	245	572	403	490	791	741	1182
203284_s_at	693	810	1563	1476	374	505	881	1168	1631	1659	568	469	440	382	652	1318	1153	1047	2373	2139	2680
203285_s_at	235	257	426	457	313	314	380	388	478	464	249	377	323	186	182	373	304	316	887	747	873
203286_at	1041	1452	1015	947	1647	1793	763	1027	995	1266	1725	1781	856	1082	1162	474	548	474	810	934	798
203287_at	1111	927	2699	1641	969	740	1618	961	4012	4264	1239	770	273	193	671	593	520	364	1794	879	714
203288_at	451	656	515	647	885	1387	476	554	541	725	1325	1059	880	1070	1325	631	691	480	565	702	710
203289_s_at	352	303	545	420	685	656	404	376	531	319	470	145	65	112	102	31	57	140	166	83	76
203290_at	20	17	15	17	41	114	8	9	13	17	14	11	21	6	2	3	2	3	7	11	4
203291_at	540	291	585	596	686	423	448	556	408	572	571	470	403	443	754	723	578	701	582	544	915
203292_s_at	583	445	483	724	577	1360	429	463	500	513	703	566	449	532	603	288	351	335	233	360	282
203293_s_at	366	145	252	163	315	375	277	413	268	451	324	214	22	28	5	78	69	120	83	74	220
203294_s_at	171	131	201	106	121	210	220	183	246	375	223	207	3	3	5	10	9	11	31	33	148
203295_s_at	21	19	73	109	115	16	33	104	36	110	54	77	40	7	5	10	4	13	8	34	3
203296_s_at	60	75	156	81	16	169	57	77	79	85	63	49	16	22	8	5	16	4	8	17	17
203297_s_at	1820	1696	666	806	881	1951	794	1095	627	834	767	660	273	327	262	455	459	331	349	371	259
203298_s_at	833	980	446	385	62	628	692	914	371	331	397	329	274	420	586	819	858	670	506	582	671
203299_s_at	136	90	174	93	401	262	129	113	103	99	338	342	621	379	198	64	53	92	76	129	151
203300_x_at	344	298	254	147	869	535	403	430	281	247	1654	1287	1326	1370	1055	327	360	347	95	145	204
203301_s_at	733	794	836	938	944	1505	476	753	524	594	706	1014	528	548	921	685	408	595	671	542	730
203302_at	740	488	461	242	423	356	515	648	317	321	379	485	1167	805	561	1076	801	1127	1484	716	1319
203303_at	1818	1867	3350	1353	2023	2920	1554	1974	2319	3002	2011	1904	2143	2087	2531	3168	3434	3124	4041	4685	6044
203304_at	300	616	248	241	5817	5409	648	603	313	446	4325	2630	8150	9005	9817	574	516	383	641	1278	1226

203305_at	176	147	203	204	216	214	131	236	248	232	134	221	110	143	121	30	28	27	53	36	30
203306_s_at	1101	1227	1432	2115	1101	1324	1089	1123	1327	1575	1113	1247	1563	1343	1942	2209	1826	1702	2846	2040	2538
203307_at	294	414	331	238	1065	1333	333	232	329	217	317	258	178	272	239	305	227	362	214	194	130
203308_x_at	263	382	258	643	73	653	184	272	363	433	186	292	150	176	229	216	40	151	226	186	229
203309_s_at	243	393	229	283	329	371	284	231	217	275	230	256	110	109	167	100	109	140	244	273	194
203310_at	515	590	661	621	876	990	310	631	401	654	514	664	1095	1043	1159	981	800	882	2217	1675	2218
203311_s_at	1937	1529	562	347	280	172	1367	1071	716	664	662	635	1150	432	235	307	267	334	379	298	270
203312_x_at	3149	2407	3017	1853	1626	1156	3102	3071	2086	2334	2334	2284	1129	1234	1637	2670	3182	2652	2436	1462	1112
203313_s_at	3647	3530	2774	3568	1563	1950	4642	5673	2669	3912	2032	3015	2109	2622	1848	4953	4624	5597	3769	2585	3534
203314_at	533	708	958	820	889	1072	629	719	1048	878	691	513	541	580	633	322	357	293	716	513	521
203315_at	1303	2664	1283	1688	951	1896	1802	1842	1256	1827	2241	2007	1123	817	1280	959	923	879	1145	1018	949
203316_s_at	7088	5814	7093	4494	5614	4357	6519	7295	5202	5451	5207	5199	12110	10774	7062	9892	9926	11436	10278	7363	6034
203317_at	206	134	326	400	526	363	189	252	205	418	559	392	223	79	88	108	87	35	140	179	187
203318_s_at	288	257	880	1308	561	517	392	553	907	1017	992	773	833	696	381	207	225	211	817	651	798
203319_s_at	112	161	608	757	359	364	235	401	581	577	577	612	601	562	416	205	172	129	549	438	520
203320_at	577	327	751	283	871	1063	815	710	617	577	1016	868	372	386	440	524	451	386	448	358	365
203321_s_at	822	703	439	406	291	339	631	655	590	567	452	368	374	335	200	278	319	327	253	170	214
203322_at	896	1152	700	557	687	673	1007	998	629	776	537	519	589	897	629	1897	2052	1928	1067	575	1036
203323_at	1746	3703	815	1292	322	887	1403	1570	945	1698	344	376	555	801	742	6151	6325	5617	3277	1396	2607
203324_s_at	3860	5915	1519	649	742	957	1990	2128	900	1455	503	368	1223	1175	1091	7749	6437	7451	4008	1653	2117
203325_s_at	11	9	8	16	51	71	27	25	5	14	7	14	28	29	28	6	4	21	27	22	11
203326_x_at	38	34	28	50	43	126	38	24	29	36	37	40	4	8	31	9	14	32	9	9	4
203327_at	872	855	996	1154	865	962	1309	1086	1219	1182	1266	1142	1146	1277	1435	1807	1459	1745	2231	1898	2662
203328_x_at	958	777	1278	920	1152	736	1517	1349	1432	1486	1396	1173	565	551	601	691	560	743	950	857	1044
203329_at	239	448	136	235	37	42	274	311	98	99	21	48	13	8	38	173	240	122	55	118	70
203330_s_at	298	236	475	459	302	302	359	478	206	300	438	402	164	205	194	228	186	147	185	205	208
203331_s_at	19	7	45	30	41	56	9	18	72	17	36	23	3	5	5	4	2	3	16	20	16

203332_s_at	329	269	1135	1092	591	638	284	369	1430	741	948
	343	180	180	183	77	113	10	320	262	205	
203333_at	475	415	476	502	449	425	413	434	294	344	135
	254	186	150	129	186	211	275	738	656	911	
203334_at	398	245	269	176	416	388	456	414	293	256	416
	425	408	375	230	367	366	322	269	237	304	
203335_at	717	816	439	909	1019	2615	635	884	405	697	622
	1468	1430	1196	2084	963	1020	1267	1183	1793	1768	
203336_s_at	1062	753	1238	526	2041	1201	796	668	1354	1175	1923
	1638	3681	3800	3563	1375	1534	1193	2540	1517	1119	
203337_x_at	323	139	308	162	1020	292	345	219	432	236	436
	258	341	395	539	277	288	112	544	289	345	
203338_at	1234	1263	1298	953	1304	1089	1730	1506	969	1038	1091
	1297	3182	1672	1055	1522	1315	1298	1589	1285	1562	
203339_at	198	151	270	312	561	392	170	161	411	148	524
	235	373	475	660	335	371	331	500	325	557	
203340_s_at	415	279	472	440	779	860	482	442	610	425	570
	574	350	501	578	653	532	620	704	508	796	
203341_at	430	410	1449	909	1471	1103	726	653	1921	1642	1607
	1267	3059	2459	2762	1747	1385	1976	4209	2955	4128	
203342_at	554	359	1454	1981	1123	1431	523	516	1128	1107	1423
	983	1503	986	927	316	304	307	580	530	313	
203343_at	1674	878	2640	2734	1478	984	694	660	4494	2256	1435
	836	1909	1392	2283	2897	2708	2836	6127	6364	8576	
203344_s_at	2486	2828	2301	1212	2521	2391	2400	2910	1632	2034	2324
	1881	2987	3139	4852	5718	5863	6391	3623	3585	4179	
203345_s_at	1670	1025	960	808	792	509	1538	1890	1295	1071	901
	764	1098	959	372	1228	1353	900	1142	697	682	
203346_s_at	431	330	515	495	536	356	620	616	450	569	642
	437	446	537	219	371	448	473	563	318	472	
203347_s_at	578	240	298	188	234	113	415	520	227	284	240
	241	439	396	285	644	481	752	577	460	642	
203348_s_at	878	281	669	70	793	366	531	808	343	405	1491
	2534	1099	1599	1354	1500	1681	1439	534	696	1162	
203349_s_at	1680	663	1677	72	2465	814	1709	1875	748	1057	3189
	3594	2502	3523	4560	4151	3249	3572	1303	2029	2667	
203350_at	491	460	559	643	572	586	766	796	627	418	439
	408	473	474	582	543	499	487	681	611	847	
203351_s_at	861	1275	1080	1216	1439	1291	1064	1229	977	1086	1428
	1715	2371	2360	2481	2091	1634	2131	3021	2648	3622	
203352_at	163	152	129	160	224	235	146	235	174	299	183
	319	321	301	444	415	222	283	369	251	367	
203353_s_at	738	609	921	683	845	445	988	637	848	544	587
	488	821	654	952	916	930	1032	1144	979	1044	
203354_s_at	57	16	296	44	36	39	200	173	290	408	18
	23	32	43	33	6	3	52	168	128	96	
203355_s_at	287	336	794	400	115	126	235	405	359	662	114
	126	110	134	92	544	439	322	979	1332	1455	
203356_at	569	541	566	534	742	853	579	563	404	393	587
	591	787	642	511	698	516	605	772	768	1128	
203357_s_at	849	714	1044	698	701	672	1185	1046	787	844	1192
	1032	428	468	399	385	375	361	306	468	573	
203358_s_at	1249	472	1009	552	1281	550	1002	900	1133	966	830
	1195	1722	1463	1434	1211	1019	1335	2760	1646	2204	

203359_s_at	1453	876	1455	1300	1392	671	1026	1186	1421	1472	1407	985	1434	1245	894	1718	1630	1683	3800	1595	1614
203360_s_at	962	554	1305	1068	1061	128	738	1147	862	1254	742	1111	1607	1063	510	667	814	835	1689	678	748
203361_s_at	423	234	431	345	537	335	274	352	413	300	395	406	254	190	254	327	378	503	528	320	346
203362_s_at	5288	1266	2816	323	3021	441	2573	3237	2225	2395	2709	2975	5373	4765	2682	3113	2823	4511	6008	2108	2438
203363_s_at	1013	740	1156	1077	1328	908	726	765	1185	1691	1231	1117	202	203	308	308	277	268	360	284	273
203364_s_at	681	577	1092	1346	958	869	484	688	827	1054	833	946	539	508	454	347	466	502	457	676	740
203365_s_at	292	296	269	662	60	87	240	257	143	352	37	38	17	15	31	51	43	23	67	75	71
203366_at	425	381	597	480	1194	1047	359	346	432	594	306	261	400	394	339	290	362	453	1035	709	666
203367_at	1187	1159	1686	945	2033	2001	1248	1056	1711	2154	2274	2640	2109	1978	2261	1520	1608	1235	1963	1942	1793
203368_at	270	207	321	378	109	215	207	239	209	287	163	287	91	57	74	169	236	164	89	254	233
203369_x_at	220	150	119	18	209	247	240	272	136	212	262	219	14	11	19	5	19	45	15	5	7
203370_s_at	851	893	781	398	401	124	949	1073	440	769	1178	665	171	255	300	259	361	389	152	104	98
203371_s_at	1844	1926	2834	3081	2928	3603	2850	2253	2650	2724	2763	3544	3984	5506	5472	2124	2061	2170	3102	1738	1448
203372_s_at	323	354	159	12	274	316	297	220	50	31	337	157	195	129	105	167	217	217	41	47	57
203373_at	923	1074	285	122	537	697	592	464	67	183	392	297	635	536	770	1560	1388	1174	188	295	363
203374_s_at	487	437	974	617	889	563	576	618	709	551	956	1046	603	643	511	377	294	372	718	642	855
203375_s_at	626	332	724	556	931	888	464	484	622	664	760	872	869	968	1247	807	811	957	1944	1501	1652
203376_at	1856	1038	1279	1798	1362	1419	1711	1561	1916	1965	1158	1239	848	943	1529	2024	2205	1833	2184	2177	2928
203377_s_at	376	95	267	243	214	257	423	310	365	458	249	289	130	130	94	160	149	166	320	180	287
203378_at	261	192	601	574	382	523	257	330	324	423	375	454	501	475	393	702	623	302	807	464	747
203379_at	504	714	378	607	856	594	758	640	614	657	823	376	344	419	412	488	384	400	413	227	195
203380_x_at	2106	2431	1624	2531	3249	3495	1900	1997	1825	1803	2174	3026	3548	3188	2872	3276	3507	3167	2829	2891	2999
203381_s_at	12	12	243	742	803	1377	15	30	211	239	627	119	462	259	239	5	43	29	107	169	155
203382_s_at	15	10	213	648	49	1684	8	12	110	122	476	197	438	379	321	12	7	28	7	192	162
203383_s_at	450	411	578	751	1002	1016	901	908	823	721	783	833	301	257	333	234	219	272	194	258	181
203384_s_at	280	268	278	292	290	443	407	330	194	306	290	327	147	202	213	157	221	84	129	185	169
203385_at	708	457	366	728	495	810	704	670	627	509	610	412	202	171	335	345	503	508	207	171	241

203386_at	673	735	806	477	1234	1047	771	478	906	671	659	706	1057	978	1154	1274	1062	822	2178	1311	1443
203387_s_at	726	735	956	463	1221	1003	844	878	1167	1159	1235	1057	609	647	639	903	868	733	1353	694	946
203388_at	511	789	635	552	65	121	506	792	634	590	634	401	108	54	134	127	186	117	161	95	78
203389_at	191	61	267	255	459	758	201	309	226	355	364	325	61	75	96	65	47	46	123	175	116
203390_s_at	357	358	476	563	598	480	474	544	425	734	691	581	230	244	324	196	246	179	316	283	346
203391_at	1089	1124	1181	985	1337	1029	990	862	1331	1452	947	703	1466	1159	1171	1517	1801	1064	1165	2432	1374
203392_s_at	3114	4428	2345	1729	1293	1572	4093	3072	3073	3592	2494	2051	1585	1409	1745	3311	2843	2169	3434	1778	1417
203393_at	13	15	11	22	43	40	11	13	13	17	10	21	2	3	2	2	2	8	27	4	15
203394_s_at	607	554	414	732	521	314	537	602	590	746	890	738	3226	2896	3821	470	387	392	1682	1623	1209
203395_s_at	689	298	589	704	783	439	478	445	458	742	807	735	1883	1599	2136	539	505	661	1498	1304	1277
203396_at	3178	3106	6331	5103	4775	4377	5550	4802	5702	6531	4762	4005	9130	10306	6424	8609	6913	8313	9344	6326	6364
203397_s_at	365	622	1266	1764	146	284	380	495	550	783	112	78	76	53	224	573	562	642	1810	1832	2043
203398_s_at	139	10	183	107	132	203	15	123	131	84	108	88	11	45	37	12	29	17	71	39	45
203399_x_at	77	51	84	54	43	316	86	75	60	87	185	101	85	96	121	3	4	14	37	4	5
203400_s_at	75	57	76	106	12	42	98	103	56	108	64	62	2	3	7	2	5	1	1	2	2
203401_at	1312	734	532	252	1346	1329	937	1013	681	861	968	1213	2161	2061	2384	2727	1807	2419	3274	1339	2378
203402_at	185	470	262	433	260	249	270	284	446	266	156	145	42	138	197	115	112	96	217	260	179
203403_s_at	1904	1867	1997	1586	4273	5666	1632	1510	1634	1595	2969	2514	6112	5415	6776	3776	3326	3190	4217	4509	6513
203404_at	12	54	20	17	37	36	69	100	99	43	107	23	39	32	35	4	3	2	24	36	63
203405_at	2745	1461	2400	1619	4547	3365	2453	1770	3741	2901	3159	2921	6727	4867	4359	2595	2297	2454	5636	3644	3030
203406_at	1594	1510	1877	2162	1832	1658	2425	2578	1927	1996	1661	1684	1699	1331	1712	2603	2117	2585	2272	2003	2749
203407_at	837	1951	2614	2669	2361	3323	542	724	1449	2383	2079	2109	1374	1688	2442	1328	2005	985	1648	3122	2983
203408_s_at	370	603	1873	3259	103	212	582	550	1370	1385	122	55	40	6	14	279	297	126	2474	1879	2310
203409_at	530	710	334	326	786	281	553	790	277	444	959	1289	1229	1187	779	746	729	616	439	256	195
203410_at	623	664	594	433	528	461	693	695	617	731	620	659	382	250	292	417	396	266	425	217	241
203411_s_at	2746	1825	5551	4422	4738	3925	3942	3009	8642	11009	7528	4644	4422	4487	4337	1599	1697	1081	3160	2060	1678
203412_at	785	692	277	335	794	845	444	527	318	379	465	344	174	173	218	165	107	380	239	200	157

203413_at	172	181	227	241	3839	1902	245	395	165	354	3337	1359	7051	10780	14133	71	104	78	43	73	10
203414_at	585	973	731	409	1735	960	1093	2179	712	1588	2334	1746	827	1367	1653	1836	2021	1217	2057	1403	2296
203415_at	1905	2523	3124	6477	1516	1098	2702	2449	4622	4632	2230	1707	3678	3046	2584	2129	2496	1828	4082	3807	3105
203416_at	263	373	463	440	465	695	530	876	423	505	243	540	84	65	68	66	37	46	129	77	125
203417_at	1132	2158	54	30	378	423	1412	1931	62	35	374	180	873	1205	1121	1899	2058	1514	114	49	104
203418_at	1790	493	1557	253	1753	218	2543	2097	2277	1508	2943	2183	1497	1574	1070	859	890	1123	1143	158	262
203419_at	245	316	435	673	496	617	272	344	603	614	453	197	190	198	211	186	185	299	313	288	279
203420_at	885	635	765	696	1020	904	791	1007	591	781	769	934	918	744	908	1405	1099	929	1138	1380	1546
203421_at	40	178	119	890	44	42	64	92	233	141	20	35	14	13	15	64	79	70	93	101	50
203422_at	634	599	1245	576	793	211	779	1190	1452	1298	885	770	686	629	412	290	267	304	769	397	375
203423_at	2471	1769	176	181	16507	12082	2696	2192	178	126	21724	26075	19823	27098	27076	4360	5285	3024	76	337	240
203424_s_at	12	11	52	71	38	30	17	54	12	42	18	9	25	4	15	5	2	9	3	6	3
203425_s_at	123	103	109	147	361	453	114	42	127	115	98	114	37	66	39	41	31	73	63	47	37
203426_s_at	118	78	56	135	407	516	25	106	112	134	95	40	32	41	35	49	14	11	29	27	26
203427_at	1212	962	1011	1152	510	648	844	1003	783	723	577	597	774	790	801	1468	1202	1284	1775	1533	1962
203428_s_at	1263	1221	1282	969	1225	860	1468	1280	1420	1065	1020	933	1129	1528	1097	1346	1229	1241	1459	973	1222
203429_s_at	664	552	1263	601	1057	1840	461	664	763	1521	502	666	1201	986	532	1074	998	1032	2652	1670	2416
203430_at	2709	4820	2904	4016	1809	2070	2096	2567	2796	3079	2096	2072	2440	2382	3611	3507	3435	2953	3656	3732	2431
203431_s_at	677	850	1396	1750	764	417	648	841	2146	2507	627	338	522	661	1837	469	427	376	2926	3225	3861
203432_at	2182	815	2750	1223	1645	531	2694	2322	3041	2330	1870	2035	2466	2729	2934	2714	2066	2668	4582	2410	3796
203433_at	974	679	1152	1102	929	1047	649	649	859	1407	711	764	741	674	888	577	724	286	950	505	316
203434_s_at	12	9	9	16	30	16	9	11	8	8	6	5	2	6	6	9	4	6	20	39	86
203435_s_at	33	15	53	35	36	42	42	36	37	43	47	33	4	7	62	17	12	5	10	71	62
203436_at	1110	737	1129	945	1199	1136	1406	1304	1266	1106	2062	1633	2283	2960	3055	1966	1787	1767	2026	1250	1102
203437_at	869	760	537	339	1321	868	754	592	658	423	1170	1068	1599	1197	864	742	583	710	472	390	269
203438_at	582	2286	16	26	1057	6175	574	186	19	27	952	860	849	979	1209	471	393	774	18	263	357
203439_s_at	823	1771	243	202	755	3146	745	384	155	211	622	381	493	244	155	125	95	170	57	104	59

203440_at	28	46	7	98	111	12	29	15	25	10	13	14	11	2	6	27	5	24	4	1	3
203441_s_at	98	92	138	107	91	230	159	92	171	88	117	53	19	60	13	52	71	2	42	42	24
203442_x_at	941	927	715	1344	329	544	1140	1140	967	1331	637	240	128	115	242	172	239	179	443	357	240
203443_at	110	33	60	75	33	24	146	218	199	154	118	153	27	18	27	37	12	3	34	8	10
203444_s_at	265	150	52	36	49	40	209	158	124	25	151	16	87	82	96	23	28	9	108	39	70
203445_s_at	1091	1187	2376	3407	1524	1519	1193	1590	1584	2201	1650	1592	926	658	731	947	851	718	1092	1589	2038
203446_s_at	480	738	1164	1331	1084	1295	749	722	990	1027	1202	1204	653	435	543	364	358	294	822	503	733
203447_at	856	725	997	1151	1476	1275	1138	1521	1497	1630	1413	1146	1494	1488	2468	1858	1715	2007	2221	1559	2378
203448_s_at	594	760	465	385	491	254	725	950	387	327	482	300	469	435	462	875	751	1129	731	486	868
203449_s_at	812	783	431	495	586	347	784	911	300	295	428	494	496	547	542	1118	1072	1082	756	597	799
203450_at	905	573	520	675	917	993	1100	800	412	505	571	562	460	480	615	951	851	717	376	449	303
203451_at	307	248	224	216	201	163	542	496	241	259	296	186	120	150	125	140	99	99	209	152	99
203452_at	172	386	353	50	106	70	197	153	149	295	166	29	145	79	21	60	88	9	55	69	12
203453_at	2813	4067	2427	2497	1095	512	2308	2059	2092	3128	2342	1518	1058	1656	1961	4535	5580	4105	3419	5205	5464
203454_s_at	2057	798	1119	1247	1890	513	1256	1134	1062	1117	2389	4265	7217	4213	3814	996	1091	1057	1003	786	479
203455_s_at	3768	4547	1510	1897	2871	7054	2023	5569	593	546	2714	3088	4598	4508	5937	8294	7932	7498	4497	5889	7112
203456_at	388	698	133	312	426	665	466	942	142	231	995	1253	329	389	483	465	535	464	60	102	78
203457_at	180	180	300	326	247	558	231	243	136	319	307	230	89	80	96	140	157	134	146	123	107
203458_at	1711	1556	1443	1918	1155	1310	1830	1384	2002	1379	1428	1135	1207	659	589	660	645	589	938	543	391
203459_s_at	751	358	301	354	862	914	465	484	329	224	527	474	476	501	543	427	486	428	251	293	307
203460_s_at	675	605	826	881	1234	1138	538	889	573	943	1578	1206	1438	1642	1268	1001	1183	981	1174	1272	1530
203461_at	163	145	104	116	212	219	85	166	171	161	132	94	33	27	13	45	32	23	39	37	13
203462_x_at	4769	4899	6147	3641	4319	4260	6232	4943	9224	7188	7376	4360	5306	4562	3947	3032	3002	2476	4204	2927	2721
203463_s_at	54	152	71	31	139	242	166	86	31	45	41	21	90	76	132	173	133	133	77	49	55
203464_s_at	618	326	353	269	45	199	420	426	375	289	284	352	171	186	145	195	183	211	182	98	149
203465_at	2286	1279	1012	535	1354	1003	1020	1114	868	710	863	569	2406	2445	1761	1969	1326	1955	3343	1437	1697
203466_at	464	192	784	579	78	539	435	317	625	597	577	489	575	647	432	264	366	292	527	435	331

203467_at	400	373	217	1261	609	534	433	520	159	377	284	375	353	362	239	501	368	362	332	338	329
203468_at	423	411	109	162	631	644	103	102	134	143	11	18	120	179	105	287	260	292	219	147	140
203469_s_at	376	682	156	275	246	392	126	344	124	64	43	21	118	79	58	194	179	179	179	59	64
203470_s_at	204	40	138	81	67	118	44	78	71	45	54	36	29	40	61	61	17	17	67	67	5
203471_s_at	200	74	191	200	555	116	237	208	91	221	237	196	45	74	94	18	41	40	27	34	31
203472_s_at	12	6	11	8	27	36	23	15	24	13	13	8	16	19	6	6	29	13	6	23	3
203473_at	132	122	180	222	154	220	207	94	138	177	119	104	61	70	59	73	50	61	86	17	27
203474_at	101	78	541	380	1388	882	143	82	670	1409	1330	1399	1618	1553	1291	56	27	50	1660	1142	1828
203475_at	136	26	91	63	34	22	133	50	139	148	114	106	28	13	37	34	28	11	44	61	36
203476_at	1864	4374	1976	3491	1879	1746	1627	1965	1559	1649	2086	2022	1377	2030	2561	3024	3235	2504	1464	1735	1953
203477_at	8	38	54	17	49	40	56	62	52	50	16	46	2	18	18	9	4	8	10	15	25
203478_at	7094	6681	3409	2152	2382	1992	7798	5348	3691	1681	3353	2262	5733	4824	4093	7354	7406	7600	4216	3400	2363
203479_s_at	102	56	58	93	45	60	136	306	139	117	85	24	36	101	13	75	94	86	53	46	84
203480_s_at	905	886	932	755	1299	1451	743	1131	836	654	1093	868	1014	1169	1252	1651	1558	1470	1450	1266	1560
203481_at	308	364	145	177	386	497	425	689	123	175	337	455	181	179	172	294	242	267	148	150	160
203482_at	372	322	309	380	858	706	521	673	284	403	507	582	365	400	329	277	285	244	158	234	219
203483_at	25	21	27	31	30	11	13	24	13	21	20	29	7	8	42	6	3	8	15	22	2
203484_at	2473	2066	4141	2910	5321	8853	3967	3729	3381	3125	5261	5354	7894	7583	6013	4262	3636	4003	2713	3727	2595
203485_at	22	88	41	67	87	23	11	15	7	55	37	9	20	2	5	86	76	68	2	17	20
203486_s_at	605	547	735	1291	772	1238	708	680	718	1040	1039	753	591	476	425	368	375	364	573	432	573
203487_s_at	284	190	247	413	591	560	245	259	277	233	286	282	303	407	376	417	402	407	440	603	771
203488_at	582	481	294	365	415	519	767	512	446	275	313	190	81	53	153	173	256	189	137	155	129
203489_at	2119	1557	1020	918	1501	223	2060	1821	1106	1131	2416	2339	4668	2716	1133	776	776	719	993	617	409
203490_at	122	281	563	506	356	63	260	75	603	717	708	93	218	195	304	208	246	200	374	176	235
203491_s_at	206	78	471	261	543	461	178	193	421	435	397	481	724	750	514	273	316	207	673	1115	1198
203492_x_at	672	266	1552	1350	1069	1337	570	608	1084	910	1110	905	910	1080	1631	610	604	636	1450	2040	2838
203493_s_at	331	234	867	520	810	1074	224	296	436	489	452	524	940	544	475	396	247	410	1841	1715	2953

203494_s_at	598	654	1381	1438	1116	1480	343	404	733	808	613	827	1204	1586	2624	1591	1343	1671	2699	4650	5835
203495_at	455	405	88	57	491	500	125	161	40	25	53	69	101	102	119	145	158	193	123	101	86
203496_s_at	13	21	119	27	26	19	126	51	83	138	162	73	214	67	98	82	59	72	122	139	133
203497_at	680	736	1252	1033	1359	1352	749	860	967	1041	1001	1115	1611	934	518	330	224	277	693	555	412
203498_at	146	481	121	113	129	145	237	249	128	150	163	173	16	4	26	91	108	68	19	18	14
203499_at	2621	1255	2332	587	1398	887	1529	1412	1430	2785	2717	2110	1785	1626	846	1163	1474	1879	606	571	622
203500_at	340	442	678	416	517	441	325	238	535	418	416	264	381	320	411	327	265	299	423	344	375
203501_at	153	191	9	54	38	105	86	67	91	17	23	11	20	20	2	12	34	26	30	4	15
203502_at	274	139	165	228	216	250	208	212	111	205	188	150	127	155	120	204	189	203	237	170	221
203503_s_at	340	562	578	495	443	323	947	1060	728	695	574	395	117	144	241	194	232	189	200	200	171
203504_s_at	224	165	163	458	213	249	155	152	122	176	145	136	29	65	31	48	58	48	90	177	251
203505_at	5	15	65	295	21	200	27	9	59	55	45	8	19	24	2	45	48	44	130	397	395
203506_s_at	455	765	555	962	389	421	457	512	551	702	605	358	110	126	220	180	187	176	200	194	172
203507_at	17	35	22	150	25	31	21	50	52	13	18	23	5	4	4	12	31	7	11	6	11
203508_at	17	10	92	36	1603	1046	48	21	154	102	1589	757	1812	2023	1075	15	10	9	153	161	157
203509_at	1302	1181	1435	4007	2033	3024	901	1032	1175	1139	1451	1656	642	514	578	477	430	354	870	1748	1506
203510_at	5439	4623	7124	4208	280	629	1998	4080	4738	6777	220	346	226	273	335	6569	5979	6339	10755	9826	14938
203511_s_at	1728	1827	1610	1236	1669	1555	1703	1569	1897	1915	1700	1159	2110	1227	1014	1114	1071	915	1036	653	557
203512_at	749	1165	732	1072	796	1070	680	994	1218	1460	874	1116	1253	970	920	852	756	662	1093	1457	1518
203513_at	1128	1264	1297	2903	1087	2524	998	1116	1032	1673	659	909	703	537	953	1088	1038	1100	2182	2725	4522
203514_at	341	446	343	489	936	919	431	544	395	491	573	460	390	461	525	282	299	213	248	275	275
203515_s_at	638	572	610	495	969	802	632	541	557	581	732	622	971	726	467	492	567	371	608	594	374
203516_at	447	335	233	321	509	568	448	387	312	211	553	476	183	276	133	81	125	70	12	83	43
203517_at	1699	1780	1378	1194	1042	1200	1777	1413	1605	1406	1300	1360	2177	1861	2879	2363	2320	2388	2832	1707	1763
203518_at	115	137	383	331	232	340	118	197	90	123	82	153	35	33	66	122	116	105	105	329	329
203519_s_at	1044	558	2039	2279	859	611	902	928	2115	2169	701	640	543	644	728	1392	1324	1220	5102	3378	4795
203520_s_at	36	119	65	101	478	331	262	323	166	141	416	408	157	142	165	175	131	126	98	117	193

203521_s_at	445	208	260	420	498	347	697	595	367	429	674	358	246	308	363	352	273	348	267	270	264
203522_at	380	786	410	982	650	782	325	329	464	901	762	594	703	485	291	241	152	112	287	369	198
203523_at	16	12	20	19	69	40	17	16	17	18	11	18	2	6	12	9	5	13	3	32	5
203524_s_at	1732	1478	1453	1631	925	982	1385	1287	1019	1227	968	882	503	639	416	579	529	720	487	615	373
203525_s_at	278	358	426	362	627	735	216	231	328	215	334	259	512	290	259	436	320	534	993	934	1196
203526_s_at	151	157	376	277	371	316	359	284	363	305	303	227	105	90	60	109	77	135	189	101	194
203527_s_at	16	19	90	47	37	38	20	25	32	21	23	19	64	35	35	80	57	9	146	104	107
203528_at	314	462	233	634	517	270	248	479	262	439	600	542	268	226	81	142	96	154	163	113	101
203529_at	1533	1358	1978	2120	2873	2681	1897	1833	2380	2247	3003	2164	3261	4722	8046	5218	4817	5001	4900	4672	4354
203530_s_at	441	498	551	833	470	977	553	409	788	658	1131	847	1122	654	976	215	281	151	631	712	558
203531_at	1146	1410	1803	1855	1299	1978	1132	1083	1486	1368	1253	882	2347	1185	2394	3040	2774	2485	4125	4119	5640
203532_x_at	119	178	377	211	161	86	201	245	463	303	297	191	127	151	141	177	184	131	193	87	124
203533_s_at	171	90	198	260	274	511	89	164	72	147	114	131	305	231	178	206	205	214	410	274	297
203534_at	2474	2132	1312	1425	1883	1152	2014	1825	1869	1227	2101	1178	2729	2483	2061	2268	2666	1906	2368	2696	1372
203535_at	101	66	90	36	77	51	32	38	74	46	67	39	4	6	7	6	17	3	16	4	3
203536_s_at	860	658	884	605	569	453	798	574	1308	991	1168	1117	928	419	519	210	252	175	720	513	355
203537_at	1171	1189	945	534	841	775	946	1082	764	797	847	757	832	1063	1540	2068	1976	1651	1152	1103	1483
203538_at	1058	1502	1196	1066	1245	1144	860	995	796	1010	1906	2126	2252	1555	2016	1080	1101	959	1296	1710	2508
203539_s_at	15	11	12	12	12	17	17	13	12	14	10	14	5	13	9	2	2	57	6	3	1
203540_at	22	57	19	18	19	20	28	69	59	129	18	25	3	3	2	7	6	3	6	40	6
203541_s_at	112	111	90	34	172	16	110	123	74	117	85	122	43	11	9	4	47	30	42	30	25
203542_s_at	75	63	49	106	208	142	110	179	83	71	301	211	96	63	64	30	18	35	18	34	38
203543_s_at	22	35	60	31	197	261	36	26	1	4	156	150	108	98	48	33	20	51	1	41	18
203544_s_at	1429	831	761	539	704	782	798	953	633	889	532	584	666	702	696	2134	1914	1593	1960	1216	2223
203545_at	1146	1221	3072	2195	1687	1399	1946	1602	3630	3828	2217	2040	1928	2251	3328	1909	2093	1975	5336	3671	4014
203546_at	716	418	738	626	619	547	786	747	653	633	509	476	141	196	198	202	242	316	165	275	234
203547_at	159	103	290	371	148	275	236	190	215	275	216	216	67	40	5	23	44	8	33	75	35

203548_s_at	7	6	7	16	144	40	13	21	27	8	23	18	27	6	3	2	1	6	6	4	3
203549_s_at	187	96	138	69	382	310	131	125	87	116	168	172	32	28	44	17	23	3	12	22	3
203550_s_at	1647	1907	1335	1001	638	878	2234	1537	1737	783	1197	485	490	596	654	1982	2193	2041	1335	815	913
203551_s_at	478	611	315	407	482	519	451	360	368	368	459	359	721	709	740	909	624	872	653	799	925
203552_at	434	418	475	273	568	556	327	438	272	376	412	246	493	275	230	318	287	335	608	432	616
203553_s_at	357	292	575	310	697	736	527	598	464	632	594	452	386	249	219	352	350	330	381	271	542
203554_x_at	3844	1506	7121	1910	8971	1705	5564	6124	7817	7263	11279	13611	10131	11163	12132	6285	7306	7659	8997	2929	2937
203555_at	168	425	468	846	1023	970	464	451	602	826	833	730	390	295	460	62	90	101	295	162	112
203556_at	205	338	252	301	434	667	118	177	235	151	391	465	552	516	845	351	491	318	667	856	963
203557_s_at	2266	1010	1717	1205	1034	806	2479	1969	2614	1494	1482	1705	1347	1199	1252	1478	1189	846	1851	1894	885
203558_at	82	324	16	38	19	239	183	404	21	14	14	14	60	9	10	119	169	106	64	98	42
203559_s_at	188	124	593	2046	1326	1859	191	160	575	1284	1047	730	342	390	485	47	57	34	524	553	416
203560_at	1868	1051	673	231	3840	3412	1324	1961	330	437	2869	3245	6474	7880	9672	2303	2113	2618	1722	2886	3341
203561_at	17	29	18	89	146	215	12	71	35	60	88	108	19	28	20	22	5	1	14	2	5
203562_at	128	141	137	172	241	250	139	185	103	152	330	446	86	72	60	52	76	41	22	23	22
203563_at	105	58	38	19	3	58	27	41	48	7	23	34	22	27	18	71	73	109	39	28	35
203564_at	1560	572	1072	467	1876	739	1124	1011	1247	1171	1795	1447	1120	1404	1061	936	892	1084	1498	986	1022
203565_s_at	257	388	336	241	632	400	401	284	120	362	435	313	723	563	563	540	446	441	487	439	489
203566_s_at	1035	1075	1460	1452	986	1545	863	1062	1329	1855	763	679	1092	811	1346	1863	1317	1880	4729	2637	3624
203567_s_at	447	583	380	667	180	454	579	598	375	341	311	290	121	93	140	309	245	177	95	170	208
203568_s_at	389	554	414	614	401	414	408	521	305	275	307	293	185	137	125	316	196	405	175	255	253
203569_s_at	646	837	765	841	1515	1555	640	740	515	633	1091	935	821	678	1440	880	757	760	913	1472	1502
203570_at	486	630	30	22	62	38	427	369	11	17	505	434	342	264	224	189	227	159	35	103	50
203571_s_at	2475	5146	15	12	588	618	2793	4513	9	13	644	924	1274	1711	1345	4659	5666	4678	6	67	6
203572_s_at	1236	1736	1540	1573	1502	1837	1260	1397	1429	1465	2751	1906	752	764	827	657	886	843	754	480	670
203573_s_at	524	453	216	270	242	355	551	429	241	307	244	210	163	159	173	379	365	427	193	164	238
203574_at	1160	3086	564	596	643	1355	1187	842	432	365	519	503	502	405	704	2242	1997	2763	933	1517	2033

203575_at	1123	912	591	1121	1115	950	753	942	668	822	600	735	732	935	1199	977	1041	849	757	2399	1910
203576_at	1004	1345	1317	1419	1703	1325	867	825	1279	976	1126	1171	960	750	769	605	726	577	1504	1050	804
203577_at	491	557	290	300	375	380	428	387	300	306	314	253	489	337	226	429	423	533	309	285	238
203578_s_at	254	118	254	76	595	304	307	191	351	271	506	158	205	233	403	141	127	171	146	172	156
203579_s_at	374	217	518	374	715	601	623	413	751	780	1134	459	302	347	367	276	193	241	303	268	317
203580_s_at	541	522	1146	779	1649	632	914	813	922	1755	1518	1386	663	517	931	462	632	591	1315	1249	1210
203581_at	1703	2151	845	1432	889	1121	1000	1174	1275	875	827	832	1017	1018	1209	2307	1897	1490	2784	2281	2270
203582_s_at	1771	1600	1035	1258	484	550	1350	1391	1292	1440	1181	1080	1070	1027	718	1202	1082	671	1181	1328	959
203583_at	1618	1512	935	1001	1144	1821	1693	1473	690	872	1545	1220	2061	2065	1544	1964	1805	1637	1351	1469	1521
203584_at	685	905	562	568	685	1188	1154	1243	666	764	551	558	974	869	1085	2296	2025	2269	2008	801	1242
203585_at	496	776	1808	2821	715	1165	591	680	437	607	769	1310	349	425	392	501	601	442	728	619	640
203586_s_at	785	504	355	322	707	1278	881	619	237	312	1474	1003	531	405	287	329	321	231	87	83	75
203587_at	37	32	57	38	14	130	36	63	47	36	10	8	2	4	2	27	26	5	3	4	17
203588_s_at	517	372	254	431	728	618	701	653	343	352	890	817	511	629	819	781	875	935	313	300	325
203589_s_at	270	271	118	181	404	497	530	329	198	225	360	483	216	112	192	230	240	281	149	117	134
203590_at	152	281	967	2263	720	1238	281	277	1017	1125	1077	1170	827	672	700	101	105	75	698	867	715
203591_s_at	20	29	122	72	25	19	117	106	37	48	41	28	7	2	9	22	18	27	9	21	5
203592_s_at	463	893	521	384	48	59	413	389	302	299	344	195	110	112	205	98	212	129	83	125	74
203593_at	640	600	979	1271	954	1075	640	684	636	952	600	439	1084	960	1030	1590	1393	1383	2404	1648	2375
203594_at	2195	3100	2359	1994	1851	2807	1927	1268	1731	1575	1606	1374	2572	1992	2359	2531	2550	2301	3542	4680	5317
203595_s_at	487	865	244	217	154	301	413	521	181	122	168	146	159	85	68	1090	1305	2560	363	833	1267
203596_s_at	83	364	39	107	88	160	129	187	48	43	47	85	54	15	82	278	481	761	106	648	789
203597_s_at	325	331	285	304	364	570	354	421	278	376	610	488	1090	951	572	565	485	391	459	301	274
203598_s_at	321	214	222	116	786	1036	217	292	153	239	455	446	1174	891	655	437	494	594	710	427	666
203599_s_at	598	791	617	579	643	1223	607	907	464	735	955	1023	724	741	917	1158	1038	973	777	603	943
203600_s_at	713	1068	765	623	741	839	655	719	573	605	641	413	361	314	290	330	375	422	301	493	298
203601_s_at	22	27	37	40	52	34	32	16	15	8	168	10	11	9	7	38	47	44	45	12	27

203602_s_at	569	479	184	279	352	289	527	545	478	266	523	434	339	328	237	280	349	332	121	119	137
203603_s_at	1	27	1	10	18	3	21	1	3	27	13	3	2	2	5	1	15	2	8	3	5
203604_at	255	400	77	146	419	476	150	185	87	89	124	162	25	27	63	104	138	166	147	111	121
203605_at	980	487	1228	917	1258	1427	1579	1330	1209	1202	1397	1214	1667	1828	2224	1796	2264	1888	1783	1501	1627
203606_at	3245	2001	3582	2299	3992	3701	2317	1709	4245	3419	4311	4112	8576	7258	7701	4748	3542	3775	6841	4818	2655
203607_at	987	755	475	379	822	818	786	871	538	771	470	865	294	272	360	836	676	798	774	473	642
203608_at	319	653	106	190	247	249	262	322	58	166	402	470	634	452	537	423	483	294	350	387	630
203609_s_at	26	24	16	22	410	398	17	28	25	20	26	26	21	3	8	6	26	24	54	13	7
203610_s_at	418	251	339	402	293	234	363	435	317	232	267	239	84	74	100	265	202	237	65	71	136
203611_at	1691	1779	1097	1468	1229	1087	2449	2138	1472	1864	1727	1517	615	686	983	1698	1504	1716	906	1281	1141
203612_at	1205	943	555	431	103	71	1223	851	692	612	676	405	725	808	1059	1491	1451	1206	890	644	577
203613_s_at	4382	2102	3070	3060	2656	2590	3130	3083	2645	2820	2250	2757	3966	2704	2673	3209	2383	2957	3759	3102	3178
203614_at	856	979	948	1195	1127	1594	755	794	752	939	1070	1139	1108	1117	1866	1241	1181	1254	1478	2031	3040
203615_x_at	1556	1421	777	1906	708	1318	1583	1347	945	1177	1350	1111	1273	1589	843	1192	1139	1096	716	827	618
203616_at	1376	1841	1101	797	898	836	2141	1698	1014	843	1309	1245	1086	1246	1002	1651	2103	1726	1346	1577	1313
203617_x_at	708	671	1473	722	900	908	905	911	1232	1117	1013	513	576	460	490	360	419	293	629	359	416
203618_at	222	106	182	225	286	146	265	290	199	328	210	221	90	167	107	82	94	88	125	114	70
203619_s_at	19	6	15	13	32	32	24	24	17	36	75	25	34	27	68	9	14	40	3	26	5
203620_s_at	270	352	636	554	383	265	235	327	450	622	362	418	407	259	277	127	245	200	1143	762	982
203621_at	4232	3941	2771	5164	3682	5222	4031	3726	2363	3075	4524	5040	6630	7453	7662	5865	5545	6425	5208	4301	4754
203622_s_at	2855	2474	2395	631	2173	1510	2317	1602	2610	2071	2579	1866	2826	1998	2843	2789	2223	2546	3234	1585	1866
203623_at	327	572	506	846	131	493	305	438	491	709	367	287	104	127	124	109	68	118	125	196	125
203624_at	902	920	528	350	1013	1216	489	530	347	366	554	642	859	764	673	702	810	924	564	1027	1381
203625_x_at	1584	628	1070	1143	936	404	1040	1065	1531	1589	850	670	1199	2144	1772	2254	1583	1863	3901	2178	2989
203626_s_at	439	192	408	299	285	22	301	344	528	428	401	251	35	20	34	64	7	37	129	32	96
203627_at	110	114	601	485	616	621	138	346	654	1145	1036	866	318	234	148	47	66	96	447	432	497
203628_at	44	18	198	414	18	20	37	57	325	467	186	271	57	48	11	12	6	11	132	190	76

203629_s_at	368	339	572	361	436	1157	315	356	328	295	480	559	545	438	635	422	486	340	856	942	1396
203630_s_at	844	901	1480	1492	1725	2532	1292	1226	1001	1409	1963	1687	918	954	1333	689	955	721	1555	2082	2742
203631_s_at	21	67	5	140	60	31	89	145	104	34	80	114	3	14	17	3	5	3	34	37	13
203632_s_at	273	229	27	376	43	238	276	236	98	31	257	341	63	36	84	186	187	109	9	28	64
203633_at	1299	1470	1899	1574	2095	938	2533	2354	2080	2662	1595	1752	1128	1561	1303	860	869	1043	1636	1743	1086
203634_s_at	374	191	149	113	209	124	461	530	281	370	260	275	86	101	105	104	66	114	137	119	100
203635_at	452	439	313	451	477	534	567	553	285	239	709	430	599	422	498	628	594	408	429	585	595
203636_at	837	1130	176	220	330	433	1052	1490	178	145	604	723	628	1054	716	2218	2160	1935	121	245	389
203637_s_at	1585	1114	114	125	812	980	1236	1995	102	24	487	750	769	723	479	1687	1309	1211	116	245	351
203638_s_at	365	505	340	1588	199	1115	461	429	181	713	41	137	127	125	176	576	571	423	683	1307	1514
203639_s_at	91	91	31	616	25	337	70	32	20	210	17	11	10	3	7	39	11	36	83	123	178
203640_at	1311	2935	1839	751	988	2157	725	1339	396	910	603	747	1079	632	1003	4303	4152	3752	1504	3400	4769
203641_s_at	177	108	320	428	18	58	187	137	336	232	144	126	33	8	10	5	65	42	155	143	274
203642_s_at	148	119	269	419	206	216	127	139	237	172	78	102	125	71	112	146	130	189	1327	1352	2104
203643_at	572	350	1229	917	279	241	586	670	1296	1245	570	294	327	194	66	92	91	217	163	82	54
203644_s_at	160	137	259	348	243	74	117	104	269	351	175	263	94	103	112	108	130	71	159	143	188
203645_s_at	60	10	23	59	26	153	23	21	70	164	16	5	20	33	3	3	19	31	5	33	2
203646_at	654	648	460	242	1236	828	352	347	578	264	719	376	1781	1184	878	1103	640	1100	1248	1338	901
203647_s_at	2090	1926	1881	1650	1784	1994	2141	1684	2509	1897	2579	2510	2500	2646	3249	1959	2067	1915	2263	2176	1703
203648_at	761	731	1156	970	1101	828	954	940	1618	1220	1087	828	652	611	484	545	474	465	876	706	595
203649_s_at	16	84	161	577	460	384	171	82	78	22	63	64	46	11	48	47	38	14	259	141	126
203650_at	1090	1254	2356	1155	660	1046	864	744	1995	2250	335	368	172	218	212	955	962	919	1873	1541	1208
203651_at	298	333	221	286	579	495	305	468	231	344	338	325	333	280	344	490	425	469	547	584	834
203652_at	609	827	1266	1974	641	542	959	739	1445	1712	955	760	372	440	395	265	335	273	592	406	255
203653_s_at	544	150	256	257	49	138	315	399	233	232	186	234	351	251	113	173	214	148	173	129	47
203654_s_at	1274	527	511	577	816	871	1071	986	807	816	583	607	1052	1153	1256	1951	1526	1322	1454	937	1032
203655_at	332	148	415	442	313	364	189	331	369	365	625	299	457	356	393	184	184	209	343	299	213

203656_at	691	811	525	791	383	778	543	649	569	773	448	682	288	349	526	801	882	591	888	857	1115
203657_s_at	54	34	111	49	278	253	32	104	178	138	85	128	17	37	37	26	29	9	6	6	8
203658_at	696	564	642	895	544	738	727	648	870	636	637	674	355	393	304	338	349	294	408	357	308
203659_s_at	601	680	602	645	979	1043	986	1070	785	791	1006	1116	962	1049	1091	1242	1215	1255	1114	990	1250
203660_s_at	900	820	791	522	921	583	576	947	704	696	968	968	733	545	513	401	295	402	536	548	512
203661_s_at	294	304	239	210	329	413	362	395	211	314	277	328	119	122	91	112	106	106	71	65	48
203662_s_at	19	44	26	21	36	24	90	154	24	22	21	28	62	163	103	110	151	94	72	22	49
203663_s_at	8087	8546	9182	8364	5055	3565	6661	6734	8729	7509	5902	6497	9944	9457	7020	7795	7665	7488	11352	12153	7723
203664_s_at	583	302	906	367	1197	532	1056	759	1318	1205	1974	1441	1330	1462	1197	663	483	594	949	395	432
203665_at	759	1130	528	1060	194	210	590	400	825	702	259	376	262	212	243	477	466	397	325	122	132
203666_at	229	113	229	328	205	306	380	290	226	267	300	226	45	30	42	20	42	36	39	46	22
203667_at	5552	6142	4460	3458	3785	5850	6249	5384	4374	3873	3695	3415	8560	8007	9629	12791	12896	13462	9733	11384	7838
203668_at	468	720	513	979	444	504	382	478	569	774	294	343	108	109	96	194	142	104	261	202	258
203669_s_at	460	892	201	354	287	331	543	655	356	309	61	197	116	156	113	503	439	394	340	253	287
203670_at	147	83	103	166	250	56	146	156	28	29	60	69	4	22	53	11	13	47	9	54	5
203671_at	426	219	320	299	111	202	364	220	288	453	323	236	112	96	110	53	76	100	282	89	86
203672_x_at	426	450	924	731	632	687	628	406	801	1437	762	554	476	677	722	1037	775	318	1009	410	479
203673_at	257	146	239	191	223	155	164	236	162	179	166	50	35	12	64	36	16	55	43	32	24
203674_at	1230	1163	1389	1755	735	696	1381	1211	1449	1310	1359	1145	224	267	179	226	181	228	491	478	448
203675_at	746	410	430	321	566	2518	456	484	114	120	586	527	1022	1133	1078	2005	2160	1944	496	2111	2797
203676_at	492	428	335	661	92	603	501	726	478	581	463	349	87	70	120	113	134	158	83	129	217
203677_s_at	926	731	789	741	1293	1094	865	747	1024	702	861	695	964	655	645	574	461	466	739	527	409
203678_at	705	600	545	467	575	663	538	627	463	539	593	797	400	311	358	384	318	237	365	429	515
203679_at	474	536	326	120	566	722	382	298	293	352	526	315	716	782	467	354	297	370	312	185	153
203680_at	407	264	52	6	137	66	443	476	5	46	9	103	41	32	28	792	747	685	2	2	13
203681_at	11	6	12	13	19	11	11	15	23	14	6	15	2	8	5	2	4	4	4	4	4
203682_s_at	365	366	585	617	308	343	449	468	748	763	526	498	350	271	187	173	157	152	425	217	221

203683_s_at	370	387	492	1020	210	302	387	256	464	538	432	231	164	150	192	58	50	68	102	80	74
203684_s_at	29	24	26	56	59	32	27	17	24	24	20	24	4	6	17	12	31	19	5	9	7
203685_at	124	95	62	137	192	206	135	99	40	64	58	97	26	40	27	51	37	36	54	66	67
203686_at	525	651	846	1527	432	529	551	545	1125	1031	877	755	1137	483	391	219	223	268	926	668	466
203687_at	44	24	164	106	70	78	33	45	135	150	156	55	6	43	18	18	8	65	8	9	35
203688_at	221	454	168	125	261	474	469	834	144	264	586	390	436	619	454	672	712	633	302	279	480
203689_s_at	665	609	2356	1645	1886	1400	602	767	1734	2247	1468	2043	1654	942	1350	601	604	548	2828	2070	2124
203690_at	2115	1286	1073	543	983	861	983	966	712	948	1002	1103	1469	1679	836	2109	1701	2729	2195	3513	2783
203691_at	25	11	377	247	269	184	44	24	296	278	149	490	29	29	59	33	56	41	1101	1928	634
203692_s_at	312	94	430	406	496	351	863	512	491	584	398	566	156	139	76	120	112	107	128	102	106
203693_s_at	647	378	376	316	104	90	607	628	336	383	394	366	576	310	468	804	582	895	904	914	1117
203694_s_at	1396	1105	917	861	1199	632	1165	1040	812	820	1170	768	967	893	831	1002	912	1217	738	638	585
203695_s_at	516	1111	99	164	212	189	505	647	124	105	115	127	25	23	33	924	1006	687	51	24	56
203696_s_at	1283	516	762	354	1282	379	957	957	957	819	1531	1968	3799	1904	931	569	398	523	713	408	302
203697_at	94	2	8	66	109	62	69	70	75	99	72	45	12	12	18	23	19	34	4	14	16
203698_s_at	4	7	3	25	41	176	8	32	3	3	10	4	3	5	21	33	28	4	6	26	17
203699_s_at	87	80	69	22	22	125	28	21	71	28	20	25	5	36	10	5	5	35	24	14	27
203700_s_at	106	136	203	228	44	191	175	193	151	184	148	90	11	22	15	12	23	7	42	32	5
203701_s_at	1036	999	444	319	309	250	784	570	514	437	500	298	375	367	311	414	455	634	421	331	265
203702_s_at	95	80	492	199	139	532	293	34	450	285	641	481	209	176	277	216	255	221	309	207	221
203703_s_at	251	176	385	316	312	509	223	224	355	300	263	231	77	44	65	66	56	61	118	80	80
203704_s_at	874	740	813	1088	1014	953	900	870	740	732	966	1009	889	432	677	413	464	410	1843	2154	1810
203705_s_at	545	611	305	428	668	640	480	666	246	562	617	583	777	843	521	333	361	246	263	163	229
203706_s_at	393	689	450	415	761	1429	372	738	281	654	932	887	1133	1170	1098	467	409	391	589	285	535
203707_at	939	729	1104	762	679	453	839	623	856	767	847	461	367	514	478	763	763	715	665	743	739
203708_at	1973	1400	8	26	23	13	1434	2135	8	11	14	16	42	13	10	2073	1817	2161	3	2	3
203709_at	443	412	354	723	77	90	388	339	532	444	414	315	236	275	107	109	109	106	191	106	85

203710_at	107	215	11	243	23	86	135	129	43	89	23	74	32	7	4	78	60	86	67	67	189
203711_s_at	320	152	467	512	500	587	309	432	411	481	259	369	801	784	641	646	401	515	1755	762	844
203712_at	1708	743	1968	811	1353	1252	1668	1198	2545	2436	1471	1062	1501	1199	1490	1247	1004	1381	2160	1360	1604
203713_s_at	753	1029	594	966	744	1681	727	846	668	693	409	396	249	257	330	452	438	427	294	373	363
203714_s_at	1130	921	751	572	504	1355	823	830	673	802	517	845	645	837	880	1318	1103	1205	1438	1828	1774
203715_at	298	209	343	150	187	511	266	297	249	292	138	246	305	252	161	269	229	243	311	380	350
203716_s_at	12	15	33	278	15	16	24	12	111	496	7	9	6	2	2	8	2	25	223	338	387
203717_at	148	384	353	779	364	269	224	165	260	544	23	111	27	12	3	43	57	72	421	497	491
203718_at	1103	1819	414	683	495	552	1308	1112	205	587	701	210	113	166	152	150	201	218	81	137	112
203719_at	573	748	1175	1502	900	1013	718	537	615	1178	1107	1078	1266	1507	1613	1127	1182	768	826	1629	892
203720_s_at	951	757	2005	2275	893	900	1007	1013	1054	2120	1778	1641	1869	1252	1081	788	750	653	683	1188	613
203721_s_at	3905	3173	2092	898	3065	1924	3432	3017	2438	2488	2942	3161	4675	4352	6862	6076	5678	5471	5839	3959	5209
203722_at	344	506	517	740	81	161	260	384	314	456	308	244	175	111	88	95	90	103	116	126	106
203723_at	198	63	128	119	412	636	186	38	71	32	438	55	284	219	346	73	99	17	77	56	62
203724_s_at	50	77	60	10	22	124	46	67	84	57	43	38	49	48	40	62	51	58	113	49	107
203725_at	2437	5131	1359	1314	1447	4398	1113	2272	648	876	1792	2291	2651	2599	1800	2542	2758	3501	1244	1324	1198
203726_s_at	89	442	1690	1336	1161	1165	38	36	538	809	1165	1102	371	353	616	79	82	114	229	708	608
203727_at	868	794	1205	1254	816	1117	757	806	1143	1262	1312	755	345	236	345	145	241	249	283	231	209
203728_at	487	214	369	295	91	73	551	376	189	312	424	231	133	98	57	149	103	93	67	86	46
203729_at	1994	1018	404	164	1643	2146	1469	1130	181	150	5132	1832	1320	2528	1692	2085	2350	1965	226	799	357
203730_s_at	336	114	278	224	731	841	318	326	312	316	702	597	365	271	259	182	167	190	161	166	175
203731_s_at	303	215	277	428	1159	731	274	354	372	422	310	385	308	231	212	199	152	163	208	157	76
203732_at	783	872	773	892	1010	1070	885	839	834	703	894	856	800	707	969	998	1087	920	777	901	1133
203733_at	925	875	536	793	830	637	1040	1007	874	887	681	469	502	358	331	719	758	672	596	403	277
203734_at	597	673	307	603	1181	1326	913	862	462	491	1501	940	747	621	376	520	471	396	224	189	201
203735_x_at	1140	549	860	810	2885	2412	745	994	815	978	2183	2656	3324	2896	1659	1262	1337	713	907	866	910
203736_s_at	294	82	265	101	977	739	252	198	213	284	1731	1338	891	630	850	358	351	509	112	153	172

203737_s_at	1363	1120	719	253	619	366	1318	909	1159	955	431	387	372	340	268	541	486	728	484	421	502
203738_at	1148	844	1027	1306	940	1324	1120	1032	1230	1451	873	875	743	813	973	2031	1668	1589	2896	1697	2765
203739_at	204	300	605	542	1178	1365	186	228	479	633	1049	1044	1717	1063	428	466	407	568	1332	1030	903
203740_at	1896	2562	2062	1415	1375	1242	3591	3100	2486	3143	2362	1908	3212	3484	5806	5447	5771	6468	4440	3719	3521
203741_s_at	262	355	191	84	165	386	178	132	166	108	111	118	54	59	68	179	242	206	186	150	225
203742_s_at	319	242	330	300	227	177	217	351	432	415	236	314	132	111	109	146	134	120	164	190	134
203743_s_at	994	1007	987	745	462	755	786	662	1113	874	398	367	725	818	560	1408	1463	2045	2369	2382	2824
203744_at	2092	1061	4118	1173	5057	2381	3540	3592	4152	3266	4438	4487	4574	3779	3411	1532	1387	1783	3668	1572	1679
203745_at	598	747	526	560	1194	1227	505	607	385	537	1187	1096	1358	1543	1372	948	1041	779	736	632	873
203746_s_at	1169	1390	1009	1032	1901	2443	1500	1234	1363	1058	2663	2157	2264	1866	1641	842	788	727	810	748	546
203747_at	225	28	345	374	239	120	118	58	775	323	145	102	12	9	17	58	66	8	74	74	84
203748_x_at	702	1270	521	1225	825	914	738	565	673	572	1103	567	1025	1535	2220	972	1045	772	1216	1097	1251
203749_s_at	365	359	380	206	346	138	371	273	317	295	637	284	305	290	298	130	175	168	203	139	129
203750_s_at	21	100	115	191	1116	921	58	78	52	120	291	53	59	98	90	58	82	47	100	92	56
203751_x_at	1072	3258	530	835	482	468	1089	1040	217	390	455	212	82	50	31	103	77	81	20	24	13
203752_s_at	7456	15081	5438	6541	2631	4874	6781	9415	3068	5638	5059	4391	3182	2904	5497	9363	9712	8802	3327	5531	4314
203753_at	20	21	42	103	48	50	57	78	56	55	44	23	18	29	27	6	26	12	64	29	31
203754_s_at	163	260	64	96	27	71	77	189	12	75	71	87	115	93	23	134	95	55	41	22	59
203755_at	1554	594	1834	396	1072	195	2268	1915	1890	1473	1522	1230	2244	1858	1485	2221	2213	2107	3774	892	1652
203756_at	46	229	92	614	59	69	52	96	209	300	109	34	19	22	37	59	106	88	101	18	87
203757_s_at	38	39	518	167	55	67	106	103	297	426	104	25	4	27	33	5	2	21	376	365	431
203758_at	282	384	266	473	261	216	288	331	317	421	262	259	29	52	49	96	114	114	200	155	272
203759_at	114	231	240	383	254	343	82	102	316	635	219	206	149	172	149	100	119	82	97	115	66
203760_s_at	52	22	33	35	66	43	20	40	32	48	31	41	5	6	3	8	5	11	9	4	6
203761_at	226	159	81	114	433	484	155	235	257	189	273	225	59	9	53	47	35	11	64	33	50
203762_s_at	270	344	171	748	111	273	515	326	58	197	108	101	123	156	125	349	331	295	327	614	716
203763_at	311	364	228	459	275	615	229	289	126	138	91	68	193	223	231	576	459	558	391	804	988

203764_at	1080	253	1004	147	1999	181	1497	1905	982	953	1501	1315	2067	1816	2102	1743	1502	2085	2521	662	1206
203765_at	218	291	426	682	526	632	298	315	508	880	419	468	446	491	451	383	341	480	1393	630	978
203766_s_at	46	40	14	32	21	15	28	26	27	28	11	11	5	9	7	6	19	9	9	10	6
203767_s_at	74	118	90	193	36	32	77	96	91	130	44	50	10	3	2	70	74	63	94	170	191
203768_s_at	93	88	137	197	132	51	135	95	88	77	88	26	10	44	8	44	10	32	34	43	35
203769_s_at	123	88	145	257	213	24	98	61	162	191	138	131	14	23	1	54	77	68	93	132	80
203770_s_at	16	5	15	22	44	39	11	5	11	10	7	6	2	2	2	1	2	4	2	1	1
203771_s_at	315	52	15	27	422	382	325	372	15	28	544	651	1116	734	349	86	116	84	6	27	20
203772_at	9	4	23	8	49	38	16	9	8	10	11	6	22	4	26	51	6	20	21	2	23
203773_x_at	1140	620	274	451	2232	4311	1076	1134	341	317	2614	2378	3557	4505	8801	1476	2650	1429	404	466	497
203774_at	345	431	488	193	412	433	346	451	413	530	446	559	694	706	584	338	389	577	879	764	890
203775_at	287	240	743	464	1023	1187	368	442	847	1005	1411	695	2083	2202	2423	525	450	510	2406	1286	1873
203776_at	589	454	648	738	1159	1107	739	600	851	769	1022	604	1435	1394	995	496	441	461	773	962	616
203777_s_at	347	215	479	309	247	235	159	292	611	407	316	293	239	171	76	65	106	123	260	210	156
203778_at	552	584	458	726	465	511	510	619	297	479	180	320	203	195	195	351	383	452	264	518	800
203779_s_at	30	33	148	229	984	567	205	123	203	243	1546	529	506	593	480	47	6	59	122	92	127
203780_at	220	103	510	885	3572	2985	175	111	379	260	2858	1723	3558	4039	4103	286	233	291	1487	1462	2106
203781_at	2205	3044	2698	3559	2903	3548	2401	2912	3151	2075	4323	4664	5224	6176	5486	4275	3846	3917	5040	3493	2588
203782_s_at	2180	1742	977	660	564	452	2064	1557	1192	876	715	432	394	438	576	780	801	890	696	520	434
203783_x_at	142	151	206	185	40	31	249	202	239	193	153	30	14	30	5	47	26	59	32	29	21
203784_s_at	17	15	27	8	19	67	61	15	64	15	17	5	50	9	5	5	5	5	27	4	16
203785_s_at	433	241	304	36	12	16	390	399	543	355	333	217	352	211	85	66	136	121	132	120	127
203786_s_at	3499	7245	3349	5716	2109	1660	3676	3189	3875	3115	2436	1854	2313	3117	5926	4878	5732	4733	3271	4072	3520
203787_at	97	69	9	84	40	55	66	86	13	8	34	6	34	18	38	59	82	101	86	129	146
203788_s_at	230	182	309	188	381	176	171	228	385	546	179	194	31	43	48	60	28	73	99	76	73
203789_s_at	307	485	1466	561	30	63	139	304	871	1384	34	5	11	8	11	1026	1277	1106	2653	1192	2664
203790_s_at	1077	994	973	982	944	821	1554	1462	696	956	976	872	1487	1646	1521	1569	1567	1858	899	589	589

203791_at	226	418	393	486	205	505	174	379	171	361	212	244	323	140	218	220	122	312	655	918	1246
203792_x_at	611	671	723	649	77	59	559	429	524	498	591	245	110	205	263	225	378	275	157	157	113
203793_x_at	716	663	850	874	432	434	754	879	756	725	604	586	282	202	195	187	193	207	199	194	104
203794_at	1252	822	1012	1357	283	332	1096	1062	631	1648	638	337	376	396	349	788	775	761	865	648	864
203795_s_at	323	500	176	338	102	180	391	375	187	200	178	142	76	33	105	202	233	231	104	137	135
203796_s_at	148	238	99	175	18	93	221	249	51	113	68	74	68	56	109	174	175	101	70	91	70
203797_at	917	569	1002	855	2617	2536	1014	955	1377	1187	2075	1184	2580	1653	5725	1889	1164	2011	2439	1667	2263
203798_s_at	205	113	403	94	759	586	363	356	542	485	723	519	1026	926	1533	422	212	480	522	385	528
203799_at	11	4	289	735	411	278	32	45	300	376	314	336	446	353	419	3	6	22	768	782	1164
203800_s_at	1386	1757	2378	1909	2201	1707	2254	1741	1713	1639	1578	1962	1627	1428	1804	1601	1532	1591	1203	1373	914
203801_at	146	146	96	301	275	314	167	240	181	298	223	274	223	223	246	182	136	206	344	300	426
203802_x_at	1558	2113	1465	1401	1555	1023	1442	1174	1596	1092	1562	1453	1995	1572	1522	996	1074	1025	1126	1263	1302
203803_at	172	94	164	406	473	458	168	318	165	338	509	736	904	745	459	182	154	140	428	320	374
203804_s_at	3151	4289	2178	4661	3175	4419	2021	3287	2543	2634	2264	1776	2323	1932	2276	3732	3241	2730	5136	3485	4847
203805_s_at	1011	327	476	414	379	329	921	720	657	847	513	660	237	327	532	368	430	450	393	180	171
203806_s_at	267	187	197	58	319	172	294	406	203	228	75	84	90	31	70	135	235	139	170	91	46
203807_x_at	11	11	9	10	33	56	7	7	8	7	6	9	4	2	5	23	5	6	2	5	3
203808_at	140	111	156	122	173	86	106	140	120	137	119	123	11	30	64	44	46	35	30	37	29
203809_s_at	238	252	369	402	280	156	318	300	237	307	328	239	19	52	44	2	20	25	17	18	28
203810_at	224	294	265	272	282	339	232	330	126	243	138	239	266	216	278	489	561	582	386	408	590
203811_s_at	103	167	236	198	106	204	276	331	162	201	119	256	166	99	101	203	238	249	240	162	436
203812_at	197	47	179	137	109	581	213	205	237	254	171	137	50	71	93	75	52	38	88	83	31
203813_s_at	41	18	26	26	78	28	24	18	23	8	10	29	5	34	5	5	1	6	5	3	3
203814_s_at	2782	1810	734	379	1046	1220	2278	2097	884	918	1047	1180	1204	1291	1179	1788	2085	1444	667	690	479
203815_at	24	16	496	1911	323	478	17	21	583	815	667	628	620	796	608	7	14	4	679	518	263
203816_at	524	191	477	409	432	262	437	530	372	414	532	705	1010	801	561	658	665	672	721	543	475
203817_at	69	94	92	132	132	198	34	70	50	28	78	84	37	30	4	15	29	21	17	21	18

203818_s_at	520	366	956	705	1390	942	925	777	1588	1294	1244	919	1527	1485	1205	1248	1108	1025	2293	1521	1703
203819_s_at	804	265	1141	1072	653	563	1473	1482	958	2126	944	921	1674	1870	1227	964	1107	1082	1595	1776	2459
203820_s_at	1630	558	2485	2124	1563	1666	1601	1461	1670	3549	1541	1031	1578	1334	990	1246	1788	2076	3130	2995	5063
203821_at	448	297	439	34	151	132	117	211	239	790	333	413	272	267	318	271	311	356	280	468	498
203822_s_at	480	432	690	738	878	861	631	706	680	693	584	636	460	317	333	601	593	537	716	485	767
203823_at	540	376	446	303	518	876	558	412	528	358	664	408	296	232	310	333	276	332	275	359	236
203824_at	93	150	9784	25549	225	318	265	187	11989	13634	104	126	89	83	80	5	26	9	15879	18209	16931
203825_at	679	1794	1076	1276	998	1114	1134	1855	886	1556	1310	802	914	979	478	676	614	527	1252	1160	992
203826_s_at	448	847	567	895	118	242	500	507	425	569	372	268	144	128	83	163	168	162	210	170	135
203827_at	405	394	302	189	668	1294	396	595	140	169	286	357	512	468	304	475	485	351	95	356	417
203828_s_at	13	78	15	19	29	71	17	12	9	11	685	216	4	6	17	12	10	22	8	52	13
203829_at	819	400	794	850	1218	1071	762	796	630	911	981	1059	1059	926	1059	328	415	386	648	565	797
203830_at	188	96	235	138	477	626	121	83	190	109	240	275	672	370	265	119	153	193	667	561	652
203831_at	541	1210	842	1042	745	1474	1055	1140	618	766	790	824	518	663	1337	1119	996	809	1250	1768	1246
203832_at	4079	1871	5024	1967	2393	661	5808	5793	9543	8088	4470	3524	7380	4114	2661	3274	3391	3341	4613	2681	1778
203833_s_at	394	319	716	1044	239	484	598	629	1058	1563	971	495	291	128	92	89	108	69	423	293	193
203834_s_at	553	463	1153	1481	642	606	974	941	1611	2101	1576	866	153	229	299	154	156	146	254	218	187
203835_at	40	18	46	18	40	82	32	34	32	57	51	36	2	4	3	3	4	8	6	10	5
203836_s_at	291	227	465	332	242	160	435	350	601	661	141	131	136	113	105	112	121	99	366	302	444
203837_at	610	513	669	451	312	284	474	405	739	875	141	175	140	112	153	344	266	314	1918	2067	2571
203838_s_at	103	103	178	260	103	28	111	124	357	356	459	239	25	8	3	3	3	2	3	3	8
203839_s_at	205	461	974	770	1523	1101	331	416	718	832	986	362	509	432	310	58	65	75	516	277	212
203840_at	177	116	170	160	125	202	69	108	87	112	74	78	153	104	100	323	211	238	362	485	611
203841_x_at	11	58	312	32	12	31	27	13	119	344	185	10	3	34	34	16	6	4	40	42	2
203842_s_at	15	27	134	85	573	658	87	18	100	14	36	18	5	33	11	10	69	40	32	19	7
203843_at	507	474	1336	1540	1105	961	637	701	1151	1644	1475	955	795	678	639	423	418	352	1249	1043	1549
203844_at	41	61	24	21	85	159	38	83	25	24	38	57	19	44	41	23	17	31	41	14	26

203845_at	130	38	170	199	166	222	117	135	195	141	190	143	173	202	98	129	143	92	232	310	476
203846_at	1304	1130	1138	1477	1025	677	1760	1496	1589	1343	1447	1051	620	760	1130	1577	1447	1359	1141	772	1460
203847_s_at	1015	978	734	667	948	775	1259	1110	1082	778	1001	519	462	488	426	559	557	571	559	241	336
203848_at	639	710	494	517	349	516	474	556	597	509	505	479	376	461	838	874	788	601	640	753	747
203849_s_at	103	6	85	131	454	456	36	140	104	108	101	108	75	104	87	45	21	36	17	4	14
203850_s_at	62	34	28	52	135	108	73	92	51	10	54	50	2	11	2	2	14	11	9	27	2
203851_at	1300	1918	3314	5447	2606	3147	1439	908	1686	1909	4056	2831	5773	4510	1614	632	1104	1033	1052	1634	741
203852_s_at	1371	836	544	212	1016	763	708	790	352	429	412	645	1177	1038	693	1636	1310	1603	1102	925	759
203853_s_at	52	95	595	921	216	586	76	53	355	375	252	131	224	129	210	160	130	88	675	707	616
203854_at	25	38	54	57	124	81	60	20	60	110	64	50	5	28	22	23	28	20	22	29	5
203855_at	253	292	233	379	382	481	351	376	181	295	401	450	276	153	244	316	394	328	271	267	421
203856_at	958	471	642	336	970	222	1097	1577	868	794	863	1066	1793	1744	1732	1455	1265	1785	2160	719	846
203857_s_at	1656	1360	1791	922	1787	1467	1662	1704	1765	1817	1587	1318	879	1207	948	1401	1596	1559	1048	1298	1379
203858_s_at	1699	1156	1407	1178	1445	1236	1532	1482	994	1185	1524	904	316	552	1035	779	605	845	791	519	676
203859_s_at	402	444	130	176	503	228	437	570	334	266	397	220	52	74	22	226	28	228	14	50	10
203860_at	216	165	258	219	925	980	285	248	227	210	259	241	823	934	1381	391	330	369	819	1376	1411
203861_s_at	17	28	14	21	25	22	15	13	15	13	36	21	2	13	4	4	4	11	6	2	2
203862_s_at	11	6	15	17	85	46	52	20	12	42	6	13	1	4	1	20	6	4	21	11	2
203863_at	40	19	37	49	47	36	52	25	19	84	17	54	11	9	14	9	4	18	13	6	5
203864_s_at	333	197	301	272	349	281	249	298	239	254	220	190	47	20	7	40	32	29	70	37	35
203865_s_at	130	210	9	185	210	435	113	210	45	138	290	457	244	208	264	156	110	95	95	185	161
203866_at	340	109	297	185	205	85	295	326	589	443	274	337	91	118	150	107	100	134	190	197	143
203867_s_at	943	828	951	220	436	535	1120	653	1329	1142	578	338	478	474	461	780	641	767	827	483	416
203868_s_at	143	102	77	88	181	168	117	78	80	145	30	111	17	27	3	28	26	20	11	14	17
203869_at	520	316	288	290	500	271	734	730	500	293	399	227	361	358	287	1063	918	622	732	368	464
203870_at	979	758	374	475	421	546	531	765	219	263	273	256	423	347	387	1464	1370	998	554	538	562
203871_at	369	257	444	292	595	425	598	321	1053	637	1369	583	805	304	396	108	313	332	474	337	291

203872_at	33	96	27	17	36	172	21	86	21	21	87
	13	14	24	44	43	42	52	24	30	24	
203873_at	79	141	119	124	147	97	196	156	198	278	195
	124	27	39	22	64	25	48	51	6	22	
203874_s_at	1255	1133	1276	2152	2306	2352	933	1167	1177	1293	1934
	1164	1287	1564	2685	2295	2251	1869	2227	1311	1616	
203875_at	582	1832	581	1984	1255	1176	431	1202	741	697	911
	1017	1277	999	992	1427	1622	1124	1199	1421	2101	
203876_s_at	79	180	180	72	59	20	187	216	78	38	104
	92	5	17	44	6	5	11	4	4	8	
203877_at	13	27	16	31	40	46	42	38	27	25	24
	25	13	4	6	4	3	9	4	6	9	
203878_s_at	266	470	84	181	60	269	295	459	140	172	148
	163	86	101	78	140	160	126	95	64	80	
203879_at	385	459	129	278	487	882	405	391	149	158	412
	390	73	102	189	173	200	161	48	91	84	
203880_at	2731	1123	2683	1984	2152	2458	2022	1779	2360	2534	3063
	2746	3989	2985	2033	1679	1762	1725	2633	1582	1028	
203881_s_at	26	6	50	30	102	19	4	7	5	4	30
	30	11	14	2	2	1	14	9	1	7	
203882_at	340	939	402	835	236	313	203	340	351	453	207
	270	98	49	102	212	735	996	155	954	836	
203883_s_at	405	485	411	221	513	634	755	772	286	219	341
	315	452	430	228	648	798	565	406	312	449	
203884_s_at	337	307	229	147	389	390	440	371	155	157	227
	201	239	197	215	952	859	1021	508	430	1034	
203885_at	1437	914	1613	1571	1182	1212	1150	1101	1496	1713	1336
	1086	1227	1231	1893	1780	1614	1569	2554	1742	2529	
203886_s_at	24	12	12	18	74	44	16	21	20	10	14
	15	4	18	41	14	5	6	13	27	5	
203887_s_at	81	71	66	74	230	106	66	110	36	87	37
	48	3	7	35	3	25	20	23	34	3	
203888_at	37	21	18	18	69	99	37	18	24	20	18
	13	7	8	10	31	34	21	8	3	4	
203889_at	77	34	174	94	81	17	60	103	100	179	47
	48	4	3	7	56	55	45	97	332	193	
203890_s_at	407	522	278	235	422	380	558	614	504	692	595
	403	53	44	50	50	74	53	37	34	30	
203891_s_at	296	355	118	220	760	793	179	304	170	194	249
	163	133	114	98	113	81	88	86	108	62	
203892_at	161	185	260	530	748	1325	216	304	274	319	1275
	1361	665	320	372	59	61	79	122	166	76	
203893_at	934	616	947	837	849	1119	708	813	966	911	709
	1455	1692	2023	2403	2760	2663	2691	3355	3117	3596	
203894_at	192	159	176	198	173	133	228	165	190	172	223
	238	108	144	139	191	178	186	214	167	186	
203895_at	77	105	408	653	246	447	93	75	317	672	344
	353	205	270	251	114	178	192	743	957	1303	
203896_s_at	9	35	243	313	227	301	17	33	194	319	168
	197	102	97	30	19	2	15	191	199	240	
203897_at	1572	1786	985	1120	2324	1775	1397	1292	743	721	1745
	1627	3637	4904	5437	3700	3069	2423	1552	3933	2965	
203898_at	359	207	412	507	902	701	253	212	444	407	604
	602	719	465	393	357	346	292	622	426	423	

203899_s_at	462	204	453	604	898	558	484	387	634	537	648	280	157	175	135	79	56	78	101	88	68
203900_at	173	133	114	206	355	321	150	190	135	180	131	109	26	22	11	31	25	53	74	44	37
203901_at	255	196	100	110	484	491	242	228	107	253	139	83	29	40	42	44	63	48	46	46	42
203902_at	9	6	7	109	27	23	64	9	15	11	10	9	19	4	10	3	5	2	4	16	2
203903_s_at	140	246	521	643	103	79	284	355	306	587	75	70	14	1	6	30	30	33	1215	1037	1307
203904_x_at	228	270	886	1791	624	628	313	219	1022	2380	1357	548	369	228	271	58	24	13	395	184	219
203905_at	1680	1343	1145	1201	973	1259	1676	1335	1370	1252	884	1017	714	548	743	1484	1347	1762	1273	1170	1271
203906_at	415	363	244	398	801	835	348	410	352	187	199	219	136	158	179	251	257	154	168	140	146
203907_s_at	386	560	88	309	104	222	485	449	99	164	358	192	169	95	108	223	180	149	143	122	112
203908_at	97	68	311	528	269	129	50	66	256	418	53	88	7	18	29	19	29	19	549	385	531
203909_at	788	566	943	1394	1278	1266	1219	1053	1102	1393	1330	1449	1390	1564	1057	808	901	760	1215	1188	1597
203910_at	4157	3437	6818	3028	1939	2099	2340	2902	4158	6077	1769	2092	1424	1483	2433	11598	12027	12085	7428	7483	10683
203911_at	556	900	765	563	570	947	749	895	313	823	530	540	156	95	111	119	161	119	244	509	324
203912_s_at	286	473	818	1141	319	579	245	309	653	812	490	205	299	406	407	321	292	222	917	915	883
203913_s_at	12	39	175	1835	849	804	49	100	62	101	883	834	1850	2333	1406	19	48	40	749	295	397
203914_x_at	140	119	202	2299	847	1015	140	205	213	140	649	731	1421	1740	2461	47	38	46	566	367	638
203915_at	172	119	170	155	74	285	208	179	193	208	182	163	19	8	21	33	45	24	36	63	4
203916_at	261	425	353	345	175	668	278	227	268	295	435	588	186	216	122	159	109	117	121	177	192
203917_at	1970	2918	2238	1478	4797	6145	2423	2782	1631	2890	4585	3702	3696	5527	6066	2395	2188	1784	2664	3411	4549
203918_at	417	240	703	314	433	492	254	253	296	686	439	280	112	162	104	132	143	169	212	131	126
203919_at	118	151	18	19	55	50	24	193	25	18	279	138	831	725	482	262	269	165	4	9	19
203920_at	427	384	970	2404	352	280	465	430	1109	1111	377	258	226	194	237	220	175	192	881	658	669
203921_at	218	201	118	107	359	308	134	176	48	124	198	232	119	119	69	120	100	90	7	21	38
203922_s_at	204	140	164	193	65	81	131	141	136	182	117	128	11	3	51	48	25	6	15	56	15
203923_s_at	53	85	121	81	148	175	72	107	104	129	97	33	25	18	40	76	81	39	133	67	74
203924_at	38	77	49	529	89	188	60	71	128	77	27	134	1	2	26	2	24	7	3	9	4
203925_at	3269	2985	3223	2518	3204	2479	2417	1803	2617	3126	2029	1639	3336	3069	2135	2261	1978	2117	4188	2661	3406

203926_x_at	906	585	1106	458	1230	710	1696	567	1276	791	637	436	719	347	487	561	646	588	979	871	425
203927_at	495	481	251	294	423	355	330	327	265	201	435	545	162	186	137	145	125	127	161	142	87
203928_x_at	646	517	47	283	401	203	439	722	50	27	183	214	190	321	183	196	175	149	105	92	46
203929_s_at	832	971	91	190	416	629	730	723	132	211	203	348	212	317	195	439	390	558	139	116	139
203930_s_at	156	169	85	146	54	149	176	176	108	116	84	78	29	22	25	30	6	4	4	17	1
203931_s_at	2893	3521	1725	713	1864	1156	2663	1434	2280	2857	2083	1107	3550	2498	1377	1447	1830	1365	1513	806	517
203932_at	377	455	338	312	711	590	568	472	326	373	367	329	126	164	211	207	188	181	190	219	165
203933_at	410	422	483	356	814	503	380	409	404	496	513	475	324	152	97	168	150	126	142	195	109
203934_at	83	39	45	32	274	169	23	65	108	41	34	8	17	21	4	4	19	18	19	27	9
203935_at	898	976	1120	921	987	1564	689	666	518	758	728	834	681	1013	1122	1851	1460	1567	1436	1764	1989
203936_s_at	304	203	316	292	733	434	405	377	237	352	438	455	104	80	100	75	86	46	70	85	47
203937_s_at	57	265	221	275	36	31	242	277	175	190	146	45	56	10	42	16	73	55	18	46	32
203938_s_at	590	859	220	343	44	93	560	503	468	256	175	18	174	128	141	231	316	180	302	300	230
203939_at	1394	494	3943	960	738	568	639	852	982	2595	671	2177	395	698	369	1466	2191	2016	2531	3679	4997
203940_s_at	275	290	91	43	557	527	244	273	83	73	247	180	89	71	41	249	256	94	63	71	33
203941_at	728	378	598	559	840	817	824	718	652	741	813	655	301	309	243	395	374	395	363	138	218
203942_s_at	276	446	490	604	19	48	298	211	591	553	277	106	121	100	60	101	13	78	119	117	126
203943_at	767	536	1172	2090	884	706	934	933	907	1611	992	547	758	629	615	519	593	540	1194	1655	1511
203944_x_at	661	568	582	559	778	603	600	604	633	580	678	582	281	241	305	214	228	314	238	253	238
203945_at	422	232	277	224	639	1342	342	272	341	416	837	469	278	769	972	285	268	287	313	556	401
203946_s_at	222	418	670	261	1534	3716	359	207	347	317	2415	1238	2343	4564	3731	642	926	738	550	1840	1598
203947_at	1475	1534	2048	1243	1589	1098	2238	1781	1703	1344	1420	1205	1055	232	364	710	757	531	1990	1595	2718
203948_s_at	9	15	42	18	47	26	13	21	5	38	4	24	2	26	3	2	2	4	1	11	5
203949_at	114	84	151	111	307	277	118	144	111	61	114	88	29	35	10	13	24	26	27	20	14
203950_s_at	255	101	252	208	52	93	178	164	211	193	207	201	179	124	180	129	168	149	104	174	206
203951_at	22	19	104	80	109	184	86	71	45	41	11	59	8	12	3	7	8	7	18	5	3
203952_at	332	499	506	383	195	165	518	516	309	372	424	383	428	323	233	717	649	400	355	566	714

203953_s_at	374	849	1172	4675	210	540	394	500	1601	1539	696	769	256	205	84	146	140	156	579	434	355
203954_x_at	435	663	746	3820	208	476	242	327	871	868	406	412	190	124	184	213	202	131	1035	714	566
203955_at	710	519	648	516	2245	1603	738	612	672	760	952	818	799	597	433	311	291	280	559	606	496
203956_at	603	506	465	321	921	903	550	472	502	528	679	783	1356	518	464	690	618	374	893	743	1006
203957_at	614	596	457	353	741	903	542	620	363	484	780	811	1197	1221	1159	1508	1477	1288	784	767	1028
203958_s_at	287	219	201	270	286	274	288	314	190	151	247	219	71	89	180	284	234	212	125	134	185
203959_s_at	439	631	515	755	155	484	660	527	510	228	509	550	245	253	177	276	251	350	193	222	256
203960_s_at	611	408	604	528	939	597	784	827	483	601	1182	1146	2372	2210	1593	983	1014	1226	1258	405	465
203961_at	568	1238	1205	1395	2856	3673	539	831	361	579	3081	3588	2480	3598	5629	1043	1214	966	838	4748	5967
203962_s_at	934	1232	1423	1319	4166	5824	851	1404	421	748	3712	3803	2710	3117	4129	1035	1091	838	605	2867	3537
203963_at	216	410	1318	2671	206	31	342	290	1737	3542	155	118	32	7	29	108	99	98	980	791	1186
203964_at	504	1248	642	1006	1149	1836	507	480	495	535	1352	1087	1392	1551	978	741	909	1484	1124	1453	1509
203965_at	210	251	201	295	700	603	178	84	268	193	155	194	103	118	157	122	136	97	158	120	135
203966_s_at	672	872	475	851	1347	1767	591	637	454	629	705	606	682	828	941	1345	1349	1154	1208	1521	1548
203967_at	1814	322	2155	239	2065	927	938	1159	1579	1553	2531	1733	1767	1740	1692	1887	1959	2767	2072	1238	2084
203968_s_at	1912	307	2097	375	2047	898	1178	1126	1322	1335	2001	1561	1799	1473	1419	1271	1422	1432	1340	835	1409
203969_at	136	10	16	76	43	60	82	67	80	55	88	106	32	35	70	31	25	12	69	50	58
203970_s_at	307	187	581	498	458	466	323	358	601	433	406	312	693	568	855	917	596	880	1665	1447	1984
203971_at	323	163	601	230	642	439	339	321	397	264	439	465	915	794	1086	571	780	694	942	1392	1365
203972_s_at	486	394	1457	971	792	563	1223	884	1239	832	961	765	962	736	1002	860	981	863	1466	985	1589
203973_s_at	937	1466	1143	2810	1343	470	571	544	710	653	1211	522	1542	1750	1776	539	618	513	698	729	466
203974_at	958	708	100	43	73	98	772	914	107	119	129	49	11	30	9	1442	1530	1393	4	8	15
203975_s_at	486	352	317	80	623	364	555	579	577	422	537	310	791	563	279	343	387	283	295	244	153
203976_s_at	489	360	342	31	378	40	656	521	454	300	468	212	254	310	140	145	136	151	192	91	84
203977_at	26	26	43	54	30	24	27	18	21	52	37	34	60	13	7	6	3	11	22	20	21
203978_at	605	461	399	482	558	607	746	833	704	704	550	695	816	658	609	452	514	491	652	496	430
203979_at	50	26	42	54	465	646	21	36	25	36	99	410	280	346	327	82	148	202	104	62	92

203980_at	48	58	9	115	30	226	53	158	138	59	40	19	1	3	5	7	8	3	22	9	3
203981_s_at	504	327	190	281	279	231	309	296	252	187	244	331	461	307	229	227	290	204	229	337	384
203982_s_at	89	63	126	123	287	116	111	78	189	113	90	99	5	44	51	48	50	75	44	54	42
203983_at	1257	658	704	694	575	525	924	907	404	591	539	491	1246	1522	1751	2807	2156	2917	2342	2283	3285
203984_s_at	540	656	547	415	713	646	485	367	391	387	483	407	217	304	375	358	323	407	261	290	304
203985_at	422	569	707	859	290	293	476	641	583	676	595	451	263	301	282	397	445	323	543	427	298
203986_at	340	449	136	307	186	484	379	391	124	267	290	241	59	146	184	206	261	220	114	151	43
203987_at	1920	2220	575	616	770	878	1627	2243	519	714	948	1051	647	927	873	2606	2330	2470	632	445	805
203988_s_at	1364	828	316	194	943	1279	757	649	84	68	745	1037	650	548	1054	1005	887	817	264	261	451
203989_x_at	544	271	250	88	82	26	227	396	62	198	55	89	39	38	41	586	517	442	800	345	374
203990_s_at	135	63	193	154	41	47	209	336	187	162	348	265	31	42	30	30	29	30	14	31	43
203991_s_at	138	69	52	26	169	351	49	62	50	56	63	148	210	253	158	156	153	203	107	179	245
203992_s_at	325	219	263	352	327	470	235	297	178	250	490	483	280	221	329	227	224	261	189	187	238
203993_x_at	24	13	15	13	41	20	28	17	15	15	13	20	4	6	5	6	5	7	5	5	3
203994_s_at	22	24	18	39	82	101	12	36	29	15	17	14	117	93	66	55	38	61	68	43	38
203995_at	40	163	125	80	51	35	42	40	19	80	34	26	5	36	13	9	33	13	7	12	3
203996_s_at	275	291	224	225	557	289	182	160	254	214	198	124	104	68	186	51	41	36	108	52	106
203997_at	1154	811	1556	1362	1318	899	1218	1181	1098	1381	1758	862	509	511	446	280	172	160	374	317	331
203998_s_at	401	247	9	13	74	32	338	557	9	4	74	31	158	98	160	854	688	474	32	111	120
203999_at	2152	3353	241	195	460	509	1772	2002	104	177	451	285	573	393	500	4888	5413	4963	170	917	1130
204000_at	423	586	357	490	663	511	346	383	444	648	448	426	328	518	457	621	622	570	758	958	998
204001_at	565	440	328	367	524	493	538	366	384	363	347	343	354	287	395	597	581	668	754	694	1120
204002_s_at	475	353	488	711	334	419	303	267	278	362	289	258	114	130	135	138	140	117	178	201	284
204003_s_at	499	397	505	332	1041	935	465	391	411	415	647	695	573	652	700	503	447	476	659	579	442
204004_at	478	572	1919	1571	1796	1725	837	933	2282	2267	1740	1855	1163	768	760	316	281	252	1251	862	1179
204005_s_at	459	762	773	469	697	1361	458	327	668	893	686	1116	622	406	610	467	402	551	1261	1424	1545
204006_s_at	74	68	123	127	241	196	73	92	43	71	30	107	8	1	2	17	7	3	4	18	14

204007_at	102	130	232	213	279	232	164	182	209	208	183	196	16	18	12	26	16	5	40	36	20
204008_at	426	414	109	376	206	165	232	428	107	46	125	150	129	55	84	110	73	63	39	64	91
204009_s_at	1464	1129	1805	2230	2955	3971	1439	1554	1703	1771	3587	2782	5024	3369	3927	2845	2045	1907	4250	4225	4837
204010_s_at	71	49	30	115	148	234	42	30	25	28	63	60	71	95	222	76	58	60	79	166	129
204011_at	246	111	633	195	311	116	232	186	673	1393	372	537	475	685	775	417	221	245	2706	1960	3580
204012_s_at	368	344	709	555	793	572	439	542	716	946	647	636	730	787	1072	488	516	492	1477	1028	1238
204013_s_at	21	11	5	16	29	16	19	17	12	6	4	9	16	22	4	3	6	47	3	2	19
204014_at	2523	1603	514	62	118	69	778	2156	306	551	252	317	242	281	624	5218	4457	3475	931	1333	1067
204015_s_at	1319	1150	576	12	520	59	1418	1615	227	408	264	232	199	369	198	1529	1385	1664	362	394	375
204016_at	718	483	460	440	722	513	637	426	423	408	449	533	499	239	286	238	171	276	428	353	375
204017_at	1459	1920	1030	449	1534	2305	1469	1415	1181	1522	2226	2601	2990	2372	2040	2112	2769	2166	2519	2099	2455
204018_x_at	349	193	298	102	253	82	109	193	238	197	136	287	110	48	152	12	60	71	162	107	60
204019_s_at	1461	2780	2417	3889	1134	2540	1051	1737	1956	2373	600	980	844	467	1191	2010	2271	1489	3965	4887	4686
204020_at	259	238	1065	1666	1479	1578	284	284	1223	1553	1488	1078	1095	1153	1430	297	237	305	2240	2086	3174
204021_s_at	247	263	345	365	374	352	310	412	520	573	512	313	1140	912	489	75	83	60	365	415	197
204022_at	747	662	492	639	693	614	488	527	538	449	424	510	461	288	396	367	327	376	578	577	680
204023_at	2548	1466	2250	987	3773	1282	2825	2772	2179	1730	3840	3496	4111	5230	4610	3412	2980	3988	4448	3020	2900
204024_at	294	311	109	188	166	290	227	340	177	217	183	209	98	108	208	447	321	299	218	229	262
204025_s_at	736	639	331	200	612	878	456	293	165	169	237	284	717	443	353	852	682	889	697	838	996
204026_s_at	3293	1618	3236	921	4080	457	5018	3870	5971	4742	4747	3612	7125	5719	6974	6584	6445	7124	10071	3965	4077
204027_s_at	941	614	730	592	981	945	827	1049	756	837	777	738	585	302	463	565	617	519	702	437	383
204028_s_at	523	399	1371	2541	620	833	392	726	1317	1922	1022	1067	1389	1221	1579	523	569	631	1644	2638	3377
204029_at	589	712	236	414	525	652	723	627	206	261	685	481	179	224	329	257	274	249	151	166	177
204030_s_at	406	491	265	132	1288	1200	270	272	181	222	969	775	1434	1533	1591	1151	1100	1272	507	362	660
204031_s_at	11801	9934	8323	10121	7618	10710	9084	11995	8205	11017	13267	12879	11358	12477	14269	12210	12241	9897	10774	14050	10780
204032_at	640	813	621	771	1177	1222	298	280	314	396	821	1122	924	916	746	744	814	662	691	664	1088
204033_at	1978	931	2965	522	2404	491	2564	2027	3980	3078	2696	2624	2435	2551	3920	2356	1989	2978	4891	1171	1961

204034_at	2062	2310	2511	2628	1994	2443	2624	2043	2305	3479	2159	3159	2003	2025	2227	2131	2367	1792	3201	2703	1756
204035_at	658	740	90	58	261	241	542	569	11	108	63	46	13	17	11	533	667	651	41	42	21
204036_at	344	905	46	48	95	19	239	252	62	41	23	28	1	11	11	743	814	815	1	6	19
204037_at	402	382	151	92	620	437	256	179	92	59	31	52	62	44	87	313	379	343	81	45	86
204038_s_at	581	749	254	246	476	489	665	520	368	362	253	214	71	77	68	231	273	247	115	74	45
204039_at	292	186	237	1442	311	207	284	399	318	330	243	201	48	69	96	81	70	73	118	145	60
204040_at	394	454	18	25	34	23	404	421	24	27	26	158	68	8	56	282	230	339	66	96	129
204041_at	1214	714	419	899	694	361	808	1250	458	2043	682	593	993	662	685	308	421	443	395	170	284
204042_at	374	363	170	110	1884	1877	400	355	91	115	1821	1948	1445	1809	2490	323	276	150	54	122	133
204043_at	276	169	155	476	421	472	192	153	218	205	239	171	157	196	159	58	72	130	161	131	174
204044_at	20	21	753	1761	375	734	12	18	415	358	378	222	307	291	304	24	21	3	794	1398	868
204045_at	165	50	703	2241	1131	1720	61	231	495	752	782	843	1396	1632	1304	41	39	6	1144	1616	1552
204046_at	86	163	72	98	34	50	91	107	88	63	74	87	12	15	17	19	26	32	36	25	72
204047_s_at	147	130	189	291	198	164	235	183	330	296	210	111	64	123	56	61	53	50	87	120	98
204048_s_at	238	241	604	1115	331	351	364	300	567	523	399	334	457	542	613	203	239	175	923	1082	1387
204049_s_at	202	229	665	1036	489	495	249	326	499	341	414	264	283	273	233	116	140	151	428	593	761
204050_s_at	3162	3551	2223	3870	2932	3431	3605	3304	3580	3361	4730	4440	8294	7557	5985	3204	2719	2311	2646	3361	2139
204051_s_at	239	116	229	221	157	145	178	227	225	175	163	221	25	19	37	23	28	56	38	31	9
204052_s_at	91	83	49	63	128	314	107	121	13	99	41	109	1	2	13	6	23	21	25	20	25
204053_x_at	1159	2174	1727	3216	1278	1334	860	1177	1857	1827	1208	953	846	726	1172	1124	1132	1203	2740	2806	3060
204054_at	315	314	221	534	261	146	468	375	396	331	141	99	138	218	191	607	549	401	748	526	688
204055_s_at	38	95	73	114	109	120	81	115	40	61	72	63	13	2	6	19	19	42	29	41	48
204056_s_at	599	333	433	59	360	341	166	215	170	116	277	30	124	153	163	234	161	163	171	115	86
204057_at	42	84	102	186	285	348	9	42	66	144	192	117	89	96	73	50	43	6	60	9	54
204058_at	2293	2579	1673	830	1058	1876	1468	1271	899	1779	770	405	1191	1032	1863	3689	2661	2233	3600	3411	4987
204059_s_at	2982	3766	3006	1179	1805	2637	2235	2229	1860	2456	1076	941	1962	1853	2924	4245	4090	3781	3664	4638	5137
204060_s_at	605	844	331	349	361	297	460	516	467	453	175	214	114	83	170	243	208	176	238	204	171

204061_at	415	433	246	146	125	152	280	268	324	250	148	67	69	60	38	108	97	58	128	79	122
204062_s_at	243	365	54	35	212	324	208	286	82	48	152	235	70	73	124	175	255	159	29	6	11
204063_s_at	160	140	68	87	148	183	189	243	99	110	142	195	47	30	38	103	79	78	14	6	36
204064_at	553	988	1781	1189	768	1532	412	528	1894	1488	1234	968	1128	1392	1923	731	709	789	2617	1674	1923
204065_at	263	141	404	292	294	196	371	351	420	306	382	447	197	225	133	74	91	25	12	76	66
204066_s_at	788	1086	1061	887	495	902	947	903	1032	1140	1795	701	822	999	993	646	787	429	793	788	733
204067_at	700	596	673	1297	423	132	649	660	649	815	563	464	205	329	405	604	701	480	649	549	350
204068_at	720	1185	411	190	305	333	480	798	131	314	169	240	636	458	648	1936	1788	1514	765	1143	1381
204069_at	103	95	126	199	179	140	12	117	146	152	153	165	65	34	78	13	5	2	102	66	72
204070_at	77	161	140	1681	98	476	113	100	213	198	183	129	34	24	32	11	50	248	288	861	576
204071_s_at	261	263	332	375	334	375	213	319	282	382	269	224	663	617	331	695	447	583	762	530	662
204072_s_at	30	43	255	554	81	375	61	13	591	667	114	74	48	52	37	38	52	32	375	566	967
204073_s_at	708	736	724	940	643	515	705	581	867	1646	522	422	181	174	204	270	235	333	674	519	569
204074_s_at	528	365	439	427	844	950	359	442	498	516	586	500	239	223	238	284	248	244	248	272	232
204075_s_at	157	140	133	89	201	251	61	74	45	91	47	102	140	86	126	205	162	198	279	565	403
204076_at	1451	2387	726	1172	1260	2559	881	1226	657	813	1532	1999	1409	2079	1538	2248	2107	1398	1134	1458	1432
204077_x_at	191	175	209	154	423	499	152	179	193	180	327	214	117	135	102	83	120	59	88	66	57
204078_at	318	297	186	169	322	403	139	179	149	184	168	122	355	244	239	417	538	415	640	706	771
204079_at	349	444	378	299	467	465	350	368	569	376	281	225	345	236	334	538	480	321	797	460	548
204080_at	758	249	539	539	624	275	652	433	735	678	343	192	419	551	360	552	585	619	812	408	466
204081_at	630	367	712	475	581	86	904	1039	704	1361	685	540	251	217	184	270	243	283	447	173	120
204082_at	228	175	395	361	675	410	245	330	400	488	971	589	425	535	837	235	161	141	362	305	353
204083_s_at	3270	1873	644	910	44	191	1940	1902	492	730	70	13	147	76	56	2839	2138	2044	781	870	430
204084_s_at	824	921	502	619	434	744	551	579	400	465	418	447	333	484	792	1612	1547	1374	840	1064	1225
204085_s_at	204	433	236	345	221	504	297	342	122	210	217	377	169	223	175	515	496	447	254	525	703
204086_at	656	386	100	80	241	23	510	388	142	157	87	103	2	7	11	254	238	313	18	19	13
204087_s_at	799	783	859	535	613	461	1043	719	1300	1050	918	633	756	872	917	641	653	654	962	593	785

204088_at	430	609	277	547	583	220	400	546	273	393	860	902	828	810	206	113	215	159	58	70	99
204089_x_at	1049	1103	1590	1026	741	423	871	1007	1189	1427	709	633	309	266	170	337	365	326	904	757	757
204090_at	164	550	88	211	56	120	213	106	123	141	190	202	166	290	93	45	47	59	83	191	133
204091_at	827	985	454	380	992	708	404	641	242	435	443	1131	755	794	680	868	985	698	382	611	391
204092_s_at	2327	418	1904	407	3523	473	1749	1797	2803	1653	4347	3115	5621	3973	3412	1524	1522	1617	4243	1543	2092
204093_at	1737	1225	2043	1642	1472	1716	2434	2249	2594	2791	2362	1894	1945	2078	2323	2029	1874	1633	3873	3067	2545
204094_s_at	930	737	2169	927	1225	1341	407	536	876	1054	810	740	1513	1227	1091	1405	1018	1233	3571	2107	2690
204095_s_at	164	202	132	151	231	99	195	244	171	196	158	176	25	79	41	44	44	76	48	43	53
204096_s_at	229	85	266	65	253	137	142	150	142	131	148	207	69	113	53	82	143	63	70	53	32
204097_s_at	544	562	552	820	1005	982	663	636	753	738	757	695	803	468	400	297	264	237	595	700	676
204098_at	468	324	517	641	390	339	396	480	619	548	614	699	440	226	502	574	467	495	1143	934	854
204099_at	609	1060	179	266	313	417	542	513	189	187	280	175	130	165	222	448	486	431	37	127	41
204100_at	33	36	16	65	59	71	13	21	33	18	18	23	7	13	6	78	15	33	18	57	45
204101_at	57	13	184	155	170	280	73	34	98	232	193	163	274	224	91	85	87	74	245	162	143
204102_s_at	15985	25530	10862	12959	11003	17494	14225	15409	12629	11614	10088	12069	15956	14531	12949	21540	19757	18186	15846	23560	21574
204103_at	50	32	42	39	122	87	36	67	23	21	27	64	4	3	4	9	4	4	6	6	6
204104_at	327	347	194	259	169	74	338	369	230	183	237	256	84	65	87	183	72	191	55	80	49
204105_s_at	78	35	39	92	36	79	174	127	60	46	44	13	8	24	40	6	24	8	48	51	70
204106_at	548	634	326	437	221	316	293	392	237	323	627	555	485	372	455	283	381	230	171	472	444
204107_at	458	161	365	175	367	349	576	611	281	419	340	367	102	91	95	156	88	168	111	64	70
204108_at	743	714	836	511	786	396	885	941	665	641	516	558	334	347	373	659	752	781	478	374	343
204109_s_at	467	350	482	392	326	348	718	715	508	600	568	457	126	117	124	123	144	131	107	135	118
204110_at	77	38	95	92	14	65	115	75	41	105	47	29	13	25	26	3	6	22	86	64	64
204111_at	9	2	46	18	37	7	48	45	75	17	9	54	14	6	16	3	4	23	39	29	60
204112_s_at	8	22	476	1080	51	36	5	7	392	342	24	48	9	4	30	6	17	1	1496	1856	2364
204113_at	306	46	484	406	374	238	502	533	662	650	667	437	148	168	360	123	138	151	227	136	170
204114_at	114	35	142	54	70	204	138	88	84	84	53	10	2	2	19	29	38	85	74	40	37

204115_at	8	1	35	4	838	433	17	24	41	10	573	354	2059	2370	935	57	39	37	13	2	3
204116_at	230	189	283	69	555	624	195	244	229	245	388	351	32	48	6	54	54	75	70	55	42
204117_at	1593	852	944	625	663	689	1342	1145	1533	1445	831	748	604	469	446	1202	984	803	1070	740	1106
204118_at	253	258	296	244	377	394	285	300	308	335	254	238	32	74	72	66	48	56	74	57	53
204119_s_at	2707	1873	2722	1342	1846	1412	2613	2184	2303	1681	1931	2008	2085	2210	3111	3559	3858	3678	2613	2633	3210
204120_s_at	640	355	568	136	528	438	172	317	126	281	239	238	184	227	232	500	241	314	451	409	445
204121_at	12	75	65	17	210	179	16	20	58	13	81	107	84	59	52	8	11	3	44	81	34
204122_at	98	74	62	120	139	133	65	80	72	60	26	78	17	4	13	3	33	5	7	14	3
204123_at	418	353	780	186	470	383	420	495	83	457	371	226	189	119	255	203	197	171	298	189	242
204124_at	78	13	125	113	77	121	136	132	115	159	45	121	11	23	13	3	22	18	8	43	32
204125_at	545	488	585	634	415	759	873	702	589	470	570	527	727	393	574	998	680	614	1014	611	688
204126_s_at	574	26	1135	146	663	40	481	305	1707	1289	659	612	856	731	916	248	418	529	1711	628	710
204127_at	1262	661	2029	578	2477	439	1716	1357	2571	2351	3256	2581	3569	2857	4779	2176	1697	2714	4046	1259	2334
204128_s_at	779	237	819	243	1204	263	876	690	1060	1047	1828	1144	1428	907	1124	467	512	733	1406	393	837
204129_at	276	266	259	332	36	121	368	458	237	352	219	158	73	100	103	196	190	106	176	82	145
204130_at	204	182	109	32	99	48	159	117	234	221	30	83	3	6	21	69	65	37	130	81	64
204131_s_at	1416	1932	1378	2212	1721	1633	1251	1147	1003	1174	1219	1372	672	1094	1385	1229	1315	1025	1510	2226	1887
204132_s_at	642	639	947	1315	638	640	637	867	502	682	665	493	195	228	127	110	148	142	289	326	413
204133_at	693	502	1186	319	316	243	1522	699	1333	1869	961	344	515	418	277	394	259	251	690	227	269
204134_at	303	133	50	45	118	47	600	255	48	59	161	206	81	70	92	17	69	64	8	69	5
204135_at	114	41	488	428	115	24	111	144	326	155	109	74	38	26	74	34	19	40	279	433	640
204136_at	119	381	45	125	561	491	58	29	47	43	142	36	102	110	84	19	70	52	34	92	55
204137_at	238	481	240	388	620	727	285	468	158	366	372	767	603	757	871	445	499	277	266	417	395
204138_s_at	353	172	395	207	404	367	366	271	326	288	307	226	44	47	75	29	57	40	55	11	11
204139_x_at	460	805	397	1270	143	437	343	397	678	594	215	182	148	234	160	282	271	216	268	431	391
204140_at	370	285	174	124	1315	910	351	356	183	164	1269	783	1254	381	572	526	676	277	239	237	296
204141_at	4149	4236	727	768	12430	10426	2829	2960	389	813	7437	6662	11931	12832	12648	3881	4105	3518	1967	1589	1493

204142_at	1185	522	922	380	445	556	433	567	801	546	365	271	506	485	537	1511	1602	1834	3493	1183	1840
204143_s_at	1015	757	1530	722	733	548	876	724	1721	1358	594	617	723	772	1054	1731	2298	2002	2643	1704	1997
204144_s_at	181	128	270	188	115	274	189	248	244	254	230	216	80	111	105	33	55	68	88	95	67
204145_at	872	318	361	322	1533	1622	631	569	297	404	787	720	1898	1063	622	853	927	1071	843	764	669
204146_at	587	245	1188	136	1318	7	656	616	1084	874	1067	1325	3672	1681	1299	596	405	914	2744	1089	2219
204147_s_at	945	496	932	495	1199	83	784	610	1549	924	1408	583	491	573	392	388	340	384	496	262	359
204148_s_at	773	1098	418	643	353	1146	818	632	308	386	442	314	296	300	484	537	437	382	339	286	218
204149_s_at	65	66	3	32	459	426	175	160	4	4	867	692	219	189	128	47	85	79	4	6	2
204150_at	90	85	148	217	180	133	105	140	159	140	149	170	8	11	6	41	9	9	35	14	41
204151_x_at	353	248	659	12103	465	465	359	265	609	985	207	300	101	140	144	119	127	154	799	1948	1464
204152_s_at	33	77	54	137	22	20	19	77	28	131	84	109	10	16	7	23	3	28	7	49	41
204153_s_at	134	107	152	250	170	171	150	154	171	243	153	132	11	33	6	54	64	40	85	235	134
204154_at	134	56	176	147	361	273	216	150	115	169	136	152	28	80	34	30	38	37	46	37	7
204155_s_at	258	350	250	319	401	527	355	438	304	352	320	392	87	222	195	156	168	197	168	153	107
204156_at	255	235	190	151	205	270	175	325	218	215	182	204	108	108	92	73	87	139	113	157	132
204157_s_at	271	305	224	250	247	470	368	274	270	252	384	258	174	168	150	269	321	394	250	290	367
204158_s_at	163	512	431	499	95	309	49	90	356	253	158	55	254	288	228	140	162	159	421	360	306
204159_at	644	94	126	66	613	91	182	261	40	155	507	538	751	277	265	77	145	191	332	129	78
204160_s_at	519	533	429	673	568	632	608	515	314	414	368	378	418	418	593	714	584	622	436	676	824
204161_s_at	194	332	103	344	103	282	103	145	66	94	175	158	175	103	70	182	188	215	130	192	261
204162_at	351	195	800	119	1517	5	611	602	518	686	1113	1017	2135	2731	2036	1198	987	1350	2865	940	1531
204163_at	9	5	23	21	16	12	5	9	5	6	9	11	5	5	21	6	10	30	4	4	14
204164_at	271	690	180	581	553	375	286	240	237	296	250	189	142	139	49	193	179	121	109	116	58
204165_at	1037	813	703	625	624	714	533	727	458	548	583	633	598	767	875	1443	1285	1344	1368	1319	2500
204166_at	22	420	229	41	172	73	50	32	50	50	131	29	15	6	29	110	108	55	104	49	26
204167_at	16	135	229	744	38	20	117	117	227	433	306	273	215	236	190	13	61	66	128	94	112
204168_at	434	523	1001	1333	783	435	718	541	977	1343	1018	788	1417	1095	1675	803	902	613	2239	1342	1061

204169_at	587	701	1150	993	811	501	1336	1009	2036	1473	840	628	436	315	394	395	378	316	1292	578	504
204170_s_at	4247	1389	5121	1544	7739	1721	4037	3170	5108	4102	6761	7489	13378	14377	14240	9160	8941	9169	10334	5766	4537
204171_at	881	434	222	235	504	493	470	534	191	228	352	185	562	431	369	1239	933	916	1007	702	834
204172_at	9677	6962	680	515	643	528	4626	10880	550	1064	605	490	668	450	678	7992	6951	7310	2308	1263	1637
204173_at	1819	1465	1209	2193	1256	1644	1602	1324	1125	914	1617	1517	3268	1407	1406	1366	1440	1021	873	1309	668
204174_at	70	109	248	181	279	908	260	128	294	222	514	302	109	147	88	27	56	34	90	35	66
204175_at	1502	1560	891	801	818	970	1377	900	1028	897	1234	983	2123	716	626	657	477	770	863	1273	495
204176_at	269	229	244	239	346	466	85	143	151	238	182	226	385	229	305	456	402	322	676	463	840
204177_s_at	458	304	460	817	691	671	636	581	518	629	551	684	312	231	194	141	228	200	233	207	286
204178_s_at	1498	650	1381	813	1168	246	1114	640	1195	1022	1529	883	818	566	630	538	423	413	860	254	339
204179_at	165	590	190	755	184	16	245	208	209	273	144	161	12	22	61	161	141	67	32	75	64
204180_s_at	319	187	326	189	371	523	310	311	238	270	232	333	120	140	125	125	91	102	106	121	93
204181_s_at	447	288	467	318	92	474	473	491	420	433	448	318	183	252	200	302	289	199	200	194	178
204182_s_at	22	17	99	87	33	36	20	12	146	31	159	26	129	147	144	18	7	61	121	122	118
204183_s_at	187	116	156	191	287	288	319	170	155	166	122	132	65	13	49	53	61	82	135	69	114
204184_s_at	253	219	199	190	209	198	335	313	225	266	267	201	5	51	65	82	145	92	55	45	52
204185_x_at	4470	1647	1902	964	2629	2489	2320	1776	1668	1455	1636	1496	2998	2819	3662	4196	3929	3704	3599	2874	3381
204186_s_at	2973	1086	1443	594	1773	872	1658	1230	1128	927	1192	871	1891	1178	1012	2032	2198	1822	2368	1717	2215
204187_at	292	266	152	181	147	78	596	657	131	143	385	318	206	296	275	360	396	414	43	39	48
204188_s_at	361	523	495	630	104	134	460	359	458	386	391	124	49	70	13	123	103	161	105	89	72
204189_at	364	342	415	515	212	231	474	273	466	372	222	94	54	117	69	174	159	158	146	134	128
204190_at	407	403	876	674	664	1259	469	615	642	457	668	840	353	259	454	683	544	634	704	914	1359
204191_at	226	137	80	154	188	253	193	189	111	126	209	288	71	108	105	78	65	85	60	92	118
204192_at	46	35	16	93	131	47	8	58	27	113	68	97	8	2	6	3	3	2	4	29	14
204193_at	471	836	266	489	47	496	431	723	217	189	61	176	228	337	235	242	420	327	172	224	237
204194_at	365	268	422	459	342	972	374	292	266	456	388	385	478	398	416	316	405	375	660	776	1232
204195_s_at	192	125	57	18	93	47	136	170	127	134	210	232	285	394	168	154	206	108	210	142	161

204196_x_at	91	77	138	114	91	106	160	198	185	190	178	153	17	28	14	3	2	7	58	23	25
204197_s_at	496	356	109	146	700	895	519	482	100	130	850	332	443	401	492	429	397	358	70	169	122
204198_s_at	728	807	11	9	935	972	705	488	21	15	1055	434	386	242	492	337	334	336	62	226	242
204199_at	75	208	87	583	109	335	121	189	114	207	67	87	35	26	58	146	118	50	139	191	232
204200_s_at	153	263	209	235	378	302	136	164	37	159	387	276	237	88	116	120	41	16	104	76	72
204201_s_at	191	272	266	160	117	124	160	294	174	196	48	49	21	6	13	300	243	297	316	357	428
204202_at	622	631	457	733	296	548	559	533	405	675	244	192	126	79	72	206	168	182	173	174	134
204203_at	1131	1167	1283	429	555	1545	925	468	1010	925	543	455	637	414	871	822	854	1273	1351	1964	1726
204204_at	259	274	176	204	276	269	327	336	198	242	297	518	264	300	304	298	256	292	284	264	225
204205_at	148	32	80	43	1989	2178	229	149	21	92	2093	2955	4312	3556	2188	735	880	651	26	64	93
204206_at	960	983	566	815	646	640	429	622	717	721	379	547	460	451	454	620	895	537	1217	974	1272
204207_s_at	826	585	1331	704	914	726	1055	1350	1019	1086	989	875	345	351	312	643	681	632	777	543	711
204208_at	523	375	469	215	106	281	386	490	261	303	273	309	444	365	652	954	809	921	1016	697	1016
204209_at	615	437	454	607	678	800	647	686	649	544	962	569	135	95	113	193	179	150	104	74	103
204210_s_at	155	4	64	89	192	208	106	11	62	67	298	150	209	112	99	109	79	81	72	76	51
204211_x_at	398	619	732	823	951	684	370	455	1048	906	1072	1382	909	952	742	450	390	977	782	904	1410
204212_at	405	280	362	525	712	634	310	252	337	498	600	574	1231	717	858	256	203	249	350	579	393
204213_at	302	270	210	255	99	132	241	354	423	169	216	250	76	92	69	82	156	112	156	119	88
204214_s_at	169	174	250	462	576	684	182	298	310	200	486	558	529	441	737	57	47	98	285	392	299
204215_at	643	1006	1822	1834	1526	2021	533	529	1983	1992	2055	2059	2748	2657	3884	1145	1087	1204	3248	2793	2808
204216_s_at	568	776	1006	773	1002	1082	1256	1193	1041	717	1074	975	880	780	404	1153	954	843	967	977	1116
204217_s_at	257	242	317	951	379	512	241	314	286	713	240	186	65	40	21	119	63	61	93	126	79
204218_at	874	617	861	1133	723	263	1200	1099	1375	1459	1013	899	539	684	710	708	726	666	1304	828	485
204219_s_at	3986	2922	3285	2487	7469	4529	6212	4553	4177	3325	8142	7264	8027	9185	6840	4072	4185	4653	3300	2636	2151
204220_at	16	40	8	19	60	42	24	17	19	22	179	204	78	134	117	18	19	10	63	9	3
204221_x_at	124	105	199	49	234	192	133	164	86	122	65	79	56	7	73	104	54	108	218	101	98
204222_s_at	32	29	96	32	15	7	29	37	56	11	60	14	3	9	6	9	42	9	40	76	79

204223_at	25	24	20	176	59	239	37	28	28	35	31	25	8	5	2	3	6	2	25	77	21
204224_s_at	1134	1325	977	653	808	917	1113	974	955	939	1020	992	1409	918	1435	2710	2577	2417	2058	2420	3428
204225_at	605	275	435	250	1024	914	333	400	328	326	588	586	677	653	1077	273	433	148	460	765	820
204226_at	262	328	345	269	434	347	580	323	313	347	324	274	293	339	368	621	486	452	531	443	668
204227_s_at	516	454	381	335	329	277	360	352	371	377	347	271	121	116	113	277	237	190	191	243	246
204228_at	1355	697	2117	661	1205	382	2218	1927	1917	1884	1323	1010	1591	1143	1352	1743	1713	1835	2060	1235	924
204229_at	151	226	130	59	280	324	152	228	205	183	242	292	84	20	172	102	16	69	93	90	52
204230_s_at	180	73	134	199	198	235	229	181	248	231	262	282	41	91	101	83	45	55	50	57	46
204231_s_at	261	231	254	347	150	176	228	282	268	295	175	136	87	62	52	74	52	32	123	75	84
204232_at	233	202	160	193	412	425	254	240	258	194	188	180	38	59	30	51	62	32	40	35	33
204233_s_at	380	512	2317	2412	1126	656	553	418	2221	2180	1521	654	450	468	468	342	354	285	954	1075	513
204234_s_at	557	370	700	458	532	484	926	1136	573	443	989	533	573	647	483	800	681	834	525	581	777
204235_s_at	1721	2592	595	406	403	663	1249	1514	169	92	385	197	1417	1096	1494	3572	3201	2732	438	1130	1555
204236_at	41	17	4	16	12	15	28	24	51	20	3	41	9	6	25	3	6	1	15	2	3
204237_at	1705	2008	301	269	415	997	1011	1136	199	159	371	117	2132	1635	3133	5472	5865	6802	784	2247	4061
204238_s_at	774	1534	210	186	250	460	1118	1213	631	545	431	676	974	606	232	558	416	191	191	131	136
204239_s_at	83	114	64	206	201	566	240	218	194	224	54	219	23	9	17	40	34	2	14	29	29
204240_s_at	451	154	945	490	1754	375	955	804	1128	1110	1538	1082	1327	1162	1417	599	576	690	1867	873	1307
204241_at	336	388	118	221	173	185	303	317	163	208	226	183	160	200	206	245	291	323	180	180	200
204242_s_at	187	181	79	219	44	67	76	169	229	171	114	166	30	53	17	78	114	134	102	132	120
204243_at	398	388	395	453	537	204	429	478	385	368	384	496	356	421	359	564	450	475	395	340	410
204244_s_at	1364	414	1282	150	2833	833	984	941	1460	758	2936	1438	5398	4711	4546	2031	2126	2105	4066	2004	3126
204245_s_at	753	510	680	517	1244	938	965	868	834	662	1094	821	960	889	896	702	744	647	674	607	587
204246_s_at	1746	1234	1781	2690	1970	3333	1933	1548	1561	1472	3354	2756	2541	2236	1656	1341	1584	1509	1499	1419	842
204247_s_at	668	659	806	1460	610	634	746	657	840	730	908	469	931	1091	856	539	641	506	673	845	471
204248_at	1005	1302	670	358	836	617	509	628	183	396	681	334	788	505	401	671	645	469	588	417	231
204249_s_at	331	828	190	786	2595	1942	404	397	163	159	2224	1945	1097	724	3554	168	199	227	168	271	227

204250_s_at	52	11	110	93	166	102	115	107	134	180	223	165	30	46	28	3	5	35	49	48	45
204251_s_at	271	257	282	244	473	554	348	342	361	397	402	268	216	130	192	83	117	66	206	268	211
204252_at	2388	1844	1979	1019	3291	1314	3093	2869	2315	2189	2973	2735	1891	1737	1571	1819	1676	1824	2350	1284	1536
204253_s_at	185	206	220	392	865	371	184	129	404	254	550	160	131	124	144	74	92	55	88	11	33
204254_s_at	294	277	581	481	830	556	407	269	507	710	1343	857	1117	697	1240	298	426	337	984	767	714
204255_s_at	206	192	610	307	323	224	232	133	562	572	969	563	876	663	668	126	273	106	528	288	387
204256_at	1629	1844	1194	175	532	355	1075	1577	967	657	281	155	182	173	274	3182	4802	2237	1636	897	993
204257_at	646	1645	506	678	1019	2229	468	409	343	280	489	416	435	488	786	656	552	510	456	443	450
204258_at	470	342	960	669	980	820	888	843	1139	791	1120	818	1226	1100	1160	990	985	1145	1330	1263	1590
204259_at	250	276	7758	8831	236	267	136	220	3709	1768	151	146	35	34	41	99	88	42	8231	11272	10862
204260_at	254	131	113	150	235	280	90	206	274	109	169	295	41	19	60	5	11	18	33	60	49
204261_s_at	160	46	118	59	272	669	57	92	115	123	117	73	50	35	10	10	24	42	31	8	8
204262_s_at	355	348	259	200	271	1152	269	301	293	266	331	298	197	187	178	119	141	166	209	139	101
204263_s_at	254	365	495	835	333	184	510	414	183	703	364	243	347	583	893	530	642	663	855	924	1285
204264_at	300	201	271	450	513	357	242	201	136	327	151	290	579	372	496	530	377	614	1247	1208	1602
204265_s_at	130	141	282	238	279	110	217	143	183	211	426	338	96	191	202	22	17	74	57	15	39
204266_s_at	815	989	4151	3811	657	192	1165	757	4442	4082	2042	1538	839	985	588	477	407	314	1030	1753	822
204267_x_at	782	470	933	517	961	548	1166	1196	1916	1213	1117	636	529	526	478	342	322	352	633	290	235
204268_at	560	724	1354	888	7387	4970	1569	1482	1683	1744	11304	7292	13517	11163	9043	1202	1058	876	2216	1235	541
204269_at	577	367	414	238	504	513	461	413	491	519	513	390	117	112	210	230	247	221	240	108	141
204270_at	152	144	194	150	99	39	174	193	194	222	134	132	144	77	22	59	73	87	67	47	39
204271_s_at	102	75	172	164	400	267	97	77	58	126	71	99	58	62	50	6	30	51	45	44	32
204272_at	130	72	8395	12788	231	269	225	132	6669	12710	144	124	75	56	43	3	31	3	8240	10756	7969
204273_at	13	5	4	3	15	40	28	8	1	1	23	70	1	9	1	1	12	4	9	2	1
204274_at	1918	1820	576	561	521	953	1275	1474	380	416	604	568	1148	1242	1609	3932	3977	4221	2129	2746	2668
204275_at	347	367	903	467	283	254	375	384	1006	868	517	290	164	118	68	68	32	88	143	78	72
204276_at	273	316	376	535	136	211	213	210	143	307	144	102	181	77	117	238	258	117	275	498	433

204277_s_at	78	99	146	83	102	82	25	45	36	27	17	13	65	11	45	38	11	20	96	70	70
204278_s_at	558	449	319	235	351	462	676	669	330	282	343	243	470	350	246	689	781	777	424	364	433
204279_at	438	1500	110	376	823	1263	379	284	142	211	1424	1634	663	555	272	165	280	454	41	137	91
204280_at	192	146	153	66	351	386	228	208	178	161	192	45	50	94	21	56	68	101	74	86	41
204281_at	930	866	1015	573	1248	868	702	487	850	756	1627	895	1092	519	633	370	334	286	444	491	296
204282_s_at	542	436	354	453	738	679	533	293	408	317	351	308	215	294	276	280	245	200	336	289	217
204283_at	601	238	380	401	408	531	465	350	372	363	468	357	561	494	387	385	423	304	508	378	375
204284_at	181	10	95	110	192	367	162	172	96	172	155	263	54	20	63	20	46	22	19	34	17
204285_s_at	2290	4102	728	118	1065	1222	2131	1872	387	764	1174	1835	2200	1214	1030	2050	2383	4270	1048	751	1268
204286_s_at	1311	2331	351	45	356	790	338	785	92	363	530	691	1366	845	793	1063	1153	2880	761	857	1113
204287_at	258	399	159	145	220	212	260	128	162	143	286	92	32	90	197	141	177	113	93	82	64
204288_s_at	37	9	75	542	517	469	17	32	29	15	189	123	87	71	118	40	24	4	81	166	154
204289_at	26	13	12	21	175	253	52	32	43	17	14	18	1	1	3	2	10	4	4	6	5
204290_s_at	558	715	332	1551	357	919	425	607	178	310	377	651	231	242	172	386	491	274	249	435	562
204291_at	362	400	332	325	203	284	276	389	207	305	131	158	194	133	98	367	376	447	524	468	836
204292_x_at	153	197	119	94	364	267	48	148	33	34	124	46	48	17	9	70	47	52	48	25	25
204293_at	53	130	16	39	49	71	32	115	45	25	14	29	8	9	5	37	73	116	22	12	57
204294_at	235	618	206	601	351	529	329	327	238	319	233	225	54	49	108	116	146	138	79	144	89
204295_at	3065	2799	1149	2200	849	1547	2504	2692	910	1646	1362	1737	2024	2267	2277	3322	4542	2847	1648	2127	937
204296_at	179	134	26	18	78	36	171	87	36	27	135	16	31	25	45	44	37	39	60	86	55
204297_at	421	423	389	451	331	238	401	623	245	365	217	171	217	357	312	1076	949	1252	969	860	1427
204298_s_at	54	9	24	38	190	203	77	107	62	81	36	94	13	22	14	2	8	13	36	41	51
204299_at	743	363	419	288	746	453	514	484	270	386	439	563	1244	754	914	1042	854	923	1772	931	1937
204300_at	495	179	134	191	312	633	417	342	127	59	246	260	429	494	368	494	519	642	340	275	287
204301_at	217	186	155	191	23	161	203	152	215	168	47	19	5	11	10	129	139	84	224	160	177
204302_s_at	75	63	90	146	205	195	40	48	39	116	43	16	18	56	60	52	57	67	23	49	39
204303_s_at	200	161	198	316	300	418	189	127	225	320	186	249	30	92	92	104	57	80	45	89	34

204304_s_at	987	835	922	2447	104	89	694	494	756	581	85	82	15	9	40	2602	2533	2308	1738	3706	4956
204305_at	988	508	766	878	1131	1406	1012	1106	681	1090	910	1193	691	568	800	537	510	521	812	799	830
204306_s_at	1677	1181	1677	1045	1170	875	1279	1099	918	1199	1681	1022	1582	1464	1454	1297	1330	1181	899	666	588
204307_at	73	57	50	14	82	286	33	63	23	18	196	73	60	134	93	85	112	59	12	64	15
204308_s_at	359	560	216	437	572	743	261	385	161	256	445	659	229	245	261	222	217	238	156	141	133
204309_at	56	23	49	88	148	11	73	131	94	89	68	36	34	27	23	2	3	2	13	9	22
204310_s_at	101	247	39	266	16	11	48	30	171	87	14	15	32	38	45	144	144	85	207	90	105
204311_at	94	32	83	237	23	55	150	133	118	109	142	112	26	49	44	22	28	6	106	39	22
204312_x_at	327	220	402	479	562	648	558	592	570	486	603	640	238	246	228	202	154	208	242	215	223
204313_s_at	427	343	408	516	522	593	392	556	443	541	608	616	504	446	589	733	641	774	826	695	923
204314_s_at	205	223	220	237	308	417	186	319	179	231	244	362	418	124	218	235	214	178	552	387	492
204315_s_at	276	124	384	138	566	198	529	480	440	375	483	346	418	414	444	332	384	380	421	139	160
204316_at	318	485	267	340	195	200	325	393	399	263	216	331	48	65	72	267	178	196	135	167	148
204317_at	228	113	206	40	722	391	205	282	266	189	328	166	233	236	156	111	122	130	196	72	78
204318_s_at	636	186	581	114	515	43	596	736	499	516	823	640	1095	928	658	378	372	465	644	152	188
204319_s_at	1767	2456	1009	1155	537	704	2014	1825	1056	1100	765	1204	310	278	451	2864	3214	2586	1236	1406	1133
204320_at	98	55	90	14	95	20	17	16	21	60	85	83	7	14	6	22	1	3	24	22	33
204321_at	966	572	663	874	630	1082	953	868	796	1097	694	691	352	347	232	364	301	295	488	608	610
204322_at	11	15	11	21	23	23	17	13	12	10	11	16	2	2	3	4	5	5	4	2	2
204323_x_at	142	153	307	507	271	218	224	257	543	362	264	231	23	34	52	26	40	44	49	58	55
204324_s_at	67	13	47	12	88	106	133	79	58	59	23	69	98	138	138	80	117	89	127	76	110
204325_s_at	97	84	376	357	323	156	156	144	326	467	230	229	29	38	58	6	13	44	93	92	73
204326_x_at	9696	5473	1400	1478	4716	6850	6527	7511	2090	1483	4286	7397	7666	5525	7380	7096	7416	6197	3808	3614	1934
204327_s_at	556	575	420	325	388	503	500	454	437	363	469	393	277	243	215	326	290	333	289	318	374
204328_at	609	854	758	993	796	742	769	759	637	950	581	351	186	229	249	271	370	229	332	273	215
204329_s_at	146	123	152	22	89	117	94	9	15	22	146	113	98	44	42	136	129	88	105	114	69
204330_s_at	233	174	168	119	234	243	250	112	274	245	254	138	336	223	127	209	169	202	137	114	86

204331_s_at	3387	3013	4726	3350	3506	3454	3375	2107	5440	4102	4370	3495	7092	4488	2483	1863	1778	1789	3343	2370	1504
204332_s_at	549	339	433	251	513	433	620	400	393	588	482	632	220	300	258	307	299	301	275	452	461
204333_s_at	151	131	75	88	115	285	172	133	86	106	77	197	155	75	116	221	252	177	202	236	444
204334_at	291	339	309	307	335	255	288	446	257	295	246	298	152	88	102	171	197	108	38	43	35
204335_at	413	388	159	113	199	65	268	278	110	109	145	41	304	260	204	241	280	327	224	217	128
204336_s_at	896	833	955	676	1182	903	872	827	919	918	1272	975	770	595	1142	593	468	364	624	621	414
204337_at	42	118	156	25	84	106	123	146	139	98	27	80	5	6	14	6	5	57	6	6	4
204338_s_at	5	4	8	5	10	12	7	4	4	7	6	6	1	6	2	3	3	1	1	1	2
204339_s_at	115	30	66	47	26	60	45	124	56	155	53	48	8	4	11	6	24	8	47	9	6
204340_at	206	358	427	901	558	762	290	385	487	549	406	498	235	227	275	192	185	131	533	523	358
204341_at	1677	1714	1838	1711	1588	2244	1165	949	1361	985	1775	943	962	1113	1416	1321	1508	1244	1832	1116	1026
204342_at	1410	1523	2248	1782	3182	3369	1173	1405	1757	2484	2115	1599	3411	2142	2127	2420	1790	1970	6424	3712	6014
204343_at	153	202	46	118	256	630	85	174	33	108	90	172	57	71	86	101	123	84	57	59	38
204344_s_at	355	326	404	336	452	368	659	639	421	675	547	273	211	220	160	453	347	278	185	94	193
204345_at	132	92	203	641	430	474	122	38	144	137	213	141	228	400	398	61	201	127	193	309	258
204346_s_at	533	437	148	150	33	38	269	195	223	253	827	285	664	752	374	352	452	337	246	282	217
204347_at	341	274	190	185	474	744	433	342	415	347	753	436	344	316	239	303	303	259	259	172	167
204348_s_at	1240	890	659	764	992	663	1355	1153	1231	1107	2446	1421	1164	633	598	1006	674	459	768	287	359
204349_at	197	328	353	582	236	456	156	387	329	235	280	289	608	628	693	562	466	490	1166	633	847
204350_s_at	595	499	621	1179	763	798	657	612	554	636	705	1083	891	841	892	506	600	555	791	431	587
204351_at	29	16	13590	9886	52	67	90	16	6785	6451	30	15	79	59	182	56	60	21	7389	16127	10304
204352_at	243	238	378	476	234	60	289	191	553	439	388	298	198	253	326	293	368	232	570	1232	1540
204353_s_at	329	512	605	701	477	645	606	720	542	514	473	587	321	317	513	670	632	610	643	498	964
204354_at	368	453	628	797	764	914	551	776	524	653	486	412	597	474	598	860	661	839	1370	1397	2488
204355_at	1707	1534	955	885	715	410	1744	1461	1254	969	1418	657	787	882	565	927	793	917	683	427	411
204356_at	24	18	30	16	34	39	69	26	28	38	20	13	3	9	6	5	6	7	7	6	5
204357_s_at	99	283	27	13	672	245	99	36	23	21	311	260	169	98	67	81	29	56	6	43	11

204358_s_at	49	84	23	88	132	60	115	104	183	25	70	10	59	36	29	10	22	69	35	25	19
204359_at	94	6	61	91	59	28	103	54	91	87	75	78	14	16	13	17	13	13	15	2	12
204360_s_at	217	275	252	244	946	1011	276	139	203	173	399	221	366	369	265	154	147	133	170	189	174
204361_s_at	246	162	332	467	469	603	162	256	149	326	446	407	513	441	336	213	188	134	313	293	314
204362_at	409	425	595	1214	1660	1232	492	557	416	643	894	1010	1623	1502	1642	606	642	523	1158	1557	2070
204363_at	706	356	863	608	326	396	624	402	923	1335	325	268	323	348	177	683	587	558	1289	858	988
204364_s_at	303	213	38	113	586	187	376	272	51	59	273	392	117	320	286	117	99	96	12	1	20
204365_s_at	501	390	309	307	643	164	511	524	297	285	465	446	275	313	235	287	243	154	34	41	37
204366_s_at	1688	1682	1811	1862	1310	1197	2251	1974	1976	2180	1804	1472	2208	1874	2937	2438	2428	2565	2806	1940	2667
204367_at	642	534	651	519	867	689	443	651	654	674	689	524	534	594	495	735	753	705	1368	571	802
204368_at	148	235	227	297	103	376	240	333	214	247	273	207	48	38	44	44	46	26	15	9	40
204369_at	201	213	488	305	716	786	241	314	265	345	701	775	400	294	231	148	183	190	203	219	300
204370_at	1106	626	1244	1460	1370	1211	1110	1240	1833	1979	1713	1188	1515	1405	1224	1277	1290	1756	1874	1374	1443
204371_s_at	351	240	496	338	561	348	485	463	621	534	534	226	59	62	52	22	41	22	60	37	31
204372_s_at	3109	2079	2234	1504	1380	938	2418	2586	2218	2368	2280	1387	2733	2235	2810	3202	3442	3281	4181	3265	3088
204373_s_at	392	440	524	442	422	485	600	586	395	527	694	573	620	623	541	364	363	570	420	677	811
204374_s_at	73	224	34	18	47	15	134	79	13	36	70	121	217	172	29	6	32	37	12	13	3
204375_at	210	262	209	194	93	290	138	124	86	141	213	106	116	164	50	24	112	107	111	132	13
204376_at	28	99	49	102	19	12	106	111	143	112	102	98	53	56	33	12	25	25	62	14	82
204377_s_at	191	50	137	124	489	141	146	123	135	106	88	121	120	76	45	58	55	58	79	36	31
204378_at	64	54	321	1388	311	304	58	120	238	231	115	35	37	39	18	26	19	26	479	398	494
204379_s_at	483	858	140	41	146	40	297	377	286	479	95	26	80	115	41	475	467	386	320	250	209
204380_s_at	70	215	126	52	397	403	123	172	119	183	142	14	26	28	13	19	7	12	18	26	3
204381_at	328	178	28	85	484	615	286	249	27	36	158	142	171	46	53	89	106	108	15	17	43
204382_at	767	804	652	534	548	656	792	533	490	458	769	224	635	653	869	395	455	407	323	506	401
204383_at	496	421	457	502	368	285	458	395	432	372	340	214	213	97	176	66	149	182	220	154	201
204384_at	198	240	297	194	19	22	204	247	286	327	219	112	307	111	36	3	3	3	4	47	43

204385_at	179	123	340	326	120	168	295	276	340	242	178	173	65	69	119	47	80	56	140	102	86
204386_s_at	5826	7369	5374	4622	7536	8575	4979	4212	5227	4904	7981	10495	9751	9992	13740	7470	8726	8984	8125	7288	5110
204387_x_at	729	253	779	666	1222	716	425	665	472	833	1002	380	656	370	419	113	211	156	463	219	238
204388_s_at	179	83	137	243	531	380	144	273	187	138	490	308	342	144	132	133	92	111	336	483	537
204389_at	90	64	80	199	290	391	102	92	110	175	441	285	233	189	124	87	80	87	176	171	232
204390_at	7	7	15	9	16	38	4	11	7	8	3	10	7	3	4	4	6	8	11	7	5
204391_x_at	360	272	441	1271	669	1046	313	442	815	528	429	490	607	582	767	504	433	519	1199	1356	1900
204392_at	254	191	190	233	484	563	350	351	128	212	394	246	269	295	219	114	114	108	79	7	52
204393_s_at	25	51	156	270	21	27	60	103	149	89	80	82	11	33	14	9	21	8	608	212	361
204394_at	352	293	898	445	539	806	280	269	862	704	418	229	181	214	299	108	66	122	531	703	808
204395_s_at	433	500	404	322	245	216	464	470	405	798	526	464	60	8	29	34	3	8	38	11	26
204396_s_at	410	554	161	292	267	261	229	375	253	770	406	364	212	196	215	283	325	288	595	373	353
204397_at	142	111	126	274	48	43	183	121	265	349	223	128	13	3	58	8	23	45	13	9	36
204398_s_at	915	1272	494	596	564	1089	669	751	425	591	495	518	211	202	237	288	376	260	203	139	197
204399_s_at	286	667	163	220	56	79	160	202	165	281	215	167	26	19	5	37	51	18	4	36	9
204400_at	11	13	7	10	55	47	11	12	12	18	6	6	5	6	21	3	6	3	4	1	6
204401_at	1918	479	142	235	605	598	762	752	189	189	927	480	433	603	336	582	688	710	107	149	122
204402_at	266	262	296	200	715	634	250	269	405	288	257	261	195	189	78	84	109	53	116	147	62
204403_x_at	492	550	986	979	411	343	542	955	708	1100	473	610	561	380	308	275	220	228	1009	795	866
204404_at	951	680	2134	2897	1008	493	2078	2274	5381	4129	1447	1022	520	804	885	781	820	982	4832	2449	3784
204405_x_at	2052	2820	2484	899	1512	1754	1773	1400	1948	2430	1600	1723	1594	1422	3308	4711	3978	3709	6262	4783	5476
204406_at	20	19	134	18	30	13	24	65	35	21	20	10	3	6	27	19	6	6	5	14	19
204407_at	460	234	339	106	1355	876	337	402	361	368	364	457	721	395	276	421	324	498	1084	555	903
204408_at	876	665	529	618	1849	730	659	940	689	1319	1924	944	961	958	897	412	425	485	425	297	268
204409_s_at	180	116	14	35	58	38	95	135	43	8	6	4	5	30	16	46	50	68	7	4	2
204410_at	71	107	8	19	19	34	78	115	12	13	34	45	2	1	4	17	27	22	2	10	9
204411_at	26	11	22	28	36	26	38	22	29	20	21	34	11	22	66	55	92	20	29	87	110

204412_s_at	21	38	58	31	93	47	12	24	17	38	17	60	15	6	13	22	3	4	9	6	5
204413_at	111	19	203	125	118	106	113	95	144	161	320	93	102	144	78	81	47	40	61	8	7
204414_at	4	2	7	13	33	87	8	8	4	13	7	10	8	12	3	13	1	2	6	2	23
204415_at	356	1789	27	331	56	191	322	499	269	516	367	462	373	314	501	484	3670	19371	369	3785	2557
204416_x_at	28	40	237	511	45	36	27	55	452	396	347	226	345	324	412	7	9	9	1157	724	556
204417_at	73	12	22	12	29	269	49	83	40	78	151	99	116	108	74	2	1	3	32	5	1
204418_x_at	396	485	191	490	1122	929	437	396	364	340	1030	891	166	101	190	119	139	114	74	87	73
204419_x_at	411	617	195	146	54	50	507	578	161	68	219	186	6	4	76	266	343	249	15	22	5
204420_at	2871	1794	4061	48	1770	486	1546	1405	1630	2004	4272	2941	1093	1898	2617	2071	2753	1939	1215	688	656
204421_s_at	30	16	16	10	34	31	44	15	25	13	14	21	2	11	5	68	63	194	4	2	4
204422_s_at	37	22	3	4	106	16	37	38	27	15	3	46	3	19	18	64	93	249	8	16	13
204423_at	331	182	326	313	341	374	408	321	437	416	424	284	426	335	610	321	314	404	581	252	402
204424_s_at	69	122	178	374	69	259	241	272	226	189	213	180	16	15	13	1	3	4	6	11	5
204425_at	167	223	28	76	507	602	37	173	12	20	153	43	192	156	135	199	127	173	53	177	63
204426_at	1947	971	1042	845	911	1630	1520	2114	1041	1426	1133	1631	1271	796	271	1153	940	1448	1201	860	3151
204427_s_at	2826	1581	2159	1368	1115	1485	3026	3034	2955	3275	2437	1919	1741	1534	1049	2448	2090	2399	1885	1881	4171
204428_s_at	13	18	15	19	22	28	8	12	9	10	9	11	29	43	6	2	7	9	4	11	3
204429_s_at	462	209	327	182	735	637	368	330	282	446	333	269	64	60	112	82	87	59	103	95	29
204430_s_at	8	10	9	26	19	32	21	13	13	15	13	8	3	27	7	15	5	2	8	11	7
204431_at	423	743	113	151	206	242	384	414	87	124	114	90	45	48	59	197	191	194	26	72	39
204432_at	483	365	187	168	345	364	302	261	127	205	195	92	209	198	128	298	254	334	178	209	161
204433_s_at	229	187	123	217	305	70	229	197	408	299	638	240	166	225	372	311	198	187	341	191	298
204434_at	230	265	282	410	344	310	299	292	321	334	377	300	176	268	198	275	359	256	250	216	241
204435_at	425	499	684	283	936	796	329	355	334	358	962	1076	975	1365	742	807	891	671	704	728	779
204436_at	695	319	547	532	1076	888	541	652	621	832	681	258	229	196	141	205	184	150	233	144	149
204437_s_at	515	355	445	961	605	2501	558	678	472	502	556	645	414	586	299	206	335	182	222	546	229
204438_at	34	51	165	102	246	269	87	98	79	129	85	57	23	24	7	26	42	14	11	34	23

204439_at	7	10	7	16	11	101	9	8	41	10	9	5	1	2	6	4	5	139	19	751	1079
204440_at	448	320	255	235	737	446	364	373	181	123	458	425	200	129	151	407	557	287	140	67	75
204441_s_at	454	241	850	241	1182	74	423	376	991	841	1505	1458	1025	836	587	189	194	227	565	272	339
204442_x_at	229	640	58	150	583	546	478	472	44	123	259	170	111	134	214	283	246	161	34	76	11
204443_at	130	330	159	616	66	60	292	289	252	211	158	217	39	66	69	75	67	17	35	87	15
204444_at	800	223	1112	225	1027	85	712	827	1224	1255	1126	1064	1392	1227	1144	802	625	755	3257	824	1537
204445_s_at	102	353	189	810	298	290	85	185	312	323	101	16	7	38	3	51	8	11	157	143	171
204446_s_at	161	1631	338	2197	131	59	217	256	312	461	60	5	17	17	18	143	142	112	1669	2091	2180
204447_at	359	336	237	579	164	895	201	255	312	218	274	176	92	61	95	129	159	177	176	360	341
204448_s_at	413	158	391	432	482	375	356	438	508	465	563	461	435	451	460	406	476	353	321	190	320
204449_at	160	123	180	263	466	461	140	286	245	226	153	352	251	153	192	206	127	170	390	294	440
204450_x_at	136	145	109	197	194	110	183	40	153	52	145	21	26	48	24	47	12	50	53	28	15
204451_at	241	293	121	116	388	301	213	343	70	117	321	403	409	362	240	377	402	250	81	91	76
204452_s_at	220	147	113	111	186	133	276	322	155	150	318	251	83	66	22	13	21	16	27	27	29
204453_at	90	153	132	207	192	336	155	182	118	96	124	219	294	362	346	405	329	396	335	419	606
204454_at	175	41	113	98	286	282	179	158	110	187	43	217	32	34	29	43	34	28	43	45	32
204455_at	126	46	133	138	87	43	147	193	166	115	94	54	9	20	25	94	90	139	113	74	93
204456_s_at	7	6	5	8	11	9	7	9	7	8	4	8	2	3	1	2	2	2	1	3	1
204457_s_at	15	7	11	13	23	40	8	4	11	14	9	13	2	19	2	1	1	2	4	6	6
204458_at	160	161	132	255	206	58	148	166	205	228	169	151	94	154	147	80	99	106	90	92	88
204459_at	1359	530	1651	841	1012	589	1173	957	2319	1590	1640	1357	1119	1302	1045	504	663	521	1185	819	608
204460_s_at	482	369	986	546	542	423	386	414	823	1045	765	493	541	567	595	496	449	508	1053	751	612
204461_x_at	705	426	1286	591	871	520	574	647	1351	1310	610	783	1058	646	594	250	283	369	2224	1787	1667
204462_s_at	180	106	144	91	352	243	30	110	104	129	566	584	102	147	112	35	10	42	20	46	9
204463_s_at	53	21	4	5	58	9	16	34	3	6	3	3	1	3	1	2	16	1	1	2	1
204464_s_at	165	209	134	75	65	133	207	191	47	123	58	117	24	19	21	175	180	128	29	29	18
204465_s_at	197	231	190	246	451	434	281	177	202	254	213	231	5	23	5	31	35	31	45	52	29

204466_s_at	216	158	104	84	41	22	140	124	177	158	82	128	4	7	26	105	88	32	27	18	19
204467_s_at	118	46	213	176	324	239	130	115	66	82	99	124	24	4	5	36	7	10	36	31	31
204468_s_at	5	6	4	5	12	11	4	4	4	3	4	4	13	7	2	19	2	3	7	4	8
204469_at	46	2	50	49	78	134	46	26	36	36	72	78	189	227	356	13	30	25	8	38	37
204470_at	61	201	144	113	131	210	94	99	171	92	699	744	42	50	41	110	268	218	367	284	269
204471_at	20	2	5	66	110	58	9	54	43	29	77	45	25	15	11	2	8	3	3	57	88
204472_at	463	434	285	203	192	189	367	557	214	256	186	221	74	36	94	466	569	585	97	111	136
204473_s_at	565	483	1006	758	411	328	575	488	933	915	556	454	129	133	160	126	124	151	340	224	250
204474_at	430	403	206	210	179	116	523	311	218	137	479	393	290	238	258	242	252	265	236	221	240
204475_at	17	11	73	6	8	11	41	1	3	6	36	5	2	19	25	7	17	9	20	18	25
204476_s_at	400	355	602	499	52	39	331	172	524	348	448	278	121	145	66	35	38	92	111	164	141
204477_at	183	255	193	273	231	173	224	240	195	303	159	338	148	245	338	226	155	163	271	288	392
204478_s_at	679	766	890	807	652	513	745	848	886	1033	763	674	502	508	651	585	726	737	880	540	504
204479_at	818	564	456	559	775	653	755	656	508	525	493	736	748	750	705	441	458	482	554	674	507
204480_s_at	2195	6029	2827	8065	929	1129	3676	3251	3353	5491	3259	3207	1392	1164	1842	1716	2107	1001	1637	1925	713
204481_at	414	365	395	294	517	445	376	371	444	403	352	333	344	337	135	219	178	194	375	230	253
204482_at	17	16	16	28	258	98	36	15	32	32	28	19	29	28	10	64	16	52	58	44	4
204483_at	294	105	243	176	29	31	258	169	239	180	193	129	106	150	111	101	135	90	82	201	142
204484_at	689	488	732	1662	625	1136	372	286	961	1476	544	249	276	110	235	356	453	331	798	1114	1044
204485_s_at	1019	1293	1282	1577	922	1027	1224	1301	908	1031	966	958	1227	1354	1696	1915	2246	1522	1830	2170	2919
204486_at	8	34	5	38	157	223	13	5	4	7	6	4	40	8	3	3	15	8	12	4	16
204487_s_at	423	439	11	145	18	16	72	16	11	145	7	8	65	33	5	196	375	114	339	378	184
204488_at	677	592	636	740	687	691	686	724	847	682	1286	787	557	748	784	514	499	424	576	325	266
204489_s_at	2250	1545	2648	1755	1358	2334	2388	3500	3091	4327	2160	1634	716	1403	1562	3969	4042	3129	3519	2922	3517
204490_s_at	2058	1258	2037	1225	1071	1927	1896	3419	2498	3799	1347	1293	624	965	659	1510	1271	1106	1562	1371	1243
204491_at	78	10	187	239	2770	1058	103	75	187	266	2082	1224	3032	2296	2922	60	66	54	178	234	266
204492_at	230	62	210	35	370	81	166	187	217	168	145	160	184	149	122	114	125	170	423	188	102

204493_at	844	950	602	538	542	808	731	689	649	573	864	591	491	394	465	699	736	608	906	540	470
204494_s_at	516	734	575	925	749	515	572	606	629	609	742	455	133	186	178	259	248	186	324	220	192
204495_s_at	523	319	472	563	466	450	494	500	777	514	591	510	256	191	196	243	319	351	439	367	304
204496_at	794	862	510	317	498	638	657	765	298	266	446	282	1070	701	728	1408	1099	1362	1605	1076	1664
204497_at	230	653	12	58	361	880	142	182	7	6	351	187	285	297	321	536	496	284	67	139	149
204498_s_at	276	208	160	228	63	52	288	317	215	229	453	143	62	54	62	5	29	84	66	26	35
204499_at	19	114	115	98	22	60	101	185	166	53	16	72	37	56	87	71	51	57	97	102	80
204500_s_at	398	972	503	433	374	1820	362	685	379	723	539	847	625	461	677	585	470	667	1332	1378	1773
204501_at	267	737	217	194	55	130	330	322	272	175	135	126	32	26	63	47	94	99	34	19	60
204502_at	242	97	233	286	364	349	246	265	268	264	604	534	273	389	477	207	196	451	279	383	343
204503_at	349	414	452	489	509	142	468	607	471	607	375	53	135	69	23	46	127	111	164	136	111
204504_s_at	630	56	362	81	411	73	690	401	491	539	482	816	536	388	229	194	276	261	344	219	178
204505_s_at	775	652	543	398	308	570	739	740	496	429	586	260	188	170	351	186	269	344	224	177	153
204506_at	504	352	327	273	416	343	497	391	384	471	345	314	573	416	446	572	462	446	447	406	330
204507_s_at	474	310	194	172	212	160	451	322	293	257	313	274	470	435	330	627	476	634	490	355	373
204508_s_at	21	18	290	856	14	26	23	29	479	784	16	13	3	3	3	5	8	32	379	375	500
204509_at	183	96	77	127	605	473	172	116	154	112	92	85	11	77	48	6	50	46	43	71	59
204510_at	705	327	711	415	797	356	910	959	1072	788	698	591	655	581	480	673	584	558	1021	418	578
204511_at	254	237	222	634	320	55	182	243	272	359	171	168	20	68	53	75	52	97	125	121	102
204512_at	185	119	98	58	153	144	107	149	110	84	151	153	171	132	80	188	128	141	254	142	302
204513_s_at	57	30	72	84	45	90	93	73	64	77	88	25	12	5	37	1	37	4	20	12	16
204514_at	720	535	539	451	485	419	545	476	668	492	395	327	239	166	155	120	143	136	292	176	248
204515_at	19	12	39	44	16	247	5	13	15	13	4	31	14	35	22	2	2	5	25	1	7
204516_at	93	152	673	516	1043	1013	155	211	281	421	372	402	533	378	230	93	94	160	695	576	636
204517_at	1966	4204	1495	4192	1227	3130	1444	1240	1453	1504	1271	926	1727	2069	1799	3100	3301	3310	3650	4258	4396
204518_s_at	888	1281	769	1652	951	1351	946	921	745	728	641	490	517	1126	753	974	1366	1099	761	1025	781
204519_s_at	263	220	164	440	372	138	253	340	306	328	264	251	49	68	145	163	269	216	308	496	290

204520_x_at	1033	1353	486	510	838	727	507	563	435	678	557	500	327	313	545	678	604	562	605	761	702
204521_at	562	506	764	367	1079	763	490	362	876	618	1035	725	1373	1096	1650	737	672	914	1610	1239	1337
204522_at	168	214	183	226	113	89	142	98	82	137	138	78	186	89	148	108	51	87	119	160	87
204523_at	7	4	342	357	525	794	44	11	249	207	334	504	377	414	547	1	1	1	412	455	698
204524_at	459	586	330	304	643	555	333	355	443	510	304	324	470	486	477	717	603	475	839	840	735
204525_at	20	32	58	74	115	148	42	20	47	39	74	52	90	67	100	8	9	30	71	39	68
204526_s_at	1599	1034	654	932	976	735	1137	1702	1056	1344	992	1553	406	541	513	745	741	687	606	621	686
204527_at	392	348	104	40	335	384	354	281	74	68	281	407	342	388	406	234	222	208	37	60	66
204528_s_at	1725	2946	3201	2125	3039	2008	1712	2205	2873	3112	1997	2534	3426	2606	1792	2981	1845	1981	5183	6213	8155
204529_s_at	33	29	76	75	143	140	73	74	45	66	55	84	6	2	17	8	30	35	21	7	15
204530_s_at	26	24	35	25	25	22	29	28	35	35	41	24	15	21	15	3	19	27	27	24	9
204531_s_at	560	288	514	162	561	11	457	470	657	621	642	699	1051	931	1617	1163	960	1449	2661	1444	2470
204532_x_at	443	279	7862	19136	2775	3515	473	240	8092	11761	1804	533	2079	1984	2048	47	43	60	5960	4342	5731
204533_at	263	120	285	246	345	130	208	298	235	257	195	131	7	7	6	38	10	147	116	88	106
204534_at	71	19	7	131	290	40	125	102	55	57	108	20	3	4	3	3	26	4	6	9	3
204535_s_at	152	58	100	61	82	253	126	22	17	35	37	20	36	92	70	86	87	53	62	51	4
204536_s_at	82	22	96	39	209	214	221	42	41	143	31	13	26	41	11	93	23	57	78	55	69
204537_s_at	83	92	534	1683	18	39	140	82	522	354	114	82	5	2	26	19	16	4	221	141	136
204538_x_at	1020	2341	1792	1997	338	1170	1776	2253	1624	2085	1154	1175	1010	1164	1945	2372	2853	1825	2175	2956	2907
204539_s_at	21	9	22	6	136	202	7	24	7	14	9	6	14	3	13	3	12	21	6	3	9
204540_at	5761	8249	214	529	1498	2161	7400	6314	222	250	1410	916	557	731	2073	7250	5136	7436	305	440	290
204541_at	131	88	170	149	276	95	158	141	80	205	252	160	96	114	155	158	111	90	106	118	40
204542_at	303	372	57	334	88	60	162	384	41	68	41	44	35	50	80	224	161	173	23	131	71
204543_at	30	169	148	34	214	308	142	157	246	182	257	205	2	4	3	5	5	66	10	4	7
204544_at	361	280	711	321	606	746	592	656	685	619	901	1023	876	911	749	496	329	507	658	486	427
204545_at	107	377	75	303	92	216	34	29	23	34	14	21	84	75	80	104	100	130	72	141	65
204546_at	94	285	45	317	224	167	240	252	163	152	99	43	28	11	10	132	107	107	39	107	80

204547_at	499	630	184	1185	487	497	713	843	218	403	358	417	414	493	759	2457	1601	1432	865	639	747
204548_at	22	22	19	84	22	99	28	21	23	22	30	15	5	7	3	3	3	6	4	2	1
204549_at	294	235	316	291	550	302	306	241	324	379	736	381	203	153	184	91	133	93	186	159	128
204550_x_at	261	330	153	282	691	629	443	360	198	236	1096	735	151	54	139	119	57	37	68	95	18
204551_s_at	33	60	18	74	37	55	74	48	50	25	60	49	3	1	36	3	15	16	6	11	17
204552_at	434	314	243	269	219	477	220	338	210	245	262	264	114	138	114	133	100	95	146	51	83
204553_x_at	155	41	90	34	286	340	264	112	47	106	277	46	52	108	18	70	19	46	52	4	2
204554_at	161	217	490	394	396	607	237	224	367	264	416	395	278	320	389	118	146	83	434	437	628
204555_s_at	87	7	175	92	63	31	13	15	191	70	71	116	8	4	11	6	3	7	8	3	5
204556_s_at	155	109	160	224	212	234	82	191	96	92	84	104	35	7	2	11	3	7	18	4	3
204557_s_at	4	2	3	25	38	4	1	3	1	4	1	1	1	2	21	2	10	2	1	11	1
204558_at	434	210	236	141	761	379	394	416	674	348	307	517	327	269	385	211	279	222	326	177	126
204559_s_at	2569	2147	1864	1346	1311	1184	2166	1665	1793	1554	2271	1257	4973	2967	2958	3518	2784	2419	2972	2041	1497
204560_at	13	17	16	10	146	30	323	211	195	197	173	302	37	8	3	38	5	37	7	4	5
204561_x_at	48	43	20	57	84	83	48	41	52	49	53	39	18	15	13	30	16	7	12	7	34
204562_at	183	101	209	21	315	438	174	127	249	232	144	155	65	38	93	72	39	40	62	59	61
204563_at	192	131	80	190	315	137	257	274	197	157	119	60	22	7	19	82	118	132	47	34	29
204564_at	504	650	164	233	348	601	530	540	226	233	206	157	73	95	84	238	295	239	133	84	96
204565_at	1799	1359	829	980	1072	1299	2104	1518	918	914	1145	1343	1038	861	1163	1149	1210	1129	796	716	541
204566_at	1005	1231	315	269	477	403	767	829	356	429	347	586	609	818	1101	3569	3768	3607	1081	1096	1948
204567_s_at	54	99	129	266	82	687	49	78	45	292	75	73	56	108	38	72	64	41	186	242	459
204568_at	508	588	269	460	507	609	551	682	276	314	323	385	364	375	271	458	590	595	219	272	278
204569_at	238	120	171	168	126	97	72	154	50	64	63	53	93	70	50	165	101	142	271	267	284
204570_at	13	12	22	14	34	27	17	21	12	13	13	18	2	7	34	5	4	5	6	6	20
204571_x_at	810	1137	2050	2539	3001	2302	1212	1531	1945	2034	2561	2786	3775	3007	2820	1002	1091	1087	1867	1424	963
204572_s_at	173	303	252	607	471	437	265	425	326	423	486	411	246	296	222	141	181	146	230	143	149
204573_at	93	105	307	410	1203	710	90	17	182	405	860	607	630	1121	725	7	42	41	308	189	203

204574_s_at	262	133	279	124	187	42	249	226	63	345	297
	150	11	49	63	13	7	7	25	13	29	
204575_s_at	20	12	138	71	14	58	29	28	20	27	175
	182	114	123	94	15	76	22	76	32	64	
204576_s_at	603	521	347	475	495	476	774	735	367	335	368
	468	241	174	277	610	902	646	239	254	283	
204577_s_at	204	94	98	65	319	261	182	179	86	123	173
	249	166	192	257	93	84	119	76	78	105	
204578_at	169	243	225	535	36	36	174	257	63	78	242
	220	107	126	97	90	120	70	283	189	127	
204579_at	413	232	370	305	423	437	299	381	330	607	163
	93	148	121	123	419	406	306	988	658	583	
204580_at	147	86	201	83	172	71	114	104	86	148	168
	112	2	22	32	36	7	22	25	39	23	
204581_at	16	10	30	21	18	19	20	22	9	17	11
	46	25	47	39	47	64	7	6	5	4	
204582_s_at	19	11	15	14	52	93	16	13	28	20	14
	23	6	62	22	14	11	29	10	9	9	
204583_x_at	56	27	84	123	111	204	77	25	120	45	97
	31	51	28	35	27	5	23	34	27	4	
204584_at	238	568	76	226	1313	3432	213	339	44	17	2561
	1851	2089	1381	1259	204	199	139	30	135	105	
204585_s_at	16	86	15	22	82	265	33	129	20	27	313
	180	6	4	40	20	7	7	3	7	6	
204586_at	29	24	35	39	25	23	33	28	35	34	36
	30	5	3	7	5	23	7	5	2	7	
204587_at	303	241	301	330	995	683	326	387	372	337	733
	559	710	501	713	421	336	362	501	592	646	
204588_s_at	130	119	754	1883	2825	3075	203	41	631	727	2960
	1012	1352	1384	2347	13	50	8	1118	951	1088	
204589_at	577	502	229	340	1134	2102	583	449	238	165	866
	1129	1199	1197	1154	372	518	335	200	359	358	
204590_x_at	283	237	189	198	322	204	272	113	239	219	209
	225	166	119	200	198	175	182	176	162	129	
204591_at	13	27	5	13	71	90	4	41	7	35	20
	9	19	1	36	3	2	3	26	2	4	
204592_at	126	82	41	76	73	65	115	90	88	105	70
	39	36	36	40	66	92	83	54	46	2	
204593_s_at	885	613	601	400	583	512	942	734	625	520	715
	529	476	343	491	895	835	677	687	741	672	
204594_s_at	787	554	480	557	807	822	803	838	678	506	873
	610	323	285	419	515	522	438	403	531	486	
204595_s_at	210	290	153	141	254	200	354	255	187	229	209
	156	28	66	11	32	45	55	53	58	22	
204596_s_at	147	230	111	151	23	195	117	193	158	150	161
	167	42	49	22	30	8	46	43	4	18	
204597_x_at	38	247	90	59	120	32	231	115	52	56	63
	44	25	38	36	6	21	25	13	4	5	
204598_at	357	248	224	132	341	540	329	396	356	351	223
	302	102	64	120	121	150	144	89	72	124	
204599_s_at	693	665	1209	1117	1012	1058	560	472	1086	668	1033
	474	1397	1292	790	452	449	319	632	503	252	
204600_at	183	154	473	335	863	821	228	259	701	488	381
	265	193	198	119	72	79	96	285	332	347	

204601_at	290	653	165	131	37	35	221	190	244	198	277	216	153	167	143	219	245	260	222	269	290
204602_at	5558	6685	9836	3215	755	398	3254	4301	5662	7340	924	690	1488	2093	1636	14189	15198	15045	11423	5364	6546
204603_at	769	200	505	286	521	56	698	684	1094	660	728	971	426	431	501	399	342	402	775	290	375
204604_at	345	405	106	110	370	297	368	314	84	102	134	143	17	40	42	540	456	339	34	61	65
204605_at	396	460	296	357	535	687	481	509	257	201	397	310	411	505	460	664	634	607	255	381	460
204606_at	102	23	76	96	52	77	49	22	94	22	47	23	6	6	7	15	3	22	6	21	21
204607_at	20	13	2976	10873	38	89	33	22	6710	9719	31	33	44	7	36	5	11	8	8132	3218	2647
204608_at	848	954	1240	1454	1003	1259	584	517	957	1256	804	891	979	1196	686	776	975	650	1412	1165	935
204609_at	11	5	7	6	14	17	30	5	5	14	6	6	2	19	3	12	2	17	14	3	2
204610_s_at	1998	4719	1814	722	936	1256	1817	1788	2300	2850	1059	2042	1905	983	631	432	579	485	567	520	173
204611_s_at	180	151	167	173	263	585	110	71	140	63	125	49	70	83	80	108	90	113	57	114	81
204612_at	315	307	66	91	170	56	274	285	28	190	81	28	20	16	27	222	250	144	27	56	83
204613_at	570	1457	267	278	544	849	550	775	238	232	298	320	119	129	127	451	598	667	84	76	80
204614_at	17	41	95	48	18	43	27	55	78	50	90	121	19	29	28	29	4	13	13	4	17
204615_x_at	2496	1296	2922	1451	2572	443	1412	1672	1971	1467	1285	1097	1939	1378	2176	4861	4332	3918	2647	3824	4491
204616_at	1417	1589	4940	2159	3045	3169	1216	879	3825	3213	3769	2738	5226	5516	6998	1658	1652	2515	5491	4550	4791
204617_s_at	1189	898	827	464	1005	339	864	879	1120	998	861	741	860	898	816	1035	962	1314	1435	1266	950
204618_s_at	500	540	537	437	548	328	644	379	582	616	594	436	575	581	919	1262	1474	1106	991	661	998
204619_s_at	442	103	11	5	77	16	64	145	9	6	7	1	10	20	21	323	313	455	27	21	2
204620_s_at	1476	310	109	132	161	83	867	1057	94	123	114	134	11	22	48	467	566	518	89	46	29
204621_s_at	450	536	191	69	177	120	207	887	24	119	17	19	16	49	52	511	485	733	135	141	268
204622_x_at	672	933	224	166	33	69	258	868	123	165	81	114	37	22	42	1302	1082	1661	352	931	1208
204623_at	95	16	100	282	164	267	76	108	329	284	152	60	21	24	24	22	50	80	189	338	252
204624_at	402	241	312	411	586	449	306	364	359	394	621	561	269	187	179	69	51	55	191	219	316
204625_s_at	45	114	34	81	69	79	42	186	40	43	40	87	9	3	4	22	8	2	5	7	4
204626_s_at	73	101	118	153	102	47	160	185	149	176	215	178	35	6	25	4	14	25	36	30	19
204627_s_at	20	11	4	38	67	124	17	16	5	22	71	23	20	2	6	5	2	10	15	20	3

204628_s_at	198	127	167	180	213	224	130	173	150	144	165	73	86	45	49	29	42	69	86	27	45
204629_at	626	554	255	247	805	923	628	590	218	333	1299	679	376	634	1058	341	331	313	43	162	177
204630_s_at	58	345	1175	1326	935	1136	864	639	1168	984	918	1098	1235	1157	732	371	369	375	1360	1181	936
204631_at	94	46	77	101	256	285	68	107	103	57	94	54	26	31	14	31	30	21	27	31	11
204632_at	803	749	1517	1205	646	402	1224	1040	1357	1638	830	892	286	262	225	166	212	203	393	204	136
204633_s_at	484	633	389	411	757	683	470	581	316	591	864	1403	607	802	774	743	587	640	472	280	553
204634_at	1095	898	654	347	1076	1168	766	772	484	477	877	799	1235	788	956	1269	917	1301	1269	1018	1437
204635_at	386	425	342	344	775	578	223	413	221	467	564	679	309	292	333	415	387	421	400	285	517
204636_at	30	63	351	124	27	23	38	21	537	446	10	10	6	6	4	3	2	4	12	7	13
204637_at	48	33	42	30	95	151	91	38	50	43	28	147	19	2	7	5	26	34	9	31	23
204638_at	97	36	76	65	132	270	52	136	110	106	87	88	31	65	56	38	53	37	48	44	37
204639_at	575	1157	206	107	440	547	394	449	429	379	416	338	214	234	452	540	520	883	263	199	179
204640_s_at	1974	1410	1885	1998	2482	2056	2272	2242	1579	2120	1745	1522	2198	1733	1378	1599	1503	1751	1547	1975	2003
204641_at	1860	461	1799	221	1373	42	1203	1789	1275	1716	1164	1551	1432	1062	907	1292	1234	1401	3339	915	1445
204642_at	30	88	43	59	125	98	28	50	68	38	77	108	10	21	20	1	2	2	10	11	7
204643_s_at	108	319	361	405	816	986	265	416	285	651	516	638	481	393	326	187	242	263	422	317	458
204644_at	123	73	163	207	96	156	160	117	138	207	67	50	107	145	86	111	70	72	118	61	99
204645_at	278	134	323	204	318	312	254	377	278	242	226	183	272	262	238	291	290	283	370	298	448
204646_at	69	32	119	128	48	70	28	53	102	101	33	28	4	9	6	48	28	74	489	411	789
204647_at	515	425	328	188	203	372	639	466	174	186	151	108	209	151	103	287	300	247	93	157	75
204648_at	37	91	91	52	71	163	50	124	17	15	51	20	8	17	68	24	47	28	67	24	35
204649_at	794	344	937	388	944	124	922	945	668	598	917	586	520	615	597	432	575	469	469	320	335
204650_s_at	421	545	419	645	309	310	496	523	460	471	361	337	69	12	81	64	90	124	121	104	45
204651_at	505	338	361	318	639	583	359	451	356	305	328	359	362	362	269	428	489	449	426	302	219
204652_s_at	361	144	283	188	367	167	223	301	158	75	249	170	147	111	159	241	205	254	167	104	108
204653_at	810	1684	128	265	372	472	873	966	229	211	213	238	70	77	84	1382	1308	1106	169	197	269
204654_s_at	369	429	107	186	88	249	348	364	71	95	102	112	26	15	6	98	66	78	18	29	35

204655_at	135	97	109	93	351	281	106	84	150	127	145	181	23	5	7	7	36	213	86	142	197
204656_at	318	197	319	176	487	474	289	425	373	521	499	385	261	196	250	256	219	266	306	212	284
204657_s_at	732	613	589	423	827	386	637	528	553	714	938	484	71	147	278	323	275	279	297	213	166
204658_at	2245	574	1457	983	1941	1255	2107	1767	1406	1357	1084	989	2796	1823	831	1842	1486	1581	2734	1153	1220
204659_s_at	386	538	193	286	341	250	384	399	177	191	293	194	311	215	482	509	613	459	287	373	180
204660_at	24	30	30	31	98	43	21	34	16	27	16	14	22	18	58	23	92	80	21	49	60
204661_at	25	11	39	30	165	355	11	36	11	28	14	28	44	17	19	12	34	5	8	6	2
204662_at	583	263	456	239	709	470	498	548	504	558	540	241	669	573	806	794	721	846	999	828	927
204663_at	69	117	515	504	286	302	68	25	416	531	333	278	203	158	243	38	27	43	470	453	458
204664_at	9	7	8	9	45	1076	11	11	8	8	421	23	101	74	8	3	3	2	4	2	1
204665_at	107	94	167	106	309	148	143	145	182	147	101	87	41	48	157	67	60	45	128	111	41
204666_s_at	171	94	209	78	191	156	193	263	116	175	99	104	33	37	40	61	36	90	104	96	73
204667_at	107	157	1320	1542	169	36	97	120	890	820	158	122	38	19	52	91	38	63	948	710	776
204668_at	193	191	203	58	16	168	211	194	66	126	321	309	112	134	196	163	128	130	55	130	115
204669_s_at	19	229	15	182	25	73	68	16	13	17	372	195	300	366	347	231	241	151	105	415	363
204670_x_at	366	517	454	480	213	32	698	524	433	349	405	397	81	71	105	200	242	173	53	102	34
204671_s_at	194	361	47	12	56	172	264	185	7	24	30	10	9	3	7	291	336	266	2	13	5
204672_s_at	209	169	53	79	111	13	200	214	19	42	119	46	6	23	38	111	169	135	27	47	33
204673_at	81	80	125	166	307	304	178	190	187	236	45	166	60	53	9	10	6	52	27	30	22
204674_at	65	45	140	74	40	32	54	71	51	53	68	46	4	10	43	33	32	42	67	14	11
204675_at	1123	1670	971	997	1583	1340	637	818	1167	1621	1153	1286	1894	495	1083	1176	542	893	2309	1896	2460
204676_at	676	356	534	574	554	622	482	483	903	910	472	349	558	308	314	441	452	352	630	521	360
204677_at	8	16	15	3	77	69	20	17	5	14	16	11	1	3	5	3	3	12	4	6	3
204678_s_at	459	662	625	402	32	66	515	590	591	851	156	137	171	132	57	408	232	368	447	434	353
204679_at	1856	2153	1500	1381	511	798	784	1210	1306	1873	630	545	718	552	503	2803	2966	3094	3744	3870	4049
204680_s_at	214	178	193	159	349	314	189	144	223	205	185	167	57	63	65	49	45	35	40	15	43
204681_s_at	159	158	250	150	206	86	180	239	270	299	186	205	150	119	99	221	123	200	386	113	179

204682_at	255	215	240	288	1128	1041	280	245	198	191	1526	1012	874	929	829	75	126	41	35	59	24
204683_at	220	154	391	261	653	875	174	239	274	224	699	774	500	488	344	14	6	33	157	185	155
204684_at	169	27	141	172	315	177	117	55	87	161	68	88	29	33	17	40	30	118	22	59	61
204685_s_at	143	26	152	71	194	157	118	143	157	166	54	129	29	54	16	30	80	11	36	7	3
204686_at	1330	1081	639	555	429	911	790	842	431	981	156	161	316	337	197	2323	2432	2313	2725	2394	2486
204687_at	489	381	247	458	546	384	533	352	352	324	279	372	50	41	66	80	84	80	113	117	88
204688_at	385	297	9	72	180	224	331	389	15	15	11	59	9	36	51	741	852	577	51	107	119
204689_at	459	345	194	145	467	232	394	352	150	148	425	357	203	135	267	338	201	257	103	154	217
204690_at	582	794	804	1472	979	2451	788	784	498	723	1147	1512	1168	1190	2080	1183	947	885	1013	1334	801
204691_x_at	540	670	495	746	631	820	555	615	565	558	458	391	120	71	187	174	150	147	146	164	162
204692_at	333	280	388	328	558	351	317	265	417	499	303	358	66	149	139	159	132	79	125	122	65
204693_at	2036	1236	1706	442	1009	607	1238	668	823	1037	667	259	65	59	218	522	708	407	244	162	123
204694_at	33	114	14	102	114	48	37	40	32	43	13	14	25	8	38	34	10	19	54	42	25
204695_at	175	74	332	26	509	50	154	194	983	289	777	432	813	1036	575	423	365	266	489	204	139
204696_s_at	98	29	289	136	351	228	189	37	344	217	465	249	77	73	57	30	34	23	43	35	21
204697_s_at	58	39	65	80	107	137	144	185	83	127	91	165	35	44	59	3	10	7	5	16	6
204698_at	15	10	27	21	19	38	25	17	19	24	38	41	109	149	217	87	201	214	62	683	388
204699_s_at	497	502	468	295	564	671	559	529	615	583	360	435	310	292	295	457	437	550	678	576	606
204700_x_at	684	782	636	327	460	540	559	560	640	573	421	564	534	418	596	956	966	853	1180	962	1272
204701_s_at	81	33	53	115	361	484	45	139	48	117	33	25	107	29	37	52	12	30	79	57	44
204702_s_at	393	549	1093	521	4008	2689	518	496	739	1364	2815	3868	4167	3212	1551	415	487	598	1666	795	1548
204703_at	439	412	750	735	812	1158	541	772	440	788	496	651	323	326	423	354	396	423	428	451	524
204704_s_at	1	7	0	3	29	5	1	7	1	3	1	1	11	13	1	2	15	0	1	2	1
204705_x_at	28	15	18	23	44	32	19	20	15	18	14	19	4	8	30	8	6	3	31	6	5
204706_at	667	909	358	537	584	715	705	718	531	440	510	485	372	335	394	449	340	347	374	265	347
204707_s_at	90	103	83	13	54	73	148	123	110	45	60	117	16	3	17	7	3	13	32	11	2
204708_at	20	10	7	10	21	28	109	20	8	10	7	15	14	2	26	3	3	4	15	34	11

204709_s_at	397	118	688	48	847	77	343	866	662	593	591	537	1308	1656	848	864	820	938	2405	380	719
204710_s_at	1187	1798	1687	2570	1226	1283	1646	2301	1531	2837	2842	2568	2960	1698	2792	1334	986	1321	1811	1248	1517
204711_at	344	460	719	652	586	637	582	632	578	639	634	553	336	266	374	269	265	260	340	338	472
204712_at	7	10	19	59	157	151	12	8	29	11	4	34	18	2	14	18	3	17	4	16	4
204713_s_at	46	64	393	671	269	316	239	165	504	914	149	201	2	16	9	5	3	24	191	265	299
204714_s_at	89	122	453	885	37	51	129	62	704	1690	58	85	31	33	16	4	12	13	951	1514	1798
204715_at	541	451	795	864	570	529	766	612	796	913	723	535	570	644	467	500	498	543	865	836	949
204716_at	658	483	1149	1230	500	677	1317	1420	1482	2116	667	623	110	107	186	334	205	364	376	455	470
204717_s_at	396	696	665	418	525	593	195	276	476	577	271	161	212	327	429	393	379	434	574	690	402
204718_at	233	322	1408	1103	613	720	486	327	931	1072	505	275	213	298	405	198	183	165	1006	594	466
204719_at	11	6	7	76	114	77	32	62	52	22	3	40	1	1	11	2	2	1	4	4	2
204720_s_at	340	383	58	10	316	379	227	293	7	89	341	464	507	463	945	862	702	882	218	463	730
204721_s_at	44	16	11	57	95	15	16	82	31	50	18	72	9	27	5	13	8	18	14	2	11
204722_at	20	19	18	31	34	34	65	24	41	17	16	24	8	16	51	36	5	4	13	17	8
204723_at	44	28	49	142	47	48	57	32	32	151	132	124	4	3	11	5	18	13	11	38	31
204724_s_at	105	303	96	40	2403	1845	217	186	166	398	2044	1087	2186	2557	1800	275	137	146	69	87	76
204725_s_at	765	551	1352	1026	1094	1211	917	887	619	1096	955	1327	678	513	622	636	610	808	796	890	1204
204726_at	21	5	20	41	274	280	12	20	21	11	149	210	213	107	210	2	2	3	3	26	3
204727_at	61	61	106	52	133	58	86	103	51	73	38	45	94	53	10	60	52	74	149	55	64
204728_s_at	189	74	369	101	276	187	240	343	229	382	105	234	160	107	205	269	228	283	372	207	207
204729_s_at	607	371	990	402	165	69	451	508	1019	745	470	309	114	114	118	124	103	190	304	215	199
204730_at	340	299	23	150	394	500	367	391	68	112	189	152	95	52	108	160	150	78	77	36	54
204731_at	149	142	141	266	151	360	184	186	294	377	189	124	151	209	156	229	148	131	732	519	840
204732_s_at	140	57	152	67	184	141	133	322	86	103	64	172	167	233	77	469	334	356	152	217	248
204733_at	2813	2572	4927	4590	2492	2324	3266	1746	2376	1031	3702	978	2202	2528	2417	2618	3044	2088	1063	2465	1643
204734_at	328	348	49	48	543	447	311	148	50	56	173	126	186	222	129	189	177	127	20	47	16
204735_at	959	1111	227	215	414	618	1041	944	265	152	651	645	272	186	198	656	669	754	94	132	132

204736_s_at	94	102	24	34	29	22	98	71	111	496	7
	14	1	5	3	27	57	46	74	35	6	
204737_s_at	3	4	5	9	16	47	3	5	13	4	3
	3	1	3	31	2	3	8	5	1	2	
204738_s_at	291	114	315	317	748	500	325	325	288	391	462
	434	227	198	190	132	93	116	161	112	164	
204739_at	165	116	152	122	408	138	464	383	270	203	225
	323	185	192	196	332	313	333	364	182	294	
204740_at	486	539	454	420	602	634	423	321	522	527	435
	222	136	170	279	354	283	310	307	168	141	
204741_at	396	353	271	235	466	529	303	418	56	278	269
	69	92	44	120	202	161	163	23	156	108	
204742_s_at	95	15	209	177	246	177	66	116	215	175	136
	114	196	162	147	55	42	79	403	144	213	
204743_at	220	99	267	308	71	210	160	216	179	259	200
	297	68	56	23	47	62	64	55	92	66	
204744_s_at	8737	9031	9373	2202	5631	8873	14351	7582	8484	4797	3807
	3768	5542	5529	12634	15907	14805	15592	11966	17419	18881	
204745_x_at	8520	4638	3455	2202	2700	3545	5413	8140	2683	2286	3502
	5849	3845	1673	1794	1735	2244	1783	2616	2682	1317	
204746_s_at	142	168	231	248	232	269	294	218	265	235	318
	175	38	108	114	81	78	84	90	66	9	
204747_at	216	230	327	493	346	404	272	247	328	354	232
	255	96	85	86	155	230	799	332	816	907	
204748_at	87	46	852	207	19	20	48	90	90	115	18
	5	37	6	1	30	34	74	2165	1283	2155	
204749_at	5	21	33	17	93	132	28	45	52	32	16
	63	2	23	14	2	2	10	15	17	3	
204750_s_at	26	28	69	146	15	16	136	172	62	68	11
	5	2	4	5	36	1	2	11	6	3	
204751_x_at	486	219	315	295	103	241	603	748	395	316	115
	67	19	20	22	424	569	341	237	311	229	
204752_x_at	1560	1282	1091	917	1142	938	2010	1656	1397	1048	1333
	1165	1138	1112	942	1092	1047	1437	1078	671	819	
204753_s_at	24	88	11	18	118	155	85	120	23	7	11
	45	10	5	10	58	55	38	51	21	23	
204754_at	111	100	92	88	26	20	66	100	51	39	58
	29	4	34	38	57	63	16	41	38	12	
204755_x_at	33	91	12	19	27	79	17	115	98	61	14
	11	4	19	14	62	86	34	35	21	41	
204756_at	159	272	212	184	60	79	159	165	104	186	219
	167	131	85	174	83	76	105	148	166	161	
204757_s_at	306	294	228	131	470	69	325	321	174	180	367
	90	217	132	181	181	221	192	161	269	222	
204758_s_at	20	5	11	27	19	17	30	18	8	8	16
	6	40	15	69	9	13	4	13	18	28	
204759_at	107	127	98	228	322	245	101	156	79	171	161
	337	245	184	346	107	64	141	368	451	494	
204760_s_at	249	280	122	43	29	42	333	450	186	31	193
	13	105	240	163	389	515	438	235	122	182	
204761_at	287	240	246	111	411	360	197	189	181	157	267
	288	408	464	326	484	411	421	564	443	678	
204762_s_at	218	130	175	191	342	293	154	153	201	197	168
	119	38	3	45	27	40	17	49	39	43	

204763_s_at	30	30	28	186	246	200	25	29	131	81	30	145	5	15	29	5	6	16	10	34	5
204764_at	631	580	361	409	81	82	775	796	518	516	449	338	35	4	29	156	98	161	83	16	116
204765_at	437	608	1371	1472	515	934	363	338	1252	1508	914	343	455	346	275	230	248	216	624	360	436
204766_s_at	283	12	492	129	1119	650	254	108	427	342	411	547	602	591	419	236	153	184	304	160	48
204767_s_at	2324	1179	2419	954	3523	820	3140	1919	4600	3483	3378	3362	5592	5791	6200	5627	4699	4360	8749	3502	3359
204768_s_at	2328	841	2622	815	2818	603	2515	2242	4471	3400	3282	2312	5260	2901	691	727	947	1253	1919	561	666
204769_s_at	746	797	246	433	579	460	433	278	269	254	360	269	172	257	306	373	283	291	192	302	162
204770_at	163	164	183	171	305	265	329	280	246	144	432	250	51	46	78	52	86	121	56	72	63
204771_s_at	380	449	461	482	716	535	776	735	603	556	559	610	853	684	860	669	649	687	997	767	657
204772_s_at	415	321	587	338	584	419	619	616	670	512	638	396	625	478	343	388	313	338	447	317	310
204773_at	138	277	22	173	58	81	148	139	147	122	87	97	81	87	62	77	105	40	81	87	67
204774_at	97	54	92	84	125	142	62	141	119	77	84	84	11	15	37	17	3	18	18	23	17
204775_at	550	251	308	113	870	370	731	562	361	366	705	829	436	406	447	414	288	424	336	137	172
204776_at	290	192	225	200	576	505	262	284	238	186	193	88	20	78	103	27	35	104	67	21	38
204777_s_at	142	91	109	147	168	38	228	135	229	177	163	113	57	32	46	28	22	40	51	57	41
204778_x_at	328	459	319	601	658	426	549	623	396	423	844	796	2418	1081	693	744	612	501	586	433	307
204779_s_at	2249	1792	1563	2675	2678	2059	1842	2056	1611	2885	3564	2820	4430	3431	4376	3760	4169	3747	4205	3437	3675
204780_s_at	333	563	353	118	1021	478	538	618	302	391	812	604	854	1236	1590	778	756	1012	572	378	632
204781_s_at	335	336	289	194	526	262	454	362	272	352	598	405	576	954	917	569	616	966	519	265	422
204782_at	58	13	5	10	26	42	68	115	37	46	16	6	39	49	48	135	57	96	60	36	9
204783_at	433	429	170	356	383	398	604	624	143	260	178	197	211	261	278	878	583	889	465	186	371
204784_s_at	696	617	332	592	855	644	1041	1371	306	498	557	587	513	289	318	771	1222	970	463	317	397
204785_x_at	616	687	380	415	579	367	513	717	448	463	782	740	428	474	688	413	437	406	386	410	313
204786_s_at	11	16	16	66	205	243	33	33	36	29	121	129	161	210	98	76	6	32	27	17	49
204787_at	164	34	35	228	355	253	143	216	33	119	152	194	7	40	61	9	3	4	7	22	21
204788_s_at	545	382	378	475	777	641	421	449	264	354	289	540	284	361	303	339	350	304	205	285	236
204789_at	22	24	19	32	304	247	30	17	15	11	316	116	99	154	314	12	41	7	25	62	71

204790_at	302	411	530	388	750	566	378	501	338	349	999	685	554	386	630	201	270	135	307	257	254
204791_at	519	796	553	684	333	559	436	462	385	534	311	552	341	446	660	637	587	607	1012	1033	1535
204792_s_at	93	198	109	106	223	368	30	187	75	36	58	59	35	19	46	138	145	67	95	86	67
204793_at	94	32	90	151	63	172	58	73	144	147	124	57	28	4	18	17	28	55	43	49	92
204794_at	362	240	186	32	51	56	260	293	290	49	142	78	116	212	367	290	269	222	428	243	232
204795_at	513	148	269	189	261	130	444	435	433	288	271	240	245	153	165	465	479	561	538	293	381
204796_at	52	90	34	4	11	30	83	61	15	27	21	16	21	2	29	57	56	52	1	1	11
204797_s_at	304	245	68	69	155	188	216	224	66	87	104	67	44	35	51	235	224	172	16	2	9
204798_at	176	154	505	2312	261	274	262	274	2222	1068	159	138	80	65	113	133	177	142	2795	2096	3177
204799_at	594	428	507	287	488	218	509	534	446	491	425	314	214	173	202	465	481	340	425	243	291
204800_s_at	216	281	236	517	425	458	239	245	215	458	387	348	172	195	170	147	128	103	148	190	178
204801_s_at	46	35	87	21	62	42	12	30	80	22	14	84	45	54	89	8	12	9	32	24	7
204802_at	61	24	4	9	14	17	32	59	16	49	20	19	32	3	19	27	4	27	1	6	9
204803_s_at	146	129	50	19	704	481	115	70	21	31	21	21	23	6	43	31	30	15	8	11	5
204804_at	519	543	1252	2023	423	525	762	653	871	1093	929	772	171	184	211	223	221	469	531	380	506
204805_s_at	1435	2109	2899	2417	1052	985	1387	2330	1575	4574	2314	1912	1289	673	510	469	539	498	1046	772	527
204806_x_at	1339	5175	1588	1834	1561	2598	1685	1672	1437	1980	3260	2036	1493	1388	714	784	864	1292	477	665	668
204807_at	352	472	208	144	216	289	253	231	41	133	186	238	317	247	438	863	797	923	536	726	1173
204808_s_at	785	1512	958	667	844	632	1375	1403	713	1124	759	1181	1137	1524	2136	2483	2580	2345	1302	2698	2409
204809_at	988	706	715	480	865	856	606	684	557	616	586	563	815	774	724	988	794	913	1499	1267	1218
204810_s_at	30	24	38	32	175	77	16	66	37	28	23	39	2	8	3	3	5	3	3	3	1
204811_s_at	333	298	163	141	691	406	505	437	226	232	97	201	51	54	91	154	204	204	132	59	69
204812_at	941	517	1409	791	1293	814	1378	1280	1397	1835	1533	1152	882	684	481	511	508	500	1083	776	618
204813_at	323	285	282	331	488	466	428	327	466	389	335	319	50	89	111	85	69	101	128	175	115
204814_at	60	35	41	89	18	151	41	69	80	140	67	73	13	22	10	17	9	31	13	12	18
204815_s_at	192	113	233	56	322	305	367	59	462	296	367	176	14	77	8	65	65	35	32	39	7
204816_s_at	75	39	39	9	157	206	20	24	36	38	16	8	20	3	41	13	35	23	21	32	3

204817_at	986	408	741	235	1177	108	913	1097	672	737	1654	1548	803	875	970	523	498	571	561	325	254
204818_at	33	15	448	542	27	339	15	18	644	3069	17	34	39	43	17	14	7	1	1767	2031	1610
204819_at	415	332	312	325	389	313	464	445	337	284	327	253	59	76	129	142	142	121	63	93	72
204820_s_at	614	1231	305	349	217	339	485	546	302	288	237	167	91	196	222	933	1249	1541	546	1172	1283
204821_at	19	107	88	116	43	27	21	67	12	17	10	6	16	25	34	44	126	157	52	146	197
204822_at	785	292	795	154	1251	146	906	1194	821	911	1455	1751	1924	1308	1715	1176	1228	1335	2167	652	1130
204823_at	279	277	191	19	48	44	133	41	44	36	34	35	12	5	7	268	293	377	229	251	408
204824_at	386	481	425	366	346	339	441	325	367	306	412	369	373	288	428	262	220	200	339	398	294
204825_at	3161	859	1641	398	4841	667	3362	2673	1761	1649	4433	3608	5110	5677	6540	4761	4185	4565	3035	1722	2051
204826_at	575	296	566	314	638	274	685	538	437	841	490	390	358	433	636	740	763	592	745	297	336
204827_s_at	413	123	480	28	88	48	576	607	361	382	308	308	119	71	67	64	67	80	126	39	39
204828_at	695	544	661	691	573	310	607	718	668	664	551	328	303	295	390	428	449	553	559	548	525
204829_s_at	60	30	148	49	56	34	138	128	67	38	33	31	10	21	17	16	11	12	14	12	3
204830_x_at	77	26	49	19	91	801	30	95	58	117	296	166	166	112	165	5	34	5	6	6	2
204831_at	668	524	513	331	2753	1553	535	454	512	418	1708	1302	4416	4502	3800	741	740	780	1295	1175	2021
204832_s_at	644	1106	734	1013	713	586	606	822	704	637	715	490	355	215	156	445	334	367	495	404	442
204833_at	558	629	290	297	181	230	807	664	234	351	787	540	659	740	380	768	818	1064	410	274	347
204834_at	67	61	79	114	136	99	37	30	170	59	74	90	8	30	31	6	17	3	32	32	11
204835_at	565	226	458	305	991	288	816	680	514	443	1201	935	1158	838	897	397	359	421	652	507	593
204836_at	332	176	54	120	114	235	133	158	123	136	95	85	3	56	3	134	214	155	68	54	50
204837_at	548	300	416	423	566	602	688	735	792	758	378	368	200	185	317	287	263	213	458	418	551
204838_s_at	201	225	134	172	148	35	193	232	24	189	152	189	113	154	111	200	168	214	245	229	258
204839_at	3036	1623	3723	3065	1751	1154	3172	2511	4443	3737	2194	2278	2548	1979	1530	1697	1970	1530	4134	2515	1633
204840_s_at	26	1	28	22	12	12	3	5	3	10	24	3	23	38	3	15	2	19	8	16	15
204841_s_at	81	7	292	135	257	169	175	197	198	180	142	97	63	47	88	82	91	126	157	138	153
204842_x_at	912	689	1358	1073	1445	1594	859	864	1484	1092	1548	1712	1430	1379	1371	630	664	546	1233	881	939
204843_s_at	278	178	450	208	694	544	322	203	433	291	458	325	98	129	136	74	47	96	86	109	113

204844_at	114	168	56	27	74	60	78	112	39	10	37	18	2	1	2	195	160	223	21	14	27
204845_s_at	71	120	5	27	165	188	95	111	24	29	10	4	2	11	16	45	46	50	13	1	7
204846_at	44	38	83	350	19	32	81	25	72	98	70	41	4	13	4	11	3	4	42	26	43
204847_at	818	741	814	601	675	675	644	669	583	648	438	380	585	602	624	1683	1434	1467	1426	1653	2441
204848_x_at	167	473	34	34	45	48	87	108	36	28	38	15	23	30	10	255	312	178	8	67	15
204849_at	896	1438	567	837	847	1911	669	1077	484	897	941	1521	1050	1309	1728	1386	1175	884	1011	1160	1505
204850_s_at	108	57	145	92	261	366	113	80	120	140	74	162	7	17	27	7	10	6	19	19	4
204851_s_at	26	5	45	5	10	9	4	11	8	11	11	48	5	14	3	26	4	2	6	1	1
204852_s_at	183	174	286	261	198	214	219	309	235	207	371	199	48	57	62	7	8	18	43	4	5
204853_at	803	754	788	555	1128	1063	811	607	1144	957	956	808	570	587	665	646	546	497	963	467	664
204854_at	187	280	95	74	59	32	155	198	92	89	149	171	71	25	13	5	5	8	4	4	13
204855_at	575	592	1658	970	73	73	893	889	1667	1460	290	217	115	83	261	687	603	620	2256	1052	1369
204856_at	343	405	525	454	540	199	337	241	535	307	311	265	92	96	151	126	178	116	200	208	136
204857_at	210	262	308	247	323	562	474	338	361	502	787	418	737	823	618	171	208	180	214	144	133
204858_s_at	406	613	134	140	33	250	659	474	227	232	152	227	54	7	19	143	109	136	27	36	29
204859_s_at	254	156	172	243	764	542	142	154	171	189	180	239	238	260	178	157	136	205	306	370	312
204860_s_at	28	4	14	17	19	22	60	170	11	15	11	21	26	21	4	41	18	32	3	9	34
204861_s_at	130	84	15	10	27	157	72	153	9	38	57	127	48	28	72	101	92	90	59	66	50
204862_s_at	1306	2146	673	1267	80	360	811	689	672	451	189	375	291	143	163	612	445	623	622	626	325
204863_s_at	184	62	183	228	158	79	168	218	138	306	399	273	87	52	19	14	14	37	34	44	27
204864_s_at	81	57	270	244	320	392	197	195	233	274	506	290	44	45	42	26	28	15	42	48	33
204865_at	3	21	8	18	16	7	30	25	21	7	0	16	16	1	3	14	1	21	6	15	1
204866_at	374	454	723	436	843	805	306	387	721	763	628	922	880	727	539	525	473	462	1283	986	1204
204867_at	91	101	994	3003	489	349	193	305	863	1620	941	1715	970	490	474	68	55	37	970	970	406
204868_at	2201	1736	1959	1243	2188	2013	2804	2074	2042	1474	2560	1611	3900	3554	2836	1683	1764	1574	1665	1728	1060
204869_at	74	7	64	57	65	153	56	66	29	119	98	10	16	3	49	17	30	25	37	19	23
204870_s_at	7	5	50	27	11	28	17	21	45	61	4	4	1	4	2	2	2	40	7	8	4

204871_at	177	179	194	125	460	357	143	158	278	173	182	289	314	321	196	229	258	281	444	244	255
204872_at	13	36	110	138	357	527	72	44	16	147	277	400	360	205	527	3	24	18	76	199	241
204873_at	106	178	90	103	399	732	90	115	32	74	124	195	415	337	407	344	238	279	451	337	635
204874_x_at	20	12	14	53	22	38	11	50	11	13	11	10	8	12	13	5	12	12	16	10	5
204875_s_at	863	786	1397	2660	335	218	651	596	1480	1163	613	356	543	440	666	711	830	565	1264	1333	1203
204876_at	188	179	212	193	34	30	143	272	163	134	132	143	14	73	9	6	14	17	6	15	13
204877_s_at	267	324	228	277	375	304	314	278	211	233	219	162	105	35	44	160	132	104	50	96	37
204878_s_at	430	116	296	210	359	113	305	201	210	110	107	63	37	64	56	86	66	45	88	61	57
204879_at	12	5	16	12	99	47	28	16	8	10	17	15	7	2	2	12	7	4	4	3	2
204880_at	353	405	559	825	52	55	407	325	626	851	257	263	202	240	204	253	166	199	404	348	184
204881_s_at	459	691	796	1207	1093	1133	763	854	1009	1712	3442	3795	883	1203	508	181	262	246	295	195	185
204882_at	17	17	20	17	55	23	8	20	21	17	13	14	1	7	3	5	2	5	6	6	5
204883_s_at	267	246	449	135	690	228	436	247	571	266	1022	569	509	530	874	403	402	459	702	563	636
204884_s_at	50	29	81	70	78	258	83	153	118	115	102	89	54	28	31	34	21	32	62	38	106
204885_s_at	224	864	53	1504	58	465	135	274	15	29	144	68	68	92	89	181	241	136	57	126	101
204886_at	234	80	144	4	140	23	73	211	106	119	152	152	311	153	173	247	185	282	507	124	224
204887_s_at	523	170	627	40	1019	155	653	814	875	737	857	626	583	572	837	746	770	628	1089	197	379
204888_s_at	115	94	126	109	129	172	81	127	127	116	91	113	23	8	44	7	30	45	36	59	35
204889_s_at	153	101	155	150	242	391	152	179	179	157	200	101	23	42	108	26	47	42	79	46	36
204890_s_at	26	21	38	38	159	117	41	40	90	39	374	206	89	63	78	11	2	24	63	122	112
204891_s_at	46	22	114	43	414	77	60	57	74	92	544	227	314	108	324	41	7	20	168	439	397
204892_x_at	32104	42645	35768	47453	42508	45848	39805	38334	37710	36111	28356	46530	21401	26900	28668	30830	33158	33465	20066	30748	26657
204893_s_at	390	549	458	569	536	653	594	509	388	516	514	478	145	116	132	101	154	128	102	92	117
204894_s_at	98	63	60	41	246	54	48	11	84	57	64	54	13	27	4	68	77	80	53	57	22
204895_x_at	44	40	53	43	74	97	98	41	51	49	9	18	7	7	3	9	35	36	4	4	24
204896_s_at	28	22	26	30	27	30	28	30	25	36	20	26	6	6	9	2	4	4	3	12	7
204897_at	180	179	152	106	80	7	122	137	127	282	60	29	33	23	22	230	212	266	450	229	374

204898_at	123	68	81	153	241	200	174	50	107	180	92	18	44	26	9	24	3	40	36	21	19
204899_s_at	241	207	193	125	907	762	268	117	199	236	409	478	363	313	85	62	66	55	42	61	50
204900_x_at	632	327	237	97	1036	778	364	350	139	298	902	934	1364	1197	998	740	525	552	1011	742	749
204901_at	284	165	137	84	66	149	345	432	165	113	182	230	91	109	71	131	184	185	88	58	92
204902_s_at	381	476	370	488	377	597	134	199	234	356	196	166	92	69	93	96	131	120	121	143	125
204903_x_at	1274	1143	992	878	1042	946	1312	996	1173	1115	925	716	747	717	774	1101	1442	1085	933	807	814
204904_at	54	40	45	107	27	30	45	51	44	55	45	45	5	15	14	11	15	17	66	7	5
204905_s_at	2795	2058	2383	1222	1451	1766	3093	2191	2333	1379	2250	1183	2588	3730	3888	4272	4701	4769	4237	2641	2739
204906_at	398	616	289	285	1454	1137	372	490	316	233	289	308	119	210	207	242	171	188	111	112	151
204907_s_at	315	508	541	573	47	214	327	418	466	654	374	141	7	5	9	6	7	24	46	22	42
204908_s_at	180	617	349	510	103	250	231	223	425	567	291	185	59	73	57	330	300	207	252	219	144
204909_at	159	80	121	66	298	284	107	141	110	21	119	123	35	31	46	41	97	42	30	37	26
204910_s_at	8	15	12	18	56	28	28	26	12	13	23	16	1	3	5	6	2	2	2	7	6
204911_s_at	142	179	180	225	254	241	154	220	143	144	134	134	42	11	5	16	38	11	58	34	11
204912_at	142	91	104	181	366	318	186	135	177	133	148	134	56	49	61	55	45	58	32	59	44
204913_s_at	38	16	58	62	181	136	34	9	41	46	70	64	8	2	31	8	17	2	13	1	8
204914_s_at	13	13	47	18	34	44	12	5	52	15	11	16	1	2	6	3	2	5	4	2	3
204915_s_at	46	12	26	17	30	34	9	16	17	17	10	8	33	27	5	20	24	16	6	18	3
204916_at	622	702	153	522	999	879	444	437	45	21	308	254	281	351	320	563	616	376	140	135	130
204917_s_at	183	264	562	700	63	348	242	400	446	488	198	239	78	58	72	82	80	144	372	526	571
204918_s_at	135	135	296	431	297	171	175	219	309	264	105	72	54	33	54	85	126	51	334	362	490
204919_at	228	150	161	243	705	328	192	248	185	219	171	162	66	43	34	92	78	69	90	94	87
204920_at	570	496	161	84	355	226	550	846	110	143	179	74	48	13	11	502	429	644	55	34	43
204921_at	414	533	471	555	114	126	427	503	213	330	225	303	83	59	111	198	125	188	74	85	160
204922_at	95	131	71	279	234	246	52	140	20	115	257	182	230	383	224	121	96	59	76	116	69
204923_at	172	122	195	248	434	275	187	235	210	271	220	199	51	4	31	33	19	25	25	11	18
204924_at	119	19	22	63	73	138	21	33	21	22	124	183	56	83	128	17	20	2	4	6	4

204925_at	396	308	370	273	18	99	334	360	355	412	354
	361	299	294	205	182	270	267	297	484	478	
204926_at	66	67	30	101	102	161	70	3	54	35	18
	102	7	20	7	15	17	13	18	44	21	
204927_at	579	979	770	1578	821	1056	477	388	665	555	1016
	675	265	274	132	175	97	101	241	247	120	
204928_s_at	419	287	1192	685	788	429	246	219	868	1012	713
	641	541	556	789	359	510	303	1254	978	1253	
204929_s_at	28	26	248	282	834	1123	90	51	179	124	345
	410	332	323	213	30	20	46	104	142	62	
204930_s_at	253	189	103	97	337	318	264	298	178	245	206
	191	322	226	178	242	205	214	196	147	119	
204931_at	8	19	9	14	95	90	29	40	8	50	34
	8	2	3	5	67	34	73	30	4	33	
204932_at	21	26	15	21	194	105	16	30	31	22	78
	49	784	1121	1061	23	45	23	30	43	33	
204933_s_at	9	4	5	9	342	216	4	4	3	6	118
	11	970	1490	644	2	3	18	12	17	1	
204934_s_at	212	30	144	54	291	280	136	116	131	115	131
	190	74	65	83	74	21	77	92	62	88	
204935_at	81	47	54	22	38	39	41	62	41	36	36
	64	126	141	77	74	87	92	237	91	183	
204936_at	19	29	11	47	21	27	21	18	28	11	9
	8	4	46	28	131	187	184	165	50	81	
204937_s_at	503	873	476	404	951	965	437	417	338	289	1066
	999	604	774	892	441	459	427	346	359	501	
204938_s_at	16	10	46	17	21	16	12	13	17	63	11
	20	19	9	2	4	3	2	2	3	4	
204939_s_at	115	12	22	65	19	30	68	99	79	103	65
	59	17	4	9	31	7	3	8	31	35	
204940_at	34	26	4	26	44	8	20	7	4	28	33
	46	7	26	1	1	6	7	6	10	6	
204941_s_at	9	17	9	12	34	60	8	5	8	10	6
	9	1	5	3	5	4	1	1	4	1	
204942_s_at	57	61	119	206	168	94	73	120	116	88	92
	50	29	28	43	27	42	47	56	59	43	
204943_at	201	237	167	200	429	411	237	231	181	208	145
	176	60	98	39	29	45	28	25	16	22	
204944_at	234	258	4	4	188	293	370	309	8	4	232
	172	202	231	352	575	433	362	1	1	1	
204945_at	188	123	138	93	709	312	136	127	131	109	136
	162	53	71	83	53	37	71	88	38	33	
204946_s_at	497	258	446	440	608	374	555	355	360	274	510
	403	236	269	155	163	104	143	167	142	105	
204947_at	280	168	393	322	192	78	318	358	565	749	736
	383	119	283	76	78	167	128	137	84	164	
204948_s_at	385	291	203	63	249	78	321	364	363	319	378
	322	45	45	9	23	48	36	11	44	32	
204949_at	220	130	183	331	383	359	233	145	320	256	337
	251	602	466	510	312	356	147	441	357	157	
204950_at	148	161	178	288	459	374	219	189	193	252	213
	182	122	144	233	106	144	98	186	326	298	
204951_at	26	21	20	39	27	16	32	37	72	57	37
	41	3	5	6	20	8	35	14	7	9	

204952_at	209	43	49	65	81	69	212	177	47	59	217	72	37	44	80	6	59	46	9	6	5
204953_at	112	17	12	67	44	38	21	45	20	48	10	82	2	4	18	13	3	2	7	2	23
204954_s_at	17	13	113	155	392	141	15	22	28	64	16	23	7	7	61	6	6	10	32	11	35
204955_at	1091	1504	117	105	188	328	939	967	90	98	182	216	145	255	286	2085	2288	1968	97	89	103
204956_at	331	153	415	198	212	156	286	388	334	594	117	172	190	121	86	335	195	335	1327	320	438
204957_at	1274	769	1089	707	1992	1301	1834	1502	1113	978	1957	1536	2311	3119	3204	2088	1552	1881	1417	1542	2001
204958_at	332	258	343	138	243	50	352	338	195	264	159	145	93	126	93	215	228	320	116	148	82
204959_at	9	4	5	4	114	40	8	7	55	3	3	5	1	17	7	3	19	2	10	9	5
204960_at	26	15	15	85	43	71	20	36	41	17	14	49	7	4	16	5	5	19	27	6	9
204961_s_at	11	9	12	16	55	113	9	9	16	7	16	10	5	6	3	6	8	7	7	6	3
204962_s_at	1082	338	1222	127	1526	62	884	969	1129	875	2172	1790	3165	3029	3912	1178	1061	1429	2216	548	911
204963_at	5	43	113	142	25	142	12	13	63	164	94	14	48	53	34	33	14	51	367	263	459
204964_s_at	32	11	27	23	98	132	7	44	86	169	24	25	35	33	3	3	7	3	72	129	33
204965_at	73	13	8	65	73	11	57	94	76	122	51	68	17	2	35	1	2	3	18	5	2
204966_at	206	162	114	255	460	318	261	234	95	113	343	166	97	90	57	37	59	68	55	59	59
204967_at	476	538	240	200	529	950	462	488	142	238	686	559	247	259	383	298	379	261	98	99	67
204968_at	434	507	275	550	451	755	485	414	329	482	490	510	121	108	69	106	50	79	88	74	66
204969_s_at	91	68	106	56	56	55	371	363	132	98	303	432	98	127	29	105	93	140	64	24	60
204970_s_at	1124	1578	661	631	786	683	823	567	511	387	495	579	782	687	646	558	434	443	421	521	305
204971_at	595	942	24	144	187	211	1597	516	13	20	27	59	4	5	37	1551	1303	939	25	67	31
204972_at	168	158	186	526	520	1418	99	223	330	342	445	322	317	421	425	37	35	63	130	1090	2384
204973_at	130	125	345	842	1762	785	215	231	421	390	1514	672	1232	1038	597	6	16	31	64	134	82
204974_at	316	354	197	158	613	501	270	413	157	46	134	101	33	67	81	80	260	312	131	92	116
204975_at	851	679	1474	1349	1559	621	867	868	1440	1576	1350	902	1314	1046	1558	674	651	356	1844	1078	750
204976_s_at	581	546	993	532	742	460	825	643	846	552	804	421	1126	1471	1661	1067	1287	852	2581	2188	3361
204977_at	1623	1418	2887	1213	880	951	2274	1804	3496	2890	1337	992	1257	1001	2011	2824	2326	2428	3954	2681	3028
204978_at	976	1271	1031	1230	836	766	1034	738	1005	883	756	955	464	288	302	230	345	139	285	214	140

204979_s_at	26	105	31	26	131	567	36	63	75	74	71	108	128	121	26	88	162	180	6	49	26
204980_at	283	399	1131	830	1190	1337	717	1019	808	901	1198	836	376	493	419	108	17	79	692	445	421
204981_at	953	2063	882	2089	286	1990	815	841	765	1157	480	406	303	416	258	814	863	539	883	1074	819
204982_at	16	16	30	26	21	30	30	30	29	31	27	26	56	58	25	131	94	56	126	21	91
204983_s_at	247	242	347	413	462	425	284	321	306	292	388	366	99	77	78	137	157	174	254	207	223
204984_at	247	364	414	559	294	329	423	211	359	254	313	346	133	82	139	149	217	226	449	520	350
204985_s_at	413	942	617	1547	462	921	356	527	648	2063	716	1234	643	466	259	210	243	132	416	664	248
204986_s_at	310	349	380	374	366	140	254	458	478	418	232	313	11	53	90	71	51	96	49	67	52
204987_at	157	165	228	116	282	265	288	205	226	267	259	178	12	15	9	30	39	29	23	12	29
204988_at	151	19	24	34	213	79	106	15	92	46	18	33	7	22	7	19	7	2	10	7	8
204989_s_at	2094	882	4177	3461	675	477	2764	5318	2406	10291	3088	1477	126	232	235	186	249	265	341	169	310
204990_s_at	2703	1164	3566	4452	1785	950	2283	5005	2430	9504	2717	2678	958	1067	1085	968	820	923	1628	1527	1429
204991_s_at	95	123	85	70	34	74	115	124	63	82	119	103	95	56	47	112	63	60	54	30	43
204992_s_at	7030	7282	8516	8863	7487	7023	11248	7452	10652	6626	9043	6716	7197	7545	7391	6737	5741	6739	7670	5676	5980
204993_at	357	386	222	235	319	511	417	501	203	256	264	216	61	43	76	298	251	194	53	80	64
204994_at	221	107	221	288	500	994	193	245	270	152	328	420	101	144	188	58	47	65	89	428	654
204995_at	169	97	151	92	225	281	83	111	104	134	179	153	217	159	258	246	230	199	456	196	385
204996_s_at	28	9	38	19	18	16	13	16	33	15	38	21	6	7	55	3	6	4	5	9	9
204997_at	57	9	16	27	49	56	12	20	19	32	14	15	5	12	3	4	5	21	18	16	19
204998_s_at	877	715	695	662	990	667	602	453	603	579	858	358	371	349	234	185	180	207	315	313	106
204999_s_at	143	148	210	274	107	289	207	150	171	179	195	161	145	157	108	98	68	87	133	105	56
205000_at	385	226	11	56	66	106	135	162	28	27	68	18	7	4	15	75	58	103	10	19	9
205001_s_at	135	120	65	41	40	94	129	162	40	32	47	44	2	6	11	22	11	7	7	13	7
205002_at	394	355	160	278	22	13	244	285	171	200	257	143	61	98	105	120	114	104	80	131	75
205003_at	107	144	251	163	546	954	146	197	225	287	341	377	603	670	1423	140	131	118	411	478	714
205004_at	569	586	709	736	1035	628	786	697	910	777	664	557	699	572	601	764	571	734	1344	1234	1554
205005_s_at	318	446	267	295	823	2384	265	257	290	517	1163	1067	1105	1327	956	513	680	545	1276	412	719

205006_s_at	291	512	212	219	804	2670	178	276	333	450	763	1212	808	971	836	473	511	457	872	383	649
205007_s_at	210	221	87	147	187	137	439	284	88	103	183	147	62	93	56	126	127	132	41	34	28
205008_s_at	632	624	159	219	396	628	399	826	214	221	324	371	126	192	355	526	513	446	172	192	132
205009_at	144	118	414	266	351	797	187	161	499	196	148	45	23	38	47	44	12	36	210	137	90
205010_at	893	579	1008	691	525	529	791	603	1300	1346	1015	611	400	294	356	290	273	242	661	560	489
205011_at	339	549	256	62	302	167	307	352	211	254	166	225	53	5	11	120	103	103	76	85	50
205012_s_at	640	513	658	494	906	926	551	717	345	742	620	569	564	567	383	350	442	271	278	383	243
205013_s_at	205	191	216	239	315	379	168	162	149	147	95	123	63	53	46	66	108	106	91	71	64
205014_at	507	1113	558	253	67	136	562	467	298	214	172	52	70	107	155	740	788	595	250	478	348
205015_s_at	19	13	28	163	34	38	36	234	120	270	28	23	4	61	53	15	30	25	13	5	7
205016_at	2085	1365	1815	1582	1659	2180	1257	1384	1260	2006	1533	1781	776	843	1176	1958	1775	1434	1745	1445	1809
205017_s_at	184	367	340	292	147	255	310	344	221	377	253	248	27	5	36	73	86	90	44	53	99
205018_s_at	497	880	674	387	341	535	584	780	353	602	379	401	50	28	41	221	189	328	36	74	238
205019_s_at	229	903	833	4913	256	85	444	494	1282	1834	785	564	336	532	358	109	74	112	1171	889	818
205020_s_at	623	651	982	1134	126	340	1363	1341	1018	1082	631	532	1338	1773	2113	1686	1846	1662	1989	2141	2507
205021_s_at	336	431	539	515	528	251	537	519	474	537	705	451	123	111	123	108	91	103	113	160	149
205022_s_at	307	215	153	277	856	796	264	413	127	157	1059	724	1031	1460	1125	126	250	156	176	224	168
205023_at	13	68	88	22	22	13	9	9	91	80	11	82	29	62	25	37	30	27	118	62	66
205024_s_at	1050	893	651	266	1052	300	506	779	669	1072	495	1061	604	634	689	578	577	632	1035	661	550
205025_at	167	303	134	246	32	32	282	166	43	94	118	16	31	67	113	157	229	176	41	102	36
205026_at	78	101	213	191	333	312	72	74	154	218	202	141	28	57	27	23	20	48	51	51	37
205027_s_at	42	79	64	119	98	149	5	24	62	87	129	107	199	144	88	21	28	21	261	195	359
205028_at	46	6	42	41	184	47	5	24	7	28	11	31	29	5	4	6	3	2	4	2	2
205029_s_at	7	6	4	6	43	19	7	7	4	6	6	3	2	2	1	16	10	2	8	2	2
205030_at	161	56	109	149	203	289	138	150	116	164	141	157	14	29	36	30	3	17	57	41	28
205031_at	52	41	38	40	118	1020	28	42	44	48	442	403	323	255	414	7	12	7	13	6	6
205032_at	401	152	1266	498	746	474	555	765	573	995	561	589	172	123	164	180	181	140	229	323	174

205033_s_at	114	102	81	171	214	223	170	161	178	205	139	160	11	4	2	6	2	12	36	41	1
205034_at	439	265	247	109	564	102	280	408	203	260	742	558	1052	887	660	1093	800	697	466	329	488
205035_at	376	412	225	272	77	71	405	405	261	419	260	191	81	108	84	161	224	133	118	40	86
205036_at	1591	871	534	491	871	650	1859	1462	702	732	1420	1162	1876	2081	1428	1566	1981	1936	1584	570	493
205037_at	807	805	308	437	720	1048	731	680	201	177	705	383	300	259	250	270	229	166	84	179	73
205038_at	11	17	8	10	22	95	25	13	12	13	11	14	15	4	9	6	23	35	11	2	5
205039_s_at	257	220	224	114	179	232	227	267	202	335	199	317	83	63	64	70	64	103	33	92	50
205040_at	50	19	18	12	81	34	25	20	21	34	36	26	22	3	9	24	45	31	8	2	18
205041_s_at	36	23	5	32	26	24	13	34	9	11	13	62	2	23	24	47	43	19	2	4	1
205042_at	435	488	490	2008	470	564	339	412	551	991	294	312	305	286	447	1184	856	865	1293	1789	2241
205043_at	16	16	119	1525	30	11	19	21	191	917	21	18	2	4	3	3	4	3	637	772	1016
205044_at	110	68	12	2226	157	31	23	20	19	84	13	26	16	2	7	5	5	12	187	39	102
205045_at	287	75	174	141	147	103	207	214	126	208	223	215	62	69	34	41	37	73	27	31	96
205046_at	333	91	563	21	979	35	553	697	451	422	810	290	686	652	909	377	333	560	765	180	446
205047_s_at	5838	5149	7093	256	3930	8515	9494	2262	1915	600	1340	3011	3061	2372	7857	9908	12404	11104	2459	10635	11353
205048_s_at	2408	2275	1336	414	1413	1486	1079	1330	437	363	587	636	1080	516	860	1244	996	1220	899	1446	1908
205049_s_at	200	73	170	181	323	427	150	104	56	157	55	45	7	7	38	45	7	41	11	5	25
205050_s_at	149	60	146	153	349	318	211	179	140	92	126	122	61	101	95	94	146	130	69	81	60
205051_s_at	78	41	14	35	18	42	73	57	19	32	70	118	2	9	6	6	3	7	29	2	3
205052_at	1380	1814	929	1274	573	726	810	963	838	1027	631	719	685	939	901	1854	1851	1533	1583	1469	1473
205053_at	1095	595	1038	462	1366	868	2038	2160	1391	1244	2035	2291	2674	3315	4164	2883	2739	3364	3124	1879	2587
205054_at	126	101	71	135	522	442	127	75	95	68	101	93	31	16	18	59	47	28	59	27	37
205055_at	2090	1412	1747	1441	2399	1836	2487	2319	2125	1753	4862	4122	4926	5118	5035	3026	4083	3570	3832	2372	1922
205056_s_at	15	52	12	26	34	26	30	30	24	31	16	28	7	6	5	9	9	5	6	6	7
205057_s_at	15	34	14	22	40	32	15	20	13	20	17	15	1	2	7	9	4	25	3	3	3
205058_at	152	102	161	101	466	442	140	121	127	88	51	165	43	59	72	31	73	25	6	47	5
205059_s_at	353	1128	286	614	302	414	342	428	193	457	227	244	90	166	74	177	165	142	115	127	120

205060_at	572	771	754	770	1032	1644	551	435	1034	924	526	295	294	266	301	539	449	364	1087	772	840
205061_s_at	983	515	510	208	460	390	1087	1050	780	605	620	646	1341	1679	1383	3872	2401	3021	1879	614	889
205062_x_at	265	61	285	273	401	316	163	239	162	247	168	141	111	137	172	196	129	126	330	181	306
205063_at	249	147	256	107	227	249	274	288	175	218	129	127	338	198	133	302	292	412	570	299	357
205064_at	131	101	471	449	49	19	143	106	241	154	90	92	4	34	22	21	26	37	32	26	23
205065_at	50	12	24	9	15	27	24	46	24	8	4	5	1	3	4	1	9	1	4	2	1
205066_s_at	167	134	24	27	298	302	156	145	130	52	17	40	5	16	9	84	104	106	39	32	7
205067_at	164	127	263	285	110	120	254	186	272	208	220	199	13	31	50	5	11	14	172	89	123
205068_s_at	343	221	397	396	522	849	578	542	528	809	490	555	198	265	215	141	170	138	331	154	294
205069_s_at	57	56	107	164	113	112	65	80	136	143	55	11	29	50	5	37	5	3	94	55	70
205070_at	282	123	433	563	293	486	313	310	409	468	381	363	830	844	719	314	264	382	653	545	816
205071_x_at	352	184	263	153	139	38	492	474	186	191	193	160	415	288	344	758	726	684	554	242	326
205072_s_at	198	119	87	84	272	87	129	276	104	143	72	102	232	137	113	314	274	285	381	235	243
205073_at	102	99	46	127	58	113	117	63	35	133	41	107	83	44	153	44	30	52	86	93	209
205074_at	471	640	593	741	865	949	701	707	425	523	813	753	533	420	586	252	307	294	281	451	376
205075_at	206	114	271	372	74	211	191	145	150	249	229	176	124	135	151	78	92	93	132	174	126
205076_s_at	245	467	853	697	454	391	274	387	391	530	220	194	58	45	69	125	203	108	340	232	213
205077_s_at	1199	1524	718	857	946	1226	1232	1458	702	1055	692	1168	968	879	1235	2049	1743	1414	1243	1581	1284
205078_at	712	996	606	665	680	692	1026	1405	480	616	688	784	795	563	392	1462	1216	1047	764	701	557
205079_s_at	164	84	118	30	195	230	85	100	33	176	155	136	4	59	38	12	24	23	15	34	18
205080_at	177	114	176	164	290	265	245	144	123	159	163	141	18	25	29	37	17	31	43	37	42
205081_at	2327	2203	1683	8427	1218	1320	1779	1763	1624	1850	1072	1742	4253	2458	1937	1871	2230	1520	3285	2715	941
205082_s_at	13	26	19	21	25	46	60	34	56	25	61	19	23	71	28	15	28	8	23	5	16
205083_at	24	28	118	107	293	242	107	133	20	89	64	139	20	43	6	17	26	7	6	10	26
205084_at	386	294	452	363	1232	1005	429	513	352	451	1077	1149	1500	1988	1176	294	313	601	571	238	599
205085_at	417	134	339	114	246	32	453	342	668	436	337	398	375	294	251	458	328	357	688	279	307
205086_s_at	353	298	339	252	85	42	333	235	340	263	338	229	188	165	188	115	239	217	245	79	84

205087_at	400	180	183	210	583	583	249	274	213	131	176	215	479	322	112	363	229	583	1059	558	698
205088_at	286	206	151	184	654	290	249	282	166	193	539	552	259	274	262	138	132	116	42	7	32
205089_at	667	439	187	221	481	525	685	710	186	198	280	300	254	286	238	669	685	671	324	290	325
205090_s_at	323	450	218	447	319	142	492	377	341	256	372	41	280	211	191	206	315	261	199	252	179
205091_x_at	263	96	381	216	1309	892	269	284	401	456	783	833	1066	545	515	181	206	194	730	491	504
205092_x_at	77	9	9	19	91	133	36	13	13	31	23	48	34	39	30	75	58	68	15	46	52
205093_at	48	19	251	155	349	63	45	21	197	109	61	58	8	63	28	6	12	4	107	56	60
205094_at	729	445	491	586	429	789	473	367	324	391	384	517	305	339	345	392	337	442	475	598	781
205095_s_at	161	347	408	425	183	200	416	625	476	577	499	322	75	86	183	129	62	105	75	93	65
205096_at	249	172	184	106	690	994	260	338	389	211	297	243	77	76	60	108	105	114	103	85	125
205097_at	148	240	170	203	166	300	270	421	244	349	455	581	266	175	279	131	125	86	240	147	199
205098_at	115	67	205	85	250	172	215	215	235	165	178	111	133	115	125	39	23	41	48	45	48
205099_s_at	7	10	16	12	223	44	9	15	17	11	16	15	41	54	3	3	19	2	11	4	4
205100_at	299	282	136	167	408	310	289	400	124	116	182	222	86	97	107	349	335	238	46	38	29
205101_at	19	99	18	31	212	351	40	46	78	60	63	63	1	4	33	20	6	7	4	7	9
205102_at	61	29	122	405	59	196	52	61	177	243	53	43	16	75	78	10	10	92	12	76	94
205103_at	360	275	243	340	285	345	197	276	214	351	283	361	136	79	117	220	263	190	125	290	124
205104_at	126	192	23	74	154	214	66	146	21	43	308	113	102	96	124	104	55	35	26	17	48
205105_at	201	281	2086	2135	910	1532	486	589	2262	3072	715	941	496	695	930	174	153	173	3253	2864	4346
205106_at	196	82	161	220	313	337	144	165	227	232	193	160	124	72	70	57	38	41	134	102	104
205107_s_at	310	247	203	308	404	73	400	336	202	275	362	324	513	415	344	221	246	173	219	99	161
205108_s_at	115	43	132	212	114	108	139	173	127	159	152	94	23	20	5	9	6	4	22	24	8
205109_s_at	398	510	102	122	338	564	425	389	66	84	274	289	147	105	96	114	146	129	13	24	39
205110_s_at	65	35	88	32	598	491	62	45	29	7	561	417	423	417	532	31	30	28	43	26	27
205111_s_at	111	122	231	366	77	34	45	69	154	268	44	116	20	17	56	38	36	51	304	250	278
205112_at	20	89	297	566	136	151	78	17	297	415	47	39	11	8	2	44	17	15	199	155	175
205113_at	89	39	75	28	82	187	73	8	11	34	53	18	5	4	4	5	2	6	20	18	3

205114_s_at	71	96	58	21	140	281	44	120	74	17	74	57	17	7	28	25	3	9	23	19	21
205115_s_at	266	186	438	238	383	78	411	397	644	391	496	356	200	231	366	138	118	136	335	182	180
205116_at	118	127	119	72	293	206	235	124	122	88	64	60	29	35	34	190	152	120	82	65	59
205117_at	44	21	37	21	40	159	86	94	68	14	104	92	9	6	9	34	7	17	17	24	27
205118_at	60	18	42	75	22	19	68	9	20	13	36	30	2	14	7	26	49	3	8	20	3
205119_s_at	102	157	258	53	249	78	260	249	219	61	153	126	7	8	6	6	45	27	34	42	24
205120_s_at	418	472	286	283	334	253	661	688	314	430	381	464	435	517	415	585	587	666	384	270	197
205121_at	107	148	11	69	159	93	136	190	21	64	54	103	3	17	36	31	19	31	18	11	37
205122_at	296	290	68	35	150	336	133	125	36	35	61	72	125	79	49	194	154	227	100	167	271
205123_s_at	146	208	87	59	88	98	113	119	62	60	61	70	47	36	60	160	121	144	51	47	126
205124_at	24	13	15	21	59	58	30	26	9	13	10	20	17	21	4	2	41	7	6	16	9
205125_at	394	734	505	2201	290	758	342	662	549	983	560	760	89	135	120	143	135	161	423	270	208
205126_at	1345	1628	1115	1089	719	969	1472	1028	1272	1191	698	484	687	610	1108	1866	1573	2188	2217	1642	2021
205127_at	50	26	43	107	276	109	23	40	71	53	67	43	22	6	6	8	10	9	16	6	5
205128_x_at	425	310	255	838	241	305	351	354	470	218	350	212	38	74	84	102	131	122	238	175	160
205129_at	3919	2069	1857	848	1083	1240	1978	1499	1734	1019	684	706	961	731	624	2327	2183	1166	1471	1454	540
205130_at	331	214	157	182	120	239	236	214	227	179	246	176	93	129	82	159	140	119	121	144	95
205131_x_at	130	43	81	30	179	46	196	111	54	155	72	55	90	83	140	110	151	56	83	71	34
205132_at	336	165	334	295	335	439	362	425	321	330	254	147	111	59	93	127	110	73	62	120	52
205133_s_at	7277	5971	7657	2037	6679	5967	4244	5628	9487	5644	5534	6394	13152	7879	5681	4997	3995	4689	8730	3592	2799
205134_s_at	519	524	407	287	280	469	685	554	656	484	601	432	1046	668	514	595	754	498	695	346	218
205135_s_at	419	328	491	332	859	731	606	698	740	576	867	617	906	1002	745	1114	1065	1482	1125	799	879
205136_s_at	280	229	246	149	320	441	244	239	550	242	232	173	221	151	170	224	240	188	386	193	281
205137_x_at	269	326	426	2049	352	399	306	149	871	1363	463	383	87	132	309	121	54	50	681	607	535
205138_s_at	11	18	9	17	27	22	13	15	17	32	7	9	4	2	3	2	5	15	79	14	13
205139_s_at	599	766	198	113	136	218	347	467	239	759	121	151	37	39	43	431	439	293	1184	415	379
205140_at	336	341	293	332	213	430	342	391	194	252	189	189	76	60	104	210	179	231	358	232	360

205141_at	201	178	140	334	231	48	272	178	143	84	142	109	52	69	68	133	213	170	90	302	219
205142_x_at	448	527	549	1124	576	449	428	432	349	470	921	314	120	263	172	109	159	121	117	164	94
205143_at	196	83	107	81	254	254	138	170	207	109	144	103	10	9	43	38	32	53	43	42	25
205144_at	300	178	345	275	272	404	321	244	268	288	335	347	57	166	194	125	123	92	115	131	75
205145_s_at	209	226	123	323	426	462	73	146	21	136	124	139	92	124	77	41	30	18	43	114	35
205146_x_at	148	378	53	31	25	30	34	110	20	22	77	24	65	38	10	29	33	48	10	46	3
205147_x_at	32	75	157	85	474	735	193	70	33	84	129	104	32	13	14	36	46	49	48	55	39
205148_s_at	37	28	46	41	192	188	121	100	29	32	145	82	62	81	92	49	68	65	53	77	31
205149_s_at	34	28	52	43	36	43	106	61	39	35	80	36	5	7	2	3	2	2	10	2	4
205150_s_at	90	90	58	142	188	165	105	119	124	127	87	138	1	18	35	21	9	34	53	45	17
205151_s_at	102	168	33	159	60	254	107	153	106	158	153	72	3	32	10	5	14	2	21	31	27
205152_at	48	54	22	57	51	157	36	32	52	63	13	43	12	1	16	22	14	28	1	18	1
205153_s_at	177	280	35	23	16	20	307	336	23	85	24	79	8	9	7	179	214	111	6	4	5
205154_at	44	35	35	45	32	54	42	124	32	28	10	13	7	11	8	17	59	46	7	6	11
205155_s_at	132	60	345	466	168	304	74	17	231	256	141	94	8	9	64	86	19	12	59	84	104
205156_s_at	429	449	282	308	485	355	494	545	641	373	236	14	156	185	184	328	496	321	468	239	362
205157_s_at	19	12	414	1605	76	310	8	8	9	91	60	25	58	8	21	6	6	42	134	154	130
205158_at	191	109	102	885	16	189	164	202	103	105	144	137	50	67	26	149	233	236	52	267	547
205159_at	172	90	107	193	221	136	103	158	150	140	107	119	4	9	30	29	2	35	43	28	1
205160_at	70	118	259	1133	62	429	56	44	472	762	129	114	144	58	134	126	200	106	323	333	431
205161_s_at	9	18	27	279	29	17	34	26	252	231	27	26	65	3	72	11	10	21	131	49	90
205162_at	425	552	377	406	296	427	571	553	407	465	347	215	201	184	238	664	686	625	419	394	587
205163_at	30	36	9	120	22	56	12	37	35	24	24	31	38	27	6	8	6	10	11	44	54
205164_at	2172	724	250	215	36	56	1068	644	40	49	33	33	250	211	22	934	1149	985	189	427	235
205165_at	118	185	216	385	368	477	143	87	96	133	246	235	163	114	66	15	54	11	50	138	48
205166_at	278	404	320	1746	128	129	372	232	157	552	215	278	40	23	43	76	27	77	70	258	176
205167_s_at	284	151	342	147	757	250	339	635	490	432	601	1125	764	720	491	270	358	304	553	219	270

205168_at	52	57	39	28	52	39	111	84	31	20	30	46	2	3	22	17	30	6	27	8	5
205169_at	265	69	156	155	214	300	356	330	190	242	166	178	293	211	141	144	99	155	430	367	403
205170_at	271	150	266	330	426	441	186	368	197	240	237	276	43	46	77	48	90	147	76	55	82
205171_at	356	279	605	595	517	414	546	556	933	693	522	499	248	371	518	438	477	422	943	565	644
205172_x_at	561	679	884	428	1419	1670	670	350	599	573	1694	848	685	526	559	204	283	250	317	256	137
205173_x_at	1711	965	1997	1275	1622	1250	1126	870	1152	1783	1698	978	2585	4719	4920	2866	3238	2657	2512	2237	2572
205174_s_at	5	2	955	656	2565	6448	3	15	879	1325	2334	2237	7664	8659	7401	13	6	2	4789	3590	5490
205175_s_at	15	9	8	9	38	40	12	8	8	7	11	11	69	9	10	6	17	8	37	7	9
205176_s_at	1246	763	1675	949	1095	914	1431	1792	1235	1350	918	1174	1215	1710	1979	2197	2328	2841	3292	1961	2319
205177_at	19	6	14	14	16	32	7	15	13	13	11	9	3	4	5	2	3	2	3	2	3
205178_s_at	479	501	487	534	258	177	488	499	446	246	469	258	569	370	522	763	896	341	637	737	633
205179_s_at	19	16	75	76	190	113	8	21	130	105	81	72	21	42	17	8	42	28	13	51	17
205180_s_at	30	27	28	272	80	253	33	22	161	48	48	97	77	64	30	43	66	68	113	67	11
205181_at	168	164	296	339	242	456	358	136	205	214	283	245	141	161	202	245	274	193	123	218	200
205182_s_at	40	28	42	89	47	54	41	34	44	127	155	26	146	11	70	20	30	44	125	48	122
205183_at	7	6	9	12	34	43	7	8	11	10	9	13	5	2	9	2	2	11	15	5	5
205184_at	376	384	149	182	499	258	437	478	264	236	786	621	820	606	585	192	264	169	145	339	329
205185_at	131	108	232	296	65	163	54	67	210	94	119	69	4	3	2	5	3	32	786	1105	666
205186_at	67	5	8	14	58	43	15	25	15	17	16	15	6	28	3	5	7	6	4	6	4
205187_at	112	56	191	40	131	145	119	131	76	171	155	276	137	134	92	73	37	82	146	81	165
205188_s_at	148	94	201	246	268	60	288	380	289	321	399	435	122	138	52	52	60	109	86	76	105
205189_s_at	700	702	799	616	796	617	598	684	843	804	836	767	326	266	251	210	153	162	285	222	246
205190_at	956	1406	3521	5013	2030	2044	1280	1176	3073	4074	1408	1474	2048	1922	3987	3629	2503	2868	7636	5298	8631
205191_at	180	55	255	180	334	242	45	67	107	200	188	186	487	458	324	220	175	199	1058	744	934
205192_at	382	309	338	261	289	245	254	265	163	273	499	260	177	263	132	82	164	190	88	94	64
205193_at	561	611	803	335	553	781	314	491	256	798	640	728	272	365	333	226	238	251	172	200	114
205194_at	1270	807	1145	454	1555	2450	447	429	510	456	721	779	1896	1352	1035	1493	1106	1320	2331	3493	3281

205195_at	736	389	604	502	685	547	547	313	721	478	863	607	2061	828	487	247	343	208	485	219	161
205196_s_at	898	637	750	879	991	810	1022	622	920	848	2061	882	2401	1413	668	389	413	294	520	317	328
205197_s_at	230	85	321	715	158	337	224	234	403	442	180	201	59	48	44	48	42	59	114	104	157
205198_s_at	192	220	302	930	345	460	179	252	304	310	118	202	89	49	97	170	118	174	486	586	676
205199_at	160	154	612	923	319	313	207	98	1038	1347	308	161	42	44	34	44	15	31	282	159	143
205200_at	32	18	8	63	43	52	33	22	19	20	10	33	8	9	15	37	13	38	20	24	20
205201_at	246	50	231	374	1707	1947	216	199	242	184	1613	1418	1861	1700	1323	23	58	85	109	99	90
205202_at	2570	1639	1283	909	1721	1739	1468	1808	1081	1226	1624	2284	2870	2971	4026	4384	3387	3118	3370	3671	4661
205203_at	344	336	213	467	357	120	280	236	147	429	330	259	92	86	56	51	85	82	178	225	181
205204_at	651	422	1169	1257	227	238	632	627	1390	1133	286	238	183	126	186	374	280	242	1540	485	300
205205_at	241	648	446	366	344	622	298	296	309	435	664	611	136	195	141	142	127	139	75	111	88
205206_at	122	103	130	120	388	277	133	107	84	94	118	113	4	4	3	3	8	6	29	6	19
205207_at	124	99	81	156	250	66	131	100	94	102	94	167	33	14	29	38	38	16	42	42	23
205208_at	67	34	129	116	276	453	20	75	99	141	23	152	66	135	85	68	28	69	110	117	96
205209_at	206	144	220	424	668	826	367	344	338	359	571	538	318	144	99	22	8	9	55	62	21
205210_at	140	57	31	63	36	52	242	108	306	239	365	191	227	118	124	4	3	7	89	112	94
205211_s_at	614	642	529	617	351	184	649	711	554	653	497	236	117	170	149	192	247	260	277	132	93
205212_s_at	110	19	12	88	44	24	77	26	19	15	82	39	55	14	75	60	28	85	47	44	37
205213_at	12	10	34	14	26	35	23	18	21	18	17	14	3	5	3	5	5	3	4	4	4
205214_at	176	78	259	154	168	16	191	174	320	467	21	166	50	45	78	111	70	90	348	71	219
205215_at	143	196	102	141	60	345	156	88	86	88	55	29	76	163	99	224	150	168	163	186	126
205216_s_at	36	144	981	2010	216	191	30	7	1013	1196	92	9	25	21	21	6	39	16	2123	1529	1983
205217_at	329	302	236	220	345	339	468	499	298	407	391	372	530	584	655	562	527	491	648	581	348
205218_at	348	153	134	132	256	152	192	182	134	197	162	160	372	87	248	447	368	317	326	141	199
205219_s_at	250	134	515	352	606	605	276	257	431	449	317	388	351	313	346	180	167	162	685	526	752
205220_at	17	85	142	191	11	97	12	108	230	179	68	57	10	40	9	26	19	14	107	87	105
205221_at	29	9	350	2221	62	47	23	26	280	171	27	10	4	3	3	9	1	11	271	279	249

205222_at	107	148	111	120	225	214	87	177	44	55	117
	305	404	195	162	101	110	121	161	72	141	
205223_at	139	157	76	54	142	188	50	136	41	41	33
	94	56	20	21	81	64	80	57	6	70	
205224_at	287	246	235	119	100	99	268	143	539	477	425
	418	755	457	352	114	125	88	238	277	159	
205225_at	147	79	157	231	180	198	106	139	126	171	152
	195	31	19	30	4	29	44	31	42	15	
205226_at	380	184	61	65	30	50	339	373	13	13	41
	45	5	28	14	397	527	493	69	84	80	
205227_at	26	18	64	76	71	103	83	38	59	45	21
	20	24	32	5	50	54	45	55	85	90	
205228_at	217	349	415	366	381	374	220	131	322	228	496
	611	241	191	195	148	153	109	151	232	189	
205229_s_at	336	561	138	110	290	168	399	488	79	113	102
	167	128	106	106	499	496	269	197	157	129	
205230_at	146	50	88	207	341	361	150	148	150	110	72
	153	26	20	49	78	72	39	93	46	24	
205231_s_at	128	147	126	124	241	271	93	160	72	137	65
	118	95	97	149	159	148	216	118	193	221	
205232_s_at	386	147	448	418	301	453	333	309	214	345	311
	243	51	80	76	90	125	74	83	76	65	
205233_s_at	197	152	155	219	181	89	180	181	91	179	99
	106	65	70	63	105	88	123	81	136	113	
205234_at	1	4	255	420	55	1342	3	1	54	155	95
	143	193	155	129	31	17	44	170	942	1415	
205235_s_at	345	134	1249	34	537	91	290	484	765	864	799
	180	585	648	1095	670	439	897	2240	976	1322	
205236_x_at	261	204	315	378	254	226	252	354	193	506	220
	254	5	63	55	29	15	16	163	82	106	
205237_at	132	119	35	114	392	243	70	115	95	108	90
	93	11	29	18	15	23	25	15	4	9	
205238_at	537	462	992	797	957	984	545	591	1151	1062	1099
	666	217	217	647	223	197	176	425	536	571	
205239_at	7611	5265	1362	312	247	141	3616	5536	524	387	102
	145	146	139	141	5066	4791	7113	1096	2000	2384	
205240_at	549	151	1089	292	3167	981	497	814	1141	1146	3915
	1923	1748	2021	1844	276	252	340	873	206	381	
205241_at	1020	1791	521	486	434	512	788	470	640	493	587
	598	806	677	877	1309	1116	1139	834	1190	678	
205242_at	12	15	19	31	23	22	24	12	11	56	7
	31	3	10	6	5	1	15	1	1	9	
205243_at	40	50	128	257	41	58	184	104	198	64	153
	114	76	8	107	40	23	43	10	7	7	
205244_s_at	24	10	15	19	66	103	13	18	13	17	31
	73	6	8	5	5	13	6	11	4	2	
205245_at	336	293	410	441	263	419	343	277	488	679	240
	231	150	138	190	207	206	149	437	400	184	
205246_at	351	214	456	501	467	488	481	401	429	373	540
	483	493	503	479	378	303	467	360	368	416	
205247_at	131	64	140	67	315	245	90	91	100	106	139
	77	94	49	77	53	74	49	10	37	37	
205248_at	274	247	256	295	335	501	241	342	178	300	215
	377	153	113	90	55	83	34	107	195	137	

205249_at	147	21	60	35	95	34	94	220	116	191	104	35	121	83	203	469	264	446	360	673	1012
205250_s_at	161	232	156	89	143	93	107	164	79	190	115	57	148	59	153	265	206	279	313	262	269
205251_at	755	757	404	587	405	630	539	422	391	498	340	373	241	292	462	674	637	719	1855	1721	1873
205252_at	278	231	232	323	334	400	252	256	231	240	253	276	136	133	94	103	144	160	136	129	106
205253_at	13	28	5	132	92	39	201	131	5	4	4	6	3	2	8	70	90	84	10	32	7
205254_x_at	102	77	308	419	363	468	241	208	537	588	426	187	113	143	118	58	30	82	226	64	51
205255_x_at	849	1001	2345	3867	2994	2548	1015	1288	2791	3494	3071	2240	2435	2510	2737	757	788	1204	6360	3841	3774
205256_at	270	242	191	230	595	581	260	216	178	180	244	263	140	133	215	213	250	245	275	244	229
205257_s_at	148	84	189	78	329	301	126	61	76	109	104	82	28	35	5	26	50	47	27	19	11
205258_at	30	49	54	27	1084	1982	86	94	108	101	952	1067	1096	446	349	37	43	6	73	195	171
205259_at	111	111	68	71	49	28	139	133	25	42	27	72	29	18	17	72	77	8	35	67	47
205260_s_at	495	355	536	325	700	528	474	719	416	658	790	728	1821	968	932	885	556	701	1250	682	415
205261_at	205	212	197	273	348	156	187	198	225	144	291	151	107	98	88	21	10	46	81	74	37
205262_at	111	319	20	16	38	142	225	148	15	21	28	19	32	61	57	176	146	99	8	91	47
205263_at	1371	1578	2040	1164	1072	1434	939	938	908	1350	893	1277	856	516	523	1301	1120	1258	2598	2606	3410
205264_at	1447	727	1337	510	869	786	1215	1071	1699	1593	1045	992	822	761	862	1142	885	983	5304	1411	1752
205265_s_at	456	575	46	38	41	70	555	464	56	39	317	142	51	35	34	88	107	115	47	10	17
205266_at	1823	783	449	188	932	410	718	789	365	408	1042	723	649	482	570	473	629	486	227	257	229
205267_at	61	88	88	79	128	103	91	95	96	115	82	79	17	20	17	3	24	8	141	94	129
205268_s_at	28	23	104	466	51	31	28	20	75	35	27	19	4	24	8	11	37	6	41	103	64
205269_at	30	1	30	22	80	125	24	1	11	21	34	4	7	1	2	10	13	2	4	9	6
205270_s_at	41	56	15	21	77	172	29	13	23	18	16	41	4	3	2	3	1	2	5	4	4
205271_s_at	198	184	179	305	181	122	224	162	150	196	198	147	69	74	85	61	133	99	55	109	80
205272_s_at	12	13	9	13	49	63	8	15	9	10	11	13	5	11	7	10	5	7	6	4	5
205273_s_at	1967	816	2676	1423	1060	1103	1336	990	2535	2679	728	753	416	316	497	725	686	795	1487	952	1194
205274_at	28	24	47	70	33	17	29	46	64	31	28	33	6	5	7	4	3	4	9	6	3
205275_at	93	77	30	21	121	36	19	17	39	15	21	33	51	13	63	12	46	20	27	41	28

205276_s_at	163	182	339	127	405	304	241	251	183	179	362	137	8	45	51	19	50	8	26	13	7
205277_at	366	293	342	348	503	578	363	314	325	422	368	347	59	103	73	70	87	58	68	117	98
205278_at	77	283	103	304	668	766	117	106	198	169	343	210	306	438	574	97	120	82	175	295	462
205279_s_at	131	44	83	176	280	112	133	141	118	131	141	132	19	24	36	27	21	16	39	41	33
205280_at	16	6	3	3	7	9	3	3	4	6	6	25	1	4	3	1	10	1	1	2	1
205281_s_at	115	66	319	217	318	332	199	145	127	168	266	292	438	374	325	302	261	423	538	363	634
205282_at	929	607	2872	1309	921	688	796	681	3920	5396	743	406	615	531	616	671	510	552	3024	1362	1771
205283_at	331	315	308	330	265	297	452	474	363	418	387	337	242	260	347	318	374	456	495	450	741
205284_at	787	673	681	268	759	679	677	579	1034	738	786	437	359	379	526	734	561	655	725	554	425
205285_s_at	40	30	30	31	21	17	101	107	29	25	141	33	4	3	6	13	2	3	6	2	2
205286_at	316	1134	103	79	547	1410	600	371	29	34	897	899	448	452	992	683	499	455	36	29	28
205287_s_at	60	67	71	14	45	42	123	63	11	21	109	64	23	57	30	51	59	23	7	10	2
205288_at	69	52	71	57	106	152	38	25	28	38	41	79	107	82	70	71	30	50	58	140	104
205289_at	32	13	75	171	32	17	29	9	35	157	18	83	14	13	3	2	17	3	189	204	490
205290_s_at	143	43	75	318	114	78	109	69	127	182	104	77	4	36	30	4	9	3	101	157	167
205291_at	388	332	362	248	660	293	311	387	355	457	556	457	75	76	94	51	130	107	79	47	78
205292_s_at	11163	3758	10415	3983	16556	4275	10494	9563	11545	10274	9596	7823	16499	16342	11505	11343	8786	12990	15354	8553	10030
205293_x_at	394	417	34	50	40	44	379	309	99	55	215	152	74	72	120	185	217	196	69	40	64
205294_at	663	554	269	194	471	431	904	599	412	481	654	308	192	164	184	201	212	226	127	100	51
205295_at	119	169	155	136	606	426	176	239	103	225	554	424	611	891	1061	26	7	15	57	10	30
205296_at	131	60	111	59	436	179	91	137	106	105	172	254	376	237	141	125	91	183	262	110	176
205297_s_at	11	9	22	16	437	567	23	120	28	22	47	20	19	27	35	45	59	53	25	20	7
205298_s_at	308	399	305	294	471	839	423	461	293	273	360	317	206	199	259	368	430	425	161	159	179
205299_s_at	4	38	11	87	19	121	46	33	5	60	63	40	27	25	9	33	54	23	27	29	28
205300_s_at	164	94	233	380	562	439	164	220	463	144	216	283	196	224	137	196	189	138	257	139	171
205301_s_at	149	63	182	123	118	243	89	84	98	144	129	101	86	194	164	128	157	185	252	137	157
205302_at	56	63	98	28	111	95	41	48	11	53	33	58	47	33	42	28	6	2	23	84	78

205303_at	34	29	54	23	81	222	233	154	40	113	101	98	156	214	283	3	24	4	7	5	5
205304_s_at	12	12	27	76	199	501	24	13	8	8	10	10	111	135	144	2	8	1	6	3	25
205305_at	194	102	151	147	102	211	158	190	131	122	166	122	14	23	68	51	62	37	127	162	173
205306_x_at	98	89	122	162	253	175	138	154	198	214	112	101	19	34	3	26	43	30	17	2	3
205307_s_at	77	86	16	63	235	332	72	75	78	75	55	109	4	2	35	27	12	45	20	26	21
205308_at	261	328	199	172	401	289	295	439	174	221	249	325	217	185	322	277	202	142	231	145	94
205309_at	101	460	183	151	11	15	248	36	297	116	9	6	33	146	61	241	223	328	255	180	188
205310_at	411	523	632	852	528	447	525	545	565	724	645	681	284	335	596	501	449	433	599	696	705
205311_at	79	10	604	441	18	16	44	25	979	432	20	15	10	7	22	6	29	44	2945	1316	1780
205312_at	7	19	8	8	132	11	8	18	7	10	11	77	4	4	5	7	5	6	3	4	1
205313_at	445	347	990	3562	335	355	262	346	1121	1744	391	294	22	33	47	104	138	102	1003	752	923
205314_x_at	5	5	8	9	10	11	4	4	4	7	4	4	2	3	3	2	3	4	3	2	3
205315_s_at	266	316	579	610	404	289	379	484	578	594	709	617	481	648	1121	618	729	312	969	904	584
205316_at	114	64	149	318	96	195	77	104	151	140	94	109	65	74	42	29	33	21	254	203	228
205317_s_at	175	63	274	220	223	99	76	121	80	119	135	143	53	36	40	45	31	45	105	107	64
205318_at	276	180	157	432	269	146	196	150	190	176	112	122	24	33	30	30	53	49	38	91	25
205319_at	52	109	9	54	214	284	97	77	58	15	67	24	24	24	25	28	14	7	53	32	28
205320_at	90	99	52	56	432	149	81	42	104	70	98	33	40	60	83	54	83	102	70	58	69
205321_at	5107	841	510	561	2790	2805	3187	2155	954	682	2609	2011	2722	3372	4900	4186	4233	3821	1641	2152	3516
205322_s_at	407	348	444	398	924	873	494	400	347	375	374	236	149	206	250	272	190	333	343	396	391
205323_s_at	360	347	657	519	707	392	584	462	779	643	479	383	169	166	171	195	154	176	185	182	169
205324_s_at	2252	1556	2561	1470	2220	4010	1476	1521	2339	2007	3361	2199	2768	4213	3471	2807	3396	2842	3615	3536	3717
205325_at	15	39	39	123	216	97	42	66	100	35	71	13	2	12	6	29	17	23	6	4	5
205326_at	331	186	279	283	320	335	299	238	254	271	163	238	47	35	45	9	12	50	36	67	41
205327_s_at	639	418	452	454	667	648	666	636	524	498	529	310	228	239	387	514	558	658	449	398	597
205328_at	220	157	178	256	287	269	298	260	163	277	230	235	20	29	54	47	63	48	57	27	54
205329_s_at	368	200	332	318	536	509	322	334	479	482	480	249	372	652	578	467	456	476	556	355	700

205330_at	209	141	79	59	87	71	246	259	40	165	246
	172	101	58	42	88	59	71	20	6	4	
205331_s_at	34	50	73	66	454	808	66	74	70	39	131
	312	51	112	132	109	135	7	6	17	15	
205332_at	38	26	30	22	302	156	19	11	13	20	21
	15	8	8	9	68	19	66	10	14	4	
205333_s_at	479	648	756	1220	140	419	582	701	1136	801	631
	263	127	210	178	148	198	200	421	244	221	
205334_at	233	131	248	238	254	192	130	226	206	180	232
	114	73	42	3	55	60	22	76	70	49	
205335_s_at	3618	3233	2608	1914	2423	2814	2726	2453	2260	1850	2567
	1901	4576	4750	6350	6450	6243	7784	4583	4866	4082	
205336_at	30	52	26	27	48	42	76	26	27	29	14
	109	12	43	17	10	3	11	7	9	15	
205337_at	58	49	64	5	55	28	50	8	43	29	9
	34	1	29	14	10	2	14	4	4	19	
205338_s_at	312	101	221	273	396	414	232	234	278	274	274
	244	28	24	23	19	37	79	27	41	31	
205339_at	1511	382	867	352	1761	550	816	872	685	771	767
	1116	1203	840	671	1088	942	1264	1640	903	1329	
205340_at	847	446	594	307	524	574	563	495	831	513	458
	416	317	135	239	607	640	559	750	618	774	
205341_at	316	333	553	334	195	237	245	255	293	674	209
	137	42	33	105	138	117	155	86	107	63	
205342_s_at	288	265	307	473	136	308	449	434	197	291	128
	106	29	43	26	107	88	79	275	106	181	
205343_at	114	85	66	217	29	128	180	220	83	141	77
	117	6	11	9	29	26	22	62	91	66	
205344_at	28	24	15	22	32	31	27	51	33	42	16
	16	17	5	8	10	7	13	16	4	7	
205345_at	48	111	453	146	893	59	256	255	425	454	691
	738	601	560	374	76	129	103	591	146	250	
205346_at	87	60	71	102	85	118	60	87	195	274	155
	23	14	27	14	29	42	12	57	47	53	
205347_s_at	679	567	137	98	184	262	1630	2411	96	123	114
	148	62	45	44	1687	2138	2203	20	22	4	
205348_s_at	201	49	209	54	454	278	172	135	197	204	219
	194	51	89	92	32	23	23	177	117	129	
205349_at	57	58	256	325	155	196	101	112	181	172	97
	33	14	32	30	44	5	25	177	203	195	
205350_at	22	57	38	50	95	38	16	16	90	119	43
	59	62	51	82	34	4	8	58	4	45	
205351_at	515	219	506	441	488	300	477	359	347	463	347
	412	248	282	238	233	243	224	217	252	262	
205352_at	460	247	81	91	246	375	270	344	27	101	51
	230	116	150	128	331	358	409	34	157	335	
205353_s_at	4215	4038	3178	3037	1706	1222	3075	3711	3024	2869	2184
	1286	2603	2251	2054	3770	3444	3688	3258	1749	1636	
205354_at	1445	1296	16	26	136	103	1110	585	12	28	352
	332	190	247	123	641	633	534	6	42	15	
205355_at	253	220	62	50	183	321	155	173	20	36	102
	172	184	133	175	206	125	240	144	101	86	
205356_at	1595	1305	1162	472	1615	859	1848	1679	966	850	1681
	1499	584	608	790	771	663	538	892	561	623	

205357_s_at	116	92	99	80	290	327	74	73	163	110	105	137	6	12	33	18	18	19	27	34	7
205358_at	8	5	5	6	12	11	5	5	7	6	7	8	1	1	5	2	1	3	2	2	2
205359_at	155	136	159	177	247	257	280	255	162	197	190	191	19	13	31	24	2	20	31	22	27
205360_at	146	11	90	72	164	43	95	92	63	92	128	59	10	8	29	24	24	72	46	24	8
205361_s_at	1738	1676	2476	1714	2851	3635	1401	1611	1694	1570	2155	2366	5366	4433	3951	3496	2753	3768	5481	4056	5432
205362_s_at	175	230	198	194	870	743	199	185	126	138	114	217	245	441	514	465	480	435	356	353	364
205363_at	11	7	28	92	110	56	25	13	12	39	6	9	4	3	3	2	6	23	11	1	2
205364_at	1107	82	22	113	150	153	832	575	52	55	188	139	69	48	81	248	494	343	19	65	24
205365_at	172	158	320	472	375	265	231	245	530	1034	712	292	69	52	36	14	47	5	41	31	36
205366_s_at	325	442	1432	2393	2513	1570	239	412	1066	2094	2642	2392	2250	1985	1340	178	158	88	770	966	739
205367_at	541	898	576	570	337	293	965	1144	682	528	543	455	104	123	212	219	185	269	189	174	131
205368_at	49	41	71	22	91	163	68	37	70	21	37	84	18	56	36	31	38	30	23	58	50
205369_x_at	220	95	368	508	51	175	286	311	400	289	149	187	60	72	93	143	166	135	204	188	298
205370_x_at	2011	1532	2250	3315	1475	1560	1326	2123	1881	2253	2048	2090	1143	1061	2007	1127	1139	1816	2748	3069	3302
205371_s_at	238	54	271	332	176	173	180	195	167	183	122	58	105	88	62	95	70	94	262	260	312
205372_at	11	6	165	147	125	293	7	45	79	113	117	106	59	116	135	19	19	26	68	91	174
205373_at	11	5	11	53	11	24	19	7	28	31	7	6	4	6	14	23	4	26	16	31	24
205374_at	324	191	381	182	532	321	337	389	245	328	267	269	21	53	67	54	56	54	63	72	49
205375_at	237	210	171	133	142	47	208	172	87	89	217	68	121	70	64	80	101	58	87	42	33
205376_at	116	141	254	300	110	125	106	119	215	119	85	62	85	116	133	771	763	745	470	401	656
205377_s_at	26	12	27	30	55	66	38	40	31	36	36	24	24	5	3	5	17	7	10	5	5
205378_s_at	20	13	11	25	11	24	12	11	5	17	16	23	2	3	3	5	8	4	3	5	3
205379_at	25	83	34	67	1170	560	101	124	24	24	1370	1361	1326	895	739	101	95	62	13	35	21
205380_at	65	75	96	775	32	97	12	75	104	105	85	41	22	31	25	29	3	5	680	188	296
205381_at	102	91	100	83	143	165	90	108	91	113	75	101	32	9	46	3	33	50	22	16	35
205382_s_at	923	1130	292	3457	81	27	1061	1027	215	324	101	287	194	102	61	451	580	491	175	219	122
205383_s_at	1499	3417	281	676	235	392	983	1081	198	331	203	284	49	30	73	676	651	667	100	184	118

205384_at	228	210	69	154	239	380	151	245	123	165	114	58	17	43	10	116	137	73	58	46	8
205385_at	110	56	8	35	8	35	44	30	84	77	30	26	5	8	6	10	27	7	4	2	24
205386_s_at	108	88	42	98	159	114	102	120	55	43	37	72	14	2	21	40	21	12	28	2	24
205387_s_at	26	39	15	57	44	187	15	18	7	11	21	9	7	12	18	48	132	19	9	6	7
205388_at	61	68	133	35	29	23	77	121	60	134	108	155	5	8	25	22	44	7	4	28	6
205389_s_at	108	84	26	18	340	151	85	111	33	18	17	14	17	7	27	15	29	32	23	6	5
205390_s_at	26	12	9	30	21	12	30	29	23	18	13	20	6	10	13	6	7	6	4	7	3
205391_x_at	17	17	19	25	19	22	27	25	27	20	11	15	4	4	10	15	9	33	5	4	9
205392_s_at	30	30	69	375	159	144	119	29	264	306	24	211	7	25	44	12	12	46	109	131	61
205393_s_at	386	174	657	114	948	271	542	689	621	570	843	1299	1037	818	517	533	397	614	1137	448	759
205394_at	610	488	920	321	1270	304	1288	990	1054	1157	1266	1298	1588	1251	1047	1076	1107	1269	2439	873	1162
205395_s_at	179	224	1065	480	683	245	168	265	830	945	446	656	731	520	740	282	281	338	1825	1590	2040
205396_at	540	234	724	533	482	434	386	492	645	878	453	328	30	46	44	59	48	100	69	47	79
205397_x_at	103	90	1266	684	30	87	289	170	460	1099	819	153	6	10	11	22	28	21	21	21	17
205398_s_at	560	490	1527	714	93	67	636	569	954	1360	902	650	110	137	244	533	450	585	419	228	380
205399_at	70	38	72	47	2305	168	38	74	63	68	1593	1110	2085	2748	1524	213	409	269	17	20	23
205400_at	19	18	15	98	96	108	65	108	33	50	68	119	22	19	19	11	32	22	5	14	17
205401_at	808	645	1055	497	1135	883	1776	1149	1608	1248	1349	845	573	531	577	541	522	607	824	426	610
205402_x_at	483	230	3352	3811	568	501	282	404	1058	1071	456	317	275	171	191	158	148	199	1040	11090	6312
205403_at	12	12	422	469	91	81	13	11	451	133	10	8	20	7	3	18	16	6	919	804	555
205404_at	171	54	141	149	265	56	110	67	127	151	114	25	28	20	35	43	10	20	25	3	25
205405_at	105	10	31	105	38	42	208	104	130	71	74	142	35	25	8	25	10	9	4	11	7
205406_s_at	324	296	270	467	727	532	501	575	262	380	348	420	416	320	563	452	450	404	542	333	308
205407_at	57	45	41	8	27	136	73	75	1	49	45	139	34	62	28	78	63	49	13	9	9
205408_at	622	341	509	367	396	527	598	647	462	479	436	429	137	135	114	281	176	231	257	257	336
205409_at	134	240	213	215	92	356	211	145	161	75	323	122	50	38	48	26	14	3	51	8	3
205410_s_at	192	50	151	407	49	26	350	383	209	259	192	211	11	6	8	3	6	5	10	2	21

205411_at	238	129	243	212	386	318	217	241	300	287	416	240	63	48	112	43	56	69	66	92	70
205412_at	3008	2761	3881	1752	2775	4550	3613	3119	3990	3955	3425	3278	3929	4539	5456	5045	4759	4812	5282	5675	5989
205413_at	25	7	38	62	15	15	29	41	33	18	18	29	1	2	10	1	1	1	5	14	9
205414_s_at	159	229	251	658	36	48	89	116	269	252	17	70	49	27	46	103	89	110	749	358	361
205415_s_at	230	117	113	140	456	542	199	222	83	179	226	269	344	299	312	249	232	317	319	295	279
205416_s_at	276	170	160	147	301	469	166	219	190	166	233	222	236	212	352	261	177	216	279	160	264
205417_s_at	3994	3586	2603	3904	2807	3665	3568	3843	2729	3873	3216	3513	2414	2037	1070	2020	2286	2528	2045	1888	1878
205418_at	205	231	11	17	19	38	193	107	15	10	7	9	14	23	2	249	273	227	4	7	6
205419_at	52	6	27	38	190	132	37	7	43	10	48	18	61	69	138	29	28	26	27	42	15
205420_at	246	286	267	282	245	280	171	154	325	299	54	117	263	227	191	236	387	300	760	695	682
205421_at	3	1	35	93	22	5	1	3	13	53	3	3	4	4	2	1	1	2	43	82	68
205422_s_at	4	17	3	36	12	5	3	4	3	4	3	4	3	4	2	64	39	35	1	3	13
205423_at	627	399	1006	736	1574	1462	542	486	761	819	695	375	356	302	368	291	267	359	530	266	219
205424_at	112	95	35	58	111	56	45	48	44	31	34	36	40	6	65	51	70	64	12	17	8
205425_at	278	102	251	237	454	325	346	380	352	274	783	579	386	273	254	50	46	44	144	100	93
205426_s_at	241	220	270	231	312	251	294	305	252	235	594	279	51	79	102	61	29	58	100	88	72
205427_at	249	390	111	123	228	657	260	202	66	91	151	189	224	93	132	477	474	525	143	208	292
205428_s_at	511	173	2861	286	742	839	586	265	5449	2401	364	254	129	52	69	219	281	117	1797	895	710
205429_s_at	406	232	293	58	719	609	360	356	363	299	804	579	786	717	778	380	302	346	517	342	533
205430_at	3	9	7	4	59	63	5	7	16	8	3	10	7	3	3	22	11	6	27	30	37
205431_s_at	135	116	96	102	212	38	213	176	84	201	111	141	37	57	45	53	25	46	50	87	83
205432_at	209	248	233	504	38	126	351	293	276	282	161	62	56	48	15	86	143	101	157	270	234
205433_at	20	34	66	54	122	65	54	67	40	50	7	44	1	11	23	12	11	3	13	18	2
205434_s_at	101	282	465	522	316	513	360	381	367	314	490	304	325	240	172	119	124	92	198	311	108
205435_s_at	108	79	155	347	47	26	95	115	197	141	128	194	57	62	67	63	36	59	81	23	20
205436_s_at	6489	3724	2769	1116	7638	1254	5399	5569	2863	3620	7184	9325	7172	5814	7943	4921	3755	4628	4532	1648	1158
205437_at	130	200	136	339	312	274	276	218	33	176	262	325	252	252	383	185	201	187	130	166	167

205438_at	20	36	39	16	144	87	23	44	25	11	28	18	7	16	9	6	32	4	8	5	3
205439_at	376	453	18	10	25	66	378	449	19	25	114	60	23	11	27	152	158	137	5	10	7
205440_s_at	9	4	4	18	14	11	49	15	9	18	6	30	1	1	2	3	22	2	13	1	2
205441_at	466	656	414	732	592	692	345	548	345	503	281	302	125	157	215	604	649	505	402	297	215
205442_at	271	311	81	35	103	17	268	201	7	42	68	77	57	55	49	518	499	429	20	24	24
205443_at	572	331	304	127	421	517	416	414	163	161	350	293	503	483	501	641	493	623	233	201	296
205444_at	85	105	118	242	37	60	164	199	158	155	142	93	4	22	21	39	12	7	11	39	17
205445_at	136	107	164	36	245	151	144	84	135	196	162	131	47	6	26	37	50	47	42	51	16
205446_s_at	189	102	604	561	579	836	322	480	309	539	603	396	212	257	290	232	202	243	222	279	401
205447_s_at	64	371	15	18	69	31	54	50	12	15	30	30	50	34	60	133	140	82	8	84	7
205448_s_at	218	300	224	334	258	271	201	319	189	300	166	369	83	82	144	94	66	69	94	113	97
205449_at	1230	1051	1352	904	484	81	995	1151	1436	1310	1474	816	3197	1271	754	749	1125	697	2842	976	593
205450_at	466	428	703	357	470	442	541	445	609	430	510	456	308	255	599	814	727	566	648	850	1125
205451_at	85	107	42	94	69	87	117	26	19	43	80	97	70	87	68	13	43	9	41	47	25
205452_at	368	574	530	671	305	477	360	396	450	468	279	265	228	202	346	597	602	537	890	747	1030
205453_at	66	34	532	751	1225	765	58	18	688	632	1826	1348	1790	1011	936	107	82	62	231	231	167
205454_at	24	38	24	138	117	309	17	12	15	27	14	15	63	26	34	30	82	89	96	80	75
205455_at	751	601	1150	1111	1112	992	526	635	961	1483	861	939	362	323	419	221	308	268	513	388	378
205456_at	153	249	326	219	73	95	160	232	207	261	216	229	23	29	29	34	24	33	63	61	47
205457_at	308	269	340	400	199	929	370	346	322	425	421	295	147	72	101	7	11	9	44	42	14
205458_at	255	184	254	372	366	572	292	244	262	285	209	137	101	98	101	110	136	103	74	110	80
205459_s_at	212	192	151	48	368	237	184	172	161	131	290	212	90	48	95	37	10	64	57	21	53
205460_at	29	74	19	202	12	23	17	17	16	25	24	13	9	43	53	27	6	23	13	22	51
205461_at	255	203	189	125	34	30	338	238	447	347	443	185	45	36	50	49	17	28	57	55	39
205462_s_at	1617	1000	1442	768	528	513	1083	856	1370	1571	1657	832	952	804	507	548	541	386	407	325	167
205463_s_at	454	574	187	207	1156	853	403	381	194	204	1126	951	2505	2333	4031	777	658	465	419	809	893
205464_at	90	22	113	34	205	164	27	82	52	98	64	60	10	8	11	22	8	6	7	4	5

205465_x_at	307	293	52	34	22	43	172	139	107	187	7	5	4	3	6	19	96	119	15	9	2
205466_s_at	2889	1251	676	396	162	198	712	720	292	862	34	26	36	45	49	2139	1201	2093	1150	513	541
205467_at	106	173	191	224	408	336	164	143	246	73	77	41	34	51	31	46	88	19	96	91	60
205468_s_at	115	85	26	26	67	156	110	103	24	29	17	89	14	4	11	5	3	9	6	22	4
205469_s_at	560	511	513	488	1031	1367	476	487	324	421	460	495	171	167	242	167	242	141	161	112	165
205470_s_at	140	16	844	1469	25	34	134	42	1137	1528	75	13	25	60	57	3	13	11	925	349	286
205471_s_at	58	5	80	54	15	7	38	41	20	28	28	8	40	55	46	2	3	7	32	14	30
205472_s_at	12	4	5	6	25	50	13	11	8	13	7	9	11	44	12	1	2	5	3	1	13
205473_at	41	32	75	2323	267	512	20	108	154	66	27	87	35	56	7	8	9	40	97	130	214
205474_at	1928	1719	1365	678	1097	1087	1829	1551	1260	1481	1077	955	1251	1531	2418	4883	4340	4411	2379	1957	2397
205475_at	148	170	187	203	359	223	216	176	131	221	141	280	39	44	52	41	33	28	32	41	23
205476_at	7	6	23	14	36	32	12	15	15	24	388	610	12	5	3	2	2	9	150	317	292
205477_s_at	9	9	15	36	19	43	11	9	17	14	10	6	2	3	4	3	3	3	6	5	15
205478_at	15	43	16	6	19	12	25	26	12	6	7	8	7	22	6	61	62	78	1	2	2
205479_s_at	1914	3849	1092	287	318	887	750	678	367	177	1046	654	204	339	135	1671	2349	2238	136	229	245
205480_s_at	1945	2080	3946	4252	5007	5832	2405	2220	3353	3100	4876	3288	6768	7581	8968	4113	3969	3808	6864	7447	9865
205481_at	267	178	163	109	290	378	268	245	197	171	210	99	75	41	65	64	44	32	46	17	23
205482_x_at	583	387	591	895	898	1066	522	511	615	1026	850	901	448	553	594	389	348	338	588	581	529
205483_s_at	4102	9091	2241	3751	2508	3759	3241	2568	2329	1887	2220	2824	2440	1957	1739	1884	3994	13318	1583	7251	3907
205484_at	66	17	24	199	121	370	60	25	63	116	54	49	11	3	15	8	3	9	11	77	12
205485_at	81	86	5	72	58	110	118	78	33	4	188	52	51	24	30	5	42	17	6	9	5
205486_at	265	271	418	694	454	641	322	428	356	566	324	544	67	98	124	127	207	199	331	332	418
205487_s_at	29	17	23	660	76	65	20	21	86	25	16	21	2	3	3	3	5	13	53	5	29
205488_at	20	58	90	56	296	220	83	107	88	183	98	111	4	47	42	27	3	2	40	24	24
205489_at	430	378	317	1037	342	386	360	368	436	485	151	300	29	124	25	197	313	283	229	239	255
205490_x_at	1050	644	1937	883	452	829	708	738	1152	1360	1401	731	784	991	1021	769	952	536	662	581	317
205491_s_at	149	54	81	147	194	118	133	123	106	136	119	142	10	4	6	24	2	4	30	5	23

205492_s_at	139	82	110	97	100	122	103	133	149	39	134	183	37	40	11	33	80	7	50	70	23
205493_s_at	90	44	18	10	102	55	118	73	43	17	17	54	19	40	22	130	126	131	9	6	3
205494_at	120	127	53	186	56	56	147	71	158	194	151	211	15	17	8	20	24	34	43	2	64
205495_s_at	81	79	31	61	67	77	32	61	106	78	77	15	2	7	5	5	8	8	20	14	5
205496_at	7	15	12	6	5	13	7	5	3	63	6	14	1	2	3	5	1	2	4	2	1
205497_at	148	189	161	136	153	247	204	252	132	236	254	273	123	121	93	145	150	197	122	126	132
205498_at	65	4	39	44	122	65	52	73	9	70	51	60	4	14	3	2	3	2	46	29	37
205499_at	134	106	137	166	150	15	105	102	32	99	14	50	34	5	40	59	31	71	46	56	55
205500_at	120	51	76	62	216	258	91	106	87	119	94	229	64	77	132	17	17	12	56	158	209
205501_at	46	29	469	238	56	12	30	32	280	461	40	46	16	20	31	12	6	3	580	538	891
205502_at	144	114	189	120	62	48	201	244	190	215	213	278	14	6	60	22	24	18	51	29	3
205503_at	184	173	246	266	258	235	143	160	203	165	375	280	59	103	46	64	94	83	57	59	84
205504_at	26	27	53	76	40	31	86	104	35	41	16	25	5	9	6	12	33	18	6	16	5
205505_at	198	147	167	305	129	558	205	280	159	190	185	227	46	59	93	133	117	98	90	104	98
205506_at	9	62	779	1042	774	896	24	33	866	1191	948	315	218	161	748	23	17	36	1055	562	942
205507_at	229	193	145	241	254	212	192	239	244	256	152	153	26	32	52	30	37	54	48	47	61
205508_at	271	184	191	210	309	318	246	338	304	212	216	222	69	67	108	57	83	87	20	52	21
205509_at	208	123	193	268	423	347	143	170	183	165	109	128	22	28	62	13	24	25	39	32	5
205510_s_at	78	90	159	107	249	192	72	87	146	607	107	118	166	206	86	142	183	123	139	63	89
205511_at	33	34	30	61	37	8	34	38	12	56	31	38	55	39	26	16	29	13	18	17	15
205512_s_at	953	783	1546	1430	1871	843	1232	1413	1647	1347	2179	1307	1916	1572	1928	1138	1004	872	1390	1657	1871
205513_at	274	191	289	217	190	231	205	271	229	177	175	229	7	4	66	220	291	230	63	74	64
205514_at	83	40	19	41	113	73	8	59	29	7	51	34	4	4	19	29	1	3	10	14	1
205515_at	238	141	80	131	54	112	256	193	87	122	91	50	12	27	3	38	35	47	18	48	39
205516_x_at	1343	2889	2155	1908	783	1090	1479	1104	1474	1384	2216	1218	451	356	863	576	549	294	572	785	482
205517_at	94	22	12	12	51	211	20	63	44	24	13	13	2	2	12	3	4	18	27	5	1
205518_s_at	188	69	168	113	188	134	152	113	112	99	99	79	16	3	11	15	19	23	14	27	26

205519_at	540	94	193	93	557	109	355	223	250	198	175	146	129	107	108	233	214	273	234	122	113
205520_at	202	96	197	154	393	352	216	305	241	238	274	260	74	60	73	44	37	40	23	34	58
205521_at	198	107	60	43	351	297	209	172	173	59	276	255	303	207	320	183	187	202	292	177	185
205522_at	175	185	132	140	221	89	160	224	248	136	105	224	25	39	53	5	23	59	11	16	25
205523_at	4	15	7	23	66	137	5	37	3	6	26	6	2	12	20	4	1	1	5	13	2
205524_s_at	34	17	30	23	45	39	28	34	33	28	23	30	8	6	10	5	37	6	5	6	41
205525_at	83	72	95	110	183	208	44	100	72	105	85	138	23	19	40	19	32	1	3	25	29
205526_s_at	749	510	567	424	1237	706	766	711	689	570	630	687	699	789	841	725	783	952	442	470	507
205527_s_at	941	496	666	257	1047	323	912	372	1026	872	1040	1009	1254	1173	1307	1039	1093	1396	1956	1056	1343
205528_s_at	3	35	4	44	70	83	4	7	28	15	6	18	3	3	2	12	3	5	3	3	21
205529_s_at	97	21	62	34	153	82	160	66	124	99	43	54	16	29	5	31	2	23	33	5	13
205530_at	233	224	160	237	144	207	282	273	206	194	121	192	74	76	45	195	111	132	166	83	120
205531_s_at	29	26	513	167	360	290	15	42	429	467	343	89	391	147	463	4	7	6	403	339	438
205532_s_at	54	30	33	56	27	161	50	77	43	38	53	72	6	2	7	10	4	12	18	17	2
205533_s_at	15	10	4	3	16	4	19	13	5	17	9	21	1	1	14	2	1	2	4	1	21
205534_at	53	148	39	57	22	24	73	104	15	36	16	8	8	2	11	62	40	47	18	28	31
205535_s_at	9	18	3	5	5	13	3	7	13	8	26	10	14	3	2	8	25	15	8	2	2
205536_at	218	204	225	71	25	24	266	330	395	300	298	173	68	107	56	35	52	30	39	51	38
205537_s_at	9	24	8	10	54	26	25	20	37	31	23	8	2	14	11	8	6	44	6	6	6
205538_at	161	258	182	341	399	546	179	194	356	520	158	170	147	144	77	119	109	147	351	210	303
205539_at	165	16	129	193	162	245	144	152	138	133	125	136	56	19	23	35	24	32	20	33	35
205540_s_at	86	91	62	150	16	30	135	124	64	109	70	69	59	92	62	137	153	110	78	96	153
205541_s_at	93	6	79	76	62	77	56	54	98	57	58	28	16	19	2	33	45	8	43	37	2
205542_at	1588	534	3072	1213	933	691	1272	768	1539	530	1175	744	1581	1493	2216	1456	1844	1265	2758	4427	4834
205543_at	586	293	795	261	300	196	753	524	654	625	409	309	270	198	274	272	300	287	1115	411	539
205544_s_at	177	249	9	16	30	9	162	198	9	18	23	23	12	2	19	157	197	155	2	7	3
205545_x_at	1074	950	890	1041	1424	1066	1937	1537	1013	893	1556	1196	3256	2769	2454	2049	2632	2798	1651	1211	1399

205546_s_at	519	626	517	479	959	710	489	635	546	584	807	422	353	414	263	249	323	215	171	166	173
205547_s_at	108	154	193	156	242	558	103	98	79	94	195	108	51	45	114	63	73	64	95	83	80
205548_s_at	2422	3023	996	341	1687	1959	2484	2326	421	660	2685	1781	2144	2156	1967	2601	2781	2557	648	289	276
205549_at	102	16	45	119	154	974	49	112	72	50	82	97	74	22	53	58	14	27	4	17	3
205550_s_at	1203	820	826	1386	1072	1433	855	829	653	843	789	852	749	1061	968	955	996	928	695	708	640
205551_at	11	18	14	14	33	151	30	28	20	20	10	30	23	8	5	5	4	23	4	5	5
205552_s_at	66	74	236	713	394	340	117	77	253	300	104	85	53	76	121	5	57	398	239	734	955
205553_s_at	8	2	8	6	11	12	8	5	5	10	4	8	3	2	2	2	2	2	1	2	1
205554_s_at	116	74	176	57	305	246	121	107	165	59	75	70	32	40	33	23	8	23	38	59	47
205555_s_at	245	153	132	74	25	227	254	326	98	94	207	240	165	182	119	93	102	65	60	51	72
205556_at	16	33	20	18	47	87	29	42	45	35	33	23	12	24	13	21	28	22	7	15	3
205557_at	32	52	31	35	37	39	29	41	21	27	23	24	22	7	14	3	2	21	7	9	7
205558_at	253	361	252	204	463	570	303	306	190	408	576	564	283	415	343	312	260	302	311	280	286
205559_s_at	126	89	170	169	228	1091	115	135	132	260	357	248	968	1530	1249	317	267	238	530	249	331
205560_at	103	139	194	222	118	251	133	127	139	198	270	205	423	579	354	94	156	123	176	69	143
205561_at	136	86	14	27	597	464	110	38	17	32	45	107	141	100	42	79	21	8	15	42	61
205562_at	988	978	1031	612	533	184	959	798	803	876	634	563	499	418	728	1492	1318	1307	1045	937	784
205563_at	25	16	12	38	43	32	23	21	11	14	23	20	60	58	27	6	16	17	24	12	6
205564_at	78	74	79	132	318	383	11	65	7	80	48	53	34	35	33	29	44	16	39	20	50
205565_s_at	630	696	566	290	691	443	576	486	530	562	532	547	865	556	477	577	517	741	723	357	241
205566_at	347	307	2204	2352	517	443	590	615	2067	2908	1381	739	232	285	342	90	99	92	367	270	460
205567_at	196	151	183	180	210	404	257	115	179	165	264	224	71	81	129	92	33	10	62	54	64
205568_at	12	10	16	23	18	23	17	16	15	14	13	15	7	35	5	3	11	6	2	3	9
205569_at	156	479	73	45	415	8179	240	227	52	29	422	907	436	445	506	493	723	1232	16	1109	1846
205570_at	311	206	182	173	88	226	326	282	250	190	175	220	70	55	107	109	96	101	76	78	64
205571_at	467	421	231	392	275	399	370	313	143	326	308	381	353	525	775	586	659	517	474	511	511
205572_at	42	11	8	16	30	66	8	20	16	13	6	9	1	17	2	16	2	28	3	2	2

205573_s_at	1540	2314	2227	2493	1073	1467	1599	1631	1857	2335	1080	1342	1111	1488	2082	2761	2921	2878	5254	3822	4873
205574_x_at	419	682	243	266	121	290	595	581	104	229	325	194	21	83	85	195	200	224	100	57	84
205575_at	298	189	248	241	400	368	276	396	229	274	398	362	66	59	44	18	60	45	60	47	43
205576_at	29	4	35	12	27	27	17	9	16	21	7	6	2	2	11	3	26	32	4	4	5
205577_at	134	24	30	220	33	31	30	162	120	184	74	122	50	30	29	4	28	5	13	34	3
205578_at	160	118	9	16	148	34	220	161	23	21	173	165	87	99	72	144	144	93	5	8	4
205579_at	267	124	222	101	59	54	186	239	258	226	252	224	75	50	88	180	151	160	174	132	110
205580_s_at	122	64	161	83	85	60	203	247	41	259	129	168	10	61	5	64	68	40	20	22	7
205581_s_at	296	303	266	109	59	70	424	24	273	267	36	29	20	4	49	114	181	104	186	81	94
205582_s_at	20	24	15	18	34	20	19	20	12	41	33	18	3	3	9	8	10	6	3	3	3
205583_s_at	295	491	266	565	290	543	186	400	376	416	149	258	256	201	209	188	189	185	584	668	1056
205584_at	114	173	118	172	221	453	129	148	111	124	91	128	147	97	111	93	123	79	209	280	421
205585_at	116	148	164	164	22	82	97	148	111	187	171	131	45	28	40	74	39	61	60	74	47
205586_x_at	519	308	369	216	1100	1141	383	405	138	271	306	275	210	189	264	200	153	221	107	129	82
205587_at	40	15	41	41	52	48	30	41	21	15	17	19	2	3	10	15	10	9	4	2	7
205588_s_at	684	566	583	330	1170	879	804	694	609	443	712	549	593	548	622	561	601	724	699	383	415
205589_at	216	163	121	111	70	59	41	46	47	70	98	63	8	60	17	18	10	7	19	72	4
205590_at	49	100	31	49	81	106	90	59	32	53	44	54	78	117	88	57	63	31	46	26	25
205591_at	12	6	18	6	73	42	13	8	4	13	431	514	306	333	149	30	5	6	4	7	3
205592_at	139	29	75	101	220	133	69	36	58	68	50	36	11	15	11	15	10	8	50	47	46
205593_s_at	492	546	302	93	60	15	445	524	357	310	212	67	114	182	222	411	316	251	168	184	147
205594_at	394	198	351	529	599	703	246	424	355	421	591	792	207	257	107	112	113	49	383	412	373
205595_at	116	46	163	87	40	103	130	173	107	115	237	33	20	50	55	7	28	16	6	8	4
205596_s_at	4331	2578	1457	437	3185	1477	2259	2315	720	746	3326	3515	3638	3491	5598	9437	10738	9504	1738	1612	2009
205597_at	40	84	570	1886	352	155	121	111	401	643	107	34	8	5	3	24	2	17	273	353	525
205598_at	202	113	301	31	161	39	239	157	250	231	320	366	213	173	168	107	74	94	178	50	124
205599_at	124	94	145	81	206	81	36	124	119	99	263	160	31	45	23	2	16	64	27	27	18

205600_x_at	521	609	723	1121	1017	1153	794	730	1246	1315	1036	1081	836	760	333	144	138	154	292	195	300
205601_s_at	529	730	749	1414	1362	1316	486	623	878	1838	1512	990	926	890	510	272	232	232	355	194	209
205602_x_at	41	180	175	63	436	997	97	146	32	169	250	109	262	216	294	4	21	9	47	52	4
205603_s_at	172	156	296	369	440	239	160	216	272	408	244	139	217	239	368	170	105	84	386	382	473
205604_at	205	84	45	47	408	140	134	132	124	131	138	229	96	46	49	9	11	34	29	29	28
205605_at	24	49	22	22	198	133	16	11	15	22	13	16	6	8	14	27	18	16	7	10	17
205606_at	73	24	163	190	26	74	140	59	190	193	226	54	61	20	9	73	77	63	20	71	84
205607_s_at	52	90	244	163	276	224	354	358	270	277	212	181	305	186	151	199	109	235	345	276	306
205608_s_at	253	150	320	220	404	165	281	273	322	464	205	221	21	55	43	36	7	38	63	47	61
205609_at	30	43	107	76	10	152	23	62	83	147	50	50	15	24	10	6	19	11	213	114	174
205610_at	12	12	87	23	65	32	76	49	84	81	63	59	5	3	18	22	3	3	6	2	9
205611_at	46	120	9	14	48	58	32	24	12	10	16	19	13	7	11	10	35	14	6	9	4
205612_at	17	38	20	31	56	19	68	13	1	25	6	6	2	6	2	8	2	1	23	15	13
205613_at	270	572	37	839	37	27	254	247	25	28	33	38	20	65	7	349	461	254	43	225	210
205614_x_at	234	510	186	188	95	94	170	78	136	99	131	64	68	90	59	100	132	151	48	14	50
205615_at	15	4	11	36	58	50	13	16	16	8	21	13	5	4	5	3	6	3	4	7	5
205616_at	179	142	129	188	155	419	97	37	100	136	34	78	21	25	7	37	51	8	66	10	5
205617_at	171	331	214	813	208	332	282	218	257	275	186	138	86	116	188	113	114	118	149	140	128
205618_at	343	476	364	485	371	1039	425	508	395	576	389	416	338	211	383	708	778	615	711	575	921
205619_s_at	61	55	49	26	51	97	76	82	17	59	47	53	16	17	14	3	43	4	6	4	3
205620_at	34	64	35	105	205	399	25	79	44	80	141	239	39	11	31	29	31	27	20	21	7
205621_at	898	803	663	649	904	769	1314	1079	696	692	1103	1121	583	615	1070	1074	920	983	595	417	695
205622_at	312	322	241	182	441	234	297	162	385	519	198	205	208	156	153	112	122	143	214	220	89
205623_at	83	28	825	7585	385	551	21	40	1464	1430	327	65	214	174	97	47	35	3	371	191	119
205624_at	127	28	96	110	213	77	25	162	100	123	91	127	16	7	38	7	28	22	33	14	6
205625_s_at	56	58	47	96	147	145	114	78	167	41	125	89	42	15	45	15	2	26	6	47	48
205626_s_at	75	57	61	79	197	237	20	38	107	60	70	67	15	31	44	20	15	17	14	24	15

205627_at	210	496	49	491	47	1446	334	282	11	109	574
	397	254	257	916	468	539	392	65	360	124	
205628_at	337	133	183	138	386	226	252	391	165	183	324
	368	302	334	273	368	340	315	418	194	268	
205629_s_at	16	6	19	16	49	38	13	22	9	6	10
	8	15	28	9	7	47	6	5	17	22	
205630_at	106	10	47	44	81	98	148	144	91	102	97
	99	4	4	9	19	42	28	53	11	13	
205631_at	370	281	435	365	433	371	664	674	400	512	424
	418	141	180	127	146	153	221	213	148	128	
205632_s_at	70	120	408	563	672	410	114	98	334	444	387
	300	563	468	452	17	10	37	289	902	937	
205633_s_at	1689	1025	1469	1467	1314	1594	1526	1132	1127	1201	1376
	1392	1618	1662	1197	1374	1262	1472	1269	1293	1529	
205634_x_at	515	556	598	670	707	534	775	693	602	766	1053
	865	673	447	315	116	145	171	226	229	170	
205635_at	25	77	53	57	117	110	24	45	74	13	61
	78	41	36	28	36	28	17	11	16	24	
205636_at	325	145	144	154	392	396	193	168	206	349	112
	129	25	36	72	42	31	49	39	82	20	
205637_s_at	45	116	42	91	80	69	322	197	288	57	43
	119	24	8	10	131	105	64	63	61	45	
205638_at	12	40	20	58	15	81	53	46	71	59	81
	60	3	1	13	1	2	6	6	8	13	
205639_at	19	16	31	25	197	51	15	18	19	18	11
	13	38	66	48	60	65	73	58	42	23	
205640_at	242	847	410	1448	100	476	220	165	325	403	325
	199	95	98	108	133	188	130	231	308	235	
205641_s_at	566	876	572	1238	614	879	333	507	273	570	576
	659	289	411	461	335	589	476	782	420	249	
205642_at	151	103	213	159	227	155	212	253	282	419	226
	175	87	107	90	85	62	104	179	91	108	
205643_s_at	298	122	228	181	243	243	228	280	205	287	222
	82	17	13	55	25	35	29	47	48	21	
205644_s_at	5509	4402	6799	3810	7935	4401	5737	5557	6731	5098	6971
	8660	16243	17353	15143	9542	10731	12471	10448	7736	6308	
205645_at	37	99	260	528	231	278	20	169	507	443	202
	332	114	180	176	152	154	84	439	851	866	
205646_s_at	194	168	37	40	1343	515	142	157	24	20	924
	1015	1011	1261	1301	102	158	64	10	41	17	
205647_at	200	335	16	150	82	328	125	210	9	18	91
	119	70	60	120	134	66	86	130	131	222	
205648_at	101	29	128	96	110	116	62	133	47	39	176
	116	28	61	24	9	25	22	8	34	7	
205649_s_at	1	4	11	57	7	13	8	7	3	8	6
	11	2	3	20	1	2	5	4	14	2	
205650_s_at	229	151	312	253	52	85	239	228	221	203	48
	151	14	7	7	25	25	2	22	6	35	
205651_x_at	40	19	9	14	70	108	1	37	4	3	43
	46	31	57	88	1	7	9	15	59	66	
205652_s_at	172	189	141	275	302	355	187	276	32	151	121
	254	74	82	38	90	99	68	50	89	64	
205653_at	134	106	65	50	63	173	48	129	44	34	61
	30	44	14	44	57	38	37	45	22	77	

205654_at	201	156	221	237	92	243	246	271	284	204	136
	157	23	48	17	10	89	69	29	44	63	
205655_at	164	50	68	96	27	79	154	149	118	108	85
	55	28	23	5	54	56	50	47	40	60	
205656_at	206	162	222	319	293	223	298	313	239	323	219
	227	39	12	52	29	45	4	47	42	53	
205657_at	46	27	156	125	60	39	30	154	155	151	97
	44	54	30	21	88	38	93	53	42	62	
205658_s_at	566	709	426	314	447	332	542	501	482	391	478
	352	272	294	286	203	243	205	216	195	153	
205659_at	89	147	160	79	143	42	57	88	86	18	92
	36	34	15	7	111	76	157	123	126	174	
205660_at	263	331	627	817	320	556	480	360	1180	846	314
	328	51	66	80	158	341	2450	924	1297	1253	
205661_s_at	1093	427	1004	680	796	687	868	715	1030	695	520
	351	447	331	299	486	426	488	477	299	250	
205662_at	336	196	338	202	749	1309	244	211	154	157	234
	427	327	188	301	86	190	170	189	186	158	
205663_at	36	64	37	53	382	308	34	127	47	21	115
	39	3	70	36	73	33	79	22	44	11	
205664_at	349	248	361	485	396	622	382	339	328	344	419
	304	909	745	775	1133	829	1034	1153	877	1243	
205665_at	86	108	27	59	70	73	12	102	20	46	175
	182	121	98	65	64	34	13	35	9	69	
205666_at	30	23	100	30	36	98	29	29	60	46	23
	18	19	6	14	3	4	7	14	6	40	
205667_at	348	345	321	263	579	478	505	433	233	472	249
	417	218	159	313	486	464	332	340	344	363	
205668_at	22	28	209	596	81	32	17	30	130	467	6
	33	2	21	5	5	1	2	544	894	1211	
205669_at	111	75	84	31	60	97	54	48	70	96	71
	64	14	20	26	4	22	32	17	1	19	
205670_at	12	6	8	85	44	39	7	8	20	35	10
	9	4	36	5	3	5	9	62	103	56	
205671_s_at	110	22	23	59	111	28	60	50	47	55	38
	26	20	11	15	33	55	40	25	35	52	
205672_at	475	769	340	520	926	1603	490	439	405	499	1121
	1240	856	1200	1032	773	887	856	895	1011	870	
205673_s_at	134	167	183	251	458	273	107	132	253	138	289
	431	283	403	324	159	216	201	598	313	354	
205674_x_at	134	99	15	107	279	363	105	111	35	45	65
	35	37	57	13	65	53	80	36	52	17	
205675_at	67	41	64	16	8	34	13	50	54	59	10
	9	1	4	7	19	4	3	3	18	3	
205676_at	507	394	407	318	422	318	437	251	433	335	243
	323	116	109	152	317	334	280	390	432	455	
205677_s_at	826	663	1162	964	958	849	971	1003	1196	1231	1406
	915	1866	1700	2519	1689	1508	1405	1868	2250	1671	
205678_at	86	52	119	85	353	325	103	121	62	61	148
	147	85	45	56	4	9	36	6	3	5	
205679_x_at	389	238	334	464	177	503	443	622	484	442	314
	260	60	44	65	64	30	49	80	62	57	
205680_at	33	46	38	65	107	113	68	66	31	85	55
	63	4	7	22	12	23	25	3	34	28	

205681_at	16	15	14	36	135	180	7	32	52	32	58	31	14	26	22	30	25	25	18	4	27
205682_x_at	254	225	419	446	683	677	196	339	383	415	307	413	174	194	181	154	139	88	206	182	151
205683_x_at	30	68	38	180	45	32	164	125	104	133	98	166	7	51	64	3	3	4	8	28	11
205684_s_at	439	299	635	1671	477	546	272	512	599	594	352	411	280	190	244	294	276	239	594	597	834
205685_at	119	15	31	54	216	239	56	20	29	42	68	68	32	53	49	43	29	37	57	26	37
205686_s_at	155	19	19	21	14	98	17	129	51	141	26	84	23	5	17	2	27	5	8	9	7
205687_at	684	634	597	432	609	425	591	694	697	567	651	483	443	356	468	578	589	421	295	486	310
205688_at	390	247	334	322	370	641	322	472	314	324	243	254	10	32	78	36	65	35	13	38	68
205689_at	284	371	205	197	258	192	127	248	95	212	108	116	53	41	38	87	76	65	88	79	56
205690_s_at	2539	2814	2706	1441	3649	3763	1965	1940	1434	1684	5077	3865	7434	7424	6135	2825	3307	3391	2442	3440	2302
205691_at	356	664	30	9	258	82	256	298	17	22	306	327	135	130	168	363	535	315	45	99	94
205692_s_at	41	68	18	26	73	63	64	75	36	136	36	153	6	7	9	3	17	6	8	8	25
205693_at	151	124	115	167	372	414	147	276	211	176	126	205	85	53	55	97	40	25	43	61	24
205694_at	34	44	62	59	107	114	49	66	88	66	68	55	23	9	20	19	2	18	18	17	18
205695_at	124	66	273	128	503	316	146	152	213	198	192	97	64	38	19	59	79	37	79	50	29
205696_s_at	29	45	54	34	109	83	57	80	78	56	111	77	10	9	25	22	7	19	21	9	9
205697_at	34	46	28	40	58	59	50	36	110	84	108	160	28	25	25	9	12	7	10	8	4
205698_s_at	254	134	262	660	282	253	204	190	365	309	240	196	20	15	26	6	10	11	220	757	638
205699_at	50	24	88	67	180	114	65	48	43	59	50	85	13	24	29	6	8	4	13	22	13
205700_at	77	11	20	23	118	78	32	210	19	28	85	127	65	36	70	160	152	100	6	61	76
205701_at	284	113	246	243	440	472	341	351	442	365	374	255	48	48	74	50	35	51	56	33	82
205702_at	343	190	267	400	443	415	319	257	336	295	516	540	195	364	227	206	151	132	168	189	206
205703_at	17	19	58	18	91	94	15	18	17	20	21	18	5	4	60	19	8	2	14	16	5
205704_s_at	362	232	704	242	467	513	266	255	585	514	500	432	284	247	333	226	260	335	520	481	743
205705_at	230	71	244	178	118	148	58	144	230	273	210	133	108	75	125	54	52	53	147	148	176
205706_s_at	15	64	136	43	258	200	68	91	48	137	135	69	113	145	270	117	102	114	212	264	409
205707_at	390	437	406	504	371	503	452	478	486	811	567	418	229	174	192	115	143	93	148	142	93

205708_s_at	665	584	305	325	803	805	690	566	253	362	780	260	832	473	336	214	319	223	71	80	67
205709_s_at	400	453	437	277	173	328	227	501	360	467	195	173	225	167	181	300	228	225	666	537	666
205710_at	75	18	84	136	243	63	68	51	165	68	117	67	42	32	50	2	26	6	39	25	14
205711_x_at	7644	6053	7446	8697	5241	5781	9855	6940	8619	7094	6735	4815	6601	8625	12618	11985	11807	12372	10292	10880	9811
205712_at	103	10	176	75	202	12	103	152	82	115	142	79	18	3	27	23	41	28	27	12	9
205713_s_at	20	9	5	17	275	241	5	8	11	7	28	9	18	3	5	8	5	4	11	4	6
205714_s_at	34	103	47	36	260	83	83	45	20	24	24	13	11	12	21	6	16	3	6	6	33
205715_at	13	18	14	21	22	22	15	17	20	18	16	10	2	2	1	37	10	2	1	4	1
205716_at	226	137	258	234	300	500	305	466	308	337	866	520	209	103	308	224	273	371	252	209	175
205717_x_at	489	633	251	375	514	499	629	612	244	348	1086	611	199	180	236	164	193	174	79	82	74
205718_at	45	18	64	41	274	306	30	16	16	17	14	8	5	9	9	8	10	8	61	19	73
205719_s_at	69	29	43	41	40	407	73	135	39	61	70	106	24	15	57	42	51	37	11	16	24
205720_at	17	12	12	12	33	46	27	24	15	27	23	16	5	6	9	20	21	6	6	4	3
205721_at	32	10	22	14	65	125	24	18	39	21	18	21	23	10	48	22	71	9	13	45	31
205722_s_at	36	17	16	40	25	19	21	20	19	29	28	21	2	9	10	26	22	6	6	7	2
205723_at	228	208	294	122	837	697	213	327	195	229	222	206	38	56	81	94	59	84	76	95	72
205724_at	183	118	72	118	44	23	155	128	175	123	108	160	26	48	20	5	8	29	32	27	8
205725_at	49	12	22	119	102	169	135	123	56	73	135	84	4	3	20	20	20	4	24	2	1
205726_at	263	294	685	1399	679	539	469	558	627	1145	622	518	311	295	873	494	447	465	1360	1510	2366
205727_at	11	12	11	13	19	31	7	13	16	36	6	15	19	6	11	13	5	22	3	26	6
205728_at	4	4	7	8	113	59	4	4	13	21	6	5	10	15	8	19	2	2	2	2	3
205729_at	597	247	99	116	142	198	354	371	39	120	71	21	8	11	13	238	208	222	15	29	16
205730_s_at	118	156	956	292	22	97	189	183	507	1023	17	29	19	25	27	75	81	130	189	127	155
205731_s_at	115	7	41	115	195	179	42	146	56	80	72	80	19	30	4	30	21	5	8	7	17
205732_s_at	83	91	47	127	82	85	189	235	56	112	85	82	17	11	9	41	30	22	36	37	37
205733_at	668	376	1043	447	821	220	816	1002	1062	1135	735	897	599	442	598	407	324	403	1349	377	473
205734_s_at	16	4	4	4	60	54	7	17	4	4	4	4	2	6	3	3	3	3	4	3	2

205735_s_at	16	15	18	19	65	122	17	17	16	13	16	18	13	15	9	3	7	12	6	4	1
205736_at	56	6	5	9	65	27	12	8	12	8	10	11	3	4	7	13	25	25	14	2	29
205737_at	261	321	26	22	282	258	229	211	95	148	129	104	25	9	41	133	144	62	29	36	16
205738_s_at	97	47	64	137	217	273	80	46	68	126	132	84	326	270	419	32	10	7	16	15	19
205739_x_at	111	79	151	32	495	231	172	172	139	95	159	145	284	383	215	283	291	438	296	206	333
205740_s_at	1068	772	1636	1402	1662	1265	1134	925	1397	1322	1893	1272	815	719	630	333	370	235	576	358	242
205741_s_at	143	77	98	39	147	70	148	146	143	161	117	122	16	2	28	83	81	76	15	16	1
205742_at	352	410	205	275	356	245	441	453	253	349	472	406	324	168	100	138	199	85	130	100	37
205743_at	332	255	193	348	617	188	262	133	273	376	276	319	31	14	31	56	47	64	46	32	66
205744_at	239	276	121	70	198	71	192	259	250	141	153	153	66	51	44	73	73	55	27	22	5
205745_x_at	499	546	628	532	327	961	505	534	389	397	456	667	459	643	694	871	639	604	507	791	895
205746_s_at	390	251	342	242	169	323	310	322	175	203	144	123	148	211	121	224	260	263	137	182	248
205747_at	21	47	91	96	143	38	90	58	88	96	129	78	20	62	10	31	29	6	3	2	13
205748_s_at	867	789	779	382	679	583	1248	955	1098	621	824	249	466	349	200	328	296	395	411	231	157
205749_at	336	1123	75	1359	319	202	374	194	102	119	152	73	63	95	65	126	172	144	18	5	3
205750_at	283	282	176	365	438	270	272	529	136	335	217	366	233	231	184	246	288	329	200	225	287
205751_at	74	57	52	47	7	27	58	59	28	41	54	21	10	28	21	2	16	26	13	21	39
205752_s_at	224	327	349	367	553	433	258	368	324	266	283	305	56	48	111	82	66	58	94	74	60
205753_at	85	22	24	39	65	48	29	94	68	67	36	11	3	4	4	4	2	4	3	4	5
205754_at	67	84	23	32	33	30	121	54	39	45	13	74	40	8	6	16	7	9	6	41	5
205755_at	78	61	71	83	95	215	95	136	143	148	90	147	31	23	19	8	8	15	4	41	15
205756_s_at	405	338	258	648	378	894	394	401	233	372	524	670	348	257	302	248	225	250	263	504	457
205757_at	146	120	186	489	287	296	236	294	284	215	186	194	44	45	62	39	47	30	92	56	94
205758_at	262	43	47	53	364	396	189	65	115	211	205	225	65	7	22	49	43	28	10	7	15
205759_s_at	263	310	684	1169	272	140	329	289	440	330	328	211	84	86	178	126	120	80	334	274	122
205760_s_at	294	332	425	587	318	590	186	272	450	548	279	245	208	150	256	268	202	364	498	431	529
205761_s_at	509	448	558	415	914	1465	453	413	479	298	1043	933	1344	1438	2166	600	553	596	935	1075	1030

205762_s_at	87	263	141	35	407	505	89	88	94	96	303	397	357	154	347	60	128	109	197	160	288
205763_s_at	575	500	1862	816	1344	1086	2182	1545	2792	2587	2285	1819	993	795	873	1274	1014	1371	1276	763	1385
205764_at	54	40	28	43	109	23	48	30	71	11	7	29	13	21	20	9	13	38	13	27	17
205765_at	48	7	529	940	170	145	7	30	322	1104	168	136	74	100	72	1	12	4	682	705	1137
205766_at	200	32	144	43	253	87	98	48	142	59	38	87	9	32	27	24	48	42	54	15	17
205767_at	6869	3455	813	181	82	71	2604	3556	384	81	88	14	162	92	231	6146	5432	9523	606	747	919
205768_s_at	860	2297	644	689	1478	1556	558	647	633	647	559	582	814	728	673	434	454	374	1486	1296	1438
205769_at	765	2463	914	935	1144	766	881	761	705	850	772	705	818	781	904	634	918	743	1194	1035	1303
205770_at	1867	1815	818	554	386	621	3282	2453	1466	2112	985	593	479	479	388	850	761	933	591	412	434
205771_s_at	144	235	205	591	169	194	196	182	459	453	159	205	132	183	142	280	163	297	1236	752	1327
205772_s_at	73	30	98	79	118	112	50	95	120	119	65	53	8	25	9	30	10	37	22	16	12
205773_at	30	52	5	65	49	59	41	42	37	31	10	5	20	45	58	88	100	152	90	99	229
205774_at	1270	718	775	623	1681	501	1246	727	1152	925	1587	959	2019	918	1515	488	451	774	1145	1040	573
205775_at	173	218	136	136	348	189	170	161	157	187	325	287	245	238	565	70	74	17	42	54	15
205776_at	44	64	76	813	170	163	69	117	78	154	117	30	35	48	55	39	52	47	143	174	249
205777_at	258	274	136	138	271	202	367	284	110	175	145	139	69	33	63	144	140	146	81	8	21
205778_at	1521	597	126	292	125	439	1558	1595	178	116	487	294	309	104	37	557	686	457	5	37	54
205779_at	26	22	22	19	65	67	19	22	24	32	16	53	5	8	5	5	10	2	30	5	3
205780_at	1606	3692	60	335	1590	1021	1121	813	62	20	1889	1597	2202	2589	2159	1349	1736	1695	442	1340	660
205781_at	189	196	176	282	542	419	178	206	136	140	129	133	58	90	42	83	57	89	64	96	74
205782_at	103	5	83	39	65	17	49	90	99	92	77	35	26	20	16	11	11	22	13	17	9
205783_at	206	200	189	255	186	202	192	325	258	239	199	200	38	25	40	40	48	32	46	39	25
205784_x_at	110	465	81	396	81	54	95	46	47	64	26	83	10	12	23	10	26	9	57	52	45
205785_at	290	165	246	259	506	593	239	239	300	240	215	260	37	53	18	86	41	52	68	86	71
205786_s_at	148	153	92	92	133	146	172	111	131	137	109	162	19	35	17	23	16	30	100	29	39
205787_x_at	812	204	648	947	368	457	827	1082	958	1099	878	738	60	40	68	69	49	4	67	61	68
205788_s_at	2994	2643	3219	4113	3343	2952	3026	3418	3674	4193	2408	2349	2750	2140	2185	3692	3855	4494	4292	4418	5513

205789_at	33	2	20	58	32	16	40	9	7	42	40	44	12	3	6	1	1	1	1	6	2
205790_at	442	558	981	676	823	949	482	369	640	801	924	1125	600	784	571	136	156	96	149	216	181
205791_x_at	82	32	145	178	131	227	99	108	68	60	57	160	101	104	110	117	97	113	99	126	126
205792_at	22	38	26	31	258	370	32	33	25	39	26	36	4	4	5	4	4	3	4	11	3
205793_x_at	400	353	376	383	125	289	396	391	281	71	321	187	113	122	108	122	88	66	30	117	50
205794_s_at	132	38	260	160	293	222	101	103	106	249	153	101	16	30	23	51	37	6	59	106	157
205795_at	8	5	8	12	25	11	8	11	12	14	10	8	5	4	17	4	5	3	1	2	20
205796_at	97	119	72	31	25	113	163	133	94	151	202	244	166	153	189	149	146	160	119	122	152
205797_s_at	220	279	75	62	32	50	183	263	177	228	240	339	126	144	156	83	109	73	21	39	63
205798_at	85	21	39	116	129	38	81	96	107	70	149	94	35	13	25	2	2	4	11	14	2
205799_s_at	78	79	130	758	142	122	136	156	197	204	95	94	28	40	37	35	38	35	425	1149	1703
205800_at	11	2	7	14	32	34	11	13	5	6	6	6	2	2	1	6	3	3	2	1	1
205801_s_at	7	15	8	8	47	36	12	7	11	10	115	34	144	170	104	7	30	3	2	29	37
205802_at	91	134	8	31	441	445	34	236	8	41	173	372	145	153	111	47	103	101	16	18	36
205803_s_at	78	102	69	54	445	587	94	162	12	73	412	495	193	135	65	57	52	87	27	32	53
205804_s_at	19	7	15	12	29	27	19	18	11	18	10	11	7	1	8	2	2	6	4	4	7
205805_s_at	152	50	724	957	326	482	164	185	1357	1544	242	178	70	62	103	73	114	74	486	473	608
205806_at	81	54	56	141	140	74	48	129	123	88	126	68	62	104	52	64	65	105	100	61	26
205807_s_at	525	943	1248	1679	804	1294	584	879	1088	1832	749	1474	630	662	652	582	655	700	2072	1377	1811
205808_at	443	173	358	70	52	63	323	304	313	352	253	222	38	11	5	66	26	16	61	14	80
205809_s_at	128	152	206	185	107	238	127	140	147	309	198	155	699	390	56	110	85	36	134	154	70
205810_s_at	25	39	171	166	89	26	56	95	210	387	280	83	11	25	7	5	8	8	11	14	8
205811_at	906	468	560	371	581	640	619	726	625	628	441	518	278	168	285	410	505	361	404	400	365
205812_s_at	5130	6009	3940	3826	7295	12658	4428	4398	3848	4858	4497	6137	7131	6916	5744	5582	5940	5079	4530	6423	5033
205813_s_at	34	26	37	26	74	129	27	22	31	34	23	26	29	8	28	5	14	59	18	11	10
205814_at	41	83	72	100	54	75	61	108	103	120	94	137	2	7	3	5	3	6	2	2	5
205815_at	283	144	122	124	220	156	152	157	110	180	148	196	39	23	58	20	38	28	40	27	26

205816_at	73	7	23	54	106	83	61	62	59	77	101	67	19	29	30	48	14	36	26	52	39
205817_at	73	22	8	8	40	16	20	28	16	11	45	48	51	12	12	8	5	9	4	2	15
205818_at	147	122	107	107	195	171	176	113	173	147	145	205	13	3	27	32	25	22	7	18	11
205819_at	25	16	23	16	25	22	21	15	12	24	21	24	7	11	27	6	10	10	5	11	5
205820_s_at	12	13	23	35	47	46	11	11	13	10	14	23	5	7	2	3	2	2	4	6	2
205821_at	373	224	103	164	96	31	143	210	122	88	33	84	32	13	19	119	113	83	15	28	23
205822_s_at	556	187	1321	674	874	449	627	722	821	597	812	325	167	186	496	759	601	251	343	475	527
205823_at	212	150	130	237	465	505	241	292	213	221	179	260	111	123	72	83	76	87	93	67	27
205824_at	11	11	12	14	26	17	11	7	7	8	14	8	18	7	4	4	18	4	7	4	3
205825_at	20	13	5	35	14	20	5	5	8	3	7	33	1	14	3	24	4	1	11	5	2
205826_at	217	136	7	27	43	44	172	145	48	89	34	102	4	8	32	118	178	97	19	7	14
205827_at	29	184	14	35	132	177	121	132	71	39	53	64	4	2	22	76	56	44	6	51	58
205828_at	165	27	123	115	181	78	179	166	122	161	90	89	13	3	48	26	10	4	42	22	43
205829_at	21	15	12	16	40	36	23	24	23	28	77	30	152	79	14	43	47	46	9	102	52
205830_at	108	99	47	98	114	957	111	103	39	56	119	50	141	74	73	181	307	183	26	960	1487
205831_at	79	144	129	133	210	122	109	154	132	119	121	141	40	20	36	47	20	20	38	31	35
205832_at	619	446	308	335	403	341	538	517	286	489	316	146	34	6	55	142	217	241	82	57	54
205833_s_at	146	74	52	91	162	297	62	100	62	71	94	150	51	3	20	33	19	28	16	30	32
205834_s_at	101	29	22	56	214	146	109	203	47	64	80	36	31	36	15	35	46	12	13	9	6
205835_s_at	93	27	152	65	137	173	123	169	130	130	226	113	86	92	67	28	37	6	88	66	36
205836_s_at	233	167	283	204	351	387	355	488	241	397	513	298	197	188	275	170	139	206	274	163	173
205837_s_at	11	1	3	1	3	4	1	1	3	1	0	1	5	2	1	15	30	6	5	21	1
205838_at	21	51	24	17	30	31	25	28	23	24	20	18	4	11	16	15	1	7	13	4	19
205839_s_at	65	9	111	211	148	275	72	120	40	50	77	118	32	22	52	16	7	4	40	45	44
205840_x_at	33	15	23	36	38	17	29	17	27	48	41	41	12	8	48	22	7	4	24	3	4
205841_at	82	6	7	101	10	7	28	12	8	6	6	29	22	33	36	45	19	49	43	17	33
205842_s_at	163	170	104	163	89	102	274	305	153	222	136	239	41	14	23	64	51	50	46	61	62

205843_x_at	65	241	91	629	293	515	213	271	56	408	431
	57	84	15	111	42	14	122	14	54	15	
205844_at	5	41	61	74	22	8	20	3	66	43	1
	14	7	5	2	1	16	12	348	221	282	
205845_at	45	58	28	32	34	17	41	53	16	39	31
	38	3	13	4	9	3	26	6	5	4	
205846_at	38	55	407	336	36	32	21	17	494	316	27
	26	8	15	4	2	16	22	142	82	122	
205847_at	355	404	682	724	771	581	323	333	452	471	426
	324	204	153	123	143	132	171	152	312	209	
205848_at	9	16	38	66	34	55	13	84	7	7	7
	6	9	1	1	17	17	17	33	39	54	
205849_s_at	8090	10304	4223	5772	5458	7495	6006	8325	3781	4017	3670
	4825	7966	7054	4432	9527	7659	8710	4527	4120	3115	
205850_s_at	49	15	56	26	47	28	42	21	59	73	48
	50	3	8	2	3	28	3	1	22	3	
205851_at	411	271	410	497	492	527	331	397	388	405	574
	403	509	446	357	256	181	154	360	210	184	
205852_at	37	6	19	6	195	396	8	61	20	41	61
	44	15	24	41	10	27	8	29	59	24	
205853_at	22	46	37	147	93	87	166	55	52	56	53
	20	13	6	16	6	4	10	11	4	8	
205854_at	466	238	501	665	558	891	553	611	460	489	918
	774	821	670	915	182	183	214	259	470	492	
205855_at	255	193	159	166	851	904	212	206	195	179	198
	185	46	37	56	144	113	170	112	118	129	
205856_at	21	32	34	41	29	55	5	3	32	32	14
	14	10	1	18	1	4	9	148	31	56	
205857_at	36	23	170	202	147	142	24	86	159	231	57
	38	5	9	27	57	41	68	71	57	132	
205858_at	107	28	9	8	1636	2385	42	74	28	14	3098
	1976	1381	558	245	20	34	17	4	18	5	
205859_at	274	195	156	300	312	351	184	284	186	59	175
	191	41	42	32	46	78	86	102	99	80	
205860_x_at	19	13	8	6	14	87	13	15	43	15	6
	11	2	13	4	5	2	11	3	4	12	
205861_at	70	97	149	155	55	69	66	41	68	77	53
	131	7	7	38	37	30	65	23	2	23	
205862_at	64	79	19	0	48	40	19	55	7	0	24
	35	14	1	38	105	81	78	4	34	15	
205863_at	131	75	130	91	30	34	201	136	140	189	104
	78	2	3	4	20	2	3	6	21	12	
205864_at	300	141	354	235	301	366	311	298	317	352	406
	303	65	36	104	31	77	39	21	37	31	
205865_at	105	302	332	181	507	288	183	248	272	309	293
	139	207	161	145	122	107	107	158	162	134	
205866_at	17	22	28	43	194	125	37	83	17	75	30
	14	6	43	54	44	31	45	6	35	19	
205867_at	142	54	141	110	129	91	262	212	142	271	195
	72	11	3	17	4	19	21	15	7	14	
205868_s_at	294	153	199	145	62	159	596	637	579	600	419
	268	29	40	30	26	9	42	6	7	18	
205869_at	30	26	648	870	44	54	102	66	142	129	58
	15	12	2	3	27	30	3	75	2931	1251	

205870_at	61	82	62	23	136	300	49	125	83	53	37	15	3	10	6	5	10	11	8	36	28
205871_at	52	22	37	22	203	192	42	29	13	29	105	145	35	19	63	33	17	29	20	44	29
205872_x_at	22	7	27	38	37	59	19	21	12	17	17	14	26	12	27	28	6	15	8	5	6
205873_at	422	196	297	305	554	547	489	471	615	570	621	313	192	263	215	207	241	278	304	157	165
205874_at	24	124	148	115	285	285	207	111	33	87	611	465	294	423	148	6	10	9	6	44	7
205875_s_at	22	99	122	343	363	305	19	28	310	84	291	132	311	398	195	99	94	177	205	147	66
205876_at	38	12	5	44	56	9	69	73	33	38	10	5	2	14	1	16	35	24	1	1	3
205877_s_at	405	364	87	217	502	462	188	293	199	173	291	288	363	177	85	119	141	113	83	66	74
205878_at	13	129	130	47	197	62	12	103	13	59	114	49	10	48	53	33	19	15	36	62	62
205879_x_at	471	245	418	374	542	443	527	616	479	665	523	172	74	68	49	72	51	61	107	47	66
205880_at	89	18	61	63	103	98	95	55	135	101	99	88	5	14	27	9	29	1	34	25	15
205881_at	249	259	301	402	143	304	256	248	340	254	209	173	69	51	96	154	105	126	157	192	200
205882_x_at	525	491	2185	4638	1649	898	1085	1580	1952	3460	1818	2092	1373	1271	1326	336	412	244	1393	1470	2606
205883_at	142	39	151	83	349	429	188	189	170	145	105	206	20	17	36	20	5	12	27	29	13
205884_at	7	2	5	10	10	23	3	9	8	11	9	40	2	1	3	3	16	1	23	15	1
205885_s_at	28	27	26	35	82	7	15	4	19	24	28	5	13	9	1	1	3	5	7	1	5
205886_at	180	54	80	160	249	332	91	161	157	157	142	122	15	14	42	25	27	32	21	39	3
205887_x_at	250	43	324	425	452	261	113	187	312	338	260	180	379	160	139	47	54	117	1028	943	831
205888_s_at	69	107	191	349	74	79	143	86	99	126	169	101	4	32	7	9	6	32	46	66	62
205889_s_at	75	19	73	106	115	173	93	55	79	60	43	39	1	1	25	10	12	1	13	20	22
205890_s_at	11	187	19	138	29	27	32	29	19	21	313	299	6	2	29	14	3	32	5	24	43
205891_at	3282	2877	1422	608	2217	839	2515	2828	1468	1256	2879	2221	1544	1504	1097	4037	3678	4016	1218	867	874
205892_s_at	9	10	9	78	12	9	11	8	15	81	7	11	4	3	2	2	2	4	48	22	3
205893_at	32	10	7	18	48	5	17	5	35	7	7	63	18	1	2	1	19	12	4	1	8
205894_at	261	129	331	490	326	306	208	227	381	309	230	216	10	17	9	22	8	49	203	204	250
205895_s_at	2133	1029	2416	727	1491	906	3409	2585	3508	3072	1747	1175	1099	416	433	858	1111	877	1507	694	1057
205896_at	278	162	246	184	502	599	209	170	186	224	520	566	226	459	381	129	71	121	72	71	63

205897_at	95	32	68	220	34	17	40	24	239	66	243
	67	4	8	58	6	5	13	8	9	5	
205898_at	16	7	4	31	180	110	83	42	68	70	21
	6	13	2	9	14	20	2	27	45	13	
205899_at	115	94	22	138	109	94	81	145	67	186	101
	98	3	15	11	1	3	1	22	27	3	
205900_at	206	173	146	184	208	378	250	123	187	151	108
	232	58	6	46	27	20	12	39	20	32	
205901_at	115	120	61	133	191	22	207	25	144	122	31
	34	47	9	6	6	7	9	38	32	21	
205902_at	13	7	9	12	16	12	9	9	9	10	10
	9	4	5	3	6	4	5	3	3	1	
205903_s_at	12	7	4	6	14	20	13	15	7	10	10
	9	3	4	19	7	3	6	14	21	3	
205904_at	908	434	392	440	1036	523	659	629	343	386	742
	390	755	1011	752	1215	1328	1015	299	458	261	
205905_s_at	695	373	323	309	363	218	647	685	305	355	674
	525	474	529	333	407	276	437	172	209	220	
205906_at	32	10	60	45	121	271	83	22	44	63	36
	29	4	28	50	19	12	7	57	56	40	
205907_s_at	164	46	39	48	15	62	46	80	122	80	51
	106	15	12	11	1	2	1	2	7	14	
205908_s_at	9	7	14	17	213	60	52	11	48	13	67
	11	1	15	1	14	3	3	30	23	17	
205909_at	1058	482	699	347	816	133	1134	1190	1337	764	699
	667	823	838	959	1282	1092	1239	1607	583	679	
205910_s_at	19	40	11	14	22	12	20	15	19	20	13
	8	2	14	7	43	44	55	7	42	4	
205911_at	251	168	269	325	333	515	196	135	138	252	242
	121	53	78	75	102	113	87	69	59	55	
205912_at	34	47	35	154	159	48	53	80	50	49	97
	40	8	7	29	5	2	30	8	4	2	
205913_at	24	94	53	164	73	70	32	59	29	64	24
	20	7	3	9	6	6	30	9	48	13	
205914_s_at	9	9	9	18	7	11	19	7	5	4	6
	8	2	6	4	3	7	4	4	2	2	
205915_x_at	60	208	140	54	359	73	186	183	51	39	26
	40	8	17	9	12	12	14	32	16	39	
205916_at	16	11	16	17	48	149	11	18	20	21	7
	18	2	3	3	2	2	2	3	2	17	
205917_at	364	405	232	486	360	185	486	718	286	384	122
	119	25	28	54	374	330	221	139	164	155	
205918_at	16	12	11	10	26	23	12	15	9	10	13
	13	34	6	14	14	16	44	4	3	2	
205919_at	3137	5456	109	36	183	329	2865	3769	169	197	151
	134	75	75	218	6166	7308	4876	4	19	7	
205920_at	41	24	30	706	48	44	109	129	706	755	985
	46	38	13	7	6	6	7	6	3	7	
205921_s_at	98	85	47	215	30	24	126	144	293	271	496
	235	6	30	5	29	8	41	13	28	17	
205922_at	8	7	18	6	56	56	7	8	7	7	6
	8	2	3	3	12	2	19	3	34	22	
205923_at	118	133	83	106	175	159	129	131	157	88	92
	99	8	36	62	30	7	39	25	7	50	

205924_at	147	136	140	19	74	70	261	351	20	53	254	354	187	62	40	49	20	7	29	6	7
205925_s_at	224	128	104	142	66	93	250	294	132	144	257	273	123	60	26	63	35	24	34	47	14
205926_at	623	446	167	115	449	255	417	432	194	352	370	304	144	194	116	219	173	183	110	92	62
205927_s_at	155	99	2086	782	187	173	204	278	2220	3797	244	206	60	71	53	40	29	31	2564	2873	2866
205928_at	233	249	250	348	322	298	489	416	177	246	435	508	389	314	273	358	590	338	424	286	411
205929_at	156	120	233	54	213	189	191	172	298	226	121	145	29	7	58	9	41	4	214	89	87
205930_at	759	505	852	666	1313	904	1034	740	916	780	1133	1048	821	684	943	722	673	759	1193	774	1074
205931_s_at	37	51	235	81	115	181	56	33	115	133	63	14	4	4	3	31	14	6	40	36	35
205932_s_at	7034	6209	119	69	13553	4957	5304	5092	90	95	9481	6073	12292	13055	12564	9029	7403	7216	123	332	251
205933_at	52	10	87	74	188	246	53	41	27	32	43	53	11	48	29	4	3	2	34	7	4
205934_at	42	45	79	57	102	23	48	48	35	59	34	84	32	10	22	2	6	21	19	22	10
205935_at	161	299	83	66	267	278	212	197	124	99	186	181	83	39	81	141	101	166	29	30	15
205936_s_at	34	17	18	17	34	27	24	22	25	25	23	24	5	4	2	4	4	3	4	6	3
205937_at	58	49	118	71	29	27	16	16	211	191	293	166	227	310	216	16	19	12	224	83	80
205938_at	41	19	24	5	191	191	27	12	90	15	84	31	4	17	21	5	15	23	39	11	17
205939_at	119	152	114	257	131	443	23	84	177	193	242	83	37	87	56	31	7	27	57	31	29
205940_at	4	6	5	10	22	179	37	21	3	7	3	5	2	32	25	23	25	4	12	5	29
205941_s_at	70	60	38	40	165	207	103	34	43	41	136	102	13	6	63	31	20	32	39	4	27
205942_s_at	168	15	321	975	26	15	182	214	720	700	109	77	3	4	6	62	64	34	948	417	777
205943_at	132	62	94	75	78	216	183	170	202	165	91	82	20	2	22	12	17	2	630	168	334
205944_s_at	165	262	100	133	378	366	171	245	163	182	135	160	41	55	100	66	74	91	59	79	50
205945_at	131	113	137	50	69	184	158	146	12	82	240	75	72	153	109	56	102	51	40	147	71
205946_at	22	22	18	27	51	66	23	15	27	18	21	34	7	8	2	3	2	2	6	11	3
205947_s_at	13	9	8	10	21	11	8	16	27	14	41	13	1	5	5	4	3	3	6	3	2
205948_at	132	29	148	45	47	114	203	42	55	94	34	34	11	2	6	2	4	4	9	6	37
205949_at	149	86	130	114	245	188	126	172	126	224	139	178	14	23	33	17	16	28	27	3	33
205950_s_at	295	225	286	72	484	529	197	244	285	233	193	190	53	36	47	40	35	37	55	37	39

205951_at	52	5	7	4	51	43	8	26	31	4	4	35	1	2	19	1	6	6	12	1	1
205952_at	95	94	121	131	249	263	135	117	100	123	94	21	3	2	9	3	24	8	28	19	5
205953_at	386	344	357	312	951	426	489	459	476	453	335	489	93	77	127	122	141	202	146	132	114
205954_at	142	79	69	75	81	27	156	80	67	94	108	150	22	11	42	24	5	4	13	51	11
205955_at	263	207	144	190	197	163	262	239	163	200	229	302	48	38	56	73	56	65	65	49	74
205956_x_at	763	488	829	270	1082	280	1020	755	888	1034	1265	713	609	362	376	275	364	365	384	260	313
205957_at	22	36	172	548	87	89	40	21	47	106	202	39	54	81	104	6	16	7	52	111	100
205958_x_at	12	17	18	16	37	55	17	12	12	14	13	10	4	15	2	2	4	2	6	2	1
205959_at	21	7	12	12	12	20	17	12	12	13	9	14	1	20	7	2	4	4	2	14	26
205960_at	48	13	58	83	331	253	24	34	127	130	38	50	5	7	6	6	43	20	6	3	4
205961_s_at	1240	946	1431	876	1340	1000	1953	2109	1627	2219	1292	1384	1680	1139	1215	2497	1890	2106	2478	1478	2690
205962_at	40	55	15	5	71	28	29	32	25	13	21	58	6	26	7	7	12	28	7	2	23
205963_s_at	2778	1815	2153	1152	2335	1820	2447	1498	2700	1918	1656	1279	947	1097	1469	1659	1682	1506	1835	1919	1526
205964_at	60	10	20	65	11	56	9	37	66	38	71	97	44	45	36	30	17	31	35	29	66
205965_at	57	106	37	50	592	1004	69	57	58	66	411	77	315	346	390	9	13	51	11	95	62
205966_at	36	60	8	35	76	133	86	84	35	17	26	34	46	39	9	46	33	56	48	2	11
205967_at	1177	669	1206	759	639	313	1756	1974	1509	1217	749	1250	16481	9773	1400	2442	1875	1333	3066	1961	721
205968_at	349	354	41	17	203	69	334	350	37	95	303	339	68	62	89	146	157	186	1	1	29
205969_at	33	35	42	384	26	35	93	95	217	99	23	15	2	32	7	86	167	116	327	137	143
205970_at	397	202	265	155	609	605	376	239	153	208	259	475	104	107	148	91	174	18	88	60	9
205971_s_at	12	67	75	91	51	17	94	82	40	14	41	36	8	7	6	8	8	8	13	8	3
205972_at	58	75	113	101	37	58	134	203	158	145	94	156	18	8	12	3	45	9	14	9	27
205973_at	214	120	146	216	363	241	156	106	84	246	168	227	17	46	26	24	35	6	13	6	17
205974_at	57	39	16	76	77	24	13	9	59	13	11	69	3	2	33	5	17	3	24	2	3
205975_s_at	209	136	354	340	415	129	212	339	384	396	297	591	60	38	9	4	18	17	36	37	16
205976_at	134	23	81	107	103	78	89	115	103	116	98	150	50	37	21	36	26	30	58	61	84
205977_s_at	587	641	1272	824	197	58	600	497	1193	1112	460	214	126	50	77	55	87	59	361	181	273

205978_at	46	35	23	5	88	167	25	25	27	55	44	64	38	44	64	11	1	12	1	11	8
205979_at	4	2	5	13	12	12	3	3	3	3	1	3	3	2	5	3	2	2	1	3	1
205980_s_at	1113	1296	700	1194	283	371	1505	1153	752	903	560	279	253	207	300	450	443	398	375	300	356
205981_s_at	381	347	282	301	617	611	227	288	305	233	436	493	565	342	196	220	163	183	296	228	298
205982_x_at	24	11	16	21	25	32	21	17	23	18	14	15	4	6	3	6	11	5	9	6	5
205983_at	143	73	301	278	107	122	179	236	448	320	195	190	23	17	22	22	10	58	476	295	265
205984_at	62	45	43	41	137	35	11	78	20	105	112	99	20	19	32	23	16	21	6	2	7
205985_x_at	32	24	22	36	52	43	28	46	36	25	27	20	2	36	40	22	6	5	7	19	10
205986_at	396	679	403	1040	254	228	509	515	615	433	320	270	96	46	148	196	174	151	226	215	232
205987_at	11	12	11	25	488	603	15	12	12	13	20	21	47	12	11	30	12	4	12	12	12
205988_at	251	165	319	393	357	226	284	347	268	405	318	206	28	28	61	77	38	42	64	39	54
205989_s_at	38	54	26	23	38	117	32	49	20	34	31	45	11	3	3	6	1	3	7	14	1
205990_s_at	70	21	57	16	2668	2422	30	75	55	36	2592	1665	2103	1743	1450	9	20	2	13	12	25
205991_s_at	138	105	81	184	269	258	204	148	82	204	161	118	28	95	53	38	34	44	43	72	67
205992_s_at	183	412	132	208	87	601	82	143	177	117	212	93	87	93	82	125	169	260	331	615	701
205993_s_at	77	15	62	43	66	270	12	21	16	17	117	69	37	21	28	45	33	35	43	14	11
205994_at	77	12	18	50	135	146	11	28	67	74	36	15	43	29	32	45	3	47	56	41	43
205995_x_at	536	575	986	819	1183	1439	835	777	1018	885	787	773	418	407	703	504	431	616	746	1058	1155
205996_s_at	819	378	704	385	562	409	1179	834	1089	799	493	522	224	198	78	216	214	238	388	236	339
205997_at	22	4	3	8	10	12	5	7	5	15	6	5	1	3	4	3	6	1	1	4	1
205998_x_at	413	296	463	559	728	574	374	410	423	594	313	375	69	87	178	88	55	117	13	57	89
205999_x_at	26	78	278	107	291	271	140	133	225	247	162	93	61	65	40	29	5	23	88	107	149
206000_at	22	19	22	19	69	23	13	13	16	24	16	28	11	8	4	4	2	4	17	31	20
206001_at	233	207	161	241	415	195	256	271	269	225	146	139	22	26	20	13	11	29	6	54	3
206002_at	83	16	85	59	522	364	64	112	98	116	439	416	472	403	476	8	17	2	9	17	1
206003_at	419	88	315	92	301	175	172	351	206	338	243	192	229	239	325	278	265	289	424	237	380
206004_at	144	186	111	163	69	77	274	253	165	67	148	204	25	54	29	9	5	25	33	11	37

206005_s_at	26	35	106	173	396	323	57	50	78	105	121	31	92	61	55	35	12	30	130	146	250
206006_s_at	180	68	165	409	238	370	195	305	298	247	190	191	87	73	145	85	73	89	186	164	194
206007_at	8	44	73	92	100	133	45	58	7	45	6	40	1	1	13	2	6	9	2	16	6
206008_at	41	33	18	43	15	83	37	22	91	18	54	20	36	21	7	20	8	16	4	21	21
206009_at	130	69	91	98	263	204	139	135	104	133	47	75	29	45	5	52	21	7	20	16	31
206010_at	247	176	151	273	403	370	176	202	223	243	151	150	50	43	46	62	6	43	57	57	35
206011_at	24	68	8	23	15	24	69	66	161	95	53	8	14	7	10	12	5	5	12	5	26
206012_at	228	146	145	133	177	234	97	166	147	145	139	157	56	20	11	29	73	9	21	31	24
206013_s_at	9	11	8	5	40	19	9	5	12	7	4	10	2	3	2	3	1	2	2	1	5
206014_at	17	11	14	18	25	26	15	9	12	13	9	13	3	4	4	4	4	6	5	4	3
206015_s_at	1340	1307	1565	1614	1434	1473	999	1219	1643	1576	881	1028	1241	897	1516	2366	2391	1958	2437	2383	2195
206016_at	385	373	585	644	554	357	327	274	454	443	715	405	408	301	324	136	106	100	224	335	191
206017_at	87	57	18	17	69	87	122	104	37	52	60	87	4	3	5	3	16	16	8	6	6
206018_at	12	6	11	6	216	75	16	8	44	10	246	158	255	262	298	2	6	2	4	98	93
206019_at	107	156	228	319	40	207	60	131	178	238	107	20	93	75	123	56	24	72	199	147	124
206020_at	892	780	250	188	199	206	486	424	122	217	75	82	111	75	89	1071	785	777	357	435	331
206021_at	77	105	79	36	41	78	33	79	59	50	36	33	14	29	3	19	15	4	31	45	41
206022_at	91	62	49	70	372	270	135	86	112	85	250	131	400	374	250	5	23	1	16	11	16
206023_at	2732	1651	35	8	645	31	3725	4882	165	166	885	1228	1471	1187	1603	4047	4959	4963	210	97	67
206024_at	25	23	16	9	18	28	29	24	17	29	23	19	4	5	6	3	6	18	5	20	12
206025_s_at	119	9	69	110	70	137	125	87	80	66	74	77	14	27	27	19	37	7	16	21	25
206026_s_at	75	13	8	67	162	66	113	110	131	120	88	90	2	3	5	15	25	24	17	21	4
206027_at	157	266	62	124	2062	340	382	348	241	342	2528	2012	936	572	796	172	115	160	81	89	58
206028_s_at	259	298	271	309	242	390	245	241	153	176	161	108	56	71	64	138	153	123	187	215	401
206029_at	5	6	5	43	403	285	50	29	64	6	189	310	1928	2005	1800	42	40	28	13	20	12
206030_at	42	27	18	25	23	202	23	37	37	41	63	18	7	2	29	8	6	25	15	35	5
206031_s_at	599	200	586	573	243	122	751	850	662	559	667	441	106	178	118	76	155	122	57	162	49

206032_at	217	169	52	89	151	168	182	195	83	106	40	60	16	5	28	43	54	72	31	45	21
206033_s_at	253	107	19	132	148	141	183	292	120	91	87	96	3	3	23	41	23	25	11	23	6
206034_at	249	60	320	52	151	56	240	135	215	246	236	181	147	166	153	144	125	126	94	137	138
206035_at	135	71	160	141	179	136	168	124	140	131	117	82	10	25	52	36	43	33	44	28	37
206036_s_at	144	208	220	304	372	409	221	212	250	305	269	211	130	98	124	168	128	136	304	293	355
206037_at	372	387	534	676	76	560	420	432	507	579	495	525	307	298	282	158	115	103	150	129	118
206038_s_at	119	112	140	105	397	292	184	186	119	152	159	123	60	60	57	55	86	92	92	90	70
206039_at	146	46	141	101	214	134	135	120	123	145	136	126	38	15	31	19	5	4	36	50	38
206040_s_at	12	11	7	13	15	22	9	13	4	8	7	13	8	3	2	2	2	3	3	3	2
206042_x_at	7	15	76	89	338	407	73	92	55	133	6	103	5	5	11	2	43	5	16	2	7
206043_s_at	149	247	1670	1183	22	129	273	256	1315	872	108	49	13	54	94	195	243	201	650	1052	935
206044_s_at	295	133	217	175	361	242	313	255	280	287	246	197	64	38	67	69	119	117	121	109	165
206045_s_at	99	6	75	13	19	38	28	32	12	15	50	55	1	2	2	1	24	6	2	1	2
206046_at	169	140	174	255	33	136	201	156	167	177	122	118	8	46	39	38	46	16	60	52	14
206047_at	173	269	265	213	51	89	302	305	235	196	351	222	75	91	77	101	53	72	79	108	82
206048_at	11	2	14	70	151	183	16	11	86	56	57	78	27	29	39	8	14	31	43	61	25
206049_at	357	378	452	325	410	332	376	492	500	503	362	295	54	11	97	76	20	42	116	14	64
206050_s_at	2135	2302	2212	1772	2445	2864	2230	1652	2407	1796	3137	1615	2835	2520	773	624	995	805	849	742	601
206051_at	228	109	28	200	394	323	260	194	246	267	125	132	3	38	11	9	6	10	34	22	2
206052_s_at	3688	3548	1731	1351	3016	1388	3063	3253	2959	2348	2382	2336	5540	5178	4040	6071	5565	5490	5189	3095	3969
206053_at	279	253	323	366	489	372	244	319	313	366	341	304	104	89	76	73	54	89	123	94	88
206054_at	25	43	22	116	66	44	15	18	12	17	16	19	7	20	12	5	18	6	4	4	6
206055_s_at	3608	1740	3159	1640	4298	1692	2792	2231	3845	2387	3351	1806	6706	4186	4534	3562	2643	3106	9471	5412	7019
206056_x_at	77	172	216	52	1434	1481	117	170	51	217	90	314	119	39	21	109	63	78	181	151	194
206057_x_at	83	145	217	100	135	232	82	128	110	176	155	85	13	53	11	23	17	11	13	12	12
206058_at	214	131	218	317	341	215	225	271	427	254	236	180	29	18	44	26	43	25	22	15	35
206059_at	579	438	237	341	586	718	525	641	190	280	661	485	349	430	212	296	334	206	101	182	162

206060_s_at	15	7	16	19	34	20	48	13	7	21	11	50	5	4	25	4	13	21	21	36	62
206061_s_at	194	133	402	419	524	400	197	285	495	328	513	484	116	185	187	60	39	79	202	159	138
206062_at	163	51	132	160	43	97	175	179	155	183	126	189	17	14	43	31	4	3	15	4	9
206063_x_at	441	245	350	420	1071	547	215	435	436	408	381	338	105	103	117	152	88	75	60	87	69
206064_s_at	32	15	221	195	74	19	64	96	205	70	63	24	8	17	13	5	11	11	10	16	3
206065_s_at	79	10	83	94	338	138	48	13	48	101	71	10	14	3	2	12	14	2	30	35	17
206066_s_at	1294	535	539	360	1878	978	1544	1232	682	607	1400	1449	1387	1040	928	850	775	881	522	198	266
206067_s_at	15	6	8	5	275	160	4	4	7	6	374	54	133	103	48	2	3	2	4	4	2
206068_s_at	12	38	11	54	26	87	94	77	78	73	50	80	4	3	26	23	5	15	41	26	20
206069_s_at	13	28	9	12	184	16	17	21	15	29	16	28	14	17	2	6	1	18	4	16	20
206070_s_at	5	4	4	3	12	12	41	20	3	14	21	5	2	8	1	2	2	2	0	2	3
206071_s_at	24	9	9	19	32	24	4	5	8	10	7	13	1	24	3	14	5	15	4	1	4
206072_at	44	129	14	53	38	54	30	108	68	15	48	18	8	33	44	12	33	78	42	47	71
206073_at	40	75	20	62	331	58	23	158	20	71	63	16	17	25	7	45	63	64	38	17	39
206074_s_at	13456	9892	10449	4383	6029	2549	11165	11822	8474	8251	7642	5359	3654	3928	4972	8303	10002	8196	4997	4509	3499
206075_s_at	1935	454	798	586	924	590	2227	1722	1437	1022	1708	1098	349	336	319	493	679	626	334	340	395
206076_at	243	253	18	53	25	185	232	94	24	42	198	459	195	231	109	68	72	87	36	54	38
206077_at	37	66	109	35	37	81	24	38	36	110	105	78	31	14	9	9	14	11	6	9	5
206078_at	250	102	220	173	208	218	258	244	248	312	293	264	41	81	41	32	3	22	97	29	50
206079_at	144	122	332	124	44	179	127	187	503	376	94	157	65	66	69	73	97	91	469	150	247
206080_at	126	27	28	185	407	273	72	40	58	99	77	73	23	67	82	47	86	82	43	34	15
206081_at	197	279	33	156	70	216	118	174	12	17	153	212	132	174	236	330	363	390	203	294	479
206082_at	33	36	56	28	176	306	107	112	25	17	195	191	24	17	51	17	10	147	34	12	38
206083_at	54	41	42	10	518	775	48	95	119	50	47	96	11	61	60	55	116	97	83	56	45
206084_at	107	16	69	16	30	22	38	45	90	64	45	59	2	2	1	29	28	20	43	42	78
206085_s_at	212	62	403	129	309	323	284	244	142	152	192	146	159	147	137	305	283	335	137	365	460
206086_x_at	390	355	339	471	271	232	376	418	316	355	264	236	33	84	109	87	70	54	93	87	64

206087_x_at	811	870	529	1071	341	836	493	560	260	436	377	331	106	123	118	367	291	272	340	483	429
206088_at	165	107	171	190	181	165	131	218	207	289	313	238	21	30	3	2	3	4	26	12	24
206089_at	34	32	28	30	65	77	46	50	51	53	23	127	5	6	5	4	5	3	4	2	3
206090_s_at	167	67	117	71	246	163	87	219	74	112	173	151	48	75	89	31	53	69	24	36	55
206091_at	17	1	22	6	47	23	27	8	13	17	27	11	42	21	25	4	14	1	22	46	80
206092_x_at	341	207	250	190	474	728	237	203	197	190	408	256	161	209	182	130	110	136	132	21	29
206093_x_at	37	118	16	26	104	73	27	36	25	20	26	39	11	35	23	6	15	6	39	7	5
206094_x_at	295	180	4844	11991	2286	1950	360	330	5365	7091	1424	535	1530	854	1078	94	51	52	4638	3042	4372
206095_s_at	2420	1327	1937	1437	1395	826	1565	1735	1709	1895	1468	1178	1878	2008	2517	2609	2133	2393	4028	2812	3701
206096_at	184	165	206	281	459	324	235	278	194	187	310	217	198	332	361	365	362	250	243	198	199
206097_at	344	243	294	1048	151	153	333	313	383	275	209	63	19	24	89	196	214	156	239	177	114
206098_at	91	78	100	100	77	184	154	139	112	101	102	52	103	66	69	94	134	99	109	54	55
206099_at	364	347	460	460	55	67	472	385	471	393	279	133	37	52	44	180	131	96	125	71	103
206100_at	26	6	35	41	16	9	41	36	7	49	48	24	17	33	17	40	24	28	30	6	17
206101_at	111	50	41	114	291	56	81	62	139	78	67	104	4	3	9	27	3	16	21	102	110
206102_at	1380	600	1034	444	1718	263	1668	1449	1829	1572	1970	1837	2796	1757	2311	2166	2221	2528	4104	1830	2294
206103_at	669	764	153	39	33	19	749	603	173	150	173	48	200	129	21	247	276	248	90	87	6
206104_at	225	95	313	422	243	98	156	174	361	331	134	99	223	361	313	228	220	299	763	796	953
206105_at	221	95	243	251	318	164	249	223	340	415	209	225	38	56	82	81	76	52	59	37	57
206106_at	677	974	260	451	451	352	837	761	171	187	389	356	321	249	262	337	361	304	177	161	136
206107_at	24	15	19	32	22	152	20	15	7	29	14	15	8	2	9	2	5	4	4	9	1
206108_s_at	6726	5323	132	222	131	146	7364	7293	887	713	853	867	1438	2072	3013	6710	5916	7095	2740	1314	1244
206109_at	509	364	511	186	572	511	550	437	260	242	598	356	351	224	578	265	333	398	175	307	204
206110_at	15	148	144	301	257	400	70	139	45	145	311	829	206	138	208	89	85	78	64	113	118
206111_at	151	151	152	124	832	324	107	77	157	141	109	162	10	35	27	5	8	12	37	32	3
206112_at	5	27	5	10	67	81	8	32	12	3	4	26	2	2	1	8	2	2	8	2	1
206113_s_at	840	789	798	616	770	1056	735	829	661	798	877	652	419	356	153	213	242	303	243	187	342

206114_at	13	22	35	12	110	20	77	18	16	27	253	490	464	646	349	42	43	15	5	5	6
206115_at	180	54	119	93	345	148	127	145	102	122	304	383	345	326	605	244	132	248	37	112	133
206116_s_at	487	377	1054	819	579	1058	335	274	1006	851	576	426	680	619	459	354	365	307	1088	1248	1326
206117_at	11	10	118	92	213	259	42	12	130	80	107	93	89	73	60	15	14	23	222	106	88
206118_at	221	333	338	376	286	185	382	268	351	271	182	112	14	2	57	54	70	85	86	79	94
206119_at	37	23	54	88	21	59	90	195	95	102	139	74	23	36	27	8	9	5	18	23	7
206120_at	50	26	16	36	19	39	23	53	17	15	13	11	4	4	3	10	7	10	3	4	5
206121_at	130	90	136	96	120	50	115	90	90	141	124	137	17	4	16	22	6	2	14	30	21
206122_at	17	13	16	26	302	48	12	20	24	22	11	13	19	9	7	6	46	32	48	4	5
206123_at	323	453	357	153	305	368	201	298	190	126	95	261	96	63	81	25	66	55	106	69	68
206124_s_at	65	150	100	69	205	77	146	297	59	238	166	18	22	66	38	41	34	79	87	27	33
206125_s_at	1077	914	294	692	320	563	1317	889	266	204	300	234	38	67	74	628	497	429	42	84	50
206126_at	71	17	18	92	41	52	32	107	13	17	37	28	8	43	15	6	4	7	12	4	1
206127_at	302	240	193	127	322	324	284	248	211	112	382	357	25	22	93	68	60	80	28	36	6
206128_at	279	347	66	93	1046	1114	150	232	71	10	499	383	639	397	234	182	184	108	56	67	53
206129_s_at	110	128	98	78	206	145	195	131	144	116	142	155	47	36	76	34	37	34	36	57	47
206130_s_at	34	46	42	59	85	156	29	28	25	27	27	28	6	6	13	19	14	11	57	11	27
206131_at	102	85	87	38	199	108	77	63	248	171	44	116	29	66	46	48	74	40	79	43	49
206132_at	582	263	410	427	561	538	526	521	355	472	480	459	163	123	136	88	89	123	93	61	47
206133_at	11	60	19	103	179	200	9	32	28	61	64	28	37	12	24	28	11	60	25	190	243
206134_at	50	27	68	40	129	52	45	62	98	73	54	78	26	21	19	23	22	39	36	24	28
206135_at	3	6	8	3	40	8	7	11	3	7	6	23	2	3	2	2	3	2	25	7	45
206136_at	16	17	16	19	41	38	19	18	20	11	20	21	7	5	13	9	25	9	107	79	49
206137_at	224	116	195	38	249	120	184	174	248	189	294	264	105	87	95	94	88	120	52	19	56
206138_s_at	1340	1701	1210	1170	1203	1338	1271	1078	1140	922	1185	1170	601	472	489	593	628	552	822	792	666
206139_at	427	388	537	591	456	664	346	331	344	370	431	263	151	201	220	251	202	237	304	235	231
206140_at	335	237	12	8	30	8	223	271	47	22	43	65	77	81	69	954	869	761	11	2	2

206141_at	411	366	349	356	335	347	401	300	332	414	398	270	197	165	141	144	106	109	175	142	137
206142_at	9	12	7	9	8	7	8	4	4	46	9	4	2	3	2	2	4	3	2	2	2
206143_at	34	7	4	18	92	28	3	7	27	7	6	5	16	2	25	23	2	2	2	1	2
206144_at	171	101	170	180	224	173	125	185	227	232	200	185	73	57	74	43	41	47	88	57	58
206145_at	50	36	49	43	81	24	58	36	8	43	7	52	1	4	9	11	3	14	21	5	2
206146_s_at	19	6	28	21	139	55	12	32	12	10	11	15	12	4	9	23	30	25	16	6	5
206147_x_at	434	327	458	397	1387	1270	476	455	626	623	510	602	429	560	354	494	478	275	678	558	771
206148_at	25	30	22	32	65	46	25	24	28	29	36	28	4	4	13	9	5	13	10	5	9
206149_at	42	12	24	43	23	28	16	18	20	22	26	18	7	5	10	5	3	4	8	11	7
206150_at	146	18	161	138	274	384	152	145	181	186	111	98	56	49	58	36	35	69	46	74	35
206151_x_at	16	26	35	34	34	93	20	40	37	34	63	38	8	22	3	3	3	8	27	34	4
206152_at	106	237	134	146	583	728	204	165	183	214	48	93	44	9	18	73	24	39	98	54	47
206153_at	69	151	5	10	8	17	73	125	5	10	7	5	1	2	2	136	120	83	1	2	3
206154_at	160	109	265	233	322	332	309	166	185	285	480	314	370	320	420	12	67	13	36	24	7
206155_at	1025	573	11	12	40	17	1121	1069	33	15	10	21	1	4	6	501	434	575	9	32	42
206156_at	57	40	24	23	22	16	61	48	24	31	44	29	27	11	45	88	68	104	8	4	30
206157_at	34	79	4	39	44	48	19	4	31	36	48	31	4	6	2	20	16	13	6	4	10
206158_s_at	8966	6820	8056	7086	8977	8949	6487	5786	9105	7311	6631	7127	9997	10004	7099	9108	8031	8864	9352	8492	8233
206159_at	36	57	182	301	282	52	105	169	179	129	151	153	3	6	13	19	2	2	8	19	3
206160_at	255	172	167	239	249	302	163	207	158	247	159	146	38	12	40	43	54	16	45	51	25
206161_s_at	93	71	106	87	177	54	119	133	75	109	101	128	26	35	45	30	59	53	37	26	17
206162_x_at	24	12	43	116	227	375	9	7	17	21	20	14	4	18	39	47	8	3	35	5	4
206163_at	108	9	69	81	186	78	66	32	67	42	47	62	2	35	34	14	10	4	31	31	21
206164_at	44	6	5	25	37	26	4	37	31	32	9	9	3	3	6	8	4	18	13	2	1
206165_s_at	146	94	194	76	117	192	205	191	120	155	45	77	13	3	19	8	17	17	14	11	13
206166_s_at	99	99	65	14	122	19	15	50	45	43	60	62	13	30	23	15	25	20	27	3	33
206167_s_at	34	21	38	67	136	59	9	51	47	27	7	55	4	19	2	23	11	6	24	21	13

206168_at	224	189	203	193	216	185	122	244	209	232	159	143	12	29	13	3	3	3	22	3	4
206169_x_at	122	157	125	194	687	884	50	59	193	162	162	255	98	202	152	149	64	323	183	199	156
206170_at	81	62	246	206	47	133	52	96	120	108	153	119	34	33	25	10	21	30	138	59	82
206171_at	212	252	288	219	353	180	241	205	206	345	216	234	42	40	63	36	20	38	51	12	39
206172_at	16	9	18	12	19	22	19	28	13	13	9	11	20	2	3	1	2	3	13	2	9
206173_x_at	385	286	132	57	554	378	269	231	293	239	303	128	299	347	282	288	368	435	478	267	412
206174_s_at	897	757	1592	1405	2159	1321	2033	1613	2060	1849	2980	1822	2234	2335	3035	2279	2114	1607	2397	1679	1611
206175_x_at	37	49	168	48	44	246	61	63	23	43	97	268	121	137	159	73	51	116	102	141	145
206176_at	163	157	167	169	283	298	260	298	150	254	291	312	123	125	135	110	52	64	60	46	50
206177_s_at	22	50	71	91	208	153	52	123	92	106	60	58	1	19	2	19	16	4	32	9	21
206178_at	136	169	119	397	131	105	269	235	310	355	190	211	3	35	5	12	5	17	33	35	37
206179_s_at	11	88	20	75	80	22	77	99	75	50	63	18	5	47	34	26	43	66	32	13	25
206180_x_at	537	416	411	458	223	301	360	446	333	430	581	469	183	104	206	128	93	147	90	160	181
206181_at	127	100	123	109	118	267	114	127	138	161	98	79	6	15	22	36	9	30	42	34	29
206182_at	140	111	31	66	427	280	227	154	15	98	372	214	200	168	224	149	104	43	8	11	28
206183_s_at	11	113	23	133	84	157	110	71	60	145	109	85	29	62	72	26	22	52	18	52	27
206184_at	458	392	619	705	609	744	771	781	1112	1449	766	515	41	44	25	55	54	32	69	71	52
206185_at	11	7	12	13	81	46	15	24	15	25	13	11	3	14	8	6	15	9	4	3	2
206186_at	389	599	574	648	445	359	563	612	535	407	448	292	67	62	80	114	193	129	142	78	106
206187_at	57	83	50	135	150	351	34	57	39	25	121	25	5	47	31	49	54	21	46	47	31
206188_at	197	237	113	36	194	344	61	191	40	61	57	160	122	88	62	240	165	242	149	137	185
206189_at	64	35	15	47	95	144	20	73	20	20	9	11	3	4	19	6	3	39	39	9	15
206190_at	7	5	7	10	23	13	9	5	21	15	10	9	3	6	5	5	4	3	4	6	5
206191_at	153	44	193	122	231	318	309	166	149	252	145	196	11	7	17	5	6	20	32	61	29
206192_at	30	39	45	31	58	48	41	50	36	50	38	31	7	6	10	9	17	30	4	24	9
206193_s_at	16	16	12	16	36	35	20	21	40	29	20	21	4	6	5	5	5	4	8	2	1
206194_at	233	252	95	228	322	672	215	193	253	242	242	441	90	151	162	181	246	111	147	144	145

206195_x_at	13	12	33	31	51	43	17	22	27	28	30
	19	4	4	5	3	3	3	3	7	35	
206196_s_at	292	133	168	118	78	312	217	216	234	129	341
	260	129	92	230	62	52	38	56	64	62	
206197_at	430	282	126	22	177	184	420	391	120	166	129
	143	13	22	3	286	240	269	36	17	41	
206198_s_at	30	9	16	44	30	55	34	4	16	46	20
	23	1	21	8	10	2	1	15	4	14	
206199_at	153	190	228	283	307	216	197	173	217	238	216
	178	26	39	31	21	32	65	134	109	113	
206200_s_at	2420	4371	3215	8916	1894	2457	1924	1896	3772	2503	2065
	2418	3775	3743	1673	3792	2993	3175	3918	4443	2915	
206201_s_at	1	4	4	3	19	31	36	5	3	4	1
	3	3	4	1	1	10	3	13	35	43	
206202_at	3	1	1	3	5	13	3	12	8	6	1
	16	4	2	3	16	2	1	1	8	2	
206203_at	30	90	50	45	84	102	13	42	20	39	34
	23	5	11	9	3	6	19	14	7	11	
206204_at	146	254	449	374	15	32	240	257	467	566	40
	3	4	2	24	209	174	192	1108	904	1315	
206205_at	161	118	370	127	224	215	168	281	158	401	155
	176	279	237	349	354	270	250	660	412	584	
206206_at	30	30	122	30	264	77	37	111	66	106	68
	60	9	9	9	6	8	6	8	31	9	
206207_at	124	67	122	57	128	159	134	112	58	98	124
	29	31	7	37	25	1	5	25	34	16	
206208_at	41	11	15	18	85	30	16	15	19	13	10
	20	15	3	40	6	38	4	16	11	3	
206209_s_at	192	131	191	344	93	70	147	206	333	210	252
	315	35	33	40	23	16	3	5	51	23	
206210_s_at	11	9	15	12	12	9	8	11	11	11	9
	10	2	2	6	3	2	4	3	5	1	
206211_at	33	45	41	43	22	46	41	50	163	36	45
	18	15	2	16	5	2	11	17	4	3	
206212_at	393	229	373	339	371	425	378	371	355	375	324
	366	68	74	35	41	34	52	67	68	66	
206213_at	105	72	90	54	40	125	174	74	155	56	71
	59	23	23	21	128	97	176	25	24	10	
206214_at	291	403	106	54	140	148	261	239	79	63	81
	124	101	46	19	172	190	177	17	10	3	
206215_at	99	47	102	270	49	297	156	113	147	217	220
	98	4	29	9	7	63	3	4	6	5	
206216_at	205	173	279	266	768	809	245	219	373	281	68
	185	12	7	9	13	12	9	43	162	143	
206217_at	296	224	316	273	224	270	262	355	312	316	240
	346	60	62	60	41	28	81	102	61	60	
206218_at	5540	3550	141	122	392	305	5471	4703	203	222	108
	113	45	4	35	5487	4758	6114	31	37	23	
206219_s_at	460	387	130	221	40	23	375	292	210	136	144
	142	19	56	7	200	245	284	55	85	50	
206220_s_at	71	56	134	246	33	36	239	136	266	307	334
	40	35	9	2	7	7	3	15	8	39	
206221_at	7	4	9	4	19	22	5	13	5	6	6
	60	1	2	3	2	3	3	2	1	1	

206222_at	140	330	79	23	264	141	253	199	127	94	95	68	14	39	68	29	37	40	67	9	17
206223_at	67	69	262	191	300	383	167	187	260	303	234	139	59	8	35	33	31	35	49	37	62
206224_at	69	12	53	34	742	5428	24	13	40	35	1603	853	662	704	1188	9	5	22	24	9	3
206225_at	216	120	342	270	495	379	310	300	334	372	250	283	35	5	18	5	22	18	46	68	22
206226_at	130	105	129	149	180	187	93	223	187	129	122	127	29	36	23	59	81	55	20	43	32
206227_at	280	178	323	222	345	321	221	257	284	351	175	253	54	3	12	30	23	39	50	36	52
206228_at	253	193	73	57	263	183	244	302	9	129	141	84	32	41	54	193	229	196	41	45	30
206229_x_at	173	202	136	31	947	890	249	253	158	271	121	64	47	35	67	36	84	70	53	21	29
206230_at	7	5	15	8	95	47	8	7	5	14	108	69	83	31	97	20	10	6	6	19	3
206231_at	292	262	281	378	293	175	270	410	265	358	229	294	7	69	49	79	84	52	59	52	45
206232_s_at	127	11	247	22	11	22	156	181	87	219	188	97	8	3	5	2	2	2	27	36	9
206233_at	185	119	389	109	338	325	276	199	211	207	304	145	196	71	106	82	46	81	132	76	142
206234_s_at	146	288	33	31	241	246	306	226	140	75	71	75	28	24	19	87	12	8	46	49	39
206235_at	258	158	227	208	410	327	257	336	195	177	252	146	143	142	137	191	104	182	146	100	213
206236_at	38	40	115	35	305	353	16	21	118	92	81	107	48	45	94	57	88	77	41	54	38
206237_s_at	17	10	19	26	67	63	28	9	15	21	7	9	3	5	12	5	6	32	4	7	5
206238_s_at	501	364	384	309	498	579	485	401	487	493	566	466	363	439	342	326	332	407	425	352	442
206239_s_at	25	64	2748	22036	353	348	77	53	697	428	91	87	23	9	22	20	26	34	2571	8680	6026
206240_s_at	202	113	186	195	379	355	262	319	187	196	259	189	119	65	95	74	86	107	63	78	88
206241_at	99	49	56	79	47	196	148	129	82	67	58	70	12	22	42	144	89	153	51	72	66
206242_at	28	96	83	182	201	82	90	144	154	268	250	165	91	71	145	3	12	12	91	108	39
206243_at	7	6	282	160	22	312	21	8	185	169	126	103	66	41	45	2	2	4	626	658	539
206244_at	9	10	14	40	22	26	11	17	23	11	16	9	2	6	6	6	16	14	4	12	7
206245_s_at	987	1308	1455	1482	1021	1429	815	1281	1770	1586	839	559	849	745	483	1247	1153	1301	2696	1583	2348
206246_at	130	38	87	72	77	55	90	102	50	31	155	33	26	11	6	12	42	13	8	15	8
206247_at	914	420	43	31	1120	851	949	813	35	57	640	745	1274	1332	757	2249	1947	2646	75	252	221
206248_at	228	152	123	229	95	71	244	226	325	173	192	219	8	2	5	8	12	8	9	9	7

206249_at	75	89	85	235	180	196	27	50	139	53	41	36	2	5	31	41	42	19	95	98	66
206250_x_at	20	18	20	17	66	118	23	30	21	17	18	21	8	27	4	5	9	15	6	7	8
206251_s_at	78	36	94	30	120	128	37	57	31	41	30	41	15	7	2	2	16	21	18	22	3
206252_s_at	48	22	69	10	62	168	53	61	9	6	36	8	13	22	5	5	4	4	35	2	17
206253_at	85	4	75	47	16	16	40	7	29	45	54	64	2	3	11	3	1	3	3	26	13
206254_at	44	77	31	62	14	81	90	45	28	49	48	62	2	21	29	26	5	17	30	3	5
206255_at	15	7	12	53	21	23	30	18	59	20	16	113	26	4	4	3	3	6	5	1	2
206256_at	235	232	180	278	84	69	184	222	215	186	210	212	19	56	54	43	50	49	57	46	39
206257_at	513	585	595	610	767	664	746	479	537	685	573	528	135	173	144	190	220	165	177	112	128
206258_at	8	9	11	10	21	17	15	7	8	10	7	14	6	4	2	11	5	9	6	12	19
206259_at	29	9	14	122	88	180	7	13	7	15	64	41	48	66	86	4	7	8	24	96	45
206260_at	197	156	92	144	220	153	133	94	136	134	145	192	31	43	49	36	24	4	20	11	13
206261_at	362	280	526	318	191	187	398	294	492	549	171	187	59	45	71	207	294	211	748	455	512
206262_at	17	28	22	171	121	149	56	18	39	38	10	14	16	32	41	11	22	34	30	7	32
206263_at	388	299	457	498	415	491	526	545	389	433	335	372	35	48	36	138	103	102	93	76	60
206264_at	116	120	194	199	195	128	178	197	165	182	152	190	6	6	26	15	5	8	22	24	29
206265_s_at	21	24	38	70	14	9	9	40	45	48	43	57	11	40	2	2	3	2	3	14	3
206266_at	16	16	15	31	81	42	13	15	15	10	13	24	2	6	5	5	15	4	4	3	3
206267_s_at	34	27	42	39	106	314	27	28	66	20	36	24	38	13	50	39	27	46	61	87	57
206268_at	172	91	153	169	153	257	127	154	167	124	203	90	14	3	27	45	50	44	95	74	39
206269_at	71	15	54	27	26	8	64	42	27	20	60	69	10	9	26	5	17	4	4	4	22
206270_at	17	10	7	6	16	19	8	8	7	18	6	6	32	4	3	9	4	4	4	4	3
206271_at	54	197	83	202	118	245	115	108	62	36	13	68	34	23	47	65	105	287	34	82	155
206272_at	655	886	385	1125	331	652	525	603	479	439	262	303	403	323	732	1097	800	543	1080	837	1210
206273_at	32	84	34	28	16	54	19	33	35	22	90	21	28	7	37	19	101	65	103	61	58
206274_s_at	218	238	24	93	249	157	131	240	51	172	118	117	71	20	37	14	49	29	8	34	38
206275_s_at	122	34	167	132	451	454	57	103	162	328	162	122	25	24	48	42	28	49	54	32	9

206276_at	29	28	14	21	188	310	17	33	17	15	17	10	4	7	7	29	9	7	5	3	2
206277_at	34	51	483	415	26	28	66	17	698	527	80	14	25	9	11	47	28	48	211	104	84
206278_at	20	116	7	35	25	23	25	33	50	35	28	13	33	45	22	47	45	55	18	39	51
206279_at	75	123	60	100	107	19	123	63	48	109	107	69	29	39	3	34	16	42	25	35	16
206280_at	112	69	193	62	278	210	220	172	131	173	104	99	3	10	4	9	6	6	7	21	5
206281_at	156	134	161	185	390	216	204	182	119	246	149	166	11	8	21	19	7	6	44	21	4
206282_at	17	9	38	18	26	70	66	9	9	18	16	15	2	9	13	2	2	3	5	4	3
206283_s_at	102	58	132	78	70	155	106	88	99	155	139	143	3	30	29	35	49	3	22	37	14
206284_x_at	1164	1605	1282	983	1875	2080	1627	794	1349	949	3277	1976	1891	1287	1475	291	377	319	392	342	197
206285_at	21	39	16	36	19	24	27	33	27	75	17	19	10	8	15	12	7	30	27	25	5
206286_s_at	19	27	57	32	351	243	13	103	23	14	196	98	200	363	701	8	5	55	220	706	975
206287_s_at	17	22	15	17	23	23	17	15	20	21	13	25	2	12	3	2	4	6	10	4	2
206288_at	218	176	197	159	18	11	412	307	274	238	94	14	225	207	105	225	241	256	304	195	230
206289_at	169	94	221	164	263	105	175	62	151	122	286	239	122	158	171	69	84	69	91	153	88
206290_s_at	11	56	68	50	165	124	54	58	92	102	30	57	15	3	13	9	6	29	20	46	39
206291_at	20	7	46	62	183	138	77	44	55	77	9	13	2	3	2	5	5	34	205	160	231
206292_s_at	65	58	90	136	74	51	66	137	17	95	99	75	32	2	41	5	23	27	34	39	4
206293_at	45	55	87	36	74	19	50	32	56	24	26	44	1	12	3	5	1	3	10	19	1
206294_at	29	142	41	65	195	220	109	124	100	28	163	55	4	16	5	39	21	6	20	40	13
206295_at	832	999	517	301	5	9	518	691	139	377	20	5	2	3	1	1370	1352	790	423	943	527
206296_x_at	30	11	19	10	38	27	15	25	17	20	18	20	46	8	18	37	10	13	6	8	3
206297_at	245	169	189	109	452	238	232	143	98	187	193	128	45	35	8	40	25	33	58	57	19
206298_at	131	131	16	78	16	13	60	91	20	17	24	33	2	6	5	14	52	35	3	2	2
206299_at	479	409	123	123	360	153	686	366	135	180	87	52	9	38	50	234	257	198	57	16	50
206300_s_at	115	47	81	78	175	172	40	25	50	64	91	68	29	55	44	29	16	12	69	58	56
206301_at	19	10	8	14	25	35	15	15	12	14	11	10	7	8	34	4	3	7	4	7	21
206302_s_at	1037	507	2648	3087	1839	1529	782	805	1630	2463	1730	1680	1816	1737	2140	768	698	593	2997	4123	3822

206303_s_at	348	457	1606	1923	859	825	403	451	1714	2105	1414	716	934	502	470	350	282	253	760	648	610
206304_at	79	64	22	38	44	36	110	84	60	49	70	108	11	7	4	7	14	5	12	7	1
206305_s_at	38	57	46	34	45	189	62	55	33	38	24	33	11	6	11	8	15	14	7	11	5
206306_at	151	80	76	128	148	230	115	104	60	122	75	74	25	26	24	17	15	29	41	28	29
206307_s_at	761	771	113	138	492	521	421	784	134	200	610	484	485	965	850	1016	1236	1115	175	281	250
206308_at	200	173	138	57	96	185	125	156	207	123	146	127	69	84	37	160	140	125	188	127	196
206309_at	271	133	220	286	290	132	268	191	218	253	212	293	9	9	6	26	16	11	31	39	29
206310_at	107	118	37	40	47	52	118	88	35	96	30	69	1	15	7	47	70	71	10	45	27
206311_s_at	19	6	47	80	104	132	44	45	28	34	45	84	15	19	6	24	6	11	9	22	16
206312_at	74	63	27	52	249	266	73	96	60	13	38	57	11	19	13	7	3	6	6	6	2
206313_at	124	120	195	31	356	243	146	117	128	141	95	145	35	30	28	46	51	40	28	49	37
206314_at	16	33	7	6	11	19	12	5	7	13	4	4	3	29	2	2	14	10	17	21	1
206315_at	284	246	12	13	430	267	383	496	13	7	10	10	25	57	39	300	399	252	47	30	5
206316_s_at	425	206	468	226	425	257	507	697	591	725	419	517	271	293	356	269	247	359	601	232	451
206317_s_at	33	51	31	65	36	47	24	157	23	133	37	31	42	91	127	110	71	89	144	76	59
206318_at	107	73	61	6	146	175	72	83	100	67	77	74	14	10	2	13	23	36	29	32	11
206319_s_at	194	64	168	125	252	138	143	120	99	108	146	111	16	4	42	13	42	23	39	17	19
206320_s_at	13	16	12	27	44	38	11	11	8	14	7	10	2	7	10	5	4	9	6	7	4
206321_at	20	6	18	18	16	24	12	8	32	8	11	9	3	7	5	3	3	2	3	2	2
206322_at	22	18	9	12	320	50	50	24	27	15	11	49	13	20	37	7	16	7	26	15	6
206323_x_at	1102	1535	2208	2761	1458	1412	2145	3084	2749	2600	2207	1896	925	1093	2650	2130	1835	2934	2553	2687	2146
206324_s_at	107	154	194	212	250	308	93	131	48	59	81	59	6	11	11	15	8	35	7	37	11
206325_at	36	12	39	41	22	17	38	44	35	41	27	57	7	6	5	5	21	22	55	11	43
206326_at	30	21	28	22	208	94	66	17	17	20	18	21	2	7	10	3	12	6	4	7	21
206327_s_at	311	109	145	43	59	67	374	346	290	273	124	214	20	19	16	12	43	13	43	44	17
206328_at	17	27	11	18	272	309	9	20	7	8	7	8	2	6	6	30	71	15	8	7	2
206329_at	52	12	45	21	92	124	9	55	25	24	13	10	5	4	50	11	24	12	14	12	9

206330_s_at	17	16	15	19	55	98	24	29	24	14	13	23	4	5	3	5	7	3	5	12	9
206331_at	46	88	84	138	47	163	80	144	76	144	85	73	2	3	28	41	30	37	15	29	33
206332_s_at	8	68	7	28	23	19	58	26	44	21	10	16	10	13	9	8	12	26	11	97	118
206333_at	22	26	8	16	137	151	19	88	11	17	11	44	6	5	4	24	8	34	4	22	5
206334_at	38	39	14	76	71	22	87	16	82	61	23	14	2	14	14	2	18	40	32	23	6
206335_at	718	994	699	1265	388	570	436	625	636	780	404	400	388	395	401	802	1324	851	1057	1346	897
206336_at	45	52	220	129	120	109	73	51	138	99	94	79	15	9	13	9	20	22	139	93	190
206337_at	103	46	87	110	250	56	94	116	132	109	370	251	171	237	148	18	17	9	19	2	21
206338_at	29	18	27	23	421	349	21	22	25	22	13	20	8	23	10	11	15	15	10	11	7
206339_at	191	151	91	52	298	270	216	186	185	198	77	274	5	36	13	50	7	5	66	41	37
206340_at	48	82	56	28	93	90	85	66	76	66	20	33	10	12	4	5	23	1	36	9	12
206341_at	5	9	12	12	132	83	40	32	19	18	6	9	17	31	3	2	6	11	19	2	3
206342_x_at	347	417	1068	970	374	599	433	519	813	418	1138	1044	740	823	1128	554	608	515	819	606	469
206343_s_at	21	72	52	16	29	9	86	34	58	10	16	6	30	8	11	126	237	314	3	9	1
206344_at	15	19	68	21	202	140	76	53	111	73	61	64	26	2	2	12	2	2	1	13	25
206345_s_at	263	78	76	216	71	160	168	172	193	158	112	158	30	26	32	26	9	52	71	109	151
206346_at	61	41	30	44	11	70	32	30	21	36	7	24	7	40	2	8	3	2	49	4	28
206347_at	161	112	76	127	311	300	172	150	186	177	149	170	61	67	98	68	71	72	89	111	70
206348_s_at	9	7	23	25	33	20	15	21	56	24	38	117	201	391	310	69	68	61	164	48	77
206349_at	7	9	8	17	22	34	23	9	12	8	11	10	1	2	2	1	2	1	1	1	1
206350_at	132	106	117	67	260	151	154	103	135	136	94	165	44	17	42	19	38	23	39	40	38
206351_s_at	388	457	361	304	515	360	387	466	301	429	550	270	155	142	103	128	126	95	186	77	38
206352_s_at	443	781	427	392	471	594	513	392	377	579	590	464	395	330	498	428	492	327	346	338	186
206353_at	202	164	307	334	447	200	297	314	284	198	239	211	64	62	46	112	47	58	47	82	78
206354_at	200	156	1337	1172	18	40	144	143	577	759	10	36	26	51	42	445	365	351	1090	1910	3125
206355_at	97	159	31	96	245	290	127	149	45	42	523	160	238	557	420	50	69	64	76	63	70
206356_s_at	75	129	7	22	44	17	103	80	7	8	195	70	99	195	207	55	29	4	44	36	29

206357_at	61	22	115	107	374	430	62	66	151	196	163	70	242	71	27	12	4	8	101	77	62
206358_at	36	13	23	19	84	366	57	18	55	61	36	63	37	40	6	31	36	36	19	6	20
206359_at	58	38	39	49	65	65	12	34	9	11	48	29	41	45	15	18	10	34	4	12	1
206360_s_at	192	208	184	286	558	435	229	256	193	296	190	178	92	100	36	51	53	35	55	69	50
206361_at	101	151	72	19	34	28	140	164	134	102	165	92	31	86	56	47	103	45	66	46	40
206362_x_at	33	32	42	36	66	63	36	36	33	34	40	38	11	10	6	8	8	13	15	10	8
206363_at	25	15	8	23	41	20	17	18	16	21	11	14	4	3	5	2	7	2	2	3	1
206364_at	1004	157	1153	129	1304	202	808	1082	1118	938	986	840	868	537	604	430	490	667	1239	338	436
206365_at	20	54	58	120	22	91	23	28	76	50	57	57	1	3	6	3	28	3	18	9	15
206366_x_at	34	7	14	31	96	43	30	24	12	20	20	16	3	7	2	2	2	15	2	82	26
206367_at	34	102	141	65	44	126	122	15	182	42	40	116	6	11	9	5	6	5	20	11	11
206368_at	64	11	39	45	59	93	29	77	48	73	134	16	18	4	34	29	11	11	32	2	3
206369_s_at	17	18	23	31	37	35	11	12	12	22	11	11	3	5	3	1	3	1	6	4	3
206370_at	46	2	7	45	7	12	1	22	45	15	3	36	19	11	9	2	16	1	3	21	7
206371_at	94	21	26	28	131	228	125	26	41	56	149	139	41	51	29	45	23	15	17	11	8
206372_at	25	19	27	89	56	210	27	36	88	39	27	21	2	6	9	2	3	31	9	3	4
206373_at	20	6	8	9	10	17	11	11	13	21	10	20	2	5	5	2	3	24	1	2	3
206374_at	238	1103	339	415	345	539	176	195	260	180	131	83	56	37	54	57	73	92	97	152	90
206375_s_at	130	46	107	47	85	71	99	58	123	157	102	54	5	9	10	7	21	6	13	17	1
206376_at	9	11	7	8	11	28	8	5	7	7	4	6	3	2	1	2	14	3	2	4	4
206377_at	134	54	178	49	717	535	76	121	107	89	769	450	531	578	493	19	15	5	16	101	108
206378_at	29	41	20	16	34	20	64	12	41	7	20	9	3	7	26	27	16	24	4	8	13
206379_at	32	27	49	43	49	78	146	182	27	49	38	59	11	34	47	2	59	2	34	37	8
206380_s_at	7	6	7	8	10	11	8	8	7	7	6	8	2	1	3	7	4	17	1	10	2
206381_at	26	15	27	26	76	40	16	54	44	36	17	78	14	13	10	14	3	18	19	2	5
206382_s_at	286	189	60	25	239	102	147	161	44	13	146	85	458	314	308	1151	944	1111	21	38	3
206383_s_at	495	251	437	338	473	234	912	699	779	799	648	435	172	184	54	169	160	174	204	137	133

206384_at	15	22	12	18	300	27	12	18	21	14	13	11	3	5	6	3	3	2	4	32	1
206385_s_at	224	429	598	885	236	230	285	259	476	559	146	43	87	83	326	300	284	353	919	929	1036
206386_at	77	106	141	26	470	419	20	92	62	106	51	15	21	6	19	36	13	12	46	18	32
206387_at	110	235	9	13	164	124	33	96	19	15	220	33	151	161	52	8	13	9	6	3	9
206388_at	171	112	197	281	110	134	167	251	158	189	129	101	14	39	26	3	16	22	18	18	13
206389_s_at	98	17	50	50	59	105	95	21	32	14	45	20	5	6	5	9	5	1	29	24	6
206390_x_at	164	164	145	310	84	79	277	146	203	224	81	77	13	12	12	10	29	11	89	69	86
206391_at	12	11	58	343	26	54	41	13	12	15	14	16	7	6	6	2	47	2	15	62	17
206392_s_at	11	4	26	1323	12	9	7	21	17	85	10	9	3	2	3	2	7	2	137	114	110
206393_at	15	7	8	25	32	23	11	18	15	11	17	11	3	3	5	3	3	3	5	2	3
206394_at	11	6	5	10	16	78	8	7	13	6	21	6	25	21	47	19	37	2	12	29	35
206395_at	110	52	31	19	454	614	122	146	20	49	441	182	215	259	191	138	99	92	25	36	32
206396_at	232	248	199	229	110	187	435	402	317	1152	21	14	14	27	1	31	59	37	50	50	160
206397_x_at	28	41	27	19	80	58	48	26	29	21	28	168	96	141	113	8	6	8	5	5	15
206398_s_at	57	51	174	199	44	89	86	48	143	75	159	97	4	24	50	19	30	34	60	6	4
206399_x_at	17	9	15	13	286	200	13	29	15	10	9	13	9	6	22	18	8	8	11	6	6
206400_at	24	112	88	76	224	67	17	15	54	28	51	5	42	55	36	16	38	8	8	94	69
206401_s_at	593	519	85	107	198	300	651	578	41	49	138	141	151	133	58	107	167	96	16	14	22
206402_s_at	28	23	20	35	38	65	13	25	21	27	27	16	11	8	6	5	8	12	12	19	13
206403_at	181	127	170	164	381	284	147	238	186	184	173	97	3	39	87	43	28	40	60	59	33
206404_at	882	506	107	502	834	875	681	616	281	221	414	514	298	224	194	206	197	144	192	153	268
206405_x_at	852	636	916	926	760	723	576	827	644	984	664	642	174	198	208	209	220	180	401	362	321
206406_at	22	22	35	22	33	17	32	26	27	28	18	15	2	3	3	2	3	3	8	4	6
206407_s_at	56	54	19	48	43	30	139	88	149	101	54	90	6	8	7	8	48	4	10	32	13
206408_at	22	17	49	28	166	116	81	28	24	24	58	25	5	15	28	1	2	1	25	8	11
206409_at	249	44	19	36	199	42	74	170	17	20	146	177	57	36	19	41	20	41	11	10	8
206410_at	139	114	159	268	93	164	111	129	95	50	99	28	28	6	63	15	5	11	95	62	37

206411_s_at	401	287	316	268	381	231	231	406	381	389	226	122	56	26	30	48	50	70	58	61	4
206412_at	336	210	140	162	62	31	400	491	60	108	111	128	107	187	187	429	548	422	135	141	199
206413_s_at	119	106	47	83	82	50	57	86	92	74	21	73	6	10	9	9	14	12	48	46	12
206414_s_at	521	659	3142	2704	1823	1414	444	479	2415	4196	1990	2262	1829	2419	2566	1204	1239	997	4148	2921	4443
206415_at	4	10	7	38	78	50	37	33	33	29	48	23	1	4	2	8	8	2	9	2	2
206416_at	49	140	22	26	830	443	85	58	25	10	20	38	107	113	73	148	189	125	121	129	100
206417_at	147	271	155	357	179	280	154	133	221	277	104	119	26	36	44	92	45	33	69	103	82
206418_at	4	2	393	275	10	24	17	12	640	1093	7	4	10	4	2	2	9	15	2294	1753	3535
206419_at	232	201	300	288	56	73	363	285	78	300	183	234	60	26	81	69	57	46	8	53	11
206420_at	34	6	77	72	65	78	95	59	7	55	24	53	23	22	17	2	24	3	27	11	8
206421_s_at	19	21	24	81	32	31	20	16	17	34	14	21	2	4	5	3	3	4	4	7	19
206422_at	20	10	9	12	91	39	9	9	21	24	11	6	2	8	19	1	2	6	3	3	15
206423_at	19	13	7	9	52	36	11	41	12	18	10	72	4	34	3	8	3	7	13	33	23
206424_at	46	142	35	57	305	387	58	55	78	81	119	29	99	118	27	38	38	44	7	3	28
206425_s_at	98	56	113	92	122	34	90	86	108	115	94	113	22	15	17	13	29	9	18	2	11
206426_at	20	39	38	59	14	66	57	54	60	25	43	10	17	19	23	11	12	20	2	24	3
206427_s_at	146	90	136	76	135	168	178	166	122	134	153	30	14	3	8	16	2	16	5	24	4
206428_s_at	9	13	9	13	14	12	12	13	7	11	9	8	4	2	2	3	3	2	3	4	1
206429_at	2113	1815	2257	1133	261	452	2200	2854	860	2279	662	501	72	56	80	952	765	942	358	214	585
206430_at	36	17	23	40	98	86	21	29	28	55	26	18	4	22	5	20	12	18	15	7	11
206431_x_at	992	1437	992	1610	1021	1152	1179	1281	1231	1332	978	1017	382	366	378	258	185	393	360	386	309
206432_at	46	17	53	87	154	38	61	34	78	57	78	50	342	336	160	12	23	24	60	2	52
206433_s_at	56	17	5	32	44	116	64	86	80	4	3	53	25	14	19	18	5	6	20	19	3
206434_at	48	5	1	10	41	34	28	63	1	7	24	49	19	11	1	27	15	2	11	1	7
206435_at	406	460	225	198	69	173	458	455	285	242	176	195	89	93	43	192	203	112	81	69	108
206436_at	142	100	144	260	304	54	189	228	68	126	81	35	7	58	39	89	65	73	66	9	29
206437_at	20	84	34	100	77	89	37	29	31	196	124	65	34	15	54	13	61	44	46	50	19

206438_x_at	823	448	484	701	421	603	829	587	510	545	436	555	150	188	250	263	207	120	477	301	268
206439_at	44	4	4	75	62	188	7	37	54	34	78	23	13	3	9	1	3	6	5	9	3
206440_at	13	9	8	19	29	30	53	77	15	18	9	11	20	21	3	59	8	54	5	15	6
206441_s_at	1229	1125	1447	1244	742	509	1019	987	1973	1589	1145	984	2346	1680	1078	849	890	859	2354	1356	1029
206442_at	16	41	69	116	151	15	57	44	71	64	10	72	10	3	29	3	5	1	50	93	61
206443_at	25	7	7	13	37	19	17	16	5	11	11	16	3	3	7	3	4	8	3	6	1
206444_at	21	17	23	62	183	286	37	22	64	28	40	36	5	8	6	12	10	7	8	11	7
206445_s_at	9328	10509	11405	5371	7663	4620	9654	8340	15233	9874	10808	5194	10157	9093	5086	6214	6211	7513	7224	7077	5426
206446_s_at	17	26	47	14	41	24	8	13	45	6	27	58	4	4	26	2	4	7	29	29	3
206447_at	9	11	12	13	70	54	37	20	8	10	4	9	11	3	7	3	3	8	2	7	2
206448_at	283	204	72	92	239	309	254	193	92	77	128	147	42	48	74	226	154	190	57	47	29
206449_s_at	118	60	71	153	60	30	78	92	120	106	70	113	10	4	25	28	28	23	29	14	29
206450_at	24	44	23	32	132	22	11	11	21	27	40	28	10	4	52	4	6	3	11	11	9
206451_at	597	319	292	281	686	568	315	347	186	190	308	431	383	412	363	473	459	462	641	470	665
206452_x_at	1283	1066	891	1169	772	757	1179	852	1136	637	1163	351	429	236	222	247	245	218	311	231	153
206453_s_at	319	119	189	407	173	141	400	113	138	238	125	212	18	65	29	132	114	139	153	27	31
206454_s_at	21	13	7	31	55	69	66	16	39	14	23	63	29	6	2	5	25	4	2	6	2
206455_s_at	164	88	122	190	205	65	158	94	163	105	47	129	44	6	27	7	1	35	47	54	14
206456_at	50	44	14	4	21	22	19	40	20	6	4	36	10	1	11	13	1	10	2	3	9
206457_s_at	99	71	33	48	96	103	119	87	41	85	87	65	4	9	25	17	1	17	25	17	3
206458_s_at	57	72	79	132	87	35	127	150	39	137	18	60	23	30	19	38	34	5	21	29	19
206459_s_at	78	103	80	17	307	294	114	98	79	102	70	102	32	12	44	22	12	31	54	62	31
206460_at	116	60	75	89	155	161	110	78	87	77	68	93	13	4	35	35	15	4	3	46	5
206461_x_at	17635	12993	4575	2927	4650	4806	12052	16900	3519	3199	5267	20171	6752	5148	4318	9385	12154	6797	4556	3982	1675
206462_s_at	58	40	61	164	143	55	146	79	124	94	121	70	32	28	42	17	26	52	30	31	36
206463_s_at	45	38	228	26	1436	78	424	786	281	632	1737	3387	1072	348	466	29	21	34	125	83	71
206464_at	42	18	34	25	25	38	28	33	28	32	26	28	5	3	8	4	3	6	6	6	4

206465_at	126	73	90	120	175	69	111	123	36	80	48	133	11	3	7	25	12	9	8	17	21
206466_at	16	9	14	75	124	56	86	32	16	22	33	58	6	5	26	4	2	6	15	32	7
206467_x_at	2314	887	270	296	599	445	884	1153	314	259	1376	1443	409	197	171	529	455	308	64	49	51
206468_s_at	714	675	888	600	667	159	864	859	916	928	647	495	207	253	187	245	237	256	360	162	286
206469_x_at	841	859	906	1459	570	489	447	583	864	855	466	460	291	281	234	309	291	230	627	531	424
206470_at	22	16	15	12	8	34	8	11	9	15	16	15	2	3	9	3	7	9	2	6	3
206471_s_at	83	38	38	39	33	54	119	106	41	92	14	13	5	18	15	3	8	17	39	34	2
206472_s_at	266	170	199	153	444	202	217	218	221	147	702	280	333	350	222	78	121	112	121	76	29
206473_at	192	236	213	171	401	294	498	561	203	270	598	397	148	73	137	129	154	99	69	20	118
206474_at	237	111	201	155	236	235	237	248	193	226	203	239	34	33	66	52	45	43	49	32	45
206475_x_at	16	17	23	21	55	42	23	25	23	25	23	14	42	2	6	6	13	3	22	37	5
206476_s_at	12	7	9	6	23	17	4	15	4	15	9	5	7	6	3	10	1	2	10	11	13
206477_s_at	176	109	60	93	309	421	56	103	72	80	179	297	20	15	67	65	57	93	111	65	39
206478_at	254	164	233	194	458	306	236	286	173	205	172	132	42	31	48	17	33	3	36	33	23
206479_at	36	12	68	47	30	38	50	11	63	78	40	1	20	39	36	9	4	2	27	7	5
206480_at	19	21	16	14	32	20	17	17	16	15	11	20	4	8	3	3	2	9	4	11	3
206481_s_at	45	10	9	40	59	74	33	42	5	34	40	68	5	3	2	3	2	4	1	4	3
206482_at	142	109	370	519	422	669	193	108	178	129	317	250	322	223	833	141	173	139	417	474	666
206483_at	275	202	62	242	155	126	315	360	123	112	132	146	64	46	54	242	251	243	88	112	96
206484_s_at	40	27	132	57	111	187	38	61	80	144	131	96	25	38	52	18	15	51	34	59	5
206485_at	82	169	248	378	261	399	73	80	127	201	183	221	63	61	27	13	15	28	21	20	17
206486_at	69	36	20	17	203	81	28	44	25	11	24	70	11	2	5	8	2	2	39	2	11
206487_at	24	32	66	23	175	231	17	44	75	57	31	53	202	169	343	97	92	127	264	232	219
206488_s_at	48	56	90	8	230	130	76	12	25	96	47	101	2	5	5	4	12	25	12	7	2
206489_s_at	38	35	12	61	78	27	34	36	9	34	53	98	10	12	21	5	13	3	1	24	2
206490_at	67	4	83	100	37	270	56	82	48	45	45	40	18	23	18	3	34	3	21	14	3
206491_s_at	761	935	1849	1702	341	443	987	1053	1893	1563	1433	775	619	678	754	614	635	835	1181	1258	1048

206492_at	409	345	53	92	169	228	311	307	127	116	107	147	48	58	85	511	432	330	29	28	29
206493_at	175	167	206	173	756	458	216	181	127	161	206	161	27	90	86	67	65	58	60	41	31
206494_s_at	22	9	20	8	29	9	21	18	11	15	10	15	2	2	3	5	3	19	1	2	2
206495_s_at	458	431	171	356	623	843	391	520	221	210	303	475	292	276	287	353	357	423	230	313	323
206496_at	9	4	16	8	36	42	20	12	13	29	10	14	1	3	2	27	6	19	20	42	49
206497_at	17	44	65	36	213	155	34	20	20	64	121	92	78	75	5	2	34	29	49	19	36
206498_at	91	60	43	119	93	200	24	131	29	82	17	38	20	5	53	33	37	59	14	22	3
206499_s_at	2582	2100	3076	841	2146	926	3933	2381	3243	2762	2844	1924	1147	757	545	912	1032	1133	899	562	350
206500_s_at	9	40	72	17	18	16	101	77	64	63	13	67	151	104	53	46	34	54	104	44	50
206501_x_at	255	210	256	283	441	415	333	430	276	442	269	348	68	58	44	79	66	85	57	82	77
206502_s_at	16	7	56	6	84	11	32	7	76	66	13	8	17	3	27	1	7	10	2	26	8
206503_x_at	19	16	16	36	19	20	11	26	13	14	189	40	47	109	65	10	24	165	93	93	122
206504_at	574	247	95	5	220	58	113	121	99	57	225	294	270	133	163	462	425	1025	86	43	45
206505_at	4	23	1	18	5	15	20	48	4	1	4	5	1	17	12	1	1	20	11	16	2
206506_s_at	504	269	184	85	111	254	302	241	122	71	217	310	243	231	226	306	401	272	151	262	251
206507_at	138	52	110	103	260	265	9	84	92	64	102	136	87	70	66	55	86	127	144	134	160
206508_at	311	229	129	85	876	887	62	183	108	126	600	464	113	252	196	38	32	92	42	7	26
206509_at	175	108	16	128	220	176	205	102	124	48	88	157	48	48	25	55	11	50	25	66	39
206510_at	197	63	115	144	285	245	107	123	174	165	217	202	66	40	61	29	34	37	45	56	63
206511_s_at	48	94	102	111	267	284	126	117	48	159	185	131	22	14	57	4	25	8	6	31	6
206512_at	9	6	7	12	59	31	11	8	9	7	7	6	8	6	7	4	29	9	6	39	19
206513_at	201	125	87	140	106	17	146	183	37	259	162	199	16	19	17	24	30	4	17	43	32
206514_s_at	13	39	23	17	52	20	15	41	7	15	11	13	7	2	2	4	5	17	8	16	4
206515_at	97	272	54	122	96	19	134	92	98	48	77	67	4	29	26	144	116	80	27	27	11
206516_at	28	85	95	171	37	93	27	16	24	131	18	30	52	11	10	52	11	82	88	59	53
206517_at	32	16	64	30	15	16	19	17	104	20	6	6	2	10	27	16	6	30	39	8	9
206518_s_at	46	38	5	31	616	692	78	62	21	10	495	167	256	165	121	59	10	29	27	14	16

206519_x_at	8	6	7	4	54	8	3	3	5	4	6
	6	25	22	2	2	3	1	6	3	20	
206520_x_at	270	246	441	396	245	411	403	508	365	524	377
	413	51	33	57	49	54	64	67	91	72	
206521_s_at	118	19	15	59	239	176	147	178	32	85	126
	92	25	45	3	33	7	6	34	7	3	
206522_at	5	69	5	14	25	23	13	8	11	4	7
	8	2	5	3	3	6	7	4	6	20	
206523_at	16	24	14	13	876	394	23	16	13	14	459
	323	111	85	131	17	25	51	6	19	7	
206524_at	66	40	68	153	855	468	41	94	45	55	370
	273	219	156	231	43	59	70	114	64	82	
206525_at	126	49	114	145	165	168	195	98	131	113	114
	116	21	13	8	11	12	7	21	21	20	
206526_at	12	124	14	19	33	9	13	12	13	14	171
	41	87	88	112	34	90	47	43	104	67	
206527_at	243	129	164	274	608	587	261	240	230	212	529
	305	94	105	111	59	46	46	64	119	54	
206528_at	53	12	11	13	32	12	61	83	60	60	51
	65	3	3	10	2	3	2	29	26	36	
206529_x_at	67	32	12	52	110	296	30	42	24	45	68
	43	292	173	209	27	39	2	19	27	20	
206530_at	168	95	167	213	224	46	171	172	225	225	175
	182	48	64	27	31	22	15	63	61	45	
206531_at	77	28	42	40	71	58	53	45	23	42	47
	28	17	13	17	17	19	20	12	11	8	
206532_at	164	16	117	18	153	106	91	20	31	49	163
	35	31	19	18	41	10	52	46	41	20	
206533_at	382	237	1039	466	532	310	331	438	1033	1808	496
	418	262	304	403	322	315	306	882	603	652	
206534_at	24	79	88	168	102	156	103	102	87	136	71
	64	29	35	2	9	3	2	8	3	39	
206535_at	4	7	4	12	12	22	7	9	4	10	16
	8	3	1	5	2	1	17	28	3	32	
206536_s_at	167	109	228	226	436	308	121	176	186	229	190
	151	132	69	123	61	123	120	132	148	203	
206537_at	136	92	221	116	241	336	111	137	206	233	179
	158	120	125	122	78	89	85	104	87	151	
206538_at	33	43	18	164	298	24	53	88	41	17	23
	68	41	24	41	56	43	8	25	41	33	
206539_s_at	62	184	41	288	293	253	48	66	52	53	34
	63	4	11	71	40	104	104	74	12	22	
206540_at	149	185	189	475	304	60	347	276	171	268	232
	113	64	74	79	79	38	35	90	94	43	
206541_at	58	60	47	57	45	40	74	32	20	43	54
	14	3	34	17	7	16	6	8	6	13	
206542_s_at	153	105	289	338	529	673	200	144	268	257	449
	528	579	378	135	58	93	24	529	279	349	
206543_at	97	71	69	57	74	194	76	44	32	77	44
	55	16	9	34	38	38	47	60	31	40	
206544_x_at	160	56	336	250	327	391	152	116	292	340	466
	524	385	156	34	29	21	11	151	112	175	
206545_at	62	67	62	76	80	112	86	139	68	110	70
	85	10	3	9	3	2	7	8	13	8	

206546_at	3	5	28	36	67	8	16	22	16	10	23	52	9	13	8	4	13	1	19	41	25
206547_s_at	91	23	26	115	18	99	103	24	106	109	104	112	22	23	27	15	45	31	32	27	15
206548_at	327	364	231	228	142	128	209	777	340	358	68	72	10	23	14	46	13	96	41	37	35
206549_at	175	79	142	220	126	316	191	211	165	175	136	136	28	8	11	8	35	35	27	11	15
206550_s_at	794	370	746	491	704	746	1182	710	974	872	833	719	630	576	819	821	649	984	1255	540	719
206551_x_at	188	168	232	283	73	597	199	227	347	310	203	408	213	126	107	73	99	117	168	278	290
206552_s_at	38	21	61	49	58	121	37	48	28	56	20	52	13	2	1	5	17	17	8	1	7
206553_at	34	91	69	246	104	266	94	92	28	91	284	134	123	131	158	10	8	41	57	177	253
206554_x_at	206	157	96	16	239	242	211	165	139	87	111	150	196	119	108	213	277	233	156	107	126
206555_s_at	512	505	713	1013	368	343	628	897	638	628	334	480	820	706	1002	1520	1113	940	3255	2022	3200
206556_at	108	32	20	88	180	77	42	83	86	91	48	43	10	2	32	12	20	4	6	6	7
206557_at	128	46	203	252	159	137	97	157	122	148	51	108	26	5	18	24	25	3	81	78	126
206558_at	303	466	220	247	415	505	388	369	170	233	188	161	57	59	73	205	180	169	90	124	129
206559_x_at	32630	50140	35570	48352	40551	45908	39495	39762	43920	35428	30607	49127	20245	28166	30444	28378	30123	30720	18638	27458	25537
206560_s_at	12	41	269	331	2511	2442	175	58	396	495	3830	4216	4283	3512	1446	100	111	92	466	298	189
206561_s_at	152	154	1875	3601	98	312	265	131	8038	13368	65	70	34	3	37	73	48	69	4956	1782	1405
206562_s_at	3098	2438	2792	1900	2201	1902	4207	3754	2929	2644	3971	3093	2673	2062	1432	1475	1230	1383	1114	1500	2082
206563_s_at	126	30	123	114	38	65	52	206	71	87	81	50	37	5	7	29	7	31	36	32	19
206564_at	155	140	168	243	650	774	97	141	151	187	135	151	12	61	57	50	69	66	69	50	7
206565_x_at	265	203	144	246	38	77	290	400	51	127	132	157	52	60	62	190	198	177	137	102	98
206566_at	108	67	289	63	158	203	282	174	343	481	459	132	8	38	31	17	10	31	29	32	29
206567_s_at	398	423	233	392	531	525	302	395	408	408	425	530	483	637	781	603	614	433	701	721	757
206568_at	21	50	22	96	197	132	45	38	79	15	51	124	20	27	60	40	10	28	25	25	5
206569_at	91	11	16	115	286	238	23	77	21	45	121	15	63	81	91	30	7	30	32	31	33
206570_s_at	32	18	20	67	312	124	94	74	143	190	81	28	158	66	117	19	30	11	22	64	6
206571_s_at	480	884	1073	277	979	806	1092	726	716	829	794	768	473	336	352	361	391	280	368	495	367
206572_x_at	422	270	307	166	466	446	511	595	190	207	269	393	51	28	40	46	62	189	57	34	61

206573_at	217	66	132	107	296	184	192	203	181	169	112	88	29	59	37	64	13	43	48	80	50
206574_s_at	108	253	80	211	44	50	273	235	157	190	159	111	20	8	50	61	64	82	71	91	58
206575_at	15	13	7	106	21	54	17	11	31	22	16	20	1	3	3	3	2	3	4	3	2
206576_s_at	212	64	232	548	522	422	232	146	551	723	419	245	51	55	51	44	21	88	121	132	192
206577_at	46	7	5	19	19	15	15	3	7	11	43	9	2	2	4	1	5	3	1	9	27
206578_at	33	17	60	35	32	36	30	36	35	36	57	26	31	53	29	12	8	44	28	113	37
206579_at	65	82	141	43	191	22	217	309	157	175	171	192	7	7	3	15	19	14	8	7	31
206580_s_at	220	264	176	285	599	710	223	212	233	154	348	426	172	245	228	59	65	49	79	46	43
206581_at	99	68	9	106	147	146	98	100	75	124	65	45	29	18	25	14	2	21	19	37	11
206582_s_at	351	315	1061	938	43	255	282	137	886	910	500	102	10	77	78	41	90	108	147	99	149
206583_at	101	128	235	328	151	242	244	228	111	176	217	200	488	354	421	435	468	346	265	349	470
206584_at	21	64	52	75	164	177	64	49	41	68	72	74	46	42	53	29	9	1	18	37	29
206585_at	15	52	16	40	154	137	86	106	91	59	71	69	26	22	22	50	23	15	7	31	5
206586_at	370	260	391	370	498	219	337	389	330	488	412	303	40	55	17	27	65	35	57	82	35
206587_at	307	254	225	369	503	469	227	393	273	422	391	547	218	195	324	194	237	167	182	466	523
206588_at	28	9	113	14	103	109	33	11	13	45	60	15	19	10	33	15	1	4	13	4	4
206589_at	25	36	26	16	38	31	65	107	11	20	36	30	54	11	37	62	36	41	8	28	11
206590_x_at	263	314	316	442	575	391	457	256	415	453	323	426	86	57	68	39	54	39	40	57	101
206591_at	81	38	5	9	249	52	23	13	79	13	91	53	23	3	21	5	13	35	11	3	10
206592_s_at	4163	4100	2994	1304	2280	2973	2397	2527	2693	2254	2899	2374	2128	2029	1749	2381	3223	2598	2053	2026	1924
206593_s_at	411	412	463	388	720	590	400	368	633	442	770	518	491	618	368	168	209	183	294	215	211
206594_at	73	78	189	84	153	52	219	268	169	126	193	215	71	49	77	26	65	41	121	52	52
206595_at	1097	1191	1853	748	249	434	510	632	472	428	354	274	373	208	237	958	939	940	709	752	264
206596_s_at	25	10	18	14	22	20	16	16	15	22	14	14	68	17	27	19	8	6	59	9	39
206597_at	66	7	30	47	192	98	21	57	8	10	20	13	28	11	55	3	3	4	12	5	6
206598_at	9	7	8	8	51	86	9	7	7	7	6	8	3	4	7	3	2	2	5	3	2
206599_at	466	583	581	932	81	349	408	462	661	1096	333	134	256	360	284	472	473	274	730	513	658

206600_s_at	930	1073	1082	1552	480	1162	623	837	1040	1331	527	709	504	609	541	985	928	1014	1832	1749	1786
206601_s_at	90	57	72	16	45	27	25	94	17	28	6	18	10	30	6	34	8	15	4	20	8
206602_s_at	78	21	80	35	158	39	65	62	19	48	38	36	1	1	2	1	1	3	4	23	8
206603_at	15	72	23	53	87	38	23	15	64	138	17	24	10	10	4	14	25	3	4	2	3
206604_at	101	41	113	250	26	20	154	231	175	119	117	34	16	7	8	30	30	22	25	11	50
206605_at	24	21	20	19	51	79	50	21	27	34	81	48	20	28	7	13	6	2	6	34	9
206606_at	16	12	12	14	22	19	11	9	17	14	11	11	3	5	3	5	4	4	45	82	247
206607_at	105	46	91	120	390	97	129	96	149	205	146	142	33	13	143	17	39	16	101	14	49
206608_s_at	239	122	190	162	452	445	250	226	222	215	132	210	52	55	56	50	29	53	34	44	58
206609_at	45	67	76	61	62	130	66	91	88	106	101	46	12	46	6	19	11	4	34	39	22
206610_s_at	181	107	168	147	121	289	166	176	169	131	26	119	29	55	86	43	28	78	67	24	59
206611_at	89	131	156	263	106	47	189	230	111	145	33	29	29	10	50	50	51	56	74	109	71
206612_at	9	15	12	13	158	102	126	11	13	50	31	46	4	53	27	47	14	21	4	16	4
206613_s_at	369	241	397	67	301	254	245	315	234	257	193	263	336	140	340	920	815	783	636	468	689
206614_at	258	62	132	54	122	95	58	70	63	42	50	93	15	34	5	6	7	17	4	5	25
206615_s_at	86	43	81	59	103	20	76	90	33	103	78	79	51	24	18	41	4	28	68	61	60
206616_s_at	24	46	9	9	45	66	4	13	9	7	9	14	2	26	3	28	4	19	22	4	12
206617_s_at	38	13	33	41	43	48	12	16	39	36	34	31	5	4	9	6	7	2	5	4	5
206618_at	12	30	19	4	10	11	41	29	29	10	13	8	2	9	15	31	18	33	2	12	17
206619_at	19	7	16	14	103	85	34	45	52	68	20	54	87	39	167	2	8	2	3	2	21
206620_at	54	60	39	31	71	113	50	129	13	108	136	11	62	14	12	40	46	32	38	24	32
206621_s_at	7854	7406	5076	4140	7681	5033	6740	7552	5954	5364	9492	6000	7957	6629	3786	3236	3427	2924	3733	3241	2142
206622_at	33	18	37	19	106	128	40	38	15	36	36	11	9	2	4	11	6	5	40	32	24
206623_at	135	128	83	129	364	290	129	185	142	226	102	176	45	64	26	7	7	36	27	4	9
206624_at	74	11	8	6	16	31	72	86	9	91	90	8	3	5	3	2	15	2	1	2	15
206625_at	270	180	302	294	88	349	201	241	352	280	179	57	37	45	61	29	24	30	53	56	43
206626_x_at	101	73	84	91	177	243	102	120	107	66	132	215	78	51	56	7	32	34	22	4	4

206627_s_at	1	1	26	6	69	51	16	25	24	7	3	6	4	3	2	5	5	13	14	9	1
206628_at	188	195	256	277	144	59	158	263	337	650	233	195	11	10	77	29	10	25	178	255	199
206629_at	9	9	7	8	14	15	11	9	8	6	11	6	3	8	7	3	8	7	6	2	7
206630_at	233	84	237	229	687	582	148	228	189	285	217	225	29	15	33	8	25	41	79	51	48
206631_at	17	24	34	54	93	71	16	36	19	17	37	94	15	22	101	26	48	37	33	7	5
206632_s_at	384	453	378	120	3803	880	317	335	239	386	2764	4394	4640	4164	2617	788	735	953	1303	713	847
206633_at	54	82	172	125	102	51	142	117	154	98	119	58	17	27	32	35	20	6	27	39	22
206634_at	29	102	62	41	62	199	64	74	70	78	70	45	9	17	13	39	41	52	5	36	1
206635_at	155	176	232	300	466	367	73	203	288	108	222	98	99	68	150	71	105	128	177	172	105
206636_at	107	92	189	167	250	189	195	197	215	228	243	344	135	218	98	128	77	71	90	74	99
206637_at	29	2	22	1	19	38	7	7	15	15	37	60	11	2	6	19	1	16	14	17	12
206638_at	13	22	4	4	44	17	4	4	13	7	21	5	15	18	3	1	1	6	10	5	11
206639_x_at	103	44	41	52	109	69	114	44	58	22	44	14	13	32	27	10	19	26	12	2	10
206640_x_at	172	90	75	88	30	27	48	131	116	66	55	94	12	5	11	7	4	6	2	15	5
206641_at	8	4	18	10	66	47	4	11	5	4	6	6	7	2	9	12	12	21	6	4	9
206642_at	74	11	19	34	14	11	15	46	41	38	37	74	4	2	5	12	3	6	11	3	9
206643_at	103	44	81	83	230	43	107	102	82	120	33	109	4	8	26	4	14	21	34	40	31
206644_at	106	68	68	102	201	163	101	92	64	99	67	112	4	16	22	2	8	7	15	14	14
206645_s_at	9	88	45	132	367	396	21	86	25	75	119	19	29	39	2	31	38	7	21	19	4
206646_at	99	38	296	193	84	97	139	48	226	189	70	170	5	12	41	153	62	114	118	147	134
206647_at	7	5	4	9	15	22	7	7	7	7	4	5	4	4	2	4	6	16	3	4	1
206648_at	28	6	7	10	164	185	52	74	50	70	166	145	92	83	127	13	2	18	8	44	35
206649_s_at	234	326	663	625	386	370	188	230	419	442	1158	369	260	306	289	125	110	95	192	108	130
206650_at	114	66	30	144	892	792	61	67	96	55	136	36	71	93	55	123	61	42	91	64	74
206651_s_at	98	49	50	22	49	9	102	21	41	77	61	102	13	25	42	19	20	2	15	4	23
206652_at	28	26	65	32	118	82	49	96	75	68	87	157	109	66	3	15	15	32	29	52	51
206653_at	677	476	392	79	194	164	616	541	597	274	117	97	85	170	213	761	783	1002	594	331	498

206654_s_at	122	60	107	80	367	87	331	216	234	98	27	49	38	59	54	123	107	137	65	74	86
206655_s_at	16	23	9	23	81	87	82	21	23	15	16	11	2	6	3	4	3	8	4	5	2
206656_s_at	2983	3229	2174	2510	3985	3721	3212	3562	2922	3442	4792	5019	3868	4374	3820	2968	3109	2199	2069	1645	1521
206657_s_at	49	23	26	36	30	58	38	45	48	29	27	138	7	4	9	5	3	6	8	3	7
206658_at	32	77	312	6208	195	496	36	9	686	344	74	20	40	48	41	83	54	34	366	236	166
206659_at	73	168	72	10	41	91	11	92	15	18	129	197	43	54	43	34	26	6	6	7	3
206660_at	26	57	68	19	71	395	57	57	35	102	37	30	17	38	41	24	31	13	54	6	11
206661_at	60	78	163	140	133	31	176	223	159	247	213	176	35	24	26	3	6	3	4	4	5
206662_at	1857	1292	1171	1763	3939	4286	1056	1163	574	864	3894	5944	6357	860	4470	1467	1848	2568	1606	3542	2528
206663_at	153	58	118	109	41	44	183	145	173	109	118	143	3	3	23	3	7	1	10	21	27
206664_at	3	1	5	8	74	15	5	5	3	3	18	6	17	6	2	1	3	12	9	3	1
206665_s_at	525	501	1156	718	1057	746	557	624	697	1254	2265	1178	229	177	168	132	130	150	152	57	63
206666_at	65	32	56	69	146	220	62	57	83	41	38	65	11	10	11	4	42	7	35	6	7
206667_s_at	118	63	233	392	195	265	325	395	341	419	283	243	113	80	83	140	134	170	132	128	337
206668_s_at	156	108	365	476	289	262	612	602	450	477	493	381	114	112	115	240	160	259	203	146	236
206669_at	126	270	275	224	973	528	229	158	266	71	839	397	1128	728	1197	164	128	123	240	316	314
206670_s_at	134	328	290	217	528	308	50	145	298	212	787	380	1064	971	1432	114	119	98	232	273	250
206671_at	169	94	152	54	136	87	27	146	37	198	82	101	7	6	21	8	6	24	6	19	3
206672_at	353	307	415	411	564	415	335	405	355	375	432	235	46	75	76	85	80	83	118	62	49
206673_at	108	77	163	109	85	43	163	133	75	87	172	127	20	8	16	15	15	17	22	19	4
206674_at	163	145	225	119	268	86	231	160	223	274	149	133	34	6	22	35	38	18	6	25	19
206675_s_at	54	28	209	52	370	210	52	37	90	112	126	19	90	75	56	19	21	30	86	42	50
206676_at	9	51	4	10	18	47	8	15	28	38	7	5	2	12	11	2	2	11	1	2	2
206677_at	134	107	199	87	524	258	62	115	130	92	142	141	38	71	61	9	12	21	36	44	17
206678_at	33	78	28	100	40	58	73	71	39	29	31	41	2	3	2	24	5	31	4	3	6
206679_at	52	62	45	72	242	308	36	40	75	101	75	101	5	12	33	58	23	6	6	8	39
206680_at	12	15	83	50	243	274	89	70	88	39	41	24	15	5	32	36	18	14	6	16	23

206681_x_at	204	54	213	217	150	234	245	177	114	257	141
	300	4	38	11	22	7	29	8	2	5	
206682_at	89	9	50	72	168	151	103	129	115	36	102
	107	15	12	10	15	24	20	32	6	6	
206683_at	836	173	285	66	334	259	265	301	45	57	145
	79	209	253	482	560	597	1304	311	437	589	
206684_s_at	98	152	168	132	190	163	178	166	116	219	159
	118	35	25	72	50	51	35	70	36	48	
206685_at	48	7	19	4	92	71	11	4	3	10	11
	13	34	17	11	11	3	13	15	2	3	
206686_at	33	26	95	19	179	211	77	44	309	166	91
	88	102	107	40	33	74	68	177	139	193	
206687_s_at	681	833	406	737	911	1403	682	718	360	454	588
	547	343	332	406	303	371	283	304	399	304	
206688_s_at	1752	1525	1419	1295	3652	2326	1782	1644	1817	1530	2528
	1930	2058	2867	1479	1129	1131	1196	1255	1233	1049	
206689_x_at	1053	858	882	1370	943	988	1104	938	924	872	878
	613	467	742	446	636	536	361	521	450	335	
206690_at	87	16	18	16	26	27	23	22	23	43	26
	33	12	3	4	33	12	11	13	7	33	
206691_s_at	116	111	96	273	120	51	123	87	122	105	53
	128	6	7	12	9	33	6	11	26	37	
206692_at	193	19	109	76	249	247	74	183	76	165	188
	192	4	15	33	2	4	2	23	6	19	
206693_at	66	34	19	5	16	28	46	41	52	36	44
	63	24	27	20	24	56	69	12	4	9	
206694_at	9	67	134	34	228	156	162	87	55	45	30
	131	18	12	6	9	5	3	10	37	7	
206695_x_at	136	44	100	106	307	212	99	160	116	130	115
	162	44	42	47	41	38	44	38	51	52	
206696_at	22	105	24	8	56	102	50	54	41	15	105
	215	96	97	131	42	66	20	6	7	17	
206697_s_at	32	61	46	35	225	86	28	67	66	20	31
	65	23	19	14	36	11	52	8	29	18	
206698_at	229	285	484	358	173	153	205	268	467	607	95
	134	20	15	21	225	226	272	1063	670	938	
206699_x_at	50	16	52	58	63	24	49	103	59	17	26
	67	2	25	15	38	10	12	22	26	4	
206700_s_at	167	154	37	35	65	47	236	271	153	38	153
	39	37	4	4	23	45	13	15	21	36	
206701_x_at	128	55	19	5	19	60	20	36	17	48	63
	53	2	16	16	4	14	2	5	18	18	
206702_at	17	7	68	210	65	40	15	8	43	38	9
	6	10	20	3	4	2	3	72	12	52	
206703_at	284	181	252	296	536	336	258	339	242	293	730
	544	223	225	95	90	65	99	59	57	60	
206704_at	138	96	161	171	135	171	126	205	165	229	271
	139	68	52	76	48	21	40	52	56	70	
206705_at	83	19	22	78	353	544	103	25	43	71	34
	102	3	45	18	32	9	47	22	9	35	
206706_at	56	36	61	98	410	595	53	48	40	48	424
	418	321	357	290	52	10	54	29	20	8	
206707_x_at	229	92	214	149	188	51	156	182	149	173	132
	113	8	4	28	29	25	30	23	22	39	

206708_at	148	55	88	137	194	180	217	227	197	162	146
	123	44	47	30	31	37	40	32	51	40	
206709_x_at	20	18	81	149	280	71	13	30	90	14	44
	14	103	135	123	111	143	169	138	112	89	
206710_s_at	7	2	5	5	12	4	5	7	7	21	3
	5	19	2	4	3	11	1	11	2	1	
206711_at	36	7	24	8	26	75	5	17	12	15	10
	14	2	15	7	6	2	3	5	4	4	
206712_at	60	28	15	17	93	95	21	82	71	73	9
	36	9	25	15	3	2	3	118	34	22	
206713_at	167	159	221	290	415	343	244	241	277	313	213
	215	5	20	26	22	34	6	39	16	26	
206714_at	103	145	146	234	98	138	34	140	146	36	131
	83	4	30	39	9	21	33	36	4	7	
206715_at	82	17	91	118	194	133	60	69	43	74	72
	65	4	12	15	3	5	1	21	39	3	
206716_at	70	19	95	28	80	36	30	115	92	35	98
	29	2	6	9	12	9	8	8	4	7	
206717_at	41	10	37	79	70	74	33	34	9	85	37
	10	1	1	10	3	4	32	43	11	9	
206718_at	69	99	100	248	322	314	134	129	120	61	60
	63	18	17	25	52	66	68	46	27	15	
206719_at	151	114	52	172	341	129	143	69	111	172	91
	134	41	31	20	6	38	12	54	12	27	
206720_at	93	60	90	19	125	62	12	57	33	73	125
	75	42	54	57	4	10	4	18	47	5	
206721_at	57	27	107	41	210	191	52	65	51	20	37
	55	19	12	33	1	29	20	7	7	8	
206722_s_at	598	781	602	387	120	105	563	363	337	447	549
	308	223	229	159	175	196	230	165	144	127	
206723_s_at	554	518	434	362	542	607	564	454	447	461	689
	439	430	364	276	328	363	519	266	530	359	
206724_at	445	519	484	428	397	538	584	435	310	416	510
	259	48	33	7	33	33	56	20	58	78	
206725_x_at	71	74	27	27	38	69	30	55	19	45	18
	26	9	17	22	16	79	17	20	14	15	
206726_at	160	38	4	50	40	113	49	162	37	96	111
	72	7	4	29	16	2	4	6	36	18	
206727_at	11	18	8	48	26	28	27	54	43	17	17
	8	4	1	1	1	15	1	4	2	6	
206728_at	257	117	144	160	87	110	76	84	124	115	81
	102	5	54	67	5	96	15	5	7	3	
206729_at	266	162	136	274	537	474	146	179	306	240	151
	183	44	61	35	93	38	55	48	36	54	
206730_at	26	10	11	32	33	22	25	3	13	14	3
	8	1	2	23	2	2	2	17	1	4	
206731_at	81	6	54	89	349	48	82	78	68	66	34
	62	1	13	42	35	4	17	38	17	17	
206732_at	21	56	115	84	25	47	106	106	83	77	70
	93	5	17	6	6	19	3	4	2	11	
206733_at	98	136	163	93	40	22	162	211	166	117	168
	158	11	17	18	23	7	31	39	38	13	
206734_at	239	167	441	411	111	305	154	297	393	559	273
	248	344	186	242	181	161	137	878	671	670	

206735_at	22	21	79	97	32	31	45	129	59	36	94	109	45	4	5	16	23	6	8	4	7
206736_x_at	130	49	30	140	27	30	121	120	95	140	7	168	4	2	10	3	3	5	5	2	1
206737_at	12	12	53	662	18	603	12	9	66	28	16	11	14	43	80	3	6	4	269	108	97
206738_at	249	277	187	217	438	249	228	234	186	266	165	290	38	88	40	12	67	14	68	52	60
206739_at	82	41	217	212	298	67	164	63	395	249	107	65	110	55	121	30	19	18	169	154	110
206740_x_at	163	88	34	62	246	129	106	158	120	131	108	102	37	22	9	31	33	26	45	35	26
206741_at	71	32	47	53	58	47	54	42	48	56	45	44	7	13	6	4	8	28	12	4	4
206742_at	36	45	16	102	195	85	106	135	28	45	124	40	5	4	1	3	7	7	27	28	11
206743_s_at	38	51	57	22	26	16	107	16	47	17	63	19	31	29	48	17	33	3	47	46	35
206744_s_at	108	15	132	72	159	175	37	96	28	70	92	109	119	117	105	38	34	48	69	61	65
206745_at	287	176	201	239	440	413	233	244	310	261	360	265	137	95	165	58	48	83	79	107	76
206746_at	167	140	47	12	36	191	183	139	15	4	55	57	80	64	66	122	178	111	39	91	35
206747_at	314	208	312	230	598	258	58	128	218	240	178	280	65	35	80	55	46	73	159	132	97
206748_s_at	441	257	667	279	998	939	579	673	755	897	854	613	108	115	100	114	70	168	107	81	100
206749_at	142	34	35	26	19	23	24	36	91	116	91	20	12	17	4	5	2	35	3	7	3
206750_at	52	61	91	110	326	343	195	102	178	217	172	34	20	17	41	50	78	23	55	47	23
206751_s_at	74	46	80	28	62	31	38	53	98	84	53	79	1	19	5	6	3	3	1	1	3
206752_s_at	503	246	149	125	282	142	454	256	281	159	171	114	182	87	148	189	229	205	427	264	302
206753_at	53	17	31	52	110	175	13	24	54	27	18	16	10	7	5	6	9	11	8	21	9
206754_s_at	122	97	1500	552	76	102	76	143	1254	1782	88	93	24	7	65	37	3	2	2013	5307	5984
206755_at	22	13	772	432	121	231	32	86	566	806	37	34	10	20	11	8	2	15	1213	3228	4333
206756_at	239	23	18	34	36	50	148	120	21	14	101	138	178	155	137	272	361	204	43	105	64
206757_at	61	38	5	16	19	23	13	50	20	77	48	58	2	3	13	29	2	12	4	2	1
206758_at	29	27	42	39	298	344	37	29	24	39	125	31	105	8	24	8	68	15	29	4	7
206759_at	20	11	15	8	37	17	11	11	12	17	14	20	7	4	25	16	6	36	6	6	8
206760_s_at	8	5	8	10	29	27	7	7	7	7	7	9	5	7	9	6	2	8	3	5	4
206761_at	78	11	43	153	147	85	131	25	92	17	47	84	19	1	5	33	10	49	57	36	77

206762_at	13	21	9	62	15	32	12	15	12	8	10	15	18	4	3	3	2	5	1	2	1
206763_at	168	136	225	366	386	173	158	148	207	127	135	176	36	44	44	48	73	18	40	56	41
206764_x_at	210	496	270	621	176	556	91	356	258	147	50	153	17	26	110	91	135	74	203	284	267
206765_at	95	61	134	146	67	208	95	143	222	179	101	52	5	13	19	13	25	3	48	65	70
206766_at	430	259	255	322	403	484	295	329	312	256	233	256	63	51	67	36	60	46	88	79	32
206767_at	196	82	290	243	236	192	227	251	215	229	175	152	30	41	11	45	36	49	31	45	33
206768_at	91	13	35	27	37	65	17	20	21	87	20	35	1	11	3	16	20	27	9	9	3
206769_at	164	221	110	169	854	1091	98	41	140	41	33	141	29	13	53	70	48	68	76	73	15
206770_s_at	873	770	1230	1380	726	750	445	446	748	790	317	538	576	490	561	815	748	788	2814	2367	3388
206771_at	12	11	9	23	30	40	9	21	11	18	21	10	4	9	5	9	9	7	4	4	9
206772_at	112	12	133	40	132	130	105	150	59	140	30	54	14	11	16	12	39	25	27	29	32
206773_at	62	96	75	100	169	191	85	129	139	64	60	85	30	10	56	29	35	36	15	25	23
206774_at	16	61	38	125	80	56	15	108	39	42	33	77	37	14	54	27	38	38	21	37	34
206775_at	44	95	128	40	190	151	60	44	52	88	50	102	8	3	7	8	24	8	5	37	44
206776_x_at	261	151	254	256	495	218	231	170	298	281	189	236	27	25	45	55	81	73	77	78	56
206777_s_at	222	57	350	392	581	840	335	243	484	305	802	1003	853	427	447	125	100	149	315	282	170
206778_at	42	24	18	23	170	87	32	49	48	43	90	99	14	8	7	7	5	4	8	4	17
206779_s_at	11	60	46	8	56	130	9	11	60	11	10	10	13	40	26	3	24	50	18	10	23
206780_at	86	30	28	188	385	309	109	231	254	92	63	109	29	11	14	23	10	47	11	19	9
206781_at	288	232	423	319	257	148	227	226	67	136	415	156	45	106	57	38	114	83	20	104	17
206782_s_at	243	584	216	647	294	380	266	248	213	271	216	255	169	154	90	119	137	150	39	157	150
206783_at	261	213	218	405	580	462	303	434	194	189	294	192	23	24	75	30	41	11	51	30	13
206784_at	101	23	224	54	80	47	46	44	63	157	47	75	8	5	2	4	13	6	10	5	23
206785_s_at	249	109	9	133	132	27	111	90	9	11	10	8	28	55	54	918	939	689	55	453	388
206786_at	8	6	4	10	11	8	12	4	4	4	7	5	1	2	1	1	1	4	15	11	3
206787_at	15	6	33	14	77	149	40	78	75	49	21	5	1	11	3	13	3	2	10	14	2
206788_s_at	1158	884	1013	473	1541	523	906	1045	1715	2021	1484	1445	300	108	133	205	85	240	188	201	265

206789_s_at	568	476	366	449	224	70	347	578	514	514	391	338	178	324	269	240	219	230	468	317	450
206790_s_at	2465	3913	3264	4609	4434	4070	3809	4281	3578	4221	6789	8246	11451	9731	7654	3426	2954	3069	4513	3366	2065
206791_s_at	361	336	368	369	419	293	533	363	478	437	391	415	77	65	97	68	37	73	130	106	66
206792_x_at	1500	1988	1375	2408	2605	2113	1211	2089	2464	1610	1245	3325	498	506	576	294	248	876	818	959	772
206793_at	177	146	199	138	205	152	186	94	171	152	114	160	60	44	14	13	33	35	59	51	20
206794_at	70	51	34	54	89	206	32	29	52	81	63	40	17	14	46	5	37	15	11	26	11
206795_at	94	68	45	10	38	23	91	80	68	56	16	4	3	6	3	26	20	21	4	5	4
206796_at	49	69	18	22	23	62	32	59	28	102	10	53	12	2	11	1	6	21	20	2	4
206797_at	42	24	65	47	36	30	95	106	44	38	30	36	5	6	3	10	8	4	8	7	5
206798_x_at	196	113	136	350	67	51	38	44	157	131	75	24	7	12	33	64	34	41	87	46	42
206799_at	12	6	12	16	15	9	15	9	15	15	10	9	1	14	2	2	2	2	18	2	2
206800_at	58	33	52	56	78	112	41	33	36	61	80	28	32	9	30	10	10	51	36	7	9
206801_at	128	86	72	67	324	194	73	17	72	24	37	53	3	46	2	30	3	6	11	4	5
206802_at	34	18	61	43	89	54	58	41	36	70	70	89	3	12	27	3	7	3	9	8	5
206803_at	36	22	75	28	18	23	66	84	23	27	53	24	2	2	2	4	5	45	2	2	3
206804_at	30	19	28	32	115	286	29	29	28	31	24	21	10	9	6	6	7	6	6	6	3
206805_at	123	136	525	285	197	304	350	294	359	189	92	123	68	67	74	299	227	311	191	289	342
206806_at	16	24	69	14	73	63	80	62	5	71	74	57	2	3	1	22	4	1	14	33	12
206807_s_at	324	247	282	340	366	254	253	434	226	282	296	332	55	87	144	87	133	79	74	53	76
206808_at	12	12	57	6	158	152	113	127	88	144	27	18	62	61	31	34	17	55	37	13	43
206809_s_at	1203	886	1240	718	1031	306	2669	2408	1754	2053	1316	1857	883	362	244	460	350	368	813	396	579
206810_at	16	7	31	6	109	409	8	32	35	3	21	40	7	6	19	2	19	3	2	23	1
206811_at	90	9	12	66	16	28	37	45	9	53	10	44	2	8	10	3	5	9	16	6	1
206812_at	98	62	18	22	85	24	40	65	72	18	45	34	4	4	9	6	5	28	4	7	18
206813_at	126	213	41	44	103	40	213	165	24	32	31	14	5	95	6	160	131	130	42	36	7
206814_at	52	10	11	48	48	39	40	59	20	82	94	109	10	9	14	5	11	21	5	21	13
206815_at	11	7	16	32	341	406	8	15	12	11	14	11	3	5	4	3	2	1	4	2	2

206816_s_at	110	91	103	102	489	431	101	33	87	134	78	64	29	51	44	10	43	49	34	87	33
206817_x_at	94	54	60	56	74	90	57	94	63	57	45	55	9	18	128	26	100	70	51	20	12
206818_s_at	579	583	410	532	441	349	627	682	400	521	557	460	96	137	174	282	303	258	159	240	240
206819_at	83	21	38	118	278	81	50	104	39	28	31	18	26	2	3	23	24	15	23	41	15
206820_at	12	15	11	28	37	105	13	12	13	14	18	14	5	8	5	2	7	7	6	5	3
206821_x_at	91	40	58	156	151	243	134	190	131	56	281	99	46	32	36	61	16	18	20	21	23
206822_s_at	127	203	133	200	32	56	129	202	138	157	98	224	34	39	73	15	41	23	62	64	53
206823_at	64	30	47	52	56	52	212	42	43	45	37	24	4	11	15	5	54	71	6	6	29
206824_at	15	6	34	13	41	36	8	20	7	53	20	20	5	6	14	5	39	12	4	7	2
206825_at	216	174	155	193	570	598	166	177	126	157	277	258	53	95	58	68	62	67	87	53	80
206826_at	66	28	62	84	357	185	56	78	64	35	78	68	13	9	28	5	28	4	15	2	7
206827_s_at	25	108	28	175	419	753	30	18	51	27	20	23	40	105	67	100	70	30	67	152	169
206828_at	71	17	152	219	128	15	103	120	72	73	111	13	15	14	21	90	96	69	104	206	280
206829_x_at	406	173	536	350	334	103	364	422	280	268	449	346	272	242	275	409	317	351	233	204	234
206830_at	32	21	33	41	47	90	56	65	19	25	54	41	8	17	6	9	1	5	8	3	15
206831_s_at	12	30	12	85	313	241	13	46	13	21	60	80	26	45	21	49	8	31	43	52	36
206832_s_at	65	105	85	226	99	19	109	22	84	158	23	24	4	4	3	5	35	22	22	38	7
206833_s_at	534	505	350	473	542	856	392	578	213	324	360	377	326	359	364	582	534	566	352	624	613
206834_at	234	49	24	159	247	321	57	50	36	36	151	73	7	3	2	40	8	9	15	16	7
206835_at	36	11	87	61	340	184	87	75	72	77	55	13	2	36	30	17	16	22	21	6	16
206836_at	288	271	206	283	45	157	131	166	123	295	195	292	74	104	66	151	90	109	63	56	68
206837_at	29	21	38	23	33	24	41	38	41	27	31	35	4	5	24	18	17	20	6	5	7
206838_at	75	227	58	66	22	48	27	54	16	48	47	113	74	54	7	118	128	130	40	99	90
206839_at	65	35	71	25	570	527	8	116	106	122	105	90	20	8	14	30	66	54	53	32	19
206840_at	135	72	163	66	109	60	175	141	112	161	58	94	5	29	37	24	20	4	25	45	13
206841_at	15	10	77	12	62	16	13	20	24	22	21	31	3	3	7	6	2	10	3	21	3
206842_at	83	19	16	18	209	44	16	20	12	17	28	21	5	5	6	22	7	7	4	21	9

206843_at	16	13	7	9	56	19	8	17	7	10	10	16	2	8	5	6	5	7	8	2	1
206844_at	12	9	16	9	18	24	12	9	9	8	7	9	2	7	6	2	3	2	2	7	2
206845_s_at	1036	663	1177	953	455	363	1043	909	1283	1192	1008	383	277	452	332	375	541	638	468	371	295
206846_s_at	946	1422	973	1500	1021	1154	1101	1259	688	1157	1053	1024	294	491	281	284	248	296	278	228	191
206847_s_at	156	227	157	58	261	394	245	238	90	175	286	303	624	517	292	302	309	239	130	209	128
206848_at	389	333	123	295	210	230	301	458	334	289	310	328	105	99	23	64	60	77	98	78	46
206849_at	151	94	121	111	201	47	186	284	25	161	92	162	5	27	25	54	41	59	20	7	9
206850_at	213	38	61	106	36	66	183	201	235	263	142	102	45	38	54	26	9	44	13	47	11
206851_at	28	33	37	40	205	308	40	40	35	56	33	54	4	8	15	8	20	6	10	8	9
206852_at	5	2	34	39	26	9	5	9	5	22	18	36	2	7	18	3	3	16	13	7	1
206853_s_at	566	692	937	714	683	568	647	868	744	892	618	613	411	379	352	445	326	444	880	554	626
206854_s_at	620	584	810	720	797	736	704	755	752	769	567	646	542	407	388	797	647	645	901	490	709
206855_s_at	1001	1254	865	1080	863	852	735	975	702	622	1438	631	1172	1104	515	507	508	438	416	188	193
206856_at	82	102	23	109	22	22	91	61	74	71	81	186	20	22	3	3	3	2	1	3	2
206857_s_at	700	1072	7	36	636	622	725	707	21	24	530	466	356	327	435	560	651	490	13	37	19
206858_s_at	671	871	956	1681	1431	1016	466	743	760	516	1059	1120	2298	1164	1228	836	545	893	679	2682	2150
206859_s_at	21	18	19	38	33	36	16	21	15	20	20	16	23	10	9	5	3	7	2	6	2
206860_s_at	548	712	947	532	1117	787	628	624	804	655	1019	1048	600	648	1084	561	495	464	871	605	900
206861_s_at	946	800	781	720	1527	1762	1371	1572	739	864	2000	1687	715	986	785	515	483	493	300	333	233
206862_at	15	27	18	41	150	24	9	69	11	27	54	72	40	63	25	34	48	28	36	32	46
206863_x_at	57	56	43	23	21	122	24	22	5	7	9	11	34	8	20	6	5	6	8	17	9
206864_s_at	75	108	26	17	386	59	58	58	13	67	13	11	52	45	30	108	136	124	3	44	19
206865_at	26	23	16	12	32	121	24	16	28	15	26	10	13	24	7	4	29	3	5	2	3
206866_at	9	6	7	27	12	24	15	9	7	7	10	10	7	41	16	11	7	12	2	11	1
206867_at	107	123	278	191	401	54	209	164	149	246	151	128	5	9	46	9	39	58	62	66	58
206868_at	155	174	324	176	368	91	154	231	286	327	209	162	62	70	70	90	140	143	117	41	49
206869_at	15	11	23	10	15	17	13	17	9	7	10	14	2	8	7	6	5	5	3	3	3

206870_at	90	116	161	266	66	274	139	127	149	254	189
	117	25	28	33	33	57	48	63	42	27	
206871_at	16	7	24	14	23	71	30	21	11	18	14
	19	3	22	6	3	5	2	7	5	4	
206872_at	202	101	126	129	287	199	160	140	182	151	165
	161	34	4	43	6	8	18	15	49	19	
206873_at	28	9	109	59	44	60	58	232	43	113	136
	137	29	12	5	12	29	34	13	59	22	
206874_s_at	328	184	817	894	906	996	370	542	974	1020	506
	695	490	819	671	411	413	280	1374	1590	1348	
206875_s_at	827	502	1042	680	683	1020	718	1375	863	1024	630
	539	809	760	798	947	569	1172	1824	1411	1921	
206876_at	5	23	4	98	18	51	68	36	41	8	50
	24	4	6	3	3	5	2	4	10	2	
206877_at	21	12	14	21	16	23	23	16	20	48	24
	30	3	6	6	2	4	8	8	11	25	
206878_at	385	147	388	297	546	390	269	278	282	299	291
	209	46	69	123	97	45	113	83	72	68	
206879_s_at	45	40	35	17	313	239	86	84	31	42	134
	176	54	77	57	22	17	9	32	61	14	
206880_at	234	302	134	362	375	290	360	236	215	331	273
	450	62	47	86	63	52	69	12	67	6	
206881_s_at	50	18	18	62	49	32	106	13	23	20	14
	49	3	6	2	5	2	5	4	4	2	
206882_at	17	15	19	41	54	101	24	26	23	22	60
	20	6	5	5	5	10	8	6	7	5	
206883_x_at	138	94	122	180	27	54	77	107	122	282	155
	204	63	30	13	13	16	7	28	46	37	
206884_s_at	5	51	1153	85	47	16	11	17	158	43	3
	11	2	2	13	1	11	1	319	1120	1515	
206885_x_at	29	28	23	28	48	52	30	34	21	27	37
	29	7	6	5	6	28	6	5	4	3	
206886_x_at	11	16	26	35	34	117	64	16	21	29	14
	8	37	7	3	6	4	6	1	17	1	
206887_at	153	29	113	166	95	50	140	157	186	120	173
	160	34	20	31	22	40	35	34	28	31	
206888_s_at	143	102	146	345	220	199	95	57	150	112	141
	150	42	47	78	48	20	59	56	53	11	
206889_at	40	22	50	18	37	34	42	20	23	21	16
	26	13	5	6	5	15	8	6	10	3	
206890_at	143	108	100	58	287	395	130	28	206	94	117
	62	34	27	3	19	15	44	62	29	33	
206891_at	172	99	88	97	197	285	134	210	132	131	161
	173	11	7	37	15	32	12	35	17	29	
206892_at	25	39	33	19	66	55	19	24	31	48	23
	26	3	6	4	5	6	3	3	3	3	
206893_at	48	24	37	50	45	4	28	18	16	10	14
	15	7	2	16	11	20	1	13	20	14	
206894_at	30	57	42	49	429	488	85	50	20	10	30
	13	10	10	8	35	18	57	17	32	10	
206895_at	8	7	16	6	85	17	37	30	8	11	14
	38	1	23	34	1	5	7	3	3	9	
206896_s_at	161	287	31	69	359	695	233	260	123	41	78
	133	63	51	133	222	316	223	66	50	35	

206897_at	26	13	11	17	242	46	7	63	32	67	102	30	42	27	9	38	25	26	34	34	3
206898_at	7	18	22	23	99	94	21	44	33	10	36	23	14	14	16	9	20	16	15	21	19
206899_at	8	6	9	8	15	13	7	13	12	10	7	9	1	2	2	3	2	2	1	2	1
206900_x_at	210	69	168	217	225	216	146	170	144	154	247	133	81	65	103	27	16	28	27	26	21
206901_at	38	36	47	62	63	55	159	131	58	46	166	165	20	25	76	41	41	15	66	15	11
206902_s_at	62	85	35	9	118	55	103	45	66	22	53	15	72	42	36	54	38	48	17	46	52
206903_at	26	24	31	131	60	82	24	26	87	55	70	15	5	17	44	9	9	3	13	3	8
206904_at	142	96	85	49	269	215	99	69	116	120	105	112	13	59	14	52	66	20	43	67	5
206905_s_at	146	60	53	47	92	163	86	53	27	82	80	21	29	43	67	37	10	12	29	15	7
206906_at	221	215	27	66	377	443	231	149	63	64	144	88	42	52	9	77	14	47	25	2	13
206907_at	673	740	189	194	290	366	856	690	197	211	368	358	144	522	174	901	935	991	146	148	74
206908_s_at	288	217	176	272	208	124	285	327	282	193	252	197	106	82	133	13	18	48	29	45	19
206909_at	15	16	45	40	87	22	17	62	45	18	13	26	2	21	13	3	6	10	13	3	5
206910_x_at	163	141	64	260	199	230	164	106	151	144	118	123	28	49	36	26	35	29	74	41	51
206911_at	431	523	567	208	639	550	497	618	450	436	419	289	75	63	115	161	136	126	104	81	65
206912_at	15	12	5	93	59	20	29	42	9	48	10	26	4	3	2	6	1	7	22	11	2
206913_at	32	12	35	58	82	62	16	37	37	14	16	11	28	7	7	16	12	60	40	105	92
206914_at	15	1	43	8	132	22	7	21	70	8	10	31	6	6	10	8	2	4	2	1	1
206915_at	54	4	7	21	4	11	15	4	50	14	7	10	1	4	2	5	2	3	2	1	17
206916_x_at	67	61	94	18	274	352	164	95	107	61	74	58	35	62	70	107	69	65	62	55	62
206917_at	441	253	258	224	399	314	456	533	294	407	550	351	45	58	63	86	81	73	52	28	53
206918_s_at	2531	2713	2001	2424	1949	2977	2377	2827	2803	3270	2798	2331	2742	2640	2015	2082	2028	1778	1752	2345	2793
206919_at	112	61	111	71	55	39	83	90	140	55	92	57	24	18	25	86	96	88	175	73	99
206920_s_at	816	161	773	724	986	888	843	784	919	847	1091	655	275	193	172	176	189	255	183	126	185
206921_at	45	60	92	25	55	97	78	102	83	98	54	82	74	49	66	50	60	54	71	46	43
206922_at	94	108	193	206	180	347	130	162	17	53	81	168	8	61	49	31	17	28	18	44	51
206923_at	8	15	46	25	36	145	32	15	80	115	85	15	8	9	6	5	2	17	16	6	5

206924_at	103	73	110	39	67	17	98	82	13	101	74	129	57	7	40	40	41	9	15	35	26
206925_at	83	15	31	56	121	94	52	92	103	56	41	18	13	11	5	8	3	11	15	35	17
206926_s_at	40	169	183	163	92	122	246	276	118	112	60	278	7	14	40	17	17	14	6	14	13
206927_s_at	71	44	69	84	26	12	48	30	76	41	71	38	2	4	39	10	25	7	6	4	11
206928_at	156	68	159	162	125	222	115	96	116	122	74	116	111	37	58	207	163	200	183	179	234
206929_s_at	377	633	176	138	504	324	484	434	310	201	279	200	284	279	288	484	425	389	316	259	171
206930_at	107	136	134	70	172	284	115	146	124	148	108	150	24	24	34	38	43	22	18	38	21
206931_at	5	4	35	6	43	89	24	5	7	6	61	16	26	33	24	10	2	15	12	17	13
206932_at	83	41	41	102	36	165	94	172	107	137	144	85	18	23	30	34	15	19	23	19	41
206933_s_at	128	38	81	47	319	316	290	206	170	212	118	214	10	15	14	8	7	11	6	11	2
206934_at	9	69	128	44	161	83	110	38	107	46	47	14	5	22	3	33	41	23	52	41	39
206935_at	30	21	3	8	43	73	3	24	4	43	6	31	6	1	5	3	4	11	1	1	6
206936_x_at	396	422	450	634	405	462	435	529	478	347	310	576	514	469	423	365	436	242	765	684	734
206937_at	102	69	106	45	106	145	97	74	122	94	64	78	29	20	9	38	42	21	29	43	26
206938_at	102	27	85	56	335	231	122	165	75	108	115	151	34	11	28	6	31	27	40	6	3
206939_at	93	41	85	182	107	70	109	112	209	158	105	25	6	19	19	6	3	5	4	42	3
206940_s_at	167	78	117	147	206	235	106	127	185	117	239	171	50	43	46	9	37	17	39	31	63
206941_x_at	54	5	54	10	18	66	11	40	5	21	11	10	20	5	13	10	7	17	39	51	51
206942_s_at	24	12	23	6	170	34	21	28	151	80	31	15	30	6	80	65	35	82	165	57	84
206943_at	224	72	152	182	425	277	252	281	230	285	358	378	53	35	44	68	28	38	65	42	44
206944_at	270	161	244	307	521	151	269	65	276	207	176	156	24	20	43	99	92	103	62	89	15
206945_at	280	186	224	229	449	363	321	314	292	252	273	214	1	47	76	59	45	46	89	39	29
206946_at	38	17	30	44	103	51	30	48	29	32	31	25	5	13	4	30	17	6	9	4	5
206947_at	159	89	87	153	111	12	102	121	162	143	144	104	44	24	2	33	4	24	42	34	38
206948_at	402	195	471	242	282	284	318	348	447	335	281	181	6	14	36	51	52	31	101	24	46
206949_s_at	1781	2013	1853	1936	1446	1209	2103	2077	2596	2103	1477	1064	702	614	1061	1811	2016	1397	2376	1700	1560
206950_at	12	49	15	10	63	101	13	26	37	14	1	9	2	3	3	21	41	3	4	4	1

206951_at	131	73	80	113	213	60	69	113	175	140	175	243	98	29	73	19	76	68	48	63	32
206952_at	4	5	3	8	4	5	3	8	4	3	6	43	3	2	1	1	1	1	1	6	3
206953_s_at	60	39	84	25	23	20	76	11	54	20	7	55	11	3	10	11	1	6	32	4	19
206954_at	37	33	160	93	87	125	111	117	71	131	121	157	11	56	8	6	22	4	31	22	3
206955_at	45	105	43	166	693	707	48	58	37	39	48	25	11	16	88	51	16	30	41	16	13
206956_at	198	173	113	142	201	263	107	94	127	127	151	119	132	59	57	71	47	31	90	184	125
206957_at	16	9	9	71	15	28	53	40	84	109	53	103	2	6	16	4	6	39	22	7	18
206958_s_at	902	754	865	909	929	1219	753	773	941	1215	1147	953	1062	838	677	610	586	500	1452	1300	1721
206959_s_at	245	180	387	314	386	371	457	296	297	439	365	270	177	113	115	88	84	75	170	168	187
206960_at	7	5	9	3	51	192	7	34	9	14	24	33	13	1	13	1	11	14	4	9	1
206961_s_at	445	186	315	410	437	316	444	548	379	296	496	371	63	36	69	78	90	108	89	28	74
206962_x_at	28	12	46	28	286	259	21	30	151	66	27	26	18	2	7	9	26	20	4	56	35
206963_s_at	77	57	22	34	26	34	32	87	87	119	51	60	35	6	7	58	4	12	43	14	40
206964_at	101	30	95	58	43	95	73	73	45	43	14	45	5	30	36	17	4	4	30	24	13
206965_at	20	23	4	35	80	108	19	40	25	67	54	58	10	3	11	9	1	2	14	16	6
206966_s_at	318	163	294	212	170	218	221	277	365	400	301	323	63	65	86	41	13	35	33	79	72
206967_at	226	336	216	270	113	129	269	143	250	259	254	94	166	147	141	132	225	155	131	144	105
206968_s_at	590	417	747	657	679	892	602	658	788	848	1047	626	157	222	253	124	121	185	205	124	139
206969_at	15	38	14	14	18	36	13	17	15	17	13	35	20	7	15	3	6	19	1	2	3
206970_at	12	18	41	23	51	47	27	26	19	27	17	15	3	11	76	30	7	9	6	12	9
206971_at	140	230	85	56	71	151	156	150	104	144	266	156	73	75	72	63	52	70	67	52	31
206972_s_at	189	307	11	22	38	63	186	260	12	10	294	191	111	23	7	46	51	21	13	7	19
206973_at	16	17	110	21	132	55	34	22	15	22	27	85	5	12	6	5	20	4	5	4	3
206974_at	69	86	88	72	324	370	123	91	108	73	85	87	35	40	14	10	11	30	43	31	25
206975_at	13	10	11	9	37	40	15	13	13	14	13	10	2	8	5	6	6	3	6	7	3
206976_s_at	3809	2510	3628	1739	8633	6796	2455	2243	4691	3184	6805	4387	7965	8733	13225	6474	5472	6181	8559	6188	8107
206977_at	30	17	15	12	10	54	15	24	5	6	3	30	4	14	3	2	1	2	2	21	10

206978_at	122	75	161	133	301	90	135	264	195	172	148	133	44	15	38	48	22	9	16	25	17
206979_at	176	36	163	156	195	214	236	182	128	198	165	220	25	19	11	12	12	8	28	12	13
206980_s_at	19	17	24	30	23	19	27	18	19	25	38	25	15	15	11	12	9	10	4	11	5
206981_at	21	22	27	21	60	70	9	22	43	38	23	16	8	7	6	4	8	2	20	16	11
206982_at	21	17	3	56	80	11	23	18	19	38	21	14	12	3	6	2	1	13	4	4	2
206983_at	56	39	34	56	26	9	16	17	47	31	13	11	20	26	15	16	6	11	22	26	25
206984_s_at	8	5	12	13	16	137	4	13	4	8	14	16	10	20	5	19	16	7	22	17	1
206985_at	99	16	35	74	164	292	191	120	119	196	188	85	5	9	67	12	59	20	18	64	7
206986_at	73	40	18	66	455	129	54	70	29	96	75	26	19	27	12	4	21	50	57	55	7
206987_x_at	44	94	15	32	186	199	94	96	19	48	182	132	417	403	372	146	76	39	88	207	161
206988_at	102	39	37	52	56	24	45	37	127	91	33	31	14	30	9	16	5	5	24	15	7
206989_s_at	2056	892	1225	1190	1353	1512	1493	1221	979	1140	1502	713	1258	1206	1099	1358	845	1177	2104	2071	3090
206990_at	149	118	165	163	353	341	192	165	80	212	144	162	32	7	37	41	27	28	15	40	22
206991_s_at	107	35	39	67	56	71	65	70	170	68	63	49	10	6	4	3	3	3	6	7	5
206992_s_at	251	446	735	469	489	809	249	340	532	647	439	529	517	378	255	140	145	114	339	319	196
206993_at	221	145	239	189	441	370	178	182	347	338	264	297	171	167	131	39	56	58	229	134	113
206994_at	81	174	91	36	805	2830	196	239	98	193	685	491	436	435	803	22	35	22	55	97	49
206995_x_at	91	47	38	97	110	86	34	57	12	38	50	49	25	21	34	30	45	34	32	69	50
206996_x_at	148	57	279	171	117	179	133	143	119	152	171	121	9	14	9	8	2	31	27	21	37
206997_s_at	194	208	125	210	34	42	356	358	122	256	213	155	4	4	11	34	8	36	4	42	18
206998_x_at	24	28	24	40	686	407	32	36	47	28	24	40	37	46	73	63	20	16	48	41	9
206999_at	53	4	47	9	150	89	48	11	9	34	47	20	19	27	17	20	10	40	5	17	16
207000_s_at	194	180	37	6	115	258	183	107	12	10	169	138	165	150	183	239	248	250	100	177	176
207001_x_at	110	148	31	124	150	235	110	46	134	98	72	25	58	41	26	56	39	99	49	97	106
207002_s_at	796	403	34	19	595	614	307	389	8	21	232	406	528	425	941	613	675	594	29	42	76
207003_at	188	130	160	43	85	63	235	140	225	193	195	145	35	33	39	23	25	13	41	7	5
207004_at	148	105	145	63	307	296	113	77	21	210	57	137	58	135	56	71	58	169	83	71	75

207005_s_at	58	80	18	100	32	26	27	22	75	75	57	96	4	32	13	44	28	8	43	19	26
207006_s_at	5	6	62	317	19	78	5	8	50	10	17	11	44	18	31	6	35	12	154	136	66
207007_at	181	92	123	171	192	259	144	96	189	144	97	97	35	9	27	16	2	2	39	47	5
207008_at	8	9	11	40	140	52	24	22	32	13	11	53	8	11	30	20	11	17	20	9	22
207009_at	176	111	35	150	51	34	171	154	68	59	78	60	1	8	16	16	2	32	36	33	25
207010_at	48	21	98	101	27	90	57	73	62	127	51	111	4	9	5	2	5	39	4	4	13
207011_s_at	663	693	334	704	462	536	925	1193	567	527	635	566	332	315	259	488	413	351	288	259	208
207012_at	97	39	12	83	85	125	93	94	35	82	98	60	14	9	19	3	15	16	27	2	31
207013_s_at	254	120	179	180	598	770	187	248	47	180	257	117	47	44	39	64	57	51	62	59	47
207014_at	74	34	52	89	128	73	27	30	48	42	37	39	1	7	22	30	12	25	15	41	33
207015_s_at	38	73	22	75	84	67	109	65	7	61	78	20	4	10	7	21	33	23	36	9	17
207016_s_at	70	86	98	168	223	32	143	124	32	102	128	41	4	3	36	9	29	51	39	2	3
207017_at	8	5	58	54	216	185	19	9	59	60	34	30	5	3	11	2	19	4	13	39	57
207018_s_at	70	45	119	119	92	125	41	77	135	91	60	9	30	24	26	26	27	21	57	97	80
207019_s_at	37	43	22	9	296	511	65	24	31	17	16	19	6	7	54	15	8	40	11	9	4
207020_at	278	234	220	213	403	344	262	193	136	201	308	273	83	66	84	45	8	7	60	72	71
207021_at	118	47	20	87	230	120	57	148	79	126	75	126	16	4	6	5	17	37	44	27	29
207022_s_at	107	10	98	78	216	39	175	185	99	103	199	132	73	117	87	80	95	114	125	12	64
207023_x_at	2862	3285	1871	2011	1894	2326	2883	2218	1992	1495	2645	2424	2388	1264	966	1127	1216	1035	915	880	462
207024_at	29	17	33	39	43	59	33	32	19	42	27	18	7	15	13	3	3	18	11	11	17
207025_at	52	35	241	62	256	210	42	49	472	261	38	36	31	11	17	13	30	15	121	82	15
207026_s_at	108	54	19	35	40	77	27	28	33	10	21	14	9	17	5	13	3	24	32	27	8
207027_at	19	28	18	27	32	69	20	21	17	20	18	21	6	3	3	12	5	2	4	14	7
207028_at	56	23	31	25	49	20	30	25	31	21	24	34	5	8	9	5	5	4	8	16	3
207029_at	1384	382	705	188	109	113	2112	3166	1425	851	71	80	13	6	42	289	283	496	172	42	130
207030_s_at	1003	935	16	65	948	550	1283	1417	67	10	310	1816	2168	1546	2217	2248	1962	2042	167	159	126
207031_at	48	142	42	34	242	195	44	53	19	48	128	109	42	43	50	74	123	82	8	22	21

207032_s_at	22	44	68	23	66	91	82	90	36	74	26	34	25	3	3	2	25	6	4	24	3
207033_at	17	17	18	98	187	40	127	115	127	24	17	16	22	6	7	27	2	12	6	2	13
207034_s_at	165	167	271	194	175	169	170	218	258	225	340	204	78	91	84	69	47	55	104	26	50
207035_at	77	50	26	25	88	52	41	46	36	28	26	29	63	38	30	233	127	126	11	7	12
207036_x_at	101	33	11	52	36	106	95	77	66	99	18	9	2	3	3	9	4	2	10	2	6
207037_at	90	66	165	250	201	195	86	70	474	465	10	8	11	9	5	36	6	4	289	99	157
207038_at	151	146	12	28	492	176	229	278	21	15	567	462	676	1104	245	250	317	222	29	42	40
207039_at	423	400	1361	1138	232	137	372	454	1110	988	485	498	400	177	357	336	246	299	1556	989	992
207040_s_at	6154	5907	5236	6309	5436	5416	8059	8576	7154	5673	7018	6203	6658	7459	6361	8737	8849	6922	5113	6381	5775
207041_at	291	102	144	260	260	361	184	149	186	208	126	123	34	29	38	31	54	50	56	41	41
207042_at	101	29	23	31	103	134	56	37	27	34	24	15	5	3	8	2	5	7	25	9	17
207043_s_at	83	195	168	44	216	433	74	22	39	52	60	19	6	37	11	8	10	26	9	66	41
207044_at	349	243	302	435	558	456	273	327	375	299	274	314	29	28	31	10	48	9	82	69	70
207045_at	105	16	30	19	49	177	83	29	59	31	92	112	141	102	115	66	65	45	95	54	86
207046_at	155	73	66	122	80	130	8	95	71	87	122	186	145	116	70	9	17	19	20	61	37
207047_s_at	107	77	23	115	194	121	25	94	35	60	90	117	23	30	3	38	63	31	12	46	27
207048_at	77	73	117	74	888	668	72	117	28	24	70	118	10	64	70	68	50	50	51	54	31
207049_at	13	9	8	9	14	27	11	15	11	15	20	15	19	4	2	24	7	2	5	10	3
207050_at	114	85	134	178	60	66	46	211	134	113	98	122	20	4	37	29	18	6	46	4	21
207051_at	242	24	209	331	386	148	178	315	219	201	139	261	22	7	26	11	23	27	28	31	21
207052_at	74	66	98	81	30	85	91	108	140	95	74	46	16	4	40	17	19	15	35	12	10
207053_at	12	10	7	10	19	23	15	8	9	13	10	11	2	3	2	2	1	2	1	6	3
207054_at	28	1	3	1	12	17	5	3	5	3	3	3	8	1	1	1	1	1	15	7	2
207055_at	16	19	16	16	29	31	25	16	20	24	10	25	2	3	3	2	5	4	4	3	2
207056_s_at	13	5	26	74	120	55	12	8	12	8	38	9	51	59	46	13	37	38	25	19	5
207057_at	57	12	9	54	19	17	54	8	39	27	21	24	1	9	2	26	49	31	12	26	32
207058_s_at	40	38	187	193	287	148	68	104	102	134	78	49	15	28	5	3	46	15	2	46	38

207059_at	172	150	157	116	291	296	166	154	250	186	135	139	48	45	45	39	43	23	29	56	22
207060_at	64	67	23	70	592	495	50	63	55	82	829	1062	649	661	559	35	20	21	49	164	172
207061_at	343	254	312	365	276	485	303	325	253	298	281	249	38	55	81	43	60	37	53	78	60
207062_at	19	6	11	8	30	47	19	7	8	20	6	18	8	2	2	2	2	4	2	23	29
207063_at	21	16	14	28	18	46	12	18	8	18	9	10	2	2	2	8	7	2	3	9	3
207064_s_at	213	242	222	194	197	117	229	243	166	208	142	146	28	41	61	95	111	80	65	89	64
207065_at	53	39	184	140	415	305	111	69	235	70	421	270	90	66	64	17	19	12	45	20	25
207066_at	15	50	22	45	14	16	11	54	63	45	45	34	5	4	3	2	2	4	8	6	6
207067_s_at	53	137	37	41	33	75	122	153	197	133	98	24	28	3	4	26	34	35	29	8	27
207068_at	114	69	30	16	172	218	99	53	37	48	159	134	92	81	83	104	83	41	34	70	58
207069_s_at	95	453	549	678	154	429	78	129	559	724	249	192	139	121	170	289	181	236	701	433	422
207070_at	33	41	19	27	173	94	89	11	64	102	34	90	39	8	11	12	12	7	31	27	24
207071_s_at	1793	1555	1139	1495	819	980	1553	1796	1330	1582	1066	814	462	424	707	1049	893	556	1014	611	792
207072_at	44	24	104	92	54	114	102	92	82	164	27	101	10	3	3	2	4	4	7	6	5
207073_at	26	84	15	22	19	54	11	15	13	24	23	13	16	15	27	15	24	33	6	4	10
207074_s_at	53	56	155	136	125	36	97	145	123	92	84	133	6	6	46	9	9	8	13	5	5
207075_at	29	6	168	26	238	298	17	29	108	98	26	10	19	26	34	54	3	34	257	195	196
207076_s_at	2377	3315	1171	2403	1353	4241	3405	2714	714	878	2115	1441	1958	1506	3793	3483	4132	1999	1364	7093	6558
207077_at	49	106	69	16	291	75	73	104	67	68	91	20	24	42	3	33	7	11	12	2	3
207078_at	61	94	87	72	69	15	34	78	60	74	55	77	32	21	32	9	7	15	53	41	32
207079_s_at	852	320	370	415	738	574	1116	905	664	546	1404	1140	2058	2214	1152	1040	945	823	783	649	664
207080_s_at	8	33	5	13	118	155	28	16	60	6	7	11	7	3	6	5	7	4	6	14	24
207081_s_at	714	824	825	806	374	344	507	705	570	802	453	544	286	323	146	255	364	326	524	562	574
207082_at	90	35	26	16	78	17	15	83	59	73	70	41	4	6	7	55	5	34	4	4	7
207083_s_at	12	16	16	13	27	81	11	22	36	14	71	14	29	7	2	5	7	7	7	2	17
207084_at	17	35	26	47	15	47	13	44	21	21	77	18	27	4	6	23	6	16	3	3	1
207085_x_at	19	18	15	28	22	15	32	24	17	20	21	19	5	4	4	27	96	60	4	6	33

207086_x_at	221	134	123	101	63	73	154	170	104	133	98	131	7	19	49	42	4	20	32	7	37
207087_x_at	87	38	52	62	22	17	73	12	90	34	60	87	20	6	17	8	31	28	6	4	5
207088_s_at	1274	1358	932	930	881	939	1709	1387	1240	1145	1495	694	1340	1087	539	518	594	775	712	388	379
207089_at	8	23	8	17	15	31	54	113	92	80	13	74	23	2	26	9	21	18	27	7	2
207090_x_at	128	113	34	44	320	312	183	231	16	35	296	542	351	210	161	107	101	90	32	34	44
207091_at	126	34	9	109	32	16	97	74	111	60	97	186	27	37	15	15	3	6	4	45	26
207092_at	11	5	12	10	60	34	4	13	9	11	4	9	13	23	5	3	8	24	4	4	5
207093_s_at	251	186	142	244	447	388	204	234	199	190	162	97	25	74	72	51	22	68	63	34	27
207094_at	13	18	15	26	111	85	20	16	21	27	17	24	7	16	8	4	6	2	7	19	5
207095_at	116	122	160	105	279	98	191	87	98	208	65	143	24	50	21	28	16	19	25	16	22
207096_at	200	140	195	156	400	431	135	241	229	198	479	788	93	92	123	61	7	87	62	52	50
207097_s_at	24	12	18	14	29	51	13	26	16	11	51	11	2	3	7	6	33	2	12	3	2
207098_s_at	437	493	914	526	1293	1256	674	570	832	682	982	642	1335	1396	1582	1248	1050	1055	1585	747	1338
207099_s_at	20	9	31	34	44	34	20	22	27	27	30	44	15	5	9	7	8	4	43	20	27
207100_s_at	37	9	62	120	133	40	30	61	108	71	144	68	34	33	59	27	17	4	22	32	50
207101_at	82	92	91	78	135	74	114	96	169	46	68	118	14	43	23	28	16	12	45	16	19
207102_at	75	10	50	102	34	79	53	62	33	52	34	39	18	33	12	10	5	2	10	11	19
207103_at	24	22	19	13	14	43	27	17	20	1	24	16	1	2	8	2	3	4	15	14	9
207104_x_at	16	15	15	18	38	30	19	20	20	17	13	20	5	6	3	6	5	4	5	4	5
207105_s_at	373	443	289	440	201	259	518	553	260	403	281	256	36	79	65	146	66	90	67	69	60
207106_s_at	116	52	65	105	125	246	57	75	82	39	90	74	50	67	27	68	53	84	12	14	4
207107_at	25	43	46	3	4	95	16	22	39	20	45	28	1	4	13	2	1	1	8	11	1
207108_s_at	310	127	555	489	396	333	338	352	460	440	300	177	166	127	126	183	162	165	438	446	361
207109_at	15	10	9	8	124	17	7	7	17	4	18	6	2	8	3	22	20	13	16	27	9
207110_at	85	13	84	75	12	55	9	34	27	17	179	84	83	48	32	9	33	2	2	16	12
207111_at	212	74	140	151	93	63	166	150	169	150	190	186	35	39	31	27	8	5	39	27	3
207112_s_at	20	61	178	193	120	176	119	198	177	246	109	128	41	48	7	34	16	35	58	36	43

207113_s_at	132	32	104	105	87	165	37	58	56	35	259
	358	28	45	38	45	45	27	36	33	9	
207114_at	209	117	241	164	289	262	225	248	231	217	254
	357	78	72	148	62	87	41	72	78	65	
207115_x_at	36	49	42	10	43	39	83	160	95	150	24
	119	19	9	49	101	64	108	74	102	78	
207116_s_at	155	102	156	176	136	314	159	135	203	177	109
	175	45	48	26	31	3	32	6	7	5	
207117_at	9	36	11	109	47	126	109	49	44	52	68
	67	11	1	22	3	12	9	6	7	10	
207118_s_at	12	7	8	17	19	26	11	11	7	13	11
	11	2	9	3	4	5	2	3	6	3	
207119_at	3	1	1	18	34	55	46	3	4	8	7
	5	1	2	5	1	10	1	1	1	1	
207120_at	13	4	11	6	29	19	36	61	5	6	4
	6	4	2	2	2	6	1	3	2	1	
207121_s_at	2774	3062	3867	1977	1306	2500	2931	2770	2890	2590	1411
	1159	1910	2726	3359	6835	7770	6988	4821	5872	7344	
207122_x_at	1266	1050	718	983	995	1701	1248	994	684	773	1072
	1058	628	702	668	701	590	632	417	373	316	
207123_s_at	26	62	43	57	125	232	53	34	17	52	92
	16	48	56	45	34	59	30	79	34	48	
207124_s_at	458	650	463	614	157	281	435	666	486	1013	574
	635	352	411	403	441	448	309	578	509	502	
207125_at	5	6	31	97	32	120	89	73	54	71	20
	70	33	14	31	41	15	48	47	15	41	
207126_x_at	298	215	9695	21679	2739	3239	313	239	8896	12773	2055
	425	2192	1823	1999	86	47	70	5201	4458	5581	
207127_s_at	1414	887	1621	890	3009	2384	1181	1003	1390	1245	1563
	1225	2605	1950	1133	968	778	748	1419	1006	870	
207128_s_at	58	63	281	326	228	288	72	80	157	226	246
	270	73	40	76	2	17	4	87	69	79	
207129_at	60	40	34	115	60	43	68	48	59	77	48
	35	8	27	7	7	7	20	9	45	7	
207130_at	75	6	16	65	21	13	13	127	24	20	70
	18	22	41	40	33	25	16	49	41	31	
207131_x_at	329	457	266	410	492	507	425	358	306	309	287
	333	101	83	57	137	197	150	149	149	130	
207132_x_at	2830	4232	5129	9234	5051	9552	5749	4538	3653	4137	6266
	6969	6745	7584	7588	7157	7343	7587	4915	6076	5374	
207133_x_at	116	181	294	259	522	507	60	215	318	102	99
	255	77	62	66	5	19	14	125	112	76	
207134_x_at	98	54	77	229	55	26	27	36	59	112	40
	73	31	5	31	24	10	3	6	32	10	
207135_at	177	146	178	149	228	125	223	201	298	245	192
	181	30	52	24	17	20	47	43	31	34	
207136_at	259	74	208	253	449	603	265	276	286	239	219
	263	42	47	70	16	39	50	41	41	37	
207137_at	41	10	11	8	55	82	15	90	33	67	17
	25	5	8	39	6	12	7	10	8	19	
207138_at	17	78	23	19	98	126	85	205	31	17	20
	25	9	4	5	4	23	35	4	4	2	
207139_at	49	79	83	116	286	258	93	98	134	36	101
	30	11	2	5	22	15	13	34	29	3	

207140_at	9	5	8	8	18	19	8	7	5	7	7
	9	2	6	3	3	3	2	2	3	1	
207141_s_at	112	109	167	113	231	171	121	113	162	141	182
	131	17	18	43	48	6	28	28	27	32	
207142_at	49	9	5	10	155	19	32	8	4	13	36
	23	8	3	20	20	20	4	17	12	11	
207143_at	50	36	39	34	81	42	205	125	197	66	101
	171	25	3	33	16	39	19	37	64	7	
207144_s_at	37	21	34	27	32	28	41	59	56	31	273
	116	166	113	155	85	110	43	8	53	56	
207145_at	1	2	19	21	4	5	24	12	15	1	10
	1	1	2	9	2	13	11	1	6	1	
207146_at	138	331	151	21	36	26	224	259	108	238	53
	181	25	10	68	41	127	144	15	30	22	
207147_at	241	139	26	18	143	251	231	319	12	85	146
	185	85	87	237	406	316	410	86	124	74	
207148_x_at	13	7	12	12	19	16	12	12	13	14	13
	13	1	3	2	2	6	1	2	11	4	
207149_at	65	44	24	41	151	112	11	13	13	73	27
	10	7	15	14	8	1	39	29	10	1	
207150_at	22	106	68	32	117	26	40	40	29	91	80
	79	25	33	39	27	12	57	11	56	8	
207151_at	86	91	95	41	430	247	65	98	41	129	61
	85	19	24	9	23	13	39	19	16	20	
207152_at	265	123	262	397	414	196	209	207	280	204	267
	275	28	30	74	57	52	27	88	71	60	
207153_s_at	267	226	227	221	297	404	396	455	371	393	242
	410	560	433	609	703	554	736	794	511	890	
207154_at	71	86	114	76	62	144	148	98	190	184	115
	153	75	144	39	78	48	55	58	43	61	
207155_at	21	45	83	22	206	257	105	25	64	80	21
	137	19	19	9	3	4	4	9	7	5	
207156_at	99	129	53	105	394	470	23	87	8	15	199
	664	220	150	353	57	45	106	64	145	229	
207157_s_at	7701	8703	8150	8973	4930	4607	6881	7153	8387	7128	6327
	5205	8344	8527	7877	8494	8868	8722	10321	9251	5888	
207158_at	128	54	142	151	485	176	217	202	178	152	152
	106	38	8	48	26	39	40	48	47	32	
207159_x_at	434	272	279	288	455	450	367	438	301	278	360
	165	48	88	68	29	64	65	67	69	21	
207160_at	89	78	94	62	199	101	123	58	47	59	77
	72	17	49	30	83	62	78	21	43	45	
207161_at	8	23	11	10	18	191	8	34	13	21	51
	39	6	20	25	3	2	5	2	19	6	
207162_s_at	12	16	16	16	33	28	16	17	24	20	11
	15	2	7	5	10	11	4	6	5	6	
207163_s_at	1271	1409	1224	1092	1067	926	1742	1399	1509	2333	1718
	1119	618	413	286	447	300	384	646	230	227	
207164_s_at	29	58	91	180	12	102	62	48	50	137	91
	65	19	23	13	1	1	17	15	20	15	
207165_at	1269	318	1484	147	2951	46	1407	1836	1227	1128	2167
	2116	5276	4395	4790	3221	2685	3070	5967	1868	4573	
207166_at	5	45	3	8	8	56	4	4	7	17	3
	5	2	3	27	16	14	17	18	11	3	

207167_at	44	74	119	149	82	211	156	166	161	210	129
	123	7	70	7	5	6	11	6	25	10	
207168_s_at	5873	5075	7033	5641	6556	4640	7130	6681	7626	9203	7095
	6137	10873	12203	11255	11109	10006	12741	12884	10577	10656	
207169_x_at	4507	6513	1423	2049	2610	2444	3694	3378	851	1781	3471
	1854	1113	851	860	1557	1402	1152	391	775	568	
207170_s_at	1343	1474	1694	2621	1384	1383	1709	1606	1212	1947	1461
	1663	760	907	1467	1370	1422	1478	1218	2284	2836	
207172_s_at	25	19	71	26	60	47	33	67	31	32	97
	11	7	4	48	5	8	4	11	11	7	
207173_x_at	56	45	9	52	144	142	53	73	64	45	50
	55	22	34	29	20	15	15	15	26	15	
207174_at	9	5	5	61	11	8	7	5	24	6	7
	39	13	3	8	17	3	17	18	2	1	
207175_at	66	57	61	70	115	38	78	71	98	74	45
	55	26	8	13	14	3	29	22	8	16	
207176_s_at	269	240	262	347	282	263	317	356	343	432	287
	255	60	69	92	55	120	68	64	55	80	
207177_at	7	9	4	4	19	8	5	21	7	7	7
	8	1	2	3	1	6	6	4	1	1	
207178_s_at	73	101	68	109	62	12	73	73	56	35	33
	28	7	1	2	61	76	58	64	94	153	
207179_at	21	7	20	17	126	275	16	12	9	10	9
	14	2	6	23	2	15	15	13	6	3	
207180_s_at	619	1480	1522	2060	2689	3002	935	718	1244	1985	1647
	1484	2016	2495	1534	758	877	679	1142	1414	1172	
207181_s_at	939	988	619	907	514	957	922	975	515	696	418
	523	290	367	317	812	1008	1027	627	535	725	
207182_at	8	4	8	6	12	11	7	5	7	11	9
	18	1	2	3	1	2	25	11	2	3	
207183_at	25	15	47	34	54	48	50	21	63	45	48
	94	45	38	63	52	57	61	114	21	91	
207184_at	365	213	159	576	289	306	164	355	313	287	404
	255	33	31	7	49	55	11	55	36	35	
207185_at	184	185	156	239	180	98	146	176	135	197	188
	176	22	46	41	52	52	41	62	32	4	
207186_s_at	447	629	642	969	403	399	857	1297	923	1071	567
	430	1130	994	841	1261	1279	714	1203	1643	1184	
207187_at	511	410	461	759	603	629	600	990	622	704	574
	538	115	73	120	89	108	96	102	94	85	
207188_at	134	165	275	491	201	202	148	249	183	305	145
	109	16	11	10	53	12	11	34	4	17	
207189_s_at	152	47	214	203	750	180	86	117	244	222	249
	29	51	23	30	12	46	17	60	37	36	
207190_at	11	6	8	13	41	24	8	8	13	8	9
	9	3	3	6	6	5	4	8	7	5	
207191_s_at	98	60	122	50	225	234	32	128	100	171	45
	124	21	9	5	17	12	6	3	8	4	
207192_at	49	103	41	114	44	40	73	92	55	110	172
	109	4	26	36	11	65	37	6	11	9	
207193_at	218	287	72	145	32	23	189	187	195	330	256
	270	6	11	13	13	3	3	11	44	37	
207194_s_at	53	43	46	35	47	113	5	16	33	61	124
	73	23	24	31	1	2	13	20	4	18	

207195_at	132	27	60	58	14	46	49	9	9	15	17	26	4	37	25	19	3	28	4	9	27
207196_s_at	615	1191	920	1751	2016	2146	482	544	796	1139	2571	2620	1047	1021	957	551	691	683	1130	1262	1279
207197_at	62	13	15	89	106	105	25	18	74	34	43	54	16	3	34	6	34	4	12	5	11
207198_s_at	835	909	819	502	3335	2141	1064	1371	887	1238	4471	4377	6826	4863	1775	446	462	427	593	422	335
207199_at	40	38	30	28	234	198	17	18	23	11	18	13	28	9	11	57	78	29	59	56	25
207200_at	22	17	27	36	25	28	56	34	28	20	21	13	1	3	3	3	5	26	4	4	1
207201_s_at	13	12	19	17	65	133	21	21	9	14	13	15	23	12	5	11	11	23	20	4	2
207202_s_at	42	50	28	22	212	67	30	49	37	57	27	55	4	6	11	8	6	6	26	6	5
207203_s_at	173	57	132	47	66	27	93	70	76	94	95	161	8	7	3	6	12	6	5	4	7
207204_at	3	4	5	8	7	7	25	5	8	7	6	6	9	17	3	11	2	17	2	22	4
207205_at	204	152	345	275	153	160	89	259	214	219	259	256	53	56	61	75	61	57	53	79	41
207206_s_at	54	19	45	145	21	31	118	67	95	38	65	72	25	2	15	30	19	1	36	48	10
207207_at	127	92	60	116	114	85	89	113	126	116	108	133	13	27	18	29	28	11	5	28	21
207208_at	52	10	66	12	19	26	20	18	17	17	13	45	19	3	6	17	5	5	6	4	6
207209_at	286	232	338	184	221	383	306	322	317	309	227	270	11	29	115	63	8	56	105	16	7
207210_at	17	4	5	22	29	12	5	5	4	6	4	6	51	65	19	2	2	2	22	5	1
207211_at	242	124	212	260	77	157	261	318	292	204	202	325	11	4	10	5	6	6	5	11	19
207212_at	29	7	20	16	32	50	24	18	15	22	53	9	4	3	11	3	16	7	5	6	5
207213_s_at	36	27	27	31	12	24	15	41	17	25	27	20	5	2	2	2	2	2	2	4	5
207214_at	181	100	94	135	324	356	131	129	155	99	87	73	28	14	57	30	39	41	94	12	9
207215_at	32	19	28	21	103	91	15	16	15	25	28	14	5	14	12	6	12	12	6	5	3
207216_at	131	189	80	35	99	259	248	168	25	210	80	79	8	66	13	9	42	32	17	6	14
207217_s_at	138	74	551	388	176	374	184	162	676	1744	152	118	7	4	25	33	7	51	970	1241	2389
207218_at	116	84	106	71	225	184	140	94	66	91	65	96	2	2	53	3	1	9	12	28	30
207219_at	57	7	90	61	122	112	68	5	48	35	13	55	38	33	50	8	11	25	56	49	25
207220_at	74	66	107	131	77	159	109	110	122	70	85	58	2	26	4	12	37	23	12	21	19
207221_at	57	9	15	16	38	42	25	30	8	13	58	14	3	4	2	31	14	26	15	24	9

207222_at	192	165	92	938	173	56	180	205	238	232	84	69	43	46	50	110	99	151	79	251	70
207223_s_at	319	260	604	581	341	339	588	425	763	813	526	435	114	130	119	97	129	107	127	78	181
207224_s_at	120	129	111	122	220	151	113	36	35	113	128	64	3	5	5	8	26	2	30	4	16
207225_at	12	4	12	10	21	15	13	15	15	10	16	10	22	4	3	6	6	4	6	1	5
207226_at	21	44	22	61	52	110	32	62	27	34	33	35	7	2	7	4	5	3	2	8	8
207227_x_at	97	40	113	120	190	15	131	166	36	154	141	133	9	18	88	34	11	33	21	19	36
207228_at	111	85	164	35	411	219	65	32	54	92	51	15	6	33	46	26	51	12	47	37	18
207229_at	67	40	47	26	117	83	61	84	70	7	54	60	10	7	18	17	28	49	32	32	24
207230_at	32	7	8	36	408	474	12	29	21	36	9	30	2	40	5	26	5	2	1	6	2
207231_at	179	96	121	105	155	141	129	156	62	36	30	45	32	76	53	126	78	70	58	38	60
207232_s_at	127	35	28	67	21	35	78	113	28	42	17	58	65	50	53	110	86	156	69	62	35
207233_s_at	197	180	90	135	599	1386	205	251	178	154	209	206	77	101	179	128	50	44	61	85	41
207234_at	124	92	100	66	216	95	78	88	54	63	97	68	32	29	37	36	63	37	52	41	76
207235_s_at	7	9	19	21	136	196	40	13	51	57	47	15	6	10	3	10	21	17	7	14	8
207236_at	30	7	3	31	95	93	3	38	7	39	104	87	119	75	18	1	19	12	15	13	3
207237_at	53	21	18	26	117	86	13	24	135	29	31	21	2	2	6	10	29	3	8	15	1
207238_s_at	15	6	12	16	12	13	20	13	13	34	34	13	1	8	3	16	6	6	6	2	5
207239_s_at	656	589	875	772	650	685	1100	1128	731	1008	2001	797	251	406	529	266	301	196	396	207	196
207240_s_at	46	2	42	21	36	153	12	45	41	45	3	8	2	1	1	2	20	2	2	1	1
207241_at	56	57	53	94	91	155	45	107	52	63	44	8	19	30	6	9	14	2	17	16	1
207242_s_at	25	16	14	144	74	42	103	63	48	14	80	26	19	10	48	61	65	32	11	59	43
207243_s_at	9038	10617	19876	18255	25057	19311	14763	16528	22711	20165	22847	30837	21465	24623	26232	22506	22631	22145	17162	18508	18380
207244_x_at	54	52	266	27	543	564	138	48	82	82	50	165	53	50	7	31	21	41	107	74	62
207245_at	7	4	5	6	10	15	9	7	4	4	9	5	2	1	2	2	12	5	1	1	12
207246_at	134	64	57	47	235	262	52	28	19	73	112	60	21	38	28	1	18	13	17	3	15
207247_s_at	81	51	24	41	82	9	76	84	72	39	98	57	75	42	31	30	12	37	51	54	56
207248_at	69	112	149	70	78	203	21	61	52	38	68	44	9	10	9	4	23	7	5	6	14

207249_s_at	29	10	11	10	18	12	21	8	16	13	10	10	2	3	10	3	5	3	5	4	6
207250_at	116	68	107	120	166	247	105	131	158	110	91	112	14	6	5	2	7	4	34	30	3
207251_at	132	16	28	31	236	216	64	140	187	173	115	26	9	33	5	8	6	23	17	27	13
207252_at	124	114	109	176	349	425	130	91	48	144	129	180	34	23	27	51	47	52	40	79	36
207253_s_at	652	729	479	640	1309	1252	480	474	623	755	610	764	415	335	257	200	287	182	357	312	285
207254_at	61	24	57	14	47	59	20	95	12	53	64	15	23	7	85	69	61	63	3	22	2
207255_at	200	13	110	65	161	196	113	123	146	203	111	162	3	4	19	13	34	23	10	31	39
207256_at	4	5	28	36	10	47	25	24	12	10	7	10	10	3	5	9	1	1	1	9	2
207257_at	15	9	7	6	421	348	4	9	8	7	7	8	13	17	6	17	45	7	6	24	6
207258_at	24	17	37	25	22	55	29	21	23	28	81	23	9	7	7	8	32	7	4	22	5
207259_at	5	2	5	9	8	8	5	7	3	6	6	5	7	10	1	1	3	2	43	21	8
207260_at	33	21	45	36	96	184	25	84	94	22	27	36	17	9	8	10	6	6	10	6	5
207261_at	7	5	11	9	10	11	9	5	7	13	6	15	1	9	2	1	1	1	1	1	1
207262_at	30	39	1	6	10	9	19	33	11	39	21	11	3	23	20	21	24	20	22	20	21
207263_x_at	234	105	254	211	197	113	125	178	25	116	202	132	4	15	9	40	17	19	30	34	19
207264_at	179	118	138	13	225	43	158	172	94	105	61	145	88	97	89	149	152	123	115	44	64
207265_s_at	1804	1507	1350	1066	1325	1356	1981	2440	1733	2123	2543	2207	1611	1314	720	1319	1420	1217	673	546	715
207266_x_at	647	1313	628	901	613	844	721	529	652	615	1258	759	1141	1437	2442	995	1009	785	1308	1042	1190
207267_s_at	15	43	19	79	165	159	150	125	147	74	77	92	53	75	88	2	1	19	10	22	14
207268_x_at	872	727	1194	766	1306	1029	823	929	1069	1222	1435	1047	622	451	761	442	330	391	423	360	409
207269_at	102	71	140	124	205	160	152	128	88	85	94	35	4	9	51	31	38	45	46	34	41
207270_x_at	163	127	64	151	357	308	48	104	197	197	266	192	57	51	77	23	12	30	71	59	47
207271_x_at	9	6	12	23	44	60	25	8	12	22	11	25	16	2	2	2	4	2	3	2	3
207272_at	29	16	1	12	7	8	53	30	12	25	16	20	14	3	3	12	1	1	1	8	2
207273_at	13	21	22	1	80	132	48	26	28	41	38	30	21	11	24	18	14	2	19	27	13
207274_at	66	22	64	131	256	290	66	20	13	24	41	79	47	39	6	31	58	29	48	49	5
207275_s_at	422	299	620	881	1095	2154	451	335	554	351	1501	1113	659	677	808	216	183	173	319	469	774

207276_at	3	5	4	19	16	12	32	4	9	3	3	4	2	21	3	2	1	1	2	2	4
207277_at	258	186	199	184	297	586	167	169	182	145	152	114	31	14	11	5	39	37	83	44	29
207278_s_at	29	24	33	43	323	367	172	38	27	193	16	41	33	7	6	24	12	36	29	5	33
207279_s_at	253	237	214	253	393	640	269	356	210	282	878	621	244	162	243	73	47	84	81	172	250
207280_at	45	77	34	92	56	54	45	74	171	27	60	134	2	18	38	23	2	7	4	7	6
207281_x_at	42	100	80	54	110	101	41	86	50	119	74	57	26	38	7	13	31	50	62	9	15
207282_s_at	146	41	38	43	18	19	41	50	124	59	50	31	4	22	32	11	11	27	6	14	1
207283_at	198	184	251	151	932	477	200	187	282	300	217	429	266	55	81	103	98	85	228	252	264
207284_s_at	25	19	24	12	11	19	9	8	68	8	4	6	11	4	1	14	1	12	1	2	8
207285_x_at	20	12	11	23	162	309	53	12	12	15	13	14	4	3	40	43	21	6	2	2	7
207286_at	16	2	7	3	43	26	3	15	13	3	17	3	14	11	1	6	2	13	27	31	19
207287_at	153	142	167	164	23	79	98	193	177	122	155	109	26	53	47	36	36	23	11	22	28
207288_at	9	6	8	8	14	12	8	8	7	8	7	9	4	32	7	2	5	2	3	2	3
207289_at	271	156	315	374	838	982	248	340	203	288	267	253	44	15	53	58	75	26	29	47	45
207290_at	98	28	104	175	213	156	101	37	149	193	92	118	17	14	7	30	49	2	33	5	39
207291_at	291	633	610	343	302	172	294	343	371	630	252	250	247	104	162	230	190	149	405	400	284
207292_s_at	116	73	66	79	414	200	78	70	55	59	190	222	154	59	48	25	14	119	48	21	16
207293_s_at	52	51	28	12	91	31	20	70	94	67	45	31	4	3	24	39	33	4	17	30	5
207294_at	5	4	5	5	30	16	8	15	7	11	7	5	9	25	19	26	44	17	1	7	3
207295_at	136	22	151	44	191	145	106	201	90	120	60	46	2	23	46	27	8	52	44	20	24
207296_at	155	63	19	91	29	59	44	164	136	95	107	96	8	36	9	7	28	32	23	11	2
207298_at	169	32	126	169	177	125	131	152	235	150	112	212	25	1	16	19	45	22	49	56	14
207299_s_at	4	68	15	12	14	12	83	12	39	99	20	33	3	2	4	4	6	10	3	3	10
207300_s_at	161	131	186	247	599	927	175	156	254	200	229	161	41	25	50	16	34	46	34	59	45
207301_at	33	6	24	52	34	27	16	20	27	32	18	9	25	3	2	15	4	4	21	6	1
207302_at	16	12	60	8	16	11	9	12	5	32	21	93	1	12	5	2	3	13	4	25	9
207303_at	230	190	236	222	283	263	183	146	241	159	175	248	38	36	14	22	30	37	53	42	15

207304_at	102	79	141	186	290	360	182	199	217	288	310	313	187	189	146	50	78	77	90	54	98
207305_s_at	829	861	883	1137	455	575	942	1140	710	1106	385	495	703	752	730	1638	1716	1792	1719	1441	2413
207306_at	22	52	14	110	11	39	12	11	15	15	20	15	7	11	5	9	12	9	8	4	3
207307_at	22	19	3	12	100	70	5	29	9	6	6	11	2	3	13	2	20	1	2	1	1
207308_at	95	68	49	113	128	87	69	69	154	105	71	133	17	1	19	18	17	13	6	4	5
207309_at	294	227	312	304	393	484	326	305	249	305	206	224	4	45	31	69	82	44	69	70	45
207310_s_at	37	47	106	237	30	19	65	67	50	95	23	162	2	6	7	4	43	7	4	4	3
207311_at	73	73	122	43	419	285	227	120	17	22	36	38	11	19	39	87	86	87	71	33	12
207312_at	128	148	199	217	285	198	193	124	112	154	105	172	20	7	1	27	30	31	27	42	20
207313_x_at	167	102	113	177	203	212	144	154	136	211	111	85	40	40	48	8	38	3	22	48	11
207314_x_at	81	92	56	67	162	108	103	13	32	68	72	8	4	23	36	17	20	8	4	4	2
207315_at	17	56	46	74	98	226	81	121	51	87	63	18	2	2	22	19	1	3	17	11	13
207316_at	87	40	28	67	168	89	65	74	74	52	26	24	22	6	12	57	7	5	8	13	5
207317_s_at	41	79	22	44	26	36	45	33	23	49	38	26	6	20	3	24	20	9	6	39	5
207318_s_at	396	614	711	1068	1425	1641	403	746	622	746	975	1223	672	680	792	423	407	450	564	714	707
207319_s_at	200	127	335	266	294	446	252	253	290	316	337	298	58	46	50	46	41	40	57	119	45
207320_x_at	3321	3185	5317	5225	7545	4205	4109	3774	6795	7217	7855	10402	6382	6644	11847	5176	4901	5641	5473	7274	8151
207321_s_at	147	109	174	62	419	575	315	153	161	238	142	11	35	26	14	54	15	76	5	49	19
207322_at	91	45	64	23	85	52	58	44	47	48	109	70	14	3	10	26	2	31	13	14	16
207323_s_at	123	164	122	330	480	409	111	190	210	171	144	148	26	71	128	90	63	52	104	92	36
207324_s_at	28	15	81	52	11	17	49	70	67	38	53	25	2	4	1	1	4	2	3	2	1
207325_x_at	73	107	294	210	129	159	123	135	211	271	11	25	2	15	16	80	78	83	188	120	104
207326_at	81	148	20	56	11	13	58	124	21	25	18	23	7	2	9	44	48	72	11	46	31
207327_at	15	39	136	140	34	79	72	61	87	124	149	50	4	6	14	3	12	3	8	16	21
207328_at	9	12	5	10	22	20	15	12	24	24	7	11	11	4	26	3	6	11	5	20	18
207329_at	156	102	156	171	186	133	125	117	146	141	125	24	4	17	27	19	21	26	4	42	3
207330_at	188	43	216	216	393	202	163	232	262	218	162	202	5	3	6	42	19	15	39	5	16

207331_at	5	1	5	61	5	3	1	9	25	31	10	58	15	1	5	1	3	16	1	11	3
207332_s_at	5052	5951	8151	7946	9843	9873	10097	7548	14092	11963	10838	7870	9986	10817	13650	12209	11852	10675	11136	11893	12854
207333_at	21	24	104	137	45	191	61	57	96	29	45	23	15	3	20	3	17	30	31	11	14
207334_s_at	9	23	129	56	21	36	40	67	91	285	102	64	4	34	5	2	30	23	26	24	29
207335_x_at	2387	1724	944	1205	1919	2497	1835	1516	1127	981	1042	985	3030	2130	1032	1450	1529	2098	1558	1174	774
207336_at	11	7	5	6	10	9	7	12	13	6	9	9	1	2	1	1	2	3	1	5	3
207337_at	19	11	52	17	991	1398	8	8	21	24	10	6	11	32	42	18	28	33	47	25	31
207338_s_at	316	360	688	519	346	824	534	509	577	573	698	656	555	534	627	708	722	655	880	566	793
207339_s_at	74	49	53	125	271	384	72	69	47	21	621	273	64	90	55	50	72	56	72	60	19
207341_at	54	49	14	122	81	44	127	86	16	20	75	41	14	18	18	24	32	30	21	24	1
207342_at	40	21	20	54	26	26	12	53	8	4	40	18	8	2	30	2	2	30	6	4	4
207343_at	105	178	136	263	84	204	235	219	272	291	243	224	57	47	34	30	32	49	52	47	66
207344_at	56	32	34	35	110	60	41	59	27	29	31	20	6	12	10	21	72	11	6	12	9
207345_at	151	106	123	110	25	34	172	120	37	130	135	126	34	35	52	21	66	11	39	19	29
207346_at	311	140	27	18	316	81	160	212	163	119	279	123	34	67	38	79	7	114	13	46	33
207347_at	282	213	239	297	448	614	318	334	274	302	293	312	68	61	87	64	74	103	93	121	112
207348_s_at	396	227	475	401	169	464	394	397	482	422	432	229	85	67	125	71	81	110	111	100	149
207349_s_at	120	16	125	9	48	242	64	59	52	78	90	83	13	38	2	1	20	11	32	22	23
207350_s_at	250	90	220	189	500	509	131	166	126	124	190	238	71	73	89	66	83	59	88	57	74
207351_s_at	311	247	384	202	305	280	224	269	389	373	266	249	58	58	140	48	54	59	261	164	100
207352_s_at	299	172	91	220	219	138	292	278	230	326	249	176	5	59	28	82	78	74	41	12	17
207353_s_at	83	71	99	229	110	93	242	189	98	48	144	268	10	11	11	23	11	21	11	7	5
207354_at	30	63	90	66	147	39	28	131	33	32	94	89	2	5	21	20	16	12	8	19	23
207355_at	50	40	129	45	107	24	46	51	41	22	105	77	52	36	37	8	39	10	18	22	6
207356_at	33	26	38	58	55	27	40	57	70	45	50	21	8	4	18	17	8	44	9	16	12
207357_s_at	307	328	544	376	91	200	460	396	464	401	306	411	89	83	77	160	76	93	155	60	92
207358_x_at	1060	545	1602	1066	2346	1793	682	914	1015	1166	2096	2679	971	1008	961	544	729	644	1031	1299	1398

207359_at	65	55	33	88	192	51	105	75	32	25	55	106	22	51	31	30	35	7	10	56	10
207360_s_at	1692	463	1956	45	258	74	1550	1884	856	2193	426	62	156	158	256	835	1220	854	278	340	220
207361_at	95	50	35	56	131	144	74	111	60	59	81	68	23	45	36	33	41	49	29	5	35
207362_at	134	94	50	102	25	19	45	78	58	110	81	119	1	27	25	3	1	16	2	14	15
207363_at	20	18	8	5	93	67	45	77	83	66	43	65	8	41	24	14	14	14	13	12	10
207364_at	19	17	23	26	114	191	23	18	21	20	16	31	2	3	5	3	9	3	6	4	4
207365_x_at	751	642	686	1111	1656	1361	974	1223	1312	1321	1005	1237	327	409	344	232	260	598	417	666	305
207366_at	325	252	380	354	188	183	229	313	325	351	244	289	26	26	12	8	42	30	45	10	7
207367_at	176	77	71	106	227	235	146	150	124	112	149	132	38	9	17	19	27	7	29	15	26
207368_at	11	36	20	23	110	9	9	16	51	32	9	8	1	2	3	5	6	21	4	6	21
207369_at	247	152	99	100	440	333	68	183	182	95	88	96	25	47	49	33	19	46	37	45	26
207370_at	32	27	22	49	438	587	30	40	44	29	50	63	5	22	6	37	6	35	4	8	31
207371_at	11	24	5	8	47	5	40	17	12	27	7	8	5	8	21	1	20	12	16	9	5
207372_s_at	20	27	14	30	69	39	65	63	20	64	7	70	4	10	8	3	5	4	11	15	7
207373_at	8	7	7	8	11	12	11	7	9	7	10	6	10	12	2	2	4	4	6	1	2
207374_at	120	30	46	88	236	208	58	87	63	88	60	111	11	27	34	29	10	59	33	56	19
207375_s_at	374	539	275	234	423	626	355	264	187	152	706	398	315	290	262	296	234	363	154	185	195
207376_at	124	128	92	106	77	65	117	170	155	254	122	216	7	6	9	6	6	2	6	8	4
207377_at	13	6	7	18	38	23	9	13	7	10	10	6	2	4	2	2	2	2	2	2	1
207378_at	9	6	9	6	197	113	7	7	15	6	61	15	13	6	14	2	29	18	4	6	3
207379_at	54	30	47	50	129	184	70	92	92	64	47	35	40	56	34	9	41	25	37	43	11
207380_x_at	156	103	402	357	32	133	82	70	614	1213	111	107	18	11	4	7	14	6	293	340	982
207381_at	22	21	15	22	32	128	24	17	9	18	200	240	162	34	68	10	35	16	3	4	6
207382_at	97	189	175	93	47	55	117	115	144	140	136	196	27	13	23	5	51	26	46	31	35
207383_s_at	12	39	8	59	69	35	9	20	31	14	9	25	4	3	6	15	8	3	7	9	11
207384_at	17	11	11	10	96	83	9	13	12	15	10	14	7	26	10	23	14	8	13	11	6
207385_at	26	12	7	22	23	32	12	16	8	17	16	15	2	3	13	2	5	1	3	2	1

207386_at	64	45	99	109	146	117	87	84	122	137	95	83	11	8	27	5	20	20	31	21	27
207387_s_at	83	46	45	81	133	66	48	79	40	74	126	133	107	103	86	70	44	46	86	56	100
207388_s_at	419	404	345	207	441	337	574	401	207	338	433	398	75	133	67	147	165	74	71	12	45
207389_at	278	223	301	462	528	226	323	538	343	394	287	307	47	69	117	82	15	61	95	59	65
207390_s_at	982	1124	1053	552	356	109	1252	1502	712	720	934	627	139	168	227	370	324	373	212	185	130
207391_s_at	679	611	1110	865	1501	1505	983	921	912	1238	2130	881	518	231	362	398	420	503	372	313	321
207392_x_at	167	97	33	674	58	28	94	34	52	112	109	138	2	51	79	6	28	27	53	36	60
207393_at	103	15	62	114	60	54	142	73	79	124	51	15	2	25	7	19	34	17	4	21	3
207394_at	127	111	80	115	132	122	129	115	92	70	188	166	31	11	29	31	25	28	32	30	19
207395_at	258	186	134	171	250	336	196	212	170	203	158	54	13	2	8	38	20	6	41	21	15
207396_s_at	1341	948	1499	746	1265	861	1524	1182	1786	1624	1902	1195	1008	780	1173	976	878	825	1799	1548	1424
207397_s_at	8	2	5	4	10	11	5	3	3	6	9	3	2	1	8	2	3	1	2	1	1
207398_at	33	45	94	44	29	55	164	80	37	177	102	145	3	7	26	6	3	8	4	7	2
207399_at	77	46	30	32	54	232	19	74	20	92	48	18	4	12	52	8	27	8	8	30	33
207400_at	212	111	184	200	198	263	240	207	193	231	205	210	28	38	43	61	55	21	46	55	32
207401_at	77	38	45	49	107	78	106	66	99	78	82	132	64	81	45	19	6	19	37	44	39
207402_at	32	38	64	97	36	44	46	59	50	77	68	94	10	2	1	8	1	9	3	2	1
207403_at	123	34	141	44	426	234	228	58	106	116	102	133	36	38	44	27	52	50	24	43	55
207404_s_at	86	15	111	22	432	206	19	51	33	60	135	46	63	30	43	23	19	35	74	50	31
207405_s_at	497	544	452	343	459	1101	358	505	290	432	270	325	737	629	869	1594	1456	1187	1265	1673	2170
207406_at	24	9	20	8	48	15	38	12	4	42	30	4	11	30	11	2	1	1	1	1	9
207407_x_at	89	16	84	19	187	105	87	116	142	66	30	29	17	6	6	21	15	35	16	17	25
207408_at	310	224	301	402	697	495	379	408	371	280	250	153	64	153	136	100	104	62	99	97	93
207409_at	25	61	15	18	93	31	28	24	11	25	20	24	2	3	3	5	9	4	3	4	2
207410_s_at	5	11	4	16	16	24	1	3	3	10	1	4	2	2	5	2	2	3	2	2	1
207412_x_at	21	11	26	19	23	26	17	20	11	35	17	29	4	3	6	2	3	3	4	12	1
207413_s_at	185	277	66	146	598	704	163	201	122	161	334	407	133	136	106	72	41	90	79	116	52

207414_s_at	168	146	479	767	245	251	139	41	524	647	590	703	278	339	466	85	131	59	755	724	794
207415_at	87	71	88	348	66	86	48	69	88	171	65	54	8	19	7	2	11	3	95	22	52
207416_s_at	265	268	397	718	616	930	362	267	437	581	281	238	135	201	228	199	198	147	458	470	490
207417_s_at	155	139	42	94	170	74	167	211	70	73	101	111	40	51	63	124	104	127	34	52	39
207418_s_at	26	11	30	25	55	17	16	17	35	15	33	25	4	6	5	6	26	6	4	7	3
207419_s_at	410	599	174	278	16	50	380	429	171	150	308	196	104	49	18	128	104	164	51	79	107
207420_at	70	41	76	36	192	203	147	117	120	91	91	26	2	5	11	8	39	28	32	16	4
207421_at	120	26	134	67	63	65	125	120	131	56	161	89	20	8	2	47	5	26	62	7	6
207422_at	15	18	26	72	184	38	105	41	19	68	40	31	5	25	17	33	58	7	37	8	17
207423_s_at	194	130	241	277	278	345	200	330	273	289	198	206	32	11	33	7	43	17	34	33	9
207424_at	8	5	24	59	102	78	15	13	12	6	24	13	2	7	19	3	20	9	4	2	5
207425_s_at	549	505	423	497	243	212	439	657	443	598	1309	642	72	64	37	34	41	49	45	48	4
207426_s_at	187	179	132	129	232	181	235	215	45	194	92	167	20	39	22	205	161	123	46	49	42
207427_at	148	27	146	159	186	75	30	96	191	96	33	70	48	39	26	47	36	37	13	6	17
207428_x_at	844	736	509	632	700	364	633	713	737	432	915	450	307	152	100	154	102	154	159	96	102
207429_at	12	28	12	101	44	48	30	33	17	49	40	43	10	6	6	6	5	2	35	37	41
207430_s_at	7	30	22	8	12	34	11	5	3	45	6	6	1	36	1	1	1	5	1	1	1
207431_s_at	971	824	728	313	914	591	673	736	423	681	1359	1116	1134	838	1048	856	997	811	484	392	447
207432_at	95	28	102	67	32	43	119	61	45	52	74	97	22	55	44	2	40	24	7	5	43
207433_at	28	86	115	22	356	24	28	67	27	53	316	269	406	176	160	9	21	1	11	19	5
207434_s_at	87	105	61	57	283	265	131	55	21	50	45	45	44	14	69	47	22	61	46	48	21
207435_s_at	3224	3828	2374	1967	707	921	2345	2709	2016	2102	1522	1164	506	260	275	220	360	409	515	407	378
207436_x_at	1081	833	958	892	1519	1477	735	933	767	962	885	1587	434	532	468	456	316	743	627	570	598
207437_at	3	2	1	62	32	5	15	1	24	1	21	1	1	3	5	12	17	18	14	19	9
207438_s_at	1393	1142	1511	1511	595	524	786	862	1500	1332	537	674	678	659	748	1026	1197	892	2533	1571	1795
207439_s_at	323	358	490	405	372	181	362	425	456	570	546	368	95	101	55	96	83	122	107	92	99
207440_at	164	94	94	128	150	282	53	71	116	61	82	88	35	46	72	54	94	30	47	39	48

207441_at	110	11	31	14	23	48	19	15	16	15	11	21	16	3	3	27	2	20	6	2	1
207442_at	15	34	15	96	25	17	19	16	64	29	13	18	10	6	6	6	12	4	5	5	3
207443_at	37	18	14	10	14	20	34	24	8	18	16	20	3	8	1	4	24	16	8	7	5
207444_at	16	9	14	17	26	31	12	12	16	14	11	9	4	33	5	3	6	10	4	3	3
207445_s_at	131	63	110	175	190	179	126	125	120	144	94	92	13	18	41	36	20	2	40	34	41
207446_at	144	82	144	142	147	140	127	95	78	70	78	83	27	38	17	46	9	21	20	13	27
207447_s_at	30	28	28	53	91	79	13	28	40	27	31	54	10	8	14	22	13	12	4	10	13
207448_at	16	16	12	18	60	47	15	8	8	10	7	11	30	10	21	24	7	6	22	4	3
207449_s_at	239	144	212	177	269	66	195	224	185	187	188	147	35	46	48	40	44	23	44	25	26
207450_s_at	7	4	46	6	19	106	11	9	12	11	9	9	1	3	4	2	1	4	1	1	5
207451_at	169	215	30	27	331	370	73	82	23	13	65	16	10	4	7	42	13	12	7	21	5
207452_s_at	16	26	20	62	132	168	9	58	13	29	10	50	26	31	12	36	3	6	4	27	9
207453_s_at	11	7	8	13	15	34	8	9	13	8	11	6	1	2	2	2	3	4	1	2	1
207454_at	28	24	28	41	23	106	30	11	20	15	14	29	3	3	3	1	7	5	2	2	17
207455_at	101	74	110	150	66	146	122	98	92	113	136	9	1	16	14	10	19	1	45	29	48
207456_at	15	7	38	63	14	8	11	11	29	103	24	13	3	6	4	5	3	31	15	2	35
207457_s_at	142	60	125	239	843	1275	98	194	123	191	755	555	601	548	909	10	33	4	25	24	17
207458_at	349	248	269	350	536	379	289	297	376	478	340	352	97	152	135	120	129	122	170	125	91
207459_x_at	34	120	23	184	73	181	114	38	190	271	230	40	19	53	5	9	6	9	20	62	34
207460_at	89	84	22	26	485	284	72	26	37	29	41	43	57	42	15	4	44	12	3	27	14
207461_at	22	29	30	6	33	11	36	46	23	18	33	10	22	1	9	1	2	2	6	17	11
207462_at	205	13	156	178	36	263	32	96	13	280	99	146	2	41	8	9	11	33	7	37	18
207463_x_at	1286	1094	5140	2501	1146	1004	1328	1103	3116	3621	1217	976	928	543	813	490	632	661	2142	2497	1701
207464_at	123	54	140	158	201	146	131	111	175	138	122	113	32	26	30	20	25	46	41	18	11
207465_at	62	1	38	45	5	62	38	17	43	55	36	36	2	1	10	4	25	18	6	10	14
207466_at	169	99	126	127	412	452	189	154	146	127	129	92	35	47	90	62	50	43	43	51	45
207467_x_at	1819	2156	1860	1543	2803	4214	1610	1648	1706	1821	2095	2256	2319	2137	2548	2315	2396	2767	1824	2552	3810

207468_s_at	12	6	16	13	95	379	7	8	5	8	425	212	108	88	18	6	10	11	46	12	32
207469_s_at	1205	1507	411	852	841	1934	1059	1214	356	349	857	951	976	1166	562	905	645	722	475	500	438
207470_at	19	7	14	12	23	36	16	62	58	43	90	68	1	6	4	5	2	7	15	19	19
207471_at	41	5	41	48	40	4	25	17	60	10	18	30	3	2	10	1	10	11	8	15	2
207472_at	4	6	5	31	7	65	7	22	11	18	4	10	1	9	13	3	10	2	3	13	1
207473_at	205	146	145	275	125	196	184	162	181	159	159	136	25	44	10	19	44	21	32	67	16
207474_at	49	66	4	48	58	122	44	86	36	77	27	60	10	27	25	14	13	28	48	20	27
207475_at	15	26	18	32	246	106	56	25	40	119	72	59	4	21	5	13	50	39	7	37	5
207476_at	37	10	9	30	103	138	29	17	13	10	18	9	2	7	6	16	7	3	13	9	4
207477_at	114	84	38	62	74	140	34	40	83	95	31	68	10	17	14	17	1	8	4	21	3
207478_at	87	79	115	80	120	125	62	69	118	78	54	62	15	19	8	16	2	12	34	14	9
207479_at	7	4	5	12	37	103	36	12	3	10	1	3	6	17	1	16	2	10	2	23	11
207480_s_at	442	695	336	419	355	486	345	290	301	268	269	234	436	270	210	790	560	705	451	492	579
207481_at	44	7	3	23	115	26	42	9	11	67	11	15	2	6	3	4	5	11	5	4	7
207482_at	152	78	153	159	148	321	200	113	82	182	132	147	8	30	47	43	40	91	32	32	7
207483_s_at	1599	832	1355	1242	1473	1287	1333	1454	1840	1424	1475	1248	1456	1277	1579	1739	1534	2100	2891	1559	2532
207484_s_at	250	243	96	69	203	28	264	317	250	211	252	72	50	83	15	47	19	37	6	33	21
207485_x_at	13	15	14	116	36	203	15	22	21	25	20	11	2	14	57	38	6	92	5	46	39
207486_x_at	266	195	252	297	500	402	221	272	286	285	243	182	45	77	85	58	33	18	70	94	111
207487_at	0	10	34	44	70	77	30	16	8	28	3	3	2	34	15	3	14	8	6	2	2
207488_at	13	11	14	28	48	43	13	22	24	18	21	18	8	7	11	4	10	11	8	12	9
207489_at	13	51	54	52	76	67	102	75	76	70	63	94	5	11	19	13	2	21	6	16	38
207490_at	242	72	201	202	645	423	195	46	298	214	327	268	110	135	70	57	48	150	123	92	80
207491_at	193	34	68	49	183	173	42	102	153	48	38	43	6	43	30	37	83	9	58	64	57
207492_at	85	78	65	110	22	59	166	124	153	193	109	118	4	36	40	22	5	2	21	46	2
207493_x_at	30	10	20	28	181	234	27	74	19	74	30	36	15	44	36	13	7	17	5	5	2
207494_s_at	26	78	27	54	37	39	42	41	25	25	30	64	99	72	100	23	83	44	11	7	72

207495_at	151	75	37	12	224	262	98	107	33	31	87	117	127	80	37	118	95	60	31	59	40
207496_at	25	35	37	66	52	12	111	96	100	78	50	5	5	3	23	1	3	10	22	30	20
207497_s_at	108	90	212	274	212	185	146	206	98	159	142	82	5	7	29	5	18	30	10	7	8
207498_s_at	233	245	220	459	515	517	175	211	186	256	78	194	46	48	41	66	121	78	84	59	44
207499_x_at	1077	1252	1358	975	1093	701	974	949	1026	1389	600	415	56	165	77	98	100	163	159	159	77
207500_at	97	26	20	75	62	40	9	45	58	80	36	53	15	6	2	5	6	7	26	31	27
207501_s_at	97	95	80	114	208	211	95	144	96	85	104	59	24	33	34	21	14	36	21	28	18
207502_at	8	5	7	12	265	392	5	8	5	6	4	6	2	32	19	10	12	3	4	9	3
207503_at	124	43	79	88	150	38	138	73	146	84	51	114	41	47	57	6	28	28	29	28	19
207504_at	405	165	225	191	109	58	278	367	301	383	276	359	10	9	41	9	47	7	6	27	22
207505_at	202	107	205	203	386	410	182	176	119	194	98	264	25	27	42	31	16	41	17	20	46
207506_at	28	135	138	135	173	99	151	149	110	189	216	147	69	76	41	17	5	15	70	64	60
207507_s_at	13170	7413	7064	5917	8761	5597	9172	7679	9250	6861	8365	8509	13161	10939	8430	7031	7650	7788	7122	5784	3324
207508_at	12094	7803	7287	5842	6844	3966	8252	8254	8676	7585	5870	8883	10716	10155	9140	8230	7390	7842	8964	5943	3388
207509_s_at	180	99	199	166	63	30	154	84	135	189	109	204	4	7	9	40	9	28	8	7	5
207510_at	139	148	109	176	334	266	139	121	138	88	196	134	22	22	27	9	6	27	32	31	15
207511_s_at	62	34	161	100	280	103	142	80	99	280	196	177	100	15	13	13	13	9	10	17	21
207513_s_at	523	588	251	479	460	779	554	569	219	231	431	601	458	384	367	621	532	720	442	711	956
207514_s_at	12	6	11	12	15	13	11	16	9	13	10	9	4	11	4	7	8	2	4	2	5
207515_s_at	583	404	713	530	904	938	1122	895	772	641	615	645	728	438	515	715	625	940	551	616	438
207516_at	20	22	42	61	22	34	93	98	82	68	111	11	31	13	38	19	1	22	21	6	19
207517_at	52	40	267	14	487	118	118	67	162	155	132	98	78	91	56	25	30	45	93	57	37
207518_at	53	21	60	47	144	122	40	59	54	35	98	82	24	57	33	5	15	33	43	66	102
207519_at	204	140	197	189	256	133	244	157	128	211	114	200	5	8	9	29	11	9	10	16	5
207520_at	118	82	34	70	158	56	85	125	88	70	64	84	23	13	14	21	27	39	27	21	26
207521_s_at	11	36	8	25	33	15	13	18	13	15	11	11	5	10	2	36	23	32	6	3	21
207522_s_at	155	190	94	301	70	27	170	44	33	131	112	55	17	5	8	33	131	134	38	44	47

207523_at	38	15	49	94	33	12	37	26	40	28	37	19	9	2	2	2	4	1	20	2	3
207524_at	456	276	530	415	333	156	407	381	537	458	453	342	93	45	185	87	119	131	186	139	158
207525_s_at	1302	1120	1363	1680	1134	954	1087	897	1123	1013	1160	861	485	446	554	637	508	697	627	392	395
207526_s_at	75	75	149	111	82	22	113	104	84	115	24	21	22	22	13	7	25	20	19	15	11
207527_at	140	92	136	127	56	52	86	152	169	159	90	111	57	49	52	12	6	45	33	42	38
207528_s_at	261	163	477	162	175	175	497	286	380	485	179	126	38	31	54	126	118	135	103	113	131
207529_at	218	201	130	178	125	194	260	264	277	236	270	209	14	41	10	3	19	45	39	19	29
207530_s_at	9	12	34	14	43	20	8	8	32	38	41	16	14	6	6	37	12	8	8	1	5
207531_at	102	34	20	59	378	65	77	67	54	102	60	30	6	9	42	5	23	8	6	11	12
207532_at	49	106	66	70	179	13	77	29	88	52	57	83	39	38	43	59	54	44	59	59	54
207533_at	11	6	8	9	21	17	13	13	7	14	10	10	5	6	3	5	2	4	6	9	8
207534_at	102	10	47	25	32	62	80	45	37	32	33	73	23	12	1	87	98	91	8	3	9
207535_s_at	429	858	469	326	643	586	477	414	306	306	668	325	57	122	129	138	93	120	73	61	57
207536_s_at	148	106	146	44	378	519	121	102	79	143	333	388	14	62	51	16	26	19	43	29	43
207537_at	147	136	56	125	48	48	158	239	135	183	149	161	18	36	17	27	68	43	36	49	27
207538_at	11	32	14	13	27	28	40	18	31	46	10	15	14	6	9	3	2	4	4	1	5
207539_s_at	5	5	9	4	32	75	41	57	5	6	6	6	11	3	1	13	4	15	19	3	17
207540_s_at	58	46	772	528	5042	1610	73	45	796	808	3768	1306	3692	3204	5515	91	30	73	827	944	1125
207541_s_at	697	533	418	224	745	633	643	681	325	285	517	251	489	644	1032	1487	1353	1663	1004	932	1217
207542_s_at	134	73	76	135	45	145	134	96	33	84	31	90	4	3	8	46	34	30	51	8	39
207543_s_at	1046	1240	1206	951	1020	1281	922	1226	704	938	719	782	735	843	1351	1817	1771	1391	1014	1499	2315
207544_s_at	22	58	98	70	114	89	57	29	40	36	44	19	1	38	23	3	7	4	6	19	6
207545_s_at	155	85	171	154	619	540	102	140	115	119	171	339	364	286	160	144	95	106	244	197	285
207546_at	97	75	132	96	253	251	256	113	95	130	99	15	4	52	23	15	17	9	6	2	10
207547_s_at	42	203	159	98	165	169	176	182	203	105	75	131	5	34	31	10	4	16	11	19	27
207548_at	25	9	8	17	27	66	12	34	44	21	30	82	1	7	4	4	14	2	4	5	1
207549_x_at	1659	1918	2393	2911	2283	1314	2253	1905	2419	3051	2746	2507	332	416	684	1018	871	954	582	701	1366

207550_at	82	60	16	78	45	43	36	53	28	28	58	73	76	73	152	65	74	47	260	330	512
207551_s_at	483	314	402	519	1060	802	632	512	400	512	752	809	821	549	752	413	278	329	473	424	705
207552_at	66	68	123	119	392	309	191	41	150	155	233	82	27	39	26	4	21	34	48	8	19
207553_at	156	94	87	109	349	470	140	78	128	112	169	107	19	33	25	22	4	27	28	9	7
207554_x_at	335	288	311	268	300	204	427	395	239	349	384	263	75	98	19	184	127	118	69	54	43
207555_s_at	106	140	113	128	697	563	156	57	82	131	146	94	80	59	93	65	130	140	177	115	99
207556_s_at	819	652	1025	603	1336	595	828	585	1157	1212	1381	933	458	568	403	275	347	216	508	430	311
207557_s_at	13	4	11	10	15	22	9	16	12	10	20	10	3	18	2	2	26	18	4	1	3
207558_s_at	148	151	140	133	58	120	118	83	138	92	14	20	43	28	27	85	135	113	303	236	268
207559_s_at	741	532	623	1120	1390	1379	615	670	808	727	549	871	321	387	194	247	175	256	230	246	233
207560_at	233	116	186	219	513	654	166	116	139	155	277	107	7	17	82	49	78	51	61	49	45
207561_s_at	87	114	72	141	47	230	105	80	120	136	115	132	6	3	13	8	3	34	27	12	28
207562_at	11	23	20	17	48	15	24	13	20	35	23	19	3	27	3	7	8	5	6	4	40
207563_s_at	332	331	1043	1997	911	1157	712	936	1021	1448	1646	1204	468	433	277	157	179	197	285	203	318
207564_x_at	527	399	1647	1611	969	1437	1130	1258	1219	1539	1440	1090	523	501	436	458	348	451	531	657	1073
207565_s_at	127	113	123	198	51	200	158	150	179	134	219	175	99	40	74	79	127	101	86	147	133
207566_at	53	28	26	75	220	102	54	36	47	41	54	38	8	11	5	4	5	11	8	5	36
207567_at	29	27	43	31	23	26	11	36	32	63	10	34	5	42	3	27	17	31	23	34	5
207568_at	270	182	283	213	146	124	294	251	249	309	209	293	64	32	50	14	12	59	22	29	53
207569_at	101	62	92	107	133	187	125	158	67	122	108	57	16	27	44	12	32	16	17	31	15
207570_at	19	18	41	17	21	42	19	18	20	17	20	24	4	3	7	8	2	3	4	2	5
207571_x_at	163	153	31	89	377	356	188	230	99	94	94	131	77	99	80	195	101	107	9	49	25
207572_at	75	64	94	137	126	165	53	110	110	81	60	69	17	31	22	22	20	47	14	19	28
207573_x_at	5403	6258	7876	8042	6504	4714	8544	6396	8585	7272	10079	9296	12875	12056	9846	6906	7618	7139	6991	6506	4574
207574_s_at	718	2215	208	63	904	1687	258	323	12	49	696	567	825	670	413	353	447	462	144	154	165
207575_at	56	108	125	243	23	103	82	102	139	138	10	10	21	15	6	8	13	25	29	32	37
207576_x_at	116	36	102	58	309	87	62	61	111	60	125	109	33	14	74	9	12	28	62	35	44

207577_at	64	50	95	149	142	112	89	117	123	96	98	118	4	35	13	13	41	45	17	16	16
207578_s_at	323	246	79	191	88	200	77	57	163	74	115	266	162	76	167	116	86	62	115	149	107
207579_at	24	22	14	9	33	30	16	34	39	28	51	62	2	27	3	5	12	17	15	2	17
207580_at	49	2	50	10	18	79	11	24	8	55	17	19	6	7	14	7	16	14	8	2	11
207581_s_at	52	26	16	14	32	20	5	17	19	4	10	62	1	2	18	3	9	2	2	2	1
207582_at	118	80	52	135	135	321	30	108	32	74	97	25	89	39	60	65	39	59	46	81	49
207583_at	8	50	79	47	120	56	62	66	5	53	55	43	1	3	6	6	3	1	2	15	2
207584_at	22	32	45	12	69	105	8	38	17	35	6	43	6	10	2	16	2	2	2	4	1
207585_s_at	4696	5964	4852	5812	4946	6692	8259	6462	5479	5279	5287	6892	7608	7815	4798	7492	7072	6246	4888	5329	2903
207586_at	79	13	167	18	103	56	28	22	114	172	185	166	41	4	26	2	15	11	8	32	52
207587_at	98	36	19	41	122	94	49	153	154	143	159	74	23	25	9	33	34	6	7	2	27
207588_at	37	21	22	57	62	38	4	13	12	11	23	4	14	12	5	7	14	4	4	5	5
207589_at	73	16	19	22	89	54	30	65	17	75	131	112	38	50	11	29	3	4	2	26	9
207590_s_at	94	54	217	40	346	161	150	183	276	352	317	309	178	155	151	46	99	60	156	82	143
207591_s_at	13	43	24	18	82	15	147	120	72	60	74	8	26	63	61	48	77	22	25	18	17
207592_s_at	37	12	85	87	74	142	38	59	84	50	124	63	31	44	48	16	31	27	7	8	13
207593_at	70	116	205	184	370	321	192	189	223	222	236	141	36	13	9	42	14	37	34	22	27
207594_s_at	163	157	144	123	107	207	186	169	181	215	280	210	40	42	68	26	33	30	55	24	35
207595_s_at	319	566	191	300	172	418	500	629	186	282	311	214	51	12	11	115	121	140	87	66	86
207596_at	70	38	71	78	136	34	54	65	66	50	40	44	14	19	33	17	10	7	11	5	9
207597_at	74	44	65	41	234	145	74	98	56	92	48	48	17	36	19	36	51	39	32	29	10
207598_x_at	421	241	414	367	764	456	470	573	495	633	435	459	143	198	98	97	94	168	224	234	115
207599_at	38	24	24	39	25	7	72	22	71	17	9	6	1	11	3	2	2	9	4	6	2
207600_at	196	131	293	119	38	32	167	152	158	210	198	189	8	32	11	5	6	3	23	4	20
207601_at	312	187	290	281	349	275	367	261	310	274	229	96	28	36	10	23	40	27	53	7	7
207602_at	328	173	298	281	198	220	293	331	217	289	297	202	31	38	49	66	52	18	95	24	35
207603_at	12	11	7	25	16	16	41	33	28	21	9	13	3	14	37	3	1	3	22	1	16

207604_s_at	138	134	351	239	21	67	281	313	363	376	119	142	4	26	34	56	25	50	84	39	78
207605_x_at	200	144	225	168	423	31	310	391	195	154	270	137	79	11	80	41	50	63	76	70	56
207606_s_at	1285	741	1187	845	866	797	1746	2023	933	885	730	620	928	962	1534	2387	1747	2011	1477	985	1388
207607_at	3	1	1	3	5	3	3	1	5	3	3	3	58	4	6	5	1	1	1	1	1
207608_x_at	389	347	377	444	404	536	293	405	561	425	480	292	75	117	93	175	127	192	248	131	151
207609_s_at	41	28	19	18	55	132	28	29	19	18	16	8	13	20	3	5	22	5	13	19	7
207610_s_at	176	97	64	36	66	99	306	277	78	31	331	187	127	206	318	204	261	232	41	132	177
207611_at	20	52	92	66	18	17	37	49	114	120	48	50	5	41	11	9	29	6	6	25	27
207612_at	15	19	15	9	117	55	20	8	9	15	10	13	14	12	3	7	9	17	16	5	28
207613_s_at	29	16	18	21	21	38	24	18	9	27	20	20	2	5	7	4	2	23	4	4	4
207614_s_at	1790	1043	1772	1530	1895	1773	1385	1635	2276	1747	1984	1522	1512	1994	2360	1435	1263	1229	2129	1843	2553
207615_s_at	36	11	31	30	55	50	134	42	63	75	34	18	1	14	2	2	8	3	18	19	19
207616_s_at	1118	849	3072	2874	983	1181	1944	2408	3849	4423	1005	1018	443	421	550	2147	2377	1983	4034	2424	3367
207617_at	48	46	7	27	48	40	52	15	17	32	10	43	2	2	3	26	2	2	2	3	3
207618_s_at	1466	858	1282	857	1546	1367	1143	912	1366	931	1153	1181	1157	957	1104	710	698	561	1173	874	698
207619_at	60	52	8	19	34	26	15	46	68	18	17	15	2	3	33	2	7	4	2	5	11
207620_s_at	214	197	156	137	157	152	368	268	135	278	253	119	102	131	146	123	133	132	107	153	152
207621_s_at	761	701	521	463	192	486	719	748	670	671	466	313	474	523	553	769	911	752	963	735	472
207622_s_at	1631	566	1834	907	649	474	1746	998	2269	1431	1332	743	617	753	1759	1248	1204	1225	1821	1266	1218
207623_at	102	150	7	54	33	360	16	21	7	31	14	10	21	10	19	47	130	52	19	66	42
207624_s_at	153	179	113	93	216	126	188	218	106	143	207	275	177	212	255	157	229	225	153	100	219
207625_s_at	291	269	278	608	493	620	307	434	399	528	509	567	202	246	210	152	173	195	332	289	322
207626_s_at	11	9	90	87	59	67	33	80	19	80	16	14	5	24	23	6	25	5	4	6	5
207627_s_at	565	189	479	345	572	615	606	508	640	519	587	396	288	145	175	181	154	142	231	254	245
207628_s_at	3647	1558	4733	1702	3201	3022	3442	2717	4917	4609	3647	4047	4775	3788	3376	2098	2031	1449	3495	2473	1643
207629_s_at	422	530	1165	798	432	521	564	487	626	890	556	442	108	103	50	81	91	77	94	190	150
207630_s_at	549	532	438	516	550	728	523	512	344	292	441	527	229	225	195	210	231	210	137	167	186

207631_at	13	67	134	114	29	60	36	71	17	98	102	117	51	56	84	52	61	44	6	42	91
207632_at	41	22	12	12	29	26	36	17	8	14	28	46	3	1	2	4	10	3	1	4	30
207633_s_at	90	44	85	72	188	251	17	30	76	103	68	132	2	14	12	1	7	14	7	6	21
207634_at	138	73	231	263	104	270	188	135	102	157	158	175	21	30	58	12	19	7	45	56	5
207635_s_at	65	7	12	10	34	113	11	21	16	14	4	6	2	2	38	5	10	5	1	2	16
207636_at	172	41	201	102	147	267	131	201	140	171	144	103	4	18	50	2	11	22	10	5	26
207637_at	12	18	33	27	33	38	12	24	17	10	17	20	2	9	5	8	3	4	4	7	31
207638_at	40	11	57	25	11	74	78	36	13	52	53	5	4	6	19	15	1	17	20	24	11
207639_at	25	56	15	13	60	38	30	16	13	20	153	75	46	47	6	19	17	54	7	11	3
207640_x_at	9	6	34	5	87	99	15	9	8	31	4	5	13	4	62	5	46	19	21	34	35
207641_at	175	260	56	358	253	202	348	282	245	274	289	269	39	54	66	98	85	123	80	36	24
207642_at	181	46	87	162	294	208	133	154	106	140	99	84	17	14	58	2	26	32	33	36	34
207643_s_at	2318	2320	1706	1677	962	912	1436	1561	1153	1702	1312	784	585	765	837	1153	993	1074	719	564	583
207644_at	48	57	79	145	278	331	94	121	163	82	84	49	4	5	8	21	8	19	25	9	15
207645_s_at	328	210	355	173	186	312	457	410	532	458	190	171	72	20	33	59	17	43	76	38	40
207646_s_at	164	139	195	107	239	185	166	194	171	182	159	201	20	64	5	23	7	26	41	57	17
207647_at	7	32	26	25	23	19	29	9	11	7	17	30	15	3	21	3	19	2	5	7	13
207648_at	147	119	52	62	60	89	292	79	162	193	77	54	11	7	7	2	10	6	40	21	37
207649_at	122	91	140	160	236	265	158	129	159	198	117	202	42	41	84	27	35	3	39	45	29
207650_x_at	29	21	26	30	117	181	27	30	28	29	27	30	7	4	15	5	34	13	8	10	7
207651_at	12	12	11	23	12	30	27	16	12	14	20	9	2	8	1	3	5	2	2	2	2
207652_s_at	70	22	15	125	253	85	148	100	146	144	78	20	7	3	11	10	20	8	43	6	33
207653_at	118	92	85	88	139	198	139	169	80	77	108	15	17	45	37	25	33	48	60	40	32
207654_x_at	1217	1243	2041	1287	953	611	1504	1359	1506	1718	1798	925	1047	961	1557	1231	1008	1215	1485	1444	1453
207655_s_at	61	63	69	354	33	16	77	57	71	82	45	40	7	3	17	31	25	7	42	64	68
207656_s_at	22	80	11	13	30	19	21	44	17	7	17	10	35	8	11	40	3	18	11	6	23
207657_x_at	890	731	2102	1661	1938	1107	1354	1268	2712	2467	2238	2348	2706	2574	1946	690	711	534	2172	2193	2297

207658_s_at	15	64	39	26	34	19	40	32	35	36	44	35	23	52	47	41	26	87	18	12	5
207659_s_at	147	83	79	100	148	74	106	125	64	124	71	141	9	35	19	33	25	45	36	42	8
207660_at	180	49	79	175	227	270	76	128	128	190	122	108	16	6	36	25	14	28	26	26	12
207661_s_at	97	58	73	101	144	54	57	106	13	52	67	63	2	9	10	2	3	12	20	19	3
207662_at	105	75	109	16	374	380	89	133	107	91	6	111	20	31	26	19	57	31	39	13	40
207663_x_at	106	61	104	146	65	36	98	182	144	215	60	102	20	15	17	4	15	4	25	7	2
207664_at	3	10	7	75	120	50	5	3	4	7	6	3	1	6	11	7	5	27	15	14	21
207665_at	269	176	170	219	304	243	302	220	260	208	185	94	25	32	35	66	54	90	50	32	31
207666_x_at	15	58	141	22	113	331	21	113	98	81	171	14	47	51	46	3	16	27	3	11	17
207667_s_at	865	724	1080	746	867	348	1060	834	994	1342	1354	699	636	544	463	396	459	351	507	300	234
207668_x_at	9564	4398	13379	9153	9183	8054	6845	5121	8393	10513	9519	8469	9343	9525	8046	6806	8716	6634	9237	11304	12748
207669_at	17	96	31	38	30	42	24	38	24	41	20	13	7	6	10	6	14	9	8	56	6
207670_at	213	60	197	257	195	196	270	125	138	221	145	94	40	20	10	33	24	59	4	19	56
207671_s_at	134	61	104	47	58	113	81	32	91	98	71	87	50	43	4	43	33	37	11	44	25
207672_at	42	10	85	8	14	103	9	32	13	24	11	9	1	2	27	2	5	4	6	3	9
207673_at	24	17	8	19	40	44	16	17	21	17	18	74	16	23	21	8	9	8	15	22	9
207674_at	25	9	43	57	103	34	33	11	32	41	36	45	13	7	20	2	1	4	15	16	29
207675_x_at	623	998	134	160	544	1000	492	322	254	238	286	266	77	86	100	185	207	193	104	57	68
207676_at	176	56	194	180	287	536	138	162	193	229	135	126	6	5	4	21	8	3	51	38	38
207677_s_at	8	13	12	10	27	46	3	12	13	8	6	38	3	11	2	2	6	22	3	8	3
207678_s_at	66	29	61	59	44	73	46	38	20	59	40	99	2	29	24	23	22	12	40	32	67
207679_at	8	73	3	9	62	66	11	4	45	42	57	60	20	4	17	22	14	18	12	6	5
207680_x_at	93	21	5	133	330	305	117	135	51	84	112	131	15	58	51	7	21	38	18	43	30
207681_at	48	40	126	98	76	44	29	79	40	25	31	65	2	3	7	12	11	6	14	12	5
207682_s_at	40	44	16	25	213	216	8	16	8	15	9	8	6	3	8	8	15	22	4	26	4
207683_at	16	10	11	12	27	34	16	21	15	13	7	8	4	2	2	2	16	6	13	3	3
207684_at	296	257	247	398	478	390	323	289	284	366	249	214	35	49	45	40	47	64	74	55	34

207685_at	11	7	11	12	22	47	11	12	11	11	10	9	3	2	6	5	3	7	3	4	4
207686_s_at	385	431	373	278	147	281	313	314	310	629	338	323	80	57	80	110	117	202	233	116	215
207687_at	259	163	259	295	294	296	207	264	242	319	274	202	42	50	93	66	62	68	79	56	98
207688_s_at	730	526	365	198	437	341	567	672	442	502	310	307	247	186	446	625	425	648	1030	399	313
207689_at	200	72	205	166	209	157	150	205	103	131	135	192	42	43	61	45	30	37	41	71	29
207690_at	128	97	19	144	645	251	121	115	128	110	28	10	17	9	7	34	6	21	46	23	36
207691_x_at	160	128	152	189	315	180	90	136	154	159	199	145	3	73	66	16	20	15	26	29	29
207692_s_at	243	136	148	156	532	476	207	121	210	260	226	234	59	34	49	28	19	29	48	79	43
207693_at	79	26	104	19	65	98	11	21	9	73	78	93	1	3	29	4	6	23	3	7	3
207694_at	110	57	62	85	82	63	83	45	115	57	99	88	13	22	25	17	18	40	32	10	13
207695_s_at	40	154	201	433	198	251	73	145	397	205	24	64	2	6	17	62	46	56	165	106	104
207696_at	3	2	1	3	4	12	4	4	1	3	1	4	0	10	1	1	3	1	1	1	1
207697_x_at	9	13	8	62	23	24	33	8	11	34	21	21	4	15	5	5	15	10	8	6	7
207698_at	38	21	87	150	125	223	184	154	162	217	138	101	5	9	7	7	27	8	50	83	58
207699_at	200	170	239	198	775	379	144	199	179	147	168	134	56	90	96	76	88	74	114	86	35
207700_s_at	201	169	487	623	393	321	443	523	519	618	586	554	596	520	373	292	392	277	420	402	662
207701_at	161	236	195	297	430	427	276	315	301	252	301	249	60	62	59	91	51	62	67	50	46
207702_s_at	8	4	4	5	12	20	5	9	9	3	6	13	5	4	3	1	2	1	1	2	1
207703_at	11	9	35	8	18	75	48	41	12	66	43	64	5	3	18	2	2	2	1	5	5
207704_s_at	58	10	9	67	45	39	111	70	35	50	105	150	9	9	3	4	7	3	5	9	1
207705_s_at	191	311	91	136	526	438	284	351	159	143	350	353	306	348	354	194	225	120	59	65	75
207706_at	34	4	31	44	22	13	8	5	41	8	20	8	7	1	2	4	1	18	4	11	21
207707_s_at	3133	1299	2956	1758	2891	2272	3195	2253	2460	2215	2153	1281	3045	3101	1739	2932	3660	3248	2427	2577	1877
207708_at	378	108	355	404	445	656	375	358	355	328	333	253	19	14	26	42	21	90	99	71	64
207709_at	82	45	49	107	296	282	138	115	43	63	145	158	68	55	97	57	43	42	35	27	70
207710_at	81	11	7	78	45	95	77	20	44	48	54	40	14	25	16	29	23	27	18	36	18
207711_at	98	45	20	76	95	153	127	178	27	99	180	129	29	49	31	14	5	17	24	31	5

207712_at	3	2	50	54	96	36	32	29	15	43	28	34	8	17	3	1	24	2	18	2	17
207713_s_at	630	1839	585	794	532	867	570	377	361	391	684	435	193	243	343	534	742	274	161	955	654
207714_s_at	1790	1826	2298	2718	1274	892	981	888	1409	846	3141	530	1808	2152	539	559	692	506	577	483	291
207715_at	52	57	76	53	81	22	182	63	64	96	172	74	25	3	21	5	51	32	38	2	27
207716_at	284	231	254	229	279	852	301	252	249	305	186	185	69	28	86	22	26	26	50	39	30
207717_s_at	419	557	1416	1024	1769	1429	380	426	1236	1737	1644	1656	1295	1591	1252	734	649	672	5735	1916	2750
207718_x_at	254	193	326	244	319	192	211	321	404	331	338	234	40	41	73	9	34	18	55	11	35
207719_x_at	668	449	1318	599	698	810	678	856	1132	1198	674	513	792	662	805	2010	1615	1929	2730	1804	2398
207720_at	34	16	34	35	122	13	30	70	91	67	16	8	4	3	9	5	3	3	6	7	13
207721_x_at	13789	13064	11703	13364	9580	9009	12578	12818	9153	8987	13820	12495	13664	19637	20647	16052	18444	16843	9209	11772	7850
207722_s_at	642	1338	507	1033	48	210	1083	969	531	764	497	64	66	72	56	140	159	162	172	89	64
207723_s_at	95	82	8	78	14	16	83	103	35	3	40	21	42	20	46	347	284	252	19	62	49
207724_s_at	225	151	339	220	296	247	196	274	353	393	419	346	131	116	165	200	146	172	292	247	437
207725_at	153	85	75	69	176	239	144	120	111	131	65	90	39	20	40	26	32	36	55	28	3
207726_at	30	19	11	31	44	31	21	25	19	14	9	28	3	9	3	3	5	4	6	5	3
207727_s_at	1340	1995	935	1081	643	732	1022	1613	541	977	524	699	353	399	453	762	743	864	871	888	690
207728_at	56	64	11	83	246	129	50	90	78	103	94	58	10	40	23	5	2	15	24	32	24
207729_at	41	41	38	120	23	32	66	82	21	99	55	73	2	17	3	9	18	4	4	9	3
207730_x_at	586	695	834	951	1181	1243	551	826	872	1135	996	1213	275	477	372	289	219	472	336	388	536
207731_at	99	16	19	79	89	110	95	94	13	17	37	18	10	3	18	2	10	4	4	12	3
207732_s_at	448	184	648	619	377	259	485	585	642	774	475	285	142	88	154	98	123	141	189	168	167
207733_x_at	253	151	300	330	542	980	266	302	359	253	485	200	257	253	279	12	47	8	48	81	31
207734_at	180	91	222	199	588	402	250	137	249	242	230	139	31	71	66	66	59	77	57	66	58
207735_at	332	190	133	193	320	280	307	359	217	211	145	108	74	73	63	163	162	113	130	172	96
207736_s_at	9	4	4	4	23	36	8	7	8	6	4	5	1	2	1	5	1	1	1	2	2
207737_at	3	30	9	61	40	31	34	17	25	42	7	4	20	10	5	3	4	14	6	2	3
207738_s_at	3457	2077	4193	2461	3117	3593	1889	1975	2454	2695	2263	1354	7101	5901	2689	5749	3697	5828	7742	7173	9294

207739_s_at	37	7	69	93	180	85	36	49	52	73	20	64	40	29	40	10	8	11	20	7	9
207740_s_at	599	682	954	576	351	78	943	834	1129	931	885	363	292	336	218	248	213	289	692	329	329
207741_x_at	103	153	153	307	33	27	221	240	149	173	99	141	52	8	25	5	5	7	11	6	3
207742_s_at	177	63	174	328	52	118	302	325	223	331	279	128	10	15	25	8	17	55	9	24	8
207743_at	17	26	38	31	21	60	27	41	31	22	30	16	8	7	4	5	9	7	4	7	5
207744_at	4	5	9	32	23	27	1	7	13	3	14	36	9	9	12	3	24	25	11	4	9
207745_at	13	10	104	97	16	112	8	11	9	10	11	34	8	24	27	2	6	12	13	29	2
207746_at	176	84	258	204	301	169	134	191	621	366	291	447	32	47	54	14	5	4	40	41	24
207747_s_at	146	139	368	720	352	285	293	238	439	637	244	99	32	195	64	73	81	57	111	104	76
207748_at	148	75	23	19	107	79	50	168	100	119	87	123	65	70	58	10	3	36	59	8	33
207749_s_at	205	296	397	533	535	625	489	570	534	450	550	667	139	161	179	128	147	115	57	94	72
207750_at	19	16	4	1	5	4	1	32	15	8	26	20	4	24	2	6	8	8	7	4	17
207751_at	152	69	122	131	188	262	115	176	106	137	132	141	45	31	21	3	12	8	27	26	2
207752_x_at	64	56	62	146	405	509	44	62	72	270	63	325	8	7	27	40	24	6	18	12	37
207753_at	224	215	23	50	125	71	228	227	16	11	21	40	12	1	23	187	191	155	1	6	13
207754_at	97	16	52	110	209	223	78	66	74	59	122	119	53	40	8	19	17	34	23	10	33
207755_at	118	73	134	208	458	481	172	168	140	180	139	123	29	13	52	39	29	9	74	34	7
207756_at	17	63	12	75	19	19	7	33	11	45	36	69	1	5	4	6	6	3	3	5	2
207757_at	17	9	14	18	16	16	12	9	11	7	1	10	2	2	2	11	11	15	3	4	5
207758_at	179	64	75	58	865	973	61	131	70	119	109	99	57	60	69	71	72	94	92	32	36
207759_s_at	206	62	174	141	153	235	80	99	147	190	114	77	26	7	5	5	19	25	5	24	6
207760_s_at	1431	2052	2016	2157	1223	1521	1617	1628	2558	3029	1585	765	1217	1119	1513	1492	1778	1241	2307	2241	1390
207761_s_at	247	109	159	1463	36	75	166	249	248	108	40	39	34	60	72	291	191	247	519	824	953
207762_at	21	11	5	32	115	56	78	11	8	85	17	24	2	20	2	12	16	2	4	3	2
207763_at	28	21	8	39	69	70	20	20	62	59	99	112	44	5	44	77	60	71	13	5	3
207764_s_at	138	63	247	190	56	109	182	238	245	302	229	158	17	8	32	23	17	35	32	19	52
207765_s_at	308	243	536	845	316	495	313	318	244	334	368	167	81	93	138	80	154	107	121	71	108

207766_at	15	19	9	9	27	19	12	12	9	24	10	6	2	3	4	3	23	1	18	14	29
207767_s_at	205	229	361	344	337	371	196	319	261	268	206	295	50	116	93	80	60	75	58	70	49
207768_at	11	2	9	8	22	23	5	11	11	4	9	9	76	64	93	16	24	50	8	8	25
207769_s_at	519	315	700	532	1201	1279	476	355	601	587	1167	949	2889	1912	1220	518	661	404	553	882	614
207770_x_at	120	190	80	182	279	384	223	268	199	319	371	333	50	83	40	43	31	60	54	49	15
207771_at	287	154	49	241	370	501	242	199	157	270	152	199	12	54	58	24	35	40	46	13	80
207772_s_at	32	6	4	63	92	52	9	54	88	56	55	13	2	6	5	6	2	4	2	1	4
207773_x_at	9	7	38	39	146	206	20	7	27	46	27	23	22	14	23	19	15	9	36	24	9
207774_at	167	86	156	191	311	292	215	199	144	184	269	106	23	22	63	41	41	40	21	49	33
207775_at	21	29	9	43	81	7	53	25	63	55	40	43	11	12	3	23	18	3	20	21	17
207776_s_at	99	89	102	116	38	89	74	144	118	137	77	123	8	3	6	22	39	16	24	24	23
207777_s_at	105	34	98	101	155	125	44	57	92	75	58	60	24	31	75	48	26	19	48	29	29
207778_at	34	23	20	32	18	11	34	22	39	36	20	58	2	6	5	3	3	3	7	2	2
207779_at	135	71	80	32	133	17	98	95	150	110	94	59	2	22	27	8	11	18	32	10	13
207780_at	151	64	132	103	180	106	186	100	126	119	78	127	11	26	21	24	17	23	23	21	11
207781_s_at	91	67	111	47	58	141	89	127	95	28	36	24	5	2	21	57	37	37	40	54	76
207782_s_at	525	585	735	731	520	638	745	881	549	1044	1228	1209	399	472	243	231	265	284	231	202	251
207783_x_at	33292	50659	33560	42177	25602	34100	35384	35856	30921	36054	30277	46388	23738	33778	39037	34271	39067	38552	21532	31617	26380
207784_at	16	12	38	23	785	704	7	36	31	18	13	11	4	43	6	10	13	8	11	6	2
207785_s_at	754	744	1042	852	579	626	1469	1310	735	865	703	680	600	640	659	1596	1280	1071	1152	832	1252
207786_at	131	63	119	166	113	44	175	143	128	148	161	134	67	64	58	61	95	88	97	182	107
207787_at	26	19	26	16	37	52	21	21	16	32	27	24	16	8	42	22	10	20	25	35	15
207788_s_at	488	630	100	176	507	495	814	565	12	101	659	266	62	35	85	107	111	108	85	39	55
207789_s_at	210	78	164	115	153	185	162	238	221	201	223	177	26	41	31	32	40	60	62	9	26
207790_at	103	78	138	118	397	395	146	169	170	184	102	69	18	8	15	3	17	4	32	20	13
207791_s_at	2779	2831	1941	1751	2206	2849	1671	1611	1757	1876	1727	2157	2688	1707	1234	1766	1881	1747	2003	1506	2128
207792_at	28	21	9	75	188	130	49	75	17	43	72	52	44	7	23	3	3	6	38	9	38

207793_s_at	50	63	56	62	7	74	76	98	51	66	40	24	20	10	6	2	23	26	8	2	4
207794_at	87	34	136	52	220	318	44	86	54	113	23	25	2	3	21	26	18	36	22	27	26
207795_s_at	128	33	178	197	236	39	182	156	169	155	148	118	10	41	45	19	27	23	10	27	4
207796_x_at	81	69	119	87	96	85	123	131	126	109	92	162	18	48	42	33	6	32	34	11	14
207797_s_at	71	84	12	14	21	19	140	133	56	21	18	84	25	36	27	3	23	19	4	2	2
207798_s_at	89	128	174	72	38	163	207	128	187	80	109	20	24	8	3	6	2	23	18	5	5
207799_x_at	15	13	12	23	245	175	12	11	17	20	20	13	21	6	13	5	45	7	67	22	50
207800_at	20	41	91	35	56	59	24	46	62	85	58	75	12	7	16	3	4	20	26	9	3
207801_s_at	1113	1287	1489	2384	2484	1165	674	822	634	966	1056	1027	1129	930	901	877	1155	779	1206	1316	1135
207802_at	3	2	14	5	22	19	3	8	1	4	23	5	4	11	3	13	4	2	6	2	2
207803_s_at	24	22	18	28	41	43	20	24	25	28	17	10	13	25	3	5	4	11	32	7	3
207804_s_at	102	71	56	69	48	238	11	37	107	55	82	127	2	29	5	2	5	3	3	6	17
207805_s_at	2388	2070	1606	1517	1328	1392	1558	1400	1441	1796	1703	1871	2199	2374	2098	2016	2105	1775	1769	1784	1149
207808_s_at	204	412	435	1164	114	87	356	322	357	646	94	49	56	18	19	241	194	234	1010	681	790
207809_s_at	2056	2114	3687	6045	1265	2380	1660	1529	3021	3796	2585	1144	1612	1529	2289	1902	2049	2007	2153	3601	2958
207810_at	50	30	41	12	73	117	48	67	32	55	23	16	12	2	13	12	7	17	2	1	2
207811_at	16	16	50	8	51	43	15	7	8	7	6	6	1	2	5	16	2	3	16	31	3
207812_s_at	2955	2513	2885	2387	3350	3029	2205	2518	2764	2478	3306	2395	3433	3462	4877	3581	4028	3268	3406	3710	4506
207813_s_at	1217	2113	321	347	366	257	1434	1506	351	507	365	457	380	458	160	353	454	416	235	167	121
207814_at	16	28	38	22	33	48	27	29	27	36	31	34	6	2	3	10	11	10	20	6	6
207815_at	24	11	18	25	12	23	1	9	31	20	3	3	17	20	7	14	14	15	6	7	1
207816_at	64	15	28	38	169	59	27	66	29	88	72	26	29	28	11	6	16	3	8	6	15
207817_at	13	15	14	28	29	16	19	21	16	17	74	21	5	43	29	3	6	4	13	10	4
207818_s_at	171	79	12	5	184	360	133	94	3	4	9	18	26	25	28	32	12	50	23	31	9
207819_s_at	17	11	20	40	25	44	12	8	56	14	227	70	114	281	148	6	2	4	12	24	11
207820_at	61	72	92	111	135	185	95	87	71	94	102	50	2	32	21	15	6	17	21	6	25
207821_s_at	1361	813	2389	2574	645	478	1041	1236	1830	2233	345	354	119	119	120	509	373	432	695	586	756

207822_at	94	75	128	103	155	134	94	79	56	110	114	63	28	20	68	31	29	37	32	7	21
207823_s_at	54	127	85	61	45	65	167	132	155	145	111	38	4	18	10	9	7	6	5	6	5
207824_s_at	429	327	410	563	33	31	391	271	412	472	510	18	20	8	28	58	41	12	60	57	3
207825_s_at	44	12	19	22	36	26	44	48	36	25	44	49	12	33	22	5	12	3	11	2	7
207826_s_at	1282	4224	33	16	355	109	1067	2355	25	24	31	28	45	9	74	2510	1802	2331	39	31	8
207827_x_at	134	118	110	106	232	277	129	182	147	99	134	124	89	35	17	17	114	58	81	21	17
207828_s_at	1752	356	1774	237	2730	22	1949	2468	1905	2302	2137	1398	1526	1470	1266	600	632	1254	1636	771	879
207829_s_at	423	180	138	109	329	206	306	269	122	84	401	160	360	206	182	272	321	201	220	91	88
207830_s_at	1421	772	986	816	1083	783	1534	1643	1199	963	945	602	1335	1244	1810	2781	2483	2593	1650	1101	1517
207831_x_at	671	998	358	345	253	269	612	586	230	414	173	258	791	762	631	1109	1117	1037	585	795	646
207832_at	13	11	18	10	29	40	9	13	25	10	11	16	31	9	11	41	91	7	27	2	10
207833_s_at	37	26	95	131	36	517	34	74	166	158	36	28	87	83	84	57	60	34	61	75	68
207834_at	143	106	144	109	139	189	138	115	218	155	139	136	5	8	7	3	10	29	22	35	30
207835_at	218	190	233	233	177	47	278	201	225	214	141	225	45	22	33	31	15	32	30	74	29
207836_s_at	181	311	176	263	115	125	109	158	222	159	141	92	55	3	37	149	136	92	323	348	256
207837_at	11	7	5	50	12	22	11	15	7	92	60	8	1	6	22	1	18	15	19	41	11
207838_x_at	19	22	19	203	19	89	13	34	12	43	11	11	5	3	4	4	3	3	4	10	5
207839_s_at	193	316	506	498	54	202	144	321	830	724	202	239	59	65	104	94	122	115	441	319	197
207840_at	85	113	53	124	238	356	33	150	106	105	117	132	26	9	15	1	25	2	17	21	30
207841_at	67	23	71	124	155	28	253	141	170	119	70	53	32	9	22	14	11	10	6	17	5
207842_s_at	962	1095	1081	1033	1087	1243	792	909	1333	973	917	1457	1019	926	827	744	694	810	880	613	842
207843_x_at	1610	2203	2736	4722	827	1367	1496	1383	3242	2948	652	598	616	226	568	881	802	925	3417	1923	1702
207844_at	73	137	164	44	411	534	37	91	84	88	145	23	37	32	58	66	38	85	78	39	48
207845_s_at	552	586	342	216	440	562	606	731	215	291	402	396	903	797	580	988	855	886	823	577	614
207846_at	45	13	3	14	12	8	3	29	3	22	43	1	1	8	23	1	23	29	1	1	7
207847_s_at	217	173	278	4308	360	740	232	203	310	162	328	234	40	44	57	43	39	38	109	285	142
207848_at	138	22	24	30	18	23	27	28	33	24	28	53	6	8	10	4	9	9	3	3	7

207849_at	32	10	49	25	41	16	33	7	28	31	26	20	7	4	6	5	2	4	1	17	3
207850_at	77	277	45	62	254	375	4	59	72	41	212	396	30	44	36	17	53	58	156	196	219
207851_s_at	128	101	75	178	445	495	215	194	136	141	61	11	23	55	41	66	55	61	48	37	62
207852_at	30	19	22	5	26	50	28	12	23	15	33	4	3	18	6	10	3	14	6	3	13
207853_s_at	30	15	31	23	49	44	30	24	17	36	91	82	11	9	10	5	12	6	10	12	6
207854_at	79	6	4	79	184	214	9	67	20	34	31	19	2	6	1	3	47	1	4	27	31
207855_s_at	164	105	277	149	102	207	73	170	198	208	74	113	42	45	45	84	111	119	557	323	343
207856_s_at	1434	904	1257	771	1416	710	1215	1254	1949	1282	1738	890	658	850	420	474	248	502	756	288	268
207857_at	20	46	18	44	146	176	36	70	44	7	34	26	17	27	21	2	2	6	22	24	29
207858_s_at	20	11	18	23	18	44	16	36	23	17	30	15	5	13	6	9	20	3	5	2	7
207859_s_at	165	89	151	238	191	95	250	202	202	196	149	232	24	26	41	22	7	4	34	42	33
207860_at	85	10	22	84	80	142	42	67	66	74	16	65	2	11	26	2	24	4	4	1	1
207861_at	21	30	30	30	51	22	28	21	37	56	23	33	8	7	9	3	11	4	8	12	7
207862_at	79	77	153	114	212	284	121	168	62	205	111	123	6	4	10	15	45	55	27	15	22
207863_at	26	18	39	39	58	39	93	38	20	42	51	30	2	3	18	35	8	10	29	14	29
207864_at	8	6	19	52	21	19	27	29	45	17	14	41	13	11	7	6	2	2	5	2	23
207865_s_at	142	66	64	115	154	103	160	128	102	98	104	143	29	16	31	17	35	42	13	15	30
207866_at	5	24	81	17	27	46	41	106	8	24	9	65	23	4	3	2	2	4	1	4	15
207867_at	87	24	52	1	106	120	91	107	74	112	37	35	11	22	9	4	20	19	3	7	13
207868_at	291	304	198	257	1359	1141	399	350	252	287	266	309	41	85	124	107	79	127	82	54	88
207869_s_at	52	19	16	14	140	56	29	29	5	3	26	9	12	19	18	38	25	25	22	1	9
207870_at	45	12	130	50	143	142	38	11	107	59	98	58	2	14	4	13	3	10	4	3	4
207871_s_at	204	215	414	503	602	540	184	218	396	389	539	501	714	861	1150	401	495	384	921	1112	1266
207872_s_at	91	29	133	36	379	261	87	82	112	134	98	98	22	34	33	31	7	31	32	7	28
207873_x_at	167	209	37	195	59	56	62	63	123	172	105	243	29	11	25	10	10	11	41	23	7
207874_s_at	21	13	27	149	85	42	20	20	20	43	17	65	2	21	6	13	2	5	62	36	44
207875_at	7	4	7	14	16	8	5	4	12	4	4	5	2	2	8	2	10	1	1	2	1

207876_s_at	591	1446	96	122	422	781	352	322	182	274	146
	256	129	132	68	335	323	262	29	66	18	
207877_s_at	208	32	42	74	29	19	176	36	36	25	41
	23	337	349	402	273	398	335	324	271	478	
207878_at	22	15	14	13	73	44	19	17	13	17	18
	14	13	11	3	6	12	7	9	4	3	
207879_at	19	4	23	14	7	17	27	25	7	41	16
	23	8	9	3	8	24	17	1	32	9	
207880_at	26	51	20	65	66	35	38	30	23	36	23
	54	2	4	7	8	14	6	6	5	3	
207881_at	62	50	37	63	140	79	56	48	33	95	38
	41	19	18	21	2	7	17	19	25	10	
207882_at	176	129	132	167	291	120	170	143	138	152	109
	161	57	85	73	38	68	74	76	92	34	
207883_s_at	152	124	34	154	348	333	106	104	82	115	71
	118	63	64	116	62	110	86	99	71	30	
207884_at	179	170	60	151	188	70	140	179	146	171	108
	118	45	29	47	49	56	30	45	30	51	
207885_at	21	12	18	6	49	77	17	15	16	7	14
	15	4	3	2	3	5	11	5	4	2	
207886_s_at	53	9	12	10	16	8	7	7	8	21	3
	13	2	7	1	36	31	7	4	1	3	
207887_s_at	111	113	90	83	285	433	30	117	206	171	135
	99	19	18	8	6	7	12	27	52	7	
207888_at	20	5	8	5	7	32	36	58	5	6	3
	34	2	31	8	3	1	1	1	1	5	
207889_at	38	6	42	16	23	137	61	30	15	64	48
	23	26	20	9	4	33	27	46	12	11	
207890_s_at	254	213	255	330	147	253	236	313	265	320	216
	210	35	73	67	50	69	55	76	81	67	
207891_s_at	249	119	332	158	444	173	306	193	709	753	421
	334	684	346	252	37	52	67	384	111	62	
207892_at	19	21	37	19	41	48	28	22	19	36	23
	16	11	14	10	3	10	11	5	7	9	
207893_at	8	19	47	87	33	46	130	71	44	91	41
	112	3	6	6	10	21	2	26	3	3	
207894_s_at	106	21	19	10	102	164	83	66	106	84	54
	93	5	7	19	8	8	4	7	15	29	
207895_at	9	7	5	10	25	19	8	11	7	13	9
	11	2	4	6	7	5	8	15	9	3	
207896_s_at	25	10	19	138	47	39	15	16	11	13	16
	10	3	7	4	4	10	6	6	10	8	
207897_at	220	131	205	265	355	257	219	187	300	208	189
	274	5	30	44	36	19	43	20	9	2	
207899_at	28	18	20	10	118	200	28	18	31	60	122
	16	72	40	5	26	7	23	21	32	28	
207900_at	20	13	18	27	286	224	12	71	39	25	10
	72	25	8	21	5	18	15	36	32	20	
207901_at	44	66	43	5	154	155	73	46	48	49	57
	117	16	6	45	15	11	16	24	16	13	
207902_at	180	85	110	114	121	258	30	67	50	56	34
	41	17	8	36	2	25	39	36	9	22	
207904_s_at	77	51	129	153	186	176	130	222	191	159	254
	206	86	55	93	48	33	49	58	62	99	

207906_at	8	7	8	10	12	22	9	11	11	8	9
	9	10	3	18	3	2	3	4	1	9	
207907_at	9	13	27	9	71	90	7	8	8	11	9
	6	3	3	2	2	3	3	2	2	1	
207908_at	276	258	180	231	485	492	371	367	285	310	328
	339	5	30	93	53	47	80	65	71	31	
207909_x_at	38	34	7	45	93	136	53	16	11	7	37
	8	17	3	3	23	7	5	9	14	4	
207910_at	101	41	84	17	154	17	60	66	70	35	102
	33	3	3	21	1	9	2	24	2	3	
207911_s_at	75	125	222	97	241	73	56	100	149	186	286
	165	32	77	19	49	115	74	63	15	63	
207912_s_at	4	4	5	34	5	8	3	1	20	1	3
	21	2	5	15	2	1	9	1	6	11	
207913_at	11	10	11	31	11	12	24	13	23	24	43
	11	28	6	4	5	3	17	6	4	1	
207914_x_at	89	27	88	70	93	109	40	71	59	55	78
	97	10	19	14	11	9	11	19	17	11	
207915_at	208	92	174	100	70	146	136	176	183	191	152
	185	21	13	55	17	10	34	32	11	11	
207916_at	83	26	99	122	81	180	65	18	17	34	67
	20	16	12	13	25	11	3	23	22	11	
207917_at	69	109	229	273	536	208	217	199	239	222	193
	155	107	86	74	55	44	58	117	114	54	
207918_s_at	7	4	5	4	10	17	11	8	7	8	4
	6	6	3	8	4	2	2	3	2	3	
207919_at	239	187	262	325	657	456	260	202	221	247	200
	153	74	55	45	66	43	22	39	48	44	
207920_x_at	232	164	184	142	302	235	232	375	154	141	293
	289	145	105	116	59	139	141	121	147	128	
207921_x_at	37	187	151	100	297	99	213	137	205	133	232
	273	48	20	33	28	3	25	23	2	7	
207922_s_at	3207	2777	1793	1637	1970	1461	2521	2780	1660	1771	1846
	1768	1431	665	1215	2108	2102	2035	1364	1468	1858	
207923_x_at	147	129	107	39	181	148	212	199	96	138	125
	240	27	3	2	6	21	8	8	20	2	
207924_x_at	26	62	19	21	40	52	110	38	23	31	95
	80	3	4	15	20	4	8	12	9	2	
207925_at	74	10	107	27	271	609	15	51	15	119	330
	147	178	196	128	2	41	25	8	5	3	
207926_at	41	9	14	66	11	23	48	108	55	87	43
	52	5	2	4	9	28	21	20	31	13	
207927_at	70	101	14	23	77	62	83	16	15	17	13
	78	8	10	3	5	13	8	15	5	9	
207928_s_at	20	2	4	8	21	30	3	17	0	20	1
	25	1	2	1	2	7	1	12	5	2	
207929_at	345	190	184	246	282	243	343	350	297	382	325
	180	25	53	42	8	46	54	8	18	9	
207930_at	86	105	81	140	272	271	130	63	154	99	135
	139	2	4	5	7	3	5	6	14	3	
207931_s_at	132	52	9	16	293	640	15	104	51	55	94
	25	4	30	47	72	165	35	12	27	3	
207932_at	7	30	3	3	78	39	9	4	9	6	4
	3	5	19	3	4	1	7	4	7	1	

207933_at	9	6	8	8	21	17	8	7	8	7	6	9	2	10	2	31	17	4	2	2	15
207934_at	5	2	3	8	7	4	1	26	3	6	6	6	1	11	6	2	9	15	15	2	6
207935_s_at	94	163	117	476	2470	2830	201	135	183	647	4049	1566	4415	2457	1595	67	94	31	141	42	8
207936_x_at	316	236	399	300	361	254	355	343	348	373	277	264	60	53	94	24	67	74	84	92	45
207937_x_at	442	276	312	239	580	472	598	640	405	328	648	440	138	170	84	65	73	61	89	66	65
207938_at	132	96	61	72	198	77	60	133	165	180	105	108	32	29	18	54	15	54	61	8	23
207939_x_at	2076	1980	1282	1417	794	644	1708	1316	2280	1354	935	891	537	362	557	797	967	476	1965	612	539
207940_x_at	85	39	26	87	56	20	54	46	123	56	151	38	4	9	6	4	3	6	18	22	27
207941_s_at	2922	2924	2950	3016	4546	5119	2018	2406	2186	2305	2619	2634	3774	3283	4592	5121	4101	4801	5563	5089	7440
207943_x_at	781	594	60	53	827	1248	317	636	54	61	306	499	375	349	562	475	323	414	51	75	69
207944_at	26	5	99	76	155	212	19	71	63	94	117	49	7	18	10	26	3	35	4	2	4
207945_s_at	1861	1901	1101	1331	911	1251	1782	1566	1010	1152	1532	787	634	641	734	929	1142	909	585	543	545
207946_at	101	79	168	216	250	155	221	161	138	203	175	202	23	55	28	17	6	9	18	52	31
207949_s_at	237	265	513	488	756	1360	315	256	364	278	283	240	277	323	408	234	320	247	479	942	1110
207950_s_at	147	125	144	292	34	114	155	96	136	141	30	31	4	6	8	41	29	22	64	80	74
207951_at	235	179	206	208	405	253	182	210	276	150	165	172	10	23	36	16	30	16	39	42	10
207952_at	33	34	9	49	22	20	76	29	9	7	21	59	13	13	7	5	5	20	5	17	4
207953_at	194	131	142	235	276	489	151	166	94	92	128	126	35	30	16	9	11	44	52	48	31
207954_at	290	96	402	275	641	790	318	226	359	439	325	352	60	50	57	33	82	32	92	46	65
207955_at	127	108	72	79	449	314	81	71	37	80	23	40	28	25	32	31	8	25	27	42	1
207956_x_at	574	349	826	781	1233	988	509	616	1106	1029	1391	945	1205	1157	1152	676	618	707	2542	1236	1646
207957_s_at	22	7	18	67	16	16	60	20	87	17	14	40	2	4	5	5	24	7	9	2	3
207958_at	42	15	7	87	210	165	57	44	35	48	37	70	8	33	52	50	28	16	24	19	24
207959_s_at	17	19	34	34	693	400	16	9	15	48	82	13	2	3	2	3	5	23	4	6	3
207960_at	464	187	326	322	370	300	404	384	407	510	334	342	44	70	73	65	59	59	95	104	48
207961_x_at	38	28	28	26	88	156	19	26	19	22	23	30	5	19	5	5	6	17	6	5	25
207962_at	102	100	31	72	137	179	37	42	132	88	82	20	4	19	22	16	4	7	50	10	17

207963_at	12	32	54	32	323	222	65	36	102	110	33	52	38	11	54	41	11	41	23	4	29
207964_x_at	151	140	187	138	257	227	170	177	174	212	156	171	2	36	39	15	18	5	32	6	6
207965_at	64	54	15	48	232	312	15	17	13	15	9	29	29	6	5	17	40	5	15	9	9
207966_s_at	1676	1740	1392	1424	553	802	2047	2346	1654	2503	1043	1116	780	775	381	1696	2146	1968	832	999	1204
207967_at	42	24	14	54	44	120	56	62	45	45	21	45	39	20	6	23	15	5	6	21	43
207968_s_at	70	90	77	56	92	15	178	230	20	29	61	20	5	1	14	51	46	39	28	28	3
207969_x_at	230	111	267	241	349	257	232	236	328	340	232	246	44	33	42	56	32	45	95	69	64
207971_s_at	217	246	176	274	304	204	261	319	219	240	323	338	130	143	53	100	64	80	59	72	47
207972_at	95	57	56	36	146	180	110	103	95	80	107	62	1	29	32	26	3	30	21	6	20
207973_x_at	238	116	271	269	438	296	201	255	292	305	210	168	26	55	42	69	66	56	62	57	36
207974_s_at	3285	3597	4326	4749	6409	6618	4982	5483	3322	4594	7126	10269	10157	11155	10328	7244	6289	6663	5475	4700	3745
207976_at	156	97	170	211	110	324	179	127	143	236	222	138	20	35	49	41	3	34	60	53	25
207977_s_at	17	13	22	12	41	344	34	38	23	22	10	21	5	32	33	41	8	5	39	33	23
207978_s_at	71	78	126	27	117	101	154	168	59	53	102	63	7	4	5	6	37	3	7	2	3
207979_s_at	78	122	42	167	456	341	133	210	162	182	433	178	209	166	624	23	10	21	21	22	4
207980_s_at	312	1064	90	184	356	402	443	556	95	129	162	372	138	66	95	177	296	273	101	177	193
207981_s_at	19	29	37	45	137	173	23	54	75	52	7	43	21	29	37	2	22	3	4	19	2
207982_at	19	17	20	63	59	275	21	69	20	31	97	23	4	7	14	10	34	12	6	9	5
207983_s_at	160	111	699	727	704	654	396	519	751	962	947	684	886	885	492	142	140	258	660	573	720
207984_s_at	627	465	327	308	366	396	676	656	293	423	544	323	90	65	86	206	140	184	111	35	33
207985_at	32	18	24	38	169	63	36	49	19	95	41	23	7	36	10	9	11	6	6	3	9
207986_x_at	1166	838	893	1041	531	687	931	821	929	780	689	478	1026	431	394	848	1192	1372	837	561	420
207987_s_at	15	32	14	3	15	77	27	30	9	4	6	8	2	8	8	10	28	4	16	2	9
207988_s_at	2663	2572	3696	3824	3813	4436	2757	2606	3944	3962	3522	3366	6932	6800	7135	4230	3716	3537	6034	5011	5029
207989_at	15	7	3	19	5	26	81	94	13	27	31	26	7	19	18	30	5	8	32	3	1
207990_x_at	291	173	255	277	344	282	260	203	322	361	216	206	28	66	81	97	44	62	86	49	49
207991_x_at	105	186	209	256	324	353	249	269	254	277	274	254	26	46	29	89	52	97	63	44	40

207992_s_at	462	586	266	219	902	1295	496	355	219	299	792	442	444	522	566	595	716	496	226	212	294
207993_s_at	290	472	472	964	188	222	819	834	737	843	483	264	97	66	117	301	358	376	224	269	282
207994_s_at	11	4	7	13	18	12	4	5	5	11	10	4	3	6	5	2	26	2	10	1	1
207995_s_at	147	23	148	80	199	65	187	42	162	197	139	112	52	34	108	45	66	31	46	27	32
207996_s_at	8	16	3	3	76	17	3	5	16	10	26	6	2	2	2	8	13	5	4	2	2
207998_s_at	91	145	102	216	63	50	156	182	118	250	168	194	10	15	15	12	16	16	9	25	8
207999_s_at	307	258	202	332	500	644	301	289	324	375	381	504	80	71	82	80	59	69	103	75	67
208000_at	250	198	304	190	272	316	199	290	300	291	262	244	32	25	46	34	6	25	18	21	9
208002_s_at	3229	1827	1205	231	2587	1242	2498	2256	1069	988	2501	2534	3316	3561	3740	4012	3987	3547	1923	1160	941
208003_s_at	167	148	96	225	254	245	265	264	199	189	242	230	201	143	101	86	76	80	50	72	76
208004_at	138	136	275	273	235	302	229	193	195	147	192	239	31	33	12	12	33	28	22	16	29
208005_at	78	91	137	115	221	34	94	117	100	123	102	126	19	32	7	16	10	49	34	11	17
208006_at	197	36	34	34	256	116	188	144	139	108	85	72	31	8	40	5	32	10	37	44	23
208007_at	40	117	84	39	34	113	64	84	86	157	124	64	5	2	21	28	3	3	8	7	6
208008_at	142	167	184	133	143	426	179	190	110	204	87	121	5	35	12	9	12	7	34	33	33
208009_s_at	529	654	547	488	579	539	847	604	915	704	503	319	48	28	131	142	128	50	176	230	145
208010_s_at	1	2	4	5	5	4	3	4	4	3	45	31	19	8	11	1	26	13	1	2	4
208011_at	56	28	11	19	18	5	4	12	3	50	23	59	18	13	15	8	3	4	10	2	2
208012_x_at	310	262	482	784	425	387	391	274	499	542	308	248	117	119	93	331	393	1401	645	1516	1808
208013_s_at	146	66	206	222	418	188	200	235	365	198	196	172	32	28	34	47	54	31	64	19	8
208014_x_at	194	157	111	136	129	280	146	165	122	116	139	111	25	39	50	61	42	39	34	37	7
208015_at	54	58	11	28	113	270	36	106	76	39	71	82	4	25	19	14	12	18	20	22	18
208016_s_at	86	10	79	21	148	79	64	49	21	21	80	68	1	24	17	2	12	16	1	4	5
208017_s_at	17	9	22	13	48	161	23	53	8	67	9	11	17	4	41	26	5	9	4	11	12
208018_s_at	148	24	94	27	186	352	152	92	108	133	67	94	4	16	18	36	8	3	22	32	29
208019_at	5	4	3	22	12	23	5	5	5	25	27	6	1	2	3	1	2	10	10	4	1
208020_s_at	20	10	24	16	302	251	21	73	17	20	30	19	26	7	6	19	14	42	32	39	5

208021_s_at	929	474	669	609	980	728	937	971	876	1037	701	726	786	666	618	945	940	859	1035	781	1085
208022_s_at	153	259	347	1284	257	383	244	253	392	446	252	248	336	312	145	64	45	65	310	320	383
208023_at	85	74	85	27	341	79	93	29	36	74	88	19	21	52	76	62	11	45	41	17	19
208024_s_at	1484	842	1487	1348	1063	981	1108	909	1081	1885	1258	870	1539	1376	1472	856	1120	860	1409	1253	759
208025_s_at	2445	1657	2112	716	1483	2169	2743	3567	871	692	1948	1445	1659	2741	2126	4357	5269	3997	1201	869	967
208026_at	46	21	80	74	120	148	49	66	64	35	98	69	11	1	9	8	1	15	18	24	3
208027_s_at	42	4	46	66	223	228	11	48	59	53	68	48	2	32	4	23	15	31	4	3	34
208028_s_at	97	11	22	23	197	188	32	16	25	43	21	65	32	52	7	6	17	5	5	6	4
208029_s_at	8136	8119	2082	1533	1550	1545	8326	7065	3731	2231	2624	1842	2374	1941	2498	8941	11298	10677	4195	2456	3803
208030_s_at	933	934	427	609	614	900	416	594	298	394	394	411	902	541	313	720	611	607	748	630	724
208031_s_at	157	64	87	159	91	118	93	174	44	144	169	111	51	65	34	48	7	7	75	11	21
208032_s_at	20	16	15	19	34	171	11	18	9	11	10	30	14	2	3	3	20	15	31	4	13
208033_s_at	136	381	376	396	197	95	205	189	173	284	694	329	45	53	72	48	17	56	34	51	81
208034_s_at	147	100	163	181	223	51	155	179	239	175	138	160	3	6	12	8	10	11	8	9	14
208035_at	241	274	377	347	346	325	411	306	460	495	313	329	39	32	68	63	33	47	88	39	48
208036_at	34	32	62	101	34	32	99	106	96	34	18	34	6	9	7	1	8	8	9	26	9
208037_s_at	344	198	164	263	493	630	167	202	357	207	196	268	4	37	11	29	26	50	79	66	42
208038_at	152	21	54	92	242	163	9	21	20	15	11	21	34	8	7	9	24	8	7	26	29
208039_at	173	192	202	178	390	321	248	214	214	236	196	238	60	34	38	23	35	15	32	31	43
208040_s_at	148	75	145	137	515	232	68	199	115	94	78	83	19	56	70	68	62	68	67	26	33
208041_at	94	86	84	127	30	28	54	103	13	119	95	153	10	33	7	8	3	25	8	9	3
208042_at	241	221	232	467	228	228	236	166	206	296	300	165	96	62	57	111	63	27	95	86	105
208043_at	19	11	20	17	82	58	16	8	16	15	17	35	5	13	14	5	5	17	4	28	8
208044_s_at	265	249	236	169	513	447	187	247	111	184	340	180	97	118	200	108	85	60	119	84	64
208045_at	17	5	54	17	150	40	74	37	59	56	53	43	18	13	14	3	15	2	9	14	13
208046_at	20	15	31	106	44	90	90	54	78	59	65	16	8	37	22	15	5	32	6	2	2
208047_s_at	191	68	269	197	243	263	82	127	213	259	195	240	157	144	89	37	46	47	421	205	402

208048_at	149	44	98	88	165	281	107	149	163	89	85	131	20	29	6	16	2	23	2	16	29
208049_s_at	15	5	5	16	104	24	12	32	16	39	10	85	4	22	3	4	5	2	6	7	2
208050_s_at	689	332	248	219	297	551	1016	1156	407	444	351	305	221	227	181	203	182	225	210	135	194
208051_s_at	1192	1466	1431	1539	649	832	888	1057	1092	1034	813	812	1149	811	988	2360	1830	1854	3224	2550	3051
208052_x_at	466	380	506	504	691	663	457	399	463	484	442	460	87	108	81	79	48	33	84	86	66
208053_at	21	13	19	18	27	15	13	26	21	17	20	20	5	7	7	2	3	4	8	2	13
208054_at	28	17	35	26	23	180	56	63	60	15	20	8	8	2	10	5	21	2	15	9	11
208055_s_at	45	33	156	186	109	169	197	208	112	122	121	102	80	52	34	57	41	55	80	44	67
208056_s_at	327	397	100	132	159	281	274	282	17	103	168	98	30	3	52	161	181	192	19	42	32
208057_s_at	127	100	41	136	297	536	125	127	67	129	146	73	20	55	4	34	47	49	34	31	33
208058_s_at	90	27	34	31	47	39	40	46	32	122	151	39	4	7	10	8	11	6	6	9	9
208059_at	126	64	49	168	113	27	152	153	163	110	82	134	2	6	39	18	2	6	5	4	42
208060_at	26	16	15	13	43	27	25	18	17	25	14	35	19	27	7	40	42	7	13	24	19
208061_at	19	52	18	52	34	34	7	63	27	10	3	8	4	4	8	3	23	36	6	2	22
208062_s_at	12	13	7	8	8	15	12	13	5	7	23	19	2	1	7	5	3	3	1	1	2
208063_s_at	22	23	60	1163	186	32	8	20	193	144	40	16	7	32	48	3	11	35	230	362	312
208064_s_at	180	105	88	89	433	456	29	116	183	50	36	85	39	14	53	12	37	33	35	22	13
208065_at	17	46	7	4	23	58	41	5	4	4	14	35	5	1	29	12	3	17	1	6	15
208066_s_at	999	869	1034	1070	970	1146	1423	1405	1114	1199	932	992	1160	1708	1547	2475	2833	2329	1243	1524	1689
208067_x_at	333	129	125	288	253	239	172	359	211	275	303	240	19	5	47	33	14	41	64	45	27
208068_x_at	30	24	30	36	493	355	34	33	27	27	31	49	27	38	89	76	42	42	22	59	41
208069_x_at	15	10	23	27	51	63	37	36	28	29	11	25	2	2	3	7	6	8	4	2	4
208070_s_at	445	805	818	1107	800	648	474	592	615	513	730	307	435	562	683	1024	1138	961	1267	1537	1926
208071_s_at	34	17	30	23	158	69	24	40	25	22	20	19	3	11	11	7	25	7	4	4	5
208072_s_at	508	935	678	670	441	560	647	338	732	1023	298	178	274	273	194	164	256	171	184	282	163
208073_x_at	3154	3530	3521	5366	4056	6592	2402	3050	3821	5200	3391	3455	3051	3934	5173	3531	3607	2376	4375	4406	6632
208074_s_at	4231	3475	3222	2634	2401	1442	4419	3283	4647	3631	4592	2364	6250	5326	3522	3505	3794	3802	4273	2712	1667

208075_s_at	98	17	45	61	210	313	56	62	87	68	85	68	22	38	13	21	7	13	15	6	9
208076_at	185	117	155	109	342	175	164	199	199	183	237	196	60	60	88	51	28	63	39	48	44
208077_at	62	47	122	57	190	97	139	103	183	219	132	215	26	14	18	1	3	8	25	16	11
208078_s_at	447	906	246	991	658	1181	428	458	401	333	890	912	411	435	269	319	304	316	417	358	342
208079_s_at	1598	198	1930	464	4822	496	1264	988	2526	1610	3898	3281	6104	9232	7072	2907	2867	2927	6443	2528	3271
208080_at	164	49	130	53	612	237	144	243	294	124	331	160	90	111	147	113	90	105	305	99	60
208081_s_at	50	13	12	50	23	11	16	54	15	11	68	44	20	19	29	16	7	13	11	12	25
208082_x_at	624	560	925	1423	969	1156	696	988	1029	1150	897	1204	497	531	502	290	295	691	555	927	818
208083_s_at	46	24	442	445	47	203	56	86	274	479	70	63	1	4	3	2	1	3	37	19	90
208084_at	66	71	342	270	40	26	66	33	231	328	41	29	3	7	22	3	20	2	35	6	62
208085_s_at	8	9	12	17	19	16	12	11	8	8	11	9	3	2	3	8	6	2	2	7	3
208086_s_at	54	57	113	91	69	146	13	83	35	61	20	18	2	20	2	2	25	4	5	3	5
208087_s_at	78	24	15	30	44	58	17	28	13	53	48	13	10	15	4	3	8	6	11	8	3
208088_s_at	3	2	4	4	8	7	3	3	7	4	4	4	1	2	2	1	5	1	1	7	1
208089_s_at	280	352	157	154	474	499	242	169	179	53	298	175	350	269	116	155	144	217	209	201	250
208090_s_at	36	23	109	178	89	59	37	110	40	18	28	79	69	55	56	31	54	9	36	29	29
208091_s_at	1070	1585	2257	3307	3568	3744	1693	1518	3281	2749	3870	3186	2025	2592	2942	1246	1271	1261	3629	2840	2768
208092_s_at	164	201	190	221	184	828	240	239	211	309	247	440	34	57	106	27	51	20	28	37	5
208093_s_at	1151	1268	785	630	1744	1451	681	619	676	840	1423	1470	1212	1086	1176	986	971	948	553	1032	966
208094_s_at	329	533	149	233	109	122	121	305	74	169	213	253	160	66	93	78	171	88	75	144	65
208095_s_at	2884	2535	2470	1430	1836	2439	3778	2609	2561	1945	1910	1345	4528	2930	2083	3868	3462	4815	4421	5344	5879
208096_s_at	58	27	88	154	73	110	34	55	64	84	20	9	22	9	17	29	7	25	140	286	199
208097_s_at	1749	963	1347	909	999	1000	2001	1599	2062	1856	1980	1880	1187	1305	1255	2095	1981	1907	1759	964	2515
208098_at	28	9	23	34	150	175	24	8	33	21	65	53	4	5	14	34	17	16	9	7	1
208099_x_at	208	78	174	160	293	185	146	123	198	218	168	207	86	70	74	14	5	23	57	57	67
208100_x_at	42	136	33	28	36	70	40	20	27	29	21	23	6	10	6	10	7	17	11	4	4
208101_s_at	1028	1000	1015	939	1284	1033	1040	761	1205	1065	1494	787	695	481	492	373	345	311	513	301	277

208102_s_at	53	55	68	22	12	145	24	111	11	52	28	34	42	8	30	3	15	23	13	31	9
208103_s_at	951	187	623	171	1016	468	1218	897	931	472	837	319	1016	488	587	639	552	769	1802	583	519
208104_s_at	257	285	349	458	613	538	248	223	340	180	397	132	171	175	96	120	141	89	170	54	56
208105_at	21	9	11	12	12	15	9	16	8	39	10	6	37	29	11	5	22	25	11	9	1
208106_x_at	308	252	354	352	401	689	245	274	277	321	381	341	206	173	210	90	78	86	111	88	80
208107_s_at	282	84	319	168	473	274	270	269	126	365	330	378	268	286	292	234	221	235	437	215	347
208108_s_at	26	23	19	26	22	28	17	25	27	15	34	26	8	8	6	13	11	9	13	15	4
208109_s_at	5	40	53	18	19	7	21	65	103	64	7	31	3	9	1	10	17	17	12	36	13
208110_x_at	40	56	50	75	81	74	56	78	59	42	40	46	6	7	15	135	14	11	17	11	6
208111_at	13	5	16	63	32	27	12	37	15	74	14	43	2	7	4	3	6	6	4	4	17
208112_x_at	419	375	499	647	679	447	330	252	741	792	1181	930	500	530	399	174	303	226	323	458	230
208113_x_at	21821	21853	12831	8944	8710	16226	10258	19986	7318	11620	9009	11347	15096	17956	16958	26000	26542	28417	17235	25478	21785
208114_s_at	874	386	1613	1234	521	482	1064	962	2181	1892	969	651	731	763	727	433	388	418	1499	1072	1011
208115_x_at	261	92	133	80	74	58	268	247	175	123	112	52	78	48	52	86	50	120	70	57	84
208116_s_at	489	426	64	75	239	214	1035	1697	87	143	200	152	60	63	64	399	334	370	40	49	72
208117_s_at	853	161	990	444	1924	423	851	722	1925	1500	1592	2183	1307	1047	539	212	303	262	775	501	331
208118_x_at	590	516	567	498	537	681	543	412	718	521	482	535	209	112	123	182	146	230	184	149	185
208119_s_at	33	131	26	80	71	20	24	33	78	165	128	145	54	51	97	27	23	23	53	44	20
208120_x_at	836	1377	1514	2377	827	696	1220	1597	2118	2267	1219	1028	182	244	275	191	151	150	217	292	267
208121_s_at	101	38	117	160	235	63	49	87	111	108	71	46	9	29	3	21	33	15	315	43	137
208122_x_at	26	17	37	38	47	46	36	36	35	34	30	23	4	6	10	6	8	4	9	4	5
208123_at	87	33	9	62	54	38	21	15	62	63	40	15	25	3	11	13	35	18	11	12	25
208124_s_at	291	281	273	317	499	531	398	633	219	382	340	407	60	88	94	78	92	127	42	8	28
208126_s_at	161	106	225	200	184	86	83	156	166	171	141	131	44	10	42	14	6	22	50	51	58
208127_s_at	410	401	255	317	449	833	317	342	254	328	652	631	743	916	824	960	875	667	637	483	647
208128_x_at	153	157	152	164	735	765	180	179	142	246	44	46	46	44	65	42	99	30	81	66	28
208129_x_at	216	27	176	159	158	218	91	95	66	95	156	189	76	76	123	69	74	59	81	127	79

208130_s_at	50	23	210	150	293	262	15	113	128	217	70	57	38	62	40	32	68	43	157	175	147
208131_s_at	56	11	19	80	38	46	58	42	40	66	75	26	29	15	17	51	31	15	33	29	38
208132_x_at	974	1347	758	793	447	543	867	1071	916	662	783	314	351	303	480	482	455	608	452	264	277
208133_at	20	9	5	4	27	43	12	11	11	10	30	8	1	6	5	28	2	7	6	14	25
208134_x_at	66	16	68	116	121	509	110	123	138	147	131	35	94	93	208	20	24	12	35	21	13
208135_at	54	24	96	737	33	43	170	58	199	249	77	31	4	6	11	3	8	4	21	85	50
208136_s_at	48	49	104	118	40	34	91	36	107	34	40	146	36	21	6	44	19	13	25	37	72
208137_x_at	359	347	533	463	1567	1102	351	405	419	724	560	870	298	285	361	163	190	278	364	344	249
208138_at	229	198	137	303	282	339	245	271	226	268	200	220	47	116	109	45	32	39	34	36	30
208139_s_at	17	52	26	31	36	70	69	102	52	70	80	21	25	11	10	2	14	23	22	2	9
208140_s_at	175	131	187	237	352	348	207	162	187	313	202	195	62	39	64	47	19	31	107	75	121
208141_s_at	544	433	442	296	1377	861	525	380	515	356	428	288	153	224	135	223	187	135	172	163	74
208142_at	7	4	7	6	3	12	1	12	3	3	3	8	7	17	5	5	3	3	1	10	1
208143_s_at	9	5	34	31	181	12	5	29	31	8	1	16	2	25	1	1	1	22	21	9	2
208144_s_at	99	40	28	103	125	28	95	104	122	88	51	138	35	20	52	44	42	15	48	26	33
208145_at	28	10	12	8	66	60	11	15	13	20	16	19	44	8	37	32	9	16	29	26	14
208146_s_at	413	561	39	10	29	27	380	450	13	25	17	77	34	82	127	505	545	451	126	172	159
208147_s_at	126	44	43	10	82	31	53	58	64	74	75	35	7	2	5	2	17	42	27	14	11
208148_at	9	4	4	39	11	22	4	8	5	6	3	104	14	2	2	3	2	2	1	26	8
208149_x_at	1128	973	1651	1225	1384	1295	1049	1042	1873	1417	1124	946	323	495	432	328	290	464	849	497	470
208151_x_at	284	243	179	397	115	192	223	271	135	278	249	317	95	77	168	65	77	55	93	82	41
208152_s_at	8805	5614	6531	2625	3443	3839	6930	6202	8941	7571	4194	2040	4617	4968	6579	8354	7018	6627	9946	10292	10922
208153_s_at	22	19	71	72	162	231	56	55	88	88	36	29	25	7	10	7	6	6	6	42	23
208154_at	13	33	95	91	158	65	118	53	144	96	153	54	23	4	27	17	33	13	27	29	20
208155_x_at	164	95	56	110	49	56	126	263	126	198	104	132	14	27	37	7	41	26	27	38	9
208156_x_at	733	1130	445	472	308	364	1125	1832	712	791	472	408	74	72	172	268	223	183	416	291	242
208157_at	20	12	5	9	18	17	12	12	16	13	10	10	3	3	13	3	2	2	2	2	2

208158_s_at	488	938	737	713	918	2248	407	625	579	931	881	1575	1894	1401	1287	1217	1237	1054	1931	1848	2196
208159_x_at	1482	1066	1464	1235	1372	1060	1212	1028	1880	1150	857	626	314	407	295	228	198	281	767	540	614
208160_at	24	17	19	26	45	23	24	21	25	28	23	21	7	7	9	23	10	12	7	7	5
208161_s_at	632	2306	2823	7422	532	1204	672	1149	2953	7813	947	1903	582	619	336	1246	1229	472	3184	4123	4340
208162_s_at	15	11	11	17	38	30	12	11	9	11	10	11	2	15	3	27	8	3	4	2	3
208163_s_at	144	279	170	269	34	31	294	205	173	219	109	94	25	20	22	5	31	12	43	7	13
208164_s_at	28	18	179	111	267	38	142	199	159	232	139	168	16	9	9	8	61	7	43	10	5
208165_s_at	335	353	225	238	341	583	299	321	154	162	210	226	175	104	223	333	474	331	130	446	620
208166_at	8	5	39	40	71	176	30	38	17	6	14	29	24	12	1	9	9	5	6	6	11
208167_s_at	83	29	39	27	194	164	97	42	58	22	45	8	18	9	21	43	25	3	3	2	3
208168_s_at	50	21	37	44	26	23	30	29	31	29	11	31	2	4	15	4	3	3	7	6	4
208169_s_at	15	4	60	120	25	50	30	74	15	80	23	85	5	1	31	2	4	2	4	22	17
208170_s_at	90	34	445	3461	176	71	111	125	803	457	87	99	13	13	3	1	6	11	287	474	560
208172_s_at	180	118	159	113	280	410	237	216	178	151	111	212	3	4	19	2	5	3	41	9	21
208173_at	13	9	18	75	33	32	9	11	9	8	10	16	1	6	5	5	4	22	4	1	3
208174_x_at	615	533	429	441	819	1005	545	542	455	451	658	587	299	336	394	178	213	117	132	282	146
208175_s_at	4	4	1	5	56	56	5	4	3	3	4	11	3	6	3	9	3	2	1	1	2
208176_at	180	29	30	88	454	110	16	24	33	29	21	31	34	13	36	16	13	21	8	18	19
208177_at	46	90	77	142	212	151	60	156	186	91	132	134	10	60	4	43	7	6	32	15	42
208178_x_at	390	618	758	790	221	133	371	430	705	770	715	462	116	80	50	86	66	152	144	128	100
208179_x_at	53	163	210	36	553	309	265	141	248	331	198	249	7	6	35	55	23	51	65	31	26
208180_s_at	79	28	94	150	351	484	62	79	119	91	418	749	245	113	76	5	5	13	25	76	29
208181_at	8	32	37	21	56	142	33	29	60	29	64	78	3	9	14	12	3	18	19	4	7
208182_x_at	153	102	206	158	285	214	171	174	162	196	189	89	19	25	17	31	3	23	43	22	41
208183_at	12	45	72	27	82	42	24	12	23	22	24	74	1	7	3	5	3	4	1	12	7
208184_s_at	470	347	370	310	840	667	416	362	341	443	512	322	287	161	159	163	178	211	202	124	124
208185_x_at	315	151	106	176	109	159	184	311	268	235	171	367	92	18	137	101	8	58	111	71	39

208186_s_at	151	55	141	263	54	15	50	45	377	414	138	79	26	49	10	57	70	53	123	77	60
208187_s_at	19	58	76	135	58	36	60	121	94	123	146	29	4	19	26	13	38	7	37	34	20
208188_at	177	116	172	285	425	430	58	73	124	203	135	137	46	55	82	50	70	22	59	74	51
208189_s_at	42	56	278	327	62	39	133	38	209	187	198	177	3	4	5	8	16	5	10	7	84
208190_s_at	2340	3782	3152	3414	921	738	2178	2462	2217	3184	2139	1377	1126	662	93	237	367	194	309	250	119
208191_x_at	501	314	311	296	867	798	400	442	439	397	1050	596	272	277	272	29	6	42	69	71	48
208192_at	8	29	5	62	78	95	19	71	9	29	33	40	1	8	3	10	3	7	1	26	5
208193_at	83	12	72	17	175	77	82	54	52	18	9	43	2	11	15	2	16	2	3	3	4
208194_s_at	134	35	41	8	129	38	136	103	75	133	101	96	61	96	76	54	3	59	46	36	66
208195_at	196	92	118	119	158	58	167	169	130	154	121	104	7	58	6	19	32	30	20	17	11
208196_x_at	57	142	27	69	158	173	138	154	20	45	38	118	55	19	25	85	12	9	15	29	11
208198_x_at	149	52	65	81	129	24	56	346	111	162	60	25	8	11	13	67	73	64	34	74	39
208199_s_at	94	44	15	91	355	187	87	115	66	67	63	74	65	89	69	90	74	96	123	90	108
208200_at	73	78	170	202	390	157	76	135	174	203	135	165	10	27	59	18	2	12	49	59	35
208201_at	22	39	34	6	78	94	19	32	64	60	16	16	2	22	2	3	6	45	20	2	5
208202_s_at	46	15	12	12	70	79	11	8	91	14	176	10	89	75	91	31	19	18	62	30	5
208203_x_at	90	26	53	62	162	132	48	48	64	53	26	63	16	13	10	9	6	9	10	8	13
208204_s_at	149	168	170	171	118	185	78	110	277	401	202	148	7	42	11	72	43	18	6	19	44
208205_at	193	172	206	141	272	98	195	107	159	176	203	148	28	42	49	52	51	32	45	4	55
208206_s_at	15	23	19	30	191	65	103	29	9	17	21	31	45	8	9	37	52	17	8	9	13
208207_at	73	9	18	14	40	27	17	44	44	11	11	8	2	3	3	2	26	4	1	5	2
208208_at	112	57	57	105	26	23	113	165	122	103	71	20	23	5	4	2	2	4	14	24	4
208209_s_at	13	7	71	239	84	83	21	30	239	91	21	9	20	11	7	8	6	7	220	262	134
208210_at	138	101	98	246	133	102	285	268	270	193	199	166	29	39	7	36	45	26	46	47	42
208211_s_at	33	78	65	85	201	56	115	30	37	32	200	170	46	94	66	22	61	33	7	12	36
208212_s_at	62	94	26	94	405	258	126	100	130	43	243	142	153	233	249	6	64	8	36	8	30
208213_s_at	99	68	128	160	131	13	20	12	98	38	78	122	43	8	13	30	8	29	15	6	32

208214_at	9	7	7	13	15	103	8	11	48	18	9	11	1	4	1	1	3	4	1	4	1
208215_x_at	13	17	5	69	15	11	44	15	7	11	9	11	5	2	3	2	5	4	3	5	1
208216_at	221	248	141	265	412	656	298	352	187	165	186	108	68	98	155	99	99	123	81	91	93
208217_at	228	127	213	270	366	360	200	289	284	323	262	155	42	6	50	26	21	28	81	46	45
208218_s_at	50	71	34	110	236	94	33	125	149	197	325	141	38	30	22	39	25	45	55	39	23
208219_at	20	16	33	17	40	36	23	15	16	21	97	65	22	41	56	3	6	29	6	6	21
208220_x_at	57	135	95	116	234	206	94	20	64	141	18	99	23	24	8	10	7	3	8	19	32
208221_s_at	29	33	113	43	315	325	107	120	25	126	111	136	90	136	52	62	47	21	18	44	25
208222_at	157	23	69	260	44	126	129	170	92	20	171	90	2	8	3	16	19	6	3	3	7
208223_s_at	103	107	16	106	76	42	113	73	68	147	338	171	16	13	33	6	4	3	9	24	3
208224_at	49	36	60	199	329	577	117	203	191	164	80	55	7	19	7	5	3	40	44	33	9
208225_at	12	6	7	10	16	23	9	13	7	7	9	13	20	49	10	3	3	14	8	6	2
208226_x_at	40	22	18	14	36	74	32	25	19	20	34	62	57	70	83	37	47	14	20	31	48
208227_x_at	40	162	80	102	154	122	265	139	182	256	314	227	64	67	131	57	60	75	56	71	43
208228_s_at	316	270	302	1245	766	1090	338	492	325	470	138	222	109	79	33	164	208	175	380	629	623
208229_at	112	175	107	105	33	140	155	214	150	152	125	21	53	141	151	240	143	212	186	162	88
208230_s_at	81	123	170	50	51	51	87	120	90	129	190	75	6	16	22	23	4	27	6	21	13
208231_at	90	47	27	91	52	211	62	80	11	45	58	98	19	14	19	18	24	20	14	37	36
208232_x_at	15	69	100	14	979	577	28	15	62	15	31	18	59	25	50	38	54	48	71	75	37
208233_at	61	23	60	26	18	12	73	55	3	11	33	26	11	27	18	19	23	22	32	17	6
208234_x_at	22	18	18	23	41	85	17	18	17	20	14	21	4	4	5	2	6	3	6	11	5
208235_x_at	78	73	66	38	26	30	110	98	110	212	57	94	7	3	45	2	33	7	6	35	11
208237_x_at	217	219	213	182	436	313	220	405	318	377	381	479	129	103	116	121	81	89	88	51	120
208238_x_at	929	708	932	1275	973	878	661	1028	866	938	958	1113	338	275	228	224	205	254	409	483	440
208239_at	9	13	11	8	12	19	8	9	8	13	4	3	4	33	3	3	5	2	2	41	15
208240_s_at	130	94	172	137	256	199	155	152	131	168	212	141	20	18	4	31	42	17	11	33	23
208241_at	185	135	163	195	175	191	246	212	182	228	159	146	12	20	25	8	2	51	44	44	20

208242_at	22	9	45	18	114	65	13	12	15	17	16	24	4	4	9	5	9	12	6	4	6
208243_s_at	16	12	16	17	43	13	13	15	20	21	18	14	3	3	9	1	8	6	4	2	1
208244_at	171	140	164	184	32	9	146	104	62	176	98	143	23	5	7	10	32	6	3	4	3
208245_at	36	4	39	34	29	9	12	4	7	43	16	35	2	1	2	15	2	9	3	6	15
208246_x_at	426	350	563	671	1688	1336	448	665	705	769	479	705	572	548	526	329	245	558	651	767	876
208247_at	71	10	87	39	389	151	106	154	79	82	28	113	12	28	32	33	37	26	19	43	25
208248_x_at	5424	5467	6313	8858	6310	8512	6285	7495	5447	7813	7463	7802	5747	7012	4332	3442	3311	2547	3523	3683	2949
208249_s_at	771	493	686	502	770	738	856	636	722	844	634	753	782	812	1079	1514	1244	1613	1238	927	1584
208250_s_at	294	206	194	348	484	284	355	404	226	470	378	364	66	19	66	79	58	86	74	111	98
208251_at	161	274	37	66	203	157	60	186	110	133	244	62	5	9	64	11	12	15	38	12	20
208252_s_at	25	30	56	48	51	27	15	22	54	22	30	26	24	12	19	37	36	9	46	32	29
208253_at	140	6	54	83	81	406	66	16	16	25	72	21	14	21	7	5	7	30	24	2	11
208254_at	17	44	117	59	95	196	20	12	12	49	48	62	7	18	36	31	9	5	23	7	19
208255_s_at	586	591	332	574	32	31	632	599	345	401	492	264	59	56	88	163	221	99	91	22	37
208256_at	26	36	31	16	43	19	90	28	80	41	74	13	9	13	16	8	10	9	11	7	16
208257_x_at	40	33	46	43	125	632	17	36	35	34	229	29	703	743	1217	7	6	9	6	9	3
208258_s_at	25	28	41	38	76	42	193	75	206	63	37	20	7	6	11	5	8	11	11	9	11
208259_x_at	153	146	148	160	73	134	89	157	203	164	126	170	10	19	10	24	18	13	32	47	23
208260_at	95	80	80	110	66	52	106	77	110	109	90	167	12	12	12	12	11	40	16	6	13
208261_x_at	98	54	98	210	175	26	138	153	205	131	166	156	29	33	46	41	24	43	71	80	13
208262_x_at	198	169	247	171	74	38	298	146	257	179	155	166	50	74	66	72	59	35	76	31	44
208263_at	99	74	75	54	170	180	118	88	63	82	98	103	12	20	12	16	15	18	19	20	21
208264_s_at	598	404	1245	447	620	681	462	371	1234	1125	624	408	613	603	611	652	660	667	2572	1511	2142
208265_at	30	19	15	45	29	87	36	63	63	21	51	54	19	10	2	17	29	2	8	1	4
208266_at	78	63	66	43	190	204	76	67	92	113	92	82	7	54	37	20	8	16	13	16	21
208267_at	160	172	197	140	249	231	134	123	215	159	168	187	45	35	58	52	58	18	53	33	13
208268_at	50	36	41	28	69	24	27	42	51	49	33	21	9	18	25	2	10	11	14	11	24

208269_s_at	25	21	144	31	275	51	20	34	31	22	21	25	7	8	12	4	14	9	10	12	6
208270_s_at	2991	2848	3196	4532	1806	1845	2308	2993	2778	4064	1408	1766	1142	994	950	1883	1848	1726	2837	2500	2411
208271_at	11	10	4	14	34	219	12	12	15	10	13	11	8	4	7	5	2	4	3	2	6
208272_at	74	41	60	74	66	110	61	51	32	60	27	60	7	64	5	10	65	37	33	33	47
208273_at	89	18	61	88	18	86	94	83	179	59	68	44	7	23	10	30	15	32	21	2	41
208274_at	120	140	141	125	414	423	186	170	209	126	124	108	34	61	71	27	72	46	43	27	30
208275_x_at	25	68	83	28	201	164	30	69	28	39	23	6	38	9	46	16	25	23	58	30	58
208276_at	131	52	121	115	285	188	130	129	161	151	102	103	32	61	56	64	67	50	113	61	64
208277_at	25	66	22	23	23	51	44	42	40	24	26	28	5	10	26	13	10	11	4	6	8
208278_s_at	13	5	12	6	12	23	9	5	5	7	6	18	4	7	6	3	3	4	15	4	4
208279_s_at	37	46	24	159	55	56	46	38	37	43	27	150	30	8	64	6	3	13	17	40	21
208280_at	149	62	45	116	136	97	114	100	103	71	50	108	23	37	15	51	35	59	15	12	9
208281_x_at	152	158	170	177	150	171	172	245	296	218	141	101	40	26	44	50	6	23	6	21	12
208282_x_at	12	7	18	12	234	124	56	12	60	36	24	14	4	2	26	3	33	18	34	17	2
208283_at	5	11	18	16	21	16	9	8	11	13	9	9	1	2	4	3	4	3	1	3	1
208284_x_at	405	530	311	398	603	695	405	314	377	317	293	309	82	60	55	135	132	146	183	162	174
208285_at	29	33	41	27	23	17	44	38	45	43	20	30	4	2	3	2	3	6	5	5	4
208286_x_at	26	52	24	212	78	117	62	62	202	133	95	30	7	14	40	47	20	28	46	46	11
208287_at	122	108	142	97	177	400	87	67	115	152	139	48	48	69	5	40	54	42	24	71	25
208288_at	17	7	18	6	26	16	44	11	19	39	6	10	4	4	8	21	3	2	7	2	14
208289_s_at	1700	1645	1129	873	1567	1458	1408	1288	1212	1111	989	662	1576	833	1039	2319	2497	2470	3364	2518	2037
208290_s_at	1650	1244	1849	1120	1850	2568	2372	1983	1659	1497	2879	2094	2141	2307	3627	2553	1957	3289	1833	1570	2659
208291_s_at	30	10	18	12	293	333	7	15	20	7	165	16	223	122	267	10	19	11	36	26	3
208292_at	176	140	186	131	41	130	252	169	155	203	125	202	5	28	7	18	6	31	14	14	29
208293_x_at	21	16	18	21	82	40	24	17	15	18	16	20	4	5	20	5	5	15	6	4	3
208294_x_at	17	19	18	17	59	128	20	9	15	17	10	15	2	5	9	3	1	14	4	4	5
208295_x_at	112	18	12	16	51	54	17	18	3	11	13	15	2	9	19	3	29	35	6	2	1

208296_x_at	529	516	1670	1637	224	344	633	722	985	2253	213	263	203	173	115	465	468	424	2671	1091	1625
208297_s_at	74	84	113	129	148	181	101	137	84	148	138	109	53	52	13	10	25	21	32	16	21
208298_at	94	18	19	116	32	26	17	17	102	109	68	113	1	8	2	4	2	6	6	6	6
208299_at	118	119	155	162	847	649	148	132	110	124	125	118	19	8	90	79	83	107	100	49	41
208300_at	183	147	507	770	137	181	103	168	202	477	168	72	13	11	20	48	57	45	243	341	325
208301_at	93	128	113	44	241	304	49	96	130	87	27	108	51	53	49	67	13	27	64	40	7
208302_at	106	91	121	125	264	208	107	119	86	157	107	111	25	36	28	15	35	27	19	26	21
208303_s_at	36	89	20	124	469	370	32	45	82	56	21	33	5	4	9	3	3	3	8	5	6
208304_at	60	10	9	25	236	173	45	83	126	131	70	50	6	43	21	3	11	6	10	4	26
208305_at	15	5	14	18	7	15	8	7	7	8	9	5	1	5	11	3	1	4	2	1	1
208306_x_at	370	488	193	387	236	421	578	504	223	300	259	284	35	21	68	108	108	75	36	32	35
208307_at	19	50	19	88	71	85	52	90	28	35	53	45	2	4	10	5	6	6	4	5	3
208308_s_at	10621	5181	11695	7289	6940	5694	8260	6195	10848	7065	8887	4718	5985	6705	8853	6979	7980	7838	6457	5746	5222
208309_s_at	1220	470	269	392	575	98	1398	856	512	335	1096	511	508	610	879	1936	2044	1936	510	293	507
208310_s_at	1443	1120	1833	1310	1714	1700	1651	1595	1592	1793	1886	1937	3328	1325	1414	1211	841	873	2127	1803	2522
208311_at	32	22	53	34	58	73	52	131	72	35	27	26	3	9	3	5	4	29	3	5	6
208312_s_at	37	39	52	58	66	71	40	41	70	92	81	40	2	6	6	5	6	6	6	3	3
208313_s_at	4749	3748	3557	2121	2010	1357	5097	4318	4941	3901	3891	2047	2268	1849	2319	2284	2425	2145	2534	1432	1792
208314_at	164	47	175	202	216	207	136	125	154	162	121	165	6	3	56	43	16	26	80	41	19
208315_x_at	365	408	302	238	671	722	417	434	359	376	603	475	214	248	262	202	179	265	207	147	127
208316_s_at	463	364	902	1313	1060	759	762	780	1082	1473	1317	1022	234	155	152	72	53	79	128	148	169
208317_at	13	6	8	5	25	16	13	4	11	11	9	11	2	1	26	3	2	2	2	18	4
208318_s_at	13	12	18	10	19	19	19	13	13	14	11	19	28	12	10	10	27	7	67	13	5
208319_s_at	2753	794	2191	1028	2282	2434	1990	2189	2444	2778	2442	2810	4282	2574	1952	2370	2595	1516	5791	3572	3903
208320_at	15	17	46	89	70	97	16	15	99	11	9	8	3	6	7	3	7	4	9	3	3
208321_s_at	13	15	117	151	111	202	16	25	329	376	18	14	18	12	29	34	26	24	122	68	70
208322_s_at	57	33	951	287	267	450	12	15	987	1393	108	11	84	96	78	170	119	108	1937	1712	2071

208323_s_at	155	26	133	349	67	32	19	22	241	210	44
	58	21	4	41	44	10	9	148	607	282	
208324_at	60	52	62	38	143	130	37	115	47	102	112
	88	19	19	23	33	26	21	27	17	23	
208325_s_at	175	172	396	344	55	91	219	300	330	314	92
	128	42	56	22	13	7	17	62	51	58	
208327_at	38	12	75	110	30	42	15	38	23	24	105
	11	5	9	7	33	19	8	3	7	2	
208328_s_at	148	86	240	142	129	165	156	127	56	240	75
	180	107	125	53	62	95	92	182	158	190	
208329_at	13	9	5	18	89	185	13	18	11	10	13
	13	4	9	3	11	3	3	18	2	2	
208330_at	28	22	28	106	26	39	40	36	39	36	14
	89	13	8	13	5	17	13	7	4	21	
208331_at	9	7	8	8	48	83	7	17	8	6	6
	8	2	24	9	4	2	16	1	3	12	
208332_at	226	186	151	147	287	384	212	181	159	215	162
	139	45	44	38	78	39	63	54	9	53	
208333_at	19	13	12	31	21	19	20	21	48	32	18
	14	5	4	5	5	6	6	8	7	3	
208334_at	143	92	92	118	312	112	107	156	110	134	132
	148	10	12	6	14	5	10	29	4	29	
208335_s_at	16	21	23	19	59	59	25	13	13	32	23
	25	3	8	10	9	11	8	4	9	6	
208336_s_at	2215	664	1541	1855	1550	1095	1611	1755	1442	1789	1929
	1492	2649	2377	2424	2652	2646	2160	2029	1870	1192	
208337_s_at	147	72	148	151	179	313	133	185	186	148	90
	84	17	19	2	29	24	28	39	3	53	
208338_at	28	33	20	45	329	187	23	28	84	18	16
	13	16	19	25	7	25	11	15	13	12	
208339_at	48	49	54	8	16	73	57	34	24	34	71
	35	19	13	16	17	9	14	29	31	18	
208340_at	7	9	7	18	16	59	9	66	21	7	47
	18	16	9	33	4	20	5	3	1	4	
208341_x_at	33	19	23	23	37	43	24	22	25	29	24
	45	5	8	9	3	2	4	4	12	4	
208342_x_at	26	15	19	21	40	67	24	25	20	22	18
	23	4	10	2	8	5	2	5	3	3	
208343_s_at	7	7	22	26	59	69	4	4	4	39	37
	18	10	1	6	40	55	75	17	23	45	
208344_x_at	116	64	113	100	48	98	193	194	222	48	185
	185	10	7	19	7	2	15	6	6	3	
208345_s_at	20	83	68	93	21	16	38	113	29	84	63
	8	27	39	23	20	10	27	27	43	19	
208346_at	44	7	30	22	25	12	50	17	29	38	33
	13	3	20	24	5	3	5	4	9	6	
208347_at	213	40	103	222	43	60	86	95	37	81	33
	206	104	97	57	67	70	29	79	41	55	
208348_s_at	48	6	9	26	84	95	9	58	7	73	27
	40	7	13	2	27	2	17	3	13	30	
208349_at	167	170	152	180	184	175	147	154	130	164	138
	160	2	24	5	10	23	13	50	9	29	
208350_at	12	55	11	66	58	26	60	29	15	10	61
	54	26	2	18	2	35	22	14	9	16	

208351_s_at	441	394	387	336	447	531	725	833	706	558	864
	648	171	199	179	136	94	106	133	150	109	
208352_x_at	30	60	71	59	21	24	29	17	92	38	99
	29	21	6	6	37	46	37	18	26	4	
208353_x_at	98	78	76	98	276	255	135	120	170	117	108
	80	32	16	8	58	25	32	40	63	49	
208354_s_at	9	9	20	17	19	32	20	12	13	15	10
	21	2	13	4	2	7	4	4	2	3	
208356_x_at	13	10	9	12	34	55	15	12	8	13	9
	15	2	4	7	2	3	2	2	5	1	
208357_x_at	17	11	11	9	19	110	13	13	9	11	10
	20	6	15	7	2	2	2	2	3	1	
208358_s_at	205	204	368	360	322	310	407	445	353	491	335
	278	225	145	166	161	183	136	355	167	229	
208359_s_at	41	19	5	54	253	904	28	100	23	36	539
	505	335	228	169	34	6	13	23	5	13	
208360_s_at	189	175	175	256	537	595	302	284	310	334	254
	240	28	27	19	39	30	51	36	12	35	
208361_s_at	624	286	359	149	286	173	378	302	210	284	205
	172	181	133	110	155	240	194	121	137	124	
208363_s_at	151	200	144	102	175	395	239	197	147	154	247
	206	69	108	57	67	33	64	48	24	25	
208364_at	108	103	115	222	51	43	216	115	13	168	151
	157	32	13	29	4	31	2	11	28	4	
208365_s_at	110	33	126	57	126	12	36	83	62	15	80
	98	28	42	44	34	22	40	2	34	19	
208366_at	41	66	46	80	96	134	111	78	104	119	87
	94	3	54	14	10	14	25	5	34	19	
208367_x_at	20	13	20	21	18	13	87	21	45	25	17
	11	2	4	5	2	3	6	4	3	2	
208368_s_at	53	21	114	4	525	24	80	65	144	175	165
	128	130	88	62	72	42	23	135	54	90	
208369_s_at	386	443	576	318	403	366	404	256	385	500	318
	263	234	238	172	163	297	273	217	166	167	
208370_s_at	487	662	571	775	1269	2240	549	309	369	194	671
	325	803	697	1314	583	745	508	549	792	1018	
208371_s_at	722	1304	449	923	470	1118	820	1033	459	544	645
	669	423	319	162	345	465	349	261	247	158	
208372_s_at	205	237	155	163	418	559	240	91	123	151	465
	202	48	41	73	46	36	55	66	41	34	
208373_s_at	86	180	182	84	67	66	211	119	182	171	121
	187	10	2	6	4	2	3	4	7	10	
208374_s_at	3227	3500	3603	4768	4441	4431	4082	4014	4036	3672	4085
	3645	7460	6102	9129	9248	8970	10453	13156	8195	11194	
208375_at	9	4	46	13	12	16	25	42	67	11	10
	13	16	1	3	5	3	4	3	4	3	
208376_at	24	82	194	120	169	132	57	83	16	85	117
	89	43	6	21	27	11	13	32	56	30	
208377_s_at	34	26	33	38	67	245	30	33	36	34	28
	33	4	4	5	3	5	3	5	5	4	
208378_x_at	16	10	46	26	30	85	87	17	25	18	54
	93	4	3	7	3	5	10	14	3	12	
208379_x_at	93	12	193	101	205	161	58	113	115	143	134
	116	19	12	13	14	39	6	16	29	26	

208380_at	5	11	9	6	15	13	7	8	5	8	7	5	1	2	2	3	3	2	2	2	1
208381_s_at	42	32	11	27	32	69	156	148	27	31	37	20	31	65	78	56	61	66	81	46	79
208382_s_at	122	55	194	114	43	35	114	152	107	176	192	191	46	6	35	27	18	7	17	9	26
208383_s_at	17	4	3	12	14	78	3	38	4	7	7	5	2	7	1	1	3	17	27	16	13
208384_s_at	578	380	403	574	572	826	591	684	571	594	621	363	224	337	420	257	219	260	71	139	104
208385_at	11	6	7	6	87	215	19	9	8	8	28	36	46	7	5	8	2	8	11	22	11
208386_x_at	33	23	47	58	70	129	11	33	33	14	34	44	19	42	50	15	27	16	19	19	26
208387_s_at	135	97	134	182	25	117	87	33	209	120	138	35	13	35	8	17	6	39	23	44	26
208388_at	69	84	83	62	126	171	50	77	151	96	65	49	39	3	19	26	37	17	49	31	37
208389_s_at	114	108	83	168	213	130	78	124	103	29	141	156	75	113	41	19	22	16	6	5	11
208390_s_at	377	283	246	138	60	129	416	385	272	358	310	366	65	20	14	19	117	111	29	16	56
208391_s_at	26	30	18	19	54	73	30	30	15	25	13	18	7	2	9	2	7	6	4	4	5
208392_x_at	33	7	33	22	51	82	9	40	41	38	7	33	20	11	10	82	61	258	73	121	180
208393_s_at	934	757	1222	627	973	591	1047	1002	888	964	580	230	302	192	476	864	899	980	1218	1236	1503
208394_x_at	65	29	53	19	525	148	23	8	67	43	173	268	172	180	196	1	24	29	20	23	25
208395_s_at	139	12	23	116	205	73	142	177	118	126	138	113	41	15	39	28	41	27	22	22	18
208396_s_at	54	6	87	16	14	19	69	92	58	80	47	96	1	4	6	2	19	21	3	3	11
208397_x_at	37	32	8	48	102	85	30	37	70	60	109	64	9	8	17	8	5	6	6	6	9
208398_s_at	1158	962	1333	1046	1019	918	674	661	850	850	824	554	817	824	1079	1579	1731	2065	2250	2644	2930
208399_s_at	15	71	61	65	25	62	21	59	124	8	202	92	101	139	132	3	2	7	4	3	4
208400_at	75	56	53	69	157	151	23	9	51	29	132	19	4	3	7	16	12	7	17	6	3
208401_s_at	54	6	11	74	265	212	16	15	33	68	13	10	13	8	12	5	56	18	58	19	62
208402_at	16	9	102	22	30	108	130	33	17	82	85	69	4	22	4	16	4	4	3	16	11
208403_x_at	194	316	277	221	269	349	374	373	130	215	436	315	165	198	156	169	109	124	88	77	98
208404_x_at	93	128	80	62	118	277	199	194	138	102	298	185	88	200	237	52	81	72	13	38	9
208405_s_at	4183	3999	4730	9705	3611	4941	2627	3116	3119	4059	3320	3431	4239	5186	4557	6313	5627	5621	7694	9130	10966
208406_s_at	13	17	9	16	81	31	15	9	16	17	18	13	5	7	18	5	6	4	9	6	2

208407_s_at	700	1460	1915	4779	750	1223	983	1301	1790	2724	739
	1092	944	709	706	1588	1293	2113	4909	3894	6180	
208408_at	205	50	152	111	318	198	265	115	71	129	124
	65	4	19	13	15	16	21	8	21	13	
208409_at	22	21	114	103	22	36	29	67	31	34	145
	23	23	27	2	5	25	18	18	11	3	
208410_x_at	102	83	104	96	247	269	70	107	67	68	64
	89	39	33	30	28	30	22	24	34	30	
208411_x_at	9	6	14	8	19	26	42	5	8	7	7
	11	3	3	2	1	3	3	4	4	9	
208412_s_at	11	12	34	10	372	183	8	13	9	27	21
	59	22	55	44	27	12	6	60	9	4	
208413_at	13	17	16	21	22	26	12	20	9	20	24
	13	2	3	5	2	6	3	6	2	4	
208414_s_at	107	109	194	356	482	200	77	77	143	219	166
	319	79	120	77	19	28	44	129	9	38	
208415_x_at	1311	1305	1087	905	1042	610	961	1510	919	1836	1691
	1185	1117	841	948	1920	1322	1730	1327	2073	2039	
208416_s_at	52	86	22	114	282	247	155	208	157	236	267
	183	158	161	239	6	16	74	21	7	6	
208417_at	143	33	81	79	543	691	41	136	40	173	33
	29	78	48	76	62	48	147	107	40	80	
208420_x_at	660	511	804	561	672	392	822	661	660	819	1184
	532	261	258	228	170	175	190	151	124	115	
208421_at	131	32	73	45	195	230	53	95	52	50	119
	108	35	22	30	29	10	17	12	22	5	
208422_at	12	57	42	36	199	220	42	25	71	35	64
	62	4	11	19	24	2	20	18	15	9	
208423_s_at	32	67	65	101	100	60	12	71	84	55	98
	77	2	8	17	19	2	17	29	10	13	
208424_s_at	728	508	392	308	665	383	778	596	550	495	692
	451	1142	1117	1127	1658	1478	1469	1259	787	918	
208425_s_at	97	127	14	17	41	241	109	141	12	13	64
	21	4	4	5	2	2	12	2	3	2	
208426_x_at	173	57	130	156	201	31	175	117	174	208	129
	89	22	16	26	8	28	2	41	22	23	
208427_s_at	13	94	18	81	107	82	58	28	8	11	14
	44	10	2	23	33	17	20	46	29	15	
208428_at	40	71	18	10	113	140	28	4	11	10	54
	53	2	19	68	3	25	37	18	34	23	
208429_x_at	242	84	359	326	87	69	258	371	359	535	395
	219	37	61	77	51	20	45	111	85	56	
208430_s_at	156	73	186	119	60	35	114	158	135	162	99
	118	7	24	18	27	14	6	19	13	32	
208431_s_at	116	77	103	109	104	22	123	140	84	204	128
	148	38	48	24	12	41	15	46	42	34	
208432_s_at	101	18	26	35	23	60	49	38	62	35	26
	93	7	8	5	5	10	9	8	6	26	
208433_s_at	611	422	2854	1734	357	387	845	689	3398	5943	711
	415	436	572	493	234	256	239	1930	947	764	
208434_at	16	10	12	14	12	8	13	73	31	21	9
	13	18	4	3	3	1	3	8	4	3	
208435_s_at	16	13	16	17	23	28	19	12	21	36	17
	18	4	34	6	5	7	2	5	4	1	

208436_s_at	168	344	415	388	619	943	121	334	209	376	499	784	327	296	372	175	274	755	283	659	476
208437_at	26	35	84	106	38	23	56	50	153	89	47	73	47	8	11	10	5	6	26	36	39
208438_s_at	36	86	69	85	43	75	34	30	32	38	87	30	7	16	53	15	30	3	43	8	25
208439_s_at	229	164	175	347	135	54	237	331	239	293	215	53	6	16	69	69	6	63	40	62	25
208440_at	25	10	14	21	40	34	20	15	21	18	9	18	13	8	42	6	45	5	4	2	7
208441_at	86	112	103	124	338	277	113	153	123	122	145	126	19	46	55	50	29	33	55	43	27
208442_s_at	229	196	380	326	427	399	159	230	248	690	469	416	130	146	200	110	63	80	275	466	448
208443_x_at	48	97	16	28	128	27	133	106	27	25	41	21	32	48	26	111	83	85	41	6	3
208445_s_at	1498	718	1310	597	2518	1656	1458	1248	1208	1066	1612	1606	1779	1608	1678	1455	1488	1311	1037	672	616
208446_s_at	173	116	88	107	293	280	231	187	108	208	210	161	8	50	40	33	17	8	27	47	27
208447_s_at	1208	262	1313	754	1391	1189	1265	1227	2410	1796	1850	1253	1529	1418	1016	722	785	818	1180	469	697
208448_x_at	123	77	137	84	169	110	89	135	116	120	94	106	23	27	20	16	15	19	26	41	31
208449_s_at	13	6	7	3	21	36	7	12	11	10	4	4	4	3	17	27	3	11	2	6	3
208450_at	41	11	15	19	33	27	19	21	15	126	53	10	8	9	10	9	5	13	62	4	2
208451_s_at	77	78	142	241	26	23	58	67	244	150	121	101	44	36	37	21	50	7	22	3	7
208452_x_at	364	375	467	501	484	246	408	401	471	419	397	349	50	27	42	48	37	12	49	47	21
208453_s_at	811	363	1466	1309	308	195	1842	1624	1783	1477	780	294	281	289	305	769	771	981	1080	825	1354
208454_s_at	12	97	19	10	250	372	28	12	12	15	9	38	11	3	10	5	28	52	16	7	19
208455_at	74	71	61	52	74	156	45	92	80	80	107	38	13	34	9	21	16	25	9	32	5
208456_s_at	643	741	1104	854	711	1058	729	711	998	1209	988	770	956	635	400	475	379	304	959	623	630
208457_at	155	86	15	61	117	62	313	143	28	52	18	123	2	4	5	5	6	8	5	6	5
208458_at	128	29	50	106	80	180	163	107	144	144	131	63	4	4	1	16	7	4	5	16	23
208459_s_at	814	375	1019	903	1149	755	1308	1337	1231	1275	1539	1483	205	169	216	114	101	68	181	158	141
208460_at	314	193	69	111	208	199	231	268	116	180	465	266	144	75	36	79	57	88	27	51	1
208461_at	8	7	7	26	18	20	7	4	8	8	10	10	1	7	5	2	10	8	4	3	2
208462_s_at	205	79	153	171	271	149	102	169	159	168	153	151	3	15	52	38	24	28	43	37	27
208463_at	171	117	220	215	252	151	136	96	161	190	152	196	29	39	27	5	29	34	76	41	43

208464_at	213	55	189	125	76	172	152	210	88	182	27	197	17	3	25	7	7	30	16	10	54
208465_at	198	21	24	26	96	112	66	24	44	21	54	77	8	45	5	5	5	5	6	29	9
208466_at	151	180	240	274	224	113	147	172	193	182	105	48	31	30	29	37	12	17	26	33	43
208467_at	22	10	12	56	26	34	68	67	70	80	102	19	56	46	56	1	14	3	32	7	72
208468_at	172	35	33	175	30	27	52	141	178	243	78	212	5	4	5	3	2	1	4	9	1
208469_s_at	70	179	96	217	29	44	129	174	28	21	20	123	32	39	26	10	34	36	29	9	9
208470_s_at	20	19	18	16	49	48	11	15	21	29	28	29	5	34	5	15	7	35	11	7	18
208471_at	93	74	94	18	29	239	46	8	20	17	9	28	35	9	8	2	3	17	4	4	10
208472_at	140	107	126	154	425	523	139	183	114	183	121	156	17	73	59	39	105	79	46	48	30
208473_s_at	273	150	66	203	71	254	228	185	92	180	196	255	25	8	5	3	3	2	3	2	19
208474_at	173	106	190	182	330	266	155	311	173	151	243	408	34	64	25	48	47	18	25	97	48
208475_at	9	10	85	96	23	23	13	29	5	15	26	10	5	9	5	4	12	20	5	2	2
208476_s_at	77	46	31	17	29	36	60	55	51	15	80	108	20	3	6	16	11	27	4	13	17
208477_at	22	27	75	94	43	35	105	116	39	103	70	171	24	5	14	30	16	11	17	7	4
208478_s_at	329	366	422	314	333	269	311	216	443	716	514	408	770	414	211	198	184	155	230	126	74
208479_at	82	22	76	103	243	56	134	125	174	165	132	196	16	3	18	5	8	4	9	11	5
208480_s_at	17	12	28	27	34	28	11	15	9	29	21	20	7	20	3	3	4	5	4	4	5
208481_at	11	32	61	81	242	114	13	59	35	15	126	117	61	62	99	1	36	30	13	22	16
208482_at	13	23	80	72	52	19	76	33	76	74	13	62	5	27	34	6	5	4	8	11	6
208483_x_at	16	15	8	9	23	23	8	13	8	7	38	18	4	6	5	6	5	2	2	6	2
208484_at	20	19	9	10	137	153	15	13	17	22	9	20	8	3	14	5	8	8	2	7	5
208485_x_at	221	257	328	623	580	864	277	315	272	235	351	342	58	104	84	105	110	91	104	141	125
208486_at	320	294	351	502	570	443	400	396	360	281	395	313	37	41	40	46	32	45	48	69	35
208487_at	8	9	22	12	62	50	8	24	16	11	9	9	4	9	14	4	5	8	18	12	7
208488_s_at	112	140	119	181	375	339	188	194	146	143	156	150	30	64	26	38	39	25	68	59	26
208489_at	30	15	15	16	104	55	24	18	29	13	23	34	5	23	5	9	3	9	11	9	3
208490_x_at	369	858	327	788	999	1646	313	218	199	162	1482	2648	1545	753	470	139	79	94	100	185	127

208491_s_at	17	13	16	22	19	13	34	32	23	45	21
	18	6	8	6	3	7	3	5	5	4	
208492_at	66	71	72	35	166	324	74	20	51	85	182
	75	81	46	46	35	5	40	44	52	28	
208493_at	17	43	263	255	18	16	147	83	244	208	21
	126	64	60	5	15	28	20	51	46	51	
208494_at	12	10	14	16	25	26	9	17	12	13	10
	11	5	4	5	3	4	5	4	5	5	
208495_at	16	47	7	22	8	19	8	41	28	73	63
	69	1	4	3	4	17	2	6	3	3	
208496_x_at	21	13	12	16	19	20	13	67	12	22	159
	418	180	239	16	13	46	15	7	6	3	
208497_x_at	78	32	118	96	11	31	76	135	20	103	70
	15	28	25	13	3	6	23	10	23	20	
208498_s_at	21	39	8	41	14	19	27	34	48	20	1
	13	4	13	3	12	25	15	1	2	5	
208499_s_at	291	49	91	27	25	23	252	125	127	137	202
	187	171	180	157	301	385	367	74	347	285	
208500_x_at	16	15	9	9	22	12	12	15	8	10	6
	10	1	3	4	3	1	3	1	2	2	
208501_at	25	16	50	62	14	9	53	91	84	63	81
	74	34	16	5	1	2	2	5	3	5	
208502_s_at	201	490	894	1467	753	646	339	141	731	449	1384
	397	547	327	407	133	91	168	343	247	153	
208503_s_at	513	481	1316	1627	646	1067	706	573	912	1117	1217
	1174	1022	559	748	98	116	91	786	992	863	
208504_x_at	13	11	9	12	18	17	19	13	8	24	14
	9	2	3	5	3	3	2	2	3	5	
208505_s_at	302	221	47	400	67	66	314	241	289	298	155
	44	13	14	28	68	23	31	43	35	38	
208506_at	110	5	98	98	117	51	64	91	58	95	88
	112	62	66	31	41	7	14	35	29	21	
208507_at	40	22	27	26	30	36	25	120	16	29	16
	38	4	5	5	5	10	13	8	7	3	
208508_s_at	17	29	9	10	87	144	30	29	20	53	31
	29	1	3	6	10	12	2	11	21	1	
208509_s_at	177	128	23	54	221	65	151	36	110	116	114
	161	13	32	42	26	14	21	32	7	14	
208510_s_at	1279	1257	2397	2557	52	320	1434	1393	2208	2577	296
	113	280	339	180	979	785	675	3742	2311	3143	
208511_at	278	86	312	215	803	167	204	293	356	412	416
	657	339	181	153	86	132	134	376	93	133	
208512_s_at	9	15	11	9	25	26	17	17	8	21	21
	15	3	37	1	3	2	4	1	2	1	
208513_at	19	16	23	16	30	9	15	17	12	18	16
	8	2	28	6	3	8	17	7	16	19	
208514_at	209	111	96	141	96	121	160	205	127	81	112
	112	31	25	48	26	12	13	22	48	37	
208515_at	4	100	47	28	7	266	50	12	16	41	98
	142	51	64	45	27	14	3	20	21	23	
208516_at	20	17	20	83	41	113	19	34	23	29	24
	21	17	6	7	3	6	10	16	15	5	
208517_x_at	14226	14499	15104	16854	9596	8562	15439	15456	15567	13581	
	12492	12438	14871	17202	16101	17266	19390	18312	13401	16182	12927

208518_s_at	107	41	100	83	41	103	101	120	39	31	88	62	2	4	5	16	12	16	4	6	5
208519_x_at	191	174	227	304	430	473	126	150	110	180	136	279	29	12	34	8	24	51	45	58	25
208520_at	280	203	330	270	249	344	250	191	288	198	222	244	26	11	44	16	14	17	49	37	35
208521_at	119	118	134	184	469	296	162	111	155	191	183	156	47	27	43	19	5	30	40	23	2
208522_s_at	49	6	20	18	136	156	64	29	47	17	74	25	2	4	13	6	7	3	6	4	17
208523_x_at	193	279	180	341	401	443	160	123	163	175	618	1171	721	166	192	18	37	122	114	145	77
208524_at	193	141	71	140	85	157	127	255	198	112	31	128	15	7	45	15	51	11	43	7	12
208525_s_at	13	52	11	12	38	16	57	54	47	29	23	116	14	14	4	17	2	12	11	7	21
208526_at	42	79	96	203	44	91	127	104	118	98	117	148	57	71	21	23	10	4	17	11	6
208527_x_at	400	928	296	900	959	1807	221	425	256	235	1950	2573	2150	531	438	152	122	166	149	347	331
208528_x_at	12	17	19	22	29	38	38	21	19	31	17	46	14	3	4	2	4	2	4	2	2
208529_at	11	1	4	3	166	165	3	7	5	13	20	4	1	8	18	22	14	12	8	13	1
208530_s_at	64	67	159	149	34	83	57	222	198	50	95	129	34	32	8	34	25	3	20	23	7
208531_at	44	15	33	18	45	106	65	96	25	66	91	85	6	12	20	5	6	1	2	6	3
208532_x_at	122	88	88	83	245	188	90	121	111	115	102	99	7	13	11	19	11	9	8	9	7
208533_at	5	7	5	9	14	12	5	5	7	7	4	8	5	3	6	2	3	2	2	2	5
208534_s_at	16	35	16	85	129	196	11	15	7	17	104	24	44	68	62	19	46	20	16	7	11
208535_x_at	152	33	83	47	95	74	76	51	122	129	99	168	103	186	68	68	7	11	21	14	32
208536_s_at	29	9	5	6	23	13	45	69	12	27	97	94	42	39	10	1	2	17	23	2	8
208537_at	29	10	14	32	26	27	23	24	17	29	27	25	4	2	5	3	3	7	6	6	2
208538_at	22	10	15	10	37	32	15	11	84	10	9	19	16	29	4	38	21	22	12	9	1
208539_x_at	29	22	58	19	58	50	34	116	63	48	21	30	41	41	33	26	6	20	8	38	19
208540_x_at	1732	1803	6702	4735	4642	3810	2037	1494	3919	5430	4512	7474	14444	928	1782	655	683	510	6543	2776	1388
208541_x_at	144	116	327	186	1318	847	401	272	821	700	543	400	551	322	189	179	197	193	310	150	317
208542_x_at	29	5	16	14	25	15	9	7	5	13	7	10	1	4	19	20	1	4	4	3	1
208543_at	25	9	15	13	19	11	19	20	20	53	14	35	1	9	5	5	7	3	5	7	5
208544_at	139	89	46	101	583	520	192	119	130	126	114	145	38	13	11	9	25	51	35	50	27

208545_x_at	9	12	20	9	18	19	11	15	63	22	16	19	7	13	6	17	10	3	19	2	17
208546_x_at	135	521	38	604	518	805	144	172	149	28	1052	2379	1763	557	613	84	63	47	82	322	151
208547_at	34	15	12	23	71	22	5	21	35	4	20	52	35	40	11	8	7	5	3	26	5
208548_at	16	49	53	52	263	305	82	102	45	59	44	89	4	3	64	30	6	2	36	2	25
208549_x_at	9184	10208	5838	2755	5443	2849	8605	10115	4640	6440	4714	5789	6733	1417	1424	2842	1894	2068	2789	2986	1773
208550_x_at	37	18	24	13	25	38	21	34	11	20	23	20	2	3	3	4	3	6	1	1	1
208551_at	212	82	80	141	151	168	159	146	154	92	97	116	34	33	18	6	29	3	20	32	17
208552_at	17	5	31	49	48	24	54	45	13	42	50	44	2	2	2	2	3	10	4	4	3
208553_at	24	17	5	22	41	50	7	24	11	10	28	40	27	33	7	6	6	7	22	30	1
208554_at	12	51	56	57	16	42	17	55	15	13	9	16	4	35	17	23	2	4	15	31	8
208555_x_at	22	35	28	172	605	2242	46	63	32	31	604	331	215	274	920	6	3	10	79	4	7
208556_at	12	13	16	19	22	15	13	13	17	14	11	25	4	7	2	3	2	2	6	6	2
208557_at	144	90	130	146	438	496	125	133	131	131	149	121	56	46	10	39	33	4	36	27	4
208558_at	130	99	182	91	888	672	135	216	144	154	155	178	10	26	31	25	22	45	13	54	6
208559_at	8	9	22	18	80	28	17	21	16	8	14	25	7	6	14	3	11	7	3	6	5
208560_at	17	10	12	13	192	157	15	16	15	14	13	13	41	52	7	24	6	6	6	4	5
208561_at	73	34	47	54	47	97	64	5	70	108	14	52	17	18	5	8	11	1	15	27	13
208562_s_at	16	19	43	6	110	24	29	38	11	41	11	9	6	9	3	3	6	23	3	3	6
208563_x_at	25	18	9	12	23	31	23	21	20	50	20	28	3	3	2	16	2	4	2	2	1
208564_at	9	7	9	12	128	90	85	9	27	13	10	18	53	6	45	25	3	24	22	4	11
208565_at	41	44	172	43	181	56	113	156	151	151	134	113	8	45	78	8	30	35	18	25	6
208566_at	4	1	1	4	73	99	7	5	7	1	7	3	20	3	9	2	11	6	8	2	5
208567_s_at	29	26	8	9	16	36	16	54	13	20	28	19	28	30	33	5	3	6	7	1	1
208568_at	234	173	263	199	330	434	166	201	123	113	210	138	48	18	19	22	30	17	51	31	22
208569_at	106	99	23	131	418	456	41	91	142	136	88	202	42	38	37	24	32	21	57	59	26
208570_at	95	55	87	76	151	26	70	102	144	126	87	102	5	6	5	6	35	3	3	1	16
208571_at	9	4	19	6	15	8	25	5	13	6	23	6	4	12	1	17	3	2	4	4	1

208572_at	16	4	14	5	15	38	15	9	7	8	13	9	16	43	63	16	19	7	18	37	16
208573_s_at	217	170	271	178	366	411	286	219	153	136	271	205	12	5	63	7	55	21	41	21	27
208574_at	20	10	24	26	23	39	15	16	13	22	34	9	2	2	7	2	5	4	3	2	2
208575_at	30	19	11	5	11	8	8	9	9	10	9	49	2	10	2	7	1	7	15	2	1
208576_s_at	7	15	11	6	66	65	44	5	8	7	51	82	34	33	38	15	3	7	20	16	3
208577_at	16	15	19	19	19	15	5	7	7	17	14	18	13	40	11	5	5	3	20	10	3
208578_at	294	232	110	331	374	275	276	186	246	411	244	258	42	77	98	69	51	78	35	48	32
208579_x_at	730	1690	506	1705	2374	3264	513	664	459	377	7066	10313	6768	3018	2086	512	498	359	458	1035	839
208580_x_at	11	19	8	19	16	28	16	8	9	10	47	215	218	181	136	52	94	65	34	62	15
208581_x_at	25640	22416	7444	4505	9718	10391	18150	25079	4454	4286	12450	25071	11984	12764	12025	15267	18284	14003	7024	6696	2756
208582_s_at	5	10	8	40	175	177	3	3	3	10	6	4	2	3	5	13	26	2	12	15	2
208583_x_at	271	302	111	507	131	992	250	218	135	182	401	675	294	186	178	68	29	100	75	87	60
208584_at	32	12	23	17	162	336	77	15	25	13	11	24	35	5	6	9	11	22	15	24	8
208585_at	123	57	100	105	217	62	158	239	189	210	129	138	4	61	46	38	37	7	35	41	31
208586_s_at	4	9	28	6	122	99	28	34	4	42	4	3	4	29	13	10	10	2	4	17	2
208587_s_at	198	159	87	154	99	336	152	236	173	205	138	201	8	39	59	47	72	58	101	70	54
208588_at	271	145	161	137	425	460	119	146	140	115	206	229	42	34	52	41	21	30	25	42	23
208589_at	8	9	7	10	26	26	5	9	5	6	7	9	2	2	2	4	1	4	3	2	2
208590_x_at	12	13	12	8	33	58	9	11	8	7	13	9	4	4	5	3	3	7	3	3	3
208591_s_at	235	236	579	645	455	447	453	358	868	720	327	276	70	35	41	81	55	55	222	93	173
208592_s_at	95	46	106	96	109	17	110	50	114	84	80	75	40	24	19	65	54	58	20	3	5
208593_x_at	112	77	141	156	463	120	156	111	202	130	102	137	14	6	50	13	65	39	54	31	25
208594_x_at	139	82	94	109	241	79	217	156	154	140	179	156	24	46	50	75	33	48	48	48	37
208595_s_at	181	336	163	100	144	110	314	302	84	165	77	52	32	16	14	16	42	84	16	89	100
208596_s_at	144	112	7275	16193	1647	2208	98	129	8963	12398	1059	430	2544	1465	1255	22	5	6	4973	2809	5310
208597_at	25	122	94	122	25	102	180	148	124	180	128	82	6	31	4	22	2	22	4	20	5
208598_s_at	4499	2828	4954	5079	5814	5253	5638	4997	4418	4660	7609	6461	1772	1282	1646	1190	1291	1312	1676	2292	1738

208599_at	7	18	41	45	43	44	9	45	107	38	14	26	3	13	21	10	7	6	10	28	9
208600_s_at	28	15	187	38	69	19	24	12	182	140	11	14	3	4	5	19	18	18	32	31	15
208601_s_at	15	23	56	18	93	24	21	96	106	115	23	65	4	43	9	21	2	60	6	7	7
208602_x_at	16	11	18	19	26	15	15	16	20	27	17	19	4	7	7	3	3	5	4	3	3
208603_s_at	48	17	31	36	78	86	66	103	23	34	47	20	7	9	13	5	9	12	5	5	7
208604_s_at	66	32	168	81	297	46	33	70	124	75	153	108	28	29	10	28	55	46	39	67	32
208605_s_at	44	9	16	10	58	23	16	40	24	18	6	6	1	2	3	2	6	21	4	4	5
208606_s_at	24	9	5	9	21	19	20	18	23	22	16	21	2	3	2	1	3	2	4	5	3
208607_s_at	12	40	35	6	528	470	19	12	12	10	1335	3628	1150	990	332	26	38	37	7	4	4
208608_s_at	156	158	373	1002	188	238	268	174	431	335	355	129	42	23	89	2	23	24	39	63	137
208609_s_at	28	200	34	65	92	71	37	54	48	48	24	33	11	16	18	16	17	5	11	5	41
208610_s_at	374	687	372	737	98	185	955	1091	904	966	587	597	345	297	326	465	380	271	501	573	190
208611_s_at	627	837	1801	2305	1002	1442	898	1118	1611	2714	2801	1802	317	568	773	237	254	219	540	648	519
208612_at	5629	5997	8607	4799	5214	5487	3518	3447	5420	4848	4180	4069	5438	5456	4161	9508	9967	8444	6688	11866	12003
208613_s_at	634	967	5001	2028	3262	2412	673	759	3133	3083	5862	1903	1121	767	310	155	183	137	437	458	369
208614_s_at	779	1248	5534	4168	2841	2872	979	783	3620	5097	6668	3161	1395	1720	593	318	339	347	1227	1355	1238
208615_s_at	778	584	1133	936	1380	1289	1414	1706	1141	1435	1259	1376	2830	1577	693	1040	1123	920	1579	1143	1536
208616_s_at	8316	8849	6683	3528	6514	5632	9125	9027	5622	5662	6447	5836	8182	8206	8443	13839	11543	11014	9133	9628	9575
208617_s_at	1908	1761	1877	1557	3350	3256	2043	2551	1953	2349	2354	2397	4616	5728	5000	7897	9496	8509	6001	6359	9298
208619_at	6105	3445	4479	3378	3626	2729	4765	4055	5748	4820	4299	3927	3455	1566	1452	1388	1388	1538	2198	1677	1789
208620_at	7246	5879	6143	5447	5821	4807	6514	6156	6732	6440	7802	4888	6740	7931	8923	6617	6547	7010	7415	5232	4470
208621_s_at	6131	7385	1805	1235	1744	1510	4082	4650	1434	1509	3375	1903	968	529	255	1857	2098	870	219	179	291
208622_s_at	10233	10860	2101	1407	2665	4213	5626	5104	1482	1263	4173	2569	2301	2105	1375	6951	8153	5737	988	1064	1136
208623_s_at	8964	10750	2948	2681	4251	4116	4514	5676	1381	1845	3232	4786	4378	4015	2789	10169	9681	9083	3528	3624	4120
208624_s_at	1061	760	1272	886	1039	900	1444	1281	1597	1561	1978	769	724	899	371	409	359	225	319	318	217
208625_s_at	3430	2898	3372	1870	3413	3182	3714	2659	4378	3677	4195	2125	2854	2213	734	525	495	644	882	653	474
208626_s_at	2152	2827	1272	2941	1685	3870	3672	3649	1386	1547	1995	1934	1035	1007	899	741	787	638	341	601	260

208627_s_at 17940 14592 16779 10441 10141 6669 20738 16342 23678 15755
13578 10235 14185 12352 8212 8750 10379 10200 8236 7180 9559
208628_s_at 22067 19472 20146 16694 13139 10186 17176 19960 21020 17298
14695 12135 18401 21488 19774 20806 21161 21903 16171 18426 15706
208629_s_at 1057 286 1141 1699 1519 1442 1497 1475 1523 1958 2621
2056 1004 1018 840 602 623 565 525 446 537
208630_at 964 670 834 1095 2313 2866 772 731 618 885 978
1266 3028 2543 2541 1585 1614 1278 1424 2301 2656
208631_s_at 1992 838 1774 2389 2302 1668 1778 1799 1610 2130 2821
2497 1555 1760 1705 1276 1846 1519 1335 1136 1341
208632_at 255 421 636 983 389 528 439 475 696 825 375
537 272 312 245 351 396 359 634 626 666
208633_s_at 868 490 1457 1194 2012 1008 955 717 1177 1119 2024
1601 310 435 157 144 155 147 230 196 200
208634_s_at 1716 1163 4280 2985 5608 2301 1344 1467 2376 3168 5038
4926 2203 3044 3579 1772 2036 1912 2406 3375 3763
208635_x_at 17536 18718 20853 24103 18179 21617 21855 20050 22500 21019
19738 16815 21947 28330 26472 24987 26646 25662 18851 26384 20536
208636_at 3960 4447 3603 2991 9333 7845 3086 2526 2335 3438 9847
13930 11209 13819 16267 9452 8012 8870 10138 7889 8783
208637_x_at 2240 2401 3273 2051 5675 3467 2230 2173 2042 3157
10437 8995 6150 4648 3596 1015 822 1231 1316 1018 1016
208638_at 3153 2626 4421 2554 5871 6946 2235 1964 3867 4147 4704
5684 6078 7836 7276 6309 7015 6365 7537 11321 12237
208639_x_at 7164 5435 11703 6159 7954 5994 4883 4429 7763 9550 8642
7789 9927 9748 7043 6716 7968 6123 9842 12336 12973
208640_at 7547 9189 11942 10028 11616 6784 8992 9079 11691 12542
11954 11420 12612 14572 17709 14021 15739 14883 11873 14550 16047
208641_s_at 6258 7120 6477 6403 7523 6489 5291 5813 6564 7560 8853
6712 12291 10391 8982 7013 5852 6661 6544 7003 6342
208642_s_at 4314 3321 5896 4611 7655 4433 8635 8093 7965 5964 7731
7356 8479 9493 9803 8582 7821 8517 8061 5786 6938
208643_s_at 3667 2866 4639 3987 4361 3810 8401 7626 6013 6089 6049
4722 4163 4176 4564 5658 5552 6360 5528 3112 4494
208644_at 4403 2650 4163 1432 2939 2177 3136 3009 3164 3919 2939
2819 2555 1828 1429 2373 2441 2215 2748 1592 1954
208645_s_at 27030 25523 23338 28406 24853 36246 24280 24399 24223 23199
21656 31267 21346 29404 24321 24815 25465 22706 15490 23199 16884
208646_at 5525 3597 4246 4692 5384 10288 4131 4308 3773 5114 5338
7431 11133 4895 6934 4657 4777 2410 4641 9145 3475
208647_at 7420 5401 7706 7381 4240 2090 3421 3986 5012 5210 2767
1651 3537 4153 6780 12930 11552 10105 9732 13838 13335
208648_at 1000 856 1206 1182 1348 1003 913 1073 1330 1711 1927
1494 2011 2027 1634 966 842 704 1591 956 1109
208649_s_at 3758 2776 4467 2977 6636 2635 3456 3151 4780 5298 7220
6190 4009 4250 2455 1094 1320 1289 1951 1014 1216
208650_s_at 107 265 4967 6178 359 766 182 183 6509 7043 166
246 214 216 107 205 217 264 10888 7407 9436
208651_x_at 291 631 6408 8904 572 1648 317 310 5685 7178 266
513 215 262 183 307 237 275 9890 7901 8317
208652_at 4102 4087 4175 3218 5510 3524 3755 3870 4348 3782 5537
4336 8211 7226 7064 6401 5673 5699 6332 4975 6110
208653_s_at 1421 1322 985 2262 858 1236 868 978 957 1501 1050
1354 613 344 462 895 415 575 986 936 2317

208654_s_at	2130	2265	3224	6108	1747	2104	1965	3168	3463	3961	2345	2013	2177	1654	1195	2439	2203	2439	3512	4130	7065
208655_at	5051	9458	4604	7085	3608	6113	4885	5334	3842	5237	5912	4304	4164	5115	6919	9675	9184	7999	6250	6685	8497
208656_s_at	4478	11609	5793	9494	5025	7401	6148	6744	4523	5135	5689	4062	4236	5569	6384	8729	8606	6454	3876	5941	6368
208657_s_at	1230	1470	1039	627	1170	575	1215	1256	800	900	1775	1139	715	607	428	275	227	218	247	200	100
208658_at	2441	1405	1412	857	1084	1348	690	759	1010	1201	708	802	865	544	530	997	968	1114	684	1781	2053
208659_at	7537	4859	7010	6575	8582	5953	6667	6426	5160	4735	9256	8266	10313	11264	8307	6618	7270	5307	4342	5408	3355
208660_at	4015	3364	5156	4567	5688	4166	5434	4890	7781	4988	8560	5278	6040	5090	7874	6017	5767	6701	7461	6430	7264
208661_s_at	640	1247	1126	2213	869	1247	841	1408	1072	1599	1124	1440	1901	1530	871	859	765	391	1294	1542	1649
208662_s_at	1918	3366	2739	5383	2548	6704	2088	2869	2747	3367	2166	2967	3380	2050	1106	1100	1040	791	1911	2545	3082
208663_s_at	225	264	392	740	51	611	302	759	735	983	507	592	1382	1602	1117	862	890	575	1056	1439	1443
208664_s_at	247	395	646	736	335	711	657	458	678	690	689	726	119	170	77	78	48	89	119	19	156
208666_s_at	1093	1089	818	825	1012	1664	1929	2130	896	897	1089	1552	1269	753	698	1308	1051	909	1185	840	1239
208667_s_at	2985	4045	2187	2447	2416	3698	3354	3865	2099	2143	2643	2987	3533	3291	5725	6960	6661	5685	5058	5161	7326
208668_x_at	14133	13384	10830	9494	15259	9727	13826	15915	11268	11061	14029	17536	17305	20300	18989	17078	17608	19749	12695	13091	12373
208669_s_at	2909	3365	1748	2336	2715	3485	1643	2676	1181	2324	2665	3270	4054	4587	4785	4102	3855	4013	3855	2554	2964
208670_s_at	591	939	392	714	1036	830	553	909	514	837	762	1061	2949	2015	377	560	589	527	393	282	447
208671_at	1009	905	1266	1485	1236	1910	884	1231	1077	1729	1154	1552	1411	1667	1689	2208	1942	1903	3206	2940	4139
208672_s_at	5203	2854	7142	4012	8468	4742	6739	6063	7253	5459	6039	5883	10060	9770	9353	11524	10331	11418	11924	8566	9692
208673_s_at	1128	836	1312	685	1457	701	1416	1528	1102	1133	1005	1134	2410	2516	2776	5175	4361	3991	4246	2003	3281
208674_x_at	5198	5861	2576	1997	2441	3103	3135	4839	1931	2045	2302	2219	3596	2685	2499	6809	5567	4881	3626	3755	3363
208675_s_at	10797	10484	5932	4318	3619	6038	8038	7569	5084	4711	5659	4255	6013	4648	2876	5438	7171	7765	2851	3338	3378
208676_s_at	5117	1974	3690	1566	1746	1227	5573	3881	5036	3685	3486	1675	3501	4503	8065	7244	6542	6855	5884	5836	5230
208677_s_at	2568	3514	2504	1773	1153	708	3505	2453	2309	3484	3704	2844	1736	2736	2808	3431	3799	3175	2482	1976	2515
208678_at	1289	1879	1641	2155	1193	1519	1942	1565	1266	1275	1587	965	2791	2594	2338	2498	2866	2283	1733	2388	1535
208679_s_at	5556	4810	8548	7649	6331	5568	5805	6028	8323	8933	8712	6544	16932	13183	9830	7668	9020	5602	10416	7126	6281
208680_at	13715	16763	17527	14610	19532	16094	22648	21848	22569	18552	14994	16487	17027	18029	17638	18935	19013	18884	13711	11320	6586
208682_s_at	501	243	435	601	598	2010	493	552	480	442	1170	1499	1451	2031	739	330	362	324	306	361	412

208683_at 8812 10426 8886 5777 9876 10026 8132 7523 6232 7395
11769 10103 10009 12486 14470 13044 15181 13672 7449 7843 9350
208684_at 2524 1519 4209 3341 1960 1986 2274 3042 2867 3751 2733
2536 1068 1125 1446 2154 2337 2262 1775 2806 3120
208685_x_at 2946 3497 2427 2292 972 761 3823 4092 2139 2028 2004
2167 581 743 463 738 760 922 279 551 354
208686_s_at 2369 1218 1017 989 634 680 2433 2295 627 978 1371
842 556 464 357 505 561 440 212 237 205
208687_x_at 14862 12350 20344 10661 21955 13394 27135 18395 18905 17118
21882 21979 21188 24957 23978 20149 18014 18601 17644 17080 16714
208688_x_at 5809 5599 6078 3943 5497 4439 5797 4934 8995 6935 7308
4255 6978 4259 3458 2813 3183 2682 4603 3659 3137
208689_s_at 6505 5764 9577 7836 6191 7653 6114 6647 8002 8123 7994
6598 10588 11122 9268 8900 11085 9505 9130 10358 12335
208690_s_at 1942 4821 2915 3981 2089 2516 2234 2177 2688 3712 2967
3411 2771 3394 3535 4339 5396 4924 3805 4031 3710
208691_at 7738 8366 10199 8793 11255 14919 10685 9786 13989 11783
10660 9708 14669 12124 12976 12725 10703 14506 15081 17116 18862
208692_at 30019 22321 27132 32088 26776 33454 25110 24140 31822 27125
22790 28170 21530 25682 25090 23112 26007 25518 17609 24726 20963
208693_s_at 5203 4488 9822 2086 4911 6836 7366 3593 5803 4147 5382
3406 7823 7976 13871 6949 7694 8243 6476 11444 13426
208694_at 4276 2180 2085 736 3296 2047 2347 2260 1458 1815 1924
1684 2189 1785 1853 1945 1516 2933 2919 2149 2155
208695_s_at 20908 30410 23724 31547 26315 32306 24852 23341 27767 24514
21396 31052 22315 26741 29779 27864 29979 30493 22908 28362 23356
208696_at 8423 6567 8500 3948 8490 4630 7855 6496 10476 7592 8051
5414 10419 15108 13284 10666 10837 10760 10401 10046 10122
208697_s_at 11748 16297 7975 8673 7395 8684 16374 15458 7824 8725 7962
7362 12838 18771 19332 23526 27475 26899 14034 19199 19013
208698_s_at 5134 682 4895 3848 3983 3507 4728 5726 4816 5530 5844
4631 2867 2577 1819 1702 1422 1883 2238 1525 3643
208699_x_at 9222 11447 11804 21457 2493 3548 7114 7039 9590 7741 5496
1800 3138 3751 2728 4279 3893 3221 3514 3632 3073
208700_s_at 9437 10873 10512 16753 4768 4968 5524 6463 7082 9312 5924
2092 5571 8257 7276 9044 8549 7341 7551 8682 7692
208701_at 296 280 381 216 467 352 371 466 407 401 463
337 32 44 70 69 38 8 99 62 49
208702_x_at 3888 5672 4281 5927 3998 5049 4110 4761 3851 5713 6452
5487 3142 3916 2416 1374 2124 1601 1292 1592 1560
208703_s_at 2068 2999 2935 4237 3163 5205 2999 3567 3069 4921 5084
4963 2232 2955 1493 933 1127 929 1337 1230 1101
208704_x_at 4194 5817 5944 8639 5236 6377 5482 6089 5079 8577 8863
9191 4943 6117 3950 2206 2580 1954 2585 2632 2160
208705_s_at 4976 6029 4052 3657 5182 7848 4647 4299 3635 3082 4788
4841 9317 9714 12460 9061 8323 8414 6822 10191 8208
208706_s_at 2474 3109 2839 2041 3970 4947 2814 2961 2725 2869 3772
4673 4574 4578 5619 4210 3884 5267 4082 4622 7043
208707_at 142 176 239 278 507 407 285 300 277 233 308
217 217 298 184 108 172 117 203 137 153
208708_x_at 2334 2120 2386 1234 3500 4014 2581 2559 2527 1966 3772
2676 3672 4386 6208 4065 3687 6354 2814 4071 5769
208709_s_at 1722 2097 1746 1786 1605 1850 2839 1961 1643 1447 1643
1268 1228 1145 1286 1521 1399 1522 1058 1002 1155

208710_s_at	274	645	473	105	58	206	587	487	472	513	405	246	1055	771	230	378	505	354	225	378	319
208711_s_at	915	844	589	270	2562	1007	1253	1209	619	513	3866	4058	8214	4628	1696	539	615	237	305	232	107
208712_at	3993	4070	1862	879	14423	6120	3886	3274	1608	991	9616	10165	13055	10498	6728	2665	2727	1948	1705	1599	1075
208713_at	1849	1693	3197	1694	2454	1181	1424	1741	2464	2982	2420	1977	3881	2672	1238	2429	1635	1615	6123	2503	2962
208714_at	2560	2474	5374	4946	1956	2759	2862	1748	4768	5057	3459	2951	4791	5005	3870	2867	3090	2738	5529	5710	4934
208715_at	414	318	578	649	575	755	530	471	409	580	540	415	659	783	816	1034	1124	847	1056	942	934
208716_s_at	1519	1636	1922	2707	2356	1994	2337	1902	2365	2531	2339	2854	3723	2959	3884	3724	4032	3982	5066	3519	4423
208717_at	2555	3314	1584	2099	966	1907	3616	3195	1717	1209	2025	1749	1985	2202	2636	3569	4515	3428	1719	2039	1953
208718_at	3766	7067	5093	8110	7431	11710	6879	7275	6554	6657	7461	7386	6679	6029	8820	8505	8311	6779	6758	8090	8909
208719_s_at	11	11	9	23	25	9	25	8	19	10	36	52	20	10	25	3	3	12	5	2	21
208720_s_at	3012	2939	4095	4656	3854	4343	2608	2275	3267	2742	3678	2251	4403	4578	6080	4258	4097	3767	5096	4005	4722
208721_s_at	814	1073	709	612	583	605	849	843	1627	1479	1158	958	130	144	154	221	187	345	345	212	417
208722_s_at	1386	1724	1508	1318	704	650	1719	1708	2341	2345	1317	1498	1236	951	920	1172	1192	1274	2235	1587	1877
208723_at	1154	1586	1412	1460	2311	1807	969	1603	1090	1673	3051	2585	3212	2854	3280	780	858	1111	1747	1788	2179
208724_s_at	11308	10305	8213	7601	8484	10350	7745	7477	9901	7604	7485	7621	11926	12702	13915	10808	12082	12566	11762	12115	15195
208725_at	62	36	210	66	109	153	133	36	278	386	155	160	236	242	302	183	183	195	456	433	574
208726_s_at	8409	9229	10232	5244	13825	13399	7935	5094	9129	6697	8857	7122	12288	14105	16396	11755	12569	13939	11604	16652	15187
208727_s_at	540	338	1325	2224	1076	974	3009	2291	1090	1268	472	633	583	914	862	752	729	745	622	451	382
208728_s_at	949	1248	804	943	642	904	1525	1449	614	985	829	528	1152	1123	467	1192	896	491	1267	784	599
208729_x_at	2322	8144	1523	1626	1368	1416	3415	2611	970	1282	4610	2941	2090	1951	681	744	1202	2090	332	432	495
208730_x_at	569	485	768	560	1555	1570	514	649	557	531	607	514	355	583	624	587	543	603	477	483	353
208731_at	1725	2642	1529	2102	1215	1946	2468	2173	1923	1688	1610	1318	1776	1571	1780	2732	2618	2708	2889	1632	2532
208732_at	663	825	549	676	520	663	738	808	459	558	580	577	606	595	801	1824	1366	1285	917	586	923
208733_at	79	63	111	105	70	102	117	99	63	56	80	85	45	61	58	132	219	109	50	47	72
208734_x_at	1061	1090	864	910	598	770	1736	1672	1399	1351	1445	1327	1336	1147	817	835	1063	785	872	674	566
208735_s_at	636	405	567	583	749	390	563	707	672	890	800	496	335	288	392	241	305	326	349	285	273
208736_at	1959	1858	3394	4401	2207	2498	2177	2142	3157	3723	2575	2604	6687	4635	4676	3439	3893	3625	6780	6627	4522

208737_at	3285	2282	1856	2825	2136	4226	2541	2573	1252	1621	2840	2551	6697	6814	4765	5046	5141	3983	3085	3842	3187
208738_x_at	8936	5668	3936	3823	8272	7515	5281	7379	4532	3898	4518	4730	9264	10896	8535	13869	11757	11886	11066	6807	5630
208739_x_at	8976	5630	5547	5654	6321	5674	8285	9777	5093	6049	7321	8709	12320	11626	10858	14028	15109	14721	7853	6683	6313
208740_at	743	515	278	582	660	735	527	505	297	443	662	721	1015	876	625	883	885	592	495	514	437
208741_at	193	221	164	393	324	431	217	195	264	396	269	341	498	282	202	226	224	186	247	339	291
208742_s_at	4235	3737	4597	4952	6349	5394	5674	5327	4451	4137	6565	7683	11401	8984	5185	3825	3522	4245	3131	2713	2002
208743_s_at	2340	1851	6104	4786	4441	3079	1681	2280	4273	4327	5615	3294	10968	9291	6202	3220	2370	2620	10454	6798	6217
208744_x_at	1565	679	2321	828	3282	1734	1587	1433	2546	1734	4038	3460	2279	2285	1153	363	385	381	615	291	525
208745_at	929	1387	1171	2015	958	1764	763	736	914	1247	820	1037	1252	1010	1179	1185	1126	1076	1868	1517	1633
208746_x_at	6037	7198	8497	8638	7481	6071	9080	7310	9308	8007	10477	11015	16188	15307	12400	8954	9672	9631	8483	7868	5357
208747_s_at	34	22	35	67	84	86	37	30	48	59	33	31	5	39	31	6	16	12	4	31	5
208748_s_at	42	214	90	47	30	43	46	58	235	205	33	38	139	13	21	96	61	133	18	39	13
208749_x_at	2274	5445	3024	2843	1739	2524	2675	2849	2394	3071	2216	1797	1774	1769	2058	2454	2577	2197	1773	1941	1610
208750_s_at	2611	2716	2874	2276	1317	972	2899	2293	3820	3474	2312	1695	2663	1836	744	1187	1213	1557	1160	1027	1406
208751_at	499	454	1074	887	485	538	443	486	879	781	647	408	261	369	306	255	224	283	565	494	515
208752_x_at	7188	10170	12527	10447	5765	5875	7338	10000	10493	11544	7579	6707	10117	10527	9995	14308	15203	11185	15436	14521	14690
208753_s_at	758	720	2823	1485	1460	1250	847	1140	1952	2034	1406	1620	2375	1927	2252	1376	1758	1809	3470	2902	3953
208754_s_at	1543	1317	3174	1516	1471	1235	1285	1990	2145	2373	1416	1587	3197	2825	3120	3883	3423	3354	6534	5618	8034
208755_x_at	12758	15194	19065	30298	15718	12152	13877	20055	19270	24902	15058	24407	17177	18759	19567	17316	18224	19386	17853	22319	19801
208756_at	4990	2760	4261	2585	4172	3300	6669	4182	4323	3969	4151	3743	5545	4286	3099	4254	4566	3854	3469	3203	2844
208757_at	2724	2092	2914	1764	1692	1680	2268	1620	2187	2233	2841	1306	1977	1255	801	893	1464	907	830	1116	884
208758_at	2110	2092	4605	2078	4771	3386	3873	3164	5150	4762	5048	4250	7015	7021	9963	4263	4200	4041	6030	6100	6215
208759_at	754	558	920	1636	562	1086	977	954	561	896	804	617	302	202	224	311	424	363	484	772	1331
208760_at	489	1197	407	772	493	468	371	516	294	351	210	559	268	133	175	575	523	304	495	483	489
208761_s_at	3034	1484	3125	3665	3045	2870	3008	3749	3500	4066	3989	3717	6354	4986	3788	3962	3653	3336	4463	2370	2714
208762_at	243	161	186	184	120	163	162	145	100	194	141	230	493	226	143	360	143	337	850	289	471
208763_s_at	607	878	671	2250	594	895	460	317	363	264	487	450	247	189	275	366	288	355	158	553	464

208764_s_at	5386	3778	5241	7779	4242	4607	3371	3192	4133	3835	2915	4469	6360	5591	5085	5964	5741	4224	6090	8127	4360
208765_s_at	3191	1524	2296	978	1368	625	3522	3884	3667	2995	2254	2625	1575	1527	819	1090	1053	806	1010	503	577
208766_s_at	6808	4344	4824	2280	5090	2846	5044	5406	5694	5329	4933	4000	6774	5031	6545	8563	7925	8018	9429	5541	7911
208767_s_at	8104	9008	2794	1816	1876	1485	10018	10185	5253	3683	3240	2409	2286	2151	2205	7538	7549	6874	2556	1984	2334
208768_x_at	15928	19518	17185	23702	17437	25284	22781	19963	21300	19448	16079	17092	16334	19052	18528	19846	22036	18840	13927	18060	11834
208769_at	308	129	214	415	283	99	303	527	215	432	473	310	40	50	12	57	32	53	49	24	80
208770_s_at	679	661	597	766	1036	736	996	1091	720	1001	948	1144	603	666	736	616	709	674	677	501	514
208771_s_at	5425	5958	4143	4860	3508	4038	5413	5980	3689	6426	3031	5046	3143	3274	5902	7773	7768	6253	5251	6435	6853
208772_at	673	751	1101	807	1078	1076	872	680	890	752	696	736	850	583	1034	624	529	624	878	1530	1803
208773_s_at	1377	1148	1708	1961	1846	2133	1207	1539	1011	1652	1715	1968	875	1027	1693	1049	1014	1097	1532	2213	2931
208774_at	1298	2310	1592	2751	873	740	1106	1463	1521	1950	1060	1142	799	753	569	578	718	537	890	643	581
208775_at	4158	3427	5118	4744	6910	9647	4608	5259	5500	4124	6714	5962	9656	7543	7743	5384	4635	4935	8151	5987	9142
208776_at	228	106	583	860	525	267	321	339	2331	2233	1371	620	539	604	228	295	215	206	1732	588	822
208777_s_at	3551	1864	5833	3017	5909	5734	4962	2856	7044	5400	6213	5223	6002	6843	6091	4192	3347	3880	6414	4093	3307
208778_s_at	10540	6911	5064	2612	7572	3646	11244	8125	5987	4615	5543	4394	6770	8050	9839	11982	11330	11886	7910	5905	5957
208779_x_at	5482	6706	1888	2027	2348	2443	3248	3728	1048	1448	3499	1610	1163	915	510	895	809	918	340	689	491
208780_x_at	9712	8914	8235	9039	7439	9373	7612	6818	9218	8102	9194	6368	10927	10966	15797	13079	14122	13914	12938	12609	13197
208781_x_at	4829	2909	4635	2750	3351	2930	4782	4756	5358	3916	3719	3627	4085	5530	5488	6257	6771	5416	4257	5010	4651
208782_at	445	1535	208	287	227	155	700	991	213	137	144	88	11	31	16	484	432	524	62	6	35
208783_s_at	1412	1337	2556	2848	3091	2450	833	879	1659	2418	2453	2282	4887	3537	5372	2906	3066	2141	5089	6258	9494
208784_s_at	618	462	570	600	660	747	1132	980	760	948	540	332	166	237	153	342	259	322	513	308	268
208785_s_at	2406	2771	3348	1856	2217	2092	2690	2902	1600	2013	2741	3730	3022	3446	3795	2995	3190	3034	1734	2564	2275
208786_s_at	1728	2662	2142	1489	1902	2000	1991	2753	807	1490	2561	4095	2310	3080	3047	2963	3917	3837	1021	2067	2502
208787_at	7783	7659	10536	4140	6976	5765	6836	6663	9391	8168	7730	5882	11479	11043	12832	15070	13389	14709	15919	14039	15442
208788_at	2646	3170	7	78	3325	2414	1729	1834	13	42	2625	1800	4013	3527	5492	4565	4640	3415	263	837	1315
208789_at	1245	1126	3344	3082	2335	2289	1030	1312	2336	3994	3695	3100	2397	1817	1680	985	754	472	1276	837	535
208790_s_at	241	282	507	663	296	387	241	249	383	760	649	362	254	263	86	72	52	65	69	66	29

208791_at	3034	3249	1073	943	1879	5142	1151	1029	819	760	2862	2501	3466	3434	2512	1311	1948	1949	746	777	776
208792_s_at	1716	1916	640	683	2465	6202	900	868	308	393	1718	1783	3017	3609	2748	1635	1627	1940	784	819	856
208793_x_at	581	529	719	488	737	543	798	747	867	826	652	395	145	158	57	93	87	13	114	119	88
208794_s_at	1663	1619	1328	845	1248	1130	974	1211	1647	1630	1707	1428	1567	1647	1459	1156	1173	1183	2324	1518	1654
208795_s_at	3583	1343	2816	930	7014	1060	3893	3959	3868	2854	6691	4206	5577	6552	2905	1357	1366	2118	3008	1444	1471
208796_s_at	3747	6507	4657	5887	2485	4452	4244	5727	5855	7588	3562	4670	4763	5773	8527	9602	8721	6031	10551	11100	14471
208797_s_at	106	102	91	93	658	509	118	153	120	120	111	97	31	8	7	10	11	38	47	25	3
208798_x_at	516	1238	845	1066	491	495	665	883	985	1448	551	386	321	230	257	367	441	368	948	667	779
208799_at	7234	5181	3798	3419	3068	1926	7720	6224	4355	2745	5094	3578	5233	4931	4827	6197	6577	5048	2802	2637	1702
208800_at	1404	967	1200	983	1255	1422	1970	1553	1638	1249	1664	632	1086	1066	1127	2298	1853	1940	2228	1745	2807
208801_at	3720	3593	3131	1856	2131	3124	4615	3596	3773	2591	2990	2180	3812	4345	5639	10271	9388	8715	6981	6542	9491
208802_at	877	1141	680	411	485	1031	925	713	443	451	435	398	883	702	1463	2458	2266	2116	1722	1524	2415
208803_s_at	554	356	670	310	641	470	1089	615	599	376	573	416	584	697	476	1019	936	958	833	628	749
208804_s_at	4322	3225	2853	4296	2118	1908	6045	5416	3666	3459	2702	2858	3789	4644	5979	8411	8279	7643	7703	4717	7094
208805_at	4764	6603	3213	3844	5471	4549	7190	5938	5439	3810	6822	5395	10029	10899	11904	11017	11080	11460	9047	9354	7021
208806_at	157	40	270	341	52	79	50	58	223	122	145	52	28	54	5	44	48	26	89	88	25
208807_s_at	591	954	713	1586	484	1110	608	483	527	400	668	308	192	191	154	164	167	80	170	149	10
208808_s_at	8047	2305	3448	597	8980	2018	5859	8580	3481	3711	6958	7979	13941	13236	11542	10595	13025	13341	10333	5164	7167
208809_s_at	1577	2046	1046	1185	1651	2223	1288	1368	863	1143	1153	1478	2732	2362	1748	3849	3204	3744	3644	3220	5172
208810_at	1037	449	735	501	1281	1287	627	789	656	625	986	1482	2626	2719	1118	1768	1213	1470	2070	554	860
208811_s_at	984	691	937	614	2152	1700	855	880	1196	1171	1714	1787	2519	1399	600	768	897	630	807	501	983
208812_x_at	8450	23492	2071	5631	4128	5151	8191	8682	1670	2611	8251	6882	4136	4712	4131	6279	8003	12218	1429	3357	2029
208813_at	2188	1220	1683	1098	1464	1363	2866	1767	1137	1300	1289	1096	1250	1648	1554	2626	3098	2690	1345	1012	1218
208814_at	234	347	427	524	290	145	566	466	593	822	425	819	915	718	386	167	166	159	435	448	264
208815_x_at	2422	1634	1947	1260	4514	2833	2947	2560	2816	1927	3286	2218	4136	3268	3446	2209	2256	2557	3407	2211	2817
208816_x_at	3346	4693	4479	3150	8155	19428	4506	6532	3438	6341	9324	13539	16811	19037	14666	8522	7806	5497	4853	2887	3935
208817_at	1154	1527	2762	3930	71	112	1884	1260	3913	2735	547	554	260	250	271	801	988	667	2311	2216	1537

208818_s_at 3209 4696 7233 9895 657 1011 4031 2970 8636 6094 1110
483 438 572 480 2537 2639 1784 5242 4748 3111
208819_at 2678 1926 1553 2932 2514 1699 2337 2125 1738 1680 2484
2456 2407 2965 3341 2818 2855 2961 2256 1674 1668
208820_at 2074 2614 3637 5107 660 755 1667 1684 2799 3037 364
264 372 401 488 3073 3010 2871 6258 4991 5660
208821_at 4077 2881 3515 1595 3420 1774 3988 3651 4758 3423 5824
3795 8409 6930 5838 4044 3484 3670 4731 4072 2588
208822_s_at 1769 2062 2857 1513 1519 1615 2743 2399 2692 3153 1720
2451 2867 2741 1315 2588 2720 2159 2986 3631 3189
208823_s_at 931 715 944 729 1069 880 1030 1097 1002 1124 1188
939 482 382 337 198 255 400 311 179 227
208824_x_at 1087 984 1091 1468 898 939 1505 1580 1303 1558 1634
1396 414 533 787 409 633 482 494 402 373
208825_x_at 36659 49272 33900 49190 37084 42151 37188 38652 39341 33288
30883 47203 24561 34646 41376 35628 40538 42806 21790 32503 26894
208826_x_at 12289 12741 11237 10191 9389 9936 11358 11311 9036 8569
11871 13442 12420 16209 15508 13283 14610 12823 10182 11792 8149
208827_at 6035 2265 3874 2762 5093 3706 5437 3642 3743 2619 6086
3632 10472 8217 5860 3890 3856 3608 3704 3125 1937
208828_at 2043 1050 2817 1434 3313 1558 2275 2357 3465 3697 3390
2747 4224 3838 4147 3550 3377 2767 5245 3140 4202
208829_at 1083 4010 773 3741 1277 1910 955 1003 682 1012 3165
1573 628 487 702 837 508 1315 612 659 741
208830_s_at 671 811 840 803 770 669 714 752 771 846 868
648 177 271 215 289 309 271 312 320 256
208831_x_at 696 507 608 400 192 183 716 611 482 558 972
416 210 192 111 140 203 157 141 92 64
208832_at 638 836 458 758 766 779 474 530 466 626 549
589 330 288 310 444 430 479 462 365 345
208833_s_at 4628 6208 2549 4674 3555 4159 5455 5231 3200 3733 4849
3423 3222 4688 6088 7669 8614 8896 4833 3762 4343
208834_x_at 36006 50810 35097 48848 39304 41656 37323 38910 40376 33981
31293 49572 25798 38016 44044 38085 40429 43284 22548 34712 28898
208835_s_at 1626 1585 1233 1339 1317 675 1965 1841 1644 1620 1411
1254 4886 3840 3833 4624 3977 2880 4251 4567 3557
208836_at 5882 6747 6044 3968 15760 10521 7041 5394 7391 6938
12498 10823 12627 13968 14830 12334 11196 11213 10877 10970 10070
208837_at 1155 1349 1808 2350 275 218 1663 1570 2436 1842 186
240 21 18 34 826 859 975 2034 1510 1151
208838_at 832 530 665 591 947 542 1153 856 581 661 530
832 807 634 641 1229 1108 1347 1654 1200 1984
208839_s_at 790 629 844 877 676 628 1055 897 1076 759 752
542 715 535 836 1490 1192 1401 1980 1145 1778
208840_s_at 1386 762 1259 882 953 380 2120 1957 2186 2066 1721
1178 612 716 663 1125 1296 1177 1381 832 923
208841_s_at 1898 1968 1919 2021 2777 3665 2339 2415 2014 2236 1897
2278 3045 2328 2004 3653 3333 2840 4133 2970 3420
208842_s_at 3989 2973 4048 3189 3516 4663 4653 3915 5122 4393 6661
3633 2302 1803 2013 2133 1931 2366 2174 2274 2674
208843_s_at 2338 2281 1747 1610 2826 3177 1628 1820 1861 1961 2304
1512 3822 3324 5678 4765 5507 4769 4469 5347 5541
208844_at 284 235 336 288 377 122 440 432 383 326 378
173 105 182 345 218 128 207 189 141 110

208845_at	6650	5288	4559	3034	7328	5537	7246	7052	5431	3863	6043	7101	7561	7821	9390	10815	10117	9935	9105	8132	6918
208846_s_at	1337	1022	1921	1058	2272	1639	2879	3254	2385	1536	2719	2716	3483	3597	2605	3221	2858	2779	2275	1604	1836
208847_s_at	1814	1733	1928	1706	2777	2164	2874	2324	1897	1522	1956	2185	3057	3340	3102	4849	3936	4706	3434	3164	3363
208848_at	1039	1313	1169	1185	808	841	1662	1492	1291	812	969	975	1067	937	1039	2420	2168	2323	2111	1003	1662
208849_at	164	39	146	76	198	314	150	75	107	122	118	94	24	49	48	65	86	100	85	41	23
208850_s_at	221	243	203	216	313	120	257	298	211	282	274	183	27	37	46	65	78	78	46	22	21
208851_s_at	183	164	164	133	399	351	148	154	246	236	189	136	38	46	56	33	55	79	55	43	23
208852_s_at	6343	4014	7159	4494	5581	10516	7245	5961	6857	5610	6646	5439	5437	4839	3634	5483	5072	4930	3645	4761	6406
208853_s_at	4932	1487	3414	2881	3215	3807	4392	4265	3793	3294	6128	3657	1090	1581	1534	1948	1909	1294	922	1062	1863
208854_s_at	2271	2347	3615	3950	2573	4714	2234	2237	3192	4470	5118	3290	2669	2438	3004	1909	1866	1737	2034	2461	2356
208855_s_at	5615	7144	6865	7983	8853	13297	4327	4252	7070	8120	8799	7760	12186	9923	13401	9170	9096	9267	11082	15064	15511
208856_x_at	35240	47475	32871	38398	25907	27593	36953	39583	35578	30247	26159	38790	19401	25536	25425	28049	29074	29247	17112	24172	20667
208857_s_at	2336	1496	1374	1064	2210	1872	1685	1872	1173	1279	1875	2017	2496	2398	1883	1959	2020	1785	2146	1574	1621
208858_s_at	804	990	1149	1063	1331	1549	957	856	1312	1572	1343	1274	1341	1544	1445	587	508	795	1147	821	1058
208859_s_at	103	50	79	144	114	142	109	144	178	296	192	212	483	442	225	210	172	148	211	300	269
208860_s_at	332	318	1077	1157	1079	950	486	804	777	956	810	469	583	353	369	159	168	204	984	1126	1443
208861_s_at	966	1118	2016	2169	1993	1731	1276	1581	1753	2024	1849	689	915	1247	2649	1829	1565	1889	3913	4134	6241
208862_s_at	1065	881	3539	4113	1260	1168	1704	1855	2743	3147	1593	1480	438	377	295	332	302	353	859	726	721
208863_s_at	1589	955	2036	685	2481	1247	2490	1353	1904	1841	2678	1780	2861	1992	1525	1210	1032	1171	2069	980	1020
208864_s_at	7324	9452	13006	9592	12022	12691	8634	8319	14732	13525	11182	13554	19364	19034	15272	9099	8763	11069	14774	11527	8175
208865_at	3891	4505	4457	4662	4647	6329	5015	4070	3741	3646	4733	4837	6749	6432	7587	6708	6076	6055	5997	7740	9645
208866_at	512	191	692	617	1005	821	457	603	640	759	777	770	878	592	525	213	255	268	735	661	1058
208867_s_at	1098	1275	822	1079	660	935	1839	1610	785	1106	1393	1327	1090	892	1265	931	800	951	1343	1258	1783
208868_s_at	183	254	80	163	623	637	158	244	62	137	631	538	640	537	159	125	63	107	77	44	57
208869_s_at	267	478	172	335	603	645	211	231	79	136	630	880	734	680	309	295	245	290	94	159	143
208870_x_at	8791	7284	7949	9888	6699	6258	10811	8744	10155	8462	8713	5769	7283	9313	14352	13355	13507	13726	12222	11476	10921
208871_at	243	290	391	304	340	278	248	281	333	298	311	189	49	45	90	65	51	87	81	79	39

208872_s_at	2248	1573	2009	1839	2304	1688	3476	2878	2194	1779	4629	2696	3610	3698	2997	2465	2529	2318	1871	1833	1211
208873_s_at	897	1133	1211	1846	2284	1936	1280	1160	1205	1119	1502	1852	2736	3045	3655	3101	2899	2337	3337	3663	4508
208874_x_at	1418	1456	1257	1441	2040	1598	1465	1285	1125	681	1809	584	324	271	277	267	216	144	357	267	203
208875_s_at	722	483	1104	644	1215	1040	1212	1246	1300	1282	2245	1485	1095	934	1408	592	473	532	770	552	943
208876_s_at	172	125	254	163	319	563	323	355	343	345	490	364	154	135	175	147	113	136	226	151	153
208877_at	1005	450	1331	993	2184	2466	1035	1020	1262	1526	1775	2087	1748	1640	1387	1297	1098	1137	2595	1996	2646
208878_s_at	529	528	720	535	877	1192	696	852	856	832	1293	1071	646	490	595	664	419	654	759	626	924
208879_x_at	496	646	846	867	521	375	746	718	1145	1249	1622	829	242	249	57	68	40	40	77	78	54
208880_s_at	950	1213	1465	1461	1028	1035	1052	900	1692	1609	1538	1431	1349	1058	568	297	410	145	543	414	473
208881_x_at	3090	1291	2998	1891	2711	687	1752	1684	2561	1719	1182	1151	1933	1655	2187	5281	4940	4051	3262	4271	4636
208882_s_at	1119	1148	620	698	812	726	1247	1407	894	711	681	513	271	320	431	959	665	1134	673	465	610
208883_at	462	321	176	132	175	159	519	565	218	82	111	143	128	48	182	298	251	240	221	202	369
208884_s_at	1521	988	1474	1196	1174	852	2842	2977	1805	1617	1619	978	466	480	493	1011	692	882	668	614	679
208885_at	53	35	146	74	113	85	166	223	235	282	200	199	13	31	63	61	51	52	185	375	577
208886_at	5402	4028	5909	4572	4350	5428	3531	4650	3380	5716	4103	4668	3629	2571	5137	5054	4561	4361	5132	5104	3964
208887_at	3174	4154	1331	1809	1950	3192	2768	2155	1758	1298	2150	2090	4011	3205	4533	5128	5510	6846	2651	4630	3317
208888_s_at	26	18	14	34	91	95	38	15	13	34	13	15	2	4	6	5	3	3	3	3	2
208889_s_at	82	161	170	110	166	102	139	158	118	182	118	46	36	25	13	47	12	55	10	9	22
208890_s_at	1588	3151	1238	3268	636	763	1597	1928	1317	1986	1589	1057	1393	1446	1118	1388	1682	1502	1058	1388	1058
208891_at	4667	614	1594	102	10806	246	3077	4488	1299	3178	8863	15043	14365	16603	13542	8562	8368	7919	4567	5722	9342
208892_s_at	3470	556	1636	113	4150	110	1672	3263	813	2604	4247	5649	9951	7540	5051	7777	6264	6303	5624	4337	6839
208893_s_at	1778	137	572	17	4234	13	1443	2288	266	1493	5065	7028	6456	6300	4064	3185	2691	3314	1635	1441	2425
208894_at	102	55	99	129	95	67	126	73	198	136	92	92	11	2	2	82	17	44	6	21	20
208895_s_at	2552	2443	2846	1402	2568	2164	2808	2155	2392	1966	2199	2500	3281	2413	2557	2070	1562	1811	2609	1485	1222
208896_at	606	382	1405	630	1142	1122	1100	1083	1702	1585	1515	1359	2575	1663	1351	1303	1096	1338	3384	1707	2141
208897_s_at	1081	1204	2763	1326	2713	1635	3358	2634	3251	3194	2967	2878	1536	1705	2458	1968	2066	2325	2251	1371	1773
208898_at	1070	1236	1628	2143	3410	3481	1716	1314	1521	1578	3704	3569	5317	5468	5808	2732	2782	2413	3404	2647	2859

208899_x_at	994	948	1852	2545	3472	2913	1997	2027	1710	1873	4254	4226	4718	4428	3037	1266	1367	1086	1496	1273	1210
208900_s_at	483	209	503	202	312	47	678	651	677	660	625	599	1653	1373	529	910	788	537	501	505	209
208901_s_at	4124	2541	6229	2840	4801	2325	5066	5193	5570	5135	4089	4283	5573	5496	5573	5528	7637	7292	5697	4743	5713
208902_s_at	78	17	65	136	25	39	46	146	62	94	67	60	42	15	35	38	12	48	65	43	37
208903_at	605	338	247	398	335	327	337	586	325	470	311	415	98	213	171	446	460	346	261	228	157
208904_s_at	25406	33963	20405	26496	19122	16626	25536	24795	21875	19214	16969	26731	14243	17161	13420	16405	17439	17416	9941	15626	10280
208905_at	10128	10715	14837	9252	13220	13598	14073	13812	13989	14017	16290	13049	19348	20216	21347	20182	17733	20666	17304	15821	17738
208906_at	565	501	579	1071	753	661	1053	816	788	875	1448	1278	1467	1118	1058	419	389	480	662	288	337
208907_s_at	2036	335	893	674	975	1111	1676	1023	1110	892	1219	922	2330	1598	1247	1399	1728	1472	908	983	811
208908_s_at	1017	1365	1468	1079	1988	3068	1138	1268	1208	1115	1450	1411	1310	1346	1674	1160	1386	1400	1030	1366	1868
208909_at	7954	9434	8500	8931	5520	4808	8932	6342	9925	6872	7066	5438	9905	10538	12045	10130	12554	10780	10605	9290	6548
208910_s_at	10203	9826	8698	3077	6603	4060	8517	6929	9482	6552	6027	5629	12069	8722	6896	8056	7992	8207	9352	9023	7656
208911_s_at	1226	830	1294	1239	1032	1133	901	1009	1403	1648	1674	1448	2139	2318	2072	1829	1659	1626	2195	1358	1698
208912_s_at	1688	1557	1076	2072	1993	1568	1295	1349	1053	1238	1772	2177	3497	3468	1828	740	956	944	1079	756	816
208913_at	581	603	460	340	425	452	461	664	566	843	476	366	308	176	185	246	257	361	531	327	236
208914_at	385	293	709	757	348	168	404	554	868	1036	753	597	250	232	199	142	266	211	346	215	238
208915_s_at	479	453	837	651	183	134	704	496	1413	1033	669	459	148	83	99	156	68	190	172	202	127
208916_at	2557	2046	7237	1284	4000	1619	2922	1763	4536	4409	9572	5126	3835	2977	2670	399	493	643	1188	1266	1210
208917_x_at	452	353	236	356	304	368	429	439	454	415	438	138	79	128	89	58	70	116	85	36	63
208918_s_at	1439	1463	939	609	889	1228	868	1238	768	1125	1185	773	1334	927	966	808	961	1139	1016	573	534
208919_s_at	1265	1684	754	1051	863	793	982	1262	1006	1163	1067	1161	723	615	570	835	925	855	951	721	789
208920_at	287	372	274	657	477	495	289	245	203	264	273	625	720	546	728	510	405	517	774	448	700
208921_s_at	3284	3634	3710	6019	3398	2866	3310	3038	3549	3646	4797	4468	5738	5505	10450	5958	4749	6325	4787	5003	4242
208922_s_at	1282	684	1362	1134	1586	968	1255	1186	1248	1417	1693	1470	1381	1817	1103	641	854	767	862	1157	1233
208923_at	5714	7422	4666	3858	2926	3655	4826	4302	4748	5558	3057	3158	3033	2502	3943	6014	5852	5996	6646	5693	7337
208924_at	1773	2659	1780	2063	1131	2079	1689	2040	1188	1195	1091	1351	1679	1105	1283	3336	3085	2828	2840	2144	3456
208925_at	2850	3551	754	287	568	641	3126	5557	448	608	810	734	667	509	203	3889	2702	3504	540	246	556

208926_at	741	596	832	1707	569	998	545	534	566	738	645
	738	1131	1033	836	439	344	394	258	544	583	
208927_at	767	1001	1565	1361	705	899	1108	1603	997	1093	1397
	680	1150	1763	2677	2436	2425	2676	2045	1287	1263	
208928_at	978	1190	695	675	1244	925	975	850	500	436	1767
	597	967	1183	1117	498	418	501	306	355	310	
208929_x_at	24669	41171	18056	19134	14890	17031	20577	22230	15925	16576	
	13618	22287	16516	17299	15745	18506	20325	18118	10842	17305	11010
208930_s_at	696	718	545	341	245	62	1022	1087	986	860	813
	332	680	415	85	150	132	118	123	102	113	
208931_s_at	2332	1036	1301	605	1273	689	2186	2696	1610	1877	2258
	976	929	595	459	599	509	619	805	475	541	
208932_at	1241	1334	1750	2020	1122	1153	1730	1570	2062	2116	1234
	1318	1280	1124	1017	1268	1104	1047	1417	1132	913	
208933_s_at	411	353	389	638	837	1039	229	570	144	333	1192
	821	1160	1852	1857	1012	1066	918	819	1531	2375	
208934_s_at	390	274	487	445	1274	1230	521	604	450	457	1683
	1206	1193	1560	1412	541	601	585	485	647	642	
208935_s_at	77	67	100	169	544	761	106	136	23	88	323
	246	318	321	260	124	72	171	203	181	191	
208936_x_at	294	221	327	482	1094	926	338	399	288	380	1559
	711	789	1202	1369	512	450	455	435	655	605	
208937_s_at	12516	22381	5391	3610	389	347	12130	17082	3757	6633	333
	528	195	171	141	4872	5411	3860	2304	2015	1439	
208938_at	938	476	699	657	628	601	611	718	680	524	495
	425	329	226	202	298	198	258	325	296	245	
208939_at	1098	870	985	654	1106	782	1239	973	989	887	1340
	783	923	1084	1271	1714	1711	1533	1925	1198	1929	
208940_at	241	303	477	380	416	223	427	366	496	387	456
	478	543	425	412	469	495	414	597	568	827	
208941_s_at	667	468	681	422	716	468	712	635	765	669	816
	606	1166	913	988	750	776	635	1232	888	898	
208942_s_at	1082	1349	1229	1357	1913	2168	875	1198	915	1089	1396
	1141	4285	2523	1134	1218	949	857	1393	1297	1051	
208943_s_at	1267	1219	2081	3099	2399	2727	2029	1602	1539	2194	2709
	1830	3029	3044	4392	3240	2515	2673	3466	3305	4659	
208944_at	785	809	1934	1886	1364	765	819	967	1789	2757	900
	1161	867	1564	1046	733	959	1015	2231	2030	2626	
208945_s_at	933	804	1126	1754	1005	1033	1097	1189	832	1318	1050
	1376	1303	1098	801	823	903	844	863	678	990	
208946_s_at	1434	2028	1461	2733	1348	1609	2055	2093	1691	2470	1667
	2413	2040	2502	2645	3531	3329	2953	3196	2296	2749	
208947_s_at	220	204	262	345	456	345	719	537	456	436	581
	273	195	119	18	62	77	66	80	118	56	
208948_s_at	4063	3068	4780	5966	6464	5127	3132	3569	5295	5118	5870
	5987	7448	8823	12170	7948	7041	7489	9016	8864	9980	
208949_s_at	5144	7210	3942	10245	6831	12582	3380	4178	2744	3521	6667
	7086	13456	17961	21822	10404	10626	7088	10915	12194	10391	
208950_s_at	1397	895	1244	1610	1649	1816	1459	1587	989	1268	1431
	2082	2229	1862	1259	1726	1636	1611	1243	1171	1267	
208951_at	659	581	711	1062	965	933	1117	1240	599	880	1032
	1546	1818	1174	574	1007	1126	1040	845	601	699	
208952_s_at	1226	1218	1257	1245	564	672	1357	1205	1226	1227	672
	470	198	243	281	504	414	507	894	683	777	

208953_at	1001	1012	996	832	469	427	912	838	916	1166	362	430	290	279	245	572	517	661	817	546	739
208954_s_at	1162	1307	1108	720	654	548	775	859	813	1085	409	304	216	288	310	542	466	423	486	614	562
208955_at	455	253	711	400	962	472	690	670	1304	1160	1275	1435	1584	1419	1911	487	486	636	1780	766	1015
208956_x_at	4479	3244	3589	2539	6119	2478	3159	3600	4253	3737	5540	6184	9360	7102	6866	3964	3801	3878	8043	6147	5261
208957_at	310	254	480	658	595	798	264	292	341	456	281	356	85	83	134	135	135	127	177	249	180
208958_at	15	41	109	76	14	46	68	48	60	67	70	83	60	63	32	24	43	60	73	41	129
208959_s_at	1400	1227	1894	1976	2727	3408	1408	1252	1255	1560	2793	3001	2727	3370	4120	2478	2965	2973	2266	4028	5295
208960_s_at	365	766	294	44	113	403	95	123	47	338	370	347	517	567	248	246	287	209	168	355	368
208961_s_at	968	990	712	246	377	137	224	472	266	572	475	597	1007	822	795	700	760	605	586	1203	1082
208962_s_at	1458	1452	117	35	1817	950	661	821	41	25	1711	640	1763	2021	1098	507	490	508	32	211	102
208963_x_at	996	596	9	23	544	551	704	790	8	7	1062	583	1490	1806	1368	803	884	754	19	170	162
208964_s_at	2490	2199	316	328	2293	2137	1252	1808	242	348	2699	921	1259	1648	2471	1718	1943	1723	172	416	445
208965_s_at	8	9	12	17	18	58	8	50	12	18	13	8	2	9	4	1	1	23	1	26	15
208966_x_at	37	28	28	50	47	183	52	70	35	56	40	45	1	4	2	1	2	6	7	61	102
208967_s_at	2502	1002	2793	1502	2268	1637	3554	2718	3884	2882	3340	2927	3525	3189	2901	1369	1482	1469	1694	1552	1170
208968_s_at	1726	1319	1671	1458	1766	1218	3473	2136	2497	1915	2805	1596	2523	3085	3266	3187	3542	3231	2513	1768	1701
208969_at	2402	1855	2849	2356	3226	2709	2564	1711	2566	2194	2714	2605	3888	4836	6191	2790	2723	2765	3938	3571	3767
208970_s_at	1965	791	913	1599	2162	3307	1155	975	971	549	1248	1548	2623	2820	2667	1764	1495	2101	1431	2132	1331
208971_at	462	186	507	591	1919	1188	579	464	458	298	722	704	1174	984	826	696	460	400	633	526	405
208972_s_at	2680	1401	1498	1523	1527	1375	2645	1544	2771	1543	2049	2359	2217	1595	1590	1817	2030	1488	1436	1066	442
208973_at	587	359	605	653	416	376	558	620	545	576	475	390	258	338	539	605	569	488	730	596	404
208974_x_at	4595	2895	5890	2894	6401	2768	5480	4535	7400	6029	6813	4950	9049	5594	2797	2162	2383	1851	5635	4522	4304
208975_s_at	2568	1943	1983	638	2587	1301	2058	2047	1751	1564	2385	1881	3325	3578	2185	2080	1987	1834	2713	1659	1333
208977_x_at	12674	8057	19426	5484	16182	6311	13609	15356	18407	18405	21737	23021	18730	18631	11138	3892	3913	3389	10506	4894	3326
208978_at	36	156	464	1999	38	26	380	435	603	718	31	39	5	28	31	93	108	64	75	97	66
208979_at	1386	1536	1241	1270	1603	1501	1253	1250	1120	1233	2241	1000	1663	1958	2206	1652	1528	1855	1517	1399	2410
208980_s_at	14087	14803	18034	21272	9771	14462	20646	20163	15751	15515	14019	12511	13995	14647	13101	13797	15350	20738	11444	14996	15389

208981_at	20	67	20	52	241	69	36	34	25	28	30	35	8	16	5	29	4	10	44	29	11
208982_at	160	179	212	210	686	593	199	127	209	271	134	190	19	43	22	34	5	49	55	42	17
208983_s_at	19	11	20	12	27	19	30	20	25	27	18	19	3	5	5	5	18	2	3	20	5
208984_x_at	1281	1309	1595	1260	877	332	1432	1225	1737	1557	2166	1245	1265	1384	1350	637	787	595	1086	601	597
208985_s_at	2212	1641	4490	1844	2720	1764	1599	1127	4594	3817	2385	1781	3123	2569	4790	1906	1549	2437	7642	6075	7271
208986_at	1544	1603	2128	1667	1431	1841	1427	1726	1164	1349	1278	1205	1153	922	2217	2609	2331	2569	3106	3907	5202
208987_s_at	528	515	1080	1516	1038	1102	913	684	1330	1189	1147	886	309	393	248	145	145	187	329	285	211
208988_at	480	629	997	1088	1384	3309	395	433	844	879	657	1384	1182	729	735	532	506	490	1869	1687	1926
208989_s_at	230	206	655	684	768	906	327	384	602	524	613	359	233	271	127	127	116	128	198	125	193
208990_s_at	1262	1157	1141	1341	1300	1273	1459	1176	1330	1061	952	1200	2331	1911	1533	1966	2071	2053	2290	1720	2391
208991_at	922	1386	2832	3757	1545	2162	1186	1268	2438	2981	2135	1509	754	1047	928	670	896	677	2044	1436	1758
208992_s_at	388	433	948	1533	855	1095	266	523	847	784	742	587	238	196	371	326	387	209	414	386	353
208993_s_at	403	432	340	349	423	404	783	779	541	525	655	452	2554	1796	1849	2087	1508	1422	1905	1211	1206
208994_s_at	589	366	644	891	859	497	750	1075	1068	860	902	752	1527	1381	1132	945	870	807	803	543	428
208995_s_at	242	258	231	182	674	439	217	322	217	215	313	210	645	629	413	586	411	499	820	456	621
208996_s_at	2596	1916	2671	1985	1549	1215	2547	2417	2687	2393	2462	1389	1804	1495	1211	1212	1684	1409	1261	842	768
208997_s_at	598	495	1734	15576	198	251	710	986	2487	4493	1153	1168	129	239	228	200	311	290	1220	915	863
208998_at	340	423	2016	20753	804	910	515	628	4466	7678	1001	1462	1174	2385	2531	891	1383	1205	8169	5358	8442
208999_at	1164	1539	1008	1235	1475	1735	1023	1151	1314	1652	1494	1375	821	853	622	609	581	456	1119	823	841
209000_s_at	331	238	180	35	179	86	329	252	270	326	766	269	261	300	284	132	97	159	172	63	106
209001_s_at	2838	3168	3832	3732	3248	4279	3383	3193	4755	3465	3456	2831	4659	5258	6723	7924	8612	7013	8156	7707	8186
209002_s_at	442	487	753	2526	739	945	635	780	791	847	1111	1075	194	316	521	288	224	228	348	483	470
209003_at	1540	1742	1087	1298	852	1027	1688	1478	1330	1454	1533	975	862	352	351	304	354	441	557	268	248
209004_s_at	624	980	319	577	946	1824	611	573	190	270	514	606	837	728	630	1056	867	872	577	819	1450
209005_at	159	365	144	119	111	262	192	265	106	82	122	58	141	177	210	375	406	397	193	333	369
209006_s_at	319	363	214	588	434	431	207	241	218	418	227	308	167	162	207	51	60	79	221	370	355
209007_s_at	628	1564	1214	2036	1740	2267	578	806	599	985	820	902	985	1083	1530	737	505	701	1242	1660	2207

209008_x_at	10553	18293	19339	25016	14387	6880	9135	6669	17062	21081	17711	16491	21377	18485	11272	6201	6922	5779	9454	9106	8255
209009_at	3179	3521	3869	3431	3642	5885	6701	5123	3277	2593	4256	4195	6373	6500	8238	8336	7661	7647	6426	4910	5085
209010_s_at	12	11	41	22	11	17	5	16	83	138	101	15	10	8	25	9	2	5	19	17	9
209011_at	794	826	901	742	647	1060	623	776	1045	1080	877	861	159	161	253	324	275	307	317	599	516
209012_at	299	410	605	481	643	716	253	282	417	584	384	675	276	258	215	221	207	242	425	515	597
209013_x_at	81	710	764	433	66	99	352	338	468	681	691	553	107	87	54	65	118	123	109	148	116
209014_at	2417	1372	1716	3258	2356	2174	2292	2142	2019	1924	2065	1782	2361	2356	2321	2681	3289	3148	2903	3148	3834
209015_s_at	486	375	628	877	1922	1590	351	373	626	787	1416	1136	1535	1640	991	776	711	241	817	580	753
209016_s_at	304	3817	186	102	175	146	440	274	118	56	199	84	117	66	123	791	1252	666	126	290	159
209017_s_at	2295	2370	2444	515	1559	1783	2255	1565	1635	995	1600	852	904	1032	864	1001	1496	1097	615	942	1113
209018_s_at	762	476	536	949	1177	1578	869	1054	649	735	937	925	388	505	249	261	321	235	183	296	185
209019_s_at	359	568	351	939	1052	1610	526	685	213	400	900	677	656	544	276	409	414	536	198	233	256
209020_at	437	461	1374	1111	1531	1365	666	688	850	1010	1487	2248	1409	1612	807	457	473	436	837	1145	1147
209021_x_at	427	388	850	825	404	216	519	497	670	625	823	464	305	383	375	419	390	378	496	422	320
209022_at	1172	1137	2702	1989	3526	2903	1575	1693	2003	2769	2919	1865	4661	4993	5636	3423	2809	3873	5907	5811	8468
209023_s_at	101	67	525	428	467	474	160	216	355	546	674	460	1374	1242	388	172	175	185	1124	796	1120
209024_s_at	4069	793	3074	1160	3607	1746	4047	3360	4629	3737	4852	2918	5532	4172	1987	2029	1805	1317	3087	1807	1435
209025_s_at	2486	1214	1748	1119	2070	1142	2140	2371	2490	2288	2521	2061	4121	3873	4007	4322	3695	4198	4771	3468	4277
209026_x_at	16850	4265	15671	3057	11136	4874	23655	18375	17507	12432	20195	16662	12320	11704	9442	7206	7302	9175	6579	3684	3440
209027_s_at	757	676	1989	1799	1458	1064	1057	1565	1264	2017	1019	1470	851	711	1002	1027	791	1001	1126	1021	1450
209028_s_at	254	414	548	758	488	512	337	430	598	858	304	548	509	506	502	820	736	579	1608	1043	2117
209029_at	1603	1181	1280	1258	844	1148	1700	1809	1496	1237	1181	1073	1058	962	589	641	503	499	622	474	358
209030_s_at	168	112	87	109	1069	2844	60	141	71	88	1151	1121	635	700	569	12	10	59	60	32	15
209031_at	46	24	12	5	1695	6768	34	70	16	17	1916	2095	746	741	1224	22	3	9	25	46	21
209032_s_at	187	127	64	113	329	1450	162	112	138	95	459	501	276	324	381	5	19	37	26	32	27
209033_s_at	1086	1444	998	1286	2459	2943	1809	1488	1283	1441	2245	1948	2230	1987	2892	2006	2014	3043	2162	1925	2585
209034_at	542	1247	509	1851	405	551	405	992	300	837	381	402	310	429	284	993	1071	810	338	619	2023

209035_at	1494	3362	156	198	1528	1969	1595	1284	62	96	556	587	466	338	242	791	774	810	69	154	101
209036_s_at	6744	6806	5191	4671	8364	8687	7163	5995	5352	4569	6584	8013	12403	9559	8500	9447	9446	7939	8542	7988	7344
209037_s_at	216	117	307	185	423	108	187	141	321	430	763	325	275	326	212	164	139	230	250	199	183
209038_s_at	15	158	296	393	91	34	85	13	177	282	556	93	64	99	50	61	91	48	41	11	2
209039_x_at	835	808	1088	1070	845	715	820	537	1072	1554	1877	1584	665	781	622	481	384	377	491	425	363
209040_s_at	15	11	33	234	558	676	9	12	15	25	1380	604	673	493	313	3	5	5	103	245	163
209041_s_at	398	343	350	210	188	78	1002	640	780	710	743	501	347	247	162	154	90	132	128	94	80
209042_s_at	1192	764	867	388	788	452	984	903	1189	1322	1262	982	1054	1260	2303	2548	2663	2198	2281	1509	1670
209043_at	2418	2485	1888	4882	2305	1602	3795	4349	1761	3314	1880	3149	2205	1915	2001	4531	4268	4453	2199	1746	2391
209044_x_at	2443	1575	2676	2258	958	656	2790	2391	2645	2626	2027	1017	813	880	1231	2085	2122	1888	2999	1551	1361
209045_at	1646	1237	2086	2597	948	1879	2129	1203	1775	1372	1040	876	841	808	1028	2662	2408	2926	3600	3202	3383
209046_s_at	3908	4851	4609	5211	2065	2424	3803	4831	5611	5648	3171	3619	3226	3692	4360	5392	5327	4538	6340	5168	4154
209047_at	156	140	111	114	44	38	154	164	297	284	72	153	4	2	25	3	24	18	32	24	15
209048_s_at	913	703	1232	1767	1416	1091	775	676	1231	1076	1816	958	1205	1455	1325	417	468	526	951	997	1313
209049_s_at	462	355	987	1183	1097	1070	599	437	910	1016	1792	1214	1750	1559	1401	677	530	759	1219	1159	1619
209050_s_at	734	961	567	823	610	633	751	984	554	675	374	349	356	377	565	1085	1135	802	587	711	804
209051_s_at	417	551	273	279	70	172	504	610	357	408	257	157	92	83	113	198	159	103	159	111	133
209052_s_at	544	534	772	199	383	231	1081	1029	965	811	779	372	171	135	85	57	63	68	97	79	58
209053_s_at	1541	1187	1153	345	668	215	1334	1390	1577	1189	1126	911	767	558	582	553	590	580	711	334	414
209054_s_at	1322	1029	1143	547	731	544	910	996	965	924	715	656	676	520	307	557	557	594	1011	615	670
209055_s_at	284	168	570	428	753	757	947	1122	692	671	948	984	375	358	279	402	437	519	345	202	440
209056_s_at	701	743	792	631	1067	654	1014	1330	856	914	1066	1127	1210	1188	933	1290	1415	1790	1431	1178	1715
209057_x_at	857	579	595	568	441	441	961	888	761	749	658	747	464	300	478	638	486	484	599	303	347
209058_at	2826	1876	2522	2206	2082	1928	2263	2059	2312	1841	2257	2467	4541	3931	2455	2818	3457	2899	3800	3741	2302
209059_s_at	2789	1189	2229	2287	1402	1931	2577	1887	2220	2024	2346	2055	5588	4691	3162	2416	2892	2270	2382	2404	1275
209060_x_at	191	204	362	384	250	231	472	381	443	432	412	469	158	184	137	167	174	147	176	181	307
209061_at	388	358	536	595	840	833	280	471	526	422	578	480	376	385	406	530	371	457	430	584	591

209062_x_at	139	46	106	211	150	153	144	248	351	169	327	287	131	147	226	111	156	137	126	77	144
209063_x_at	2351	2524	3215	2448	1839	2283	2091	2139	2695	1931	1791	1410	2076	1184	1031	2112	1922	2313	2903	2445	2928
209064_x_at	1699	1923	1773	2108	918	1461	1397	1554	1414	1498	1086	1160	1676	1041	1412	3030	2439	2074	3720	2731	3949
209065_at	628	866	275	344	243	425	698	682	281	198	252	274	280	325	395	934	1034	928	409	391	367
209066_x_at	7406	10485	4898	4113	4679	6476	5264	8769	3699	2581	3516	3494	7517	6665	4152	11579	11136	10954	4448	3246	2441
209067_s_at	4294	1247	2249	1270	2805	1748	2753	2853	3180	2461	2825	2179	5959	4935	3268	4457	3942	5046	6276	3684	3512
209068_at	725	429	676	834	799	484	611	666	915	924	694	578	719	579	572	1174	936	741	1206	982	1552
209069_s_at	13403	8540	9944	7271	11485	5650	11994	14858	10733	11263	10952	15458	16352	14868	9981	11143	10862	11753	8753	8169	6631
209070_s_at	42	35	41	54	23	52	126	50	31	48	65	14	5	12	29	16	76	35	8	9	5
209071_s_at	46	18	19	21	27	20	29	32	25	28	18	25	15	14	11	42	41	42	4	44	55
209072_at	34	117	22	129	197	280	41	135	64	109	97	48	8	7	40	45	27	27	24	24	7
209073_s_at	730	386	794	801	1035	1320	472	508	482	584	995	819	613	452	175	234	194	278	434	323	333
209074_s_at	15	16	11	18	36	15	15	12	20	8	14	16	16	8	22	17	7	8	5	9	3
209075_s_at	3405	3923	2564	2474	4000	3817	3571	2941	2842	2274	2459	2847	5548	3313	2115	3939	5273	4858	5746	6785	4645
209076_s_at	4522	5280	2360	1959	2121	2286	3748	3521	2905	2273	2868	2721	2180	2150	2129	3027	3034	2648	1966	1713	1823
209077_at	1506	1431	1055	1222	1006	1029	1525	1291	1057	928	853	616	871	576	988	1859	1863	2013	1612	1057	929
209078_s_at	1410	656	947	952	950	1277	1723	1314	1092	872	1229	730	1408	667	403	947	852	1072	911	696	662
209079_x_at	537	639	236	319	421	531	663	625	289	337	1195	657	203	237	304	150	212	128	70	134	92
209080_x_at	2395	1597	6288	2090	3209	2494	2764	2092	5273	4194	4228	3104	5030	5861	6986	2721	2571	2587	6384	4201	3798
209081_s_at	501	1334	638	4150	1203	1051	351	606	594	710	850	660	685	824	476	261	330	237	325	323	255
209082_s_at	374	754	323	1751	1065	1407	432	412	334	281	489	466	370	395	304	277	336	217	282	215	290
209083_at	126	27	62	9	291	191	123	111	55	48	271	181	324	185	195	46	70	84	61	6	41
209084_s_at	274	427	137	74	192	392	221	269	84	50	244	141	333	301	163	360	304	316	148	116	232
209085_x_at	761	446	616	407	874	622	1305	1268	776	830	1050	860	715	640	809	911	842	1191	847	543	702
209086_x_at	116	238	289	366	429	480	288	197	476	602	736	450	394	409	178	98	81	115	88	71	70
209087_x_at	511	673	373	248	1652	2587	343	336	641	426	1893	1398	919	1210	877	418	490	375	407	423	327
209088_s_at	279	237	422	484	444	298	553	589	662	718	586	670	705	523	348	354	423	262	360	281	242

209089_at	2539	3250	2462	2340	2107	3213	2149	1999	1975	2169	2177	2296	3367	3478	3550	3761	3747	4121	3951	4595	5111
209090_s_at	803	640	796	1392	663	1007	713	790	682	915	841	468	435	491	499	703	684	815	1265	953	1188
209091_s_at	2017	1979	3257	2718	1141	1461	2245	1702	2222	2376	1772	1352	1675	1851	1624	1824	1751	1627	2197	2530	2268
209092_s_at	1343	1162	2009	1601	2789	1893	1964	1993	1765	2119	3057	2868	4128	3969	4127	2661	2186	2016	2553	2217	2633
209093_s_at	325	242	395	557	286	391	433	455	381	479	449	450	187	264	183	185	239	214	266	174	250
209094_at	1357	2141	4197	6420	2482	1860	1393	1127	4778	4752	2160	3999	2653	3568	4220	2958	2600	2636	10213	8849	11584
209095_at	3106	2912	3236	2945	3590	3588	3880	3923	3255	3019	3567	2539	4820	4450	5804	6658	6025	6123	7114	5009	7656
209096_at	1076	622	899	555	2474	2130	1196	1297	1026	908	1370	960	4698	4263	3295	4206	4128	4113	4272	2009	2321
209097_s_at	60	41	7	14	15	19	17	20	20	70	10	14	31	30	56	16	6	23	8	31	2
209098_s_at	832	359	815	153	254	199	497	497	330	492	173	173	62	53	54	89	86	115	104	167	173
209099_x_at	3370	1523	2009	247	638	839	1246	1006	698	1242	307	479	700	746	1516	2054	1904	2228	2092	4331	5236
209100_at	2004	1401	2842	1152	1836	1923	2471	1393	2737	1982	2388	1474	2685	2012	2096	1275	1382	1181	2183	1341	1263
209101_at	58	118	286	163	301	141	44	78	33	98	270	807	470	545	221	480	455	495	1611	2355	3366
209102_s_at	476	448	334	1291	422	590	436	655	276	397	502	664	607	780	739	629	744	551	541	856	1056
209103_s_at	2688	1848	3022	2081	1498	1033	3646	3127	3706	3055	2895	2745	4049	4619	3936	4071	3232	2907	3934	3649	2393
209104_s_at	7423	6425	6822	3967	3799	3932	5558	4963	7591	6155	3981	4981	9036	6469	6859	8794	9950	10772	11047	9529	6676
209105_at	298	156	159	251	162	649	229	223	131	147	382	254	275	306	387	103	95	84	183	175	235
209106_at	228	214	175	323	474	1114	317	311	122	145	648	368	452	456	527	267	263	298	443	471	828
209107_x_at	815	715	1024	1567	838	1732	827	771	777	897	1647	1327	509	686	767	226	200	209	500	480	533
209108_at	951	1796	1521	2986	1229	1512	1664	2198	1474	1637	1614	1748	1874	1535	1736	2873	2499	2424	2381	2158	2984
209109_s_at	545	783	1272	1935	1086	1164	1185	2111	1350	1852	1779	1894	2283	2074	1739	2519	2268	2060	2197	2255	2819
209110_s_at	1073	1299	712	1542	581	724	672	756	582	566	550	689	285	372	350	344	252	252	252	451	349
209111_at	806	1410	654	1009	698	742	745	1147	375	658	568	718	866	636	649	1294	1232	944	721	789	632
209112_at	1082	1285	1023	1653	1850	2965	843	1202	599	805	1373	1379	1964	2442	1857	1895	1683	1933	2479	2555	3231
209113_s_at	356	982	434	754	40	169	694	1006	555	674	539	489	308	215	93	185	177	119	200	79	83
209114_at	590	571	5515	15790	355	704	753	594	3468	9005	836	625	722	774	464	576	904	611	3866	5278	3353
209115_at	799	1083	1191	1377	2025	2348	901	1029	985	777	1205	985	2341	2505	2136	2943	2492	2808	4278	2290	3324

209116_x_at	209	206	45	39	51	32	36	78	23	14	26	35	10	17	47	59	19	75	12	11	2
209117_at	1365	1918	1476	1005	1023	1361	1724	1847	821	1451	1347	1234	268	286	262	438	408	367	144	267	207
209118_s_at	2041	1337	136	94	4144	6923	1219	2020	62	151	2564	4632	4902	8437	8044	6940	7845	6039	147	1555	1225
209119_x_at	167	139	246	248	238	207	265	354	278	260	171	181	191	147	127	143	129	90	160	238	136
209120_at	1959	1136	1295	1613	1812	926	1566	2249	1177	1048	1175	1268	2666	1854	2121	2120	1609	1587	5059	4148	5890
209121_x_at	1155	1077	1761	2539	1306	637	2026	1660	1929	1606	1882	1629	1342	913	880	401	383	407	1006	718	681
209122_at	497	602	1142	882	1325	1305	653	463	1511	992	1036	1704	1191	1392	1685	694	989	863	1525	2229	2496
209123_at	2193	2125	681	432	1109	1299	2706	2119	554	390	914	1462	2909	2454	1224	2378	2151	2332	935	1037	743
209124_at	1154	2046	2500	3288	1795	1855	1419	1215	2739	2208	2744	1875	1689	1056	1091	841	974	1370	2528	1836	1837
209125_at	152	91	155	63	216	99	212	271	92	154	195	396	16	18	17	5	32	6	15	26	17
209126_x_at	259	192	202	180	1878	1166	180	286	269	159	908	802	281	143	98	129	88	75	29	122	124
209127_s_at	852	740	711	484	37	75	941	901	1185	858	725	684	739	651	204	395	481	303	187	169	213
209128_s_at	1884	1229	1008	758	2268	707	1061	1116	1086	992	1086	970	812	714	857	1213	1130	1226	1304	831	955
209129_at	2064	4107	791	992	2308	2337	2597	1403	919	390	2463	1338	3250	3040	2927	2471	3008	2761	813	786	557
209130_at	1365	980	1986	2588	1012	1793	934	1324	1046	1371	1164	1346	1747	1161	2094	2743	2006	2343	3337	3519	4391
209131_s_at	86	11	175	286	60	181	208	239	246	214	141	275	116	90	6	95	49	99	85	88	224
209132_s_at	1564	1157	1333	1592	992	609	1219	1469	1840	1829	1201	1318	2068	1445	943	688	597	675	1410	1036	658
209133_s_at	222	169	222	304	241	51	269	241	234	250	236	205	63	80	71	13	32	35	125	94	55
209134_s_at	20962	29745	24445	27550	25943	29169	26279	27311	25510	24916	19438	28130	21153	26394	24053	26915	29512	30246	18539	26606	23920
209135_at	3248	3439	4315	651	1538	2388	1489	2213	1987	2702	1187	1293	1297	1548	1061	2924	2685	3329	5732	3622	8368
209136_s_at	1076	242	628	538	588	563	1602	2130	1409	1329	1281	875	139	179	106	189	139	270	142	96	154
209137_s_at	2450	1092	1313	612	1300	777	2337	2288	1664	1614	1272	1423	570	835	848	1661	1510	2046	1214	719	881
209138_x_at	130	117	33	45	47	62	166	145	170	84	117	33	25	22	40	38	12	16	35	21	28
209139_s_at	1192	1789	1062	895	1685	1876	1075	1421	1489	1393	1886	1832	2980	3497	3339	2359	2178	2085	2479	1623	2056
209140_x_at	8634	22254	1683	3547	5723	5394	8112	7692	1136	2250	10338	8991	6847	5307	3595	3143	4577	6120	677	1750	957
209141_at	1786	1736	2378	2563	1994	2127	2029	1847	2644	2697	1966	2095	2056	1370	2809	2432	2394	2958	3680	4023	5921
209142_s_at	1111	953	1728	1379	1167	1078	1040	1325	1588	1574	1424	1230	1967	1868	2433	1588	1537	1684	3171	2033	2658

209143_s_at	3458	3263	5572	3888	2640	2352	3334	3754	6964	6885	3412	4013	5600	4156	4739	4843	4195	4382	8025	5621	5873
209144_s_at	119	131	110	197	69	74	38	100	87	210	132	132	25	9	69	8	5	13	14	21	54
209145_s_at	91	144	113	230	48	270	81	103	197	115	126	136	7	54	50	38	41	37	48	5	15
209146_at	2909	1581	1572	1022	3385	2092	1357	1292	860	605	1749	807	2774	2828	3313	5763	5318	4454	2281	4343	5053
209147_s_at	620	820	632	4546	271	277	570	713	906	762	499	596	255	281	319	560	534	348	767	633	880
209148_at	717	535	570	823	675	805	550	747	630	730	557	633	273	252	107	356	336	263	310	290	246
209149_s_at	934	1027	682	865	539	615	931	1065	700	815	1103	857	514	440	397	551	629	561	290	342	416
209150_s_at	1144	1535	806	1138	1248	1422	920	949	665	910	1342	1058	1249	1088	1445	1630	2068	1748	1217	2178	2383
209151_x_at	284	280	397	176	38	17	415	379	527	419	401	96	62	24	13	2	20	31	15	19	8
209152_s_at	906	434	491	405	430	167	431	380	393	437	379	234	268	100	139	166	259	189	205	152	138
209153_s_at	1797	2145	1498	1507	625	404	1043	1141	2006	1761	460	454	792	842	783	2250	1895	1385	2107	2666	1690
209154_at	2772	6492	3832	5597	7264	7134	2507	1956	3445	4025	5359	6486	9560	8544	10768	5131	5690	6314	7588	7907	5671
209155_s_at	1521	1418	1194	1225	962	2255	486	1052	981	1237	740	1051	1284	912	648	1503	1253	1481	3006	2112	2342
209156_s_at	433	966	8	45	27	22	666	916	17	49	14	18	4	4	6	287	327	328	1	2	3
209157_at	2086	1724	1487	1243	2012	1426	1900	1366	1979	1748	1452	1439	2487	1207	1611	2718	1821	1493	3334	2386	4823
209158_s_at	447	353	526	619	924	925	325	467	555	661	530	534	1139	1072	493	333	445	275	386	684	529
209159_s_at	176	27	68	62	379	267	147	186	15	101	367	194	100	170	144	86	93	149	60	21	38
209160_at	15	90	513	8612	18	56	81	113	470	977	3	18	3	19	14	54	25	65	777	2274	1525
209161_at	2525	1439	1994	973	2833	1463	2341	1983	1640	1810	2855	2410	2647	2826	4580	3287	3261	3612	2565	1913	2575
209162_s_at	1508	343	1450	671	1516	1064	2394	1866	1898	1445	2419	1761	1347	981	886	702	616	811	520	335	446
209163_at	2401	2249	1960	1786	810	1068	1940	1634	1605	1467	1336	553	693	499	842	2312	2705	1967	1394	1855	1943
209164_s_at	1162	944	1150	1068	493	513	994	1044	887	897	1104	553	608	536	334	740	823	509	618	868	573
209165_at	1666	1933	2462	1167	3105	1924	2178	1671	3121	2273	3381	2270	5093	4979	5983	2876	2667	2198	3607	2998	2891
209166_s_at	325	695	214	503	78	130	391	438	225	337	460	499	326	338	179	242	274	186	111	220	214
209167_at	17	7	18	19	15	26	13	21	7	21	16	19	16	42	2	20	6	21	23	32	3
209168_at	115	102	164	96	275	245	150	144	167	110	90	147	26	24	84	40	41	25	26	24	7
209169_at	11	72	8	40	316	173	30	48	11	8	10	13	3	6	38	34	13	45	29	29	32

209170_s_at	7	2	11	9	21	74	11	5	11	11	17	11	4	1	1	2	1	2	3	1	9
209171_at	2068	2437	1219	971	1073	978	2272	1611	1989	1842	1887	1840	3010	2163	1396	1228	1244	1037	1377	1348	925
209172_s_at	475	101	289	101	506	7	220	395	261	305	306	397	375	258	262	195	111	211	388	137	164
209173_at	7	12	4821	1959	21	38	16	9	1767	1980	9	5	26	4	5	27	13	4	6481	16824	15812
209174_s_at	1023	887	834	1311	1328	1041	1272	1136	1086	1092	1253	1626	661	583	439	635	818	805	747	618	818
209175_at	1146	978	913	674	896	735	1476	1363	1080	980	661	671	789	480	452	1538	1286	1515	1292	759	1151
209176_at	306	150	129	226	374	425	109	206	227	219	202	160	151	99	158	274	266	161	413	425	432
209177_at	1484	1872	1063	1399	887	1447	1643	1948	1319	1796	1255	1346	1772	1582	699	902	1000	752	933	812	546
209178_at	676	648	543	389	425	491	750	563	708	609	551	221	166	97	9	13	76	44	69	63	42
209179_s_at	1218	1595	994	2737	623	620	1275	1523	1203	1686	1595	700	727	730	755	599	561	755	505	261	301
209180_at	1459	2178	1039	817	1196	1347	2079	1896	1428	1412	995	963	1906	933	1250	2428	2392	2007	1318	1461	1390
209181_s_at	2680	4546	2855	1398	1398	2354	3854	3236	2266	2975	1663	894	3501	2689	3646	6604	7316	6599	5702	4623	5325
209182_s_at	115	257	19	206	179	31	133	53	90	98	78	116	35	17	46	26	31	18	31	42	39
209183_s_at	42	910	24	532	32	55	34	26	5	18	33	93	13	8	5	55	80	9	57	130	91
209184_s_at	472	774	308	407	1155	1411	355	878	868	1291	1003	1132	865	977	957	370	326	271	579	804	700
209185_s_at	431	1457	262	477	2066	4130	370	677	847	2556	2243	3898	2981	2929	4220	1320	1183	1124	2734	3661	4611
209186_at	8663	4905	10555	4635	5337	2905	7224	6215	8897	7678	6096	4982	3225	3307	5208	7635	8002	7772	6655	8286	9558
209187_at	1835	1601	1603	614	961	1012	1243	1321	1222	1101	983	975	1585	1116	1444	3705	2524	2515	3697	2251	3674
209188_x_at	1335	1264	1967	1468	896	735	1590	1502	1568	1845	1741	1072	1123	955	1240	1307	1054	1338	1523	1382	1630
209189_at	89	9	328	23	693	12	27	439	460	1030	1185	961	2725	3069	2320	2890	3231	3450	2320	3697	4350
209190_s_at	1740	1345	2748	1340	2972	1760	1810	1822	2756	3009	3256	2769	2674	2218	1168	1066	956	923	1916	1130	1508
209191_at	2720	2957	26	35	4759	2000	2808	3043	25	172	6667	6040	8343	9721	7020	2888	3065	3154	53	42	61
209192_x_at	836	676	879	1009	810	773	864	829	704	662	669	607	477	734	336	404	424	374	439	503	318
209193_at	487	418	416	422	675	805	295	562	397	889	559	871	228	181	220	273	326	204	221	161	179
209194_at	669	870	2714	4590	1494	1670	1120	1370	1754	3363	1245	1857	1930	1900	3146	2713	2616	2150	5681	4805	5573
209195_s_at	493	377	704	1405	511	363	598	664	1157	955	505	362	214	139	95	103	70	78	226	202	217
209196_at	515	61	160	19	590	328	343	297	282	231	362	150	749	544	417	365	323	188	254	202	180

209197_at	105	112	141	189	315	265	180	176	142	106	84	113	19	46	27	10	51	30	41	31	44
209198_s_at	85	74	90	78	159	106	85	125	78	91	72	111	19	4	21	29	11	11	25	44	33
209199_s_at	120	449	106	27	173	159	514	529	32	136	87	84	19	5	7	588	630	548	26	47	45
209200_at	33	331	14	14	18	19	264	194	5	8	4	5	1	4	2	440	541	514	8	4	23
209201_x_at	53	46	1222	908	1798	87	46	59	1810	5637	1681	1195	1600	1951	1024	9	8	45	1071	1298	2631
209202_s_at	397	240	239	237	482	348	408	339	277	289	267	206	98	113	69	109	128	118	95	125	84
209203_s_at	403	204	349	239	99	206	372	437	349	293	469	269	405	290	150	235	261	136	193	112	110
209204_at	152	184	222	211	78	94	187	165	144	197	97	65	199	151	77	75	48	67	289	251	105
209205_s_at	1537	1781	2115	1315	393	574	841	798	1032	860	416	323	363	278	451	684	629	435	1146	1219	820
209206_at	712	522	719	587	397	615	322	515	349	394	313	397	490	555	730	532	532	700	1023	1292	1682
209207_s_at	1287	982	355	737	612	609	1268	1151	550	605	532	610	526	756	993	1242	1350	1353	1747	1423	1299
209208_at	1107	328	1058	693	1933	1129	1069	1178	1603	1358	1920	1274	2155	1597	391	354	418	490	535	342	328
209209_s_at	249	252	52	16	351	278	327	344	3	35	554	466	266	378	313	342	331	301	22	49	78
209210_s_at	962	1045	130	52	838	1146	882	739	12	70	1136	1120	1224	1470	1571	1232	1237	1034	48	245	339
209211_at	578	552	1264	1833	1259	1286	241	532	617	960	445	671	2064	1780	1057	713	510	800	3742	3124	3679
209212_s_at	557	793	2362	4622	936	710	788	897	2700	4088	1325	958	1438	1624	1373	725	719	595	4112	3583	3186
209213_at	12	6	358	522	961	1033	15	15	589	583	1101	1007	1813	1214	1066	7	19	43	653	415	197
209214_s_at	4179	3001	3116	2209	4359	1545	2525	2936	3508	3323	2639	2390	4240	2640	1470	1645	1562	1495	2962	2287	1781
209215_at	685	1378	739	662	680	435	590	641	860	1010	507	285	484	285	190	264	183	240	432	302	192
209216_at	537	715	1008	1363	878	910	292	509	678	966	1069	983	889	978	1011	728	1049	947	877	2602	2094
209217_s_at	615	595	925	1029	790	849	578	581	451	970	959	824	538	369	392	312	310	345	423	762	552
209218_at	3150	1946	11577	4331	3861	1666	2238	2540	8707	6661	3145	1625	2346	2365	3688	3557	2920	2634	7699	9116	9057
209219_at	1753	1466	1005	1278	1408	1381	1525	1163	906	1040	1340	1188	2274	2234	2121	1967	2448	1416	1400	1647	1345
209220_at	116	24	142	186	213	290	118	113	112	141	109	185	26	38	72	53	46	36	79	44	45
209221_s_at	365	238	343	557	577	801	293	306	470	616	469	406	338	309	354	187	214	249	298	280	343
209222_s_at	534	522	981	1084	1409	1258	439	478	1109	1104	989	993	714	951	1003	601	517	469	1097	1160	1044
209223_at	135	91	155	44	45	39	135	214	154	140	61	142	63	49	57	41	5	3	28	12	4

209224_s_at	2965	1787	2553	4612	3153	3674	2333	2619	2588	3507	2906	5340	4441	3771	2399	1435	1441	1362	1891	1663	858
209225_x_at	831	686	1620	1031	1825	1670	1212	909	1432	1656	1804	1708	1928	1564	1515	1053	776	824	1594	1448	1441
209226_s_at	673	561	2804	1586	1796	1168	1489	1273	2312	2207	2004	1673	2287	2318	1557	501	439	409	2242	2035	1978
209227_at	89	38	100	147	47	173	65	90	98	87	102	73	8	16	33	20	35	23	15	14	24
209228_x_at	124	84	79	66	173	254	179	123	86	85	144	77	8	8	8	52	88	65	10	6	9
209229_s_at	1122	923	1207	825	1281	687	1059	909	1121	1066	1110	636	621	480	291	279	226	256	404	242	175
209230_s_at	146	122	681	996	154	585	200	199	304	194	168	111	44	67	79	98	139	112	179	1096	500
209231_s_at	790	248	1011	635	588	469	616	471	1365	893	965	665	309	422	446	291	340	344	329	316	262
209232_s_at	341	89	719	697	676	486	587	296	961	776	681	798	542	558	628	352	278	244	782	637	570
209233_at	3285	1602	2126	891	2349	1676	4626	2563	2996	1994	2886	2045	4765	1865	1886	3097	2460	2224	3408	1940	1506
209234_at	979	886	681	763	979	1195	787	905	763	879	1337	877	438	326	598	683	709	678	680	714	888
209235_at	364	41	419	257	808	610	54	248	298	396	341	54	207	27	38	63	21	145	128	117	114
209236_at	452	555	358	72	972	1963	216	252	305	161	655	498	564	398	401	282	339	202	230	212	205
209237_s_at	299	189	218	43	216	759	326	239	292	228	347	245	67	110	158	14	7	59	21	37	39
209238_at	788	848	1805	1671	1326	2104	930	842	1904	2401	1468	779	2353	1828	2921	937	1133	755	2453	2919	3782
209239_at	1036	1032	1198	821	966	695	1289	940	1009	770	1212	1111	521	594	581	789	663	757	750	424	538
209240_at	1748	3356	4899	4979	3021	4388	2475	2998	4339	3699	3856	3470	2287	2409	5459	3605	3439	6236	5479	9896	10256
209241_x_at	447	445	657	799	117	351	488	499	443	630	499	334	59	91	88	71	83	125	86	101	82
209242_at	135	103	119	101	253	324	156	136	203	161	112	155	25	5	23	3	3	6	31	6	23
209243_s_at	15	6	52	6	74	71	21	4	13	4	4	4	2	2	5	16	10	12	11	6	17
209244_s_at	443	387	647	445	1058	529	295	359	396	559	387	178	103	345	109	178	139	104	203	169	50
209245_s_at	13	15	15	87	25	30	25	9	13	14	17	49	29	6	2	5	3	5	4	5	39
209246_at	163	147	240	49	379	81	129	141	304	197	236	143	240	227	132	228	118	195	266	175	212
209247_s_at	2381	1249	2844	1390	1711	1364	3154	2121	2914	2166	2156	1162	1195	845	1271	1545	1633	1684	2474	1702	1743
209248_at	3710	3353	5061	5001	4730	7354	4142	3063	3355	4052	4689	4253	7685	8030	11645	9661	8735	9894	9493	14560	16006
209249_s_at	5252	3694	8772	9274	8189	11517	8296	5926	7363	8479	8949	8034	8171	7722	7042	6334	5793	6000	7578	10811	11390
209250_at	697	776	256	244	1078	1365	331	447	308	365	975	651	1591	1376	1444	1767	1597	1568	1096	1807	1441

209251_x_at	26819	17714	26127	9995	28732	19584	25300	26397	32889	21116	22769	30111	17591	24239	22900	15917	18758	20422	13512	14340	11798
209252_at	1376	1376	1294	1315	1034	1050	1263	1507	1263	959	929	809	855	375	473	785	781	687	784	900	972
209253_at	758	1006	263	360	653	102	966	744	322	225	568	359	138	107	227	343	166	350	132	154	88
209254_at	237	300	275	369	113	134	403	533	455	451	384	455	154	97	196	80	59	87	156	230	126
209255_at	448	263	846	1095	469	949	719	625	682	801	783	518	186	174	271	287	224	192	904	977	1264
209256_s_at	296	292	509	961	247	691	477	306	268	465	641	320	397	455	488	447	454	462	866	905	1564
209257_s_at	353	161	454	349	682	333	518	763	1226	1136	887	623	2141	1287	247	361	351	286	470	251	204
209258_s_at	160	21	178	124	289	275	154	236	269	259	277	171	1196	789	162	153	131	121	279	161	131
209259_s_at	1089	521	1426	499	1280	1329	941	1197	1299	1442	1316	912	2026	1091	813	1348	947	1049	2695	2198	2065
209260_at	1864	1929	1578	961	909	1183	1726	1254	1353	1458	1578	1043	628	566	1574	761	769	650	558	274	175
209261_s_at	118	234	60	376	33	54	40	40	252	173	47	19	9	7	5	13	8	47	18	7	9
209262_s_at	1418	1558	1323	2418	188	353	1134	1210	2241	1419	735	676	713	715	679	1193	1241	1661	1702	1301	1101
209263_x_at	787	554	530	358	1380	1355	579	354	351	263	773	593	967	832	401	187	313	383	357	222	165
209264_s_at	442	370	327	93	296	271	468	294	479	187	844	303	951	874	220	184	250	152	161	121	102
209265_s_at	1044	557	849	521	926	802	949	794	1003	784	912	916	1454	2049	2417	1823	1681	1622	1917	1394	1964
209266_s_at	8	50	23	23	19	8	38	12	74	18	14	26	5	12	19	8	17	34	6	6	7
209267_s_at	581	504	501	132	752	310	628	592	559	370	937	636	1260	1104	1487	981	869	1044	1490	1094	2213
209268_at	426	370	570	780	811	692	636	651	435	352	669	488	782	700	807	831	728	786	458	496	601
209269_s_at	17	47	274	243	1414	954	91	38	320	345	1386	371	584	614	527	68	76	58	164	112	110
209270_at	713	1412	1971	2795	1229	1875	566	446	1089	1314	2031	1449	722	655	399	269	458	246	295	1790	1115
209271_at	779	887	1124	1300	913	529	863	1102	796	1275	944	385	490	519	992	1784	1660	2077	1992	1960	3794
209272_at	577	560	1381	1220	1277	1083	611	658	784	1076	1212	1174	1561	1500	2089	1042	889	835	1739	1483	1940
209273_s_at	696	901	792	667	838	949	901	814	863	988	821	819	1431	960	1114	883	891	1169	2401	1101	1427
209274_s_at	1162	1367	1194	864	1886	2044	1337	1264	1342	1532	1303	1534	2620	1516	2375	3354	2667	2722	4105	2072	3695
209275_s_at	743	614	594	1295	359	480	767	635	887	458	567	304	414	250	129	118	189	262	340	310	205
209276_s_at	652	512	716	1134	2264	2448	898	1119	446	795	2005	3612	3115	2474	2107	1374	1453	1281	353	674	585
209277_at	15	9	1	25	34	5	21	4	29	15	13	34	8	4	4	1	16	4	1	8	1

209278_s_at	7	6	7	10	18	17	9	9	13	11	7	8	1	2	12	3	4	2	1	1	1
209279_s_at	702	243	1320	1429	641	572	474	359	1299	932	868	613	1108	926	882	298	399	348	834	907	700
209280_at	29	117	172	225	947	945	272	238	193	162	281	161	68	70	64	29	119	26	67	36	6
209281_s_at	378	293	841	728	341	288	733	862	1068	1206	374	400	311	374	139	190	165	87	305	415	331
209282_at	785	1053	874	909	877	554	790	914	1025	804	1067	466	327	317	268	234	254	260	376	313	276
209283_at	149	94	60	156	110	629	166	74	206	70	274	59	19	58	15	14	32	12	69	62	25
209284_s_at	746	522	1266	991	1343	1437	933	895	815	1044	991	943	1146	947	948	802	654	640	1919	1028	1497
209285_s_at	131	134	190	401	766	954	172	189	242	271	325	366	265	424	306	123	137	123	487	309	365
209286_at	488	449	564	1558	1340	2764	559	747	607	454	983	1139	1342	1589	1365	584	577	541	370	470	518
209287_s_at	333	238	239	962	678	742	298	402	328	330	1124	691	688	484	293	166	162	184	112	101	160
209288_s_at	749	575	571	1711	1405	4087	555	455	343	435	948	1335	2488	1659	1712	1097	1016	1082	860	1067	1303
209289_at	276	552	1464	1549	161	204	350	329	1036	1249	246	160	41	58	155	483	279	342	1859	1862	2500
209290_s_at	217	354	1312	1759	202	224	233	310	1165	1125	173	20	64	69	261	668	606	632	1811	1842	2088
209291_at	58	74	7	49	49	30	61	53	44	8	17	14	4	23	8	69	34	83	10	18	15
209292_at	26	5	9	17	16	59	17	34	41	15	16	19	2	6	1	11	12	13	4	6	2
209293_x_at	128	139	96	151	434	332	125	166	99	137	104	118	41	54	53	78	14	73	46	72	43
209294_x_at	672	769	602	379	249	398	1162	732	615	605	669	386	62	110	104	194	189	165	112	107	58
209295_at	2679	2607	1297	559	1008	2663	1361	1300	1068	1185	1032	1087	1508	1664	1170	2727	2888	2557	1396	2189	2454
209296_at	704	892	1016	943	1057	1063	485	629	862	1055	1022	710	1030	900	1344	1417	1537	1160	2544	2567	3986
209297_at	472	220	400	256	502	219	299	397	332	296	613	387	474	394	388	482	540	643	282	347	391
209298_s_at	173	224	239	107	344	215	114	215	95	109	247	249	206	98	93	61	83	82	124	135	138
209299_x_at	82	181	71	163	109	274	41	48	241	49	215	109	167	140	170	149	152	170	232	139	167
209300_s_at	701	583	633	416	700	1148	831	835	475	562	793	602	760	721	1009	1415	1263	1285	874	514	925
209301_at	1073	1143	231	193	22	22	912	845	96	191	27	11	5	9	2	1196	1080	1049	112	90	110
209302_at	4395	2663	3095	2416	3332	3100	3470	3116	4119	3748	3932	4445	4391	3126	3597	3016	2939	2992	3462	2855	2025
209303_at	1773	1793	1983	2305	1825	2795	2105	2033	1583	1986	1131	1044	1271	1394	1518	3292	3485	3353	2890	2164	1746
209304_x_at	384	1293	151	39	928	2379	217	150	39	20	443	372	414	546	329	225	272	248	23	109	66

209305_s_at	324	577	142	39	537	654	338	264	44	242	451	369	436	440	92	105	52	83	4	29	39
209306_s_at	286	170	506	513	1111	1039	362	434	466	601	1018	812	1110	1266	1057	725	625	595	1508	964	1698
209307_at	274	118	391	402	675	603	260	433	309	411	779	643	1395	1428	831	607	476	414	750	629	806
209308_s_at	684	533	629	344	793	646	698	554	601	449	543	466	1418	410	972	1438	820	1305	1276	967	1792
209309_at	60	23	134	387	107	137	64	9	174	307	99	26	13	23	30	26	30	28	179	347	219
209310_s_at	254	540	349	702	45	349	256	263	221	314	161	87	72	102	123	333	310	345	437	945	765
209311_at	810	732	658	636	576	746	1030	903	851	690	721	342	523	323	219	543	548	407	350	583	430
209312_x_at	355	498	222	172	290	425	511	494	230	240	270	283	62	54	47	133	148	179	39	71	41
209313_at	1745	1325	1827	1933	1421	1701	2030	1023	2080	1501	1288	1631	1699	1537	2129	2083	2294	1776	2683	1722	1891
209314_s_at	536	466	326	233	316	450	425	454	306	264	104	155	557	343	355	1038	759	701	1447	698	1091
209315_at	38	60	171	103	37	38	50	149	36	119	87	15	17	24	21	28	14	49	51	36	27
209316_s_at	1404	1606	1563	2350	1149	1607	2472	2411	1854	1968	1271	1390	1232	1408	1626	2834	2562	2114	3086	1744	2939
209317_at	885	821	692	760	643	693	1034	726	676	751	586	613	596	435	383	701	813	690	717	654	545
209318_x_at	632	766	170	144	671	556	624	1035	80	73	482	788	611	480	644	1033	927	1024	29	64	62
209320_at	450	367	392	334	848	724	453	442	519	679	414	498	335	290	378	224	251	218	350	216	112
209321_s_at	487	513	484	281	715	535	560	442	880	1019	645	691	502	415	417	268	172	259	536	332	338
209322_s_at	341	151	217	263	800	786	252	227	238	302	175	129	77	68	31	44	83	164	161	94	52
209323_at	2302	2257	5008	3306	2345	3056	1560	1854	4164	4063	3183	2211	3293	2441	1934	2673	2631	2150	7022	5844	7205
209324_s_at	259	274	254	198	48	70	232	199	248	278	124	132	71	82	129	105	66	135	170	325	195
209325_s_at	201	254	316	182	357	282	215	168	351	217	171	206	74	36	17	61	66	42	95	157	74
209326_at	804	909	1249	1064	1166	1107	1035	798	1147	1467	1104	948	1692	665	618	856	911	1176	2205	1786	2609
209327_s_at	33	7	4	16	44	40	24	16	11	29	31	18	19	72	3	55	29	44	9	39	7
209328_x_at	176	154	121	233	15	50	94	143	166	64	92	63	62	124	53	66	17	81	61	96	87
209329_x_at	3176	4902	2884	4420	1779	2129	3483	4059	3484	4456	3297	3197	4108	3489	3017	3925	3763	3368	2658	3140	1534
209330_s_at	4227	2791	2656	1287	3241	2377	3514	2941	2349	1912	2993	3078	5096	5199	3428	4168	3059	3373	5193	2712	3791
209331_s_at	934	1008	726	614	871	890	1023	790	1078	804	1047	1101	540	441	238	357	427	270	264	262	373
209332_s_at	1580	2140	1489	982	1732	2029	1554	1541	864	1501	2231	2224	2437	1574	1820	1522	1697	1608	2151	2360	2518

209333_at	172	151	179	292	77	136	221	335	171	131	55	157	53	60	68	73	45	74	57	13	43
209334_s_at	242	123	221	207	327	235	309	263	223	310	297	271	114	58	131	123	67	138	142	124	170
209335_at	12	79	35	65	132	152	34	40	84	66	14	4	15	25	28	20	26	24	11	25	15
209336_at	1155	1073	423	243	579	669	1170	806	851	614	743	454	877	542	507	621	540	532	388	248	248
209337_at	1697	1624	1923	1551	2970	1418	2525	3426	2395	3154	2261	3474	3316	3157	3574	4579	4609	4592	5996	4778	7211
209338_at	705	588	784	1000	869	599	931	863	831	897	753	689	546	583	630	624	814	790	752	614	672
209339_at	624	594	1122	763	187	293	970	647	1262	1034	725	558	268	398	374	548	612	538	606	728	556
209340_at	1410	1785	1917	1313	1061	2051	1308	1182	2212	2006	942	1359	1430	1372	2225	3267	3865	3177	4746	3871	5674
209341_s_at	447	989	395	462	279	262	303	507	266	307	314	364	266	171	153	297	279	337	271	217	254
209342_s_at	124	224	94	66	16	59	154	166	92	136	57	106	63	29	65	12	56	51	52	31	52
209343_at	245	148	171	49	202	169	138	203	16	211	172	78	23	30	45	44	18	54	2	55	9
209344_at	2586	2282	3696	856	1139	782	2141	2832	1147	1596	4053	2131	636	696	549	425	464	232	893	281	405
209345_s_at	1023	788	800	736	968	672	881	1028	712	748	942	866	627	849	596	681	682	1009	408	420	487
209346_s_at	191	60	178	159	49	65	98	227	143	184	341	177	13	51	66	65	106	132	80	52	5
209347_s_at	11	5	22	5	27	78	16	9	12	7	6	6	4	4	3	5	2	2	4	3	3
209348_s_at	44	28	49	52	121	179	46	30	24	39	71	20	26	27	4	4	15	16	22	11	11
209349_at	204	195	393	334	286	112	401	339	403	458	237	44	101	110	113	159	131	137	207	313	256
209350_s_at	1311	1488	1063	1319	887	848	1260	1384	1476	1494	1903	1930	1851	1537	1541	1037	942	838	1311	1007	734
209351_at	66	46	15	25	36	274	13	25	8	11	84	41	46	83	10	16	5	16	22	27	3
209352_s_at	369	720	297	322	62	142	482	446	186	292	311	210	78	58	97	202	156	242	119	79	81
209353_s_at	98	101	75	69	29	17	122	67	112	141	23	85	6	4	5	48	3	49	9	20	31
209354_at	79	27	27	305	331	595	58	87	158	60	68	132	99	80	49	6	25	15	125	125	60
209355_s_at	29	208	23	28	37	44	110	119	70	87	104	72	124	89	83	47	44	31	89	139	111
209356_x_at	61	112	129	156	238	378	85	94	95	101	196	191	81	62	37	13	6	27	13	21	17
209357_at	491	1808	170	241	256	571	338	527	100	105	141	561	290	244	400	1025	941	738	355	593	861
209358_at	829	527	429	534	543	649	775	540	515	464	394	259	965	795	897	1146	1141	1061	1369	777	844
209359_x_at	151	95	80	106	342	292	65	100	175	63	311	155	84	91	83	36	55	89	71	38	72

209360_s_at	685	68	305	538	1553	1610	197	311	364	429	1067	1656	639	636	517	333	241	212	230	438	462
209361_s_at	853	934	879	780	649	515	863	684	506	521	1073	372	300	354	187	182	332	322	231	237	190
209362_at	696	637	1786	1618	2690	1395	1090	1095	1572	1549	2912	2290	3136	3244	4352	1893	1804	1697	2701	1492	2574
209363_s_at	280	330	1035	910	1435	762	685	711	1025	915	1667	1502	1805	2099	1554	552	477	564	959	527	872
209364_at	532	580	595	556	499	626	486	392	475	498	726	447	597	539	391	251	336	460	397	390	212
209365_s_at	238	181	338	406	448	206	216	249	581	517	524	380	114	165	202	91	101	135	214	147	138
209366_x_at	1568	2326	3015	6001	911	1208	1728	1566	3635	3020	891	1013	734	346	839	1401	1336	1348	4223	2187	1952
209367_at	651	1463	589	979	252	380	822	569	591	590	536	351	134	196	395	483	534	515	456	324	248
209368_at	212	320	30	206	220	528	34	135	17	21	109	297	263	243	367	135	143	89	27	11	56
209369_at	2973	3929	3680	2435	4612	7054	1505	1039	1161	1342	4283	2466	5811	5297	6279	10240	10430	11372	6362	6813	7388
209370_s_at	280	203	227	610	253	476	317	207	373	85	131	183	165	174	186	154	144	192	168	179	170
209371_s_at	232	280	290	499	132	226	386	330	408	377	289	239	68	73	81	74	47	74	128	95	124
209372_x_at	529	502	241	56	1728	1953	481	433	376	407	3382	1797	4006	3123	527	235	145	263	221	95	76
209373_at	6947	3545	8402	1716	2691	818	4326	2755	4157	4732	1912	1142	2314	1717	1248	4550	4151	3896	3477	1816	2198
209374_s_at	74	27	60	115	132	189	56	106	47	136	128	85	16	23	74	66	30	15	26	20	32
209375_at	994	943	703	1199	627	730	892	1421	625	1022	976	783	449	367	365	456	564	461	428	428	403
209376_x_at	538	291	708	811	518	585	851	1279	862	910	1131	804	470	498	369	408	269	280	383	393	340
209377_s_at	3974	4735	6232	10211	4060	2242	3358	6453	5413	5438	4775	3372	6706	7828	6406	6124	8874	7239	10469	6722	6698
209378_s_at	269	300	397	215	301	364	326	417	84	252	392	337	183	232	307	248	300	313	152	301	309
209379_s_at	528	416	415	376	206	731	354	429	337	416	300	216	366	404	684	937	792	672	519	899	975
209380_s_at	627	743	621	750	1137	1209	897	856	627	812	1127	1031	557	790	428	351	408	391	231	305	270
209381_x_at	447	571	349	479	70	58	617	690	423	354	406	90	76	143	151	280	310	390	301	274	180
209382_at	630	561	586	654	562	1251	619	554	487	437	746	579	548	629	736	495	578	641	641	746	753
209383_at	945	640	989	594	691	2411	786	596	478	527	379	364	746	1159	797	169	223	1184	76	1547	748
209384_at	1846	2244	1025	1344	1897	1536	1870	1957	959	1114	1978	2260	2068	2111	3915	2965	3124	2543	2212	2183	3609
209385_s_at	1396	1154	1104	1225	1561	1334	1526	1413	728	1099	1774	1512	4035	3357	3188	1295	1375	1195	1733	1992	1803
209386_at	86	90	12540	8665	2378	1441	58	70	10266	8974	1732	1435	4129	3664	2636	9	58	38	11948	13648	14019

209387_s_at	17	11	6297	3152	1473	589	11	62	5381	4036	1035	769	1645	1491	575	11	1	27	5877	4311	4687
209388_at	974	1011	407	480	977	1302	449	558	393	534	726	836	1274	1278	740	1169	1328	1211	1672	1060	1204
209389_x_at	4183	4598	7798	9640	5380	2529	5191	5400	9816	8970	5406	4287	5434	4435	4373	3414	3248	2751	4703	3925	2195
209390_at	579	543	510	751	598	1054	448	421	396	451	489	473	343	428	432	397	353	405	504	541	727
209391_at	1025	1075	482	475	1027	849	954	802	618	668	929	955	1116	955	666	537	386	623	496	463	333
209392_at	85	47	58	21	106	86	73	61	39	52	67	77	17	1	37	25	12	4	14	12	8
209393_s_at	419	457	1077	1231	845	787	1267	983	1622	1016	809	714	1033	633	558	694	534	403	948	751	413
209394_at	852	1251	237	350	183	435	939	870	660	440	402	406	179	165	183	339	395	288	164	83	76
209395_at	12	24	4	27	22	218	12	15	3	13	124	8	269	146	459	2	3	2	1	4	2
209396_s_at	65	28	68	27	91	175	80	45	90	96	199	123	172	138	468	5	2	8	1	2	3
209397_at	2295	3398	2005	1042	693	994	2149	1681	1900	1588	760	721	669	649	1477	3022	2911	4176	3088	2698	3930
209398_at	314	517	205	592	929	1166	314	278	155	221	2847	6192	3877	1906	2170	202	205	211	71	302	153
209399_at	251	369	425	449	239	118	322	391	380	347	286	338	180	223	140	138	141	128	155	110	185
209400_at	22	88	100	164	291	208	24	41	68	68	109	108	28	35	35	33	50	47	43	16	25
209401_s_at	146	225	209	142	392	212	184	219	223	226	327	219	47	11	42	24	50	35	29	18	12
209402_s_at	189	478	282	197	617	552	184	156	76	316	357	342	194	300	88	24	125	94	83	34	13
209403_at	589	1775	779	3295	449	656	472	705	807	1012	443	319	119	168	188	271	305	223	516	779	690
209404_s_at	1549	1154	1200	895	1307	2091	832	900	684	871	1087	1010	1092	633	888	1568	1562	1736	1218	1865	2167
209405_s_at	124	204	438	415	356	435	289	276	340	335	408	224	134	163	124	153	106	102	146	136	84
209406_at	2134	1466	1666	850	1377	900	1770	1692	1561	1638	1329	1077	1748	1725	1658	1842	2085	2052	2730	1410	1797
209407_s_at	585	740	422	457	521	392	431	389	353	488	779	403	287	410	294	208	205	242	182	147	130
209408_at	1971	702	2029	577	2239	404	1860	2245	1972	1957	2328	2350	2347	1781	1241	1071	1032	1134	1743	646	826
209409_at	1409	1721	2096	621	606	2806	1071	703	801	1385	424	494	317	257	996	1036	1360	1453	1014	2372	2855
209410_s_at	434	377	589	340	212	347	493	376	494	565	466	300	111	152	261	148	205	198	182	177	201
209411_s_at	808	816	434	586	349	470	794	847	573	668	557	530	273	262	208	314	202	324	255	144	192
209412_at	275	206	290	326	381	677	213	276	253	401	479	255	361	299	294	279	284	333	517	461	593
209413_at	915	727	770	608	382	492	995	911	974	875	523	349	104	187	104	208	146	244	243	194	91

209414_at	295	341	152	133	463	228	297	281	151	98	298	156	99	87	154	110	149	149	48	35	33
209415_at	229	242	288	242	569	555	158	310	203	208	249	317	134	106	137	122	83	111	100	106	128
209416_s_at	684	538	509	372	882	626	578	715	524	479	675	522	140	150	144	161	166	203	202	119	108
209417_s_at	341	449	376	1119	392	972	388	352	372	591	594	583	327	364	165	198	286	916	241	448	283
209418_s_at	1175	699	1017	607	854	1019	786	644	1088	629	578	622	700	724	759	858	653	896	1277	909	1092
209419_at	5	32	27	48	187	138	61	88	70	81	43	39	10	19	5	4	28	10	18	11	17
209420_s_at	226	154	107	253	466	516	314	219	134	177	658	424	236	373	254	48	17	66	13	60	54
209421_at	1291	785	1639	891	2080	915	1336	1503	1490	1680	1850	1751	2315	2207	1890	1451	1069	1331	3073	1444	2974
209422_at	746	923	435	688	708	1418	493	672	581	668	709	1483	1037	940	1607	1226	1328	1211	1965	2476	3031
209423_s_at	175	122	225	155	351	344	136	255	260	194	260	289	29	52	76	66	48	67	63	61	51
209424_s_at	155	241	222	389	100	222	148	235	242	198	284	254	257	237	312	71	114	66	248	283	294
209425_at	25	82	113	193	231	90	80	123	114	126	134	181	118	144	496	51	85	53	194	365	415
209426_s_at	206	190	270	379	291	255	184	248	276	292	281	289	165	98	505	85	52	91	261	274	315
209427_at	671	595	492	313	125	78	894	876	463	478	703	217	230	207	188	242	201	302	348	139	171
209428_s_at	496	678	769	1000	874	744	718	730	726	799	1040	909	672	385	317	171	197	161	263	230	234
209429_x_at	1830	1051	1416	1324	1482	1558	1936	1452	1529	1338	1670	1374	1593	1739	1622	1465	1346	1313	1225	1233	1087
209430_at	554	462	833	603	638	687	592	682	520	700	459	417	418	492	381	699	461	819	852	793	1009
209431_s_at	430	563	384	450	216	164	488	340	419	396	308	139	204	181	159	230	318	239	315	242	207
209432_s_at	1012	613	1077	947	1185	1720	718	672	676	611	1796	1651	1418	1419	1429	826	912	680	450	938	583
209433_s_at	959	554	1278	524	1295	1325	1435	1221	1314	1444	1282	897	1070	1110	956	1074	931	924	1083	694	960
209434_s_at	955	592	677	233	987	712	810	937	535	865	755	456	807	524	703	1246	1333	1274	1312	766	1305
209435_s_at	949	1207	1621	1959	609	1015	861	780	1038	1959	1127	1010	368	450	561	540	747	750	500	1183	1000
209436_at	365	162	266	215	250	234	284	352	320	358	289	273	51	63	60	48	50	62	65	54	102
209437_s_at	144	29	122	186	296	134	147	125	103	154	166	114	25	38	17	9	3	35	37	42	31
209438_at	319	213	273	338	473	478	151	210	328	326	153	156	107	165	128	148	179	133	272	166	208
209439_s_at	292	535	600	709	521	605	421	402	678	741	520	362	314	245	243	188	260	163	512	392	454
209440_at	2049	1039	2336	1535	2187	1316	2311	1999	3992	3045	3081	2270	2772	3623	3227	3267	3653	3213	5134	2176	2674

209441_at	85	112	46	36	172	105	56	74	111	39	186	132	108	86	15	14	18	7	15	6	9
209442_x_at	238	223	387	428	280	265	260	286	322	418	109	112	29	79	133	149	114	84	266	287	318
209443_at	49	30	58	180	187	435	48	45	52	197	36	45	35	96	66	223	267	193	72	102	63
209444_at	1189	901	746	645	720	634	1275	1367	870	625	809	615	1016	1100	1576	2112	2021	1946	1682	1783	2023
209445_x_at	749	794	1004	649	1755	1275	1997	1371	1189	787	1752	1644	1669	1348	1921	921	776	811	941	404	425
209446_s_at	172	134	313	231	187	122	168	234	269	321	193	136	156	143	124	61	43	80	131	68	133
209447_at	24	9	24	35	22	38	8	32	24	27	11	10	1	2	3	5	35	45	3	85	98
209448_at	1697	2248	2242	3504	2712	7058	917	1006	1545	1597	1921	2505	4639	4969	3832	2070	2215	1909	4987	5975	4682
209449_at	1599	1497	772	666	1064	775	1504	1209	986	934	1143	827	1571	1132	881	1160	1112	954	724	677	385
209450_at	226	208	220	181	597	547	193	157	437	436	361	315	463	363	231	168	71	110	264	180	160
209451_at	467	234	1327	572	495	738	231	392	605	1396	149	410	522	337	194	990	1158	1406	5177	2889	4061
209452_s_at	2565	2730	2296	2581	2610	2170	2837	2354	2584	2060	3471	3107	3261	3724	3481	1529	1903	1260	2018	1501	921
209453_at	93	320	266	502	298	419	142	71	165	242	271	72	137	111	179	235	203	204	239	257	240
209454_s_at	410	448	369	436	393	249	436	480	330	429	298	241	120	141	199	308	267	300	323	242	171
209455_at	1285	1215	1129	1044	1292	1598	1190	866	1193	1168	1478	1342	1216	1333	1665	1841	1817	1588	1590	1359	1485
209456_s_at	692	388	616	476	506	394	685	883	701	826	982	753	71	79	119	99	77	108	81	49	71
209457_at	2615	3412	1747	285	1218	864	1827	1631	491	593	2034	1594	685	749	612	2133	2278	2777	164	352	263
209458_x_at	298	36	202	198	194	661	127	55	108	310	207	44	69	77	227	55	94	60	181	82	52
209459_s_at	138	214	18	389	510	978	111	120	1	1	591	1204	509	389	340	309	308	229	30	134	209
209460_at	83	154	54	467	231	357	201	143	11	102	877	1262	308	432	229	142	260	294	29	81	80
209461_x_at	870	674	686	300	82	83	933	637	480	482	264	58	570	421	183	627	564	735	630	493	440
209462_at	242	309	15	21	52	42	374	388	12	15	61	23	103	101	24	282	286	305	6	40	12
209463_s_at	365	448	349	343	52	179	513	548	185	384	367	608	690	462	332	659	658	660	300	406	355
209464_at	861	100	1080	237	2012	269	967	1203	1203	1325	1961	1690	2472	1546	958	550	530	814	1457	360	339
209465_x_at	26	7	19	41	22	82	5	37	55	41	23	8	15	11	2	5	34	8	20	21	18
209466_x_at	407	291	282	517	1421	904	484	529	435	380	335	332	44	58	60	61	41	49	62	65	25
209467_s_at	975	1174	993	1244	649	1170	1230	1190	745	1558	793	812	291	270	609	1102	677	715	725	589	482

209468_at	372	811	1438	3016	440	208	391	393	1049	1760	641	207	98	155	258	147	199	212	531	517	545
209469_at	52	91	1	17	4	5	50	66	4	14	3	4	1	33	1	176	215	227	3	1	10
209470_s_at	119	128	9	28	74	26	85	104	47	52	51	19	25	32	7	200	212	171	18	1	13
209471_s_at	848	1092	1028	863	1138	853	1034	1297	809	952	1370	1002	1677	2304	2113	1896	1907	1406	1295	1679	1950
209472_at	541	768	897	1214	775	1007	868	1012	673	1041	561	513	815	971	1134	1884	1792	2103	2060	1456	2377
209473_at	119	94	176	237	383	511	122	256	252	184	185	132	51	33	13	97	15	122	73	112	34
209474_s_at	42	9	56	10	209	52	122	51	54	70	54	38	18	4	8	2	3	24	20	6	5
209475_at	364	350	410	454	315	341	546	442	550	491	314	368	186	192	287	435	370	295	567	430	741
209476_at	3589	2819	2615	2956	3297	3296	3885	3729	3335	3230	3640	3565	4556	4067	4077	6929	6151	7258	6502	5110	7844
209477_at	1124	1317	1645	1260	1175	1074	815	829	1477	1350	935	868	1008	601	443	434	519	385	805	579	383
209478_at	2944	1850	1310	1033	1471	1376	2564	1834	2886	1987	2349	1993	2679	1308	967	1961	2708	1650	2762	1225	738
209479_at	438	393	1236	2201	1171	505	493	823	1044	1399	583	1286	528	552	504	522	589	548	1129	1102	1081
209480_at	13	9	9	16	209	333	46	59	59	4	126	150	99	137	184	3	3	3	1	3	9
209481_at	725	702	528	629	609	1184	697	875	542	706	920	946	617	771	672	780	706	835	675	535	670
209482_at	1593	1775	1888	997	2067	648	1662	1396	2392	1732	3466	3211	4304	2936	3036	1773	1701	1318	2559	1823	972
209483_s_at	79	21	39	9	157	212	11	83	50	28	23	30	22	8	69	54	45	71	60	27	33
209484_s_at	1803	1298	890	1128	1373	1084	1416	1291	947	1013	871	1470	1169	1055	729	2126	1555	1783	1964	1857	1877
209485_s_at	156	245	425	432	539	907	288	231	415	604	610	857	1071	836	408	280	305	228	435	402	573
209486_at	495	416	650	610	803	714	1041	1004	924	851	824	674	1412	1069	530	834	886	642	693	475	351
209487_at	263	698	396	706	187	289	256	194	416	335	179	170	242	199	246	245	229	226	1170	1230	1000
209488_s_at	270	628	658	1434	136	40	368	302	682	583	313	153	155	216	177	144	186	162	571	533	326
209489_at	1648	1657	1944	1534	1046	1033	1673	1492	1868	1884	1316	1019	661	1110	1138	1888	1774	1875	2138	1961	1893
209490_s_at	446	409	473	419	151	232	613	418	515	222	745	491	144	218	443	180	206	252	175	139	143
209491_s_at	197	167	178	176	252	230	159	210	175	180	220	238	19	13	25	40	29	64	16	35	17
209492_x_at	5137	3885	2454	2990	2618	2924	3503	3847	2530	2312	2419	2360	6511	3951	1893	2135	2400	3483	3879	2289	1196
209493_at	19	15	53	59	669	1090	13	8	50	10	812	806	364	323	605	19	7	25	4	5	1
209494_s_at	493	472	408	529	349	251	769	600	482	439	402	327	218	203	317	255	416	279	343	241	236

209495_at	540	485	551	512	335	395	616	734	610	537	641	613	259	260	199	173	125	150	190	147	143
209496_at	26	23	42	597	401	934	16	61	37	31	270	139	454	145	102	5	7	5	146	72	35
209497_s_at	590	214	646	847	590	403	482	483	621	710	493	382	357	354	387	594	728	691	599	691	701
209498_at	58	55	825	1130	305	247	107	67	949	1364	378	255	89	139	222	84	76	206	1356	1623	2386
209499_x_at	171	140	629	665	302	380	268	240	520	366	490	207	196	190	121	67	78	50	229	257	184
209500_x_at	210	234	720	1079	279	447	274	263	868	548	662	366	258	267	311	129	167	127	737	989	464
209501_at	982	1062	1369	532	614	693	712	685	895	1265	567	718	527	434	615	1283	1300	925	1788	1422	1652
209502_s_at	347	646	263	278	487	374	497	282	244	177	212	480	235	187	231	372	418	287	213	240	199
209503_s_at	5551	2272	3098	2875	4485	3342	4227	3438	3586	3644	5108	5241	6448	6930	5946	5189	4802	4764	4262	4239	2993
209504_s_at	438	678	689	2034	437	1150	416	533	537	1795	866	806	405	270	404	309	398	269	507	907	535
209505_at	1323	1482	696	601	1065	2254	382	549	720	275	605	190	1209	987	1580	6939	6146	8330	1391	806	2231
209506_s_at	1000	652	473	370	819	1059	372	399	559	305	505	225	261	322	260	549	481	470	141	185	70
209507_at	1832	976	2471	1376	3605	1091	2703	2519	1572	2559	4308	7297	9357	9843	6251	3070	2685	3664	3935	1737	1628
209508_x_at	181	230	304	458	438	447	203	214	266	312	311	312	103	103	108	87	103	70	103	162	132
209509_s_at	672	375	1047	713	1167	1141	742	637	1070	1111	1157	868	1251	1226	1468	778	832	854	1169	1079	1281
209510_at	1181	1754	2037	1835	741	711	1487	1726	2474	2049	635	603	710	901	1510	3250	3741	2864	3575	2780	3491
209511_at	2369	1158	1473	748	2346	1368	2243	1165	1915	1399	2979	1247	4820	2312	1957	935	963	648	997	749	348
209512_at	347	269	1328	1300	691	636	590	703	1363	1617	739	916	498	312	297	247	192	211	1194	814	981
209513_s_at	459	516	1597	1846	1017	801	779	810	1433	1878	904	1269	1411	1507	1186	846	820	847	2512	2413	2706
209514_s_at	447	418	495	667	625	468	513	421	646	440	647	535	271	401	297	345	279	300	434	436	373
209515_s_at	232	113	202	437	258	181	248	199	285	177	210	187	170	180	245	320	295	236	383	372	494
209516_at	489	321	80	52	301	288	159	186	470	59	398	131	143	322	316	361	449	330	363	188	173
209517_s_at	1668	1103	1214	918	1654	1695	1940	2143	1164	1400	1525	1369	733	965	1189	1928	1592	2051	917	713	913
209518_at	540	422	494	592	364	437	734	698	809	809	1143	506	396	567	942	595	648	596	666	503	551
209519_at	29	2	114	28	137	90	73	55	103	166	75	75	87	82	60	35	38	63	108	51	116
209520_s_at	747	446	1210	537	1593	1019	991	760	1228	907	1246	1242	1334	1007	900	546	424	734	1184	941	1232
209521_s_at	105	22	73	76	307	160	143	58	62	123	125	101	11	43	11	40	30	4	19	19	29

209522_s_at	343	421	172	925	1359	1607	326	343	312	426	486	824	775	589	610	163	184	186	291	334	329
209523_at	2184	2171	3390	2660	1391	1157	1561	1520	2454	2717	1072	984	1589	1392	1994	3755	3087	3692	6367	5272	6790
209524_at	4	5	7	12	1049	1056	5	5	7	4	812	633	1119	804	1050	3	5	8	63	221	312
209525_at	26	4	75	38	113	168	69	66	62	71	163	204	130	67	66	1	3	2	2	25	20
209526_s_at	152	97	142	102	1073	1107	139	145	154	99	1536	1015	1108	929	1107	23	11	44	55	211	203
209527_at	579	528	435	238	770	551	567	602	933	591	675	572	688	769	1150	854	940	886	1159	805	735
209528_s_at	214	192	157	79	30	54	225	51	265	217	227	134	194	222	171	191	160	272	270	204	108
209529_at	2166	2280	1652	1803	669	633	2362	1967	1888	1635	1865	675	1341	1164	783	1082	1007	1042	763	675	573
209530_at	198	302	277	440	114	124	342	232	130	303	379	243	97	153	173	118	186	73	159	120	90
209531_at	243	219	194	213	529	708	345	524	391	505	544	796	867	772	243	255	370	256	298	269	274
209532_at	163	122	194	204	158	204	302	323	270	274	145	187	71	36	30	42	33	51	102	64	62
209533_s_at	376	339	822	614	564	517	563	492	969	681	759	778	886	755	625	440	402	430	1446	1021	1313
209534_x_at	452	463	705	1045	584	691	301	434	494	618	414	353	20	46	77	136	157	112	200	266	180
209535_s_at	144	108	292	301	307	430	156	234	347	292	259	315	89	112	111	108	121	78	263	94	108
209536_s_at	2195	1780	2191	1597	2647	2643	1627	1623	1912	2078	3226	2399	2539	2520	2545	1941	2181	2080	2032	1274	1604
209537_at	614	417	1040	931	592	546	1403	1279	1180	1128	634	544	379	542	731	1986	2297	2267	1428	1072	1695
209538_at	366	404	490	361	437	609	375	273	355	249	262	368	202	179	196	297	445	255	233	388	318
209539_at	200	175	91	39	252	163	223	174	37	154	146	150	47	64	9	156	154	109	44	16	25
209540_at	40	12	31	25	91	26	13	91	78	32	14	15	23	13	46	2	15	4	3	17	22
209541_at	12	21	91	71	181	102	42	92	74	95	44	48	21	23	66	6	4	16	18	4	3
209542_x_at	48	19	34	49	32	113	29	15	25	20	13	80	54	59	25	2	3	48	52	3	9
209543_s_at	239	118	146	156	444	409	170	245	252	277	242	244	43	8	57	22	12	4	16	18	58
209544_at	220	130	130	32	117	332	148	103	62	95	55	79	208	143	139	181	181	183	196	202	355
209545_s_at	2224	2389	1251	649	731	1059	1483	1609	880	723	1143	1013	561	593	959	2112	1943	2297	897	1201	1608
209546_s_at	38	29	52	883	230	504	33	36	66	45	87	133	17	92	32	13	56	15	80	139	118
209547_s_at	66	56	81	160	98	42	57	61	51	73	64	94	151	369	149	132	76	49	107	238	37
209549_s_at	2647	1993	1792	1717	2278	2407	2532	2348	1718	1971	2214	2180	2848	2432	2323	3244	3256	3501	2701	2073	2000

209550_at	79	124	24	128	109	38	174	125	70	250	205	192	8	32	14	5	8	8	28	27	41
209551_at	228	133	182	239	344	615	81	103	166	240	179	239	361	328	285	352	246	314	629	467	828
209552_at	155	77	30	75	33	23	105	123	41	106	188	173	102	144	97	110	74	121	41	71	61
209553_at	722	485	418	697	738	1455	386	596	334	347	678	912	522	463	323	287	322	322	346	447	442
209554_at	77	7	23	63	293	34	41	29	60	53	65	10	18	6	3	9	6	23	4	36	2
209555_s_at	17	90	5	54	11	22	32	70	48	18	6	16	7	30	31	23	34	22	25	31	3
209556_at	464	392	536	389	155	164	712	348	617	535	298	199	74	72	119	168	143	96	193	119	89
209557_s_at	57	168	117	34	146	74	57	55	36	52	50	36	20	53	5	9	7	12	16	28	7
209558_s_at	631	802	419	647	91	203	519	661	591	697	250	60	132	194	81	316	308	252	372	261	234
209559_at	45	51	90	173	78	63	110	140	79	147	48	54	26	84	61	13	158	54	15	56	6
209560_s_at	65	97	144	4	157	204	126	83	83	131	70	133	4	25	50	28	6	37	36	49	37
209561_at	204	220	77	410	58	258	167	128	157	143	139	106	48	47	40	69	65	51	57	90	111
209563_x_at	6111	8294	5351	8615	5733	3373	5662	6144	5646	5964	6772	7415	7070	9078	10054	9334	8967	11805	9160	7068	7571
209565_at	423	388	992	921	1069	1103	392	322	844	703	1289	1384	1156	1320	623	234	184	166	463	1079	595
209566_at	279	431	859	1014	1278	1751	378	371	621	616	762	794	825	714	1215	632	682	624	732	908	1321
209567_at	2014	1617	832	503	1227	1399	1130	1244	1327	1414	1057	1008	1563	1045	762	1566	1786	1333	1093	702	711
209568_s_at	310	277	283	263	469	418	293	351	265	324	377	424	243	265	291	85	65	109	114	187	202
209569_x_at	177	95	125	173	278	325	168	154	218	208	286	232	89	50	84	16	24	13	19	36	33
209570_s_at	32	38	38	43	488	388	45	53	60	18	82	114	64	60	70	5	20	47	34	24	34
209571_at	168	185	235	290	739	1017	233	284	257	236	287	294	217	294	373	241	252	253	304	335	282
209572_s_at	950	867	1409	1298	1747	1540	1059	779	2063	1758	1659	1562	3059	2442	2015	1457	1323	1531	4178	2778	3746
209573_s_at	74	12	46	101	734	468	40	94	41	43	99	14	14	8	25	20	10	45	17	7	25
209574_s_at	155	55	138	140	208	253	135	157	149	173	176	102	31	8	16	29	35	37	36	41	8
209575_at	534	376	307	378	950	1445	274	335	170	221	797	572	861	947	766	604	571	424	465	531	501
209576_at	1574	2676	236	356	106	30	1170	1890	321	456	78	57	530	485	1015	3424	2795	3228	1009	710	1238
209577_at	696	886	581	998	895	1619	688	661	835	655	770	390	603	509	863	460	747	429	569	512	509
209578_s_at	499	423	507	524	757	888	366	406	332	356	333	341	137	249	262	249	371	333	242	163	197

209579_s_at	474	567	1446	1491	683	688	606	653	1805	1305	1457	1534	1305	1276	1426	902	891	928	2528	1971	2130
209580_s_at	426	449	1257	1182	1116	822	399	347	1262	970	935	720	833	766	670	555	385	722	1871	1150	1982
209581_at	978	736	5471	4886	1024	1984	796	560	5134	4910	696	1068	785	755	610	561	808	482	4243	5205	2523
209582_s_at	115	71	79	144	158	188	126	92	135	145	99	119	23	8	13	15	3	40	27	21	8
209583_s_at	34	66	31	56	263	31	40	11	41	15	71	24	13	18	7	8	8	3	34	17	6
209584_x_at	516	203	123	200	2492	1007	629	454	67	77	1734	1688	1958	1779	3052	1098	1049	604	115	218	207
209585_s_at	1278	1678	701	1172	682	809	787	1085	413	906	523	647	839	709	564	1688	1501	1290	1437	1905	3075
209586_s_at	541	495	612	1218	621	794	1044	1186	872	955	686	703	405	381	442	629	756	853	721	629	675
209587_at	69	26	145	282	205	305	174	98	231	115	269	94	218	166	253	96	54	69	79	85	52
209588_at	409	315	246	239	532	568	362	380	277	263	307	250	104	155	82	187	163	140	235	139	151
209589_s_at	311	493	222	169	274	751	298	362	268	239	301	312	180	171	96	140	180	171	362	352	218
209590_at	237	209	183	87	1440	1189	270	260	100	28	1769	1199	1457	1084	1292	220	130	136	60	165	149
209591_s_at	181	68	96	120	1137	500	224	235	19	109	3455	807	1229	1104	1439	65	43	58	3	53	49
209592_s_at	697	481	412	246	601	331	865	1023	498	500	860	535	406	434	484	815	620	662	374	317	249
209593_s_at	620	516	703	837	1339	1156	566	765	535	894	1052	1122	823	409	638	303	428	325	415	570	701
209594_x_at	255	260	293	287	608	1270	307	304	313	338	568	297	462	337	314	56	48	53	81	64	42
209595_at	1263	1084	496	178	1249	759	542	620	365	368	389	736	986	535	441	739	544	515	475	282	277
209596_at	102	47	172	128	67	31	103	165	157	126	158	53	20	19	12	25	36	59	33	41	7
209597_s_at	21	16	14	30	26	17	21	16	59	70	38	137	19	11	6	2	2	3	11	4	5
209598_at	110	74	165	94	749	676	162	148	87	64	664	1213	597	1039	734	82	105	42	19	34	9
209599_s_at	28	57	138	211	197	336	200	252	205	182	172	147	32	71	9	47	43	95	74	39	70
209600_s_at	675	747	511	693	515	722	719	808	519	862	370	470	495	313	342	873	807	727	976	594	836
209601_at	21	19	43	27	45	30	17	32	15	27	13	18	6	4	3	2	3	8	5	7	9
209602_s_at	7	7	8	89	7	9	24	5	11	8	10	4	23	29	12	15	15	40	38	84	169
209603_at	16	9	24	133	21	13	46	34	80	18	17	18	3	45	60	22	8	58	71	116	137
209604_s_at	183	226	387	864	140	324	292	296	213	328	198	275	106	84	176	122	105	207	397	500	513
209605_at	996	1721	1416	4110	1314	1308	655	1107	1426	1754	1293	1850	1872	2274	2362	1447	1807	1063	3134	7400	3250

209606_at	20	23	39	114	27	63	15	69	44	81	28	72	11	6	6	4	19	24	16	29	25
209607_x_at	1908	1646	855	1218	896	998	2090	1469	809	853	885	733	604	707	540	874	1161	891	339	387	288
209608_s_at	1643	449	2391	991	2401	1047	1150	1211	2078	1433	2894	1807	3070	2554	3406	1177	953	714	1834	1509	1068
209609_s_at	1953	2033	2189	1450	1754	1656	1719	1690	2602	1765	1885	1307	3942	3438	3911	3866	3496	2917	4205	4384	3907
209610_s_at	265	298	1076	719	755	1763	272	193	450	408	1036	998	642	687	1232	337	371	238	523	1155	1190
209611_s_at	251	253	441	405	129	429	245	256	324	344	526	373	62	77	99	52	52	45	62	94	72
209612_s_at	176	43	28	23	36	31	77	33	56	35	172	21	2	12	22	18	26	3	6	16	2
209613_s_at	5	6	11	25	27	5	7	15	4	11	3	5	10	1	12	20	17	2	1	2	1
209614_at	11	15	15	16	98	44	23	21	20	31	21	77	2	13	3	2	2	4	4	11	3
209615_s_at	135	36	292	272	4	15	110	189	344	326	53	44	22	3	3	72	83	136	198	150	324
209616_s_at	123	28	148	31	34	74	76	16	86	22	14	15	4	29	31	21	39	29	39	4	4
209617_s_at	16	83	18	48	124	183	91	75	20	78	43	75	37	19	30	27	6	32	25	17	14
209618_at	19	43	15	56	58	136	25	25	9	38	60	30	2	4	3	4	4	2	2	2	1
209619_at	209	113	130	132	392	582	156	157	110	101	1390	1149	109	189	259	42	42	59	4	4	21
209620_s_at	429	438	621	551	603	829	529	352	541	516	703	457	936	795	1127	850	600	701	1022	1018	1376
209621_s_at	251	252	102	47	308	185	228	228	29	59	334	299	228	473	314	99	179	69	39	30	33
209622_at	705	460	525	670	713	542	599	532	381	532	644	523	490	342	298	284	274	372	266	293	290
209623_at	1457	1432	3443	3873	1744	1152	2075	2233	4661	5391	2867	2092	2099	1863	4245	2450	2527	2714	5634	4542	6616
209624_s_at	418	196	1082	813	581	555	641	672	1244	1495	1005	628	262	438	590	364	346	480	959	483	807
209625_at	434	457	315	193	819	1093	355	418	214	250	549	984	523	425	207	279	338	342	227	190	206
209626_s_at	474	330	385	211	1681	1117	596	537	396	338	2093	1386	1436	1350	852	530	488	715	351	311	327
209627_s_at	456	541	454	444	1364	1261	738	734	455	411	1528	1756	1967	1918	2918	1472	1477	1317	745	735	917
209628_at	565	472	454	379	620	554	635	755	502	500	657	778	1233	1327	1516	1824	1110	1673	1770	903	1695
209629_s_at	105	130	167	278	392	275	673	487	412	426	486	703	116	73	79	133	96	114	110	63	138
209630_s_at	910	842	730	1355	889	1283	920	949	856	1118	962	1020	828	805	1283	1261	1162	995	1319	861	967
209631_s_at	119	116	134	182	12	73	99	80	95	134	6	29	1	8	2	209	127	128	189	253	526
209632_at	78	75	103	193	194	302	93	123	47	70	148	168	242	130	41	45	48	36	56	25	44

209633_at	148	176	190	462	274	603	220	265	222	302	387
	555	528	491	499	445	501	412	286	195	299	
209635_at	1511	1225	1850	2041	1050	747	1104	1267	1737	1537	1123
	625	1406	711	538	589	483	579	742	825	458	
209636_at	37	291	73	102	102	227	191	205	78	17	144
	53	66	106	68	103	66	118	58	28	15	
209637_s_at	230	259	228	250	210	294	301	317	198	306	236
	280	27	39	30	38	8	15	5	6	13	
209638_x_at	34	4	61	50	399	474	4	54	58	34	33
	23	31	15	18	10	2	9	22	38	8	
209639_s_at	98	176	14	17	114	134	90	145	15	74	117
	55	39	48	81	35	38	23	49	39	13	
209640_at	302	235	178	286	364	332	195	234	221	243	209
	288	59	24	48	51	35	61	59	99	101	
209641_s_at	188	1016	1898	3920	605	821	74	430	1840	3669	411
	555	127	216	78	200	255	168	591	841	775	
209642_at	1098	264	897	175	1949	171	1321	1097	1296	1240	1984
	1699	1955	1409	1409	907	867	1425	2099	417	943	
209643_s_at	456	681	458	546	841	536	549	765	532	565	422
	392	95	258	176	258	291	279	213	200	168	
209644_x_at	1450	1697	4254	2740	2077	1927	1308	1222	3954	4232	1289
	1250	1643	983	1586	1452	747	1396	4088	2267	1924	
209645_s_at	767	456	273	261	588	562	714	590	631	569	573
	353	141	166	178	117	143	183	99	47	78	
209646_x_at	556	314	193	94	264	396	404	263	181	106	142
	192	255	140	331	674	424	399	529	180	326	
209647_s_at	455	668	551	704	1133	1063	1053	747	819	777	1727
	1150	1265	1240	1523	1144	1211	995	754	719	1107	
209648_x_at	253	270	125	181	388	636	207	238	186	123	307
	361	675	645	352	634	517	555	532	370	445	
209649_at	302	214	320	307	217	550	211	261	282	340	237
	376	666	468	464	461	461	394	1222	927	1486	
209650_s_at	49	236	46	120	67	146	58	53	28	46	43
	225	180	145	152	31	106	155	146	147	229	
209651_at	270	326	220	233	630	693	212	323	123	225	1229
	532	1133	1023	960	418	413	350	166	286	180	
209652_s_at	30	24	14	13	164	74	155	107	39	77	51
	62	4	32	9	68	75	26	36	54	5	
209653_at	382	315	704	539	674	904	531	562	1094	1099	2091
	1468	795	602	818	364	255	315	622	464	513	
209654_at	1409	1333	1332	1024	2092	1756	937	940	1409	1347	1141
	1058	1283	1406	1802	1951	1773	1724	2464	2325	2997	
209655_s_at	12	4	85	43	340	826	11	30	25	7	374
	422	935	1060	841	2	4	6	23	119	153	
209656_s_at	29	4	7	41	1592	3271	8	17	7	17	1104
	1487	5502	5124	6750	2	26	15	217	1169	1307	
209657_s_at	406	355	258	401	293	462	582	837	450	355	418
	382	507	390	419	810	620	829	796	760	1096	
209658_at	906	1575	1074	821	1548	2607	1036	996	689	1097	868
	1732	1848	1580	2433	1522	1526	1211	1627	1991	2640	
209659_s_at	1302	1892	1095	1156	1721	2620	1150	1161	1159	1163	1541
	1810	2706	2489	2724	2068	2080	2083	2024	3225	3942	
209660_at	94	24	31	84	282	99	44	28	82	94	36
	80	41	11	43	49	38	15	10	39	4	

209661_at	175	190	334	256	96	308	388	264	401	379	327	133	5	96	120	152	136	111	34	112	32
209662_at	1376	1193	891	450	1222	969	1159	1396	537	861	860	971	2059	1883	1960	3556	2835	3550	2650	2060	2233
209663_s_at	29	86	23	62	371	56	21	36	20	22	419	72	131	102	127	16	35	28	64	104	38
209664_x_at	12	7	9	10	19	27	9	9	5	7	16	10	5	7	2	3	3	3	2	6	3
209665_at	652	493	530	733	614	990	509	525	606	731	947	857	897	740	421	231	322	270	318	222	169
209666_s_at	812	558	480	221	603	472	314	474	364	407	290	284	599	468	592	1732	1537	1621	2297	1094	1730
209667_at	594	777	540	905	467	927	545	747	512	650	523	525	356	504	526	736	790	761	731	1184	1491
209668_x_at	663	959	633	1071	661	839	975	1151	583	1008	722	646	308	320	272	221	254	220	214	373	348
209669_s_at	4400	3752	2922	1948	2998	1828	5365	4075	4928	4734	3029	2537	6919	6727	4427	4333	3955	3847	5542	3738	4500
209670_at	28	19	210	153	77	202	70	176	37	157	34	31	24	61	7	45	16	20	50	4	6
209671_x_at	120	44	80	132	238	149	126	132	102	59	72	45	5	14	7	40	7	41	37	49	25
209672_s_at	71	41	57	25	70	167	17	75	41	78	38	111	59	76	54	89	60	47	106	76	110
209674_at	546	690	827	199	841	1459	288	420	555	586	816	459	1739	1283	1361	1111	661	753	2155	2248	2937
209675_s_at	1035	720	1569	1480	877	781	794	980	1603	1553	1323	494	418	253	105	145	162	150	409	247	185
209676_at	91	192	346	479	62	86	89	162	304	285	17	41	3	9	1	207	156	193	435	556	900
209677_at	87	6	347	268	308	284	135	230	222	355	310	235	114	103	50	59	46	23	178	72	153
209678_s_at	611	330	2703	2532	1603	1935	686	748	2794	4070	2842	2907	1757	1611	2410	254	170	215	1704	1200	1321
209679_s_at	983	1171	2691	2967	636	645	1248	969	2562	1701	1251	1037	1037	1027	1385	1109	967	846	2590	1850	1627
209680_s_at	1003	491	789	199	929	30	930	1192	1044	949	1288	739	446	425	443	392	453	398	485	119	141
209681_at	411	404	967	709	176	292	395	360	797	630	229	180	216	249	527	1244	823	831	1581	1291	2062
209682_at	212	221	187	410	135	184	166	305	158	224	101	98	36	17	29	234	243	249	144	313	511
209683_at	12	7	7	6	15	411	9	12	4	7	38	6	1	2	15	1	6	7	2	3	3
209684_at	57	122	452	817	503	1919	144	66	321	292	1019	633	844	1535	1262	94	96	80	1463	1361	2243
209685_s_at	171	89	149	210	370	308	158	139	169	179	146	137	37	50	36	30	7	12	36	49	31
209686_at	16	11	15	10	73	23	11	13	23	11	20	19	2	4	1	3	7	2	4	5	3
209687_at	38	89	37	8	135	36	81	17	7	43	11	34	2	2	17	29	10	1	10	1	18
209688_s_at	394	303	598	680	480	758	407	409	574	657	547	883	437	442	371	248	225	266	477	467	562

209689_at	147	142	199	269	67	54	195	176	146	250	300	405	199	217	98	114	108	70	135	194	138
209690_s_at	160	105	273	319	88	156	205	240	363	319	230	118	93	43	33	41	59	61	118	86	62
209691_s_at	54	175	330	953	43	52	237	265	510	570	153	108	194	210	247	201	188	168	665	375	303
209692_at	28	15	31	25	80	97	34	24	20	28	26	30	16	6	3	4	4	9	2	6	4
209693_at	90	64	58	47	357	347	118	95	67	77	122	82	126	121	81	89	91	70	80	36	40
209694_at	2405	2468	2311	1419	2377	1720	2278	2250	1627	2045	2892	1808	3662	2951	2853	1878	2033	1508	1905	967	933
209695_at	411	403	282	415	396	297	345	428	452	334	273	325	31	85	174	110	146	217	137	193	134
209696_at	159	169	1068	5904	216	194	240	231	1567	2868	247	259	81	143	69	66	34	28	1871	1698	1257
209697_at	128	96	87	133	267	294	106	24	86	78	60	73	6	10	31	33	11	35	16	9	15
209698_at	282	483	72	343	197	113	388	261	45	198	503	480	259	264	192	86	31	50	115	108	45
209699_x_at	173	210	419	5697	38	23	182	189	409	764	203	200	100	68	119	39	90	59	463	824	669
209700_x_at	1	1	11	41	8	70	5	1	7	13	1	20	3	20	1	20	2	11	8	7	1
209701_at	257	147	243	190	515	637	330	249	269	293	306	182	52	62	129	138	97	101	137	99	70
209702_at	955	726	760	1516	899	915	1236	1207	892	742	755	989	919	1000	1421	2298	2320	2494	1531	1654	2088
209703_x_at	329	140	229	477	524	577	253	216	214	218	166	232	75	76	98	77	89	107	70	97	118
209704_at	228	136	191	208	309	280	345	369	231	240	139	176	199	274	161	571	496	523	437	255	511
209705_at	474	534	606	564	433	427	1032	764	741	822	530	517	180	180	179	850	1046	706	418	248	240
209706_at	348	566	279	356	465	590	362	557	328	407	368	503	246	266	318	297	293	288	370	287	278
209707_at	586	370	606	476	394	501	656	632	508	576	478	279	550	377	676	896	806	916	1248	1042	1427
209708_at	251	227	157	114	327	216	257	185	171	233	185	148	24	28	52	72	45	77	70	41	48
209709_s_at	777	283	1268	308	2273	345	1483	2015	979	1147	1953	1661	2194	2183	2894	1929	1376	1870	2905	919	1945
209710_at	757	774	199	275	2271	1786	758	914	157	171	928	591	897	720	418	379	263	513	253	378	308
209711_at	310	268	307	237	511	375	342	314	209	274	301	381	260	288	215	229	169	249	320	284	409
209712_at	393	275	283	228	353	396	339	355	249	256	409	435	301	335	306	410	351	465	275	293	332
209713_s_at	81	60	30	13	37	17	41	59	63	39	90	54	49	63	48	58	49	26	37	22	22
209714_s_at	1195	564	1860	579	3181	949	2442	2313	2949	1845	3515	3681	6000	5500	3323	901	1029	1049	2399	502	563
209715_at	212	58	338	195	366	118	242	260	470	232	485	533	742	561	637	388	401	398	814	419	332

209716_at	163	198	202	147	282	370	195	195	165	109	141	259	44	67	70	109	190	125	53	86	74
209717_at	341	307	536	233	338	574	212	347	341	369	207	162	600	542	1168	1148	940	743	900	1976	1174
209718_at	275	182	172	191	414	441	158	117	225	164	61	294	53	106	122	131	166	141	164	90	173
209719_x_at	167	86	197	93	268	231	174	157	179	204	166	113	31	48	59	16	15	42	43	59	33
209720_s_at	13	28	11	13	44	4	36	4	8	4	3	8	1	1	1	11	2	1	1	1	1
209721_s_at	142	142	130	180	106	106	119	165	136	130	172	191	5	14	8	11	11	6	8	7	3
209722_s_at	180	122	91	123	243	129	144	160	80	73	124	178	19	37	46	22	6	50	31	4	3
209723_at	980	809	220	176	866	625	1153	1229	329	586	1070	997	856	1019	1005	1721	1831	1466	672	351	399
209724_s_at	246	200	267	193	289	372	193	191	261	186	216	312	181	105	154	269	203	123	266	252	333
209725_at	820	269	895	547	1054	751	734	515	1014	1006	760	635	822	665	326	173	260	376	677	311	382
209726_at	353	401	75	356	405	371	219	474	199	250	340	60	136	97	60	224	292	176	90	78	86
209727_at	269	36	106	251	51	528	678	661	253	296	290	421	55	24	79	75	78	48	72	11	7
209728_at	21	9	19	30	14	13	19	26	11	18	20	16	2	2	1	4	3	6	3	2	1
209729_at	561	444	486	566	164	202	441	544	484	450	297	234	110	103	168	356	256	347	420	171	150
209730_at	30	52	53	66	124	242	50	110	72	45	77	41	53	25	109	44	115	170	47	72	88
209731_at	468	519	429	459	334	439	367	340	657	425	227	177	339	317	351	370	274	289	758	360	264
209732_at	64	74	11	65	360	298	86	17	17	8	54	44	34	42	9	16	10	40	25	22	9
209733_at	102	180	132	176	302	461	164	108	114	77	354	191	142	131	216	154	116	76	32	72	41
209734_at	222	46	115	128	322	312	101	144	158	45	193	98	12	39	10	84	12	65	54	6	11
209735_at	263	320	564	489	164	83	382	321	954	1465	82	231	40	30	42	136	146	151	925	511	686
209736_at	336	377	437	585	463	480	429	437	605	1133	804	405	177	164	330	72	193	245	309	251	247
209737_at	83	62	39	57	195	132	82	104	68	59	109	194	85	68	129	18	45	30	17	30	33
209738_x_at	204	280	174	275	477	843	179	198	185	266	284	288	245	348	253	82	55	51	65	36	41
209739_s_at	523	657	20	30	390	923	493	529	19	24	533	848	1306	1055	1045	723	861	509	4	7	5
209740_s_at	142	196	14	14	44	52	282	478	13	38	101	205	147	124	269	385	236	250	4	51	9
209741_x_at	224	113	224	370	194	321	160	141	153	182	135	139	104	81	129	154	175	152	362	313	537
209742_s_at	169	130	160	142	80	331	219	238	165	119	159	131	48	28	22	98	104	20	88	65	34

209743_s_at	226	198	496	316	526	278	486	363	421	355	426	456	176	143	215	100	116	99	175	117	185
209744_x_at	546	247	652	446	720	902	680	494	644	580	888	730	708	514	562	340	238	384	484	528	610
209745_at	212	180	87	141	313	44	174	215	203	245	144	134	117	143	234	254	221	215	295	237	235
209746_s_at	855	541	548	446	885	739	488	627	507	569	448	511	291	289	306	505	495	451	461	273	217
209747_at	312	325	128	530	359	460	212	286	316	278	236	236	45	82	66	87	85	6	80	67	62
209748_at	245	163	275	235	410	427	136	124	293	401	355	207	966	933	843	858	643	729	1723	1296	1970
209749_s_at	25	15	27	22	143	110	36	22	16	21	28	21	5	29	29	5	15	21	15	9	5
209750_at	214	220	221	347	485	661	413	379	219	338	615	574	317	174	96	38	34	66	55	63	78
209751_s_at	429	283	351	687	693	622	342	472	391	426	992	738	1061	1035	616	449	609	404	530	460	399
209752_at	20	12	18	19	47	480	24	94	41	24	13	64	5	28	38	11	15	5	5	2	13
209753_s_at	919	307	719	482	482	313	1265	1065	1218	1017	861	522	234	286	304	546	452	589	540	320	361
209754_s_at	853	214	1046	453	381	273	1219	1170	1395	1029	1056	607	180	110	73	219	184	147	582	148	157
209755_at	99	28	27	28	335	130	28	70	33	36	480	253	200	132	132	2	1	4	6	6	3
209756_s_at	15	21	14	8	78	89	9	13	5	8	9	26	8	3	7	11	10	9	7	11	3
209757_s_at	11	9	8	12	19	23	17	17	12	10	16	87	23	27	21	5	4	28	50	43	35
209758_s_at	124	123	99	111	291	310	148	149	197	166	114	162	42	61	40	33	39	56	47	29	23
209759_s_at	1278	2117	1221	1037	1008	774	1284	1104	903	2204	870	973	1062	890	409	988	1060	868	765	1201	604
209760_at	692	828	517	524	383	361	613	764	617	512	401	444	149	144	172	330	408	457	302	165	268
209761_s_at	306	137	326	530	293	638	294	447	520	377	274	196	56	71	52	114	125	438	182	335	294
209762_x_at	287	254	494	673	334	309	310	329	458	531	237	241	92	91	91	243	328	999	542	1100	1382
209763_at	103	85	134	84	217	237	42	82	115	103	77	88	14	2	33	5	2	40	14	11	45
209764_at	13	50	14	50	85	185	17	11	47	13	139	11	68	57	41	47	7	15	15	10	3
209765_at	1931	397	1297	331	1306	992	2356	1760	926	1145	1251	775	1119	2149	1928	349	423	369	955	451	624
209766_at	153	123	170	206	429	431	228	381	213	320	230	176	47	36	46	68	79	59	62	68	48
209767_s_at	79	22	23	49	922	740	48	15	16	31	10	16	11	30	14	115	51	73	55	20	37
209768_s_at	74	63	7	12	859	730	8	20	9	10	28	9	31	8	42	65	98	39	52	85	69
209769_s_at	15	17	14	32	91	66	28	22	31	24	11	16	5	8	8	8	14	8	8	6	6

209770_at	357	470	91	382	481	625	319	364	55	243	229	224	67	76	116	284	461	314	125	250	251
209771_x_at	437	1092	17299	28617	917	2547	633	743	20784	19804	406	963	746	1100	1136	1874	2135	1544	20009	22808	23439
209772_s_at	130	78	3993	6941	48	335	119	161	6930	7734	128	109	81	85	54	47	119	77	4061	1610	3607
209773_s_at	8272	3198	5050	1064	17924	391	10213	9203	5687	4806	14495	16255	16616	20725	19569	8880	11139	10997	7734	3697	3509
209774_x_at	50	462	132	84	26	122	66	37	47	48	453	858	38	45	60	144	212	330	110	194	187
209775_x_at	19	4	14	26	21	13	41	46	28	14	13	6	5	6	7	3	7	7	4	3	2
209776_s_at	246	161	128	88	43	42	318	148	233	242	244	21	20	48	50	103	86	61	81	34	35
209777_s_at	594	529	441	261	65	105	811	523	482	238	556	276	217	224	149	202	218	310	148	157	118
209778_at	161	89	233	239	473	638	172	235	158	231	338	303	201	240	238	88	146	146	158	220	218
209779_at	91	63	77	106	377	403	147	83	92	94	68	85	32	14	36	8	39	27	55	38	7
209780_at	675	325	704	163	2308	1712	791	479	486	256	1441	1269	3035	3719	3195	1274	1049	1127	1388	1092	1699
209781_s_at	524	483	254	21	355	384	425	301	179	344	310	588	386	289	522	273	229	213	416	146	189
209782_s_at	356	502	26	275	470	453	407	363	138	119	182	161	114	101	120	346	340	284	66	90	56
209783_at	58	43	41	63	203	55	191	32	108	108	13	58	35	50	65	65	29	26	75	42	15
209784_s_at	1048	1128	625	685	701	513	1101	780	787	387	493	333	263	242	570	479	549	394	388	418	302
209785_s_at	62	596	47	136	38	406	103	65	20	61	53	161	10	48	18	108	91	89	18	33	19
209786_at	2187	1837	1519	1796	1417	2551	1843	2245	1341	1799	2093	2008	1740	1184	1786	3090	2804	2761	2209	2097	3396
209787_s_at	2150	2199	1544	1463	2109	3131	1302	1781	911	1066	1538	1821	2516	2084	2836	4161	3443	3838	3180	3946	4400
209788_s_at	123	631	113	283	272	617	105	80	84	109	263	342	315	438	616	216	276	327	322	904	1194
209789_at	226	106	20	154	320	306	204	296	31	21	554	383	155	156	140	28	37	42	26	12	20
209790_s_at	437	215	606	898	474	409	453	517	776	717	519	775	769	569	329	329	317	374	981	643	667
209791_at	230	236	689	1805	173	125	240	272	633	267	249	251	63	44	13	64	49	60	182	156	177
209792_s_at	876	2622	3421	2341	361	566	930	1002	1726	1198	1002	1044	209	207	386	1009	944	1139	1558	1831	1224
209793_at	85	11	156	133	49	94	29	20	12	70	14	18	2	15	18	15	19	10	6	14	2
209794_at	337	398	136	147	113	141	343	400	209	217	171	199	38	46	26	132	159	123	45	59	56
209795_at	19	24	57	19	69	8	49	9	3	46	23	33	32	7	18	1	1	2	17	2	1
209796_s_at	1524	1642	1435	1323	1706	1962	1720	1387	1453	1409	2028	1594	3912	2847	1298	930	908	694	1171	1521	843

209797_at	665	554	632	522	1164	730	808	645	1034	758	1313	1165	1718	1386	1508	623	597	694	1276	714	597
209798_at	270	270	898	1042	514	343	530	434	891	1129	550	603	583	511	618	738	607	785	1513	1430	2039
209799_at	165	92	556	450	348	398	284	267	593	868	331	327	217	281	295	296	258	186	465	435	536
209800_at	9	6	12	5	56	60	7	8	5	7	27	5	2	19	48	16	6	5	17	28	1
209802_at	136	130	87	105	392	392	117	77	149	148	104	113	52	68	43	105	49	69	56	52	43
209803_s_at	10072	5108	7067	2296	2386	1625	5507	4670	5083	6047	2071	1739	3502	2513	2563	8961	9511	8619	7341	6936	3356
209804_at	343	248	397	253	278	265	590	527	516	295	327	287	256	177	245	443	388	332	478	247	349
209805_at	493	416	410	433	98	544	477	404	371	453	382	398	241	343	259	238	199	263	316	282	349
209806_at	1032	4702	1438	5268	7340	6424	1218	1878	1021	1009	11763	15162	12409	10209	12037	2254	2117	1762	2147	5305	3417
209807_s_at	118	19	81	158	187	390	117	77	94	120	112	89	35	7	32	44	19	14	57	24	25
209808_x_at	696	483	735	473	1101	611	607	639	677	784	817	664	371	237	212	177	159	202	170	132	102
209810_at	90	34	90	144	463	265	122	124	82	115	36	36	7	48	52	38	21	36	27	51	30
209811_at	517	344	137	136	49	16	599	757	302	333	179	145	302	278	159	253	186	232	427	174	219
209812_x_at	67	45	43	34	41	34	534	645	74	56	43	45	71	65	7	14	7	46	10	6	47
209813_x_at	21	10	20	21	41	31	21	12	15	14	21	6	5	4	22	3	6	2	6	40	1
209814_at	1746	1101	819	484	1014	681	1318	1229	1179	864	796	855	1251	1353	1558	2427	2195	2581	1768	1227	1648
209815_at	228	326	336	429	2144	1209	258	402	316	323	1856	1674	2005	2297	2454	168	171	191	591	784	1050
209816_at	86	72	14	122	279	234	130	131	92	143	277	128	125	64	152	10	2	32	57	41	60
209817_at	450	831	499	433	741	761	527	578	495	804	598	740	835	654	1093	755	718	919	1384	816	1297
209818_s_at	280	360	189	190	344	652	239	347	35	126	159	152	114	96	115	180	120	110	85	66	52
209819_at	46	10	43	22	316	250	45	51	27	42	24	11	5	25	10	5	30	25	2	26	23
209820_s_at	630	444	503	156	569	367	582	632	771	702	475	329	373	513	415	601	646	699	705	389	476
209821_at	38	4	20	8	122	4	58	38	21	21	21	55	1	11	2	11	10	15	15	12	23
209822_s_at	94	63	380	330	687	800	111	59	191	134	517	391	442	442	563	19	22	9	151	357	684
209823_x_at	22	191	19	25	56	35	93	28	19	18	44	46	65	11	61	83	90	50	18	19	10
209824_s_at	540	641	346	600	307	409	567	468	233	482	598	955	535	674	889	459	705	581	183	101	282
209825_s_at	1787	1235	1925	441	1208	505	1742	1053	2879	1876	1504	982	1056	1326	1404	1174	1040	1001	1546	1161	756

209826_at	225	405	41	291	78	63	266	236	213	299	23	64	35	64	30	135	165	133	109	42	41
209827_s_at	254	224	313	330	619	566	299	330	281	344	281	369	48	36	46	46	17	34	56	56	39
209828_s_at	112	158	72	114	38	34	58	128	114	52	105	141	1	14	25	1	1	3	4	29	8
209829_at	42	33	142	155	32	19	36	25	126	95	34	46	12	3	8	10	17	3	205	134	248
209830_s_at	67	45	80	162	286	16	135	18	82	67	18	3	3	4	1	15	17	4	55	3	3
209831_x_at	1461	1524	1529	1716	1375	1400	1483	1317	1486	1187	1636	1462	1011	844	722	653	713	665	463	738	610
209832_s_at	867	600	374	200	418	215	786	731	511	428	456	353	166	92	69	157	165	199	97	84	85
209833_at	447	530	300	458	495	597	530	553	348	296	298	243	143	106	170	294	246	385	302	303	218
209834_at	149	463	50	246	87	83	429	106	33	31	92	30	45	9	93	396	467	174	48	93	95
209835_x_at	3912	2836	4225	2810	1604	3042	4774	6525	5123	7047	2589	2185	1041	1206	1118	2050	2116	1768	2756	1821	1624
209836_x_at	2021	1581	1957	740	1192	1349	2590	1628	2739	2078	1794	1452	2435	1617	1375	1811	1990	2123	2047	1296	1133
209837_at	303	169	146	344	190	262	338	346	206	232	300	229	339	330	355	146	153	183	133	179	145
209838_at	220	167	285	259	265	460	266	203	304	231	172	163	366	291	230	304	287	312	650	503	563
209839_at	172	90	125	100	300	151	167	215	118	105	190	183	108	129	179	65	74	66	49	79	55
209840_s_at	21	22	50	21	60	34	36	29	32	41	18	20	2	19	19	7	16	21	21	24	11
209841_s_at	69	140	102	162	278	146	231	131	25	194	136	82	2	25	57	17	42	6	18	65	39
209842_at	34	86	39	31	51	60	34	41	60	35	36	54	13	43	9	24	50	65	20	16	5
209843_s_at	46	34	42	43	59	99	17	55	60	46	38	23	14	7	5	10	37	11	8	9	7
209844_at	25	23	16	14	19	30	36	36	8	21	26	28	28	3	8	6	6	17	2	6	1
209845_at	355	377	913	1253	487	680	305	300	969	707	482	361	485	511	748	485	526	451	1503	1511	1208
209846_s_at	328	246	239	185	25	255	225	310	207	180	155	77	50	60	50	122	187	192	151	270	179
209847_at	26	78	20	114	162	36	82	74	25	35	30	20	43	8	56	47	9	26	139	138	128
209848_s_at	192	204	115	123	18	16	297	259	29	210	142	119	37	155	106	69	86	68	28	27	27
209849_s_at	1536	974	852	502	2258	1127	1704	1615	965	963	1990	1973	2917	1710	2399	1580	1272	1623	812	652	761
209850_s_at	614	575	734	764	613	644	372	591	587	1212	600	631	140	118	137	238	241	177	254	247	169
209851_at	278	122	193	225	344	298	99	65	203	274	227	196	4	38	104	20	58	49	66	7	27
209852_x_at	2355	1917	3117	2783	2426	2014	4316	3208	5096	3778	4232	2110	1232	1522	2819	2300	1840	1884	2603	1677	1568

209853_s_at	1269	1053	1099	1168	1381	1067	2052	1656	2242	1945	1873	1494	1152	1251	1292	772	583	1041	1270	736	885
209854_s_at	26	7	19	17	73	58	34	33	35	27	38	24	12	11	13	4	40	27	28	32	27
209855_s_at	22	15	20	32	52	73	23	21	21	22	14	15	4	6	7	6	6	4	4	11	4
209856_x_at	593	258	726	473	547	110	631	582	820	816	985	586	349	295	354	213	127	209	263	199	227
209857_s_at	101	82	168	591	38	118	175	67	341	175	202	53	105	26	11	6	12	24	93	64	72
209858_x_at	345	871	442	987	214	707	391	139	116	84	36	162	37	104	118	287	418	359	505	774	663
209859_at	123	131	5	13	313	241	95	106	15	11	31	101	33	30	34	38	52	58	44	57	47
209860_s_at	3027	1922	2858	2527	2698	2952	2511	2553	2874	2862	2281	2684	3010	3372	3416	3794	3641	3854	3970	3764	3996
209861_s_at	1937	1586	2509	1721	1480	1782	3497	3481	3168	2893	2722	1561	4188	4269	3341	4892	4919	4931	5413	3173	4233
209862_s_at	599	236	1264	833	947	1289	458	478	717	741	829	906	634	729	904	363	409	426	873	1283	1899
209863_s_at	181	105	151	198	312	391	160	207	223	173	175	121	11	30	29	2	8	50	32	44	33
209864_at	327	348	477	660	544	430	270	393	598	773	357	342	192	106	130	111	163	102	664	664	436
209865_at	382	291	515	450	356	417	115	292	388	219	209	238	172	196	273	462	340	174	1180	977	1204
209866_s_at	13	26	14	62	140	47	68	46	51	68	63	62	1	6	3	2	2	6	15	18	15
209867_s_at	5	12	12	18	44	19	17	24	15	17	13	8	2	11	4	5	6	10	5	3	7
209868_s_at	847	1067	857	1182	1009	896	982	864	926	983	1391	850	959	1064	1128	808	852	452	1056	802	741
209869_at	99	128	252	594	162	175	140	86	537	1635	57	79	2	6	4	9	4	17	638	322	205
209870_s_at	25	19	27	47	30	35	36	26	79	137	38	30	7	8	14	6	8	6	7	8	12
209871_s_at	11	13	8	10	49	31	19	21	19	18	17	15	5	4	9	2	3	18	6	8	3
209872_s_at	527	352	461	396	760	503	783	504	522	689	533	294	64	60	42	51	63	68	77	28	21
209873_s_at	1820	1730	1480	1080	2063	2255	1786	1506	1824	1665	2278	1048	872	586	487	328	426	265	372	158	181
209874_x_at	550	586	307	555	206	379	615	447	330	372	350	238	40	94	132	415	288	279	216	247	303
209875_s_at	146	30	244	398	85	42	180	82	56	48	68	107	10	9	6	124	60	100	399	161	268
209876_at	73	33	26	185	27	24	38	38	24	20	98	31	56	71	98	78	60	99	11	11	115
209877_at	139	299	170	1542	67	56	252	170	842	579	158	77	29	37	50	72	77	64	256	244	166
209878_s_at	553	693	713	657	372	628	603	540	840	872	757	405	126	98	152	182	169	241	218	200	183
209879_at	33	101	45	32	348	372	34	42	32	41	267	34	148	130	136	93	47	23	39	5	16

209880_s_at	30	29	33	67	40	93	30	67	28	28	186	65	9	27	31	6	14	23	6	6	10
209881_s_at	40	201	345	58	889	270	54	42	193	182	411	293	270	311	304	5	18	80	150	121	46
209882_at	102	71	302	469	282	433	166	149	324	291	395	265	268	216	145	31	2	31	306	298	317
209883_at	134	125	182	164	352	439	191	160	154	158	233	243	136	118	110	10	32	25	100	511	559
209884_s_at	217	154	768	277	213	305	163	210	372	467	124	121	269	233	243	238	128	260	1515	728	959
209885_at	787	838	1028	694	652	618	1112	773	409	277	1650	377	975	660	777	624	594	421	350	521	240
209886_s_at	17	26	128	138	565	159	76	29	193	224	21	28	41	45	38	57	13	7	51	34	19
209887_at	85	5	43	58	37	52	81	9	48	84	99	77	2	2	6	29	41	3	56	4	30
209888_s_at	131	125	45	27	48	32	106	165	52	29	138	211	25	7	40	27	41	9	29	6	34
209889_at	28	43	33	159	48	36	91	70	35	71	23	57	5	9	12	12	15	16	13	8	16
209890_at	454	251	319	360	254	172	421	668	234	351	493	479	766	598	488	561	563	357	212	420	298
209891_at	470	167	342	120	1252	17	514	606	507	375	1036	985	2434	2188	1422	565	485	594	1331	250	179
209892_at	217	174	4806	3520	653	390	281	323	4485	3606	736	501	752	866	864	156	202	191	4368	2937	4519
209893_s_at	52	61	1335	653	272	27	215	107	1742	1108	232	238	230	249	307	64	170	78	1567	828	593
209894_at	122	196	45	100	142	153	81	153	37	55	84	77	106	74	66	321	235	188	89	289	322
209895_at	1000	741	1481	718	1043	1262	2221	2024	1678	1867	1370	1164	280	139	101	280	201	241	303	220	251
209896_s_at	2061	1140	2200	1401	1659	1404	3022	3568	2877	3518	2223	1800	968	456	400	1080	971	754	981	796	1019
209897_s_at	120	122	81	149	320	375	262	202	44	53	36	79	13	40	45	36	58	61	44	31	13
209898_x_at	402	445	677	158	998	1402	322	380	253	316	632	677	1004	988	1983	812	756	1133	1147	1637	1883
209899_s_at	5313	4074	2748	2151	2759	1798	4057	3664	2662	2211	2521	2021	2851	2131	1477	2752	2828	2724	1847	1352	1277
209900_s_at	798	783	806	400	1539	1164	547	578	958	1198	1245	2168	1635	2121	1623	1385	1107	1505	2694	1369	1651
209901_x_at	19	19	23	31	26	32	25	26	25	31	23	19	4	4	6	3	6	4	4	4	1
209902_at	189	164	442	250	540	340	246	218	585	597	537	445	596	563	510	417	339	346	1106	487	779
209903_s_at	681	647	1210	925	1428	1025	655	748	1145	1192	1289	1199	1141	1140	1732	940	840	1024	3043	2096	2822
209904_at	9	189	110	1004	348	2600	131	82	292	91	854	270	662	281	369	22	77	54	627	225	153
209905_at	2119	4003	331	726	1634	2576	1533	2801	395	393	1529	2601	1540	1655	1970	2770	2512	1696	1565	1873	2005
209906_at	64	40	109	97	474	442	21	91	102	130	75	129	37	44	60	8	12	28	32	33	23

209907_s_at	635	471	728	815	583	517	514	534	555	774	753	426	214	339	262	314	233	229	260	284	259
209908_s_at	42	19	94	107	66	81	102	119	83	71	58	73	5	1	15	2	3	1	1	8	13
209909_s_at	19	4	56	96	26	83	33	4	55	10	24	29	31	34	17	20	20	26	62	90	116
209910_at	163	161	231	70	209	203	68	98	153	126	210	227	51	53	52	47	64	60	110	57	58
209911_x_at	652	5095	543	4339	2521	7722	602	908	429	630	4563	8323	4026	2231	3324	731	824	471	229	1343	935
209912_s_at	165	145	168	102	566	478	57	83	112	108	220	196	351	271	130	52	76	68	100	170	75
209913_x_at	144	185	210	159	95	71	101	230	123	211	179	121	236	157	127	85	106	118	205	209	182
209914_s_at	90	16	27	28	95	275	19	26	29	35	74	62	4	4	7	3	1	2	25	8	1
209915_s_at	30	10	34	22	21	22	24	20	15	50	14	20	10	31	9	3	3	6	13	2	2
209916_at	213	100	297	949	412	218	207	182	258	435	279	189	506	520	521	231	230	203	594	771	963
209917_s_at	22	11	26	295	74	173	9	18	59	166	145	232	135	51	60	17	27	12	63	90	32
209918_at	45	44	54	34	107	148	41	25	24	48	34	25	7	6	9	44	57	13	11	7	9
209919_x_at	493	523	304	504	551	697	484	421	428	432	415	313	75	44	42	144	148	159	175	221	122
209920_at	139	112	203	331	619	782	201	161	207	196	252	260	180	219	115	55	62	32	64	63	83
209921_at	2209	1333	2570	283	547	1032	1545	723	815	1686	230	99	333	216	452	3004	3322	3810	1389	3596	4851
209922_at	17	28	142	14	37	99	80	46	78	10	44	52	110	178	71	60	49	96	139	96	67
209923_s_at	336	383	220	222	176	305	356	245	250	80	286	189	173	179	199	171	218	217	181	139	147
209924_at	167	119	157	26	48	39	89	166	103	164	112	26	29	15	41	34	26	28	29	46	9
209925_at	413	370	289	550	318	435	337	387	175	264	257	177	122	116	152	435	350	365	258	239	460
209926_at	122	72	140	85	232	91	85	95	171	22	64	92	138	92	140	61	156	40	138	74	49
209927_s_at	816	577	521	340	833	744	599	402	661	570	428	547	573	258	174	231	254	200	220	236	232
209928_s_at	89	84	52	40	285	120	57	29	55	87	14	33	5	33	33	29	64	31	27	24	3
209929_s_at	573	539	1270	1167	698	613	457	420	1201	1349	671	718	426	471	468	291	313	254	690	523	366
209930_s_at	26	7	37	49	289	447	60	15	63	10	605	269	332	239	334	4	10	35	27	34	7
209931_s_at	300	210	100	125	256	357	215	285	138	80	246	172	79	97	63	79	48	81	42	47	20
209932_s_at	2539	1551	2726	1495	4363	1787	2207	2623	3525	3173	4488	4953	7259	6391	4429	1901	1882	2038	3307	2164	1655
209933_s_at	78	46	47	35	30	22	127	100	75	39	41	31	22	5	60	24	30	13	3	2	21

209934_s_at	361	456	494	463	361	196	249	202	306	330	205	124	158	182	170	366	326	354	552	753	648
209935_at	368	640	472	493	440	352	220	367	356	260	139	191	204	149	75	318	295	259	1009	1046	1068
209936_at	21	15	27	208	213	254	56	77	225	119	107	102	26	36	2	22	15	4	11	4	13
209937_at	45	46	7559	4761	34	35	53	36	8642	4773	45	41	5	41	5	5	4	12	6645	8767	7838
209938_at	173	196	331	208	323	32	229	302	355	247	111	171	73	50	88	132	81	93	172	125	162
209939_x_at	630	720	1900	1048	676	922	412	490	1453	1223	1164	838	651	872	1087	686	590	838	1968	1080	1052
209940_at	163	271	191	266	625	919	201	235	60	154	207	220	142	127	52	123	91	160	135	106	114
209941_at	478	326	282	495	285	282	322	409	272	421	490	494	75	95	68	85	86	111	122	124	108
209942_x_at	9270	6299	12870	11124	51	55	7076	5949	15612	13311	33	43	34	20	13	5811	5716	4845	10725	7849	7320
209943_at	230	285	148	97	220	332	171	260	162	143	74	133	121	138	258	444	360	376	405	576	802
209944_at	1537	1764	1686	1584	2045	3216	1891	1578	1003	1226	2301	2054	3283	2797	5594	5032	4641	5020	2881	3341	3453
209945_s_at	516	457	461	573	756	500	506	570	637	500	695	475	526	528	873	519	567	393	557	466	492
209946_at	38	122	87	129	192	208	90	73	134	129	77	40	6	11	30	6	2	2	12	24	1
209947_at	1336	676	628	846	433	414	1416	1231	569	844	597	630	153	153	259	646	487	497	422	468	402
209948_at	295	167	374	242	315	300	343	288	301	328	301	185	17	35	63	28	38	70	49	59	28
209949_at	70	18	109	89	29	231	54	62	27	115	112	113	53	36	11	6	5	7	27	62	53
209950_s_at	41	21	43	383	56	86	78	29	31	56	48	113	45	15	19	9	5	8	29	19	40
209951_s_at	172	141	168	153	309	278	250	248	199	260	183	133	45	23	11	24	38	49	36	24	3
209952_s_at	44	34	123	12	186	101	125	139	178	183	178	19	22	28	74	10	5	6	7	17	25
209953_s_at	1597	1850	1241	533	540	344	1705	1362	1176	1135	1278	731	1611	1539	712	1506	1455	1243	925	680	504
209954_x_at	624	309	589	389	608	728	677	846	476	577	463	191	204	187	141	239	203	228	167	88	114
209955_s_at	50	19	15	12	93	24	21	16	20	13	65	80	4	49	40	3	8	4	3	4	3
209956_s_at	107	12	91	8	125	159	118	77	67	88	98	102	23	17	23	3	16	27	25	10	1
209957_s_at	283	212	304	371	858	536	282	292	235	161	162	69	53	151	178	125	125	119	85	44	33
209958_s_at	44	24	23	43	32	339	32	28	19	20	45	29	95	106	51	84	74	44	5	151	108
209959_at	29	29	26	56	62	51	54	42	29	29	26	34	5	8	6	3	19	7	11	8	5
209960_at	12	1	4	6	38	9	3	18	19	63	13	10	4	1	1	3	8	2	1	2	1

209961_s_at	311	220	190	137	71	44	192	223	210	281	156	269	41	36	85	72	57	109	66	77	52
209962_at	95	363	121	189	18	214	186	251	83	70	206	131	5	12	43	87	68	95	33	33	33
209963_s_at	220	271	163	189	85	132	197	236	124	218	166	137	32	36	68	36	42	70	41	8	30
209964_s_at	110	135	278	397	386	129	203	231	367	359	325	245	35	70	56	22	34	38	104	90	67
209965_s_at	202	159	532	287	98	67	241	234	583	414	243	322	141	174	147	110	67	108	323	299	220
209966_x_at	52	57	87	43	47	23	38	103	55	46	104	92	23	42	3	24	19	10	41	23	18
209967_s_at	172	51	37	58	110	105	98	136	9	11	148	131	193	165	67	143	186	244	69	162	195
209968_s_at	20	12	16	25	55	71	17	22	16	15	14	14	44	4	5	7	10	6	13	11	13
209969_s_at	356	589	205	437	63	40	257	274	261	284	472	347	296	302	440	421	794	2697	533	1821	3032
209970_x_at	148	241	49	168	445	238	103	129	136	201	139	103	54	66	85	89	40	78	72	52	67
209971_x_at	4628	5039	4019	2118	5884	9178	4505	3430	3962	4207	5462	3905	5968	6859	8429	3102	3685	3072	4227	5457	3626
209972_s_at	276	314	246	239	720	669	269	210	210	254	307	173	218	203	299	248	255	300	361	311	308
209973_at	143	62	72	59	103	99	65	62	233	56	51	82	125	40	32	65	7	8	8	66	33
209974_s_at	4764	1834	3068	2292	3570	2521	2357	3942	3012	2682	2921	2735	7022	5760	6578	8287	7084	8091	7185	7249	8276
209975_at	30	56	90	151	210	356	95	71	233	74	101	52	19	5	54	66	32	43	43	94	66
209976_s_at	9	9	8	10	148	214	7	7	7	7	6	8	6	3	2	5	17	12	29	6	31
209977_at	11	18	9	8	67	38	33	7	15	7	7	13	3	38	36	3	17	15	1	9	3
209978_s_at	97	21	24	21	62	26	36	30	16	34	18	8	16	4	12	6	2	34	6	3	1
209979_at	20	11	11	23	43	36	13	25	17	18	14	13	4	12	17	10	6	12	8	6	6
209980_s_at	523	236	575	744	49	31	526	773	827	998	686	753	794	524	335	226	296	293	435	308	280
209981_at	67	39	27	48	30	24	34	22	12	14	23	49	40	27	13	70	40	28	6	29	33
209982_s_at	25	12	8	17	239	223	13	36	94	36	53	16	18	22	7	14	8	14	8	34	26
209983_s_at	8	9	42	16	22	16	25	49	24	13	6	13	7	28	3	3	29	8	34	9	1
209984_at	646	296	572	583	697	804	359	401	451	584	345	421	195	373	412	363	435	466	497	535	560
209985_s_at	62	11	7	19	69	152	28	18	16	15	11	18	6	9	31	21	23	18	8	15	8
209986_at	107	7	43	22	14	120	58	71	16	78	104	90	3	2	4	2	3	6	4	9	19
209987_s_at	9	6	15	9	12	17	41	9	27	46	17	13	12	2	2	23	6	5	6	1	19

209988_s_at	34	24	35	34	25	12	54	13	39	60	43
	44	1	18	22	16	5	9	3	30	2	
209989_at	225	182	96	111	258	237	179	219	62	75	212
	178	288	249	143	213	137	168	167	122	190	
209990_s_at	67	13	35	67	210	75	50	74	13	18	27
	84	23	25	26	14	32	46	22	22	19	
209991_x_at	156	118	251	190	291	316	216	314	189	215	190
	156	25	6	27	20	6	40	20	26	27	
209992_at	209	251	300	190	371	400	192	212	250	275	289
	157	48	105	71	83	61	105	111	84	102	
209993_at	193	410	109	74	366	138	144	125	59	81	131
	182	105	176	156	31	24	27	6	86	132	
209994_s_at	49	107	8	6	609	189	16	15	7	8	161
	36	196	478	282	31	11	37	18	99	122	
209995_s_at	134	100	76	113	18	44	178	153	120	256	253
	200	40	8	49	7	34	18	33	59	8	
209996_x_at	13	13	7	10	63	286	12	34	5	17	51
	137	23	56	88	8	9	6	23	7	9	
209997_x_at	197	236	369	288	580	466	295	527	270	299	539
	660	82	139	177	71	96	55	88	36	79	
209998_at	515	338	709	568	669	880	559	533	741	900	857
	576	607	1038	709	255	331	394	615	348	449	
209999_x_at	36	71	31	40	55	75	131	45	74	57	68
	141	11	8	3	9	10	9	11	11	9	
210000_s_at	26	2	54	5	7	7	70	51	3	52	18
	3	1	2	3	2	1	2	3	15	2	
210001_s_at	155	99	107	70	203	329	56	108	66	46	146
	119	90	83	54	115	76	104	97	73	52	
210002_at	205	245	875	1458	8	129	152	245	764	1377	36
	58	3	12	2	256	277	290	3862	1836	2941	
210004_at	66	36	73	136	106	58	87	61	88	64	67
	52	198	82	119	7	38	24	22	29	26	
210005_at	530	324	449	132	324	324	298	375	384	263	207
	162	300	286	190	902	571	743	914	388	458	
210006_at	459	640	506	716	542	478	429	333	527	619	723
	594	901	752	697	421	482	389	756	599	481	
210007_s_at	191	214	305	177	522	187	358	424	355	358	277
	240	202	141	199	196	174	255	175	116	151	
210008_s_at	298	230	283	231	636	125	225	214	784	486	632
	386	752	305	235	178	135	103	248	238	194	
210009_s_at	245	135	240	160	525	285	268	292	302	250	428
	555	516	375	216	259	254	254	208	177	104	
210010_s_at	2273	2738	2825	3783	948	625	2388	2594	3485	3701	1309
	588	886	931	1256	1898	2030	1634	4394	2981	2000	
210011_s_at	1803	1282	2035	1238	1538	486	1725	1507	2190	2158	1528
	1395	1359	777	262	272	296	318	538	273	306	
210012_s_at	49	118	106	158	7	7	110	141	253	109	26
	9	20	18	17	48	46	55	46	3	51	
210013_at	97	17	35	85	45	43	38	104	88	55	41
	20	10	8	8	47	3	8	4	17	9	
210014_x_at	3240	2129	2189	1275	2598	1543	3461	3067	2577	2169	3343
	2092	3919	3719	3131	2431	2610	2482	2010	2327	1790	
210015_s_at	220	236	180	177	192	44	209	289	215	147	109
	103	2	15	3	47	60	36	16	51	43	

210016_at	40	62	106	78	131	146	62	100	55	123	45
	98	14	15	2	30	17	2	12	16	14	
210017_at	359	186	134	167	429	234	265	257	74	103	347
	197	353	568	479	1034	796	978	280	232	333	
210018_x_at	1017	518	418	394	499	419	1151	794	448	400	1046
	466	463	659	782	1809	1938	1835	362	244	439	
210019_at	123	19	26	31	180	113	25	48	21	21	21
	28	19	31	8	33	31	21	46	16	21	
210020_x_at	180	92	100	146	136	328	155	113	143	112	165
	178	38	44	23	44	28	22	65	27	39	
210021_s_at	479	748	602	473	401	366	509	255	425	323	534
	202	221	106	87	168	100	120	187	319	186	
210022_at	463	381	293	144	100	128	478	435	63	74	229
	59	160	159	350	708	716	609	348	422	436	
210023_s_at	1016	679	773	438	304	214	905	949	432	532	553
	304	223	296	685	1139	1240	1231	570	593	554	
210024_s_at	3269	2479	2930	2611	3921	3021	2196	2253	2296	1954	3989
	2726	6165	6504	6489	5789	4936	4562	5760	4492	5832	
210025_s_at	48	64	104	53	34	23	23	26	24	36	28
	16	4	8	9	6	11	12	6	4	9	
210026_s_at	982	994	522	480	745	681	730	482	567	843	655
	515	579	662	555	867	1050	766	1466	1205	1113	
210027_s_at	5961	4424	3452	2457	2863	3438	4697	5947	3157	3476	3483
	2565	5449	4655	4747	8808	8881	8356	3914	5539	4456	
210028_s_at	712	541	1112	943	1152	972	1187	1477	761	767	1093
	1675	1020	1142	1457	951	882	1153	1404	1444	1903	
210029_at	253	182	288	277	78	59	276	314	282	341	294
	204	80	38	46	9	76	58	92	96	90	
210030_at	13	18	12	23	12	7	21	55	23	18	9
	24	25	4	13	19	6	7	4	6	13	
210031_at	149	13	23	66	65	155	87	117	47	31	21
	43	23	7	5	5	8	4	16	40	40	
210032_s_at	52	44	50	109	256	318	13	73	40	67	48
	14	17	5	29	5	34	8	7	24	7	
210033_s_at	91	57	83	124	271	181	93	140	80	116	84
	74	2	7	12	20	33	12	32	27	6	
210034_s_at	77	63	85	65	106	132	111	55	63	106	34
	40	33	41	84	44	68	66	61	31	64	
210035_s_at	19	5	9	4	19	26	9	42	33	3	14
	5	4	15	6	23	16	5	18	15	14	
210036_s_at	359	271	298	738	308	1003	358	344	514	394	402
	287	195	137	240	223	305	239	436	496	277	
210037_s_at	163	77	123	147	293	270	130	177	138	179	195
	93	25	19	31	6	21	34	2	5	5	
210038_at	197	253	496	445	359	339	327	285	459	446	308
	410	130	112	146	134	102	95	508	293	471	
210039_s_at	292	330	600	370	359	382	474	362	404	482	433
	358	40	76	56	129	67	91	219	182	229	
210040_at	87	33	117	135	179	74	69	139	131	143	98
	155	22	23	9	2	22	21	15	16	1	
210041_s_at	299	339	506	356	374	746	437	611	437	495	449
	757	828	725	333	475	529	727	524	417	639	
210042_s_at	441	399	184	230	873	570	235	252	161	130	378
	221	2661	1832	1709	364	381	244	352	280	161	

210043_at	33	47	262	175	45	328	46	58	285	437	379	67	75	71	59	74	42	21	59	21	41
210044_s_at	20	21	72	54	117	67	29	29	32	28	38	21	91	81	66	61	12	12	15	14	13
210045_at	212	175	479	1063	301	355	256	268	666	1315	296	232	124	108	135	62	50	68	503	315	288
210046_s_at	709	970	2769	6479	1030	590	1088	969	3699	6632	1084	1033	849	896	401	370	407	309	1668	1411	1200
210047_at	20	49	69	97	47	124	54	49	20	43	98	210	72	95	73	87	102	70	144	182	160
210048_at	438	307	552	282	1104	895	586	521	609	474	1319	803	639	750	724	298	264	292	286	242	312
210049_at	12	5	15	10	10	13	12	25	21	14	7	5	7	3	3	2	3	2	3	2	9
210050_at	143	242	171	233	313	288	174	241	209	165	172	205	151	190	236	150	205	211	282	265	177
210051_at	15	9	20	14	132	85	13	12	13	21	340	173	224	277	129	7	4	4	56	21	11
210052_s_at	3157	852	3919	947	4157	437	4104	4699	4728	4326	5573	4544	2876	3295	2503	1399	1515	1562	2804	731	915
210053_at	401	208	362	119	569	383	413	300	440	419	354	309	412	487	673	942	812	1034	1086	597	894
210054_at	483	330	308	175	443	349	496	414	225	268	202	369	505	239	350	772	690	640	950	658	1062
210055_at	15	9	9	13	26	34	15	9	5	66	10	9	2	3	2	4	2	8	1	13	4
210056_at	66	112	46	56	206	117	69	79	94	143	112	126	53	22	37	22	35	62	118	43	61
210057_at	22	33	114	109	74	46	62	99	104	152	80	98	36	20	7	7	8	20	46	26	18
210058_at	1000	690	1238	680	764	642	1112	839	1199	1235	1064	645	715	490	797	422	521	425	859	611	443
210059_s_at	512	327	434	314	1098	966	358	173	557	495	598	354	584	525	522	369	490	379	1018	548	541
210060_at	54	47	52	53	40	44	45	42	41	66	51	49	11	13	7	7	13	7	11	9	10
210061_at	87	77	111	89	383	492	97	187	106	106	156	142	59	72	3	5	39	8	43	52	22
210062_s_at	156	84	251	188	113	368	322	334	399	467	337	331	77	50	65	29	12	46	64	18	17
210063_at	19	69	47	256	91	67	138	61	48	57	48	46	18	24	42	70	30	74	50	26	23
210064_s_at	283	201	320	299	337	298	176	300	257	342	230	314	28	2	66	31	38	34	38	32	39
210065_s_at	15	79	46	45	135	198	46	84	25	29	55	21	5	13	8	5	37	4	6	3	16
210066_s_at	4	15	7	6	12	5	5	5	11	3	14	29	13	4	3	13	1	11	1	1	1
210067_at	7	4	4	16	16	23	7	4	4	7	3	4	1	1	1	1	8	1	1	3	1
210068_s_at	108	12	50	122	18	35	87	33	54	10	63	82	2	15	18	6	3	2	5	3	1
210069_at	156	287	27	18	117	156	195	235	92	74	75	114	59	23	74	66	127	190	1	86	91

210070_s_at	232	297	95	115	360	191	187	218	58	96	64	26	31	19	50	66	125	81	48	37	44
210072_at	112	9	132	160	223	250	109	139	154	193	142	69	59	43	9	20	11	26	43	29	48
210073_at	3	11	5	14	98	133	7	36	5	42	3	6	1	14	21	2	11	1	20	2	13
210074_at	585	335	770	603	1332	501	965	1276	807	1121	1184	2596	1431	1002	1029	451	649	593	1004	448	462
210075_at	475	983	52	521	348	383	538	432	219	240	115	83	108	107	136	369	381	305	127	106	75
210076_x_at	2205	1339	2580	2212	1607	1060	1887	3480	2028	3319	1722	1556	738	353	432	719	549	687	1091	646	711
210077_s_at	180	19	114	111	316	211	151	137	114	130	185	102	35	33	54	36	16	38	37	24	33
210078_s_at	38	2	47	22	15	8	7	7	66	60	71	39	32	49	40	36	25	1	90	44	82
210079_x_at	78	22	53	27	34	67	24	29	110	81	27	52	16	8	5	11	28	5	6	12	17
210080_x_at	85	61	69	93	175	185	53	62	149	115	87	118	13	3	3	4	23	3	15	18	1
210081_at	147	105	155	97	102	78	163	104	122	124	111	123	7	8	69	69	78	14	43	13	27
210082_at	53	35	96	32	63	54	50	38	60	41	40	13	9	4	7	32	15	19	8	36	31
210083_at	153	170	156	224	219	58	286	231	249	253	148	187	31	9	24	5	11	7	8	11	19
210084_x_at	25	24	33	22	33	17	17	34	25	13	20	19	9	8	9	32	8	4	16	22	3
210085_s_at	99	116	221	691	23	112	102	38	80	123	78	38	77	41	81	52	87	68	285	323	313
210086_at	40	84	14	35	58	74	28	57	33	31	26	23	38	34	33	59	46	42	9	4	7
210087_s_at	549	467	604	405	349	445	657	891	609	809	792	387	238	242	227	228	277	218	232	192	151
210088_x_at	349	265	224	352	433	538	327	379	322	416	237	381	165	116	129	79	84	93	95	87	9
210089_s_at	28	13	43	32	55	50	19	11	25	15	20	10	2	3	7	24	11	2	9	4	6
210090_at	29	13	16	9	22	20	38	22	16	18	13	21	27	29	37	219	251	204	79	97	72
210091_s_at	13	12	14	26	25	12	27	15	15	15	13	15	1	2	4	2	9	3	4	2	1
210092_at	1078	588	1607	679	1949	1087	1263	1178	1498	1247	1445	915	3406	1377	707	838	690	779	1417	974	652
210093_s_at	800	470	996	616	933	1340	706	612	808	653	854	367	2422	807	719	1413	947	981	2446	1539	1279
210094_s_at	486	348	913	645	800	1087	431	693	433	611	338	334	522	391	385	670	712	556	553	682	426
210095_s_at	200	204	46	70	9930	7606	205	95	116	25	7896	3777	4176	3854	1275	152	153	59	6	42	40
210096_at	5	34	47	78	33	56	17	66	63	11	7	8	1	2	23	2	5	5	21	25	10
210097_s_at	4453	4266	2918	1443	3921	4042	2984	2720	2173	2478	2741	2907	4772	4179	3924	3830	3203	3498	4126	4078	3532

210098_s_at	373	212	202	111	416	396	228	211	203	238	281	113	146	197	514	555	390	404	582	523	422
210099_at	19	6	8	9	54	78	16	12	16	8	9	8	9	4	5	3	4	3	6	2	3
210100_s_at	328	474	297	358	45	52	354	389	308	458	115	45	11	13	3	137	98	99	132	31	83
210101_x_at	2354	2400	3814	3646	1251	1355	2316	2202	2523	2400	2169	1534	1168	1674	1537	1543	1420	1454	1924	2357	1980
210102_at	153	88	72	56	210	404	125	212	99	137	14	75	35	18	21	108	107	115	29	24	25
210103_s_at	29	29	229	176	77	85	27	18	205	229	53	35	7	9	62	5	5	2	106	97	123
210104_at	857	217	498	444	594	371	608	713	459	383	874	865	1703	1533	1049	1030	1014	742	859	707	691
210105_s_at	378	727	1327	422	2484	3210	451	362	662	1437	2106	2615	2007	2130	5609	596	760	620	2182	4339	5715
210106_at	98	34	106	596	78	65	53	62	32	74	242	232	132	28	86	5	6	13	47	19	43
210107_at	152	142	213	149	235	310	158	127	244	201	145	73	14	23	11	35	29	39	35	11	3
210108_at	19	75	38	144	66	17	74	58	95	172	48	26	11	12	24	19	20	11	100	160	151
210109_at	60	39	213	251	109	206	87	131	195	278	128	126	5	12	6	6	11	7	37	17	17
210110_x_at	1443	712	1078	594	3222	1773	1293	858	1571	1328	1815	1362	2265	2050	1957	1023	783	574	1093	925	749
210111_s_at	1069	1193	1255	1703	1163	2317	999	1013	1189	1255	1244	1080	1371	1434	1637	1857	1670	1554	3993	4585	5523
210112_at	517	979	327	202	614	706	563	507	294	281	352	304	226	210	271	427	433	409	345	231	195
210113_s_at	28	12	14	12	21	28	9	22	9	10	14	18	2	3	5	6	3	6	4	3	3
210114_at	546	685	347	119	539	648	488	527	185	240	557	486	382	461	567	400	486	360	367	440	460
210115_at	5	5	8	8	14	15	21	5	4	8	4	6	4	2	2	5	2	2	4	3	2
210116_at	16	6	11	13	63	58	9	12	8	7	16	8	5	4	26	31	7	31	7	21	18
210117_at	142	129	326	243	254	180	196	165	309	161	289	253	541	450	333	441	363	481	1147	992	1634
210118_s_at	74	79	39	116	41	333	167	90	16	60	40	35	13	10	7	3	6	2	39	5	21
210119_at	4	6	18	40	27	31	19	9	52	4	4	14	1	2	3	27	3	2	1	4	17
210120_s_at	504	375	365	409	228	254	613	480	340	387	446	238	133	227	234	194	296	259	182	116	152
210121_at	1	7	1	1	8	4	3	3	5	1	1	3	4	12	1	2	1	7	4	1	1
210122_at	8	5	8	6	55	47	7	7	8	6	6	9	3	3	5	2	3	2	7	2	3
210123_s_at	29	32	24	162	62	130	34	22	27	21	23	14	19	11	10	57	44	107	118	122	128
210124_x_at	426	333	327	317	742	763	515	574	329	396	618	829	268	343	350	247	309	308	159	136	115

210125_s_at	5132	1428	7698	5820	3200	2360	4950	3969	8196	6288	5544
	4226	2246	2363	4923	4134	3209	3937	6827	5629	2917	
210126_at	41	23	42	18	48	102	19	20	27	27	11
	15	5	50	29	17	10	39	18	9	7	
210127_at	400	384	118	184	372	300	370	356	223	204	394
	338	120	66	52	99	34	21	27	15	1	
210128_s_at	206	99	110	194	74	81	98	41	126	102	37
	45	24	12	7	43	59	12	13	35	38	
210129_s_at	111	344	38	309	62	74	133	191	47	52	27
	33	14	11	10	96	42	37	18	51	4	
210130_s_at	906	1637	642	2358	1179	899	795	1143	349	491	1018
	818	675	697	480	406	432	275	224	384	218	
210131_x_at	2658	1600	2680	2606	2886	1580	3483	3370	3211	3326	3263
	2657	2706	2286	1733	1669	1468	1228	1618	1208	1074	
210132_at	262	437	43	125	41	36	485	422	86	24	131
	87	63	29	102	286	188	187	47	103	49	
210133_at	60	33	53	88	191	128	183	99	71	115	58
	160	11	4	7	23	23	9	21	21	7	
210134_x_at	111	52	34	14	70	195	28	33	23	29	31
	19	11	46	19	9	10	17	7	6	6	
210135_s_at	156	405	7	23	25	26	245	257	27	25	65
	67	50	35	61	661	801	453	7	38	16	
210136_at	278	450	697	763	16	30	388	351	809	763	193
	113	54	96	118	255	320	157	678	593	472	
210137_s_at	1334	1069	882	449	836	962	1447	1477	1106	551	2581
	1263	1305	1052	1116	928	973	673	688	691	741	
210138_at	314	508	26	34	34	43	273	286	35	59	23
	16	2	20	1	449	393	448	26	16	7	
210139_s_at	1570	566	126	74	2432	1384	1102	1015	115	124	1806
	2325	3618	5135	6350	2093	2152	1627	135	93	40	
210140_at	172	46	46	35	1086	3009	105	38	28	32	1590
	772	1926	2910	7435	8	38	21	55	1307	1008	
210141_s_at	102	169	72	122	168	113	159	199	134	137	176
	93	31	41	13	7	5	39	19	67	48	
210142_x_at	1846	4559	2896	2702	1670	2615	2483	2361	2460	2316	1880
	1434	1396	1863	2001	2424	2743	2103	1776	2029	1604	
210143_at	742	400	9189	3619	43	36	947	375	4475	13151	20
	90	3	4	2	733	1002	534	3518	4760	5433	
210144_at	242	390	248	352	63	210	179	154	169	243	217
	199	129	128	103	126	122	152	125	150	142	
210145_at	205	148	279	277	4	12	249	172	326	486	17
	21	2	6	3	371	255	405	1326	510	873	
210146_x_at	24	28	5	13	78	73	56	12	12	10	11
	13	3	16	1	1	1	3	8	1	25	
210147_at	50	30	137	123	181	97	90	84	91	102	72
	94	10	30	22	3	9	17	25	2	11	
210148_at	142	108	404	323	234	489	386	360	313	446	290
	275	52	32	49	97	88	82	95	49	108	
210149_s_at	9348	9497	8992	11517	8055	9610	12658	12789	7752	8517	9056
	10703	13506	12902	9921	10788	10516	9138	7414	7160	4279	
210150_s_at	1228	2398	1601	1924	2862	2751	942	1297	1141	1133	1805
	914	806	400	476	407	241	469	634	1368	1058	
210151_s_at	275	201	141	255	401	164	180	317	183	291	259
	466	108	112	184	100	132	106	64	108	132	

210152_at	64	74	20	78	463	443	113	106	67	48	44	67	31	50	31	47	26	42	56	30	41
210153_s_at	1172	1601	1118	775	674	402	1850	1279	1114	1210	871	567	343	382	628	1252	1212	1538	1115	693	1243
210154_at	1070	1248	1015	409	385	492	1110	1306	862	878	517	318	156	156	224	708	670	581	545	379	612
210155_at	57	66	182	195	360	216	48	302	235	284	44	250	25	43	53	41	37	44	66	59	22
210156_s_at	2533	1038	1782	534	3113	2293	1777	1380	1631	1304	2240	2369	3450	2749	2465	2229	1904	1847	1673	1665	1505
210157_at	91	116	118	290	23	59	38	107	217	66	36	102	169	100	96	194	216	171	437	265	333
210158_at	179	207	270	296	250	151	362	323	241	317	240	186	8	26	9	34	29	20	49	60	69
210159_s_at	82	58	266	1824	360	132	41	83	357	338	18	25	18	6	12	10	18	27	207	210	207
210160_at	987	519	178	206	394	237	971	971	193	243	271	253	268	381	253	295	245	287	289	165	131
210161_at	138	67	79	114	664	477	95	77	135	75	102	138	40	13	78	83	51	15	99	33	19
210162_s_at	255	259	134	96	287	228	359	360	155	194	190	279	96	174	175	133	173	160	36	12	38
210163_at	34	18	42	35	30	26	17	36	7	42	9	1	6	7	15	1	21	32	50	46	48
210164_at	91	84	43	98	166	156	90	45	45	78	186	72	479	1339	1481	42	35	27	22	46	30
210165_at	12	9	11	12	16	16	8	9	8	11	11	11	4	4	3	3	5	3	4	3	2
210166_at	179	198	109	138	286	302	232	207	138	211	129	206	32	8	29	45	60	84	32	5	56
210167_s_at	124	144	69	74	15	28	87	33	54	89	118	8	3	6	3	2	12	34	3	9	3
210168_at	176	91	140	173	209	83	175	202	193	150	213	201	42	24	10	24	5	17	37	24	18
210169_at	263	221	265	263	353	425	200	253	223	260	283	205	44	81	44	58	65	76	70	92	66
210170_at	112	69	62	58	254	116	54	75	20	41	88	165	119	91	73	48	28	13	9	4	4
210171_s_at	11	2	3	34	21	24	9	4	13	8	10	6	3	1	1	1	3	2	2	3	1
210172_at	160	163	115	123	41	192	200	317	227	247	138	187	38	34	41	66	82	38	39	37	25
210173_at	20	45	15	31	114	46	82	108	80	162	301	171	28	7	5	20	18	3	3	5	1
210174_at	115	18	68	171	29	62	58	7	100	91	55	89	36	24	4	104	156	152	79	117	140
210175_at	452	343	343	150	366	266	305	214	318	317	392	271	402	565	373	287	267	303	779	355	328
210176_at	64	27	71	80	164	70	89	75	72	73	58	75	16	7	15	23	28	26	15	11	25
210177_at	184	164	34	80	257	137	139	190	191	85	273	79	227	167	198	128	193	252	256	169	340
210178_x_at	844	640	1248	863	907	323	1427	1221	1854	1427	1444	799	1836	1765	1342	1090	1096	1426	1463	787	1426

210179_at	32	9	8	28	30	36	41	26	12	35	13	31	3	11	5	9	6	7	6	7	6
210180_s_at	298	91	248	67	322	36	425	376	524	415	497	387	692	378	80	63	89	78	53	48	56
210181_s_at	49	5	115	72	16	30	28	13	197	249	9	57	13	25	10	3	6	2	36	44	29
210182_at	161	82	157	207	506	493	256	99	108	162	109	176	35	33	58	52	46	62	63	54	30
210183_x_at	5476	7220	7673	8898	3390	5362	5805	5863	5679	6295	3584	5867	4534	4027	3667	4951	5079	4232	4546	5877	3090
210184_at	24	15	24	16	99	74	20	22	29	34	30	20	40	3	11	28	10	9	48	51	11
210185_at	340	167	270	229	482	410	162	212	235	222	300	239	8	16	16	49	46	5	20	18	34
210186_s_at	1107	1232	1035	971	1484	1322	1716	1859	1461	1602	2005	1968	856	415	387	265	463	410	447	287	435
210187_at	225	195	221	119	341	277	182	169	169	131	196	153	113	116	201	238	234	288	250	142	116
210188_at	228	154	168	178	48	90	278	379	165	194	209	347	174	132	141	186	130	206	90	97	118
210189_at	21	55	80	132	14	65	52	49	37	55	24	70	31	19	14	6	28	15	49	39	28
210190_at	7	23	4	5	30	23	5	7	15	7	44	11	6	3	17	9	8	7	4	3	5
210191_s_at	304	246	471	521	606	513	309	425	450	657	725	578	336	466	394	247	181	87	228	182	232
210192_at	74	10	31	132	25	16	123	88	98	180	43	102	1	28	12	33	3	8	4	4	26
210193_at	111	51	111	97	146	113	53	79	75	75	50	97	25	31	25	30	27	29	55	14	43
210194_at	8	5	4	88	54	9	38	12	5	24	4	3	3	2	2	8	6	1	32	7	15
210195_s_at	149	167	148	166	423	574	142	140	103	183	192	190	368	345	543	30	30	31	36	29	40
210196_s_at	30	1	5	4	81	15	24	13	29	3	23	5	217	167	124	23	8	23	4	20	3
210197_at	235	131	250	263	107	108	242	264	316	268	205	175	65	46	86	117	96	64	195	107	92
210198_s_at	60	7	56	16	133	184	65	34	20	49	63	36	18	36	3	14	6	6	30	31	25
210199_at	110	136	152	111	19	38	187	241	106	138	145	28	45	8	7	3	10	4	8	2	3
210200_at	487	302	342	345	550	434	280	624	292	253	232	288	35	7	38	89	55	28	90	64	43
210201_x_at	1110	1331	1224	1549	1043	1230	1090	887	1595	1690	1342	711	466	459	340	450	485	399	394	724	599
210202_s_at	270	352	448	517	976	484	356	244	681	861	502	240	272	245	192	203	175	232	295	255	269
210203_at	42	32	71	45	38	70	52	54	55	80	87	82	32	10	5	24	24	30	53	33	49
210204_s_at	120	94	88	172	65	73	135	154	119	184	121	124	23	24	46	43	52	79	37	22	36
210205_at	50	27	37	433	177	347	27	48	155	179	21	200	93	71	65	75	86	67	114	172	71

210206_s_at	347	215	625	164	73	55	445	420	726	629	426	234	47	1	25	4	28	31	91	52	31
210208_x_at	2985	2871	1903	1649	2128	1603	3383	3686	1605	2155	2601	1499	1602	1891	3717	4405	4402	2849	1677	1978	1839
210210_at	320	204	233	239	418	450	253	342	246	394	303	133	58	36	66	59	32	37	93	84	76
210211_s_at	15719	10743	10617	6745	21275	14887	15483	17528	12728	9736	13629	16744	18208	19215	18875	16828	15965	17721	15625	9356	10618
210212_x_at	537	569	728	775	515	806	448	321	812	405	770	684	618	663	775	515	540	432	923	704	590
210213_s_at	3348	1425	4452	2585	3237	2810	3660	2598	3681	3863	5767	3093	8146	5906	4312	2791	2866	2893	3499	3866	2497
210214_s_at	135	142	391	287	329	310	242	244	245	314	414	271	169	142	100	38	58	31	44	74	81
210215_at	210	184	20	21	136	97	129	84	138	22	148	89	67	86	64	114	189	172	69	51	52
210216_x_at	824	421	1308	837	723	508	867	678	1337	1718	881	745	974	754	777	673	641	514	1713	1228	1158
210218_s_at	102	39	171	281	117	165	133	26	102	165	68	70	119	173	144	115	117	231	207	407	581
210219_at	97	80	208	253	216	145	74	169	127	175	250	172	42	45	85	26	51	75	88	143	174
210220_at	275	566	275	702	429	809	335	315	126	99	694	895	631	482	537	399	379	432	295	496	622
210221_at	149	61	119	125	55	191	127	123	153	175	101	116	44	15	43	22	38	7	18	34	7
210222_s_at	48	90	75	39	26	48	97	73	55	32	53	70	29	5	27	34	82	50	15	4	5
210223_s_at	389	271	423	583	986	1179	413	399	314	340	358	347	80	81	159	101	144	181	95	139	158
210224_at	402	308	290	625	224	406	239	261	151	200	327	245	66	61	137	112	101	122	113	174	150
210225_x_at	49	46	88	71	338	781	36	51	32	55	20	46	10	22	13	15	30	25	21	13	33
210226_at	259	159	69	446	720	652	266	348	377	113	193	163	44	20	121	62	55	63	50	29	17
210227_at	124	86	118	92	21	94	97	139	110	131	78	77	18	17	20	1	35	20	18	9	18
210228_at	101	107	96	149	612	371	19	102	161	42	88	59	7	9	26	65	11	49	50	77	42
210229_s_at	37	22	27	164	60	69	74	28	41	21	850	866	133	104	94	21	7	40	45	7	4
210230_at	377	539	492	757	40	30	606	1213	642	654	196	238	72	67	38	82	61	215	168	207	110
210231_x_at	11814	15007	8563	5141	3686	3112	14525	12324	8139	7315	8200	4651	13746	12509	13885	10428	10478	8370	10012	13158	9507
210232_at	157	187	151	263	327	288	225	212	278	270	246	238	29	49	11	20	38	36	74	18	47
210233_at	12	12	33	75	82	94	48	26	37	38	31	67	43	45	57	23	28	32	168	46	135
210234_at	339	369	129	463	169	230	211	319	373	246	334	320	7	13	47	47	51	41	38	58	42
210235_s_at	572	395	1095	718	535	543	545	562	1078	1027	608	446	234	174	176	140	203	214	402	303	378

210236_at	183	154	241	138	205	258	125	224	494	323	114	165	103	122	218	347	270	375	748	533	753
210237_at	67	326	61	48	252	198	50	45	44	35	30	38	15	56	85	72	188	203	14	16	7
210239_at	41	33	221	175	49	181	53	90	122	196	259	146	100	75	96	37	46	16	102	40	50
210240_s_at	183	152	171	159	129	94	163	255	84	280	182	150	144	88	17	16	35	15	41	5	15
210241_s_at	24	6	12	234	212	435	8	5	60	85	171	224	85	183	125	12	21	31	137	74	62
210242_x_at	433	331	545	621	1339	1158	162	342	318	404	365	381	260	137	207	160	144	90	243	255	210
210243_s_at	1659	1133	1192	1028	515	700	1133	1039	1347	1308	540	315	381	421	302	494	564	526	700	633	590
210244_at	21	30	22	48	43	54	33	36	37	38	26	35	32	8	7	7	5	12	6	11	14
210245_at	13	16	18	12	34	35	13	22	15	27	53	19	7	11	5	8	11	7	8	11	3
210246_s_at	45	18	37	23	33	38	38	44	23	42	21	45	13	13	7	5	10	2	8	6	9
210247_at	410	572	316	361	452	359	513	552	252	330	298	166	28	64	84	282	328	302	113	54	47
210248_at	189	50	71	213	19	255	207	80	28	217	128	52	59	28	2	3	2	7	15	6	21
210249_s_at	478	460	753	996	1127	1926	505	453	554	601	1006	944	423	343	457	160	138	144	391	377	458
210250_x_at	1652	1083	1921	1068	1563	1379	2160	1791	2301	2302	1738	2348	3307	5995	4062	4421	4289	4719	2242	2118	2517
210251_s_at	79	67	81	78	166	269	103	83	71	73	34	72	62	90	38	65	99	81	74	96	76
210252_s_at	445	489	638	803	759	599	518	417	729	1062	617	550	542	452	334	235	245	230	593	704	674
210253_at	201	241	426	411	256	273	229	189	396	435	239	235	408	574	262	352	356	377	1230	571	383
210254_at	11	10	37	8	142	42	8	5	33	38	53	30	3	3	5	3	3	25	6	17	2
210255_at	89	79	115	177	198	304	110	128	102	84	128	202	182	174	161	90	117	139	90	100	129
210256_s_at	33	19	156	43	110	387	36	211	39	42	234	19	61	60	62	45	66	113	80	17	80
210257_x_at	263	292	515	557	1405	3177	726	813	582	552	1829	1560	1045	869	850	748	583	767	443	597	764
210258_at	33	12	1	27	15	39	7	7	12	1	3	3	1	10	7	1	6	1	1	9	2
210259_s_at	161	161	167	150	40	90	224	164	127	164	165	217	50	38	21	7	14	18	10	6	3
210260_s_at	651	706	1710	2124	179	220	551	810	1741	2200	259	450	261	253	234	720	675	632	2940	1274	1972
210261_at	25	12	27	13	23	23	74	77	7	15	84	9	4	4	5	2	5	4	18	12	27
210262_at	20	11	30	18	117	27	12	7	15	11	14	39	15	9	19	3	15	13	6	7	12
210263_at	131	117	179	156	410	636	60	129	29	109	55	26	25	28	44	72	59	53	71	51	42

210264_at	287	145	1264	616	793	634	155	247	755	927	785
	303	99	147	214	47	14	22	257	301	265	
210265_x_at	22	11	19	39	219	172	21	22	32	28	18
	28	5	12	9	6	20	1	29	34	7	
210266_s_at	1228	1040	1261	1304	1425	860	1132	1188	1148	1138	1330
	1010	772	877	1205	2245	1851	1517	1660	1786	1854	
210267_at	17	13	15	30	11	11	28	17	11	57	23
	14	12	26	11	15	28	6	5	35	3	
210268_at	71	23	73	72	137	83	70	121	119	85	91
	5	11	17	18	56	51	55	82	49	47	
210269_s_at	228	260	129	96	121	425	203	256	222	124	323
	211	135	90	53	79	74	51	48	71	66	
210270_at	13	16	15	17	40	23	9	11	19	10	10
	9	10	7	6	51	26	7	5	5	2	
210271_at	201	131	184	171	279	266	246	198	218	242	264
	220	29	7	33	36	35	16	13	35	21	
210272_at	58	82	27	48	503	316	44	55	76	50	48
	18	48	25	33	30	38	20	55	7	37	
210273_at	791	1308	167	338	232	258	1395	1070	278	203	166
	186	26	15	19	1240	1193	650	27	52	29	
210274_at	108	43	39	91	250	179	64	18	86	88	82
	147	1	10	18	54	3	32	21	30	1	
210275_s_at	4093	7135	5248	5605	5655	4768	3482	3564	4823	4418	5101
	3915	5802	6723	7844	7516	7307	7618	6540	6617	10517	
210276_s_at	2344	1748	1354	1789	371	431	3240	3728	740	1936	711
	1003	373	428	155	682	775	512	264	451	309	
210277_at	143	119	148	53	298	293	86	127	83	45	209
	204	264	262	316	251	285	179	252	176	190	
210278_s_at	99	108	95	67	161	267	86	96	96	64	180
	165	275	261	151	164	105	110	100	66	114	
210279_at	45	5	11	14	70	128	36	70	11	41	85
	24	7	13	5	20	6	24	15	32	16	
210280_at	58	109	134	28	117	146	113	106	95	165	21
	24	8	9	39	43	70	64	45	21	71	
210281_s_at	116	39	134	203	132	241	93	98	261	191	306
	250	124	130	46	30	56	53	124	107	148	
210282_at	36	34	38	58	7	8	23	26	79	70	55
	75	67	35	4	9	19	4	64	48	56	
210283_x_at	2717	2139	2640	2030	2507	2874	1786	1953	2141	1747	1582
	1398	1481	1240	1351	2173	2078	2165	2517	2143	2804	
210284_s_at	557	579	822	801	880	972	1170	1441	894	1054	1256
	872	317	412	278	448	383	567	313	329	501	
210285_x_at	1722	1691	926	791	988	1141	1407	1269	1005	769	1430
	1330	991	602	825	1525	1240	1302	792	603	983	
210286_s_at	71	38	239	93	77	89	94	69	159	228	60
	97	17	6	18	38	11	55	135	43	122	
210287_s_at	13	9	19	10	26	32	7	30	20	7	21
	16	2	15	15	20	8	11	36	17	8	
210288_at	382	212	373	414	460	266	396	408	429	390	325
	276	58	53	105	59	35	78	61	46	52	
210289_at	13	21	12	84	192	163	101	16	67	18	21
	10	5	14	5	12	16	8	24	3	3	
210290_at	127	79	151	145	356	277	40	124	107	110	180
	255	195	186	249	58	83	77	137	180	143	

210291_s_at	48	39	66	40	230	43	69	66	75	108	119	111	106	105	178	79	62	51	44	86	114
210292_s_at	67	30	84	13	170	60	81	69	67	32	45	53	8	22	15	13	6	23	11	21	3
210293_s_at	4290	1540	1097	491	1735	2561	1982	2123	836	938	854	1038	1983	1567	2477	5113	5015	4896	4040	3343	4071
210294_at	38	192	37	48	65	44	37	40	45	35	77	41	11	6	8	29	73	11	27	7	39
210295_at	17	9	22	9	27	24	16	15	28	22	13	30	3	3	3	2	3	6	4	3	4
210296_s_at	939	1005	595	952	823	1352	1479	1692	487	443	446	681	539	658	853	2584	2652	2747	1037	871	1315
210297_s_at	7	9	23	48	12	46	9	8	8	22	37	23	2	36	2	27	21	23	12	8	10
210298_x_at	235	164	30	124	173	317	235	243	82	140	728	552	53	117	117	264	108	78	4	4	13
210299_s_at	167	136	39	28	513	927	136	260	43	1	320	401	99	113	129	314	228	166	15	31	33
210300_at	112	75	172	129	878	863	136	168	170	159	149	108	68	17	57	73	41	18	99	59	25
210301_at	208	208	450	457	535	523	305	290	388	391	399	305	56	35	51	44	51	29	52	48	41
210302_s_at	79	38	168	122	256	247	155	149	112	166	141	97	8	6	31	16	3	21	28	39	11
210303_at	7	6	18	65	73	55	57	24	27	3	10	6	1	9	4	2	5	3	1	8	23
210304_at	22	47	20	30	352	271	25	15	8	10	30	14	7	15	41	97	50	89	53	38	27
210305_at	8	10	5	43	36	218	16	8	9	10	7	40	2	2	12	10	1	4	5	1	21
210306_at	176	251	72	268	106	220	99	212	103	126	91	107	54	14	50	46	48	41	50	64	52
210307_s_at	156	221	578	304	47	95	256	238	328	359	222	209	75	47	115	40	97	62	103	73	48
210309_at	37	11	15	31	48	43	45	22	59	22	61	35	8	3	10	9	7	8	6	4	5
210310_s_at	11	7	11	17	16	16	7	9	7	6	4	16	3	12	2	2	3	1	8	7	1
210311_at	101	68	126	123	198	245	162	80	55	180	169	112	11	12	42	5	37	47	36	17	45
210312_s_at	349	1249	1305	1496	841	1820	469	581	558	869	1060	1556	1443	1602	1485	805	866	802	1159	2108	1703
210313_at	36	22	103	59	1021	681	16	15	31	10	24	34	10	16	67	44	34	47	29	14	22
210314_x_at	19	91	722	878	104	183	126	62	743	305	352	173	254	254	267	140	167	117	597	810	608
210315_at	140	108	20	61	224	206	146	153	96	34	26	23	20	15	17	36	25	45	41	34	33
210316_at	49	78	77	65	285	293	23	79	70	46	64	10	24	33	25	15	13	18	62	37	35
210317_s_at	3007	494	4388	2856	1097	1044	4611	4483	4430	4780	4983	2963	1524	1506	3607	3813	3597	3675	4496	1586	3194
210318_at	36	17	14	19	32	23	23	21	59	18	16	28	10	22	10	27	12	28	7	34	6

210319_x_at	273	260	208	107	322	359	636	802	110	233	408	348	339	254	306	204	339	408	176	256	281
210320_s_at	562	337	775	705	493	589	621	550	1053	860	550	455	563	478	511	336	397	477	1568	693	1114
210321_at	20	10	16	12	48	43	11	12	8	21	7	9	6	6	53	5	5	4	4	2	2
210322_x_at	24	117	141	217	51	30	103	143	70	168	104	136	16	12	7	7	20	20	17	6	3
210323_at	17	57	68	144	51	54	36	9	20	32	27	24	40	17	19	34	10	35	19	25	31
210324_at	7	5	5	25	71	35	7	5	15	7	7	5	4	4	16	5	32	30	11	4	2
210325_at	262	129	289	208	276	259	227	281	387	270	213	245	7	25	22	37	34	22	47	17	27
210326_at	151	49	194	43	19	16	53	67	215	25	124	232	3	12	10	3	7	6	9	24	24
210327_s_at	17	16	4	47	69	26	4	9	7	6	9	26	48	40	7	4	14	18	47	50	2
210328_at	13	89	16	25	59	60	53	13	23	39	45	60	26	4	3	15	5	2	6	8	3
210329_s_at	60	10	75	25	330	184	28	16	95	24	4	87	32	4	5	36	53	43	27	39	21
210330_at	77	32	90	23	98	122	36	42	70	85	17	60	11	22	26	20	5	10	23	3	13
210331_at	226	190	224	155	482	262	187	226	173	233	196	194	39	31	37	61	42	49	42	4	21
210332_at	28	47	52	35	242	269	74	34	64	136	47	28	35	40	77	54	47	49	55	27	47
210333_at	11	22	15	14	15	79	81	25	54	14	18	63	13	11	60	29	12	35	10	2	26
210334_x_at	1207	459	789	290	1041	86	962	1423	1164	1234	1430	1263	984	672	479	428	399	619	354	242	211
210335_at	64	105	151	67	150	69	61	30	84	113	11	69	18	15	36	21	17	8	43	85	135
210336_x_at	601	858	609	1121	59	231	197	371	498	622	375	175	196	225	257	328	484	394	358	577	528
210337_s_at	3757	2164	4937	1900	3112	1419	3334	2635	4069	3706	3728	2250	1279	1284	1409	1136	1104	1095	1203	1045	1056
210338_s_at	11966	7275	22910	13339	26212	16559	27581	23937	28151	20207	23865	27870	18057	20267	18726	15897	16558	18123	14728	11155	13548
210339_s_at	110	130	184	176	289	323	27	88	111	89	60	33	37	7	50	44	5	8	13	25	35
210340_s_at	15	16	3	6	11	39	12	44	27	34	6	38	2	9	3	9	28	3	2	1	7
210341_at	52	12	15	10	186	220	30	48	16	15	38	13	37	10	7	28	36	35	10	56	46
210342_s_at	9	5	9	8	15	22	23	21	7	42	9	13	6	2	4	5	5	1	7	5	2
210343_s_at	83	55	16	136	69	121	45	103	74	74	38	80	14	6	4	10	5	7	6	2	3
210344_at	17	12	8	17	36	34	8	8	12	14	9	6	4	6	7	6	7	7	6	6	6
210345_s_at	114	103	163	84	26	28	131	148	202	191	136	186	28	7	5	21	5	9	12	3	19

210346_s_at	282	257	307	431	595	692	246	416	272	333	394	442	337	357	407	535	432	600	364	398	719
210347_s_at	269	157	194	200	153	180	244	205	206	239	180	300	28	32	178	46	34	3	43	50	38
210348_at	161	49	60	39	32	121	37	116	92	120	27	13	23	27	16	10	26	12	11	17	5
210349_at	110	109	76	17	47	44	133	91	84	53	134	113	10	24	57	81	121	80	41	7	56
210350_x_at	189	125	100	76	37	19	245	278	246	147	149	114	245	222	110	111	117	112	71	66	98
210352_at	122	119	153	188	209	134	121	80	76	106	126	73	10	9	15	17	23	14	36	19	31
210353_s_at	12	12	12	12	19	35	13	17	13	15	17	15	2	2	4	2	3	4	8	2	1
210354_at	127	100	111	105	239	125	110	116	119	122	128	158	16	3	55	17	28	17	20	17	17
210355_at	62	38	37	23	91	133	42	26	83	78	30	156	54	106	52	20	14	5	36	34	35
210356_x_at	139	40	121	153	74	48	180	205	242	226	180	142	11	5	61	58	26	30	28	57	40
210357_s_at	491	387	1221	231	771	789	305	391	494	890	581	459	258	284	370	618	789	982	651	927	731
210358_x_at	366	423	233	237	261	75	525	418	242	280	436	380	181	247	126	142	86	127	96	119	82
210359_at	32	41	110	162	201	95	20	128	139	215	71	28	20	2	5	30	6	18	10	7	5
210360_s_at	107	112	114	159	126	222	91	96	106	224	131	129	20	22	5	33	30	16	38	11	36
210361_s_at	118	207	227	266	216	378	119	187	197	187	104	197	185	194	156	216	207	197	236	157	222
210362_x_at	66	187	119	103	18	12	286	185	149	126	472	82	14	58	6	4	76	23	6	73	35
210363_s_at	12	22	8	18	16	63	5	9	17	11	4	16	4	8	5	2	9	3	4	7	3
210364_at	57	18	14	14	21	26	23	44	64	45	43	68	17	35	5	6	16	21	8	40	20
210365_at	147	69	75	113	342	292	168	132	179	152	335	295	215	160	150	19	3	20	20	76	25
210366_at	86	131	77	175	47	106	21	162	68	57	57	48	13	35	34	8	19	50	40	53	45
210367_s_at	558	977	298	314	818	660	677	449	207	208	478	259	209	147	175	513	509	397	65	119	88
210368_at	7	10	5	12	10	23	7	11	11	10	7	10	4	7	5	22	19	38	6	7	3
210369_at	61	36	47	45	40	22	32	78	169	53	98	128	19	46	7	14	2	17	34	32	6
210370_s_at	87	13	15	14	63	30	23	21	23	10	18	23	7	7	6	2	3	10	8	2	4
210371_s_at	2639	2848	2062	1552	2540	2165	2900	3441	1773	1704	2174	2899	4517	3077	5500	7869	6267	6247	5834	3608	5108
210372_s_at	1213	1978	799	1380	620	657	1477	1039	1224	889	728	493	1031	766	791	829	842	765	539	600	634
210373_at	136	33	43	44	41	36	33	22	41	35	18	33	5	54	11	7	61	5	6	9	39

210374_x_at	114	54	155	47	228	227	115	148	103	117	131	68	20	29	30	26	48	50	39	51	18
210375_at	42	23	28	25	103	188	32	8	31	35	13	25	2	19	18	14	3	13	6	1	2
210376_x_at	609	432	875	608	496	298	676	764	752	1036	816	383	175	96	57	45	102	84	108	84	78
210377_at	30	17	251	1023	30	90	73	157	312	370	67	28	27	7	2	101	74	54	836	568	790
210378_s_at	972	742	1116	966	1402	669	1166	1149	1196	1351	1772	1346	1357	892	488	262	273	248	517	362	183
210379_s_at	132	139	282	241	231	314	351	376	349	317	341	299	332	222	250	324	193	236	496	367	504
210380_s_at	54	74	8	70	126	44	53	90	54	46	37	52	17	42	5	40	48	51	29	32	23
210381_s_at	177	92	138	175	102	99	83	129	127	127	151	126	20	4	26	12	32	8	26	3	3
210382_at	28	12	16	25	73	81	102	63	28	81	58	15	4	25	3	31	10	12	8	44	3
210383_at	36	34	31	4	70	39	44	22	8	42	10	34	3	2	23	12	11	1	2	11	1
210384_at	11	33	58	6	51	59	61	34	60	49	31	83	6	17	3	6	11	3	29	3	4
210385_s_at	213	311	167	309	514	660	248	177	155	179	422	525	178	142	176	80	93	89	67	149	241
210386_s_at	2814	2673	1152	1331	1419	1333	2361	1698	1123	818	1391	811	1658	1290	971	1438	1257	1449	1536	1521	686
210387_at	13	2	98	268	195	191	4	5	194	127	142	314	195	127	191	24	15	15	81	120	138
210388_at	33	32	34	43	95	85	25	33	47	39	38	38	7	9	8	9	7	7	7	6	7
210389_x_at	421	181	353	281	777	554	391	428	268	393	375	371	396	453	269	514	583	577	432	285	359
210390_s_at	21	6	402	577	12	43	45	44	454	788	17	90	13	10	13	2	19	4	1104	486	299
210391_at	44	34	20	30	23	17	29	26	21	24	26	31	4	7	4	13	8	4	4	3	7
210392_x_at	146	77	141	234	309	636	215	189	140	137	260	166	91	105	51	48	71	35	69	60	62
210393_at	26	12	186	210	287	31	29	25	326	914	13	23	3	25	2	9	24	4	165	102	129
210394_x_at	108	79	126	169	313	300	196	166	132	182	213	165	108	65	97	35	5	40	28	17	4
210395_x_at	329	296	364	304	346	540	391	271	363	261	320	410	171	85	78	59	76	88	73	122	43
210396_s_at	3026	3021	1329	693	1436	1588	1891	1642	1044	1140	1424	1157	2118	2364	3222	5170	4493	5005	4274	3504	3598
210397_at	77	72	96	304	383	318	103	135	153	91	48	82	4	5	29	6	45	4	32	62	40
210398_x_at	17	51	15	19	15	20	8	9	9	10	4	9	1	6	3	7	3	2	5	4	6
210399_x_at	411	337	525	495	394	249	537	268	391	552	195	393	72	68	107	86	51	39	77	82	39
210400_at	15	21	18	13	22	15	17	17	11	15	9	10	4	14	8	4	7	6	4	3	2

210401_at	102	134	141	175	128	109	127	129	80	122	153	226	43	33	73	42	11	3	6	10	3
210402_at	15	12	15	12	29	35	21	29	11	11	51	28	12	15	11	2	12	12	3	11	17
210403_s_at	26	19	20	22	26	54	25	28	20	24	21	24	1	8	2	2	7	2	1	2	9
210404_x_at	37	69	41	31	210	102	192	176	110	154	185	162	13	59	41	6	20	35	3	6	11
210405_x_at	593	712	533	265	69	267	886	676	684	530	605	481	82	71	101	191	167	179	142	84	128
210406_s_at	2737	2119	5411	3487	2590	4415	2135	2066	3194	3606	3037	2770	3419	2590	5546	4685	3583	3656	6126	7347	8530
210407_at	105	55	87	76	175	15	87	92	75	89	70	127	86	98	49	72	70	61	85	31	36
210408_s_at	15	27	8	27	56	62	19	26	21	22	18	21	4	6	3	3	28	3	5	5	2
210409_at	78	5	18	175	76	50	93	59	27	99	21	73	16	4	2	15	32	17	39	27	33
210410_s_at	319	417	109	63	47	32	350	274	54	50	24	113	41	76	61	143	146	193	145	141	123
210411_s_at	74	7	46	25	169	142	34	50	140	52	24	28	7	12	10	5	7	4	9	9	3
210412_at	216	100	142	155	203	266	193	172	166	180	192	80	28	25	14	30	10	39	49	27	50
210413_x_at	135	88	129	94	190	152	156	79	187	161	129	114	13	7	62	44	49	35	50	53	40
210414_at	108	82	235	219	235	280	167	248	234	173	200	129	17	18	10	7	19	4	6	24	18
210415_s_at	173	28	117	18	65	67	46	80	43	66	98	258	99	70	127	35	35	66	77	72	96
210416_s_at	746	319	846	406	329	160	709	685	983	959	371	456	254	244	204	428	433	550	939	402	395
210417_s_at	1064	1071	1019	1442	720	765	1179	1061	995	864	1106	655	239	190	226	200	225	311	256	217	218
210418_s_at	3209	2143	1489	1185	1894	1677	3269	2423	2234	1228	3439	1439	3329	3405	3230	2299	2501	2401	1642	1707	1342
210419_at	20	10	31	241	444	501	23	16	27	24	23	18	51	56	67	13	8	7	12	24	18
210420_at	83	73	24	133	382	433	135	73	134	91	118	136	7	35	27	3	29	34	39	35	46
210421_s_at	42	19	19	21	32	35	30	30	19	25	61	15	4	4	5	6	7	5	4	21	28
210422_x_at	550	478	438	438	855	926	762	623	684	588	652	573	91	163	63	120	16	70	130	125	129
210423_s_at	193	175	255	304	70	46	272	272	265	404	257	239	33	40	86	12	37	78	60	69	16
210424_s_at	226	251	114	253	323	386	305	459	393	328	244	121	6	9	9	5	17	5	7	18	9
210425_x_at	615	1918	494	1296	650	1007	929	1252	1018	1205	715	496	163	142	219	357	177	185	236	387	350
210426_x_at	86	4	83	23	285	189	111	49	7	99	242	192	139	208	279	21	14	11	61	61	38
210427_x_at	18155	29329	23815	17604	30215	32018	21732	24282	20751	26784	25626	45879	17102	24406	26127	21010	23139	23420	12890	16560	13976

210428_s_at	3729	5269	3397	2082	1409	935	3503	3540	2790	2461	3201	1585	943	1027	1179	1356	1366	1382	1093	721	634
210429_at	65	36	7	59	21	13	27	67	4	14	11	50	1	1	15	29	8	28	1	1	2
210430_x_at	67	49	33	61	29	27	66	75	35	74	45	113	7	11	27	6	9	13	10	14	8
210431_at	32	202	24	137	51	173	38	49	44	41	44	44	38	6	6	25	11	42	14	11	11
210432_s_at	9	7	5	10	32	42	38	9	7	4	6	3	1	9	2	3	12	7	1	2	14
210433_at	8	28	23	16	34	98	33	17	5	15	47	15	37	4	5	30	5	3	8	7	21
210434_x_at	6460	12098	9224	8084	5752	4929	6444	5739	8977	8458	5897	6795	8418	8884	11026	8888	9970	9467	10462	14063	10790
210435_at	12	34	8	9	154	114	13	44	5	10	16	11	28	11	3	6	6	11	23	17	21
210436_at	173	101	170	164	198	113	256	205	199	239	173	129	17	27	25	15	18	26	18	41	19
210437_at	118	72	125	102	216	352	156	84	35	64	112	49	19	66	44	52	41	67	53	27	15
210438_x_at	2261	2451	1743	1580	1196	1195	2196	2487	1874	1694	1487	1161	809	991	1169	1339	1142	913	1014	967	986
210439_at	8	7	14	10	63	94	12	7	5	10	6	8	2	1	3	2	19	6	2	3	1
210440_s_at	144	84	199	125	360	325	150	160	118	201	153	129	26	21	7	7	20	13	25	44	39
210441_at	40	40	30	47	98	66	78	26	39	157	125	68	26	11	9	13	4	3	6	7	2
210442_at	73	47	212	53	99	171	61	53	40	92	43	87	7	11	17	14	3	3	13	34	7
210443_x_at	855	816	842	817	223	542	869	870	753	1034	890	562	242	257	246	226	389	368	338	298	248
210444_at	110	124	209	189	282	386	307	252	182	245	153	190	11	24	21	23	2	6	15	46	8
210445_at	71	22	33	35	26	30	29	45	13	34	7	9	2	4	6	98	98	65	56	56	30
210446_at	130	134	118	71	85	67	102	82	119	82	112	123	10	32	27	45	32	40	16	17	29
210447_at	26	29	16	25	100	130	34	49	41	35	36	41	1	15	11	15	3	18	27	19	2
210448_s_at	487	437	31	41	836	586	437	135	52	45	728	484	500	398	840	389	389	341	71	283	236
210449_x_at	523	358	347	612	264	379	851	730	783	665	786	559	87	142	158	135	123	113	120	139	122
210450_at	89	6	11	75	213	16	12	17	7	25	6	6	3	1	3	20	6	7	21	3	2
210451_at	48	11	28	21	44	23	41	15	20	31	27	21	18	38	11	2	2	10	2	2	1
210452_x_at	36	49	42	23	92	441	60	24	25	29	37	35	8	41	5	56	70	10	44	19	5
210453_x_at	7123	7593	8783	10120	8266	6772	10165	7945	10503	8725	10623	11359	15913	14559	12240	9073	9419	9613	8170	7701	5707
210454_s_at	114	32	104	115	263	188	30	33	54	29	53	36	23	7	23	27	7	9	8	10	12

210455_at	114	46	129	118	187	129	99	173	181	172	172	183	35	60	15	33	41	31	29	37	19
210456_at	20	128	113	59	216	274	15	123	28	96	159	33	10	62	42	26	10	40	11	11	10
210457_x_at	2831	1701	3348	449	448	218	3669	3918	1952	2704	2487	1338	145	117	121	317	216	251	156	69	171
210458_s_at	94	96	227	186	416	290	107	177	231	250	84	165	145	114	42	71	83	91	353	170	418
210459_at	17	36	14	17	427	734	40	18	23	17	21	33	7	12	14	23	10	17	8	7	8
210460_s_at	3549	3811	2676	1623	2620	2189	2953	2469	1889	3184	3328	3181	3935	3321	1325	1655	1888	1270	1481	1760	1173
210461_s_at	185	72	273	341	502	388	580	554	565	556	819	686	980	679	338	280	201	184	378	422	212
210462_at	16	5	8	17	66	51	8	11	11	49	9	15	1	3	3	5	2	2	39	19	16
210463_x_at	873	980	339	137	70	86	677	537	395	335	510	271	370	432	267	404	461	645	360	300	289
210464_at	8	5	53	14	18	16	17	48	19	63	11	9	2	7	22	18	12	1	4	24	5
210465_s_at	400	415	346	345	407	538	260	464	293	309	236	180	149	185	205	273	225	312	279	197	327
210466_s_at	8901	9396	8833	5628	5033	3085	6819	7181	10586	10279	7197	6481	10562	8150	11060	9466	9908	11776	15991	12435	14900
210467_x_at	1036	623	5519	3563	194	15	613	686	5682	5698	97	89	34	45	31	621	665	442	5755	3756	3721
210469_at	134	197	331	316	363	374	195	165	298	282	163	136	34	57	6	18	6	20	60	31	7
210470_x_at	6928	1379	7322	6331	6570	4308	8367	7089	7195	8059	8172	6957	3733	3489	3501	3426	3514	3486	2775	2996	5243
210471_s_at	114	89	136	118	100	188	142	162	151	141	148	107	14	51	69	107	29	25	68	80	37
210472_at	112	120	123	116	88	19	160	129	132	165	125	119	8	17	7	63	11	23	2	17	3
210473_s_at	795	966	553	477	393	394	1179	1192	692	815	492	471	274	213	261	863	828	659	493	425	475
210474_s_at	254	341	155	132	202	361	143	136	110	52	158	109	168	150	55	91	112	121	104	139	99
210475_at	16	21	27	27	88	16	30	18	31	28	45	26	29	11	18	7	26	25	76	30	18
210476_s_at	49	74	19	40	65	78	32	86	35	64	44	89	4	17	7	8	5	8	41	9	8
210477_x_at	351	326	350	304	82	60	242	385	301	309	293	194	36	25	78	110	129	92	63	76	85
210479_s_at	90	35	90	158	296	157	65	113	151	109	189	138	136	182	155	9	5	15	33	63	46
210480_s_at	376	314	629	1092	81	273	477	793	744	917	121	31	89	75	46	187	175	240	275	362	430
210481_s_at	11	9	11	16	113	196	13	17	15	10	24	34	6	9	10	4	11	9	9	7	10
210482_x_at	135	377	58	409	37	62	56	88	62	116	141	28	84	82	57	72	65	48	25	132	64
210483_at	44	101	23	75	312	160	11	87	12	15	18	58	40	65	48	83	117	95	71	51	42

210484_s_at	138	162	113	36	444	663	46	128	155	148	80	69	23	12	21	46	80	32	44	45	43
210486_at	202	112	225	159	132	313	186	269	127	259	23	118	34	55	77	117	127	117	109	68	45
210487_at	20	11	16	14	65	55	12	18	8	15	9	14	3	4	6	4	1	4	6	6	1
210491_at	20	50	8	49	291	58	53	84	29	77	10	70	22	38	7	11	28	72	32	35	27
210492_at	45	38	47	75	48	153	21	29	21	22	13	35	56	48	7	74	57	47	34	3	48
210493_s_at	94	95	28	12	225	141	109	70	45	60	61	106	68	67	72	44	30	42	50	33	24
210495_x_at	344	209	1439	410	1440	1148	596	736	1286	186	1275	1912	1316	1269	1095	131	251	240	250	382	448
210496_at	8	15	3	12	89	125	8	15	11	6	41	5	8	13	5	2	12	28	22	4	2
210497_x_at	29	24	39	31	65	232	33	40	47	34	44	68	75	76	72	3	36	44	41	4	6
210498_at	25	26	7	72	84	66	5	11	11	70	40	3	18	13	10	23	29	23	20	34	7
210499_s_at	177	119	217	243	609	610	126	230	138	177	102	129	68	61	42	69	154	116	156	86	38
210500_at	54	19	8	100	82	13	73	50	116	50	53	49	20	23	25	21	12	16	13	13	9
210501_x_at	6367	5379	7810	7846	5139	5203	5691	5148	8678	6660	5442	5361	7172	6831	7791	5033	4875	4656	6343	7674	4153
210502_s_at	2481	1066	1108	1092	874	1259	2272	1577	1259	1224	745	660	922	956	732	1779	1646	1502	1087	1200	775
210503_at	7	12	16	39	21	118	48	3	7	15	6	19	1	2	6	3	2	8	5	27	3
210504_at	103	88	122	131	175	192	142	88	140	117	82	103	27	66	37	22	24	8	18	21	29
210505_at	183	80	179	172	289	195	138	128	124	184	111	113	30	22	34	28	30	31	29	41	22
210506_at	9	11	19	32	125	28	25	34	28	27	20	20	7	15	9	24	8	9	3	3	11
210507_s_at	21	33	19	79	60	47	11	17	7	13	10	6	11	11	3	5	29	6	6	6	21
210508_s_at	482	431	20	9	30	73	596	669	32	24	27	29	25	40	79	197	225	246	6	4	9
210510_s_at	33	9	34	13	121	36	29	8	39	11	151	97	263	373	214	2	5	4	102	32	53
210511_s_at	16	7	12	8	30	65	17	7	7	18	16	15	4	5	6	19	3	8	6	3	5
210512_s_at	1261	1209	1347	308	169	1009	474	501	167	211	196	68	271	222	917	2377	1908	2317	1023	4801	5302
210513_s_at	569	478	799	347	403	371	689	554	516	531	389	420	75	83	60	91	84	69	64	174	100
210514_x_at	1961	2690	792	957	621	1576	1863	2052	777	671	2537	2095	715	621	423	636	486	779	156	383	223
210515_at	136	105	308	230	705	747	178	166	301	240	161	52	86	116	141	114	144	122	234	217	218
210516_at	48	45	60	61	135	163	69	41	51	60	48	67	15	3	32	19	16	3	15	37	7

210517_s_at	14538	17295	4316	208	4473	865	14178	17791	593	790	5324	2511	5764	2118	2515	15949	15766	15133	195	282	498
210518_at	89	120	92	88	305	275	133	186	107	137	134	132	10	27	36	23	29	25	46	39	48
210519_s_at	13110	20798	9632	16990	5965	8378	17053	16053	10517	10880	7177	6338	7436	6862	5591	8270	7644	6994	5731	3912	3398
210520_at	12	7	30	27	136	220	8	17	9	10	9	20	3	2	2	3	10	16	5	4	7
210521_s_at	11	4	8	12	25	12	5	7	8	8	11	8	4	3	1	1	4	1	4	3	7
210523_at	26	44	27	27	21	13	25	26	23	89	84	26	8	28	3	35	29	22	27	2	12
210524_x_at	1887	1549	1480	1337	1453	2134	1679	1849	864	969	1410	1981	1799	792	940	757	957	758	1306	1752	996
210525_x_at	57	12	7	8	74	44	40	46	5	3	18	74	31	28	1	20	8	9	7	25	21
210527_x_at	550	369	638	481	3034	2334	814	1011	562	658	999	813	518	650	116	280	433	379	456	282	219
210528_at	114	22	94	100	43	74	8	38	50	39	53	29	12	19	15	6	19	21	15	39	20
210529_s_at	721	939	1537	1399	333	294	1137	1767	1470	1708	897	999	188	149	67	124	86	127	245	257	205
210530_s_at	93	43	347	204	334	203	87	66	163	190	99	185	94	75	124	35	51	64	363	152	260
210531_at	73	79	187	125	120	28	101	61	181	161	104	108	60	59	60	46	6	32	132	45	90
210532_s_at	4616	4915	5378	4501	9106	8054	6540	6813	4303	3908	8976	10405	11421	14370	11379	9929	9847	9976	6373	6378	4837
210533_at	149	180	68	50	99	44	117	113	91	103	95	94	17	11	14	43	24	21	20	33	3
210534_s_at	523	338	376	263	737	1031	617	530	452	506	669	504	716	805	1343	651	743	507	398	340	350
210535_at	42	23	22	49	92	140	20	12	27	25	16	43	35	58	11	17	64	17	14	4	19
210536_s_at	29	69	118	188	418	242	94	191	146	29	37	138	12	37	3	8	22	22	10	44	19
210537_s_at	21	11	42	23	89	106	106	144	199	61	64	46	14	68	9	57	22	41	74	22	57
210538_s_at	75	314	240	147	304	320	57	83	108	63	422	633	127	146	35	79	118	239	209	326	422
210539_at	33	34	31	110	436	153	27	25	37	21	30	45	12	72	62	4	34	66	41	26	19
210540_s_at	286	229	416	413	478	703	192	282	297	425	320	377	493	300	184	127	136	114	479	367	273
210541_s_at	1482	1432	1480	680	774	883	1626	1215	959	1031	1545	919	467	328	313	292	337	384	177	301	379
210542_s_at	208	130	346	371	44	93	188	248	364	291	210	219	57	89	96	45	61	78	220	242	234
210543_s_at	2959	1471	1805	956	2192	1317	2694	2666	2074	1957	2497	1724	608	685	320	521	416	670	392	333	373
210544_s_at	794	674	993	2376	322	671	988	1071	2042	2168	803	696	143	62	120	277	269	272	390	180	187
210545_at	242	116	203	198	272	121	125	227	249	231	210	82	20	4	9	17	8	5	41	41	27

210546_x_at	7	13	11	13	40	46	12	12	8	11	17	19	3	3	6	3	3	7	3	8	4
210547_x_at	409	291	753	1308	477	1562	321	278	373	560	452	222	293	477	971	452	571	413	671	1419	1299
210548_at	17	2	22	31	33	20	41	28	5	34	43	20	1	2	5	19	4	2	9	2	3
210549_s_at	17	2	45	13	37	85	19	7	17	39	20	36	9	12	5	23	2	16	1	7	6
210550_s_at	36	17	19	14	55	50	29	12	16	18	47	43	65	134	165	71	42	41	36	7	6
210551_s_at	22	15	15	14	32	28	70	29	39	20	68	13	2	3	1	9	2	35	3	21	1
210552_s_at	65	11	76	119	410	558	23	124	92	15	80	107	84	83	68	41	23	62	89	47	39
210553_x_at	140	156	81	85	41	35	152	143	99	198	144	171	75	43	14	57	90	23	13	17	11
210554_s_at	5163	4466	6773	5096	3009	2750	4726	4607	5895	5930	3695	1795	3226	2455	1851	2899	2066	3957	7015	5200	6238
210555_s_at	295	271	491	384	355	340	436	464	549	408	334	153	207	149	148	233	338	221	250	367	433
210556_at	87	33	103	118	151	120	65	110	127	130	87	96	26	21	34	20	23	5	47	54	66
210557_x_at	148	88	50	109	191	120	28	40	48	18	31	13	5	57	90	62	103	11	36	11	25
210558_at	79	19	24	36	107	43	21	50	56	49	10	54	1	28	34	36	7	13	22	62	32
210559_s_at	2703	948	3334	504	2260	168	3172	4302	4165	3876	3353	3911	3401	4037	2916	2837	2820	2966	4284	1576	2254
210560_at	57	54	5	8	32	22	70	65	3	4	71	9	7	8	10	33	2	22	8	4	2
210561_s_at	881	847	1420	1114	887	1826	767	1565	777	1326	1029	1297	915	505	556	671	737	562	1084	856	1262
210562_at	15	6	12	10	62	8	12	13	9	8	7	9	1	14	22	3	2	3	1	2	9
210563_x_at	524	397	1123	1134	759	978	582	404	1271	1027	934	675	578	572	596	391	415	367	836	490	474
210564_x_at	434	320	494	623	410	443	219	259	298	363	455	253	74	161	115	134	116	111	189	189	120
210565_at	15	9	41	74	147	507	13	9	11	53	30	9	3	4	9	37	12	3	50	26	7
210567_s_at	552	164	388	206	280	145	529	438	760	502	469	165	199	169	168	229	221	224	674	93	151
210568_s_at	201	145	402	273	1240	812	244	379	479	442	782	966	890	698	537	247	165	231	550	281	524
210569_s_at	22	5	19	39	29	22	25	20	19	24	14	16	7	3	3	15	5	2	4	25	3
210570_x_at	411	530	576	366	613	606	722	603	500	903	1217	974	663	584	502	411	408	260	578	391	470
210571_s_at	12	18	24	18	40	66	8	17	8	10	9	15	5	2	1	1	2	3	2	2	9
210572_at	26	4	54	78	93	48	7	22	37	27	30	5	14	2	2	2	2	22	20	1	8
210573_s_at	733	463	610	538	731	883	641	387	562	542	841	623	392	188	327	237	197	202	325	242	367

210574_s_at	3761	2510	2554	1434	2815	1724	3266	2408	2745	2832	2645
	1910	3461	2699	2586	2914	2470	2228	2659	1879	1646	
210575_at	19	10	4	4	25	23	4	4	7	4	11
	5	25	24	27	9	21	2	14	1	9	
210576_at	15	2	12	8	144	32	4	3	4	20	7
	4	3	2	1	2	10	35	5	4	13	
210577_at	184	90	88	213	48	52	136	202	207	136	146
	68	29	33	48	35	26	10	8	8	14	
210578_at	176	36	110	226	155	355	159	149	59	110	135
	103	34	19	20	30	35	39	17	14	26	
210579_s_at	134	142	136	176	258	148	123	139	173	158	114
	133	37	4	19	23	6	2	43	28	26	
210580_x_at	1823	2027	903	1327	862	685	1931	1285	916	924	902
	1059	459	572	451	1005	1273	1224	477	518	567	
210581_x_at	369	325	374	335	197	257	439	429	387	419	360
	294	57	61	43	43	31	35	96	44	39	
210582_s_at	360	367	625	855	782	1369	640	521	1029	1194	1319
	519	506	257	223	200	140	227	428	230	246	
210583_at	71	24	133	119	227	191	195	107	151	134	117
	52	15	67	33	29	55	16	38	18	30	
210584_s_at	127	40	121	106	121	228	107	99	123	92	97
	96	17	27	17	6	11	22	25	4	23	
210585_s_at	112	66	30	10	25	58	11	9	66	13	63
	26	9	6	14	6	8	32	11	10	6	
210586_x_at	73	145	31	57	25	44	148	66	63	28	41
	155	7	11	16	13	64	10	8	9	8	
210587_at	87	9	64	38	305	1020	29	55	21	24	70
	48	54	17	267	24	61	76	41	672	583	
210588_x_at	1335	961	1561	1081	3313	1704	1571	1247	2064	1595	1894
	1356	2369	1845	2009	1248	1266	922	1180	1084	963	
210589_s_at	69	46	129	238	76	55	286	183	126	193	223
	136	266	605	296	235	162	345	367	256	396	
210592_s_at	5837	7848	2716	4243	3963	9747	5194	11582	1110	847	5088
	5440	4415	6274	8591	13348	16149	12135	5215	6571	7621	
210593_at	163	106	69	146	129	146	189	128	112	171	139
	85	4	40	34	19	26	44	24	5	13	
210594_x_at	598	568	760	428	587	535	926	839	931	924	1018
	675	296	267	208	203	274	241	279	202	219	
210595_at	26	21	20	124	117	195	23	18	11	57	13
	21	5	3	34	12	2	3	31	22	1	
210596_at	422	412	570	511	466	493	506	536	401	628	647
	563	332	771	1550	1543	1213	1811	967	1687	1685	
210597_x_at	67	193	153	343	829	636	87	219	143	238	267
	241	23	94	86	118	58	68	77	57	62	
210598_at	120	88	111	141	195	169	77	156	115	157	112
	156	33	56	6	36	50	37	68	49	36	
210599_at	57	34	35	50	147	7	9	65	12	17	7
	9	1	14	25	7	4	21	37	31	25	
210600_s_at	8	2	4	54	91	62	17	3	5	3	6
	25	7	2	1	2	1	15	1	6	3	
210601_at	54	40	142	67	111	421	86	110	55	115	78
	99	24	44	67	39	23	18	69	29	11	
210602_s_at	169	7	52	38	188	30	110	92	54	113	92
	13	26	41	30	30	3	2	11	8	20	

210603_at	160	69	260	132	209	237	176	177	235	169	166
	146	27	3	33	41	39	49	28	35	14	
210604_at	15	10	33	22	15	30	16	20	29	15	21
	11	3	6	4	20	16	7	19	7	12	
210605_s_at	263	446	475	547	56	44	398	429	237	516	205
	312	32	34	97	75	92	163	138	97	84	
210606_x_at	123	124	34	26	65	169	32	40	27	38	97
	36	35	5	10	5	7	5	6	21	3	
210607_at	64	34	9	18	140	28	78	11	35	3	47
	30	32	50	38	29	28	34	24	57	39	
210608_s_at	410	337	304	534	646	435	356	327	304	425	412
	309	57	81	114	218	201	245	271	247	196	
210609_s_at	1205	1633	869	1624	1232	1309	1393	1246	788	885	1145
	1085	697	647	635	550	626	376	287	592	325	
210610_at	98	169	30	328	47	66	101	74	150	277	111
	109	21	51	27	11	41	16	62	42	18	
210611_s_at	13	23	16	25	11	50	17	45	9	35	3
	24	1	1	2	2	10	2	3	4	11	
210612_s_at	42	185	23	21	30	42	183	113	84	25	87
	264	253	236	93	99	86	102	161	116	143	
210613_s_at	187	120	26	9	359	410	154	206	11	7	216
	113	134	217	138	228	261	245	51	71	15	
210614_at	94	63	117	168	256	163	127	146	147	211	166
	82	59	51	94	12	13	17	4	3	17	
210615_at	53	16	20	25	180	159	58	21	19	29	44
	18	2	29	4	12	9	4	18	26	7	
210616_s_at	1254	1080	1658	1496	1167	1295	1375	1277	1365	1350	1128
	1155	889	509	399	702	1034	836	957	1307	1572	
210617_at	222	192	190	263	33	219	167	247	186	257	225
	273	39	54	74	53	11	13	55	49	48	
210618_at	123	45	30	63	76	23	131	67	100	53	88
	155	30	8	20	5	9	8	8	31	24	
210619_s_at	230	82	156	238	85	102	175	86	140	106	438
	89	120	75	140	74	43	50	68	86	92	
210620_s_at	572	118	647	515	225	353	880	698	899	692	730
	341	136	160	155	167	214	178	156	74	84	
210621_s_at	579	664	1633	1276	577	790	737	763	1299	1265	556
	594	415	511	744	1189	881	830	1603	1230	2069	
210622_x_at	579	1057	439	576	150	189	623	637	569	517	274
	217	195	125	74	187	207	171	184	88	102	
210623_at	57	17	30	31	210	198	24	26	86	75	63
	19	34	8	15	16	9	61	47	28	5	
210624_s_at	917	935	811	587	686	738	991	947	895	769	742
	866	579	553	557	692	792	775	880	729	838	
210625_s_at	757	577	1210	1676	568	671	969	837	1557	1593	1064
	577	375	332	262	210	194	230	462	381	511	
210626_at	124	30	163	59	191	165	107	38	126	91	153
	98	54	10	32	5	42	47	27	41	29	
210627_s_at	574	667	874	679	764	478	623	523	625	653	679
	758	421	259	234	157	121	116	196	182	173	
210628_x_at	85	179	151	191	529	520	245	220	123	221	209
	158	113	81	104	83	71	86	102	49	65	
210629_x_at	42	23	38	49	63	44	69	115	43	55	135
	64	21	14	53	20	37	28	22	40	1	

210630_s_at	221	302	46	4	43	62	156	220	9	11	48	98	84	81	99	193	138	230	42	47	84
210631_at	12	80	95	87	14	151	12	79	90	169	97	75	70	73	38	51	31	59	165	101	243
210632_s_at	7	6	8	13	36	52	5	7	5	6	4	10	1	4	4	8	2	15	6	2	2
210633_x_at	3993	7220	3485	3017	2605	4924	3978	3013	2509	2175	4772	4406	3379	2156	1392	1735	1859	1610	1534	1375	687
210634_at	22	86	132	96	201	38	57	103	111	130	155	104	31	20	62	3	3	23	64	36	78
210635_s_at	136	92	254	225	278	320	266	307	242	356	318	356	101	107	38	48	70	64	77	78	81
210636_at	16	30	96	44	84	160	91	88	78	70	71	58	56	58	57	36	35	58	66	27	25
210637_at	37	24	68	38	73	28	16	30	29	55	27	46	5	46	24	22	24	18	32	37	3
210638_s_at	725	1264	1382	1736	1566	728	1379	1342	1664	1648	1995	1714	1697	2196	2627	2962	2685	2128	1934	2320	2234
210639_s_at	938	1140	1233	779	680	707	1024	970	1136	890	866	828	601	422	396	684	597	772	873	617	977
210640_s_at	86	36	16	67	63	235	36	42	51	50	91	178	101	114	70	104	144	114	97	126	126
210641_at	224	99	142	797	208	145	280	98	187	296	180	269	14	27	36	52	19	25	175	292	232
210642_at	28	34	33	23	38	47	61	18	19	25	27	38	2	8	6	4	3	6	4	14	2
210643_at	32	6	41	16	37	19	40	58	21	42	21	54	2	4	2	1	3	3	13	6	9
210644_s_at	267	213	342	202	404	462	317	356	230	373	313	293	32	49	44	41	33	46	27	60	62
210645_s_at	1629	1921	1814	3200	3779	4851	1454	2348	2384	3071	1961	2658	3223	3042	2512	2528	1974	1899	3507	3478	5364
210646_x_at	35024	50144	38458	53730	30973	33912	41301	37595	45070	36960	32314	48766	25848	32903	31123	28186	29957	28362	19726	30230	22836
210647_x_at	471	698	374	554	653	508	527	454	375	444	354	287	121	99	121	174	220	137	149	106	145
210648_x_at	9675	9439	7794	9229	5380	7297	5827	6537	6718	6163	6364	5360	6086	8258	11177	10334	10732	10587	8584	10596	9595
210649_s_at	1195	1552	935	771	363	359	1571	1170	739	577	1062	416	96	85	57	96	110	64	118	69	103
210650_s_at	61	44	34	19	96	237	94	66	70	57	43	177	29	22	8	20	24	3	20	20	13
210651_s_at	288	416	359	425	690	614	437	441	475	444	541	449	159	112	91	136	97	84	245	170	161
210652_s_at	335	331	156	967	404	761	416	174	108	46	192	73	98	103	138	296	371	411	238	540	532
210653_s_at	155	377	730	1406	272	649	284	271	1053	1252	500	483	405	251	283	153	206	139	1213	1071	1229
210654_at	235	7	84	58	54	39	159	198	56	64	192	92	37	11	19	48	19	2	1	16	2
210655_s_at	445	545	548	1238	36	54	453	738	348	551	605	151	23	80	80	112	68	75	89	193	146
210656_at	12	30	31	35	69	5	29	32	55	38	27	8	29	32	33	22	20	26	43	19	31

210657_s_at	34	92	8	9	522	945	180	149	21	98	361	397	274	396	221	40	59	40	7	4	25
210658_s_at	497	461	814	563	862	775	656	557	1407	1050	870	483	230	155	182	223	183	276	327	164	199
210659_at	99	106	96	54	361	286	152	125	127	151	81	163	32	9	59	8	9	18	32	19	10
210660_at	64	18	11	13	302	309	27	25	21	25	38	57	31	10	5	4	5	5	41	19	6
210661_at	78	23	77	6	44	85	57	45	64	39	20	38	20	4	10	18	14	3	13	14	18
210662_at	15	2	14	5	11	48	21	15	1	3	14	3	4	2	18	11	17	4	32	18	36
210663_s_at	26	9	121	233	41	59	21	13	40	17	20	14	29	9	35	3	28	4	79	86	137
210664_s_at	171	251	638	826	206	227	208	223	751	484	17	83	1	5	7	569	504	440	1473	1875	2308
210665_at	57	122	189	166	8	122	62	9	306	99	3	4	3	6	9	142	177	145	657	433	515
210666_at	32	12	28	16	500	306	17	22	15	21	14	31	10	11	11	12	15	12	9	7	7
210667_s_at	810	937	407	469	553	450	731	611	308	412	725	867	851	652	364	438	316	350	214	192	179
210669_at	101	90	66	84	37	43	72	113	79	102	47	68	5	6	7	38	45	6	6	6	19
210670_at	159	86	27	97	195	210	156	143	91	112	114	103	16	4	18	5	15	3	18	21	3
210671_x_at	347	415	416	326	188	210	366	432	289	499	337	240	19	44	89	139	115	97	122	120	108
210672_s_at	822	654	762	288	654	519	796	619	752	525	833	664	245	275	372	310	190	240	325	289	343
210673_x_at	34	21	35	31	96	51	24	29	17	15	68	18	28	20	41	40	70	37	13	15	5
210674_s_at	135	159	50	101	43	82	138	190	33	71	9	53	2	27	39	95	118	156	53	159	91
210675_s_at	36	27	52	94	121	184	54	18	114	59	48	30	3	35	30	45	26	17	51	42	74
210676_x_at	438	386	903	901	874	1040	861	1033	1007	1215	1155	713	378	453	688	248	195	277	384	403	568
210677_at	82	18	24	22	87	91	24	88	44	63	14	16	11	11	11	9	5	6	5	74	56
210678_s_at	175	151	376	832	1003	960	93	277	388	706	330	138	570	292	105	94	127	97	185	129	102
210679_x_at	295	316	430	450	1002	515	154	395	321	384	343	454	255	314	304	172	184	306	222	276	296
210680_s_at	119	84	151	160	52	140	53	119	173	95	134	186	44	58	41	10	54	54	34	11	56
210681_s_at	726	382	623	471	583	586	730	594	475	435	537	302	547	625	532	915	954	1085	1069	860	1315
210682_at	40	22	33	27	58	39	34	99	37	38	34	23	2	6	8	11	27	2	6	4	5
210683_at	101	11	5	3	4	7	11	55	5	4	4	9	35	9	1	120	126	92	1	3	1
210684_s_at	42	13	27	103	65	28	62	15	75	28	30	21	13	8	9	9	16	9	7	9	4

210685_s_at	496	263	243	343	437	421	321	446	231	341	350	376	236	251	153	203	185	194	245	145	189
210686_x_at	1187	2518	3087	2341	1153	1359	2166	2791	2866	3509	2393	1903	288	607	703	473	272	782	598	654	415
210687_at	235	12	126	74	190	368	20	218	91	75	98	155	65	95	58	93	75	108	132	87	98
210688_s_at	340	218	256	199	352	60	576	566	281	326	317	236	105	102	178	124	119	72	145	108	113
210689_at	164	39	129	70	115	208	72	115	12	165	128	141	37	41	80	8	64	38	61	57	35
210690_at	21	36	18	13	76	8	38	24	15	11	10	28	1	2	0	17	37	13	9	11	11
210691_s_at	1877	674	1038	453	1138	836	938	889	1108	886	826	865	3410	3560	1670	2645	2247	2334	4806	2000	1877
210692_s_at	413	161	60	103	129	95	543	384	20	87	446	304	50	12	71	74	80	118	53	87	116
210693_at	11	7	15	92	40	51	9	15	19	8	20	23	24	3	29	40	3	7	5	4	8
210694_s_at	108	223	155	237	313	339	205	182	114	193	269	400	130	102	123	44	53	79	71	88	39
210695_s_at	1062	841	593	903	401	620	722	876	611	932	759	833	187	135	204	233	207	196	215	317	240
210697_at	7	11	9	38	10	13	7	3	4	4	9	5	2	3	1	3	2	2	1	2	1
210698_at	19	10	7	21	22	67	13	11	8	42	9	92	5	6	4	29	2	3	6	7	2
210699_at	90	7	87	87	172	98	97	67	87	112	63	41	4	19	10	75	60	20	55	24	11
210701_at	61	18	27	110	52	280	30	73	162	162	51	98	32	3	79	17	7	34	76	129	48
210702_s_at	53	41	28	23	19	19	125	113	55	89	20	24	3	5	5	5	12	4	5	1	7
210703_at	41	24	33	35	74	77	40	50	27	34	37	43	2	7	19	15	8	11	8	17	6
210704_at	152	64	122	133	252	250	158	137	163	137	102	117	19	6	32	12	69	37	19	27	20
210705_s_at	389	172	312	230	247	212	121	280	225	215	97	64	75	60	72	838	785	1186	779	1088	1488
210706_s_at	517	558	351	491	737	633	489	426	391	432	814	552	475	489	564	375	380	284	175	322	336
210707_x_at	926	650	1107	1146	1347	2025	942	1438	1062	1147	2044	1695	846	547	640	388	358	385	443	412	335
210708_x_at	245	84	103	318	16	130	248	311	217	210	104	145	32	9	9	9	42	34	19	8	21
210709_at	13	36	61	17	34	67	13	73	70	105	63	70	2	3	14	3	30	14	8	2	3
210710_at	48	163	111	78	199	250	163	158	107	36	115	156	69	61	63	83	25	85	104	52	51
210711_at	30	10	30	19	22	24	16	18	11	28	9	39	3	3	1	2	3	4	8	6	26
210712_at	50	78	129	81	109	16	113	62	92	92	99	69	22	12	24	13	22	2	11	30	3
210713_at	15	12	76	116	176	253	11	62	104	120	44	41	20	33	42	29	32	39	48	36	16

210714_at	111	56	129	125	231	255	199	203	115	133	121	143	26	14	45	40	32	26	28	19	15
210715_s_at	5653	6537	8985	11918	6323	6147	3685	3637	8260	7864	5490	4720	6285	5045	4353	4133	4961	3694	5071	6526	4490
210716_s_at	441	321	614	449	630	875	359	536	458	558	564	504	260	235	93	165	176	163	211	280	363
210717_at	33	10	23	45	4	26	12	65	68	41	31	53	15	5	3	1	22	10	14	33	10
210718_s_at	188	94	179	198	300	181	168	169	241	179	146	186	65	76	78	27	40	30	56	44	56
210719_s_at	1167	2182	903	1438	536	435	1684	1932	969	1289	915	740	607	472	277	467	616	438	426	265	171
210720_s_at	810	743	472	885	799	602	814	695	460	425	641	543	280	363	353	336	340	414	269	240	290
210721_s_at	4	4	19	27	30	11	42	73	27	1	3	6	1	13	1	2	4	18	6	2	7
210722_at	86	15	22	32	70	87	19	59	21	53	31	63	21	3	6	6	3	1	6	21	4
210723_x_at	114	208	165	142	495	269	134	149	144	89	141	224	47	41	50	45	20	25	76	57	37
210724_at	24	19	99	5	111	129	58	15	9	92	65	46	2	19	3	3	2	28	5	2	16
210726_at	116	12	68	92	221	262	37	53	43	92	43	14	37	8	13	47	38	4	40	7	33
210727_at	134	78	38	32	235	387	61	26	29	20	44	13	40	21	26	1	5	8	61	11	35
210728_s_at	8	2	8	8	48	42	7	13	7	6	7	9	5	3	11	5	7	5	4	5	3
210729_at	9	6	5	19	18	40	29	22	3	1	16	38	1	3	1	2	1	1	2	7	1
210730_s_at	169	82	163	131	173	36	110	165	170	119	112	107	17	23	32	23	25	19	45	32	6
210731_s_at	187	218	179	542	469	274	123	177	163	246	236	295	138	124	178	135	114	94	169	182	196
210732_s_at	146	156	224	116	481	409	338	342	115	116	878	518	567	553	373	173	149	153	86	137	119
210733_at	972	972	411	590	634	836	928	1363	368	541	446	380	163	168	415	1151	839	779	304	526	642
210734_x_at	409	764	465	281	421	898	493	586	379	432	854	593	361	258	187	195	195	205	143	104	185
210735_s_at	315	354	823	1412	85	224	412	344	1185	2356	249	261	72	50	94	76	56	56	136	124	154
210736_x_at	332	198	168	268	78	85	266	269	174	275	188	41	34	64	104	64	85	64	67	98	6
210737_at	70	96	76	76	175	113	44	78	87	165	55	78	53	19	12	56	60	37	17	49	32
210738_s_at	25	6	7	61	40	9	4	4	47	96	1	4	1	2	1	15	8	2	6	29	15
210739_x_at	276	237	415	557	602	570	368	348	479	546	344	329	6	27	36	15	30	45	168	147	152
210740_s_at	1230	1382	1647	1798	1845	1793	1448	1188	1561	2112	1993	1563	1080	818	869	736	630	752	1406	1014	799
210741_at	40	9	4	57	93	43	40	16	60	7	34	11	4	12	6	16	1	23	26	10	25

210742_at	97	60	47	71	21	122	91	46	75	56	67	46	14	15	10	3	1	5	24	5	16
210743_s_at	101	57	80	44	80	138	38	59	68	61	80	126	16	6	21	15	3	1	20	36	15
210744_s_at	33	13	15	40	232	189	24	26	29	24	21	13	5	8	5	2	6	7	7	7	8
210745_at	86	32	24	116	49	46	62	38	144	46	24	53	10	14	27	5	29	20	17	29	17
210746_s_at	33	13	28	19	40	27	30	26	19	35	24	29	5	3	4	13	9	2	6	5	13
210747_at	20	28	9	6	80	23	19	25	23	41	14	34	1	3	3	12	6	12	6	18	3
210748_at	48	19	96	47	118	40	62	51	36	60	27	63	19	36	51	51	45	55	36	30	27
210749_x_at	4236	5131	2225	2969	2356	2156	3445	3295	1560	2812	3260	1973	1256	865	506	988	926	958	612	931	879
210750_s_at	50	15	15	181	37	67	34	26	20	81	23	29	10	12	31	2	14	7	5	20	9
210751_s_at	97	9	87	103	109	101	111	63	126	92	144	103	3	5	20	16	10	21	41	5	24
210752_s_at	226	103	221	229	345	267	211	194	246	236	199	224	120	260	139	221	258	293	344	383	417
210753_s_at	187	109	84	234	126	97	166	124	115	214	196	192	6	39	33	66	9	49	69	92	25
210754_s_at	743	606	392	256	403	370	1085	930	527	572	611	495	305	281	230	441	470	363	255	248	260
210755_at	65	52	81	61	125	155	56	62	70	75	81	35	18	3	19	8	1	3	18	7	21
210756_s_at	435	133	827	876	609	450	983	959	1017	1040	1148	963	140	170	105	120	116	144	207	119	122
210757_x_at	316	156	463	644	216	148	314	414	367	595	421	342	60	11	73	97	68	112	185	486	258
210758_at	631	377	364	136	249	277	592	595	233	253	306	290	506	589	600	2018	1250	1863	1262	713	510
210759_s_at	6138	4784	6010	4810	8150	8005	8477	6979	6050	4586	9762	9159	10054	13274	14373	11161	10916	11128	8064	7138	7460
210760_x_at	46	16	164	204	47	52	134	157	272	74	343	162	72	75	70	66	42	76	31	46	39
210761_s_at	554	562	595	957	334	406	482	727	672	829	483	322	82	48	37	88	97	92	147	92	122
210762_s_at	172	83	117	116	316	91	34	176	50	67	374	285	191	263	241	23	69	37	38	54	20
210763_x_at	152	57	94	160	81	55	52	69	110	59	78	41	5	7	10	10	7	15	6	7	6
210764_s_at	500	977	613	438	1219	1821	282	437	249	256	1381	2780	1251	802	155	758	758	1133	217	376	310
210765_at	52	21	160	85	214	42	147	162	253	106	200	158	81	66	69	22	52	69	157	52	89
210766_s_at	2286	1240	3318	1690	3974	1869	2808	2733	5225	3641	4204	4230	6113	5640	5070	2835	2270	3306	8210	4349	7017
210767_at	45	18	52	41	308	285	61	37	119	61	36	8	65	82	60	55	70	31	145	91	64
210768_x_at	868	640	931	895	1298	1109	933	821	991	1423	826	846	915	504	527	610	581	751	1569	855	976

210769_at	11	35	24	9	170	290	13	20	17	29	38	20	19	32	38	8	15	26	44	7	28
210770_s_at	65	220	14	40	22	36	73	70	37	41	77	77	10	3	7	2	2	2	5	6	12
210771_at	45	26	69	75	576	241	49	45	66	42	45	36	37	35	33	26	30	27	41	23	27
210772_at	15	33	52	53	12	23	28	7	58	21	72	28	3	12	29	23	17	29	17	17	14
210773_s_at	108	46	73	39	208	222	115	90	92	116	68	108	40	7	16	37	4	6	60	46	19
210774_s_at	2929	4014	3485	5582	3954	5116	5199	4109	3626	4542	4053	4235	4254	4201	6036	7075	6266	6097	4959	6409	7721
210775_x_at	811	680	746	786	877	1086	663	771	637	769	748	709	172	145	161	238	189	284	173	182	189
210776_x_at	1263	1662	935	441	1101	439	1202	982	793	816	759	520	667	544	1248	1589	782	792	701	1710	568
210778_s_at	135	204	210	1125	85	60	228	205	256	321	33	55	15	54	85	70	64	42	207	326	187
210779_x_at	618	499	528	473	928	825	784	592	494	546	367	375	299	342	279	430	449	530	331	205	280
210780_at	153	137	134	111	177	31	101	177	181	150	108	156	25	30	49	41	7	35	47	50	20
210781_x_at	41	85	53	63	47	52	60	58	39	41	36	50	8	10	28	15	11	18	8	9	7
210782_x_at	73	189	71	109	385	222	50	197	106	46	50	62	21	13	53	43	80	140	102	27	39
210783_x_at	11	13	33	32	47	126	61	33	39	28	24	28	78	53	94	67	72	79	56	51	48
210784_x_at	34	32	37	21	693	693	28	34	27	27	21	52	58	10	18	63	78	85	36	61	33
210785_s_at	246	258	190	164	241	481	175	304	175	144	242	325	38	50	54	85	110	63	35	30	31
210786_s_at	70	52	73	61	279	191	58	54	45	68	112	89	15	16	3	34	42	25	23	28	33
210787_s_at	106	234	140	114	205	175	160	265	330	386	205	127	51	35	25	40	42	18	32	46	43
210788_s_at	1933	4070	921	1737	554	805	1970	2204	773	799	779	894	768	895	893	2086	2155	1559	688	664	670
210789_x_at	214	66	342	78	447	370	130	169	139	271	249	214	69	22	82	42	15	20	70	61	45
210790_s_at	249	38	423	411	503	458	555	413	645	833	735	550	125	79	153	166	113	228	171	91	161
210791_s_at	370	355	564	1006	583	653	443	396	934	1128	610	372	411	419	316	89	108	63	543	515	654
210792_x_at	1849	1948	1120	444	3245	515	2588	1661	874	774	3371	2743	4074	3020	2311	972	1175	1168	1008	727	279
210793_s_at	443	344	1143	440	1640	1040	586	451	1383	974	1164	1188	1481	785	1314	703	575	725	2001	1013	1309
210794_s_at	82	60	16	21	139	73	74	87	92	38	82	148	7	52	5	5	12	4	11	12	3
210795_s_at	50	29	27	59	121	321	40	90	50	74	45	38	13	18	18	15	10	13	15	16	58
210796_x_at	288	257	332	410	419	539	351	405	381	489	355	346	75	87	101	73	87	61	79	55	55

210797_s_at	291	275	810	663	796	826	403	401	876	1150	242	312	20	71	45	73	201	1115	472	623	724
210798_x_at	13	24	9	13	27	27	36	33	29	11	17	11	7	5	39	6	6	6	3	12	5
210799_at	148	64	214	147	205	556	164	203	201	172	34	146	3	36	9	27	32	15	28	39	44
210800_at	29	5	19	30	18	110	33	70	16	35	38	35	5	31	11	2	12	21	8	9	16
210801_at	78	91	99	54	99	112	62	73	132	103	57	79	32	6	31	27	19	75	70	36	78
210802_s_at	960	946	941	330	366	383	916	521	904	742	689	645	791	893	1007	1904	1458	1761	1808	1441	1493
210803_at	74	120	156	181	220	329	123	119	124	127	135	103	46	56	69	60	39	46	86	44	33
210804_x_at	110	26	45	21	242	155	166	98	124	182	119	26	29	59	6	45	14	40	61	87	48
210805_x_at	11	5	15	13	10	9	12	18	11	10	21	14	4	2	11	2	2	3	3	2	3
210806_at	5	4	12	14	26	27	7	16	12	14	11	16	14	18	14	6	15	1	6	4	5
210807_s_at	153	84	148	54	137	79	121	107	110	110	70	98	8	18	34	42	61	21	27	37	28
210808_s_at	16	13	118	113	16	8	20	11	217	488	17	62	66	7	94	54	17	77	402	490	820
210809_s_at	83	72	98	102	175	87	85	106	87	85	70	57	7	10	27	23	23	15	13	28	9
210810_s_at	159	17	178	38	40	11	52	92	136	31	63	36	13	8	10	6	8	7	4	6	5
210811_s_at	314	294	210	155	381	314	292	210	215	150	166	83	303	249	303	297	261	236	214	232	175
210812_at	28	28	27	19	268	290	17	21	12	35	30	158	45	46	32	54	66	35	76	24	41
210813_s_at	407	207	476	142	297	208	685	574	361	505	338	255	295	173	317	427	317	466	447	202	256
210814_at	8	7	19	3	11	13	20	3	15	4	18	9	1	1	1	1	18	1	1	3	5
210815_s_at	103	16	64	31	22	36	33	25	25	22	27	16	1	4	1	5	26	10	8	2	8
210816_s_at	257	245	359	288	252	184	396	339	310	302	259	182	91	62	7	61	120	70	110	72	71
210817_s_at	1439	984	1205	1191	1063	1826	1860	2107	951	1180	1563	1371	1074	1069	1431	2187	2951	2678	904	994	1790
210818_s_at	85	82	148	127	272	251	131	82	84	130	115	182	18	14	25	22	12	3	53	32	11
210819_x_at	213	86	193	233	221	125	187	206	112	271	232	158	65	64	48	83	68	144	51	89	46
210820_x_at	765	455	414	472	465	265	462	412	451	430	321	325	331	227	344	447	407	467	550	295	267
210821_x_at	541	403	695	264	1204	411	712	569	589	980	1384	1085	1103	1058	787	254	285	280	414	182	219
210822_at	29	196	126	97	80	271	237	78	47	119	41	178	150	206	109	14	34	27	59	126	111
210823_s_at	188	389	111	113	23	17	148	272	48	24	21	30	16	11	52	47	47	68	23	9	16

210824_at	32	18	22	27	93	48	42	55	13	22	14	25	25	20	7	10	25	9	8	6	9
210825_s_at	6631	6318	4554	3881	2825	2595	4741	5660	5045	4496	3304	2570	4842	4741	5408	8673	9235	9049	8547	5124	5857
210826_x_at	1000	977	1252	1344	891	1189	1642	1578	1137	1438	1013	910	338	420	480	921	820	738	534	725	877
210827_s_at	1085	2188	882	4245	586	685	949	1015	812	869	651	305	169	198	215	351	401	410	507	599	371
210828_s_at	130	80	26	92	52	73	197	187	83	31	88	59	5	27	4	35	40	33	26	6	33
210829_s_at	111	60	73	17	63	94	118	108	64	92	53	21	43	25	2	54	51	67	69	94	96
210830_s_at	1523	1901	1633	2018	2021	1047	1871	1614	1991	2117	2636	1333	3863	4739	3786	1372	1363	1266	2154	1470	2152
210831_s_at	46	52	102	19	179	126	62	90	103	28	33	80	2	9	16	3	8	26	13	14	20
210832_x_at	103	64	27	35	107	219	81	178	195	70	74	137	20	3	2	27	7	8	38	43	41
210833_at	22	11	16	38	129	81	56	24	15	15	33	54	2	8	3	9	3	10	12	2	9
210834_s_at	13	47	15	10	23	26	99	95	25	13	13	18	1	6	3	2	7	3	29	3	26
210835_s_at	3175	4565	5595	3325	2477	2694	3747	3201	5041	4703	2494	1962	3270	1911	3061	2433	2685	2358	4625	4664	5110
210836_x_at	65	35	88	123	639	196	83	99	136	117	695	358	235	168	161	11	35	27	31	52	37
210837_s_at	45	47	50	74	664	226	40	66	91	53	497	231	325	281	230	10	16	1	27	29	35
210838_s_at	200	130	46	198	36	27	65	53	58	48	28	121	10	25	7	44	33	31	7	16	9
210839_s_at	214	84	136	111	124	259	136	234	163	103	155	146	14	22	53	15	12	32	27	24	27
210840_s_at	992	499	3127	2623	1472	1274	1455	2078	2547	4253	1734	2086	1179	738	562	578	508	712	1447	1190	1449
210841_s_at	291	264	144	215	393	378	237	140	348	246	361	266	81	69	76	15	35	56	34	34	43
210842_at	19	5	9	8	25	56	13	9	12	11	18	6	20	6	64	2	3	3	4	4	7
210843_s_at	60	100	85	35	74	103	162	59	66	92	112	18	19	31	22	18	24	15	35	28	3
210844_x_at	5407	4614	5945	6492	9616	6575	5077	5335	5349	6017	10490	8630	7944	7164	7609	4180	4542	4295	4470	4235	5477
210845_s_at	1512	2336	1466	476	1120	1259	769	856	733	2067	2136	1678	791	755	749	720	1005	1135	438	470	421
210846_x_at	202	210	140	153	359	312	203	178	202	134	244	183	99	120	113	93	89	117	146	101	46
210847_x_at	22	253	31	14	23	23	78	12	23	34	11	13	22	23	6	27	6	39	50	37	33
210848_at	36	50	113	94	47	24	171	67	52	75	101	90	16	48	34	55	24	28	30	36	21
210849_s_at	960	1027	920	1291	1087	1582	1121	1397	910	915	1170	1124	707	535	1070	1066	1034	899	746	929	1300
210850_s_at	193	127	194	97	65	27	315	170	241	324	186	85	13	1	7	2	1	15	28	2	1

210852_s_at	12	34	14	13	99	167	5	13	11	8	45	74	157	158	115	83	87	66	6	12	17
210853_at	8	33	68	78	103	141	34	45	27	35	58	54	10	15	14	24	4	4	24	27	21
210854_x_at	1379	1693	3531	1367	1020	864	2067	1874	4477	4249	1921	827	603	316	588	466	335	455	767	631	476
210855_at	126	54	18	145	444	386	60	75	88	67	102	75	4	22	22	6	3	26	8	8	15
210858_x_at	405	367	810	702	840	1265	289	393	578	1108	995	821	341	382	523	297	300	268	693	1234	1039
210859_x_at	708	564	667	1274	261	504	881	566	761	542	847	434	309	255	159	139	170	218	219	402	234
210861_s_at	77	9	83	23	65	273	74	131	48	105	136	152	54	62	44	9	2	7	2	2	1
210862_s_at	48	105	109	150	232	112	152	137	58	144	151	177	2	5	3	3	11	2	6	4	1
210864_x_at	517	355	368	537	407	316	484	383	369	379	439	255	32	19	42	52	98	28	45	53	58
210865_at	130	79	190	182	214	130	158	132	138	208	159	136	80	88	164	26	40	26	56	25	12
210866_s_at	187	186	282	182	249	47	441	313	301	373	283	328	28	43	28	36	4	74	67	67	50
210867_at	54	44	49	80	287	271	54	84	41	39	34	85	28	50	12	33	11	39	30	22	9
210868_s_at	558	467	491	85	304	142	348	410	337	204	126	62	73	77	112	943	846	614	531	393	317
210869_s_at	164	298	251	43	964	1321	288	148	360	411	1813	1533	1290	840	368	124	305	174	129	136	139
210870_s_at	269	192	159	119	131	185	156	216	124	169	142	146	75	55	61	55	40	41	53	62	33
210871_x_at	364	349	231	304	410	313	360	380	282	366	220	270	149	166	398	546	550	512	412	395	609
210872_x_at	265	215	218	162	500	378	272	248	142	187	209	269	14	27	23	41	63	78	28	21	3
210873_x_at	81	71	53	44	294	195	17	37	33	56	112	180	20	45	66	1	12	24	32	23	5
210874_s_at	81	136	34	234	32	34	195	58	96	144	207	265	7	132	144	67	88	95	34	12	70
210875_s_at	34	5	4	61	125	136	24	30	4	1	50	58	23	35	2	3	3	21	6	1	19
210876_at	147	151	244	188	855	1087	130	190	99	240	325	455	248	205	294	103	81	123	212	84	106
210878_s_at	1468	1776	1312	1997	2324	2834	2095	2299	1345	1550	1650	2262	1248	1049	1367	1477	1427	1211	1713	1255	1351
210879_s_at	438	586	472	495	764	841	435	557	587	390	563	499	238	284	305	364	427	422	238	300	183
210880_s_at	17	73	34	94	37	42	113	18	20	20	23	118	6	14	9	2	5	25	4	7	23
210881_s_at	26	10	9	19	19	13	23	11	20	15	18	10	5	6	5	2	3	4	4	2	2
210882_s_at	75	41	28	76	147	215	5	59	78	67	36	60	14	38	15	15	12	32	40	1	3
210883_x_at	261	212	252	596	353	617	292	131	518	341	280	241	81	30	82	82	57	102	97	82	82

210884_s_at	143	61	87	156	333	101	187	194	155	203	145	156	4	6	50	27	39	29	27	7	33
210885_s_at	22	24	22	28	36	31	21	29	78	24	36	21	143	121	90	20	46	70	76	77	63
210886_x_at	222	187	260	652	771	687	286	330	347	377	411	530	177	132	207	47	48	49	163	136	95
210887_s_at	25	41	30	48	80	294	123	28	32	29	28	54	11	8	15	27	15	11	43	34	2
210888_s_at	21	19	18	26	15	23	34	22	19	66	57	28	22	9	10	5	9	3	2	32	14
210889_s_at	64	9	12	38	329	239	97	25	21	22	43	18	6	24	35	31	44	25	21	16	13
210890_x_at	34	17	24	26	49	48	20	22	20	21	24	38	5	3	13	3	3	3	10	6	6
210891_s_at	4022	2452	3030	5196	5619	6151	2543	3408	2735	2608	4690	4154	7065	6439	3443	2187	2356	1765	4199	4597	4115
210892_s_at	623	508	553	691	551	1097	829	776	830	808	881	489	106	137	151	121	49	101	151	121	92
210893_at	25	4	19	16	91	20	7	36	11	22	28	45	12	4	3	27	1	1	6	4	3
210894_s_at	183	148	224	154	334	254	175	226	162	155	182	31	40	67	21	10	21	35	73	41	45
210895_s_at	94	12	45	235	34	153	23	119	161	113	63	72	4	4	2	3	1	3	36	12	17
210896_s_at	3633	2808	5650	899	1479	984	2967	2846	3855	3441	1954	1312	1087	1978	1327	3844	3857	3455	2930	1979	3181
210904_s_at	435	360	845	605	359	610	564	637	748	1212	1144	726	539	574	469	277	300	304	693	483	571
210905_x_at	12	24	12	57	26	16	23	12	11	24	14	14	2	4	27	5	3	4	6	6	6
210906_x_at	5	12	18	8	144	133	30	30	28	46	21	31	2	12	23	15	1	25	11	1	5
210907_s_at	2551	2577	5164	3837	3921	4788	3301	3263	3888	4351	4187	3518	8344	7438	7512	4444	4266	5298	8584	8893	9989
210908_s_at	2340	3879	4543	7817	3774	5387	4893	3585	3247	3441	4369	5769	7182	7568	6985	7548	7830	8158	5535	6796	6242
210909_x_at	19	19	28	21	38	23	64	65	60	85	33	19	16	6	3	6	10	6	27	2	14
210910_s_at	746	897	410	599	418	821	799	892	433	376	662	387	283	239	254	590	445	393	353	264	187
210911_at	12	5	64	47	45	94	9	18	8	4	23	24	108	31	85	28	29	51	41	7	37
210912_x_at	398	333	258	295	833	1105	559	534	317	474	628	784	229	187	204	163	164	122	61	72	66
210913_at	15	9	5	9	169	79	15	9	15	11	11	28	18	2	9	5	3	6	23	2	4
210914_at	82	51	20	23	124	30	32	63	56	18	17	109	21	7	9	22	3	4	23	12	8
210915_x_at	93	62	136	133	562	321	172	125	110	73	620	518	752	366	530	29	34	32	81	230	159
210916_s_at	1363	1055	975	1048	531	1052	1268	1327	1624	2249	823	1007	400	501	255	430	362	435	531	462	542
210917_at	98	45	57	44	279	167	60	65	55	88	63	45	1	6	11	2	4	1	32	22	10

210918_at	253	162	137	151	161	152	193	190	206	203	225	207	26	24	52	13	10	27	45	1	17
210919_at	5	4	1	65	37	35	4	4	20	1	1	3	1	2	3	4	2	2	3	1	3
210920_x_at	45	68	122	88	26	78	29	104	35	74	71	89	17	4	15	3	15	2	18	14	13
210921_at	7	9	9	9	33	15	4	9	4	3	3	8	1	1	2	5	11	3	4	3	3
210922_at	8	6	8	17	242	36	8	11	9	13	6	10	2	5	3	2	6	2	8	2	3
210923_at	310	193	263	264	454	525	317	289	424	250	243	220	64	81	35	60	42	58	31	59	64
210924_at	212	197	269	334	82	148	225	214	257	344	335	339	13	8	11	16	14	11	19	45	7
210925_at	132	198	222	158	506	380	147	157	256	98	293	202	86	56	47	13	28	65	68	47	54
210926_at	857	487	734	466	1142	1261	641	680	831	548	634	593	478	487	263	209	217	313	295	123	175
210927_x_at	7432	11040	8692	8681	5065	5291	6506	5752	9205	8176	5958	6090	8450	7485	7396	7534	7517	8873	9922	12492	9801
210928_at	60	47	52	10	16	13	46	22	29	55	38	68	14	3	38	4	24	3	6	12	3
210929_s_at	9	9	61	171	44	97	17	48	24	41	18	18	31	22	33	30	35	44	45	39	45
210930_s_at	152	146	212	393	139	165	171	194	169	285	220	104	9	2	2	14	15	13	11	24	5
210931_at	38	47	11	8	21	36	53	55	23	49	4	13	11	7	25	1	16	14	15	22	27
210932_s_at	82	45	91	65	331	171	170	124	139	141	237	204	53	49	59	66	24	37	28	29	52
210933_s_at	2023	1727	1051	358	756	878	2627	2660	1132	787	1145	420	233	288	137	406	378	558	423	245	71
210934_at	177	180	121	215	320	266	223	207	190	194	210	161	19	38	11	64	11	45	57	7	24
210935_s_at	668	821	799	210	726	832	839	946	840	1099	1046	596	312	355	214	282	274	416	343	117	260
210936_at	102	158	160	132	192	249	90	17	51	172	21	168	29	66	15	86	31	63	79	38	38
210937_s_at	29	12	65	57	150	298	34	51	71	46	65	44	43	33	34	3	6	4	25	11	4
210938_at	32	18	65	17	71	55	27	24	50	13	23	50	22	40	24	6	23	9	13	6	22
210939_s_at	140	66	95	81	238	156	127	73	54	117	26	67	5	28	37	12	30	54	32	53	29
210940_s_at	28	4	16	13	126	108	52	11	32	25	6	8	17	24	37	25	24	27	29	40	29
210941_at	8	44	26	49	18	70	30	32	28	31	34	13	15	3	11	5	8	1	17	22	7
210942_s_at	218	129	43	9	70	12	186	226	5	34	75	68	16	51	31	108	103	130	11	6	21
210943_s_at	360	201	430	632	429	520	457	484	480	478	328	447	72	87	13	43	129	51	91	101	85
210944_s_at	208	154	146	146	397	253	191	185	130	122	136	106	33	44	23	85	77	125	43	11	48

210945_at	49	7	57	53	126	93	9	21	64	25	70	19	15	36	34	9	23	20	22	2	11
210946_at	384	725	406	3491	367	325	281	471	328	437	260	364	314	320	452	934	723	583	1557	1391	1232
210947_s_at	86	39	661	503	805	345	138	140	432	647	605	410	497	571	369	111	139	173	776	538	817
210948_s_at	37	16	62	27	274	335	9	50	31	55	249	194	47	54	65	2	3	3	15	2	12
210949_s_at	10429	9349	12066	6122	8827	10942	11286	8849	12081	9690	12143	10180	8569	9914	9915	8298	9170	8815	8428	9225	8730
210950_s_at	7591	4000	10137	8423	3057	1099	4732	4455	6213	6087	3021	1419	2835	3201	4903	9155	9203	6897	5605	7249	6241
210951_x_at	378	328	467	543	416	375	441	421	650	409	682	328	347	385	488	369	314	239	654	548	481
210952_at	22	75	23	28	192	75	27	74	28	24	16	96	42	9	42	5	7	4	35	36	11
210953_at	44	50	84	47	110	168	38	36	52	57	13	69	10	3	1	3	33	4	22	37	1
210954_s_at	160	67	130	132	195	198	142	137	198	127	138	175	37	71	40	17	42	41	91	7	44
210955_at	79	144	140	263	89	294	150	137	21	232	47	26	26	30	35	45	63	31	43	31	39
210956_at	29	66	30	138	59	46	61	36	31	64	27	88	14	58	19	8	21	17	9	6	18
210957_s_at	15	9	11	16	38	245	13	13	19	13	10	15	1	4	3	3	3	3	3	4	4
210958_s_at	144	116	279	378	161	142	82	107	162	201	290	367	44	29	21	17	13	12	15	74	27
210959_s_at	554	631	491	340	559	382	515	470	591	896	853	633	573	186	372	306	193	335	412	292	420
210960_at	58	17	9	22	25	15	11	119	68	22	54	10	17	37	11	12	3	5	7	23	7
210961_s_at	26	19	130	54	184	257	24	92	50	91	99	29	22	5	33	37	22	3	21	19	9
210962_s_at	852	367	719	753	1120	1144	248	437	456	646	910	511	713	876	1171	718	598	743	938	1638	1880
210963_s_at	97	120	176	212	316	320	98	190	140	187	320	212	75	68	76	43	35	46	68	71	37
210964_s_at	41	17	144	91	564	684	28	28	88	29	352	158	129	177	113	9	3	34	22	24	25
210965_x_at	32	77	23	93	133	97	32	104	50	81	77	36	30	23	21	23	13	25	31	32	24
210966_x_at	1044	748	1649	1716	1131	1438	1271	1056	1818	1551	2012	931	497	692	1058	389	301	252	1056	1028	680
210967_x_at	314	105	178	248	184	277	237	230	381	280	165	265	30	46	3	38	24	9	17	61	6
210968_s_at	5014	4079	9689	7090	7370	4229	4456	4843	5484	6511	6913	6406	10749	13715	8917	4777	4199	3464	11834	9795	8313
210969_at	111	30	69	103	184	65	65	129	86	119	67	84	9	14	14	20	13	9	24	24	21
210970_s_at	818	689	1275	466	918	1547	466	549	476	601	451	424	841	594	618	1078	1017	1149	1531	2634	3524
210971_s_at	359	219	155	234	165	199	285	219	151	136	404	354	193	160	202	224	248	240	55	71	124

210972_x_at	202	85	167	221	285	79	54	214	170	179	267	225	19	6	87	32	6	31	34	41	5
210973_s_at	681	563	161	237	422	563	280	536	189	211	748	495	90	68	110	90	78	121	122	70	58
210974_s_at	415	404	370	253	67	159	441	392	432	407	399	204	200	121	70	20	52	66	50	37	34
210975_x_at	783	1128	971	1351	434	476	749	764	945	771	796	382	504	307	322	110	260	174	328	313	230
210976_s_at	3982	2632	2701	2270	2290	3587	3669	3216	2922	2689	2177	1815	1561	1972	2269	3027	3588	3006	2402	1712	1881
210977_s_at	36	58	42	75	100	77	57	30	19	25	26	29	5	13	20	12	19	11	24	19	9
210978_s_at	2163	910	5045	4195	2504	1583	2800	1928	6102	7298	6057	2893	2269	1804	1012	457	649	622	2277	940	913
210979_at	15	33	18	12	27	13	52	8	20	24	58	16	2	9	5	12	6	5	11	4	17
210980_s_at	1670	2178	924	2115	1370	4537	1030	1330	656	709	873	1463	1162	1131	1717	2429	2150	1563	1550	2800	2975
210981_s_at	582	546	368	372	959	642	159	381	446	444	831	510	511	844	1018	274	285	275	407	179	154
210982_s_at	17	12	16	9	15	17	16	17	15	14	14	13	3	4	3	4	5	4	6	2	3
210983_s_at	3307	1189	3066	826	5237	587	3656	3800	3923	3234	6944	4301	7042	7627	5645	2476	2446	3308	4771	1590	1819
210984_x_at	30	79	79	40	47	48	37	140	92	56	416	439	7	7	74	9	15	5	5	6	4
210985_s_at	53	33	72	141	19	105	29	71	83	42	36	55	27	28	39	46	42	92	103	363	467
210986_s_at	1109	1443	3993	3235	1153	2119	836	668	2229	2580	1278	1400	1168	567	411	510	557	598	5158	5555	6018
210987_x_at	1176	1412	3155	3060	1161	1751	1418	1086	3389	3581	1782	1266	907	556	978	692	667	517	2640	2483	2452
210988_s_at	369	408	662	945	375	379	786	796	618	741	422	481	97	41	76	181	187	181	234	126	203
210989_at	67	77	18	18	132	106	37	88	60	66	51	35	26	36	4	20	3	19	26	7	26
210990_s_at	37	41	27	25	73	87	30	34	29	31	26	33	12	7	9	6	6	9	8	11	12
210991_s_at	165	146	119	156	243	195	286	366	253	236	223	255	47	45	46	26	12	52	40	35	7
210992_x_at	32	15	27	31	21	24	28	12	36	25	9	28	10	8	17	12	6	6	12	4	9
210993_s_at	532	815	431	510	639	485	835	801	328	282	610	483	469	398	500	553	405	367	244	290	337
210994_x_at	53	19	45	28	87	79	188	356	31	92	48	70	47	40	50	171	76	123	34	71	103
210995_s_at	239	114	267	211	271	332	337	395	182	211	291	313	53	54	50	133	125	104	57	86	116
210996_s_at	7763	4214	6282	5243	3871	3232	6583	7350	6595	6158	6668	5123	6163	8644	10032	8993	7756	7987	10718	7455	8578
210997_at	5	6	9	9	80	15	28	9	5	36	4	6	1	2	3	4	1	1	7	4	1
210998_s_at	9	7	5	6	29	5	8	7	7	10	7	10	2	6	3	2	3	2	1	12	1

210999_s_at	407	360	757	182	427	812	425	205	518	538	379	329	180	79	285	269	231	229	287	388	332
211000_s_at	151	51	168	175	361	290	150	176	203	264	323	251	80	102	36	36	25	34	46	51	58
211001_at	144	203	121	129	686	719	176	179	155	148	102	102	7	61	68	40	56	16	46	27	7
211002_s_at	17	19	56	144	333	544	44	24	50	275	1363	615	570	521	382	12	19	7	135	131	116
211003_x_at	25	16	12	17	1608	216	19	11	9	10	5347	2687	428	294	250	23	49	7	6	11	7
211004_s_at	316	583	351	1498	1733	2602	337	354	368	464	416	401	206	255	145	126	103	106	177	180	125
211005_at	136	71	372	200	121	112	148	139	351	303	443	256	329	306	393	43	8	4	241	322	234
211006_s_at	74	95	100	48	206	300	94	145	83	96	111	79	24	18	27	43	33	59	34	39	42
211008_s_at	50	179	159	53	195	75	50	73	170	136	124	114	22	56	56	4	11	16	10	12	11
211009_s_at	226	156	217	285	367	422	248	257	256	162	192	192	249	173	178	244	236	255	275	365	407
211010_s_at	36	35	22	13	59	36	40	21	37	24	99	20	21	39	7	3	4	15	41	7	17
211011_at	124	64	24	89	22	28	65	34	58	71	33	85	5	12	16	4	7	16	4	3	17
211012_s_at	151	336	73	103	71	81	293	82	56	67	428	43	124	139	218	184	211	385	170	191	176
211013_x_at	15	86	34	38	19	17	13	36	12	24	399	43	66	95	154	60	99	106	100	201	161
211014_s_at	336	290	407	431	268	278	465	412	433	342	483	366	54	64	105	111	15	169	113	134	59
211015_s_at	2547	1455	1644	918	3139	1773	2445	2150	2985	1719	2646	1876	3719	3298	2544	1846	1654	1438	2379	1271	1080
211016_x_at	1322	875	1031	736	1537	1266	2276	1704	2444	1659	2682	1278	190	202	222	180	191	176	177	119	119
211017_s_at	69	97	141	79	33	124	167	140	209	122	206	147	37	25	7	4	16	55	41	24	19
211018_at	160	129	46	84	568	460	86	67	63	88	99	11	54	50	60	123	119	57	72	84	64
211019_s_at	372	209	353	246	327	396	277	176	325	324	483	276	32	45	58	82	60	70	11	41	26
211020_at	53	26	11	106	60	73	5	82	122	15	44	57	4	13	43	5	30	19	4	11	35
211021_s_at	9	6	8	26	126	79	13	20	27	27	57	9	14	4	18	6	3	2	4	9	3
211022_s_at	26	43	37	62	12	206	57	74	45	89	111	118	27	1	21	3	12	1	15	17	26
211023_at	3108	3411	3540	4611	3761	3976	2930	2949	3885	4518	4058	3854	5275	6592	7600	5304	6309	6228	6746	6949	6909
211024_s_at	107	88	20	18	63	59	13	107	19	57	16	19	12	4	3	37	38	8	4	17	7
211025_x_at	4142	6039	3154	6325	2929	4545	3295	3178	2725	3287	3526	6265	6987	6424	7544	5469	6588	5270	5890	7113	3770
211026_s_at	472	937	2111	2680	579	798	529	406	2510	2176	554	643	186	187	194	79	113	97	967	871	551

211027_s_at	89	129	62	70	109	214	144	154	118	64	61	93	7	4	5	26	32	13	40	5	3
211028_s_at	90	120	121	193	856	454	73	92	277	187	317	298	424	233	198	97	83	91	214	118	108
211029_x_at	67	78	23	17	113	185	101	121	20	14	159	156	349	358	317	96	77	48	44	190	112
211030_s_at	9	5	8	6	18	17	8	9	8	10	14	5	8	4	6	5	5	5	8	2	2
211031_s_at	413	915	76	313	484	977	313	347	130	198	686	447	508	539	547	395	464	377	36	239	156
211032_at	3	2	1	25	11	13	12	20	3	8	1	3	7	3	1	16	6	1	27	49	34
211033_s_at	458	719	566	801	388	478	500	523	692	801	411	385	316	198	254	299	307	303	535	385	411
211034_s_at	441	712	378	720	334	597	400	475	399	481	279	381	106	88	125	168	220	223	254	427	509
211036_x_at	2864	2997	3148	2834	1882	2322	3305	2755	3796	4082	2413	2683	2496	3025	3247	3312	3403	3193	4600	3761	4478
211037_s_at	164	124	178	238	115	130	179	158	221	320	173	129	68	30	56	61	49	45	114	59	54
211038_s_at	553	798	297	310	456	406	269	575	285	447	414	441	397	217	341	446	461	469	507	567	575
211039_at	85	18	49	30	232	198	20	26	56	87	41	57	7	25	33	4	12	16	27	11	3
211040_x_at	893	573	1190	733	2592	1106	867	1499	1290	1519	1269	1622	917	1329	1010	551	534	912	1042	679	733
211042_x_at	909	629	1449	1190	911	589	1252	1073	1668	843	820	797	885	1612	1331	1000	923	1059	2110	1162	1683
211043_s_at	1040	1027	1522	834	1992	2666	1041	492	1013	904	3118	1934	2815	1602	1859	645	626	644	811	867	470
211044_at	107	16	38	171	121	230	46	95	102	131	151	194	14	10	19	42	2	34	23	34	26
211045_s_at	40	7	20	13	49	36	13	11	52	11	13	19	5	7	3	6	8	12	4	4	5
211046_at	5	5	12	9	126	255	7	3	7	13	6	6	2	22	1	4	1	2	3	2	19
211047_x_at	8656	7012	7524	7727	4129	2981	9518	6263	8425	7810	8679	4449	6953	9039	6941	8103	8441	9675	7161	4567	2579
211048_s_at	6018	4898	6879	3810	2777	3841	3183	2623	3634	4429	2217	2343	2575	2485	2643	7174	5579	6872	3808	7590	8776
211049_at	17	17	8	8	261	78	19	62	19	18	9	28	35	5	3	3	3	6	21	3	2
211050_x_at	25	32	24	49	23	26	99	143	44	99	121	46	10	8	42	7	39	9	22	6	61
211051_s_at	267	195	302	204	318	363	509	620	356	377	291	195	32	42	10	67	62	65	15	47	17
211052_s_at	1032	726	545	338	1063	305	1015	892	645	629	665	363	561	616	275	460	581	559	470	251	291
211053_at	16	7	11	16	18	19	9	9	12	18	7	23	2	3	3	5	2	9	4	2	2
211054_at	1	1	5	3	33	4	5	13	23	6	3	13	1	7	18	17	28	16	13	7	1
211055_s_at	28	60	52	10	78	141	9	40	15	48	18	39	38	39	29	15	26	29	72	26	40

211056_s_at 493 1230 378 696 162 140 530 556 767 1353 541
770 527 255 815 745 355 781 634 436 801
211057_at 13 129 183 111 51 67 93 18 104 221 70
35 38 30 31 18 13 22 146 82 124
211058_x_at 27832 16966 25617 12629 30134 23286 31220 28202 35615 25960
25842 33092 20569 27658 23747 16965 19649 23933 16443 16588 12871
211059_s_at 262 230 324 138 78 595 199 284 226 236 321
221 207 230 22 89 166 96 160 66 119
211060_x_at 1802 1195 861 1103 1306 1453 1423 1256 1040 693 1199
1348 679 901 886 831 961 762 674 440 324
211061_s_at 1046 712 1038 886 929 684 901 827 920 1031 1199
982 753 588 665 627 663 659 704 641 737
211062_s_at 26 19 15 38 41 24 24 32 17 15 13
14 6 6 7 8 7 7 8 10 4
211063_s_at 697 751 960 854 676 1015 521 604 648 753 719
970 998 1201 1377 1473 1235 1406 1770 1712 2255
211064_at 3 27 7 13 71 46 3 11 1 35 37
58 51 35 23 22 2 22 42 15 40
211065_x_at 1279 1332 841 808 561 521 1077 1490 942 1227 974
851 315 283 249 307 308 289 386 175 284
211066_x_at 556 665 319 322 510 898 501 546 274 356 1099
741 290 375 343 273 230 192 94 65 101
211067_s_at 126 82 24 96 84 31 174 177 11 24 63
40 38 22 77 103 86 94 35 27 3
211068_x_at 1252 1143 1173 2020 1083 1568 1079 1221 1054 1041 1003
768 1123 1169 1717 1039 1024 973 1330 1783 2004
211069_s_at 3475 2292 3448 3625 3458 3678 3254 3311 3388 4421 4554
4959 6615 8439 6812 8622 7370 9488 8710 7041 6257
211070_x_at 6072 5895 8854 14119 6820 3745 5003 4163 8948 8295 5706
4423 7548 8201 9034 7147 8895 5771 7376 7322 4050
211071_s_at 194 238 998 582 1188 1122 203 256 665 415 2223
758 1011 1762 1475 110 84 139 981 1134 1374
211072_x_at 26440 13938 26695 8860 26899 21359 28274 26584 31551 26615
28114 34397 21631 27224 20430 14899 15823 21562 13898 13549 11593
211073_x_at 32007 41050 26566 21346 20487 31193 31109 30521 26286 24882
21214 27512 16583 19985 23731 28843 29939 26277 14141 22218 15713
211074_at 1135 1428 1158 1600 2640 3216 1770 2604 2139 2270 1988
1785 916 887 407 641 389 366 390 961 573
211075_s_at 1041 1892 3373 3907 1170 691 1859 1760 3520 3613 1904
1880 1278 1566 1336 854 809 1033 2664 2329 2112
211076_x_at 183 102 178 114 245 329 134 172 197 171 141
134 36 4 2 5 5 56 24 38 25
211077_s_at 135 125 142 191 492 392 310 354 157 263 234
158 134 82 145 149 72 111 224 129 172
211078_s_at 73 62 57 26 43 121 27 71 13 11 20
34 30 13 12 33 12 30 38 25 37
211079_s_at 198 129 91 141 209 116 89 108 106 64 136
124 42 29 7 6 24 6 41 8 4
211080_s_at 605 108 393 107 444 278 509 635 586 690 566
657 345 327 159 242 202 332 486 122 266
211081_s_at 106 122 94 38 84 124 160 150 80 171 205
89 51 46 34 35 28 31 27 22 24
211082_x_at 122 170 156 282 58 70 244 199 289 280 230
34 11 84 52 39 34 8 48 53 43

211083_s_at	7	7	11	13	12	23	7	7	4	8	4	4	3	6	7	8	16	4	6	11	1
211084_x_at	136	130	586	605	903	1740	321	344	872	758	1170	980	388	435	412	28	10	50	276	126	223
211085_s_at	65	17	73	75	74	73	73	75	99	106	124	112	196	220	64	30	15	22	41	32	35
211086_x_at	19	5	7	10	128	15	61	65	13	57	10	13	27	27	12	24	2	31	4	4	2
211087_x_at	29	137	19	16	33	42	34	110	21	29	34	46	8	22	9	5	2	6	1	6	5
211088_s_at	253	7	115	10	132	20	204	253	151	177	192	196	59	30	22	25	24	32	21	2	6
211089_s_at	173	82	225	145	371	386	252	256	258	277	588	418	150	113	86	43	32	70	74	44	94
211090_s_at	1131	410	1211	837	929	632	1132	1368	1148	1307	1100	956	373	330	273	544	467	527	503	370	480
211091_s_at	157	156	142	141	220	314	221	100	195	119	202	59	57	46	28	62	94	74	86	46	47
211092_s_at	122	120	165	31	71	288	143	183	226	243	173	201	34	7	14	22	16	26	33	44	39
211093_at	7	5	22	3	10	9	33	3	4	7	3	13	1	3	1	1	3	4	1	1	1
211094_s_at	42	47	244	297	294	235	101	99	253	334	182	191	45	29	51	62	55	7	144	80	147
211095_at	74	40	12	63	81	172	49	66	64	87	91	72	16	24	13	26	44	4	39	36	40
211096_at	180	145	203	180	323	302	242	260	195	229	173	106	69	90	120	76	160	118	40	66	82
211097_s_at	470	302	270	350	456	572	319	310	222	266	250	225	48	64	19	43	81	56	44	79	36
211098_x_at	480	342	633	722	540	989	992	870	788	946	713	777	1353	804	581	650	593	596	900	706	845
211099_s_at	8	6	12	6	14	11	12	46	7	14	60	70	2	2	2	2	2	3	8	6	1
211100_x_at	183	38	172	177	146	82	146	232	265	169	183	177	7	31	29	5	21	65	46	60	41
211101_x_at	181	29	46	49	74	69	113	145	52	77	40	158	25	19	46	13	25	5	22	27	10
211102_s_at	15	13	7	81	15	13	12	41	50	82	6	16	10	11	5	19	25	9	7	13	6
211103_at	56	18	9	13	173	20	15	12	24	22	10	25	14	15	18	7	3	2	32	28	14
211104_s_at	64	5	11	12	96	35	17	9	9	17	11	13	16	5	7	4	3	7	5	8	5
211105_s_at	373	517	180	248	356	266	616	655	324	222	324	187	71	88	59	578	588	394	77	46	53
211106_at	97	89	167	136	73	208	117	146	98	172	148	133	50	59	66	56	39	39	55	96	50
211107_s_at	126	172	198	158	139	335	188	230	225	212	115	293	7	3	37	17	12	9	41	6	12
211108_s_at	41	105	16	27	67	9	21	29	8	28	14	13	6	6	4	5	5	3	18	3	2
211109_at	33	73	53	12	38	46	48	41	48	52	17	93	4	8	6	5	2	3	13	3	7

211110_s_at	132	78	125	47	51	300	99	73	80	147	65
	162	20	38	27	6	19	54	68	35	22	
211111_at	16	47	9	18	62	331	17	83	52	20	7
	50	14	18	7	9	7	3	11	9	8	
211112_at	97	142	66	110	122	149	152	74	103	64	260
	31	86	68	114	92	20	103	106	87	26	
211113_s_at	86	86	151	253	396	710	113	128	143	295	182
	57	28	70	64	47	42	47	82	82	86	
211114_x_at	700	410	701	453	691	797	1096	784	546	741	470
	390	309	440	388	605	601	644	400	296	295	
211115_x_at	946	636	931	617	1001	970	1393	1128	830	1010	703
	770	441	560	451	921	824	1129	803	537	583	
211116_at	21	9	11	72	334	65	97	16	33	80	104
	62	8	6	29	19	3	8	5	5	2	
211117_x_at	98	19	121	40	16	160	37	75	27	87	77
	139	14	2	26	3	4	9	12	35	3	
211118_x_at	42	22	27	14	73	243	29	36	60	28	23
	122	8	51	11	6	6	9	28	9	48	
211119_at	5	4	3	12	8	8	5	3	4	6	1
	3	1	8	1	3	2	4	1	4	1	
211120_x_at	30	15	20	22	69	238	19	26	20	25	27
	53	25	12	42	6	7	5	4	14	37	
211121_s_at	505	292	235	370	811	960	307	439	395	257	640
	643	338	216	40	53	50	41	67	52	45	
211122_s_at	57	34	71	69	71	28	32	45	25	52	13
	8	1	3	13	5	21	55	48	39	76	
211123_at	250	269	349	508	478	529	333	306	310	333	244
	163	69	60	69	77	82	49	88	92	42	
211124_s_at	1420	219	461	155	8	13	1732	2535	957	636	10
	6	1	1	1	177	155	425	113	27	74	
211125_x_at	147	238	60	43	253	231	258	185	63	36	71
	77	3	4	38	5	4	25	45	13	35	
211126_s_at	1065	865	87	28	1219	789	1379	1196	142	64	503
	832	880	660	823	699	718	605	92	79	80	
211127_x_at	32	15	90	66	124	112	78	104	173	157	55
	73	35	30	20	38	28	32	50	22	34	
211128_at	11	6	22	28	47	54	23	36	66	11	20
	13	14	2	4	2	29	5	1	7	8	
211129_x_at	33	26	114	87	161	246	111	11	76	70	84
	77	13	23	20	39	42	26	25	26	25	
211130_x_at	36	101	115	118	344	357	83	128	162	196	105
	151	43	45	26	50	35	59	74	39	25	
211131_s_at	49	6	19	96	18	34	5	26	48	20	17
	36	32	2	3	13	8	9	29	6	6	
211132_at	17	21	34	39	115	15	17	34	16	27	16
	15	5	7	17	6	7	26	9	4	3	
211133_x_at	101	86	186	154	1003	973	164	177	201	165	111
	59	21	34	58	77	85	47	64	77	54	
211135_x_at	53	57	43	50	507	714	34	34	21	27	24
	41	14	29	70	30	69	39	48	50	82	
211136_s_at	1480	1629	2252	2462	1045	723	2215	2069	2546	2261	2889
	691	452	649	581	860	1033	958	1126	389	487	
211137_s_at	478	528	449	405	488	552	473	592	563	730	432
	527	520	427	544	850	647	752	1367	1035	1576	

211138_s_at	110	184	146	158	129	22	182	117	173	63	145	171	31	13	21	20	29	15	20	10	1
211139_s_at	542	409	1177	885	745	572	674	676	1064	1371	968	655	202	272	271	139	140	130	477	268	377
211140_s_at	81	29	18	32	19	32	481	380	71	55	71	78	38	27	13	64	6	78	14	5	33
211141_s_at	179	210	368	176	126	161	184	208	171	210	250	136	50	22	49	50	28	77	83	54	48
211142_x_at	30	15	79	36	104	103	19	17	24	22	27	19	22	9	14	10	32	22	22	4	5
211143_x_at	60	86	62	66	117	60	66	74	64	48	256	150	122	78	131	123	98	90	96	101	74
211144_x_at	38	26	41	41	161	47	33	38	43	39	37	113	17	16	27	15	30	42	26	27	28
211145_x_at	97	16	14	19	34	31	25	108	48	96	43	145	3	3	3	17	2	3	1	4	1
211146_at	13	15	12	12	15	22	11	12	4	14	10	9	2	3	4	3	3	9	3	2	3
211147_s_at	25	21	15	19	18	17	32	29	20	31	18	35	6	6	14	3	3	3	5	2	3
211148_s_at	54	9	4	5	71	214	8	11	9	14	36	18	14	4	28	35	30	66	26	4	3
211149_at	32	46	22	91	7	1	44	4	5	7	6	5	13	8	19	15	17	2	6	16	12
211150_s_at	994	595	954	374	961	824	1317	1126	1263	942	1333	890	597	596	704	848	677	829	901	535	783
211151_x_at	32	9	11	23	45	50	32	28	12	28	26	24	3	38	22	5	7	39	1	5	4
211152_s_at	564	420	442	297	969	1256	456	441	379	354	716	523	736	756	548	309	343	381	264	307	181
211153_s_at	26	19	31	31	52	24	48	25	54	31	45	23	2	2	9	47	6	9	4	9	7
211154_at	56	39	9	57	265	161	76	46	33	39	91	64	8	20	37	5	2	30	3	16	2
211155_s_at	5	6	26	10	89	93	24	65	51	25	53	45	12	8	2	4	25	2	17	31	14
211156_at	95	12	84	131	18	13	44	69	71	29	95	19	15	6	13	5	8	3	3	4	3
211157_at	180	168	164	215	139	62	269	285	281	300	254	253	9	18	21	80	9	64	35	59	7
211159_s_at	865	299	695	534	1416	1087	701	829	458	648	630	400	198	177	99	227	178	147	191	194	144
211160_x_at	1155	1406	2050	921	5736	1364	1300	1079	722	1590	10093	9633	5084	2213	2212	891	862	1014	636	485	812
211161_s_at	48	18	27	45	76	122	13	29	40	24	6	46	0	1	11	18	30	21	8	9	7
211162_x_at	3176	999	1526	653	205	480	1020	1098	705	1106	709	75	22	16	98	452	365	383	81	132	136
211163_s_at	17	55	7	14	29	44	87	113	15	28	17	72	3	5	5	48	82	41	10	22	2
211164_at	48	45	30	40	23	113	70	50	70	49	21	30	12	11	15	15	25	13	20	9	1
211165_x_at	20	35	38	21	23	40	52	34	17	22	20	41	26	15	11	69	55	56	90	99	137

211166_at	319	276	153	224	217	36	197	228	313	214	124	146	43	51	61	17	54	68	90	101	74
211167_s_at	11	16	20	13	52	32	20	25	24	119	24	31	6	9	11	8	4	3	4	19	6
211168_s_at	1169	878	628	462	664	429	1077	992	751	811	998	799	440	322	286	315	359	274	313	232	191
211169_s_at	7	40	53	4	19	67	4	4	12	3	33	40	1	11	1	1	4	1	0	3	9
211170_s_at	75	6	113	92	84	99	60	61	102	131	36	74	11	22	32	16	12	35	57	39	61
211171_s_at	115	154	339	204	289	285	109	191	217	298	145	67	30	17	25	31	41	17	61	75	65
211172_x_at	101	141	167	169	122	294	192	129	166	123	104	73	77	9	75	52	89	63	25	62	50
211173_at	91	26	38	87	66	32	37	33	29	29	20	46	12	14	49	36	106	12	48	25	30
211174_s_at	144	30	33	128	88	262	29	113	40	60	92	33	53	49	27	25	6	20	10	7	8
211175_at	28	22	37	14	265	177	9	16	17	20	17	45	6	11	54	8	36	10	5	20	11
211176_s_at	132	26	119	91	48	81	28	65	66	39	37	50	27	13	14	5	7	12	31	7	5
211177_s_at	52	262	743	547	775	1216	390	400	872	728	577	324	382	450	180	153	234	208	318	179	114
211178_s_at	37	117	136	144	32	191	57	121	122	95	71	103	39	29	44	22	30	57	22	19	2
211179_at	9	2	1	3	8	71	5	5	1	4	4	8	2	14	2	2	3	1	1	1	2
211180_x_at	142	129	100	31	348	216	97	90	103	110	298	303	124	4	95	83	46	77	76	54	58
211181_x_at	29	54	38	163	383	101	113	190	82	66	377	142	44	54	48	25	47	31	8	9	42
211182_x_at	22	21	26	38	88	85	103	20	35	24	112	45	31	33	66	57	20	7	52	14	43
211184_s_at	200	279	423	1870	745	585	229	260	933	1386	458	369	124	130	379	117	105	48	652	568	511
211185_s_at	6175	4068	4343	5023	6573	5405	5647	5631	6026	4832	5196	4539	6596	6054	6401	7448	6691	6515	8524	6378	8633
211186_s_at	13	12	15	14	22	50	13	9	8	13	9	16	4	5	41	3	3	4	4	2	6
211187_at	3	1	7	3	5	8	3	3	1	3	1	3	1	4	2	13	3	1	1	2	1
211188_at	82	78	23	47	113	121	61	54	23	88	104	84	14	32	36	5	30	18	36	32	17
211189_x_at	188	154	315	188	253	168	217	259	194	273	195	240	31	55	94	38	90	35	43	11	31
211190_x_at	159	129	180	208	43	27	178	193	171	183	136	187	27	50	46	10	16	7	46	57	27
211191_at	5	4	5	5	11	27	5	5	8	6	10	8	1	3	3	4	3	9	3	2	4
211192_s_at	108	52	79	10	19	22	117	82	91	82	63	77	2	4	15	2	1	1	3	1	13
211193_at	153	118	155	71	168	351	125	124	159	148	126	121	15	38	34	28	24	42	55	22	22

211194_s_at	30	15	28	84	183	146	129	145	147	87	33	59	10	11	7	11	10	41	13	3	19
211195_s_at	161	101	167	146	285	17	176	273	245	214	139	153	2	7	48	7	28	11	28	21	25
211196_at	106	52	92	101	58	120	37	86	78	141	78	63	14	3	2	13	14	31	4	1	22
211197_s_at	42	77	57	32	47	109	27	113	186	119	16	28	71	69	73	87	64	61	94	51	40
211198_s_at	26	9	28	22	44	50	16	16	19	11	11	14	8	6	44	40	7	7	32	27	5
211199_s_at	19	7	11	19	34	39	20	24	90	68	20	13	7	6	5	2	6	3	5	2	7
211200_s_at	66	58	11	9	73	179	9	51	74	57	44	104	89	218	112	214	225	205	230	144	317
211201_at	32	9	15	19	51	54	61	22	15	22	14	16	4	9	7	10	3	28	6	4	1
211202_s_at	575	657	1027	1971	274	860	657	812	816	998	510	466	211	262	347	670	672	770	793	930	1249
211203_s_at	275	218	133	166	131	109	269	208	44	194	112	194	31	29	45	59	65	90	10	62	19
211204_at	24	21	34	111	260	130	109	90	70	32	114	31	6	41	4	33	7	4	7	9	2
211205_x_at	283	101	650	246	535	648	600	579	451	611	665	424	32	66	66	128	55	200	83	61	72
211207_s_at	93	78	41	74	98	155	53	108	70	52	85	75	18	59	77	35	1	33	5	15	1
211208_s_at	984	1152	808	776	1336	1132	1190	1131	740	1177	902	995	679	744	715	973	980	924	997	673	1077
211209_x_at	69	34	68	58	106	145	16	33	45	38	23	107	25	3	23	8	15	37	36	36	29
211210_x_at	153	23	179	133	726	528	122	174	173	205	77	225	54	47	10	5	21	18	71	61	72
211211_x_at	110	24	33	87	96	452	52	139	202	172	117	172	54	6	5	9	5	20	53	11	4
211212_s_at	307	102	472	146	511	328	453	377	242	240	546	508	822	510	231	249	143	210	343	216	371
211213_at	4	22	26	17	4	9	4	3	7	14	33	29	68	21	47	17	13	54	29	14	39
211214_s_at	110	129	65	327	135	46	54	220	111	291	61	103	5	5	11	5	7	24	43	16	9
211215_x_at	155	61	50	54	84	30	171	102	282	75	155	141	14	42	15	4	8	10	6	33	20
211217_s_at	57	57	49	305	146	163	115	53	52	277	21	40	34	10	10	16	8	9	8	10	6
211218_at	12	5	28	48	198	106	42	49	94	89	82	34	3	15	25	3	30	16	11	14	3
211219_s_at	188	45	22	17	47	23	260	174	92	147	87	21	66	3	40	111	103	75	3	8	6
211220_s_at	138	73	148	129	117	81	148	214	166	96	124	116	84	28	82	124	116	125	130	79	169
211221_at	15	7	18	36	55	28	16	28	20	18	13	16	7	30	15	6	18	4	17	7	5
211222_s_at	140	165	160	185	102	97	208	238	16	166	266	266	57	87	92	80	47	40	38	52	55

211223_at	26	82	90	133	242	124	143	100	103	148	114
	143	2	15	12	40	8	30	4	16	3	
211224_s_at	134	137	95	72	37	39	163	48	62	113	122
	143	4	8	15	5	3	5	4	7	18	
211225_at	12	27	9	18	390	488	17	25	11	11	10
	15	5	17	6	13	15	15	48	14	9	
211226_at	56	7	12	53	69	48	45	11	19	102	63
	67	76	92	92	40	58	27	6	42	5	
211227_s_at	40	50	54	57	155	97	72	42	4	43	43
	31	8	2	9	13	10	2	17	1	14	
211228_s_at	534	687	770	636	407	548	1105	1016	575	890	422
	637	274	311	400	636	754	660	336	586	791	
211230_s_at	241	241	269	193	98	44	261	193	206	168	217
	232	50	49	53	114	69	96	41	21	28	
211231_x_at	366	223	301	331	37	81	366	144	296	214	256
	285	4	39	55	19	8	22	16	32	13	
211232_x_at	40	26	52	32	85	66	33	20	43	41	16
	10	7	5	3	8	20	4	8	11	6	
211233_x_at	193	33	46	162	142	120	32	44	44	49	78
	29	13	28	15	9	53	51	16	6	51	
211234_x_at	20	40	22	36	126	85	34	33	20	31	61
	35	5	11	15	8	11	6	16	17	13	
211235_s_at	48	19	22	79	95	99	29	107	29	29	50
	26	11	16	8	12	88	61	32	7	13	
211237_s_at	251	232	76	38	60	31	205	117	318	762	17
	29	17	5	6	56	37	16	132	55	88	
211238_at	48	4	75	22	23	164	9	5	94	7	4
	24	22	4	25	34	14	4	31	21	21	
211239_s_at	8	29	38	85	77	9	21	74	50	41	55
	24	13	3	19	9	1	13	11	17	1	
211240_x_at	1081	884	3344	3527	1234	1168	1634	1655	2411	3024	1651
	1183	372	318	375	364	317	399	1262	925	725	
211241_at	491	176	408	371	935	505	490	740	313	735	630
	809	149	332	207	78	203	176	132	131	89	
211242_x_at	26	32	144	164	32	48	115	80	5	112	26
	64	5	5	30	5	19	3	10	4	21	
211245_x_at	74	90	132	81	37	30	40	22	151	200	109
	18	30	33	3	3	23	9	26	15	11	
211248_s_at	130	150	125	150	36	77	109	211	76	126	115
	94	51	47	20	9	48	47	32	49	30	
211249_at	165	58	41	109	397	524	102	32	67	94	109
	97	42	26	29	2	8	41	18	8	17	
211250_s_at	210	227	140	193	221	507	146	143	112	63	175
	122	113	61	107	83	88	96	64	52	60	
211251_x_at	923	954	960	958	1135	773	855	888	879	862	789
	714	534	615	537	472	531	667	576	436	405	
211252_x_at	517	449	330	641	881	918	500	529	811	229	574
	466	93	208	162	168	167	192	177	167	136	
211253_x_at	83	88	18	38	197	185	70	87	112	35	58
	78	48	9	4	2	7	32	5	30	7	
211254_x_at	597	310	427	414	770	642	456	540	494	471	382
	457	34	32	69	71	56	45	138	63	72	
211255_x_at	401	192	351	498	44	192	497	457	412	458	358
	367	118	112	89	119	93	147	187	78	146	

211256_x_at	487	46	292	321	455	398	333	69	285	245	286	271	166	137	112	58	25	104	28	78	126
211257_x_at	1118	1067	1450	2431	1674	1258	1291	1502	1207	1528	1482	1360	946	970	1028	991	1051	836	1279	1040	1170
211258_s_at	382	254	627	588	114	194	497	346	725	724	510	567	32	44	66	80	7	51	79	39	98
211259_s_at	74	44	49	155	634	211	98	133	40	131	1182	266	536	402	434	74	90	68	36	48	40
211260_at	303	120	321	250	327	384	170	317	154	354	354	358	66	323	135	79	136	122	163	46	45
211261_at	130	136	229	178	1068	896	213	178	159	80	213	197	62	43	56	29	44	37	142	41	76
211262_at	287	175	293	297	209	293	269	263	268	310	242	260	8	34	33	32	42	44	86	52	12
211263_s_at	90	49	85	78	253	208	86	83	82	87	54	92	25	24	65	37	16	9	30	19	28
211264_at	37	5	7	78	48	9	12	28	33	56	51	11	3	1	11	2	11	13	7	25	1
211265_at	40	5	30	5	45	26	33	36	31	7	3	10	15	26	17	6	2	6	3	16	9
211266_s_at	90	47	62	231	21	81	191	59	79	207	63	118	4	3	6	9	15	3	3	3	4
211267_at	74	43	15	12	32	36	13	26	13	15	34	74	5	24	13	9	34	31	9	25	25
211269_s_at	232	74	39	191	359	263	188	74	245	222	142	96	5	5	17	23	4	5	89	9	16
211270_x_at	8042	5556	5252	2249	4787	2600	6164	5768	4212	4681	6296	5217	5336	6879	8293	7017	7143	7786	6643	6226	4970
211271_x_at	8279	5281	5537	2465	4018	1750	6740	6384	4391	5258	6825	4424	5381	6115	9446	7840	7974	6409	5831	5917	4552
211272_s_at	138	95	92	129	245	292	162	201	155	134	149	139	14	20	26	50	88	108	41	36	11
211273_s_at	32	23	8	17	30	15	61	51	8	11	4	20	7	2	5	1	1	2	4	2	1
211274_at	33	33	57	59	117	122	19	70	139	94	78	107	14	20	24	15	3	17	13	13	19
211275_s_at	1871	2377	2979	2907	2885	2856	2044	1560	3060	1918	3131	2402	4817	4325	5309	3390	3296	2947	4867	4975	6603
211276_at	45	13	68	23	12	20	27	42	84	94	63	6	17	21	15	23	7	5	9	16	16
211277_x_at	515	792	513	528	1259	934	449	515	451	562	563	611	86	68	54	97	113	72	135	103	193
211279_at	66	94	191	78	257	202	159	131	70	57	40	70	36	32	54	9	33	81	50	15	15
211280_s_at	106	35	90	65	47	66	103	108	66	55	18	24	64	70	31	51	57	51	50	45	27
211282_x_at	455	709	357	367	65	62	384	400	313	257	270	122	39	66	80	93	24	26	62	14	88
211284_s_at	799	1847	922	5380	623	1154	567	1104	765	2015	773	739	1004	843	317	358	359	390	734	1050	846
211285_s_at	1795	2029	2120	1667	1230	1476	1958	1941	1981	1711	1417	1170	2257	1463	1503	2172	1804	1659	2754	2192	2718
211286_x_at	28	97	22	30	32	79	50	20	11	24	13	18	7	13	3	46	30	67	1	9	7

211287_x_at	165	89	132	27	242	173	196	202	59	166	151	189	50	55	54	68	54	84	50	63	48
211289_x_at	347	442	304	362	672	730	429	480	371	342	310	263	435	453	332	265	282	244	327	254	179
211295_x_at	287	218	513	305	341	281	347	362	458	433	435	401	37	46	48	19	83	79	52	37	46
211296_x_at	24391	32340	25919	35479	25996	26943	34234	29866	29157	25564	21801	29432	18062	23255	22062	25472	26437	28802	16084	21408	20831
211297_s_at	1304	1046	1850	1262	1589	1368	1685	1888	1448	1778	1181	1371	1994	1554	1991	2377	2039	2383	2319	2888	2885
211298_s_at	58	32	56	72	117	191	36	32	47	46	43	55	26	30	14	8	2	18	20	32	13
211299_s_at	156	163	209	220	111	208	197	297	214	347	318	349	53	100	58	92	96	61	95	90	78
211300_s_at	732	731	549	522	506	415	1366	1475	939	1198	1241	1196	153	105	114	231	191	346	328	229	377
211301_at	81	9	138	119	27	35	86	139	75	115	102	104	5	36	10	48	5	3	6	59	2
211302_s_at	1114	277	149	63	45	43	1175	1614	71	99	107	124	32	19	7	466	343	563	11	11	14
211303_x_at	7	6	38	8	23	164	7	9	8	11	9	5	19	29	10	18	20	3	22	17	3
211304_x_at	230	272	262	113	891	566	348	224	233	320	384	266	163	116	261	55	90	84	101	66	89
211305_x_at	94	50	31	17	272	282	25	127	94	96	118	39	14	26	29	36	41	32	31	24	23
211306_s_at	36	4	3	14	8	13	4	8	15	6	4	4	4	3	19	8	4	3	1	2	2
211307_s_at	7	4	12	8	19	22	5	17	5	24	7	6	7	2	1	1	1	2	6	4	1
211310_at	67	5	22	9	30	38	4	54	7	60	41	15	17	16	1	9	2	19	3	23	23
211312_s_at	26	22	87	132	30	34	41	41	32	17	30	28	5	3	29	5	2	2	12	2	1
211313_s_at	36	61	142	36	418	85	105	62	39	130	117	35	22	20	7	6	24	34	32	26	29
211314_at	9	6	7	9	18	28	8	8	7	7	7	8	4	3	4	3	5	8	2	2	2
211315_s_at	85	7	9	19	298	206	7	48	19	6	26	23	21	35	29	12	4	42	25	6	17
211316_x_at	370	463	575	895	488	567	383	358	478	369	446	514	139	145	208	143	151	82	236	370	389
211317_s_at	157	158	239	445	426	419	179	241	207	250	263	339	99	67	55	86	59	66	144	215	184
211318_s_at	2227	1286	1827	1478	3792	2091	2056	1741	1865	1448	4004	2923	3483	3762	3688	1974	1920	1859	1906	1284	1268
211319_at	15	33	61	40	8	16	15	12	11	50	13	9	8	48	14	6	16	3	4	4	2
211320_s_at	562	476	146	212	649	443	661	656	250	129	571	403	139	224	121	64	108	103	18	69	27
211322_s_at	46	88	88	25	245	188	24	54	21	20	20	21	45	8	12	3	7	9	11	2	5
211323_s_at	155	95	37	94	40	17	99	21	8	27	14	29	11	55	18	70	18	54	25	26	33

211324_s_at	67	1	8	6	27	134	7	5	3	4	3	4	4	1	23	3	25	17	3	26	7
211325_x_at	523	703	541	811	917	946	669	560	646	605	695	686	228	263	287	294	278	212	346	219	345
211326_x_at	390	376	278	552	473	402	488	468	330	373	294	250	23	66	54	61	154	27	83	57	58
211327_x_at	365	412	218	656	265	317	481	509	288	320	217	263	56	95	92	118	71	99	96	58	81
211328_x_at	570	540	256	441	551	497	449	442	387	330	308	503	90	117	118	112	77	75	93	105	130
211329_x_at	422	314	53	354	213	333	221	358	298	183	131	127	46	87	49	42	68	117	83	79	108
211330_s_at	447	449	144	501	252	267	323	297	205	193	115	151	40	44	38	167	148	91	147	129	219
211331_x_at	71	270	23	149	34	32	170	157	75	53	43	24	5	54	5	5	41	34	13	5	5
211332_x_at	400	462	365	662	254	441	367	380	296	293	276	280	28	27	64	148	87	72	76	117	66
211333_s_at	124	77	175	129	203	117	77	117	80	123	104	114	74	73	41	48	21	44	20	47	40
211334_at	134	22	56	67	214	102	34	66	37	57	54	16	23	39	25	25	28	2	53	9	21
211336_x_at	28	28	19	25	30	34	90	40	31	39	20	19	7	3	7	6	3	4	4	11	5
211337_s_at	867	600	1141	957	991	1015	769	904	2072	1198	1262	711	457	467	747	565	661	634	1313	704	905
211338_at	17	43	11	12	67	12	4	7	4	4	6	30	5	21	2	2	7	3	3	7	11
211339_s_at	98	101	96	145	246	196	142	210	132	116	158	118	24	22	42	45	20	37	29	46	26
211340_s_at	369	746	396	186	1610	2481	464	325	446	419	1343	1247	1000	1519	1064	518	698	378	407	436	336
211341_at	11	4	11	9	187	137	15	12	11	4	10	14	23	29	34	3	2	6	36	27	16
211342_x_at	561	1119	796	1185	621	773	500	756	658	865	786	591	147	258	384	384	301	300	399	465	477
211343_s_at	521	140	416	18	937	281	150	207	201	130	1452	1635	967	1810	574	669	656	396	149	122	106
211345_x_at	27364	29067	24704	32379	21376	25278	25765	23763	25808	23954	21838	29404	17322	19899	18608	17251	20270	18649	13226	19314	17141
211347_at	110	24	15	66	263	308	91	197	140	24	28	18	41	57	134	187	151	137	81	102	48
211348_s_at	140	114	58	63	265	453	20	125	103	48	72	85	42	45	58	86	106	121	35	40	43
211349_at	87	107	123	65	293	317	89	66	144	96	114	129	19	5	24	25	41	18	32	24	16
211350_s_at	26	67	27	115	153	163	17	24	64	31	21	16	23	8	48	3	7	6	13	6	5
211351_at	4	10	7	48	93	65	7	5	7	21	20	4	3	1	18	1	1	18	13	19	1
211352_s_at	12	15	35	221	21	26	134	59	112	274	237	221	101	82	69	52	72	68	74	107	91
211353_at	25	7	5	19	25	30	5	7	8	4	6	14	2	15	4	3	4	3	3	5	2

211354_s_at	86	125	50	167	76	83	127	143	123	89	90	65	39	37	46	121	113	96	29	47	74
211355_x_at	239	67	96	142	168	203	162	421	128	165	126	148	29	60	49	175	132	99	33	67	76
211356_x_at	169	172	168	241	190	257	280	335	170	158	175	209	51	39	67	142	189	125	95	72	94
211357_s_at	5	27	5	30	144	567	11	5	9	4	6	19	29	25	15	6	7	6	25	6	11
211358_s_at	1050	1640	937	1045	389	676	802	637	672	720	992	576	200	289	356	280	248	144	241	453	290
211359_s_at	101	15	102	85	180	228	261	113	199	194	156	123	20	11	21	24	8	15	37	16	21
211360_s_at	41	142	60	76	41	215	148	132	126	168	134	194	3	7	29	2	4	34	7	46	25
211361_s_at	33	23	57	58	33	56	20	51	28	20	28	25	1	1	6	1	2	14	6	1	10
211362_s_at	144	33	107	178	227	331	142	174	159	130	125	126	22	6	24	29	28	17	29	21	16
211363_s_at	475	252	739	295	232	211	633	889	1131	1690	260	102	224	96	40	289	263	261	493	159	264
211364_at	384	272	281	326	243	228	358	447	281	418	306	279	58	44	74	72	48	62	52	47	45
211365_s_at	16	10	15	16	36	55	8	15	13	15	14	44	1	2	2	2	4	3	1	4	1
211366_x_at	250	354	311	375	533	434	266	235	336	293	207	225	101	97	65	36	59	79	117	142	106
211367_s_at	48	40	31	34	104	24	32	32	15	11	4	15	1	18	17	25	6	16	6	21	1
211368_s_at	12	57	4	26	8	5	7	8	4	13	4	38	2	5	2	2	1	3	1	2	1
211369_at	5	7	9	14	19	22	5	7	36	3	14	6	3	10	2	17	47	18	1	32	1
211370_s_at	275	398	331	480	60	234	466	318	209	288	323	202	165	153	249	123	131	129	207	296	204
211371_at	65	54	66	35	87	149	87	115	59	74	34	75	6	23	21	20	17	20	8	4	28
211372_s_at	22	12	378	307	27	35	89	32	349	214	17	18	2	3	3	8	4	7	394	327	216
211373_s_at	431	369	293	189	282	845	390	289	312	277	335	297	250	337	312	186	348	229	262	225	218
211374_x_at	142	47	80	149	223	265	80	58	58	112	102	215	34	12	58	33	56	8	88	61	31
211375_s_at	5337	4152	2530	1584	2643	2694	2557	3067	3116	2679	1921	1842	2481	1325	1077	1654	1298	1392	2039	938	879
211376_s_at	1557	1390	1000	910	1168	927	1181	1345	974	1122	1245	889	1147	1265	1254	2116	2867	2878	1836	1481	1117
211377_x_at	9	16	9	17	52	42	8	9	9	7	6	6	5	7	6	6	5	4	6	5	3
211378_x_at	31700	27517	32092	32582	35214	29668	36789	35210	35918	31603	32830	35796	23364	31958	35359	28748	31483	31821	18506	26786	21123
211379_x_at	136	62	28	38	91	103	187	108	35	38	67	82	38	40	46	163	94	163	9	22	35
211380_s_at	38	29	57	138	40	59	28	24	50	43	37	99	22	13	35	4	4	15	17	44	8

211381_x_at	8	15	11	172	19	19	9	8	108	45	80	99	2	7	6	2	4	1	7	4	17
211382_s_at	758	567	718	748	348	845	543	577	506	648	343	239	183	146	176	260	321	248	305	536	380
211383_s_at	492	331	601	733	335	409	501	610	574	720	459	363	321	396	472	758	696	402	487	430	651
211384_s_at	9	9	22	6	33	27	7	17	20	6	6	6	6	44	18	21	19	28	5	8	3
211385_x_at	1241	754	526	956	1010	1274	1302	900	597	710	749	701	628	658	527	677	610	542	321	327	379
211386_at	87	9	65	38	92	9	28	91	31	84	75	8	28	35	23	9	3	2	6	17	9
211387_x_at	370	264	278	396	352	328	431	453	498	495	321	352	41	74	70	68	63	59	112	116	115
211388_s_at	221	96	157	114	257	226	201	104	194	183	183	80	16	41	9	53	55	6	79	28	16
211389_x_at	30	30	27	119	51	38	33	107	80	129	75	34	2	66	36	9	3	17	47	25	3
211390_at	15	12	19	40	29	28	11	15	9	13	13	11	2	2	3	2	2	6	6	2	3
211391_s_at	507	404	404	398	291	246	612	606	516	428	429	226	43	35	42	97	48	77	77	51	39
211392_s_at	729	459	468	522	96	361	763	724	625	612	568	431	134	158	112	128	94	84	182	122	119
211393_at	65	91	136	128	136	51	182	153	166	171	125	103	38	30	58	38	69	24	50	69	53
211395_x_at	315	179	144	176	33	44	156	282	274	298	311	269	90	62	50	68	77	101	69	46	56
211396_at	26	18	24	17	34	101	29	15	45	28	13	19	23	28	11	6	10	11	5	35	15
211397_x_at	37	17	46	35	56	34	32	32	35	42	38	24	8	12	10	3	8	9	11	7	6
211398_at	74	22	73	38	169	91	44	32	84	21	85	11	31	62	31	22	23	18	27	13	16
211399_at	66	13	98	76	757	457	56	73	59	13	31	39	32	8	27	57	26	35	8	44	3
211400_at	64	56	43	123	33	55	184	71	40	85	27	83	6	3	15	2	2	2	5	20	17
211401_s_at	24	18	76	316	52	184	16	127	84	105	17	20	26	28	22	56	7	4	55	59	58
211402_x_at	287	127	289	234	737	751	253	363	252	291	320	225	79	114	55	23	45	92	70	17	82
211403_x_at	79	111	95	88	462	218	164	98	134	130	159	122	20	22	45	45	33	38	27	29	18
211404_s_at	2532	4128	1990	2526	3799	3991	1978	2557	2390	2910	3033	4526	2425	2076	1209	822	1175	1076	1078	1348	1606
211405_x_at	455	276	551	410	506	265	435	410	470	453	442	393	48	66	34	54	56	85	70	18	42
211406_at	512	366	308	374	217	230	563	536	380	369	273	194	140	263	576	974	925	873	714	478	286
211407_at	94	92	37	8	311	328	78	99	120	11	92	52	76	44	55	57	32	27	43	49	50
211410_x_at	210	117	81	58	434	230	80	145	108	84	63	243	27	69	13	7	8	35	12	14	7

211411_at	11	11	15	26	37	30	13	11	24	15	50	30	34	45	35	8	8	11	24	6	7
211412_at	9	7	41	10	81	7	9	30	8	27	16	11	11	7	1	2	1	3	1	2	11
211413_s_at	28	18	8	28	38	102	20	11	5	10	13	9	5	3	17	4	15	3	21	13	11
211414_at	78	40	110	94	133	91	58	59	119	98	148	142	41	26	54	27	14	35	43	37	35
211416_x_at	413	359	273	450	614	778	496	422	404	369	316	214	22	57	58	45	78	80	107	133	78
211417_x_at	577	601	336	574	525	607	603	462	405	391	364	343	91	106	76	153	232	121	186	207	158
211419_s_at	12	40	24	78	139	141	53	84	56	39	13	39	29	21	11	2	12	27	39	49	68
211421_s_at	124	79	39	113	114	194	93	102	5	17	87	21	37	20	7	67	40	45	8	24	7
211422_at	89	56	52	40	125	40	50	33	50	63	53	92	29	20	38	43	24	8	23	18	13
211423_s_at	1048	1224	1443	2350	2054	1277	1240	1326	1084	1196	2091	1468	1893	2501	3351	3556	3367	2638	1627	2675	2970
211424_x_at	40	47	45	146	184	163	32	70	37	57	38	40	9	1	18	30	12	23	37	37	22
211425_x_at	54	13	95	118	269	363	30	57	71	77	212	182	98	68	52	19	27	17	39	25	22
211426_x_at	814	797	849	828	559	800	1007	1388	910	1332	1033	577	75	78	132	70	61	59	146	97	186
211427_s_at	4	4	37	5	11	23	4	9	4	6	7	13	8	2	2	9	1	2	2	4	1
211428_at	56	89	4	26	25	22	50	8	52	80	26	73	15	3	5	1	32	10	2	2	9
211429_s_at	197	80	2474	4204	2463	3546	180	117	993	2010	6121	3074	2088	2254	2462	146	207	150	1426	1721	1465
211430_s_at	19	17	132	16	767	579	19	13	21	11	24	30	37	73	42	6	5	5	6	8	3
211431_s_at	176	79	123	25	29	141	117	174	25	122	159	123	31	56	21	31	9	29	43	37	17
211432_s_at	610	466	465	244	410	286	857	587	581	467	799	418	232	172	148	138	127	156	157	122	130
211433_x_at	511	400	1038	1079	1221	1028	672	500	411	482	578	329	105	81	119	160	191	137	116	139	119
211434_s_at	38	11	164	177	44	32	53	34	104	136	107	69	9	16	9	9	29	6	101	106	100
211435_at	71	91	128	131	38	38	86	137	103	183	84	104	5	4	15	3	17	2	15	19	19
211436_at	21	5	57	48	115	15	8	9	8	31	7	10	2	3	16	7	2	1	4	14	6
211437_at	13	5	16	5	19	13	5	8	8	13	6	11	2	33	18	3	3	3	4	2	5
211438_at	12	11	9	25	16	40	11	16	8	8	7	19	3	2	9	3	17	4	26	2	4
211439_at	460	276	311	261	252	313	196	321	341	330	279	290	68	64	106	52	82	34	84	75	134
211440_x_at	210	145	283	270	283	185	268	240	302	471	232	298	30	40	26	38	21	25	86	56	64

211441_x_at	128	27	220	186	219	73	158	125	32	229	178	131	47	56	44	9	36	39	90	81	98
211442_x_at	122	6	122	142	56	199	13	33	15	103	36	36	37	45	31	26	24	20	33	47	43
211444_at	573	296	201	374	346	351	648	647	413	411	347	442	220	109	139	272	256	158	316	190	212
211445_x_at	3303	4430	2480	2924	3359	2510	1883	2922	2792	2718	2693	2106	1523	986	1078	954	701	1240	1330	1600	1502
211446_at	151	114	80	197	85	43	72	131	130	141	84	170	7	6	22	7	2	30	8	7	3
211447_s_at	212	251	15	30	15	20	258	252	79	32	115	79	13	2	19	61	6	21	4	9	11
211448_s_at	13	13	15	14	44	42	12	13	12	8	9	9	2	6	15	4	4	11	5	4	1
211449_at	62	54	58	54	10	91	57	79	80	50	45	45	32	3	27	22	25	23	20	27	16
211450_s_at	646	390	912	679	1175	668	1112	1452	2040	1437	2706	1797	928	574	181	203	182	193	507	259	317
211451_s_at	26	6	26	32	63	56	69	25	41	36	82	45	70	56	15	2	7	23	22	4	26
211452_x_at	3385	2848	5863	3910	5983	4323	3370	3080	4937	5775	4202	3406	2888	2864	2940	2726	2414	3911	4073	4409	4710
211453_s_at	60	212	270	264	21	36	156	202	411	243	232	24	42	9	1	3	6	4	6	13	4
211454_x_at	867	1240	1305	1723	752	685	1234	1785	1635	1870	1255	1155	184	240	211	168	124	245	344	321	256
211455_at	151	124	191	199	249	341	101	143	187	194	107	21	35	31	125	7	11	74	26	46	29
211456_x_at	25844	21634	6641	6315	7194	6093	14081	22581	5394	3730	9877	20535	10653	9889	7915	12690	13557	7132	6046	7536	2429
211457_at	111	67	73	131	29	62	83	69	63	89	141	145	4	9	21	23	48	11	35	36	3
211458_s_at	381	1162	301	497	1725	1544	557	672	74	150	2108	2823	1392	1128	1144	425	425	462	131	501	466
211459_at	11	10	57	63	23	30	25	5	8	39	34	74	1	4	18	18	12	17	2	16	5
211460_at	181	123	174	224	241	280	209	150	126	162	182	177	59	41	59	23	30	44	45	57	49
211461_at	19	10	19	10	33	39	21	11	15	18	10	10	6	28	4	12	22	4	11	7	5
211462_s_at	116	84	23	76	30	26	62	87	20	56	14	33	2	8	11	13	5	19	6	8	27
211463_at	50	55	62	70	29	9	48	82	21	77	18	9	16	12	23	17	30	21	43	21	11
211464_x_at	418	202	350	419	429	508	295	212	276	396	259	383	542	489	479	320	281	258	1879	1290	1709
211465_x_at	751	605	796	799	1112	714	685	697	792	875	517	577	91	129	161	92	111	122	144	197	113
211466_at	17	9	9	74	14	50	4	24	45	11	4	13	8	3	27	1	8	10	25	1	3
211467_s_at	44	108	313	471	48	86	57	36	343	207	50	16	15	13	32	12	9	8	87	65	94
211468_s_at	155	41	98	67	469	159	44	28	96	25	84	96	157	185	196	30	11	54	92	67	29

211469_s_at	26	45	16	47	81	79	45	46	107	48	77	39	16	12	55	6	46	3	8	11	30
211470_s_at	578	225	373	297	89	179	447	533	239	409	21	45	31	77	26	80	105	105	305	287	252
211471_s_at	443	333	217	182	355	335	501	466	321	299	530	371	131	143	268	238	215	238	53	67	96
211472_at	40	10	30	41	54	114	21	18	17	24	30	30	3	22	6	8	32	16	11	41	2
211473_s_at	87	36	156	53	99	24	146	133	79	25	20	33	4	8	1	8	4	8	9	2	3
211474_s_at	1888	1013	1545	1511	1647	1302	2001	1700	1512	2163	1896	1433	1537	1793	959	1150	1260	1091	1235	1281	972
211475_s_at	878	927	836	1195	507	723	841	1106	1201	1219	678	681	789	777	509	829	1036	694	911	561	518
211476_at	7	9	1	5	7	63	11	5	4	4	7	1	1	15	3	10	8	1	6	2	1
211478_s_at	11	152	340	475	56	34	150	82	213	629	28	26	1	2	1	2	8	18	190	228	292
211479_s_at	77	33	61	69	165	71	76	46	75	99	36	60	26	1	28	11	6	20	22	24	27
211480_s_at	107	17	28	59	96	42	15	15	9	43	24	60	1	3	3	19	4	2	22	4	28
211481_at	108	19	19	141	18	54	54	21	170	20	63	19	22	22	46	38	23	34	6	26	20
211483_x_at	171	113	52	146	313	349	191	191	83	180	102	137	38	54	13	38	37	6	22	4	27
211484_s_at	60	97	52	74	124	129	56	45	88	89	50	57	18	15	54	8	6	13	39	54	6
211485_s_at	53	101	81	88	71	211	122	141	88	27	202	102	356	330	168	31	11	34	7	117	87
211486_s_at	15	18	11	21	22	20	15	15	17	20	14	14	4	4	5	3	3	7	13	7	3
211487_x_at	24709	44149	29510	39320	25815	28312	28681	30122	35722	29631	21966	31746	17050	25259	25289	22959	25664	27550	15600	22821	19069
211488_s_at	142	24	73	147	179	228	73	80	165	147	155	134	28	54	19	21	42	32	38	9	38
211489_at	60	61	71	133	150	231	150	165	151	169	144	214	14	27	15	18	8	17	5	17	5
211490_at	16	7	7	14	23	194	17	15	21	48	9	28	9	4	3	3	6	14	2	2	15
211491_at	139	43	31	44	324	192	82	12	54	13	54	21	7	6	4	8	8	4	25	44	7
211492_s_at	54	26	8	45	96	134	56	84	52	46	7	16	1	14	15	27	6	2	17	22	14
211493_x_at	232	231	269	186	267	214	262	207	249	211	249	196	44	111	14	64	70	93	49	42	27
211494_s_at	179	36	256	274	331	198	246	137	381	472	159	89	20	29	18	5	7	6	158	144	126
211495_x_at	118	103	446	643	241	474	68	220	409	291	375	216	126	147	105	52	63	38	212	200	176
211496_s_at	99	7	38	57	162	156	33	63	33	29	37	34	16	6	11	21	6	2	37	7	35
211497_x_at	197	210	312	317	326	220	375	327	367	316	298	271	31	65	14	94	61	18	82	59	52

211498_s_at	25	24	18	21	41	51	33	37	25	14	23	25	2	6	14	10	11	9	7	9	5
211499_s_at	394	494	186	372	33	30	323	271	90	211	145	308	50	7	45	96	76	44	6	66	32
211500_at	28	19	15	28	45	62	37	33	31	31	30	25	16	35	30	10	29	43	23	15	41
211501_s_at	2429	1933	2534	1226	2334	2594	2653	2359	3665	2885	2969	1856	1696	2394	1725	916	895	1200	1865	1067	1542
211502_s_at	107	39	72	76	330	43	164	121	135	189	20	67	10	27	38	53	65	53	41	26	31
211503_s_at	1151	1002	810	1354	939	1228	1477	1316	1472	1391	1693	1365	2052	1839	1016	1346	1302	872	1300	1012	889
211504_x_at	360	279	1291	835	1255	1230	680	554	924	1220	1100	1136	157	155	222	55	76	90	172	157	256
211505_s_at	1502	1549	2444	2797	2110	1930	1651	1891	2778	3543	4092	3167	2390	1375	1458	1289	1089	1056	2903	2301	2893
211506_s_at	50	172	252	35	203	312	78	38	33	95	171	204	4	15	3	23	50	93	51	37	110
211507_s_at	50	84	68	54	52	141	65	281	54	59	67	80	16	49	3	5	2	4	16	8	36
211508_s_at	22	44	69	56	19	27	93	63	47	67	95	75	2	23	5	2	7	25	4	4	1
211509_s_at	8348	8099	10654	7603	8901	5052	8203	7577	7777	8204	7795	7958	8070	10123	10663	10334	11489	9152	12745	9607	10930
211510_s_at	9	50	20	144	14	43	74	21	21	36	16	14	10	24	14	10	11	1	15	2	2
211512_s_at	369	437	414	449	264	153	441	438	452	521	331	226	86	27	110	118	113	102	101	67	70
211513_s_at	161	209	240	211	223	153	167	278	158	295	264	84	50	105	72	50	107	69	63	14	17
211514_at	65	77	52	35	37	55	97	107	119	117	75	103	33	17	22	12	3	2	18	3	4
211515_s_at	221	133	206	244	351	391	317	243	201	253	408	336	57	50	36	58	38	32	32	36	58
211516_at	132	64	84	133	158	71	99	117	143	119	85	60	23	15	26	28	16	20	32	2	15
211517_s_at	4	7	7	13	62	13	13	4	4	4	6	8	12	4	4	3	10	3	8	3	10
211518_s_at	1322	1467	3013	11747	696	1990	2665	2262	5738	3834	1077	351	3374	3370	2683	2313	2226	2194	2977	2204	2431
211519_s_at	1069	227	1164	327	1104	267	1204	1367	1141	1146	1381	1203	656	717	374	365	361	302	482	204	203
211520_s_at	29	36	5	4	91	141	42	53	4	4	4	5	1	23	5	1	2	17	11	22	2
211521_s_at	165	108	153	118	232	149	192	135	112	221	216	207	4	44	20	15	6	19	8	28	27
211522_s_at	5	10	5	6	7	8	33	3	28	31	4	8	1	1	14	1	3	2	2	1	1
211523_at	9	55	65	16	33	55	70	17	58	43	13	18	7	3	1	17	1	17	26	2	12
211524_at	33	36	43	96	219	90	87	111	16	22	54	123	14	2	49	20	12	22	17	6	11
211525_s_at	24	29	77	40	41	39	85	48	50	74	63	25	12	13	30	9	1	8	4	2	2

211526_s_at	25	7	38	45	16	17	24	16	83	8	28	39	1	17	33	3	16	2	4	1	3
211527_x_at	409	247	452	181	22	42	362	176	163	179	112	79	42	3	8	56	48	85	25	126	125
211528_x_at	3866	9340	2934	4527	2361	2352	4145	4337	2863	2611	5102	3054	3081	2258	671	1055	1299	1672	643	704	472
211529_x_at	3010	8856	2075	2867	1506	2298	3371	3558	2008	1821	4515	3087	2850	2021	960	1094	1209	1851	535	681	514
211530_x_at	912	1993	1095	1221	2235	1285	973	1214	780	1413	2089	2340	2516	1169	293	382	408	546	299	370	264
211531_x_at	173	134	184	283	551	775	123	131	483	330	270	276	44	43	22	91	70	46	29	41	43
211532_x_at	12	7	11	9	22	17	5	11	13	10	14	8	3	4	6	4	6	6	4	3	3
211533_at	9	6	7	16	45	51	8	13	4	14	7	4	1	28	5	3	1	3	2	3	7
211534_x_at	106	88	34	41	624	449	44	42	35	35	863	960	980	201	255	29	42	44	47	51	46
211535_s_at	865	810	20	85	792	910	688	637	62	15	782	764	318	266	256	206	225	248	81	200	112
211536_x_at	665	594	962	764	1036	754	849	1071	864	1147	703	741	164	184	243	297	260	287	259	166	244
211537_x_at	859	665	1135	820	804	560	1011	1177	1030	1224	877	789	254	313	210	435	386	390	288	242	338
211538_s_at	193	60	153	171	840	735	272	264	139	219	918	1447	595	697	643	118	123	89	287	96	108
211540_s_at	139	118	186	151	315	513	184	300	222	273	354	338	60	27	7	20	54	4	26	6	33
211541_s_at	41	61	35	75	80	67	40	38	74	46	126	29	4	17	5	11	5	5	5	6	5
211542_x_at	41362	45478	37466	48594	33841	30997	41318	39730	32477	34680	29358	36991	25891	33354	28735	28344	31882	29962	19090	28407	20899
211543_s_at	372	316	307	235	948	542	398	385	356	429	571	323	335	383	356	152	276	238	210	86	70
211544_s_at	259	68	115	98	261	48	203	238	151	169	112	28	13	12	5	3	4	33	40	32	19
211545_at	45	33	39	113	40	22	37	51	187	60	75	69	10	20	9	4	3	11	6	12	17
211546_x_at	151	212	107	203	891	181	196	136	268	187	186	124	51	54	86	91	103	136	76	123	39
211547_s_at	450	206	590	442	573	406	930	1003	755	1017	981	613	154	197	173	354	282	235	307	153	328
211548_s_at	67	97	148	1880	812	1211	90	127	131	105	632	620	1414	1585	1603	6	7	16	489	428	545
211549_s_at	33	45	95	573	1072	1408	62	102	153	122	438	440	673	921	279	25	21	7	113	56	147
211550_at	16	10	11	13	26	30	15	17	31	32	14	16	2	3	6	3	4	3	6	4	8
211551_at	249	136	236	199	517	298	166	252	227	257	132	224	58	36	62	80	60	59	73	47	22
211552_s_at	417	448	564	662	96	461	392	313	330	498	320	249	57	113	80	82	23	49	118	69	62
211553_x_at	86	124	76	141	324	324	109	169	169	126	155	152	38	27	5	3	25	7	6	13	3

211554_s_at	267	117	366	360	93	50	427	393	393	537	496	464	59	64	44	22	66	94	60	34	30
211555_s_at	173	60	138	142	133	142	130	108	99	109	71	122	3	19	41	40	24	25	22	29	16
211556_at	19	4	11	6	15	9	20	4	5	41	7	10	2	3	1	2	1	7	3	1	2
211557_x_at	98	62	64	66	173	137	121	240	33	187	95	131	10	65	11	17	55	6	12	17	5
211558_s_at	737	865	498	460	544	527	575	669	484	397	341	305	692	478	454	788	933	774	418	537	437
211559_s_at	488	440	609	503	242	431	493	688	221	553	449	501	73	116	146	292	219	218	74	88	162
211560_s_at	16	7	16	13	18	26	13	22	16	11	20	13	5	5	3	2	15	4	4	4	2
211561_x_at	529	394	358	520	423	438	714	688	673	764	745	491	112	145	161	192	184	152	136	180	151
211562_s_at	12	50	12	18	12	60	24	29	19	13	14	13	31	17	13	52	15	6	29	32	5
211563_s_at	658	678	650	931	554	642	815	823	983	1231	918	723	816	656	941	1069	839	833	1572	784	1348
211564_s_at	8	22	83	16	584	184	34	18	24	14	2342	1737	2024	1491	1508	3	32	4	2	1	4
211565_at	24	84	118	52	140	51	76	71	25	106	98	103	28	17	34	6	11	19	37	24	18
211566_x_at	364	423	388	720	340	773	538	550	407	382	426	479	326	335	331	267	312	287	313	272	306
211567_at	30	39	23	50	191	216	57	24	12	25	57	30	1	2	2	4	11	1	11	2	8
211568_at	1	1	5	3	60	130	4	1	3	18	3	3	6	1	2	2	16	8	1	8	3
211569_s_at	602	569	612	1260	1035	664	429	596	704	545	489	758	1207	1152	1201	544	549	645	1423	772	965
211570_s_at	16	19	14	17	25	93	11	5	44	21	68	8	29	7	2	3	3	27	34	4	5
211571_s_at	507	41	18	4	30	27	277	441	7	7	3	29	2	14	17	104	97	198	1	1	4
211572_s_at	213	124	118	150	151	304	171	205	138	189	280	175	61	75	107	64	14	41	29	41	55
211573_x_at	153	73	39	106	1119	556	138	74	13	74	3320	1925	384	300	227	29	14	16	4	4	3
211574_s_at	978	1040	1423	1600	1439	621	1317	1265	1610	2059	1846	1687	268	355	601	667	717	700	468	436	1154
211575_s_at	872	620	1198	742	811	779	1232	783	1002	1043	911	642	778	824	960	1324	1236	1327	1312	1086	1345
211576_s_at	1025	815	469	467	790	237	772	562	846	489	682	430	536	495	542	494	606	618	561	470	351
211577_s_at	34	113	175	28	56	78	41	218	45	103	16	43	54	6	69	35	4	5	23	12	14
211578_s_at	438	400	205	47	404	398	579	635	284	321	360	153	147	166	133	308	381	412	155	111	116
211579_at	85	9	27	13	154	267	78	92	51	98	47	6	11	23	2	2	3	15	2	27	14
211580_s_at	155	22	125	389	43	55	130	186	111	196	175	136	13	4	7	27	8	8	34	33	6

211581_x_at	46	21	24	45	36	28	36	28	37	35	41	33	10	6	13	3	6	6	5	4	7
211582_x_at	30	21	24	27	29	28	28	32	27	25	37	30	4	7	10	9	9	5	5	7	3
211583_x_at	95	49	28	35	356	54	33	24	33	27	114	55	12	2	4	4	7	2	6	6	10
211584_s_at	185	34	448	365	260	155	478	397	652	590	343	396	184	228	137	245	146	194	401	321	294
211585_at	95	24	75	9	27	69	76	96	56	92	9	54	1	1	6	2	6	3	11	29	13
211586_s_at	9	6	9	23	34	11	11	9	20	11	10	8	2	2	2	1	1	3	4	1	1
211587_x_at	238	83	119	263	122	52	105	207	107	274	254	63	42	36	17	69	33	64	158	19	135
211588_s_at	22	17	24	17	44	42	16	25	12	28	23	23	19	12	24	12	2	45	22	51	34
211589_at	91	47	50	50	30	26	168	80	67	129	104	172	5	8	9	27	5	2	2	4	2
211590_x_at	329	442	365	524	65	52	489	417	371	542	451	466	37	17	45	7	70	69	13	15	35
211591_s_at	13	17	22	32	44	71	9	15	24	17	21	9	7	16	34	6	3	10	17	3	2
211592_s_at	16	39	12	30	14	22	32	5	7	10	10	25	2	21	3	2	11	9	17	9	19
211593_s_at	675	832	762	872	427	503	775	682	872	731	857	759	392	348	314	259	282	307	360	265	252
211594_s_at	724	420	597	511	1226	724	738	499	781	556	608	677	1723	1573	949	922	685	817	1077	1176	829
211595_s_at	1217	884	1942	1640	1041	641	912	1029	2208	1480	1445	1110	1873	1405	894	596	742	493	1600	1533	702
211596_s_at	149	142	402	343	1342	778	115	98	443	516	1573	755	587	968	547	159	256	117	398	363	467
211597_s_at	15	15	26	19	89	71	110	16	124	87	134	68	4	11	36	33	2	5	308	131	114
211598_x_at	245	96	167	16	231	152	195	224	99	123	161	50	20	33	29	40	2	24	6	25	29
211599_x_at	2056	1045	5503	2327	210	417	2121	3054	5135	7052	638	552	63	83	107	532	443	562	796	458	706
211600_at	4177	6677	14492	16859	4368	8419	15557	19348	12654	13515	11129	9580	943	2273	2351	3264	5077	3823	3822	1756	1440
211601_at	4	34	11	52	7	8	4	5	13	8	6	15	1	14	2	1	1	1	1	2	8
211602_s_at	56	18	71	16	157	97	62	84	11	49	141	206	28	27	43	33	41	63	1	44	1
211603_s_at	251	95	526	91	770	79	268	268	242	507	2147	415	461	493	517	138	192	111	121	160	84
211604_x_at	12	7	8	12	25	40	13	11	28	10	16	19	4	5	21	7	16	8	15	7	6
211605_s_at	85	150	175	230	148	157	95	131	120	164	135	146	7	14	10	4	7	17	8	5	9
211606_at	33	15	72	83	26	15	109	73	88	115	68	63	1	17	11	5	12	3	12	13	7
211607_x_at	58	54	50	39	48	38	53	51	67	32	317	293	8	21	15	8	12	27	7	4	6

211608_at	36	77	28	56	37	42	44	53	62	130	47	39	4	10	10	38	14	30	8	6	14
211609_x_at	4437	4427	3243	2049	3215	2458	4445	3390	2614	4000	3012	3790	4327	4254	2981	3028	2746	2162	2840	2467	1715
211610_at	267	227	293	162	32	132	196	318	336	377	212	220	62	35	15	13	15	22	106	27	37
211611_s_at	82	64	15	62	41	43	117	120	83	74	72	121	48	12	16	31	28	41	43	70	23
211612_s_at	472	463	853	767	643	699	529	653	709	1013	850	817	426	588	640	429	383	374	754	687	692
211613_s_at	136	85	129	52	148	101	280	216	221	270	222	143	14	30	10	19	23	7	27	17	22
211614_at	26	15	9	40	30	13	28	9	19	8	10	18	4	2	2	22	4	3	2	6	2
211615_s_at	1577	1943	1871	1345	1542	1524	2284	2066	2106	2306	1330	1370	2064	1103	2306	2315	1485	2173	2406	3636	5025
211616_s_at	107	55	60	63	180	172	42	78	78	80	108	107	14	8	44	23	24	9	29	22	23
211617_at	8	9	31	13	307	187	7	13	8	7	7	5	4	2	26	4	6	21	5	3	2
211618_s_at	9	5	5	14	11	12	4	5	9	7	9	5	5	3	5	5	3	6	2	3	1
211619_s_at	22	137	31	65	719	989	12	83	20	48	252	18	28	59	93	47	51	57	51	54	4
211620_x_at	30	49	126	69	430	387	27	70	13	82	286	312	35	54	52	23	38	30	10	31	35
211621_at	159	124	151	109	191	216	118	121	104	131	114	145	28	35	12	30	4	26	27	22	14
211622_s_at	632	555	1133	993	454	747	893	805	1402	1268	1231	704	303	420	513	129	125	226	367	208	199
211623_s_at	7473	4673	10555	4645	6551	5664	8729	7051	15229	11950	8406	7430	10776	8923	8539	8516	8449	6334	10135	10296	5345
211624_s_at	87	24	132	34	76	73	90	34	88	34	38	19	5	10	6	24	11	27	15	31	14
211625_s_at	8	10	12	31	22	20	9	12	4	8	9	6	2	2	1	6	3	3	4	1	3
211626_x_at	48	13	16	88	10	27	25	69	92	14	38	70	2	2	5	37	10	5	3	12	7
211627_x_at	7	27	66	47	11	30	5	46	58	6	30	13	4	22	1	2	2	6	2	21	1
211628_x_at	21777	41249	25337	39386	22109	30618	24250	23756	25836	21727	15248	22859	20023	21753	22932	23946	25746	21509	17785	24521	22070
211630_s_at	1999	1570	1738	2030	2172	1868	2270	1884	1246	1760	2935	1908	2331	2432	1381	990	995	1085	896	829	751
211631_x_at	324	394	209	203	313	134	517	400	258	228	239	151	22	52	30	41	24	45	64	31	27
211632_at	7	9	12	12	55	15	8	12	33	8	11	8	17	7	35	9	37	4	17	2	3
211633_x_at	30	16	68	25	296	188	16	16	19	8	18	28	7	3	6	6	21	18	5	9	4
211634_x_at	20	21	15	18	44	55	20	20	87	28	20	52	4	3	3	5	3	3	6	9	5
211635_x_at	73	7	15	21	52	42	50	111	56	36	10	31	5	6	15	5	6	4	9	8	4

211636_at	8	2	5	12	16	20	15	4	5	4	3	5	2	4	5	3	3	1	3	3	2
211637_x_at	9	7	12	13	144	237	12	11	11	14	10	14	7	19	5	29	46	87	27	31	4
211638_at	56	10	8	35	36	31	32	9	23	35	10	43	14	3	3	2	2	3	8	9	3
211639_x_at	11	12	11	17	34	13	15	7	13	20	14	20	25	9	9	2	7	4	4	36	6
211640_x_at	19	4	8	23	43	36	24	20	19	15	14	39	3	5	4	9	3	4	10	6	7
211641_x_at	93	57	65	87	275	419	72	111	64	116	71	129	33	38	25	20	5	30	25	40	35
211642_at	19	5	8	6	32	24	44	8	8	52	23	11	11	4	3	2	5	3	14	2	12
211643_x_at	77	125	77	211	98	46	66	133	78	61	149	67	13	10	7	9	8	8	8	11	44
211644_x_at	48	22	28	56	23	28	12	33	32	24	30	29	4	5	7	6	2	6	7	7	5
211645_x_at	56	47	80	67	115	130	217	80	80	68	21	96	29	6	5	7	8	56	8	37	17
211646_at	11	21	5	12	161	95	19	8	7	8	45	5	9	28	14	6	3	6	8	2	2
211647_x_at	7	16	9	3	36	23	16	11	12	36	10	29	5	2	5	3	3	26	4	29	2
211648_at	29	72	11	23	133	50	69	17	13	21	20	19	1	3	2	2	2	2	2	2	3
211649_x_at	16	15	16	21	30	24	32	18	20	17	18	28	29	20	18	30	4	29	11	19	23
211650_x_at	17	16	18	17	40	34	16	28	7	10	11	10	2	7	6	31	17	11	7	6	7
211651_s_at	492	445	978	321	400	238	470	711	611	580	328	254	218	275	131	129	144	61	217	370	363
211652_s_at	349	201	326	237	89	112	330	363	338	285	378	307	28	26	11	12	52	3	7	56	42
211653_x_at	131	84	545	9291	80	426	192	172	348	657	97	139	50	92	26	52	79	31	342	873	545
211654_x_at	303	292	222	216	837	867	317	289	211	205	655	790	365	546	431	87	122	154	104	81	43
211655_at	8	60	54	74	55	167	3	12	45	35	21	57	13	16	13	29	3	13	13	29	1
211656_x_at	241	316	145	48	290	375	236	215	123	38	436	369	71	91	154	138	171	92	13	29	22
211657_at	276	113	612	273	265	235	170	202	496	721	155	202	44	38	9	66	29	25	1379	1787	1524
211658_at	2848	1263	852	1504	257	224	1374	1982	725	688	904	1152	3421	369	515	768	821	353	444	501	366
211659_at	21	19	19	9	18	27	9	21	23	7	20	23	3	4	3	8	7	4	7	6	2
211660_at	230	142	151	248	371	695	162	162	150	214	135	180	51	77	58	131	30	83	60	22	78
211661_x_at	515	494	507	419	610	418	388	434	464	458	429	284	59	29	122	115	62	40	53	60	72
211662_s_at	11529	14807	9373	7705	7325	7800	9704	8866	8623	7399	6530	4609	6317	10040	10320	14035	14227	16094	10111	10736	9175

211663_x_at	173	156	80	162	275	323	159	248	111	210	169	173	81	99	78	7	23	6	23	17	10
211665_s_at	147	107	206	310	338	417	191	329	165	267	222	197	41	33	61	65	59	75	55	88	86
211666_x_at	26917	22430	18948	14431	12527	16957	22067	24683	18308	18788	15397	18819	12642	12404	7759	12896	13369	10486	7120	10249	8878
211667_x_at	8	38	22	61	41	151	13	107	103	91	61	18	65	65	39	66	77	73	59	44	42
211668_s_at	1380	2567	907	206	311	325	811	645	221	184	927	689	69	51	41	316	389	465	3	34	36
211670_x_at	48	58	85	31	100	222	78	33	33	29	77	102	34	12	11	3	16	7	8	16	15
211671_s_at	151	326	98	100	432	243	277	358	79	88	189	259	166	117	167	64	78	88	15	45	56
211672_s_at	1289	906	970	746	488	398	1695	1048	1025	903	1036	432	1298	789	318	771	775	884	910	360	590
211673_s_at	208	147	174	169	532	359	176	162	223	145	142	131	36	58	61	111	147	126	81	67	38
211674_x_at	12	10	19	21	25	26	16	18	17	17	6	19	4	6	6	4	3	5	10	3	2
211675_s_at	314	512	11	48	82	98	380	397	87	45	41	48	1	2	5	605	664	616	24	22	20
211676_s_at	667	690	3132	1141	506	739	613	740	1951	2851	502	496	337	451	340	691	649	756	2803	2045	3479
211677_x_at	53	135	113	58	447	520	54	135	171	152	41	72	26	81	97	8	21	56	76	73	67
211678_s_at	1446	1653	1465	2056	1359	1953	1252	1518	1659	1653	1731	2094	2379	2157	2766	2205	2518	1915	2714	3015	3584
211679_x_at	91	41	85	36	190	81	58	59	23	32	58	67	8	41	38	6	8	42	34	6	26
211680_at	56	13	34	40	34	9	24	13	11	32	3	25	8	2	13	2	1	20	1	17	15
211681_s_at	398	271	549	516	455	400	706	888	454	764	675	533	89	66	91	127	103	122	106	89	122
211682_x_at	45	15	30	19	170	211	34	25	20	32	24	29	29	7	34	37	37	58	79	34	22
211684_s_at	931	1268	2469	3262	3700	4384	1628	1956	2531	2500	4166	2860	5202	5324	7828	3169	3477	3497	5419	4461	5051
211685_s_at	147	109	137	164	203	151	180	135	159	129	213	170	140	154	108	39	60	22	34	7	17
211686_s_at	999	798	1318	776	800	809	1273	1021	1540	1273	861	883	700	947	1339	2000	2040	1831	2070	1275	1857
211687_x_at	19	28	49	62	76	39	29	26	52	42	81	55	5	4	6	4	20	9	7	28	1
211688_x_at	20	15	34	21	54	20	21	22	13	29	26	20	3	12	2	20	3	4	6	4	2
211689_s_at	16	6	23	402	23	51	9	9	20	130	18	23	4	3	5	1	1	2	10	39	15
211690_at	90	10	50	28	22	30	45	22	35	34	24	29	7	11	7	17	25	23	4	18	10
211691_x_at	184	208	90	233	793	667	163	226	225	295	163	217	42	76	94	128	166	48	31	91	96
211692_s_at	271	788	427	623	352	492	423	534	250	622	301	439	104	132	15	107	93	95	114	23	84

211693_at	149	173	144	85	1392	1351	152	215	167	138	202	134	75	74	103	65	97	84	186	59	86
211694_at	13	9	12	9	26	16	5	5	5	4	3	5	2	3	3	2	4	6	3	1	12
211695_x_at	112	75	179	870	63	91	136	174	203	138	178	156	23	6	49	20	17	6	41	61	60
211696_x_at	447	328	281	278	689	442	370	536	264	323	259	304	57	71	115	131	117	83	76	61	31
211697_x_at	1393	1215	1236	1151	815	1189	1683	1386	2174	1495	1676	1499	1413	1334	1275	1504	1202	706	1342	876	921
211698_at	1054	989	476	603	986	1673	221	703	324	671	746	900	2648	1415	1115	1547	1135	1479	2395	1678	2324
211699_x_at	53	18	220	23	206	140	42	44	52	137	53	103	95	54	237	92	10	30	214	144	75
211700_s_at	12	6	9	10	70	110	12	12	11	8	11	15	26	6	25	6	6	12	5	5	4
211701_s_at	50	13	42	92	88	191	52	69	25	45	149	15	15	5	37	2	5	41	26	4	8
211702_s_at	433	321	342	261	1281	1371	313	336	261	239	371	413	332	356	440	402	365	303	264	282	278
211703_s_at	937	1027	617	512	588	1076	586	643	397	546	519	496	684	626	567	985	1008	945	934	792	922
211704_s_at	209	196	293	406	337	564	122	144	359	184	190	271	251	156	182	154	97	51	339	231	254
211705_s_at	16	21	20	26	74	146	13	15	17	14	14	6	6	3	42	29	35	9	42	29	8
211706_s_at	8	17	18	39	18	13	15	51	16	18	30	29	7	3	4	4	6	8	6	7	5
211707_s_at	409	408	627	679	683	863	449	544	660	690	595	618	400	478	640	494	467	588	788	919	1096
211708_s_at	3907	1553	2268	879	422	149	1299	1664	1654	1121	1039	88	90	65	79	555	388	347	97	219	161
211709_s_at	335	423	183	416	309	297	264	326	198	405	280	503	288	191	393	305	201	235	139	145	64
211710_x_at	31318	27782	28205	28844	20706	25608	25318	26372	21920	20710	16482	23340	16333	21395	21685	24720	27036	25313	17142	25432	21485
211711_s_at	587	1024	551	1168	286	337	611	697	627	762	328	300	349	357	398	961	717	675	1213	1552	1652
211712_s_at	97	227	294	733	231	318	183	198	258	314	269	117	111	112	224	129	165	92	621	638	731
211713_x_at	495	227	920	407	1295	646	640	668	1176	1308	1074	1117	1000	1107	577	633	545	667	1504	640	550
211714_x_at	20261	5610	15994	3784	12787	7652	24407	19691	20078	12672	20052	16607	16122	18857	11880	9074	9192	9610	10228	5085	4404
211715_s_at	316	445	415	508	445	382	424	323	448	458	524	422	951	731	434	235	250	184	333	269	202
211716_x_at	5599	5783	5110	4104	3178	1637	5969	4813	5168	4745	5698	3444	1009	327	1460	1449	833	1350	1099	829	817
211717_at	1324	961	688	409	443	771	1569	1252	939	871	655	225	263	201	340	538	374	383	502	350	312
211718_at	165	57	133	80	27	157	144	38	130	43	122	40	20	18	33	3	40	21	27	11	3
211719_x_at	19	22	1259	113	1292	1265	341	356	785	25	932	1527	964	1096	1136	123	220	209	271	327	528

211720_x_at 36029 42090 31594 40472 24645 29366 36120 39288 37230 30416
23883 34294 19889 26685 27285 27748 29802 29813 17235 24994 21391
211721_s_at 159 136 171 182 209 35 143 189 186 215 118
102 150 114 59 76 78 103 236 109 174
211722_s_at 119 79 52 74 302 191 49 95 95 75 129
57 32 6 46 10 30 42 53 24 26
211724_x_at 757 704 746 660 1098 1160 558 537 860 707 1032
1117 829 682 1157 546 541 446 840 769 962
211725_s_at 1277 1786 1009 1095 1093 988 1157 792 1448 1180 1315
1009 1338 1298 979 1317 1094 1148 1397 644 504
211726_s_at 243 170 258 261 289 347 258 251 221 295 205
273 26 28 22 24 34 6 43 56 35
211727_s_at 2450 3469 2367 1858 2579 3096 2319 2382 2664 2943 3878
3352 5375 4086 4487 4053 3336 3343 5575 3025 3508
211728_s_at 200 163 251 215 206 189 174 120 286 232 169
145 52 44 23 52 90 40 110 97 72
211729_x_at 1504 928 484 675 2657 3479 1287 1185 468 400 2447
2179 3270 4119 5933 1546 1742 1316 481 658 470
211730_s_at 7824 9341 5922 5308 6570 3052 8664 9208 6607 7217
10507 11161 14111 10259 6323 5053 6235 5981 4367 4134 1823
211731_x_at 176 62 69 131 408 250 16 137 193 103 114
137 31 35 41 49 35 45 31 19 13
211732_x_at 32 33 137 384 65 120 53 54 206 162 63
11 7 7 4 2 1 11 176 164 165
211733_x_at 1509 2028 1686 3193 1432 3356 1158 1189 1532 1518 1128
1022 2461 1952 2556 4304 3434 3580 6663 4304 5401
211734_s_at 69 55 31 65 148 90 56 62 59 66 61
10 22 8 13 30 3 11 15 11 3
211735_x_at 20 16 22 44 29 19 21 20 23 45 28
34 5 5 8 17 6 6 6 5 3
211736_at 48 34 39 65 104 93 3 3 21 41 24
3 4 32 1 8 18 21 24 19 29
211737_x_at 132 79 52 128 48 74 98 95 82 137 74
90 11 23 10 23 38 23 31 47 16
211738_x_at 214 112 187 186 404 187 208 143 213 225 156
254 41 35 63 18 5 9 53 48 26
211739_x_at 9 9 9 10 19 26 8 7 5 7 7
14 4 2 7 2 6 2 2 12 3
211740_at 37 6 64 22 55 112 69 49 5 85 67
68 29 40 38 8 12 20 23 85 92
211741_x_at 116 83 7 47 32 527 94 88 110 71 193
78 129 12 153 5 3 23 31 9 3
211742_s_at 17 69 65 100 34 43 93 34 35 42 30
10 2 7 17 13 2 24 18 17 23
211743_s_at 58 72 23 35 48 31 24 78 75 34 34
58 5 3 10 5 37 24 8 24 14
211744_s_at 1013 631 1474 735 947 528 718 578 918 1159 1167
867 1478 2150 1755 1264 1446 1341 1187 1171 1097
211745_x_at 202 117 224 168 404 376 199 164 244 316 215
240 90 89 235 79 62 71 288 109 65
211746_x_at 9172 9203 7039 5559 9165 11577 8867 7330 5642 4783
10386 9840 11826 17330 15726 13061 14175 13480 10070 11273 9926
211747_s_at 3844 2536 4205 1620 5427 2270 3547 3291 3879 3480 5331
5824 12707 10339 11676 9085 7641 8705 10360 6353 8184

211748_x_at	216	153	83	259	125	121	102	327	198	91	234	415	280	196	269	115	17	90	79	62	129
211749_s_at	3179	1721	3112	3500	2458	1947	4563	5625	3659	4165	5270	4302	2220	3233	2893	3343	3586	3141	2958	2285	2694
211750_x_at	25030	14372	27399	7792	27712	17798	25513	26824	32572	21217	23721	34303	17807	23571	26236	16315	19530	21731	13618	12797	11330
211751_at	36	22	43	76	51	125	64	53	25	57	20	36	1	20	21	1	2	11	15	18	9
211752_s_at	1132	754	760	914	777	962	859	466	519	704	669	770	1417	972	677	662	692	701	746	1033	628
211753_s_at	146	122	75	92	235	269	106	116	104	115	81	124	27	15	27	14	12	2	15	11	14
211754_s_at	403	287	305	231	854	620	354	396	522	498	617	460	688	478	629	449	428	472	888	447	634
211755_s_at	12298	9592	11481	9795	8057	6506	10445	9464	10385	9010	8351	6822	11497	13046	11762	13225	14872	14657	13796	11319	11879
211756_at	46	64	125	116	89	63	46	26	83	60	71	35	37	51	42	11	5	11	73	44	104
211758_x_at	1045	572	2786	2624	3858	3794	1032	1008	2676	2741	3974	3226	4345	4929	6753	1465	1283	1077	5217	4343	4921
211759_x_at	2634	3736	2526	2448	1652	2092	3173	3199	3219	4039	3741	3622	4183	2439	1291	1057	1139	1023	1084	831	454
211760_s_at	97	109	119	125	143	155	95	104	43	75	84	129	63	43	79	106	99	102	62	104	138
211761_s_at	3011	2433	2168	1204	1333	1261	2877	2155	2460	1961	1590	1571	2705	3106	4491	5693	4750	5306	5317	3862	4803
211762_s_at	7944	2677	7673	1949	13436	4367	9708	9289	8290	5888	11119	13105	16916	14532	11161	8673	10110	9146	10747	5167	7884
211763_s_at	958	575	1095	887	1049	851	959	872	1032	1100	1101	1228	1369	1162	1217	1102	1524	1372	1644	1365	1855
211764_s_at	722	782	708	290	756	777	339	517	272	408	314	582	727	514	503	941	700	841	1391	1137	1360
211765_x_at	30430	25249	31018	32146	34293	28106	36015	35041	37017	29210	30776	32818	24226	31509	35988	30015	32669	32748	19086	27354	22138
211766_s_at	64	13	12	14	25	40	20	7	9	3	7	8	20	18	30	19	9	26	24	8	20
211767_at	693	182	362	128	536	16	453	658	429	516	620	584	347	253	181	127	148	129	271	137	66
211768_at	217	178	236	285	405	122	211	245	412	288	229	274	21	61	15	31	59	35	50	36	45
211769_x_at	1093	625	3038	3947	2229	2977	1352	1273	2174	3266	3616	3525	1651	2003	1481	544	723	484	871	1343	1290
211771_s_at	12	13	9	6	55	98	17	16	12	18	21	23	14	24	16	12	15	9	18	19	28
211772_x_at	22	16	20	26	29	26	20	28	27	34	21	20	41	46	6	5	11	13	40	24	7
211773_s_at	343	535	191	296	521	469	435	367	363	224	448	253	86	98	73	185	288	264	74	75	98
211774_s_at	321	282	563	295	109	42	412	351	502	356	240	376	135	118	179	291	187	249	492	328	272
211775_x_at	42	35	46	34	44	8	9	24	29	22	17	21	22	29	1	9	16	27	13	12	5
211776_s_at	33	2	18	87	36	98	101	21	50	61	48	16	14	18	28	27	7	28	12	17	18

211778_s_at	308	201	540	469	621	542	150	135	455	484	510	386	416	323	330	56	119	75	563	515	343
211779_x_at	2139	2510	1295	1746	1265	1697	2396	2074	1181	1512	1995	1708	965	1160	1459	1300	1346	1419	782	990	807
211780_x_at	1193	1195	1099	1211	858	1001	1365	1016	757	1089	1110	768	448	579	818	740	737	667	812	520	624
211781_x_at	128	23	130	98	32	30	16	193	139	200	202	29	6	20	11	23	10	40	4	28	46
211782_at	52	22	62	75	21	83	50	58	88	96	71	112	54	58	44	16	38	18	67	26	44
211783_s_at	3618	4809	2622	1610	2073	1459	2256	2101	2331	1817	1842	1068	1087	861	1102	1522	1581	1337	1562	1329	1049
211784_s_at	4751	3454	3892	2334	6158	2579	3744	3771	4469	4129	4293	5468	8861	7263	5656	5536	4928	4776	7584	5831	6372
211786_at	9	15	4	65	40	274	13	17	9	80	90	92	29	3	11	2	3	16	10	3	6
211787_s_at	21252	10153	20785	10513	20152	11810	20094	18074	22192	17140	18450	13230	18796	23439	17857	14674	16951	17519	13853	16466	14367
211788_s_at	8	6	7	8	18	17	7	7	7	8	6	9	2	3	2	2	5	3	3	2	2
211789_s_at	87	157	248	491	11	9	225	177	123	176	159	53	1	2	1	2	1	32	17	1	2
211790_s_at	101	57	91	32	27	28	66	36	122	127	30	119	6	3	3	5	4	3	2	3	4
211791_s_at	82	137	149	123	29	28	152	164	174	106	17	142	29	38	14	10	42	33	11	7	18
211792_s_at	99	99	49	107	282	191	151	113	45	48	264	123	172	231	121	74	86	113	58	100	52
211793_s_at	913	793	966	675	1559	988	800	1132	1053	1343	1529	1263	667	578	642	403	323	335	409	441	403
211794_at	26	23	26	25	29	22	21	24	24	25	17	19	4	10	27	5	8	10	6	4	3
211795_s_at	32	49	12	25	55	106	60	86	59	11	40	69	11	5	13	17	33	29	25	13	4
211796_s_at	17	9	9	10	1600	603	11	13	12	7	730	518	1549	1144	1128	4	8	21	100	320	319
211797_s_at	486	482	585	613	639	810	562	476	467	531	472	407	557	571	269	287	300	300	340	289	276
211798_x_at	17	30	27	9	201	433	28	61	36	27	37	65	10	9	42	29	37	77	30	57	7
211799_x_at	745	2764	701	1062	1002	1533	926	1169	753	1010	2349	700	1177	789	324	270	351	474	218	257	304
211800_s_at	713	420	600	788	1122	1398	533	623	846	555	945	1124	1227	1179	1137	466	515	473	696	634	699
211801_x_at	332	337	907	665	844	1195	717	624	781	660	938	665	250	239	212	182	171	179	264	128	206
211802_x_at	28	32	65	44	307	112	57	45	44	45	40	39	16	22	32	20	24	21	22	11	10
211803_at	34	24	114	97	65	47	25	49	8	29	18	28	19	36	14	4	63	4	9	3	2
211804_s_at	929	662	713	380	772	263	999	1025	907	839	1171	1022	493	357	238	248	300	344	454	149	335
211805_s_at	58	12	43	22	333	86	42	117	48	43	43	35	32	39	70	22	32	56	76	44	11

211806_s_at	319	324	294	394	393	325	351	404	281	513	289
498	88	64	52	20	41	19	33	66	44		
211807_x_at	194	343	256	230	389	269	228	197	165	257	246
239	75	88	107	87	89	91	117	110	97		
211808_s_at	17	137	88	114	45	60	77	57	165	112	162
96	20	61	36	21	43	32	43	6	51		
211809_x_at	237	72	30	97	249	243	168	103	167	169	269
339	198	180	105	92	92	72	32	46	56		
211810_s_at	171	100	129	153	309	280	183	189	195	270	183
196	44	44	18	30	16	37	55	31	19		
211811_s_at	79	35	4	69	23	42	19	65	79	67	84
44	5	40	1	8	2	20	23	7	3		
211812_s_at	156	95	3	9	21	23	200	127	47	11	26
88	45	28	5	50	45	70	2	32	41		
211813_x_at	143	28	183	185	51	77	147	267	171	212	223
158	16	64	40	8	5	7	12	26	17		
211814_s_at	251	165	239	178	566	194	608	598	252	362	543
538	143	196	208	309	202	196	106	67	169		
211815_s_at	549	454	415	437	348	308	545	560	384	442	378
317	72	55	92	96	72	111	116	117	35		
211816_x_at	81	96	73	69	87	59	142	127	114	95	87
54	13	40	5	1	26	6	4	25	23		
211817_s_at	85	179	174	217	27	19	183	260	91	157	368
274	127	84	123	3	15	4	10	26	9		
211818_s_at	250	106	233	127	164	227	129	178	108	176	91
49	25	4	44	19	9	65	45	29	28		
211819_s_at	115	88	62	83	399	439	65	170	104	136	1160
760	67	35	29	21	28	16	32	16	27		
211820_x_at	50	15	24	13	34	184	25	16	16	31	14
13	4	5	19	2	2	6	4	17	5		
211821_x_at	9	6	27	13	32	40	27	8	7	13	10
29	11	4	3	4	5	6	13	8	4		
211822_s_at	53	46	45	57	158	60	122	102	74	129	45
59	5	6	3	8	6	8	8	6	6		
211823_s_at	568	484	1062	419	489	513	832	932	934	1300	1425
643	11	6	5	77	57	94	11	16	31		
211824_x_at	58	75	23	12	27	22	82	29	43	48	90
41	10	3	18	2	23	2	2	2	16		
211825_s_at	135	148	94	71	59	77	57	57	153	46	161
180	29	2	5	23	40	12	31	4	34		
211826_s_at	171	136	171	206	276	340	170	195	249	177	223
207	46	61	10	8	9	52	64	60	53		
211827_s_at	29	30	58	41	104	75	85	30	23	27	24
23	7	30	5	2	35	27	8	50	8		
211828_s_at	300	119	425	437	269	185	257	302	541	1073	452
259	283	301	163	168	153	73	122	105	90		
211829_s_at	69	29	72	106	16	15	24	28	36	20	102
13	13	4	37	2	2	3	3	9	12		
211830_s_at	54	39	33	75	366	125	42	41	50	45	34
41	3	17	8	8	6	7	10	9	9		
211831_s_at	30	49	64	38	56	78	23	37	16	21	24
29	11	3	8	26	6	2	6	14	9		
211832_s_at	16	12	72	22	25	28	28	206	147	138	16
97	2	4	6	3	3	4	4	16	15		

211833_s_at	406	547	640	466	484	316	333	264	768	832	691	525	736	661	438	256	185	228	264	216	76
211834_s_at	130	18	151	92	341	274	114	42	178	25	40	128	12	28	17	8	35	6	4	20	3
211835_at	15	11	14	18	19	31	16	13	12	14	11	13	5	7	3	3	3	2	4	6	3
211836_s_at	70	150	75	38	67	75	89	38	23	59	24	26	5	4	18	5	10	13	5	3	6
211837_s_at	33	10	34	30	372	509	87	32	43	64	85	39	7	35	44	70	36	21	61	44	47
211838_x_at	136	146	107	89	95	202	119	110	50	42	122	78	29	47	46	8	60	9	28	26	7
211839_s_at	17	16	53	27	80	78	27	13	21	18	18	26	17	2	21	13	7	9	29	7	7
211840_s_at	44	10	7	14	32	183	19	17	33	15	223	117	112	109	83	15	34	46	6	17	17
211841_s_at	34	266	12	43	56	36	33	92	19	32	13	19	11	18	4	43	65	12	20	12	44
211842_s_at	147	82	160	96	279	343	201	156	139	179	179	173	29	4	46	51	39	41	36	17	34
211843_x_at	33	23	210	402	225	280	42	26	297	546	234	234	63	91	27	6	8	5	144	142	155
211844_s_at	11	6	7	9	95	146	15	11	12	20	20	18	31	3	13	9	21	4	4	2	1
211845_at	7	7	9	6	43	67	7	7	5	6	6	10	5	8	5	2	8	4	3	2	3
211846_s_at	106	84	172	194	51	59	171	144	234	218	266	143	107	113	90	48	63	35	89	40	23
211848_s_at	22	10	12	12	92	34	21	11	31	14	16	20	8	16	7	20	11	7	13	7	25
211849_s_at	258	187	328	202	30	13	380	478	325	358	284	278	57	83	70	119	104	109	188	80	143
211851_x_at	353	147	520	154	555	391	493	596	597	579	553	520	168	190	146	105	88	113	221	116	145
211852_s_at	315	172	231	327	477	290	372	329	268	331	458	514	199	216	120	83	106	75	74	76	91
211855_s_at	26	21	209	190	203	134	199	284	248	289	416	405	509	277	274	120	113	132	219	205	201
211856_x_at	108	109	151	76	115	219	109	88	94	152	97	46	15	29	17	9	20	8	32	8	24
211858_x_at	12909	15631	12129	23685	8987	10737	11325	11908	13441	13030	14849	11341	10565	17870	21394	15720	17248	11781	12995	20605	17682
211861_x_at	46	19	27	25	102	39	9	7	9	6	21	82	1	9	7	1	2	9	4	2	3
211862_x_at	318	269	373	638	811	536	297	273	317	345	442	375	69	58	141	112	90	77	130	154	173
211863_x_at	353	406	389	485	228	238	417	509	379	401	362	339	53	60	55	64	43	43	85	3	45
211864_s_at	3524	5086	2832	3006	1451	3580	3370	4198	1577	2295	2102	2595	610	561	507	1443	1232	1226	913	1112	1393
211865_s_at	259	225	163	155	566	524	268	282	194	235	263	150	80	76	77	71	69	92	104	59	37
211866_x_at	269	339	281	362	205	348	458	449	349	344	274	373	30	79	21	48	16	5	41	48	4

211867_s_at	34	47	11	32	89	212	11	21	62	21	7	28	15	4	14	6	19	3	3	2	2
211868_x_at	79	13	94	62	21	203	168	70	91	147	74	20	35	50	61	34	34	22	34	47	43
211869_at	9	9	22	31	52	36	8	32	32	10	28	8	4	6	3	5	7	5	6	2	3
211870_s_at	21	19	23	18	47	56	13	29	33	24	17	14	2	4	7	27	5	13	3	17	19
211871_x_at	261	415	378	206	212	356	306	464	336	566	360	319	190	109	125	172	178	102	208	249	344
211872_s_at	49	62	37	105	56	47	143	293	142	285	40	185	16	52	9	12	72	3	10	45	31
211873_s_at	19	15	20	41	27	52	28	21	23	14	16	24	10	9	18	8	2	6	4	4	3
211874_s_at	241	208	385	405	142	388	347	396	535	579	338	325	74	45	13	38	32	61	83	74	101
211875_x_at	17	11	15	23	19	16	17	22	32	25	10	23	4	8	6	1	21	6	3	8	6
211876_x_at	9	15	8	17	56	137	23	15	8	8	112	14	4	2	23	15	5	4	5	1	1
211877_s_at	19	13	11	13	80	259	41	9	20	22	14	19	1	5	3	16	13	4	4	3	23
211878_at	24	9	8	13	63	42	8	59	11	31	7	4	1	15	3	2	3	2	3	4	3
211879_x_at	180	135	172	40	154	230	38	113	127	29	53	35	2	21	19	2	2	2	4	3	15
211880_x_at	177	157	202	303	283	337	156	199	198	180	182	114	22	29	39	18	50	11	29	21	15
211881_x_at	157	92	151	72	27	567	135	127	193	122	126	166	6	51	6	5	7	20	6	73	11
211882_x_at	20	94	83	39	48	40	17	46	51	15	31	25	6	8	10	8	7	6	5	9	4
211883_x_at	198	197	543	440	333	152	131	274	672	858	311	318	27	45	40	13	23	38	156	127	183
211884_s_at	64	43	117	177	43	43	38	96	100	127	124	137	12	3	19	3	3	3	4	3	3
211885_x_at	26	99	117	30	60	46	174	38	40	34	23	20	6	24	13	27	10	24	2	11	5
211886_s_at	103	80	208	199	455	611	42	178	134	194	175	74	7	48	31	36	17	21	33	39	27
211887_x_at	42	46	85	41	34	26	85	51	74	85	80	99	11	34	21	40	6	4	20	1	23
211888_x_at	156	56	161	253	43	67	213	290	203	204	192	146	8	8	13	20	8	8	17	9	22
211889_x_at	82	134	248	257	49	30	107	179	384	450	176	185	37	36	28	36	9	56	143	97	153
211890_x_at	9	12	8	10	26	52	12	9	11	15	6	9	11	17	2	44	6	17	4	2	3
211891_s_at	206	270	132	131	181	202	240	224	123	88	240	227	10	6	36	19	30	11	32	29	17
211892_s_at	111	61	77	91	271	22	122	111	140	96	97	141	40	39	33	43	19	16	21	14	28
211893_x_at	34	46	45	72	135	101	21	45	88	116	61	49	8	3	24	6	8	4	8	10	5

211894_x_at	262	248	283	286	85	67	240	238	288	247	193	312	6	25	10	39	10	33	15	31	25
211896_s_at	126	11	94	53	144	199	45	73	67	95	13	40	15	15	24	10	20	22	17	5	4
211897_s_at	70	24	14	39	14	20	45	24	31	127	44	55	2	5	14	5	24	3	3	15	3
211898_s_at	22	7	7	17	19	38	9	8	7	10	11	15	7	4	3	2	5	1	4	2	2
211899_s_at	807	993	865	828	260	242	963	887	824	1346	721	209	196	133	70	107	54	82	111	56	73
211900_x_at	212	147	72	72	132	185	68	154	186	60	68	145	8	31	50	33	16	24	16	49	19
211901_s_at	83	90	41	36	44	196	89	22	25	10	9	36	5	4	5	5	11	23	6	5	2
211902_x_at	213	74	125	123	253	211	209	219	190	123	111	114	9	65	43	27	9	58	17	29	20
211903_s_at	66	12	26	59	52	59	97	104	5	85	20	64	23	43	42	12	2	5	19	31	31
211904_x_at	181	106	107	81	447	441	148	235	116	179	128	127	53	55	40	62	45	70	46	26	52
211905_s_at	448	254	925	912	612	559	772	1305	717	3576	976	533	32	18	14	38	52	42	43	46	27
211906_s_at	11	2	18	69	8	19	73	18	47	38	17	6	9	2	11	5	6	1	1	19	1
211907_s_at	220	22	15	97	249	55	69	141	19	29	146	137	20	14	32	15	27	42	25	4	24
211908_x_at	37	30	56	31	52	71	17	29	21	28	23	46	16	9	26	26	25	10	11	9	33
211909_x_at	30	13	27	12	441	812	8	5	41	8	44	6	7	43	25	64	43	39	60	25	34
211910_at	30	22	30	40	22	38	32	32	29	18	50	25	2	3	2	2	5	2	2	3	3
211911_x_at	4517	13173	1671	3052	1915	2143	4659	4365	1311	2270	5618	3824	3246	2026	574	1082	1553	2679	410	616	610
211912_at	86	9	15	67	177	38	159	80	62	95	67	171	14	6	3	1	5	28	32	15	13
211913_s_at	197	170	179	212	363	481	200	203	190	141	178	204	42	26	29	13	60	22	32	55	84
211914_x_at	94	88	202	410	43	54	167	111	399	464	316	290	19	46	38	21	6	2	37	32	36
211915_s_at	8	7	7	9	312	125	9	8	8	7	7	9	5	8	8	9	8	12	8	6	1
211916_s_at	278	210	288	252	243	185	220	269	257	256	250	220	25	19	41	7	33	46	59	38	42
211917_s_at	75	51	109	81	137	117	106	73	123	113	63	79	1	45	30	34	3	3	27	17	27
211918_x_at	499	353	505	501	959	826	326	344	542	489	506	377	82	59	135	101	83	67	74	104	92
211919_s_at	288	173	1453	1000	1988	654	277	325	2355	6756	1923	1560	2339	2255	1020	89	86	79	1736	1764	3317
211920_at	15	39	19	66	268	52	8	13	12	14	13	20	3	12	10	8	20	23	14	14	3
211921_x_at	21645	22492	16026	8446	12537	4116	18757	22416	17464	17722	13825	16612	19993	17457	8748	12838	11944	14254	10479	11808	7894

211922_s_at	1064	257	857	2768	47	411	1277	1453	1276	1437	543	848	299	431	256	465	529	612	726	436	672
211923_s_at	11	7	62	10	21	5	9	100	43	53	67	16	6	6	3	2	1	16	8	26	15
211924_s_at	677	1393	604	272	854	442	840	740	284	1115	1053	755	484	323	238	287	445	701	188	305	225
211925_s_at	32	10	19	39	41	43	8	61	15	52	17	24	16	4	3	8	4	5	29	25	2
211926_s_at	1672	2800	2336	4305	2377	1594	1664	1888	2399	3270	3363	3222	646	380	531	477	460	328	770	630	478
211927_x_at	25021	30809	27505	33225	22948	26749	26373	25969	28709	25656	24230	34425	18490	22886	23445	23434	24654	22864	15412	21643	19022
211928_at	3403	3656	1808	1507	2678	4229	1813	2113	1438	1254	3502	2003	1296	1585	964	986	870	1583	606	1241	744
211929_at	1379	922	1053	670	1585	1160	734	1244	708	976	921	1171	3184	3081	3733	2747	2325	3181	4211	3364	4445
211930_at	414	268	578	694	1127	638	727	892	791	936	713	1261	1225	716	258	156	171	287	478	375	779
211931_s_at	3721	1811	3185	1207	6566	2501	5528	5512	4645	4904	3129	4659	6949	4593	2765	4330	3707	4270	4747	2304	2024
211932_at	1386	850	1167	464	1805	626	1663	1985	1124	1630	1151	1594	2307	1699	1606	2541	2118	2544	2372	1439	1742
211933_s_at	2671	2244	3378	1904	4298	2114	4961	5158	3887	4319	3691	4167	4939	3991	5058	4994	4662	4978	4905	3209	3453
211934_x_at	3785	2175	5073	5191	2981	2588	5247	5621	5016	7368	3938	3528	1801	1567	718	745	1111	1006	1129	1151	1204
211935_at	2518	1375	3749	2894	6115	3182	2833	5105	4034	5407	3925	4818	5948	7331	7146	7859	6750	8592	7987	4336	7332
211936_at	19007	14625	12547	4529	10100	22251	11253	7181	8894	9143	7241	8618	10656	13593	11269	21582	22596	24766	8541	18202	20881
211937_at	3520	4924	6911	7743	5018	5823	8542	8839	7376	6390	6313	4808	3748	3798	4773	5007	4397	2078	3910	5359	3671
211938_at	3008	2993	3786	3656	4508	4823	4649	4558	3947	3549	3995	3995	4633	4263	5318	6345	5782	4022	4227	5441	4597
211939_x_at	14085	15243	16230	17234	11150	10022	16038	16723	15882	15336	14668	12981	15578	18469	18616	19571	20582	20580	15684	17888	15945
211940_x_at	11549	14542	18758	26878	14897	11236	12851	16899	18197	23801	15244	24420	15837	19353	20152	15901	17413	17758	16722	20838	17964
211941_s_at	6248	7155	7414	4492	3691	3532	5818	5327	6090	6766	4811	3498	5615	4122	2692	3693	3869	3372	2928	2430	2298
211942_x_at	21483	26226	20523	29261	12288	19858	21906	22376	22225	21306	15885	23539	17166	16774	16495	14717	12703	14011	10383	16361	14729
211943_x_at	23256	27230	23906	24977	29270	24649	20153	24118	20684	23248	19743	28888	20792	27427	24267	27878	27107	36594	16459	24120	22061
211944_at	165	195	312	370	126	106	412	439	456	375	259	298	390	347	140	189	162	183	253	203	151
211945_s_at	9679	10013	10926	8829	4111	7063	6082	7694	7197	12577	4771	3785	5828	7476	5711	12254	11758	10990	10265	13222	13364
211946_s_at	2058	2017	2524	2356	2915	1986	2398	2378	3349	2985	2049	1470	2128	1803	2524	3904	2397	4305	6506	5903	7280
211947_s_at	25	27	202	30	33	15	107	235	282	277	119	14	65	91	72	138	74	93	101	144	88
211948_x_at	636	764	1110	1111	120	368	1730	1824	1436	1561	918	951	537	355	299	546	542	437	864	893	470

211949_s_at	1023	303	975	503	612	544	1424	1255	1818	1312	867	647	266	178	259	284	218	316	259	123	180
211950_at	1976	1463	2356	1698	1288	1786	1695	1925	2115	1860	1973	1159	516	574	549	821	816	1013	1385	2029	1201
211951_at	4966	3819	4697	1516	3619	2362	5304	4884	6125	5979	3517	3706	5060	4187	6685	9077	9722	10280	8069	8040	8855
211952_at	483	382	629	233	506	386	515	520	442	499	426	375	474	401	293	378	385	307	482	375	596
211953_s_at	1814	864	1846	654	2010	939	1599	1599	2008	1570	1987	1519	2639	1974	2891	2101	2235	2299	4104	2749	4518
211954_s_at	2843	1934	3314	1167	3476	2012	3905	3024	3770	3076	3647	3026	3187	2160	2426	1961	2187	1974	3585	3340	4756
211955_at	1885	1201	2196	903	2455	1293	2992	2833	3013	2444	3056	2398	2487	2608	3512	2764	3111	1755	2759	3300	4481
211956_s_at	22117	26954	23388	19417	17799	24563	18740	17824	15581	13073	17646	18430	20372	22005	24131	24610	26931	27260	14899	26913	23238
211958_at	208	147	194	208	301	345	211	198	221	221	158	172	29	4	15	25	10	19	30	14	41
211959_at	7	12	4	6	115	26	42	15	51	13	43	8	1	2	2	1	31	8	36	20	17
211960_s_at	2039	1119	1991	1758	1563	1981	1658	1217	2192	2361	1558	1398	2679	2790	2178	2101	1654	1760	4201	3002	2700
211961_s_at	2381	2721	4168	4798	2366	3296	2143	2092	3501	3986	2788	3093	3484	3148	2102	2724	1940	1917	4561	4602	5373
211962_s_at	3609	3977	1818	2492	4570	4630	2507	3274	941	802	4013	4497	5929	4263	3150	3817	3489	4218	2630	2179	3019
211963_s_at	3784	5524	3793	5969	2789	3303	3800	4062	4331	5358	3365	3435	2999	3596	4044	4513	4229	4238	5656	4646	5551
211964_at	115	27	252	896	904	1080	110	107	373	356	1485	400	1405	1470	1149	1	21	28	653	668	518
211965_at	564	539	529	488	621	731	326	360	207	254	937	586	199	113	101	52	26	84	102	82	76
211966_at	36	91	220	288	247	356	167	57	318	225	685	222	669	425	186	19	34	20	112	100	82
211967_at	3241	3508	5279	5482	3539	5245	2475	3316	5970	6340	3826	3730	8151	7724	7292	7701	6384	7459	13957	15121	18884
211968_s_at	14862	9000	13791	6164	25774	14066	20275	19807	18362	10444	17181	18306	17355	16291	14953	12083	12805	13459	10476	5161	5870
211969_at	19644	13767	13449	7038	24011	13480	18601	21062	12990	10254	17069	20270	22146	25573	22819	21795	20242	21948	16758	13403	11287
211970_x_at	37152	44789	32027	24336	31925	30947	37748	33544	31907	29721	27337	41176	21882	25968	30729	27878	29306	31745	16521	23759	21372
211971_s_at	3445	3269	5055	3167	3506	6227	5300	5884	4730	5051	3837	3212	4669	4313	5493	6485	6153	6409	5697	8146	9147
211972_x_at	29959	37496	25701	30532	20230	21574	30950	30627	28916	26855	20734	30899	17961	22780	23511	28282	28831	27308	16858	24194	20129
211973_at	246	131	191	151	309	179	507	364	238	235	262	238	53	64	52	33	19	39	46	51	31
211974_x_at	2738	1635	1782	1785	2066	2005	2572	2800	1603	1599	1917	1441	1269	1375	1138	3425	3491	2614	1768	1897	2251
211975_at	1087	751	1526	1564	1196	1033	966	1114	1429	1997	1438	843	493	593	507	418	454	548	547	473	405
211976_at	530	781	534	773	994	693	1026	1049	751	848	854	550	486	644	529	558	489	345	434	497	391

211977_at	509	311	473	347	653	539	649	542	522	674	748	701	366	271	278	192	250	179	290	211	280
211978_x_at	31403	28986	34598	33951	36047	31286	39730	37695	38327	34626	34976	39582	24230	32623	35181	29326	32418	32643	19448	26123	21451
211979_at	820	620	404	656	858	1294	368	519	483	470	483	390	263	242	241	286	324	288	299	317	373
211980_at	89	94	58	84	98	161	98	143	103	74	64	78	23	29	42	30	8	45	41	36	21
211981_at	21	17	23	18	21	26	24	34	28	29	26	15	5	6	5	2	2	3	5	4	5
211982_x_at	2343	3484	2523	1286	1094	1154	2622	2342	2800	2106	1907	1341	891	924	712	1085	1185	1457	1581	775	1051
211983_x_at	37386	47563	34862	24183	32591	28507	35208	35276	31006	29888	26119	44893	22522	26283	30117	29031	30150	30624	18543	24589	24415
211984_at	528	684	1070	1977	1770	779	1252	1209	1231	1822	2355	2878	2420	3078	3005	1647	1618	1283	2076	1637	1902
211985_s_at	517	428	1013	1556	1273	806	1193	1042	1140	1948	2089	2576	1799	1741	1414	808	818	562	1480	1206	1436
211986_at	2413	1455	5953	9804	3012	2979	1985	2440	5211	6221	5287	3240	1026	1456	769	409	444	358	1268	1024	556
211987_at	996	769	1671	1000	1072	734	1002	1635	1056	1198	1143	571	1269	1230	1049	2031	1560	1758	2985	2665	3436
211988_at	2834	2860	990	1855	2507	1881	3388	3901	1401	1117	1917	2212	3118	3233	3189	6573	5752	5014	3660	3182	5507
211989_at	1382	1108	803	731	3060	1869	2412	2289	1007	709	1467	1622	1963	1748	1194	1956	2171	1756	1499	1238	1297
211990_at	77	152	103	8	125	101	114	84	78	74	78	112	6	3	5	136	114	135	41	46	24
211991_s_at	20	26	15	17	77	164	57	61	13	15	43	18	19	13	11	10	34	25	20	6	3
211992_at	433	292	903	1141	859	829	570	845	823	1016	1337	1311	1005	497	522	340	251	283	695	708	525
211993_at	45	35	126	131	408	282	94	113	226	184	163	123	75	82	90	58	56	48	58	102	53
211994_at	897	929	1762	1659	1832	2194	1285	1128	1500	1737	1887	1836	1174	819	837	425	416	350	648	847	667
211995_x_at	36747	46383	35211	27063	35487	28860	36128	37736	34487	34422	29875	46075	21750	28258	31092	30595	31878	35764	17161	24251	22817
211996_s_at	1234	1133	1674	2178	2302	2540	1230	1277	1960	1919	836	382	710	777	796	938	833	858	1565	2194	2144
211997_x_at	24603	16256	18214	15596	22134	14802	22890	24415	20711	20626	21054	24331	17909	20070	9214	9351	9526	11747	7117	6288	5425
211998_at	397	184	483	766	709	458	460	612	878	978	1340	1403	2058	2428	1748	1298	1430	963	1752	1115	1738
211999_at	11561	9217	9536	7461	10302	7521	8072	13082	10184	10664	11574	11257	14399	14870	19566	17358	15628	16168	13369	10722	12638
212000_at	135	123	148	177	392	228	127	158	98	198	183	138	214	148	106	2	4	9	80	65	56
212001_at	523	697	422	449	2044	1869	669	635	598	755	671	689	269	388	423	138	196	259	323	256	155
212002_at	1240	1268	678	925	427	360	1341	1549	824	1142	1447	666	365	431	388	212	321	313	138	205	82
212003_at	605	568	564	305	67	69	864	1107	755	512	944	559	246	169	48	99	116	154	89	71	42

212004_at	1390	1462	899	773	1116	793	1166	1230	1062	1043	1138
	1328	568	506	366	488	334	546	275	177	136	
212005_at	1119	897	438	665	383	499	1265	1238	744	546	642
	468	473	302	101	146	113	136	109	17	7	
212006_at	1151	1000	1438	1402	1700	2233	1610	1455	1168	1079	1762
	1563	2968	2211	1445	1471	1307	968	1869	1472	1916	
212007_at	593	645	746	784	462	558	872	1089	793	936	1387
	1430	2036	1859	821	710	622	388	372	709	349	
212008_at	949	771	1681	1712	1489	1352	1412	1882	1476	1646	2393
	1534	517	453	401	445	357	450	458	362	646	
212009_s_at	2945	158	2642	1121	2034	1591	2614	1611	3287	3016	2749
	1568	2168	1886	1032	1116	1248	1202	1470	530	784	
212010_s_at	2062	3317	4417	3730	3377	5412	1958	2214	4038	3343	3544
	3851	6979	5254	4444	4197	3617	4810	7831	11035	11299	
212012_at	753	1088	47	188	49	32	570	548	130	117	75
	65	10	8	38	786	1049	776	47	76	85	
212013_at	693	664	64	56	158	89	522	464	84	21	13
	16	4	23	22	273	341	184	3	22	23	
212014_x_at	3122	2494	4451	1955	1095	2510	3894	5935	4156	6025	2611
	2129	833	1013	1051	1859	1915	1310	2468	1844	1473	
212015_x_at	7033	4108	4860	2358	3831	1743	5309	5169	4280	4510	6006
	3305	4957	7148	9079	8669	8851	7786	6705	6854	3624	
212016_s_at	2346	1555	2235	1205	1148	958	3151	2509	2497	2246	2665
	1245	283	419	229	381	436	576	355	238	239	
212017_at	2064	1438	2170	1446	1526	1244	2524	1752	2170	3044	2102
	2066	1894	1684	1120	1146	1284	1093	1415	1093	841	
212018_s_at	3004	1590	2843	1371	1867	1449	2336	2103	2838	2556	1835
	2005	3371	2822	2406	3904	3817	2891	4154	4153	4120	
212019_at	212	68	256	219	62	218	274	301	136	253	304
	180	196	162	282	220	253	168	204	481	377	
212020_s_at	1036	185	973	338	1123	208	1314	1462	1453	1624	1263
	1178	326	303	266	262	264	284	555	225	195	
212021_s_at	2854	372	2409	507	3027	384	2934	3266	3269	3315	3815
	1958	1311	1082	1011	710	654	1053	1029	515	553	
212022_s_at	2315	349	1890	307	3038	171	2418	2341	2897	1934	2877
	1596	1495	1230	1280	754	605	1176	1507	628	709	
212023_s_at	693	135	853	278	871	206	787	1041	911	1388	1040
	704	317	292	269	126	152	219	641	216	185	
212024_x_at	1447	2427	1282	1208	1324	1588	1173	1483	1282	1082	2322
	2280	2207	2011	1175	946	1069	657	1129	1128	1016	
212025_s_at	743	1178	857	667	1311	1845	807	858	775	854	1833
	841	790	665	425	310	257	228	341	323	283	
212026_s_at	151	375	179	288	15	443	256	296	194	141	377
	362	156	170	129	114	129	182	175	107	161	
212027_at	188	140	260	225	569	439	318	488	337	404	337
	474	1313	552	251	388	311	442	807	578	595	
212028_at	673	223	471	490	737	353	1113	1093	1216	855	708
	608	1859	1354	452	677	525	504	503	345	257	
212029_s_at	17	6	11	9	188	90	8	8	13	14	7
	8	6	8	5	27	8	8	27	18	11	
212030_at	578	376	638	384	720	376	852	797	733	734	674
	563	695	536	409	404	421	479	666	422	579	
212031_at	1315	888	1432	1076	1425	550	2039	1727	1812	1922	1831
	959	1532	1409	1614	1932	1546	2092	1682	816	1304	

212032_s_at 1750 1659 1862 2713 49 300 1728 1795 1208 1062 1492
485 554 735 516 526 539 487 524 383 198
212033_at 1438 1018 2069 1458 1922 1097 2034 1962 1282 2074 1423
1198 1896 1552 2360 2222 2245 2015 2924 1417 2135
212034_s_at 1655 2544 1008 1623 1394 1668 1729 1718 957 1398 1560
1108 1119 859 432 582 575 635 576 540 531
212035_s_at 873 918 616 954 315 396 1006 1110 694 769 814
524 119 188 66 155 173 146 100 128 76
212036_s_at 1169 1226 1762 1630 1647 918 2429 2175 3232 2159 1777
896 1824 1830 1644 1751 1967 1761 2491 1327 1922
212037_at 1614 1366 1328 956 1517 968 1847 1533 1325 1381 1185
949 1163 1069 1209 1036 1179 1384 1178 668 1073
212038_s_at 5212 2951 2977 2565 4935 3804 5119 5111 3602 4404 8139
6051 12183 10092 10901 7823 7638 8485 7141 4966 6833
212039_x_at 34357 39356 27174 24816 20094 39741 32248 32376 27529 26367
21800 27574 16564 18220 19458 26368 28637 24903 13694 21023 14885
212040_at 583 564 1441 2087 1054 1106 747 620 1576 2136 1529
978 787 696 539 220 207 199 968 579 1174
212041_at 2389 951 3261 4454 2419 2255 2381 1603 2238 2830 2636
2072 3389 3933 3281 2173 2192 2054 2724 4244 3548
212042_x_at 17660 19528 12287 13084 15643 23004 14676 17622 11610 11955
11152 15046 18158 18534 16886 24858 20971 24220 16757 21514 19142
212043_at 1963 2141 2948 5106 3222 3688 1467 1646 2897 4660 2881
2371 4081 4717 4610 2760 2293 2366 7506 5974 7235
212044_s_at 851 1255 1008 1035 844 754 1012 1218 1201 1126 978
465 240 557 1357 1480 602 1061 802 960 518
212045_at 1359 1199 534 115 261 551 812 924 454 890 534
239 188 208 135 381 392 414 196 219 326
212046_x_at 364 450 251 468 500 675 399 614 290 127 351
353 250 254 442 280 401 319 300 269 201
212047_s_at 1278 1201 1628 1803 1435 1209 1133 1156 1032 1924 2180
1254 739 763 1019 703 980 607 838 814 590
212048_s_at 5406 3538 3671 1086 2357 3654 5178 2049 2871 1751 1769
1474 1855 1769 4438 4935 4959 5160 2780 4648 5265
212049_at 766 815 1134 1243 948 1099 1036 1213 875 1129 794
1190 564 469 357 285 274 298 377 387 345
212050_at 1230 1864 1617 1943 999 1325 1326 2172 1500 1659 1575
1714 684 564 295 678 621 463 495 346 467
212051_at 701 1332 1050 1327 782 985 1349 1362 1480 2229 1431
824 809 892 1719 2156 2163 2422 2319 1502 2084
212052_s_at 1991 2898 1371 1693 1307 1455 1556 1738 1393 1540 1752
801 928 733 748 674 590 700 808 731 761
212053_at 1861 1862 1754 2862 1303 1287 1797 1614 2110 1959 1292
1201 740 559 821 955 1315 877 1694 1946 2140
212054_x_at 1081 1595 1206 1331 726 1433 1174 1279 1319 1630 955
961 433 348 431 406 511 374 582 474 362
212055_at 1599 982 1412 1639 495 337 1810 1763 1911 1778 1049
892 865 870 1019 1518 1279 1696 2337 905 1340
212056_at 1136 1215 1253 1106 598 653 1595 1780 1342 1581 642
525 148 202 227 757 626 712 572 378 500
212057_at 609 752 1095 1136 190 292 1118 998 1513 1419 259
221 63 81 81 237 267 240 613 415 398
212058_at 2225 1344 4403 1856 4003 2837 3514 2671 5028 3825 4059
2251 4163 3729 5006 3723 3873 4751 7418 4487 7157

212059_s_at	500	624	624	594	476	404	513	454	516	595	787	434	349	540	200	281	274	374	379	292	378
212060_at	521	409	1012	370	789	650	596	635	1018	752	898	544	1678	893	1335	1620	1013	1507	3208	1343	2261
212061_at	302	293	685	609	630	562	578	523	1092	1119	674	525	1002	632	641	449	338	389	1201	987	1576
212062_at	459	1030	1762	6536	2003	3256	615	769	1573	1895	1701	1627	728	774	1076	551	641	617	2371	3454	4334
212063_at	5469	5040	6596	5504	2352	4447	5234	6878	7590	10040	3020	3999	2696	3896	3242	8634	8101	8134	10051	8726	10844
212064_x_at	1135	956	1267	1756	573	585	1642	1161	1361	1452	1167	470	318	252	439	368	594	431	806	753	493
212065_s_at	169	127	887	953	374	321	429	742	646	995	1006	498	110	122	70	120	110	155	266	179	252
212066_s_at	1033	1271	1758	2294	1886	2435	1340	1583	1615	1968	1730	1447	1034	1315	1767	1842	1812	1909	2007	2734	2837
212067_s_at	107	137	18	101	88	42	97	158	21	32	75	99	59	66	31	43	79	49	27	29	48
212068_s_at	1586	1628	1754	2208	1171	1302	1434	1872	1799	2150	1471	1462	606	276	250	881	398	390	544	1043	804
212069_s_at	587	532	623	759	485	446	674	672	665	690	786	599	257	139	199	135	169	179	259	237	149
212070_at	1042	1159	3097	3403	1263	2174	823	631	2352	2143	1582	636	1557	1215	2077	1346	1220	1454	2796	4022	4797
212071_s_at	4532	4770	7296	7277	6602	6325	4524	4285	5907	9009	8674	7006	6031	8626	6974	3217	3999	3561	6613	6190	5330
212072_s_at	2617	1473	1351	795	1953	1695	2316	2317	1381	1093	1885	1594	1964	1426	1843	2754	2661	2996	1450	1930	2414
212073_at	718	420	442	429	594	349	1230	1099	891	739	925	760	472	427	295	481	368	287	287	367	282
212074_at	2563	3299	1903	1428	3682	5738	1525	2448	1488	1867	2452	3523	2851	2823	1422	1739	1707	1491	1779	1341	1693
212075_s_at	1439	401	758	634	935	1336	2010	1475	989	883	1505	1156	827	796	877	832	767	856	471	607	642
212076_at	688	274	628	660	401	379	763	1027	764	823	915	682	166	172	78	86	90	134	133	203	180
212077_at	2422	2528	49	48	1489	2736	1427	1632	27	48	1397	1542	2468	1664	1673	4557	4022	4633	233	1062	1589
212078_s_at	339	258	441	343	234	301	543	596	462	344	551	376	167	148	72	57	32	38	52	106	92
212079_s_at	65	45	98	114	89	17	184	198	147	144	125	219	29	41	26	2	6	11	4	44	3
212080_at	777	713	1012	932	851	597	1040	1177	859	1020	1100	848	192	300	310	361	314	299	332	465	382
212081_x_at	935	1405	1023	964	416	383	1000	1519	1242	1203	1281	462	461	376	481	607	494	676	491	408	353
212082_s_at	7485	7149	9407	12224	16024	15793	6886	7731	7118	8366	10359	15897	15817	13870	7813	7646	7226	6055	8232	7949	5877
212083_at	1093	1315	2549	1319	2716	1782	1888	1440	2745	2368	3644	2825	2704	3671	4941	1799	1800	1935	2671	1713	2183
212084_at	269	151	277	113	390	422	386	325	381	457	689	358	221	116	106	111	127	151	134	90	94
212085_at	8595	12638	4655	3723	4870	7333	7553	9161	3398	4305	8730	7620	11891	10913	6803	6212	5736	3932	2976	5420	4164

212086_x_at	1494	1333	3063	3203	3891	2858	1536	1525	2966	4323	3186	2874	3790	2554	1442	726	855	445	2422	1494	1555
212087_s_at	847	529	935	842	794	836	906	878	1054	889	1163	602	642	1046	881	611	722	894	952	613	547
212088_at	1357	1352	1706	1403	863	900	1540	1403	2014	1767	1448	1136	1760	2001	643	662	703	632	838	656	531
212089_at	1756	1107	1381	1177	1735	1246	1503	1622	1742	1878	1686	1056	1172	959	559	562	248	414	746	509	238
212090_at	1910	3227	1183	1235	584	458	1966	1857	915	1083	1093	455	185	195	408	548	479	551	343	278	215
212091_s_at	124	423	22	13	19	30	448	314	16	17	367	163	75	70	62	162	142	37	3	12	5
212092_at	202	50	38	16	87	26	269	387	62	98	109	87	53	43	43	75	25	41	50	94	71
212093_s_at	716	1034	1127	1809	418	550	2377	2916	1810	1549	328	201	35	29	42	533	601	588	369	358	486
212094_at	287	157	23	72	12	30	143	351	23	32	7	18	19	18	6	140	109	102	45	192	181
212095_s_at	460	359	583	715	25	237	1333	1989	1042	878	144	158	54	73	100	920	985	722	582	711	859
212096_s_at	2401	5406	2579	4223	324	824	3298	4925	2838	2544	256	310	248	153	460	4457	4154	3363	2490	2157	2262
212097_at	4887	8279	2104	648	235	353	4373	4476	1754	2109	343	451	456	527	301	13809	12443	14946	6577	1672	2459
212098_at	1195	763	1438	2259	9932	6907	1232	1013	2194	1999	10787	11511	7354	7943	6514	491	532	485	1103	2011	1831
212099_at	3161	4051	2637	4158	7450	6941	2283	2334	1268	1856	10790	10692	3999	5478	6755	3347	4088	3076	1288	1354	954
212100_s_at	1496	1046	1293	1341	575	861	1754	1913	1432	1209	1151	794	584	711	489	688	776	690	659	486	454
212101_at	1012	844	688	1010	553	645	1287	1120	1230	1124	1232	651	723	675	1678	1209	1433	732	1522	1284	1069
212102_s_at	515	210	326	299	276	223	371	392	264	327	306	220	199	132	154	165	161	138	164	89	137
212103_at	1126	744	581	824	392	290	1280	1186	631	644	863	543	523	763	583	771	779	810	451	357	498
212104_s_at	923	1614	705	543	544	883	782	834	674	707	760	794	909	1032	714	1430	1423	1023	615	770	606
212105_s_at	249	12	400	180	418	317	841	1032	1090	822	816	571	32	40	41	23	28	38	46	4	28
212106_at	414	175	346	300	341	262	509	542	383	312	465	615	263	159	90	73	64	78	129	71	99
212107_s_at	742	73	741	521	1194	740	1185	1693	1357	1388	1097	948	124	67	108	125	88	106	142	78	126
212108_at	1189	619	1702	1236	2018	1842	1504	1244	1268	1303	1606	1697	2818	2020	1693	2339	1981	2314	2198	1855	2362
212109_at	318	243	562	552	636	590	436	358	915	1040	701	557	451	445	198	246	254	249	735	425	318
212110_at	3485	2486	4370	2519	3051	4760	2557	1936	3243	3822	2925	1860	3293	3771	5193	8213	9127	8778	6941	8145	8184
212111_at	513	468	373	882	559	1140	510	730	189	481	320	613	335	441	420	709	649	596	407	566	724
212112_s_at	532	866	509	839	613	1105	771	896	417	654	456	576	603	709	701	1074	1134	1080	800	834	943

212113_at	262	189	144	197	48	54	347	461	417	488	122	101	10	8	7	6	5	6	7	4	3
212114_at	1674	2411	2244	3564	999	875	2123	2780	2610	2875	1049	1078	457	330	460	1011	1076	1023	1323	1168	1346
212115_at	3211	2583	3274	3668	2378	3446	3426	2663	3533	3902	3309	2253	2806	791	1076	1034	1166	1211	4921	2887	3334
212116_at	1582	1133	1240	566	1251	974	1312	904	912	960	1187	912	887	748	1108	1246	1271	1491	822	854	888
212117_at	1074	1479	757	689	988	1871	943	1207	462	321	890	840	839	959	1812	1778	1720	1367	571	932	1119
212118_at	859	1152	788	772	734	994	791	1139	896	1038	902	798	446	430	359	1216	1411	1032	1113	1188	1536
212119_at	1458	1738	1135	675	899	2059	2168	1503	557	349	1352	1149	1268	799	1228	1498	1525	1062	575	1077	1189
212120_at	1757	2816	1244	1022	972	2677	2256	1804	991	484	1859	1090	1070	1064	2379	2526	3030	2687	822	1672	2351
212121_at	1404	1445	1423	1980	968	914	1821	2045	1294	1673	1198	1336	669	667	922	1686	1893	1446	1032	1214	1406
212122_at	86	220	87	113	95	324	103	117	98	35	105	103	162	152	277	338	338	287	180	297	492
212123_at	1320	1386	974	1529	767	990	1179	1292	831	1064	857	931	357	453	621	1288	1447	1336	962	735	1048
212124_at	1201	1218	1571	1653	554	668	673	1434	1644	2896	624	553	394	494	327	1301	1281	1259	1476	1672	1979
212125_at	1011	667	1187	314	779	445	1182	1037	1101	1132	1391	709	405	289	192	110	113	142	126	43	62
212126_at	831	770	993	1313	1045	703	1281	1741	830	1177	1639	1199	602	436	367	354	348	324	664	449	918
212127_at	1011	712	951	168	1215	242	753	691	970	734	868	674	242	133	194	110	104	279	269	143	103
212128_s_at	560	563	609	757	694	798	733	759	563	635	614	308	192	127	90	150	164	202	166	127	90
212129_at	2973	2226	3780	1908	1813	2061	2344	1925	3198	2984	2570	1589	3396	3641	5117	4255	4289	3792	6277	6464	6458
212130_x_at	12027	15028	13425	8737	9257	12701	11587	10789	8718	8292	9688	12501	11659	7620	7576	8526	8211	7085	6134	10657	9577
212131_at	958	1369	1617	2081	1908	1828	1068	1595	1976	2219	2356	2179	3618	2552	3650	2691	2377	2131	3961	3453	3452
212132_at	1585	1525	1420	1137	2822	2610	1138	1128	1386	1969	1522	1457	3151	1410	1362	1187	1483	1807	4944	3090	4575
212133_at	606	451	601	568	278	542	537	373	542	524	371	426	305	375	325	368	458	284	707	624	902
212134_at	290	376	463	485	1144	1310	500	441	270	741	583	631	265	330	196	130	145	128	229	111	117
212135_s_at	736	689	766	1685	403	249	1261	1213	629	987	603	638	89	88	63	268	259	238	179	140	192
212136_at	579	580	442	1092	298	391	722	885	365	686	526	485	87	159	71	309	217	271	283	176	189
212137_at	4730	2865	5395	4206	5690	7800	2951	2985	5829	4025	5379	4268	6642	6447	6793	4191	4157	4511	7504	7501	7372
212138_at	1549	1427	1529	920	1926	2239	2545	2154	2092	1729	1781	1546	1779	1242	2618	3608	2934	3100	3474	3150	3836
212139_at	2487	1685	1461	929	1538	1067	1758	1553	2135	1931	1003	809	1076	761	458	1420	1184	1475	2156	1404	1655

212140_at	1397	1309	1424	997	1656	1227	1313	1498	1322	1209	951	1146	789	587	708	1427	1121	1058	1553	1554	1781
212141_at	1942	1077	964	260	2102	282	2115	1783	1845	1371	2535	2136	2735	2334	2379	688	595	780	1930	820	904
212142_at	1278	680	571	255	532	70	1320	1481	1247	1071	1802	938	169	184	184	108	63	131	254	105	135
212143_s_at	40	150	43	27	2862	1770	99	91	33	64	3519	1419	968	991	275	17	16	19	2	8	5
212144_at	525	392	488	414	793	755	470	355	337	419	570	356	310	436	246	133	267	150	184	122	150
212145_at	3695	1798	2286	1542	1824	1630	2995	2311	2344	1936	1582	1205	1344	1325	1964	3556	3085	3099	3054	2028	3216
212146_at	382	423	208	154	85	73	266	422	161	211	391	244	174	271	233	169	300	176	163	174	129
212147_at	245	111	692	304	48	79	179	164	264	340	541	236	287	142	65	66	29	28	39	47	83
212148_at	409	485	174	748	118	172	1165	1174	312	275	77	29	20	1	38	1401	1729	1365	397	438	616
212149_at	3145	4584	5308	6110	1094	1656	2789	3968	3620	6142	1008	1091	1336	1174	1381	6050	6014	6082	7731	7104	8778
212150_at	1371	1720	2834	2933	652	626	1652	1957	1976	3082	468	513	369	424	506	1916	1934	1923	2746	2366	3094
212151_at	237	603	178	1251	465	430	1097	1180	367	239	58	93	77	41	42	332	501	395	401	335	479
212152_x_at	2159	2676	2134	1777	1625	962	2834	2512	2004	1652	1885	1271	439	384	336	520	538	655	410	448	344
212153_at	116	410	989	1200	408	667	839	1360	1286	1321	938	1082	351	218	183	363	454	435	368	396	472
212154_at	28	9	8	101	48	55	91	11	44	68	10	13	26	3	23	53	5	6	2	5	4
212155_at	2617	3403	1933	1059	1082	2909	2445	1495	1553	1382	1308	1504	1050	850	1659	2388	2342	1741	1152	2537	1593
212156_at	405	512	586	660	126	165	456	648	401	541	362	385	138	69	141	94	168	162	110	210	189
212157_at	197	179	149	177	38	34	142	234	268	189	193	180	17	8	31	12	19	24	31	16	23
212158_at	11	10	9	122	8	5	4	94	12	95	7	19	5	1	1	12	5	2	6	1	2
212159_x_at	2486	2176	977	1351	1320	1692	2515	1908	1025	1270	1714	1101	943	1079	1447	1241	1390	1443	817	900	846
212160_at	2293	2098	5115	885	2412	4871	2055	1267	2365	1486	1693	1205	2722	2282	3690	4488	4066	4160	5538	7112	8478
212161_at	351	303	292	343	298	160	378	271	371	444	330	470	93	72	31	76	36	61	69	43	68
212162_at	357	212	457	441	598	1122	428	636	373	505	601	459	446	346	115	120	136	126	161	91	163
212163_at	714	690	658	922	1009	1626	721	788	506	626	974	828	973	975	765	695	641	622	1179	813	1124
212164_at	486	404	275	469	312	527	511	573	320	361	217	304	141	148	111	411	408	298	281	241	312
212165_at	2006	1590	1801	2384	694	750	1981	1651	2097	1368	996	665	678	807	753	1313	1060	1081	1412	1123	1198
212166_at	851	350	1861	653	2177	1309	1488	1297	1968	1617	2565	2155	1974	1492	807	408	314	365	607	583	446

212167_s_at	1580	1328	1145	865	1370	1075	1998	1770	1385	1220	1353	1020	631	767	1026	987	934	1103	809	613	535
212168_at	2299	1577	1845	1408	2199	935	1901	1919	2511	1860	2153	1634	2514	2108	2222	2876	2122	2581	4533	1391	1743
212169_at	1512	1976	1053	2298	986	990	1927	1879	1312	1913	1516	1414	1684	1988	2702	1365	1760	1150	1733	2616	3049
212170_at	311	296	225	303	253	110	534	519	454	356	256	287	237	330	359	579	474	440	690	260	375
212171_x_at	865	909	1020	559	539	607	824	581	523	482	509	356	164	167	371	300	366	400	301	704	608
212172_at	131	95	168	135	228	200	170	157	194	250	129	122	28	35	4	6	3	41	43	46	37
212173_at	249	179	244	172	36	42	448	416	326	361	118	64	121	124	105	469	576	544	310	144	278
212174_at	1257	718	1443	797	432	469	1688	1310	1758	1276	762	515	491	441	369	1157	1300	1178	1052	860	988
212175_s_at	3028	1352	2608	1222	1177	982	3214	2321	2988	2099	1899	1206	1886	1597	1739	2659	2992	2876	2879	2158	2072
212176_at	646	1148	643	1203	311	390	659	831	935	797	355	167	376	332	238	1017	1183	927	2176	855	1303
212177_at	180	248	225	415	153	48	240	432	310	314	119	166	133	182	76	335	297	293	414	406	387
212178_s_at	1479	876	2071	1170	1542	1286	2290	2048	1940	2168	3166	1764	1392	686	291	231	231	289	321	255	287
212179_at	578	754	526	1491	230	340	354	607	516	619	217	240	184	207	190	515	606	352	773	686	1207
212180_at	1427	1049	2121	1677	1284	1200	1354	1367	2278	2362	1107	1325	1441	1272	989	1698	1769	1640	3069	2064	2605
212181_s_at	1543	1023	3468	4052	4548	2874	1577	1425	2403	2696	2344	3946	4882	2615	7394	2689	1521	1572	5867	7308	6453
212182_at	3	5	72	9	16	5	25	5	82	56	34	40	4	13	31	1	1	1	150	62	76
212183_at	126	135	680	1367	411	318	44	201	772	1062	172	216	856	302	530	491	533	440	2216	3035	3786
212184_s_at	706	921	431	484	752	751	831	907	379	629	789	613	846	832	665	1338	971	1178	750	846	1179
212185_x_at	30241	26179	8506	5961	16347	15880	25546	29325	6407	5557	17830	33319	16723	16933	13401	17289	17351	16050	5837	6804	3274
212186_at	2160	2792	4543	3038	1054	2048	1793	1929	4300	3737	1720	1278	967	727	1447	1366	1691	1483	2242	2984	2479
212187_x_at	157	57	52	242	117	103	64	143	217	143	246	83	303	213	221	74	77	68	48	54	51
212188_at	22	22	24	17	40	26	32	21	23	56	18	16	23	34	32	46	19	31	36	27	16
212189_s_at	918	438	583	711	577	404	656	569	467	492	489	705	575	544	457	308	411	523	586	751	941
212190_at	3646	3382	1875	947	1181	1170	3209	3514	1405	3217	688	1076	890	832	667	6056	6570	4722	2504	4283	4831
212191_x_at	25520	42961	17071	19670	12684	15652	20642	21806	14380	15287	13189	20765	17323	17976	12192	18760	19544	17629	10552	16850	8127
212192_at	61	91	58	70	12	8	118	132	40	81	26	16	4	2	7	112	65	58	81	16	47
212193_s_at	570	440	1717	838	532	433	828	705	1298	914	1735	630	545	386	849	133	138	123	904	822	543

212194_s_at	855	388	886	909	962	1223	1076	1264	1192	1220	1946	765	634	424	658	332	439	470	433	327	489
212195_at	609	740	1450	1579	2480	3778	549	682	854	1138	2072	2192	3481	3879	1864	1229	1281	1322	2495	3871	5854
212196_at	216	214	1149	1059	942	1047	505	596	662	980	1470	1323	341	478	402	152	197	240	361	693	783
212197_x_at	1980	1985	3139	1472	3258	2666	2520	1673	2802	2323	3300	3084	2268	2804	1993	1181	1538	1521	1688	1146	929
212198_s_at	786	573	765	1310	669	704	957	724	797	792	1295	804	452	492	322	366	287	457	400	383	391
212199_at	2727	1178	1285	1880	1736	1484	1566	2333	1188	1455	1563	1805	2050	1397	698	1450	1633	1210	1065	768	850
212200_at	491	477	472	356	624	422	317	364	324	498	532	388	518	320	212	145	161	229	642	332	434
212201_at	609	893	552	508	493	520	502	612	729	781	698	449	205	139	84	158	157	103	193	192	161
212202_s_at	806	1319	1222	1239	992	744	802	1111	803	917	748	549	1231	1520	1686	1913	1884	1778	1804	2228	3309
212203_x_at	5731	10468	1580	1551	4893	5506	7897	7330	1884	2627	12386	4316	9416	9993	12060	9667	12378	20440	2174	6667	4130
212204_at	1578	2299	1860	2428	1298	1431	1473	2134	1853	1828	1593	824	1909	2941	3035	3758	4397	4470	3182	4293	7103
212205_at	2391	923	1633	1367	3186	1404	2026	3439	1616	2664	3556	4264	5937	5870	5134	3530	3594	2835	4359	2793	3679
212206_s_at	798	251	587	513	1102	413	967	1500	562	1291	1428	1861	1536	1154	529	457	624	386	450	345	383
212207_at	306	193	197	213	341	234	325	230	72	187	340	226	449	272	76	245	260	185	209	144	141
212208_at	508	538	444	441	801	559	694	771	518	510	750	622	402	391	242	377	290	299	177	247	168
212209_at	785	1229	482	632	829	1115	604	909	455	493	1214	846	598	499	289	564	553	686	255	259	211
212210_at	179	134	104	89	573	617	281	296	191	219	259	123	82	5	22	38	3	31	13	11	13
212211_at	717	792	855	892	590	680	1556	1376	975	966	880	616	354	294	369	836	683	863	833	450	850
212212_s_at	851	838	617	433	1145	1009	967	1019	682	885	1003	584	712	694	641	460	384	497	455	345	345
212213_x_at	1135	1148	1351	777	1568	1443	1155	1243	1311	1167	1821	1384	1073	1010	1355	1141	958	1113	1473	956	1232
212214_at	549	286	902	631	977	1133	648	587	812	897	1211	964	803	749	870	525	421	456	1119	746	932
212215_at	610	575	827	813	1112	2944	510	629	660	695	715	1552	1452	845	1852	1166	940	851	1635	1664	2190
212216_at	324	473	1275	1489	663	1114	920	838	1097	1117	907	958	321	344	618	710	665	596	981	1106	1523
212217_at	443	420	894	1102	898	1246	1047	889	945	875	874	1415	489	265	336	554	562	349	563	666	929
212218_s_at	8041	6282	4988	2120	1340	555	6416	5736	4141	3280	3346	470	626	525	452	1271	1127	1474	804	1073	810
212219_at	826	888	1424	925	1346	1629	1552	1457	2008	1901	1954	1364	1175	1197	726	891	813	827	1262	964	1017
212220_at	235	111	652	322	444	560	741	698	872	836	843	378	54	97	89	122	91	111	156	94	113

212221_x_at	765	901	1668	1302	1227	1357	656	574	1009	1082	1330	1014	1393	1039	1304	1348	1491	1538	1806	3624	3001
212222_at	249	330	496	486	394	695	500	417	668	601	509	630	320	269	336	416	357	288	628	618	642
212223_at	628	860	2326	1124	713	875	673	868	821	1744	1829	1717	667	625	948	1076	703	871	789	1018	1357
212224_at	5	6	2462	14001	44	31	11	12	5269	6904	6	6	2	9	3	12	2	1	3391	1566	2409
212225_at	131	245	194	106	11	102	127	146	75	60	70	14	155	150	58	70	92	84	69	49	61
212226_s_at	271	496	300	394	191	177	379	438	290	287	355	285	219	189	185	248	188	195	139	313	264
212227_x_at	13097	15724	12728	9374	8851	12087	11600	11077	9214	8158	9556	12512	13228	8648	8203	8542	9108	7814	7083	12771	9953
212228_s_at	2348	2057	2081	2687	980	1101	2355	1684	1559	1649	1309	1066	1217	1024	1780	1767	1539	1768	1686	1482	1518
212229_s_at	870	768	1474	1514	790	989	1292	1669	1628	2685	1443	1687	1251	1041	716	513	406	517	1330	1053	1326
212230_at	230	343	243	163	265	301	338	296	231	193	270	248	189	312	314	322	376	362	316	392	498
212231_at	1733	1534	1531	1904	1276	1351	1411	1768	1952	2710	1504	1780	1111	947	1077	1728	1517	1288	2451	1868	2286
212232_at	534	774	998	1107	1041	1050	1182	1206	1092	1263	1192	1237	833	588	451	427	439	575	702	794	1283
212233_at	336	802	153	146	2169	3963	619	484	147	165	1673	1993	938	1644	1902	627	480	494	54	689	709
212234_at	296	446	662	1194	510	458	527	682	801	903	756	701	497	650	626	418	525	433	732	641	652
212235_at	49	212	61	27	55	59	42	50	134	43	112	25	84	121	20	105	157	81	111	77	70
212236_x_at	206	235	566	2953	278	308	276	304	139	340	277	344	108	153	168	72	118	212	468	505	456
212237_at	827	917	1373	2219	1024	1281	1000	1042	1691	1880	1822	1492	660	490	412	401	555	495	770	586	887
212238_at	474	410	663	938	426	421	594	676	637	636	991	645	486	636	577	306	336	307	484	435	487
212239_at	245	293	799	1320	522	577	352	476	1145	2109	388	421	400	423	610	551	391	405	2716	2498	2553
212240_s_at	171	152	492	1068	326	259	382	348	781	935	331	210	284	235	174	31	43	49	299	177	191
212241_at	701	725	544	517	400	396	647	813	630	920	669	843	185	196	212	253	165	250	253	204	312
212242_at	1234	1028	2523	438	5578	4027	1460	901	3734	1792	5520	4695	7542	5949	3560	733	738	831	3208	1504	1189
212243_at	623	669	798	673	370	606	886	1161	751	893	469	827	448	682	548	908	767	1032	879	708	1090
212244_at	795	953	1131	909	1058	1477	721	1032	804	1059	979	885	1040	793	652	2099	1767	2007	1291	1706	2179
212245_at	1945	1199	1635	829	2569	4105	934	1023	1195	983	1863	2966	5048	4176	2664	2447	3197	2827	3873	4392	6249
212246_at	1012	330	994	486	865	1203	1114	918	1104	1022	1722	1194	1141	1001	540	537	653	724	639	368	395
212247_at	915	577	2081	784	1016	427	974	1128	2622	1786	1457	1085	1314	761	919	855	694	841	2573	1220	2092

212248_at 1365 864 861 786 763 1601 1211 1371 858 682 1036
670 2354 1945 1796 4638 3407 4724 2739 6875 5693
212249_at 45 63 153 313 59 163 166 121 376 636 139
127 37 34 46 10 11 12 90 49 47
212250_at 2578 1972 1573 759 1739 2395 2447 1954 1414 749 1695
1203 2733 2494 3439 5669 4497 5336 3308 2984 4819
212251_at 2506 3094 1723 1148 1248 1943 3364 2889 1898 1481 1919
1460 4085 3423 3621 6359 6104 4818 3152 3861 3493
212252_at 800 668 674 704 612 559 670 771 954 946 605
447 208 187 277 286 308 265 418 284 233
212253_x_at 276 245 216 31 22 24 372 381 58 77 97
52 5 18 13 96 145 152 4 62 53
212254_s_at 2109 1686 941 671 481 409 1321 1969 737 1031 293
297 178 142 144 945 1317 1557 455 895 899
212255_s_at 984 1131 1207 1073 964 1410 670 874 963 962 756
850 550 438 314 700 594 575 1484 1185 1547
212256_at 904 717 1179 975 476 851 771 814 912 973 446
765 151 242 160 717 581 622 771 455 428
212257_s_at 171 102 263 294 234 136 236 323 413 383 425
298 61 36 24 51 10 3 46 27 31
212258_s_at 26 60 94 120 335 492 52 29 197 18 17
20 59 71 34 20 7 3 28 7 6
212259_s_at 114 127 49 939 26 40 36 61 127 215 54
69 60 64 61 29 76 60 16 109 68
212260_at 632 637 515 647 580 472 784 908 690 751 745
676 209 165 228 232 244 259 235 233 335
212261_at 896 802 757 943 1144 1430 933 961 768 854 785
782 302 276 362 371 338 374 431 500 456
212262_at 242 196 41 120 331 361 268 317 98 96 256
284 167 169 207 234 239 226 35 49 91
212263_at 500 411 15 14 426 318 376 368 7 15 377
636 533 374 210 356 352 253 24 50 74
212264_s_at 594 533 1004 678 727 468 1041 1012 1453 1108 1083
749 647 648 617 538 696 613 995 807 994
212265_at 685 636 43 16 785 1701 779 701 12 20 1106
1272 1642 1171 1893 2136 2041 1777 90 661 990
212266_s_at 2756 2924 2416 3595 3972 5019 3180 3439 3073 3002 3736
5100 5291 5678 3956 4910 4658 3912 3165 3636 3603
212267_at 523 572 909 868 947 805 762 805 1102 1143 748
824 924 755 642 1227 1033 1079 1834 1256 1629
212268_at 1337 1506 2938 3334 1034 1001 863 1031 2147 3180 1641
1567 1305 1605 2013 1738 1729 1428 4121 3589 4068
212269_s_at 699 1039 571 578 318 848 636 926 541 679 1239
901 881 510 446 200 278 288 311 360 320
212270_x_at 22225 30495 16498 22781 8589 12500 21611 20585 15751 13586 9873
12368 14323 17697 19745 26287 29239 27870 14294 22241 17016
212271_at 1644 2085 1333 1147 2162 1183 2344 2693 1749 1842 2635
1914 1393 707 1912 2210 1287 1876 1407 646 1205
212272_at 228 181 335 107 246 284 118 145 174 162 262
148 39 31 22 61 24 20 36 34 35
212273_x_at 15717 21132 17240 24788 15752 19823 16049 16606 17212 18264
18443 14807 13141 18671 23473 17897 21086 18180 12180 19197 15976
212274_at 778 384 775 171 958 856 217 318 425 334 783
501 585 634 641 497 622 489 340 1169 1335

212275_s_at	345	296	373	449	591	378	241	218	286	328	347	211	49	86	77	93	259	225	144	87	35
212276_at	591	388	1000	186	818	680	269	278	348	407	773	514	481	690	1026	578	720	753	514	1080	1523
212277_at	1214	1119	1304	722	915	816	1293	961	1044	911	836	626	857	785	1008	2281	2199	2093	1750	1410	2218
212278_x_at	16	7	15	10	19	22	19	22	23	11	9	6	3	4	7	3	9	7	6	4	5
212279_at	1898	920	2584	2279	1285	427	2394	2867	3413	3757	3306	2011	1886	857	655	765	690	612	883	693	638
212280_x_at	815	736	730	572	641	434	670	707	648	897	777	535	514	576	476	1111	1197	868	911	499	628
212281_s_at	2064	1114	2813	2007	2804	379	1949	2239	2981	2999	3245	2816	2994	2928	6059	5391	4972	5014	5856	4830	5800
212282_at	1797	991	2659	1918	2062	613	2337	2115	2735	2969	3208	2952	3088	3544	6651	6070	5967	5245	5753	4699	5048
212283_at	143	139	366	519	202	211	213	140	391	635	500	197	47	61	71	33	11	11	62	42	60
212284_x_at	33399	40275	28314	41564	34334	45988	29333	32722	29771	30571	28353	35718	24373	33966	39571	34434	38434	36835	20982	31192	27607
212285_s_at	1147	1796	5564	9166	3244	2521	969	988	3768	6098	4969	2644	651	760	1038	308	420	519	2236	1648	1382
212286_at	206	174	256	266	194	392	380	445	149	246	163	99	175	126	199	249	190	213	278	289	557
212287_at	1226	1178	4669	3422	2410	2067	1789	1779	4113	3912	2551	2296	3891	3275	3048	2055	1754	2534	8069	4557	6934
212288_at	585	1113	506	357	771	915	743	907	302	327	428	612	411	302	464	590	448	679	285	466	450
212289_at	116	99	178	235	147	359	73	203	123	182	165	112	153	173	121	241	160	103	218	219	365
212290_at	448	313	1065	392	411	376	665	418	1038	1129	932	659	264	384	169	96	86	104	131	215	114
212291_at	192	279	109	242	142	28	269	218	223	140	317	207	37	29	37	16	22	6	39	6	3
212292_at	198	122	345	107	268	766	333	123	405	351	357	241	76	61	45	13	11	15	48	59	87
212293_at	661	681	612	865	1384	1971	745	879	820	946	1207	1305	712	720	506	1177	402	1030	907	606	569
212294_at	1169	1411	2136	2527	1128	1282	1738	1957	2628	3846	1281	1572	1149	1540	1227	2566	2303	1975	2754	2566	3366
212295_s_at	4638	4136	5812	2296	4114	8610	3232	2014	5305	4720	4586	3381	4889	5509	10103	6954	7494	7718	7075	10694	10639
212296_at	7207	4825	5131	2605	6742	4953	7979	6215	5904	4528	8965	6432	8770	10745	11765	9842	9395	10261	7548	6355	7955
212297_at	1015	726	1450	551	2547	2615	587	769	858	938	1607	1418	2567	889	1038	1009	1118	885	2769	1943	2156
212298_at	501	219	651	184	491	155	319	123	302	147	325	331	350	383	239	23	78	28	93	167	165
212299_at	722	987	522	621	1726	1290	871	1157	312	840	2236	2874	1645	1135	1081	727	580	783	482	532	925
212300_at	587	681	734	585	715	523	523	537	729	918	890	992	480	540	379	261	318	262	398	365	297
212301_at	762	517	1039	1060	1625	808	579	769	1169	1652	1303	1584	1704	1195	1477	868	1001	992	2678	1554	2315

212302_at	442	185	905	603	737	345	451	549	839	957	726	676	230	237	267	210	247	149	334	214	176
212303_x_at	884	313	846	510	260	145	818	958	557	918	937	359	212	89	58	78	104	82	97	121	50
212305_s_at	815	580	514	970	482	625	908	591	507	618	605	696	99	113	119	228	254	209	189	391	370
212306_at	360	241	494	264	1034	751	453	534	431	500	843	620	637	469	683	308	358	344	567	365	836
212307_s_at	294	263	1040	1507	1167	1363	861	695	895	1262	1697	1178	712	615	353	189	196	368	404	320	485
212308_at	74	56	79	44	78	105	93	61	70	75	90	96	117	71	104	74	51	70	121	93	156
212309_at	70	62	345	106	555	391	196	293	258	260	233	402	271	321	369	283	259	252	281	292	400
212310_at	148	109	214	295	198	301	142	218	177	362	125	137	87	92	96	168	150	156	208	393	548
212311_at	925	852	1282	1699	810	1144	1648	1543	1209	2008	1428	730	355	370	505	509	528	475	479	722	870
212312_at	2744	2386	4478	2924	1796	1067	1325	2729	3006	4908	3843	3196	1806	2016	1327	1658	1506	1413	1372	937	919
212313_at	1114	899	545	420	931	816	822	809	407	524	549	540	318	400	347	475	474	533	368	213	195
212314_at	1835	2146	2202	3105	1611	3249	1655	2043	1399	2664	1796	1571	1419	2108	3831	2842	3884	3014	2481	4609	5286
212315_s_at	380	557	495	220	553	327	359	457	460	471	358	205	222	235	125	173	171	154	238	150	157
212316_at	1181	1489	1385	431	1179	708	1027	1386	1552	1596	931	596	272	309	274	308	255	355	475	305	340
212317_at	1048	856	1243	1355	2071	1282	1387	1057	1539	1455	1239	929	839	426	570	466	317	306	1041	728	314
212318_at	1914	1098	2806	2107	1842	1767	1289	1511	2305	2163	1521	1405	1673	1177	1908	2204	1358	1467	3920	2463	3034
212319_at	594	946	433	815	48	63	481	491	308	363	341	219	92	126	64	138	117	111	123	59	31
212320_at	11356	6475	6397	3088	8353	3635	12022	11818	6694	6096	8574	7742	5276	6986	5920	8087	7607	7607	6099	3114	3199
212321_at	1154	1154	1065	1567	1082	1472	1322	1495	771	1198	1239	1315	878	1125	488	1194	1363	980	796	958	1115
212322_at	1639	1834	1610	1238	1669	1664	1754	1961	823	1087	1647	1352	1607	1627	1353	1365	1765	1975	1729	1188	1869
212323_s_at	430	401	388	374	1047	716	627	611	288	401	544	446	134	220	203	202	339	186	130	175	96
212324_s_at	390	257	255	279	367	668	285	331	274	341	374	410	57	10	13	80	73	83	85	86	112
212325_at	119	331	252	393	221	391	174	212	270	204	145	98	35	94	86	98	72	92	214	111	143
212326_at	475	390	220	451	74	98	364	430	50	280	355	327	86	66	33	52	97	80	130	116	146
212327_at	93	483	273	654	169	210	160	149	345	366	159	207	110	152	155	178	185	179	859	619	1016
212328_at	111	429	134	369	121	372	196	166	191	273	131	202	98	72	55	23	43	24	288	323	295
212329_at	1099	1236	1186	1040	1199	1067	1269	1374	1041	1142	1300	801	734	681	418	375	488	397	456	333	278

212330_at	3751	2482	2880	1877	4779	1812	4180	3175	5001	3677	4883	3445	5060	5084	6353	5074	5660	6200	6468	4041	5714
212331_at	695	957	760	947	679	1193	227	789	359	1164	333	939	668	547	471	955	624	440	1484	1028	1214
212332_at	131	156	296	393	21	23	235	284	318	505	281	418	35	6	9	36	7	5	48	33	85
212333_at	1984	1513	1568	967	1603	1074	2479	1573	2273	1442	2322	1599	2307	2603	2901	2949	2899	2833	3068	2749	2709
212334_at	737	865	595	1553	701	1693	635	1116	654	952	739	681	1217	1227	1209	1296	1582	1161	1628	3092	2881
212335_at	970	869	632	1570	794	2228	702	1074	689	942	794	735	455	578	420	893	1028	877	701	1194	1512
212336_at	265	238	794	1412	517	642	409	354	850	753	934	256	142	151	277	220	170	123	332	244	213
212337_at	519	689	1413	2390	1161	742	1204	1686	1474	1775	1606	1198	576	780	507	354	334	391	595	651	933
212338_at	582	743	1240	1366	3609	3091	403	780	911	1736	2784	1812	1745	1105	1079	103	40	87	499	427	299
212339_at	217	298	445	1120	605	715	375	252	579	764	1117	357	278	356	360	139	149	130	285	199	135
212340_at	664	757	830	894	933	1132	900	670	839	1027	696	604	461	500	565	752	723	747	585	566	768
212341_at	800	1505	2059	2806	1641	2321	1011	1058	1853	2007	1511	1384	2274	2481	2647	3492	3488	1982	3599	4976	5155
212342_at	374	298	415	502	543	544	464	371	341	481	503	454	488	455	542	655	698	553	665	690	912
212343_at	323	174	730	663	357	431	253	285	656	1002	541	273	324	440	471	350	381	262	822	1090	1214
212344_at	97	58	27	125	282	266	24	108	140	84	23	15	1	26	24	19	14	23	10	41	2
212345_s_at	1086	922	1015	1172	904	1496	905	1134	729	668	840	856	600	385	455	1067	1072	989	707	1195	1813
212346_s_at	213	271	229	595	59	83	231	458	235	331	60	82	36	68	69	114	163	185	68	21	89
212347_x_at	319	437	370	713	111	78	408	412	302	274	193	53	55	70	8	154	236	185	104	180	227
212348_s_at	3301	1718	2051	1573	2007	1386	3531	2889	2250	1616	1900	1751	1079	1102	1182	2576	2547	2816	1984	1403	1744
212349_at	235	173	302	392	680	961	298	241	285	268	455	287	358	287	134	106	88	84	162	229	111
212350_at	949	1959	743	905	778	517	1104	1218	689	897	813	848	335	393	391	421	425	389	357	214	206
212351_at	885	841	602	529	1227	774	588	561	595	492	1019	750	877	885	612	446	451	496	624	482	401
212352_s_at	5607	3308	3538	3709	5337	8360	4170	4361	3766	4091	5011	4659	7299	7454	8716	11386	12335	10854	6323	7315	9519
212353_at	95	57	9	32	109	130	57	51	54	43	38	6	15	3	25	45	24	25	8	22	31
212354_at	16	72	88	127	124	164	34	20	32	98	85	88	10	12	13	6	5	6	9	57	7
212355_at	959	1035	875	1170	887	933	924	1285	888	1298	1123	1327	774	736	868	534	560	654	715	469	468
212356_at	242	670	189	597	309	125	317	418	136	345	465	351	160	207	277	297	300	202	230	279	348

212357_at	527	507	589	1137	565	500	588	479	800	773	760	550	271	339	208	277	322	237	362	367	302
212358_at	54	113	114	113	48	74	77	92	80	96	41	48	10	16	19	11	16	51	11	49	17
212359_s_at	566	925	433	1068	36	77	584	483	369	521	519	209	228	112	84	144	139	88	114	272	219
212360_at	1066	1282	406	332	700	555	973	728	741	546	752	371	326	452	415	579	692	644	359	297	324
212361_s_at	1172	1242	1785	1798	730	828	1238	1180	1457	1708	776	868	591	756	371	979	1454	907	1006	1287	1783
212362_at	308	208	298	287	246	194	359	413	503	552	249	288	29	36	34	54	9	60	46	50	66
212363_x_at	37369	45930	33282	25890	28237	27304	36414	37270	35045	32447	29318	40167	20257	22912	28494	26967	26862	27413	15840	21774	20361
212364_at	763	1221	1017	510	621	568	898	937	495	317	395	527	612	694	491	1254	1007	1173	1068	817	1002
212365_at	970	1063	1579	1097	794	1050	1847	1849	1304	1360	1508	975	394	416	642	1081	892	986	795	957	1253
212366_at	187	213	187	206	382	923	187	235	114	94	215	189	368	233	283	302	274	244	339	261	402
212367_at	454	401	553	542	606	695	461	561	317	581	360	268	360	347	350	695	508	546	613	491	636
212368_at	316	527	548	812	489	751	530	713	379	387	736	435	446	359	434	238	299	351	236	256	345
212369_at	1282	1291	1221	1110	865	755	1316	1461	1001	1076	1532	968	505	432	467	541	552	452	579	491	513
212370_x_at	1252	1184	1339	1833	1644	2086	1386	1364	1334	1228	1629	1414	1423	1354	2088	1078	873	977	1368	2066	2331
212371_at	2170	1948	2400	1320	1607	2211	2861	2518	2289	2087	2514	1928	2410	2110	3485	3949	3693	3144	3382	2625	3562
212372_at	2130	3238	1175	1812	2389	2301	2100	2341	1541	1379	1984	1798	2044	2148	2617	2760	2742	2579	2910	2929	3058
212373_at	197	178	301	177	221	230	241	383	304	263	206	226	157	208	140	368	210	333	447	307	323
212374_at	37	35	81	153	217	144	91	46	162	245	146	69	81	85	69	125	78	118	167	123	183
212375_at	578	1034	499	626	503	314	553	753	607	683	361	331	242	186	267	571	468	643	651	679	633
212376_s_at	729	782	651	454	998	687	737	822	688	647	597	618	141	98	100	111	154	142	200	147	183
212377_s_at	937	923	1229	1414	1255	1254	1008	1326	1133	1417	1461	1567	847	1001	1010	846	850	947	1483	852	928
212378_at	1356	775	1560	590	2830	1794	1431	1189	966	1196	2363	2495	2288	1992	1810	1336	1173	1291	1133	1221	1518
212379_at	1085	583	1259	733	1677	907	1259	982	1085	1181	1602	1948	658	618	888	633	485	576	396	368	638
212380_at	618	450	285	357	696	511	672	528	510	457	780	763	340	306	226	174	171	230	200	214	201
212381_at	315	283	600	370	1318	974	387	300	452	525	252	239	183	243	291	359	309	323	643	298	477
212382_at	36	16	45	47	181	121	16	50	28	27	64	25	32	24	3	13	39	20	29	38	12
212383_at	1049	1220	1234	1104	1271	1381	1226	1244	1268	1199	1339	674	351	378	417	397	461	440	432	420	488

212384_at	54	10	24	62	67	136	90	108	116	67	65	20	22	33	15	19	5	37	32	33	3
212385_at	37	22	73	18	88	82	19	18	68	96	74	59	20	14	9	5	3	4	45	5	43
212386_at	94	57	91	124	60	54	61	78	111	41	55	46	16	17	37	18	3	10	20	25	38
212387_at	130	18	141	181	220	214	86	25	95	77	74	35	4	5	44	44	36	9	37	29	5
212388_at	406	294	708	451	500	894	224	356	658	559	378	460	313	304	277	319	277	345	1171	954	1291
212389_at	17	29	19	18	137	297	24	36	25	28	242	15	11	7	9	8	11	7	6	6	6
212390_at	167	52	41	309	261	40	28	87	35	127	70	99	62	55	14	30	36	29	27	77	64
212391_x_at	31485	44428	30353	34430	30719	41699	34730	37522	28352	28137	28130	39775	25707	34921	36386	36079	37602	40250	22408	32170	28755
212392_s_at	157	141	209	102	151	125	199	135	245	352	257	172	4	9	5	24	35	39	18	14	21
212393_at	335	370	313	253	280	370	266	327	369	278	368	407	128	108	81	54	8	87	21	86	62
212394_at	204	232	141	122	30	106	260	189	142	173	145	127	71	83	125	242	135	121	193	274	229
212395_s_at	339	651	290	208	100	50	515	487	281	612	239	220	120	198	199	564	458	507	249	237	271
212396_s_at	1291	350	506	159	65	79	901	875	450	435	443	253	320	210	485	831	867	1229	550	285	348
212397_at	2324	2244	1225	786	2191	2010	3278	3113	955	495	2864	2060	2884	3119	3600	3414	3707	3642	1328	1275	1796
212398_at	496	376	487	354	819	942	1183	1379	570	442	1080	808	414	340	248	394	310	426	165	105	177
212399_s_at	672	1265	628	1006	737	1195	692	790	593	545	696	509	563	655	542	786	988	513	627	1138	934
212400_at	930	1178	973	1508	1020	816	1030	802	839	1138	1084	566	323	219	215	199	192	256	123	196	143
212401_s_at	1876	2033	966	1172	2029	1490	1370	1057	1235	875	1080	1090	1147	1017	866	980	863	891	996	833	614
212402_at	1040	572	882	735	2467	1856	1098	1333	1058	970	1771	1488	1632	1540	1689	966	927	1064	1469	1283	1816
212403_at	1169	1305	808	1523	755	701	546	784	447	1012	492	621	317	254	194	284	348	236	347	406	465
212404_s_at	262	417	269	432	129	245	318	540	199	558	304	225	30	11	6	47	29	49	19	23	34
212405_s_at	853	1034	773	741	909	657	986	685	1169	910	657	747	835	842	756	1199	1267	1113	1928	1379	1396
212406_s_at	642	816	1114	2430	1720	3131	877	1178	1354	1878	1856	3022	1768	1389	2528	1162	1097	974	2512	3177	4698
212407_at	591	802	699	742	1052	550	655	497	524	720	480	681	746	787	1085	1167	958	797	1443	1323	1480
212408_at	1311	982	1705	2650	1680	1402	1053	1244	1553	1862	2029	2012	3215	3428	4787	3045	3212	3180	3354	2525	3887
212409_s_at	778	388	990	943	792	696	939	879	1109	1066	1117	1004	324	405	328	304	297	332	390	284	373
212410_at	2627	2322	1354	1583	3127	3818	1798	2111	1259	1718	2312	1962	6093	4292	2861	2443	2205	2699	4650	2296	3988

212411_at	1987	1742	1895	1123	2510	1763	2131	1486	2423	1987	1759	1562	2748	1682	1424	1030	1157	1374	1992	1383	887
212412_at	1819	1781	1865	1670	2224	1578	1822	2326	1383	2238	1157	1367	1275	1238	2146	3737	2687	3143	3532	2533	3553
212413_at	140	127	123	263	298	422	103	165	187	98	267	270	157	386	139	198	161	138	207	114	110
212414_s_at	234	287	327	501	525	790	125	194	268	233	523	496	517	832	915	505	589	588	734	751	951
212415_at	194	147	397	507	660	746	50	123	308	210	746	802	343	264	578	114	181	113	257	373	391
212416_at	1437	1710	1679	2235	1557	1867	1446	1791	1753	1781	1342	1534	1505	1794	3266	4953	4999	5037	3667	5417	7011
212417_at	165	125	231	416	256	293	250	235	215	327	217	334	344	252	158	235	200	299	227	361	697
212418_at	400	1007	931	1045	704	886	431	628	353	608	775	758	1372	1258	1774	1647	1584	1519	1755	1883	3392
212419_at	345	238	167	279	832	765	363	455	318	305	850	858	348	318	264	200	155	96	119	59	40
212420_at	183	808	775	1235	236	590	1155	504	834	795	755	753	1456	711	284	206	280	255	286	257	315
212421_at	476	652	260	383	580	466	441	511	278	565	290	398	214	159	159	351	302	246	282	204	307
212422_at	2713	2288	1601	932	1056	843	2560	2093	1667	1596	1067	726	347	193	254	574	453	706	398	342	280
212423_at	362	446	148	213	744	606	352	455	235	344	718	832	430	396	232	225	190	129	168	71	138
212424_at	1561	1128	1015	635	181	140	1993	1181	1300	887	617	762	238	192	237	645	420	747	404	370	322
212425_at	271	136	148	260	419	383	179	172	203	231	156	182	46	56	71	61	80	72	125	114	170
212426_s_at	8167	7894	6119	4294	10806	12385	3980	7713	4291	5119	8453	8692	14404	12848	11323	9911	8283	10447	9365	7999	9281
212427_at	77	75	123	105	135	181	144	57	136	120	162	136	75	81	51	76	54	78	137	137	141
212428_at	991	702	890	647	1117	1795	1231	837	999	778	1309	1130	1170	778	609	578	504	593	630	752	794
212429_s_at	1655	1802	1872	1596	1667	1119	1924	1767	2095	2070	1897	1529	1885	1726	2352	2046	2067	2078	2432	1940	2471
212430_at	430	442	362	330	804	649	441	470	424	523	672	494	523	335	200	116	91	119	177	116	82
212431_at	505	474	719	376	327	697	735	686	807	744	689	260	196	180	160	68	168	131	187	190	209
212432_at	2123	1016	1792	940	1161	1102	1879	1508	2571	1730	1606	1279	2051	2039	2088	2599	2689	1705	2102	1291	868
212433_x_at	35047	46554	28016	28024	33617	31704	32036	35633	28332	24202	19902	30300	22492	26033	23289	28311	28080	30235	16473	26240	21658
212434_at	1917	1154	1455	643	1421	1141	1321	1238	1452	1342	1199	1018	1393	957	994	2977	2746	2629	2325	1485	1665
212435_at	482	571	570	746	477	486	640	881	567	1030	392	393	448	419	476	1484	1187	1261	1759	1389	2009
212436_at	394	311	461	282	114	74	416	408	424	509	443	395	356	439	423	652	678	846	684	545	1079
212437_at	441	495	456	384	399	383	554	471	460	558	590	375	162	213	211	166	171	139	214	137	125

212438_at	1672	1152	1073	791	1862	2099	832	1349	855	1041	1325
	1157	1746	1638	1049	1782	2289	2653	1951	1499	1372	
212439_at	566	526	499	447	465	458	517	462	585	514	827
	509	361	373	220	187	223	222	205	254	213	
212440_at	1664	1276	1367	1098	1431	1853	1352	1198	1159	1498	1526
	1776	1784	1647	2773	3020	2698	2938	2744	2469	2656	
212441_at	1181	1671	652	843	1001	1044	889	1147	405	481	738
	682	606	446	276	530	520	462	282	445	426	
212442_s_at	1453	1033	2558	2747	1674	1299	1704	2014	2680	3516	1639
	2687	1831	1477	1457	1029	1063	1033	2265	2362	2683	
212443_at	213	373	1057	1270	390	364	465	501	855	801	259
	62	76	45	36	89	82	71	210	121	58	
212444_at	2167	1797	3802	2090	1196	988	1381	1777	3087	6040	2945
	3317	1279	1240	1318	1695	2471	1395	3020	2202	2737	
212445_s_at	572	488	939	1910	592	577	534	561	1076	990	812
	814	476	628	622	616	868	801	1100	1264	1662	
212446_s_at	1078	624	1160	1067	1106	1125	582	813	922	1623	780
	980	1321	1000	1386	1156	1068	936	2723	2894	3560	
212447_at	708	678	903	806	2098	1305	930	1110	1106	1234	1558
	1543	1772	2253	2013	944	859	784	1014	989	1143	
212448_at	138	331	482	2565	209	184	286	108	484	618	217
	372	138	154	154	179	225	150	394	465	438	
212449_s_at	3658	3179	2405	1573	2394	3717	1945	2561	1916	1959	1775
	1957	3813	4057	3410	5162	4847	5354	6330	5943	7690	
212450_at	347	421	288	546	313	468	325	366	205	204	281
	244	305	320	200	371	280	323	296	290	298	
212451_at	208	221	133	215	269	208	193	214	185	171	134
	147	65	49	69	76	69	31	53	61	66	
212452_x_at	364	303	461	719	258	435	492	624	559	764	436
	460	69	58	76	61	45	32	80	92	100	
212453_at	1463	1248	1134	820	1505	875	1247	1359	898	1202	1067
	1135	885	1088	1008	1606	1408	2103	2265	1221	2029	
212454_x_at	3495	2632	1921	2319	1776	1844	1840	3072	1817	2679	1742
	1695	1760	1648	1706	2591	3120	2495	3447	2577	4227	
212455_at	1856	2041	2017	2479	3065	2182	3336	3299	2356	2458	2800
	2559	1921	1517	1830	1783	1528	2433	2751	1968	2050	
212456_at	1361	1161	1078	807	1005	722	1265	953	1757	1449	1180
	960	786	598	364	251	201	323	571	332	222	
212457_at	234	495	948	566	1142	1574	147	67	543	458	1887
	958	1530	1359	1860	424	349	409	1194	1275	1348	
212458_at	1431	838	2006	1014	1270	632	1492	1375	1979	2580	1738
	1622	1280	1080	1224	1585	1242	1152	2465	2041	2893	
212459_x_at	2376	2103	1805	2166	1271	2057	1795	1882	1169	1803	1461
	1570	1997	1770	1792	2429	2234	2338	3405	1976	3372	
212460_at	1599	1045	1142	537	1596	1467	1084	1446	740	857	1516
	1426	1765	1260	1139	1394	1347	1226	1293	904	954	
212461_at	3060	3278	1921	1439	1758	1930	3665	3769	1805	1831	2190
	2194	1922	1040	1012	3445	3186	2929	2201	1171	1585	
212462_at	274	609	711	732	454	521	330	447	478	749	324
	258	396	314	464	939	692	741	1452	2168	2826	
212463_at	189	487	936	1199	434	628	258	428	459	1121	648
	921	593	767	746	300	464	155	794	839	995	
212464_s_at	105	29	945	259	1512	798	189	232	579	96	773
	1043	905	676	933	154	207	146	215	353	374	

212465_at	839	895	1074	998	1842	1606	1366	1071	1370	1582	1514	1184	1211	936	2323	1394	1544	947	3259	2068	2617
212466_at	46	101	126	56	26	26	204	230	237	309	189	153	29	29	13	33	14	23	25	24	21
212467_at	668	564	1251	1095	1251	1363	753	615	1036	669	915	687	816	874	625	513	402	493	1272	979	1164
212468_at	161	69	151	225	238	265	146	172	178	298	168	358	171	104	113	82	33	45	136	214	137
212469_at	97	219	262	494	285	481	89	98	433	552	222	104	226	216	145	216	202	261	299	589	617
212470_at	1156	779	1245	569	1556	1850	925	1122	973	1466	1407	1513	1092	1017	884	717	659	915	1249	1244	1166
212471_at	283	125	176	110	238	196	189	256	274	225	216	157	208	226	224	217	234	149	293	243	342
212472_at	683	1079	3123	1993	1491	3119	713	606	1873	3706	1235	871	366	360	515	454	596	284	1506	1061	915
212473_s_at	1458	2118	4012	2404	2043	6508	1239	907	3509	6774	1634	2128	1794	1859	2709	1631	1773	1321	5443	5232	6574
212474_at	1468	1097	1784	1729	1742	2321	1402	1293	1403	1688	1806	1491	2183	2644	3048	2856	2326	2479	3204	3493	4037
212475_at	352	114	198	226	114	314	398	317	590	454	367	378	259	309	337	162	156	85	211	260	225
212476_at	777	797	692	704	1170	1435	496	866	444	486	691	753	1219	1100	766	859	629	665	1133	1474	1938
212477_at	75	67	72	135	102	70	99	115	75	96	53	38	8	22	4	22	20	20	32	22	26
212478_at	78	10	47	72	98	52	256	388	63	172	104	84	35	11	41	31	77	40	25	32	23
212479_s_at	491	328	501	480	396	466	484	437	376	512	266	211	333	328	443	507	616	587	749	551	759
212480_at	525	538	551	425	821	742	501	362	348	298	887	968	626	485	263	198	111	142	102	316	201
212481_s_at	1890	1673	1700	570	1398	1275	1730	1807	1295	1557	3790	1622	835	879	1192	815	882	878	510	363	460
212482_at	499	422	1021	1698	543	875	776	806	1078	1665	793	620	621	691	581	663	779	389	1143	1037	1398
212483_at	302	215	266	469	304	654	213	224	260	326	165	204	226	168	217	332	222	231	452	701	831
212484_at	1127	2185	513	1543	443	402	1048	1977	743	637	711	674	474	170	300	534	309	402	289	277	143
212485_at	489	476	562	641	716	926	586	606	769	925	905	894	515	407	301	458	437	390	671	550	575
212486_s_at	142	91	125	122	344	370	72	148	157	232	200	234	272	268	565	87	154	42	335	556	896
212487_at	341	197	191	305	69	43	408	449	341	412	381	390	211	328	318	346	283	242	341	338	280
212488_at	184	89	201	195	25	28	201	133	171	210	158	126	5	9	23	27	25	6	42	29	7
212489_at	9	2	8	8	12	12	8	8	11	7	6	55	8	11	6	6	2	7	3	10	4
212490_at	217	173	103	18	260	258	160	268	147	112	139	127	175	129	129	305	236	179	203	151	177
212491_s_at	696	540	646	583	821	746	1125	891	569	532	867	674	1317	1492	923	1104	943	1226	811	591	676

212492_s_at	275	416	206	362	70	331	290	331	103	191	311
	259	163	136	101	241	150	196	93	157	100	
212493_s_at	819	985	1024	993	1276	1668	447	590	1108	1076	1022
	998	684	532	729	712	659	704	872	987	931	
212494_at	291	248	359	722	547	327	398	243	554	418	607
	298	114	176	85	68	69	62	86	81	35	
212495_at	438	405	267	341	198	380	531	432	265	264	313
	256	156	129	74	117	52	58	43	89	37	
212496_s_at	372	703	302	594	135	460	579	509	116	267	472
	238	433	399	641	694	695	571	317	639	484	
212497_at	251	139	144	241	12	15	333	236	261	207	266
	186	104	80	70	157	104	115	115	66	53	
212498_at	1117	991	1789	2530	1116	1333	676	1071	1585	2292	1049
	1335	979	1332	1773	1007	846	868	2666	3272	4221	
212499_s_at	634	543	479	473	533	1419	729	847	288	316	654
	715	1216	482	503	812	631	772	1508	896	760	
212500_at	606	1061	407	361	576	990	431	662	401	493	418
	555	518	266	301	889	700	777	709	892	1143	
212501_at	6302	6778	7497	4489	2180	8738	4603	2800	3919	4141	2881
	2637	3522	3425	5017	5275	5414	6845	5765	11246	8254	
212502_at	1770	1726	1114	661	1310	1527	1783	1754	1290	1347	1080
	988	786	864	1142	1549	1552	1373	1187	987	982	
212503_s_at	732	794	224	410	249	386	619	577	234	298	131
	162	61	65	69	434	393	343	189	314	347	
212504_at	259	297	266	422	144	120	473	533	349	324	209
	253	32	58	69	77	30	74	100	181	130	
212505_s_at	587	618	551	446	667	806	497	574	411	534	674
	386	244	242	396	392	477	412	333	373	391	
212506_at	706	1240	3215	3295	1939	2626	973	1198	2725	2899	1735
	1589	2760	1004	2708	1009	1047	920	3547	2543	2603	
212507_at	2488	1459	879	777	1214	1762	1313	1529	753	688	1128
	1271	962	1505	1470	2788	2356	3149	1188	1565	2156	
212508_at	533	1182	624	1192	1293	1285	447	603	627	1139	1379
	1880	1410	1304	1602	814	1016	584	1562	1558	2106	
212509_s_at	3495	6834	764	554	4110	3338	3526	3342	641	524	3780
	5657	4492	5162	7106	7502	7746	6405	1082	1263	1584	
212510_at	865	612	895	627	797	886	1076	620	1187	1220	1245
	905	1143	1102	2218	1639	1463	1390	1691	894	1462	
212511_at	176	390	345	494	223	413	231	186	276	247	200
	230	442	314	451	375	302	327	1157	709	1009	
212512_s_at	1699	1404	750	494	385	241	1210	1068	882	718	505
	211	390	295	570	998	705	1030	910	633	627	
212513_s_at	2166	1325	1099	1223	1425	1431	1174	1432	779	1212	1077
	1584	1712	1105	1728	2410	1815	2257	2760	2554	4000	
212514_x_at	1920	1516	1196	652	1783	1610	1911	1807	1519	1481	2409
	1240	487	450	517	640	536	519	566	288	461	
212515_s_at	1958	2073	1902	1240	2803	2415	2343	2795	2463	2028	3458
	2199	3392	2867	1947	2428	1812	2094	2502	1312	1740	
212516_at	276	254	351	497	232	379	224	143	292	425	250
	178	145	146	168	118	92	159	268	291	208	
212517_at	598	601	290	375	669	1063	478	438	260	298	594
	582	329	397	367	425	419	425	253	384	440	
212518_at	848	974	693	1139	667	523	796	999	542	760	664
	481	199	188	266	225	269	288	226	269	155	

212519_at	2037	2308	4220	3970	2862	4388	2805	2383	2927	2376	3540	2258	5786	5046	4848	4919	4861	4419	8099	6053	6797
212520_s_at	757	816	875	484	488	215	788	649	644	479	607	344	388	417	400	315	319	249	466	284	183
212521_s_at	611	776	1705	1833	891	1184	969	955	1957	1855	1278	1464	445	378	537	366	319	335	824	776	1104
212522_at	762	915	2094	1745	1311	2663	755	864	1707	1842	1222	1944	1053	1020	1740	996	1028	922	2826	2718	3996
212523_s_at	204	151	153	253	199	89	209	239	139	129	162	226	180	307	240	97	154	197	163	97	104
212524_x_at	21	9	23	23	52	52	25	50	24	28	13	23	13	35	40	25	23	14	19	6	7
212525_s_at	1093	219	505	316	1067	364	840	867	262	436	1654	1168	327	254	342	229	122	484	273	103	78
212526_at	4	10	3	9	4	4	8	9	3	3	41	10	14	8	7	12	1	8	10	1	14
212527_at	994	755	1016	466	630	481	802	636	714	618	689	407	427	265	356	343	357	321	375	305	153
212528_at	660	533	921	581	652	468	718	690	551	843	816	757	418	148	204	168	190	218	355	315	295
212529_at	714	646	343	378	216	273	1114	702	769	811	908	385	383	386	334	379	393	406	452	293	242
212530_at	902	661	1816	1376	904	1113	409	858	838	1226	793	994	1309	1272	1477	1547	1411	1660	2507	3320	4814
212531_at	280	2944	2058	10317	599	1552	627	195	1432	811	3387	1590	1341	629	539	1089	1224	338	1776	10915	4944
212532_s_at	1933	1741	1963	2019	1597	1437	3424	2512	2963	2126	2328	2326	2116	1987	1984	2277	2150	1955	2809	1889	2187
212533_at	843	833	715	619	620	318	710	813	698	823	928	1137	1093	992	1181	2446	2402	2157	2877	1870	3136
212534_at	859	991	832	1085	920	790	1280	1378	904	996	635	504	372	268	322	1366	1089	1150	669	864	858
212535_at	634	1081	1040	1117	437	736	1118	1098	838	977	803	620	410	567	341	1099	1070	884	868	1442	1353
212536_at	557	436	412	331	862	1341	307	267	268	242	448	518	636	799	542	698	411	487	832	581	775
212537_x_at	21781	32451	17837	21782	8856	11917	20926	21761	16418	14731	9991	13692	14688	18785	21604	27183	29984	28727	14760	23315	17824
212538_at	669	389	642	425	1791	1510	262	305	387	408	1838	2125	2855	3151	3667	705	592	796	1101	1024	1191
212539_at	1734	1993	2372	1467	1150	1504	2737	2858	2440	2333	1501	1372	1327	1767	1806	2298	2205	2141	2360	1598	1846
212540_at	1769	1176	871	649	782	660	1175	1269	809	745	1485	545	1183	539	380	429	455	352	401	373	208
212541_at	880	588	966	715	485	606	714	467	555	879	449	367	564	331	340	395	449	493	600	533	428
212542_s_at	860	860	924	879	1358	707	775	773	811	798	654	554	543	668	849	702	384	508	1283	1185	1319
212543_at	679	581	2431	2611	228	230	962	826	2471	2949	247	165	69	40	153	516	540	552	3429	2245	2574
212544_at	1275	1163	2801	3409	1475	1887	1540	1453	2784	2193	2444	2480	2706	2605	2717	2078	2124	1684	3516	2866	2679
212545_s_at	60	35	57	70	66	75	32	17	24	22	51	44	4	36	19	13	10	11	11	33	10

212546_s_at	692	734	781	1627	559	413	769	1031	557	801	422	480	206	195	160	387	326	475	601	564	709
212547_at	1210	1748	1009	1489	902	1317	1346	1335	1311	1852	1091	1083	746	871	618	606	636	604	1042	1017	940
212548_s_at	619	1005	799	1887	704	425	1104	1426	982	1124	468	402	300	297	377	795	758	948	1147	1250	2503
212549_at	310	196	576	556	1153	1150	213	244	444	630	676	902	318	296	203	38	68	83	313	309	282
212550_at	196	128	316	304	389	288	148	140	270	437	399	217	88	71	44	11	12	10	84	35	39
212551_at	229	146	353	312	206	351	302	216	344	306	355	212	451	587	165	78	130	73	285	197	300
212552_at	5027	3977	3639	2239	2814	2455	3238	2151	4079	4665	5347	3530	3708	3455	4760	5680	6215	4040	3812	2745	2662
212553_at	822	710	1096	1053	430	645	572	747	583	839	696	464	175	170	192	417	309	128	428	237	236
212554_at	298	165	336	353	366	353	223	220	256	236	236	207	512	443	352	337	198	322	728	755	1348
212555_at	216	86	544	39	194	675	130	90	248	365	702	473	96	62	68	37	39	75	51	42	58
212556_at	1795	1356	709	462	1423	1195	986	1103	522	580	628	851	887	721	561	863	875	822	787	624	411
212557_at	310	397	323	263	761	703	496	537	272	338	483	455	391	464	581	708	587	646	473	444	567
212558_at	284	274	282	484	267	621	376	285	500	502	210	177	90	73	141	153	155	105	539	535	788
212559_at	225	247	464	215	682	715	364	223	537	546	787	745	157	224	237	75	127	60	174	151	92
212560_at	562	779	1335	3607	1706	3348	557	711	1077	1357	1751	1321	876	1079	1330	703	753	569	2143	3007	3082
212561_at	1131	1112	632	656	1005	1512	1060	1366	789	907	1198	1678	913	1182	1149	1270	1894	1540	701	747	918
212562_s_at	73	79	197	131	377	374	49	129	124	112	37	43	62	55	93	23	48	28	15	12	33
212563_at	1667	1247	834	190	322	134	1500	901	963	865	803	276	572	697	832	1929	2122	2224	1405	736	739
212564_at	394	260	374	247	400	261	401	293	249	236	438	183	202	125	199	199	208	149	112	125	90
212565_at	134	151	349	625	399	587	110	187	397	463	200	476	672	595	705	133	178	159	1608	1273	1642
212566_at	2320	2659	1559	2777	1486	2677	1670	1978	2095	2397	1391	1345	1222	992	693	1753	2165	1255	2485	2288	1674
212567_s_at	732	678	749	1260	1058	1097	510	567	854	861	725	569	206	172	140	223	146	162	287	106	180
212568_s_at	1476	996	1219	845	1190	984	1536	1492	1519	1089	1391	873	1241	1112	1270	1772	1697	2656	2798	1420	2465
212569_at	357	298	665	309	528	442	285	587	558	562	446	407	467	431	395	570	437	619	941	931	1139
212570_at	609	586	871	692	727	680	836	899	924	1427	912	654	433	410	321	153	228	109	527	335	296
212571_at	644	266	753	945	570	852	786	896	981	812	604	706	318	244	268	132	131	200	200	197	240
212572_at	593	602	2309	3268	1572	2821	639	930	2075	3712	2253	1580	1773	1702	2317	966	850	947	4005	3242	4929

212573_at	1550	2094	2222	2389	1821	1782	2325	2183	2467	3961	2000	1494	1513	1334	1283	1341	1502	1183	3263	1630	1822
212574_x_at	937	889	859	852	539	504	1129	953	1108	1159	972	431	59	85	55	71	47	106	83	56	45
212575_at	17	99	52	25	18	17	313	156	215	268	189	9	8	9	5	16	3	42	28	6	3
212576_at	935	1398	951	788	575	407	939	953	835	1002	1120	631	296	433	674	768	750	958	734	779	880
212577_at	433	388	1141	623	647	445	1014	1293	1259	1458	1154	1046	379	322	206	370	357	470	643	530	731
212578_x_at	18996	31141	21509	28998	18600	26045	20061	20443	26701	21645	15154	22444	14975	21790	21806	20167	22056	23017	13608	20706	16914
212579_at	425	375	678	401	481	496	355	548	361	593	449	430	546	532	314	678	712	739	1164	896	1101
212580_at	135	80	123	141	164	78	98	154	122	140	129	147	50	56	93	27	51	40	51	54	60
212581_x_at	32679	39375	27928	34148	32604	30833	32597	30770	37728	27481	28285	41220	18006	26485	27911	21994	24358	25162	15169	21171	17808
212582_at	915	1008	2622	1516	2058	1673	2439	2664	2234	2338	1994	1703	2750	2173	2259	1677	1593	1579	2336	1809	2382
212583_at	130	26	241	251	298	63	133	78	202	335	92	137	56	76	58	38	21	25	129	140	130
212584_at	663	588	1717	1527	799	935	1043	1003	2106	1821	1103	916	525	628	763	743	682	759	1681	1056	1552
212585_at	1451	1701	1366	713	998	974	1526	1697	902	921	797	783	1884	1457	2239	3450	2693	3463	3527	2858	4073
212586_at	1316	1452	2222	2784	2709	3599	1638	1711	2740	3258	3272	4806	3881	4492	4995	2393	2645	2014	4507	3978	5773
212587_s_at	78	10	38	9	41	31	5	13	63	13	10	30	2	32	22	13	15	27	18	9	3
212588_at	29	23	31	49	93	4	12	25	33	38	10	36	16	6	2	18	5	18	18	19	24
212589_at	1124	2010	993	1119	697	1486	754	673	947	1002	790	1059	1025	1107	1670	2678	2600	2221	2721	2196	2973
212590_at	1429	1516	2166	1389	920	1803	1185	1305	1721	2046	1693	1306	1879	1558	1807	2200	1850	1360	3892	2295	2343
212591_at	1561	1197	1483	1248	865	1070	1401	1174	1347	1234	1060	951	1866	1939	2529	3408	3054	3758	3344	3146	3754
212592_at	8	2	7	26	29	66	5	34	16	25	3	20	1	1	1	1	14	2	1	2	0
212593_s_at	634	521	1034	5463	661	392	509	860	337	541	232	674	352	525	569	1506	1360	643	1151	2194	1978
212594_at	78	35	189	504	275	293	80	127	76	144	111	185	100	170	93	153	138	67	271	491	400
212595_s_at	1700	837	2576	3892	2687	3583	3710	5198	3619	3455	3884	3699	1935	1511	1232	1447	1298	1357	1207	780	1228
212596_s_at	1189	816	606	506	609	650	1226	1037	696	623	698	562	767	680	580	1107	902	690	597	410	436
212597_s_at	1033	961	711	447	588	645	1132	1206	718	636	854	753	805	929	958	2247	2159	1823	1124	684	1157
212598_at	61	95	151	100	30	34	77	98	138	168	82	97	30	6	19	12	65	47	58	77	68
212599_at	97	94	1373	2064	304	196	87	107	2408	2080	226	170	20	26	74	19	1	16	1559	1069	1659

212600_s_at	5125	3906	5738	6412	3332	4271	3509	3159	4944	4566	2437	2754	3789	4005	5837	7621	7532	7242	8697	8656	9204
212601_at	527	444	537	411	433	594	252	327	399	426	642	635	452	291	232	207	203	229	437	616	562
212602_at	599	760	590	741	386	460	664	805	388	397	530	336	168	170	186	280	299	374	201	234	262
212603_at	620	421	520	363	1244	1333	366	560	610	614	797	814	1708	1352	1855	976	859	783	1349	1007	1048
212604_at	533	500	732	513	1347	997	1084	926	807	690	1303	1355	2226	1656	1635	822	794	668	1164	782	916
212605_s_at	569	418	399	497	375	501	542	602	404	626	493	354	313	261	409	604	473	211	551	440	595
212606_at	291	434	201	269	191	267	310	350	153	108	230	145	94	86	128	321	293	279	134	136	199
212607_at	295	201	206	213	654	685	250	487	248	344	692	641	309	300	361	92	86	57	55	56	78
212608_s_at	695	577	589	735	744	851	886	907	759	685	702	522	539	469	543	822	646	380	740	663	806
212609_s_at	242	321	14	18	26	203	290	245	15	55	163	102	113	222	84	229	160	208	64	58	69
212610_at	5093	4513	4214	2552	4254	3959	4166	4287	3622	3645	3165	3177	5278	4165	5784	7816	6413	7069	8069	8206	9888
212611_at	574	776	1796	6248	356	736	945	938	2676	3132	570	219	123	154	153	488	430	289	1151	1005	1044
212612_at	709	601	804	576	1711	1410	498	746	539	776	1006	1450	1403	888	602	784	667	590	1213	845	1000
212613_at	32	118	12	17	15	16	15	13	9	10	9	9	24	46	11	161	252	276	88	134	108
212614_at	232	747	709	2655	801	1660	600	607	1128	1487	1218	866	745	745	736	304	328	424	1722	971	1887
212615_at	318	231	326	305	140	320	171	141	222	180	57	54	123	102	171	436	278	551	610	739	1437
212616_at	904	746	1276	1305	443	583	625	753	1080	829	580	250	250	344	365	456	377	382	780	928	1208
212617_at	245	248	403	358	210	114	329	438	329	435	263	215	80	35	90	156	99	78	144	178	164
212618_at	36	63	45	59	45	52	62	37	58	68	34	39	76	12	5	11	7	9	20	19	43
212619_at	242	50	465	197	517	157	348	476	448	738	721	641	150	148	111	52	39	90	158	86	161
212620_at	69	99	64	226	172	164	58	150	308	321	77	90	163	140	70	111	34	83	188	110	173
212621_at	528	170	905	290	1043	63	409	731	607	1027	1246	1850	1570	1641	1355	396	287	426	1295	1023	1218
212622_at	1552	1382	2786	2610	1170	1521	833	1376	1312	2013	1300	1356	1255	2098	2115	2258	2138	1754	2254	3832	4706
212623_at	2447	1803	3053	1954	1596	1654	951	1304	1877	1899	1619	1208	1229	1723	1799	2636	2755	2554	2479	3360	5005
212624_s_at	246	371	45	25	1388	1027	139	214	21	46	1369	616	1126	851	3036	395	304	421	134	523	672
212625_at	1259	1872	501	418	730	703	1458	947	784	748	897	408	882	642	289	684	658	588	346	217	187
212626_x_at	3486	3185	6074	4058	4513	4956	5641	9043	5099	5405	4973	2880	8320	4540	5993	10140	8463	9050	7127	4746	6046

212627_s_at	1516	594	1576	791	2390	1535	1393	1478	1900	1944	1796	1043	2608	2549	2252	1804	1669	1866	1618	886	573
212628_at	758	617	1226	856	929	1017	678	808	879	907	723	484	922	1067	972	1325	1005	1330	2321	1632	2554
212629_s_at	336	300	552	596	515	469	643	825	831	932	667	504	1289	1306	833	1456	1061	839	2040	1387	1631
212630_at	544	654	517	1060	592	602	419	301	744	446	442	63	376	394	454	283	403	269	793	669	749
212631_at	345	308	510	722	825	1645	497	665	344	791	584	530	429	373	907	914	792	767	1073	1052	1517
212632_at	595	792	678	763	311	396	665	959	492	773	578	850	517	492	810	906	1024	771	653	791	1254
212633_at	728	562	464	566	625	1012	386	632	391	409	397	398	716	704	617	1192	820	1091	1407	1356	2388
212634_at	284	190	125	206	231	306	325	317	225	217	141	240	294	226	161	301	269	231	200	222	391
212635_at	758	460	1306	841	900	731	567	643	1019	1635	1526	909	1382	1867	1791	1538	1356	1357	1982	1953	2256
212636_at	3	10	8	3	15	9	29	5	1	6	50	10	18	49	26	21	24	40	4	16	4
212637_s_at	321	245	220	178	305	391	184	210	165	217	129	153	349	218	148	309	341	296	1197	598	921
212638_s_at	648	841	1171	1737	614	723	1035	1163	1188	1712	1080	740	724	739	411	866	753	984	1418	774	995
212639_x_at	28451	13772	28892	8523	32823	19855	29472	29115	34206	28211	31544	36168	24996	31259	27592	21083	20924	25525	18981	16017	14100
212640_at	2467	1626	3087	2465	2623	1735	1565	1878	2390	2303	2383	2390	3411	2583	2028	3126	2837	3252	5145	3760	5345
212641_at	58	137	163	237	18	278	160	146	154	126	186	82	60	107	57	16	47	31	55	66	44
212642_s_at	191	241	331	266	474	404	180	224	213	212	378	172	202	223	219	120	244	171	217	295	263
212643_at	5275	6290	3974	3961	3735	3717	5953	5735	4540	4249	3677	5280	5013	5959	8203	10907	10207	9413	7399	6254	7431
212644_s_at	1643	1669	909	813	951	719	1654	1556	1118	1294	1141	1058	1040	1023	1019	1981	1633	1390	1777	1129	1024
212645_x_at	370	456	578	839	394	942	557	395	375	530	799	367	359	405	482	352	456	424	274	321	249
212646_at	402	546	54	182	220	223	371	326	20	141	209	103	42	19	44	357	265	248	25	6	20
212647_at	332	803	834	1421	418	766	468	373	623	872	664	745	634	672	279	378	415	352	547	352	242
212648_at	1036	646	745	400	830	488	645	1104	574	591	541	544	759	514	432	838	906	989	920	764	1006
212649_at	421	360	338	246	234	281	371	461	364	207	223	217	68	96	119	126	141	115	156	127	84
212650_at	241	102	191	164	52	228	338	343	189	266	198	210	120	87	50	83	86	25	24	42	40
212651_at	13	11	194	113	10	11	74	8	131	129	10	14	2	2	17	17	34	12	195	327	391
212652_s_at	713	527	929	1266	690	910	644	677	1147	1043	992	677	755	1337	1420	1531	1300	1200	2521	1504	2665
212653_s_at	2291	890	2105	956	1332	1316	1332	1446	1215	1508	1428	1037	1463	1421	1502	2971	3328	2473	1705	3446	4063

212654_at	77	11	221	66	58	32	52	36	79	63	10	29	32	19	19	125	114	74	83	66	64
212655_at	1726	1176	986	791	1054	1184	1165	1475	804	1048	918	943	1067	938	915	1344	1486	1101	953	1022	1036
212656_at	1636	948	1729	1075	1512	683	1159	944	1825	2320	1484	1249	1762	1137	969	770	803	853	1558	940	674
212657_s_at	216	77	650	1207	82	24	146	111	755	600	125	136	26	38	44	42	23	26	1306	897	897
212658_at	595	713	758	797	565	1060	827	635	805	991	635	621	357	426	343	278	410	301	409	372	366
212659_s_at	78	146	434	625	311	159	60	181	454	446	240	248	20	37	21	18	3	6	135	111	94
212660_at	1164	1148	1546	1694	1495	1829	913	719	1737	1460	1769	1101	1363	1043	734	952	913	772	1531	1504	1465
212661_x_at	28701	28552	33994	29628	35423	28496	37254	35977	36041	32915	34556	40221	24745	33286	37482	30097	33145	33283	20030	27842	22145
212662_at	365	327	913	361	708	704	285	281	491	390	414	258	281	260	140	118	168	180	184	255	141
212663_at	827	895	954	1395	719	1050	845	866	954	883	1235	992	590	497	644	334	372	362	424	582	547
212664_at	295	327	137	172	170	169	506	660	165	226	220	206	17	46	46	182	124	208	45	47	26
212665_at	1763	4160	2130	5600	3292	5652	2125	3269	2141	1729	3075	2820	4195	4498	5914	3092	3292	2928	2642	2398	3028
212666_at	843	831	1156	882	588	726	523	442	670	1009	648	686	284	276	197	287	285	228	575	410	456
212667_at	61	29	19	12	198	102	13	16	3	81	85	92	20	31	33	321	272	236	20	7	25
212668_at	34	157	203	216	96	82	140	49	198	217	286	131	50	35	109	4	12	42	42	20	44
212669_at	810	939	739	1011	393	638	769	591	542	671	499	380	156	157	85	167	234	195	289	294	257
212670_at	56	39	47	50	140	117	41	28	47	41	38	19	11	9	8	22	29	8	11	11	15
212671_s_at	61	4	19	76	33	47	25	61	12	61	26	16	4	6	17	5	2	2	24	26	3
212672_at	204	198	458	313	679	458	182	191	249	349	215	485	315	278	403	144	143	179	537	1114	1343
212673_at	2901	2581	2568	1761	1293	1312	2924	3401	2551	2401	1789	1547	1070	1324	1960	4560	4045	5103	4180	1548	2912
212674_s_at	1729	1809	1137	922	918	751	1860	1839	1460	1321	1711	1010	1439	936	682	727	876	901	858	560	557
212675_s_at	402	421	252	631	737	513	367	599	178	347	522	812	573	779	987	672	517	513	757	802	1118
212676_at	234	190	2046	1763	1095	849	102	219	2039	2430	1880	1455	688	502	421	80	47	135	1384	999	838
212677_s_at	286	300	148	673	506	261	396	351	314	477	465	488	282	243	484	218	299	178	242	205	178
212678_at	40	74	1142	1256	661	468	147	214	1199	1289	718	612	262	330	237	97	106	97	574	558	627
212679_at	209	105	125	100	180	132	139	95	159	133	239	99	44	27	29	37	35	64	46	31	67
212680_x_at	3662	3147	2830	1878	1388	841	2695	2301	2941	1992	2398	1176	3923	2438	2345	2418	2705	2085	2071	2030	807

212681_at	22	28	12	4	47	46	3	3	21	3	1	6	6	3	3	9	14	2	0	13	1
212682_s_at	135	383	129	182	32	89	298	330	261	229	307	232	223	155	139	71	103	131	18	90	51
212683_at	394	348	803	872	892	996	476	418	958	860	745	469	332	213	299	238	356	283	779	638	840
212684_at	335	260	359	199	137	177	461	550	288	225	438	307	620	542	420	438	418	467	249	348	442
212685_s_at	1240	1119	880	464	1449	1752	951	673	955	778	1522	1322	1963	2421	2095	2409	2994	2171	3749	3445	4184
212686_at	251	370	313	1242	646	921	295	319	392	391	207	207	272	196	283	236	249	145	571	667	724
212687_at	2297	1876	1256	1167	8611	5147	1275	1163	1002	949	6188	5497	11027	11551	13719	2810	2403	2651	4539	4263	4564
212688_at	929	980	1466	1816	1292	2342	776	643	1137	1381	1251	1243	1578	1485	1534	1160	1654	1415	2919	3423	3579
212689_s_at	538	417	402	969	590	1152	486	776	474	785	678	964	695	860	735	854	839	913	1035	947	1707
212690_at	519	752	952	825	576	590	847	843	1006	941	706	627	353	360	451	659	545	615	782	656	750
212691_at	1855	1236	1263	550	217	113	1964	1626	1480	1379	1742	1019	518	427	397	533	384	561	536	380	285
212692_s_at	2025	1506	1194	1922	1068	1832	1839	2656	1077	1434	1022	1093	766	621	513	1376	1104	1127	1302	1926	1689
212693_at	1118	644	1572	860	528	697	1488	1101	1787	1883	725	532	273	221	340	624	558	963	1015	801	642
212694_s_at	1531	1671	2234	1586	1626	1496	1522	1680	1335	1961	1487	1352	2091	2492	2233	2341	2531	2319	2340	2053	2155
212695_at	30	12	42	163	38	34	13	13	58	68	37	23	8	28	7	5	9	11	15	57	44
212696_s_at	2027	1667	1409	1062	1731	1299	1860	1805	1207	1286	1452	1680	1564	1855	1650	2202	2710	2548	1802	1171	1669
212697_at	1343	1067	1464	1847	1703	1407	957	1462	1113	1918	1869	1561	983	1020	829	612	597	452	649	618	366
212698_s_at	3248	2100	3231	2981	2889	2766	3101	2881	2315	3815	3624	3950	4377	2569	2612	4344	3185	6009	7072	5515	10313
212699_at	356	293	383	516	491	313	440	418	326	512	408	337	77	69	74	108	113	88	97	81	87
212700_x_at	208	253	180	194	245	476	260	129	138	166	250	225	166	114	102	142	136	212	156	170	137
212701_at	196	159	201	173	34	23	272	347	124	180	126	244	29	19	21	51	20	21	39	24	40
212702_s_at	426	415	433	241	588	806	531	492	541	396	847	528	687	643	283	277	257	160	301	249	255
212703_at	111	246	172	213	63	58	80	195	149	186	95	170	18	10	38	64	47	82	19	25	31
212704_at	1099	871	541	667	862	984	774	1192	514	686	603	890	288	292	404	508	474	512	476	409	506
212705_x_at	300	760	631	1019	121	90	392	569	348	653	551	270	25	111	90	105	125	125	97	81	33
212706_at	85	114	99	202	219	126	74	121	95	119	207	112	200	110	112	67	92	54	163	180	179
212707_s_at	50	15	212	76	514	175	60	30	41	52	114	109	96	6	110	22	37	13	35	9	25

212708_at	1529	1567	1161	943	1392	1215	1787	1589	1135	1059	2020	1355	1636	1604	1408	1303	1504	1400	1239	884	969
212709_at	578	371	827	322	696	583	290	416	557	475	335	421	768	584	464	889	603	994	2293	1513	2055
212710_at	545	477	806	404	580	680	518	565	617	696	618	439	512	375	373	408	386	254	738	712	632
212711_at	642	630	735	497	510	726	571	533	625	790	793	778	600	440	528	494	513	397	552	904	864
212712_at	687	555	807	427	744	521	604	542	660	711	765	523	524	471	469	332	420	294	375	446	605
212713_at	34	23	35	26	36	28	30	18	50	29	24	28	2	4	4	3	3	4	4	2	1
212714_at	1140	822	2177	1223	1901	1606	1635	1412	2119	2176	1843	1589	1799	1771	2764	3117	2525	2647	2844	2637	3141
212715_s_at	171	275	189	197	265	173	162	162	142	236	161	167	87	70	36	85	69	74	94	95	63
212716_s_at	6530	4153	6702	6623	3670	5127	5255	4297	8268	5713	5055	4909	9185	6809	5661	4998	5120	4460	6915	7793	4969
212717_at	349	495	437	554	509	1673	421	590	364	456	506	588	498	389	258	190	202	313	364	520	504
212718_at	5560	5412	3049	2629	5928	4909	4402	3502	4046	3002	5206	4668	7007	7087	10346	8272	8293	9349	7272	7156	8445
212719_at	320	611	395	504	256	360	500	520	396	365	159	173	80	74	184	236	201	175	336	219	275
212720_at	806	624	559	382	1168	982	1155	1143	1064	752	1126	1239	542	454	561	762	865	715	854	363	832
212721_at	1611	1232	1910	1992	1344	1611	1511	1537	1498	1828	1342	1046	1216	1175	1847	2703	2238	2216	2837	1950	2969
212722_s_at	785	470	528	446	598	306	521	348	708	415	617	488	672	613	326	465	474	458	317	333	231
212723_at	1658	706	1038	648	1560	1261	1137	740	807	790	908	845	1507	1342	1042	1370	1078	957	1154	1243	889
212724_at	550	1932	1656	1086	1322	1654	704	750	795	653	782	745	1058	1210	2284	1802	1986	1442	956	1616	2854
212725_s_at	635	898	1818	3691	1915	2246	1447	1709	1758	2366	1993	1555	1535	1349	1423	614	789	658	1568	1589	2694
212726_at	758	850	454	388	826	825	481	653	333	382	723	602	633	338	370	273	467	213	381	297	254
212727_at	499	574	1230	1664	1030	1090	636	759	1266	1575	517	524	307	315	250	484	452	345	786	852	714
212728_at	415	257	822	803	594	493	380	416	650	882	392	250	199	126	50	88	105	133	228	205	185
212729_at	638	322	895	709	749	607	678	657	823	1223	581	411	192	258	251	241	265	370	329	220	232
212730_at	306	584	225	215	789	640	268	294	110	134	415	620	177	205	224	196	190	128	49	30	38
212731_at	366	381	229	310	484	704	456	508	175	232	321	386	179	239	532	1001	1042	764	409	568	923
212732_at	62	44	72	62	335	403	28	46	13	17	7	44	16	59	19	10	11	35	36	16	7
212733_at	558	636	484	407	737	919	563	612	326	363	901	474	674	842	765	621	744	714	358	541	449
212734_x_at	33765	45341	22196	29515	18326	21586	26336	27818	23978	20975	16168	25385	19301	21376	17386	21682	21970	21481	12580	20904	12759

212735_at	274	296	300	275	326	359	262	264	300	348	394	312	184	233	419	173	303	195	191	302	215
212736_at	439	215	30	153	26	228	241	300	103	115	139	212	40	38	72	213	142	213	50	51	45
212737_at	1011	1235	606	1631	1047	3415	1522	1680	771	1073	1059	2070	555	375	691	798	710	973	1044	363	531
212738_at	348	92	286	50	301	50	637	714	388	307	237	117	291	269	149	372	224	270	313	149	193
212739_s_at	1275	1397	1670	1203	876	804	1190	867	1829	1219	1701	897	756	416	689	589	571	645	1072	420	346
212740_at	804	589	1124	1242	1063	778	865	746	1140	1118	804	827	657	639	681	938	795	784	1701	902	1372
212741_at	459	363	506	1984	678	577	702	594	1036	1269	1589	1607	662	569	568	457	396	451	815	1188	1477
212742_at	353	482	648	673	454	568	539	517	803	641	647	711	1020	940	882	654	645	678	783	901	889
212743_at	139	51	94	48	131	126	142	131	70	110	117	113	50	24	37	62	65	77	49	47	68
212744_at	167	206	350	499	536	775	298	347	244	370	215	366	232	263	220	194	228	224	236	200	252
212745_s_at	81	57	117	220	23	109	150	154	98	173	101	145	128	155	86	184	140	135	108	152	216
212746_s_at	38	108	510	132	32	63	277	389	107	509	105	165	206	198	126	338	281	361	538	407	428
212747_at	1042	971	700	578	367	491	690	668	677	823	478	530	415	442	385	614	885	691	1485	913	1200
212748_at	390	433	340	272	313	320	209	322	289	349	296	373	138	142	140	110	120	64	142	179	59
212749_s_at	1068	804	518	331	771	1102	1158	694	508	274	927	568	1388	1444	1649	2509	1966	2378	1258	1161	1602
212750_at	12	9	8	18	14	7	20	20	7	24	17	19	5	9	7	3	2	4	9	11	3
212751_at	929	781	918	1031	733	669	1273	1167	1296	1617	1035	798	1210	772	1234	1851	1657	1381	2567	1096	1642
212752_at	816	921	796	777	1082	1109	504	753	486	527	780	670	666	565	480	318	330	380	484	460	524
212753_at	954	1361	515	626	724	607	746	823	487	556	290	287	306	311	314	690	649	539	318	375	480
212754_s_at	587	558	695	926	396	1011	714	731	547	560	482	474	301	262	286	591	431	596	536	501	720
212755_at	9	50	23	26	25	26	16	13	23	56	14	10	35	3	5	33	30	23	47	62	34
212756_s_at	396	355	365	270	713	781	531	508	219	376	486	478	220	228	237	181	221	309	148	217	217
212757_s_at	915	713	981	1685	496	715	1059	1062	1205	1099	672	468	269	348	164	405	407	326	519	373	399
212758_s_at	46	21	41	34	327	258	30	20	27	34	44	102	80	87	41	14	8	27	17	17	34
212759_s_at	884	923	514	488	33	47	1132	1305	590	773	234	101	90	60	86	403	315	303	158	164	124
212760_at	683	634	629	961	694	1095	694	951	526	848	726	803	270	370	608	656	505	666	633	686	804
212761_at	6891	9000	4052	3325	1366	1485	5490	7131	3317	4486	1639	2121	1928	2064	3244	10695	9678	10710	5395	7387	8351

212762_s_at	3708	3883	2302	1381	326	390	2760	4468	1275	1790	782	400	176	159	53	617	721	1056	815	399	580
212763_at	220	203	366	197	334	417	436	527	491	361	274	226	139	140	79	80	103	101	83	87	85
212764_at	181	84	114	39	144	204	94	106	99	32	200	177	220	374	186	160	167	146	47	86	187
212765_at	507	523	1057	877	738	1161	655	553	891	668	817	604	942	919	1096	1186	1167	1267	1213	1632	1843
212766_s_at	1233	982	997	797	430	155	941	821	1325	1181	578	543	898	880	1061	1164	1275	1345	2373	2453	2217
212767_at	230	319	441	436	608	577	195	179	586	552	438	441	417	305	353	136	120	93	358	263	189
212768_s_at	7	6	3	9	16	13	4	5	1	7	11	4	1	33	3	18	7	2	1	1	1
212769_at	204	164	189	115	680	486	172	113	183	145	431	454	350	322	311	161	116	132	172	132	105
212770_at	530	395	521	444	672	210	527	750	538	637	1202	1219	947	898	447	132	103	84	326	197	175
212771_at	1196	1552	149	164	605	919	1415	991	206	189	961	584	673	493	1019	3266	3444	2515	64	54	59
212772_s_at	654	799	509	691	487	363	632	999	496	832	325	318	100	141	217	394	361	332	374	273	232
212773_s_at	2917	3370	2090	2262	1615	1463	2696	3216	1968	2527	1724	1688	2426	1809	1330	3355	3860	2895	2180	2452	1844
212774_at	110	272	785	885	874	1328	175	107	594	791	664	378	448	398	564	227	132	168	978	1051	1394
212775_at	8	6	39	9	867	735	5	12	8	8	1316	587	1218	1331	1228	3	3	1	23	94	100
212776_s_at	13	15	16	30	159	122	82	29	13	99	1231	476	382	272	152	2	2	3	3	6	3
212777_at	266	156	309	490	276	180	427	434	280	372	438	424	94	127	87	92	79	97	92	184	127
212778_at	573	900	521	658	448	550	549	544	528	556	516	452	310	360	250	353	339	319	373	307	284
212779_at	161	494	174	532	316	477	276	438	118	296	337	180	210	156	160	435	342	427	499	643	1317
212780_at	372	327	501	409	491	349	448	523	368	453	637	469	586	441	529	669	721	826	631	1171	1634
212781_at	751	481	458	476	661	638	588	480	260	270	367	276	977	1164	884	1737	1333	1495	2118	1761	2348
212782_x_at	3811	4174	3580	4450	3869	4884	3937	3024	2992	3378	8146	7034	11804	11028	8342	2226	2973	2193	2422	2531	912
212783_at	1169	898	695	587	1043	1043	912	976	615	556	679	400	722	868	943	1302	1271	1037	908	1047	1323
212784_at	441	376	285	750	735	840	660	602	499	826	455	369	315	225	225	279	235	281	268	308	268
212785_s_at	794	597	570	619	1211	835	1051	1048	562	630	711	545	1324	1445	1173	1382	1376	1825	1077	538	959
212786_at	595	597	507	599	257	431	502	507	535	465	480	587	165	149	147	128	130	95	95	64	104
212787_at	964	779	1168	768	1432	1256	1361	1630	1044	1259	1912	1183	1400	803	769	888	1095	701	908	766	646
212788_x_at	29921	45605	28031	39409	30359	42667	36806	30082	29335	23026	21261	28099	22832	17918	25266	23647	16892	25180	18007	21473	18374

212789_at	1560	704	1294	419	1263	294	977	1054	1943	1660	1430	1543	1014	851	608	569	441	617	1484	645	709
212790_x_at	30360	46326	37507	40378	27155	29243	30341	33186	35056	34174	29233	43596	24235	30972	25346	24850	26615	25963	17513	27128	19672
212791_at	556	571	737	1057	821	820	694	585	1037	1266	931	529	640	509	624	535	507	390	790	469	368
212792_at	4298	1799	1820	687	1186	945	2405	2445	1334	1807	1107	999	958	1110	648	1542	1810	1227	1277	950	893
212793_at	155	162	201	251	302	357	172	241	313	263	220	229	26	41	44	26	41	59	14	41	12
212794_s_at	389	330	867	940	547	415	778	846	1052	1606	641	901	292	317	370	466	344	332	639	720	1062
212795_at	894	661	1225	1295	1016	1445	504	912	708	990	866	677	977	1060	1422	1465	1246	1271	2602	3018	3962
212796_s_at	515	517	1228	1799	991	802	518	553	1141	1899	1707	1269	505	429	570	240	214	320	832	791	470
212797_at	131	32	300	147	304	298	122	140	296	503	442	280	45	8	27	27	14	35	55	11	74
212798_s_at	1249	1459	760	1545	2104	2059	1908	1706	1373	1224	1718	1874	2613	3226	4268	4212	4971	4975	3092	2470	4060
212799_at	852	1156	1145	1128	1336	1265	1036	1351	954	1065	1541	1604	1192	881	943	1139	1144	1044	1429	1231	1443
212800_at	292	187	330	219	503	414	294	224	316	280	453	381	233	230	222	214	146	149	362	178	213
212801_at	856	521	1183	466	1256	520	1008	1164	1118	1456	1248	1105	284	277	372	113	126	168	354	192	251
212802_s_at	1536	1025	1034	1324	1425	1376	1533	1438	1169	899	1403	1567	1094	1389	2482	2088	2100	2324	1276	1730	2033
212803_at	701	280	304	294	122	227	543	490	150	305	601	235	207	165	139	267	239	232	95	119	50
212804_s_at	779	254	591	502	598	679	709	709	524	510	659	735	666	666	1075	825	833	988	650	737	1090
212805_at	124	151	183	259	256	310	162	153	292	665	139	104	5	12	40	46	65	36	102	100	143
212806_at	21	36	7	85	242	216	89	58	62	245	31	41	8	29	27	18	20	23	35	37	11
212807_s_at	402	214	612	669	1105	1029	379	318	819	872	1427	1033	740	944	629	163	242	175	287	333	418
212808_at	247	161	598	423	296	144	299	442	538	1121	483	349	130	126	60	47	70	56	121	67	88
212809_at	618	425	1084	738	1502	663	759	709	1136	1215	1093	588	372	440	432	239	251	407	486	318	388
212810_s_at	258	241	723	411	613	1582	237	230	330	323	613	596	519	409	703	179	189	152	268	854	857
212811_x_at	378	378	1496	974	1229	1898	419	226	530	551	1353	1031	484	862	1412	274	340	305	489	1103	1010
212812_at	1553	2178	1928	7590	1225	3034	2494	2344	2341	2150	1367	885	2627	2615	4295	2306	2451	2429	6032	5485	7266
212813_at	111	141	125	120	285	347	81	182	154	131	102	148	56	49	92	30	47	140	81	50	18
212814_at	478	526	559	804	456	929	354	402	482	477	340	437	288	201	344	352	321	246	516	488	594
212815_at	661	984	1055	824	691	611	1088	1036	1363	1234	665	618	475	628	794	1374	1374	1509	2408	1511	1896

212816_s_at	66	51	95	32	414	1290	102	88	28	25	294	187	138	152	230	7	19	8	43	89	67
212817_at	255	129	187	178	241	169	288	141	178	154	225	132	68	15	27	121	118	118	65	82	50
212818_s_at	672	589	875	641	348	196	566	610	713	795	560	357	247	221	216	158	206	123	293	254	209
212819_at	193	105	186	91	221	187	113	135	99	145	82	101	113	68	46	78	62	15	68	54	50
212820_at	400	630	659	753	639	688	526	751	523	742	475	385	280	171	234	280	254	237	600	814	1056
212821_at	123	18	16	110	155	108	19	95	12	20	55	30	65	53	84	27	72	17	42	37	39
212822_at	132	72	123	202	1244	1330	85	107	91	81	843	679	243	148	120	87	82	50	13	32	64
212823_s_at	446	453	532	493	80	65	327	392	463	499	449	446	180	146	86	92	89	106	148	41	104
212824_at	1275	1049	882	569	1406	2115	761	695	542	797	760	747	1304	1223	801	1334	762	747	1631	1256	1361
212825_at	1230	1552	967	639	1526	886	1155	1077	1391	1135	1343	1235	1432	1142	1037	988	1085	1004	1601	995	1439
212826_s_at	9614	14664	4132	4845	3242	6468	9210	10693	2981	4490	9647	7925	11396	8252	4346	4739	4701	2901	2609	5756	4518
212827_at	22	97	174	385	429	456	223	199	206	162	196	20	23	52	73	50	57	44	67	72	32
212828_at	659	932	600	592	724	673	705	786	804	674	654	584	276	413	432	471	532	531	519	410	587
212829_at	1142	1731	861	878	782	1020	1285	1002	831	604	678	684	610	505	862	2022	1922	1663	1154	1588	1623
212830_at	364	313	274	433	460	332	493	681	206	337	384	271	210	210	317	548	470	440	236	237	330
212831_at	13	15	9	21	148	54	156	162	19	18	16	11	8	4	5	9	8	12	6	9	5
212832_s_at	1094	783	1884	1092	2190	1252	1725	2135	2455	2166	2099	1846	1248	1094	1394	745	655	742	1253	1014	1015
212833_at	1008	862	1287	1050	1336	1465	984	903	1153	1409	1801	1068	2474	2180	3399	2098	1891	2102	4104	2498	3529
212834_at	445	302	682	489	711	378	395	418	471	461	586	264	493	782	819	617	538	613	906	919	1123
212835_at	455	305	376	316	480	454	712	843	523	482	426	361	296	208	216	720	688	576	507	353	475
212836_at	463	372	810	820	954	577	522	450	1128	922	792	814	688	590	1002	631	657	465	1579	868	1138
212837_at	995	772	655	653	730	593	1159	1040	876	844	789	642	555	540	908	2203	2405	2376	1040	1369	1390
212838_at	1834	1193	1328	914	548	149	1349	1722	1033	1058	713	1119	403	377	376	1380	1486	1473	1062	691	1004
212839_s_at	213	125	153	102	287	117	170	228	194	124	153	113	58	52	60	131	64	146	56	76	51
212840_at	460	186	453	533	753	601	452	661	495	637	519	617	478	522	692	400	381	447	401	415	479
212841_s_at	12	13	315	874	44	66	15	13	380	312	30	23	91	50	61	42	8	61	629	451	771
212842_x_at	1506	1506	1380	1833	1913	1832	1408	1483	1092	1827	1983	1306	1132	712	777	1374	1171	1748	1742	1896	2427

212843_at	93	112	68	84	553	695	52	62	136	120	432	537	322	409	131	18	21	5	18	28	21
212844_at	466	462	326	216	1046	1083	608	464	554	449	654	418	162	160	20	96	50	84	104	59	121
212845_at	361	991	604	530	1333	859	409	348	583	531	1910	2485	788	665	673	574	663	537	377	448	513
212846_at	3051	2854	1729	877	3781	3505	2545	2026	2296	1930	3183	2836	5406	3183	4742	4773	3935	4672	6360	3692	6145
212847_at	183	159	361	300	257	138	215	208	403	519	297	162	310	301	257	184	179	187	980	286	393
212848_s_at	337	320	224	431	3802	2900	272	310	193	316	2177	2051	5101	6513	5734	596	527	595	487	826	715
212849_at	82	494	1030	714	78	67	400	77	645	668	367	373	147	132	187	88	108	114	391	272	240
212850_s_at	251	187	88	13	253	39	201	238	29	112	138	192	60	62	51	87	67	108	5	42	51
212851_at	411	465	624	832	416	509	701	695	717	560	313	337	156	145	215	458	412	404	399	368	519
212852_s_at	2401	2710	1529	1371	1924	1902	2590	2367	1552	1368	2261	1159	1053	989	846	1178	973	753	1577	1709	1478
212853_at	407	182	415	555	203	313	428	574	593	601	425	368	86	64	120	197	221	342	198	148	263
212854_x_at	2974	3962	2663	3303	2276	4753	3653	4545	2166	2759	2885	4136	1987	1812	2187	2916	3001	3135	2174	3605	3539
212855_at	208	225	381	768	242	356	268	226	444	284	314	269	597	587	340	454	369	306	955	748	1008
212856_at	605	560	475	372	542	540	505	483	428	472	476	424	336	289	226	241	256	220	225	223	175
212857_x_at	6324	5396	8234	5579	6158	5707	6265	6979	6687	7606	5487	5814	9831	6282	6087	7990	6036	6619	12894	12372	14971
212858_at	692	753	452	577	955	430	582	622	899	1391	1066	645	462	282	485	269	221	207	443	262	292
212859_x_at	20790	18173	9973	8117	6609	9313	15162	20358	9664	6574	8904	16217	10678	8566	8409	14711	15443	13115	8042	7947	4746
212860_at	730	533	452	649	77	328	522	569	460	572	497	280	252	292	218	153	182	92	158	102	90
212861_at	786	781	558	931	689	865	537	619	858	720	1050	577	703	393	431	499	377	447	521	435	474
212862_at	741	569	479	592	796	543	1039	1122	630	611	1165	960	476	655	561	615	536	515	292	256	333
212863_x_at	3842	5027	2458	2500	2415	2004	2920	3477	2810	3103	2402	2251	2192	2644	3814	5540	5399	4949	4596	3493	2901
212864_at	452	536	354	433	1343	525	778	603	308	517	874	692	523	801	1075	832	1004	928	379	568	502
212865_s_at	33	5	20	81	11	55	12	13	27	15	57	53	7	1	1	1	21	11	6	17	3
212866_at	896	797	515	366	404	613	916	759	491	397	553	437	408	281	306	392	398	279	247	171	151
212867_at	659	646	288	671	345	657	501	599	276	354	385	338	439	503	358	1186	1134	842	1027	891	1378
212868_x_at	155	43	52	204	74	58	99	165	111	162	72	64	62	86	56	54	54	50	8	23	28
212869_x_at	36119	49757	34387	46245	44965	40642	39060	37008	38490	36401	30959	44131	23511	33451	41071	34689	39726	38583	21386	32697	28001

212870_at	275	540	331	499	489	687	208	421	92	256	445	549	357	373	554	474	448	455	265	540	728
212871_at	683	661	1078	450	488	380	761	534	911	871	706	440	454	393	344	252	323	279	426	331	370
212872_s_at	609	348	589	729	668	758	681	656	452	633	635	798	461	506	537	793	587	670	561	654	548
212873_at	245	743	231	166	236	414	83	88	421	358	384	407	265	339	289	200	199	178	208	264	195
212874_at	12	5	11	9	14	15	13	16	15	8	6	9	2	2	3	2	2	3	3	2	1
212875_s_at	1008	734	608	423	998	1525	876	619	745	689	775	589	359	514	555	639	601	488	542	491	401
212876_at	140	197	324	335	632	1133	297	288	312	397	287	353	263	283	299	158	246	224	435	433	276
212877_at	385	300	366	228	241	516	615	482	515	439	1059	951	526	457	392	204	163	96	292	129	204
212878_s_at	951	984	484	228	942	715	1052	1148	717	574	1386	1215	851	1142	472	487	504	385	285	233	236
212879_x_at	165	156	81	27	48	62	57	51	174	42	16	15	157	60	52	48	13	11	68	76	60
212880_at	442	364	347	393	111	23	338	433	302	389	233	232	93	150	101	275	280	318	212	180	185
212881_at	331	459	294	405	334	270	329	289	351	277	259	186	218	304	208	284	397	312	312	318	201
212882_at	659	490	662	507	794	735	706	544	827	681	655	535	436	470	365	616	474	476	598	405	430
212883_at	17	9	23	74	37	23	70	12	11	48	13	23	2	6	26	8	10	25	16	25	8
212884_x_at	259	206	510	737	1078	1872	241	336	462	492	725	523	317	330	357	366	219	135	235	293	160
212885_at	849	555	737	382	1041	1060	584	706	680	704	725	406	1254	993	1660	1454	1509	1671	1739	1332	1867
212886_at	45	69	19	26	231	441	113	77	43	10	249	334	241	187	190	103	82	93	22	52	16
212887_at	1640	1241	886	542	1550	1935	986	1367	648	1038	1124	1033	1600	1731	1142	2799	2754	2650	2469	1426	2464
212888_at	1123	628	709	735	1355	1408	600	610	743	463	812	865	741	1526	728	617	596	605	1032	967	1287
212889_x_at	53	40	31	83	38	32	30	120	157	103	65	77	3	7	10	26	3	3	10	6	3
212890_at	1871	2500	1104	1952	559	883	1651	1969	931	1807	941	990	304	433	347	776	1124	578	700	801	720
212891_s_at	766	400	407	277	800	1154	677	565	574	447	711	519	1663	1096	259	350	314	350	320	176	130
212892_at	642	501	841	851	716	675	645	670	821	805	810	299	133	169	249	233	233	279	309	335	352
212893_at	1427	1310	1226	1114	1443	1748	1090	1496	1101	1220	789	871	981	865	1149	1875	1795	2032	2073	2589	3554
212894_at	1340	1179	1343	791	1292	1716	1169	957	1135	1071	732	772	456	668	1153	1134	1096	1102	1171	1266	1452
212895_s_at	1511	2005	2473	3436	3381	2620	1011	1128	1868	2060	2857	2459	1325	1077	779	624	873	551	1178	1165	995
212896_at	995	893	1762	1363	1682	1168	1883	1657	1779	1501	1337	1051	1201	1192	1086	2242	1784	2135	2420	1739	2551

212897_at	336	332	194	666	289	543	298	553	175	741	370	701	133	263	121	113	144	118	173	189	279
212898_at	1279	865	1468	1107	1326	1310	1218	1454	1627	1876	1096	1058	956	787	659	683	556	962	1405	797	1085
212899_at	775	701	747	1419	856	839	545	1190	435	915	884	1313	686	792	797	821	854	506	580	1056	969
212900_at	441	382	754	768	454	1141	486	509	389	416	505	718	786	759	933	969	711	841	1447	1869	2299
212901_s_at	291	134	286	480	137	75	207	513	297	236	206	234	212	152	87	234	356	312	319	192	146
212902_at	343	258	408	326	375	497	478	404	470	370	561	390	205	294	668	518	452	733	436	475	724
212904_at	3302	2545	1637	1674	2955	2117	3026	2610	1888	1628	2764	1859	4418	3195	4179	5028	5343	6711	4197	3248	3573
212905_at	560	423	376	669	330	398	464	615	343	542	323	372	260	150	221	474	469	396	545	660	686
212906_at	520	404	1393	1115	232	378	432	466	1228	1003	219	211	72	57	82	152	114	205	622	953	708
212907_at	652	923	1012	829	786	503	641	864	937	759	962	974	967	1217	1307	1693	1953	1490	1660	2634	3159
212908_at	720	904	606	559	414	503	962	1085	416	671	445	522	351	311	320	654	567	689	397	586	536
212909_at	20	49	16	19	36	13	17	22	20	31	23	67	16	9	19	5	42	6	6	29	4
212910_at	734	732	700	716	1016	876	579	731	1048	751	1018	824	916	809	776	537	466	521	1285	1365	1010
212911_at	381	380	197	257	280	474	510	449	181	260	162	171	168	113	78	247	343	409	274	292	339
212912_at	153	417	4	115	52	636	209	228	60	50	134	182	72	129	107	244	287	185	4	74	58
212913_at	398	364	210	175	67	157	270	397	238	266	207	163	75	45	55	180	125	127	260	127	257
212914_at	368	482	28	305	400	469	252	380	31	55	227	464	230	345	164	268	222	183	78	135	110
212915_at	4	2	23	79	7	17	4	1	51	50	44	46	39	29	164	7	7	12	218	177	264
212916_at	333	266	404	257	460	718	326	263	262	347	367	341	275	127	144	161	100	163	261	196	221
212917_x_at	271	175	610	370	1480	691	299	417	478	858	858	1255	2573	1513	790	231	230	300	1173	795	1298
212918_at	365	182	780	581	1873	1203	404	497	886	925	1256	1633	2608	1954	1916	692	559	649	2231	1783	2112
212919_at	972	731	1295	1636	735	759	1126	1000	1001	1241	772	559	632	1775	664	2008	2014	1857	3107	1623	2200
212920_at	407	321	418	270	478	953	245	315	239	328	406	603	630	398	342	537	439	484	843	732	799
212921_at	93	142	180	163	125	136	126	169	70	179	148	74	31	12	86	124	90	92	153	117	134
212922_s_at	1308	1707	2287	1527	1517	1700	1638	1454	2301	2678	2409	1636	2012	2169	3386	1600	1641	1448	2582	1765	1660
212923_s_at	324	348	365	415	355	947	240	162	186	215	470	322	389	410	404	155	275	167	141	264	146
212924_s_at	17	9	75	39	16	40	9	41	19	21	18	9	7	30	29	78	56	55	44	73	9

212925_at	1328	1163	3295	5048	1196	1062	1084	1239	2711	2442	1714	831	646	550	715	606	708	769	1286	1373	777
212926_at	193	162	140	138	364	380	224	282	218	275	347	334	232	172	136	127	136	75	102	95	99
212927_at	538	185	281	215	525	376	219	404	229	507	361	418	624	313	406	480	396	398	791	619	710
212928_at	427	563	563	1031	691	955	466	678	531	749	549	552	639	612	549	791	685	766	1013	807	1005
212929_s_at	541	457	429	479	575	738	322	525	381	349	462	407	883	741	728	512	508	536	1070	1509	1863
212930_at	156	232	221	197	5	51	155	186	274	278	54	98	127	132	101	432	459	295	721	571	1048
212931_at	873	1045	738	1405	939	1105	918	860	1114	1263	877	802	736	462	340	354	304	342	427	417	188
212932_at	339	127	305	528	477	610	371	385	451	593	505	431	378	324	163	129	150	149	243	218	264
212933_x_at	20537	28496	17483	17713	9585	12750	18767	20537	12507	15976	9040	15340	14826	15744	15694	20069	20969	22672	13718	17081	15475
212934_at	681	692	437	410	401	618	533	693	199	394	284	582	440	329	308	632	590	576	788	806	1392
212935_at	46	41	57	473	33	38	40	50	41	46	21	25	11	61	33	41	23	40	18	221	314
212936_at	400	533	244	497	436	883	323	387	201	284	226	308	421	272	372	676	646	300	388	383	405
212937_s_at	179	255	18	71	302	341	250	302	66	14	246	122	9	3	26	8	20	17	13	17	3
212938_at	83	91	106	58	399	388	215	241	32	113	97	98	36	46	52	36	36	47	64	46	19
212939_at	30	18	16	27	25	40	20	13	25	18	26	15	10	4	5	8	15	8	8	10	12
212940_at	275	313	122	211	33	44	407	401	96	171	355	210	10	12	18	3	4	2	2	6	10
212942_s_at	656	238	3268	2140	430	254	580	501	3028	5157	271	157	114	67	531	218	247	250	1925	2154	2874
212943_at	467	420	739	857	1714	1601	619	872	617	1447	1698	1928	2629	1959	2968	1141	959	1408	2797	3307	5396
212944_at	486	713	802	2338	6444	6942	681	796	1230	1483	7512	5205	7583	7350	8006	930	701	863	1668	2177	2375
212945_s_at	180	311	277	198	372	461	372	577	191	214	449	407	216	282	325	335	270	295	235	177	184
212946_at	864	675	775	784	988	1017	867	720	748	941	686	592	350	470	1078	1136	906	1091	1147	1551	2308
212947_at	242	248	343	341	238	121	127	306	277	411	281	199	229	155	17	104	140	179	258	199	280
212948_at	489	726	659	406	620	855	453	474	437	503	577	436	318	179	363	262	224	289	185	302	257
212949_at	983	146	925	35	874	19	717	896	749	983	765	946	788	709	374	155	161	290	613	118	128
212950_at	79	96	114	53	113	7	99	112	59	92	50	96	28	15	21	17	6	47	19	26	15
212951_at	34	10	14	22	26	35	12	22	24	13	14	11	5	4	40	5	7	7	4	6	3
212952_at	329	504	1435	1743	981	1379	1104	1077	1959	2145	2191	3014	547	448	880	811	855	1291	1639	1293	1277

212953_x_at	7514	4632	8008	3238	2246	2000	5199	8173	3627	3860	3856	2231	1972	1522	2036	3713	4944	4069	2947	5431	4528
212954_at	468	471	225	39	327	521	482	457	28	176	382	388	493	394	1017	537	589	513	204	335	299
212955_s_at	3168	3558	2599	2139	2421	2987	3222	2697	2791	2186	4016	3591	5130	3302	3512	2309	2365	2105	3322	2572	1326
212956_at	620	1867	391	991	419	277	590	753	329	593	382	459	219	351	329	921	1173	893	503	567	868
212957_s_at	58	56	66	297	26	113	38	94	63	32	232	54	111	151	116	86	127	156	100	235	264
212958_x_at	2094	7843	1616	3131	3321	3714	2382	3791	1335	1652	2798	2036	3802	4085	5811	3610	3270	3876	2006	2907	3450
212959_s_at	1404	837	874	1060	888	934	1208	1108	821	941	433	610	655	440	386	1147	1078	1366	1696	1459	1841
212960_at	237	399	141	312	540	177	299	222	199	217	213	261	41	52	84	47	92	15	67	59	42
212961_x_at	980	1016	2568	2550	1120	1510	888	735	2237	1817	1670	1357	1591	1320	1329	723	792	705	1728	1764	1464
212962_at	67	55	14	16	23	15	16	33	15	25	17	11	4	34	4	8	19	9	6	7	7
212963_at	152	173	229	200	14	75	162	187	132	210	129	63	7	49	151	215	170	144	72	107	105
212964_at	345	361	172	228	399	452	401	257	177	249	364	439	174	189	150	322	294	311	241	244	187
212965_at	46	144	122	9	209	241	102	139	92	46	90	45	39	56	27	44	27	5	25	19	23
212966_at	458	408	227	122	158	114	504	582	231	232	202	167	103	92	92	136	133	131	134	185	99
212967_x_at	7176	10479	11759	10896	5861	7382	8125	9547	9721	11333	7164	5883	9173	8951	9794	13853	12732	11412	13444	13767	15572
212968_at	585	1097	369	735	441	707	837	644	428	365	549	288	334	417	396	459	587	384	292	441	232
212969_x_at	433	888	1002	1062	342	442	1151	899	995	882	564	290	120	114	195	199	180	183	422	280	262
212970_at	679	848	562	493	947	1478	780	798	502	512	649	818	354	344	419	810	806	746	186	316	312
212971_at	6449	5949	4214	2497	4672	9180	5128	2391	2984	1595	3084	2776	4209	4506	10753	10401	12741	10271	4008	11339	9230
212972_x_at	261	231	457	216	283	220	423	420	373	326	306	324	107	94	231	245	200	149	132	192	147
212973_at	1912	1416	1279	649	1296	1424	1933	1215	1869	1317	1688	1049	2338	1418	1954	2335	2233	2253	3110	1343	1343
212974_at	171	231	302	516	337	339	151	198	262	284	239	273	32	45	44	45	45	17	69	56	57
212975_at	254	293	349	687	432	452	258	355	385	407	351	405	44	48	170	66	60	82	36	57	69
212976_at	12	36	102	13	15	9	4	59	70	63	4	16	32	30	49	135	125	99	250	147	191
212977_at	32	26	34	83	11	130	16	67	8	34	23	36	29	20	16	3	17	2	7	5	25
212978_at	479	384	1362	588	212	130	829	662	1222	1344	263	241	126	123	180	1199	1188	1022	1851	1030	1360
212979_s_at	352	363	1119	948	676	466	535	868	864	1163	458	801	867	374	156	189	140	199	710	458	503

212980_at	86	114	145	221	113	13	76	128	132	136	65	96	45	30	19	66	45	61	81	79	92
212981_s_at	511	532	657	560	977	1117	219	661	460	746	344	689	847	838	667	744	659	627	2739	2267	2569
212982_at	491	583	477	477	646	895	716	777	317	650	304	368	459	487	300	925	830	806	623	460	697
212983_at	1622	1116	901	328	895	437	2011	1330	1288	700	1798	542	2189	1433	604	396	485	364	540	325	193
212984_at	283	262	480	459	576	954	103	268	214	314	236	363	913	678	555	787	664	689	1259	1439	2144
212985_at	681	984	450	164	1134	1587	788	930	201	175	830	892	630	341	457	1342	1238	1164	243	358	463
212986_s_at	1380	1176	821	845	1100	933	1549	1370	959	925	1064	985	368	438	309	504	521	506	313	435	399
212987_at	1746	2298	1028	1860	1053	1169	1779	1776	1133	1115	1211	940	1599	1757	2477	5448	4960	3726	3546	3125	5272
212988_x_at	34116	40300	30067	23399	29384	28274	31966	32437	29504	28338	24281	36138	21430	25273	30682	28599	29655	31113	17704	23802	22719
212989_at	450	479	233	394	390	512	282	256	159	282	281	395	464	273	231	416	310	415	585	731	1177
212990_at	183	182	164	124	427	367	178	127	124	115	399	397	399	461	408	192	194	181	229	275	380
212991_at	105	112	179	365	188	292	236	201	333	316	190	191	41	68	68	167	161	96	170	133	128
212992_at	849	866	1238	736	543	1552	815	1115	405	1576	694	829	156	247	337	315	576	346	205	741	339
212993_at	995	1199	775	971	959	778	774	738	642	538	820	643	1089	547	786	1025	880	751	1064	704	658
212994_at	419	373	731	645	922	501	770	918	1080	1435	1181	696	569	599	357	252	267	290	411	501	387
212995_x_at	4191	4376	2382	1527	2552	1982	3901	3613	3042	2440	3489	4358	4800	3964	1661	1709	1309	1128	1135	1174	576
212996_s_at	278	283	184	54	331	134	511	379	304	309	634	378	282	262	534	304	308	431	282	244	221
212997_s_at	892	796	495	521	904	270	645	934	542	586	475	584	676	494	590	829	693	1124	545	653	996
212998_x_at	13	79	7	13	37	167	20	17	13	18	354	421	145	139	105	18	67	15	2	1	1
212999_x_at	7	10	5	9	291	309	5	40	5	4	209	366	148	203	132	31	60	26	1	1	1
213000_at	356	235	193	294	518	770	187	276	151	169	303	260	633	452	347	578	405	585	599	528	904
213001_at	333	147	362	371	378	160	311	256	415	398	247	270	48	33	31	45	10	47	49	49	33
213002_at	348	161	361	391	203	200	409	424	486	467	236	315	117	155	70	209	255	188	481	224	198
213003_s_at	112	131	129	160	265	231	94	55	52	127	138	63	51	36	139	69	44	76	25	31	46
213004_at	118	83	92	119	279	289	117	80	128	113	81	93	11	62	40	36	7	41	82	17	54
213005_s_at	550	804	632	405	235	680	655	491	652	622	236	268	168	151	164	639	580	612	1257	1033	1302
213006_at	57	68	81	62	126	60	28	48	64	41	53	45	52	69	49	28	25	11	27	19	16

213007_at	929	539	2025	516	1397	413	1187	1223	2090	2254	1197	1805	1761	1687	2225	1977	2017	3105	6605	3526	4386
213008_at	295	161	1670	287	671	42	981	1046	2565	2281	1258	1239	1066	629	629	353	383	462	1602	819	1009
213009_s_at	2742	3254	1876	1610	2173	2473	2777	2523	2133	2182	1808	1928	2308	1964	2276	3463	3952	3789	2814	2683	3339
213010_at	435	169	73	113	1105	1309	200	132	33	123	850	1108	1370	1245	667	159	238	177	69	225	152
213011_s_at	11486	7832	12816	9222	8921	5312	10213	9497	11043	9792	11181	7718	17388	15630	12976	10844	10872	10468	9893	10177	7686
213012_at	155	142	563	374	628	332	221	190	318	275	614	583	421	624	704	226	224	238	551	501	736
213013_at	266	236	168	76	142	132	178	259	103	117	109	132	51	45	13	19	18	4	18	26	12
213014_at	57	67	60	102	243	310	87	135	112	56	88	77	17	26	29	54	23	26	41	27	27
213015_at	246	196	391	544	400	257	417	463	502	349	398	418	511	506	549	415	352	350	489	935	966
213016_at	537	271	950	1225	598	739	694	682	937	594	799	488	455	356	293	406	344	420	426	505	679
213017_at	2387	3792	726	702	2316	1079	1093	1624	583	611	797	2077	1810	2103	592	2107	1606	1118	1417	1118	1800
213018_at	545	616	948	975	1046	1633	489	508	512	777	1002	735	545	706	752	350	398	419	640	571	693
213019_at	390	472	537	490	540	723	417	500	447	449	408	421	774	750	586	778	562	690	873	730	785
213020_at	511	421	604	607	410	551	482	484	403	422	364	312	330	305	221	555	630	507	682	580	817
213021_at	758	953	1030	899	598	735	969	879	1331	1038	875	747	605	483	625	867	700	761	1086	912	1538
213022_s_at	225	230	313	372	268	148	388	457	296	502	306	162	44	10	8	61	20	28	114	84	100
213023_at	149	107	128	181	51	173	143	132	143	85	95	106	22	39	56	35	16	4	46	56	58
213024_at	304	245	331	304	521	630	193	284	272	233	253	271	457	290	301	262	184	235	492	526	902
213025_at	650	534	688	757	397	293	563	862	731	706	362	358	589	521	683	1467	1262	1137	2401	1225	2179
213026_at	1664	1865	841	627	848	1041	1815	2436	523	832	1067	992	2426	2981	2899	5126	5187	5612	1575	2176	2890
213027_at	923	663	556	619	455	692	973	1111	492	527	581	402	395	487	802	1391	753	894	906	887	980
213028_at	467	358	594	687	172	579	518	571	797	710	712	704	648	496	502	312	290	302	484	389	524
213029_at	216	276	1062	1260	260	267	363	350	902	1019	236	163	36	54	86	304	265	247	1227	1368	1070
213030_s_at	112	150	715	665	66	114	76	143	627	826	71	107	7	9	5	55	31	43	238	224	171
213031_s_at	894	612	1214	1504	921	732	988	727	1498	1258	709	620	736	605	580	670	956	894	1283	1012	872
213032_at	22	86	308	388	27	42	113	120	381	306	87	28	28	10	70	198	163	152	1023	992	1077
213033_s_at	11	11	76	59	18	19	13	12	78	151	7	10	3	9	17	103	32	62	606	471	504

213034_at	627	751	524	625	617	903	555	478	500	535	605
	654	423	493	399	608	707	549	509	664	697	
213035_at	319	272	788	1084	522	336	268	339	892	1034	622
	586	708	1371	934	628	603	428	2387	2211	3099	
213036_x_at	37	192	38	949	91	132	38	48	25	53	27
	23	62	86	111	194	265	218	117	127	98	
213037_x_at	3990	3719	4784	6456	6699	5265	4049	3370	6348	7079	7194
	8389	7242	7662	11896	7676	6818	6595	6829	9387	10306	
213038_at	750	1105	633	402	401	606	590	571	345	534	490
	680	383	287	515	748	585	524	490	436	453	
213039_at	1164	2174	2910	1672	1711	1975	1015	1255	1486	2426	1893
	1873	1331	804	842	720	995	612	1688	972	743	
213040_s_at	98	223	11	14	331	257	48	54	8	6	28
	65	58	154	66	207	243	231	6	61	43	
213041_s_at	4277	3302	2078	1292	1560	793	3256	2611	2387	2004	2051
	719	2599	1816	1641	3676	3556	3643	2572	1977	1120	
213042_s_at	33	15	11	36	11	11	27	11	23	13	50
	19	3	2	11	5	10	21	3	5	2	
213043_s_at	937	1123	595	749	689	484	1196	800	805	661	1005
	445	399	382	301	317	311	513	379	224	221	
213044_at	1820	1754	1816	1822	3234	3643	1953	2155	1548	1698	2291
	1536	2168	2072	3064	3387	2743	3009	2739	4127	4984	
213045_at	345	269	88	268	180	339	242	387	193	316	273
	172	79	116	175	209	195	223	129	122	195	
213046_at	623	689	514	813	452	523	510	596	484	507	377
	258	148	140	174	145	192	97	181	184	121	
213047_x_at	5546	6297	9255	7414	6172	5930	8863	8515	10548	8690	8948
	7962	12973	12654	14011	11281	13189	10072	10856	12183	11122	
213048_s_at	1499	3860	13815	11927	14021	11056	4720	4268	18069	13731	
	12829	14093	3320	5805	11783	3058	2272	2137	10104	10253	10395
213049_at	462	535	473	489	462	570	457	529	332	387	270
	623	258	223	253	466	455	472	470	516	769	
213050_at	523	939	490	709	988	1043	504	527	621	553	1182
	954	821	640	1019	515	527	355	725	841	959	
213051_at	403	522	1225	2301	1559	1449	779	726	1561	1620	1497
	1162	872	794	1056	76	19	107	1434	2056	2552	
213052_at	1595	1116	2074	1526	2551	2313	1741	1553	2523	1736	2948
	2138	1772	2013	2285	1322	1157	999	1790	1664	2108	
213053_at	299	114	163	136	506	400	239	216	269	247	202
	241	157	211	89	97	172	151	200	76	90	
213054_at	270	163	190	141	37	161	172	182	187	186	165
	98	186	202	323	248	266	328	465	307	543	
213055_at	221	236	392	370	60	52	464	612	376	429	412
	328	80	16	22	49	26	17	137	87	117	
213056_at	110	103	99	193	221	110	66	99	72	177	111
	88	55	45	74	107	105	120	272	147	296	
213057_at	323	355	167	447	326	688	289	236	122	218	371
	342	458	393	187	203	124	149	97	112	78	
213058_at	251	161	309	217	143	239	239	227	242	432	158
	239	35	18	62	27	39	35	39	29	29	
213059_at	66	64	431	830	217	71	82	75	749	1145	51
	69	35	8	17	13	15	15	562	1669	1536	
213060_s_at	108	151	193	246	346	388	154	210	290	182	134
	225	23	7	57	42	33	65	47	31	52	

213061_s_at	587	545	327	268	506	349	505	753	254	222	419	465	790	645	511	469	456	354	399	288	226
213062_at	466	444	213	233	674	383	567	565	131	95	350	420	523	380	482	602	505	391	441	248	212
213063_at	602	788	494	473	646	618	954	847	436	477	566	553	738	692	1109	1067	943	891	837	835	1428
213064_at	1503	994	940	985	1504	1325	806	1099	710	925	1049	1387	1220	866	800	1208	867	995	1409	1110	2045
213065_at	658	586	1031	1278	1075	1308	808	945	1045	1152	900	672	394	462	614	539	505	533	686	747	1144
213066_at	69	157	79	177	181	118	85	57	33	82	81	39	54	45	53	41	69	67	24	29	44
213067_at	36	18	31	30	157	97	227	306	280	124	152	185	248	220	54	85	44	44	69	61	45
213068_at	45	49	11	48	19	15	16	36	47	13	104	45	25	9	14	14	5	15	5	3	18
213069_at	13	40	43	17	1302	2125	16	17	12	82	1305	1305	909	1407	730	75	73	51	64	74	143
213070_at	1373	1114	2345	1627	2339	2638	962	1239	1405	1417	1816	1097	2669	2450	3491	2384	1958	1995	4151	4517	6665
213071_at	20	6	14	13	34	17	21	21	19	18	18	16	35	19	9	2	5	3	6	2	7
213072_at	198	254	194	371	260	331	260	259	198	211	185	146	116	13	59	57	40	67	36	41	49
213073_at	208	221	260	336	471	1298	193	215	183	330	431	436	305	512	384	107	122	88	229	505	288
213074_at	725	989	1350	1324	1372	840	946	973	1044	1096	1083	573	903	1051	1276	1123	720	1077	1931	1911	2142
213075_at	218	359	152	89	609	1075	420	285	167	61	458	498	133	196	54	100	57	8	14	14	3
213076_at	487	426	487	592	672	652	419	438	385	482	571	373	152	116	227	105	116	146	119	142	104
213077_at	495	612	553	384	535	653	776	1083	649	651	559	606	601	354	488	621	419	614	1227	661	961
213078_x_at	491	376	697	1147	158	254	494	405	830	1160	385	256	102	171	254	268	248	225	748	312	409
213079_at	607	311	777	582	1612	869	590	649	920	928	1153	917	1862	1080	740	304	179	123	429	231	189
213080_x_at	19765	23939	17509	18899	20254	26062	17999	18118	19598	14437	12276	16191	20879	21829	19041	24212	21004	22557	19744	26143	23321
213081_at	251	398	179	230	62	106	197	261	118	147	158	143	14	52	129	83	93	53	48	29	20
213082_s_at	312	571	473	569	592	656	400	369	400	569	622	641	604	879	759	448	584	379	369	567	393
213083_at	685	663	746	713	1078	1204	546	742	423	889	738	675	944	1917	1845	1577	1822	1180	1102	1556	1190
213084_x_at	32177	48112	32981	43995	39840	39947	36658	37880	38643	32802	29905	46994	26089	38842	43998	39272	42204	45040	23593	33614	29593
213085_s_at	1147	882	1156	1328	1431	1224	1116	821	1472	1448	1769	1120	1088	1341	1968	958	1109	817	1780	1659	1602
213086_s_at	3527	3214	2683	2161	3227	3283	3963	3300	2551	2170	3944	3394	4076	4039	4912	4405	4345	5022	4030	5453	7695
213087_s_at	306	578	146	81	330	281	282	253	185	171	193	109	75	133	208	244	206	147	164	167	71

213088_s_at	2958	2293	3361	1395	2954	1001	3638	3302	4097	4161	3594	3684	5754	4773	4807	4228	4972	3499	6985	3286	3246
213089_at	438	275	483	533	351	445	415	771	510	493	260	225	53	59	45	83	31	131	111	169	38
213090_s_at	561	794	1324	949	909	754	702	830	1173	1509	1049	1054	975	289	581	746	505	625	2087	2026	3523
213091_at	123	223	77	246	66	67	117	214	79	127	63	49	7	14	21	40	37	9	14	14	19
213092_x_at	843	558	728	317	948	710	688	492	751	739	610	961	1661	1422	1760	1385	1336	1279	2955	1685	1894
213093_at	566	853	822	877	606	1605	602	629	908	903	760	836	619	595	351	573	515	345	866	626	715
213094_at	616	561	1264	385	98	39	636	884	305	590	97	90	54	32	62	2560	1611	1894	873	1071	1365
213095_x_at	25	18	16	21	23	27	23	21	23	22	17	15	3	9	6	8	4	9	6	4	3
213096_at	266	133	60	145	910	986	146	156	138	127	148	261	113	24	29	49	95	16	14	26	84
213097_s_at	1916	1443	2139	979	3662	2169	1361	1098	2349	1588	4468	1423	5786	6269	7534	3587	3482	4858	4727	3812	4832
213098_at	501	353	122	41	98	30	545	412	334	179	247	259	305	329	220	304	164	293	260	113	128
213099_at	508	460	468	344	1177	706	663	903	278	423	1103	964	321	300	461	282	275	345	290	203	323
213100_at	429	367	288	101	400	317	386	433	82	41	135	41	56	45	44	146	159	149	33	82	113
213101_s_at	10617	8153	12344	9274	12991	12362	9561	9366	11712	10182	11843	9134	12915	12386	14525	10846	9910	11040	11163	10946	12676
213102_at	2724	2334	3397	2360	3406	3077	2662	2428	2307	2600	3466	2757	5075	4927	6066	4395	3941	4557	7154	4770	6573
213103_at	101	165	81	132	25	55	172	205	112	159	135	163	38	45	116	68	31	38	6	16	19
213104_at	632	870	724	613	594	551	726	513	1007	908	648	356	645	421	221	238	165	169	367	204	110
213105_s_at	827	859	583	535	623	324	443	599	724	514	505	473	807	412	618	537	649	503	891	639	367
213106_at	36	85	107	172	125	140	61	86	74	144	44	59	28	19	44	34	27	21	168	124	186
213107_at	614	437	832	873	1108	1084	405	474	622	1458	682	539	621	818	1002	1124	1036	951	1370	831	1095
213108_at	138	40	146	207	142	152	29	48	108	138	68	65	2	6	9	52	35	61	11	49	17
213109_at	335	165	477	495	562	325	368	368	557	1068	489	308	265	280	68	104	105	102	125	138	149
213110_s_at	623	798	43	71	73	22	507	602	43	85	24	55	2	25	27	465	523	410	15	19	14
213111_at	345	302	556	422	745	912	288	480	387	456	409	462	539	669	606	931	821	808	1159	1113	1437
213112_s_at	581	800	336	566	323	1195	265	150	52	155	306	206	438	551	476	378	483	348	177	441	344
213113_s_at	964	595	343	469	716	504	975	713	213	252	893	703	273	135	247	433	491	572	118	530	613
213114_at	217	178	235	238	146	52	292	249	194	169	155	162	72	139	210	180	97	139	126	155	74

213115_at	405	294	559	665	766	845	645	467	553	331	527	454	275	425	348	323	463	366	342	458	454
213116_at	275	236	323	428	891	1216	338	319	472	338	730	700	440	410	990	298	237	290	519	530	737
213117_at	299	281	217	473	372	270	303	558	209	390	230	263	241	253	215	370	307	317	331	235	358
213118_at	146	113	183	285	294	181	80	210	264	231	198	189	398	314	284	277	312	291	705	775	1141
213119_at	271	186	171	103	536	359	342	292	241	368	459	368	397	567	659	262	162	335	181	170	145
213120_at	87	41	73	71	92	91	76	42	71	84	77	65	16	17	37	36	32	15	111	67	35
213121_at	95	103	14	150	32	24	62	125	80	126	85	10	5	7	21	15	7	10	5	12	3
213122_at	119	29	106	72	166	223	99	79	153	70	77	69	12	36	15	5	26	5	27	25	15
213123_at	507	479	735	713	709	875	484	501	526	534	698	529	625	532	774	882	655	753	973	773	1069
213124_at	344	420	650	332	533	433	400	467	508	593	648	682	475	383	404	307	298	316	361	369	417
213125_at	169	105	99	172	169	314	197	195	114	191	139	155	16	12	27	45	84	39	38	68	50
213126_at	167	75	389	137	313	191	126	41	249	323	183	167	439	336	257	231	164	173	351	367	349
213127_s_at	337	382	817	538	576	595	311	388	601	614	519	432	709	826	684	452	557	357	721	799	882
213128_s_at	696	583	643	652	707	1047	596	392	512	626	367	315	1187	696	673	991	736	1154	2904	1868	3311
213129_s_at	5892	4832	3741	2099	1970	2455	4753	5195	4493	3796	2039	1694	3586	4124	3721	8142	7628	7909	9675	7618	8004
213130_at	247	230	217	248	429	278	375	339	305	337	240	282	178	140	59	85	50	114	147	139	132
213131_at	164	79	34	76	510	382	148	190	100	110	728	716	550	465	279	77	48	27	25	10	3
213132_s_at	765	692	591	578	363	160	995	756	676	535	905	567	786	547	561	448	642	520	456	426	298
213133_s_at	4403	4584	3250	1415	1860	2258	6075	5145	4620	4162	2312	2289	2055	2040	1479	4474	4795	4248	4626	2670	2951
213134_x_at	3590	3556	770	379	2169	2595	2825	2638	569	917	3043	2502	3655	3916	4083	4400	5629	4946	1053	493	552
213135_at	1495	558	114	110	1724	1207	1301	1486	115	98	1421	1250	1403	1425	1354	2228	1962	1792	28	11	21
213136_at	1382	1395	1369	1491	1884	2243	1114	1007	1442	1314	1538	1364	2072	2280	4359	2673	2017	2684	2871	3718	3940
213137_s_at	558	699	1078	982	1544	1516	853	734	1391	1420	1393	1311	1030	1115	1192	1053	952	1241	1091	1201	1564
213138_at	71	170	210	30	54	171	162	158	100	52	90	136	88	75	98	66	77	45	102	112	96
213139_at	75	101	14	35	283	1758	54	62	41	32	168	172	147	212	240	20	61	87	28	83	90
213140_s_at	562	608	709	524	591	587	445	707	836	894	534	630	381	330	274	382	257	239	648	415	453
213141_at	369	433	430	638	322	517	604	355	416	600	459	298	184	94	179	164	148	155	216	229	159

213142_x_at	74	15	279	269	492	625	38	67	330	300	352	418	254	254	525	1	25	1	335	639	835
213143_at	188	123	138	231	194	294	50	102	167	225	99	82	45	43	10	49	85	39	257	234	191
213144_at	270	78	400	231	267	465	533	409	499	584	925	495	310	157	258	94	83	109	203	159	164
213145_at	927	1058	1031	626	1517	1520	943	1144	607	864	1389	1283	1238	932	1109	891	733	733	749	1283	1405
213146_at	132	339	271	282	197	310	97	238	190	158	166	145	42	39	103	81	47	107	61	100	75
213147_at	586	602	924	1234	576	605	800	908	1054	1149	456	449	291	301	343	329	247	361	517	525	278
213148_at	41	73	81	307	180	34	87	80	127	150	87	24	49	28	52	16	37	30	158	82	120
213149_at	1197	803	794	596	735	582	869	753	779	709	875	799	918	833	1197	1984	1257	1665	2033	1757	2378
213150_at	1019	1401	1348	2083	1067	1301	669	996	1516	1335	513	490	579	799	1126	1515	1232	987	4120	1685	1548
213151_s_at	2532	3247	4270	3735	4092	3079	4528	4551	4077	3583	4654	3915	5579	6332	8342	7526	7331	7699	7496	5536	7277
213152_s_at	1156	1080	1142	860	492	782	1076	845	1086	1251	884	780	897	780	696	786	690	605	693	726	978
213153_at	915	777	720	647	816	743	1128	868	761	615	893	730	661	631	1019	790	865	1176	829	1607	1052
213154_s_at	912	918	690	481	1243	1587	754	768	840	619	1293	1009	1420	1356	901	1075	1002	829	1321	892	686
213155_at	29	9	33	31	37	60	28	30	40	29	27	24	4	8	6	7	8	8	5	5	3
213156_at	581	826	81	212	51	125	428	490	48	82	21	36	2	9	18	690	672	718	13	129	92
213157_s_at	30	29	26	32	445	286	25	178	139	52	149	25	22	12	15	3	8	10	19	42	33
213158_at	470	991	118	367	107	118	396	487	98	123	45	15	17	36	36	928	824	885	22	129	98
213159_at	185	150	165	177	85	91	252	253	191	239	249	222	87	88	88	76	64	79	77	83	78
213160_at	13	16	8	19	113	35	5	18	12	13	11	5	2	21	17	3	11	6	2	4	7
213161_at	528	720	629	671	493	812	567	702	895	574	676	626	625	450	505	589	584	746	613	674	860
213162_at	172	18	155	217	135	87	24	153	151	120	149	99	16	27	37	31	3	37	32	40	30
213164_at	127	77	1146	2218	7460	7902	203	422	1456	1166	7667	5163	6376	5295	3494	72	69	35	670	967	706
213165_at	589	654	727	673	998	738	584	682	478	523	752	687	656	776	971	952	836	1120	758	1613	1845
213166_x_at	4124	4930	2387	1820	2082	1387	4305	3562	2994	2495	3341	4082	5105	3363	1652	1661	1581	1250	1306	1395	745
213167_s_at	58	43	99	166	685	1153	136	166	248	179	1077	656	218	112	57	29	29	23	66	41	59
213168_at	1660	1455	1310	1846	1803	2313	1891	1655	1218	1551	1914	2065	2600	2050	2799	3359	2676	3357	3740	2863	3989
213169_at	164	49	94	190	400	144	93	195	150	221	209	62	25	38	50	6	5	40	13	30	64

213170_at	176	96	157	176	227	305	155	145	123	126	286	261	171	127	128	40	24	15	24	34	12
213171_s_at	26	26	58	371	43	48	30	38	44	36	30	44	7	8	21	10	10	7	53	128	80
213172_at	12	72	8	146	203	134	81	32	21	11	185	182	160	116	104	79	53	13	13	37	41
213173_at	124	82	152	105	245	464	179	150	120	131	149	239	89	78	98	88	64	105	132	114	104
213174_at	19	164	14	189	456	527	13	94	24	13	371	466	115	55	43	31	4	5	7	5	28
213175_s_at	8764	6776	7345	2728	7256	3474	9120	7865	7056	6183	10220	6357	12293	9565	8480	6721	6229	5908	5200	5033	3134
213176_s_at	229	375	92	63	52	69	372	453	29	161	266	62	9	7	12	80	32	12	11	6	7
213177_at	16	99	99	75	106	93	13	98	12	50	72	10	5	34	11	8	28	8	8	14	4
213178_s_at	433	601	359	455	366	447	355	487	349	430	448	292	155	91	198	277	221	287	150	212	184
213179_at	1152	892	830	691	419	316	1720	1808	785	811	898	504	1159	1544	1878	1718	1725	1635	2404	1448	1417
213180_s_at	615	274	437	512	524	618	590	546	484	566	597	572	354	253	139	200	179	321	165	157	163
213181_s_at	171	86	18	74	293	241	50	111	23	20	20	20	23	4	19	112	109	96	21	29	24
213182_x_at	98	264	8	180	544	421	167	276	15	8	222	80	150	152	90	149	130	76	39	83	27
213183_s_at	243	175	27	116	179	446	163	236	108	124	144	116	91	82	297	185	191	140	119	129	40
213184_at	695	591	726	754	1112	648	494	643	920	864	1202	1008	1099	1421	1457	1035	1232	876	1192	1013	1463
213185_at	816	906	853	1393	1208	974	710	924	827	971	1127	1024	345	412	381	275	263	279	258	298	240
213186_at	663	634	326	394	419	736	423	582	269	302	243	260	196	148	132	465	351	402	264	225	310
213187_x_at	21015	26090	16314	29124	17234	34783	23512	20010	17888	14612	16197	16693	19199	13820	14810	13767	7396	12820	11172	13156	13799
213188_s_at	1721	1348	1261	751	953	1320	1214	967	1024	1006	539	672	739	788	891	2345	1827	2455	2451	2308	2847
213189_at	1697	875	1249	738	504	939	1215	1049	1064	974	482	362	442	286	493	1268	1147	1264	1130	658	794
213190_at	324	386	313	557	529	359	355	113	206	211	90	127	86	273	269	289	245	331	325	307	444
213191_at	250	455	248	234	465	464	266	241	115	182	128	150	94	113	160	170	241	280	92	147	52
213192_at	95	146	35	12	43	19	123	98	21	32	37	33	113	80	77	77	110	99	49	97	19
213193_x_at	144	208	232	189	749	485	254	286	227	157	770	676	395	257	404	49	38	45	109	187	181
213194_at	22	2	26	43	65	65	20	15	12	29	30	15	38	49	50	35	16	1	84	297	266
213195_at	324	349	175	203	786	1084	269	348	190	210	316	430	106	92	125	57	43	120	48	64	45
213196_at	1119	724	937	1165	899	890	1161	1029	1009	1114	961	749	364	414	171	289	312	244	282	252	242

213197_at	7	34	8	18	26	46	9	29	90	18	21	29	2	1	13	2	35	27	25	4	2
213198_at	1140	1348	1186	2116	2752	2668	1430	1676	1019	1347	2466	2251	1737	1430	2248	1063	1071	985	972	1196	1284
213199_at	708	589	932	1134	999	876	649	707	951	1086	1128	1091	490	330	187	275	233	313	603	513	488
213200_at	181	172	102	204	311	112	168	88	98	127	124	151	8	41	112	5	12	4	10	43	31
213201_s_at	1233	1327	157	460	258	460	1015	475	260	354	456	509	430	377	369	1412	1502	825	119	300	169
213202_at	296	191	179	285	125	203	242	102	248	211	192	133	28	14	64	93	69	9	45	14	29
213203_at	1332	1407	1229	1322	656	1713	2054	1292	1743	1389	982	954	883	991	751	1239	1477	1267	1856	1434	1012
213204_at	143	314	76	133	499	621	162	206	107	73	85	84	95	75	105	167	159	70	66	79	54
213205_s_at	36	32	427	153	58	77	23	25	123	81	671	85	87	31	23	4	3	4	19	12	98
213206_at	181	125	87	101	378	390	87	108	102	61	146	102	174	142	93	61	55	124	153	145	128
213207_s_at	299	135	267	253	371	328	302	235	273	306	387	405	194	124	129	98	130	144	150	148	112
213208_at	308	571	252	291	164	228	424	631	171	266	226	256	95	80	166	539	515	393	312	261	263
213209_at	282	235	349	423	208	114	225	244	464	463	411	327	150	183	150	115	117	127	175	82	95
213210_at	19	13	15	43	18	16	16	11	56	39	27	9	77	86	13	55	78	43	67	38	53
213211_s_at	232	21	252	123	65	164	143	149	167	287	183	145	156	107	73	62	76	47	131	89	91
213212_x_at	396	977	769	1461	257	460	370	656	606	1013	142	165	67	72	108	399	421	343	1095	1446	1872
213213_at	323	264	426	438	812	535	327	369	531	542	522	393	216	217	247	72	109	62	261	177	268
213214_x_at	38414	46689	36568	21955	33698	28753	34931	34002	30421	30680	27020	44293	22528	23643	30281	26746	28480	29610	17108	24907	23714
213215_at	411	375	467	737	245	196	515	511	570	985	577	563	257	212	266	145	122	171	580	528	567
213216_at	365	337	342	259	274	578	444	445	351	326	293	204	177	224	117	216	239	241	171	130	28
213217_at	25	33	16	13	58	60	25	18	31	48	23	24	8	4	6	8	7	2	32	9	3
213218_at	323	253	102	235	99	180	256	222	88	150	136	236	104	75	155	175	145	211	121	131	151
213219_at	74	13	49	43	151	141	77	70	16	134	34	84	11	2	6	1	15	7	10	6	2
213220_at	732	837	937	865	609	696	926	1219	726	799	800	633	1476	1340	1492	1759	1855	2454	1815	1702	1643
213221_s_at	266	376	529	512	399	527	253	441	297	298	414	470	206	511	391	420	273	289	272	330	234
213222_at	347	589	157	371	164	250	405	472	122	257	91	165	17	2	36	957	1438	1062	165	552	635
213223_at	902	1369	1814	1811	177	860	1056	1141	1506	1603	1030	1068	754	576	657	636	939	790	1257	2255	1819

213224_s_at	238	101	324	480	415	231	449	399	371	296	300
353	557	475	633	821	843	817	993	927	1360		
213225_at	728	862	445	462	1236	1662	260	490	351	373	607
632	1454	933	602	1008	711	1080	1473	1512	2005		
213226_at	1591	437	1485	180	1974	200	1292	1663	1969	1277	2263
1551	2155	2035	1703	1901	1433	2102	3161	688	881		
213227_at	364	287	225	344	63	340	245	389	281	253	114
268	300	442	413	1089	1154	923	725	481	731		
213228_at	193	179	186	145	456	357	216	169	173	205	162
137	35	69	126	48	6	40	46	27	43		
213229_at	250	102	457	657	665	767	228	293	408	321	718
763	314	458	254	83	80	47	371	322	262		
213230_at	693	538	524	303	694	1072	712	557	440	341	398
299	382	270	237	300	268	370	308	240	150		
213231_at	157	201	267	338	100	228	213	107	343	266	230
94	22	7	50	45	32	19	67	94	51		
213232_at	218	33	65	154	56	146	56	107	35	31	185
62	28	3	49	20	23	22	7	12	15		
213233_s_at	807	1216	882	1274	1012	1454	892	1252	939	1289	800
885	640	395	515	1165	1042	844	1263	905	1342		
213234_at	673	450	347	252	393	543	587	702	281	542	736
367	194	272	368	347	367	300	150	143	217		
213235_at	247	131	292	197	344	400	310	228	361	288	178
241	212	157	127	189	145	114	280	160	155		
213236_at	149	91	274	370	22	288	207	214	334	401	237
161	68	97	48	44	64	8	195	268	316		
213237_at	536	601	397	397	396	519	453	373	433	447	234
312	214	170	199	350	304	274	541	403	424		
213238_at	202	364	15	120	261	366	227	207	96	92	155
176	60	67	53	200	164	307	6	84	79		
213239_at	283	332	275	310	438	473	307	392	154	293	328
189	366	321	450	812	525	823	608	822	1265		
213240_s_at	17	16	16	17	36	17	15	18	25	25	27
33	7	6	58	8	27	15	63	9	10		
213241_at	64	68	96	65	258	235	78	74	84	110	142
255	75	65	38	33	38	34	13	23	47		
213242_x_at	853	1534	1168	1054	2049	1379	1370	1124	1219	1723	1117
767	335	297	270	326	479	307	296	317	250		
213243_at	660	828	244	450	162	418	576	661	234	282	219
212	123	127	77	664	640	695	300	553	721		
213244_at	700	1134	581	635	78	70	825	933	784	834	897
363	210	233	256	548	555	591	395	206	207		
213245_at	118	47	88	135	107	137	140	135	124	120	132
26	23	24	33	30	28	9	4	34	44		
213246_at	578	580	853	938	840	1118	594	835	824	1051	1191
1267	2173	1695	1556	840	779	560	1545	1052	799		
213247_at	54	30	58	47	99	91	32	40	56	63	37
29	28	6	10	5	6	6	8	8	3		
213248_at	187	137	172	384	206	296	107	174	219	219	179
441	128	150	144	47	28	46	262	242	267		
213249_at	130	118	129	186	491	284	237	145	132	232	145
304	32	49	74	41	11	40	53	22	21		
213250_at	69	35	79	175	41	116	52	107	45	113	175
99	3	20	74	8	51	27	58	3	7		

213251_at	832	681	787	684	1068	865	795	954	704	720	793	764	1224	830	968	1354	1116	1234	1769	1434	1909
213252_at	253	224	290	282	278	302	244	276	150	259	215	256	10	34	54	37	69	22	28	35	5
213253_at	221	54	365	212	753	216	351	379	364	519	571	484	815	380	276	164	97	139	930	461	472
213254_at	501	554	252	437	451	406	509	562	454	356	341	245	135	139	113	146	78	65	81	76	30
213256_at	196	134	194	283	280	50	105	136	298	337	180	177	95	104	125	38	64	75	517	400	399
213257_at	128	141	217	316	302	183	221	170	177	208	152	212	35	43	28	27	32	55	56	147	106
213258_at	519	966	1129	1702	12	5	204	195	842	900	33	10	10	13	2	1029	1069	809	2086	3051	4840
213259_s_at	28	61	218	119	613	614	111	78	112	152	122	166	132	88	144	69	51	60	144	158	138
213260_at	816	420	278	220	962	1138	1019	1404	131	166	476	478	321	345	377	747	763	457	135	99	102
213261_at	147	184	175	322	235	259	143	152	233	207	200	271	56	40	44	5	77	82	95	114	81
213262_at	618	578	146	72	959	566	741	664	189	171	686	464	790	899	872	843	612	1163	400	335	426
213263_s_at	258	176	473	802	957	513	56	364	527	861	931	860	613	783	662	218	130	218	497	463	374
213264_at	110	22	205	352	969	640	94	189	235	419	347	788	847	715	778	182	154	116	582	412	366
213265_at	130	163	62	175	231	314	182	170	144	148	87	151	7	67	43	9	32	11	10	42	8
213266_at	400	335	319	671	302	644	522	520	429	375	406	336	108	71	87	213	196	148	205	220	210
213267_at	41	4	19	5	135	70	5	4	19	31	31	33	30	19	9	16	23	25	33	37	39
213268_at	140	241	79	47	95	102	197	255	13	77	70	126	39	85	74	258	239	217	33	34	66
213269_at	184	179	194	231	392	699	136	249	222	219	196	191	133	127	82	80	35	69	151	168	83
213270_at	291	174	248	190	357	462	257	334	187	168	287	230	60	95	77	93	117	112	104	64	70
213271_s_at	216	145	395	301	407	543	298	272	325	310	385	439	182	239	429	197	221	310	359	447	527
213272_s_at	619	400	290	704	473	731	595	429	359	414	389	450	177	171	105	377	455	332	430	338	338
213273_at	11	28	12	102	52	73	30	71	54	108	53	58	4	6	10	23	25	6	34	36	3
213274_s_at	697	749	458	720	715	708	796	961	436	717	1572	2238	582	525	1225	519	609	558	287	239	288
213275_x_at	902	1005	395	754	1522	1207	576	1131	763	771	1897	2865	2334	1689	3154	863	800	1372	1037	981	1105
213276_at	61	16	11	21	80	24	19	13	13	25	21	10	7	8	11	3	6	6	6	6	3
213277_at	32	21	16	43	26	20	70	17	37	13	7	18	5	7	11	6	17	4	13	34	9
213278_at	439	302	210	210	368	387	399	289	229	245	192	173	167	154	296	426	461	388	376	568	498

213279_at	795	897	549	1791	991	1239	958	1023	1343	1223	1242	740	812	670	654	523	628	463	777	545	554
213280_at	1191	912	753	477	627	620	644	723	582	735	350	432	236	117	279	717	777	712	711	1431	1424
213281_at	204	620	317	374	353	363	196	160	223	423	273	260	71	52	207	94	63	145	419	443	238
213282_at	460	494	415	625	375	697	633	557	404	597	453	429	694	735	1098	1409	1054	1103	1851	1372	1738
213283_s_at	303	234	331	376	375	524	284	185	306	327	341	368	106	113	81	42	35	59	77	49	36
213284_at	41	61	16	47	118	114	73	87	19	63	9	43	11	4	22	20	13	6	25	12	14
213285_at	94	75	1492	1511	550	515	125	55	1001	1709	229	333	230	75	419	10	20	30	2296	1996	3261
213286_at	370	268	286	323	162	179	387	424	238	411	301	393	48	87	35	53	31	22	63	42	39
213287_s_at	3035	2589	1945	1645	2029	2412	2182	2136	2055	1233	1819	2603	3047	2194	1584	2095	2000	1932	1818	1698	1129
213288_at	509	529	307	427	948	998	563	532	356	338	669	745	308	487	651	288	282	240	172	320	328
213289_at	361	352	510	528	623	554	709	707	577	556	470	566	389	320	316	363	313	309	546	390	500
213290_at	308	189	275	228	374	450	160	220	194	84	141	113	76	92	84	178	162	183	110	67	63
213291_s_at	1394	1074	1705	1367	910	1445	1196	739	1536	1269	1477	881	1189	1164	1343	1665	1256	1545	2314	1386	1932
213292_s_at	614	607	650	687	643	898	851	800	637	643	649	475	409	650	781	871	860	794	641	499	596
213293_s_at	99	837	42	115	106	140	83	55	48	60	33	65	0	2	2	73	90	101	21	164	193
213294_at	892	1706	1207	1464	1368	1344	1253	1475	1024	1544	1899	1596	1260	1204	2312	1674	2178	3478	2083	2980	3933
213295_at	279	167	302	370	56	288	246	334	260	288	337	441	199	192	361	195	150	125	100	149	150
213296_at	533	934	701	753	452	753	592	689	550	862	617	606	307	317	347	312	348	293	400	477	444
213297_at	202	353	285	248	430	450	363	274	404	450	362	245	99	112	272	268	202	199	236	185	110
213298_at	253	226	138	199	173	168	384	280	227	253	233	165	110	156	117	222	206	178	186	67	70
213299_at	58	39	61	61	198	112	119	120	95	115	97	126	46	18	25	26	6	4	4	5	5
213300_at	564	473	541	1014	881	839	366	471	507	812	789	592	510	550	564	228	201	312	381	492	331
213301_x_at	314	269	528	877	661	571	355	364	648	505	661	534	584	705	916	641	557	662	1326	1299	1952
213302_at	659	455	647	204	610	442	416	655	765	1037	571	847	873	803	1007	724	726	956	2232	1006	1162
213303_x_at	20	44	68	54	491	605	50	123	43	75	82	90	39	26	46	56	6	66	58	45	41
213304_at	102	100	297	488	297	474	81	69	241	370	287	354	273	283	376	47	70	32	400	666	954
213305_s_at	511	408	838	728	1181	972	587	544	721	833	1145	946	2002	2094	1394	859	603	761	1148	910	1091

213306_at	26	22	16	34	245	48	24	62	31	68	11	33	2	20	5	9	2	11	5	7	3
213307_at	230	192	201	494	372	442	183	241	165	210	153	205	59	24	23	88	128	74	168	418	354
213308_at	230	363	384	823	62	562	248	267	175	109	126	255	87	83	62	123	128	176	243	365	373
213309_at	77	66	175	58	37	30	110	146	343	277	28	24	2	3	6	126	159	160	520	491	618
213310_at	1438	884	2286	976	573	394	916	753	2055	1911	421	339	144	134	246	1129	812	694	2695	2010	2193
213311_s_at	997	1279	418	685	393	735	1239	1520	563	544	409	382	1032	757	314	1121	1231	1220	594	630	408
213312_at	220	200	366	644	554	630	367	512	409	506	483	392	342	380	477	136	262	179	357	219	197
213313_at	1097	955	1757	3694	1597	2142	1147	1498	1686	2228	1664	1381	1247	1163	2398	1408	1320	1186	2843	3933	4660
213314_at	220	109	235	251	282	449	184	235	218	247	219	216	165	195	283	135	66	143	211	127	153
213315_x_at	1035	856	2322	2717	1309	1850	851	735	2084	1821	1632	1364	1624	1379	1260	652	719	631	1688	1853	1356
213316_at	205	116	182	146	111	176	122	189	106	264	64	85	25	23	28	95	121	82	182	119	115
213317_at	20	69	201	826	289	168	50	100	82	101	212	182	199	182	545	3	40	22	202	612	748
213318_s_at	3792	3652	2573	2481	2045	1942	5361	4308	2363	2875	3698	1697	1260	1921	1623	2230	2341	2789	1104	1210	1101
213319_s_at	83	60	90	36	85	86	53	73	72	73	41	38	13	48	43	40	50	27	44	32	11
213320_at	566	386	617	204	975	1242	477	478	680	488	723	615	1697	1042	1441	812	651	879	2255	1858	2758
213321_at	19	127	255	413	265	223	28	36	257	463	33	260	225	268	334	161	132	107	707	476	736
213322_at	403	453	445	542	689	701	559	713	532	541	625	785	805	762	782	789	807	691	759	591	660
213323_s_at	393	451	350	114	78	38	465	462	403	253	509	238	108	93	117	96	59	105	93	67	82
213324_at	62	400	730	1002	47	34	93	53	388	257	178	49	127	217	73	217	251	245	342	267	203
213325_at	1090	1355	312	1251	164	120	578	917	403	558	74	88	32	38	32	2421	1763	1699	1848	753	1255
213326_at	355	190	228	382	707	779	228	285	330	453	397	533	215	195	146	68	88	130	318	210	205
213327_s_at	564	405	779	530	1875	2305	539	524	488	608	935	1019	1304	1019	640	501	489	323	531	402	379
213328_at	155	107	184	155	118	117	215	245	210	344	308	268	79	85	68	133	106	71	76	67	68
213329_at	360	438	305	384	1348	845	432	566	338	491	713	1185	587	381	389	133	103	121	144	109	128
213330_s_at	2601	225	2598	1212	2679	1433	2290	1247	3270	2288	2255	1357	4753	3553	1764	1661	1891	1759	2641	1285	1258
213331_s_at	356	298	271	238	381	410	466	557	119	261	253	224	214	160	252	480	341	434	271	370	353
213332_at	374	277	224	246	452	453	314	389	347	412	347	429	23	52	62	33	58	49	65	56	64

213333_at	857	894	836	1119	1012	934	721	669	787	938	1013	1049	520	518	904	525	556	532	522	569	653
213334_x_at	380	249	690	226	1414	790	431	318	867	925	745	733	949	718	755	215	216	268	625	288	191
213335_s_at	8	5	15	10	54	42	7	5	5	4	3	5	12	15	1	1	19	2	1	1	1
213336_at	164	19	118	28	95	75	54	53	51	50	206	44	227	100	40	10	11	37	32	22	5
213337_s_at	151	158	129	189	320	292	167	144	195	140	148	99	39	113	58	95	132	91	89	50	9
213338_at	1126	895	24	25	562	520	652	771	28	25	1211	1000	237	264	231	605	494	440	15	14	7
213339_at	22	16	38	17	250	54	41	61	68	61	109	24	20	15	61	15	21	41	28	19	2
213340_s_at	103	56	138	98	383	200	93	157	209	225	176	99	18	20	33	33	42	25	57	50	9
213341_at	523	511	844	693	469	645	493	651	361	481	571	656	350	324	325	519	432	501	472	754	890
213342_at	926	552	1359	867	1601	718	897	1181	966	1140	1485	917	1104	1367	1788	889	1017	777	913	1648	1678
213343_s_at	259	398	103	238	51	74	189	98	67	187	594	224	164	102	86	115	161	139	244	210	142
213344_s_at	442	352	304	253	991	554	382	490	375	433	388	349	213	360	498	425	288	342	324	202	113
213345_at	48	18	30	207	121	79	33	26	16	24	71	24	26	21	18	48	20	26	12	47	36
213346_at	702	481	631	369	1060	445	507	691	365	686	929	1083	1424	1987	2049	1098	1007	1314	1247	776	955
213347_x_at	18620	25994	19662	23857	22952	33928	20273	21979	18811	17694	20191	25875	21950	24557	23858	20395	21458	20376	16322	21282	19591
213348_at	595	665	164	485	668	1203	931	859	169	180	770	495	595	592	1408	754	881	644	165	275	312
213349_at	437	425	429	343	590	509	409	507	389	310	446	368	214	285	309	316	417	385	456	318	519
213350_at	310	701	422	588	392	352	428	586	627	622	451	372	160	429	1054	799	613	673	734	378	403
213351_s_at	177	545	296	460	187	469	229	335	169	124	193	177	228	364	226	228	250	240	339	397	668
213352_at	270	513	331	476	275	638	380	505	264	327	362	243	111	126	196	184	180	142	319	191	357
213353_at	132	124	14	175	437	484	203	146	106	61	173	259	90	67	124	176	103	109	52	66	118
213354_s_at	8	5	4	8	11	35	4	5	7	7	4	10	2	3	2	2	7	1	1	5	2
213355_at	83	82	5	48	7	7	115	189	43	17	28	31	38	5	3	171	81	102	1	17	12
213356_x_at	15665	15732	17892	15297	17329	7761	17874	20762	17778	20234	18467	22857	18664	23315	26826	24748	24536	24742	15986	22858	21503
213357_at	1163	1881	887	1308	1197	2126	1560	1643	673	984	1215	1870	2180	1723	1107	1783	1628	1450	1403	1155	788
213358_at	631	989	766	458	454	758	598	709	735	924	401	557	250	359	458	902	789	745	1480	1197	1355
213359_at	837	1163	366	985	584	433	564	940	530	853	412	471	408	404	533	674	879	777	654	731	1419

213360_s_at	3880	3986	4170	3466	4807	4398	4777	4241	5979	5091	6078	5212	5401	3892	3269	2521	2593	2918	3654	2605	2834
213361_at	503	797	442	1022	1008	1383	845	562	471	976	1002	1113	708	837	534	400	468	994	400	749	978
213362_at	67	15	39	70	139	91	19	36	37	56	57	35	20	15	11	23	25	13	2	3	10
213363_at	46	114	210	207	88	69	78	62	83	144	252	102	34	11	9	23	78	45	55	80	65
213364_s_at	298	376	172	530	19	17	159	377	167	232	114	80	106	77	63	396	276	336	544	327	291
213365_at	884	642	571	495	796	554	880	780	508	429	564	476	634	766	844	1373	1295	1401	1470	842	1371
213366_x_at	8738	7277	8695	10679	6273	6433	10825	8312	9946	8063	7618	5655	7937	9760	13045	13429	13581	13901	12211	13761	11968
213367_at	323	499	174	590	493	625	317	417	300	372	260	268	69	71	88	126	167	143	179	149	154
213368_x_at	237	178	50	171	195	31	331	186	163	183	280	196	85	55	156	202	94	84	111	150	58
213369_at	28	5	14	6	67	31	12	8	8	8	13	9	4	18	3	11	2	5	15	7	2
213370_s_at	303	316	509	447	598	800	437	389	722	570	571	566	475	518	413	518	538	399	1011	476	596
213371_at	139	108	31	75	81	116	90	100	92	59	54	59	22	50	13	35	26	4	24	22	15
213372_at	802	438	320	133	322	336	286	354	163	239	237	263	625	388	507	1273	995	1108	825	588	740
213373_s_at	1041	1343	1057	856	616	1000	818	793	977	1117	682	896	587	521	642	1300	1290	1411	2120	1327	1877
213374_x_at	728	693	1017	1366	1139	1665	560	599	836	1087	692	885	1216	1172	1464	1503	1041	1199	2944	1673	1930
213375_s_at	91	117	27	57	131	329	11	16	48	42	94	122	49	77	120	13	7	33	18	104	143
213376_at	753	782	684	732	899	571	635	775	626	565	686	743	811	1007	1234	1332	1364	1227	1171	831	1811
213377_x_at	27862	39745	25257	33918	15382	18694	29450	29473	28576	26307	18196	24867	17967	23053	24020	28763	30653	32245	18120	27915	21876
213378_s_at	1216	777	1579	777	870	558	1408	1301	2428	1544	1495	822	205	333	437	244	306	331	690	245	244
213379_at	986	1040	993	385	1005	843	955	996	979	1261	1258	1059	1349	1354	1050	1177	1286	1426	2247	1114	1430
213380_x_at	634	654	265	303	107	126	481	573	201	212	283	297	77	111	15	195	139	190	18	56	29
213381_at	24	16	35	9	153	70	46	22	19	14	18	20	7	21	18	9	10	11	6	24	21
213382_at	41	43	57	26	217	136	57	55	47	45	36	63	9	7	19	20	3	22	21	7	27
213383_at	221	313	404	411	353	331	266	245	349	409	361	278	223	354	420	158	106	85	172	220	159
213384_x_at	925	815	582	658	998	1075	602	640	705	829	370	377	318	165	244	300	341	280	398	410	272
213385_at	62	80	155	149	162	235	160	178	103	224	146	113	35	65	124	105	78	89	155	376	573
213386_at	504	493	155	219	528	583	485	560	138	193	448	454	194	166	180	189	180	125	67	179	169

213387_at	130	153	309	260	423	345	295	228	356	226	242	269	113	53	90	110	136	68	193	174	189
213388_at	126	145	96	168	269	375	140	202	115	239	158	266	77	60	122	101	119	143	93	254	185
213389_at	278	322	513	457	96	95	363	306	574	595	273	212	163	110	134	142	157	139	261	213	238
213390_at	955	945	1061	1173	1233	888	795	739	1151	1114	1049	1149	1405	1196	1748	1440	1391	1266	2956	2674	2312
213391_at	761	1198	628	525	807	852	959	1124	439	711	769	1023	730	513	775	1328	1207	1273	833	946	1681
213392_at	442	450	368	622	217	336	441	509	233	282	227	210	177	67	148	562	620	367	405	561	573
213393_at	249	191	452	424	445	441	262	201	305	548	296	396	259	335	428	110	194	216	332	348	434
213394_at	168	381	58	132	52	75	235	78	43	39	155	40	157	160	168	154	199	215	132	203	146
213395_at	13	10	22	22	44	19	9	21	21	21	18	85	17	4	23	5	4	36	6	23	7
213396_s_at	583	479	418	576	580	442	535	695	415	472	773	752	293	198	149	154	213	216	227	174	269
213397_x_at	321	170	134	996	59	321	158	174	112	84	88	151	75	107	72	462	579	572	193	876	1232
213398_s_at	1634	2750	798	891	1326	961	1182	1342	839	872	1086	1350	1721	1796	1069	1046	1318	1137	986	1157	836
213399_x_at	9034	9715	13189	9326	10718	13997	8186	7643	10792	9747	10365	10178	14336	16131	13020	11025	14081	13787	12307	15445	17450
213400_s_at	1036	1454	604	290	443	1157	1003	662	664	732	370	342	606	608	890	2238	2302	1352	1143	1599	1822
213401_s_at	234	154	161	19	247	274	134	154	29	52	101	9	37	72	108	262	224	189	184	176	116
213402_at	151	163	258	235	396	292	250	150	364	261	226	148	108	44	82	55	64	56	71	91	33
213403_at	54	108	182	304	139	165	107	82	286	246	121	136	119	163	142	79	122	47	322	319	342
213404_s_at	3441	2907	4857	2663	6097	7034	2332	2515	3788	5156	4996	5331	6837	7872	6161	4378	3833	4128	8233	5195	4463
213405_at	482	367	907	1203	803	1129	466	652	569	723	884	847	645	966	1136	815	862	563	949	1510	1818
213406_at	140	105	103	106	135	194	162	183	88	133	115	63	32	27	81	50	65	51	78	83	33
213407_at	581	446	757	1098	554	423	727	850	947	1386	429	341	204	260	100	437	442	254	677	328	604
213408_s_at	2138	3205	1662	1760	636	1127	1712	1990	1873	1835	772	1031	844	1041	504	1522	1423	1763	1604	1697	1682
213409_s_at	356	269	393	305	492	798	280	278	264	324	293	222	190	330	590	423	402	375	909	503	314
213410_at	1734	1342	711	648	543	469	1101	1205	856	734	476	514	611	411	544	1255	1198	1023	1042	1147	1771
213411_at	197	162	168	212	293	149	107	153	138	224	202	303	113	141	178	71	89	83	146	117	162
213412_at	226	526	198	916	55	55	178	286	254	302	33	14	48	65	11	127	153	112	168	29	99
213413_at	75	18	65	107	25	23	98	160	12	61	11	13	16	1	6	34	56	45	37	24	4

213414_s_at	32117	37520	28842	37217	30084	37172	28394	28870	26247	24895	25519	39017	25430	36115	29061	29450	30215	31647	20955	32276	19985
213415_at	9	7	11	10	18	22	9	54	4	3	3	4	6	3	1	14	8	3	18	30	41
213416_at	40	24	42	9	54	35	12	70	11	77	13	10	14	10	4	3	2	9	5	4	9
213417_at	111	28	18	17	27	36	29	34	39	38	30	38	7	32	7	3	34	6	6	4	5
213418_at	20	39	111	103	460	83	17	18	19	42	21	79	6	25	14	55	14	25	37	19	5
213419_at	382	487	225	16	491	577	366	488	95	42	591	410	226	254	373	476	464	552	116	235	200
213420_at	287	116	174	208	76	77	330	239	191	233	290	187	106	154	65	87	84	59	123	112	126
213421_x_at	1258	1106	5537	2667	1091	1067	1367	1397	3277	3583	1481	1073	1135	783	1083	766	854	792	2672	3894	2439
213422_s_at	394	322	358	413	553	589	416	468	334	426	416	367	49	32	56	69	56	54	71	26	43
213423_x_at	67	125	46	84	104	62	76	108	82	45	117	13	3	8	8	65	59	63	32	8	2
213424_at	180	127	117	220	330	555	89	136	58	60	176	185	286	391	255	103	110	104	146	235	361
213425_at	5	18	4	14	1214	2000	5	26	21	29	812	618	2538	2283	1523	1	18	42	13	2	3
213426_s_at	222	355	146	119	23	130	341	227	108	201	124	62	44	38	71	335	376	448	86	83	67
213427_at	1319	805	680	272	1135	968	1118	1127	1155	942	959	721	1634	1560	2415	1473	1541	1624	2023	1106	1320
213428_s_at	1390	3558	178	138	1438	1404	1615	2139	149	152	1302	926	484	612	393	1029	1341	876	65	351	235
213429_at	87	5	47	81	168	149	68	22	56	85	156	116	78	67	161	16	19	1	81	64	77
213430_at	28	88	42	16	43	26	76	53	54	39	58	13	28	3	25	33	10	15	15	26	18
213431_x_at	365	378	167	282	184	231	325	474	324	390	262	388	119	104	90	100	98	176	93	82	41
213432_at	17	10	22	13	37	47	16	16	12	13	11	13	5	3	2	5	2	7	3	4	3
213433_at	163	162	115	145	77	69	205	182	128	277	80	127	35	34	66	126	143	170	152	135	90
213434_at	478	420	556	356	367	410	722	545	492	524	533	353	247	320	380	507	811	466	334	318	357
213435_at	389	347	279	153	443	563	352	658	100	198	611	468	394	342	585	536	346	366	151	185	207
213436_at	48	32	22	66	63	44	53	54	51	38	58	41	13	21	17	6	2	6	9	1	0
213437_at	143	265	38	79	70	65	256	140	45	22	17	25	60	20	50	165	114	164	86	58	120
213438_at	42	73	11	97	169	146	81	75	25	84	97	36	26	6	19	4	29	2	3	3	15
213439_x_at	185	107	187	327	253	140	42	219	62	193	190	192	99	82	72	33	71	60	87	46	52
213440_at	569	536	765	656	826	801	603	420	827	922	702	654	755	903	799	742	964	882	1464	879	961

213441_x_at	376	383	758	1104	47	46	435	538	1061	748	232	65	56	21	85	17	28	104	177	290	185
213442_x_at	66	157	157	107	59	275	125	140	177	61	109	92	51	44	96	78	29	112	98	119	46
213443_at	21	22	5	17	47	22	27	22	19	21	17	30	2	7	5	3	17	4	5	7	3
213444_at	61	52	43	350	85	148	58	99	43	43	40	48	16	8	16	17	17	20	11	14	30
213445_at	419	588	243	347	59	36	409	461	367	368	350	238	71	100	130	164	224	200	154	96	112
213446_s_at	437	137	1164	1067	326	257	686	1140	1104	2439	918	753	236	191	54	116	171	51	76	106	60
213447_at	324	220	273	283	60	35	215	208	235	305	307	282	28	31	47	20	33	27	48	19	13
213448_at	238	336	183	369	370	507	342	424	351	225	497	297	182	263	172	134	221	170	86	116	147
213449_at	572	350	399	234	296	62	984	686	627	436	401	367	239	156	254	421	389	486	278	99	95
213450_s_at	364	255	262	350	379	673	376	472	348	408	284	406	91	72	129	11	29	15	109	103	62
213451_x_at	46	120	35	16	159	97	20	29	25	25	27	36	12	13	3	9	6	17	34	7	37
213452_at	156	204	107	70	351	274	192	205	115	63	216	214	212	172	235	421	336	316	231	337	551
213453_x_at	34672	31558	26314	29312	27614	23669	28887	32313	31222	24046	24868	35359	19293	24357	24156	21057	22763	23846	13691	19978	16407
213454_at	594	732	392	331	792	548	818	747	565	556	490	816	948	856	691	690	765	732	798	466	357
213455_at	1285	1199	438	689	672	851	1114	1548	360	632	692	855	361	379	385	1928	2227	1581	282	381	614
213456_at	15	13	309	451	26	67	27	11	480	129	47	25	11	8	29	2	6	20	1780	1363	1953
213457_at	267	247	705	425	282	234	595	549	664	650	277	239	168	187	110	189	166	109	378	413	363
213458_at	5	2	5	5	15	9	28	3	25	1	3	3	2	19	19	12	4	4	1	1	15
213459_at	324	345	183	250	396	171	231	424	215	309	229	239	177	165	127	208	246	313	348	190	305
213460_x_at	894	2109	1101	1136	1024	805	864	767	870	784	949	955	983	979	818	624	524	510	833	928	958
213461_at	566	255	1002	404	800	384	774	883	1006	999	776	615	418	334	258	406	359	381	636	427	528
213462_at	572	635	399	572	96	781	443	501	231	370	284	368	120	97	77	132	203	111	123	179	110
213463_s_at	172	300	146	516	223	856	159	284	94	281	144	310	101	135	204	172	156	179	242	297	557
213464_at	167	203	121	59	338	447	285	244	155	140	172	170	67	65	62	149	52	83	26	51	17
213465_s_at	1248	354	1583	1482	1282	1252	1142	1061	1543	1220	1390	1729	2922	2600	2362	1184	1360	986	2005	2224	1879
213466_at	13	10	15	14	45	40	17	13	35	13	10	11	3	13	4	3	6	4	6	4	3
213467_at	105	34	35	93	202	235	83	99	72	28	48	134	66	26	61	87	114	33	59	9	60

213468_at	287	667	349	600	80	82	354	300	587	502	337	211	156	123	107	155	185	184	309	188	214
213469_at	110	137	163	124	154	314	109	121	202	194	112	173	145	96	87	83	56	106	325	201	365
213470_s_at	3810	535	189	125	363	102	2094	1772	317	168	451	289	697	888	538	1314	1289	1419	618	384	663
213471_at	233	279	240	259	52	52	425	397	262	408	206	147	60	86	70	174	146	172	165	189	165
213472_at	353	125	38	58	102	5	171	396	39	91	48	65	17	2	38	48	18	27	46	14	33
213473_at	210	248	346	274	157	266	213	310	314	271	209	346	449	420	446	457	445	404	512	496	689
213474_at	56	80	174	67	520	742	29	13	119	41	213	158	116	128	117	99	125	71	81	102	13
213475_s_at	58	62	167	160	500	305	158	84	23	70	33	80	13	4	10	29	69	39	42	51	30
213476_x_at	6268	5662	8902	2355	14229	6043	6925	6465	6565	7694	13027	15539	15090	14469	6462	2144	2116	1980	2863	1364	1225
213477_x_at	34260	41986	33601	39307	40904	41253	31123	36320	34784	30812	25718	43195	21956	23975	29066	29289	30876	30454	19417	27157	25521
213478_at	646	1505	383	348	536	935	961	769	460	408	451	410	260	269	306	744	631	586	368	134	204
213479_at	21	7	14	16	1234	226	46	53	47	14	1788	2371	427	572	707	18	3	10	5	6	3
213480_at	341	231	176	309	433	636	396	259	163	217	217	250	218	187	310	418	476	456	215	298	562
213481_at	235	220	232	202	311	228	282	310	272	317	269	182	40	81	90	98	82	46	60	48	54
213482_at	36	212	12	14	82	93	107	132	15	7	26	33	66	49	48	45	43	30	4	3	3
213483_at	319	381	361	391	698	442	399	381	401	460	385	390	565	522	428	450	590	374	662	578	739
213484_at	66	51	58	163	198	81	110	36	112	39	90	25	11	12	32	50	43	50	66	50	29
213485_s_at	1115	1264	308	460	958	1649	633	756	348	380	637	1018	370	391	321	420	474	291	211	255	238
213486_at	194	63	146	233	172	109	250	208	285	252	148	123	20	54	26	7	29	19	50	47	36
213487_at	37	17	34	22	51	28	42	34	25	45	37	30	5	17	31	6	5	11	6	8	3
213488_at	149	175	91	184	154	99	175	168	108	85	139	145	17	2	10	26	37	31	4	8	5
213489_at	225	217	34	88	43	59	270	281	48	21	23	124	13	14	72	135	204	292	78	117	132
213490_s_at	882	1231	590	760	33	172	974	717	570	493	765	259	291	182	151	159	185	182	91	74	91
213491_x_at	9118	9278	12462	9357	9776	12448	8068	7408	9909	9360	9431	9932	13587	12642	13497	10390	13864	13569	11832	14777	17387
213492_at	90	34	71	76	191	60	146	75	155	64	99	83	31	66	46	12	19	20	32	11	25
213493_at	13	9	9	6	45	58	4	18	4	10	14	4	34	31	2	33	6	28	49	6	3
213494_s_at	402	330	340	382	342	284	490	491	300	425	435	280	116	210	364	329	227	151	261	235	141

213495_s_at	79	56	7	63	21	108	36	50	9	48	77	44	5	3	41	15	15	27	18	24	3
213496_at	103	97	54	101	142	156	122	91	71	81	108	94	39	29	42	26	21	35	26	29	14
213497_at	467	507	680	581	368	611	472	409	553	812	748	483	261	271	171	191	224	200	358	326	358
213498_at	99	131	180	238	78	69	58	113	292	426	171	137	13	10	9	8	7	3	8	46	7
213499_at	324	303	336	416	685	769	533	284	644	349	215	201	95	123	127	177	131	160	131	168	124
213500_at	19	60	262	560	254	413	158	220	361	275	433	659	266	367	409	143	246	122	470	423	426
213501_at	385	445	468	331	265	430	363	426	261	352	337	488	265	267	160	336	313	317	424	254	393
213502_x_at	210	258	33	80	245	192	178	245	72	75	37	84	37	71	105	173	39	42	23	92	64
213503_x_at	18561	27995	22539	17487	29688	33205	21274	24311	19887	25772	24263	46198	18074	24326	27024	21390	23172	23020	12756	15967	13711
213504_at	853	702	1350	1128	1861	1371	1019	740	1743	1426	2994	2821	3043	3331	2955	913	680	877	1443	1133	767
213505_s_at	967	749	517	375	937	1035	464	724	424	386	455	519	279	279	244	352	176	214	307	298	239
213506_at	6316	5402	5074	1734	950	1110	3010	4869	1540	4110	1016	1238	1228	618	945	8437	9494	8283	3901	3435	4853
213507_s_at	6533	5218	8919	4502	13659	5729	9830	5460	10192	9378	11205	6029	12526	13323	13471	6321	6802	6887	10764	11983	13500
213508_at	620	448	468	326	1183	1028	472	454	222	363	738	1131	568	415	531	406	338	317	280	529	654
213509_x_at	1159	1248	827	1286	1284	1396	1208	1184	795	1136	1019	1201	353	324	323	344	362	264	278	436	362
213510_x_at	511	370	1019	1031	590	672	501	512	583	994	419	436	435	367	283	684	530	516	967	877	987
213511_s_at	1322	1236	1925	1655	2195	1955	1006	975	2431	2144	2386	2257	2115	1556	1081	958	871	612	2370	1914	2755
213512_at	191	214	208	171	129	363	178	263	41	173	195	186	77	53	45	80	122	88	67	59	48
213513_x_at	4424	4035	5020	4856	4148	4979	2904	3408	4593	4457	4093	3288	5542	6338	6445	5264	5183	4721	7808	6066	7191
213514_s_at	495	275	845	409	943	520	621	600	541	906	759	626	262	209	122	159	125	154	238	200	190
213515_x_at	448	742	115	109	55	54	545	558	99	85	68	106	28	37	36	275	300	227	22	11	19
213516_at	66	9	41	21	115	133	36	26	7	13	21	30	11	12	2	11	4	2	4	2	1
213517_at	173	127	300	569	238	331	176	252	379	474	232	329	74	75	70	42	35	34	91	88	44
213518_at	438	482	1777	1446	1618	2770	632	771	1113	1673	1484	1531	2141	1611	1960	1049	920	809	2608	2308	2809
213519_s_at	306	137	231	185	797	396	354	257	211	171	18	85	48	31	37	220	163	298	134	114	80
213520_at	635	378	198	27	271	46	408	560	100	57	163	256	260	242	193	252	192	280	240	88	11
213521_at	525	491	514	979	823	785	429	410	755	1090	986	591	309	384	747	319	358	287	491	405	343

213522_s_at	136	71	42	101	84	99	78	96	56	143	99
	67	3	63	65	19	39	31	27	20	17	
213523_at	1373	842	536	275	964	622	1477	1280	563	679	361
	358	368	243	246	814	793	514	508	229	278	
213524_s_at	192	49	1750	1068	610	767	28	128	931	1006	766
	576	187	147	225	34	19	20	330	405	243	
213525_at	146	44	163	97	588	456	49	65	106	59	119
	70	47	48	73	54	59	37	70	49	9	
213526_s_at	230	207	212	282	212	329	138	222	153	150	121
	224	335	333	184	204	231	265	222	339	232	
213527_s_at	119	80	160	212	27	220	208	176	118	120	172
	113	198	133	208	79	90	82	110	177	108	
213528_at	239	254	355	525	271	355	375	429	314	314	370
	294	329	312	398	548	450	529	718	531	622	
213529_at	29	97	19	127	717	934	20	88	33	39	91
	90	89	51	45	23	89	40	80	56	27	
213530_at	361	162	507	572	548	731	272	288	312	390	394
	478	530	343	182	114	115	86	224	353	269	
213531_s_at	1298	1727	2412	2503	2851	2744	1847	1388	1742	2306	2318
	3271	2324	2567	3250	1853	1495	1438	2269	3945	4111	
213532_at	1118	1060	1047	1055	650	1404	1329	1236	716	629	1128
	654	1495	2673	1992	2631	2867	2706	1668	2041	2708	
213533_at	37	94	117	62	528	335	41	50	372	180	242
	109	89	40	52	10	10	9	9	4	7	
213534_s_at	504	372	339	274	93	63	399	494	507	437	271
	300	122	88	101	165	229	171	247	145	148	
213535_s_at	7152	5310	7938	3194	4814	2424	7232	8601	6613	7287	7881
	6548	7523	5863	6855	5479	5743	5892	5834	5465	3756	
213536_s_at	89	7	65	41	27	87	101	103	74	75	20
	10	36	46	74	108	79	92	112	54	36	
213537_at	62	17	41	74	33	77	81	45	36	41	17
	58	1	2	5	9	14	14	2	2	5	
213538_at	577	721	595	493	807	687	568	705	464	586	765
	752	389	373	361	397	332	512	532	335	525	
213539_at	188	15	141	146	455	296	99	145	99	152	182
	158	11	29	57	34	21	12	32	3	22	
213540_at	418	523	533	415	191	513	551	412	345	667	465
	643	367	664	409	609	545	410	620	430	295	
213541_s_at	116	17	19	63	176	204	69	75	35	73	47
	98	9	55	20	15	16	13	13	20	13	
213542_at	101	291	695	714	43	214	70	78	640	321	156
	82	93	143	162	207	246	187	196	216	101	
213543_at	9	15	37	26	14	11	15	8	37	17	68
	8	10	35	2	3	4	17	6	2	13	
213544_at	135	106	125	27	180	269	44	48	95	117	118
	99	17	29	33	43	37	25	60	4	3	
213545_x_at	2356	3630	1801	2380	1431	2434	1777	2012	1996	1835	1337
	1654	1675	2049	4791	4335	4685	3825	3417	4298	3644	
213546_at	205	281	467	804	700	798	224	468	615	608	625
	562	227	314	310	160	212	145	317	256	277	
213547_at	159	67	175	128	228	367	102	135	225	147	138
	126	37	47	14	41	4	54	64	73	56	
213548_s_at	77	204	888	682	300	542	690	680	1201	1064	1197
	914	123	107	61	76	49	75	179	169	144	

213549_at	1107	1078	1904	2219	318	361	833	813	1630	1723	146	239	304	448	237	840	872	795	2715	2939	2928
213550_s_at	124	123	213	211	55	42	203	185	155	275	215	141	118	228	435	337	165	221	236	191	94
213551_x_at	781	843	917	957	614	708	663	816	731	936	541	834	410	241	205	240	283	203	380	560	507
213552_at	179	144	302	389	333	317	297	432	227	471	230	387	271	393	288	361	218	310	629	619	757
213553_x_at	176	113	364	773	144	102	126	203	440	365	301	369	229	153	246	6	10	15	449	469	393
213554_s_at	438	833	1380	929	678	1032	632	591	1390	1031	1376	667	698	728	580	391	426	279	627	659	347
213555_at	238	206	227	316	219	528	254	339	122	235	240	238	190	214	218	233	264	261	184	260	258
213556_at	16	27	22	23	32	43	11	16	43	42	14	74	34	34	7	30	14	8	20	23	17
213557_at	265	433	277	591	525	516	387	327	231	358	250	261	78	118	101	68	86	92	104	158	157
213558_at	243	288	113	153	335	316	235	351	166	129	109	461	95	75	34	106	79	106	36	131	64
213559_s_at	12	18	9	21	92	133	12	25	20	17	17	33	4	6	12	2	8	12	17	6	6
213560_at	282	212	145	58	87	94	130	34	24	22	47	72	44	95	152	128	76	58	33	28	38
213561_at	407	248	449	340	495	324	380	438	459	449	422	165	85	111	220	186	204	194	224	170	136
213562_s_at	392	291	2546	529	713	246	411	434	1666	1336	523	349	200	198	172	193	238	212	666	679	1124
213563_s_at	64	24	31	38	410	302	40	65	31	38	85	30	17	54	44	60	55	63	69	19	10
213564_x_at	16079	16089	17510	14097	16595	17415	18146	17989	21539	16868	13689	14264	16549	18870	20690	22649	21748	21913	17316	19009	19181
213565_s_at	32	117	121	31	62	50	27	25	31	32	84	19	17	16	44	80	114	70	29	96	56
213566_at	8	35	9	31	22	35	7	7	13	14	4	6	2	2	10	1	1	2	4	1	1
213567_at	262	350	345	319	617	671	225	307	389	450	794	974	1228	1457	1997	623	639	398	826	848	941
213568_at	308	266	102	151	689	185	253	326	127	127	156	309	153	59	54	159	156	152	208	154	146
213569_at	128	35	35	43	66	73	246	102	23	106	75	33	71	43	10	37	36	97	25	45	33
213570_at	273	181	298	372	400	289	330	401	345	317	225	196	69	99	70	112	70	101	167	93	98
213571_s_at	929	939	1070	1715	1043	864	1440	1279	1387	1277	1097	850	1214	1512	1373	1542	1695	1332	1568	1647	941
213572_s_at	1151	1858	2010	2937	958	1348	639	827	1393	2402	1101	1913	850	898	960	1502	1280	1266	3291	3702	4940
213573_at	976	1018	1515	1187	1945	915	913	999	1762	1846	1586	1465	1305	1561	1262	1187	1249	853	3177	2412	2043
213574_s_at	967	1079	2272	2060	2132	879	1344	1221	3132	2616	2537	1918	2262	3484	2457	1575	1517	1082	4150	3189	3116
213575_at	46	35	79	272	92	11	38	53	177	150	97	74	5	12	26	3	15	3	41	10	5

213577_at	691	634	1936	1152	517	386	673	855	2248	2347	1022	484	525	394	242	351	220	187	811	1250	645
213578_at	523	611	522	441	525	814	545	637	428	366	479	346	624	551	446	1691	916	858	935	844	1083
213579_s_at	152	136	256	242	533	482	236	273	241	254	479	238	391	290	294	93	72	80	355	523	573
213580_at	32	68	22	41	25	42	20	49	64	75	23	55	8	3	6	4	27	1	36	4	29
213581_at	1767	2030	1285	680	1208	734	1373	1177	1482	784	1403	936	1415	1269	1552	1359	1322	1323	2226	1135	1033
213582_at	216	180	110	69	188	35	130	94	47	106	72	13	81	60	127	206	153	243	444	340	348
213583_x_at	24957	29495	26452	30726	32441	42847	24016	29255	23589	21555	19179	30084	19868	23565	28305	27937	27922	30391	17717	24511	23839
213584_s_at	386	404	586	397	498	516	445	462	1096	879	556	367	105	136	63	91	108	116	160	87	102
213585_s_at	239	247	232	225	69	292	286	281	277	245	226	99	16	101	121	103	139	155	87	105	86
213586_at	12	7	12	8	80	28	8	17	15	10	14	16	2	15	6	5	7	12	8	19	5
213587_s_at	1726	1189	2100	1645	1814	1281	1705	1419	1860	2177	2281	1571	1233	832	1209	604	676	699	997	799	396
213588_x_at	13935	16564	10605	15413	13995	17518	10639	11259	11289	9958	10655	14592	12407	13104	13822	15042	14137	14999	9239	15537	11815
213589_s_at	33	302	145	199	91	90	16	20	52	20	11	19	66	57	29	154	200	109	280	269	150
213590_at	619	785	992	1426	504	585	632	710	1347	1677	539	454	229	284	206	388	389	414	2075	831	1115
213591_at	108	60	140	135	172	31	101	107	115	148	131	112	96	62	34	96	77	102	226	141	193
213592_at	65	40	64	43	41	34	34	94	75	60	104	70	5	7	19	8	5	7	12	31	4
213593_s_at	54	27	172	264	63	108	81	124	265	331	225	260	17	45	10	4	4	3	34	48	35
213594_x_at	1249	685	1194	935	1567	703	1305	1287	1651	1339	1801	1036	1809	1415	2293	1007	1156	956	1543	1478	1613
213595_s_at	126	22	281	105	723	371	121	152	67	166	144	59	132	102	135	82	121	113	95	175	118
213596_at	85	164	163	178	458	273	142	177	138	138	121	92	3	19	1	15	40	18	68	36	52
213597_s_at	42	107	98	147	267	239	143	80	66	168	338	113	86	89	240	77	50	31	123	104	78
213598_at	282	296	754	491	588	417	497	441	595	844	688	473	530	687	375	254	292	230	922	732	945
213599_at	1524	433	1923	287	1098	24	1140	1531	2146	1740	1356	946	1584	1445	1239	1466	1347	1790	4035	1413	1953
213600_at	562	522	1004	1341	66	191	382	425	690	804	500	362	344	168	277	86	59	112	533	224	225
213601_at	303	213	317	304	597	1209	310	348	280	190	382	550	323	294	634	105	90	74	121	157	156
213602_s_at	42	29	34	41	69	103	38	46	17	46	31	34	3	17	3	7	24	4	4	5	3
213603_s_at	2479	3598	597	1508	482	835	2634	2169	871	651	1288	916	367	590	1062	3926	4338	2911	1100	1250	1212

213604_at	1128	836	1441	1261	1270	1172	1269	1326	1779	1655	1342	1318	962	502	578	688	709	830	1124	929	786
213605_s_at	411	380	231	782	263	207	341	691	343	348	168	182	68	61	6	56	54	170	151	203	98
213606_s_at	816	717	986	322	382	237	1068	925	991	1152	1417	782	132	44	71	148	53	117	165	59	72
213607_x_at	385	586	418	422	410	544	510	523	371	472	507	468	214	203	164	123	139	164	191	75	133
213608_s_at	785	674	704	369	1025	787	984	623	652	699	817	720	656	519	817	817	858	877	777	564	456
213609_s_at	123	15	98	119	159	239	110	38	112	187	132	19	22	17	12	41	36	4	8	4	3
213610_s_at	337	248	383	194	335	270	282	358	201	268	273	332	1533	472	472	1115	385	903	2240	1507	1891
213611_at	17	21	34	66	32	67	28	8	75	39	7	20	10	11	3	3	10	16	101	17	50
213612_x_at	3807	5501	4944	4727	3421	7825	4759	5479	4145	4706	3991	4830	2776	2651	2634	3390	3904	4137	4009	5291	5434
213613_s_at	261	158	233	118	627	351	191	273	189	205	169	157	3	51	29	51	19	46	22	29	39
213614_x_at	33503	34929	33945	33314	40170	42573	29161	34900	32196	30633	26866	43119	24369	32765	30619	32860	32897	36397	20407	30319	27143
213615_at	499	262	308	330	482	387	473	317	416	380	489	466	130	141	144	75	137	132	172	189	132
213616_at	806	674	342	480	400	261	555	599	328	407	276	396	313	353	433	707	780	640	660	459	413
213617_s_at	1372	884	947	1005	432	308	1671	1931	1523	1456	686	924	880	714	709	1359	1170	1101	1237	807	1133
213618_at	261	253	392	219	219	116	286	319	194	365	199	73	178	149	231	577	428	404	442	352	391
213619_at	3338	4117	10110	7184	16008	11286	4449	6180	10773	8581	10658	14905	5607	5617	9248	3092	1983	3359	10343	8080	10697
213620_s_at	38	23	395	160	109	763	23	21	313	204	868	941	725	1289	1472	13	11	21	254	433	277
213621_s_at	147	101	156	221	313	302	152	264	197	172	163	143	51	62	79	29	25	32	93	47	63
213622_at	146	214	201	132	405	476	209	185	246	138	159	136	81	58	84	83	124	98	69	63	48
213623_at	194	158	133	198	352	442	174	190	153	256	280	334	236	271	239	161	116	161	130	110	135
213624_at	147	227	129	136	22	156	77	78	39	81	97	118	162	179	85	113	66	101	100	217	257
213625_at	218	189	60	231	271	258	187	149	63	136	173	90	138	102	67	139	112	137	212	207	96
213626_at	704	369	411	522	412	378	535	673	432	425	348	514	359	258	353	572	472	584	697	501	725
213627_at	335	251	218	543	649	1537	360	455	221	446	918	498	774	1017	642	418	316	375	348	407	492
213628_at	355	255	578	632	790	540	443	527	662	601	983	391	226	483	961	403	365	517	857	558	670
213629_x_at	2981	2179	1446	1536	1215	2388	1919	2247	1169	1275	1663	2819	2839	961	864	1075	913	1150	2207	2695	1393
213630_at	21	21	14	40	168	269	27	21	20	34	23	35	18	59	53	3	5	19	11	10	8

213631_x_at	192	145	248	216	180	208	143	253	270	260	151	98	31	30	58	112	95	103	258	144	157
213632_at	378	308	369	217	630	513	441	330	377	368	242	244	208	153	86	191	159	160	197	158	131
213633_at	217	90	189	316	56	24	209	160	218	123	239	131	92	113	144	131	184	110	141	72	50
213634_s_at	632	487	180	189	187	63	284	218	207	127	220	123	281	175	133	97	105	85	155	124	119
213635_s_at	213	148	134	91	158	198	125	112	91	122	109	77	44	33	73	123	86	136	133	111	90
213636_at	172	163	193	247	45	87	201	156	211	143	176	151	25	3	6	45	13	31	12	16	30
213637_at	599	308	636	569	606	403	522	611	769	675	638	451	947	929	664	646	608	574	1413	1487	1724
213638_at	19	13	15	17	47	19	16	34	23	31	47	30	2	3	2	4	2	4	8	20	2
213639_s_at	405	310	270	420	175	206	301	310	265	277	360	178	160	110	159	144	74	133	165	196	132
213640_s_at	17	21	80	28	55	31	24	18	68	22	145	16	5	6	3	2	6	6	7	6	9
213641_at	75	120	133	102	52	44	107	227	159	166	57	123	8	38	7	58	46	40	14	11	39
213642_at	1130	1573	897	1283	247	300	995	1663	696	890	920	758	240	426	1168	1395	770	1171	1016	501	851
213643_s_at	103	13	148	39	32	36	98	106	29	138	28	75	48	28	41	57	45	13	123	12	60
213644_at	197	107	83	248	485	473	125	228	70	64	432	439	435	422	674	283	331	260	103	277	224
213645_at	415	498	1150	954	407	491	504	537	1291	1212	540	665	177	127	169	117	136	207	828	663	905
213646_x_at	24949	19059	25120	13021	28099	16324	27660	26967	34202	26589	28174	37389	20180	26832	24912	16500	19802	22948	15891	14452	11518
213647_at	221	147	458	93	444	38	379	397	514	520	345	408	243	208	242	231	190	266	794	305	414
213648_at	451	279	400	398	531	450	489	457	416	586	492	383	345	292	253	249	245	207	308	127	167
213649_at	1007	644	1247	979	794	403	958	1069	1565	1349	1191	1091	566	776	559	555	623	683	838	433	573
213650_at	146	227	210	172	367	351	197	391	348	277	192	187	127	141	143	100	144	102	154	180	102
213651_at	50	164	90	27	110	140	74	53	66	41	43	141	16	19	20	42	148	110	17	31	26
213652_at	112	127	171	264	301	929	72	100	108	254	270	226	342	650	360	121	104	111	214	139	203
213653_at	366	321	278	198	620	335	262	409	363	235	163	288	154	156	161	376	284	275	355	160	249
213654_at	746	449	884	823	547	714	810	536	601	943	678	362	284	203	198	358	284	374	374	231	179
213655_at	2359	2555	5985	7032	8257	5963	5038	4844	11474	7536	9292	12177	6576	8462	12310	5836	5011	6243	9506	7751	9022
213656_s_at	1629	2574	526	696	1607	2005	1178	1277	412	595	918	890	941	1086	487	1183	1263	858	455	641	474
213657_s_at	189	144	267	1102	565	739	197	280	435	1048	357	442	86	61	94	41	42	9	409	327	420

213658_at	143	105	231	551	135	344	139	205	254	588	202	320	61	40	55	31	26	41	186	149	171
213659_at	81	133	198	171	74	235	54	55	82	215	111	152	80	92	113	61	100	90	122	217	302
213660_s_at	228	319	99	191	41	42	126	129	84	56	132	20	65	22	29	16	82	45	46	10	5
213661_at	32	34	38	44	23	24	40	34	28	41	28	36	14	31	17	4	21	5	9	4	4
213662_at	9	7	14	32	109	289	40	12	5	31	24	29	2	13	18	3	19	15	11	4	15
213663_s_at	70	45	43	22	51	7	23	36	43	13	24	35	2	11	2	13	14	1	1	14	7
213664_at	1414	1630	1228	1364	164	167	1289	1333	1108	3753	97	150	34	46	120	977	1074	800	2248	1620	2405
213665_at	95	105	100	106	43	54	143	124	36	24	48	53	8	15	10	24	23	20	25	2	30
213666_at	327	221	208	211	491	212	284	286	206	166	506	444	169	226	255	171	162	151	105	97	67
213667_at	71	185	312	330	150	273	118	66	227	238	180	102	10	42	49	62	70	85	95	77	91
213668_s_at	225	457	300	411	16	32	591	705	190	88	108	19	138	66	43	202	119	117	67	92	72
213669_at	610	443	216	79	38	43	559	459	273	256	259	142	74	63	68	112	169	150	197	73	84
213670_x_at	796	1654	842	1024	1012	742	615	600	1017	791	775	637	762	844	1013	522	553	521	750	588	577
213671_s_at	3576	2326	2926	640	2796	3663	2087	1181	1577	794	1481	1507	2700	2232	3302	2929	3048	3164	2692	4698	5010
213672_at	271	230	164	8	106	195	150	110	158	52	90	65	24	2	48	50	45	88	109	84	92
213673_x_at	128	91	66	229	286	130	81	59	84	217	129	80	9	7	48	80	45	34	9	45	5
213674_x_at	69	6	15	13	47	32	50	8	66	68	9	54	4	3	4	9	6	4	3	3	1
213675_at	284	269	254	462	612	458	285	302	215	323	385	469	317	239	317	212	199	153	163	228	204
213676_at	180	191	203	113	183	418	156	203	214	169	165	176	47	54	56	38	8	61	93	15	25
213677_s_at	947	1196	956	1005	1155	964	924	1364	904	1245	765	801	716	628	1167	1624	1587	1262	1501	1404	2158
213678_at	77	62	94	67	170	215	154	124	124	157	95	73	26	4	44	13	9	26	28	4	17
213679_at	3	19	26	85	136	112	56	55	41	27	64	118	84	31	34	20	27	16	22	49	50
213680_at	218	135	229	137	323	220	208	156	194	268	246	161	30	55	50	71	20	52	57	52	11
213681_at	237	221	198	233	421	293	195	276	268	198	213	221	78	80	77	97	57	92	100	78	78
213682_at	1715	1264	1302	823	2095	1527	2192	1896	1094	1270	1495	1375	2001	2206	2369	3354	3396	2962	2889	2126	2343
213683_at	20	82	62	56	26	36	103	92	130	106	88	122	3	7	23	14	14	22	4	26	15
213684_s_at	40	44	76	78	65	27	61	179	27	123	78	99	31	36	15	49	39	42	30	9	26

213685_at	69	24	73	30	60	151	188	232	48	39	189	215	144	93	145	99	191	103	89	159	94
213686_at	146	220	311	308	682	320	250	362	462	456	343	224	164	207	219	207	104	135	280	204	291
213687_s_at	9642	14091	10698	12276	14643	15536	14570	12482	11008	10907	12684	15222	19315	20138	19281	18132	18248	15019	11837	16936	11477
213688_at	212	114	76	255	499	446	196	141	269	363	193	261	39	81	54	64	61	87	114	87	66
213689_x_at	320	348	237	279	307	406	451	437	324	319	334	243	169	251	356	548	619	481	287	282	449
213690_s_at	46	39	72	163	81	90	60	145	52	31	43	24	22	41	20	57	44	18	62	61	15
213691_at	102	101	170	159	100	99	148	49	51	46	37	176	86	93	36	29	46	59	88	89	73
213692_s_at	180	111	199	189	581	431	180	146	201	177	91	25	42	91	121	3	37	79	87	61	25
213693_s_at	30	112	91	2508	118	845	27	13	107	17	77	30	85	74	18	11	119	52	353	936	929
213694_at	205	193	221	259	371	351	281	358	157	200	161	310	233	185	254	625	441	566	442	465	559
213695_at	241	305	492	1359	15	58	371	424	562	934	151	101	11	2	4	160	167	165	658	888	1446
213696_s_at	565	315	921	297	825	955	390	393	731	605	537	318	647	487	511	342	504	467	683	869	767
213697_at	65	89	179	151	147	194	131	135	169	201	125	161	26	38	40	43	34	34	21	39	19
213698_at	714	1230	872	956	616	786	733	1079	692	1037	982	1174	868	927	802	688	683	762	573	585	456
213699_s_at	10300	9546	8795	7372	15793	14858	8935	11944	9191	8678	12158	18017	16094	15637	18962	12565	12017	13859	11875	11791	13333
213700_s_at	405	358	511	494	873	332	611	379	591	601	415	341	96	143	154	101	77	82	186	79	126
213701_at	454	533	632	272	247	251	564	616	510	787	360	587	920	1037	829	1279	1122	1273	2182	1917	3322
213702_x_at	3355	4655	2265	6386	3590	7136	3213	3779	1520	1944	3932	2647	3098	3344	4556	4737	4897	3992	4229	5575	6794
213703_at	143	99	138	146	194	285	89	127	54	80	99	74	32	30	6	15	32	25	28	47	37
213704_at	648	948	427	453	263	712	600	566	380	528	202	260	377	317	333	773	637	944	980	677	1082
213705_at	257	217	214	309	209	298	208	212	214	176	271	185	156	139	290	362	267	264	772	383	252
213706_at	24	27	22	35	126	43	28	40	41	38	31	33	13	2	18	7	3	9	62	6	25
213707_s_at	22	41	16	102	16	24	34	77	16	50	16	49	21	8	30	3	17	2	7	4	17
213708_s_at	2483	1180	2085	1414	1423	1367	1593	1054	1991	1644	1575	1198	1631	2330	3869	3393	3874	3286	3626	4567	4052
213709_at	132	45	43	28	359	172	155	166	195	94	334	239	135	165	165	139	144	87	88	77	86
213710_s_at	94	168	419	656	393	188	387	556	415	742	456	826	252	122	41	56	50	53	193	160	287
213711_at	75	97	18	63	1265	6628	15	21	15	14	949	280	861	424	898	157	174	90	43	82	41

213712_at 36 4 24 6 84 23 28 16 3 11 13
5 4 4 8 7 24 18 4 27 5
213713_s_at 1335 1135 1203 714 550 359 1964 2043 1406 1733 2172
1337 563 593 1026 552 517 352 651 508 439
213714_at 70 23 75 88 142 42 56 61 48 74 18
34 3 1 3 7 16 25 1 3 3
213715_s_at 142 151 14 18 99 122 93 115 20 11 26
16 8 11 14 98 147 61 18 31 4
213716_s_at 582 1973 35 1190 738 910 1003 652 40 41 522
284 147 170 203 175 220 264 70 287 225
213717_at 13 13 39 115 44 34 37 44 44 27 18
21 4 13 19 10 45 27 6 9 13
213718_at 106 34 171 158 126 183 66 94 122 155 108
121 87 169 224 157 217 198 276 279 211
213719_s_at 13 16 47 72 29 22 15 13 12 38 36
16 27 19 26 3 2 39 6 11 19
213720_s_at 1539 1452 1057 870 1468 993 1546 1362 1611 1241 1781
1162 1201 1052 1093 762 1074 994 1932 1497 1700
213721_at 70 44 111 83 60 26 16 92 19 35 18
73 25 19 31 9 37 7 61 76 40
213722_at 12 15 14 13 10 48 9 13 11 24 13
43 8 13 9 4 5 7 50 22 5
213723_s_at 53 15 14 22 51 54 16 22 20 27 41
75 9 18 6 3 6 7 4 26 4
213724_s_at 159 204 73 391 60 234 595 309 78 87 351
339 192 127 115 145 163 171 149 53 51
213725_x_at 157 190 100 19 205 189 191 170 138 169 98
59 17 25 23 96 151 67 38 72 98
213726_x_at 14659 7659 20447 5811 17745 6473 14769 14869 19805 17083
18685 21465 18781 18828 10801 3924 3853 3253 10523 5204 3426
213727_x_at 312 1126 593 1333 375 801 592 377 534 428 146
283 75 59 167 328 385 389 433 766 773
213728_at 202 210 259 268 129 74 207 211 254 239 163
28 214 224 162 189 194 83 203 335 211
213729_at 3651 2606 3093 2294 1014 977 3654 4564 4201 4131 3135
1675 2618 2220 3193 3846 3671 3019 3095 2879 2457
213730_x_at 1184 1221 1108 920 1412 727 1277 1238 1278 1138 1147
793 597 499 803 892 927 732 730 791 481
213731_s_at 36 13 45 25 93 43 36 22 25 45 34
41 4 17 10 7 11 10 13 12 10
213732_at 70 35 145 69 261 327 53 59 91 102 94
114 11 11 11 23 16 1 15 24 28
213733_at 167 196 201 27 282 323 109 206 187 134 149
207 91 155 165 100 137 83 73 80 14
213734_at 470 456 779 1159 437 593 523 614 1591 1386 907
661 608 447 931 769 625 641 2158 1273 1254
213735_s_at 7296 9265 5500 8227 4979 7325 5148 5224 4311 5199 5810
9765 9133 8216 9465 7791 7992 7102 7776 8734 5615
213736_at 168 358 68 75 412 340 358 454 140 74 57
39 147 138 387 357 283 395 235 244 152
213737_x_at 393 864 324 370 414 735 456 410 222 300 141
373 186 135 377 547 557 570 471 342 481
213738_s_at 18111 15120 10286 9252 8771 7913 12969 12928 12075 10889 7940
7547 11147 12217 13087 20977 19455 20709 15134 15176 17779

213739_at	409	193	208	212	126	372	396	405	162	271	112	168	108	69	131	245	181	141	116	154	123
213740_s_at	22	12	27	14	69	69	25	29	23	10	14	23	19	20	39	5	6	7	11	4	4
213741_s_at	405	366	795	630	566	554	872	686	1065	781	797	638	407	335	374	472	363	456	599	373	571
213742_at	97	67	190	166	154	146	94	145	229	324	132	161	90	75	85	52	68	93	360	156	197
213743_at	414	258	283	358	261	337	171	280	175	246	199	160	277	268	250	398	401	422	443	505	620
213744_at	122	60	90	115	48	120	119	116	123	165	90	109	18	42	6	1	15	23	29	13	19
213745_at	9	36	56	57	96	39	80	94	5	35	44	63	7	2	19	2	12	3	11	1	11
213746_s_at	1438	1712	7591	1282	1890	2184	1442	1177	3480	3621	4822	2984	909	518	380	261	352	217	965	844	799
213747_at	189	315	11	65	158	265	611	801	174	261	270	371	17	12	13	30	37	56	9	2	74
213748_at	135	154	285	341	199	301	341	393	292	320	234	310	50	96	62	82	71	101	51	122	91
213749_at	19	94	19	50	318	332	15	40	19	25	14	20	8	61	89	5	24	35	37	54	33
213750_at	628	709	684	682	515	873	551	342	379	858	426	553	758	735	1386	2046	1960	1792	1206	3175	4257
213751_at	185	90	87	106	368	308	119	55	127	172	139	109	35	41	11	8	5	37	8	22	9
213752_at	111	112	35	36	279	480	17	61	27	101	104	63	8	39	6	69	46	33	8	21	4
213753_x_at	3732	1482	2170	826	1479	855	3314	2440	1829	1451	1885	1485	4291	2559	2921	2716	1968	2277	3297	2713	2307
213754_s_at	986	938	963	512	803	714	453	482	640	503	466	520	1534	1137	1160	2154	1941	2112	2885	2164	2950
213755_s_at	36	50	35	32	760	1001	32	17	29	29	24	34	59	46	25	35	28	59	61	75	30
213756_s_at	512	669	442	323	103	255	558	595	423	425	534	314	40	43	31	62	68	106	54	45	47
213757_at	280	157	605	530	2231	2009	170	245	824	539	536	627	87	173	215	72	55	184	229	302	335
213758_at	33	34	100	38	23	35	36	30	52	41	20	23	48	49	16	83	112	108	88	62	9
213759_at	16	30	60	17	206	198	17	55	31	27	68	39	28	45	72	33	10	9	9	4	10
213760_s_at	462	163	308	182	572	447	557	396	468	358	426	337	557	312	208	196	205	218	203	129	181
213761_at	295	182	151	278	245	284	292	310	175	226	188	225	251	208	183	337	252	298	364	403	596
213762_x_at	3535	2080	4129	2171	3068	2275	3813	3303	4578	3392	3839	3357	4367	4670	6952	5401	5967	5666	6515	6630	6581
213763_at	548	471	453	317	443	402	550	615	367	635	591	349	113	142	76	76	35	81	94	77	55
213764_s_at	3	6	34	21	1	5	12	0	3	1	0	21	1	1	1	1	2	6	1	1	7
213765_at	52	2	7	78	73	289	8	112	15	67	43	65	1	2	22	25	3	13	4	3	13

213766_x_at	2414	1781	1199	954	1340	1614	1786	1457	1053	1599	1970	1135	1308	1028	864	1158	1557	873	1408	875	549
213767_at	78	62	26	19	19	26	172	107	132	78	87	36	19	8	10	8	7	23	11	16	29
213768_s_at	46	38	56	66	231	456	40	42	162	75	84	273	4	43	69	3	4	4	11	11	9
213769_at	157	27	60	28	257	192	28	75	44	24	23	14	45	7	58	54	44	13	25	23	7
213770_at	24	83	61	45	102	159	81	69	28	52	27	31	18	24	33	8	23	30	34	59	62
213771_at	591	776	522	936	109	129	398	549	454	514	473	714	53	139	133	169	211	142	197	284	156
213772_s_at	247	266	324	455	66	71	270	268	569	370	280	231	172	191	90	159	239	122	266	130	189
213773_x_at	2540	3053	2107	1911	3395	3620	2523	1851	1737	1442	2429	2449	2721	2638	2734	1694	1877	1949	1790	1999	1790
213774_s_at	22	11	9	13	44	46	23	11	16	21	145	26	35	141	155	8	12	12	6	14	5
213775_x_at	1005	1201	1654	2659	1820	1552	1915	1853	1656	2372	1099	1119	1113	1411	1108	1638	1597	1454	1696	1415	1564
213776_at	75	24	24	26	366	275	32	34	23	31	33	101	66	40	40	49	30	6	34	57	50
213777_s_at	87	12	14	74	21	34	29	86	62	50	77	9	5	20	13	2	5	4	23	4	3
213778_x_at	335	443	182	34	151	157	257	424	139	137	185	24	78	84	113	216	170	215	158	106	85
213779_at	157	445	27	47	258	247	129	203	28	55	132	117	61	72	120	162	143	99	53	82	74
213780_at	48	82	23	26	364	173	37	38	17	123	195	78	99	109	104	13	29	4	19	37	22
213781_at	15	15	19	41	22	22	16	16	15	21	23	11	22	3	2	3	4	2	4	3	3
213782_s_at	4	2	3	4	15	7	9	3	4	4	6	4	3	2	3	2	2	2	3	2	2
213783_at	19	19	121	156	49	50	105	160	159	38	148	133	5	11	9	5	30	59	4	21	2
213784_at	377	468	406	493	498	316	399	542	314	299	345	338	60	58	103	71	38	51	66	17	9
213785_at	216	133	471	475	564	235	415	311	345	573	392	230	269	182	136	168	166	148	358	333	280
213786_at	189	297	410	345	378	1308	146	223	124	296	253	248	613	767	392	461	292	469	1328	1125	1953
213787_s_at	2635	613	2097	1358	103	224	2046	2092	2022	1711	571	626	3165	2731	2509	1177	1162	862	1520	1512	695
213788_s_at	127	125	383	543	639	520	33	154	385	418	418	868	145	121	175	40	4	4	199	169	357
213789_at	139	24	1435	947	5773	2474	91	117	911	985	6518	3971	519	912	2067	59	26	12	439	649	1167
213790_at	22	19	8	74	25	34	16	54	17	32	16	25	32	18	17	19	2	31	2	17	2
213791_at	86	15	53	63	221	130	32	197	92	112	95	152	59	21	18	10	33	34	41	26	34
213792_s_at	362	466	121	453	48	55	428	603	37	252	176	145	118	304	354	730	512	485	1045	1138	820

213793_s_at	362	633	1518	526	896	577	628	619	1088	897	580	574	585	359	386	383	252	281	812	545	722
213794_s_at	857	609	756	1048	1203	1587	2058	1353	929	840	1340	994	1649	1332	1058	1851	1825	1780	871	716	634
213795_s_at	638	646	445	339	382	613	533	760	254	320	613	597	300	438	446	408	621	457	355	310	383
213796_at	19	44	159	1147	14	13	21	8	19	27	14	11	2	1	3	3	3	2	18	38	3
213797_at	89	32	94	100	223	323	58	92	86	70	74	68	25	27	8	3	4	112	24	172	270
213798_s_at	4292	2506	3946	4498	2749	2361	4615	5215	4109	4871	3552	3141	6761	6455	6105	4968	4443	3675	7778	6782	7866
213799_s_at	319	335	121	122	165	379	348	497	244	236	499	390	100	58	44	26	26	81	45	41	12
213800_at	42	23	151	336	14	23	8	7	343	134	14	9	7	5	9	3	26	2	554	483	743
213801_x_at	26606	29233	20847	20236	27189	26914	24888	25286	22322	17571	18200	28873	21896	25684	24451	25162	26318	27285	15943	23305	19709
213802_at	78	108	5	61	109	151	78	67	8	63	9	8	14	7	10	100	108	70	62	49	58
213803_at	722	534	1310	938	1063	740	624	925	1552	1720	1425	1154	1531	676	826	642	635	298	3545	2187	2081
213804_at	134	265	98	226	19	30	232	247	140	198	153	256	115	95	92	161	161	173	188	202	212
213805_at	164	108	186	114	202	169	197	230	127	123	156	127	75	129	46	149	81	83	100	72	87
213806_at	11	9	19	12	40	17	13	16	19	21	17	19	2	19	3	3	1	2	3	8	8
213807_x_at	1811	1006	4942	1979	252	309	1981	2568	4218	6390	584	506	18	23	38	331	403	319	484	280	467
213808_at	5	10	11	25	21	17	52	26	24	31	10	6	7	18	5	17	17	16	15	4	11
213809_x_at	42	34	46	66	73	112	50	44	52	46	44	39	17	53	40	46	66	44	34	12	9
213810_s_at	37	63	71	28	198	238	125	161	119	99	152	116	69	106	344	119	83	108	89	59	60
213811_x_at	1754	1467	1092	736	1688	1247	1635	1685	1587	1016	948	885	962	937	2103	1845	1232	1720	1101	1384	743
213812_s_at	434	650	571	746	503	480	735	699	1465	1466	593	812	928	661	304	133	130	94	847	791	919
213813_x_at	210	426	236	173	290	266	197	179	118	224	186	196	150	174	436	514	211	366	364	337	236
213814_s_at	148	46	133	140	132	245	115	87	178	166	81	108	4	18	7	24	5	26	25	42	2
213815_x_at	636	738	441	420	311	481	702	717	347	576	618	413	171	176	144	214	197	237	202	119	64
213816_s_at	349	230	1289	477	187	66	489	965	1683	2442	132	93	10	34	1	68	55	25	93	73	52
213817_at	3	2	1	1	14	8	1	16	1	14	1	3	1	4	10	1	2	3	1	1	1
213818_x_at	33	141	102	145	367	245	93	111	153	145	101	75	14	40	88	59	45	52	55	36	23
213819_s_at	49	153	213	138	95	255	129	116	199	235	118	121	7	14	26	23	76	23	65	17	11

213820_s_at	58	141	145	366	212	312	211	305	282	250	159	216	67	87	68	93	99	89	69	107	141
213821_s_at	26	29	15	115	41	32	28	22	51	88	21	75	29	22	5	8	14	17	27	50	20
213822_s_at	397	191	319	239	146	183	447	304	488	384	487	412	374	289	673	432	388	300	477	238	222
213823_at	505	307	522	770	448	472	466	336	949	864	505	293	304	280	376	252	247	288	542	393	434
213824_at	66	34	104	135	69	27	86	131	70	63	91	89	13	29	26	4	4	5	11	21	6
213825_at	48	34	42	50	43	83	68	42	16	98	43	33	6	3	3	28	8	24	31	18	2
213826_s_at	349	335	1111	674	414	270	257	335	288	471	308	276	122	238	403	324	203	251	339	318	325
213827_at	189	167	43	38	22	54	30	156	40	35	45	19	71	49	110	56	90	65	60	52	49
213828_x_at	13842	16705	21059	32928	18812	13946	15124	19175	19381	26493	15366	27170	17275	20223	20955	16181	18048	17309	17613	22048	19364
213829_x_at	386	363	298	199	712	1003	370	296	190	293	438	241	183	184	192	170	193	199	104	57	85
213830_at	30	21	45	63	341	55	36	58	51	34	31	75	8	48	52	8	12	13	6	4	9
213831_at	13	63	14	88	113	66	15	132	48	11	81	18	17	6	44	18	9	32	14	7	5
213832_at	106	96	142	154	120	247	184	206	127	150	149	165	40	64	35	36	47	46	35	32	33
213833_x_at	253	174	281	136	81	73	207	290	181	507	298	98	63	77	99	92	79	43	30	43	37
213834_at	25	9	30	30	18	77	28	25	23	17	95	24	16	20	29	51	42	36	29	41	5
213835_x_at	1110	873	659	347	1135	900	783	644	945	709	837	773	604	242	266	943	956	299	632	630	717
213836_s_at	491	498	225	367	547	1154	228	344	154	110	270	325	306	291	326	585	801	503	179	765	603
213837_at	82	128	106	207	129	65	146	157	169	205	108	96	24	26	41	20	52	18	53	32	44
213838_at	1027	1017	1015	910	1014	1095	962	690	508	867	814	691	818	825	905	1156	1022	1181	1202	1272	1531
213839_at	280	495	338	857	23	227	235	253	222	507	87	113	39	4	36	215	280	189	320	284	364
213840_s_at	241	272	197	198	71	285	186	141	194	210	193	190	16	29	36	58	76	46	156	67	15
213841_at	30	24	27	34	52	46	41	5	51	34	38	36	8	12	9	10	50	6	13	2	7
213842_x_at	2318	3971	1864	2187	2422	2353	2402	1366	1493	1484	2078	2125	2389	2101	1850	1282	1267	1359	1534	1630	1563
213843_x_at	1474	1573	3635	1386	957	775	2054	1853	5146	4415	1843	861	575	331	636	408	429	385	749	480	505
213844_at	884	747	860	1328	635	481	717	722	598	788	851	959	345	379	534	505	528	248	609	1130	947
213845_at	13	18	19	16	25	22	19	28	13	17	17	18	2	7	3	24	35	60	3	57	46
213846_at	1041	1585	1275	2135	566	977	1306	1453	1448	1642	1042	1137	1232	918	606	1303	919	843	1405	1289	783

213847_at	422	322	160	317	333	903	437	375	285	310	314	269	127	169	68	269	409	200	53	159	104
213848_at	398	275	402	154	742	477	325	331	400	403	577	569	253	294	489	415	544	518	578	367	360
213849_s_at	98	109	95	111	216	222	113	102	82	102	74	109	14	22	25	18	24	11	36	39	31
213850_s_at	544	326	699	742	344	402	1076	1269	999	1015	1131	1023	745	919	747	664	631	421	589	808	662
213851_at	357	238	176	128	723	918	356	224	167	137	318	274	253	196	242	244	285	270	144	150	135
213852_at	1344	856	1038	1426	1310	1223	1113	1238	1011	1698	1282	1654	1351	1325	1409	1521	1336	959	1496	1710	2159
213853_at	898	805	1002	639	935	922	518	434	385	1254	949	461	449	459	1116	1778	1671	1473	1522	1481	2136
213854_at	198	257	228	110	278	399	172	90	62	28	136	106	46	66	89	45	64	41	20	9	45
213855_s_at	156	68	236	272	327	245	180	104	353	356	198	155	34	38	18	54	45	28	71	51	35
213856_at	15	43	60	10	16	7	11	40	16	85	24	6	2	3	2	3	1	13	25	26	17
213857_s_at	1077	1737	2208	3207	937	1252	1181	1131	2648	2532	1236	1561	1543	1143	1109	972	855	1263	4319	2967	4366
213858_at	351	281	28	92	195	130	187	300	98	99	172	182	95	122	148	302	338	184	144	166	167
213859_x_at	676	547	453	206	509	366	610	748	387	544	435	400	433	332	334	694	394	704	584	385	385
213860_x_at	5394	4727	3955	2951	5414	5074	5594	5264	3559	3157	5135	4100	7061	6440	7859	7114	6762	8119	6750	12221	11307
213861_s_at	403	400	456	369	528	660	568	472	502	474	662	464	229	241	332	302	266	290	308	260	299
213862_at	20	18	11	85	23	48	19	51	15	45	9	11	13	14	3	12	14	16	4	6	16
213863_s_at	11	36	33	10	11	22	20	8	7	31	16	8	32	51	46	28	32	33	37	29	26
213864_s_at	7894	10075	12532	8930	8122	7981	7576	10435	9565	10337	7770	9139	8662	6868	6401	10990	9341	7507	13040	18826	14787
213865_at	1001	220	2988	371	217	34	3176	3498	2703	3268	1804	550	445	512	147	255	210	226	146	52	168
213866_at	49	43	94	57	642	547	45	22	99	63	38	65	38	38	47	24	57	88	74	62	28
213867_x_at	34280	37923	32170	39893	32924	38592	34674	30432	41827	31611	30124	41853	21483	28439	33468	23052	22474	29127	18470	19998	16456
213868_s_at	62	113	27	62	18	15	152	133	20	64	50	28	4	3	28	66	52	53	39	25	19
213869_x_at	197	150	184	207	142	71	186	115	100	106	115	107	22	7	11	7	7	7	4	36	3
213870_at	38	147	53	158	30	81	37	34	44	39	81	31	47	45	27	19	51	44	54	78	56
213871_s_at	148	26	53	124	128	91	166	54	110	133	101	133	50	21	53	121	15	113	65	51	39
213872_at	3680	5823	7367	5244	6698	5464	6124	7192	4414	4984	6675	8579	3493	2413	4811	3347	2362	2623	4662	4367	10203
213873_at	234	167	239	173	401	118	309	338	332	263	153	165	54	191	222	470	661	624	110	100	58

213874_at	171	122	277	591	378	274	307	286	273	296	180	240	16	41	42	8	17	20	66	89	78
213875_x_at	728	751	591	586	650	722	912	880	807	660	888	793	485	732	1463	998	843	766	1557	1172	1959
213876_x_at	542	574	448	339	669	767	557	414	357	312	716	573	323	341	428	208	205	109	183	261	186
213877_x_at	339	279	484	495	296	422	425	281	546	377	414	376	148	248	125	261	268	222	281	191	169
213878_at	786	953	1306	745	496	806	1006	703	840	858	395	378	459	481	518	1354	1209	1395	1900	2015	3761
213879_at	763	782	381	649	341	439	599	537	458	570	742	625	600	536	732	912	558	1061	1309	740	566
213880_at	38	22	296	318	5	4	32	9	356	1371	4	26	4	1	3	2	2	1	2145	960	1517
213881_x_at	15960	15222	9648	11187	14784	13369	14148	14789	11382	9704	10871	13809	16599	16877	18062	20076	20202	21571	14335	16443	15165
213882_at	138	111	79	158	188	222	101	139	74	92	131	132	63	93	105	90	124	89	79	46	82
213883_s_at	1203	1381	682	1046	664	1598	529	831	396	630	543	506	959	1168	1295	2044	1195	1975	1716	1572	1710
213884_s_at	11	9	12	16	21	19	11	9	19	13	18	11	7	3	4	3	6	7	11	18	3
213885_at	339	181	330	288	1090	1142	338	281	326	352	307	313	72	89	88	112	32	77	179	92	82
213887_s_at	2705	1878	2336	1045	2907	2008	3596	2828	3030	2679	3247	1542	3284	1764	1762	1828	2090	2512	2193	949	841
213888_s_at	26	6	8	70	282	40	7	15	15	20	20	11	17	35	77	41	4	6	32	5	5
213889_at	729	639	579	770	635	621	423	581	345	464	378	495	517	567	459	641	517	580	445	650	663
213890_x_at	24093	27620	22159	30366	18726	23137	22871	24100	28563	24056	20762	31204	19438	24280	23368	22978	25530	23358	15328	23799	17131
213891_s_at	17	62	19	41	48	122	50	82	66	53	38	80	9	17	29	23	3	17	22	15	40
213892_s_at	3684	3760	2532	1851	1603	1152	2679	2745	2414	2801	1944	1928	2247	1659	1311	2231	2157	1791	2512	2023	945
213893_x_at	995	669	1336	2074	1869	2451	1582	1163	1456	1876	2682	2694	925	920	1247	716	680	677	728	708	475
213894_at	87	7	43	79	92	175	81	80	74	88	63	63	1	13	22	38	14	9	22	14	11
213895_at	106	26	157	106	166	145	72	74	119	161	320	349	119	244	154	71	60	11	57	51	25
213896_x_at	156	209	128	268	33	348	34	293	58	137	57	294	193	189	161	154	135	128	185	278	219
213897_s_at	2131	1703	1450	791	1832	853	2206	1364	1644	1198	2143	1537	3373	2727	2043	1536	1499	1030	1532	1401	701
213898_at	81	111	79	93	227	138	160	107	86	109	92	101	26	44	57	73	59	58	34	46	6
213899_at	185	105	270	199	92	87	367	371	458	231	171	117	442	222	90	233	223	204	371	192	368
213900_at	361	392	336	248	515	657	309	306	258	298	306	230	52	102	85	132	154	148	84	19	21
213901_x_at	1061	1580	1042	657	554	508	1179	1432	803	900	1055	924	555	480	244	419	387	351	204	170	179

213902_at	1390	1917	682	905	1115	2573	1283	1188	491	745	618	617	996	1500	1298	1948	2288	2756	1531	999	802
213903_s_at	119	47	20	26	289	284	61	205	48	24	31	57	42	32	71	40	43	62	92	46	57
213904_at	37	50	18	44	47	8	19	1	1	52	38	63	22	12	33	9	6	16	22	19	3
213905_x_at	16	75	425	555	85	151	42	11	693	503	44	49	1	29	87	45	34	9	503	348	379
213906_at	365	111	228	57	841	231	272	429	64	77	802	1129	745	835	502	410	342	565	82	130	187
213907_at	360	270	381	229	293	306	322	331	285	313	341	187	182	273	617	859	674	853	572	417	418
213908_at	64	46	4	5	85	246	49	30	62	20	47	53	92	49	50	41	21	59	17	39	28
213909_at	102	101	201	142	122	224	203	150	177	245	134	160	23	3	9	29	19	3	30	32	50
213910_at	8	50	4	27	55	13	11	40	15	29	33	48	5	15	34	19	2	4	8	4	1
213911_s_at	15935	7812	10399	5674	13232	7207	10332	12093	11348	9997	11833	12869	14966	16117	14059	15152	14677	16253	13387	11348	10284
213912_at	360	238	285	286	45	254	490	397	440	419	98	160	35	25	22	455	353	321	529	277	395
213913_s_at	335	287	174	113	15	105	253	194	256	197	45	75	28	4	48	298	245	286	505	501	585
213914_s_at	40	17	76	16	223	40	24	86	15	126	111	62	2	50	60	31	41	10	32	19	23
213915_at	7	5	4	8	26	16	5	5	5	6	4	5	4	4	2	2	9	3	6	2	12
213916_at	194	114	140	207	199	347	105	227	170	161	270	266	190	167	115	6	10	30	83	67	119
213917_at	110	89	88	233	54	189	319	139	166	140	124	112	58	31	34	8	26	26	18	40	31
213918_s_at	1070	313	1348	876	827	372	621	684	1015	1100	877	373	563	514	340	782	767	895	1923	1740	1647
213919_at	253	260	222	221	337	371	272	249	241	184	243	207	87	115	51	22	113	100	66	64	21
213920_at	78	10	27	48	14	42	49	24	29	22	85	45	50	40	48	17	15	20	19	7	17
213921_at	36	34	71	35	56	36	72	149	102	143	78	40	25	2	55	37	7	30	39	33	10
213922_at	77	36	53	72	93	75	72	107	36	124	88	90	141	73	33	23	35	27	44	57	48
213923_at	2449	4344	3418	2232	2543	5516	2388	2348	1998	2404	2334	2447	3536	3458	5012	6354	6467	7804	5863	5003	6308
213924_at	290	260	61	140	1252	1351	277	195	64	112	497	294	672	943	730	119	76	99	89	117	54
213925_at	181	63	832	286	59	58	179	252	613	1234	207	194	37	27	13	9	25	4	172	88	87
213926_s_at	206	187	259	153	164	206	231	230	484	356	284	190	78	66	32	32	49	60	52	41	86
213927_at	376	300	376	363	584	765	502	410	294	337	419	328	275	224	137	205	144	161	306	289	272
213928_s_at	135	102	136	19	230	81	208	165	162	133	149	29	11	4	44	12	8	12	61	50	27

213929_at	409	202	844	631	964	993	292	329	538	400	772	479	734	788	796	215	183	287	1020	761	1008
213930_at	294	258	107	168	521	523	123	248	118	131	173	182	367	410	336	263	279	345	163	216	280
213931_at	417	597	145	144	307	327	276	435	178	152	412	280	238	172	202	317	317	250	775	272	240
213932_x_at	10410	24572	11295	14756	11121	12120	9606	12508	8795	7929	15147	13508	9145	11302	11162	9223	11113	13182	7190	10424	7217
213933_at	61	83	58	98	353	241	107	150	132	108	98	98	3	7	24	30	22	15	19	26	19
213934_s_at	224	202	106	132	36	223	130	239	90	159	142	173	127	72	127	195	238	230	163	184	354
213935_at	179	220	175	146	126	91	85	107	63	152	104	102	92	138	94	241	232	271	132	162	162
213936_x_at	198	168	300	261	694	512	193	235	223	300	199	271	106	102	82	77	74	116	89	97	181
213937_s_at	530	313	727	557	617	875	579	484	1114	681	864	703	709	1032	714	483	621	580	697	489	609
213938_at	34	10	9	70	21	66	73	12	5	73	11	13	19	31	10	28	28	12	4	17	9
213939_s_at	246	343	297	200	183	203	192	339	174	177	132	131	96	29	48	118	82	96	154	61	175
213940_s_at	73	173	174	238	320	445	216	139	174	168	199	313	302	232	251	79	121	70	227	269	338
213941_x_at	14173	19370	15811	17775	19492	26837	14958	15497	15870	14486	16789	21616	18620	22688	22838	21046	21675	22396	15917	21209	18022
213942_at	192	461	54	36	27	42	204	310	43	66	34	136	3	6	14	64	50	4	85	27	33
213943_at	98	58	37	6	91	83	74	88	3	21	4	6	8	22	3	140	142	181	2	12	11
213944_x_at	1696	1775	1238	1172	1216	1329	1613	1312	1065	1321	1512	969	661	872	655	659	659	607	594	485	248
213945_s_at	246	337	330	172	304	106	264	274	282	299	161	121	62	41	198	229	286	199	251	174	146
213946_s_at	226	196	8	22	264	112	378	247	123	13	57	26	30	60	13	154	159	131	4	22	2
213947_s_at	974	1429	1460	663	434	275	1024	1160	1568	1681	911	593	512	511	329	527	516	499	684	632	467
213948_x_at	132	178	133	102	268	187	171	144	116	151	163	136	29	22	36	24	16	6	25	13	22
213949_s_at	82	56	85	22	80	90	121	87	48	87	24	16	10	42	9	10	63	36	4	4	17
213950_s_at	111	49	28	54	51	120	7	22	58	91	121	36	32	27	34	34	22	50	43	23	2
213951_s_at	333	200	289	74	726	343	209	305	261	303	372	548	609	558	523	396	399	393	518	264	213
213952_s_at	116	32	92	216	217	187	93	117	139	191	135	88	5	26	46	31	25	28	63	47	58
213953_at	159	114	9010	3254	132	164	76	91	5310	10381	10	104	22	4	27	110	141	111	6714	13858	12411
213954_at	106	109	54	16	283	198	156	222	66	63	156	191	728	480	230	99	68	58	64	94	100
213955_at	73	55	52	79	37	38	131	20	51	39	50	25	9	34	2	20	15	4	8	11	29

213956_at	177	217	157	154	333	241	223	194	191	157	144	266	42	58	56	39	61	56	60	102	66
213957_s_at	171	96	187	263	459	361	219	447	262	310	321	342	174	106	92	71	70	67	74	87	83
213958_at	128	89	141	195	302	290	42	45	166	187	82	126	10	61	93	79	110	72	79	57	46
213959_s_at	214	130	81	76	91	106	148	139	51	49	43	82	47	39	43	154	95	178	135	147	130
213960_at	226	89	179	141	188	161	164	211	195	204	189	134	11	2	42	17	5	37	22	31	5
213961_s_at	34	10	20	21	8	11	24	26	17	17	23	8	14	4	5	25	7	6	7	17	5
213962_s_at	52	38	68	28	74	31	65	158	151	46	36	83	6	4	4	17	2	17	45	34	4
213963_s_at	78	55	53	21	133	137	105	160	21	75	145	109	50	94	184	133	123	152	104	82	78
213964_x_at	53	56	94	79	21	117	45	51	79	71	68	36	36	24	81	76	70	43	177	108	240
213965_s_at	153	117	20	78	363	345	189	162	51	89	18	85	8	35	60	61	101	42	50	56	43
213966_at	52	108	69	113	99	30	66	98	116	20	16	13	3	5	18	32	6	38	25	3	7
213967_at	28	12	49	26	36	36	16	17	17	15	11	14	2	3	3	5	2	2	4	2	2
213968_at	206	46	99	92	181	65	106	137	78	158	21	132	19	13	8	16	30	20	11	37	23
213969_x_at	22576	20765	21622	29968	17032	23129	18386	16744	13546	13491	16946	22015	17969	20913	22419	20577	23217	20162	13664	22156	15560
213970_at	187	276	133	269	228	230	342	367	244	204	294	250	417	269	246	386	380	324	333	167	256
213971_s_at	327	586	579	748	296	384	507	684	1092	1087	920	782	1011	545	572	121	181	152	644	461	417
213972_at	11	6	9	9	107	146	8	8	7	10	7	14	56	18	68	16	14	54	55	36	18
213973_at	82	119	190	158	320	285	76	84	237	168	165	111	44	98	54	34	6	48	80	53	30
213974_at	87	91	58	116	214	141	114	84	82	88	97	43	1	32	28	20	17	3	27	22	27
213975_s_at	5	54	9635	13859	16	11	4	4	8884	13212	70	13	203	161	77	26	15	38	17668	18301	18206
213976_at	94	69	53	27	404	379	65	55	90	59	61	87	28	16	81	51	46	61	63	24	26
213977_s_at	1639	1870	1919	2076	286	1187	1072	789	1132	857	1715	586	276	318	537	347	391	132	343	360	223
213978_at	42	58	16	8	54	59	36	16	7	11	7	11	4	6	3	9	11	7	11	9	10
213979_s_at	1252	1508	1270	1203	904	1320	1063	1267	890	1075	750	1385	204	322	517	803	906	861	776	682	775
213980_s_at	2316	3212	1294	1291	1215	1082	1495	1850	1484	1574	1113	1215	1349	1324	2063	2775	2571	2594	3533	2452	2379
213981_at	216	122	203	75	438	558	148	169	179	99	121	33	16	38	17	64	50	107	83	49	76
213982_s_at	226	395	288	773	201	419	166	227	369	326	107	148	124	106	145	348	198	225	627	900	816

213983_s_at	282	105	369	47	326	226	472	435	387	245	269	222	125	92	149	290	325	463	286	168	320
213984_at	206	112	168	149	197	230	113	131	119	82	94	72	211	158	169	291	225	337	572	244	596
213985_s_at	17	18	19	59	87	52	21	38	45	64	38	52	4	7	18	8	6	3	4	4	5
213986_s_at	467	473	501	383	573	517	784	578	765	847	668	181	50	68	77	68	65	69	67	37	27
213987_s_at	16	6	14	10	71	34	8	11	7	11	9	6	10	21	3	9	8	7	8	6	2
213988_s_at	1420	2032	689	961	1153	2728	1859	4752	207	148	1786	2684	1974	619	477	1380	1229	1122	1122	1320	1345
213989_x_at	622	298	180	74	370	308	722	479	286	236	121	191	215	213	250	653	655	795	246	211	155
213990_s_at	83	41	75	66	351	361	42	98	19	48	30	57	7	38	14	8	34	7	11	6	4
213991_s_at	172	23	35	25	230	160	94	30	71	88	20	34	5	8	19	41	43	32	5	46	4
213992_at	263	269	152	172	176	194	281	304	161	173	168	158	8	11	52	55	157	152	29	29	51
213993_at	79	62	8	56	133	54	17	38	54	48	102	13	2	2	1	8	2	2	14	19	7
213994_s_at	11	4	11	5	21	11	5	3	4	3	3	13	4	6	32	3	1	2	2	2	15
213995_at	217	420	545	755	843	1402	228	333	452	861	483	578	784	791	683	444	508	415	1590	1178	1267
213996_at	81	67	113	141	143	136	131	96	166	194	182	304	87	112	37	26	30	34	19	11	10
213997_at	89	21	47	23	205	270	28	92	28	143	37	224	39	17	66	5	6	3	4	4	2
213998_s_at	24	97	33	75	37	40	140	223	32	88	152	108	54	29	32	17	18	4	10	4	41
213999_at	148	163	145	26	188	110	133	150	78	200	105	34	62	167	210	93	158	132	154	109	84
214000_s_at	171	185	76	61	29	35	162	208	157	138	27	52	4	6	41	151	215	157	95	59	78
214001_x_at	385	687	496	480	246	262	572	598	478	372	324	236	149	226	509	617	403	518	660	631	351
214002_at	180	191	199	352	1438	1209	182	198	375	256	232	244	37	142	96	96	132	73	75	39	77
214003_x_at	25648	35358	18909	30978	17958	31919	28314	23453	19291	20552	15990	26544	22698	28708	23634	28927	30455	29685	19174	28703	25485
214004_s_at	351	543	448	559	381	466	325	432	348	310	507	315	378	436	244	302	336	278	314	288	178
214005_at	628	326	454	278	770	434	488	416	415	383	422	460	445	468	487	373	348	395	700	354	241
214006_s_at	448	230	434	349	489	360	639	504	669	615	466	439	323	483	214	285	245	284	311	199	146
214007_s_at	1357	943	914	745	1482	1266	2352	2176	1828	1933	2551	1891	198	183	269	460	374	397	303	155	765
214008_at	149	212	138	56	38	54	382	338	199	232	252	83	20	31	41	84	82	70	74	41	71
214009_at	79	49	134	88	256	210	46	83	104	113	92	106	37	46	72	40	63	97	116	107	116

214010_s_at	30	32	39	48	27	32	25	36	35	29	24	23	5	12	10	13	44	9	22	21	63
214011_s_at	2167	1207	2050	591	2422	2009	1875	1258	3145	2354	2328	1834	2885	2319	2611	1907	1777	1494	2089	1718	1249
214012_at	28	80	30	28	124	404	3	15	28	17	146	85	90	58	36	25	8	27	12	56	80
214013_s_at	33	12	50	54	16	19	98	18	33	52	10	15	4	13	31	10	18	13	4	2	3
214014_at	50	221	193	225	52	75	19	21	40	372	87	134	19	101	82	146	162	193	201	160	166
214015_at	222	72	227	153	125	146	155	212	234	267	159	123	37	21	63	33	60	33	65	41	7
214016_s_at	716	808	540	726	876	447	603	963	653	742	385	476	397	213	172	397	387	428	872	997	901
214017_s_at	340	411	403	350	630	525	299	292	329	376	317	194	186	234	220	183	203	171	224	142	183
214018_at	5	4	15	12	11	13	5	5	8	4	9	6	2	22	2	2	2	3	1	1	1
214019_at	119	66	54	72	38	22	83	90	19	68	63	44	32	4	3	2	2	27	6	14	7
214020_x_at	963	998	650	428	1446	1220	227	2291	2674	496	775	1943	511	676	542	379	458	335	400	352	287
214021_x_at	98	135	149	85	144	142	89	127	99	99	234	142	169	186	182	288	231	140	422	195	195
214022_s_at	235	903	502	714	1438	1004	326	276	852	808	2376	1200	1047	433	888	227	409	5015	452	2701	2230
214023_x_at	25	4	60	52	4449	6958	66	38	4	52	2578	5530	6060	5240	3610	15	30	12	32	147	98
214024_s_at	28	56	22	23	131	169	103	57	17	101	55	85	10	18	25	37	34	33	11	47	23
214025_at	111	96	24	22	326	27	138	84	147	35	33	39	25	27	39	29	44	23	32	43	37
214026_s_at	19	5	5	5	7	8	11	13	13	8	7	8	2	2	2	2	7	2	1	3	3
214027_x_at	45	24	39	44	199	105	30	28	33	29	31	52	18	7	66	19	14	14	57	14	35
214028_x_at	388	180	115	81	416	234	122	199	175	80	173	129	134	124	70	77	82	64	252	164	165
214029_at	5	16	4	5	14	31	17	61	52	18	14	9	20	15	21	20	2	18	9	4	3
214030_at	422	292	852	1037	509	375	351	472	803	1015	522	416	253	192	358	530	325	414	1012	643	767
214031_s_at	13	7	4	9	27	17	8	4	4	6	20	5	1	4	4	5	20	27	4	5	5
214032_at	9	7	15	35	392	36	32	20	8	8	695	141	312	633	467	3	2	5	5	4	3
214033_at	292	260	270	744	352	392	205	280	326	355	497	366	200	283	258	144	165	165	463	360	298
214034_at	19	50	41	79	22	133	54	79	74	81	50	41	11	17	3	10	7	4	22	26	19
214035_x_at	892	2355	1873	2258	1065	1575	1467	2283	1454	2460	1342	816	694	837	1133	1934	1822	1498	1810	2207	1915
214036_at	312	161	574	648	377	438	367	441	569	718	333	341	82	106	153	52	43	55	452	387	482

214037_s_at	177	75	104	65	487	462	25	15	146	150	102	80	212	172	139	75	88	53	144	164	157
214038_at	36	44	16	136	146	137	113	62	118	21	105	93	32	22	48	1	29	26	20	24	5
214039_s_at	14563	15837	5243	4105	3913	4014	11716	12268	6938	5231	4909	3525	6192	6582	9518	22689	24084	25144	11064	11876	11829
214040_s_at	122	326	402	1035	63	113	273	238	399	1753	951	341	47	20	45	20	6	22	28	47	66
214041_x_at	1688	3777	1553	1807	394	486	2263	3201	1193	1856	2234	2356	178	363	1002	1483	948	1602	1868	821	865
214042_s_at	2942	3484	3817	3519	3934	3997	4123	4752	3646	4172	2797	3151	3568	3066	3749	5507	4476	4089	4054	5811	3909
214043_at	16	5	50	16	43	55	19	32	11	11	13	15	7	3	4	10	7	8	15	10	19
214044_at	24	9	7	28	51	15	41	9	52	34	9	68	9	17	18	7	1	10	4	15	12
214045_at	44	49	31	28	81	151	315	277	214	138	153	23	357	312	362	587	686	636	351	513	576
214046_at	26	39	34	32	65	13	25	37	64	66	64	43	1	13	18	2	15	6	8	12	11
214047_s_at	497	543	1321	1156	1240	921	460	491	1191	999	1005	1090	989	1096	630	643	408	804	2112	1811	2953
214048_at	89	41	217	307	212	167	83	140	209	175	118	118	78	80	40	47	35	53	211	134	196
214049_x_at	48	34	50	53	487	296	19	22	27	21	23	18	36	34	25	27	14	28	15	38	11
214050_at	208	130	128	122	184	66	189	149	127	88	118	21	6	8	62	30	29	36	27	30	24
214051_at	115	124	133	102	617	224	139	183	162	151	600	821	740	790	749	36	26	35	55	42	46
214052_x_at	79	164	237	137	88	97	345	292	466	337	215	157	62	75	155	201	167	168	397	428	157
214053_at	168	49	194	217	272	160	118	170	229	252	189	138	8	13	7	13	34	15	41	5	21
214054_at	26	10	50	41	91	22	27	30	29	52	95	34	10	3	5	18	37	13	41	24	27
214055_x_at	565	607	616	626	114	249	852	1029	817	830	522	646	542	589	547	728	555	622	1104	1087	650
214056_at	427	232	487	283	157	219	288	277	293	312	230	194	127	184	384	368	225	442	599	273	242
214057_at	175	275	305	260	355	473	182	177	166	287	176	214	83	144	374	358	370	335	489	391	250
214058_at	339	234	198	1001	403	652	454	383	233	400	404	542	155	166	192	178	188	118	144	204	254
214059_at	40	34	99	212	92	60	122	92	123	136	50	30	23	4	13	10	24	109	56	188	351
214060_at	149	162	279	229	324	396	156	281	285	334	234	118	101	88	49	69	73	85	122	109	104
214061_at	352	202	780	354	243	216	335	379	545	935	216	232	211	227	203	370	343	365	1589	1015	1657
214062_x_at	280	117	232	12	135	216	192	181	118	39	347	202	174	134	124	138	105	163	154	127	37
214063_s_at	173	21	20	22	25	36	95	20	17	24	21	70	18	46	3	2	4	3	30	5	9

214064_at	149	144	61	106	81	172	89	22	24	122	139	92	37	20	58	69	21	45	42	30	37
214065_s_at	101	73	45	136	40	20	80	87	140	103	18	16	8	10	5	8	6	9	15	11	14
214066_x_at	228	237	125	269	564	501	191	185	261	236	139	173	56	67	34	111	88	83	149	113	130
214067_at	57	11	14	43	341	239	49	24	15	14	21	10	5	6	9	5	8	8	6	4	5
214068_at	155	158	195	162	146	308	175	181	132	152	171	177	40	51	72	71	79	47	43	122	114
214069_at	37	107	94	79	348	228	86	55	31	74	90	72	4	30	21	54	12	4	15	37	33
214070_s_at	493	269	458	623	418	468	439	464	356	422	348	505	35	72	51	41	38	52	69	114	109
214071_at	13	69	9	67	131	120	103	96	12	45	281	226	330	333	287	47	60	25	33	21	36
214072_x_at	409	292	275	238	346	419	352	396	321	280	247	253	41	11	59	153	54	130	67	76	43
214073_at	328	319	197	219	66	269	301	280	286	194	124	307	186	205	281	403	471	330	380	335	116
214074_s_at	427	321	395	451	235	206	400	434	708	674	344	282	164	82	144	118	179	150	217	134	126
214075_at	930	1159	479	411	393	403	771	905	479	510	608	562	293	136	343	513	465	143	425	254	140
214076_at	165	58	319	166	525	340	359	136	147	422	325	295	187	153	200	100	87	106	221	286	191
214077_x_at	101	67	203	140	452	515	159	144	123	137	267	201	45	76	37	16	58	41	47	56	25
214078_at	20	21	20	32	8	8	15	1	1	3	11	3	8	6	12	3	8	1	6	13	5
214079_at	172	133	361	162	2897	320	456	1046	300	686	2602	5687	2865	890	989	85	224	261	847	251	347
214080_x_at	1991	1893	2173	2592	1321	1762	2356	2703	2205	2425	2159	1454	535	839	1275	1930	2001	1697	1716	1873	1198
214081_at	99	124	104	198	92	75	119	179	96	140	114	93	38	6	20	15	37	31	12	13	3
214082_at	127	89	27	132	36	253	119	143	83	35	21	153	4	11	7	52	17	4	26	4	17
214083_at	93	15	26	21	173	355	38	136	19	103	156	102	121	132	226	151	147	177	144	141	108
214084_x_at	93	18	9	14	49	58	9	28	21	20	30	50	6	3	4	20	17	16	8	4	15
214085_x_at	332	221	486	172	54	22	399	335	416	288	287	167	59	57	167	300	129	399	295	212	238
214086_s_at	598	686	415	217	586	169	878	709	486	365	507	627	871	677	743	629	625	1110	1089	431	678
214087_s_at	106	69	73	114	121	19	87	99	48	115	78	107	21	15	19	5	7	6	29	39	23
214088_s_at	194	327	262	159	147	261	281	129	122	98	74	138	29	26	32	59	94	64	37	64	67
214089_at	7	18	109	44	37	38	9	13	25	10	17	9	4	4	2	7	15	8	5	6	3
214090_at	127	45	85	59	34	38	27	36	63	105	74	65	8	48	38	4	31	63	69	20	29

214091_s_at	185	118	1298	4726	1234	1474	203	185	1081	1314	1843	1710	560	569	546	47	27	46	374	261	227
214092_x_at	804	774	391	277	1299	1179	472	462	514	365	422	549	324	493	505	374	476	435	403	418	411
214093_s_at	287	280	372	513	401	361	216	285	585	463	262	323	442	471	318	254	340	262	998	516	777
214094_at	37	19	22	28	73	32	20	26	87	35	18	23	44	11	24	12	26	60	44	49	39
214095_at	572	307	665	110	726	660	511	388	587	312	652	597	940	668	925	694	549	789	755	1978	1900
214096_s_at	3469	2008	6705	2707	3868	5924	3874	2675	6144	4569	3779	3984	5363	4117	8222	6488	6036	6607	5665	11155	11980
214097_at	455	276	635	450	1372	968	473	412	585	632	1232	1217	3013	1922	1623	533	617	362	530	774	373
214098_at	79	44	81	127	96	138	82	96	83	94	61	48	49	46	34	37	32	24	36	77	111
214099_s_at	22	12	12	21	58	79	24	30	43	31	21	33	22	11	62	6	51	11	59	100	110
214100_x_at	991	1937	1016	1121	1127	1058	782	612	1124	903	972	920	954	1016	1337	745	633	660	867	1000	866
214101_s_at	304	310	274	270	470	625	303	437	179	252	325	293	259	202	327	399	278	311	330	129	209
214102_at	209	140	228	266	179	142	244	222	249	217	175	112	60	51	38	104	101	63	151	108	102
214103_s_at	30	21	31	25	26	17	32	26	24	36	34	50	5	14	18	4	7	9	6	15	7
214104_at	257	574	8	66	381	355	264	385	71	14	296	206	282	383	322	327	301	250	3	86	43
214105_at	103	23	122	114	115	214	20	55	142	49	65	77	19	19	27	17	65	59	26	42	11
214106_s_at	609	645	960	1605	826	657	620	348	985	778	381	483	581	521	352	396	547	395	925	1017	989
214107_x_at	1041	883	899	1957	903	879	1390	1338	1512	1374	1883	998	699	1156	1234	551	475	512	925	588	640
214108_at	143	144	140	145	150	266	138	28	187	117	163	139	117	96	117	72	57	102	48	91	63
214109_at	1720	1659	745	1102	866	1097	1484	1710	645	867	929	755	430	319	692	1553	1588	1581	1131	1133	1297
214110_s_at	142	243	141	167	37	714	168	208	219	95	259	251	177	124	277	138	135	128	130	218	66
214111_at	58	27	38	8	230	159	4	33	58	6	16	62	37	4	5	23	5	7	36	25	31
214112_s_at	741	886	2056	1880	1203	1670	837	714	1801	1368	1248	1399	1221	977	902	548	543	528	1626	1575	1418
214113_s_at	1684	1496	834	857	944	892	951	1201	751	808	524	865	1032	828	644	1780	1686	1469	1306	1436	1592
214114_x_at	1371	1636	1565	1707	1244	1591	1378	1120	1390	1704	1154	811	804	656	395	383	372	474	710	572	488
214115_at	11	6	7	14	16	35	9	12	8	11	9	9	2	4	7	2	3	4	4	6	1
214116_at	185	94	212	291	511	321	152	197	233	161	229	234	172	252	103	51	21	74	153	97	53
214117_s_at	114	71	208	454	367	419	130	199	185	328	308	230	348	359	123	42	76	52	179	108	115

214118_x_at	868	1030	644	618	1335	1754	1056	1655	669	805	1343	1600	875	649	1142	539	476	613	799	921	1034
214119_s_at	2211	1747	2033	1680	1961	1149	3946	3804	2676	2822	4555	3441	1200	1074	1142	1387	1509	1629	993	523	494
214120_at	62	2	43	48	14	12	50	18	5	32	11	3	11	3	2	8	2	12	10	4	15
214121_x_at	226	129	189	103	327	375	175	224	95	150	381	123	145	120	335	90	59	142	20	29	33
214122_at	44	12	19	26	213	125	32	110	13	27	107	90	25	8	17	9	42	17	6	7	8
214123_s_at	24	82	38	6	15	12	46	34	21	91	30	146	82	63	153	266	140	210	114	199	97
214124_x_at	373	203	343	490	302	301	379	362	553	463	438	486	536	358	365	282	283	235	591	405	351
214125_s_at	20	43	24	26	30	23	30	40	11	38	70	25	4	5	26	59	41	30	23	4	8
214126_at	149	135	23	25	221	321	211	153	27	50	50	182	314	254	266	114	148	185	193	342	471
214127_s_at	20	9	12	13	27	46	15	15	11	7	9	14	3	3	3	3	2	3	2	3	2
214128_at	167	117	87	191	268	360	29	116	161	95	38	92	8	27	10	7	5	13	6	48	45
214129_at	360	242	449	473	576	720	367	417	436	553	452	489	479	262	286	212	166	194	376	657	333
214130_s_at	85	18	58	85	77	67	65	91	71	115	91	139	123	56	22	48	46	35	51	103	100
214131_at	12	11	79	81	85	8	65	59	83	60	54	79	15	20	10	5	7	2	15	21	3
214132_at	173	119	155	363	63	157	147	181	284	261	171	84	25	60	37	62	30	55	154	149	196
214133_at	61	36	31	31	71	38	131	84	29	101	63	39	8	7	27	10	60	48	39	9	9
214134_at	33	16	15	21	41	23	27	24	16	8	21	28	2	3	2	5	4	4	6	2	5
214135_at	167	94	172	195	172	331	122	176	155	204	139	40	5	48	6	56	39	56	50	67	31
214136_at	86	119	121	202	257	366	135	129	128	155	111	116	105	174	110	122	64	106	129	180	191
214137_at	208	71	172	185	315	290	200	157	143	267	179	73	47	28	53	16	37	27	58	44	41
214138_at	180	169	119	138	32	56	183	157	24	133	124	161	51	74	60	59	55	52	33	136	82
214139_at	191	80	115	150	228	219	215	190	150	222	145	116	7	20	28	59	26	43	36	17	17
214140_at	143	142	170	177	162	262	76	94	140	53	94	113	133	146	100	130	103	102	385	212	292
214141_x_at	5798	2163	4353	2197	5692	1865	5959	5409	7043	5315	5490	4987	6991	6314	6559	5970	6334	5617	7345	4524	4604
214142_at	17	62	22	30	26	42	20	88	17	20	27	30	2	7	22	4	17	16	16	9	22
214143_x_at	15187	24993	20507	26924	14617	18046	18783	18242	21103	18173	15315	19751	16765	20837	19450	20738	22719	20791	14592	21313	14890
214144_at	230	105	246	169	416	241	294	317	207	321	300	305	230	250	259	280	251	244	419	187	228

214145_s_at	102	23	31	58	456	309	38	95	54	35	21	10	33	13	72	7	15	12	15	6	66
214146_s_at	4	9	53	44	95	7	20	38	12	32	67	67	2	3	25	17	1	3	9	14	13
214147_at	52	33	60	106	154	60	56	36	76	55	43	44	7	7	5	12	8	6	15	34	19
214148_at	202	208	214	266	355	474	236	339	106	186	234	263	27	59	108	154	113	75	124	78	34
214149_s_at	251	422	263	389	377	399	306	346	213	309	273	273	248	365	697	547	456	533	463	442	314
214150_x_at	2675	3631	2977	5411	5255	7122	3271	2982	3355	2878	3844	4517	3949	4558	6555	4994	5054	4215	5750	5039	4692
214151_s_at	101	249	275	384	19	423	135	132	189	226	122	40	130	181	184	154	234	235	353	764	964
214152_at	106	175	201	275	216	544	99	144	78	203	104	157	119	155	172	360	313	206	585	721	863
214153_at	103	167	106	142	38	24	148	158	84	129	136	166	38	23	7	23	37	4	35	64	7
214154_s_at	134	192	266	169	139	263	52	110	321	421	381	195	19	18	17	16	25	7	65	25	29
214155_s_at	216	129	449	167	564	591	314	379	341	338	496	381	509	394	539	515	359	445	566	410	576
214156_at	11	57	3	27	10	48	8	108	12	87	9	29	31	5	22	55	81	52	52	136	252
214157_at	460	338	1725	639	408	239	534	278	2208	1097	276	192	349	156	367	274	183	192	2367	802	541
214158_s_at	30	15	14	14	109	324	20	15	15	21	23	19	8	7	73	47	3	10	12	28	29
214159_at	37	29	52	53	162	70	9	42	106	91	51	21	22	11	25	8	8	7	28	17	31
214160_at	17	90	69	23	131	144	54	20	123	66	53	18	7	30	8	40	28	12	21	26	4
214161_at	69	26	9	9	30	28	106	13	127	55	9	11	3	3	31	45	24	68	26	3	1
214162_at	89	11	16	22	32	43	76	15	39	67	14	18	9	26	4	1	12	4	4	2	23
214163_at	112	97	155	140	302	194	90	271	128	172	141	177	41	31	27	27	23	33	92	24	76
214164_x_at	413	574	1416	2735	352	359	684	602	1999	3669	254	229	59	39	45	221	263	240	2540	2670	4030
214165_s_at	15	6	4	5	10	15	7	8	5	4	9	5	4	6	2	2	6	2	6	2	1
214166_at	32	33	189	35	66	48	127	24	91	145	105	94	10	3	56	17	25	29	22	31	7
214167_s_at	19554	15443	13444	12434	9286	13070	17632	18119	11738	11977	9322	11389	15808	17196	15182	23297	23890	23245	14384	21586	18817
214168_s_at	622	466	1035	693	959	892	705	781	756	1171	1025	1249	295	310	278	238	242	342	304	299	492
214169_at	148	97	106	115	275	316	121	115	114	109	199	215	295	232	298	115	85	105	126	90	37
214170_x_at	1961	1629	2226	989	2199	1629	3464	2037	1829	1888	2439	1830	3268	4042	5967	3680	3101	3681	4668	3727	4350
214171_s_at	29	12	31	98	23	26	42	17	131	32	63	14	3	11	13	9	56	13	43	7	15

214172_x_at	705	624	1192	1084	1193	1005	754	819	1104	1076	1049	1090	712	576	310	561	584	548	1493	513	1032
214173_x_at	1654	1679	2206	1310	1333	998	1167	1919	3454	1795	1218	1254	1342	1412	2310	2730	2218	2024	7516	3481	4784
214174_s_at	40	82	50	18	361	321	74	110	126	67	95	93	17	62	123	135	105	136	29	105	67
214175_x_at	112	24	130	26	1225	1012	61	32	23	28	2747	2294	2497	890	1047	6	43	20	22	2	9
214176_s_at	32	161	142	484	324	327	178	123	94	245	94	178	6	53	20	26	101	85	74	110	96
214177_s_at	181	426	193	1743	147	516	94	311	219	412	219	498	181	262	186	258	359	294	245	539	398
214178_s_at	222	84	91	203	484	211	162	150	165	133	136	158	23	27	43	11	5	9	43	49	28
214179_s_at	3432	2074	1232	1412	1146	3310	1476	1251	677	1145	1325	1140	940	723	371	541	676	674	347	555	290
214180_at	290	111	248	295	327	352	256	310	317	273	416	236	51	24	54	49	47	69	27	29	18
214181_x_at	32	17	16	22	34	26	33	13	17	20	20	26	7	17	5	3	12	6	5	11	1
214182_at	501	490	845	701	1451	1555	269	255	633	641	1134	1365	339	378	514	318	272	231	709	397	550
214183_s_at	20	10	23	25	227	99	25	25	25	20	105	90	103	158	84	8	3	7	5	3	2
214184_at	116	61	27	185	162	65	193	100	151	184	67	78	5	14	30	11	31	33	29	51	5
214185_at	140	137	152	181	187	176	241	191	171	270	223	170	35	36	24	30	65	49	15	47	35
214186_s_at	81	141	30	92	357	349	130	33	201	18	121	157	40	25	27	67	52	8	47	6	58
214187_x_at	106	109	132	168	172	301	192	136	230	337	190	187	26	82	159	39	4	4	55	84	52
214188_at	126	196	9	167	25	55	17	124	7	8	10	10	22	17	9	97	73	38	8	52	68
214189_s_at	81	77	98	184	379	185	123	148	219	141	124	94	32	40	46	39	20	26	64	41	33
214190_x_at	144	158	225	175	300	62	156	135	330	266	256	155	36	8	41	66	12	39	48	7	31
214191_at	19	4	23	53	184	97	16	13	11	60	26	38	7	7	31	7	7	60	25	24	3
214192_at	17	21	19	21	44	35	21	21	16	22	17	23	8	5	3	5	2	3	4	6	6
214193_s_at	312	315	319	253	265	331	237	300	230	282	203	391	338	205	176	481	416	470	738	473	713
214194_at	329	308	392	498	504	492	472	468	472	607	391	469	190	236	350	334	287	309	467	495	654
214195_at	30	18	39	30	55	93	30	63	28	21	20	21	23	5	11	8	8	8	6	6	5
214196_s_at	204	325	374	883	179	426	575	945	444	920	522	376	153	125	90	69	60	75	118	91	147
214197_s_at	291	258	433	392	175	328	412	371	365	412	303	246	89	67	148	124	132	102	175	161	172
214198_s_at	1123	1405	947	885	1368	1251	497	594	813	871	817	769	632	463	213	371	477	454	907	932	660

214199_at	12	10	8	12	25	48	8	12	7	10	10	13	3	3	5	6	7	8	13	20	6
214200_s_at	16	9	12	8	349	232	19	28	12	4	11	5	21	7	11	36	2	11	26	31	23
214201_x_at	464	561	537	401	753	438	641	728	490	519	583	244	310	306	125	258	228	260	285	194	246
214202_at	246	79	195	269	319	185	175	154	221	193	227	172	223	294	171	115	88	81	237	186	118
214203_s_at	106	136	128	247	67	241	117	92	82	113	37	46	66	58	52	140	96	61	77	89	66
214204_at	75	49	77	41	69	23	90	96	27	57	91	63	8	14	11	28	32	2	13	17	12
214205_x_at	737	454	1087	557	778	504	860	611	1608	957	1292	1088	1319	1699	1386	651	495	239	1418	719	684
214206_at	29	12	18	19	19	27	15	16	15	18	11	10	2	4	4	2	3	3	5	3	5
214207_s_at	61	19	14	30	55	360	20	7	13	15	20	29	4	6	52	23	19	18	44	48	8
214208_at	74	22	27	26	412	249	36	36	32	46	26	25	6	19	29	6	7	6	10	29	7
214209_s_at	193	192	83	30	214	301	245	248	107	166	243	134	23	13	27	227	220	220	177	96	80
214210_at	209	128	283	291	188	245	225	169	377	317	215	325	235	178	257	169	181	152	307	230	314
214211_at	5064	21877	3685	17977	1957	17840	4142	3577	4190	3532	1528	3373	4259	4435	6954	8251	9695	5789	7642	12707	9382
214212_x_at	198	145	64	72	242	51	225	186	66	87	220	166	178	277	160	195	114	83	17	24	33
214213_x_at	560	538	792	912	1293	1340	600	610	613	815	767	424	210	247	334	243	295	307	301	277	166
214214_s_at	8659	6839	5639	2023	6071	4127	5046	4737	5413	4042	3878	4221	9428	8618	6847	8988	7461	10381	10938	8298	7477
214215_s_at	74	34	46	36	29	28	106	24	25	81	84	53	7	14	20	39	38	2	22	23	15
214216_s_at	209	204	100	79	73	56	257	206	285	271	34	20	5	10	6	4	6	19	3	22	5
214217_at	49	47	65	67	87	89	78	32	87	20	92	77	9	6	27	24	5	5	22	2	2
214218_s_at	143	61	84	98	187	151	61	124	82	67	67	31	17	18	5	21	15	25	25	41	23
214219_x_at	20	11	18	26	25	23	16	24	17	17	13	15	10	7	5	5	21	5	6	5	7
214220_s_at	321	189	146	215	526	271	391	343	203	257	247	265	285	208	275	204	200	271	335	351	442
214221_at	669	337	324	242	408	357	278	380	396	510	412	236	268	216	263	315	291	328	449	470	446
214222_at	177	40	98	189	525	824	87	117	102	55	102	77	37	20	8	52	39	55	61	78	60
214223_at	56	21	28	34	1890	1611	16	30	35	10	54	35	56	120	95	84	117	74	109	58	59
214224_s_at	660	645	1077	1749	2401	2074	1016	808	1081	1263	2508	3007	3890	2464	2004	630	735	714	1715	1590	995
214225_at	155	158	217	215	187	69	204	206	165	228	200	245	73	29	14	57	40	47	75	31	34

214226_at	32	236	38	52	26	137	42	161	60	49	142	44	54	38	94	152	234	156	122	37	73
214227_at	32	11	9	34	34	20	33	28	16	32	27	18	13	4	9	10	8	2	6	30	6
214228_x_at	120	49	22	52	278	40	16	18	16	17	17	20	6	11	6	6	10	12	21	9	3
214229_at	169	157	106	150	201	173	176	100	138	140	104	128	2	4	64	16	21	4	2	7	5
214230_at	105	68	87	53	232	171	85	65	119	119	67	43	16	50	55	24	9	41	90	42	40
214231_s_at	209	135	271	98	308	165	227	269	487	303	313	263	171	126	166	196	106	180	350	178	179
214232_at	283	241	256	339	115	148	322	306	302	328	242	220	50	80	62	144	118	119	134	110	28
214233_at	323	219	263	283	381	344	257	300	229	291	161	180	4	24	37	8	10	11	10	27	13
214234_s_at	16	10	833	1160	137	275	20	7	549	1665	306	244	53	43	66	5	3	11	506	432	1149
214235_at	74	33	1089	953	297	318	36	37	832	1853	284	259	33	53	52	34	2	3	479	617	525
214236_at	97	55	118	110	291	266	110	143	78	82	505	282	69	117	76	3	52	62	13	29	21
214237_x_at	143	159	172	141	307	218	151	92	186	141	175	265	104	9	9	9	25	17	8	24	39
214238_at	128	66	113	147	140	117	119	121	72	147	50	72	8	20	12	19	42	52	55	33	37
214239_x_at	755	1012	887	736	1109	988	615	864	558	901	668	904	922	947	624	781	808	768	962	1530	1368
214240_at	1376	714	64	65	606	56	1340	690	63	35	1150	424	2453	1246	1408	1253	1498	1652	201	904	488
214241_at	318	198	309	331	372	302	401	392	344	293	267	235	61	75	94	98	158	127	182	99	118
214242_at	17	12	33	28	33	26	86	21	28	53	26	63	14	12	7	12	6	6	4	22	12
214243_s_at	28	16	53	30	115	65	29	30	27	56	27	28	7	8	11	24	3	3	4	7	7
214244_s_at	134	118	123	78	246	275	87	78	19	99	105	85	53	116	196	118	95	142	146	122	94
214245_at	291	136	267	164	386	286	268	240	383	361	209	243	34	52	78	54	58	75	90	62	67
214246_x_at	1610	2439	2500	3983	1607	1459	1405	1473	1943	2163	1366	912	762	1243	1729	1716	1828	1783	2495	1910	2126
214247_s_at	8	9	27	9	1530	6045	42	65	40	59	611	395	941	1049	1157	23	27	8	46	25	25
214248_s_at	49	30	18	35	120	267	27	18	19	20	131	26	149	89	73	9	4	6	11	12	29
214249_at	44	11	35	27	27	17	20	127	11	48	81	65	11	28	7	33	24	17	7	7	5
214250_at	8	23	16	32	38	39	8	20	24	77	13	11	7	30	9	7	5	11	8	2	3
214251_s_at	381	450	821	961	198	161	452	594	844	1424	1008	484	28	49	10	17	6	20	53	5	2
214252_s_at	455	620	273	336	214	413	274	435	159	242	243	381	266	210	107	242	257	321	222	159	259

214253_s_at	42	165	34	25	118	133	21	112	31	17	97	26	4	6	5	52	8	4	7	6	3
214254_at	146	36	9	93	159	24	68	71	84	59	58	54	5	3	27	36	29	20	25	32	27
214255_at	119	97	26	176	345	169	16	91	41	64	55	20	62	20	47	2	12	30	31	20	22
214256_at	86	88	212	186	88	79	174	158	198	180	152	177	44	24	43	26	25	36	61	16	19
214257_s_at	394	319	2298	2407	2612	3231	525	528	1065	1476	1379	1904	925	1169	1117	456	332	502	3230	1837	2763
214258_x_at	1308	1383	1489	1619	991	873	1252	1289	1346	1300	1269	1048	771	809	411	508	548	274	587	438	349
214259_s_at	3703	3162	1358	1856	2010	1783	1524	1991	912	2129	1646	3614	1861	1963	1327	1857	1715	1405	1611	2219	1186
214260_at	229	175	151	154	115	235	237	179	159	243	135	106	110	43	97	156	139	177	234	143	161
214261_s_at	111	127	228	211	246	134	209	71	157	261	162	89	17	40	56	45	48	12	19	46	28
214262_at	24	36	71	13	157	23	53	13	12	56	18	23	60	56	72	85	81	51	67	37	31
214263_x_at	1442	1384	1180	1040	888	1290	1787	1335	1511	1263	846	1220	1767	1582	951	1213	1250	937	1379	1271	1060
214264_s_at	778	395	328	335	873	544	1346	1335	293	334	1282	1420	1055	858	892	772	749	801	464	395	298
214265_at	112	102	75	172	416	48	151	183	161	165	99	94	5	17	48	15	19	11	13	13	20
214266_s_at	364	325	385	154	87	280	360	434	324	284	588	367	78	90	105	55	85	182	77	15	42
214267_s_at	13	10	15	12	63	273	11	11	11	10	14	15	7	11	7	6	4	45	20	8	7
214268_s_at	1635	1084	1507	1017	1467	1552	2188	1403	1444	1226	1232	1033	473	646	978	1728	1698	1770	1062	1305	1687
214269_at	58	10	9	9	32	17	23	37	17	14	30	15	4	3	3	3	3	4	2	2	2
214270_s_at	19	120	473	255	45	195	131	161	296	744	341	29	28	53	27	22	35	42	69	85	47
214271_x_at	29462	32686	18171	24285	20621	17821	23246	24889	15167	17115	15633	29781	17499	22081	21519	24535	27896	25110	12136	21358	13723
214272_at	114	85	77	224	386	409	140	137	155	122	136	165	29	88	56	57	17	22	11	36	14
214273_x_at	441	476	610	692	372	453	606	524	474	628	412	294	176	221	197	207	146	178	274	298	266
214274_s_at	869	963	1256	2118	1451	1579	564	677	1171	1020	644	1044	1438	1023	671	665	676	678	2162	1391	1108
214275_at	91	91	136	100	52	118	68	150	150	177	92	148	5	19	34	4	2	2	2	26	4
214276_at	225	184	265	244	315	361	179	348	285	320	225	216	166	148	161	47	106	85	243	127	205
214277_at	184	198	69	101	103	212	61	88	58	57	58	54	85	94	48	105	82	116	213	89	83
214278_s_at	40	24	39	39	114	117	32	38	45	20	26	88	25	9	9	35	12	13	20	6	17
214279_s_at	306	157	140	266	32	129	284	294	280	344	233	186	25	18	16	65	12	32	77	24	27

214280_x_at	1971	3401	2684	2665	3691	2936	4167	3485	3422	3101	3257	3428	4628	4474	3738	4519	4902	3379	3376	5914	5082
214281_s_at	1013	732	520	260	657	695	910	676	443	264	816	430	1008	834	1020	1371	1358	1637	1031	1128	1322
214282_at	26	4	47	4	87	12	1	12	28	10	4	41	10	6	23	16	21	13	13	14	18
214283_at	321	277	513	305	495	312	717	379	355	379	485	401	461	530	565	674	578	503	710	614	732
214284_s_at	42	46	80	94	190	126	135	103	35	119	117	77	50	86	50	43	30	42	40	76	81
214285_at	17	43	72	19	148	70	30	16	11	73	82	118	122	148	266	23	5	32	25	7	15
214286_at	36	24	18	18	55	75	44	22	80	39	31	38	19	3	4	17	24	6	25	18	26
214287_s_at	197	122	61	63	220	105	187	121	155	143	135	147	27	3	45	37	51	36	53	32	11
214288_s_at	10621	6509	6206	4555	9304	7673	11153	10551	6624	5729	9768	10549	11835	12049	11195	13588	13194	13344	9902	7892	7118
214289_at	83	30	19	70	95	23	94	58	19	18	74	18	17	11	5	2	5	9	6	48	1
214290_s_at	470	1459	274	4599	3199	3811	568	686	183	264	6715	12999	8098	3818	2091	252	362	247	231	960	504
214291_at	325	429	165	184	177	114	334	356	321	323	189	114	50	58	100	150	202	191	163	238	203
214292_at	20	6	16	18	59	36	15	20	20	32	18	13	7	7	15	3	6	49	15	4	5
214293_at	193	189	176	171	269	234	196	220	191	176	132	139	105	100	152	220	146	116	237	188	232
214294_at	107	66	71	67	713	403	64	106	99	130	101	104	44	25	23	47	21	27	29	31	25
214295_at	71	80	175	120	22	28	130	181	347	307	149	148	10	13	21	3	22	6	43	41	33
214296_x_at	58	55	72	12	794	497	27	128	29	25	24	19	23	37	36	55	45	49	41	79	52
214297_at	171	83	30	93	34	97	142	99	52	481	88	68	4	2	6	30	37	27	107	32	30
214298_x_at	78	135	227	264	445	456	196	207	241	196	324	298	145	127	152	86	81	109	57	64	48
214299_at	157	56	180	146	665	423	170	241	213	285	216	274	215	173	185	161	114	139	200	249	217
214300_s_at	142	118	167	35	242	203	241	191	98	218	283	178	29	15	19	18	6	59	29	11	21
214301_s_at	107	10	90	133	316	140	42	55	135	99	36	93	29	6	38	38	14	16	20	10	58
214302_x_at	15	16	27	28	26	30	21	17	19	17	16	15	8	4	16	5	8	8	4	5	4
214303_x_at	16	12	427	3053	26	42	13	16	91	38	16	16	5	9	2	2	5	3	45	169	136
214304_x_at	4	2	4	4	18	13	3	5	3	4	4	6	2	2	2	2	3	2	2	1	1
214305_s_at	1916	714	1441	1642	2181	990	2067	2534	1944	1934	2004	1693	818	488	424	374	340	305	480	368	397
214306_at	221	270	252	210	272	388	246	234	285	278	304	290	384	342	512	404	304	294	536	487	674

214307_at	111	7	153	629	45	191	24	70	99	94	3	4	4	22	7	15	4	3	138	116	112
214308_s_at	64	44	293	1485	176	56	61	25	183	191	97	99	11	6	50	16	38	15	172	155	185
214309_s_at	30	32	19	13	37	35	24	22	128	61	139	21	29	9	30	27	11	4	36	9	3
214310_s_at	213	237	256	365	33	50	280	286	277	405	412	337	284	266	117	85	38	86	102	82	72
214311_at	54	16	22	23	158	554	19	51	33	66	21	50	130	77	27	13	107	73	57	106	63
214312_at	111	108	115	162	96	79	91	135	139	155	41	33	35	26	34	18	32	7	27	34	15
214313_s_at	126	103	140	114	98	20	191	236	162	257	168	45	108	65	145	162	77	101	252	110	88
214314_s_at	421	230	269	132	641	367	237	247	235	169	117	215	1245	807	1147	1155	891	944	2868	1348	1290
214315_x_at	6312	4126	5254	2515	1325	954	4451	5515	3377	3599	3199	1741	1122	1041	1496	1996	2120	1203	2483	2399	2656
214316_x_at	249	156	309	273	1311	938	159	117	364	260	441	555	195	250	398	308	306	323	339	443	386
214317_x_at	7150	5048	4438	5662	4772	4949	5577	5088	3710	4191	4231	4704	12459	5891	4514	12864	13119	10233	10532	16208	3232
214318_s_at	62	60	153	213	47	152	89	79	138	166	163	65	50	60	24	47	25	8	69	34	35
214319_at	107	55	84	80	254	403	68	71	91	87	104	101	80	76	40	29	29	13	136	32	66
214320_x_at	311	181	199	150	78	58	425	323	256	187	189	268	10	36	52	17	6	62	13	26	35
214321_at	200	1058	52	89	124	189	148	223	43	46	33	44	40	38	55	459	516	486	31	47	55
214322_at	210	215	241	476	209	200	281	371	476	355	183	175	62	61	59	71	74	8	106	74	19
214323_s_at	1396	1724	1477	1619	1856	2380	1132	1283	1725	1845	1936	1920	2082	1750	1798	1709	1871	1754	4960	4971	6639
214324_at	101	19	18	59	235	81	24	92	24	29	45	41	2	4	8	6	5	6	25	6	4
214325_at	9	11	16	26	22	20	24	20	20	22	20	13	2	4	2	3	2	4	2	2	2
214326_x_at	1094	2722	582	674	257	407	657	743	143	354	364	158	42	5	12	96	66	77	22	8	6
214327_x_at	22823	23346	19540	23485	24594	26999	10234	22983	16599	18943	15379	24952	20534	26270	25972	24930	25566	27399	17342	24108	21281
214328_s_at	24583	19405	14674	8810	35959	22789	25856	22918	19515	13659	19493	24900	21212	24516	27279	25897	25028	29809	17792	17843	19907
214329_x_at	37	85	60	203	275	349	81	44	55	61	55	82	60	57	50	23	24	27	122	320	553
214330_at	222	120	134	189	331	126	195	194	149	224	212	131	96	112	128	59	121	68	109	82	112
214331_at	119	72	248	172	30	30	121	176	317	408	115	192	254	186	178	120	100	80	667	278	375
214332_s_at	123	127	267	178	405	513	158	119	254	249	165	160	63	110	147	64	37	67	400	108	150
214333_x_at	378	298	609	1045	115	59	395	285	839	523	394	256	313	400	249	234	286	228	383	385	244

214334_x_at	4419	4866	3298	5146	6941	5522	3385	3897	3872	3680	5401	6452	3370	2221	8637	7902	6917	3978	3201	6672	3844
214335_at	131	248	269	336	187	399	205	228	338	415	283	307	51	64	78	65	59	69	106	82	62
214336_s_at	385	116	987	932	525	804	855	1077	991	1208	881	530	59	33	60	98	57	80	86	44	72
214337_at	34	38	31	40	106	70	7	40	17	28	26	15	30	32	48	95	65	59	64	86	33
214338_at	200	60	218	181	854	488	191	194	161	210	126	205	103	159	60	42	149	127	130	80	90
214339_s_at	90	73	146	119	59	34	260	238	193	226	219	216	60	87	77	70	119	59	71	44	54
214340_at	220	50	49	178	34	43	80	66	47	59	40	79	6	27	68	23	7	27	6	5	3
214341_at	21	28	71	84	327	309	13	28	78	13	81	21	20	9	40	13	38	58	80	34	29
214342_at	58	18	50	98	47	40	80	40	115	61	301	199	174	193	112	40	33	28	64	46	39
214343_s_at	15	7	11	18	368	265	16	75	135	87	244	362	591	488	305	48	17	49	96	71	32
214344_at	21	17	100	266	25	23	129	42	76	34	111	68	5	3	4	2	16	3	13	4	3
214345_at	202	168	149	230	430	364	168	241	239	217	210	147	34	72	40	24	47	61	53	46	37
214346_at	5	51	9	17	8	15	7	11	5	8	6	6	4	4	2	1	2	3	2	1	3
214347_s_at	22	6	118	76	114	59	11	36	270	50	11	8	2	4	5	8	5	27	170	142	155
214348_at	40	32	60	67	43	55	99	51	41	102	41	43	3	6	4	12	14	12	9	8	5
214349_at	315	148	319	309	1146	1052	250	359	332	403	321	212	43	58	98	79	94	73	186	80	40
214350_at	105	90	117	78	106	70	72	90	71	85	80	84	5	18	28	4	7	26	9	36	14
214351_x_at	23646	35201	14724	17673	12548	10706	17592	26126	19060	15406	11927	22314	20729	18649	10612	15415	16609	17990	10349	18994	9951
214352_s_at	667	911	815	1407	510	746	1085	1450	1428	1266	2116	2653	1242	1176	1568	731	676	445	725	682	672
214353_at	61	92	15	16	267	242	68	21	32	55	14	16	30	9	48	96	78	93	66	88	48
214354_x_at	380	315	321	472	392	489	313	352	333	495	391	468	104	106	102	124	23	125	164	157	72
214355_x_at	996	1189	1077	1508	1117	1838	1085	827	811	910	1082	1273	616	428	437	571	420	547	463	772	832
214356_s_at	1306	816	1040	673	1928	2130	734	781	686	889	1097	1100	1292	1164	661	994	867	1171	1225	1793	2092
214357_at	161	117	273	270	169	175	139	298	296	376	185	141	20	54	47	15	8	6	67	25	17
214358_at	232	197	815	384	36	46	200	136	831	416	188	83	306	299	352	300	268	245	845	422	226
214359_s_at	14740	3617	8928	3310	6930	4839	18137	14856	7950	8286	9207	5498	5879	4590	3638	7533	6440	7761	4116	2675	3305
214360_at	95	60	15	71	10	71	98	74	11	67	78	101	4	21	3	6	21	1	18	18	6

214361_s_at	138	136	9	36	65	77	9	46	54	109	31
	8	17	23	6	4	12	41	9	6	43	
214362_at	16	16	15	18	48	31	48	9	23	28	21
	18	5	4	7	7	6	7	19	7	14	
214363_s_at	6338	5528	7903	8478	7332	5056	6319	6982	7733	7744	7498
	5797	9781	9527	11062	11533	10277	11543	9674	12683	13924	
214364_at	202	198	153	257	278	164	277	176	198	126	195
	230	136	141	129	159	156	190	106	89	97	
214365_at	135	103	39	132	80	30	156	120	135	169	95
	45	42	13	9	8	22	8	40	9	5	
214366_s_at	34	268	71	694	30	91	72	49	67	155	21
	5	4	3	19	30	43	6	128	194	160	
214367_at	57	41	113	53	537	429	68	42	56	88	74
	52	53	7	28	64	45	25	51	46	37	
214368_at	61	16	41	50	322	445	42	66	75	73	40
	23	37	45	25	26	32	18	7	16	23	
214369_s_at	239	111	87	113	542	335	192	182	115	96	104
	97	58	71	70	110	126	58	44	26	39	
214370_at	3	1	8	5	15	8	4	4	4	4	3
	3	4	2	1	1	1	2	1	3	2	
214371_at	106	89	83	81	22	50	119	88	74	109	77
	85	10	8	22	16	16	12	14	25	22	
214372_x_at	208	35	324	521	704	546	276	140	258	330	401
	69	90	26	44	45	9	48	435	673	575	
214373_at	237	571	378	643	315	649	396	282	380	340	236
	231	76	57	72	55	89	70	111	219	186	
214374_s_at	119	12	41	150	412	280	76	46	31	22	445
	532	549	471	376	83	44	51	31	37	27	
214375_at	64	1	69	17	243	219	62	53	45	32	98
	302	101	102	66	16	29	24	33	19	19	
214376_at	81	12	111	67	214	235	90	121	78	50	90
	117	23	39	62	19	17	32	48	16	21	
214377_s_at	337	190	316	353	227	245	215	352	277	358	226
	351	47	14	50	55	42	77	101	99	57	
214378_at	108	86	138	272	122	109	135	95	88	109	65
	44	20	10	2	50	63	30	95	71	57	
214379_at	57	43	52	85	235	278	52	78	83	92	51
	64	39	24	23	40	28	40	33	47	36	
214380_at	26	23	23	79	66	47	13	59	11	11	21
	30	8	4	3	8	6	12	15	3	5	
214381_at	71	71	247	228	104	216	174	55	50	124	129
	94	48	11	26	12	4	4	7	21	41	
214382_at	17	13	11	131	45	43	17	15	21	15	14
	23	15	3	11	5	24	20	13	49	48	
214383_x_at	856	633	1085	1296	352	478	1064	1137	1151	1514	658
	405	297	374	321	603	594	725	888	481	432	
214384_s_at	87	26	81	93	272	215	105	119	76	36	117
	74	7	12	55	26	10	10	6	9	19	
214385_s_at	99	17	624	3435	22	83	11	16	740	479	9
	23	4	3	5	3	3	4	180	312	133	
214386_at	30	28	39	23	71	35	48	44	31	36	24
	11	7	6	54	13	10	12	8	9	6	
214387_x_at	40	46	42	44	63	39	41	42	50	43	40
	40	4	12	9	12	9	11	6	7	7	

214388_at	56	13	62	113	47	132	23	66	104	73	87	93	2	39	34	40	25	22	19	31	27
214389_at	36	4	5	4	11	12	21	33	29	1	16	19	1	1	28	4	3	19	8	2	5
214390_s_at	34	19	28	38	38	129	29	42	23	25	24	25	4	4	4	5	5	9	4	7	9
214391_x_at	48	38	54	67	109	102	57	48	58	63	50	46	80	53	27	55	15	64	80	96	20
214392_at	29	36	71	6	44	98	76	63	62	115	63	18	10	4	5	6	21	2	15	26	26
214393_at	13	11	11	10	219	65	9	12	16	13	10	9	25	26	22	13	1	14	21	9	31
214394_x_at	10693	24131	5628	8767	6316	8249	7791	7338	6066	4755	6724	7905	12027	7541	6813	8610	9924	5335	7115	12842	8596
214395_x_at	312	508	218	234	195	196	481	372	320	275	296	182	74	193	385	640	221	338	325	264	183
214396_s_at	180	170	118	56	253	313	125	102	103	35	30	31	26	6	2	48	6	29	24	22	19
214397_at	112	62	33	116	106	116	66	42	92	63	44	30	14	24	25	13	17	25	31	18	17
214398_s_at	233	120	263	297	624	465	286	290	353	327	517	469	157	149	58	59	64	88	117	72	55
214399_s_at	11	6	5	14	18	54	9	9	5	7	7	16	15	6	7	3	15	2	6	2	16
214400_at	16	19	19	36	11	59	25	104	19	14	58	49	43	20	3	3	3	3	4	3	6
214401_at	42	150	98	113	209	271	65	154	161	314	256	176	7	24	7	14	6	67	10	61	22
214402_s_at	171	209	119	93	82	219	56	119	55	101	68	44	77	46	74	95	41	55	98	67	38
214403_x_at	156	260	250	219	895	375	133	220	210	126	132	183	66	53	172	140	215	256	130	109	81
214404_x_at	64	60	244	676	60	83	66	59	583	116	74	44	7	5	6	10	5	5	189	307	191
214405_at	42	7	4	5	8	31	4	7	7	4	37	8	1	7	17	19	4	5	1	20	9
214406_s_at	19	13	28	16	25	24	34	30	27	28	13	29	40	7	5	20	22	5	8	9	6
214407_x_at	15	50	122	140	51	179	207	91	151	99	189	157	25	4	6	5	5	8	17	6	7
214408_s_at	70	66	54	88	87	55	66	102	59	53	50	93	7	50	34	50	13	20	34	17	6
214409_at	52	32	73	93	78	129	61	78	21	57	57	50	11	1	3	13	8	14	15	35	15
214410_at	11	71	47	52	27	59	12	83	23	68	91	96	4	1	3	7	3	26	6	4	13
214411_x_at	148	100	252	231	390	195	193	156	231	236	205	191	17	27	14	8	23	62	8	13	15
214412_at	8	5	7	16	41	46	12	7	28	7	11	5	32	4	34	8	2	22	49	6	20
214413_at	8	5	14	9	162	42	7	17	25	15	13	14	40	27	17	3	3	4	3	4	3
214414_x_at	176	40	168	160	195	46	131	154	217	218	125	160	138	97	365	63	12	27	419	107	59

214415_at	25	39	14	34	10	11	8	16	13	34	40	50	14	31	31	29	23	47	48	86	64
214416_at	90	56	83	132	78	250	65	95	36	75	28	38	2	13	8	2	1	4	8	14	10
214417_s_at	85	127	113	136	302	274	160	127	134	190	172	168	29	28	17	21	47	35	43	35	18
214418_at	8	15	14	6	32	36	15	12	16	20	17	24	29	7	9	5	6	2	15	8	9
214419_s_at	119	111	161	111	210	251	139	136	187	141	99	106	34	41	56	48	70	54	81	49	13
214420_s_at	16	63	19	71	52	44	86	22	13	22	26	48	8	40	12	8	5	8	13	9	15
214421_x_at	185	197	210	171	364	317	284	174	226	231	189	210	47	63	34	39	21	43	47	60	33
214422_at	28	6	56	71	60	43	37	65	24	46	13	62	13	9	8	1	1	6	1	22	11
214423_x_at	258	162	239	274	515	473	294	269	301	428	294	200	53	46	50	6	41	55	78	99	43
214424_s_at	102	92	115	98	173	118	107	117	86	67	68	101	27	2	31	22	16	4	21	9	10
214425_at	189	99	229	216	304	273	97	185	210	217	173	132	54	7	37	57	43	49	34	53	27
214426_x_at	654	505	514	207	1390	136	966	1017	648	535	661	411	533	690	505	513	714	465	662	463	304
214427_at	1959	990	1778	1195	2609	1556	1481	1504	1647	1651	1647	1533	1659	1751	2245	1387	1438	1429	1691	1354	1442
214428_x_at	172	107	183	358	495	667	90	176	181	159	239	182	46	47	44	69	50	37	44	68	33
214429_at	532	573	701	506	925	1060	392	480	468	541	1045	803	1731	725	491	347	321	534	1375	1518	2435
214430_at	1057	914	2024	1432	2807	1387	1305	1170	2240	2376	2884	3912	3355	3030	2767	737	849	1500	2155	2028	1613
214431_at	1091	984	2755	1604	3500	1579	1287	932	3200	2267	3938	3034	4257	4292	6511	2919	2372	2433	5323	3560	4483
214432_at	131	106	171	107	16	177	290	234	91	112	183	128	17	11	40	66	102	74	46	37	39
214433_s_at	138	88	321	4722	267	294	74	103	475	377	216	212	140	132	92	31	2	4	410	576	505
214434_at	429	456	144	132	620	632	692	664	135	210	696	582	490	542	1082	334	304	289	118	96	99
214435_x_at	972	1016	1226	631	1419	1537	714	951	929	1003	1226	1081	2114	964	2149	1229	492	254	2085	1151	802
214436_at	336	411	23	85	78	419	240	437	20	27	131	397	135	136	237	257	138	207	39	57	74
214437_s_at	1480	386	2448	582	1637	1965	949	662	1565	1312	1529	877	1836	1168	1775	1544	1207	1387	1447	3240	3329
214438_at	201	96	88	102	58	56	178	152	115	98	102	116	7	6	11	12	34	16	8	18	19
214439_x_at	987	1145	1058	1432	757	1149	978	864	1628	1536	1099	643	396	312	375	321	370	311	414	337	312
214440_at	349	468	461	676	1098	981	509	413	392	396	989	1087	831	692	704	459	531	523	655	606	630
214441_at	226	218	265	142	349	348	294	359	395	326	452	278	211	283	89	96	81	100	106	81	94

214442_s_at	503	445	391	451	96	184	511	612	535	454	391	241	264	254	324	607	529	517	468	217	248
214443_at	282	313	738	248	355	222	297	234	500	376	300	136	43	52	36	16	34	80	62	30	70
214444_s_at	161	249	884	353	224	265	93	293	554	475	405	243	8	41	17	13	39	41	10	11	10
214445_at	69	105	102	18	351	226	131	132	158	137	82	33	21	17	2	3	15	14	5	29	17
214446_at	126	56	152	72	51	22	110	38	41	137	41	40	34	12	49	71	57	17	62	70	146
214447_at	163	105	307	191	316	341	180	174	98	127	402	288	77	74	55	79	35	50	64	62	21
214448_x_at	380	400	487	446	256	431	478	396	479	372	441	165	303	281	269	217	250	307	172	209	144
214449_s_at	249	611	202	189	254	590	510	408	292	198	354	215	538	477	361	392	582	424	163	233	434
214450_at	26	17	26	32	47	31	15	20	25	22	21	31	7	4	6	5	7	4	7	13	27
214451_at	8	28	7	17	32	44	52	22	7	32	9	43	11	46	3	7	21	5	4	8	4
214452_at	54	12	69	57	135	43	49	34	27	56	13	29	2	27	6	8	12	1	8	19	2
214453_s_at	102	129	176	319	179	130	127	110	322	380	81	55	13	7	46	44	164	1562	308	852	1341
214454_at	111	134	99	135	33	26	33	102	108	61	98	33	45	9	37	14	12	11	8	8	41
214455_at	81	60	83	81	399	281	30	48	64	75	568	721	380	205	141	47	28	36	72	67	55
214456_x_at	40	16	9	43	1410	770	9	32	12	6	3989	13339	3055	1895	639	5	14	10	8	6	9
214457_at	11	10	117	105	11	20	25	15	138	35	7	65	16	6	42	2	18	14	115	112	125
214458_at	169	114	149	119	436	426	168	127	107	108	141	109	55	62	19	85	85	59	81	80	76
214459_x_at	7367	19292	2732	6444	4173	3622	6687	7097	2015	3466	6567	4400	4959	4124	2461	4246	6048	7322	1011	2323	1641
214460_at	30	11	23	27	21	19	12	17	17	18	17	16	2	7	3	5	2	2	2	4	1
214461_at	7	4	7	6	8	15	9	9	5	10	6	9	4	2	4	3	14	1	5	3	3
214462_at	806	601	321	226	166	149	681	656	397	219	205	210	52	45	69	277	153	335	79	70	99
214463_x_at	30	106	37	53	48	69	30	54	27	27	92	664	272	140	165	82	90	142	57	57	5
214464_at	94	10	133	118	203	204	261	288	297	559	209	239	699	641	584	942	704	416	395	638	540
214465_at	19	15	19	18	18	31	21	25	20	28	18	23	4	2	6	3	25	5	4	5	18
214466_at	66	28	26	125	25	30	82	18	28	25	58	88	4	4	4	2	11	2	8	2	3
214467_at	52	16	16	9	59	32	49	62	56	41	58	39	25	3	26	5	20	9	20	18	14
214468_at	93	45	7	14	33	28	13	15	4	25	7	13	4	4	13	12	3	5	2	4	11

214469_at	8	4	12	103	245	406	8	24	12	14	67	440	136	95	91	3	1	3	2	42	4
214470_at	15	18	102	162	60	46	34	26	62	122	102	116	23	7	4	12	3	3	4	7	30
214471_x_at	85	147	240	239	29	28	219	123	83	147	63	60	10	51	61	35	19	55	55	65	11
214472_at	83	139	92	81	70	339	46	29	76	71	212	256	251	93	52	24	25	13	64	85	95
214473_x_at	751	609	1030	1159	1487	2161	1134	1264	1033	1413	1991	1935	983	810	1083	407	368	296	703	554	458
214474_at	225	139	193	287	351	349	217	338	197	247	212	171	60	38	41	31	51	32	62	39	48
214475_x_at	7	5	4	5	15	13	7	8	8	3	6	5	2	2	10	4	12	26	1	2	1
214476_at	25	21	30	35	110	161	60	38	45	36	82	38	87	130	86	5	21	6	121	197	72
214477_at	12	9	7	6	21	36	49	15	7	13	6	14	7	3	3	5	5	4	2	6	3
214478_at	52	10	118	39	227	103	155	117	135	145	104	117	34	12	10	15	34	32	2	35	40
214479_at	267	40	37	79	67	66	37	42	48	39	41	48	5	10	56	11	11	11	11	17	19
214480_at	12	5	7	14	16	44	16	16	11	14	11	10	2	33	22	3	5	2	13	24	24
214481_at	45	120	75	111	223	59	20	141	83	56	135	293	99	62	87	23	40	28	5	27	11
214482_at	206	212	136	63	54	89	159	158	146	124	198	240	114	165	197	211	208	249	146	101	188
214483_s_at	403	195	408	654	642	629	778	756	514	669	559	711	112	103	78	143	121	144	159	107	203
214484_s_at	1611	1296	1512	603	1218	1074	949	885	1597	1488	1587	987	2706	1008	373	358	314	413	1556	688	697
214485_at	78	77	83	142	216	163	68	141	84	81	117	111	41	27	45	41	21	39	32	36	19
214486_x_at	198	328	309	498	335	328	180	219	261	221	316	256	103	117	170	193	111	163	148	220	148
214487_s_at	590	942	227	292	198	288	685	606	332	595	459	323	151	90	45	61	59	100	66	56	79
214488_at	201	274	98	8	21	12	209	170	52	78	78	68	15	6	17	37	22	52	4	3	2
214489_at	25	47	27	151	32	48	102	74	41	57	88	11	4	4	10	29	37	8	8	17	15
214490_at	36	69	80	28	27	20	30	131	135	34	34	23	38	53	14	30	9	11	11	31	7
214491_at	28	32	18	34	481	259	40	45	41	41	30	46	5	8	25	6	16	11	8	9	6
214492_at	114	17	65	39	133	155	32	139	217	200	28	26	29	12	9	2	19	6	4	26	3
214493_s_at	54	40	110	135	47	74	73	113	112	137	65	83	44	47	41	44	20	22	76	51	21
214494_s_at	668	866	850	960	679	747	1488	776	1021	1339	806	850	605	611	474	467	526	658	908	504	391
214495_at	48	30	30	16	41	24	34	34	35	36	36	60	9	8	22	4	33	4	14	16	12

214496_x_at	296	169	307	491	231	496	453	445	520	639	350	312	101	88	41	57	54	52	109	102	100
214497_s_at	7	6	5	9	10	8	4	7	5	4	4	9	2	7	2	3	8	2	2	4	1
214498_at	180	101	183	136	49	31	191	135	186	106	61	89	1	2	3	2	20	2	4	2	8
214499_s_at	191	103	509	169	717	406	770	1000	883	767	519	405	461	460	190	397	299	419	379	272	324
214500_at	77	410	182	404	15	364	150	164	115	261	360	270	400	216	78	113	97	56	117	50	113
214501_s_at	2121	1639	2773	2402	1526	1054	2967	3065	4145	3575	2481	1661	5424	4193	2489	2009	1981	2135	2480	1891	1906
214502_at	17	2	23	12	555	167	5	8	8	10	136	92	108	35	69	2	3	3	10	3	4
214503_x_at	7	10	9	13	8	13	12	11	15	11	21	9	3	4	1	12	2	2	4	4	1
214504_at	20	18	27	138	30	63	23	22	15	20	16	24	5	9	13	3	7	6	10	6	5
214505_s_at	233	148	160	243	205	442	371	282	165	119	684	468	80	154	235	431	454	432	15	44	62
214506_at	127	57	115	156	213	286	72	211	128	179	216	134	37	11	52	16	30	47	60	42	38
214507_s_at	308	92	685	369	227	40	1010	596	979	1019	1077	1269	1538	1192	992	744	660	952	1129	635	686
214508_x_at	299	282	174	334	67	219	374	317	56	274	328	297	149	145	117	189	135	160	18	114	208
214509_at	30	26	27	69	188	90	23	32	74	117	64	41	5	9	19	1	29	3	30	7	4
214510_at	143	358	22	26	76	52	87	110	33	56	40	85	4	7	3	3	3	5	23	6	3
214511_x_at	152	63	149	25	45	31	160	96	112	32	23	131	6	2	2	2	2	3	5	17	29
214512_s_at	2160	1647	4046	2254	3416	2513	2965	2254	4228	3171	2928	2568	6132	4768	4172	7238	5010	7715	7842	6456	6300
214513_s_at	251	170	309	260	168	323	265	360	332	253	327	353	142	164	149	108	144	74	114	129	137
214514_at	19	10	30	31	25	9	8	16	16	15	17	14	4	5	3	6	1	1	4	8	2
214515_at	9	111	65	140	121	277	61	7	17	48	10	68	7	5	14	51	39	3	26	20	17
214516_at	16	15	47	17	11	28	13	46	68	17	17	44	10	20	36	22	13	44	6	2	21
214517_at	26	21	43	115	25	17	21	178	58	154	26	64	5	6	13	5	5	3	20	12	7
214518_at	255	123	134	158	788	368	215	259	170	217	134	152	30	44	35	15	17	11	40	61	37
214519_s_at	91	85	83	106	471	349	148	162	140	105	178	180	145	154	230	15	11	18	54	59	41
214520_at	45	100	11	120	10	9	53	102	25	88	82	19	4	20	9	2	8	25	3	42	8
214521_at	97	137	43	5	29	32	94	78	3	15	84	132	32	18	30	26	3	4	2	6	3
214522_x_at	25	30	12	22	19	59	30	9	11	17	215	371	248	143	98	40	3	17	2	39	48

214523_at	123	107	94	219	704	586	95	90	76	169	104	33	37	14	38	74	20	98	34	59	39
214524_at	149	12	8	127	324	313	15	67	40	6	77	5	5	28	20	16	26	6	4	35	15
214525_x_at	282	296	355	308	495	410	323	297	320	411	323	333	152	161	157	85	149	148	151	215	161
214526_x_at	1081	907	1388	1663	1590	1579	1532	1486	1450	1639	2199	2282	851	1011	1231	506	541	392	779	504	380
214527_s_at	938	564	1028	907	1731	1540	902	610	1373	1135	2297	1925	3693	2844	984	575	677	485	931	1588	998
214528_s_at	9	7	8	34	55	44	57	38	28	8	75	36	35	20	22	1	5	22	5	1	5
214529_at	124	158	376	204	210	241	148	202	264	454	226	175	29	39	41	44	12	14	67	50	25
214530_x_at	12	9	12	12	16	12	11	15	13	11	13	10	2	3	3	3	2	2	1	2	1
214531_s_at	475	406	408	385	337	406	384	545	409	436	497	466	1008	578	566	359	429	444	685	599	638
214532_x_at	90	182	38	248	158	427	65	201	115	289	84	33	23	7	74	19	57	12	53	34	12
214533_at	69	35	31	71	54	222	83	73	111	57	47	87	9	17	29	14	16	20	8	7	37
214534_at	17	11	12	9	19	19	57	22	60	18	14	63	17	4	2	14	30	30	2	4	3
214535_s_at	116	50	52	91	51	144	46	210	165	124	144	139	7	8	38	20	24	4	6	11	7
214536_at	90	64	84	59	209	246	48	26	147	31	63	29	25	44	41	5	3	7	8	8	19
214537_at	57	40	47	100	32	24	49	46	59	61	185	401	159	52	180	13	14	14	18	43	17
214538_x_at	20	29	26	30	26	30	30	28	36	39	37	26	2	12	7	12	6	18	11	8	2
214539_at	218	30	146	94	224	97	150	186	183	204	132	195	5	33	21	6	19	16	13	6	25
214540_at	20	6	12	58	109	129	9	16	5	13	85	145	60	43	32	5	4	2	3	22	7
214541_s_at	9	9	12	6	26	12	13	11	11	13	9	18	5	3	1	13	3	4	4	16	3
214542_x_at	197	139	83	213	272	687	93	120	29	75	227	405	153	100	96	36	65	59	88	32	13
214543_x_at	388	210	125	122	212	58	376	293	225	249	431	320	163	186	105	135	115	65	21	41	56
214544_s_at	303	106	543	543	515	461	533	513	566	626	460	648	286	122	206	257	229	264	317	195	595
214545_s_at	384	260	236	277	602	470	449	562	273	386	512	397	1281	809	633	296	234	264	348	453	422
214546_s_at	144	193	11	18	247	341	11	13	9	29	9	38	11	9	43	30	63	31	9	36	29
214547_at	67	55	12	6	257	121	34	4	13	32	18	4	32	3	5	14	38	8	16	17	5
214548_x_at	16027	21268	17969	25326	14126	16145	13342	13856	16364	15681	18284	13015	13879	18935	24279	17880	20032	17319	14280	19251	16445
214549_x_at	200	167	358	861	781	771	366	315	397	411	418	231	61	60	89	47	47	45	64	62	43

214550_s_at	1077	395	1013	817	943	809	971	855	1501	1125	875	895	710	500	957	967	661	636	1745	1040	1541
214551_s_at	15	4	7	13	213	226	11	13	7	14	13	10	17	15	23	45	45	8	23	24	5
214552_s_at	388	215	480	446	904	1036	546	426	607	615	765	489	269	189	172	124	103	117	144	96	166
214553_s_at	664	467	392	455	279	301	762	717	661	527	557	412	393	305	262	332	310	323	212	220	230
214554_at	37	15	58	67	135	40	38	62	47	48	57	45	80	67	36	3	1	3	27	36	10
214555_at	12	62	16	13	172	453	23	25	17	17	11	14	18	39	31	32	33	17	15	9	2
214556_at	38	29	41	44	114	54	90	133	211	48	48	136	2	11	13	10	11	7	10	7	2
214557_at	95	39	60	13	34	12	102	51	64	50	63	68	34	32	34	29	25	40	11	24	13
214558_at	250	181	269	327	179	259	290	215	234	273	239	259	26	27	29	27	15	39	43	47	31
214559_at	15	6	7	10	8	32	7	7	16	10	6	88	15	4	21	2	1	4	4	1	23
214560_at	70	40	69	133	58	36	134	91	120	140	111	98	5	30	18	8	9	16	29	8	14
214561_at	98	19	138	92	184	206	60	62	83	91	82	114	5	18	21	19	32	23	19	16	6
214562_at	12	6	24	30	181	15	19	18	83	11	9	11	5	19	30	23	11	30	15	6	39
214563_at	81	68	157	178	425	54	95	37	90	45	75	78	13	5	17	45	3	30	4	44	15
214564_s_at	87	63	12	145	276	194	111	120	134	129	324	173	4	8	30	37	4	1	43	6	45
214565_s_at	25	11	12	16	22	26	16	13	17	14	9	16	3	4	5	3	4	6	11	6	3
214566_at	37	26	22	93	228	254	64	44	20	66	31	83	2	18	13	1	13	25	4	5	14
214567_s_at	8	6	5	6	30	78	7	5	9	4	6	8	1	2	2	4	2	2	1	47	15
214568_at	34	26	16	41	88	74	24	119	21	22	63	31	9	32	14	7	29	6	5	6	7
214569_at	90	58	119	67	133	132	78	74	84	60	95	64	13	11	26	23	3	8	20	23	25
214570_x_at	45	22	72	39	397	317	49	41	23	25	21	18	39	28	29	10	26	31	7	11	41
214571_at	16	7	7	12	819	517	12	11	5	6	803	230	953	922	868	2	7	8	15	142	52
214572_s_at	136	208	107	167	291	270	162	156	138	144	169	146	69	46	60	72	78	32	5	20	12
214574_x_at	53	45	42	45	49	66	41	48	32	42	36	40	12	3	11	8	13	15	11	10	12
214575_s_at	94	56	24	115	80	39	139	75	116	96	118	97	23	48	3	4	22	3	5	6	13
214576_at	7	18	12	17	16	26	20	15	5	10	11	14	7	6	3	1	2	3	1	4	2
214577_at	12	45	14	18	66	325	66	11	17	13	188	119	50	25	20	2	3	3	11	19	23

214578_s_at	586	588	936	759	1299	1639	702	895	573	588	1107	621	332	394	332	391	378	432	302	305	366
214579_at	53	47	317	250	56	36	140	69	142	36	33	131	56	13	111	12	8	15	110	95	127
214580_x_at	17	43	24	34	29	180	19	16	83	18	31	94	4	15	14	4	36	6	6	5	6
214581_x_at	947	1867	3436	1332	254	294	1179	1445	3310	5727	344	268	290	278	208	632	566	546	1803	945	1172
214582_at	138	118	745	709	164	202	179	191	828	785	125	89	43	38	58	143	114	78	914	430	591
214583_at	135	131	132	132	125	218	170	139	171	166	136	124	135	90	77	121	147	138	189	109	151
214584_x_at	61	18	22	39	58	28	52	80	28	32	71	89	11	20	4	14	5	31	11	5	20
214585_s_at	1479	1596	1309	1696	1078	1103	1715	1562	1386	1215	1356	572	722	957	1088	2170	1537	1608	1601	1311	1800
214586_at	21	11	19	32	29	93	3	5	28	10	4	3	1	2	1	3	26	1	24	20	50
214587_at	29	29	34	41	15	99	3	3	9	1	11	26	1	2	7	3	1	7	8	7	9
214588_s_at	116	95	137	169	84	66	146	120	147	280	196	287	55	8	14	82	55	51	90	54	65
214589_at	8	10	4	5	19	32	9	36	11	57	11	9	1	8	13	3	4	4	22	7	3
214590_s_at	259	192	178	114	199	208	99	182	74	171	88	224	214	173	68	194	148	180	318	199	304
214591_at	94	35	132	181	157	93	86	116	106	131	99	123	13	27	8	3	3	3	52	82	73
214592_s_at	5	4	19	23	70	211	9	5	11	11	10	75	51	24	82	36	26	25	97	55	23
214593_at	66	11	76	72	54	129	34	106	58	71	41	10	12	14	5	27	30	18	57	19	43
214594_x_at	316	343	800	718	866	650	244	438	756	1117	271	703	121	162	130	132	92	238	617	532	599
214595_at	394	530	12	21	573	930	239	149	23	8	58	82	59	82	73	251	342	294	8	21	20
214596_at	66	12	317	211	21	16	106	15	253	151	51	29	7	14	1	2	5	7	250	120	135
214597_at	11	18	24	40	19	19	42	33	59	53	78	6	2	24	7	29	3	21	43	27	42
214598_at	37	44	114	70	22	23	49	80	25	48	60	39	2	4	2	19	14	20	23	18	23
214599_at	232	136	100	233	309	293	228	216	219	235	186	185	48	36	9	31	35	4	38	53	35
214600_at	176	180	129	207	205	144	249	355	230	214	387	209	130	160	169	103	108	80	123	96	106
214601_at	22	4	22	4	8	52	45	34	5	6	6	6	2	1	7	10	23	1	19	7	7
214602_at	8	9	11	6	14	9	11	16	8	13	7	8	2	2	2	2	2	5	1	2	2
214603_at	2937	1675	137	23	26	232	1752	1755	33	89	1	26	20	7	19	1766	1790	1574	334	225	215
214604_at	93	105	65	88	89	59	66	22	68	53	124	127	144	61	124	12	14	11	34	5	3

214605_x_at	4	38	56	102	132	499	4	3	88	78	26	77	20	22	64	15	17	22	20	105	102
214606_at	21	21	9	53	187	192	25	16	12	18	63	59	101	107	82	14	9	9	3	9	9
214607_at	130	135	88	197	136	66	45	127	274	177	71	163	20	36	8	35	59	27	29	19	12
214608_s_at	67	27	28	21	120	34	114	25	36	32	18	38	7	6	5	4	2	3	6	2	3
214609_at	89	112	41	113	304	336	114	79	98	81	202	122	42	59	45	43	37	29	29	47	33
214610_at	161	49	111	94	290	124	91	181	230	165	142	141	2	7	45	13	27	15	42	19	5
214611_at	91	66	98	114	447	313	110	115	20	122	65	128	20	24	46	28	43	32	29	23	13
214612_x_at	7804	4956	11881	8928	136	52	4648	4860	10283	10198	61	75	33	33	20	3848	3226	3454	8450	6398	5709
214613_at	308	27	119	57	132	24	36	629	47	172	377	168	60	66	21	13	35	65	7	36	21
214614_at	581	279	848	630	1194	1064	388	499	1135	1008	446	677	357	207	250	73	33	291	686	515	383
214615_at	33	32	7	70	18	26	36	37	56	31	31	9	2	4	14	1	7	3	24	1	10
214616_at	304	320	289	449	279	168	368	340	413	376	276	293	45	4	22	44	34	51	57	38	54
214617_at	635	242	54	160	11840	8941	606	449	157	136	9400	7334	10775	10405	9581	541	592	541	83	144	127
214618_at	99	44	64	113	147	112	61	59	79	67	64	67	22	10	15	12	14	18	17	2	7
214619_at	58	101	199	57	85	79	201	32	23	130	92	34	31	50	65	58	29	13	47	85	51
214620_x_at	799	1833	642	1552	1596	2462	969	1271	300	414	884	768	1074	1001	778	738	605	882	425	579	696
214621_at	58	10	57	10	103	22	9	7	13	11	24	25	1	2	3	12	28	23	8	9	5
214622_at	25	44	34	31	43	28	34	36	40	36	31	20	9	5	54	30	69	4	16	9	8
214623_at	7	45	12	9	142	130	21	12	37	10	13	11	34	3	10	26	28	47	15	28	22
214624_at	220	174	88	188	136	177	239	187	119	225	179	239	41	7	67	72	69	41	8	6	17
214625_s_at	107	40	117	252	41	32	133	42	193	212	94	21	7	22	9	15	10	12	23	44	30
214626_s_at	1507	1253	1974	1496	1131	958	1581	2359	1785	1585	1693	1636	1083	902	341	455	516	429	452	573	484
214627_at	241	130	201	156	45	112	168	181	241	157	172	58	45	6	7	3	42	13	36	23	23
214628_at	34	46	50	54	70	38	27	26	21	82	23	25	5	6	60	12	17	57	13	9	4
214629_x_at	6099	4744	8078	6624	10408	6364	4355	5937	5756	6198	6887	5855	9788	11405	12763	8420	8039	5600	12724	11470	11675
214630_at	16	17	28	65	22	54	97	20	56	73	18	38	20	18	13	12	24	25	51	20	12
214631_at	9	6	50	30	148	153	90	78	51	106	98	87	9	15	6	1	13	38	17	4	39

214632_at	58	34	62	41	179	500	126	98	76	77	281	156	59	43	89	19	35	30	26	25	44
214633_at	74	26	34	62	172	243	17	55	55	74	37	39	5	6	22	29	2	11	13	38	2
214634_at	13	26	11	125	203	410	11	11	19	28	11	57	27	23	56	8	58	11	50	27	37
214635_at	119	101	220	234	547	681	201	231	201	342	271	122	27	66	69	19	69	55	71	77	45
214636_at	118	16	20	50	91	56	127	63	74	81	44	49	14	28	27	37	31	61	33	10	19
214637_at	13	12	8	9	267	206	7	12	7	7	7	8	6	9	5	3	43	4	4	5	6
214638_s_at	228	90	210	168	180	223	166	305	161	191	134	175	55	87	65	74	51	93	77	74	93
214639_s_at	153	263	123	26	720	559	178	83	75	106	898	498	637	681	873	151	145	104	101	154	134
214640_at	17	9	38	12	63	171	17	3	12	15	4	11	22	50	3	20	24	10	40	24	7
214641_at	28	79	163	138	49	48	164	135	135	144	85	111	4	3	2	3	3	6	9	36	3
214642_x_at	579	373	718	570	52	168	631	608	696	660	14	78	22	24	18	101	36	139	744	288	221
214643_x_at	298	333	281	440	426	270	454	381	657	510	549	260	132	171	179	198	176	113	406	284	231
214644_at	30	11	5	45	58	118	13	57	8	32	14	43	4	4	6	2	2	21	9	17	7
214645_at	54	27	39	81	165	144	70	48	99	31	60	59	19	28	20	17	17	16	29	21	13
214646_at	119	38	161	115	22	95	74	13	75	134	81	50	2	32	1	23	57	4	14	5	26
214647_s_at	343	300	149	314	109	189	232	243	149	147	117	72	23	31	24	67	92	56	60	34	41
214648_at	89	40	57	41	114	55	45	40	96	91	146	88	17	33	50	52	29	52	21	5	13
214649_s_at	291	180	785	583	601	753	451	315	1363	716	324	266	327	286	230	355	254	452	966	462	733
214650_x_at	62	84	126	140	25	52	80	153	140	161	179	141	2	29	19	29	2	15	27	39	25
214651_s_at	3915	4428	821	1522	2229	3228	3191	5838	1038	1027	2327	2906	2540	3531	4012	6648	4572	3866	2776	2896	3350
214652_at	21	7	7	32	117	17	8	8	11	11	37	10	1	4	5	20	19	17	1	4	7
214655_at	94	9	94	13	48	74	85	24	71	49	17	14	40	4	33	30	11	11	4	2	3
214656_x_at	1413	1181	1328	1703	1179	1712	1072	1131	1397	2020	1249	936	1465	1380	1217	704	732	683	1545	1101	1307
214657_s_at	302	869	246	488	378	431	199	368	409	844	216	268	124	71	115	37	76	117	42	64	64
214658_at	1322	752	1021	657	1226	2948	223	639	268	439	506	721	1645	1084	764	1604	1307	1911	1816	2934	3987
214659_x_at	274	219	514	312	543	246	702	643	462	477	829	522	625	390	261	166	197	145	172	179	121
214660_at	1	15	61	22	157	13	11	13	21	55	82	52	87	93	123	10	7	1	39	79	116

214661_s_at	1111	1195	853	345	283	308	1280	1164	1363	890	649	623	739	618	881	1872	1962	2606	1718	1604	1677
214662_at	1474	903	610	270	471	499	1073	740	522	486	561	402	830	912	2221	1957	1582	1804	1947	1508	1674
214663_at	198	128	246	316	56	519	204	232	229	138	388	248	354	313	215	78	161	79	153	164	125
214664_at	69	57	91	14	73	73	232	223	86	84	94	54	48	36	41	56	39	21	76	4	31
214665_s_at	2656	3575	2527	5274	1350	1265	2964	3664	3172	2820	1403	1266	3710	3318	3886	6899	7059	5396	7588	8748	9530
214666_x_at	243	80	255	147	313	282	146	162	139	204	156	172	118	93	42	44	22	44	175	152	116
214667_s_at	176	141	372	1416	210	95	253	269	420	334	117	25	20	8	10	14	6	51	70	39	45
214668_at	185	128	129	106	308	235	195	158	120	126	156	240	66	58	42	58	64	59	48	42	42
214669_x_at	111	44	47	120	263	298	81	98	52	31	101	54	5	6	37	2	4	26	35	8	1
214670_at	521	595	545	1463	928	1074	604	1153	238	516	674	1069	341	311	614	575	562	517	587	1202	2130
214671_s_at	146	234	661	762	979	840	220	248	581	714	893	416	56	71	102	75	64	77	184	106	88
214672_at	500	460	593	654	1003	898	603	622	463	653	894	1052	442	414	472	295	305	251	461	392	401
214673_s_at	179	13	179	125	109	253	172	208	194	200	350	215	5	52	2	5	40	19	43	19	3
214674_at	228	195	123	185	388	345	227	172	211	152	178	60	124	161	121	80	101	182	90	74	76
214675_at	40	88	80	21	22	30	37	25	139	61	107	80	25	3	24	14	34	7	32	54	37
214676_x_at	212	277	316	153	318	351	148	267	248	287	144	119	26	21	73	47	41	33	105	103	68
214677_x_at	25	22	23	19	117	226	21	20	24	21	21	18	3	5	5	9	5	16	19	14	19
214678_x_at	221	106	73	91	252	247	89	135	58	87	189	210	461	186	194	226	175	165	292	342	339
214679_x_at	1382	1118	895	839	889	738	1451	940	789	1097	1188	615	580	450	375	384	372	491	444	298	185
214680_at	20	7	19	10	121	93	45	21	25	39	53	19	13	11	12	3	9	7	16	3	37
214681_at	44	116	71	96	78	191	34	106	67	49	131	172	98	105	114	52	16	93	67	86	106
214682_at	82	21	38	23	48	17	9	20	120	43	11	25	3	48	34	19	66	23	55	14	3
214683_s_at	198	145	490	279	524	415	119	304	290	416	475	527	505	518	397	287	310	389	582	513	829
214684_at	382	454	434	341	261	390	213	300	341	616	419	201	508	391	285	464	522	870	1102	1315	1317
214685_at	28	9	31	19	129	40	28	49	17	13	9	16	2	30	2	13	3	13	11	4	9
214686_at	316	290	431	532	553	435	338	487	288	383	435	445	352	218	317	242	243	327	279	268	284
214687_x_at	16168	15247	16322	13528	7968	7530	12556	8885	14135	10977	13855	9991	13023	14677	13766	13113	13476	13687	11628	11468	9574

214688_at	67	33	71	21	154	254	38	29	92	66	98	83	34	52	36	15	36	26	36	47	49
214689_at	71	17	107	100	191	130	30	116	86	59	101	28	7	7	33	9	17	51	15	9	8
214690_at	98	54	114	49	179	94	41	74	79	89	77	84	105	104	109	122	97	134	183	125	304
214691_x_at	258	125	265	127	458	345	155	214	118	145	243	222	56	69	124	209	123	72	96	149	126
214692_s_at	29	60	24	25	69	251	48	131	24	21	33	15	111	66	54	44	12	98	8	28	82
214693_x_at	602	249	826	1064	1080	2146	1273	2002	1228	1557	1171	1230	443	283	174	411	402	256	184	217	106
214694_at	360	255	441	449	224	418	456	356	388	372	318	251	41	65	36	15	42	49	57	121	84
214695_at	263	159	296	404	313	167	290	255	302	348	290	243	141	148	148	156	174	171	193	187	132
214696_at	333	283	837	714	1271	3198	135	140	330	233	800	456	875	994	1303	217	370	347	261	503	624
214697_s_at	859	406	1192	975	646	646	947	1393	1564	1516	1208	638	280	240	296	255	406	494	470	245	411
214698_at	340	175	517	233	535	676	505	389	824	629	458	318	178	107	194	174	116	163	296	167	199
214699_x_at	434	339	374	498	924	1220	472	433	396	481	472	432	113	114	179	193	130	145	161	119	94
214700_x_at	242	219	351	189	838	466	265	395	458	334	399	261	550	189	218	274	171	281	826	405	769
214701_s_at	204	200	206	62	59	77	195	241	198	240	360	314	42	29	57	7	39	31	7	12	24
214702_at	26	18	35	75	5	59	21	42	7	21	33	39	13	7	13	1	11	14	8	3	1
214703_s_at	441	877	406	1156	661	992	407	560	368	503	314	461	297	190	129	214	254	295	315	355	254
214704_at	20	15	18	19	27	34	13	18	11	22	9	14	8	9	4	3	2	7	5	9	6
214705_at	69	56	75	180	78	106	52	87	82	82	50	40	22	17	43	36	27	27	84	58	82
214706_at	332	186	441	268	594	433	264	274	484	444	470	358	235	343	356	229	255	225	460	343	505
214707_x_at	609	450	431	673	793	300	384	810	582	619	540	607	125	200	183	125	153	213	224	282	229
214708_at	66	55	228	502	451	238	13	62	155	106	242	62	153	174	52	54	46	39	192	285	365
214709_s_at	1672	2243	2432	1560	3282	3778	1659	2869	2468	3073	3027	2240	5790	5489	4682	2768	1664	4032	5212	5514	6891
214710_s_at	4206	508	3657	543	4914	375	3331	3079	3646	2616	3732	3435	6954	7349	8083	5762	5241	5679	9166	3581	5077
214711_at	857	759	779	700	260	732	423	545	739	1051	632	431	426	360	549	316	391	268	590	735	413
214712_at	5	24	30	67	19	28	19	29	47	28	6	3	12	1	11	1	11	2	11	21	9
214713_at	213	303	369	304	301	535	341	366	171	386	422	309	143	96	171	59	42	83	144	126	148
214714_at	386	524	460	616	896	614	364	408	395	386	796	716	733	1140	986	640	862	692	616	537	658

214715_x_at	681	647	720	839	2026	1664	484	814	664	913	726
1039	367	370	353	213	185	319	358	501	555		
214716_at	115	5	18	43	56	102	11	53	51	61	65
33	37	57	14	24	26	32	25	35	17		
214717_at	194	72	274	292	166	47	193	501	134	361	165
217	176	119	72	65	77	69	101	90	100		
214718_at	315	260	597	834	737	1074	276	375	531	650	564
773	1161	974	865	86	67	78	1084	1714	1432		
214719_at	98	105	43	440	223	391	148	106	24	124	291
285	107	223	219	106	108	85	160	236	298		
214720_x_at	636	455	952	791	944	668	1081	961	777	1440	1040
1252	432	431	269	526	372	439	481	308	542		
214721_x_at	754	544	578	881	285	323	822	790	781	917	480
515	199	273	146	439	362	436	686	376	359		
214722_at	249	291	506	1286	594	519	321	548	563	667	453
882	208	214	324	177	131	206	386	531	658		
214723_x_at	105	40	68	87	54	15	56	157	78	61	50
94	50	27	12	43	26	44	62	30	54		
214724_at	180	68	142	281	243	399	168	91	203	193	85
102	96	81	85	127	125	73	328	247	510		
214725_at	3	9	16	58	69	69	41	11	7	29	13
1	6	6	15	8	2	14	62	50	56		
214726_x_at	915	1165	806	1192	893	523	1264	1536	888	1227	1026
661	103	188	105	146	127	175	170	113	138		
214727_at	131	28	366	88	557	247	191	286	474	495	517
320	269	207	149	77	72	123	424	144	225		
214728_x_at	1913	1923	2031	1060	1553	1037	1888	1528	2094	2144	2271
1245	1182	1307	979	761	703	588	1007	855	737		
214729_at	146	50	164	16	60	145	195	148	194	205	213
113	57	55	65	76	125	68	66	32	52		
214730_s_at	3441	3390	2321	2315	1221	1689	3901	4402	2969	3171	1744
1488	1248	1400	1947	3677	4159	3893	2016	3092	3419		
214731_at	267	308	317	325	381	293	359	426	349	442	247
205	15	25	44	52	48	70	76	109	62		
214732_at	107	88	361	325	34	43	382	335	280	333	149
275	14	39	31	27	27	15	16	51	27		
214733_s_at	233	422	229	1017	739	645	538	558	407	477	621
571	396	392	322	214	281	244	219	282	302		
214734_at	378	324	1546	1261	1357	800	496	780	1303	1040	1516
798	181	184	223	105	100	46	222	194	206		
214735_at	50	107	15	12	140	99	29	41	16	24	26
14	18	24	8	23	20	23	25	22	15		
214736_s_at	1258	1898	1062	1630	998	938	1183	1795	1196	1231	1077
709	669	521	353	530	482	412	388	310	347		
[truncated: 1,165,737 more chars]
